# Taxonomic revision of the *Temnothorax salvini* clade (Hymenoptera: Formicidae), with a key to the clades of New World *Temnothorax*

**DOI:** 10.7717/peerj.11514

**Published:** 2021-06-30

**Authors:** Matthew M. Prebus

**Affiliations:** 1 School of Life Sciences, Arizona State University, Tempe, Arizona, United States; 2 Department of Entomology & Nematology, University of California, Davis, Davis, California, United States

**Keywords:** Biodiversity, Taxonomy, Morphometry, Mesoamerica, Formicidae, Natural history, Caribbean, Greater Antilles, South America, Mexico

## Abstract

*Temnothorax* is a large myrmicine ant genus with a range spanning the northern hemisphere, including the northern half of the Neotropics. Many of the Neotropical species were originally placed in the now defunct genus *Macromischa*. Recent molecular work has revealed that distinct lineages of Neotropical *Temnothorax* have arrived by evolutionary convergenceat a morphological syndrome with characteristics that were used to diagnose the former genus *Macromischa*. One such lineage is the *salvini* clade, which in this study is redefined to contain 63 species, 35 of which are described as new. A key to all species of the *salvini* clade based on the worker caste is provided; additionally, a worker-based key to all clades of the New World is provided. The following species are redescribed: *T. albispinus* (Wheeler), *T. androsanus* (Wheeler), *T. annexus* (Baroni Urbani), *T. augusti* (Baroni Urbani), *T. aztecus* (Wheeler), *T. ciferrii* (Menozzi & Russo), *T. flavidulus* (Wheeler & Mann), *T. fuscatus* (Mann), *T. goniops* (Baroni Urbani), *T. huehuetenangoi* (Baroni Urbani), *T. ixili* (Baroni Urbani), *T. leucacanthus* (Baroni Urbani), *T. nigricans* (Baroni Urbani), *T. ocarinae* (Baroni Urbani), *T. pastinifer* (Emery), *T. pergandei* (Emery), *T. politus* (Smith), *T. pulchellus* (Emery), *T. rugosus* (Mackay), *T. salvini* (Forel), *T. schwarzi* (Mann), *T. skwarrae* (Wheeler), *T. subditivus* (Wheeler), *T. tenuisculptus* (Baroni Urbani), *T. terricola* (Mann), *T. terrigena* (Wheeler), *T. torrei* (Aguayo). The gynes of *T. ciferrii, T. fuscatus, T. ixili, T. politus, T. rugosus, T. salvini, T. tenuisculptus* and *T. torrei* are described. The males of *T. albispinus* and *T. fuscatus* are described. Lectotypes are designated for *T. androsanus, T. annexus, T. augusti, T. aztecus, T. flavidulus, T. fuscatus, T. nigricans, T. pastinifer, T. pergandei, T. politus, T. pulchellus, T. salvini, T. skwarrae, T. subditivus, T. terricola, and T. terrigena*. A neotype for *Temnothorax salvini obscurior* (Forel) is designated, the taxon is raised to species, and a replacement name is designated: *T. longicaulis* **stat. nov., nom. nov.** The following species are described as new: *T. achii* **sp. nov.**, *T. acuminatus* **sp. nov.**, *T. acutispinosus* **sp. nov.**, *T. agavicola* **sp. nov.**, *T. altinodus* **sp. nov.**, *T. arbustus* **sp. nov.**, *T. aureus* **sp. nov.**, *T. aztecoides* **sp. nov.**, *T. bahoruco* **sp. nov.**, *T. balaclava* **sp. nov.**, *T. balnearius* **sp. nov.**, *T. bison* **sp. nov.**, *T. casanovai* **sp. nov.**, *T. fortispinosus* **sp. nov.**, *T. harlequina* **sp. nov.**, *T. hippolytus* **sp. nov.**, *T. laticrus* **sp. nov.**, *T. leucacanthoides* **sp. nov.**, *T. longinoi* **sp. nov.**, *T. magnabulla* **sp. nov.**, *T. misomoschus* **sp. nov.**, *T. nebliselva* **sp. nov.**, *T. obtusigaster* **sp. nov.**, *T. paraztecus* **sp. nov.**, *T. parralensis* **sp. nov.**, *T. parvidentatus* **sp. nov.**, *T. pilicornis* **sp. nov.**, *T. quercicola* **sp. nov.**, *T. quetzal* **sp. nov**., *T. rutabulafer* **sp. nov.**, *T. terraztecus* **sp. nov.**, *T. tuxtlanus* **sp. nov.**, *T. wettereri* **sp. nov.**, *T. wilsoni* **sp. nov.**, *T. xincai* **sp. nov.**

## Introduction

### Overview

With 455 extant species and subspecies (Bolton, 2021), the genus *Temnothorax* Mayr is a large, morphologically diverse genus of small myrmicine ants, as well as one of the most taxonomically challenging. The geographic range of *Temnothorax* encompasses most of the northern hemisphere, including northern portions of the Neotropics and Afrotropics (Prebus, 2015; Janicki et al., 2016; Guénard et al., 2017; Prebus, 2017). *Temnothorax* species may be found in a wide range of habitats, from lowland tropical rainforest to the boreal zone, from hot deserts to cloud forests throughout their range. The members of *Temnothorax* also vary broadly in nesting behavior, with some species nesting directly in the ground, and others from the Greater Antilles building carton nests (Guérin-Méneville, 1852). The majority of *Temnothorax* species, however, inhabit small, preformed cavities, including rock crevices, hollow nut shells, hollow dead twigs, and epiphyte root mats. The former two nesting preferences have led to the genus monikers “rock ants” and “acorn ants”.

Historically, *Temnothorax* has been considered a northern temperate zone genus, where much of the recent taxonomic attention has been focused. Our comprehension of intraspecific morphological variability and the range of individual *Temnothorax* species has been hindered by the general paucity of specimens for many species, not to mention a lack of material beyond the type specimens for some. The preceding factors have contributed to some of the taxonomic confusion around *Temnothorax* species, but additional issues include morphological conservatism among many closely related species, vast departures from the morphological norm in others, and rampant morphological convergence throughout the genus. The latter facet is especially true in the Neotropics, where *Temnothorax* is highly diverse (Baroni Urbani, 1978; Mackay, 2000; Fontenla Rizo, 2000; Vásquez-Bolaños, 2011, 2015), and with arguably more morphological disparity concentrated in this region than in the entire Holarctic range. For example, many of the Neotropical species were previously assigned to the former genus *Macromischa* Roger, erected to hold Neotropical species that bore the following characteristics (Roger, 1863):

1. Petiole with a long peduncle
2. Postpetiole campaniform in dorsal view
3. Dorsum of mesosoma in profile view convex, without sutures

Other characteristics that often accompany those mentioned above are iridescently colored integument and incrassate, spindle-shaped femora. Past taxonomists have noted that many Neotropical and southern Nearctic *Temnothorax* species bear one or a few of the characteristics of *Macromischa*, to the point that easy distinction between the two genera was untenable. In light of this, Baroni Urbani (1978) reclassified *Macromischa* as a subgenus of *Leptothorax* Mayr. *Macromischa* was later synonymized with *Leptothorax* by Snelling (1986), then revived as a more narrowly defined, unranked taxon by Fontenla Rizo (2000). Following all of this, Bolton (2003) transferred most species of *Leptothorax* to *Temnothorax*, leaving *Leptothorax* with a handful of circumboreal species closely related to *L. acervorum* (Fabricius).

In the Americas, the counterpart to *Macromischa* is the former subgenus *Myrafant* Smith, which was erected to accommodate the bulk of Nearctic *Temnothorax* species. The worker of the subgenus was loosely defined as having the following characteristics, which here are updated to the current terminology from the original diagnosis:

- 11- or 12-segmented antennae
- In dorsal view, pronotal humeri usually rounded; occasionally subangular
- Metanotal groove usually absent; if present, barely perceptible

Recent studies incorporating morphological and molecular phylogenetics have further clarified the global placement of *Temnothorax* within the ant family, as well as relationships between the former subgenera and species within the genus. Moreau et al. (2006) and Brady et al. (2006) both placed *Temnothorax* as sister to *Leptothorax* and *Formicoxenus* Mayr; Ward et al. (2015) and Blaimer et al. (2018) elaborated on this placement, showing that *Temnothorax* was a member of a large group of myrmicines centered in the Afrotropics: the redefined tribe Crematogastrini. Furthermore, Ward et al. (2015), Prebus (2017), and Blaimer et al. (2018) placed *Temnothorax* in the *Formicoxenus* genus group (of the former tribe Formicoxenini), which includes the genera *Gauromyrmex* Menozzi, *Vombisidris* Bolton, *Harpagoxenus* Forel, *Formicoxenus*, and *Leptothorax*.

**Figure 1:**
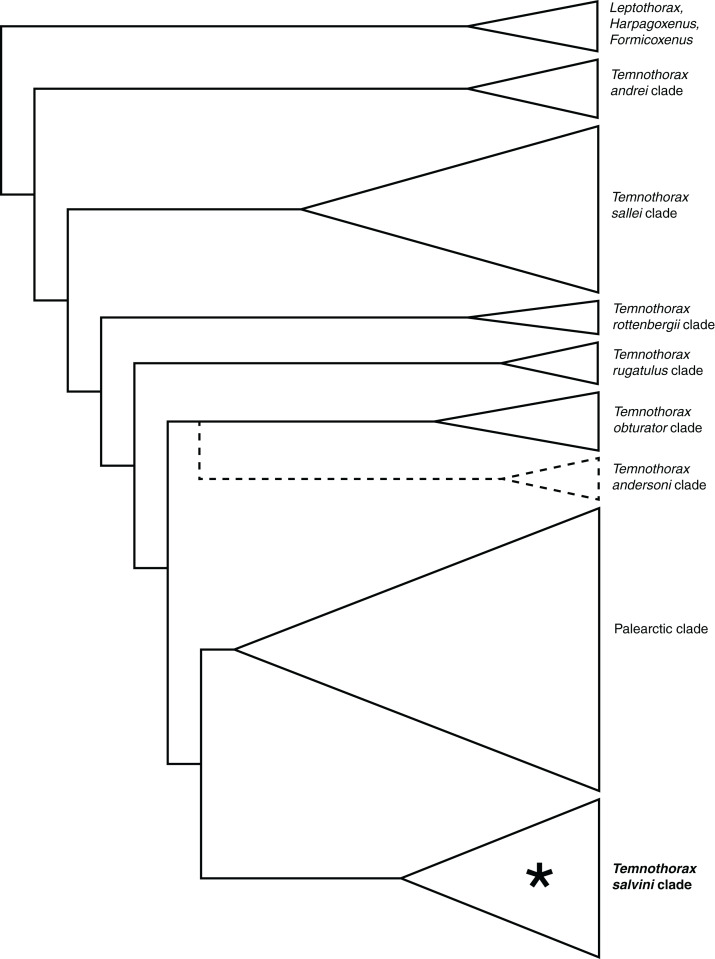
Cladogram representing relationships between the clades of *Temnothorax* found in Prebus (2017). The *andersoni* clade, which was not sampled in that study, is represented by dotted lines. The *salvini* clade is indicated by an asterisk. Triangles are proportional to the number of taxa sampled in Prebus (2017).

A recent study (Prebus, 2017) provided evidence for the Nearctic origin of the crown *Temnothorax*, followed by a rapid radiation across the majority of the present-day range. The initial radiation resolved into seven large clades, including several lineages that dispersed to the Neotropics soon after the origin of the crown group. Several of these Neotropical lineages converged upon at least part of the *Macromischa* syndrome, and among these early-arriving Neotropical lineages is the morphologically heterogenous *salvini* clade, which was recognized in Prebus (2017). See Fig. 1 for a summary of the relationships of the *Temnothorax* clades.

Fontenla Rizo (2000) held that *Macromischa* can be defined as an unranked, monophyletic taxon consisting of the arboreally- and limestone nesting species of the Greater Antilles. However, the redefined *Macromischa* is apparently paraphyletic as well, interdigitated with mainland relatives of the *sallei* clade (Prebus, 2017).

Prebus (2017) showed some uncertainty about the position of the *salvini* clade among analyses, but it was always closely associated with the Palearctic clade and the *obturator* clade in that study. The Palearctic clade, as the name suggests, is a highly diverse group found primarily in the temperate zones of Europe and Asia, but also has a small offshoot in North America in *T. furunculus* (Wheeler), *T. americanus* (Emery), and the *longispinosus* species group. The Palearctic clade contains all of the socially parasitic former satellite genera of *Temnothorax* (Ward et al., 2015; Prebus, 2017). The *obturator* clade is relatively species poor as we currently understand it, but has a broad range, extending from the American Southwest, throughout northern Meso-America and as far east as Puerto Rico. A member of the *obturator* clade was also classified as *Macromischa* by Baroni Urbani (1978), namely *T. creolus* (Baroni Urbani) of Hispaniola. Recent work has provided evidence for an additional Nearctic lineage in *Temnothorax*, the *andersoni* clade (Fig. 1; Prebus in prep.), which appears to be the sister group of the *obturator* clade.

Prebus (2017) and ongoing morphological and molecular studies (Prebus, in prep.) have revealed that the *salvini* clade is a collection morphologically heterogeneous species. The majority of *salvini* clade species have historically been classified as *Macromischa*, but another component of the *salvini* clade is the morphologically aberrant species *Temnothorax pergandei* (Emery), which was previously described as the genus *Dichothorax* Emery. While *T. pergandei* is similar to certain species of the Mediterranean region in Europe, e.g., *T. schaufussi* (Forel), *T. cagnianti* (Tinaut) (Mackay, 1993; Espadaler, 1997; see CASENT0915394 on antweb.org), this appears to be yet another case of convergent evolution in the genus (Prebus, 2017). The final component of the *salvini* clade are species which are closer to the morphological norm of the temperate zone *Temnothorax* species for which the subgenus *Myrafant* was erected, e.g. *T. terrigena* (Wheeler). Given the amount of morphological variation in the clade, morphological diagnosis (provided below) remains challenging, requiring a combination of traits. This appears to be the rule throughout the genus: in the key to the clades of New World *Temnothorax* below, the *salvini* and *sallei* clades, which broadly overlap geographically, are difficult to disentangle based on morphology and each key out in several places as a result.

**Figure 2:**
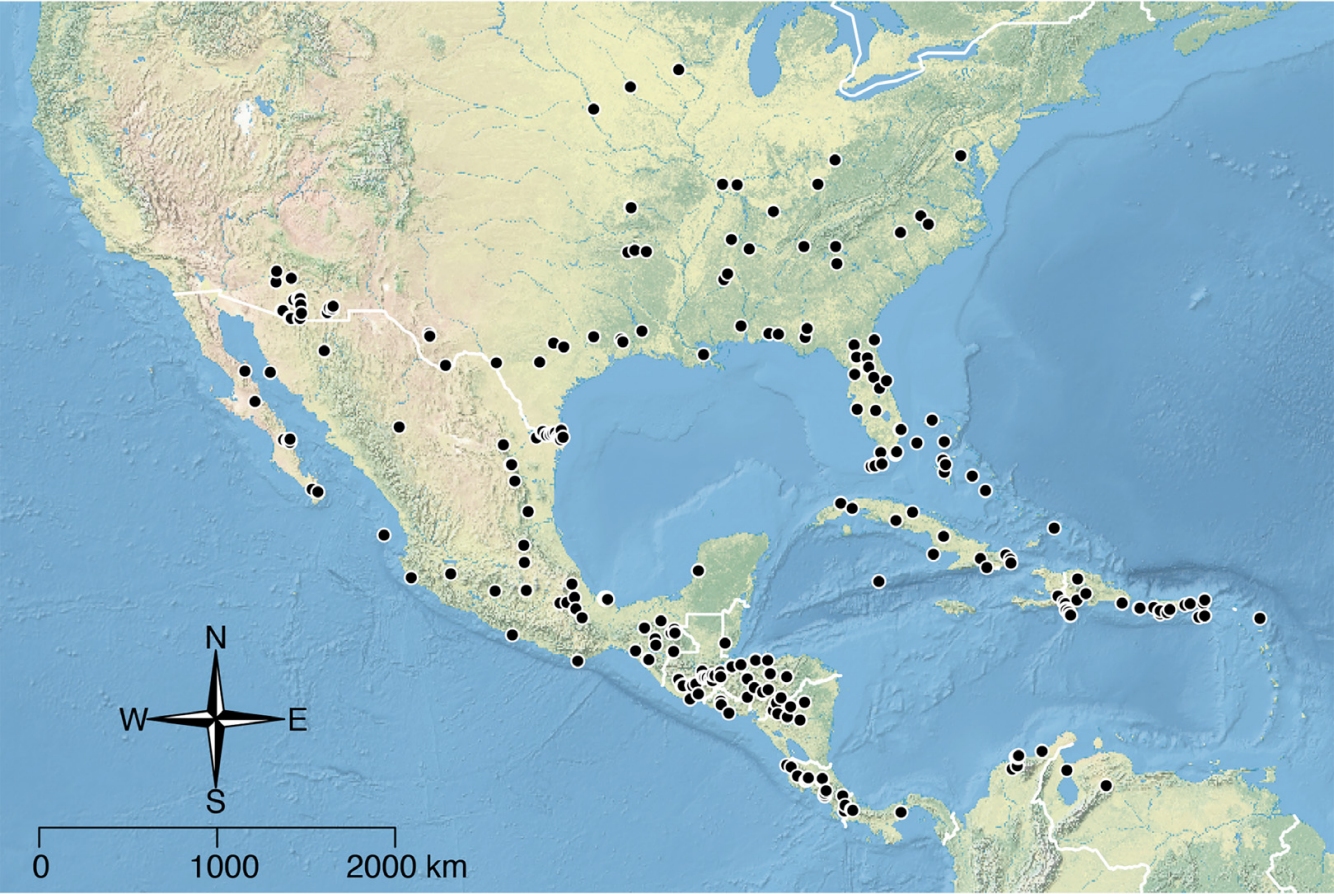
Geographical distribution of all specimens of the *salvini* clade examined in this study.

The *salvini* clade, as it is currently understood, consists of 28 described species which span eastern North America to northern South America (Fig. 2). However, recent initiatives such as the Arthropods of La Selva project (ALAS), Leaf Litter Arthropods of Meso-America (LLAMA), and Ant Diversity of the Meso-American Corridor (ADMAC) have revealed a trove of undescribed *Temnothorax* species from the Neotropics, many of which are members of the *salvini* clade. Recently, Prebus (2021) used a combination of morphological and phylogenomic data to disentangle a particularly taxonomically difficult Mesoamerican species group, the *salvini* group, consisting of the previously described species *T. salvini* (Forel) and *T. aztecus* (Wheeler), and their undescribed close relatives. In addition to the seven undescribed species detected in Prebus (2021), I describe an additional 28 species from other species groups in the clade, more than doubling the size of the *salvini* clade. To facilitate the diagnosis of the *salvini* clade, as well as all other clades identified in Prebus (2017), I have also provided a dichotomous key to all clades of *Temnothorax* in the Americas. Additionally, I have included a key to species of the *salvini* clade based on workers, and a key to species groups of the *salvini* clade based on the wings of the reproductive castes.

### Natural history of the *salvini* clade

The following is an overview of the little that is known about the natural history of the members of the *salvini* clade. Notes on the natural history of other American *Temnothorax* species can be found in Mackay (2000) and Baroni Urbani (1978). Species-specific natural history details of the *salvini* clade are elaborated on in the taxon treatments below.

With the exception of *Temnothorax pergandei*, members of the *salvini* clade have ranges that are centered around the northern Neotropics and subtropics (*T. pergandei* is found from as far north as Michigan, U.S.A. to as far south as Hidalgo, Mexico). The habitats in which the members of the *salvini* clade are found are nearly as variable as the entirety of *Temnothorax:* they live in hot deserts, temperate woodlands, cloud forests, lowland rainforests, tropical dry forests, and littoral habitats. They are found from sea level to 3,180 m (*T. subditivus* (Wheeler) from Totonicapan, Guatemala), but are especially common in mid-elevation mesic forest.

The nesting habits of the *salvini* clade are highly diverse: members typically nest in hollow, dead vegetation, such as twigs and vines, but some species are able to nest directly in the ground (for example, *Temnothorax pergandei* and *T. pastinifer* (Emery)). Another common nesting place for *salvini* clade species is between the leaves or under the root mats of epiphytes (for example *T. tenuisculptus* (Baroni Urbani), *T. skwarrae* (Wheeler), *T. ixili* (Baroni Urbani), *T. subditivus, T. fuscatus* (Mann), and *T. ocarinae* (Baroni Urbani)). Several of the species described below are known only from Winkler leaf litter extractions; they may be nesting between leaves, but more likely they are nesting in dead wood or hollow nut shells in the litter similar to other *Temnothorax* species. Some of the general characteristics of *Temnothorax* that may facilitate the flexibility of nest preference in the *salvini* clade are its small nest sizes, usually with about 200 workers per colony (Beckers et al., 1989), tendency toward polydomy (Alloway et al., 1983; Foitzik & Heinze, 2001; Partridge, Partridge & Franks, 1997), and resilience toward starvation (Rüppell & Kirkman, 2005).

While nest census data for the *salvini* clade are sparse, most of the species for which data are available are monogynous, with a couple of exceptions: *Temnothorax nigricans* (Baroni Urbani) and *T. fuscatus* both have been collected with multiple dealate gynes. This, of course, does not preclude the possibility that these species have auxiliary gynes but are functionally monogynous, like some species in the closely related genus *Leptothorax* (Heinze & Buschinger, 1987; Frumhoff & Ward, 1992). To determine true polygyny, dissections of the gynes would be necessary to examine whether the spermathecae are full and ovules are developing. As these specimens are dry mounted (and in the case of *T. nigricans*, part of the syntype series), I have not been able to confirm that these are instances of true polygyny in the *salvini* clade.

**Figure 3:**
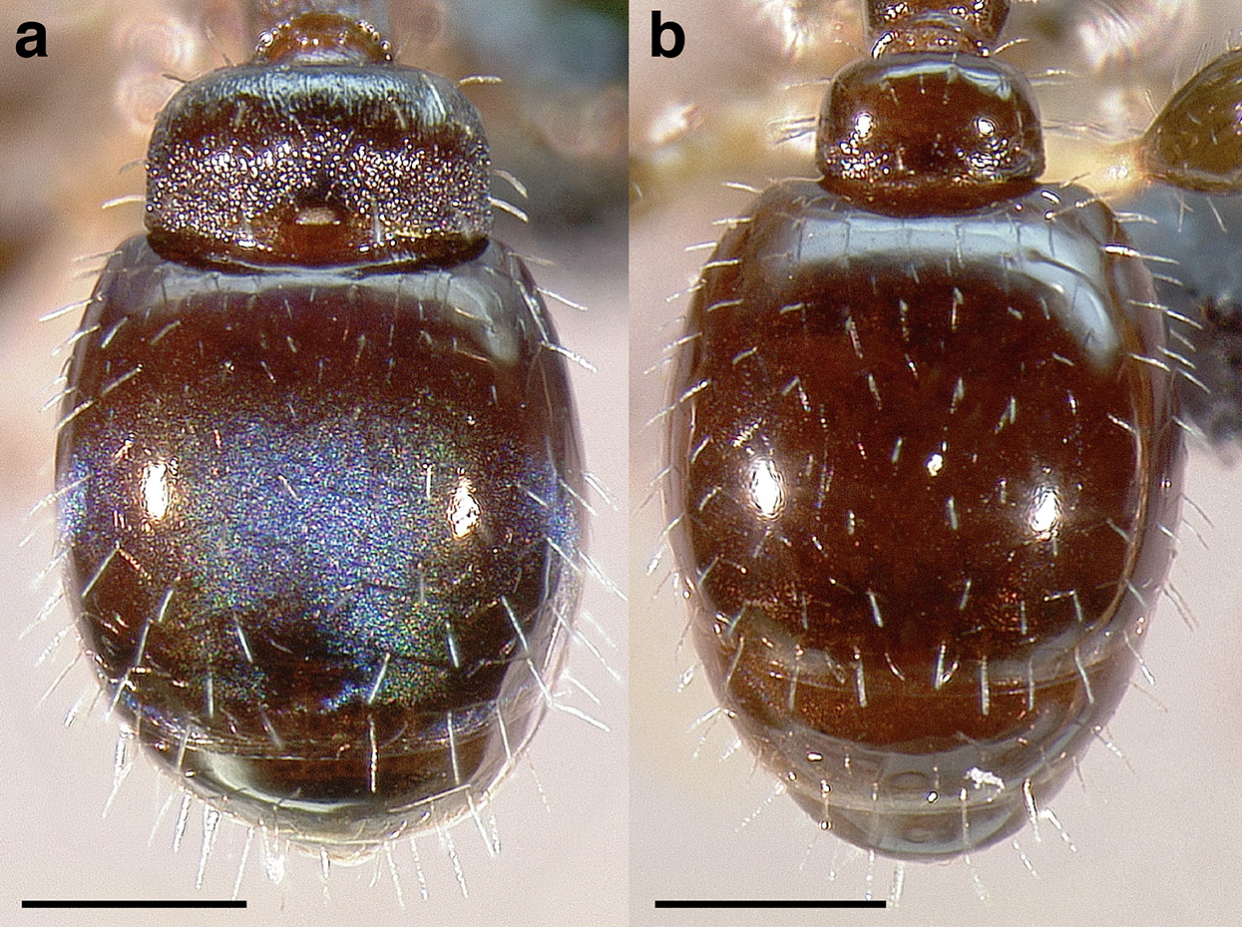
Demonstration of spectral iridescence in *Temnothorax* workers under direct illumination from fiber optic lights. Gaster in dorsal view. (A) With spectral iridescence: *T. laticrus* sp. nov. (CASENT0758263). (B) Without spectral iridescence: *T. politus* (CASENT0756845). Scale bars 0.2 mm.

**Figure 4:**
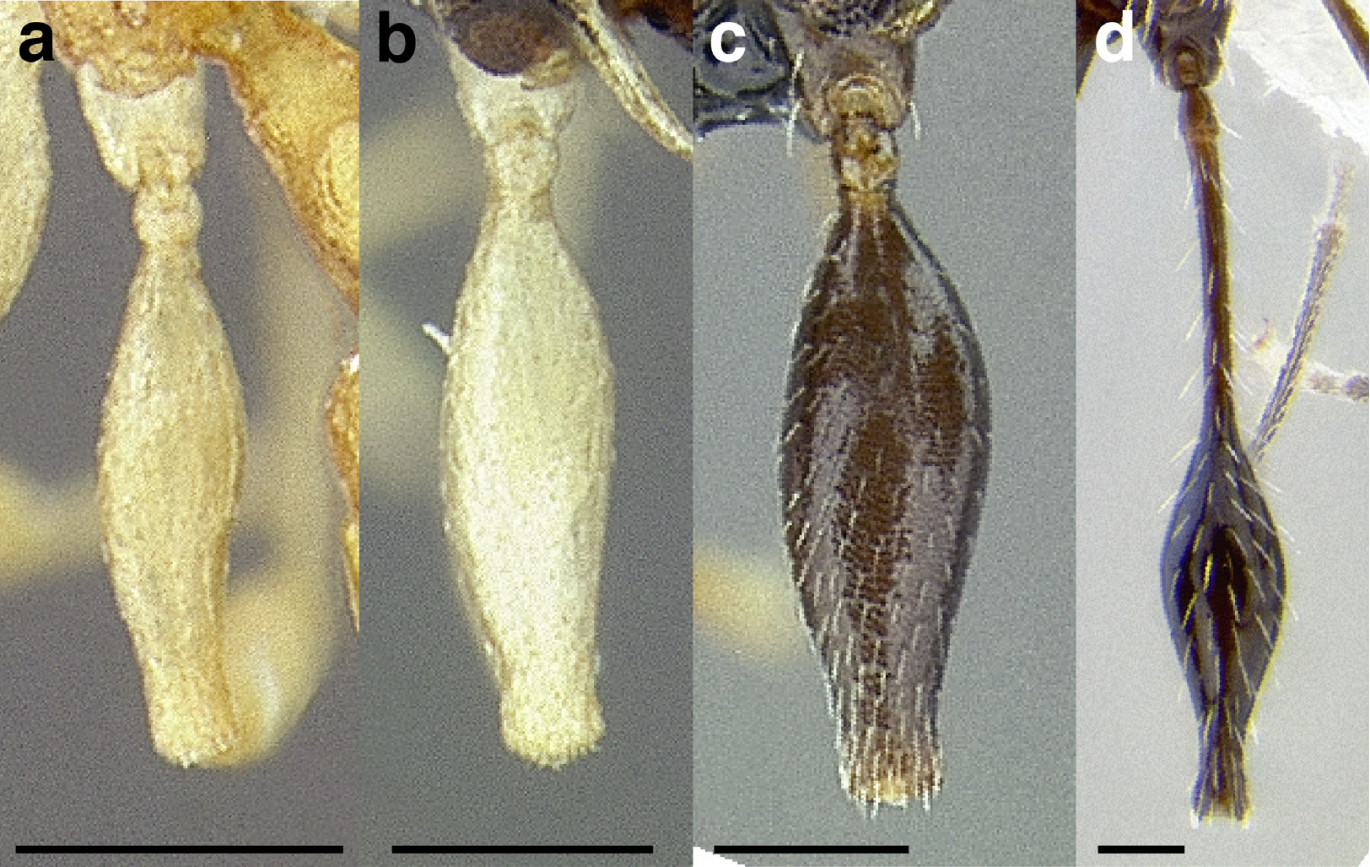
Comparison of *Temnothorax* worker femur morphology. Metafemora in dorsal view. (A) Not incrassate: *T. torrei* (MCZENT00583611). (B) Moderately incrassate: *T. albispinus* worker (CASENT0756097). (C) Strongly incrassate: *T. laticrus* sp. nov. (CASENT0758263). (D) Incrassate femur of the *sallei* clade: *T. poeyi* (CASENT0106241; photo: Michael Branstetter, from www.antweb.org). Scale bars 0.2 mm.

A couple of interesting characteristics of the *Macromischa* syndrome are found in members of the *salvini* clade: iridescent integument and incrassate femora. In the *salvini* clade, both of these characters do not reach the extremes that are attained in the *sallei* clade, but they are still notable within *Temnothorax*. While the members of the *sallei* clade often display metallic greens, blues, and purples which extend over all surfaces of the body, the members of the *salvini* clade usually have spectral iridescence (rainbow hues that are visible only under strong direct light) confined to the gaster (Fig. 3). The legs of *salvini* clade members can become extremely incrassate in some species (see *Temnothorax laticrus* sp. nov., Fig. 4C), but they never attain the spindle shaped femora seen in the *sallei* clade (Fig. 4D).

**Figure 5:**
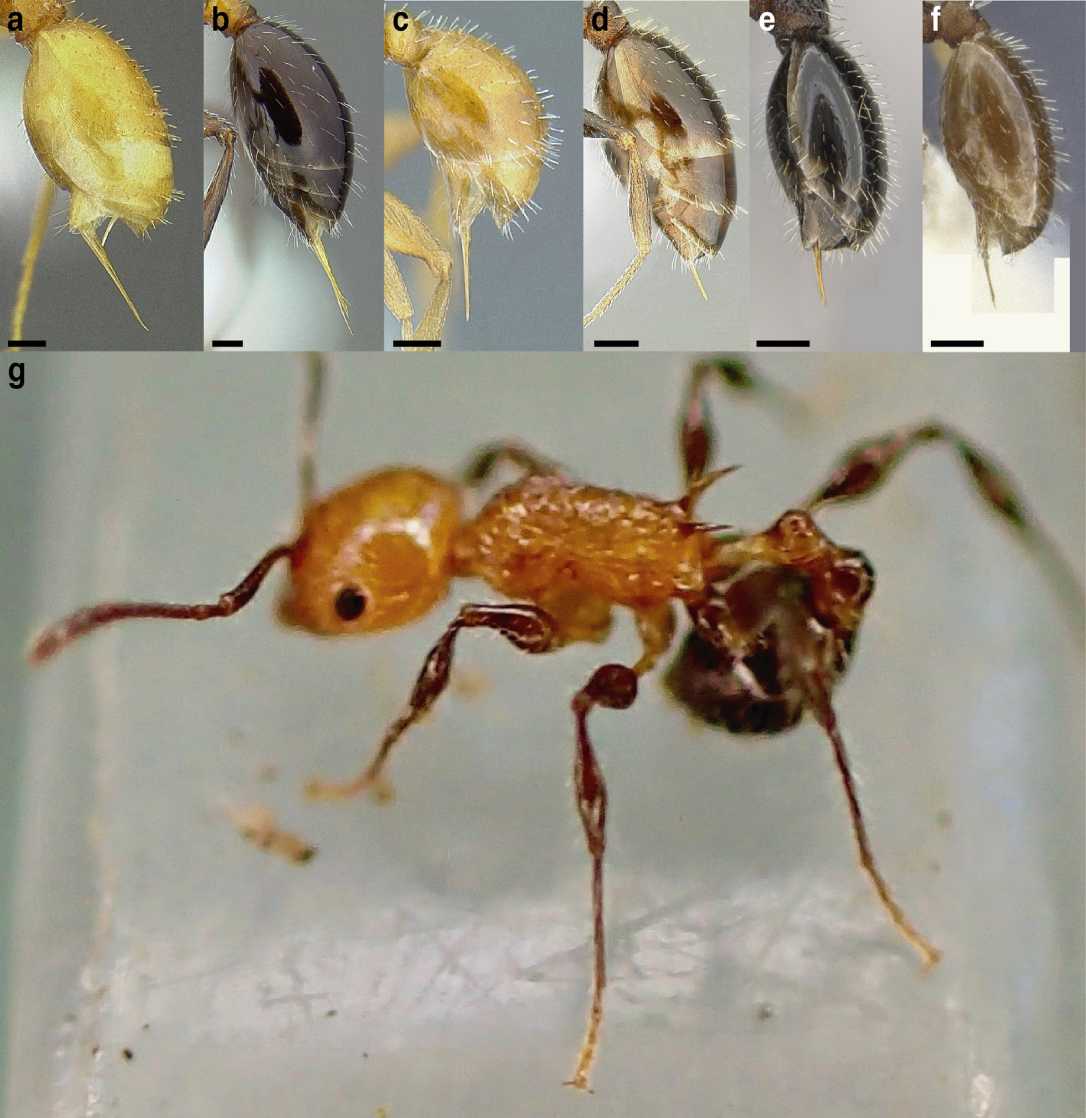
Comparison of stinging apparati and *Macromischa* stinging posture of *Temnothorax* workers. (A–F) Profile view of gasters (A) *T. obtusigaster* sp. nov. (CASENT0758654). (B) *T. quetzal* sp. nov. (CASENT0614495). (C) *T. subditivus* (CASENT0619368). (D) *T. nevadensis* (CASENT0221903; photo: Xiaofan Yang from www.antweb.org) (E) *T. texanus* (CASENT0923379; photo: Michele Esposito from www.antweb.org) (F) *T. michali* (CASENT0916698; photo: Flavia Esteves, from www.antweb.org) (G) profile view of an undescribed species of the *sallei* clade in the typical *Macromischa* stinging posture, Dominican Republic. Scale bars 0.2 mm.

The sting of many *salvini* clade members, especially the arboreally nesting species, is large in comparison to many of their congeners (compare sting of *salvini* clade workers in Figs. 5A–5C to the sting of workers from the *andrei* clade, Fig. 5D; *sallei* clade, Fig. 5E; Palearctic clade, Fig. 5F). Few observations of their behavior are available in the literature, but Wheeler (1903b) described *T. pergandei* as being particularly hostile toward workers from competing conspecific colonies; the sting may be an adaptation for defense against other arthropods. The stinging posture taken by some *salvini* clade workers is distinctive as well, with the gaster flexed under the mesosoma so that the sting in oriented anteriorly, instead of stinging straight down like the majority of *Temnothorax* species. This posture is identical to another lineage with the *Macromischa* syndrome in the Greater Antilles (the *purpuratus* species group in the *sallei* clade; Fig. 5G), which has apparently attained the same stinging posture by evolutionary convergence (Prebus pers. obs., but also see Wheeler & Mann, 1914).

## Materials & Methods

The electronic version of this article in Portable Document Format (PDF) will represent a published work according to the International Commission on Zoological Nomenclature (ICZN), and hence the new names contained in the electronic version are effectively published under that Code from the electronic edition alone. This published work and the nomenclatural acts it contains have been registered in ZooBank, the online registration system for the ICZN. The ZooBank LSIDs (Life Science Identifiers) can be resolved and the associated information viewed through any standard web browser by appending the LSID to the prefix http://zoobank.org/. The LSID for this publication is: urn:lsid:zoobank.org:pub:F8C827C6-7475-4AF0-B67E-E50786131273. The online version of this work is archived and available from the following digital repositories: PeerJ, PubMed Central and CLOCKSS.

In addition to historical collections, this study involved collection of specimens from several recent field trips. Field studies were approved by the Secretaria de Medio Ambiente y Recursos Naturales (Mexico; license numbers FAUT-0018 & FAUT-0280), Natural Resources, Parks and Recreation Department (Arizona, U.S.A.), and the Ministerio de Medio Ambiente y Recursos Naturales (Dominican Republic; license number: VAPB-02742).

### Data management

Collection and specimen data for all material used in this study, along with color images of all type specimens, have been uploaded to AntWeb (www.antweb.org). Data from AntWeb are subsequently shared with the Global Biodiversity Information Facility (GBIF), the Encylopedia of Life (EOL), Wikipedia, AntWiki, and AntMaps. All images in this study were taken or made by the author, unless otherwise noted.

Collection codes are alphanumerical assignments that link specimens to data from a unique collection event. Because multiple species and specimens may be gathered in a single collection event, this is a more general identifier than the unique specimen identifiers used by many museum collections (see below). Collection codes are associated with data on the place, date, collector name, method of collection, habitat, and microhabitat of the collection event. When collection codes were not available on label data, I requested unique “ANTC#” collection codes from AntWeb, then physically associated them with the specimens examined by attaching them to the pin on which the specimens are mounted.

Unique specimen identifiers (USIs) are physically associated with all specimens examined in this study, attached to the pin on which the specimens are mounted. When possible, a single USI was used for each individual specimen examined, but many of the specimens borrowed from museum collections had multiple specimens on one pin already associated with a USI. In these cases, rather than assign USIs to each specimen, I describe the position on the pin in combination with the USI (e.g. CASENT0123456 top, CASENT0123456 middle, CASENT0123456 bottom).

Specimen and collection data were transcribed from label data into a specimen database for standardization. Therefore, the details for each specimen in the taxon treatments are not always verbatim label data and may not match the labels exactly. Distances and elevations are stated in metric units and coordinates are provided in decimal degrees. In cases where coordinates were not given on the specimen labels, they were estimated from label data with Google Earth, with an arbitrary standard error noted in the ‘LatLonMaxError’ and ‘LocalityNotes’ columns of the collection information spreadsheet in Supplemental File 1. Data for material examined follows the following standard format: *[type status, if applicable]:* [Country]: [primary administrative district]: [secondary administrative district, if applicable]: [locality], [geographical coordinates in decimal degrees ± error radius], [elevation in meters], [collection date, with range if applicable], [collector name & collection code], [habitat], [microhabitat], [number and caste of specimens on pin] (unique specimen identifier) [specimen repository]. In order to be consistent with Prebus (2021), I discretized elevation into low (<1,000 m), mid (1,000–1,800 m), and high (>1,800 m) when describing elevational range.

Maps for each taxon treatment were generated using a custom R script (R Core Team, 2019; Hijmans, 2016; Becker & Wilks, 2017; Bivand & Lewin-Koh, 2017; Bivand & Rundel, 2018; Bivand, Keitt & Rowlingson, 2020; Wickham, 2016a, 2016b) and base maps from Natural Earth. Please find the script with example files on the Dryad Digital Repository (https://doi.org/10.25338/B87W6W).

### Specimen repositories

**ABS** Archbold Biological Station, Lake Placid, Florida, U.S.A.

**AMNH** American Museum of Natural History, New York, New York, U.S.A.

**BEBC** Brendon E. Boudinot collection. Friedrich Schiller University, Jena, Germany

**BMNH** The Natural History Museum, London, U.K.

**CASC** California Academy of Sciences, San Francisco, California, U.S.A.

**JTLC** John T. Longino collection, University of Utah, Salt Lake City, Utah, U.S.A.

**LACM** Los Angeles County Museum of Natural History, Los Angeles, California, U.S.A.

**ECOSCE** Colección Entomológica de El Colegio de la Frontera Sur, Unidad San Cristóbal, Chiapas, Mexico.

**FSCA** Florida State Collection of Arthropods, Gainesville, Florida, U.S.A.

**FMNH** Field Museum of Natural History, Chicago, Illinois, U.S.A.

**IEGG** Instituto di Entomologia "Guido Grandi", Università di Bologna, Bologna, Italy.

**INbio** Instituto Nacional de Biodiversidad, Santo Domingo de Heredia, Costa Rica.

**INHS** Illinois Natural History Survey, Champaign, Illinois, U.S.A.

**KWGC** Kyle W. Gray collection. Arizona State University, Tempe, Arizona, U.S.A.

**MCZC** Museum of Comparative Zoology, Harvard University, Cambridge, Massachusetts, U.S.A.

**MEM** Mississippi Entomological Museum, Mississippi State University, Mississippi, U.S.A.

**MHNG** Muséum d’Histoire Naturelle, Geneva, Switzerland.

**MMPC** Matthew M. Prebus collection, Arizona State University, Tempe, Arizona, U.S.A.

**MNHN** Muséum National d’Histoire Naturelle, Paris, France.

**MNHNSD** Museo Nacional de Historia Natural “Prof. Eugenio de Jesús Marcano”, Santo Domingo, Dominican Republic.

**MSNG** Museo Civico di Storia Naturale “Giacomo Doria”, Genova, Italy.

**NHMB** Naturhistorisches Museum, Basel, Switzerland.

**PSWC** Philip S. Ward collection, University of California, Davis, California, U.S.A.

**RAJC** Robert A. Johnson collection, Arizona State University, Tempe, Arizona, U.S.A.

**SIBR** Social Insect Biological Repository, Arizona State University, Tempe, Arizona, U.S.A.

**UCDC** R.M. Bohart Museum of Entomology, University of California, Davis, California, U.S.A.

**UNAM** Universidad Nacional Autonoma de Mexico, Mexico D.F., Mexico.

**USNM** National Museum of Natural History, Washington D.C., U.S.A.

**UTEP** University of Texas at El Paso Insect Collection, El Paso, Texas, U.S.A.

**UVGC** Colleción de Artrópodos, Universidad del Valle de Guatemala, Guatemala City, Guatemala.

### Measurements & indices

**Figure 6:**
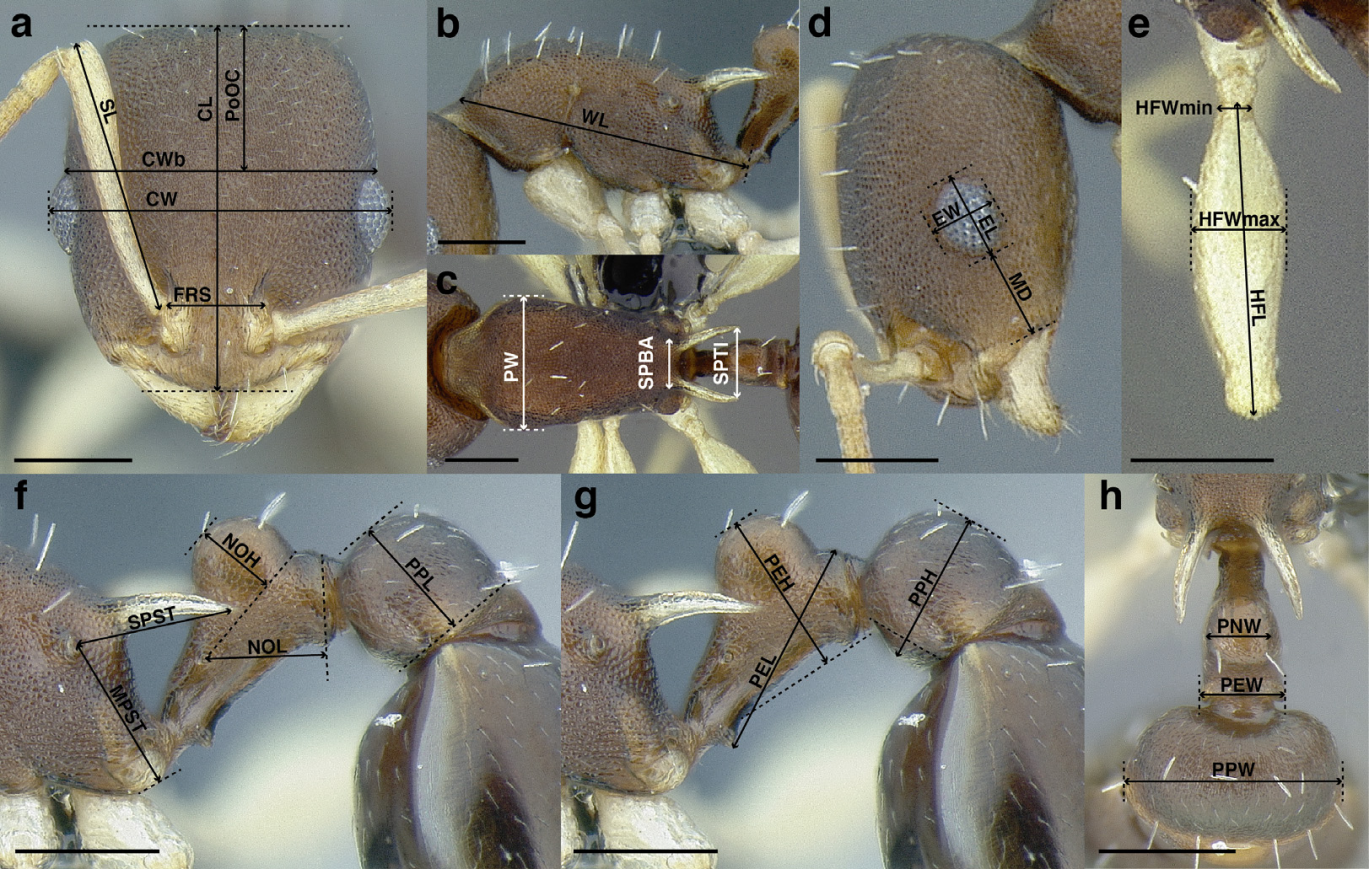
Measurements used in this study, with the worker of *Temnothorax albispinus (*CASENT0756097) as a model. (A) Head in full face view, (B) mesosoma in profile view, (C) mesosoma in dorsal view, (D) head in profile view, (E) metafemur in dorsal view, (F) and (G) propodeum and waist segments in profile view, (H) waist segments in dorsal view. Scale bars 0.2 mm.

This study is based on examination of more than 2,000 workers, 135 gynes, and 47 males, with measurements taken from more than 500 specimens. All measurements and morphological observations in thus study were performed at a maximum magnification of 63× using a Leica MZ12.5 stereomicroscope, a movable stage equipped with orthogonal digital micrometers, and an ocular graticule. The images used in this study were taken at a maximum of 115× with a JVC KY-F75U digital camera mounted on a Leica MZ16A stereomicroscope. Images were z-stacked using the program HeliconFocus v6.7.1. All measurements in this study are expressed in millimeters, up to three decimal places. The measurements in the taxon treatments are presented as ranges, from minimum to maximum, with the arithmetic mean following the range in parentheses. Many of the following morphological measurements are based on Seifert & Csősz (2015) and Csősz & Fisher (2015). See Fig. 6 for an illustration of the measurements listed below. Raw measurement data, as well as collection data for all specimens used in this study can be found in Supplemental File 1.

**SL** Scape length. Maximum scape length excluding the basal neck and the articular condyle (Fig. 6A).

**EL** Maximum diameter of the compound eye (Fig. 6D).

**EW** Minimum diameter of the compound eye (Fig. 6D).

**FRS** Frontal carina distance. Distance between the frontal carinae immediately caudal of the posterior intersection points between frontal carinae and torular lamellae. If these dorsal lamellae do not laterally surpass the frontal carinae, the deepest point of scape corner pits may be taken as the reference line. These pits take up the inner corner of the scape base when the scape is directed fully caudally and produces a dark, triangular shadow in the lateral frontal lobes immediately posterior to the dorsal lamellae of the scape joint capsule (Fig. 6A).

**CW** Maximum width of the head, including the compound eyes (Fig. 6A).

**CWb** Maximum width of head capsule without the compound eyes, measured posterior to the eyes (Fig. 6A).

**CL** Maximum cephalic length. The head must be carefully tilted to the position, providing the true maximum. If excavations of the posterior margin of the head capsule and/or anterior margin of the clypeus are present, then the measurement is taken from an imaginary line that spans the excavations from the posterior- or anterior-most margins (Fig. 6A).

**PoOC** Postocular distance. Adjust the head to the measuring position of CL. Using an ocular graticule, PoOC is the length between posterior margin of the compound eyes and the posterior margin of the head capsule. If the posterior margin of the head capsule is excavated, then the measurement is taken from an imaginary line that spans the excavation from the posterior-most margins (Fig. 6A).

**MD** Malar distance. Minimum distance from the anterior margin of the compound eye to where the mandible articulates with the head capsule (Fig. 6D).

**WL** Weber’s length. Distance between the caudalmost point of propodeal lobe to the inflection point between the pronotal neck and the pronotal declivity (Fig. 6B).

**SPST** Propodeal spine length. Distance between the center of the propodeal spiracle and tip of the propodeal spine (Fig. 6F).

**MPST** Maximum distance from the center of the propodeal spiracle to the posteroventral corner of the ventrolateral margin of the metapleuron (Fig. 6F).

**PEL** Petiole length. Diagonal petiolar length in lateral view; measured from the apex of the subpetiolar process to the posterodorsal corner of caudal cylinder (Fig. 6G).

**NOL** Petiolar node length. Measured in lateral view from the center of the petiolar spiracle to an imaginary line that joins the posterodorsal and posteroventral corners of the caudal cylinder (Fig. 6F).

**NOH** Petiolar node height. Maximum height of the petiolar node, measured in lateral view from the uppermost point of the petiolar node perpendicular to a reference line set from the petiolar spiracle to the imaginary midpoint of the transition between the dorso-caudal slope of the node and dorsal profile of caudal cylinder of the petiole (Fig. 6F).

**PPL** Postpetiole length. The longest distance, perpendicular to the posterior margin of the postpetiole, between the posterior postpetiolar margin and the anterior postpetiolar margin, excluding the helcium (Fig. 6F).

**PEH** Petiole height. The longest distance measured from the ventral petiolar profile at node level (perpendicular to the chord length of the petiolar sternum, excluding the subpetiolar process) to the distalmost point of the dorsal profile of the petiolar node (Fig. 6G).

**PPH** Maximum height of the postpetiole in lateral view measured perpendicularly to a line defined by the linear section of the segment border between postpetiolar tergite and sternite (Fig. 6G).

**PW** Pronotum width. Maximum width of the pronotum in dorsal view (Fig. 6C).

**SBPA** Minimum propodeal spine distance. The smallest distance of the lateral margins of the propodeal spines at their base. This should be measured in antero-dorsal view: the wider parts of the ventral propodeum do not interfere with the measurement in this position. If the lateral margins of propodeal spines diverge continuously from the tip to the base, a smallest distance at base is not defined. In this case, SPBA is measured at the level of the bottom of the interspinal meniscus (Fig. 6C).

**SPTI** Apical propodeal spine distance. The distance of propodeal spine tips in dorsal view; if spine tips are rounded or truncated, the centers of spine tips are taken as reference points (Fig. 6C).

**PNW** Maximum width of petiolar node in dorsal view (Fig. 6H).

**PEW** Maximum width of petiole in dorsal view, measured across the widest point of the caudal cylinder (Fig. 6H).

**PPW** Postpetiole width. Maximum width of postpetiole in dorsal view (Fig. 6H).

**HFL** Hind femur length. Maximum length of the metafemur in dorsal view (Fig. 6E).

**HFWmax** Maximum metafemur width in dorsal view (Fig. 6E).

**HFWmin** Minimum metafemur width in dorsal view, taken at the point of articulation with the trochanter (Fig. 6E).

**CS** Absolute cephalic size. The arithmetic mean of CL and CWb.

**ES** Absolute eye size. The arithmetic mean of EL and EW.

**SI** Scape index: SL/CWb × 100.

**OI** Ocular index: ES/CS × 100.

**CI** Cephalic length index: CWb/CL × 100.

**WLI** Weber’s length index: WL/CWb × 100.

**SBI** Propodeal spine base index: SBPA/CWb × 100.

**PSI** Propodeal spine length index: SPST/WL × 100.

**PWI** Postpetiole width index: PPW/PEW × 100.

**PLI** Petiole length index: PEL/PPL × 100.

**NI** Petiolar node shape index: NOL/NOH × 100.

**PNWI** Petiolar node width index: PNW/PEW × 100.

**NLI** Petiolar node length index: NOL/PEL × 100.

**FI** Metafemur width index: HFWmax/HFWmin × 100.

### Morphological analysis

The workers of the *salvini* clade, in contrast to most species of *Temnothorax*, contain a relative wealth of characters with which to determine species. Still, many species appear superficially similar due either to evolutionary convergence or symplesiomorphy, depending on the case. A majority of species in the *salvini* clade have features of the *Macromischa* syndrome: pedunculate petiole, campaniform postpetiole, arched mesosoma, incrassate femora, and iridescent coloration. However, many species in the *salvini* clade have only one of the features listed above, and some have none of them. The following is a list of characters that are used to distinguish species in this study, with details of their variability in the *salvini* clade.

**Figure 7:**
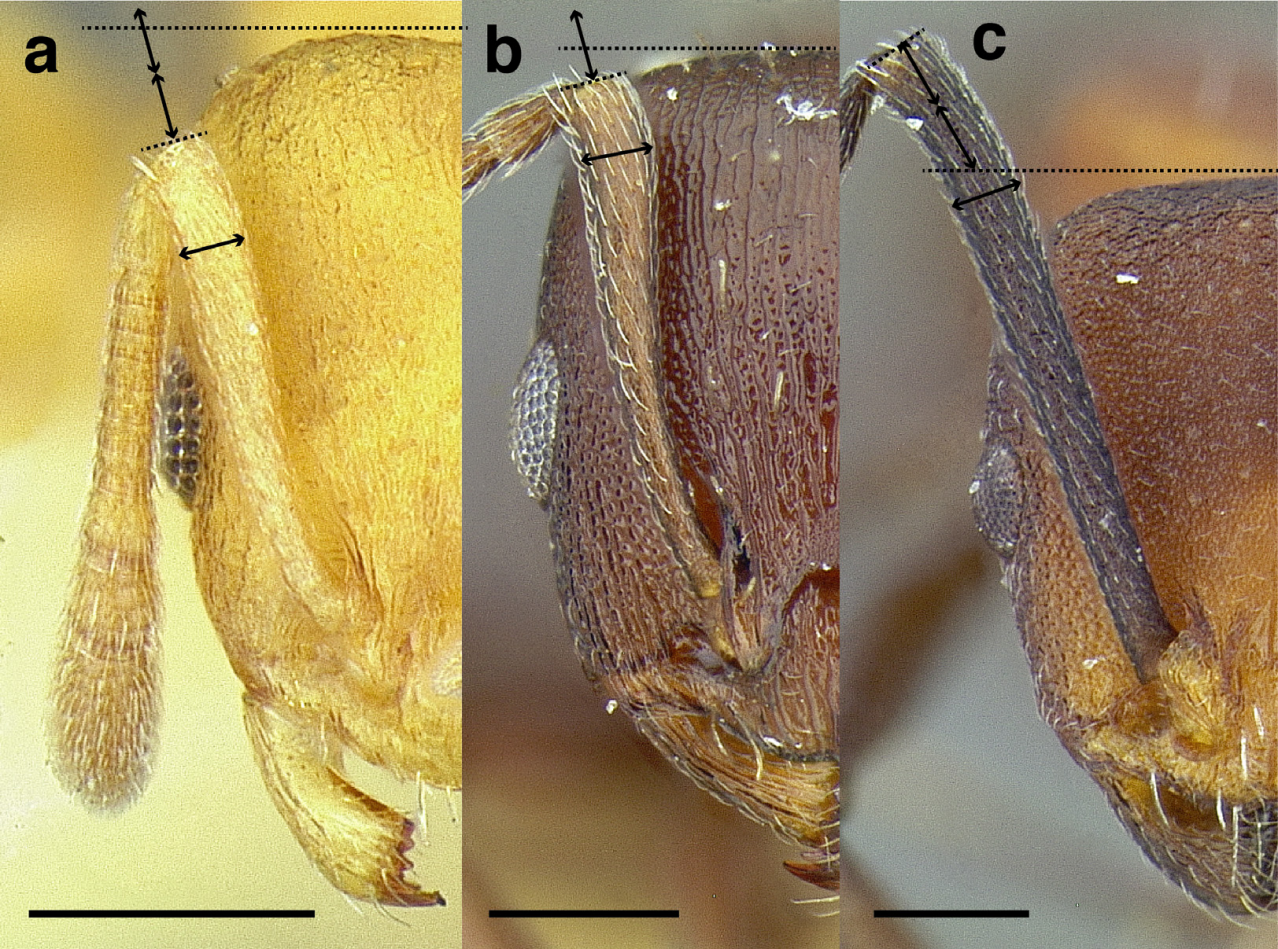
Comparison of *Temnothorax* worker scape length with head in full-face view. (A) Short: *T. terrigena* (MCZENT00522078). (B) Moderately long: *T. fuscatus* (USNMENT00921897). (C) Long: *T. pastinifer* (CASENT0758861). Scale bars 0.2 mm.

***Antennae*.** The antennae of *salvini* clade are highly variable in their length relative to head length among species. In the taxon treatments below, I have arbitrarily discretized antennal scape length into three categories:

*Short*: when fully retracted, the scape fails to reach the posterior margin of the head capsule by the maximum width of the antennal scape (Fig. 7A);

*Moderately long*: the scape fails to reach the posterior margin of the head capsule by less than the width of the antennal scape, or exceeds it by up to the width of the antennal scape (Fig. 7B);

*Very long*: exceeds it by more than the maximum width of the antennal scape (Fig. 7C).

These observations roughly translate to SI < 95, SI 95–110, and SI > 110. See Fig. 7 for a comparison of scape lengths.

***Head shape*.** Head shape is a moderately variable character in the *salvini* clade, but it can be used to separate some species. It varies from being relatively elongate in *Temnothorax terrigena* (Wheeler) (CI 76–82) to nearly square in some specimens of *T. longinoi* sp. nov. (CI 84–94). As a general rule, if head shape is used to distinguish species, the differences in CI are very slight; careful measurements are required. In some closely related species, the posterior margin of the head capsule is concave, especially in the *rugosus* and *annexus* species groups.

**Figure 8:**
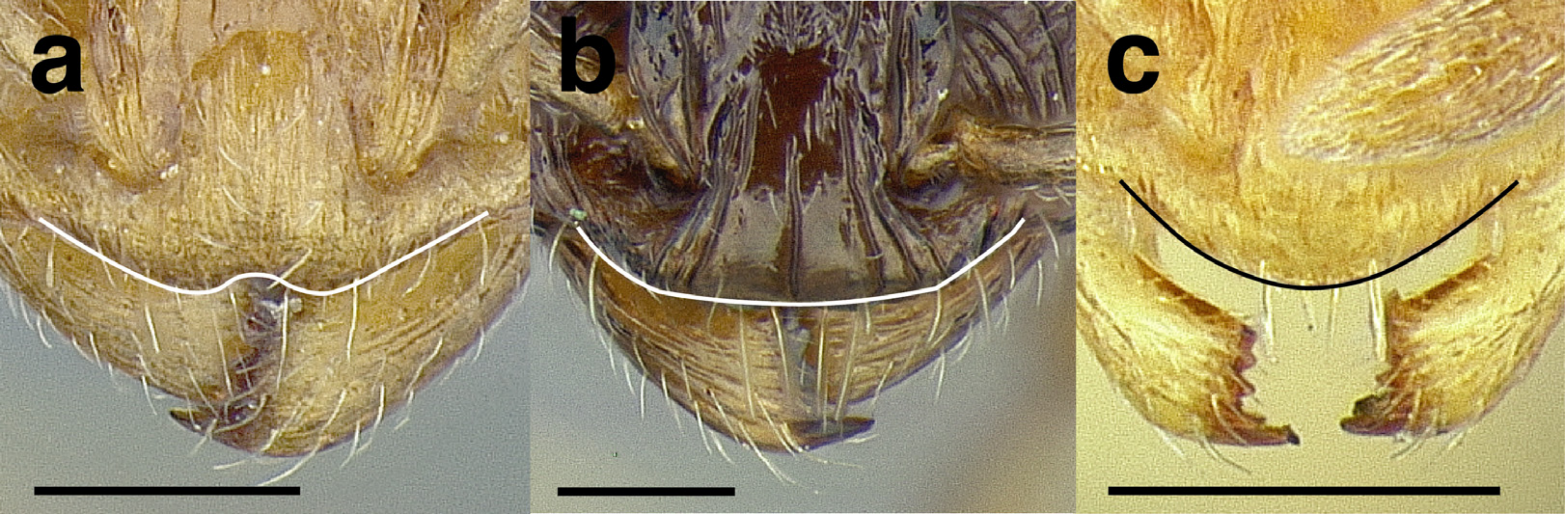
Comparison of *Temnothorax* worker clypeal shapes with head in full-face view. (A) Emarginate: *T. quercicola* sp. nov. (LACMENT323216). (B) Flat: *T. politus* worker (CASENT0756845). (C) Convex: *T. terrigena* (MCZENT00522078). Scale bars 0.2 mm.

***Clypeus*.** The shape of the anterior margin of the clypeus is a useful character for distinguishing between closely related species but does not typically delimit species groups. However, it is useful in distinguishing morphologically convergent but distantly related sympatric species from each other, especially in the case of *Temnothorax silvestrii* (Santschi) (*sallei* clade) and *T. quercicola* sp. nov. It ranges from strongly convex to strongly emarginate. Another useful clypeal character is the number and placement of the clypeal carinae, and degree of ground sculpture. See Fig. 8 for a comparison of clypeal shapes.

**Figure 9:**
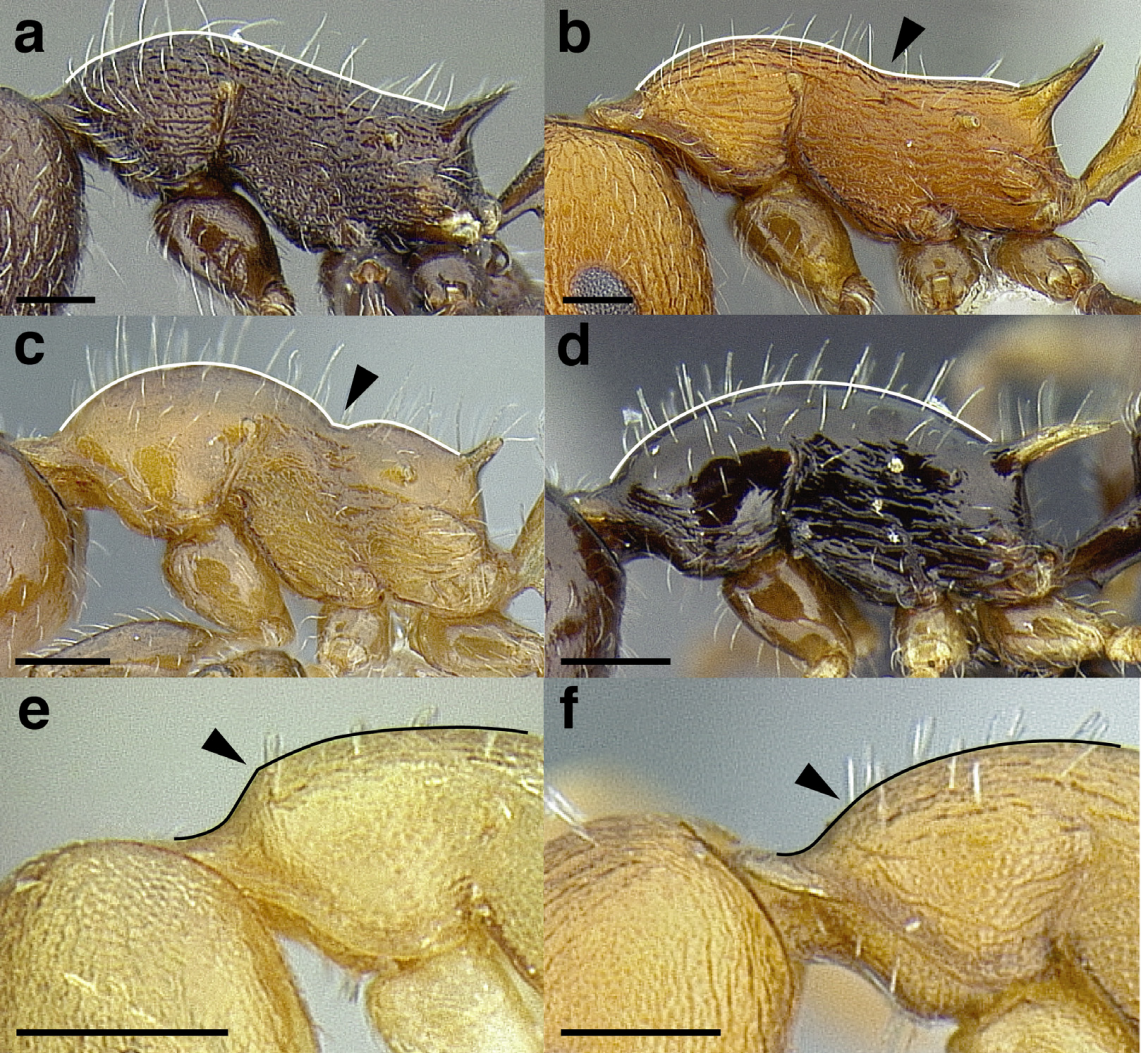
Comparison of worker mesosoma dorsum profiles in the *salvini* clade. (A) Flat: *Temnothorax longinoi* sp. nov. (CASENT0758892). (B) Sinuate: *T. quetzal* sp. nov. (CASENT0614495). (C) Humped: *T. pergandei* (MCZENT00583596). (D) Continuously convex: *T. politus* (CASENT0756845). (E) *T. pilicornis* sp. nov. (CASENT0756186). (F) *T. subditivus* (CASENT0619368). Scale bars 0.2 mm.

***Mesosoma shape*.** The shape of the mesosoma is used to delimit species groups, as well as closely related species in this study. Four types are distinguished here: flat, sinuate, humped, and continuously convex (Figs. 9A–9D). The anterior margin of the pronotum, which I refer to as the “pronotal declivity,” is often helpful in separating taxa; in the *salvini* clade it can grade continuously into the dorsal margin of the pronotum in species with a continuously convex mesosoma, or it passes through an angle as it transitions to the dorsal face (Figs. 9E & 9F ).

***Promesonotal suture*.** The promesonotal suture is always present laterally as an impression that runs from the posterior margin of the procoxal insertion to the mesothoracic spiracle and is often indicated on the dorsum of the mesosoma as an anteriorly bowed line of darker integument, a weak break in the surface sculpture with the same shape, or both. In some species the promesonotal suture is impressed on the dorsum of the mesosoma.

***Metanotal groove*.** The metanotal groove is absent or only weakly indicated in most species of the *salvini* clade, but is present and very obvious in certain species, especially the *pergandei* group. For a comparison, see Figs. 9B & 9C.

***Propodeal spines*.** Propodeal spine length is a fairly stable character within species and is useful in many instances to determine species or species group boundaries. In general, the length is expressed in relation to the length of the propodeal declivity, the most convenient character in close proximity:

*Short*: shorter than the propodeal declivity;

*Moderately long*: as long as the propodeal declivity;

*Very long*: longer than the propodeal declivity.

**Figure 10:**
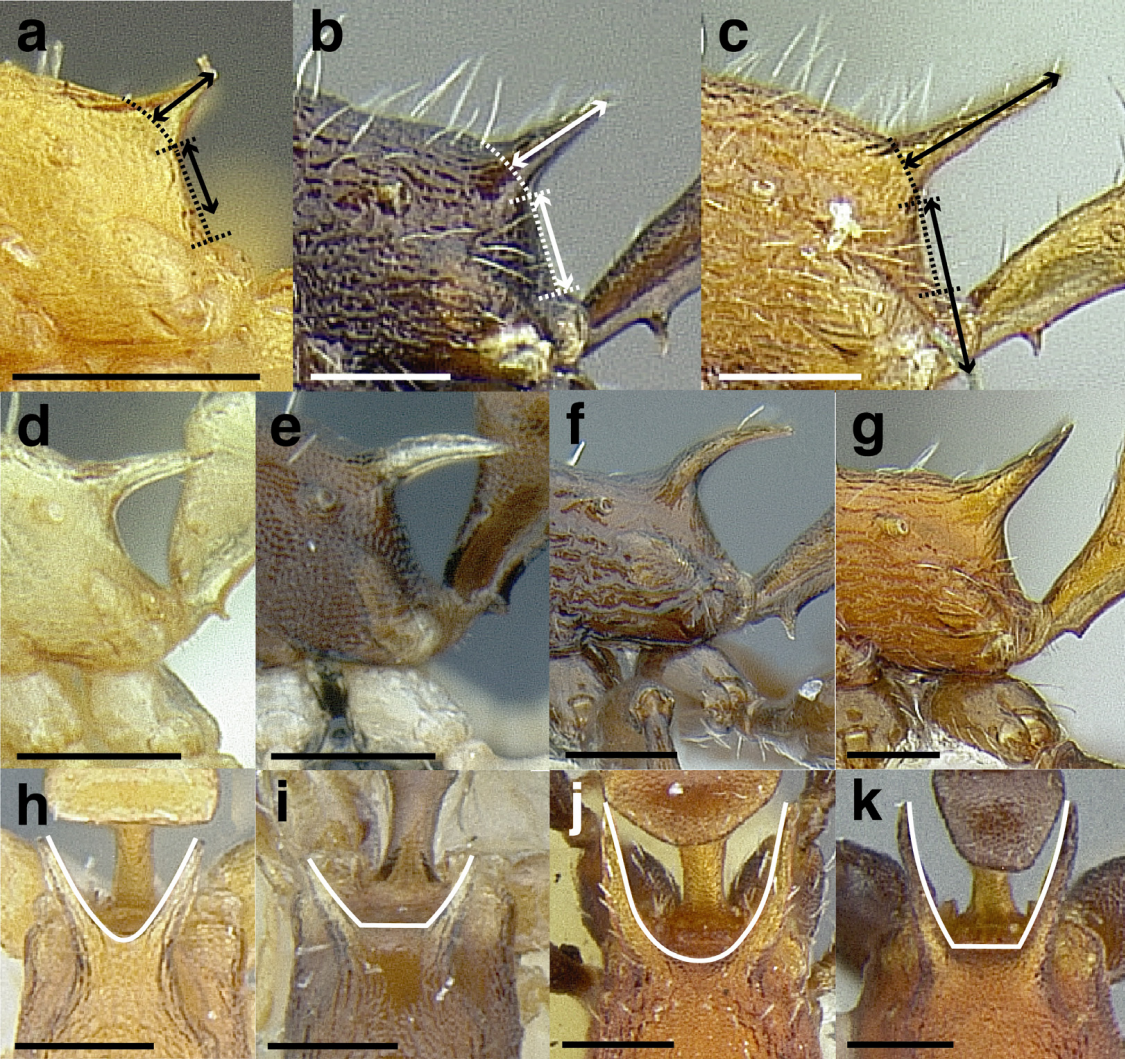
Comparison of worker propodeal spines in the *salvini* clade. (A–G) Profile view. (H–K) Dorsal view. (A) Short propodeal spines: *Temnothorax terrigena* (MCZENT00522078). (B) Moderately long propodeal spines: *T. longinoi* sp. nov. (CASENT0758892). (C) Long propodeal spines: *T. aztecoides* sp. nov. (CASENT0758791). (D) Straight: *T. agavicola* sp. nov. (MCZENT00510559). (E) Slightly downcurved: *T. albispinus* (CASENT0756097). (F) Strongly downcurved: *T. fuscatus* (CASENT0916005). (G) Slightly upcurved: *T. quetzal* sp. nov. (CASENT0614495). (H) “V” shaped: *T. subditivus* (CASENT0619368). (I) basally truncate “V” shaped: *T. subditivus* (MCZENT00016371). (J) “U” shaped: *T. rutabulafer* sp. nov. (CASENT0758266). (K) Basally truncate “U” shaped: *T. pastinifer* (CASENT0758861). Scale bars 0.2 mm.

The length of the propodeal declivity is defined as the vertically oriented portion of the propodeum between the base of the propodeal spine and the dorsalmost part of the propodeal lobe (Figs. 10A–10C). The preceding three categories roughly translate to: PSI < 28, PSI 28–30, and PSI > 30. The angularity of the propodeal spines is employed in some parts of the key and species diagnoses; this is measured as the angle between the intersection of two imaginary lines: PSL and the length of the propodeal declivity. Propodeal spine shape is also used in this study, both in profile and in dorsal view. In profile the propodeal spines are described as straight, slightly downcurved, strongly downcurved, or slightly upcurved (Figs. 10D–10G). In dorsal view, the propodeal spines are described in four ways. They may be: closely approximated basally and constantly diverging apically, so that the negative space between them is “V” shaped; broadly approximated basally and constantly diverging apically, so that the negative space between them is a basally truncated “V”; closely approximated basally and initially diverging before becoming parallel apically, so that the negative space between them is “U” shaped; or, broadly approximated basally and initially diverging before becoming parallel apically, so that the negative space between them is a basally truncated “U” (Figs. 10H–10K).

***Metapleural gland bulla*.** The size of the metapleural gland bulla is useful in some cases for delimiting species, especially in the *pulchellus* group. The size is described in relation to the position of the propodeal spiracle in the key and the diagnoses. I have categorized the sizes of the metapleural gland into three size classes:

*Small*: extending less than two thirds of the way to the propodeal spiracle;

*Moderately large*: extending about two thirds of the way to the propodeal spiracle;

*Very large*: reaching more than two thirds of the way to the propodeal spiracle.

**Figure 11:**
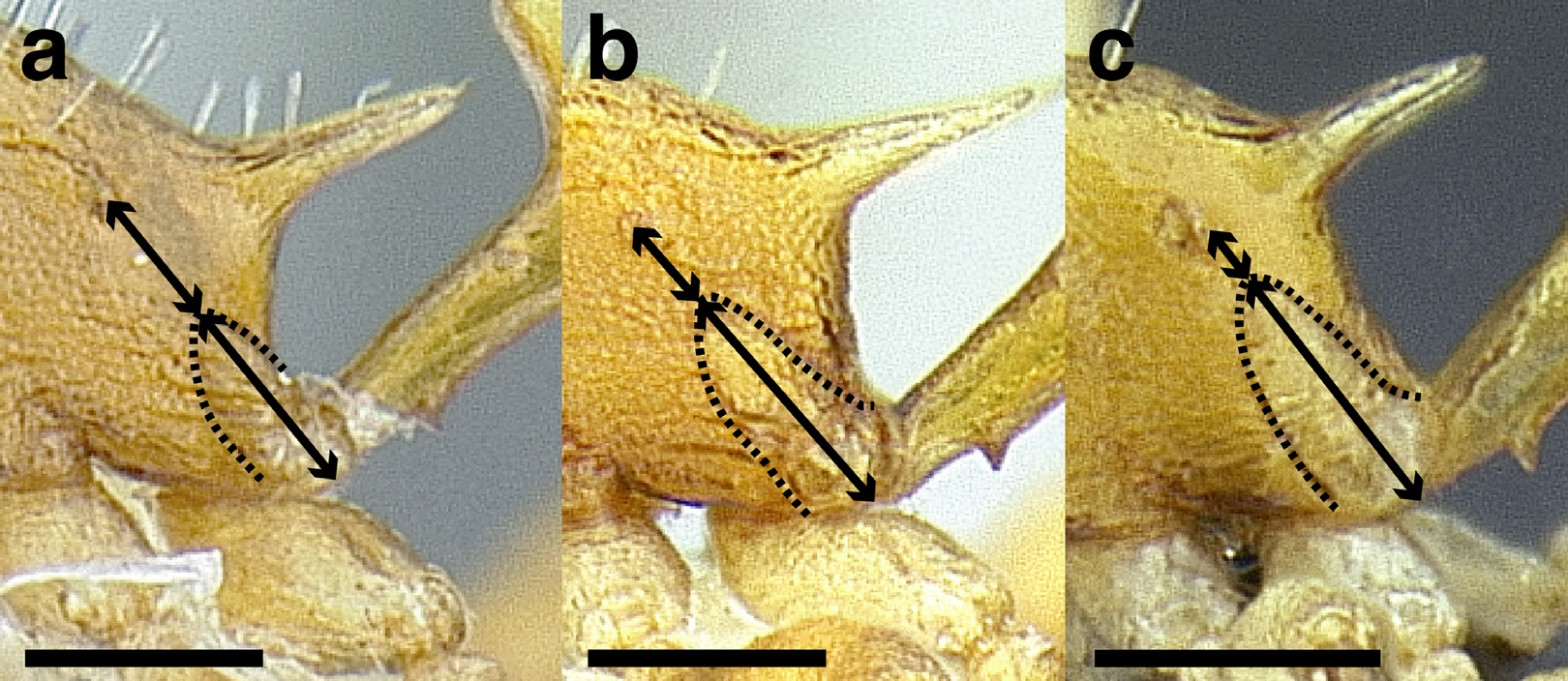
Comparison of *Temnothorax* worker metapleural gland bulla sizes in profile view. (A) Small: *T. subditivus* (CASENT0619368). (B) Moderately large: *T. aureus* sp. nov. (CASENT0619363) (C) Very large: *T. magnabulla* sp. nov. (CASENT0756093). Scale bars 0.2 mm.

See Fig. 11 for a comparison of metapleural gland bulla sizes.

**Figure 12:**
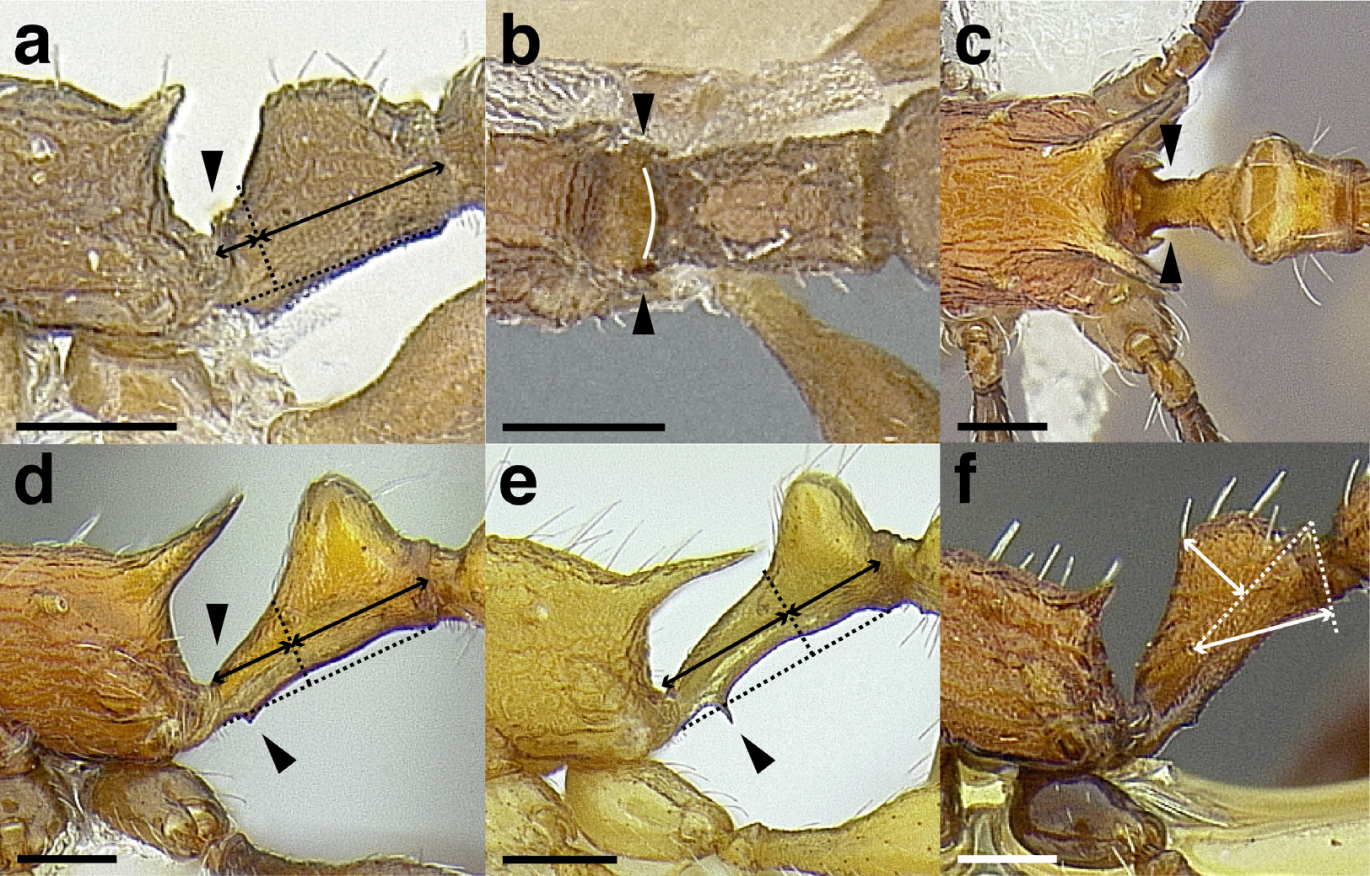
Comparison of *Temnothorax* worker petiole characters. (A) Arrow indicates the anterodorsal tubercle; measurements depict a short petiolar peduncle; profile view of *T. parralensis* sp. nov (LACMENT323355). (B) Arrows indicate the transverse carina uniting the anterodorsal petiolar tubercles; dorsal view of *T. parralensis* sp. nov. (LACMENT323355). (C) Arrows indicate the lack of a transverse carina; dorsal view of *T. quetzal* sp. nov. (CASENT0614495). (D) Arrows indicate the lack of an anterodorsal petiolar tubercle and a dentiform subpetiolar process; measurements depict a moderately long petiolar peduncle; profile view of *T. quetzal* sp. nov. (CASENT0614495). (E) Arrow indicates a spiniform subpetiolar process; measurements depict a long petiolar peduncle; profile view of *T. aztecus* (CASENT0758793). (F) Measurements of NOH and NOL, used to calculate NI, depicting a long petiolar node; profile view of *T. ocarinae* (USNMENT00531636). Scale bars 0.2 mm.

***Petiole*.** The petiole has many diagnostic characters for species determination in the *salvini* clade. Anterodorsally, the petiole may bear a couple of tubercles laterally, which are typically united by a transverse carina; this is especially common in mainland species with short petioles (Figs. 12A–12D). The subpetiolar process comes into play in the *salvini* species group, with a large degree of variability among species; it may be small and dentiform to large and spiniform (Figs. 12D & 12E). The length of the petiolar peduncle is highly variable, especially among species groups; it is described verbally in proportion to the total petiolar length and is categorized into three classes:

*Short*: comprising a third or less of the total petiole length (Fig. 12A);

*Moderately long*: comprising > a third to half the length of the petiole (Fig. 12D);

*Very long*: comprising > half the length of the petiole (Fig. 12E)

The length of the petiolar node is also diagnostic in some cases; I categorize it into two classes:

*Short*: NI < 150;

*Long*: NI > 150 (Fig. 12F).

**Figure 13:**
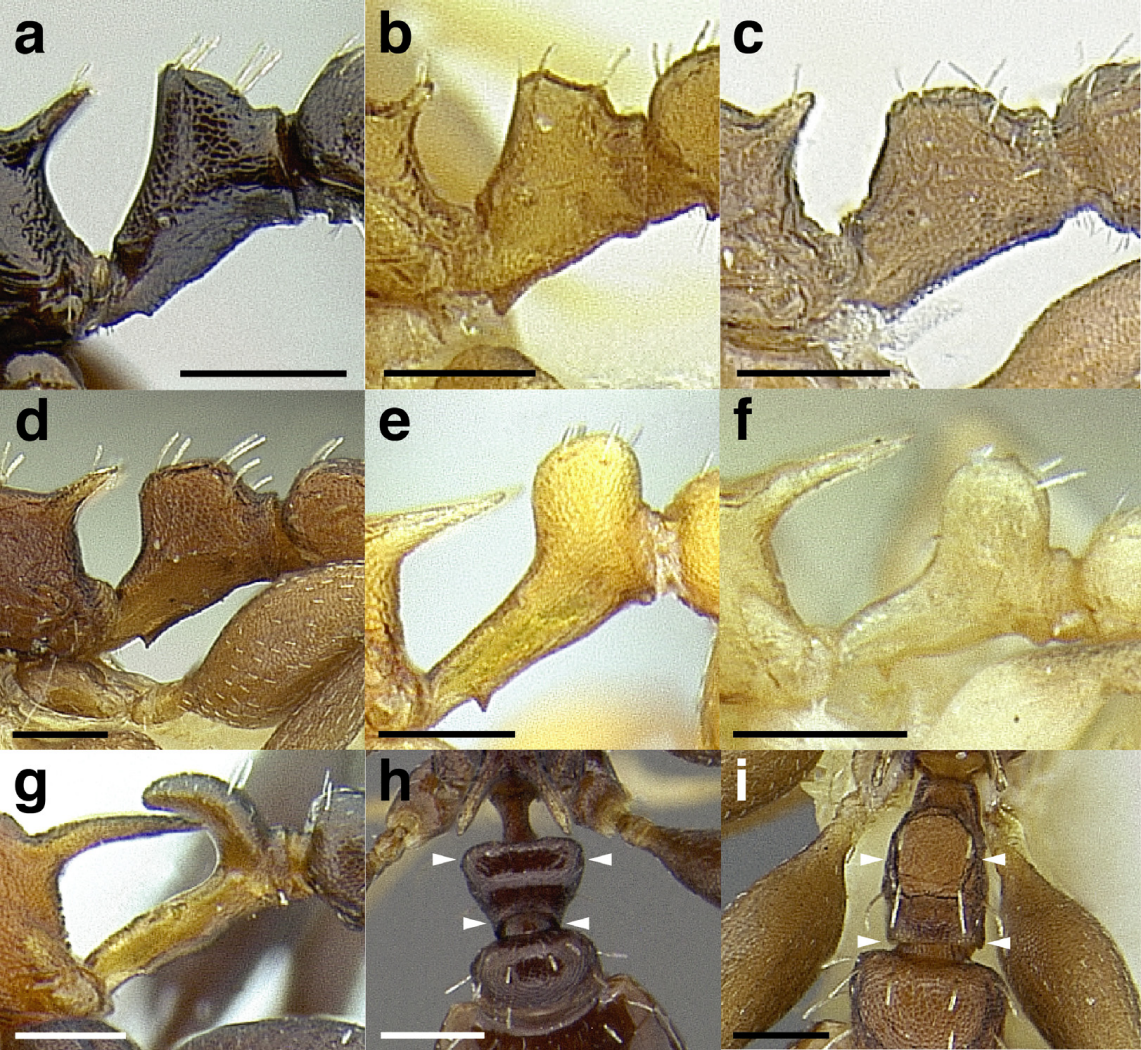
Comparison of *Temnothorax* worker petiolar node morphology. (A–G) Profile view. (H & I) Dorsal view. (A) Cuneiform: *T. acuminatus* sp. nov. (JTLC000007439). (B) Subcuneiform: *T. rugosus* (LACMENT323260). (C) Truncate: *T. parralensis* sp. nov. (LACMENT323355). (D) Subquadrate: *T. balnearius* sp. nov. (LACMENT323199). (E) Posteriorly leaning: *T. aureus* sp. nov. (CASENT0619363). (F) Dorsally rounded: *T. nigricans* (MCZENT00577111). (G) squamiform: *T. pastinifer* (CASENT0758861). (H) Squamiform: *T. subditivus* worker (CASENT0758340). (I) Non-squamiform: *T. balnearius* sp. nov. (LACMENT323199). Scale bars 0.2 mm.

Node shape is highly variable in the *salvini* clade, and is often diagnostic of species groups:

- *cuneiform*: with the node triangular in profile view. This shape is always associated with a short petiolar peduncle, and typically accompanied by a transverse carina on the anterodorsal region of the petiole (Fig. 13A).
- *subcuneiform*: with the node obliquely truncate in profile view. This shape is always associated with a short petiolar peduncle, and typically accompanied by a transverse carina on the anterodorsal region of the petiole. It is easy to see the relationships between cuneiform, subcuneiform, and truncate node shapes thanks to the retention of four dorsal setae among the *rugosus* and *annexus* groups: cuneiform nodes are simply subquadrate nodes with a highly reduced posterior face; the subcuneiform shape is intermediate (Fig. 13B).
- *truncate*: essentially this is a relatively sessile version of a subquadrate petiolar node (Fig. 13C).
- *subquadrate & erect*: with the petiolar node with nearly right angles anterodorsally and posterodorsally (Fig. 13D).
- *posteriorly leaning*: this node shape is common in the *augusti* species group. The node leans slightly posteriorly over the caudal cylinder of the petiole (Fig. 13E).
- *dorsally rounded*: with the anterior face rounding evenly into the posterior face. This node shape is rare in the *salvini* clade (Fig. 13F).
- *squamiform*: with the node antero-posteriorly compressed and dorsally expanded so that in dorsal view it is at least 1.3 times as broad as the caudal cylinder, or PNWI > 130. This node shape is common in the *pastinifer, subditivus*, *salvini, and pergandei* species groups. See Figs. 13G, and 13H & 13I for a comparison in dorsal view of squamiform vs. non-squamiform petiolar nodes.

**Figure 14:**
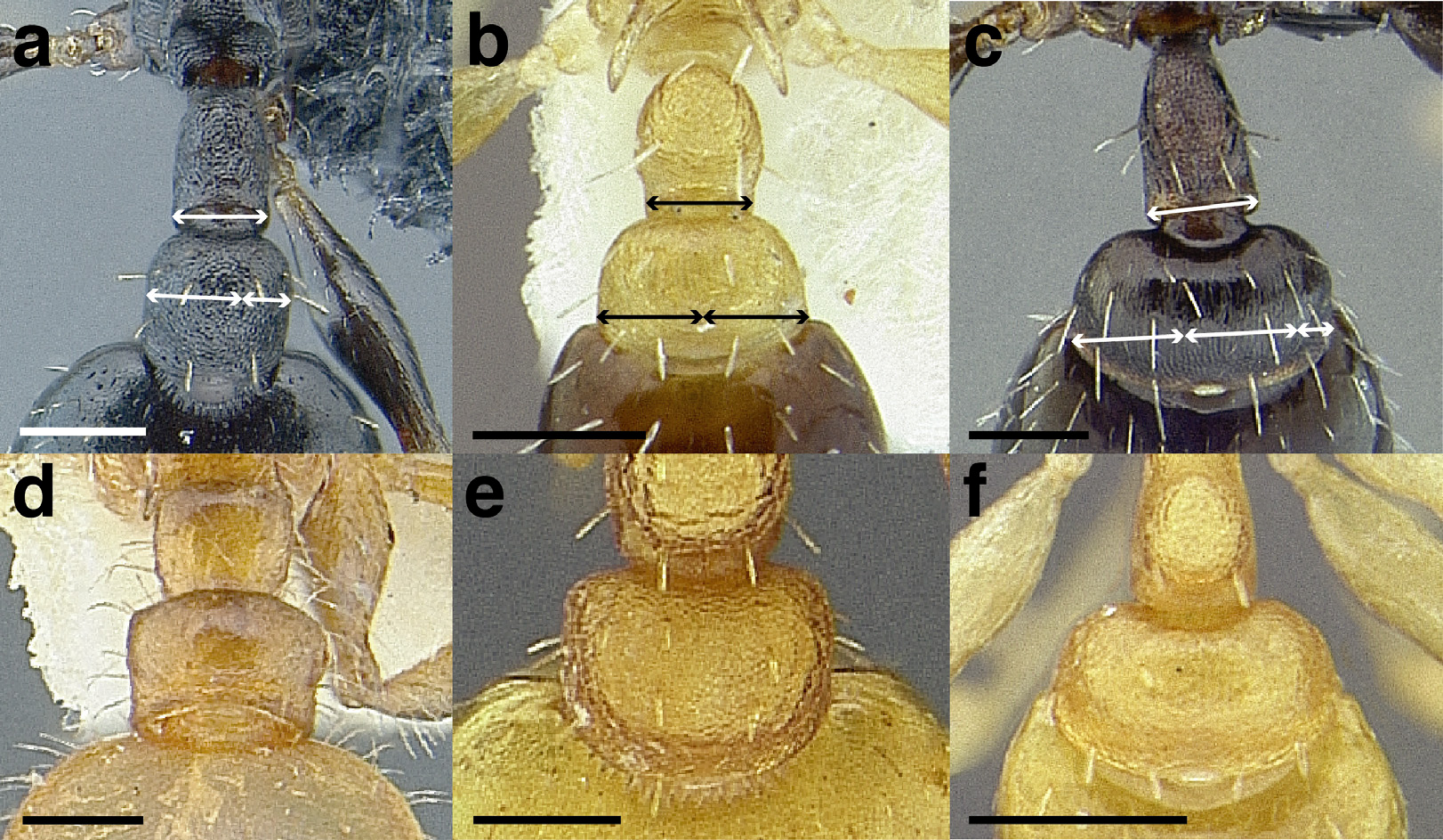
Comparison of *Temnothorax* worker postpetiole morphology in dorsal view. (A) Narrow: *T. tuxtlanus* sp. nov. (CASENT0640472). (B) Moderately broad: *T. huehuetenangoi* (USNMENT00529517). (C) Broad: *T. laticrus* sp. nov. (CASENT0758263). (D) Trapezoidal: *T. pergandei* (USNMENT00921850). (E) Subquadrate: *T. obtusigaster* sp. nov. (CASENT0758654). (F) Campaniform: *T. torrei* (MCZENT00583611). Scale bars 0.2 mm.

***Postpetiole*.** The width of the postpetiole is also quite variable in the *salvini* clade. I have discretized the width into three broad classes:

*Narrow*: with the postpetiole less than twice as wide as the caudal cylinder of the petiole in dorsal view, or PWI < 200 (Fig. 14A);

*Moderately broad*: with the postpetiole twice as broad to 2.2 times as broad as the caudal cylinder, or PWI 200–220 (Fig. 14B);

*Very broad*: PWI > 220 (Fig. 14C).

The shape of the postpetiole in dorsal view is also used in some cases. The shapes are classified into three forms:

- *trapezoidal*: with the postpetiole widest anteriorly and the anterior corners angulate (Fig. 14D)
- *subquadrate*: with the sides of the postpetiole parallel, but anterior corners angulate (Fig. 14E)
- *campaniform*: with the anterior face rounding evenly into the lateral faces, which evenly diverge from each other to the junction with the gaster (Fig. 14F).

***Legs*.** The degree of metafemur incrassation is used in some cases to determine species boundaries. I have discretized FI into three categories (Fig. 4):

*Not incrassate*: FI < 250;

*Moderately incrassate*: FI 250–300;

*Strongly incrassate*: FI > 300.

**Figure 15:**
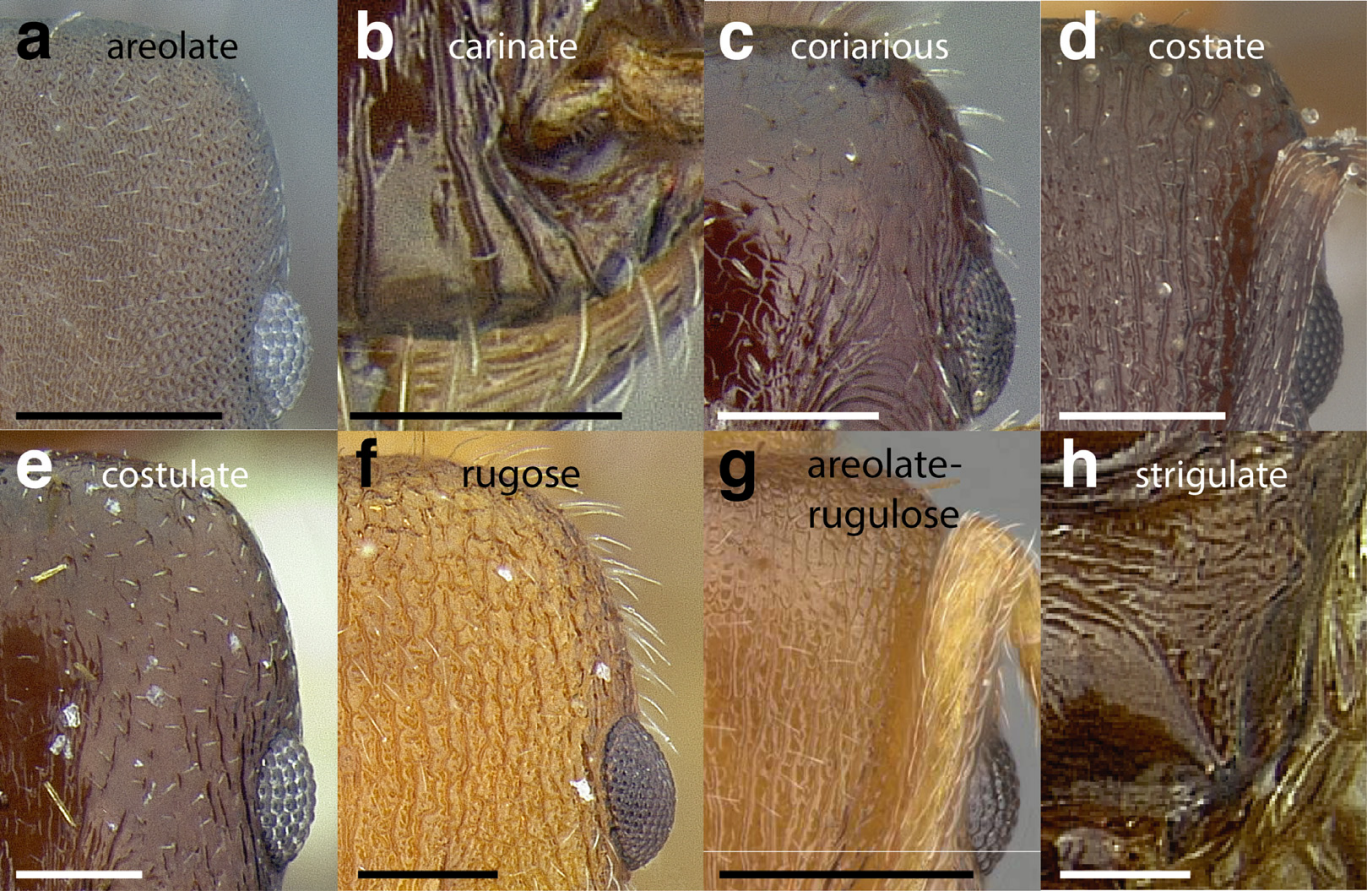
Illustrations of integument sculpture terminology. (A–G) Heads in full-face view (A) areolate; *Temnothorax albispinus* (LACMENT323245) (B) carinate (detail of clypeus); *T. politus* (CASENT0756845) (C) coriarious; *T. pergandei* (CASENT0758291) (D) costate; *T. josephi* (CASENT0102837; photo: Jen Fogarty, from www.antweb.org) (E) costulate; *T. tenuisculptus* (MCZENT00032435) (F) rugose; *T. aztecoides* sp. nov. (CASENT0758791) (G) areolate-rugulose; *T. ambiguus* (CASENT0915985; photo: Michele Esposito, from www.antweb.org) (H) strigulate; detail of propodeum in dorsal view of *T. balnearius* sp. nov. gyne (LACMENT323199). Scale bars 0.2 mm.

***Sculpture*.** In this study, I adhere to the terminology proposed by Harris (1979). The sculpture of *Temnothorax* workers can become very complex but can usually be resolved into a “ground” layer of microsculpture (areolate, rugulose, etc.) and a top layer of “overlying” sculpture which is coarser (rugose, costate, etc.). I use the following terminology in this study:

- *areolate:* divided into a number of small, irregular spaces (areolae) (Fig. 15A).
- *carinate:* keeled; having keels or carinae; with one, or several, but usually few longitudinal narrow raised ridges (Fig. 15B).
- *coriarious:* leather-like in sculpture; with minute cracks like the human skin (Fig. 15C).
- *costate:* furnished with longitudinal raised ribs or ridges (costae), much coarser than carinate (Fig. 15D).
- *costulate:* with less prominent ribs or ridges than costate (composed of costulae) (Fig. 15E).
- *reticulate*: superficially net-like or made up of a network of lines; meshed; netted.
- *rugose:* wrinkled (composed of rugae) (Fig. 15F).
- *rugulose:* minutely wrinkled (composed of rugulae) (Fig. 15G).
- *strigate:* having narrow, transverse lines or streaks, either raised or impressed; composed of fine, short lines.
- *strigulate:* finely or minutely strigate; with numerous short and fine transverse lines (Fig. 15H).

Often, the sculpture layers are composites of the defined sculpture types. I use conjugated descriptors in an effort to capture these nuances (e.g., areolate-rugulose, areolate-costulate).

***Pilosity*.** I use the terminology proposed by Wilson (1955) to describe the angle at which setae meet the body. The setae of *Temnothorax* are typically short and stout, at times nearly clavate; however, several species groups in the *salvini* clade bear long, tapering setae. For example, the *salvini* group, the *pergandei* group, and *T. politus* (Smith) of the *subditivus* group bear this type of setae.

**Figure 16:**
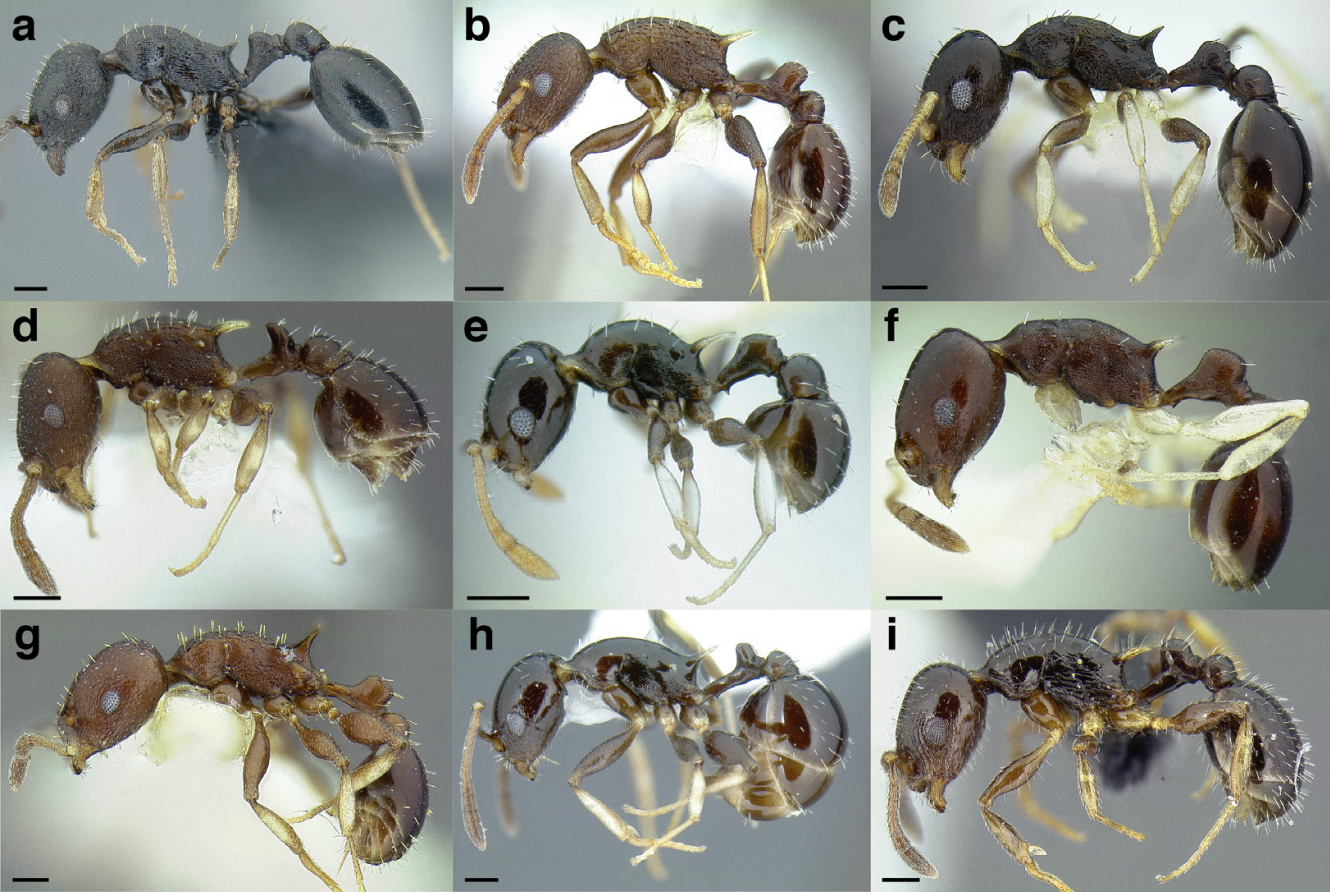
Dark body with light legs, a common theme throughout *salvini* clade workers, profile view. (A) *Temnothorax tuxtlanus* sp. nov. (CASENT0640472). (B) *T. leucacanthoides* sp. nov. (CASENT0756102). (C) *T. xincai* sp. nov. (CASENT0632983). (D) *T. androsanus*, dark form (CASENT0758350). (E) *T. ciferrii* (MCZENT00510533). (F) *T. hippolytus* sp. nov. (LACMENT323469). (G) *T. tenuisculptus* (MCZENT00032435). (H) *T. subditivus* (CASENT0758299). (I) *T. politus* (CASENT0756845). Scale bars 0.2 mm.

***Color*.** Many species in the *salvini* clade have more than one color form, so color is used sparingly as a diagnostic feature. For example, *Temnothorax pergandei, T. fuscatus, T. subditivus*, and *T. albispinus* (Wheeler) all have dark and light color variants, while *T. pergandei* and *T. longinoi* sp. nov. have additional bicolored variants; the taxon treatments include images of the different color forms. A common morphological theme across the species groups of the *salvini* clade are workers with nearly uniformly dark integument, with some or all of the leg segments lightly colored (Fig. 16).

**Figure 17:**
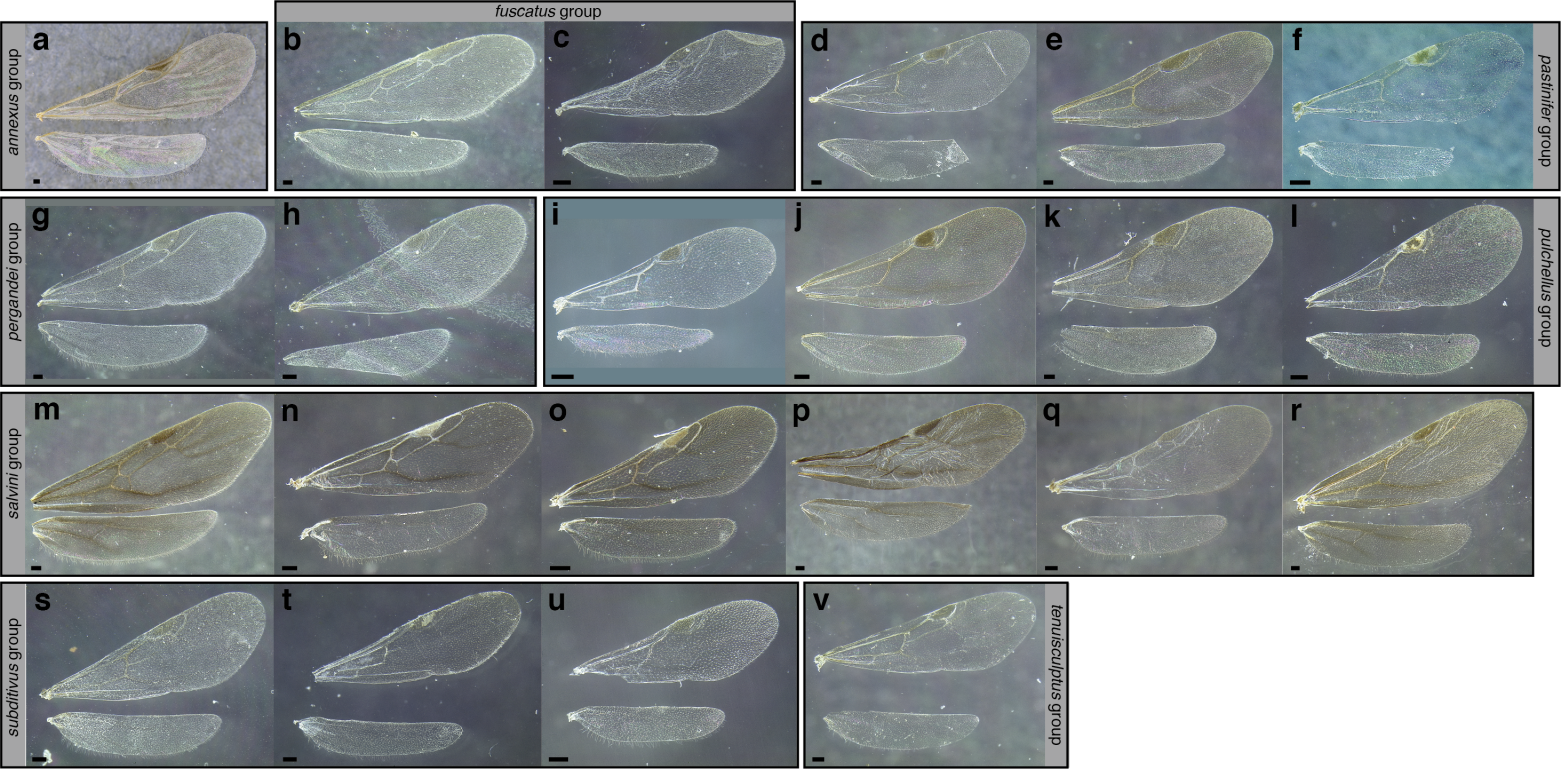
Comparison of wings of *salvini* clade reproductive organized by species group. (A) *Temnothorax quercicola* sp. nov. gyne (CASENT0869108). (B) *T. fuscatus* gyne (CASENT0615617). (C) *T. fuscatus* male (CASENT0625205). (D) *T. pastinifer* gyne (CASENT0758861). (E) *T. rutabulafer* sp. nov. gyne (CASENT0758831). (F) *T. rutabulafer* sp. nov. male (CASENT0756085). (G) *T. pergandei* gyne (CASENT0758229). (H) *T. pergandei* male (CASENT0758229). (I) *T. albispinus* male (LACMENT323246). (J) *T. laticrus* sp. nov. gyne (MCZENT00510556). (K) *T. wilsoni* sp. nov. gyne (CASENT0758813). (L) *T. wilsoni* sp. nov. male (CASENT0758813). (M) *T. aztecoides* sp. nov. gyne (CASENT0758789). (N) *T. aztecoides* sp. nov. male (CASENT0758790). (O) *T. aztecus* male (CASENT0609756). (P) *T. longinoi* sp. nov. gyne (CASENT0619354). (Q) *T. longinoi* sp. nov. male (CASENT0619355). (R) *T. parvidentatus* sp. nov. gyne (CASENT0612665). (S) *T. politus* gyne (CASENT0758021). (T) *T. subditivus* gyne (CASENT0758338). (U) *T. subditivus* male (CASENT0915978). (V) *T. tenuisculptus* gyne (CASENT0756078). Scale bars 0.2 mm.

***Reproductive caste wings*.** Although this article primarily uses workers the wings of the reproductive castes may provide additional information for determining species group membership. Although castes remain unknown for many of the species examined and described below, variation in the presence and absence of cells and angle of wing veins in the forewing appear to be useful characters in distinguishing between species groups (Fig. 17 and see dichotomous key below).

### Species & species group concepts

In this study, I endeavor to follow the biological species concept as a guide for species delimitation, in which species are defined as populations or metapopulations that actually or may potentially interbreed to produce viable offspring in nature, and are therefore reproductively isolated from other species (Mayr, 1942; Coyne & Orr, 2004). Because breeding experiments were not feasible for this study, I use a combination of morphology, genetics, and geography as a proxy to determine species boundaries. Essentially, species boundaries are established if morphological and genetic disparity are maintained in sympatry in morphologically similar specimens. The genetic data for this study comes from Prebus (2017, 2021) as well as a study in preparation. Still, I was not able to acquire genetic data for all species listed below; in these cases, species boundaries are more subjective, and based solely on my personal concept of how morphologically disparate populations of *Temnothorax* must become before they may be considered distinct species. I include a discussion of species boundaries in the comments section of each taxon treatment and include images of all known morphological variants of each species.

The species accounts are organized by species group, an informal taxonomic ranking between genus and species. The species groups used in this study correspond to morphologically diagnosable groups of species which are supported as monophyletic groups by molecular evidence from the studies mentioned above.

## Results

### Key to the clades of New World *Temnothorax* based on the worker caste

This key is based on the clades of *Temnothorax* recovered in Prebus, 2017, which was based on phylogenetic analysis of ultraconserved element data. To augment this key, please refer to the list of all described *Temnothorax* species in the Americas and their clade memberships in Supplemental File 2.

**Figure 18:**
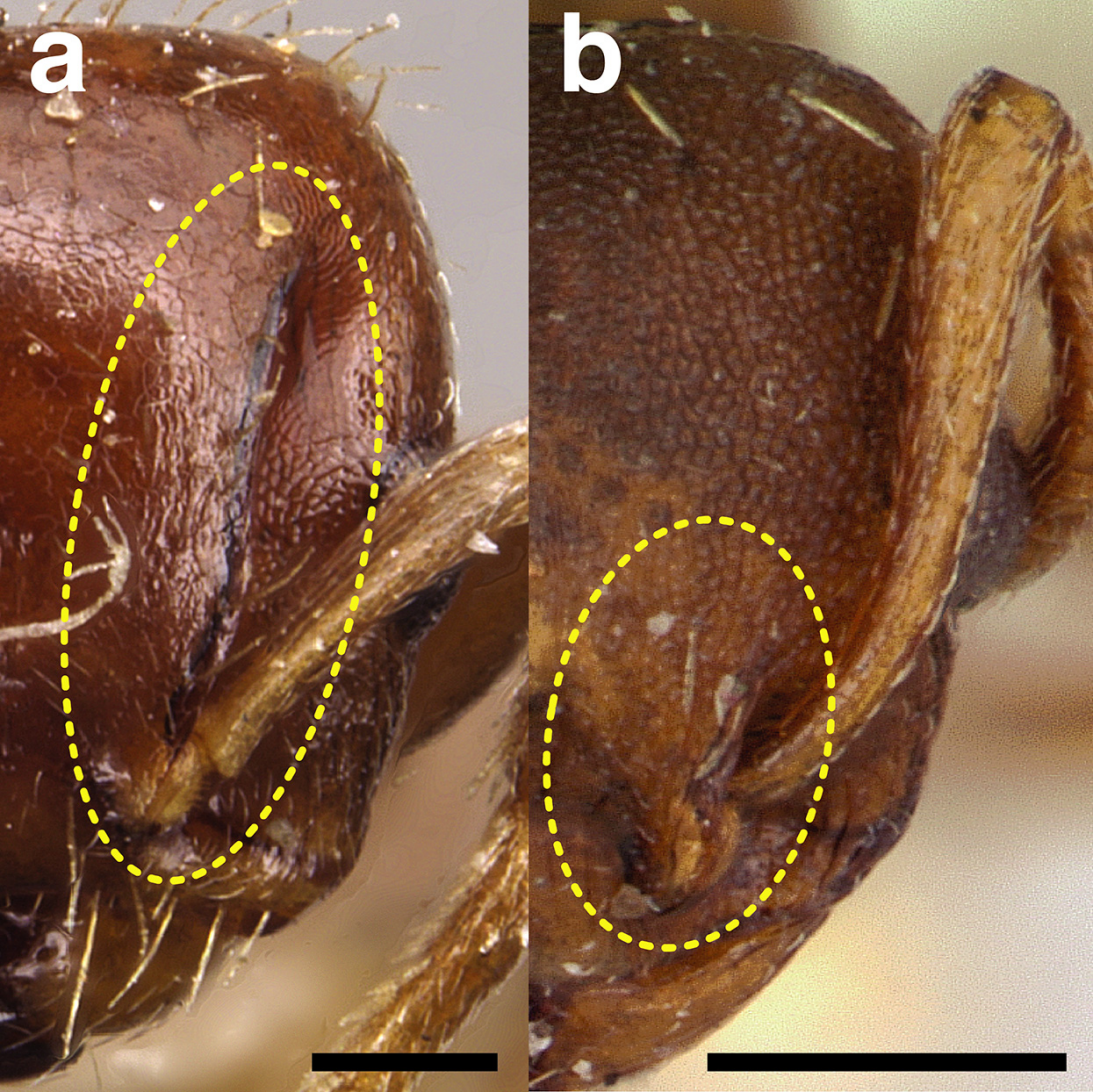
Comparison of morphology in *Temnothorax* worker heads in full-face view. (A) *T. americanus* (CASENT0904772; photo: Will Ericson, from www.antweb.org). (B) *T. duloticus* (CASENT0103163; photo: April Nobile, from www.antweb.org). Scale bars 0.2 mm.

**Figure 19:**
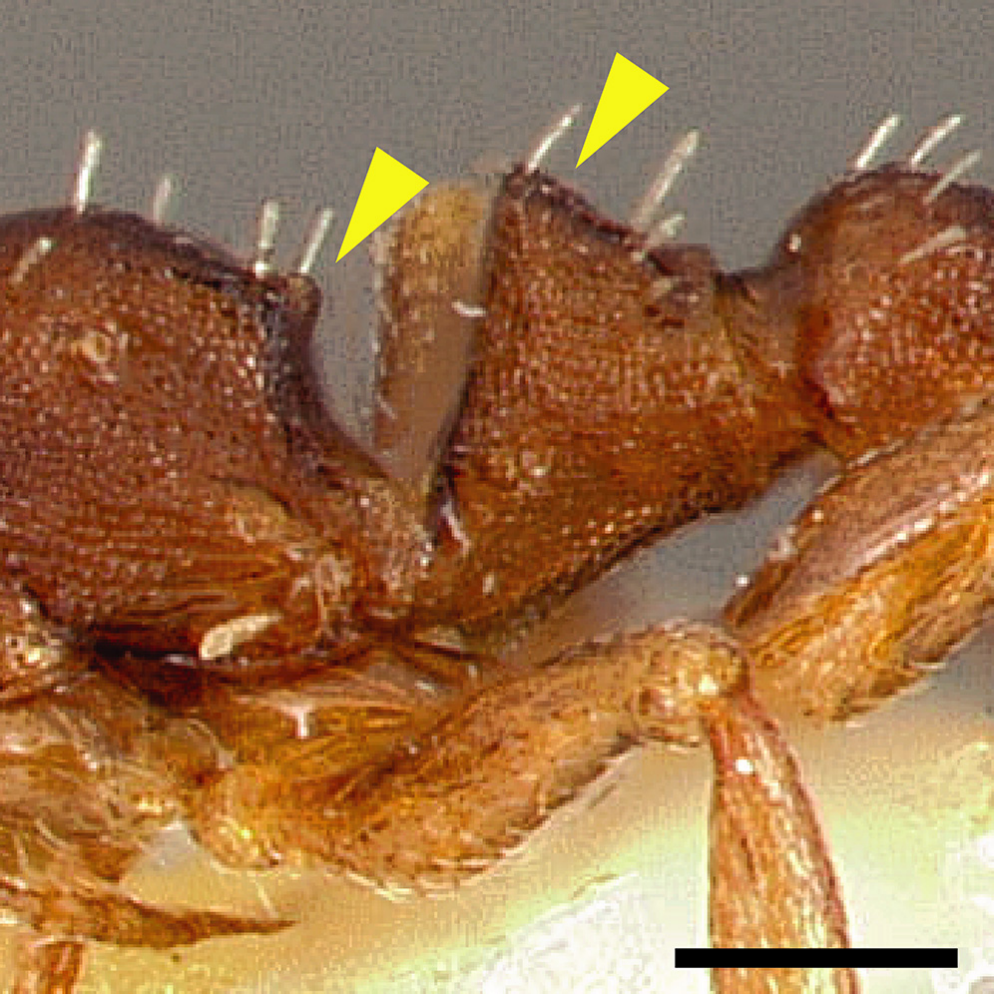
*Temnothorax oxynodis* worker propodeum and waist segments in profile view. (CASENT0005443; photo: April Nobile, from www.antweb.org). Scale bar 0.2 mm.

**Figure 20:**
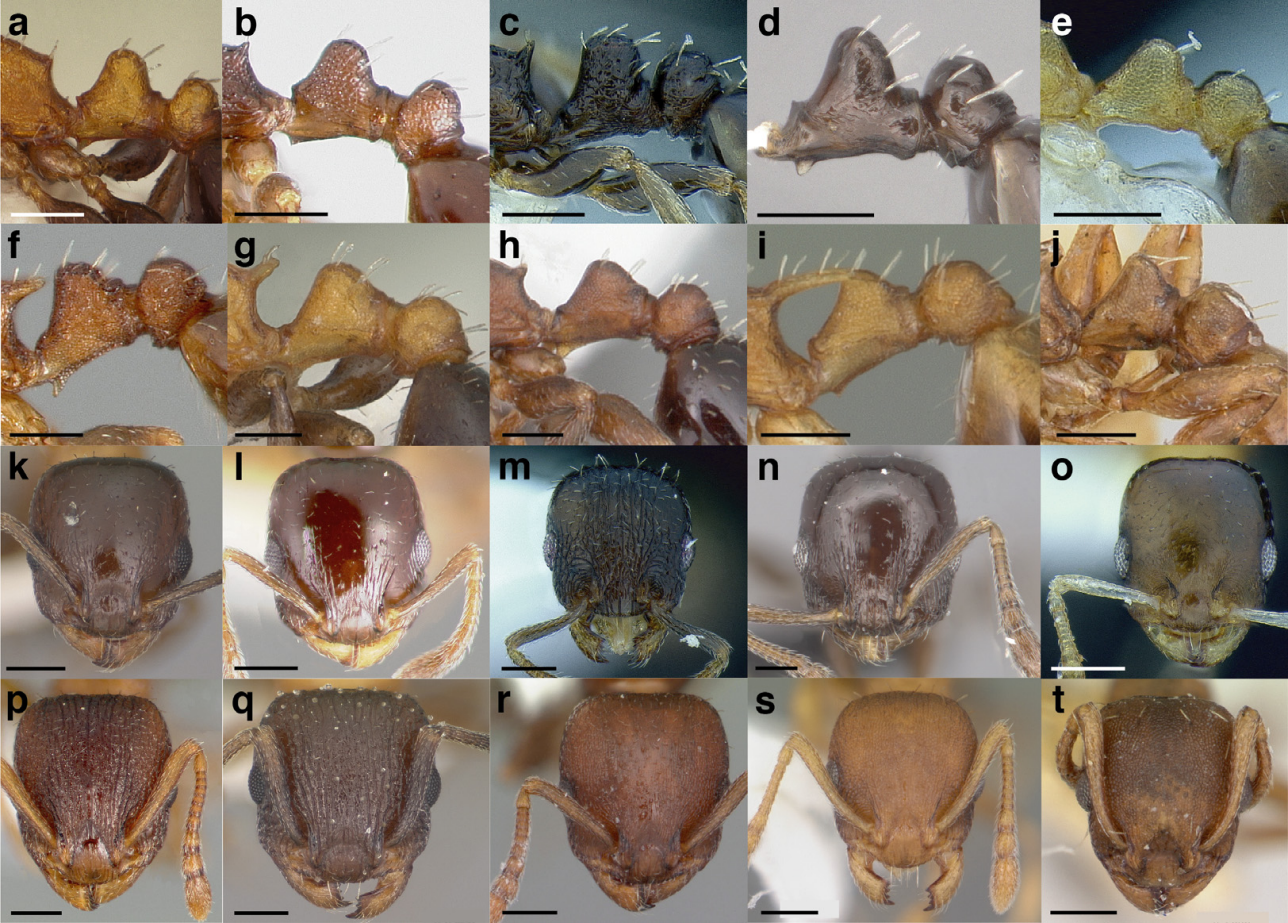
Comparison of *Temnothorax* worker waist segments and heads. (A–E) *T. obturator* clade worker propodeum and waist segments in profile view. (A) *T. emmae* (CASENT0102836; photo: Jen Fogarty, from www.antweb.org). (B) *T. whitfordi* (CASENT0005934; photo: April Nobile, from www.antweb.org). (C) *T. *mmp09 (CASENT0613041). (D) *T.* sp. nr. mmp09 (CASENT0758934) (E) *T.* mmp15 (LACMENT142344). (F–J) *T. rugatulus* and *longispinosus* clade worker propodeum and waist segments in profile view. (F) *T. rugatulus* (CASENT0005690; photo: April Nobile, from www.antweb.org). (G) *T. josephi* (CASENT0102837; photo: Jen Fogarty, from www.antweb.org). (H) *T. schaumii* (CASENT0104050; photo: April Nobile, from www.antweb.org). (I) *T. curvispinous* (CASENT0104040; photo: April Nobile, from www.antweb.org). (J) *T. duloticus* (CASENT0103163; photo: April Nobile, from www.antweb.org). (K–O) *T. obturator* clade worker heads in full-face view. (K) *T. emmae* (CASENT0102836; photo: Jen Fogarty, from www.antweb.org). (L) *T. whitfordi* (CASENT0005934; photo: April Nobile, from www.antweb.org). (M) *T.* mmp09 (CASENT0613041). (N) *T. *sp. nr. mmp09 (CASENT0758934). (O) *T.* mmp15 (LACMENT142344). (P–T) *T. rugatulus* and *longispinosus* clade worker heads in full-face view. (P) *T. rugatulus* (CASENT0005690; photo: April Nobile, from www.antweb.org). (Q) *T. josephi* (CASENT0102837; photo: Jen Fogarty, from www.antweb.org). (R) *T. schaumii* (CASENT0104050; photo: April Nobile, from www.antweb.org). (S) *T. curvispinous* (CASENT0104040; photo: April Nobile, from www.antweb.org). (T) *T. duloticus* (CASENT0103163; photo: April Nobile, from www.antweb.org). Scale bars 0.2 mm.

**Figure 21:**
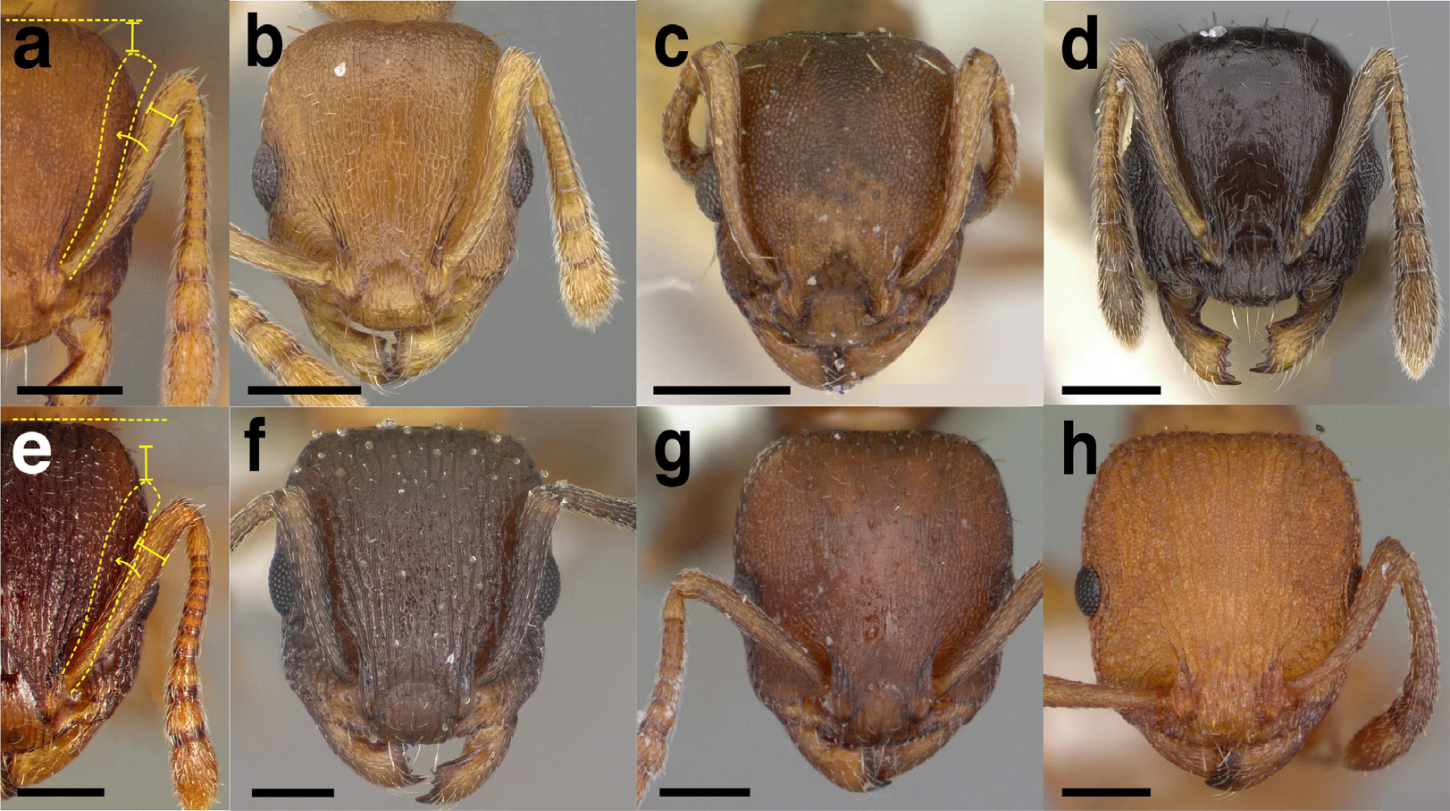
Comparison of *longispinosus* and *rugatulus* clade worker heads in full-face view. (A) *Temnothorax curvispinosus* (CASENT0104040; photo: April Nobile, from www.antweb.org). (B) *T. ambiguus* (CASENT0915985; photo: Michele Esposito, from www.antweb.org). (C) *T. duloticus* (CASENT0103163; photo: April Nobile, from www.antweb.org). (D) *T. longispinosus* (CASENT0914508; photo: Dominique Monie, from www.antweb.org). (E) *T. rugatulus* worker head in full face view (CASENT0005690; photo: April Nobile, from www.antweb.org). (F) *T. josephi* (CASENT0102837; photo: Jen Fogarty, from www.antweb.org). (G) *T. schaumii* (CASENT0104050; photo: April Nobile, from www.antweb.org). (H) *T. bradleyi* (CASENT0104011; photo: April Nobile, from www.antweb.org). Scale bars 0.2 mm.

**Figure 22:**
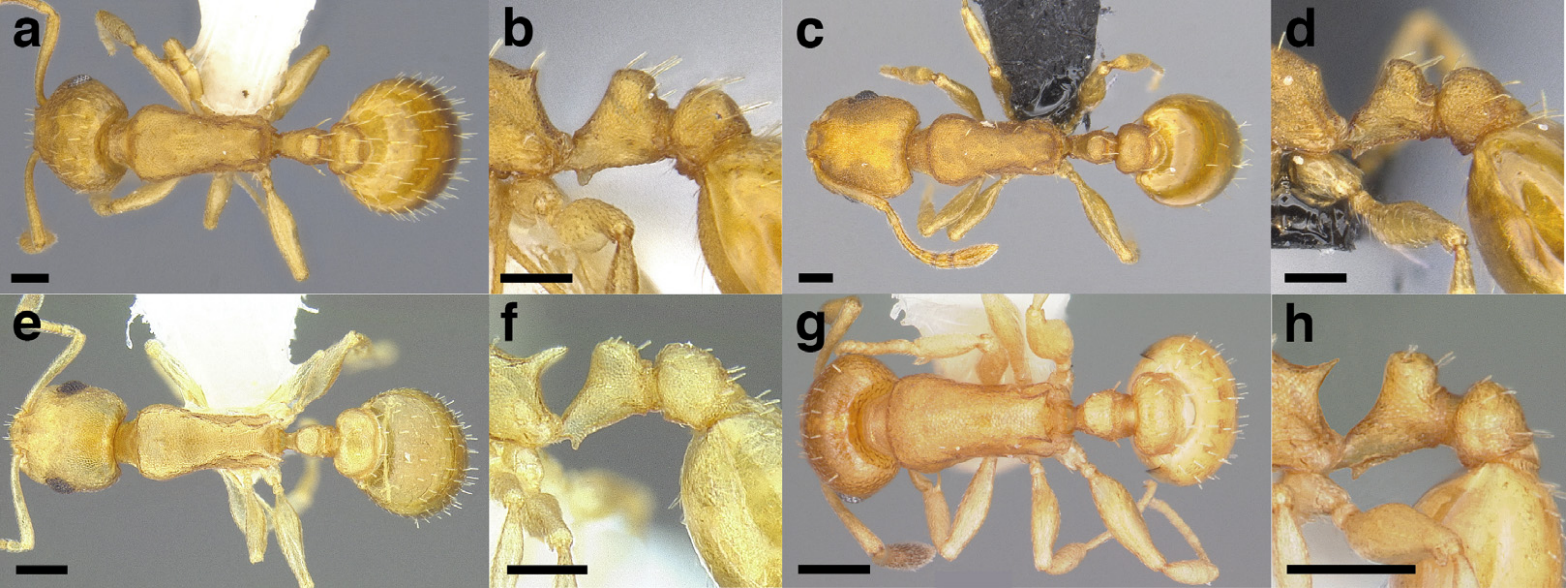
Characters distinguishing *andersoni* clade species from lookalike species. (A) *Temnothorax andersoni*, dorsal view (CASENT0635924). (B) *T. andersoni*, waist segments in profile (CASENT0635924). (C) *T. punctatissimus* (*andersoni* clade), dorsal view (CASENT0758783). (D) *T. punctatissimus* (*andersoni* clade), waist segments in profile (CASENT0758783). (E) *T. pilicornis* sp. nov. (*salvini* clade), dorsal view (CASENT0756186). (F) *T. pilicornis* sp. nov. (*salvini* clade), waist segments in profile (CASENT0756186). (G) *T. terrigena* (*salvini* clade), dorsal view (CASENT0104775; photo: April Nobile, from www.antweb.org) (H) *T. terrigena* (*salvini* clade), waist segments in profile (CASENT0104775; photo: April Nobile, from www.antweb.org). Scale bars 0.2 mm.

**Figure 23:**
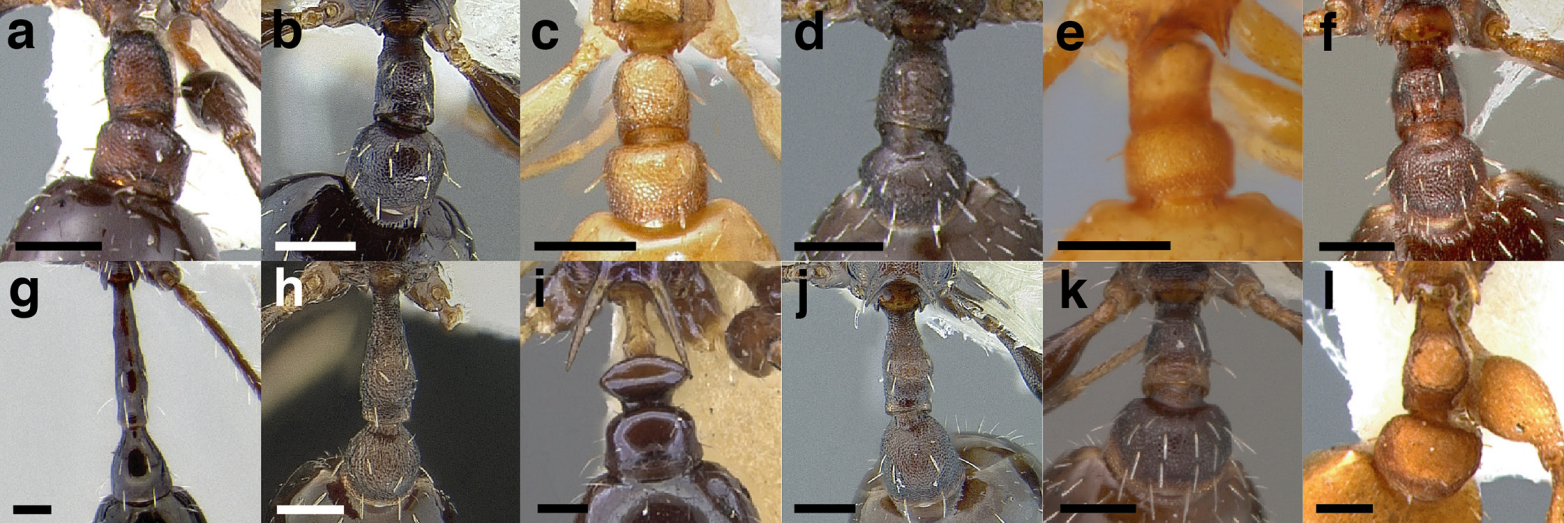
Comparison of *Temnothorax* worker waist segments in dorsal view. (A) *T. obturator* (CASENT0105860; photo: Dan Kjar, from www.antweb.org). (B) *T. acuminatus* sp. nov. (JTLC000007439). (C) *T. andrei* (CASENT0005683; photo: April Nobile, from www.antweb.org). (D) *T. adustus* (CASENT0105862; photo: Dan Kjar, from www.antweb.org). (E) *T. furunculus* (UTEPENTO60001; photo: Lindsie McCabe, from www.antweb.org). (F) *T. gallae* (CASENT0005684; photo: April Nobile, from www.antweb.org). (G) *T. poeyi* (CASENT0106241; photo: Michael Branstetter, from www.antweb.org). (H) *T. mmp11* (CASENT0617101). (I) *T. squamifer* (CASENT0901793; photo: Will Ericson, from www.antweb.org). (J) *T. fuscatus* (CASENT0625205). (K) *T. tricarinatus* (CASENT0102845; photo: Jen Fogarty, from www.antweb.org). (L) *T. quercicola* sp. nov. (CASENT0105865; photo: Dan Kjar, from www.antweb.org). Scale bars 0.2 mm.

**Figure 24:**
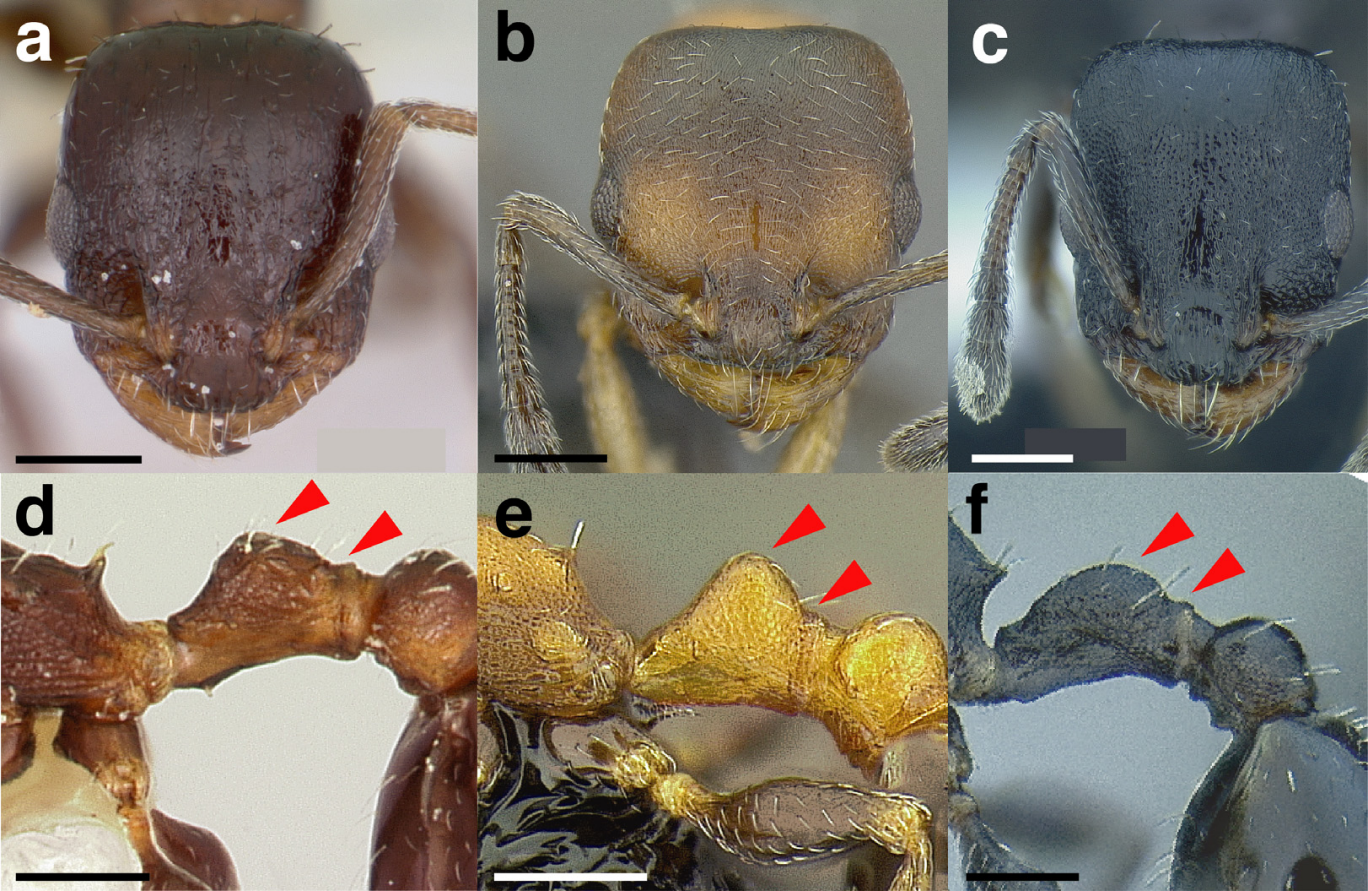
Synopsis of characters distinguishing workers of *obturator* clade species with 12 -segmented antennae from other *Temnothorax* species. (A) *T. obturator*, full face view (CASENT0104756; photo: April Nobile, from www.antweb.org). (B) *T. creolus*, full face view (CASENT0755643). (C) *T. *sp. nr. *obturator*, full face view (CASENT0733254). (D) *T. obturator* waist segments in profile (CASENT0104756; photo: April Nobile, from www.antweb.org). (E) *T. creolus* waist segments in profile (CASENT0755643). (F) *T. *sp. nr. *obturator* waist segments in profile (CASENT0733254). Scale bars 0.2 mm.

**Figure 25:**
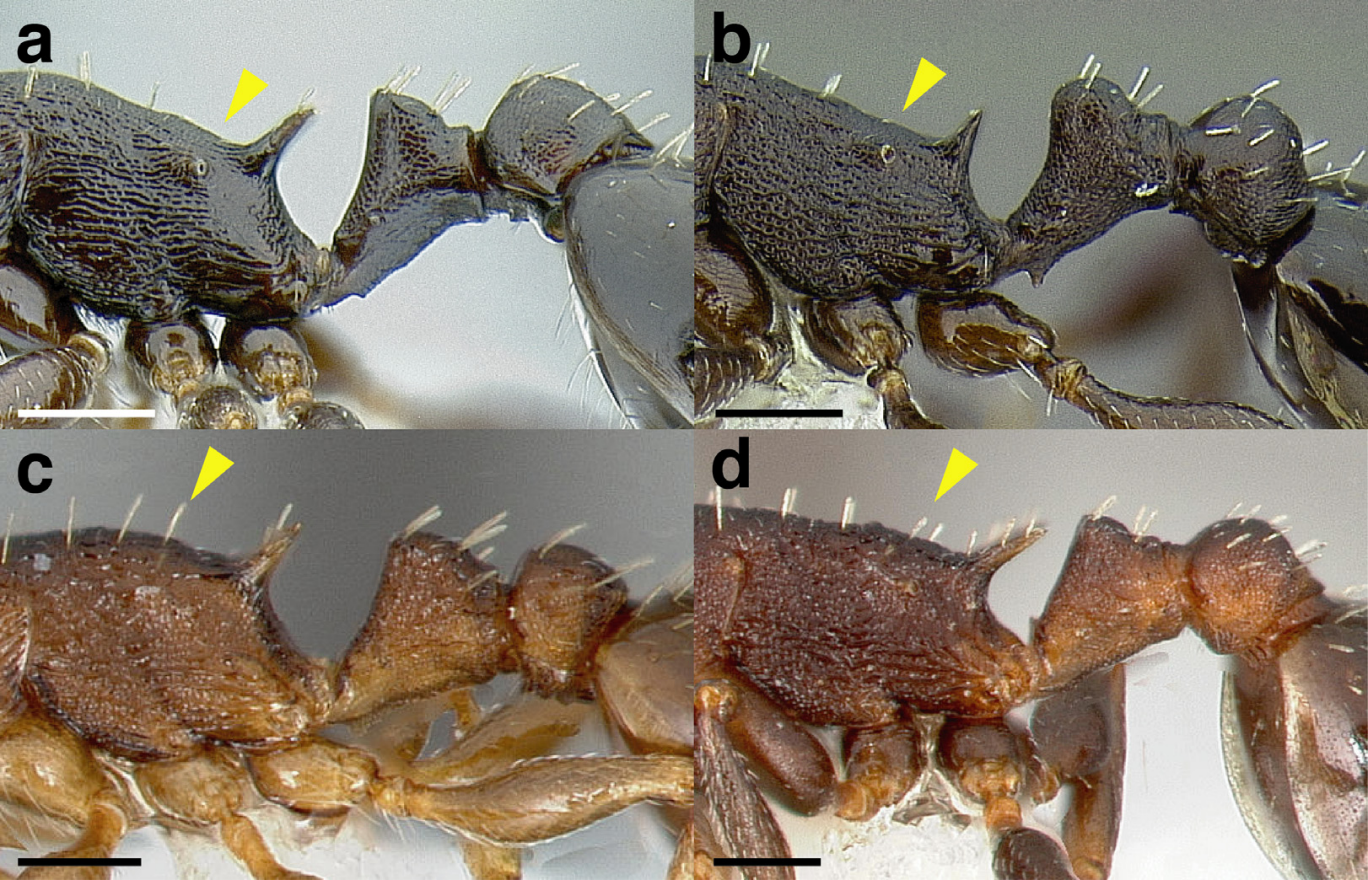
Characters separating workers of *salvini* clade species with narrow postpetioles from other *Temnothorax* species, propodeum and waist segments in profile. (A) *T. acuminatus* sp. nov. (JTLC000007439). (B) *T. acutispinosus* sp. nov. (JTLC000007447). (C) *T. rudis* (CASENT0005689; photo: April Nobile, from www.antweb.org). (D) *T. gallae* (CASENT0005684; photo: April Nobile, from www.antweb.org). Scale bars 0.2 mm.

**Figure 26:**
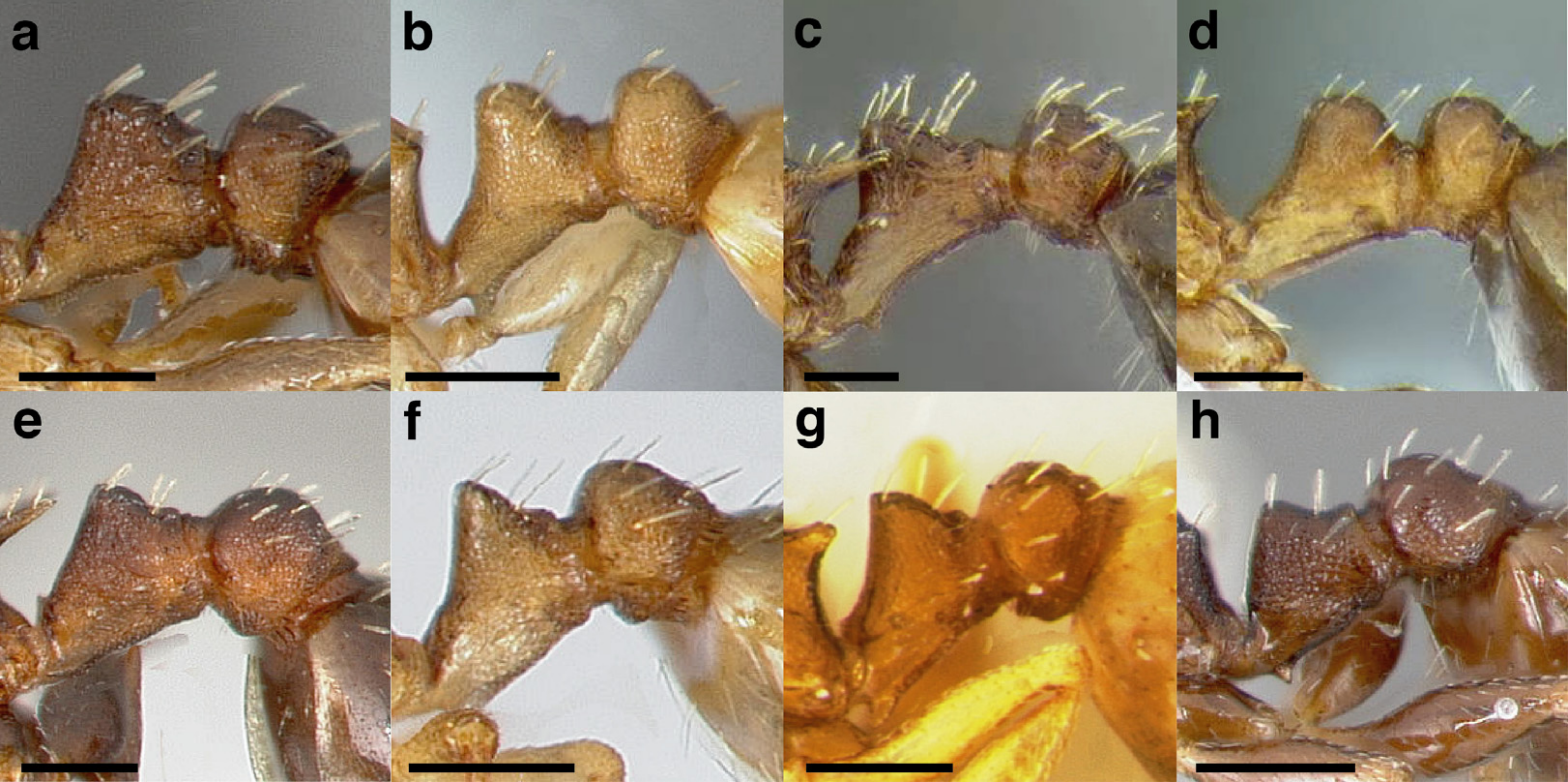
Petioles of *Temnothorax* workers in profile, comparing non-cuneiform and cuneiform. (A) *T. rudis* (CASENT0005689; photo: April Nobile, from www.antweb.org). (B) *T. andrei* (CASENT0005683; photo: April Nobile, from www.antweb.org). (C) *T. myrmiciformis* (CASENT0339323; photo Marek Borowiec, from www.antweb.org). (D) *T. paiute* (CASENT0005932; photo Marek Borowiec, from www.antweb.org). (E) *T. gallae* (CASENT0005684; photo: April Nobile, from www.antweb.org). (F) *T. nitens* (CASENT0005686; photo: April Nobile, from www.antweb.org). (G) *T. furunculus* (CASENT0105615; photo: Dan Kjar, from www.antweb.org). (H) *T. arboreus* (CASENT0005933; photo: April Nobile, from www.antweb.org). Scale bars 0.2 mm.

**Figure 27:**
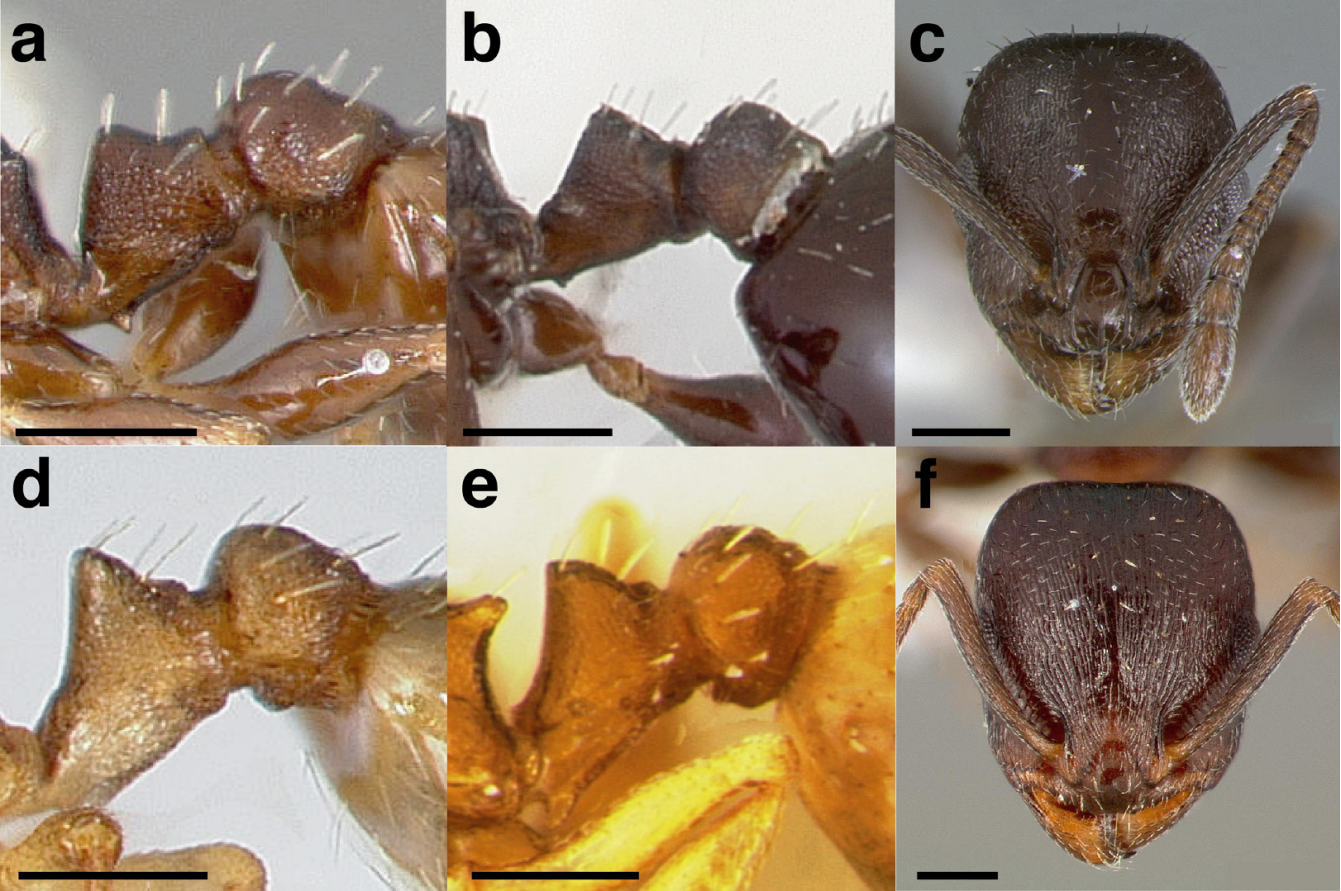
Characters separating workers of *sallei* clade species with cuneiform petioles from other *Temnothorax* species. (A) *T. arboreus* waist segments in profile (CASENT0005933; photo: April Nobile, from www.antweb.org). (B) *T. adustus* waist segments in profile (CASENT0172590; photo: April Nobile, from www.antweb.org). (C) *T. adustus* head in full-face view (CASENT0105862; photo: Dan Kjar, from www.antweb.org). (D) *T. nitens* waist segments in profile (CASENT0005686; photo: April Nobile, from www.antweb.org). (E) *T. furunculus* waist segments in profile (CASENT0105615; photo: Dan Kjar, from www.antweb.org). (F) *T. gallae* head in full-face view (CASENT0005684; photo: April Nobile, from www.antweb.org). Scale bars 0.2 mm.

**Figure 28:**
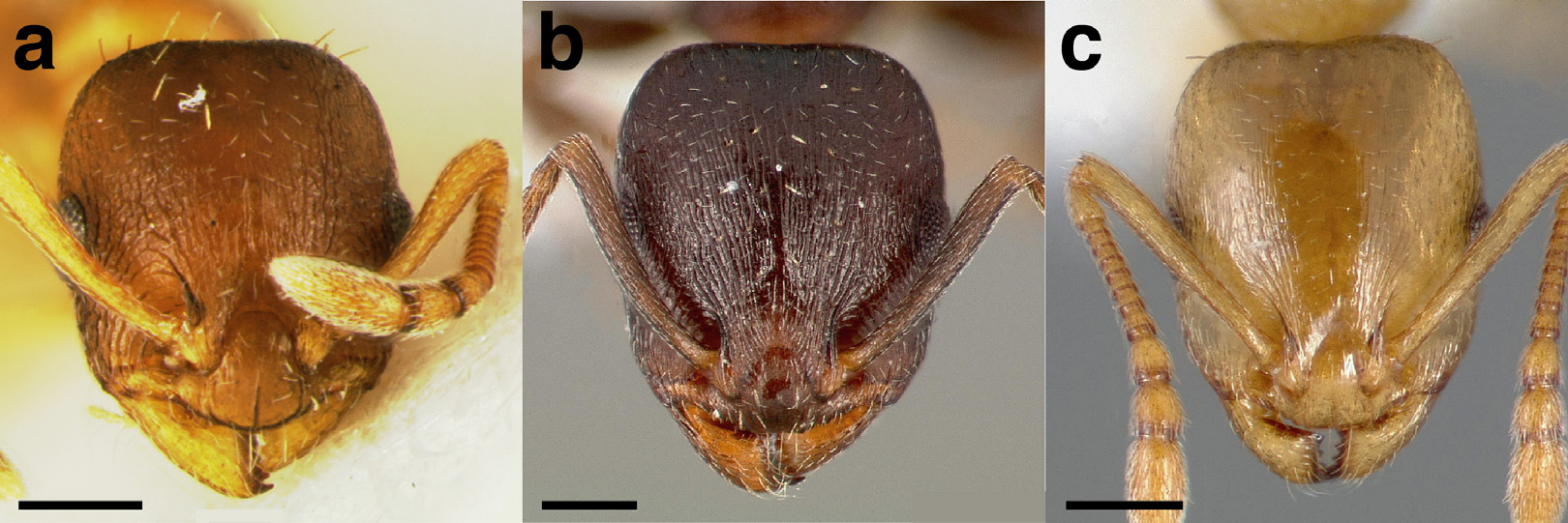
Characters separating workers of *Temnothorax furunculus* from workers of *andrei* clade workers with cuneiform petioles, heads in full-face view. (A) *T. furunculus* (CASENT0105615; photo: Dan Kjar, from www.antweb.org). (B) *T. gallae* (CASENT0005684; photo: April Nobile, from www.antweb.org). (C) *T. nitens* (CASENT0005686; photo: April Nobile, from www.antweb.org). Scale bars 0.2 mm.

**Figure 29:**
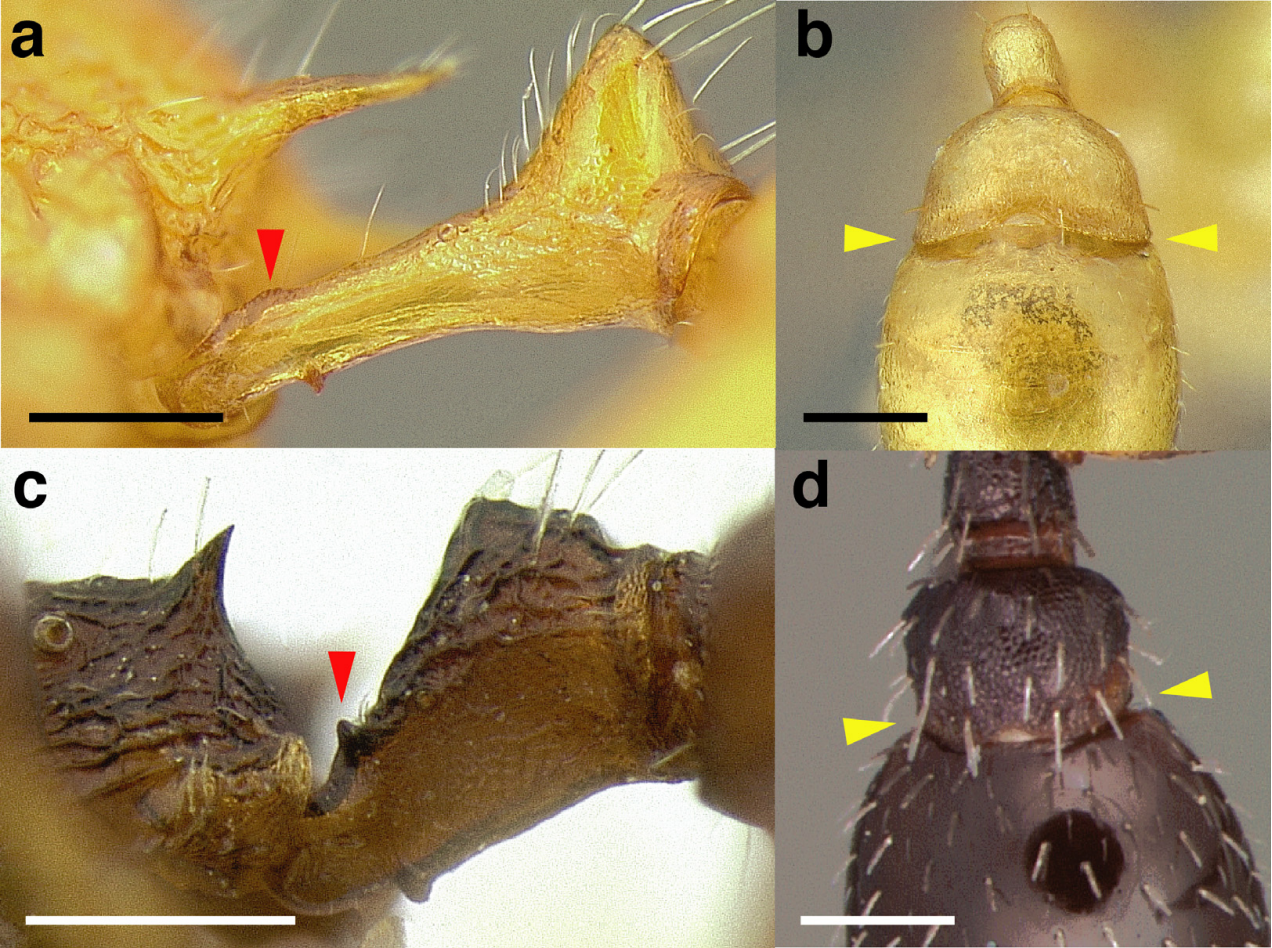
Characters separating workers of several clades of *Temnothorax*. (A) *T. aztecoides* sp. nov. petiole in ventrolateral view (CASENT0733319). (B) *T. agavicola* sp. nov. worker waist segments and gaster in dorsal view (CASENT0758360). (C) *T. tricarinatus* worker petiole in ventrolateral view (LACMENT323609). (D) *T. texanus* worker waist segments and gaster in dorsal view (CASENT0104063; photo: April Nobile, from www.antweb.org). Scale bars 0.2 mm.

**Figure 30:**
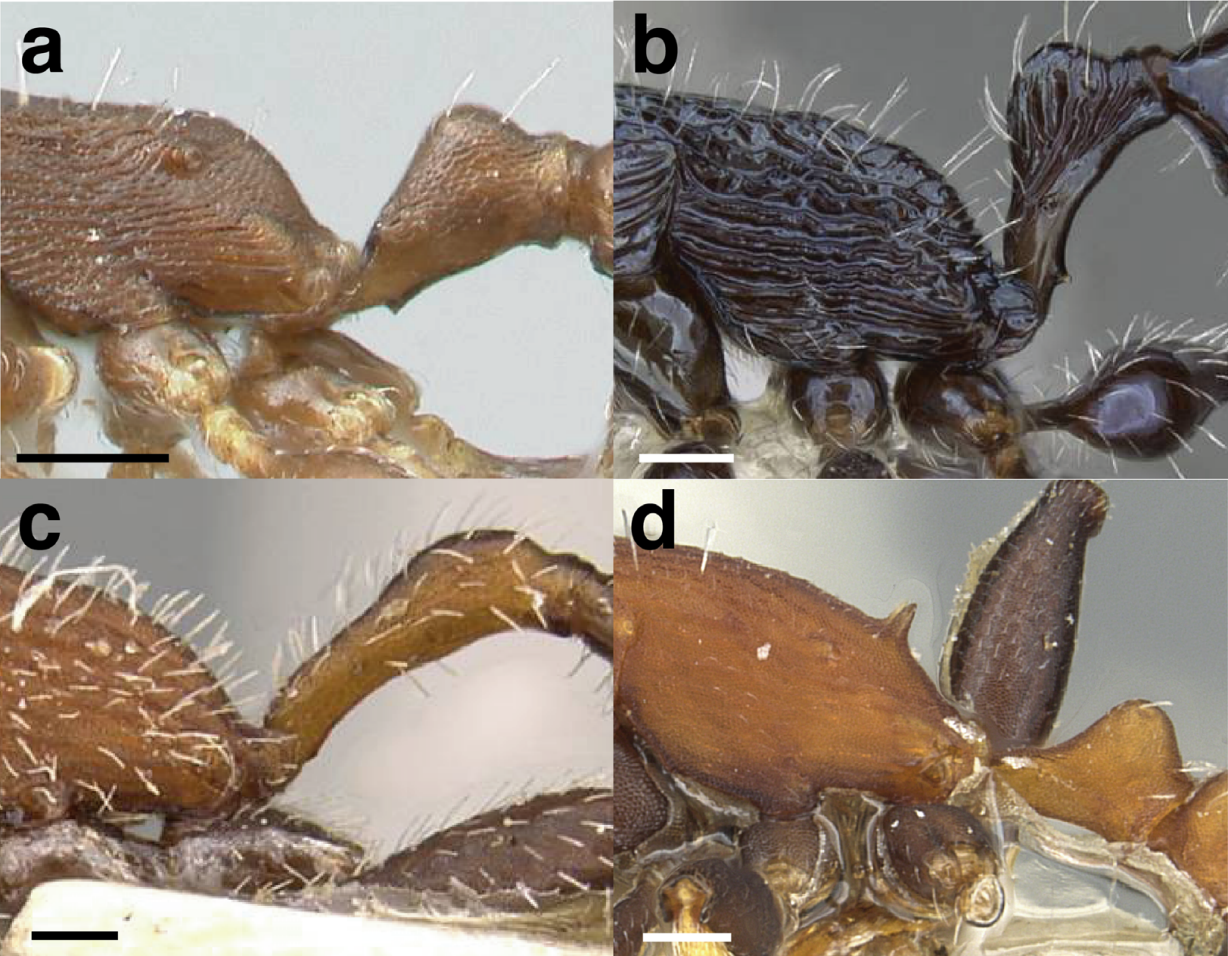
Propodeum and waist segments workers of *sallei* clade species with rounded petioles in profile view. (A) *Temnothorax striatulus* (FOCOL2038; photo: Christiana Klingenberg, from www.antweb.org). (B) *T. gundlachi* (CASENT0916006; photo: Michele Esposito, from www.antweb.org). (C) *T. versicolor* (CASENT0914180; photo: Ziv Lieberman, from www.antweb.org). (D) *T. isabellae* (CASENT0915380; photo: Will Ericson, from www.antweb.org). Scale bars 0.2 mm.

**Figure 31:**
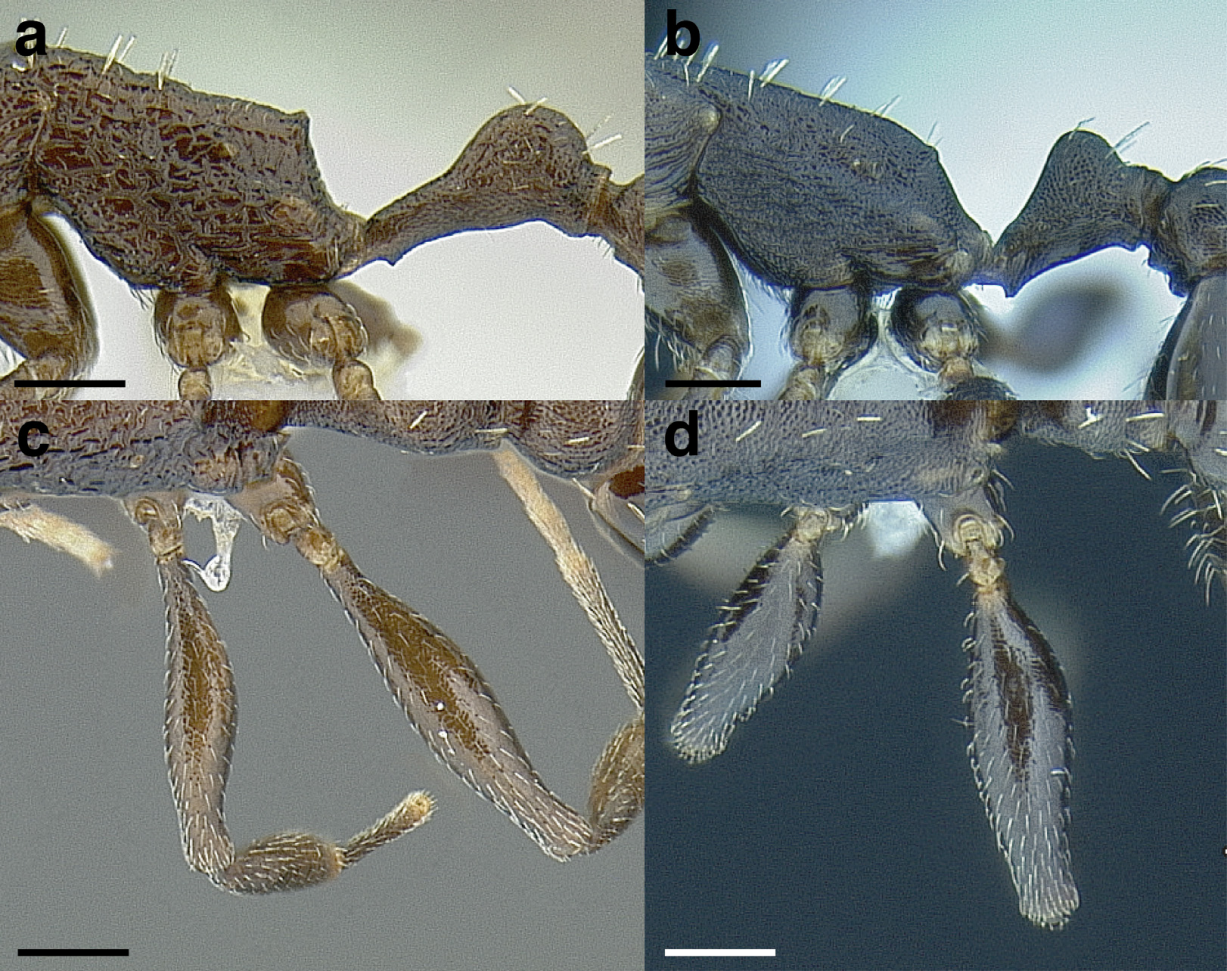
*Temnothorax sallei* clade workers with reduced propodeal teeth. Propodeum and waist segments in profile view. (A) *T.* mmp11 (CASENT0617101). (B) *T. *sp. nr. mmp49 (CASENT0733208); legs in dorsal view. (C) *T. *mmp11 (CASENT0617101). (D) *T. *sp. nr. mmp49 (CASENT0733208). Scale bars 0.2 mm.

**Figure 32:**
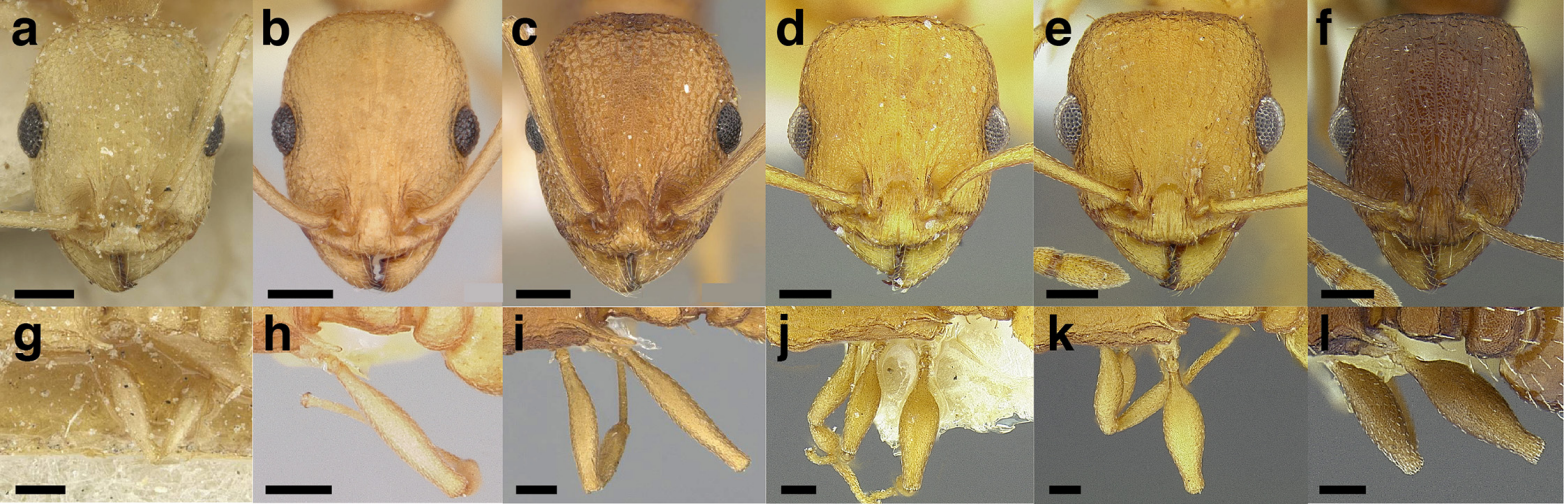
*Temnothorax sallei* clade and *salvini* clade workers. Heads in full-face view. (A) *T. silvestrii* (CASENT0913000; photo: Ziv Lieberman, from www.antweb.org). (B) *T. morongo* (CASENT0103108; photo: April Nobile, from www.antweb.org). (C) *T. *bca07 (CASENT0922107; photo: Michele Esposito, from www.antweb.org). (D) *T. quercicola* sp. nov. (LACMENT323216). (E) *T. obtusigaster* sp. nov. (CASENT0758654). (F) *T. balnearius* sp. nov. (LACMENT323199); legs in dorsal view. (G) *T. silvestrii* (CASENT0913000; photo: Ziv Lieberman, from www.antweb.org). (H) *T. morongo* (CASENT0103108; photo: April Nobile, from www.antweb.org). (I) *T. *bca07 (CASENT0922107; photo: Michele Esposito, from www.antweb.org). (J) *T. quercicola* sp. nov. (LACMENT323216). (K) *T. obtusigaster* sp. nov. (CASENT0758654). (L) *T. balnearius* sp. nov. (LACMENT323199). Scale bars 0.2 mm.

**Figure 33:**
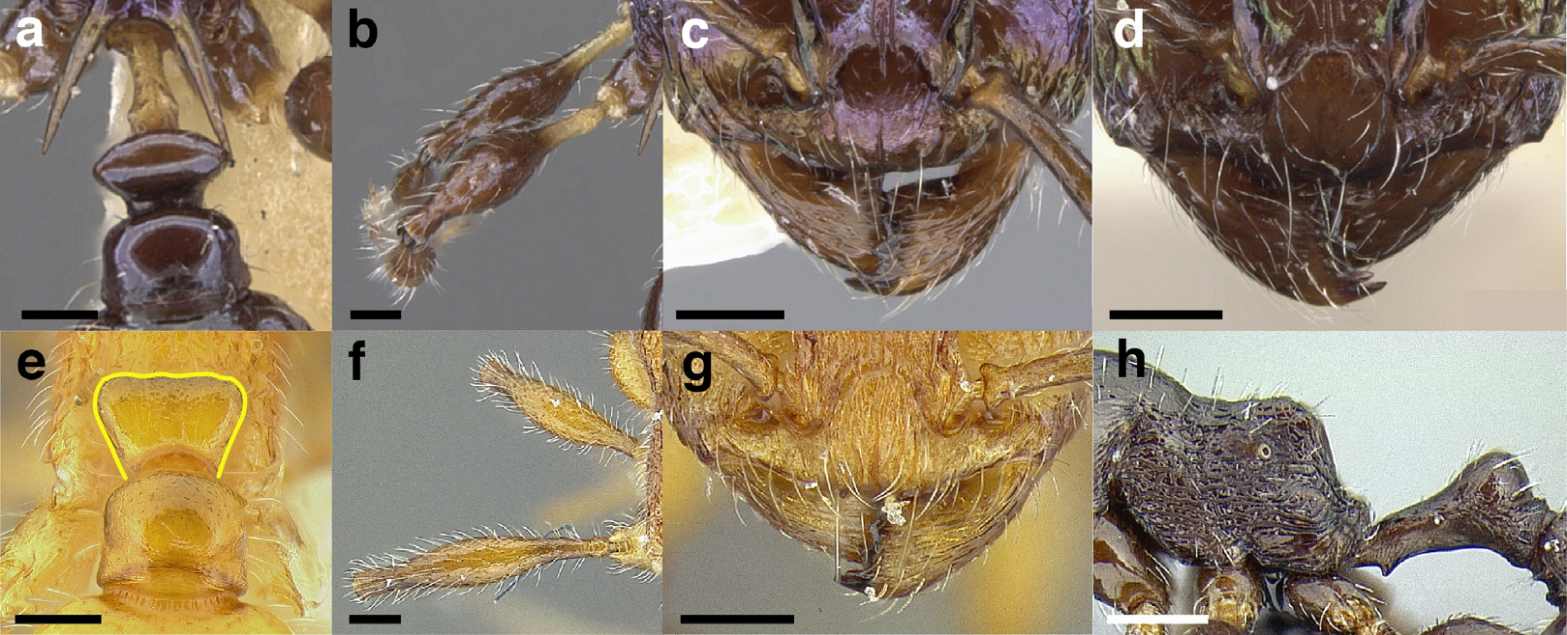
Characters separating some members of the *sallei* and *salvini* clades. (A–C) *Temnothorax squamifer* worker (CASENT0901793; photos: Will Ericson, from www.antweb.org). (A) waist segments in dorsal view. (B) legs in dorsal view. (C) clypeus and mandibles in full-face view. (D) *T. creightoni* worker, clypeus and mandibles in full-face view (CASENT0901794; photo: Will Ericson, from www.antweb.org) (E–G) *T. aztecoides* sp. nov. worker (CASENT0733319). (E) waist segments in posterodorsal view. (F) legs in dorsal view. (G) clypeus and mandibles in full-face view. (H) *T. bison* sp. nov. worker, propodeum and petiole in profile view (CASENT0636947). Scale bars 0.2 mm.

**Figure 34:**
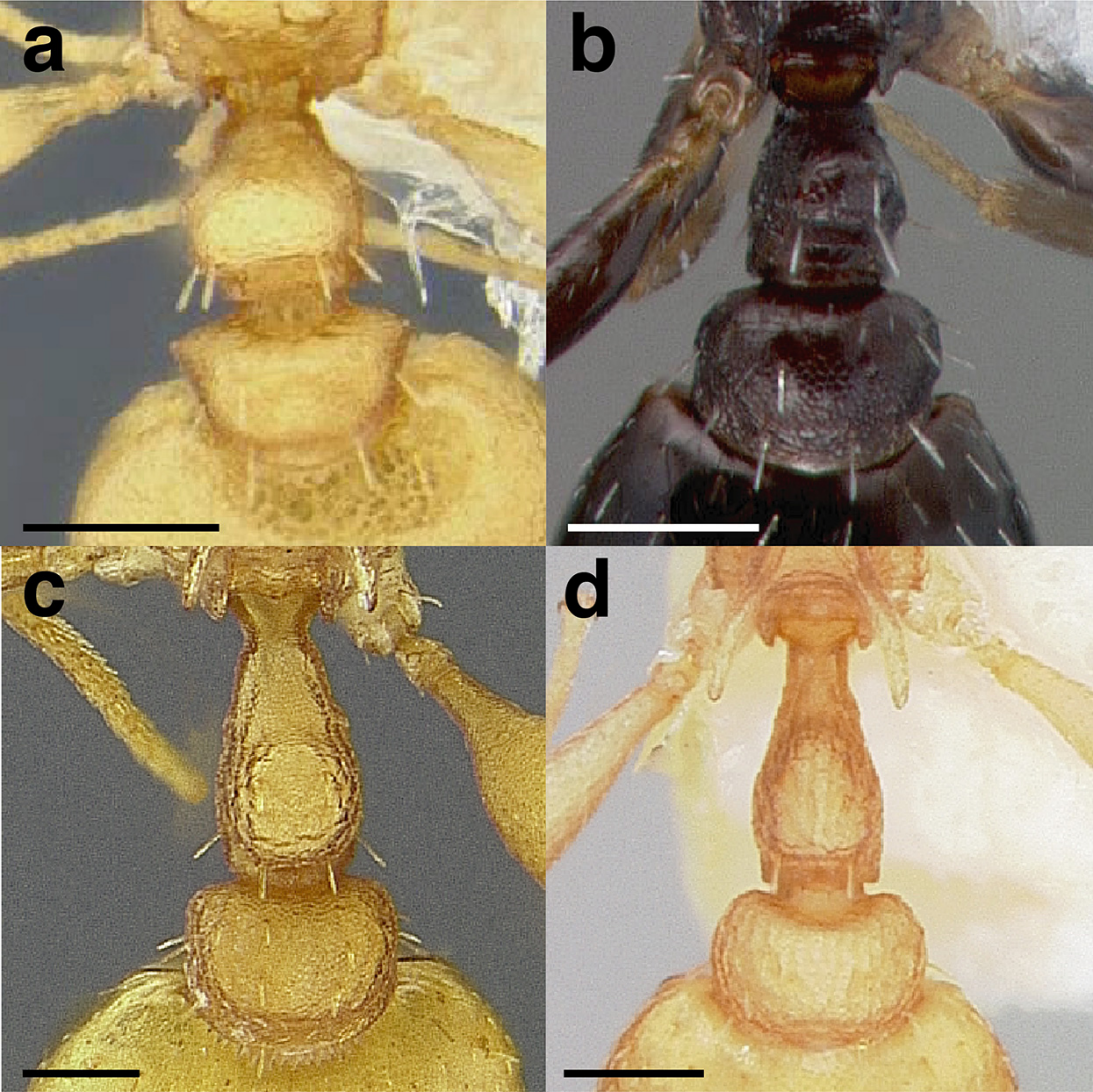
Characters separating *Temnothorax quasimodo* from *sallei* and *salvini* clade species, worker waist segments in dorsal view. (A) *T. quasimodo* (CASENT0005694; photo: Marek Borowiec, from www.antweb.org). (B) *T. neomexicanus* (*sallei* clade) (CASENT0010695; photo: April Nobile, from www.antweb.org). (C) *T. obtusigaster* sp. nov. (*salvini* clade) (CASENT0758654). (D) *T. morongo* (*sallei* clade) (CASENT0103108; photo: April Nobile, from www.antweb.org). Scale bars 0.2 mm.

**Figure 35:**
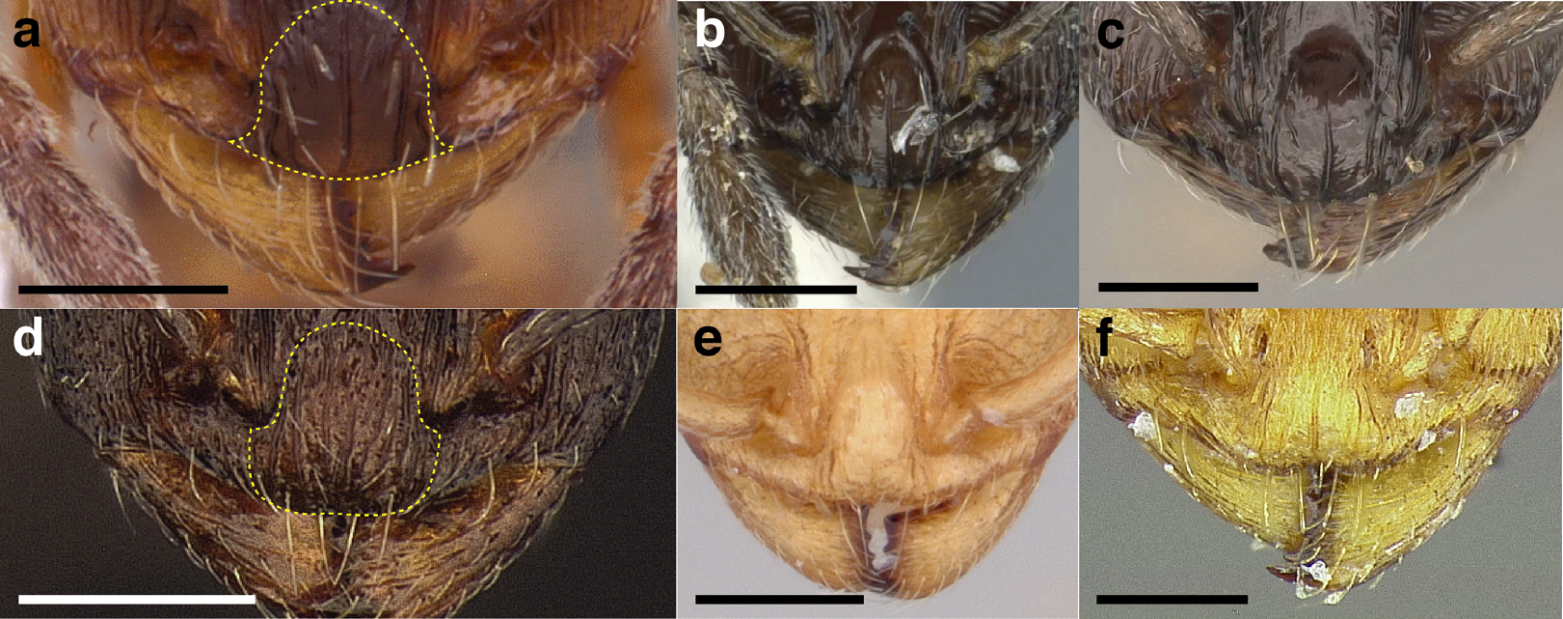
Characters separating the workers of some species of the *sallei* and *salvini* clades, clypeus and mandibles in full-face view. (A) *T. Temnothorax texanus* (*sallei* clade) (CASENT0104062; photo: April Nobile, from www.antweb.org). (B) *T. brevispinosus* (*sallei* clade) (CASENT0911187; photo: Ziv Lieberman, from www.antweb.org). (C) *T. stenotyle* (*sallei* clade) (CASENT0922576; photo: Wade Lee, from www.antweb.org). (D) *T. acuminatus* sp. nov. (*salvini* clade) (JTLC000007468). (E) *T. morongo* (*sallei* clade) (CASENT0103108; photo: April Nobile, from www.antweb.org). (F) *T. quercicola* sp. nov. (*salvini* clade) (LACMENT323216). Scale bars 0.2 mm.

**Figure 36:**
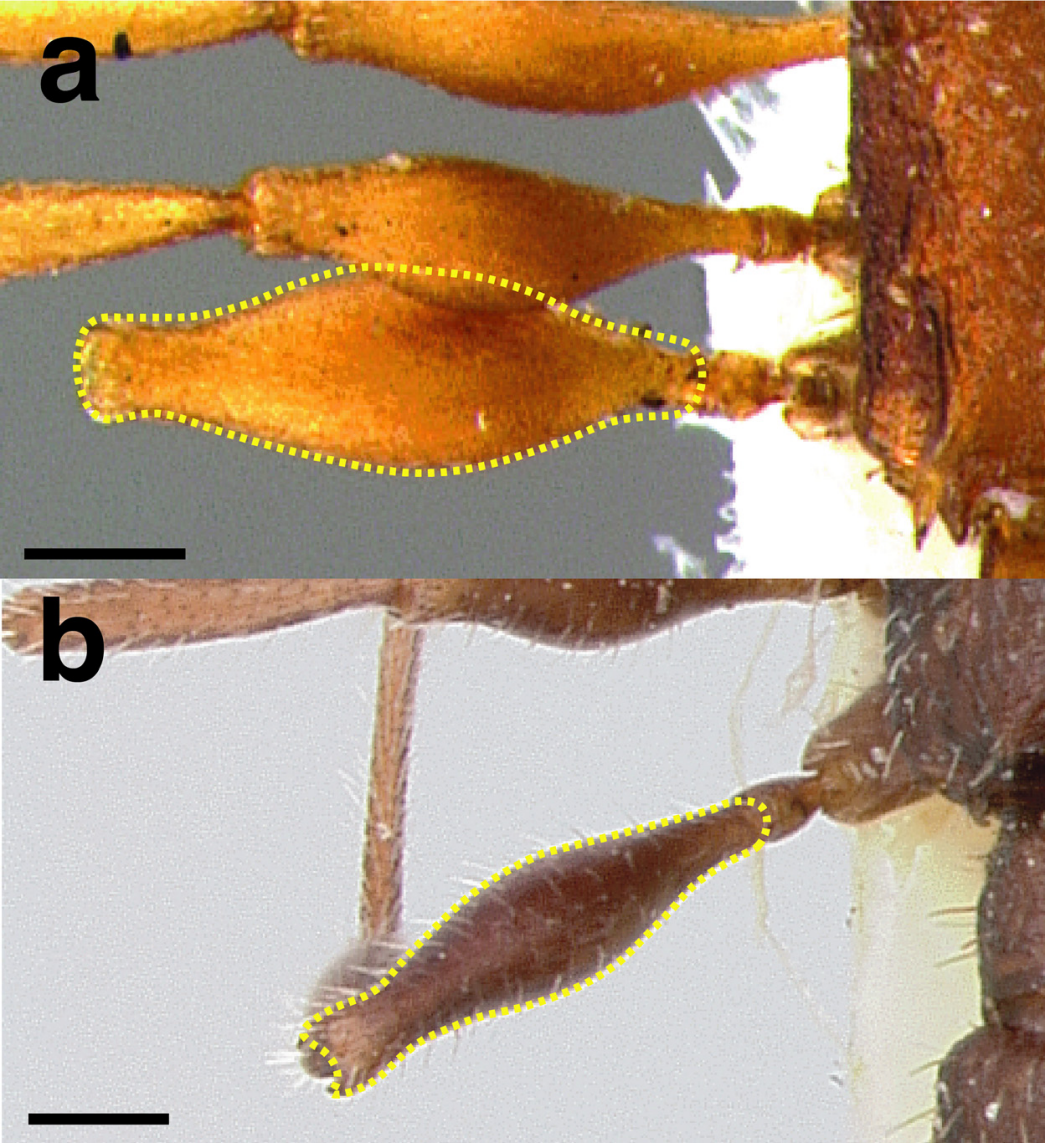
Characters separating the workers of some species of the *sallei* and *salvini* clades, legs in dorsal view. (A) *Temnothorax quercicola* sp. nov. (*salvini* clade) worker legs in dorsal view (CASENT0105865; photo: Dan Kjar, from www.antweb.org). (B) *T. hispidus* (*sallei* clade) worker legs in dorsal view (CASENT0172594; photo: April Nobile, from www.antweb.org). Scale bars 0.2 mm.

**1.** Antennae with 11 segments**2**
**-** Antennae with 12 segments**7**
**2.** Postpetiole broad and campaniform in dorsal view; mesosoma strongly arched and bearing long propodeal spines; Cuba***T. cuyaguateje*** (Fontenla) **(incertae sedis)**
**-** Not matching the above description; broadly distributed**3**
**3.** Antennal scrobes present (Fig. 18A); masticatory margin of mandible with four teeth; eastern North America***T. americanus*** (Emery) **(Palearctic clade, part)**
**-** Antennal scrobes absent (Fig. 18B); masticatory margin of mandible with five teeth; broadly distributed**4**
**4.** Propodeal spines reduced to small, rounded angles (Fig. 19); petiole acutely cuneiform in profile; arboreal species known only from west central California***T. oxynodis*** (Mackay) **(*andrei* clade, part)**
**-** Propodeal spines variable; petiole in profile shaped otherwise; broadly distributed**5**
**5.** Petiole compact and node evenly rounded in profile (Figs. 20A–20E) ***and*** laterodorsal surfaces of head directly behind compound eyes smooth and shining (Figs. 20K–20O); postpetiole ***often with*** an anterior bulge in profile view (except *T*. mmp15 from Puerto Rico; see Prebus (2017) and antweb.org) (Figs. 20A–20E); arboreal species of the American Southwest through Central America and Greater Antilles***obturator* clade, part**
**-** If petiole is rounded (some species in the *rugatulus* clade, Figs. 20G & 20H) ***then*** laterodorsal surfaces of head directly behind compound eyes sculptured (Figs. 20P–20T ) (sculpture light in *T. schaumii* (Roger), Fig. 20R); postpetiole ***often without*** an anterior bulge in profile view (except some specimens of *T. schaumii*) (Figs. 20F–20J); broadly distributed**6**
**6.** Antennal scapes long, separated from the posterior margin of head by about 1x maximum width of the antennal scape or less when fully retracted: SI > 80 (see Fig. 21A for diagram, Figs. 21B –21D for examples); ***if unclear*** (some specimens of *T. ambiguus* (Emery)) ***then*** head sculpture areolate-rugulose, ***without*** overlying strong costae (but may be costulate) (Fig. 21B); North America, east of the continental divide***longispinosus* group (Palearctic clade, part)**
**-** Antennal scapes short, separated from the posterior margin of head by >1x maximum width of the antennal scape when fully retracted: SI < 80 (see Fig. 21E for diagram, Figs. 21F–21H for examples); ***if unclear*** (some specimens of *T. rugatulus* (Emery)) ***then*** head sculpture rugulose, ***with*** overlying strong costae (Fig. 21E); widespread***rugatulus* clade**
**7.** Matching the following description: head, mesosoma, and waist segments areolate ***and*** propodeal spines reduced to small teeth or angles ***and*** petiolar peduncle short (Figs. 22A–22D) ***and*** postpetiole moderately broad: PWI 140–160; southern United States and Northern Mexico… ***andersoni* clade**
- Not matching the above description; ***if*** head, mesosoma, and waist segments areolate, ***then*** propodeal spines more developed ***and*** petiolar peduncle longer (Figs. 22E–22H) ***and*** postpetiole broader; widespread**8**
**8.** Postpetiole narrow, less than 1.5 times the maximum width of the petiolar caudal cylinder (PEW) in dorsal view: PWI < 150 (Figs. 23A–23F); if PWI is 150–155, then antennal scapes short, when fully retracted, failing to reach the posterior margin of the head by about two times the maximum width of the antennal scape: SI < 80 (Figs. 24A–24C); **NOTE:** PWI is not always obvious at first glance: careful measurements should be taken; PEW in this article is measured across the caudal cylinder of the petiole**9**
**-** Postpetiole broad, greater than 1.5 times the maximum width of the petiolar caudal cylinder in dorsal view: PWI > 150 (Figs. 23G–23L); if PWI is 150–155, then antennal scapes longer: SI > 80**14**
**9.** Antennal scapes short: when fully retracted, failing to reach the posterior margin of the head by about two times the maximum width of the antennal scape: SI < 80 (Figs. 24A–24C); petiolar node evenly rounded in profile; caudal cylinder of petiole generally elongate (Figs. 24D–24F); arboreal species from the American southwest through Central America and Greater Antilles***obturator* clade, part**
**-** Petiolar node shaped otherwise; if petiolar node is rounded, then antennal scapes long: SI > 80; arboreal or not; broadly distributed**10**
**10.** Propodeum ***without*** erect setae dorsally, but a single pair may arise from the metanotal groove (Figs. 25A & 25B); Chiapas, Mexico**… *salvini* clade, part**
**-** Propodeum ***with*** at least one pair of erect setae dorsally (Figs. 25C & 25D); North America, west of the Great Plains**11**
**11.** Petiolar node not cuneiform (Figs. 26A–26D)***andrei* clade, part**
**-** Petiolar node cuneiform (Figs. 26E–26H)**12**
**12.** Anterior face of petiolar node straight, not concave in profile view (Figs. 27A & 27B); if unclear, posterior margin of head with ten or more erect setae and medial lobe clypeus with three distinct carinae (Fig. 27C)**… *sallei* clade, part**
**-** Anterior of petiolar node concave in profile view (Figs. 27D & 27E); if unclear, posterior of head with 8 or fewer erect setae and medial lobe of clypeus without three distinct carinae (Fig. 27F)**13**
**13.** Medial lobe of clypeus convex anteriorly and bearing three distinct carinae (Fig. 28A); North America, Rocky Mountains***T. furunculus*** (Wheeler) **(Palearctic clade, part)**
**-** Medial lobe of clypeus flat to emarginate anteriorly and not bearing three distinct carinae (several to many indistinct, but median carina not distinct along the majority of the median clypeal lobe; Figs. 28B & 28C); North America, west of the Great Plains***andrei* clade, part**
**14.** Petiole ***without*** tubercles united by a distinct carina anterodorsally: profile of dorsal surface of petiole, when viewed from a ventrolateral angle, not interrupted by a tubercle anteriorly (Fig. 29A); or, if present, postpetiole is broadly attached to anterior margin of gaster (Fig. 29B**)****15**
**-** Petiole ***with*** tubercles united by a distinct carina anterodorsally: profile of dorsal surface of petiole, when viewed from a ventrolateral angle, interrupted by a tubercle anteriorly (Fig. 29C); postpetiole not broadly attached to anterior margin of gaster (Fig. 29D)**19**
**15.** Dorsum of mesosoma evenly convex in profile; dorsum of propodeum rounding evenly into the propodeal declivity; propodeum either entirely ***without*** teeth or spines (Figs. 30A–30C) ***or*** with small teeth, in which case the teeth ***do not*** mark an angulate transition between the dorsum and declivity of the propodeum (Fig. 30D); Central America, Cuba, and Puerto Rico***sallei* clade, part**
**-** Dorsum of mesosoma in profile variable: evenly convex or not; propodeum bearing teeth or spines or, if uncertain, transition from dorsum to declivity of propodeum is marked by an angle, not rounded as above; broadly distributed**16**
**16.** Matching the following description: propodeal teeth reduced, or transition from dorsum to declivity of propodeum is marked by an angle (Figs. 31A & 31B); legs moderately incrassate (FI < 300), and without abundant erect to suberect setae (Figs. 31C & 31D); petiolar node evenly rounded in profile (Figs. 31A & 31B); mid to high elevations, from central Mexico to the Nicaraguan depression***sallei* clade, part**
**-** Not matching the above description; broadly distributed**17**
**17.** Matching the following description: clypeus forming wall between antennal insertions and mandibles (Figs. 32A–32C); anterior margin of clypeus flat; posterior margin of head rounding evenly into lateral margins; legs not incrassate (Figs. 32G–32I); southwestern United States and northwestern Mexico***sallei* clade, part**
- Not matching the above description. If specimen is from southwestern United States and northwestern Mexico and the clypeal character is unclear, then the anterior margin of clypeus emarginated, posterior margin of head transitions to lateral margins through a rounded angle (Figs. 32D–32F), and legs strongly incrassate (Figs. 32J–32L); broadly distributed**18**
**18.** Postpetiole campaniform in dorsal view (Fig. 33A); legs with abundant suberect to erect setae (Fig. 33B); if petiole squamiform and increasing in width toward the apex (Fig. 33A), medial lobe of clypeus with 3 or fewer distinct carinae (Figs. 33C & 33D); southern Florida, Bahamas, Cuba, Hispaniola***sallei* clade, part**
**-** Postpetiole variable; legs usually without abundant suberect to erect setae; ***if*** present (Fig. 33F) ***and*** petiolar node squamiform and increasing in width toward the apex (Fig. 33E) ***then*** medial lobe of clypeus with many fine carinae (Fig. 33G) ***or*** propodeum is strongly depressed (Fig. 33H); broadly distributed***salvini* clade, part**
**19.** Postpetiole with a distinctive trapezoidal shape in dorsal view: anteriorly broad, with angulate anterolateral corners (Fig. 34A); southwestern North America***T. quasimodo* Snelling et al. (*andrei* clade, part)**
**-** Postpetiole in dorsal view not as above (Figs. 34B–34D); broadly distributed**20**
**20.** Medial lobe of clypeus smooth medially ***or*** bearing three distinct carinae, although these may be accompanied by additional weaker, incomplete carinae (Figs. 35A–35C)***sallei* clade, part**
**-** Medial lobe of clypeus bearing many fine carinae (Figs. 35D–35F), although these may be in addition to the three strong carinae (Fig. 35F)**21**
**21.** Metafemora incrassate: FI > 325 (Fig. 36A); or, if not incrassate, erect setae absent from the propodeal dorsum (Figs. 25A & 25B)***salvini* clade, part**
**-** Metafemora not incrassate FI < 325 (Fig. 36B); erect setae always present on the propodeal dorsum (Figs. 25C & 25D)***sallei* clade, part**

### Diagnosis of the *salvini* clade based on the worker caste

The workers of the *salvini* clade lack a singular diagnostic feature, but can be diagnosed with the following combination of characters:

1. Antennae composed of 12 antennomeres.
2. Postpetiole moderately to very broad: 1.4 to 3 times as broad as the caudal cylinder in dorsal view (PWI 140-300); if less than 1.5 times, then antennal scapes are moderately short, failing to reach the posterior margin of the head by the maximum width of the antennal scape or less, and dorsum of propodeum lacks standing setae.
3. Anterodorsal region of petiole with or without tubercles that are united by a transverse carina; if tubercles present, then median lobe of clypeus bearing many (> 3) fine carinae, femora incrassate (FI > 250), or, if femora not incrassate, then dorsum of propodeum lacks standing setae.
4. Propodeum bearing teeth or spines; or, if propodeum is unarmed, then propodeum is strongly depressed below the level of the evenly convex promesonotum.
5. Legs usually without long tapering setae, but if present, then medial lobe of clypeus bearing many (> 3) longitudinal carinae, or propodeum is strongly depressed below the level of the evenly convex promesonotum.

### Key to species of the *salvini* clade based on the worker caste

Two species, both described from Cuba, that are potentially members of the *salvini* clade were unable to be observed for this study. Therefore, *Temnothorax cuyaguateje* (Fontenla) and *T. imias* (Fontenla) are *incertae sedis* in *Temnothorax*.

**Figure 37:**
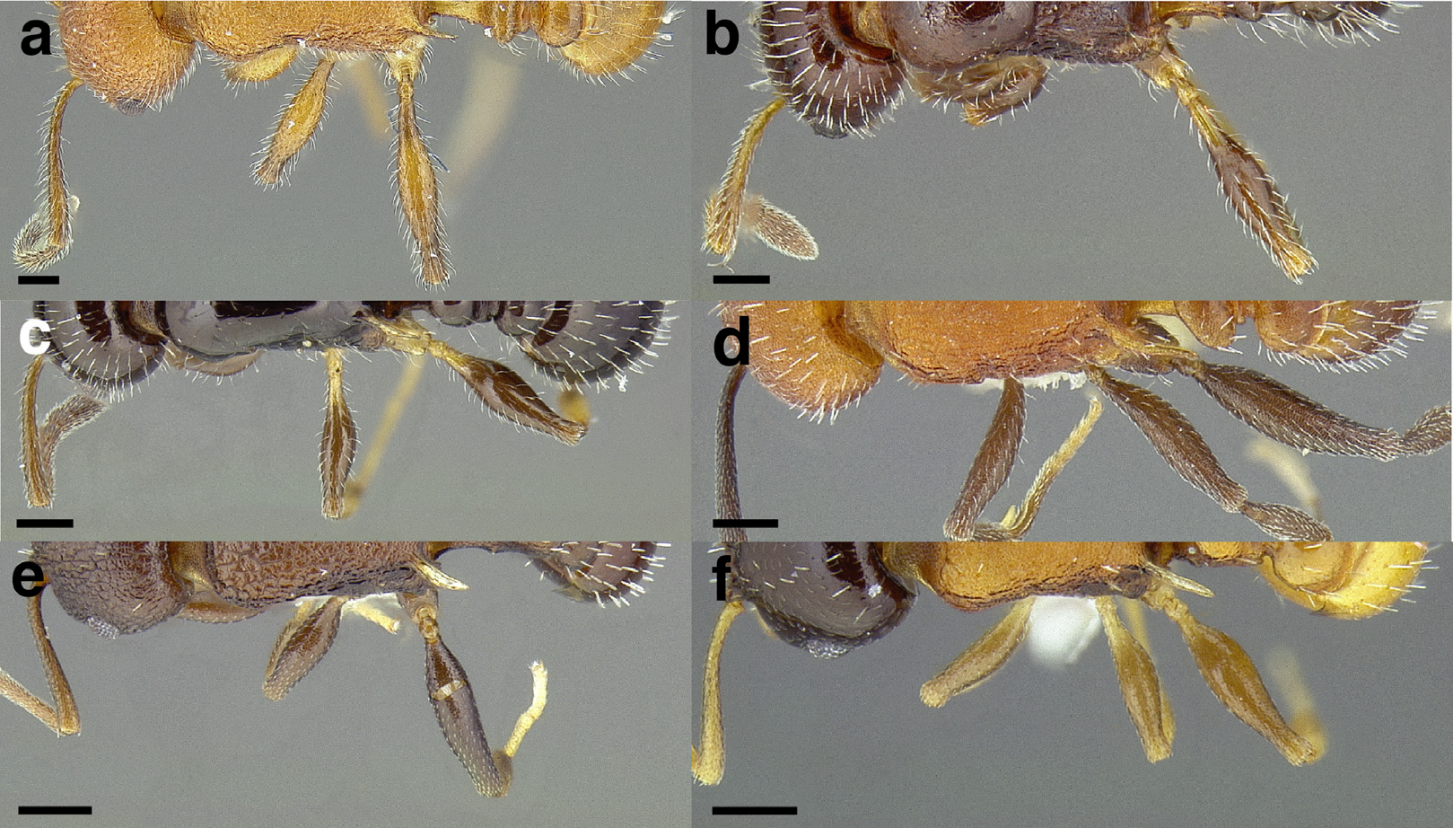
Comparison of setae morphology of *salvini* clade workers in dorsal view. (A) *Temnothorax aztecoides* sp. nov. (CASENT0758791). (B) *T. pergandei* (CASENT0758291). (C) *T. politus* (CASENT0619368). (D) *T. rutabulafer* sp. nov. (CASENT0758266). (E) *T. leucacanthoides* sp. nov. (CASENT0756102). (F) *T. wilsoni* sp. nov. (MCZENT00583608). Scale bars 0.2 mm.

**Figure 38:**
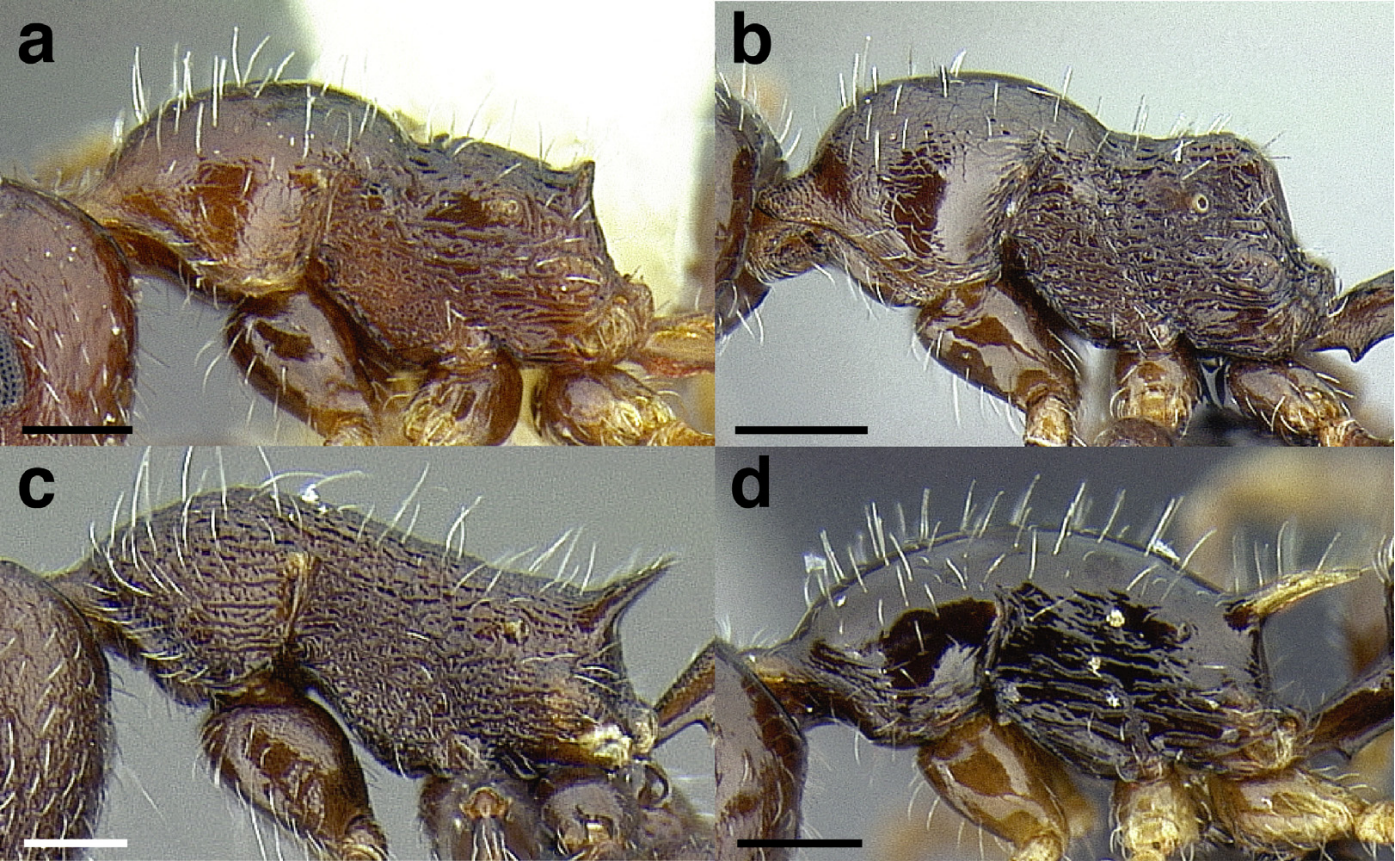
Comparison of mesosoma morphology of *salvini* clade workers in profile view. (A) *Temnothorax pergandei* (CASENT0758291). (B) *T. bison* sp. nov. (CASENT0636947). (C) *T. longinoi* sp. nov. (CASENT0758892). (D) *T. politus* (CASENT0756845). Scale bars 0.2 mm.

**Figure 39:**
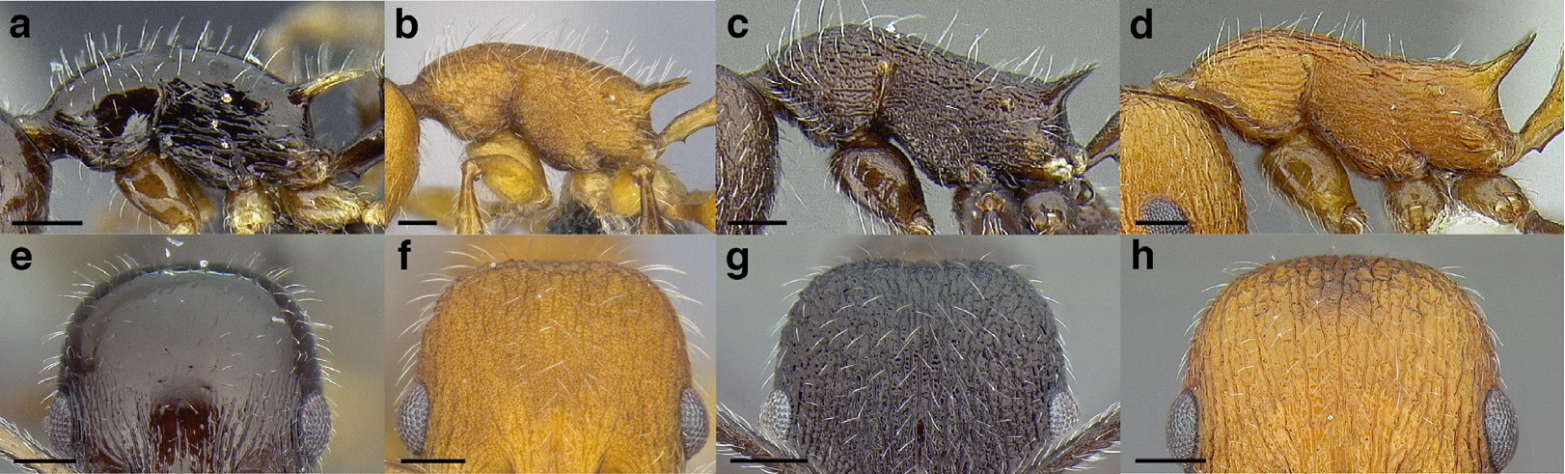
Comparison of mesosoma morphology and head sculpture of *salvini* clade workers. (A–D) Profile view. (E–H) Full-face view. (A) *Temnothorax politus* (CASENT0756845). (B) *T. terraztecus* sp. nov. (CASENT0868966). (C) *T. longinoi* sp. nov. (CASENT0758892). (D) *T. quetzal* sp. nov. (CASENT0614495). (E) *T. politus* (CASENT0756845). (F) *T. terraztecus* sp. nov. (CASENT0868966) (G) *T. longinoi* sp. nov. (CASENT0758892). (H) *T. quetzal* sp. nov. (CASENT0614495). Scale bars 0.2 mm.

**Figure 40:**
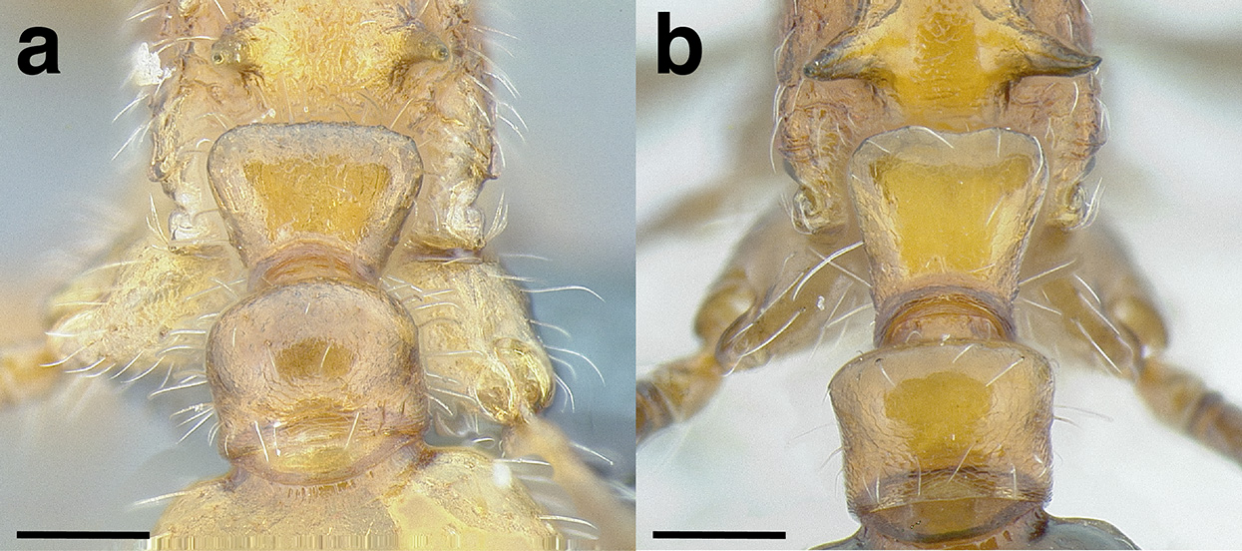
Comparison of petiolar node morphology in the *salvini* species group in posterodorsal view. (A) *Temnothorax aztecoides* sp. nov. (CASENT0758791). (B) *T. quetzal* sp. nov. (CASENT0614495). Scale bars 0.2 mm.

**Figure 41:**
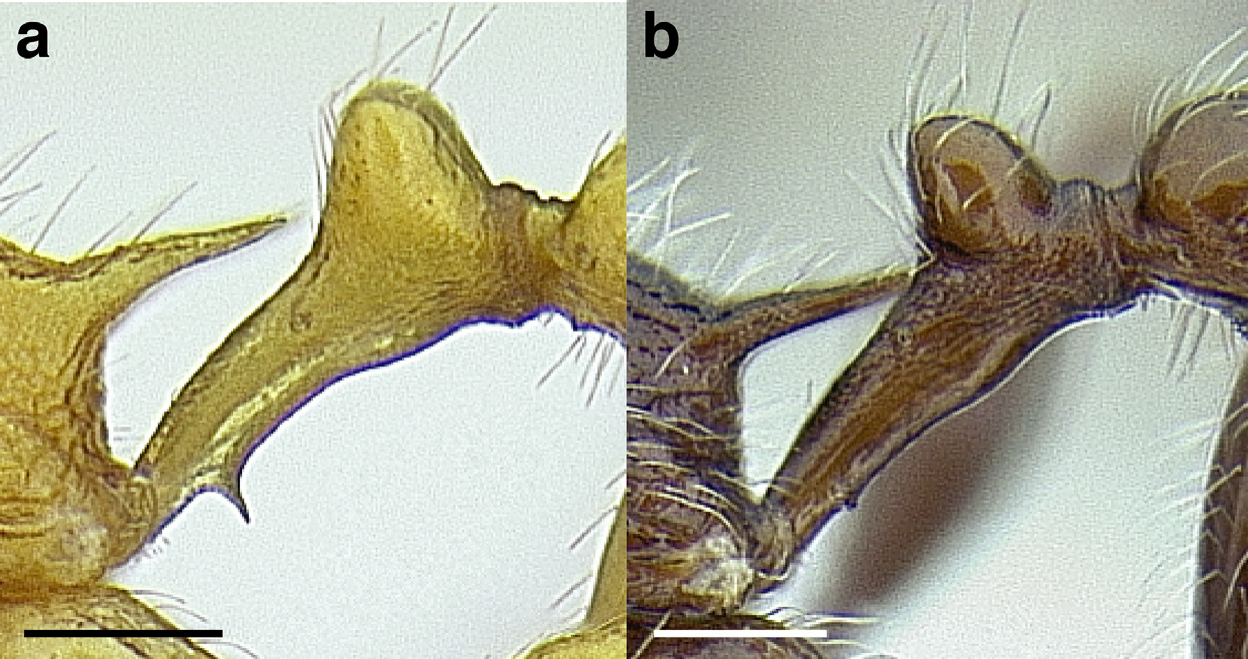
Comparison of subpetiolar tooth morphology in the *salvini* species group in profile view. (A) *Temnothorax aztecus* (CASENT0758793). (B) *T. longicaulis* stat. nov., nom. nov. (CASENT0758334). Scale bars 0.2 mm.

**Figure 42:**
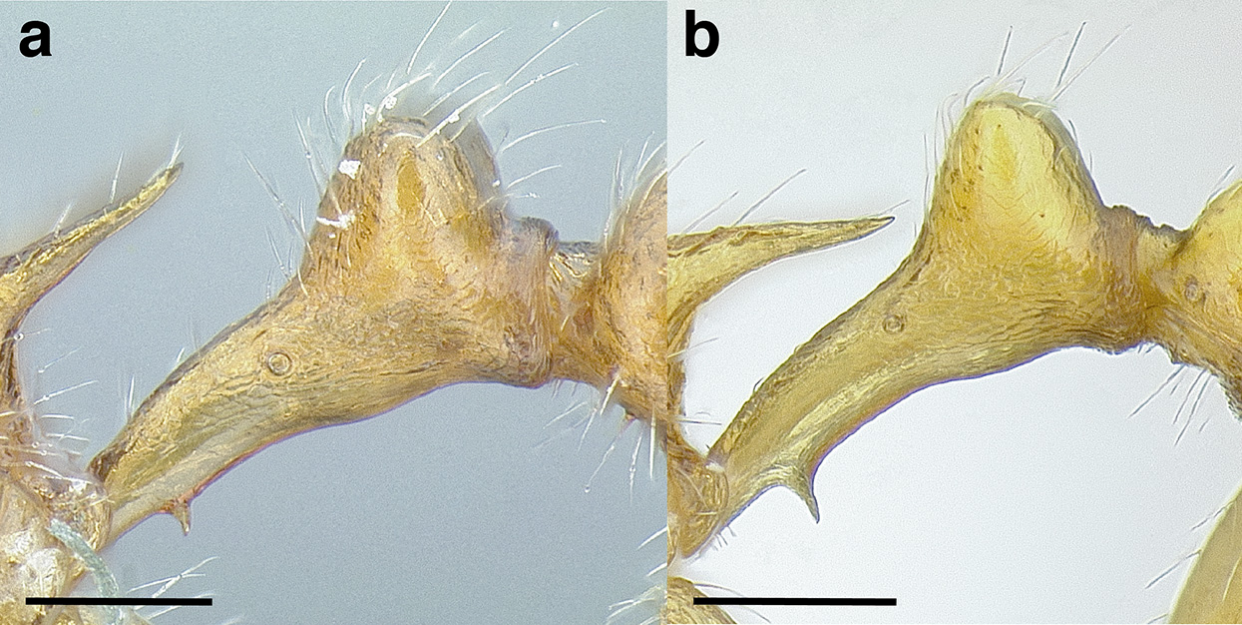
Comparison of worker petiole setae in the *salvini* species group in profile view. (A) *Temnothorax aztecoides* sp. nov. (CASENT0758791). (B) *T. aztecus* (CASENT0758793). Scale bars 0.2 mm.

**Figure 43:**
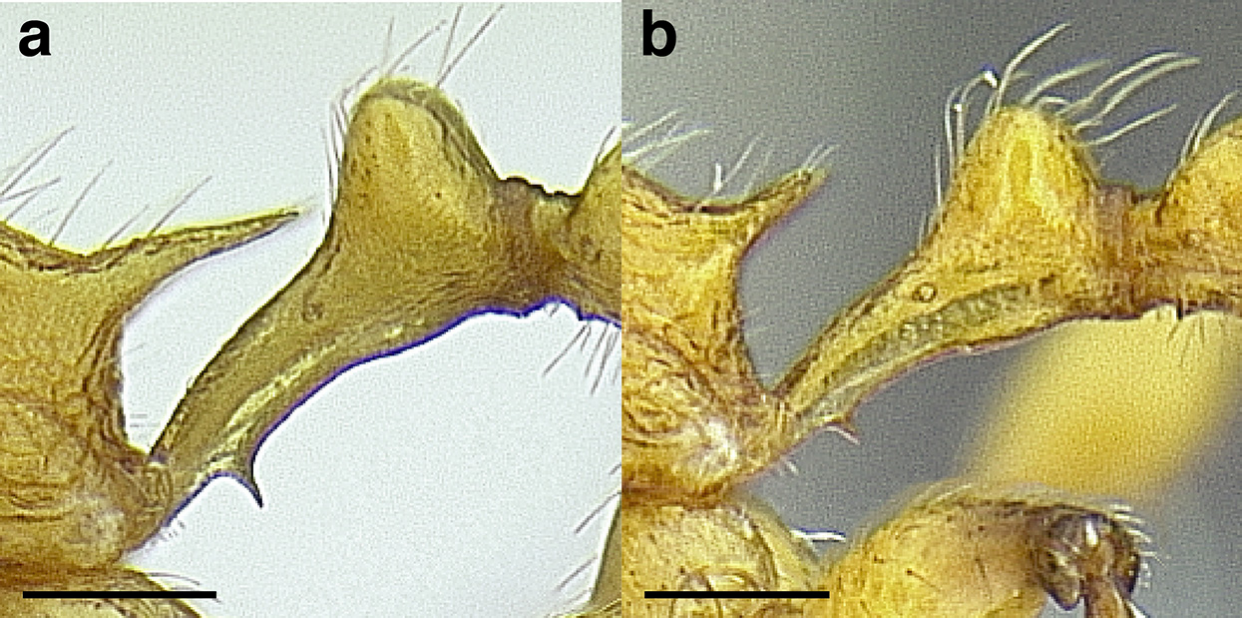
Comparison of worker propodeal spine morphology in the *salvini* species group in profile view. (A) *Temnothorax aztecus* (CASENT0758793). (B) *T. paraztecus* sp. nov. (CASENT0629030). Scale bars 0.2 mm.

**Figure 44:**
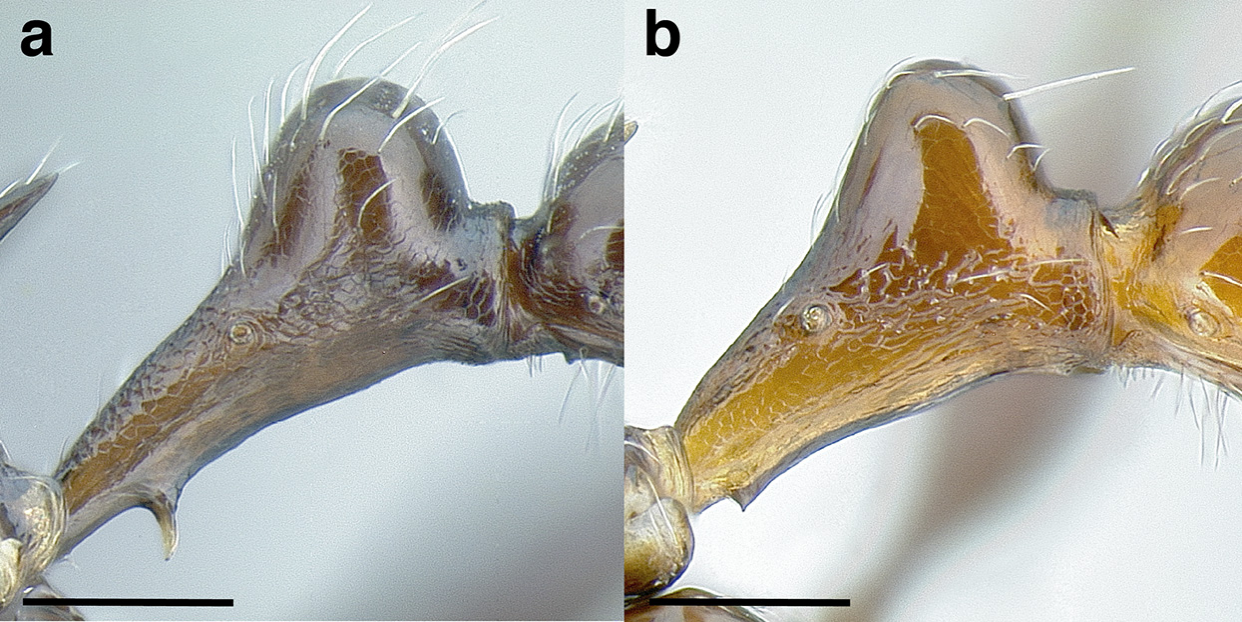
Comparison of worker subpetiolar tooth morphology in the *salvini* species group in profile view. (A) *Temnothorax longinoi* sp. nov. (CASENT0758892). (B) *T. salvini* (CASENT0756074). Scale bars 0.2 mm.

**Figure 45:**
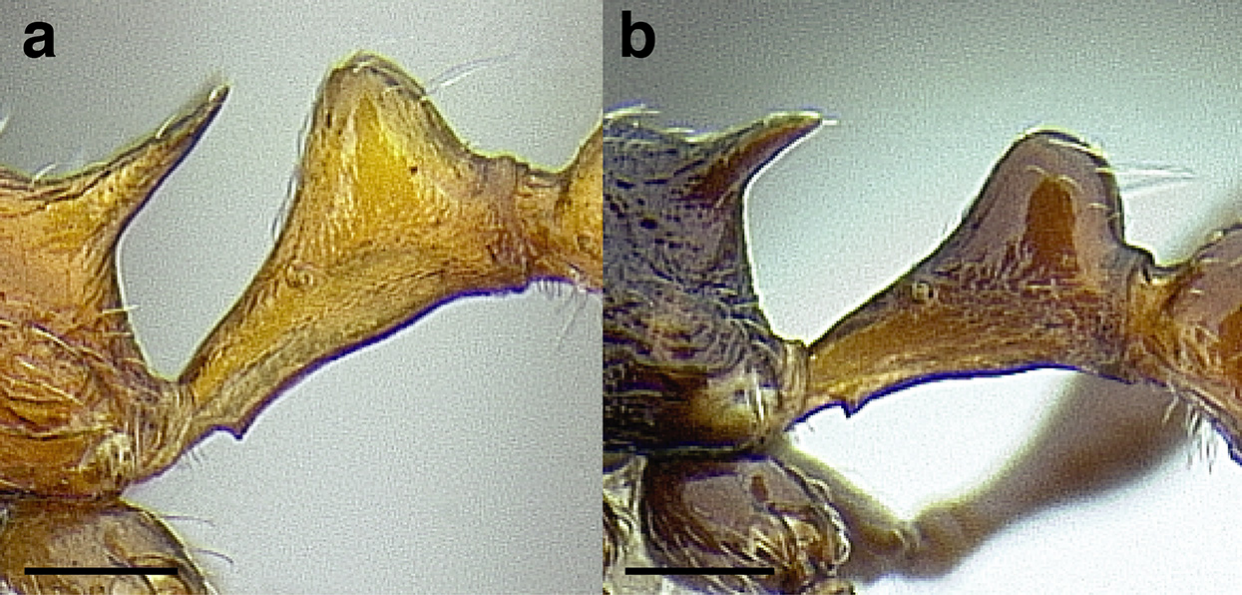
Comparison of worker propodeal spine morphology in the *salvini* species group in profile view. (A) *Temnothorax quetzal* sp. nov. (CASENT0614495). (B) *T. salvini* (CASENT0756074). Scale bars 0.2 mm.

**Figure 46:**
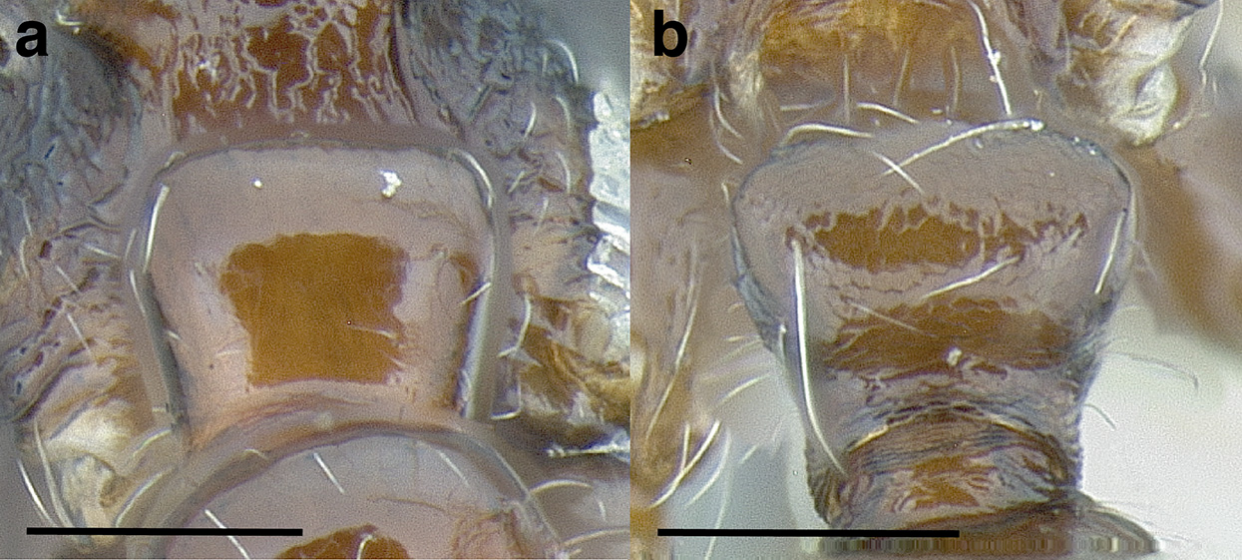
Comparison of worker petiolar node morphology in the *salvini* species group in posterodorsal view. (A) *Temnothorax salvini* (CASENT0756074). (B) *T. parvidentatus* sp. nov. (CASENT0756087). Scale bars 0.2 mm.

**Figure 47:**
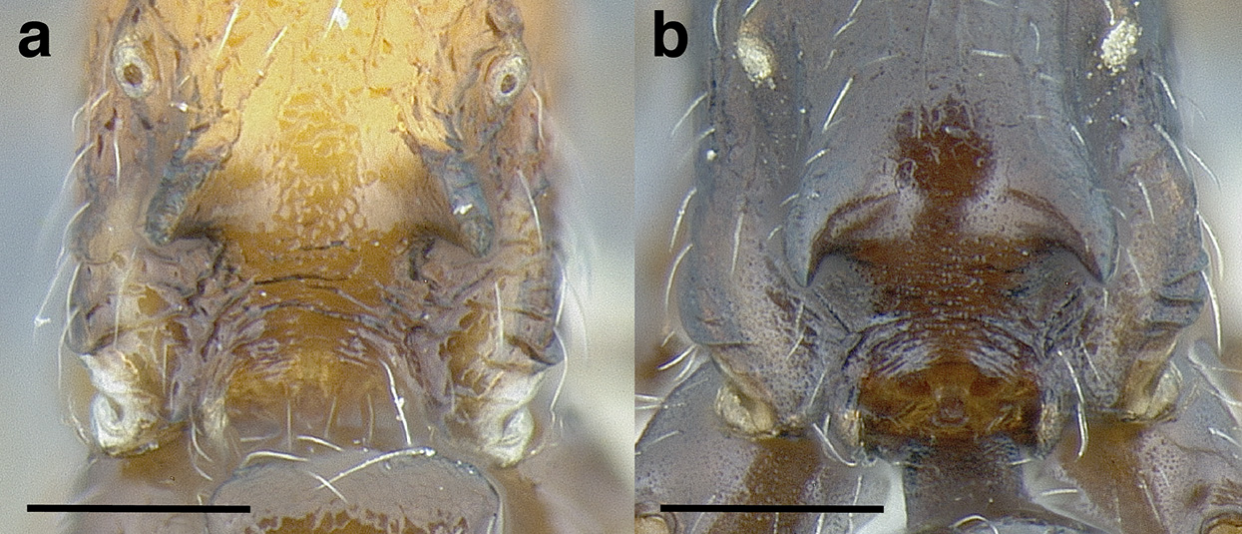
Comparison of worker propodeal declivity sculpture in the *salvini* species group in posterodorsal view. (A) *Temnothorax parvidentatus* sp. nov. (CASENT0756087). (B) *T. fortispinosus* sp. nov. (JTLC000010282). Scale bars 0.2 mm.

**Figure 48:**
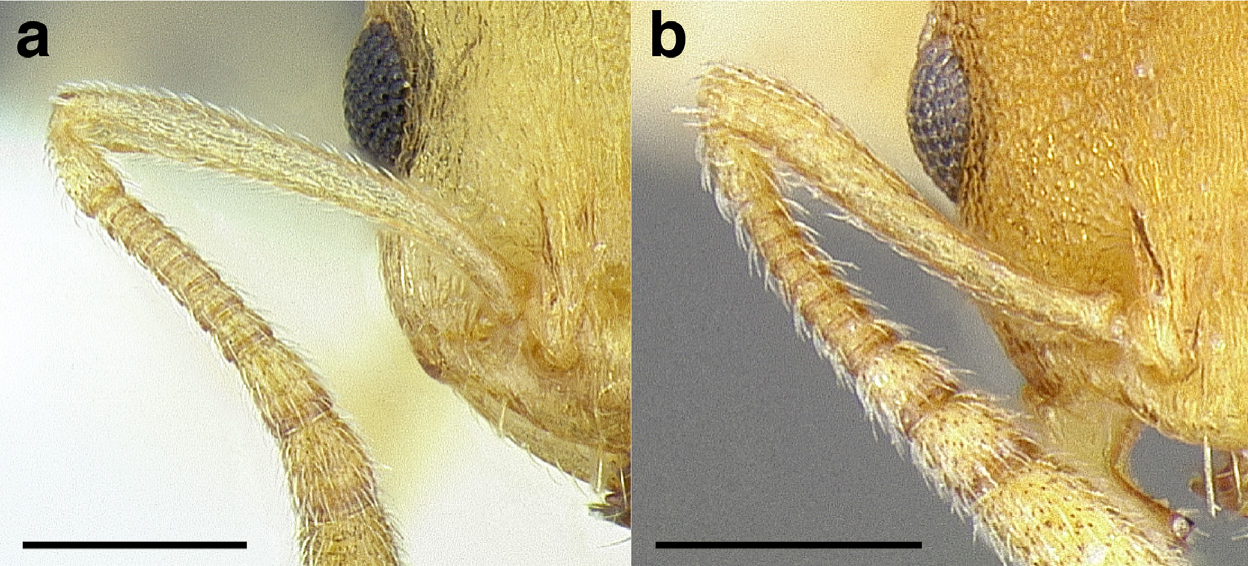
Comparison of worker antennal scape pilosity in the *salvini* clade in full-face view. (A) *Temnothorax pilicornis* sp. nov. (CASENT0756186). (B) *T. goniops* (MCZENT00032436). Scale bars 0.2 mm.

**Figure 49:**
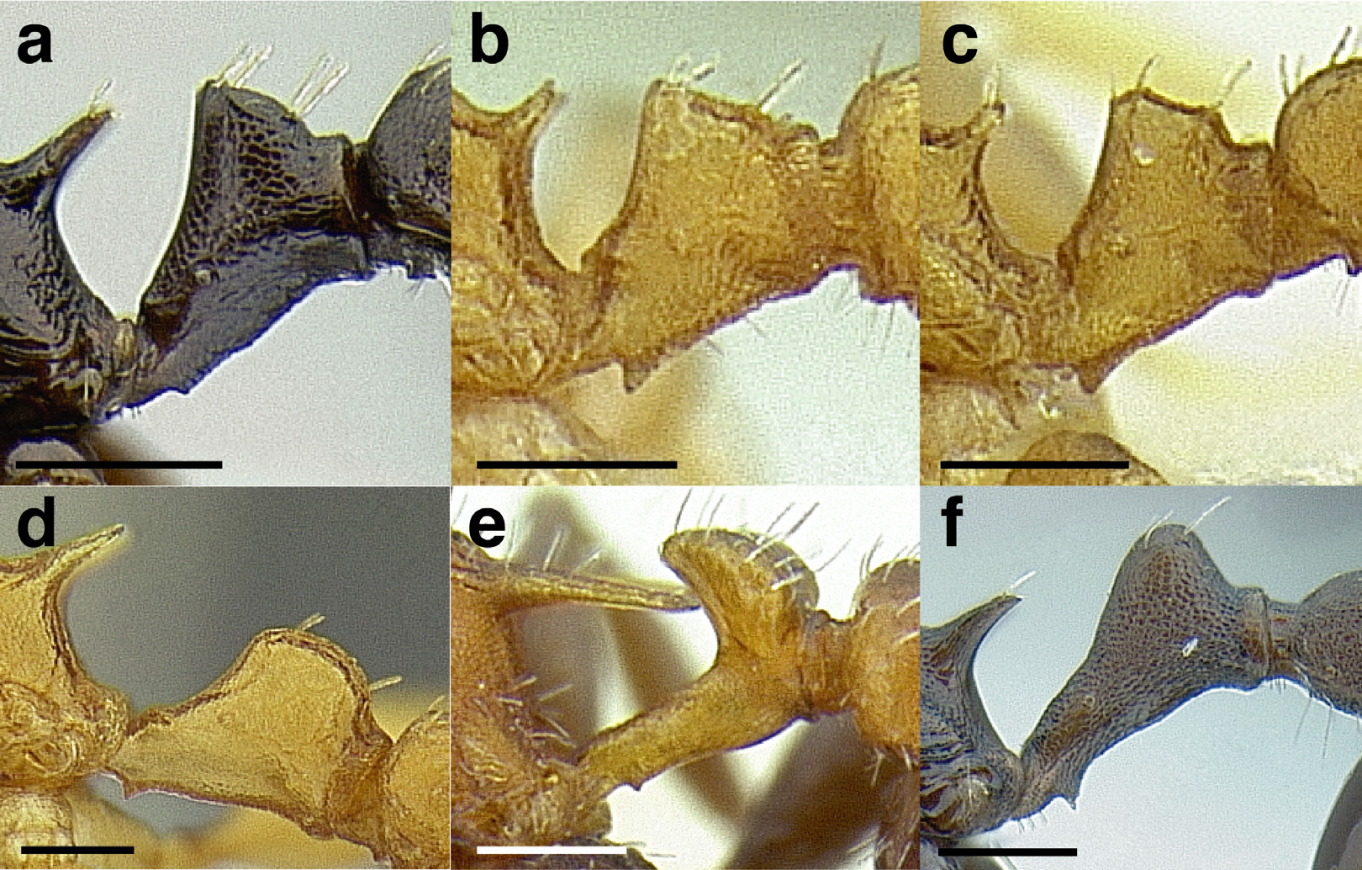
Comparison of worker petiolar node morphology in the *salvini* clade in profile view. (A) *Temnothorax acuminatus* sp. nov. (JTLC000007439). (B) *T. rugosus* (LACMENT323296). (C) *T. rugosus* (LACMENT323260). (D )*T. obtusigaster* sp. nov. (CASENT0758654). (E) *T. rutabulafer* sp. nov. (CASENT0758266). (F) *T. fuscatus* (CASENT0625205). Scale bars 0.2 mm.

**Figure 50:**
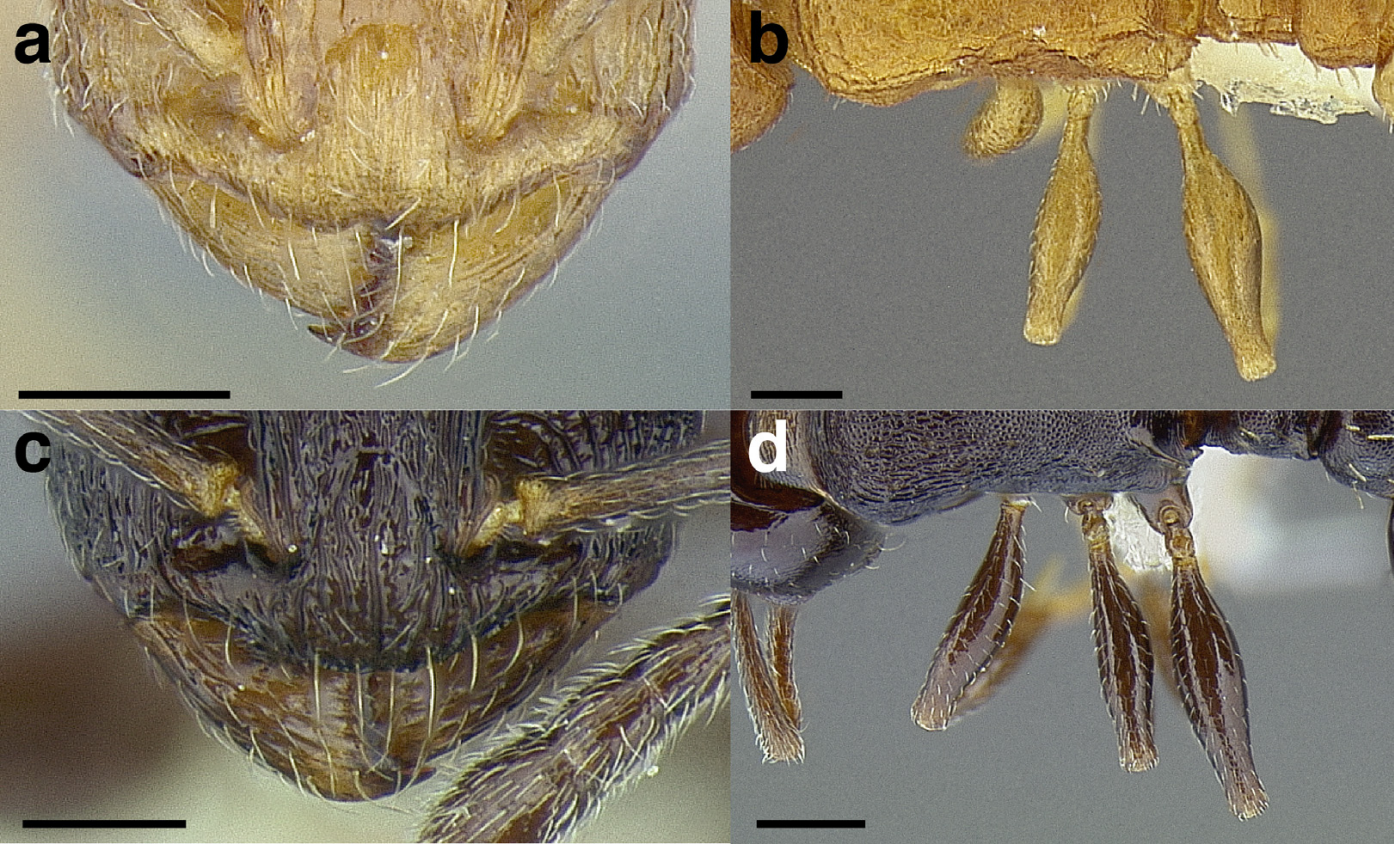
Comparison of clypeus and femur morphology in the *salvini* clade. (A) *Temnothorax rugosus* gyne, full-face view (LACMENT323259). (B) *T. rugosus* worker, dorsal view (LACMENT323260). (C) *T. acuminatus* sp. nov. worker, full-face view (JTLC000007439). (D) *T. acuminatus* sp. nov. worker, dorsal view (JTLC000007439). Scale bars 0.2 mm.

**Figure 51:**
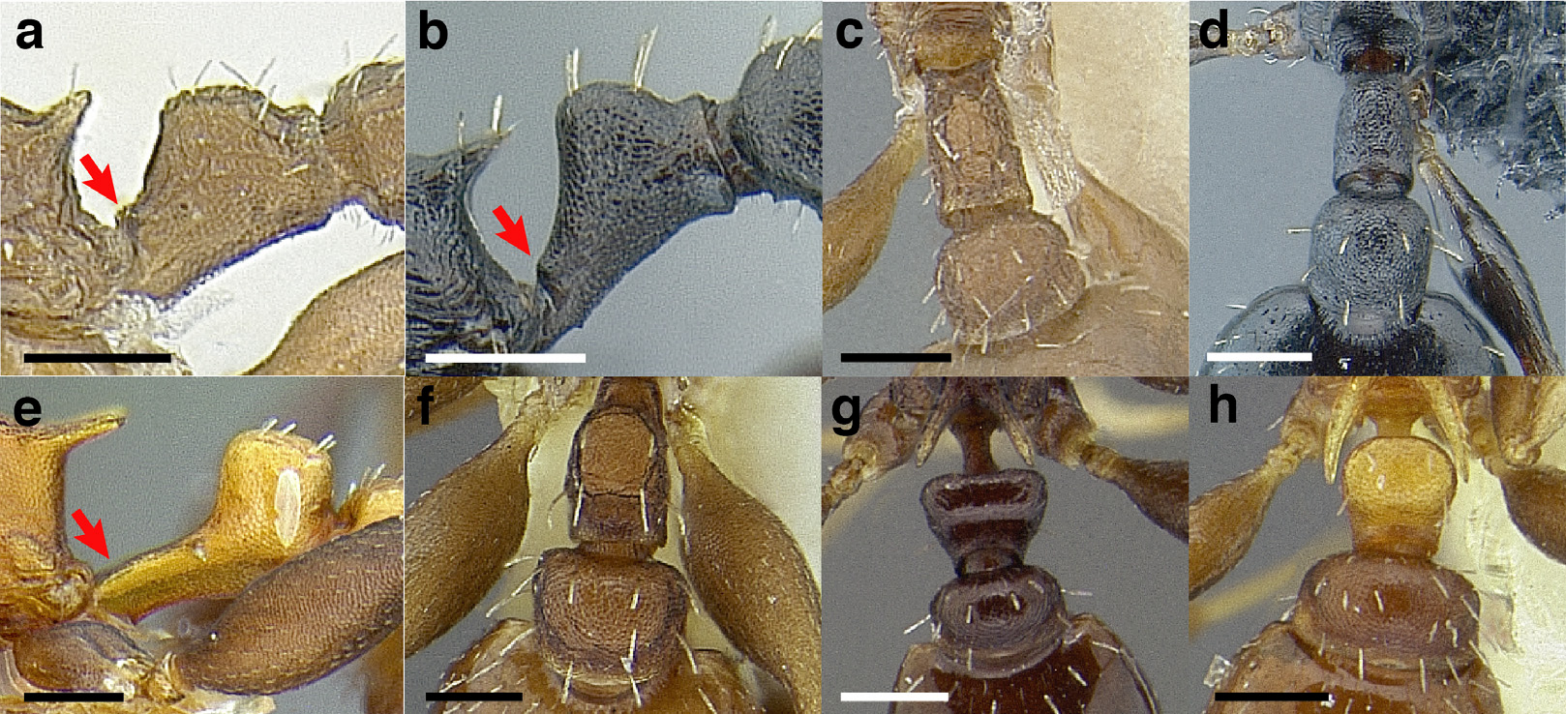
Comparison of worker waist segment morphology in the *salvini* clade. (A) *Temnothorax parralensis* sp. nov., profile view (LACMENT323355). (B) *T. tuxtlanus* sp. nov., profile view (CASENT0640472). (C) *T. parralensis* sp. nov., dorsal view (LACMENT323355). (D) *T. tuxtlanus* sp. nov., dorsal view (CASENT0640472). (E) *T. casanovai* sp. nov., profile view (LACMENT323462). (F) *T. balnearius* sp. nov., dorsal view (LACMENT323199). (G) *T. subditivus*, dorsal view (CASENT0758340). (H) *T. androsanus*, dorsal view (MCZENT00021003). Scale bars 0.2 mm.

**Figure 52:**
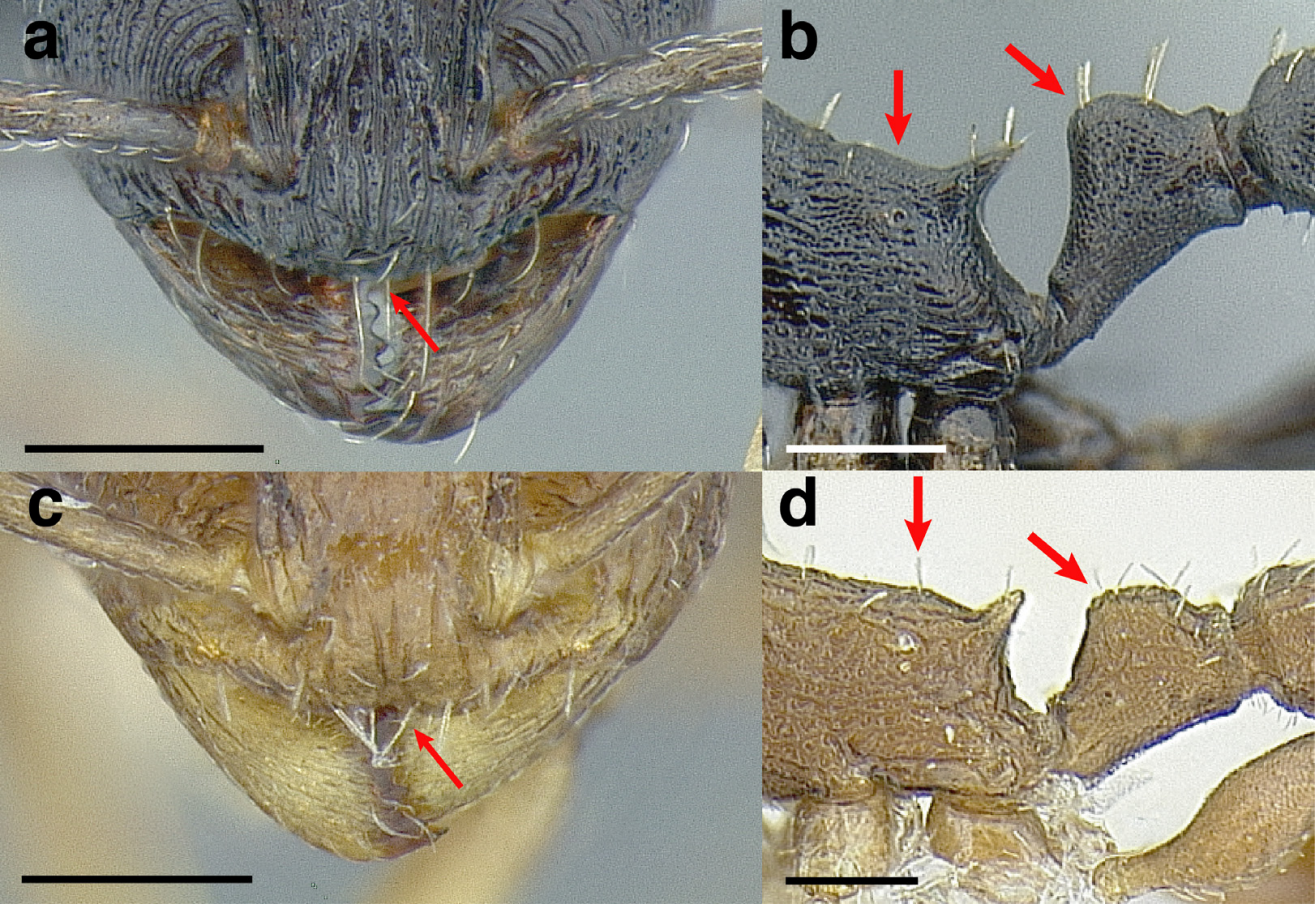
Comparison of worker clypeus and petiole morphology in the *salvini* clade. *Temnothorax tuxtlanus* sp. nov. (CASENT0640472). (A) Full-face view. (B) Profile view. *T. parralensis* sp nov. (LACMENT323355). (C) Full-face view. (D) Profile view. Scale bars 0.2 mm.

**Figure 53:**
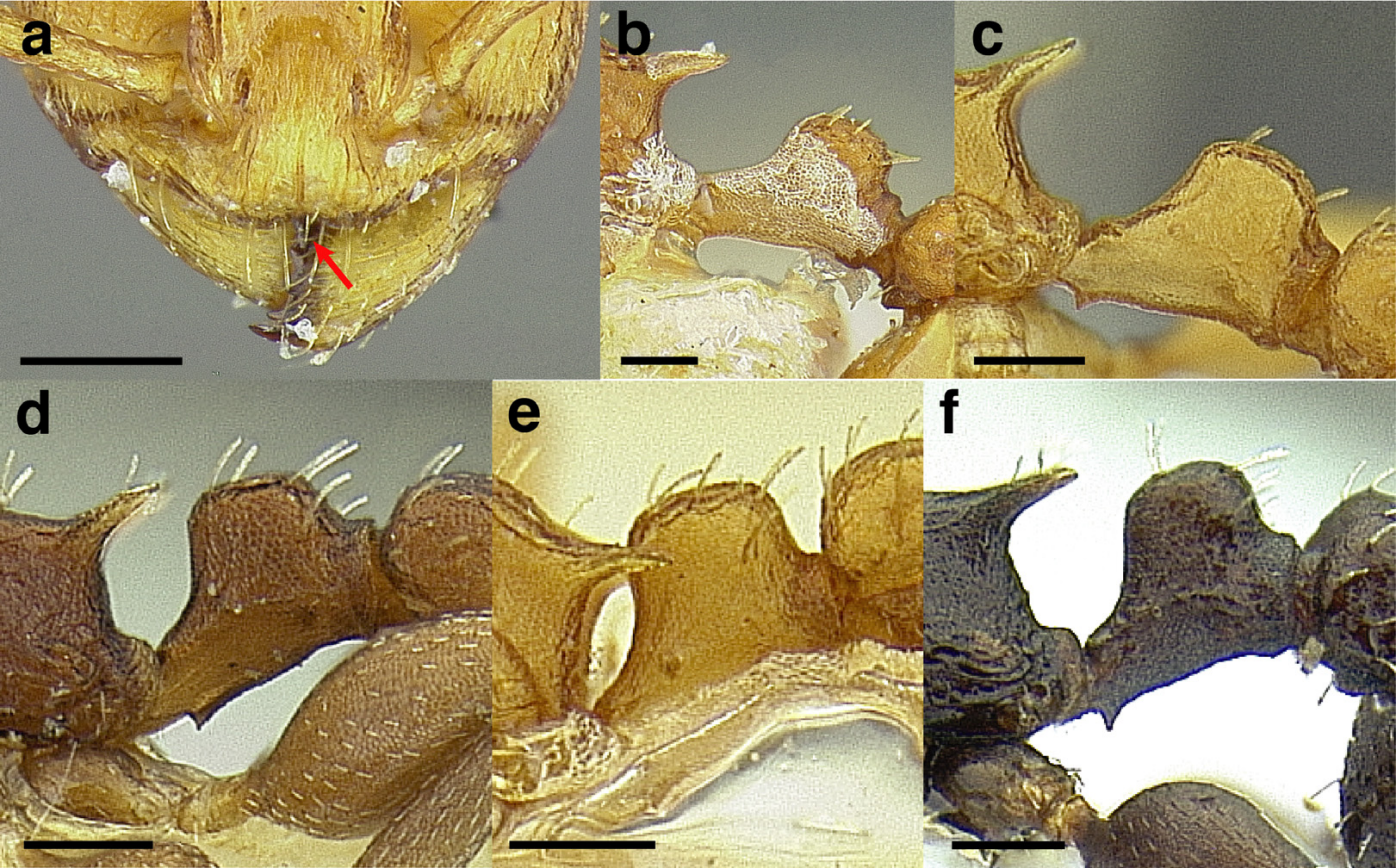
Worker clypeus and petiole morphology of the *annexus* species group. (A) *Temnothorax quercicola* sp. nov., full-face view (LACMENT323216). (B) *T. annexus*, profile view (MCZENT00561747). (C) *T. obtusigaster* sp. nov., profile view (CASENT0758654). (D) *T. balnearius* sp. nov., profile view (LACMENT323199). (E) *T. quercicola* sp. nov., profile view (LACMENT323216). (F) *T. arbustus* sp. nov., profile view (CASENT0758357). Scale bars 0.2 mm.

**Figure 54:**
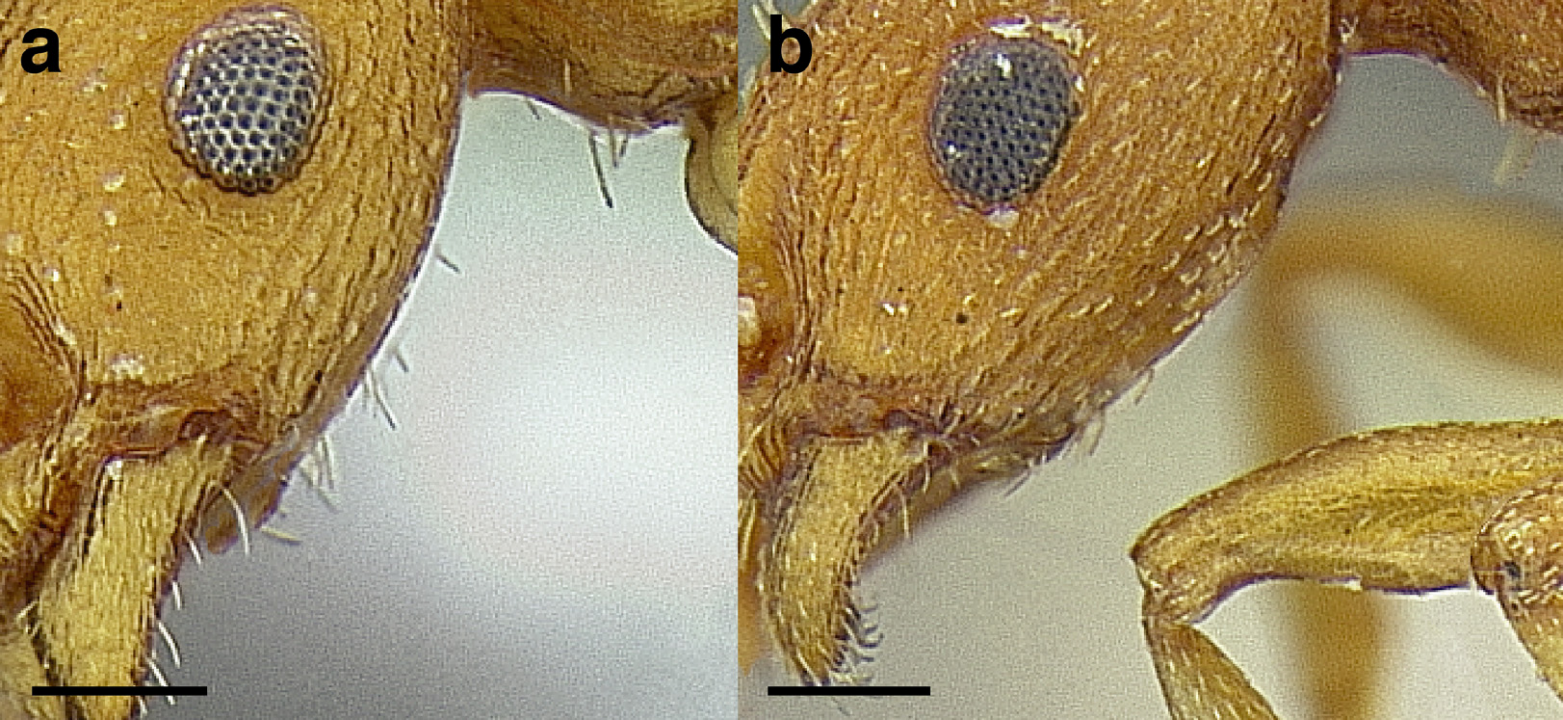
Comparison of worker gular setae in the *annexus* species group in profile view. (A) *Temnothorax obtusigaster* sp. nov. (CASENT0758654). (B) *T. annexus* (MCZENT00561747). Scale bars 0.2 mm.

**Figure 55:**
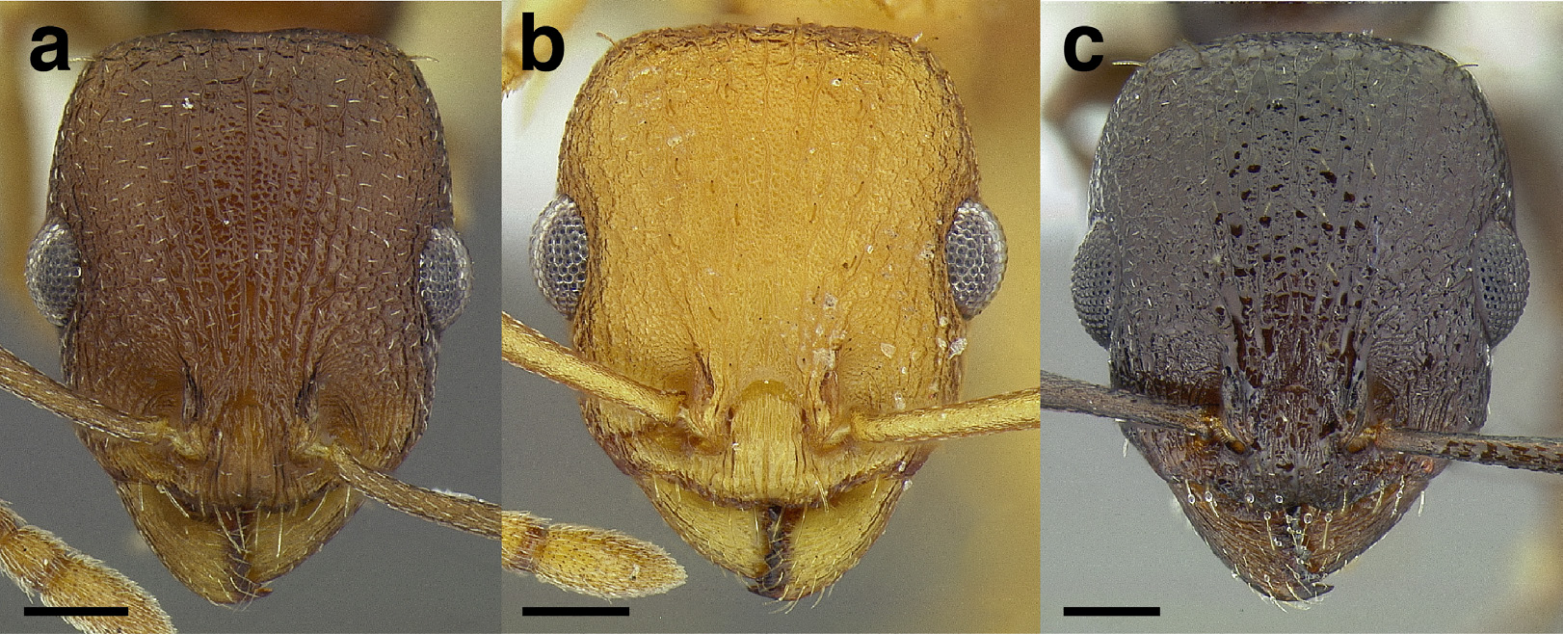
Comparison of worker head morphology in the *annexus* species group in full-face view. (A) *Temnothorax balnearius* sp. nov. (LACMENT323199). (B) *T. obtusigaster* sp. nov., (CASENT0758654). (C) *T. arbustus* sp. nov. (CASENT0758357). Scale bars 0.2 mm.

**Figure 56:**
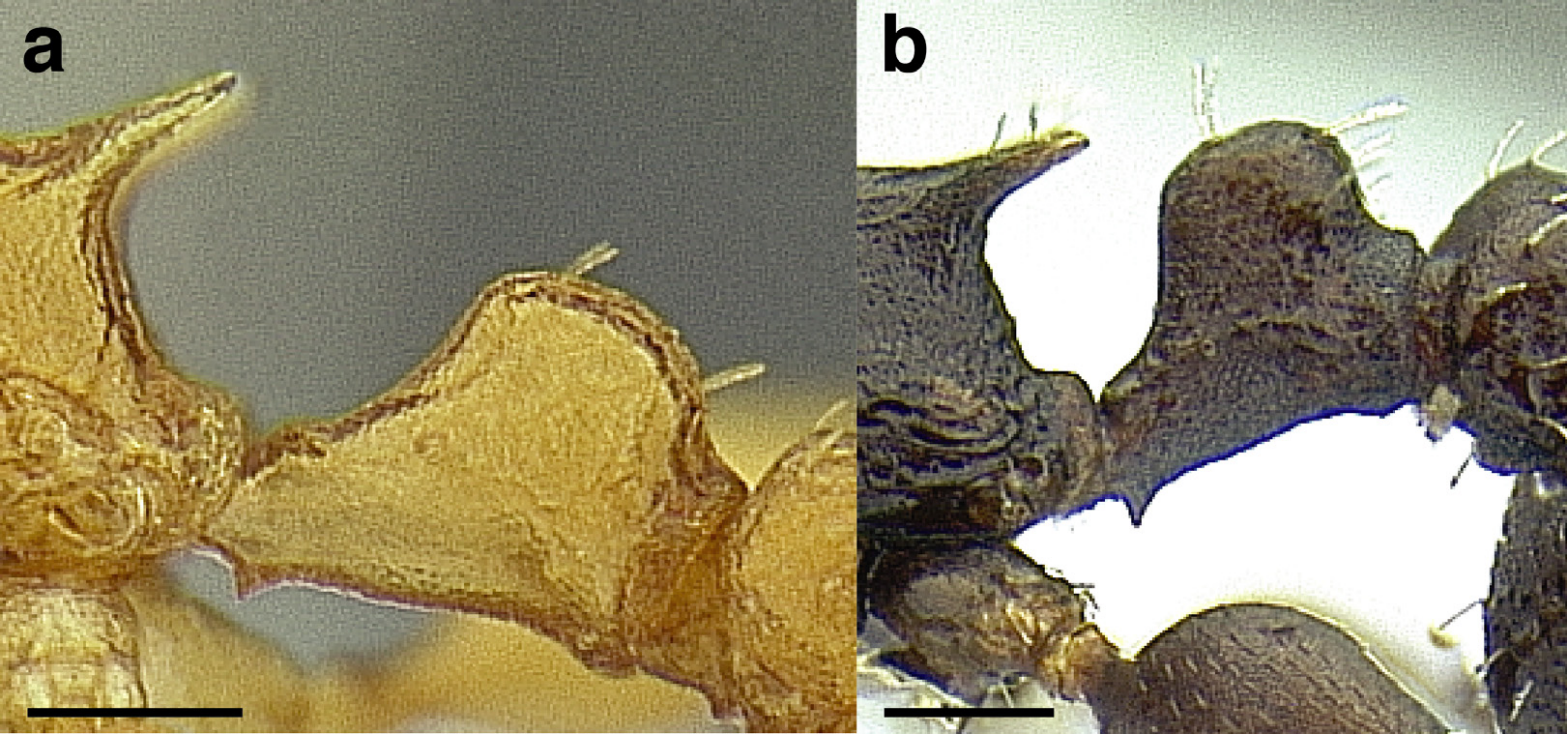
Comparison of worker petiole morphology in the *annexus* species group in profile view. (A) *Temnothorax obtusigaster* sp. nov. (CASENT0758654). (B) *T. arbustus* sp. nov. (CASENT0758357). Scale bars 0.2 mm.

**Figure 57:**
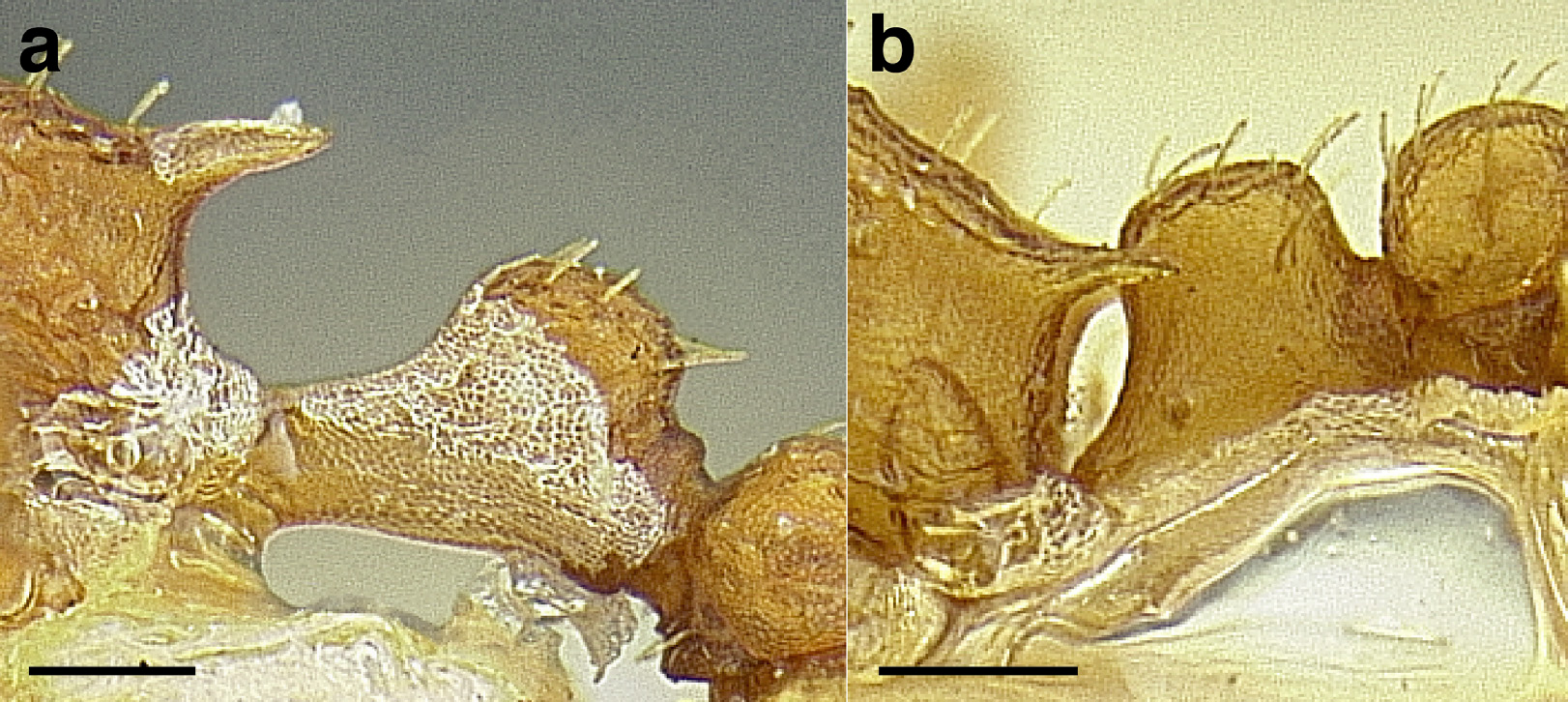
Comparison of worker petiole morphology in the *annexus* species group in profile view. (A) *Temnothorax annexus* (MCZENT00561747). (B) *T. quercicola* sp. nov. (LACMENT323216). Scale bars 0.2 mm.

**Figure 58:**
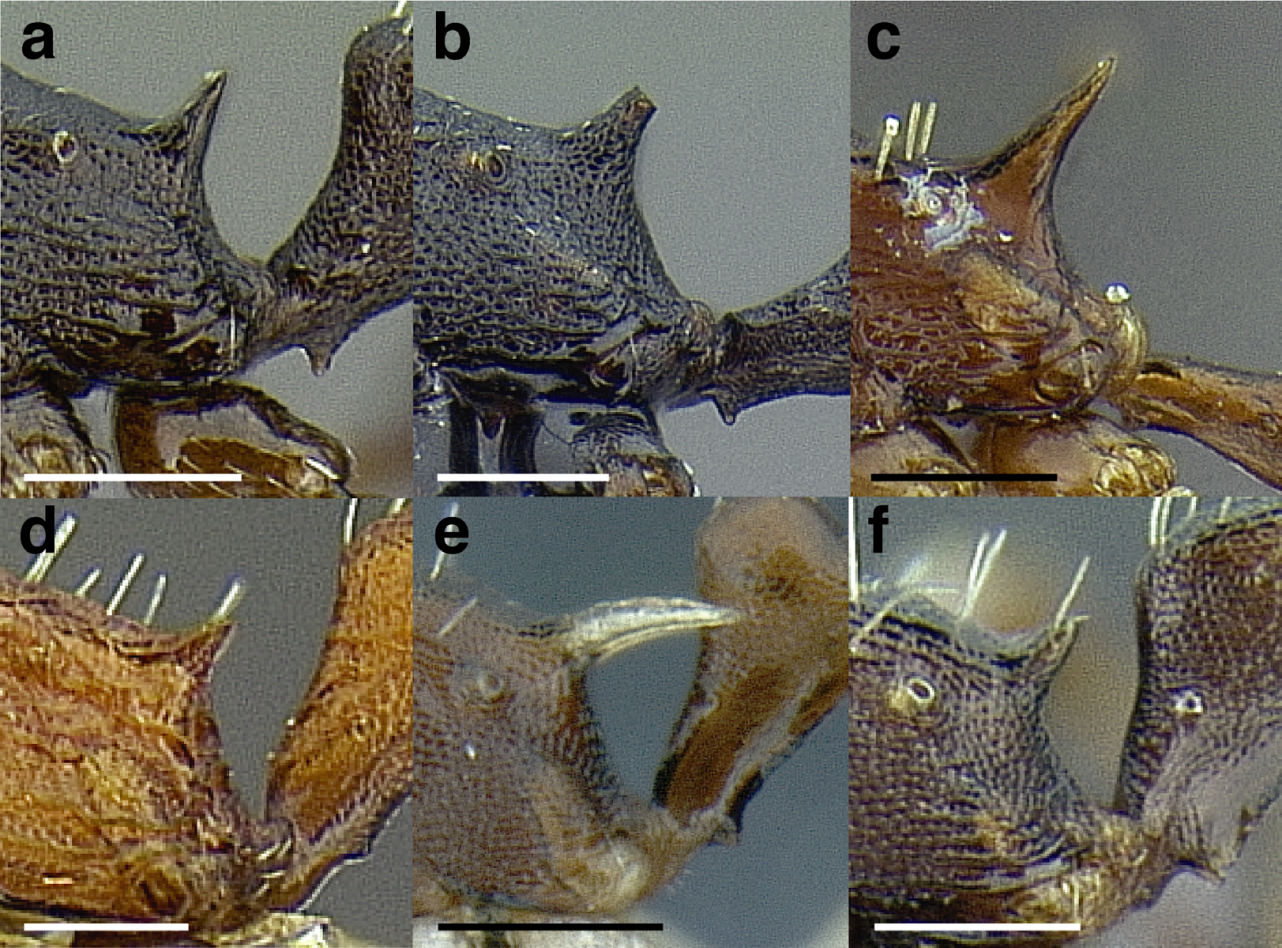
Comparison of worker propodeal spine morphology in the *salvini* clade in profile view. (A) *Temnothorax acutispinosus* sp. nov. (JTLC000007447). (B) *T. altinodus* sp. nov. (CASENT0617708; propodeal spine broken). (C) *T. tenuisculptus* (MCZENT00032435). (D) *T. ocarinae* (USNMENT00531636). (E) *T. albispinus* (CASENT0756097). (F) *T. laticrus* sp. nov. (CASENT0758263). Scale bars 0.2 mm.

**Figure 59:**
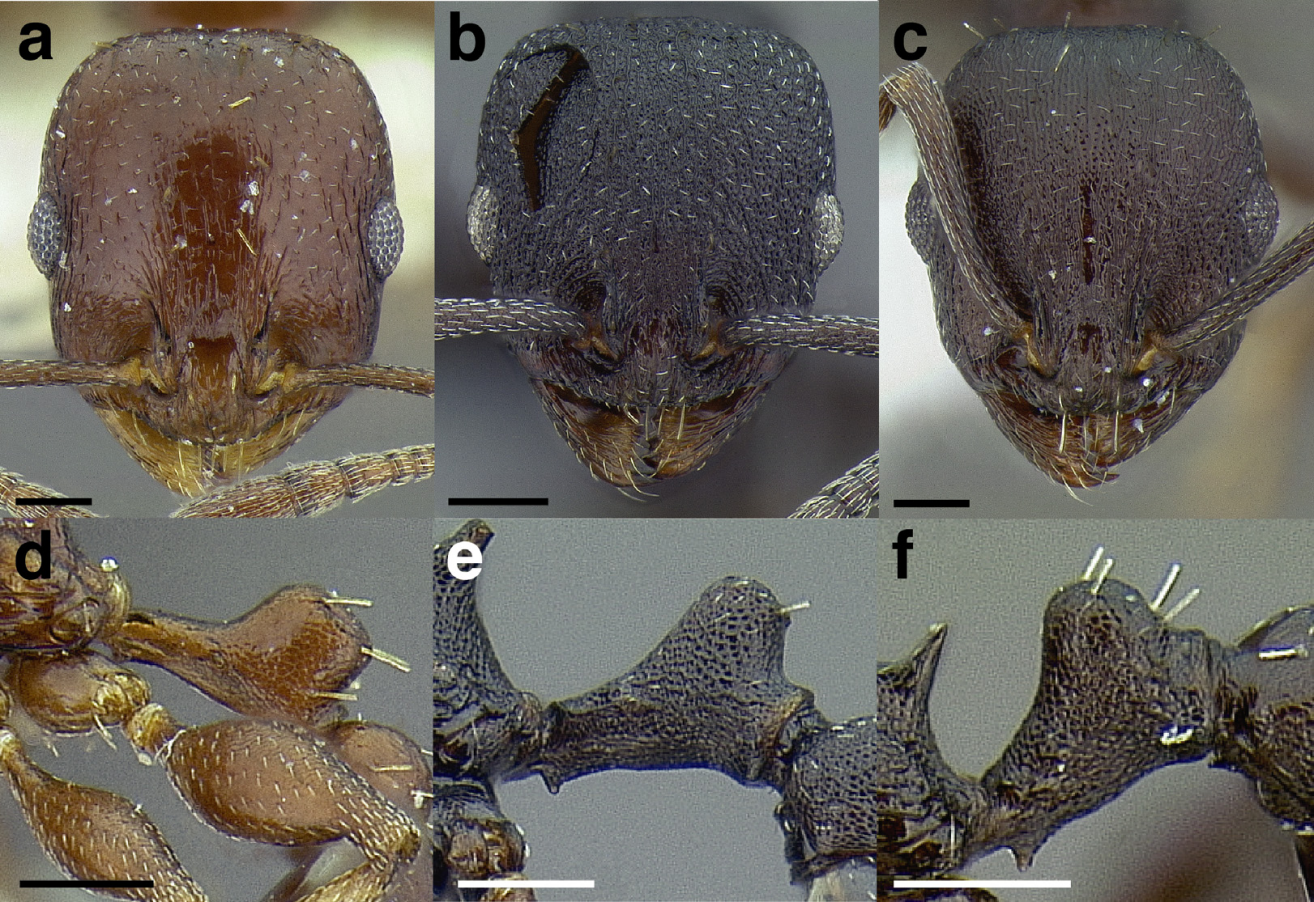
Comparison of worker head sculpture and petiole morphology in the *salvini* clade. (A) *Temnothorax tenuisculptus*, full-face view (MCZENT00032435). (B) *T. altinodus* sp. nov., full-face view (CASENT0617708). (C) *T. acutispinosus* sp. nov., full-face view (JTLC000007447). (D) *T. tenuisculptus*, profile view (MCZENT00032435). (E) *T. altinodus* sp. nov., profile view (CASENT0617708). (F) *T. acutispinosus* sp. nov., profile view (JTLC000007447). Scale bars 0.2 mm.

**Figure 60:**
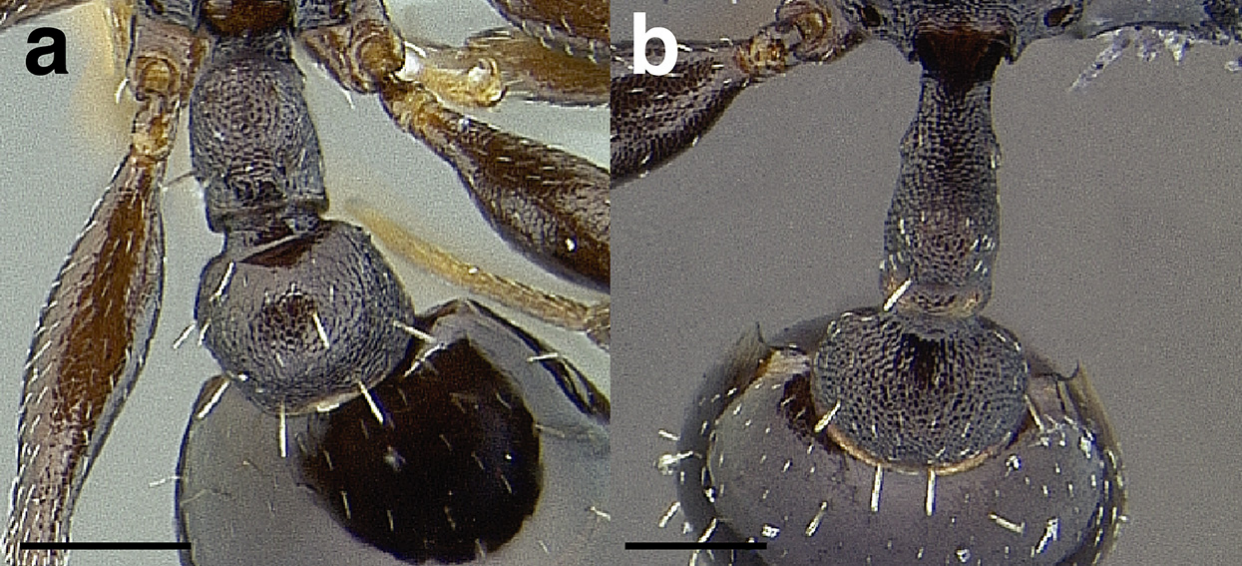
Comparison of worker waist segment morphology in the *salvini* clade in dorsal view. (A) *Temnothorax acutispinosus* sp. nov. (JTLC000007447). (B) *T. altinodus* sp. nov. (CASENT0617708). Scale bars 0.2 mm.

**Figure 61:**
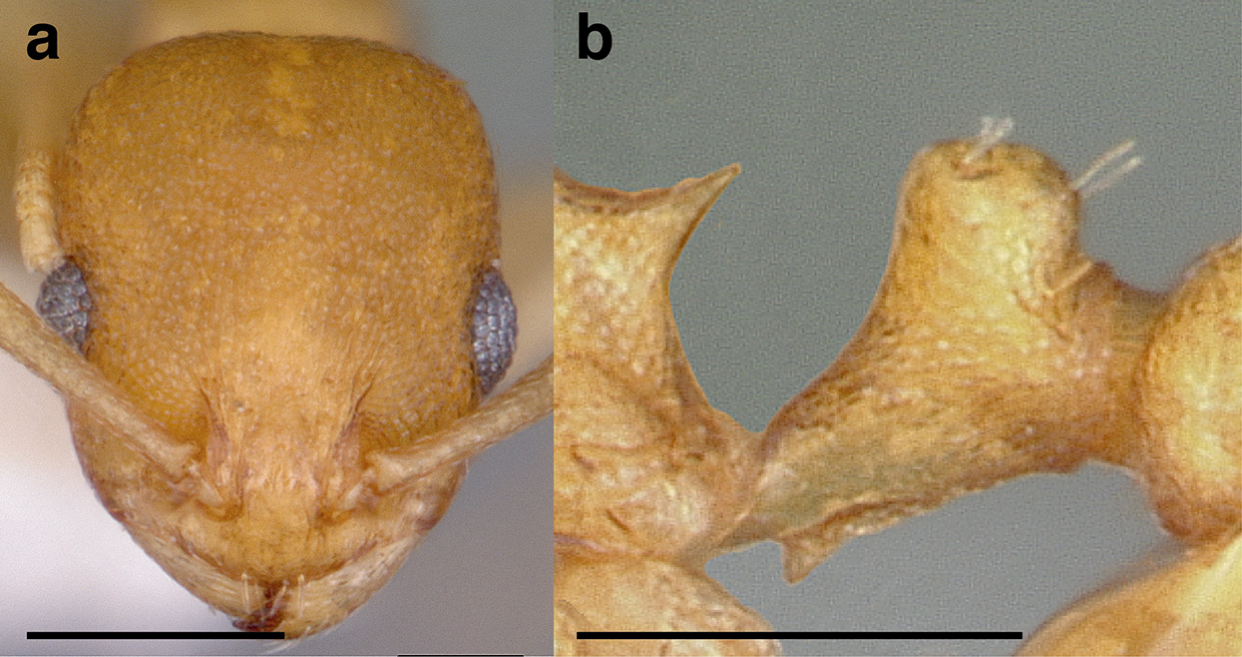
Head sculpture and petiole morphology of *Temnothorax terrigena* worker. (CASENT0104775; photo: April Nobile, from www.antweb.org). (A) Full-face view. (B) Profile view. Scale bars 0.2 mm.

**Figure 62:**
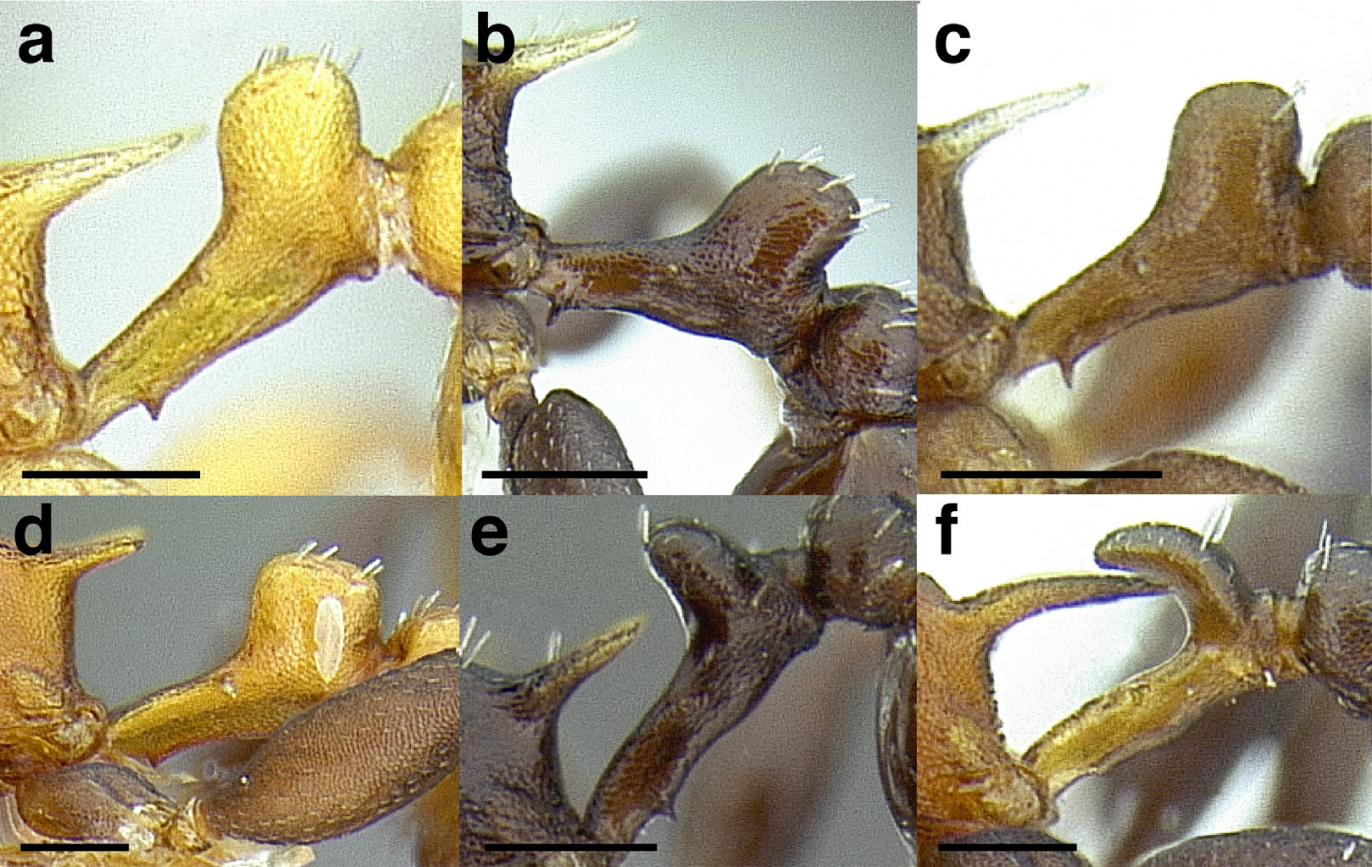
Comparison of worker petiole morphology in the *salvini* clade in profile view. (A) *Temnothorax aureus* sp. nov. (CASENT0619363). (B) *T. leucacanthoides* sp. nov. (CASENT0756102). (C) *T. leucacanthus* (MCZENT00032434). (D) *T. casanovai* sp. nov. (LACMENT323462). (E) *T. subditivus* (CASENT0758340). (F) *T. pastinifer* (CASENT0758861). Scale bars 0.2 mm.

**Figure 63:**
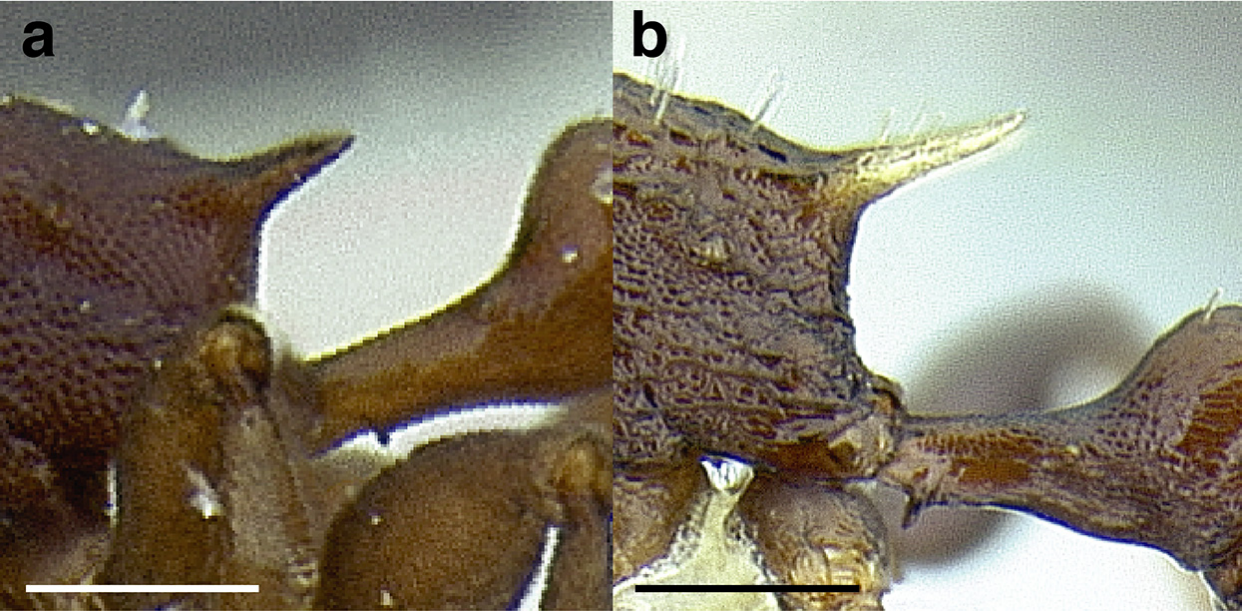
Comparison of worker propodeal spine morphology in the *augusti* species group in profile view. (A) *Temnothorax augusti* (MCZENT00021005). (B) *T. leucacanthoides* sp. nov. (CASENT0756102). Scale bars 0.2 mm.

**Figure 64:**
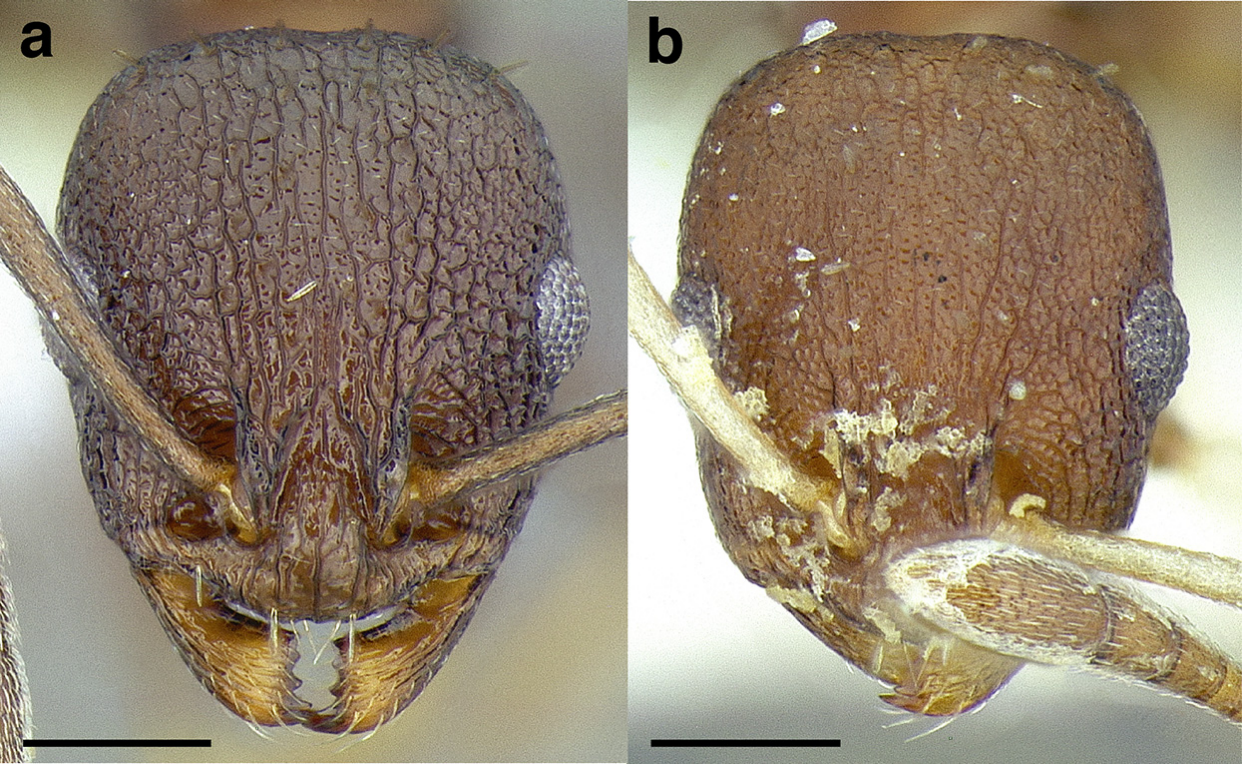
Comparison of worker head morphology in the *augusti* species group in full-face view. (A) *Temnothorax leucacanthoides* sp. nov. (CASENT0756102). (B) *T. leucacanthus* (MCZENT00032434). Scale bars 0.2 mm.

**Figure 65:**
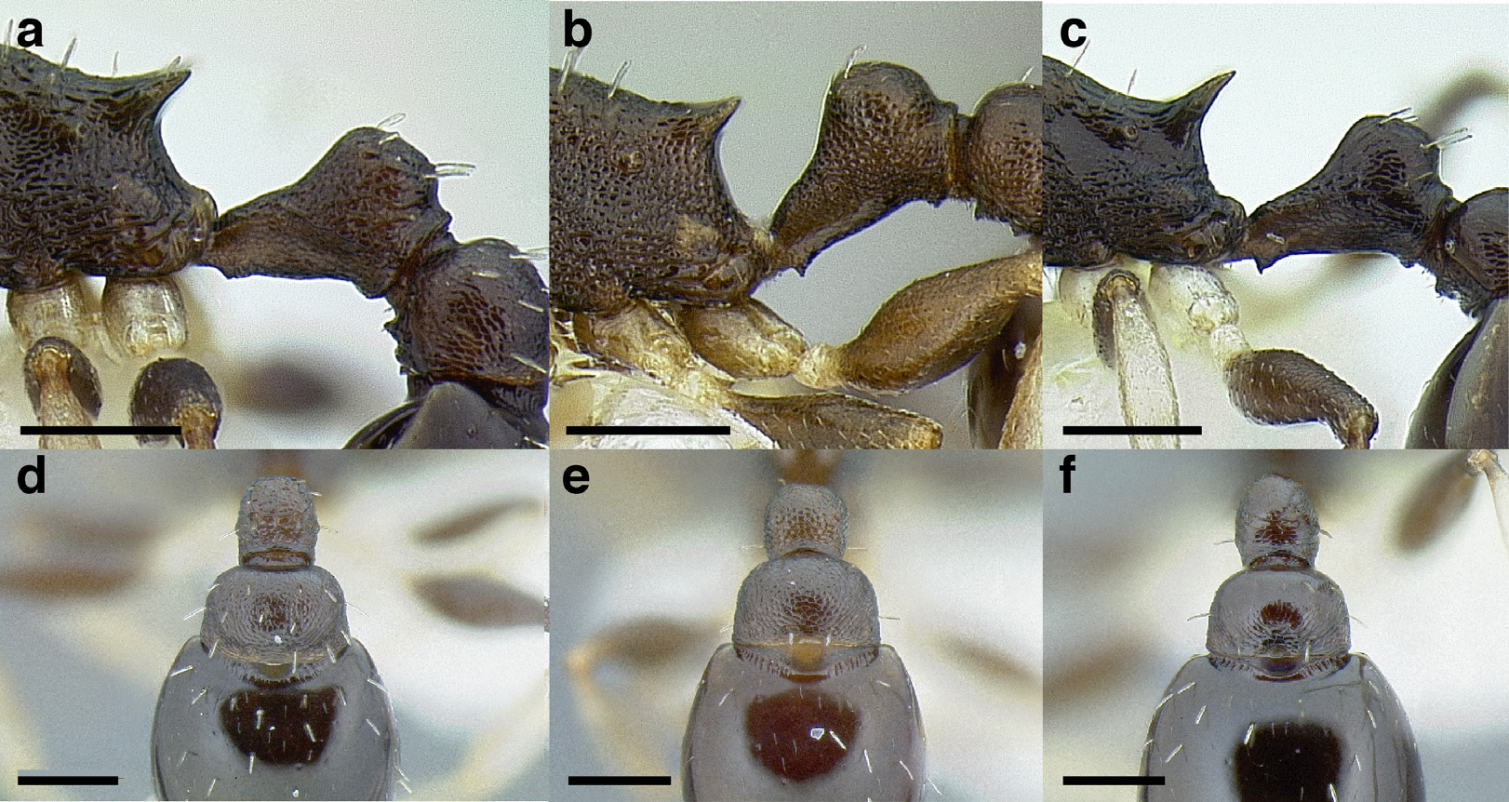
Comparison of worker petiole morphology in the *goniops* species group. (A–C) Profile view. (D–F) Dorsal view. (A) *Temnothorax achii* sp. nov. (JTLC000009876). (B) *T. ixili* (CASENT0611678). (C) *T. xincai* sp. nov. (CASENT0632983). (D) *T. achii* sp. nov. (JTLC000009876). (E) *T. ixili* (CASENT0611678). (F) *T. xincai* sp. nov.(CASENT0632983). Scale bars 0.2 mm.

**Figure 66:**
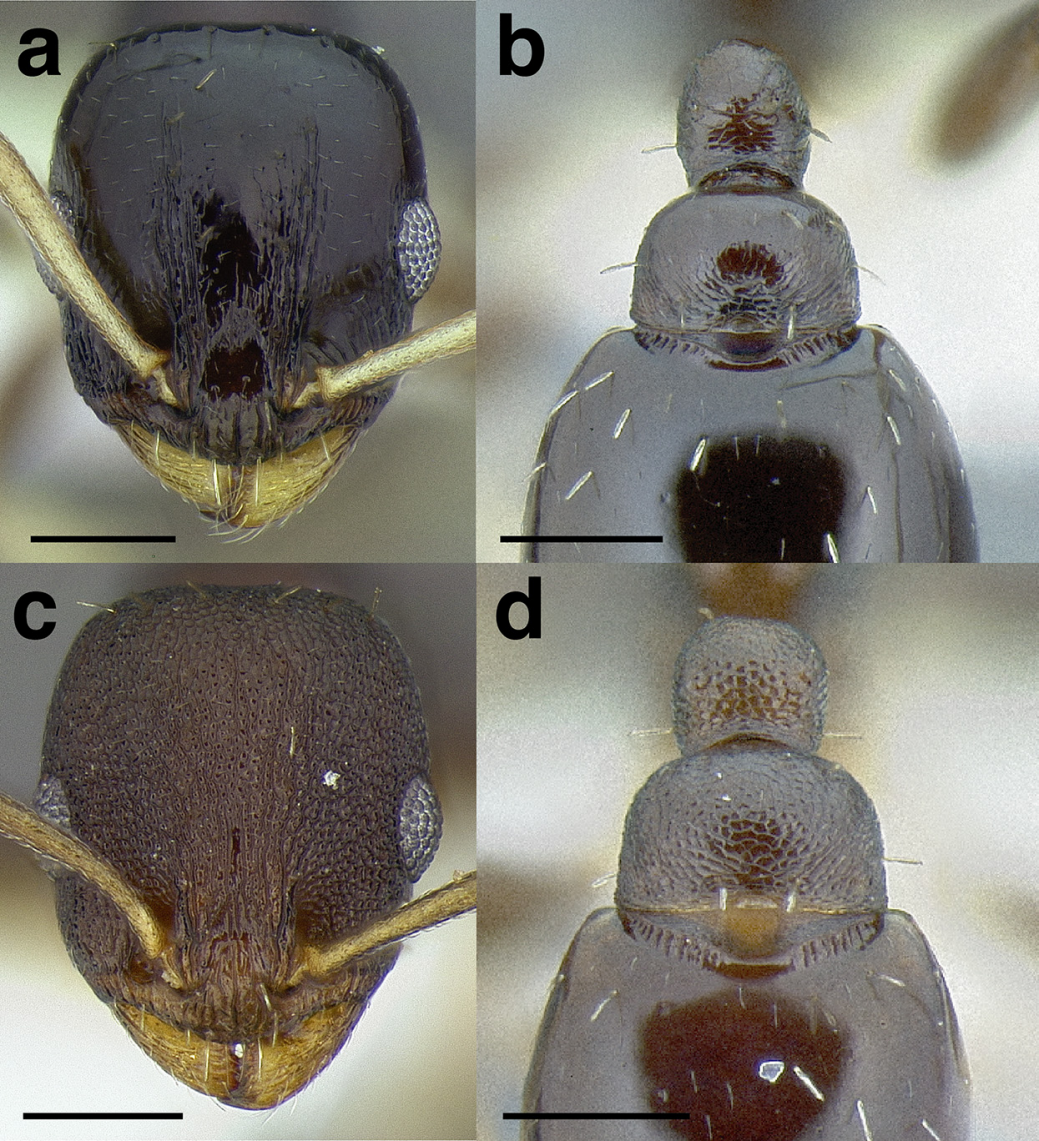
Comparison of worker head and waist segment morphology in the *goniops* species group. (A) *Temnothorax xincai* sp. nov., full-face view (CASENT0632983). (B) *T. xincai* sp. nov., dorsal view (CASENT0632983). (C) *T. ixili*, full-face view (CASENT0611678). (D) *T. ixili*, dorsal view (CASENT0611678). Scale bars 0.2 mm.

**Figure 67:**
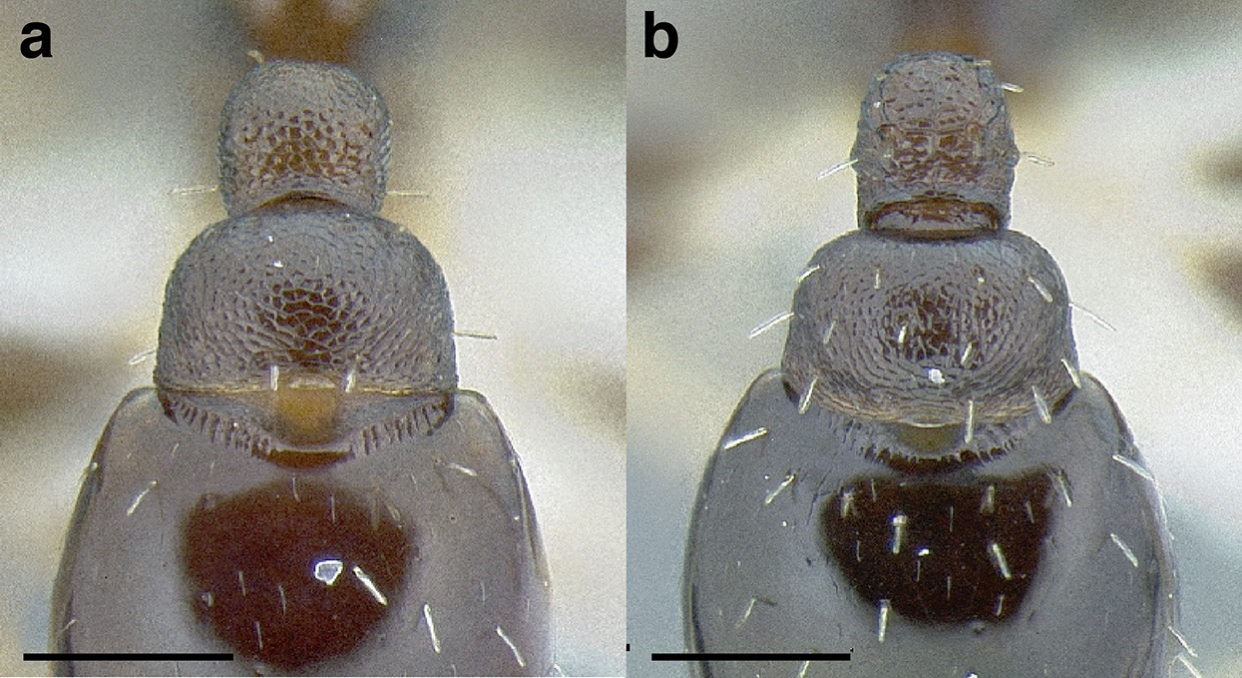
Comparison of worker waist segment morphology in the *goniops* species group in dorsal view. (A) *Temnothorax ixili* (CASENT0611678). (B) *T. achii* sp. nov. (JTLC000009876). Scale bars 0.2 mm.

**Figure 68:**
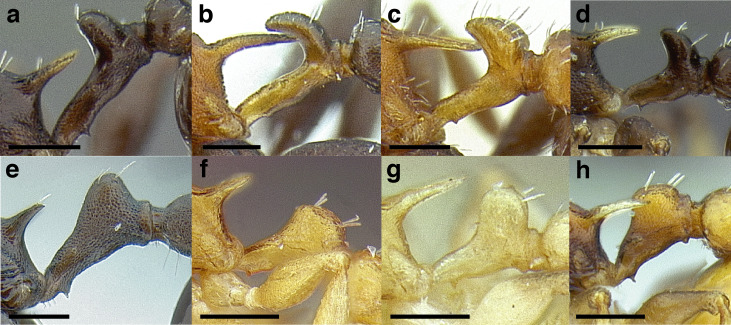
Comparison of worker petiole morphology in the *salvini* clade in profile view. (A) *Temnothorax subditivus* (CASENT0758340). (B) *T. pastinifer* (CASENT0758861). (C) *T. rutabulafer* sp. nov. (CASENT0758266). (D) *T. adrosanus* (CASENT0758350). (E) *T. fuscatus* (CASENT0625205). (F) *T. goniops* (MCZENT00032436). (G) *T. nigricans* (MCZENT00577111). (H) *T. wilsoni* sp. nov., (MCZENT00583608). Scale bars 0.2 mm.

**Figure 69:**
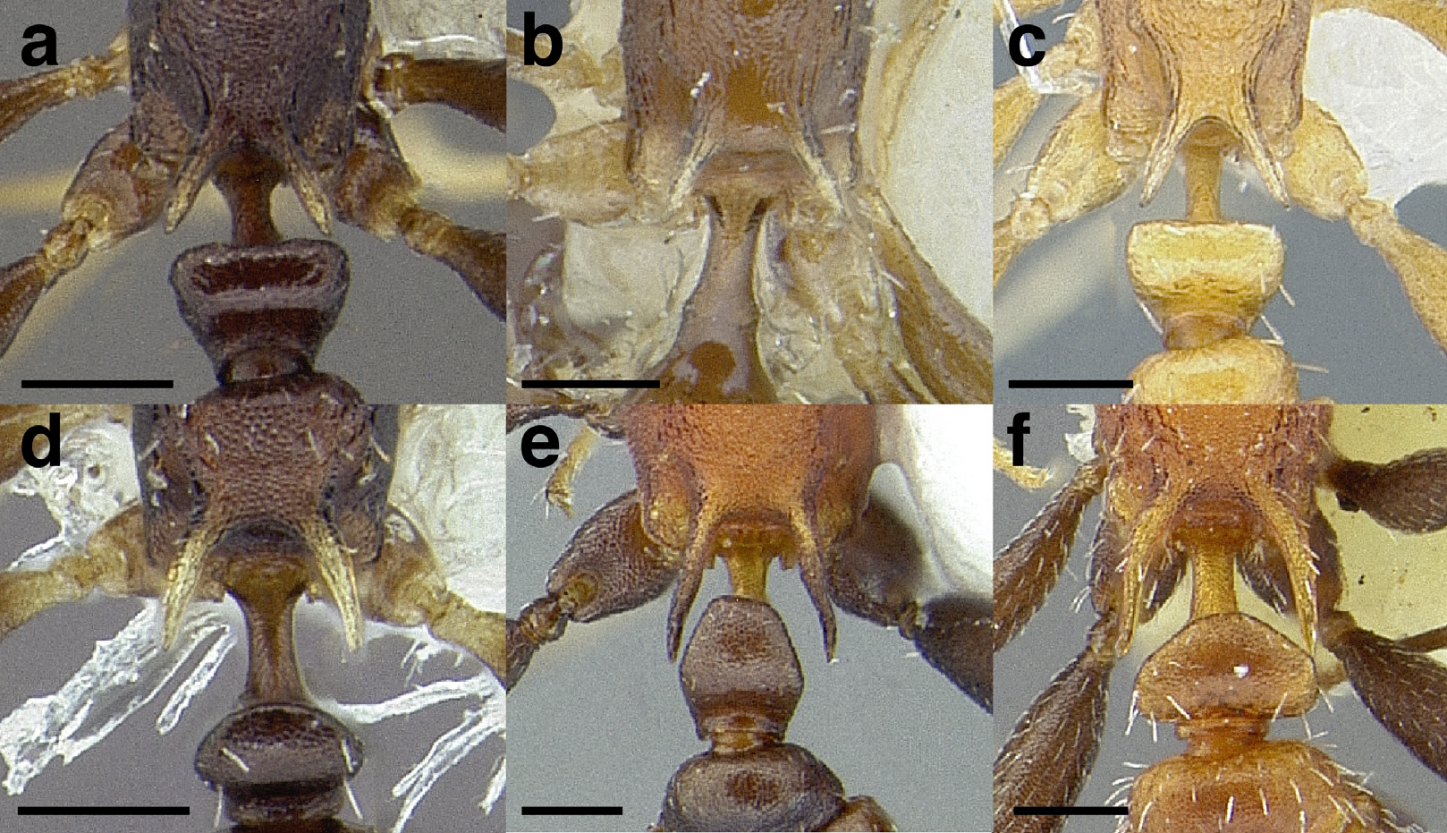
Comparison of worker propodeal spine and petiolar node morphology in the *salvini* clade in dorsal view. (A) *Temnothorax subditivus* (CASENT0758340). (B) *T. subditivus* (MCZENT00016371). (C) *T. subditivus* (CASENT0619368). (D) *T. androsanus* (CASENT0758350). (E) *T. pastinifer* (CASENT0758861). (F) *T. rutabulafer* sp. nov. (CASENT0758266). Scale bars 0.2 mm.

**Figure 70:**
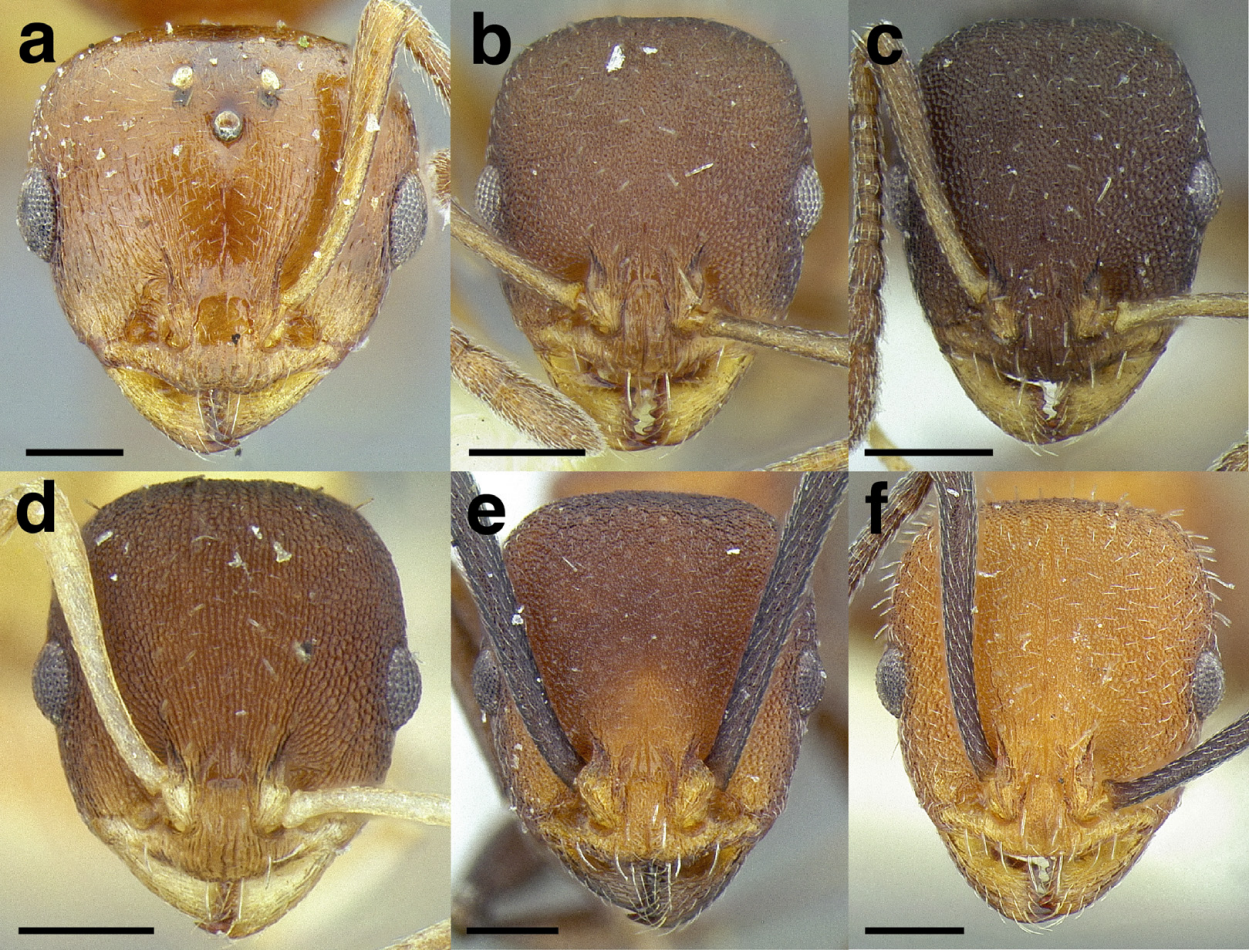
Comparison of worker head sculpture in the *pastinifer* species group in full-face view. (A) *Temnothorax schwarzi* (USNMENT00532012). (B) *T. androsanus* (MCZENT00021003). (C) *T. androsanus* (CASENT0758350).(D) *T. nigricans* (MCZENT00577111). (E) *T*. pastinifer (CASENT0758861). (F) *T. rutabulafer* sp. nov.(CASENT0758266). Scale bars 0.2 mm.

**Figure 71:**
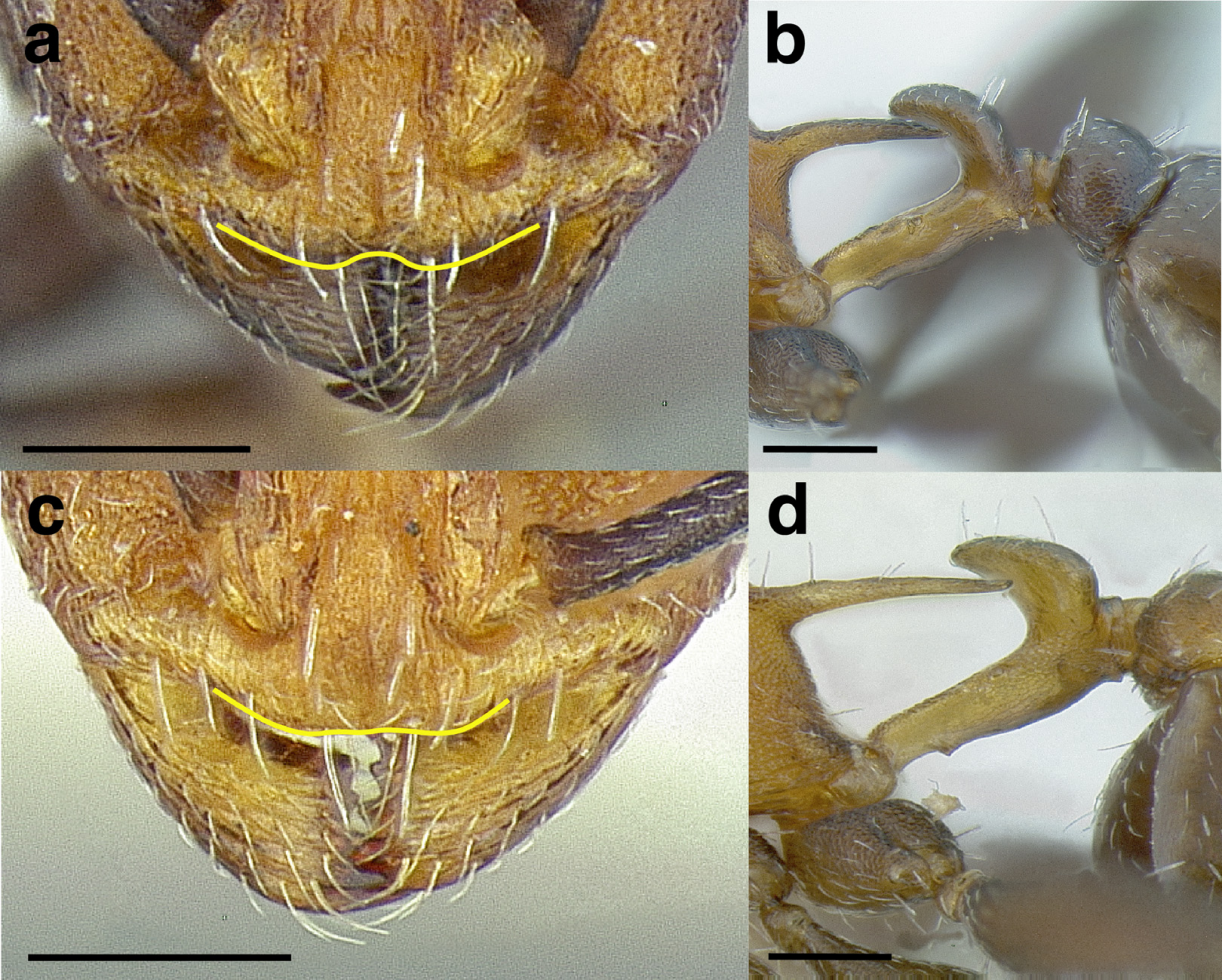
Comparison of worker clypeus and waist segment morphology in the *pastinifer* species group. (A) *Temnothorax pastinifer*, full-face view (CASENT0758861). (B) *T. pastinifer*, profile view (CASENT0758861). (C) *T. rutabulafer* sp. nov., full-face view (CASENT0758266). (D) *T. rutabulafer* sp. nov., profile view (CASENT0758266). Scale bars 0.2 mm.

**Figure 72:**
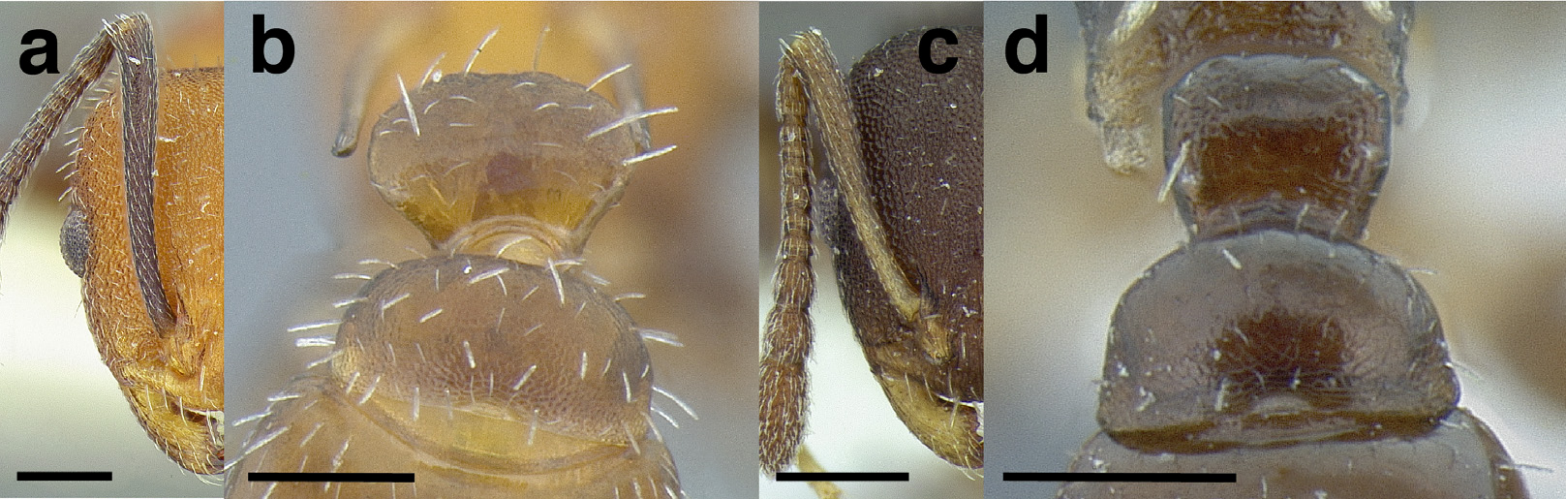
Comparison of worker petiolar node morphology and antennal scape length in the *pastinifer* species group. (A) *Temnothorax rutabulafer* sp. nov., full-face view (CASENT0758266). (B) *T. rutabulafer* sp. nov., posterodorsal view (CASENT0758266). (C) *T. androsanus*, full-face view (CASENT0758350). (D) *T. androsanus*, posterodoral view (CASENT0758350). Scale bars 0.2 mm.

**Figure 73:**
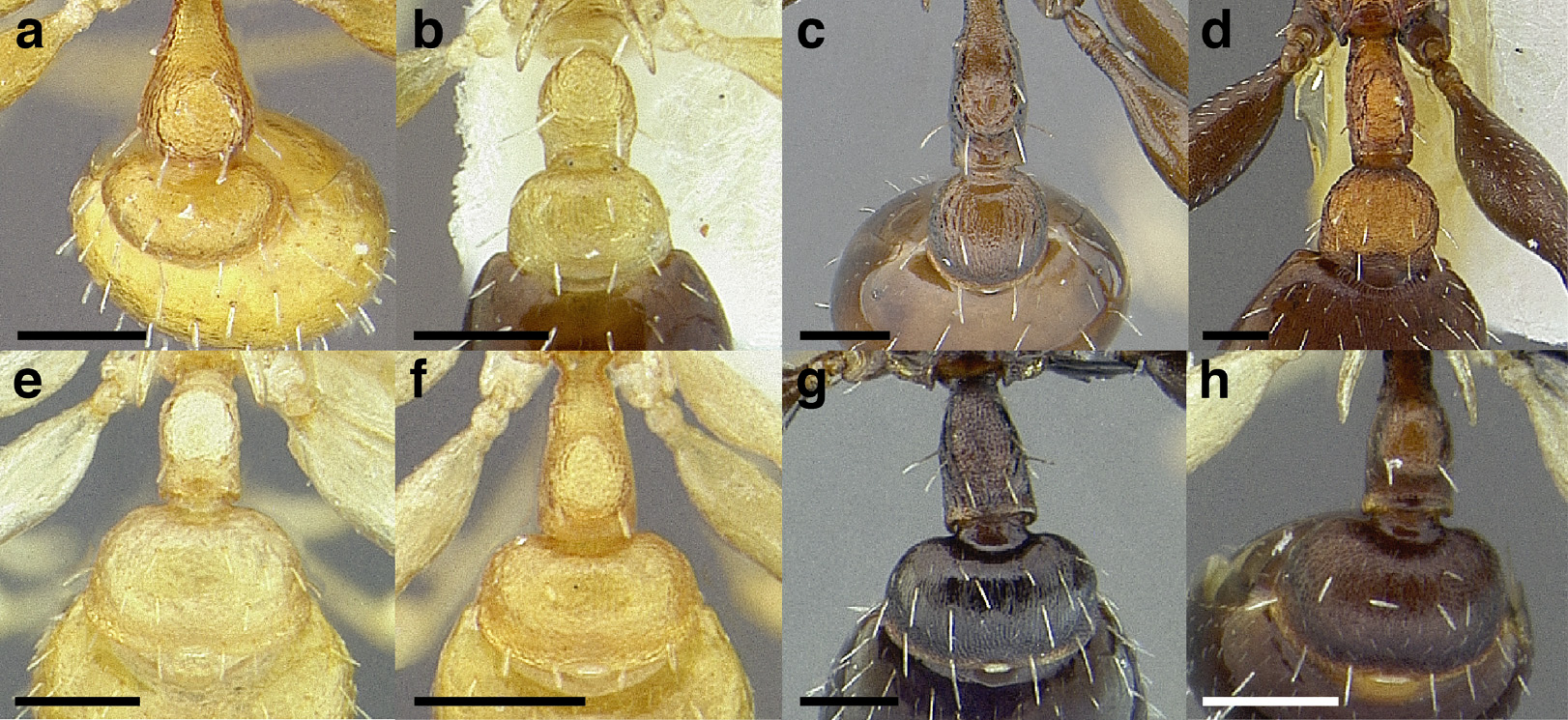
Comparison of worker waist segment morphology in the *salvini* clade in dorsal view. (A) *Temnothorax goniops* (MCZENT00032436). (B) *T. huehuetenangoi* (USNMENT00529517). (C) *T. fuscatus* (CASENT0916005). (D) *T. ocarinae* (USNMENT00531636). (E) *T. agavicola* sp. nov. (MCZENT00510559). (F) *T. torrei* (MCZENT00583611). (G) *T. laticrus* sp. nov. (CASENT0758263). (H) *T. albispinus* (CASENT0756097). Scale bars 0.2 mm.

**Figure 74:**
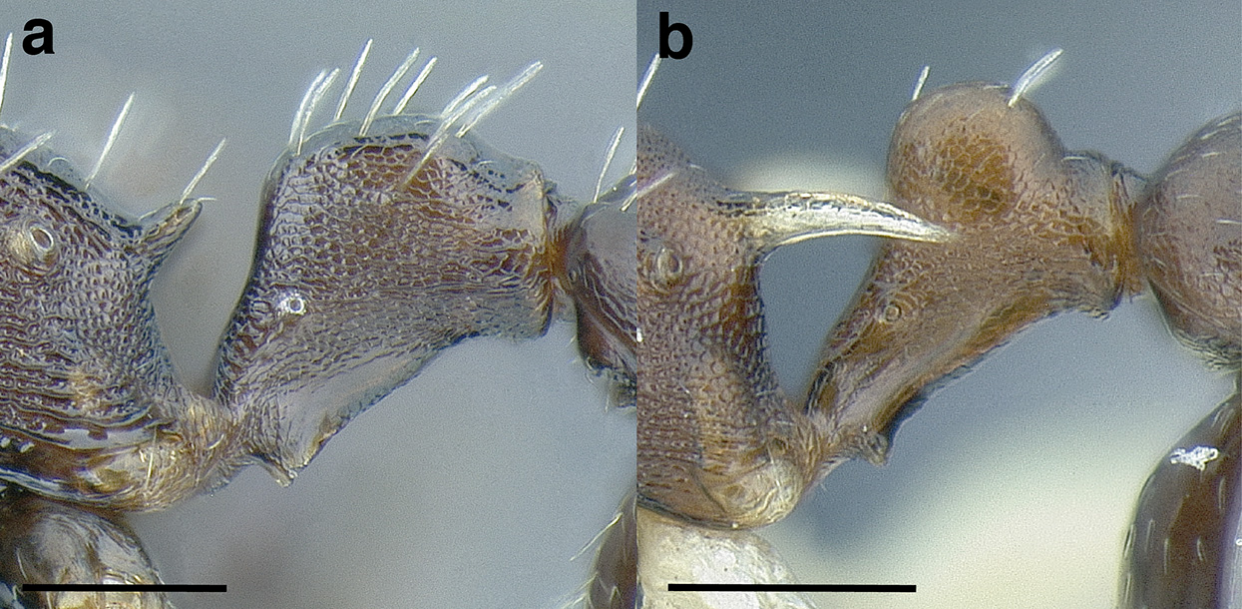
Comparison of worker propodeal spine and petiole morphology in the *pulchellus* species group in profile view. (A) *Temnothorax laticrus* sp. nov. (CASENT0758263). (B) *T. albispinus* (CASENT0756097). Scale bars 0.2 mm.

**Figure 75:**
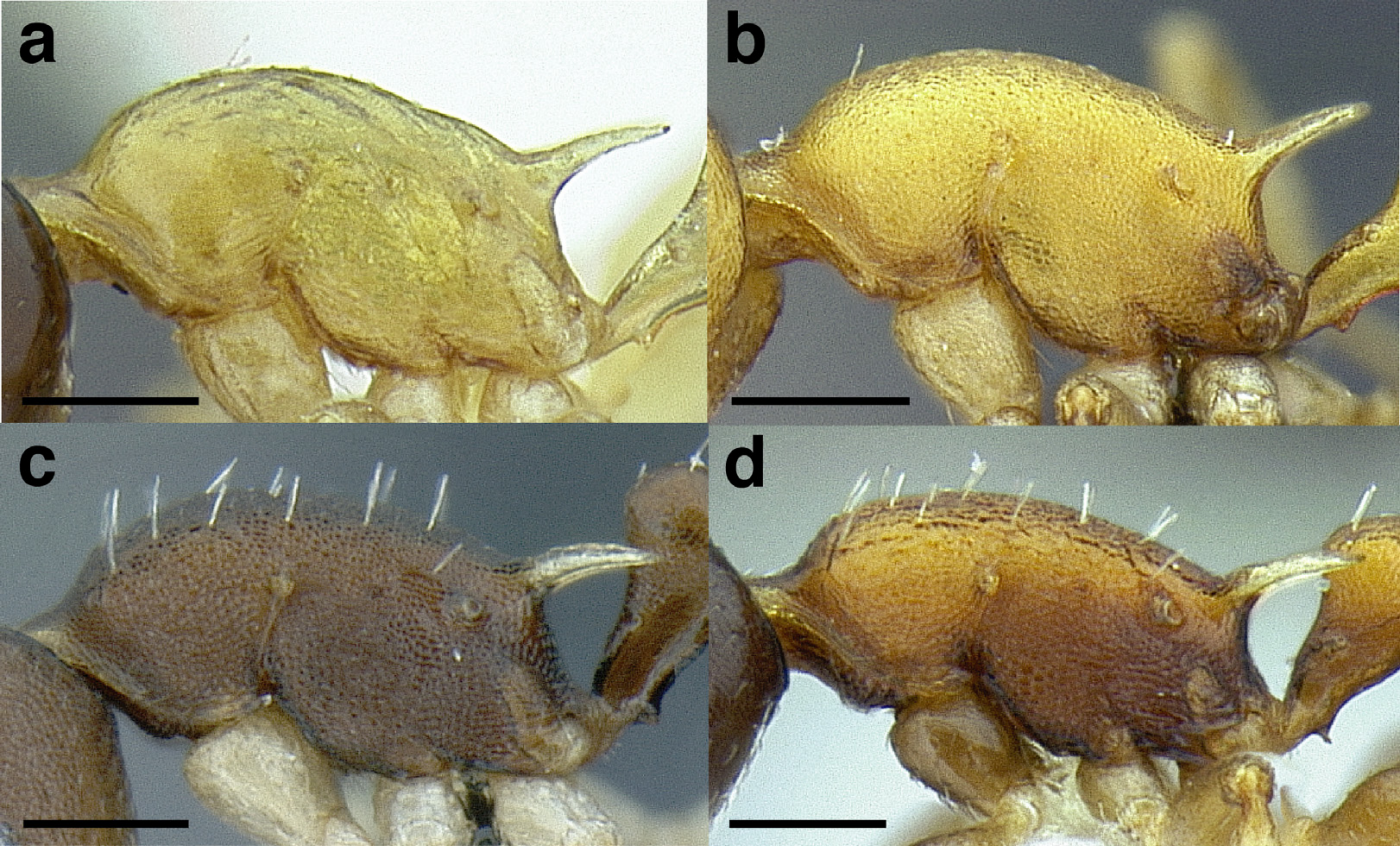
Comparison of worker mesosoma setae count in the *pulchellus* species group in profile view. (A) *Temnothorax pulchellus* (LACMENT323204). (B) *T. wettereri* sp. nov. (CASENT0756094). (C) *T. albispinus* (CASENT0756097). (D) *T. wilsoni* sp. nov. (MCZENT00583608). Scale bars 0.2 mm.

**Figure 76:**
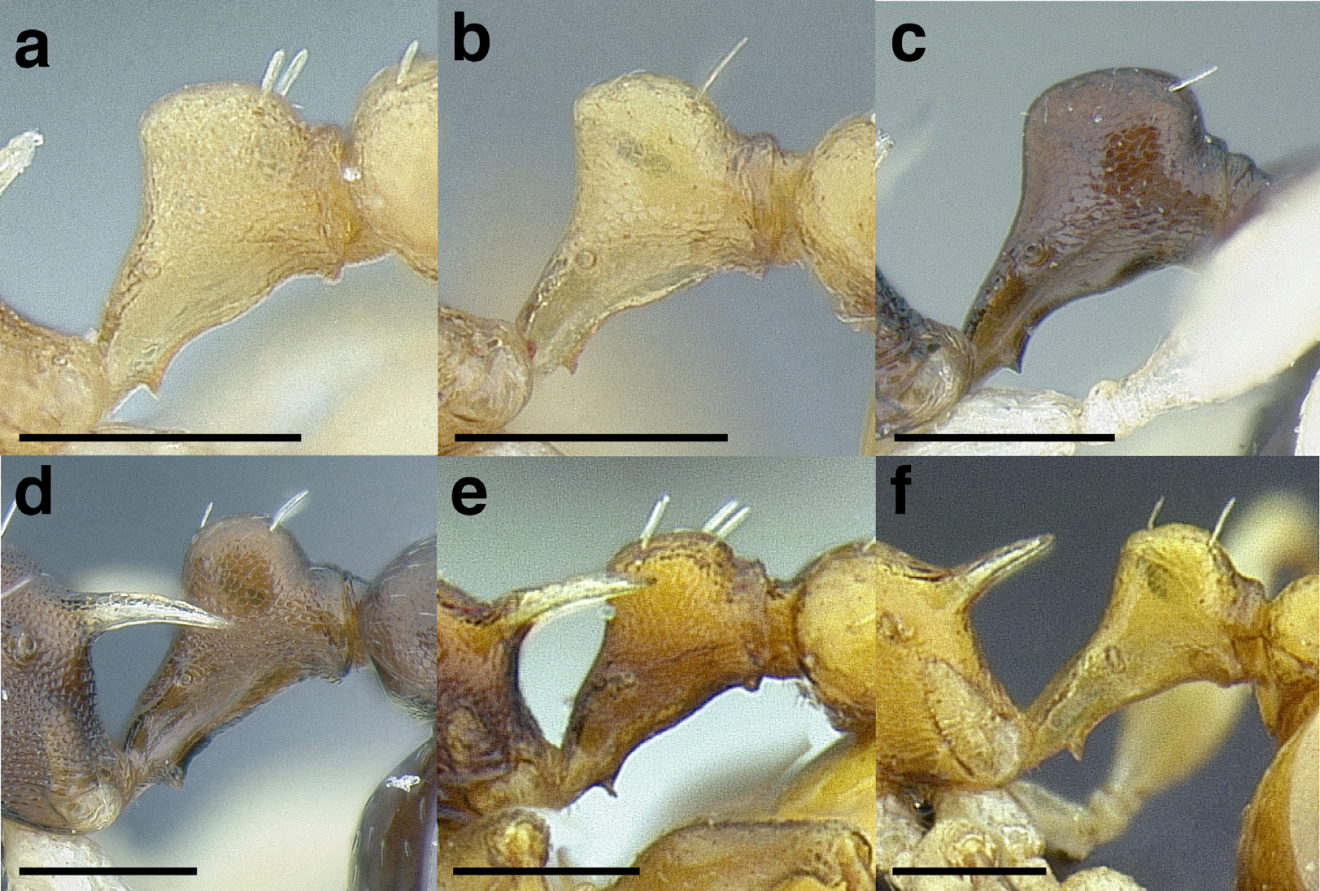
Comparison of worker petiole setae count in the *pulchellus* species group in profile view. (A) *Temnothorax torrei* (MCZENT00583611). (B) *T. terricola* (CASENT0915980). (C) *T. hippolytus* sp. nov. (LACMENT323469). (D) *T. albispinus* (CASENT0756097). (E) *T. wilsoni* sp. nov. (MCZENT00583608). (F) *T. magnabulla* sp. nov.(CASENT0756093). Scale bars 0.2 mm.

**Figure 77:**
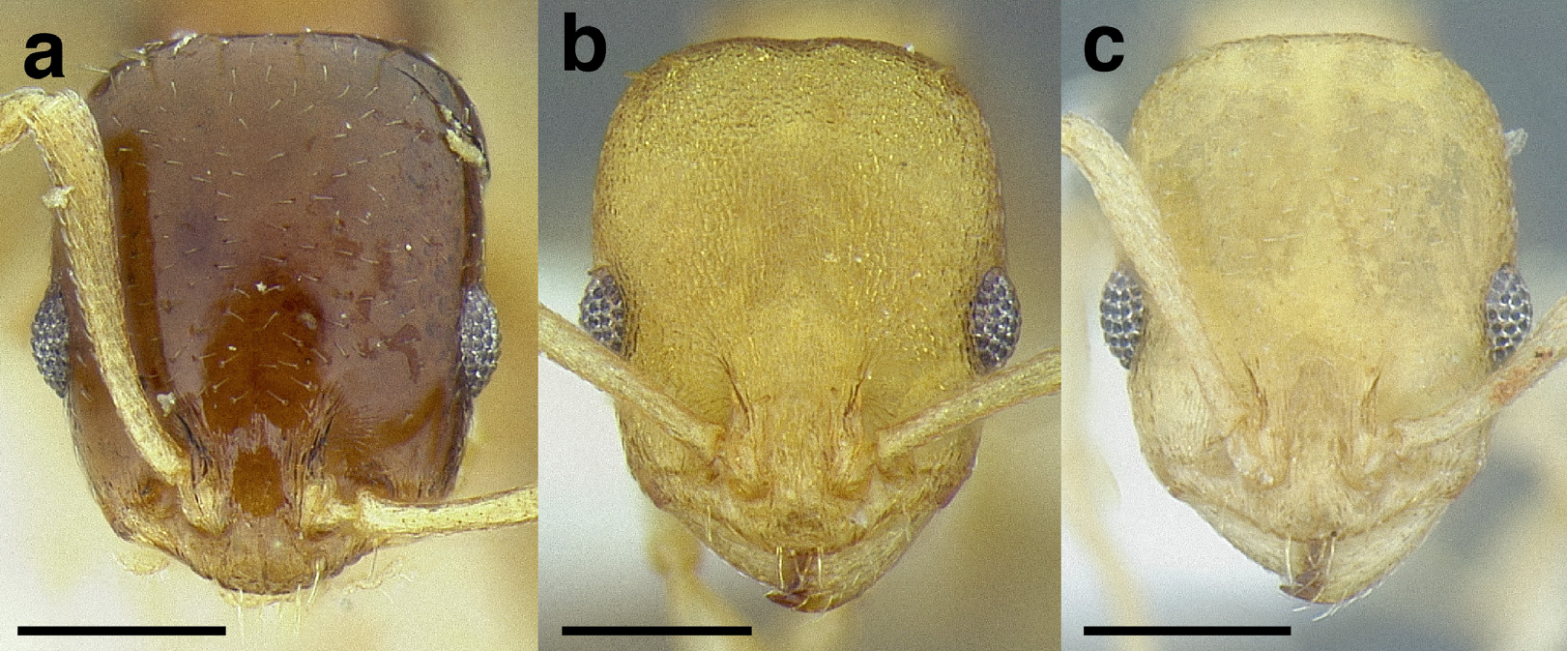
Comparison of worker head sculpture in the *pulchellus* species group in full-face view. (A) *Temnothorax terricola* (CASENT0915980). (B) *T. torrei* (MCZENT00583611). (C) *T. torrei* (CASENT0758351). Scale bars 0.2 mm.

**Figure 78:**
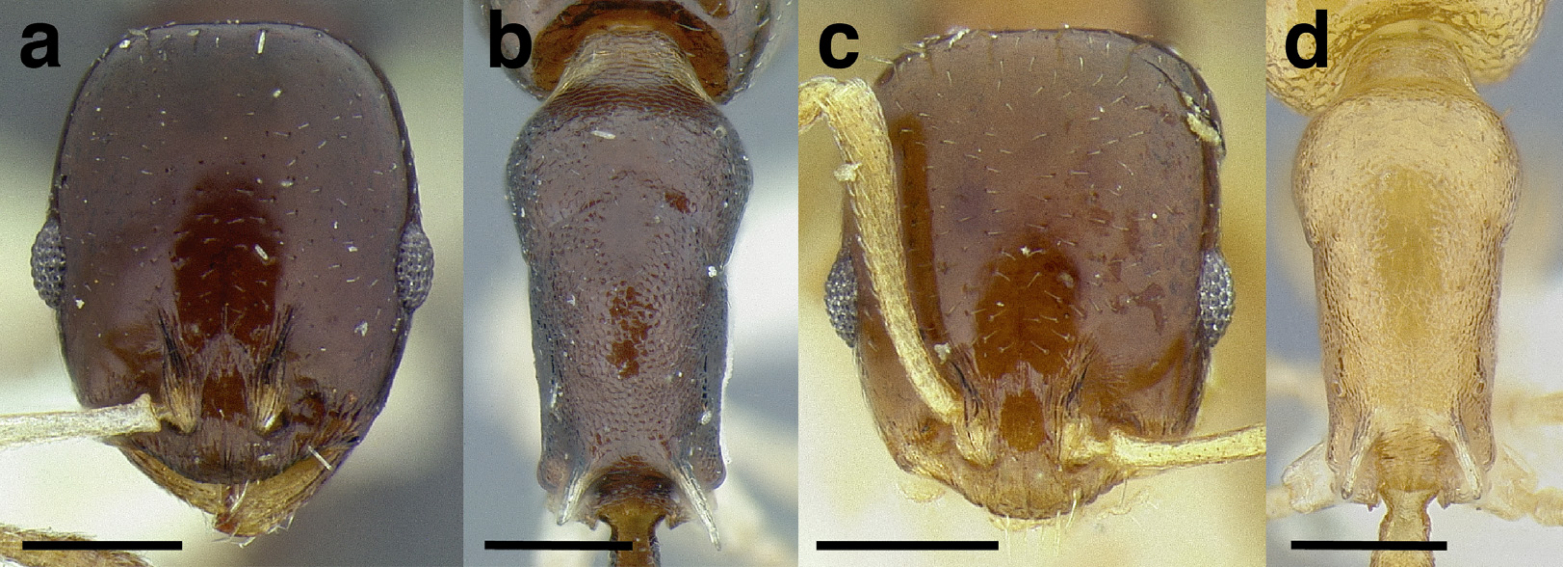
Comparison of worker head and mesosoma morphology in the *pulchellus* species group. (A) *Temnothorax hippolytus* sp. nov., full-face view (LACMENT323469). (B) *T. hippolytus* sp. nov., dorsal view (LACMENT323469). (C) *T. terricola*, full-face view (CASENT0915980). (D) *T. terricola*, dorsal view (CASENT0915980). Scale bars 0.2 mm.

**Figure 79:**
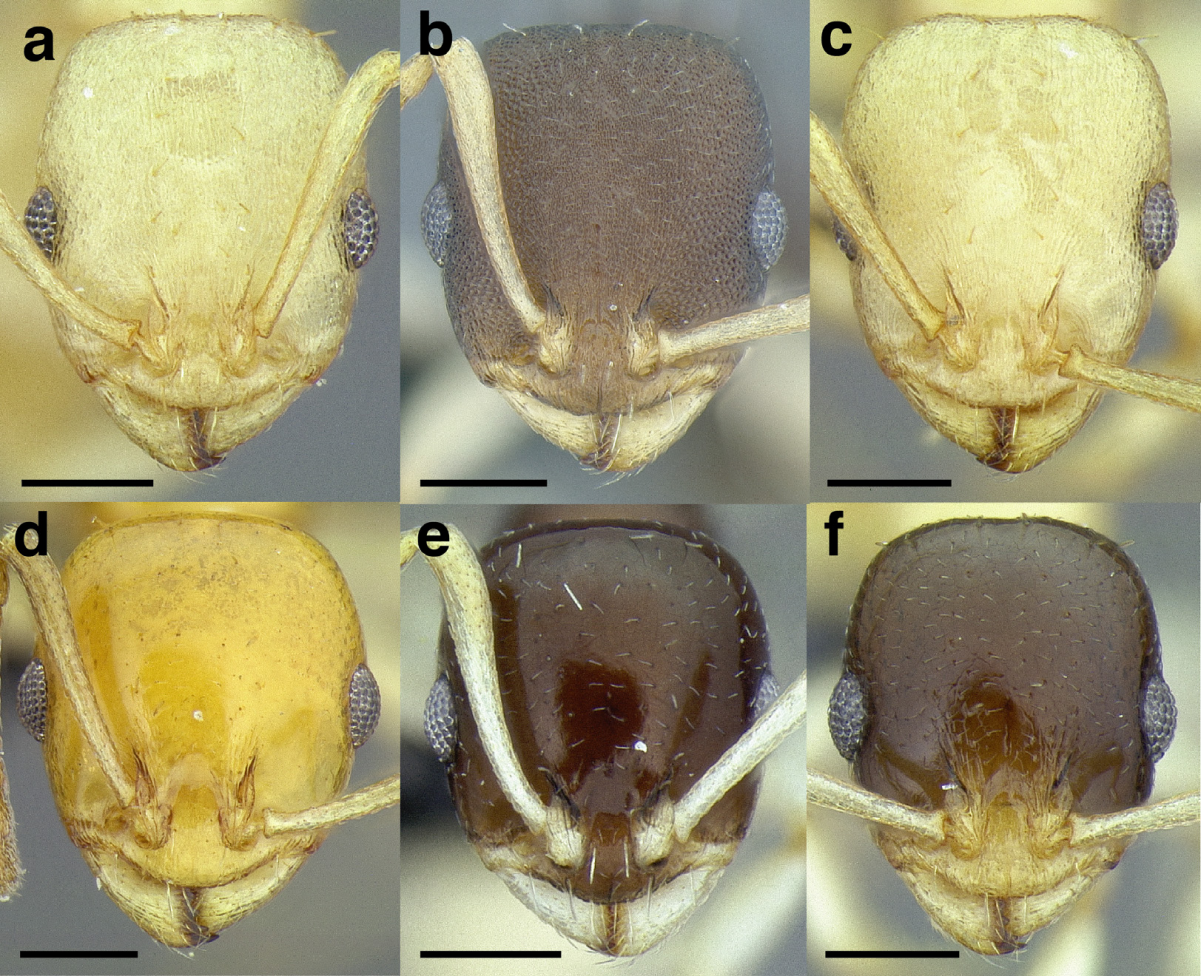
Comparison of worker head sculpture in the *pulchellus* species group in full-face view. (A) *Temnothorax agavicola* sp. nov. (MCZENT00510559). (B) *T. albispinus* (CASENT0756097). (C) *T. agavicola* sp. nov.(CASENT0756096). (D) *T. magnabulla* sp. nov. (CASENT0756093). (E) *T. harlequina* sp nov. (CASENT0756091). (F) *T. wilsoni* sp. nov. (MCZENT00583608). Scale bars 0.2 mm.

**Figure 80:**
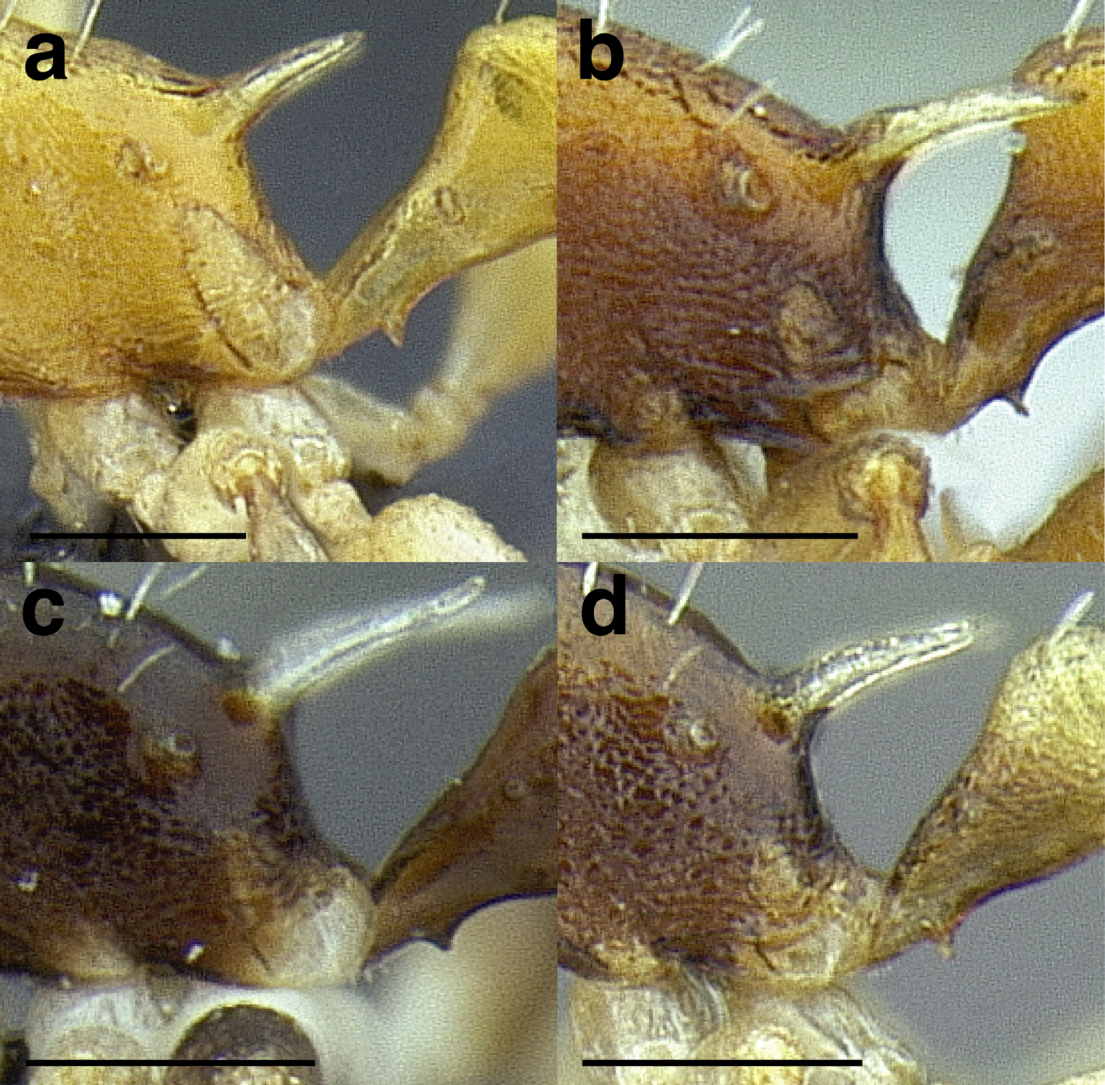
Comparison of worker metapleural gland bulla morphology in the *pulchellus* species group in profile view. (A) *Temnothorax magnabulla* sp. nov. (CASENT0756093). (B) *T. wilsoni* sp. nov. (MCZENT00583608). (C) *T. harlequina* sp. nov. (CASENT0756091). (D) *T. ciferrii* (CASENT0758829). Scale bars 0.2 mm.

**Figure 81:**
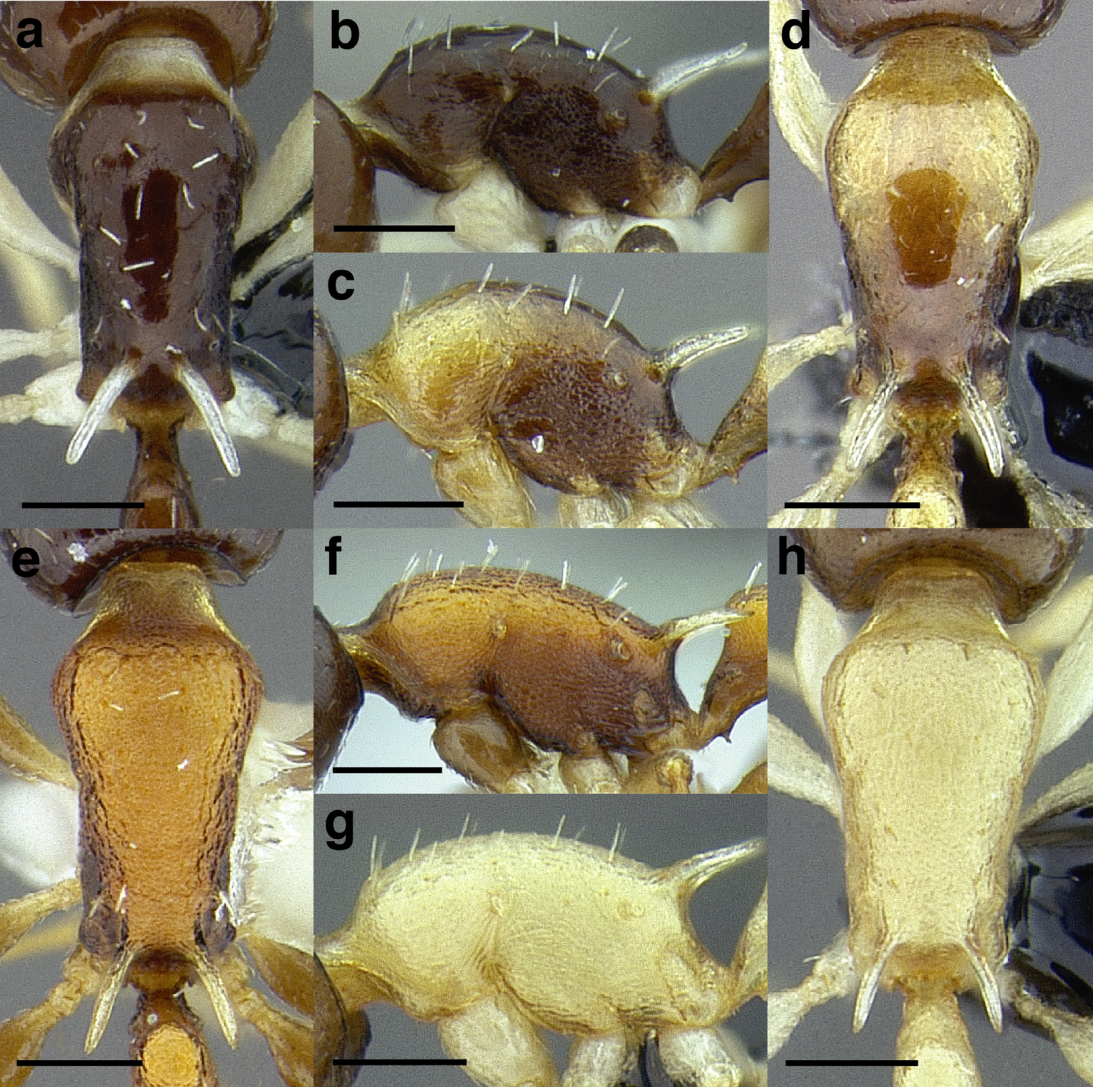
Comparison of worker mesosoma sculpture and morphology in the *pulchellus* species group. (A) *Temnothorax harlequina* sp. nov., dorsal view (CASENT0756091). (B) *T. harlequina* sp. nov., profile view (CASENT0756091). (C) *T. ciferrii*, profile view (CASENT0758829). (D) *T. ciferrii*, dorsal view (CASENT0758829). (E) *T. wilsoni* sp. nov., dorsal view (MCZENT00583608). (F) *T. wilsoni* sp. nov., profile view (MCZENT00583608). (G) *T. balaclava* sp nov., profile view (CASENT0758262). (H) *T. balaclava* sp. nov., dorsal view (CASENT0758262). Scale bars 0.2 mm.

**Figure 82:**
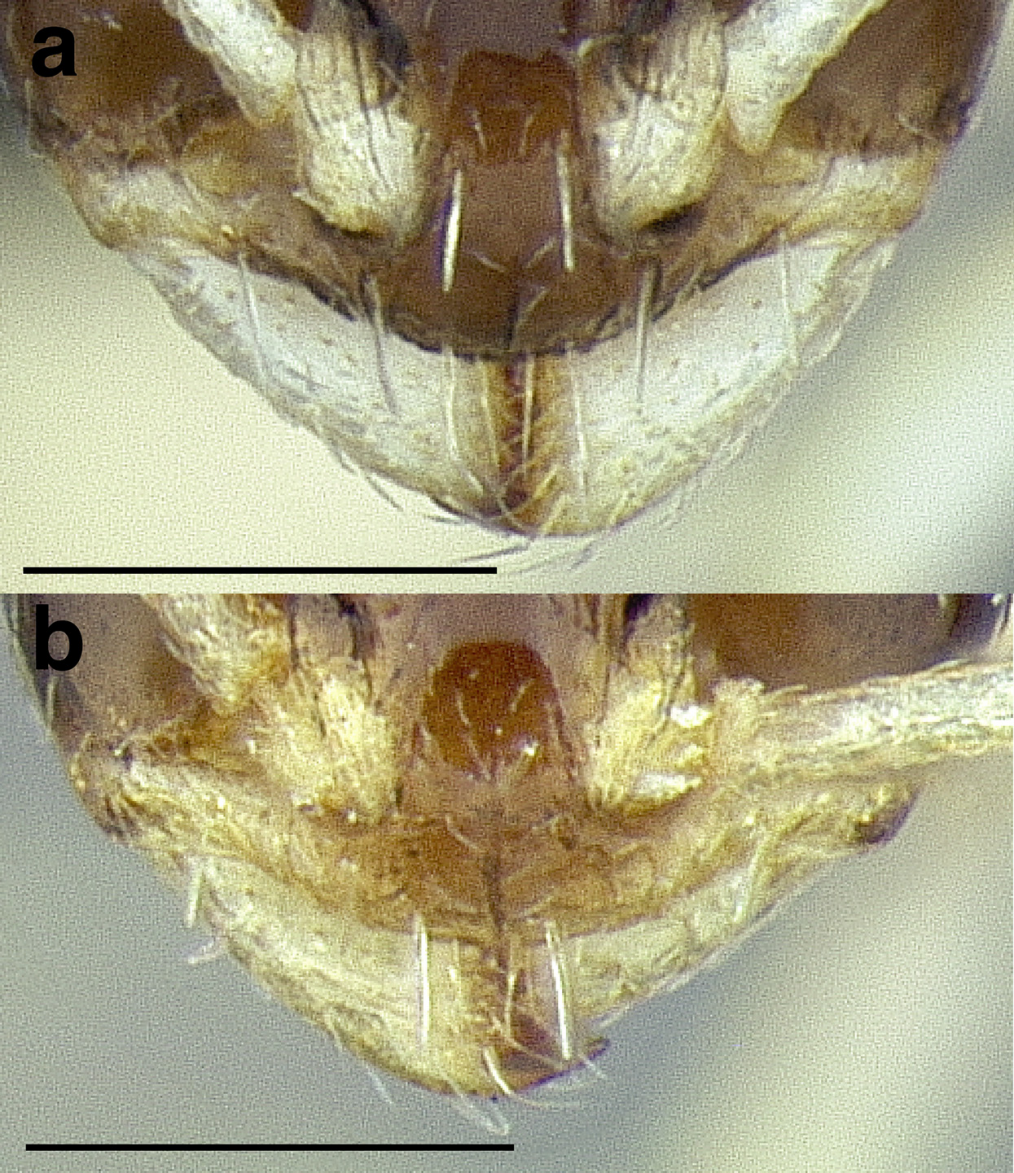
Comparison of worker clypeal setae in the *pulchellus* species group in full-face view. (A) *Temnothorax harlequina* sp. nov. (CASENT0756091). (B) *T. ciferrii* (CASENT0758829). Scale bars 0.2 mm.

**Figure 83:**
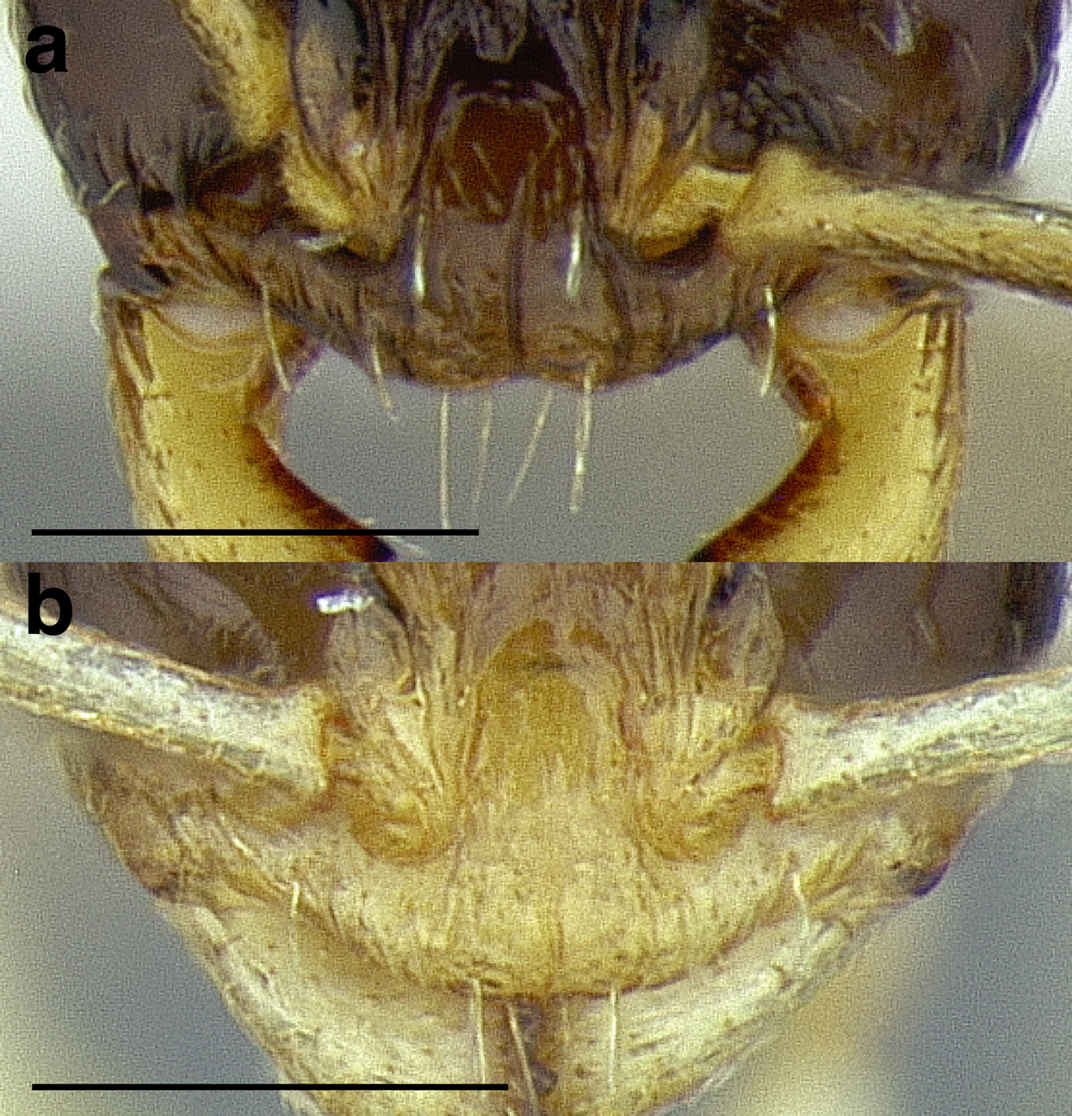
Comparison of worker clypeus morphology in the *pulchellus* species group in full-face view. (A) *Temnothorax wilsoni* sp. nov. (MCZENT00583608). (B) *T. balaclava* sp. nov (CASENT0758262). Scale bars 0.2 mm.

**Figure 84:**
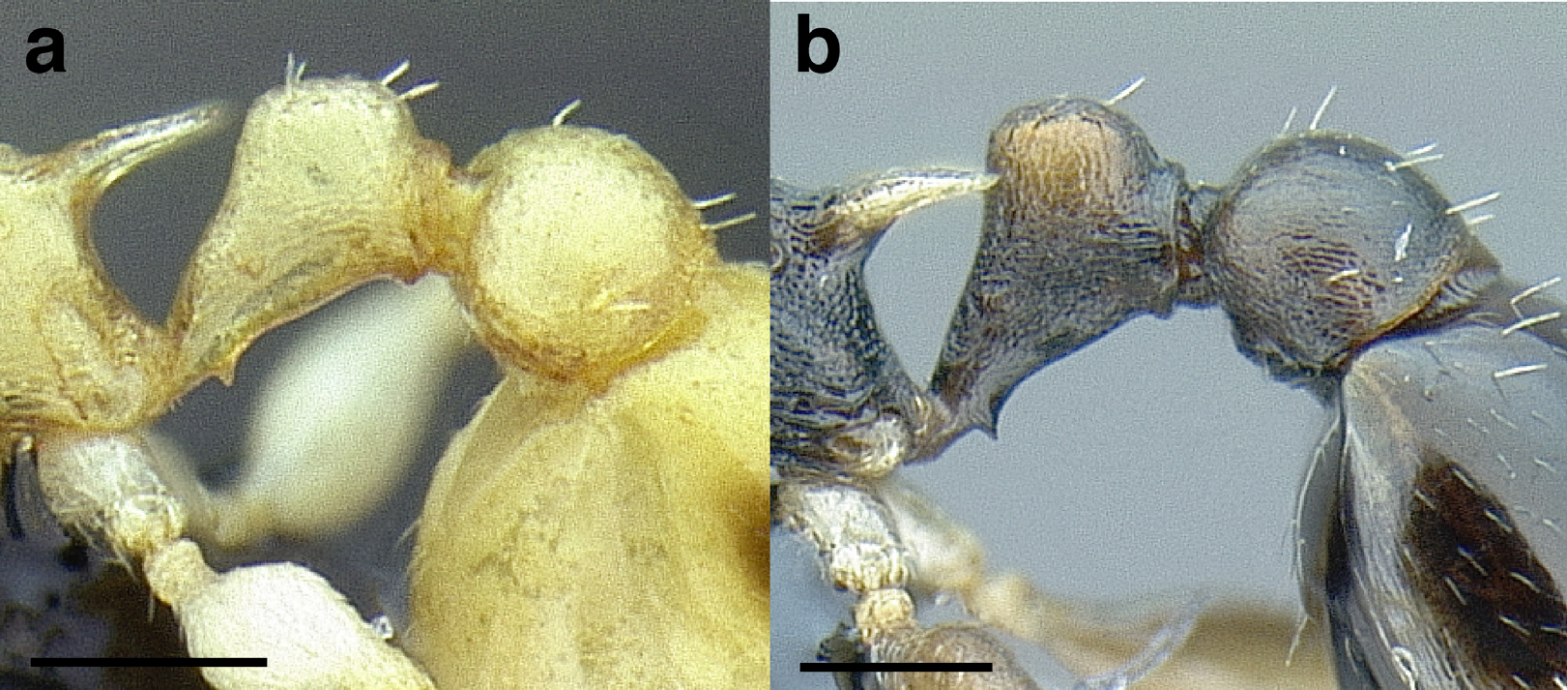
Comparison of worker petiole morphology in the *pulchellus* species group in profile view. (A) *Temnothorax balaclava* sp. nov. (CASENT0758262). (B) *T. bahoruco* sp. nov. (CASENT0758817). Scale bars 0.2 mm.

**Figure 85:**
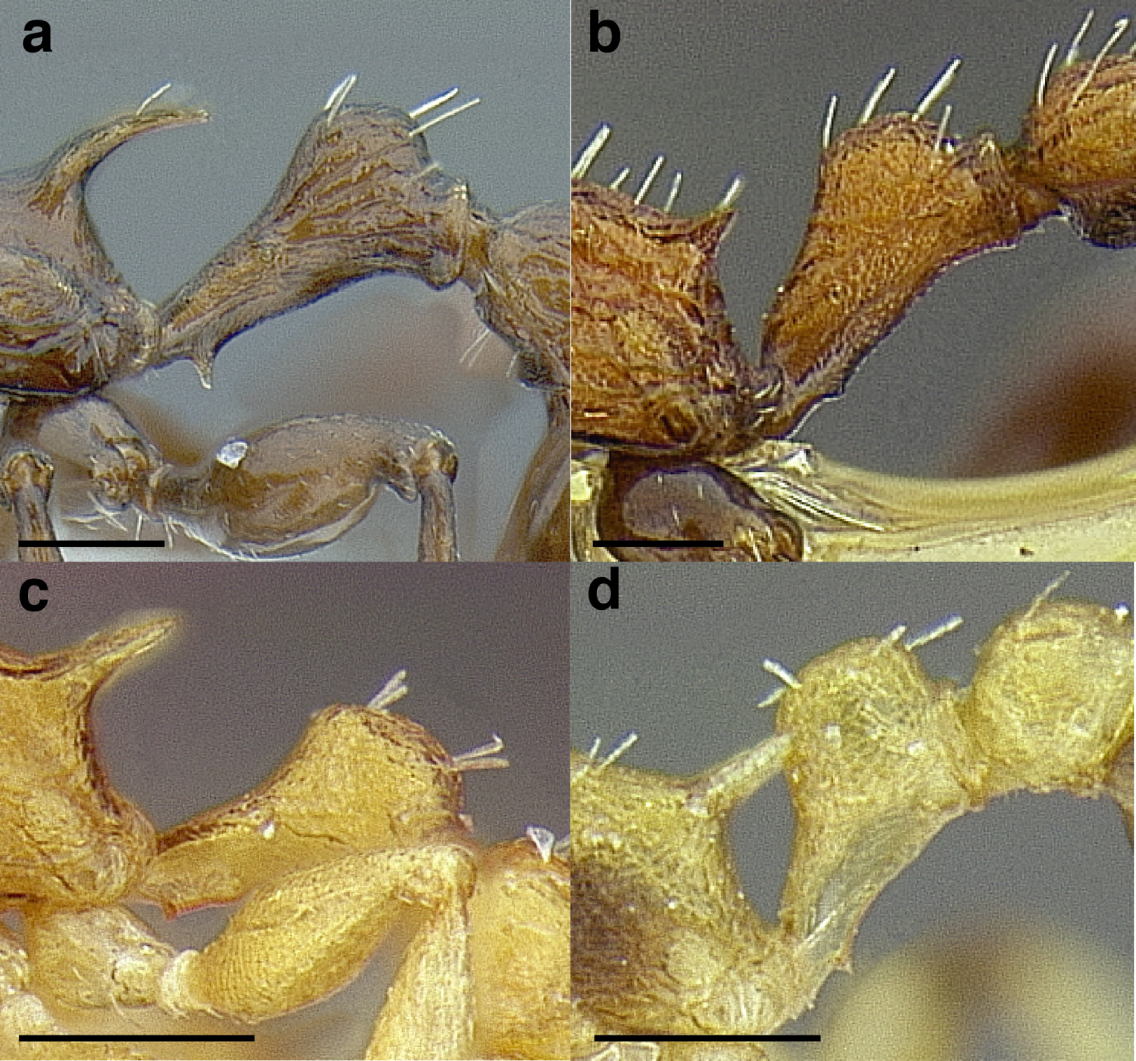
Comparison of *salvini* clade worker petiole morphology in profile view. (A) *Temnothorax fuscatus* (CASENT0916005). (B) *T. ocarinae* (USNMENT00531636). (C) *T. goniops* (MCZENT00032436). (D) *T. huehuetenangoi* (USNMENT00529517). Scale bars 0.2 mm.

**Figure 86:**
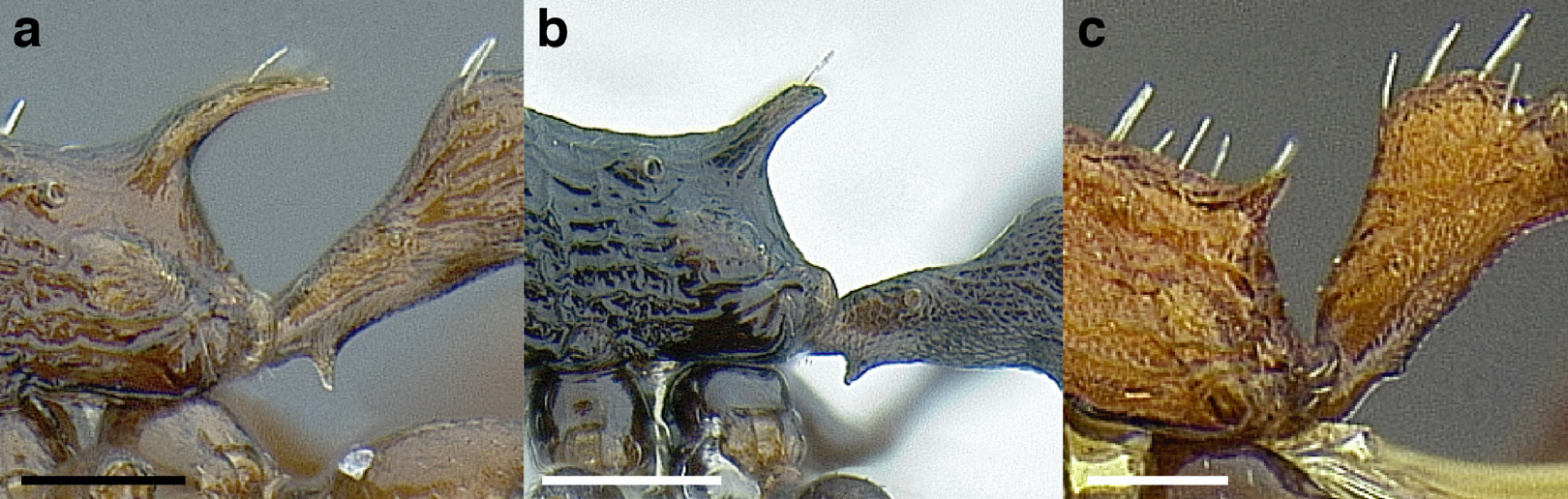
Comparison of worker propodeal spine morphology in the *fuscatus* group in profile view. (A) *Temnothorax fuscatus* (CASENT0916005) (B) *T. nebliselva* sp. nov. (INB0003659261) (C) *T. ocarinae* (USNMENT00531636). Scale bars 0.2 mm.

**Figure 87:**
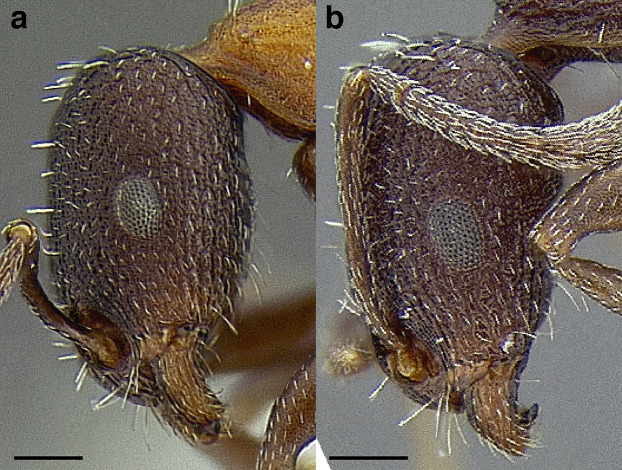
Comparison of worker compound eye morphology in the *fuscatus* species group in profile view. (A) *Temnothorax ocarinae* (USNMENT00531636). (B) *T. skwarrae* (MCZENT00016358). Scale bars 0.2 mm.

**Figure 88:**
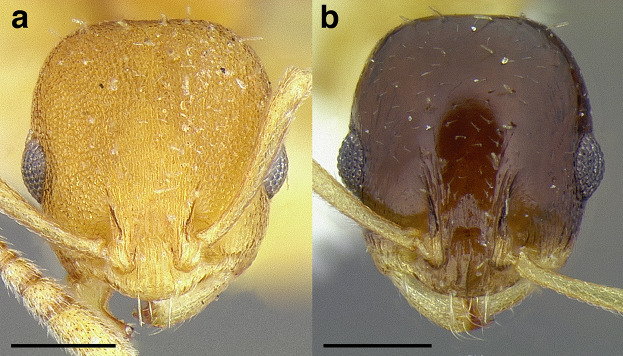
Comparison of worker head morphology in the *goniops* species group in full-face view. (A) *Temnothorax goniops* (MCZENT00032436). (B) *T. huehuetenangoi* (USNMENT00529517). Scale bars 0.2 mm.

**1.** Body without standing setae of any kind, except on the clypeus and borders of the gastral sclerites; propodeum strongly depressed; propodeal spines long and down curved; petiolar node subquadrate; southern Texas to Nicaragua***T. misomoschus* sp. nov. (*misomoschus* group)**
- Body with standing setae: may be sparse, but at least present on the dorsum of the head, dorsum of the pronotum, and posterior margin of the postpetiole; other characters variable; widespread2
**2.** Dorsal surfaces of legs and nearly all other surfaces of the body covered in long, tapering, suberect to subdecument setae (Figs. 37A–37C)3
- Dorsal surfaces of legs without long, tapering, suberect setae: long suberect setae, if present, are restricted to the ventral surface of the legs (Figs. 37D–37F)15
**3.** In profile view, promesonotum forming a bulging, even convexity; metanotal groove impressed; propodeum strongly depressed below the level of the promesonotum (Figs. 38A & 38B); ground- or litter-nesting species; U.S.A. to Nicaragua (*pergandei* group)4
- In profile view, promesonotum grading evenly into propodeum, not bulging; metanotal groove often obscure; propodeum not strongly depressed (Figs. 38C & 38D); nesting habitat variable5
**4.** Propodeal teeth present (Fig. 38A); North America, north of the Isthmus of Tehuantepec***T. pergandei* (Emery) (*pergandei* group)**
- Propodeal teeth absent (Fig. 38B); propodeal angles rounded and marked by a carina; Central America, south of the Isthmus of Tehuantepec***T. bison* sp. nov. (*pergandei* group)**
**5.** Mesosoma strongly arched in profile view (Figs. 39A & 39B)6
- Mesosoma not strongly arched in profile view: flat to slightly sinuate (Figs. 39C & 39D); arboreally-nesting species in Central America from Southern Mexico to Panama (*salvini* group, in part)7
**6.** Dorsum of head above compound eyes and dorsum of mesosoma smooth and shining (Figs. 39A & 39E); integument predominantly dark brown to black; nesting in hollow twigs in the leaf litter, in dead branches, or under bark near the ground on live trees; Sonoran Desert***T. politus* (Smith) (*subditivus* group, part)**
- Dorsum of head above compound eyes and dorsum of mesosoma areolate, with overlying rugose sculpture (Figs. 39B & 39F); integument predominantly yellow to testaceous; nesting habitat unknown, collected from leaf litter extractions; Southern Mexico to Honduras (*salvini* group, in part)***T. terraztecus* sp. nov. (*salvini* group, part)**
**7.** Petiolar node strongly squamiform, broadly expanded apically: PNWI ≥ 160, corners distinctly angulate in posterior view (Fig. 40A)8
- Petiolar node not as strongly squamiform, not as broadly expanded apically: PNWI ≤ 150, corners rounded in posterior view (Fig. 40B)11
**8.** Light yellow to color orange, subpetiolar tooth acutely spiniform (Fig. 41A)9
- Color dark brown, subpetiolar tooth minute and triangular (Fig. 41B)***T. longicaulis* stat. nov., nom. nov. (*salvini* group, part)**
**9.** Dorsum of head with strongly rugose sculpture overlying areolate sculpture; subpetiolar tooth shorter than the setae that arises from the peduncle directly above it (Fig. 42A; may be decumbent in some specimens); dorsum of petiolar node and postpetiole lightly sculptured; size larger: WL > 1.25 mm***T. aztecoides* sp. nov. (*salvini* group, part)**
- Dorsum of head with weaker, costate-rugose sculpture overlying areolate sculpture; subpetiolar tooth longer: equal to or longer than the setae that arise from the peduncle directly above it (Fig. 42B; may be decumbent in some specimens); dorsum of petiolar node and postpetiole smooth and shining to very weakly areolate-rugulose; size smaller: WL < 1.17 mm10
**10.** Propodeal spines longer than the propodeal declivity: PSI > 32 (Fig. 43A)***T. aztecus* (Wheeler) (*salvini* group, part)**
- Propodeal spines shorter than the propodeal declivity: PSI < 30 (Fig. 43B)***T. paraztecus* sp. nov. (*salvini* group, part)**
**11.** Subpetiolar tooth equal to or longer than the setae that arises from the peduncle directly above it (Fig. 44A; may be decumbent in some specimens)***T. longinoi* sp. nov. (*salvini* group, part)**
- Subpetiolar tooth shorter than the setae that arises from the peduncle directly above it (Fig. 44B; may be decumbent in some specimens)12
**12.** Propodeal spines long: PSI > 30 (Fig. 45A)***T. quetzal* sp. nov. (*salvini* group, part)**
- Propodeal spines shorter: PSI < 30 (Fig. 45B)13
**13.** Petiolar node not expanded apically: PNWI ≤ 130 (Fig. 46A)***T. salvini* (Forel) (*salvini* group, part)**
- Petiolar node expanded apically: PNWI ≥ 160 (Fig. 46B)14
**14.** Dorsum of mesosoma predominantly rugose; integument areolate between propodeal spines (Fig. 47A)***T. parvidentatus* sp. nov. (*salvini* group, part)**
- Dorsum of mesosoma predominantly costate; integument smooth and shining between propodeal spines (Fig. 47B)***T. fortispinosus* sp. nov. (*salvini* group, part)**
**15.** Antennal scapes with suberect pilosity (Fig. 48A); postpetiole moderately broad, 2.1 to 2.2 times the width of the caudal cylinder of the petiole in dorsal view (PSI 210-220); anterior quarter of first gastral tergite very weakly areolate; integument light yellow; Baja California***T. pilicornis* sp. nov. (*pilicornis* group)**
- Antennal scapes with subdecumbent to adpressed pilosity (Fig. 48B); postpetiole narrow to very broad; if first gastral tergite sculptured and integument yellow, then petiole narrower: less than 2 times the width of the caudal cylinder in dorsal view (PSI < 200); widespread16
**16.** Petiolar node cuneiform to subcuneiform (Figs. 49A–49C)17
- Petiolar node shaped otherwise (Figs. 49D–49F)18
**17.** Anterior clypeal margin emarginate; hind femora moderately to strongly incrassate (Figs. 50A & 50B); Southern U.S.A. and Northern Mexico (*rugosus* group, in part)***T. rugosus* (Mackay) (*rugosus* group, part)**
- Anterior clypeal margin entire; hind femora weakly incrassate (Figs. 50C & 50D); Southern Mexico (Chiapas)***T. acuminatus* sp. nov. (*acuminatus* group, part)**
**18.** Petiole with a transverse carina anterodorsally (Figs. 51A & 51B); postpetiole narrow, < 1.5 times as wide as the caudal cylinder of the petiole in dorsal view (PWI < 150) (Figs. 51C & 51D); Mexico19
- Petiole with a transverse carina dorsally or not (Fig. 51E); postpetiole relatively broad, ≥ 1.5 times as wide as the caudal cylinder of the petiole in dorsal view (PWI ≥ 150) (Figs. 51F–51H); widespread20
**19.** Anterior margin of clypeus entire (Fig. 52A); propodeum without many setae dorsally, except near extreme anterior near the metanotal groove and midway along the length of the propodeal spines (Fig. 52B); petiolar node dorsally rounded in profile view; first gastral sternite areolate-rugulose; Mexico (Veracruz)***T. tuxtlanus* sp. nov. (*acuminatus* group, part)**
- Anterior margin of clypeus emarginate (Fig. 52C); propodeum with at least one pair of setae dorsally (Fig. 52D); petiolar node dorsally truncate in profile view; first gastral sternite smooth; Mexico (Chihuahua; *rugosus* group, in part)***T. parralensis* sp. nov. (*rugosus* group, part)**
**20.** With the following characters in combination: anterior margin of the clypeus strongly emarginate medially; petiolar peduncle short to moderately long, comprising half the length of the petiole or less; petiolar node subquadrate, erect, and relatively short: NI < 170 (Fig. 53); arboreally-nesting species from Southwestern U.S.A. to South Central Mexico (*annexus* group)21
- Not having the above combination of characters; if clypeus is strongly emarginate, then petiolar peduncle is longer and/or petiolar node is not subquadrate; widespread25
**21.** Erect setae present on gula (Fig. 54A)22
- Setae, if present on gula, not erect (Fig. 54B)24
**22.** Head elongate: CI 80-84 (Fig. 55A)***T. balnearius* sp. nov. (*annexus* group)**
- Head less elongate: CI 86-90 (Figs. 55B & 55C)23
**23**. Petiolar node lower, not as strongly quadrate in profile view; posterior margin of petiolar node indistinct in profile view, much shorter than dorsal margin (Fig. 56A); first gastral tergite densely areolate***T. obtusigaster* sp. nov. (*annexus* group)**
- Petiolar node high and strongly quadrate in profile view; posterior margin of petiolar node distinct in profile view, about two thirds as long as dorsal margin (Fig. 56B); first gastral tergite smooth and shining***T. arbustus* sp. nov. (*annexus* group)**
**24.** Propodeal spines longer, broad; petiole pedunculate (Fig. 57A); body heavily sculptured: pronotum in profile view areolate with overlying rugose sculpture***T. annexus* (Baroni Urbani) (*annexus* group)**
- Propodeal spines shorter, less broad; peduncle of petiole shorter (Fig. 57B); body less heavily sculptured: pronotum in profile view predominantly areolate, without overlying rugose sculpture***T. quercicola* sp. nov. (*annexus* group)**
**25.** Propodeal spines long (PSI ≥ 22), acute, and directed dorsally: forming an angle of 130° or greater with the propodeal declivity in profile view (Fig. 58a–58C)26
- Propodeal spines, if directed dorsally, not long (PSI ≤ 20; Fig. 58D–58F)28
**26.** Dorsum of head weakly sculptured: smooth and shining between weak costulate sculpture; petiolar node erect and subquadrate (Figs. 59A & 59D); Southern Mexico***T. tenuisculptus* (Baroni Urbani) (*tenuisculptus* group)**
- Dorsum of head strongly sculptured: uniformly areolate-rugulose with overlying costulate or rugulose sculpture; petiolar node rounded dorsally (Figs. 59B, 59C, 59E, 59F); Southern Mexico to Honduras27
**27.** Postpetiole relatively narrow: < 1.7 times as wide as the caudal cylinder of the petiole in dorsal view (PWI < 170; Fig. 60A); Southern Mexico to Honduras***T. acutispinosus* sp. nov. (*acutispinosus* group)**
- Postpetiole relatively broad: about 2 times as wide as the caudal cylinder of the petiole in dorsal view (PWI ~ 200; Fig. 60B); Honduras***T. altinodus* sp. nov. (*altinodus* group)**
**28.** With the following characters in combination: dorsal surface of head uniformly areolate (Fig. 61A); petiolar peduncle moderately long, comprising less than half the petiolar length; petiolar node antero-posteriorly compressed and tall, with posterior face about as long as the anterior face in profile view (Fig. 61B); Texas and Northern Mexico***T. terrigena* (Wheeler) (*terrigena* group)**
- Not matching the above description; if dorsal surface of head is uniformly areolate and petiolar peduncle is moderately long, then the petiolar node is not antero-posteriorly compressed and tall; widespread29
**29.** Petiolar node tall and leaning posteriorly in profile view, overhanging the caudal cylinder of the petiole (Figs. 62A–62C); Mexico to Nicaragua (*augusti* group)30
- Petiolar node, if tall, not leaning posteriorly in profile view (Figs. 62D–62F); widespread33
**30.** Propodeal spines short: PSI < 35 (Fig. 63A)***T. augusti* (Baroni Urbani) (*augusti* group)**
- Propodeal spines longer: PSI > 35 (Fig. 63B)31
**31.** Head relatively broad: CI ≥ 84 (Fig. 64A).***T. leucacanthoides* sp. nov. (*augusti* group)**
- Head relatively narrow: CI ≤ 81 (Fig. 64B)32
**32.** Antennal scapes longer: SI > 100***T. aureus* sp. nov. (*augusti* group)**
- Antennal scapes shorter: SI ≤ 100***T. leucacanthus* (Baroni Urbani) (*augusti* group)**
**33.** Antennal scapes long, exceeding the posterior margin of the head by about two times the maximum width of the scape when fully retracted (SI > 110); dorsum of mesosoma not strongly arched dorsally in profile view; propodeal spines shorter than the propodeal declivity (PSI < 35); petiolar node subquadrate; Mexico (Puebla)***T. casanovai* sp. nov. (*casanovai* group)**
- Not matching the above description; if antennal scapes are long, mesosoma strongly arched dorsally, propodeal spines longer than the propodeal declivity, and petiolar node is squamiform; widespread34
**34.** With the following character combination: propodeal spines short (PSI < 30); in profile view, dorsum of petiolar node subquadrate to rounded, and bearing four to six erect setae (Figs. 65A–65C); in dorsal view, postpetiole moderately broad (190 < PWI < 235; Figs. 65D–65F); Southern Mexico to Guatemala (*goniops* group, in part)35
- Not matching the above description; widespread37
**35.** Dorsum of head and post-petiole predominantly smooth and shining (Figs. 66A & 66B)***T. xincai* sp. nov. (*goniops* group, part)**
- Dorsum of head and post-petiole areolate-rugulose (Figs. 66C & 66D)36
**36.** Petiolar node expanded apically: PNWI > 120 (Fig. 67A)***T. ixili* (Baroni Urbani) (*goniops* group, part)**
- Petiolar node not expanded apically: PNWI < 115 (Fig. 67B)***T. achii* sp. nov. (*goniops* group, part)**
**37.** Petiolar node squamiform (Figs. 68A–68D)38
- Petiolar node not squamiform (Figs. 68E–68H)42
**38.** In dorsal view, propodeal spines straight and evenly diverging: negative space between the propodeal spines “V” shaped, or a basally truncate “V” (Figs. 69A–69C); dorsum of petiolar node flat, sides evenly converging basally (Figs. 69A & 69C); widespread***T. subditivus* (Wheeler) (*subditivus* group, part)**
- In dorsal view, propodeal spines diverging, but curved toward the midline of the body: negative space between the propodeal spines “U” shaped, or a basally truncate “U” (Figs. 69D–69F); dorsum of petiolar node rounded to flat, sides bowed (Figs. 69D–69F); Bahamas and Cuba (*pastinifer* group, in part)39
**39.** Head predominantly smooth and shining (Fig. 70A)***T. schwarzi* (Mann) (*pastinifer* group, part)**
- Head sculptured: areolate to areolate-rugulose with overlying rugulose sculpture (Figs. 70B–70F)40
**40.** Anterior margin of clypeus strongly emarginated (Fig. 71A); in profile view, petiole strongly squamiform and overhanging the petiolar peduncle; petiole with only two erect setae dorsally, postpetiole with six or fewer (Fig. 71B)***T. pastinifer* (Emery) (*pastinifer* group, part)**
- Anterior margin of clypeus entire or, if weakly indented, head and mesosoma with well-defined rugae in addition to areolae (Fig. 71C); in profile view, petiole strongly squamiform or not; petiole with four or more erect setae, postpetiole with ten or more (Fig. 71D)41
**41.** Antennal scape long, surpassing posterior margin of head by > 2x the maximum width of the scape when fully retracted (SI ~ 106; Fig. 72A); petiolar node strongly enlarged apically (PNWI > 180; Fig. 72B)***T. rutabulafer* sp. nov. (*pastinifer* group, part)**
- Antennal scape short, barely surpassing the posterior margin of head when fully retracted (Fig. 72C); petiolar node weakly enlarged apically (PNWI < 160; Fig. 72D)***T. androsanus* (Wheeler) (*pastinifer* group, part)**
**42.** With the following character combination: antennal scapes surpassing the posterior margin of the head capsule by about one and a half times their length; dorsum of mesosoma strongly arched; petiole with a moderately long peduncle; petiolar node high, strongly rounded dorsally, and wider than the caudal cylinder of the petiole in dorsal view; postpetiole wide (215 < PWI < 240); bicolored species: head capsule dark, remainder of body light yellow; Cuba (*pastinifer* group, in part)***T. nigricans* (Baroni Urbani) (*pastinifer* group, part)**
- Not matching the above description; widespread43
**43.** Postpetiole moderately broad (150 < PWI < 220; Figs. 73A–73D); Mexico to Costa Rica58
- Postpetiole very broad (PWI > 220; Figs. 73E–73H); Southern Florida, Bahamas, Greater Antilles, Northern Lesser Antilles (*pulchellus* group)44
**44.** Hind femora strongly incrassate (FI > 370); propodeal spines short (PSI < 21) and petiole with > 4 erect setae (Fig. 74A); Hispaniola***T. laticrus* sp. nov. (*pulchellus* group)**
- Hind femora not as strongly incrassate (FI < 360); propodeal spines longer (PSI ≥ 30) and petiole with ≤ 4 erect setae (Fig. 74B); widespread, including Hispaniola45
**45.** Erect setae on dorsum of mesosoma very sparse, restricted to a pair arising from the promesonotum (Figs. 75A & 75B)46
- Standing setae on dorsum of mesosoma more abundant, with erect setae on other regions of the mesosoma, in addition to the promesonotum (Figs. 75C & 75D)47
**46.** Dorsum of head and mesosoma smooth and shining; dorsum of petiole with two erect setae; bicolored: head and gaster dark brown, remainder of body yellow; U.S. and British Virgin Islands***T. pulchellus* (Emery) (*pulchellus* group)**
- Dorsum of head and mesosoma areolate; dorsum of petiole without erect setae; uniformly testaceous yellow; Barbuda***T. wettereri* sp. nov. (*pulchellus* group)**
**47.** Petiole with only 2 erect setae (Figs. 76A –76C)48
- Petiole with 4 erect setae (Figs. 76D–76F)50
**48.** Head predominantly smooth and shining, with weak costulate sculpture along the medial edges of the frontal carinae (Fig. 77A); Cuba49
- Head with stronger sculpture, either with a smooth central strip surrounded by weakly areolate and costulate sculpture, or with the head uniformly areolate (Figs. 77B & 77C); southern Florida, Bahamas, and Cuba***T. torrei* (Aguayo) (*pulchellus* group)**
**49.** In full-face view, posterior margin of head passing into the lateral margins through a broad curve, giving the head an ovular shape (Fig. 78A); in dorsal view, promesonotal suture deeply impressed and extending to the dorsal face of the mesosoma (Fig. 78B); in profile view, petiolar node subquadrate: dorsal face transitioning to posterior face through a rounded ~90° angle (Fig. 76C)***T. hippolytus* sp. nov. (*pulchellus* group)**
- In full-face view, posterior margin of head passing into the lateral margins through a rounded angle, giving the head a boxy shape (Fig. 78C); in dorsal view, promesonotal suture not deeply impressed (Fig. 78D); in profile view, petiolar node not subquadrate: dorsal face transitioning to posterior face through broad curve (Fig. 76B)***T. terricola* (Mann) (*pulchellus* group)**
**50.** Dorsum of head uniformly areolate (Figs. 79A–79C)51
- Dorsum of head smooth and shining, or sculptured otherwise (Figs. 79D –79F )52
**51.** In full-face view, head with posterior margin weakly concave (Figs. 79A & 79C)***T. agavicola* sp. nov. (*pulchellus* group)**
- In full-face view, head with posterior margin flat to convex (Fig. 79B)***T. albispinus* (Wheeler) (*pulchellus* group)**
**52.** Metapleural gland bulla large, extending more than halfway between the metacoxal insertion and the propodeal spiracle (Fig. 80A); U.S. Virgin Islands***T. magnabulla* sp. nov. (*pulchellus* group)**
- Metapleural gland bulla smaller, extending about halfway between the metacoxal insertion and the propodeal spiracle (Figs. 80B–80D); Hispaniola53
**53.** Mesosoma smooth and shining dorsally (Figs. 81A & 81D); dorsum of mesosoma strongly convex in profile view (Figs. 81B & 81C)54
- Mesosoma areolate-rugulose dorsally (Figs. 81E & 81H); dorsum of mesosoma weakly convex in profile view (Figs. 81F & 81G)56
**54.** Uniformly yellow***T. flavidulus* (Wheeler & Mann) (*pulchellus* group)**
- Color variable, but never uniformly yellow55
**55.** On anterior margin of clypeus, medial two pairs of setae about the same width (Fig. 82A); strikingly bicolored: head, mesosoma and waist segments dark brown; remainder bright yellow***T. harlequina* sp. nov. (*pulchellus* group)**
- On anterior margin of clypeus, pair of setae flanking the medial pair about twice the width of the medial pair (Fig. 82B); integument color variable, but if bicolored, gaster never uniformly bright yellow***T. ciferrii* (Menozzi & Russo) (*pulchellus* group)**
**56.** Anterior clypeal margin emarginated medially (Fig. 83A)***T. wilsoni* sp. nov. (*pulchellus* group)**
- Anterior clypeal margin flat to evenly convex (Fig. 83B)57
**57.** Petiole long relative to the postpetiole (PLI 148–166; Fig. 84A)***T. balaclava* sp. nov. (*pulchellus* group)**
- Petiole short relative to the postpetiole (PLI 140–143; Fig. 84B)***T. bahoruco* sp. nov. (*pulchellus* group)**
**58.** Petiolar node low, elongate (NI > 170; Figs. 85A & 85B); Mexico to Costa Rica (*fuscatus* group)59
- Petiolar node high, relatively short (NI < 150; Fig. 85C & 85D); Southern Mexico and Guatemala (*goniops* group, in part)62
**59.** Propodeal spines long: PSI > 22 (Figs. 86A & 86B); body uniformly colored60
- Propodeal spines short: PSI < 19 (Fig. 86C); bicolored: either with mesosoma red and rest of body dark brown, or with gaster yellow and body dark brown61
**60.** Propodeal spines acute (Fig. 86A)***T. fuscatus* (Mann) (*fuscatus* group)**
- Propodeal spines truncate (Fig. 86B)***T. nebliselva* sp. nov. (*fuscatus* group)**
**61.** Eye small and round: in profile view, distance between the compound eye and base of mandible greater than the maximum length of the eye, OI ≤ 22 (Fig. 87A); body predominantly dark brown, contrasting with a red mesosoma***T. ocarinae* (Baroni Urbani) (*fuscatus* group)**
- Eye larger and oval: in profile view, distance between the compound eye and base of mandible roughly equal to the maximum length of the eye, OI ≥ 24 (Fig. 87B); body predominantly dark brown, with a contrasting bright yellow gaster***T. skwarrae* (Wheeler) (*fuscatus* group)**
**62.** Head sculptured: dorsum uniformly areolate-rugose (Fig. 88A)***T. goniops* (Baroni Urbani) (*goniops* group, part)**
- Dorsum of head predominantly smooth and shining (Fig. 88B)***T. huehuetenangoi* (Baroni Urbani) (*goniops* group, part)**

### Key to species groups of the *salvini* clade based on reproductive caste forewings

**Figure 89:**
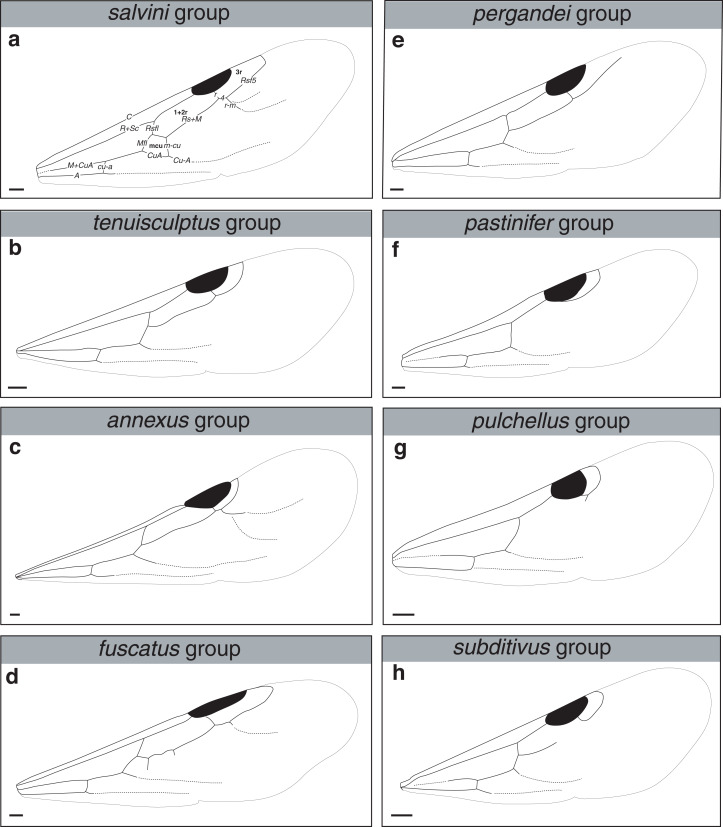
Illustrations of wing venation in the *salvini* clade based on Brown & Nutting (1950). (A) *Temnothorax aztecoides* sp. nov. gyne (CASENT0758789). (B) *T. tenuisculptus* gyne (CASENT0756078). (C) *T. quercicola* sp. nov. gyne (CASENT0869108). (D) *T. fuscatus* gyne (CASENT0615617). (E) *T. pergandei* gyne (CASENT0758229). (F) *T. rutabulafer* sp. nov. gyne (CASENT0758831). (G) *T. laticrus* sp. nov. gyne (MCZENT00510556). (H) *T. politus* gyne (CASENT0758021). Scale bars 0.2 mm.

**1.** Cell 1+2r present (Figs. 89A–89E)**2**
- Cell 1+2r absent, although vein Rs+M may be present and incomplete (Figs. 89F–89H)**6**
**2.** Vein Rsf5 not meeting anterior wing margin (Fig. 89E)***pergandei* group**
- Vein Rsf5 meeting anterior wing margin (Figs. 89A–89D)**3**
**3.** Cell mcu present or nearly complete (Figs. 89A & 89D)**4**
- Cell mcu absent (Figs. 89B & 89C)**5**
**4.** Vein r as long or longer than width of pterostigma (Fig. 89D)***fuscatus* group**
- Vein r shorter than width of pterostigma (Fig. 89A)***salvini* group**
**5.** Vein r-m present (Fig. 89C)***annexus* group**
- Vein r-m absent (Fig. 89B)***tenuisculptus* group**
**6.** Vein Rs+M present (Fig. 89H)***subditivus* group**
- Vein Rs+M absent (Figs. 89F & 89G)**7**
**7.** Vein connecting M+CuA and R+Sc straight (Fig. 89F)***pastinifer* group**
- Vein connecting M+CuA and R+Sc bent (Fig. 89g)***pulchellus* group**

### Synopsis of the *salvini* clade

### *acuminatus* group

*T. acuminatus* sp. nov.

*T. tuxtlanus* sp. nov.

### *acutispinosus* group

*T. acutispinosus* sp. nov.

### *altinodus* group

*T. altinodus* sp. nov.

### *annexus* group

*T. annexus* (Baroni Urbani)

*T. arbustus* sp. nov.

*T. balnearius* sp. nov.

*T. obtusigaster* sp. nov.

*T. quercicola* sp. nov.

### *augusti* group

*T. augusti* (Baroni Urbani)

*T. aureus* sp. nov.

*T. leucacanthoides* sp. nov.

*T. leucacanthus* (Baroni Urbani)

### *casanovai* group

*T. casanovai* sp. nov.

### *fuscatus* group

*T. fuscatus* (Mann)

*T. nebliselva* sp. nov.

*T. ocarinae* (Baroni Urbani)

*T. skwarrae* (Wheeler)

### *goniops* group

*T. achii* sp. nov.

*T. goniops* (Baroni Urbani)

*T. huehuetenangoi* (Baroni Urbani)

*T. ixili* (Baroni Urbani)

*T. xincai* sp. nov.

### *misomoschus* group

*T. misomoschus* sp. nov.

### *pastinifer* group

*T. androsanus* (Wheeler)

*T. nigricans* (Baroni Urbani)

*T. pastinifer* (Emery)

*T. rutabulafer* sp. nov.

*T. schwarzi* (Mann)

### *pergandei* group

*T. bison* sp. nov.

*T. pergandei* (Emery)

### *pilicornis* group

*T. pilicornis* sp. nov.

### *pulchellus* group

*T. agavicola* sp. nov.

*T. albispinus* (Wheeler)

*T. bahoruco* sp. nov.

*T. balaclava* sp. nov.

*T. ciferrii* (Menozzi & Russo)

*T. flavidulus* (Wheeler & Mann)

*T. harlequina* sp. nov.

*T. hippolytus* sp. nov.

*T. laticrus* sp. nov.

*T. magnabulla* sp. nov.

*T. pulchellus* (Emery)

*T. terricola* (Mann)

*T. torrei* (Aguayo)

*T. wettereri* sp. nov.

*T. wilsoni* sp. nov.

### *rugosus* group

*T. parralensis* sp. nov.

*T. rugosus* (Mackay)

### *salvini* group

*T. aztecoides* sp. nov.

*T. aztecus* (Wheeler)

*T. fortispinosus* sp. nov.

*T. longinoi* sp. nov.

*T. longicaulis* stat. nov., nom. nov.

*T. paraztecus* sp. nov.

*T. parvidentatus* sp. nov.

*T. quetzal* sp. nov.

*T. salvini* (Forel)

*T. terraztecus* sp. nov.

### *subditivus* group

*T. politus* (Smith)

*T. subditivus* (Wheeler)

### *tenuisculptus* group

*T. tenuisculptus* (Baroni Urbani)

### *terrigena* group

*T. terrigena* (Wheeler)

## Taxon Treatments

### *acuminatus* group overview

**Figure 90:**
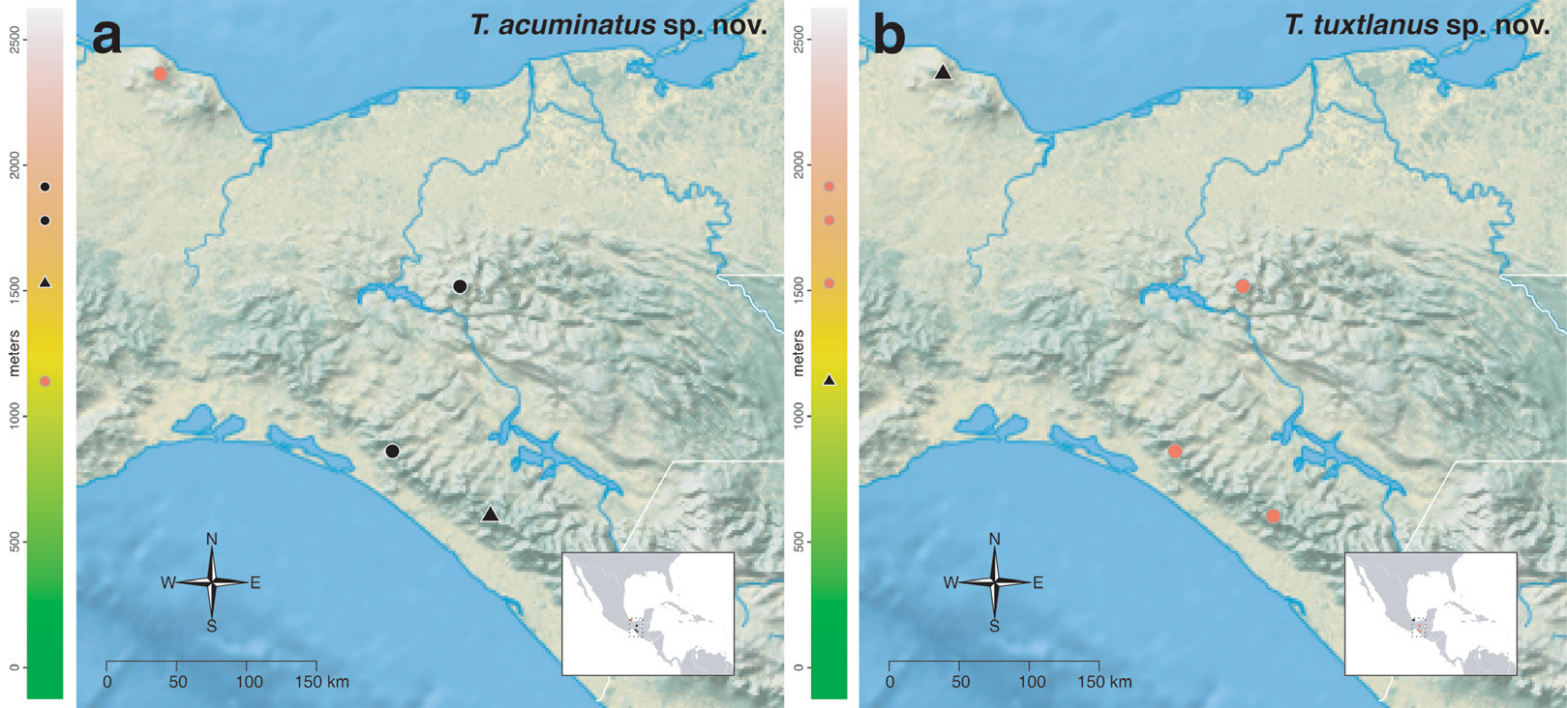
Geographical and elevational distribution of the *acuminatus* group. (A) *Temnothorax acuminatus* sp. nov. (B) *T. tuxtlanus* sp. nov. Colored scale to the left of each map represents elevation in meters. Points in black represent the species named in each subfigure, while points in red represent other members of the species group. Type localities are represented by triangles, non-type localities are represented by circles. Bounding box in inset map shows location of main map.

This group is composed of two previously undescribed species, *Temnothorax acuminatus* sp. nov. and *T. tuxtlanus* sp. nov., which are found in Southern Mexico, from the mountain complexes of Los Tuxtlas and the Sierra Madre de Chiapas at mid-to-high elevations (Fig. 90). These species are united by the lack of setae on the dorsum of the propodeum, reduced subpetiolar tooth, moderately impressed metanotal groove, posterodorsally directed propodeal spines, and dark coloration. Members of this group have been collected from bark crevices, from under epiphyte mats on treefalls, and from sifted leaf litter. Species of the *acuminatus* group may be confused with *T. acutispinosus* sp. nov., which has an overlapping distribution, similar habitus, and lack of setae on the propodeum. *Temnothorax acutispinosus* sp. nov. can be distinguished from the *acuminatus* group by the larger subpetiolar tooth and dorsally directed propodeal spines. The two species of the *acuminatus* group are sister to the remainder of the *salvini* clade (Prebus, in prep.).

***Temnothorax acuminatus* sp. nov.**

**Figure 91:**
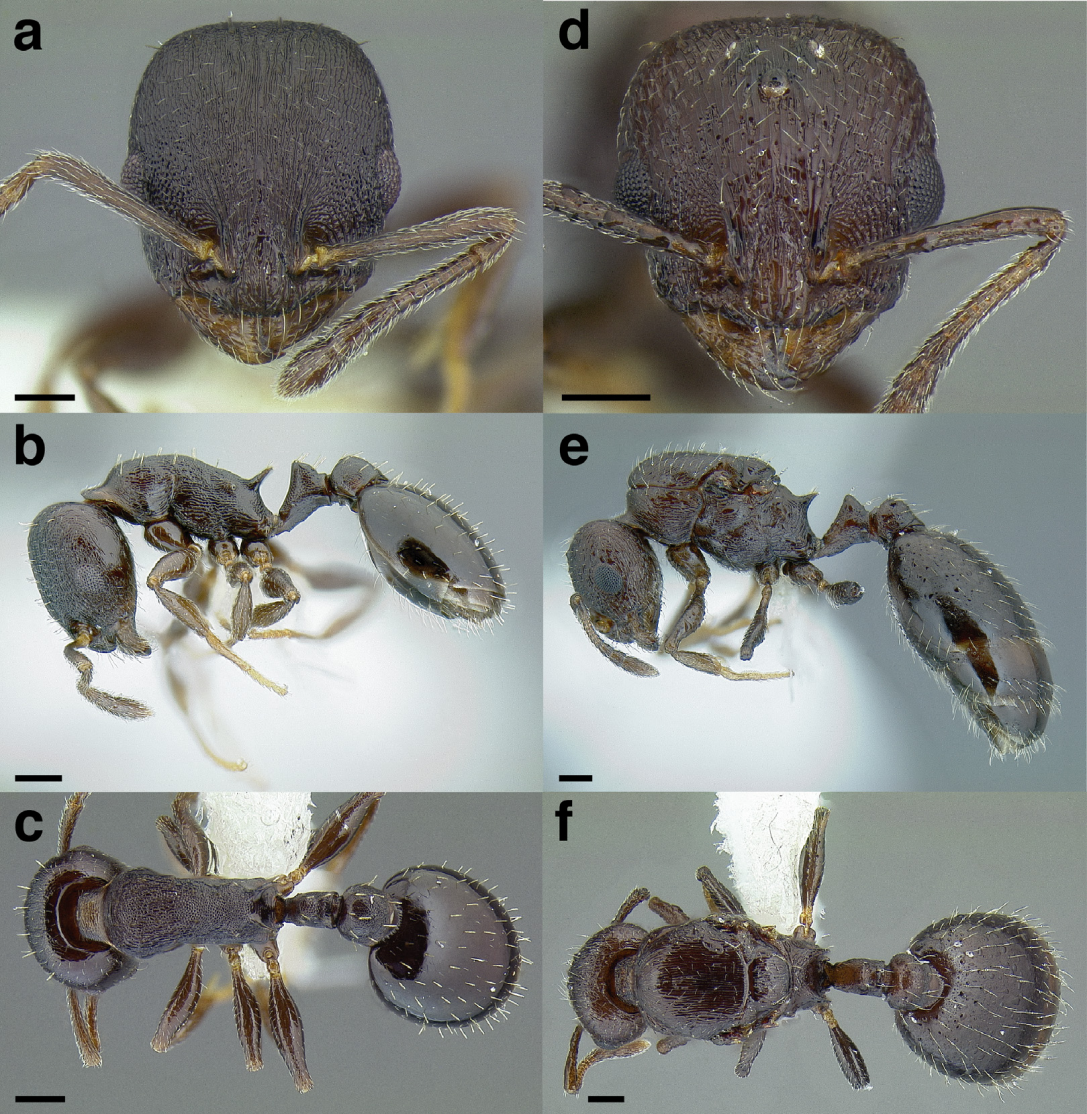
*Temnothorax acuminatus* sp. nov. (A–C) Holotype worker (JTLC000007439). (A) Full-face view. (B) Profile view. (C) Dorsal view. (D–F) Paratype gyne (JTLC000007440). (D) Full-face view. (E) Profile view. (F) Dorsal view. Scale bars 0.2 mm.

Distribution: Fig. 90A; worker & gyne: Fig. 91.

*Temnothorax* mmp07 Prebus, 2017: 8. In phylogeny.

**Type material examined:** *Holotype worker:* MEXICO: Chiapas: Custepec, 15.72196°N 92.95037°W ± 50 m, 1,530 m, 19 May 2008, J. Longino#6281, mesophyll forest, ex bark crevice in treefall (JTLC000007439) [CASC].

*Paratype workers and gynes:* same data as holotype, 1 dealate gyne (JTLC000007440) [CASC]; same data as holotype, except: J. Longino#6286, under epiphytes in treefall, 1 dealate gyne (JTLC000007452) [MCZC] 1 worker (JTLC000007451) [MCZC] 1 worker (CASENT0869118) [LACM]; same data as holotype, except J. Longino #6278-s, mesophyll forest, foragers, 1 worker (JTLC000007468) [USNM].

**Non-type material examined:** MEXICO: Chiapas: 5 km NNW Coapilla, 17.18276°N 93.15187°W, 1,915 m, 25 May 2008, LLAMA#Wa-A-04-2-20, secondary mesophyll forest, ex sifted leaf litter, 1 dealate gyne (JTLC000014336) [USNM]; same data as previous, except: 17.18330°N 93.15209°W, 1,915 m, 25 May 2008, LLAMA#Wa-A-04-2-07, secondary mesophyll forest, ex sifted leaf litter, 1 worker (JTLC000014329) [UCDC]; Sierra Morena, 16.13462°N 93.60080°W ± 100 m, 1,780 m, 14 May 2008, D.J. Cox#0031b, at bait along trail, 1 worker (CASENT0609711) [UCDC].

**Geographic range:** Southern Mexico, mid-to-high elevations in the Sierra Madre de Chiapas (Fig. 90A).

**Worker diagnosis:** *Temnothorax acuminatus* sp. nov. can be separated from all other members of the *salvini* clade by the following character combination: dorsum of mesosoma very weakly sinuate in profile view; dorsum of propodeum without erect setae; petiole with a weak transverse carina anterodorsally; petiolar peduncle short, comprising about a quarter of the total petiole length; petiolar node cuneiform; postpetiole relatively narrow (PWI < 170); setae on head, mesosoma, waist segments and gaster erect, short, sparse and blunt (never long and tapering); integument dark brown, with mandibles, pronotal neck, tibiae, tarsi, and sting testaceous brown.

**Similar species:** *Temnothorax achii* sp. nov., *T. acutispinosus* sp. nov., *T. altinodus* sp. nov., *T. ixili, T. subditivus, T. tuxtlanus* sp. nov., *T. xincai* sp. nov., members of the *augusti, goniops*, and *pulchellus* groups, and species of the *sallei* clade. *T. acuminatus* sp. nov. may be separated from *T. acutispinosus* sp. nov. by the cuneiform petiolar node (rounded dorsally in *T. acutispinosus* sp. nov.), smaller subpetiolar tooth, and more posteriorly directed propodeal spines. *Temnothorax acuminatus* sp. nov. can be distinguished from similar appearing members of the *sallei* clade (e.g. *T. manni* (Wheeler), *T. mexicanus* (Mackay), *T. punctithorax* (Mackay)) by the sculpture of the head: in similar looking *sallei* clade species, the head is mostly smooth and shining, with concentric rugulae surrounding the antennal insertions; additionally, the medial lobe of the clypeus is typically smooth, with three distinct longitudinal carina, whereas it is densely costulate-rugulose in *T. acuminatus* sp. nov. In contrast to all of the remaining species above, *T. acuminatus* sp. nov. has a cuneiform petiolar node, a feature not found in other members of the *salvini* clade, except for *T. rugosus*. *Temnothorax acuminatus* sp. nov. is easily separated from *T. rugosus* by the smaller size, lack of medial notch in the anterior margin of the clypeus, lack of setae on the propodeal dorsum, and hind femora that are not incrassate.

**Worker measurements & indices (*n* = 5):** SL = 0.450-0.509 (0.475); FRS = 0.175–0.219 (0.198); CW = 0.575–0.641 (0.611); CWb = 0.542–0.614 (0.575); PoOC = 0.282–0.316 (0.292); CL = 0.645–0.713 (0.666); EL = 0.143–0.164 (0.151); EW = 0.093–0.112 (0.102); MD = 0.132–0.153 (0.141); WL = 0.752–0.837 (0.785); SPST = 0.178–0.195 (0.189); MPST = 0.244–0.286 (0.261); PEL = 0.255–0.278 (0.269); NOL = 0.141–0.159 (0.151); NOH = 0.114–0.128 (0.119); PEH = 0.216–0.240 (0.227); PPL = 0.172–0.211 (0.191); PW = 0.363–0.418 (0.382); SBPA = 0.156–0.184 (0.168); SPTI = 0.187–0.239 (0.207); PEW = 0.147–0.166 (0.154); PNW = 0.112–0.115 (0.114); PPW = 0.227–0.243 (0.235); HFL = 0.487–0.539 (0.510); HFWmax = 0.118–0.131 (0.125); HFWmin = 0.044–0.048 (0.047); CS = 0.865–0.971 (0.908); ES = 0.192–0.218 (0.202); SI = 80–85 (83); OI = 22; CI = 84-88 (86); WLI = 132–139 (137); SBI = 27–30 (29); PSI = 22–26 (24); PWI = 144–164 (152); PLI = 131–158 (142); NI = 112–139 (127); PNWI = 68–78 (74); NLI = 52–61 (56); FI = 265–273 (269).

**Worker description:** In full-face view, head subquadrate, longer than broad (CI 84–88). Mandibles weakly, finely striate but shining and armed with five teeth: the apical-most well developed and acute, followed by a less developed preapical tooth and three equally developed smaller teeth. Anterior clypeal margin weakly convex. Antennal scapes short: when fully retracted, failing to reach the posterior margin of the head capsule by about the maximum width of the scape (SI 80–85). Antennae 12-segmented; antennal club of composed of three segments, with the apical-most segment slightly longer than the preceding two in combination. Frontal carinae short, extending past the antennal toruli by about one and a half times the maximum width of the antennal scape. Compound eyes weakly protruding past the lateral margins of the head capsule. Lateral margin of head weakly convex, forming a continuous arc from the mandibular insertions to the posterior margin of the head. Posterior head margin weakly concave medially, rounding evenly into the lateral margins.

In profile view, compound eyes elongate-ovular and small (OI 22), with 10 ommatidia in longest row. Pronotal declivity indistinct, neck and anterior face of pronotum forming a ~120° angle. Mesosoma weakly sinuate: evenly convex from where it joins the pronotal neck to the metanotal groove; propodeum slightly depressed below the level of the promesonotum, and weakly convex. Promesonotal suture extending from the posterior margin of the procoxal insertion to the mesothoracic spiracle, which is moderately well developed, then continuing dorsally as a weak sulcus. Metanotal groove visible as a disruption of the sculpture laterally from where it arises between the mid- and hind coxae to the poorly developed metathoracic spiracle, which is nearly indistinguishable against the ground sculpture, then continuing dorsally as a weak impression. Propodeal spiracle weakly developed, directed posterolaterally, and separated from the propodeal declivity by about four spiracle diameters. Propodeal spines short (PSI 22–26), about two thirds the length of the propodeal declivity, tapering evenly from the base, straight, and acute. Propodeal declivity with a weak carinae joining base of propodeal spine and propodeal lobe; weakly concave, forming a rounded ~120° angle with the base of the propodeal spines. Propodeal lobes rounded and weakly developed, but dorsal margin weakly angulate. Metapleural gland bulla small, extending from the metacoxal insertion halfway to the propodeal spiracle. Petiole short (PLI 131–158), with tubercles anterodorsally. Subpetiolar process in the form of a small, blunt, triangular tooth which grades evenly into the ventral margin of the petiole posteriorly; ventral margin of petiole weakly bulging posterior to it. Petiolar peduncle short: comprising about a quarter of the total petiole length. Petiolar node erect and cuneiform: peduncle grading evenly into the anterior node face; anterior face forming a ~70° angle with the evenly rounded posterior face; posterior face forms a ~120° angle with the caudal cylinder. Postpetiole weakly rounded anteriorly, anterior face rounds evenly into the weakly rounded dorsal face; weakly lobed ventrally.

In dorsal view, humeri developed and distinct: evenly rounded and wider than the rest of the mesosoma; mesothoracic spiracles weakly protruding past the lateral margins of the mesosoma, visible as slight angles where the pronotum meets the mesonotum. Promesonotal groove visible as a weak sulcus and disruption in the ground sculpture. Metanotal groove visible as a weak impression. Propodeal spines broadly approximated basally and weakly diverging apically, their apices separated from each other by about twice their length, the negative space between them “U” shaped. Petiolar peduncle with spiracles weakly protruding past the lateral margins, but not noticeably constricted anterior to them. Petiolar node, when viewed at a posterodorsal aspect, tapering evenly from the base, with the dorsal margin evenly and strongly convex; apex of node narrower than the peduncle and caudal cylinder. Postpetiole narrow (PWI 144–164) and subquadrate. Anterior margin of the postpetiole flat and evenly rounds into the lateral margins through a ~90° angle; lateral margins weakly converge to the rounded posterior corners; posterior margin broadly concave. Metafemur moderately incrassate (FI 265–273).

Sculpture: median clypeal carina present, extending posteriorly nearly to the frontal triangle, and flanked on either side by multiple weaker carinae; clypeus appears costulate-rugulose. Lateral clypeal lobes with additional, weaker carinae; ground sculpture weakly areolate. Antennal scapes weakly areolate. Cephalic dorsum predominantly areolate-rugulose, with the areolae arranged into longitudinal rows by fine costulae; very fine concentric rugulae surrounding the antennal insertions. Lateral surfaces of head sculptured similarly to the dorsum, but with areolae arranged into concentric whorls by fine costulae above the compound eyes; fine rugulose sculpture overlying the areolate sculpture between the compound eye and mandibular insertion. Ventral surface of head smooth and shining. Pronotal neck and anterior face of the pronotum shining through weak, transversely arranged areolae. Lateral surface of the pronotum shining medially, with weak costulae and areolae around the margins. Meso- and metapleurae areolate, with fine costulae overlying the ground sculpture. Smooth and shining between the propodeal spiracle and the propodeal spines. Dorsal surface of mesosoma uniformly areolate, with fine strigulae on the anterior margin of the pronotum. Femora shining, with weak areolate sculpture on the distal third. Petiole predominantly weakly areolate; anterior face of petiolar node smooth and shining. Postpetiole predominantly weakly areolate; anterior face smooth and shining. First gastral tergite and sternite smooth and shining, without spectral iridescence.

Setae: antennal scapes and funiculi with short, decumbent pilosity. Dorsum of the head, pronotum, waist segments, and gaster with moderately abundant, erect, blunt-tipped setae, the longest of which are about half the width of the compound eye. The head bears ~32, mesosoma ~16, petiole 4, postpetiole ~8, and first gastral tergite ~56 setae. Short, sparse pubescence present over the entire body, but difficult to detect against the densely sculptured integument.

Color: predominantly dark brown, with mandibles, pronotal neck, tibiae, tarsi, and sting testaceous brown.

**Gyne measurements & indices (*n* = 3):** SL = 0.495–0.513 (0.506); FRS = 0.22–0.231 (0.226); CW = 0.72–0.739 (0.727); CWb = 0.668–0.719 (0.691); PoOC = 0.295–0.304 (0.3); CL = 0.727–0.75 (0.739); EL = 0.212–0.22 (0.217); EW = 0.157–0.173 (0.164); MD = 0.139–0.159 (0.146); WL = 1.171–1.205 (1.193); SPST = 0.211–0.272 (0.247); MPST = 0.278–0.333 (0.307); PEL = 0.312–0.365 (0.343); NOL = 0.17–0.217 (0.197); NOH = 0.131–0.146 (0.139); PEH = 0.273–0.293 (0.28); PPL = 0.215–0.242 (0.225); APT = 1–1 (1); PW = 0.651–0.699 (0.677); SBPA = 0.305–0.325 (0.312); SPTI = 0.305–0.319 (0.311); PEW = 0.189–0.212 (0.204); PNW = 0.126–0.146 (0.138); PPW = 0.301–0.33 (0.314); HFL = 0.59–0.614 (0.603); HFWmax = 0.128–0.152 (0.138); HFWmin = 0.053–0.065 (0.059); CS = 1.032–1.094 (1.061); ES = 0.299–0.3 (0.299); SI = 71–74 (73); OI = 27–29 (28); CI = 92–96 (93); WLI = 167–176 (173); SBI = 43–47 (45); PSI = 18–23 (21); PWI = 147–159 (154); PLI = 145–168 (153); NI = 122–166 (143); PNWI = 59–75 (68); NLI = 54–62 (57); FI = 205–258 (235).

**Gyne description:** In full-face view, head subquadrate, slightly longer than broad (CI 92–96). Mandibles weakly striate but shining and armed with five teeth: the apical-most well developed, followed by a less developed preapical tooth and three equally developed smaller teeth. Anterior clypeal margin evenly convex medially. Frontal carinae moderately long, extending past the antennal toruli by about two times the maximum width of the antennal scape. Antennal scapes short: when fully retracted, failing to reach the posterior margin of the head capsule by about two times the maximum width of the scape (SI 71–74). Antennae 12-segmented; antennal club composed of three segments, with the apical-most segment slightly longer than the preceding two in combination. Compound eyes weakly protruding past the lateral margins of the head capsule. Lateral margins of head evenly convex behind the compound eyes, then parallel to each other from the mandibular insertions to below the compound eyes. Posterior head margin flat, rounding evenly into the lateral margins.

In profile view, compound eyes ovular and large (OI 27–29), with 17 ommatidia in longest row. Mesoscutum rounded evenly anteriorly, covering the dorsal surface of the pronotum, and flat dorsally. Mesoscutellum on the same plane as the mesoscutum; rounded posteriorly. Posterior margin of metanotum extending past the posterior margin of the mesoscutum. Propodeal spiracle moderately well developed, directed posterolaterally, and separated from the propodeal declivity by about four spiracle diameters. Propodeal spines thin and short (PSI 18–23), about a third as long as the propodeal declivity, tapering evenly from the base, straight, and blunt. Propodeal declivity with a carina joining base of propodeal spine and propodeal lobe; straight and flat, forming a rounded ~120° angle with the base of the propodeal spines. Propodeal lobes rounded and very weakly developed, but dorsal margin slightly angulate. Metapleural gland bulla small, extending from the metacoxal insertion halfway to the propodeal spiracle. Petiole moderately long (PLI 145–168), with flanges anterodorsally. Subpetiolar process in the form of a small, blunt, triangular tooth, which grades evenly into the ventral margin of the petiole posteriorly. Petiolar peduncle short: comprising about a third of the total petiole length. Petiolar node erect and cuneiform: peduncle transitioning evenly into the anterior node face; anterior face forming a blunt ~80° angle with the posterior face; posterior face forms a ~110° angle with the caudal cylinder. Postpetiole flat anteriorly, bulging slightly anterodorsally before it transitions into the flattened posterodorsal face; ventral surface weakly lobed.

In dorsal view, mesoscutum covering pronotum anteriorly, but humeri visible laterally as rounded sclerites. Propodeal spines parallel to each other, their apices separated from each other by about two and a half times their length. Petiolar peduncle with spiracles protruding past the lateral margins. Petiolar node, when viewed at a posterodorsal aspect, tapering dorsally; dorsal margin evenly convex. Apex of petiolar node about half as wide as the base; narrower than the peduncle caudal cylinder. Postpetiole narrow (PWI 147–159) and subquadrate. Anterior margin of postpetiole flat, with corners marked by rounded ~90° angles as it transitions to the lateral margins, which converge slightly to the rounded posterior corners; posterior margin flat. Metafemur weakly incrassate (FI 205–258).

Sculpture: median clypeal carina present, extending from the anterior margin nearly to frontal triangle, and flanked by weaker, indistinct carinae; clypeus appears costulate-rugulose. Lateral clypeal lobes with additional weaker carinae; ground sculpture weakly areolate. Antennal scapes weakly areolate. Cephalic dorsum areolate-rugulose, with fine costulae arranging the areolae into columns. Fine concentric costulae surrounding the antennal insertions. Lateral surfaces of head sculptured similarly to the dorsum, but with areolae arranged into concentric whorls by fine costulae above the compound eyes; areolate-rugulose sculpture between the compound eye and mandibular insertion. Ventral surface of head shining, with weak costulae. Pronotal neck finely strigulate. Anterior face of pronotum smooth and shining. Lateral face of pronotum, katepisternum, and anepisternum weakly areolate, with weak costulae. Metapleural gland bulla with costate sculpture overlying it. Lateral face of propodeum areolate-strigulate. Propodeal declivity weakly areolate. Mesoscutum and mesoscutellum with costulae over weak areolate ground sculpture. Dorsum of propodeum areolate with concentric costulae overlying the ground sculpture. Femora smooth and shining, with traces of weak areolate sculpture. Petiole predominantly areolate-rugulose; anterior face of petiolar node smooth and shining. Postpetiole predominantly areolate-rugulose; anterior face smooth and shining. First gastral tergite and sternite smooth and shining, without spectral iridescence.

Setae: antennal scapes and funiculi with short, decumbent pilosity. Dorsum of the head, pronotum, waist segments, and gaster with moderately abundant, flexuous, tapering setae, the longest of which are about half the width of the compound eye. Short, sparse pubescence present over the entire body, but difficult to detect against the densely sculptured integument.

Color: predominantly dark brown, with mandibles, pronotal neck, tibiae, tarsi, and sting testaceous brown.

**Male:** Unknown.

**Etymology:** Morphological, from the Latin ‘acuminatus’ (= pointed, tapering), in reference to the cuneiform petiolar node.

**Comments:** *Temnothorax acuminatus* sp. nov. has been collected from several high elevation mesophyll forest localities in Chiapas state, Mexico. The type series was collected in the same treefall as the holotype of *T. acutispinosus* sp. nov., under epiphytes and in bark crevices. Additional workers have been collected from Winkler leaf litter extraction and terrestrial baiting; it remains unclear whether this species is strictly arboreal. This species is most closely related to *T. tuxtlanus* sp. nov. from the Los Tuxtlas volcano complex in Veracruz state.

***Temnothorax tuxtlanus* sp. nov.**

**Figure 92:**
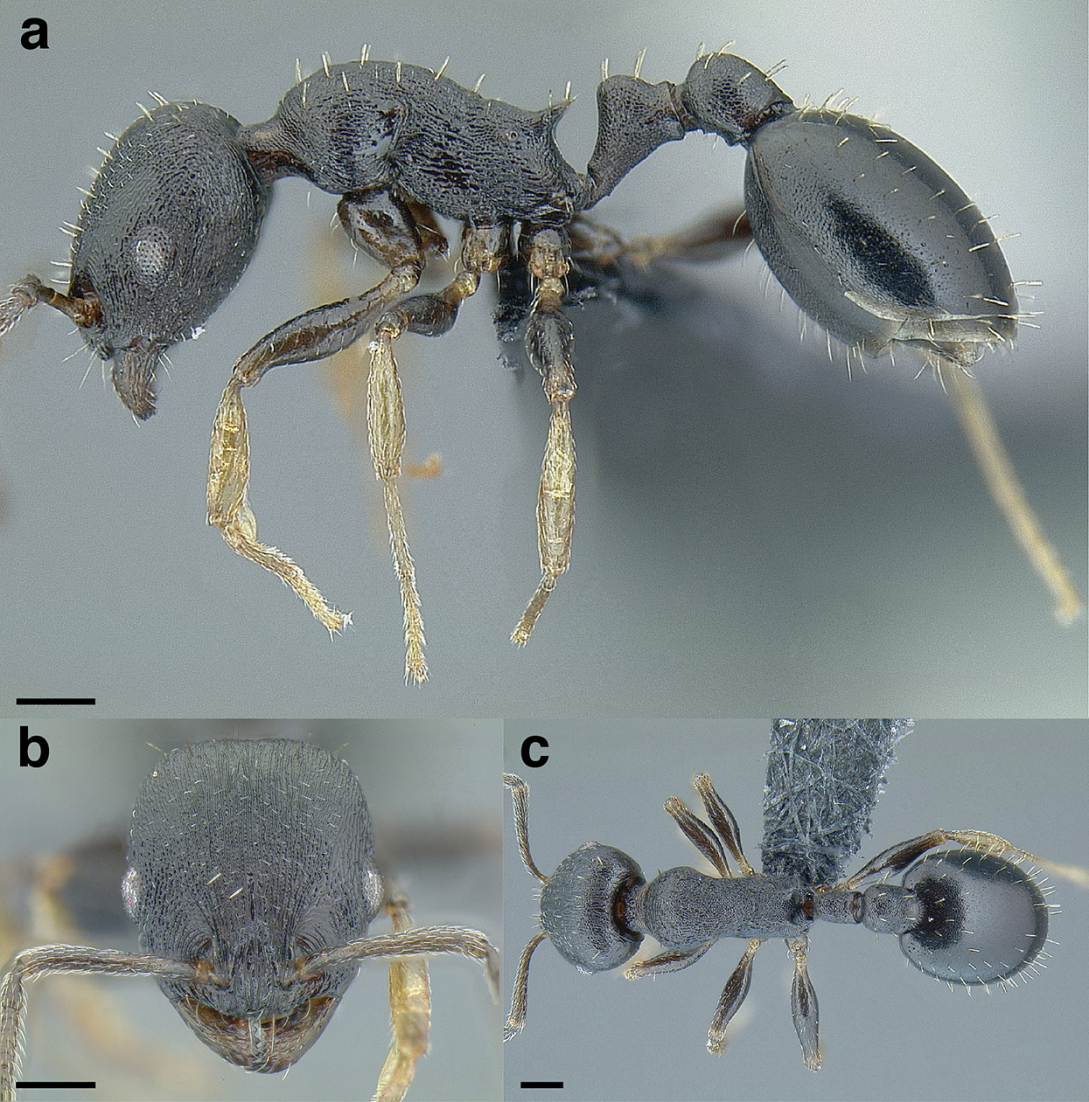
*Temnothorax tuxtlanus* sp. nov. holotype worker (CASENT0640472). (A) Profile view. (B) Full-face view. (C) Dorsal view. Scale bars 0.2 mm.

Distribution: Fig. 90B; worker: Fig. 92.

**Type material examined:** *Holotype worker:* MEXICO: Veracruz: Ruiz Cortinez, 12 km NE San Andrés Tuxtla, 18.53194°N -95.14302°W ± 20 m, 1,140 m, 3 June 2016, ADMAC#Wa-F-02-2-31, montane wet forest, ex sifted leaf litter (CASENT0640472) [CASC].

**Geographic range:** Southern Mexico, mid elevation of the Los Tuxtlas volcano complex (Fig. 90B).

**Worker diagnosis:** *Temnothorax tuxtlanus* sp. nov. can be separated from all other members of the *salvini* clade by the following character combination: dorsum of mesosoma weakly sinuate in profile view; dorsum of propodeum without erect setae; petiole with a weak transverse carina anterodorsally; petiolar peduncle short, comprising about a quarter of the total petiole length; petiolar node rounded dorsally; postpetiole relatively narrow (PWI < 170); setae on head, mesosoma, waist segments and gaster erect, short, sparse and blunt (never long and tapering); integument black, with antennae, mandibles, pronotal neck, coxae, and femora dark brown; trochanters, tibiae, and tarsi testaceous yellow.

**Similar species:** *Temnothorax achii* sp. nov., *, T. acuminatus* sp. nov., *T. acutispinosus* sp. nov., *T. altinodus* sp. nov., *T. ixili, T. subditivus, T. xincai* sp. nov., members of the *augusti* and *pulchellus* groups, and species of the *sallei* clade. *Temnothorax tuxtlanus* sp. nov. can be distinguished from similar appearing members of the *sallei* clade (e.g. *T. manni, T. mexicanus, T. punctithorax*) by the sculpture of the head: in similar looking *sallei* clade species, the head is mostly smooth and shining, with concentric costulae surrounding the antennal insertions; additionally, the medial lobe of the clypeus is typically smooth, with three distinct carinae, whereas it is densely costulate-rugulose in *T. tuxtlanus* sp. nov. In contrast to all of the remaining species above, the petiole of *T. tuxtlanus* sp. nov. has a weak transverse carina anterodorsally, a feature not found in other members of the *salvini* clade, except for *T. acuminatus* sp. nov., *T. altinodus* sp. nov., *T. rugosus*, and the *annexus* group. *Temnothorax tuxtlanus* sp. nov. is easily separated from all of the preceding species, except *T. acuminatus* sp. nov., by the weakly incrassate hind femora (FI < 300) and lack of a medial notch in the anterior margin of the clypeus. *Temnothorax tuxtlanus* sp. nov. can be distinguished from *T. acuminatus* sp. nov. by the dorsally rounded petiolar node, which is cuneiform in *T. acuminatus* sp. nov. Finally, this species may be distinguished from the morphologically similar *T. acutispinosus* sp. nov. by the smaller subpetiolar tooth and more posteriorly directed propodeal spines.

**Worker measurements & indices (*n* = 1):** SL = 0.524; FRS = 0.222; CW = 0.671; CWb = 0.630; PoOC = 0.316; CL = 0.720; EL = 0.178; EW = 0.116; MD = 0.142; WL = 0.869; SPST = 0.185; MPST = 0.268; PEL = 0.329; NOL = 0.190; NOH = 0.118; PEH = 0.224; PPL = 0.229; PW = 0.423; SBPA = 0.199; SPTI = 0.204; PEW = 0.165; PNW = 0.128; PPW = 0.239; HFL = 0.551; HFWmax = 0.132; HFWmin = 0.047; CS = 0.990; ES = 0.236; SI = 83; OI = 24; CI = 88; WLI = 138; SBI = 32; PSI = 21; PWI = 145; PLI = 144; NI = 161; PNWI = 78; NLI = 58; FI = 281.

**Worker description:** In full-face view, head subquadrate, longer than broad (CI 88). Posterior head margin flat, rounding evenly into the lateral margins. Mandibles weakly, finely striate but shining and armed with five teeth: the apical-most well developed and acute, followed by a less developed preapical tooth and three equally developed smaller teeth. Anterior clypeal margin very weakly convex medially. Antennal scapes short: when fully retracted, failing to reach the posterior margin of the head capsule by about the maximum width of the scape (SI 83). Antennae 12-segmented; antennal club of composed of three segments, with the apical-most segment slightly longer than the preceding two in combination. Frontal carinae short, extending past the antennal toruli by about two times the maximum width of the antennal scape. Compound eyes weakly protruding past the lateral margins of the head capsule. Lateral margin of head weakly convex, forming a continuous arc from the mandibular insertions to the posterior margin of the head. Posterior head margin flat, rounding evenly into the lateral margins.

In profile view, compound eyes elongate-ovular and moderately large (OI 24), with 12 ommatidia in longest row. Pronotal declivity indistinct, neck and anterior face of pronotum forming a ~120° angle. Mesosoma weakly sinuate: evenly convex from where it joins the pronotal neck to the metanotal groove; propodeum slightly depressed below the level of the promesonotum, and flat. Promesonotal suture extending from the posterior margin of the procoxal insertion to the mesothoracic spiracle, which is moderately well developed, then continuing dorsally as a very weak sulcus. Metanotal groove visible as a disruption of the sculpture laterally from where it arises between the mid- and hind coxae to the poorly developed metathoracic spiracle, which is nearly indistinguishable against the ground sculpture, then continuing dorsally as a moderately strong impression. Propodeal spiracle weakly developed, directed posterolaterally, and separated from the propodeal declivity by about three spiracle diameters. Propodeal spines short (PSI 21), about half the length of the propodeal declivity, tapering evenly from the base, straight, and acute. Propodeal declivity weakly concave, forming a rounded ~110° angle with the base of the propodeal spines. Propodeal lobes rounded and weakly developed, but dorsal margin weakly angulate. Metapleural gland bulla moderately large, extending from the metacoxal insertion two thirds of the way to the propodeal spiracle. Petiole short (PLI 144), with tubercles anterodorsally. Subpetiolar process in the form of a tiny, blunt, triangular tooth which grades evenly into the ventral margin of the petiole posteriorly; ventral margin of petiole flat posterior to it. Petiolar peduncle short: comprising about a quarter of the total petiole length. Petiolar node erect and dorsally rounded: peduncle grading evenly into the anterior node face; anterior face forming a ~100° angle with the evenly rounded dorsal face; dorsal face rounding evenly into the posterior face; posterior face forms a ~110° angle with the caudal cylinder. Postpetiole evenly rounded anteriorly; dorsal face flat; weakly lobed ventrally, subpostpetiolar process acute.

In dorsal view, humeri developed and distinct: evenly rounded and wider than the rest of the mesosoma; mesothoracic spiracles weakly protruding past the lateral margins of the mesosoma, visible as slight angles where the pronotum meets the mesonotum. Promesonotal groove visible as a weak sulcus and disruption in the ground sculpture. Metanotal groove visible as a distinct sulcus. Propodeal spines broadly approximated basally and weakly diverging apically, their apices separated from each other by about one and a half times their length, the negative space between them “U” shaped. Petiolar peduncle with spiracles weakly protruding past the lateral margins, and slightly constricted anterior to them. Petiolar node, when viewed dorsally, quadrate. Apex of node slightly narrower than the peduncle and caudal cylinder. Postpetiole narrow (PWI 145) and longitudinally elongate. Anterior margin of the postpetiole convex and evenly rounds into the lateral margins; lateral margins parallel to each other; posterior margin flat. Metafemur moderately incrassate (FI 281).

Sculpture: six equally strong clypeal carina present, extending posteriorly to the frontal triangle; clypeus appearing costulate-rugulose. Lateral clypeal lobes with additional, weaker carinae; ground sculpture weakly areolate. Antennal scapes weakly areolate. Cephalic dorsum predominantly areolate, with the areolae arranged into longitudinal rows by fine costulae; very fine concentric costulae surrounding the antennal insertions. Lateral surfaces of head sculptured similarly to the dorsum, but with areolae arranged into concentric whorls by fine costulae above the compound eyes; fine rugulose sculpture overlying the areolae between the compound eye and mandibular insertion. Ventral surface of head smooth and shining, with weak costulae. Pronotal neck areolate. Anterior face of the pronotum weakly areolate-strigulate. Lateral surface of mesosoma predominantly weakly areolate, with fine costulae overlying the ground sculpture. Propodeal declivity areolate, with fine strigulae overlying the ground sculpture. Dorsal surface of mesosoma uniformly areolate; fine strigulae on the anterior margin of the pronotum; fine costulae on the mesonotum; fine areolate-rugulose sculpture on the dorsal face of the propodeum. Femora shining, with weak areolate sculpture on the distal third. Petiole and postpetiole predominantly weakly, areolate-rugulose. First gastral tergite finely, weakly areolate-rugulose on the basal half, otherwise smooth and shining, without spectral iridescence. First gastral sternite densely areolate, without spectral iridescence.

Setae: antennal scapes and funiculi with short, decumbent pilosity. Dorsum of the head, pronotum, waist segments, and gaster with moderately abundant, erect, blunt-tipped setae, the longest of which are about half the width of the compound eye. The head bears ~30, mesosoma ~10, petiole 4, postpetiole ~12, and first gastral tergite ~68 setae. Short, sparse pubescence present over the entire body, but difficult to detect against the densely sculptured integument.

Color: predominantly black, with antennae, mandibles, pronotal neck, coxae, and femora dark brown. Trochanters, tibiae, and tarsi testaceous yellow.

**Gyne:** Unknown.

**Male:** Unknown.

**Etymology:** Geographical, from Los Tuxtlas, an isolated volcanic mountain range near the Gulf of Mexico in Veracruz state, Mexico where the holotype worker was collected.

**Comments:** *Temnothorax tuxtlanus* sp. nov. is known only from the holotype worker, collected via Winkler leaf litter extraction in mid elevation montane wet forest. The closest known relative of *T. tuxtlanus* sp. nov. is *T. acuminatus* sp. nov. from the Sierra Madre de Chiapas in Chiapas state. Together, *T. tuxtlanus* sp. nov. and *T. acuminatus* sp. nov. form the *acuminatus* species group, which is sister to the remainder of the *salvini* clade.

### *acutispinosus* group overview

**Figure 93:**
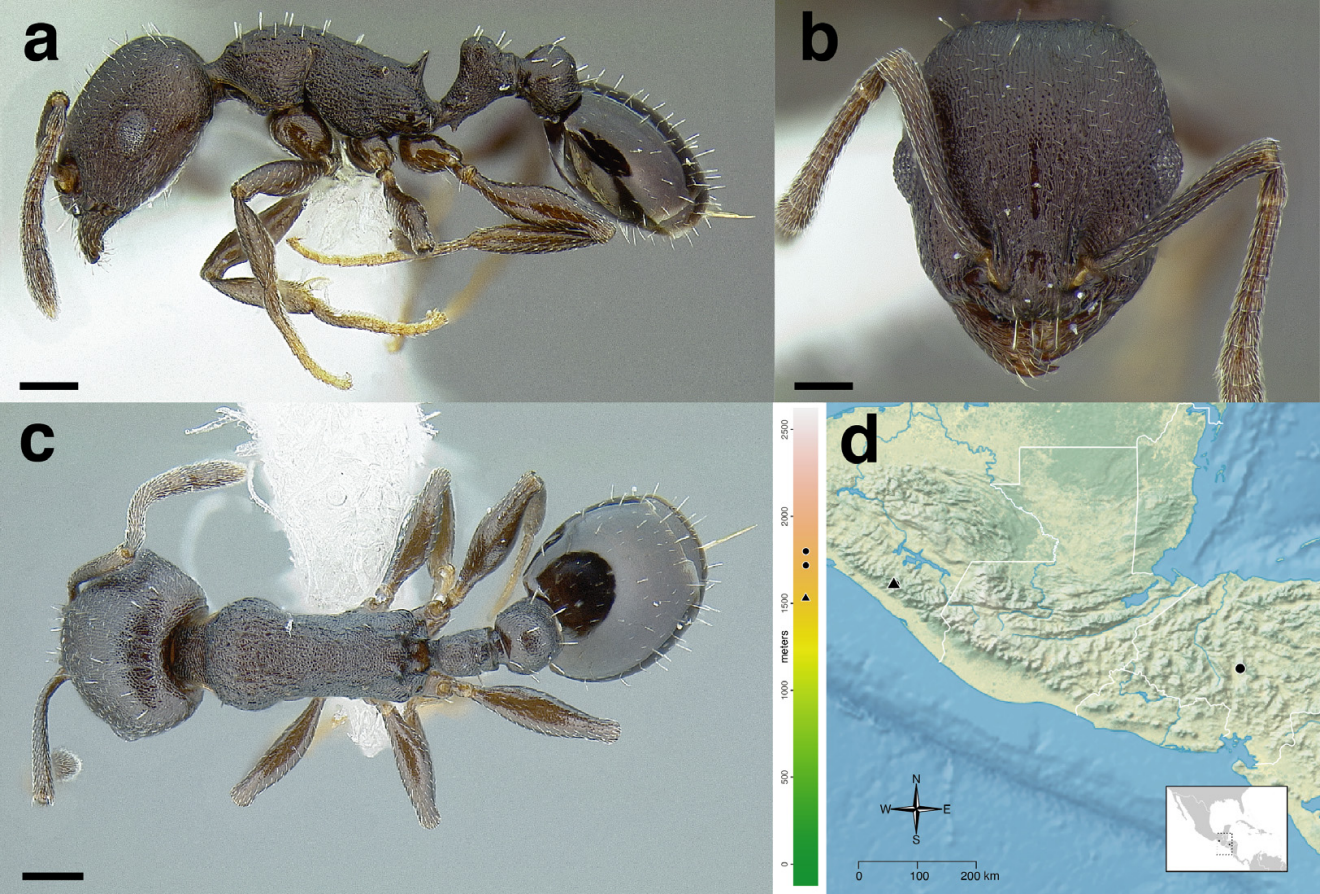
*Temnothorax acutispinosus* sp. nov. holotype worker (JTLC000007447). (A) Profile view. (B) Full-face view. (C) Dorsal view. (D) Geographical and elevational distribution of specimens examined. Type localities are represented by triangles, non-type localities are represented by circles. Bounding box in inset map shows location of main map. Scale bars 0.2 mm.

This group is monotypic, with the nominal *Temnothorax acutispinosus* sp. nov. being the only member. It is morphologically similar to the members of the *acuminatus* group, with which it overlaps geographically (Fig. 93D), but differs from them by the larger subpetiolar tooth and dorsally directed propodeal spines. However, *T. acutispinosus* sp. nov. is apparently more closely related to the members of the *rugosus* and *annexus* groups (Prebus, in prep.).

***Temnothorax acutispinosus* sp. nov.**

Distribution & worker: Fig. 93.

*Temnothorax* mmp06 Prebus, 2017: 7. In phylogeny.

**Type material examined:** *Holotype worker:* MEXICO: Chiapas: Custepec, 15.72196°N 92.95037°W ± 50 m, 1,530 m, 19 May 2008, J. Longino #6285, mesophyll forest, under epiphytes in treefall (JTLC000007447) [CASC].

*Paratype workers:* MEXICO: Chiapas: same data as holotype, 1 worker (JTLC000007448) [MCZC] 1 worker (JTLC000007449) [USNM] 1 worker (JTLC000007450) [ECOSCE] 2 workers (CASENT0869149, CASENT0869156) [LACM] 1 worker (CASENT0869150) [UNAM] 1 worker (CASENT0869151) [UCDC] 1 worker (CASENT0869152) [FSCA] 1 worker (CASENT0869153) [UVGC] 1 worker (CASENT0869154) [AMNH] 1 worker (CASENT0869155) [CASC]; same data as holotype, except: J. Longino #6286, mesophyll forest, under epiphytes in treefall (CASENT0869119) [UNAM].

**Non-type material examined:** MEXICO: Chiapas: 3 km ESE Custepec, 15.72065°N 92.94008°W ± 100 m, 1,800 m, 21 May 2008, D.J. Cox #0056, mesophyll forest, canopy baiting, 1 worker (CASENT0609722) [UCDC].

HONDURAS: Comayagua: 9 km E Comayagua, 14.44412°N 87.55429°W ± 100 m, 1,720 m, 17 May 2010, LLAMA#Go-C-03-2-04, disturbed pine-oak forest, beating vegetation, 1 worker (CASENT0615348) [JTLC].

**Geographic range:** Southern Mexico to Honduras, mid elevations of the Sierra Madre de Chiapas and Central American Nucleus (Fig. 93D).

**Worker diagnosis:** *Temnothorax acutispinosus* sp. nov. can be separated from all other species in the *salvini* clade by the following character combination: dorsum of mesosoma very weakly sinuate in profile view; hind femora weakly incrassate (FI 258–319); propodeal spines acute and directed dorsally; petiolar node rounded dorsally; postpetiole moderately broad (PWI 159–175); integument dark brown; setae on head, mesosoma, waist segments and gaster erect, short, sparse and blunt (never long and tapering).

**Similar species:** *Temnothorax achii* sp. nov., *T. altinodus* sp. nov., *T. ixili, T. subditivus, T. tuxtlanus* sp. nov., *T. xincai* sp. nov., members of the *augusti*, *goniops*, and *pulchellus* groups, and species of the *sallei* clade. *Temnothorax acutispinosus* sp. nov. can be distinguished from similar appearing members of the *sallei* clade (e.g., *T. manni, T. mexicanus, T. punctithorax*) by the petiole, which lacks a transverse carina anterodorsally. In contrast to all of the above species (except *T. altinodus* sp. nov.), *T. acutispinosus* sp. nov. has acute, dorsally directed propodeal spines. *Temnothorax acutispinosus* sp. nov. can be distinguished from *T. altinodus* sp. nov. by the relatively compact mesosoma (WLI 131–136 vs. 148 in *T. altinodus* sp. nov.) and weakly incrassate hind femora, which are enlarged in *T. altinodus* sp. nov.

**Worker measurements & indices (*n* = 3):** SL = 0.533–0.608 (0.567); FRS = 0.201–0.232 (0.217); CW = 0.689–0.770 (0.725); CWb = 0.636–0.725 (0.675); PoOC = 0.287–0.324 (0.302); CL = 0.721–0.816 (0.764); EL = 0.165–0.195 (0.179); EW = 0.122–0.124 (0.123); MD = 0.180–0.210 (0.198); WL = 0.867–0.964 (0.899); SPST = 0.188–0.233 (0.211); MPST = 0.272–0.338 (0.298); PEL = 0.325–0.337 (0.331); NOL = 0.183–0.204 (0.195); NOH = 0.137–0.139 (0.138); PEH = 0.238–0.263 (0.25); PPL = 0.200–0.231 (0.213); PW = 0.402–0.464 (0.431); SBPA = 0.157–0.180 (0.166); SPTI = 0.208–0.210 (0.209); PEW = 0.148–0.170 (0.158); PNW = 0.155–0.163 (0.158); PPW = 0.258–0.271 (0.263); HFL = 0.564–0.652 (0.599); HFWmax = 0.144–0.169 (0.158); HFWmin = 0.050–0.062 (0.055); CS = 0.997–1.133 (1.057); ES = 0.227–0.257 (0.240); SI = 84; OI = 23; CI = 88–89 (88); WLI = 131–136 (133); SBI = 24–25 (25); PSI = 22–27 (24); PWI = 159–175 (166); PLI = 146–163 (156); NI = 134–149 (141); PNWI = 96–105 (100); NLI = 56–61 (59); FI = 258–319 (288).

**Worker description:** In full-face view, head subquadrate, longer than broad (CI 88–89). Mandibles densely, finely striate but shining and armed with five teeth: the apical-most well developed and acute, followed by a less developed preapical tooth and three equally developed smaller teeth. Anterior clypeal margin emarginated medially. Antennal scapes short: when fully retracted, failing to reach the posterior margin of the head capsule by about the maximum width of the scape (SI 84). Antennae 12-segmented; antennal club of composed of three segments, with the apical-most segment slightly longer than the preceding two in combination. Compound eyes moderately protruding past the lateral margins of the head capsule. Frontal carinae short, extending past the antennal toruli by about one and a half times the maximum width of the antennal scape. Lateral margin of head weakly convex, forming a continuous arc from the mandibular insertions to the posterior margin of the head. Posterior head margin flat but rounding evenly into the lateral margins.

In profile view, compound eyes ovular and moderately large (OI 23), with 11 ommatidia in longest row. Pronotal declivity distinct: dorsal margin of anterior face of pronotum marked by a weak carina; neck and anterior face of pronotum forming a ~120° angle. Mesosoma very weakly sinuate: dorsum of promesonotum forming an even convexity between the pronotal declivity and the metanotum, metanotum and propodeum slightly depressed below the level of the promesonotum; metanotum slightly convex in some specimens; propodeal dorsum flat. Promesonotal suture extending from the posterior margin of the procoxal insertion to the mesothoracic spiracle, which is moderately well developed. Metanotal groove visible as a disruption of the sculpture laterally from where it arises between the mid- and hind coxae, continuing dorsally as a weak impression between the posterior margin of the mesonotum and the anterior margin of the metanotum. Propodeal spiracle weakly developed, directed posterolaterally, and separated from the propodeal declivity by about five spiracle diameters. Propodeal spines moderately long, about as long as the propodeal declivity (PSI 22–27), tapering evenly from the base, directed dorsally, and acute. Propodeal declivity with a fine carina joining the base of the propodeal spines and the propodeal lobes; weakly concave, forming a ~120° angle with the base of the propodeal spines. Propodeal lobes rounded and weakly developed. Metapleural gland bulla moderately large, extending from the metacoxal insertion two thirds of the way to the propodeal spiracle. Petiole short (PLI 146–163), with weakly developed tubercles anterodorsally. Subpetiolar process in the form of a moderately large, blunt, triangular tooth; ventral margin of petiole weakly bulging posterior to it. Petiolar peduncle short: comprising about a quarter of the total petiolar length. Petiolar node robust, erect, and rounded: transition between peduncle and node marked by a rounded angle of ~120°, resulting in a weakly concave anterior node face; anterior face rounding evenly into the convex dorsal face; dorsal face rounding evenly into the posterior face, which forms a ~120° angle with the caudal cylinder. Postpetiole evenly rounded anteriorly, strongly bulging anterodorsally, flattened posterodorsally; concave ventrally, with an acute, anteriorly directed subpostpetiolar process.

In dorsal view, humeri moderately well developed and distinct: evenly rounded and wider than the rest of the mesosoma; mesothoracic spiracles very weakly protruding past the lateral margins of the mesosoma, visible as slight angles where the pronotum meets the mesonotum. Promesonotal groove represented by a weak sulcus. Metanotum delineated anteriorly and posteriorly by very faint impressions. Propodeal spines narrowly approximated basally and diverging apically, their apices separated from each other by about their length, the negative space between them “U” shaped. Petiolar peduncle with spiracles weakly protruding past the lateral margins, but not noticeably constricted anterior to them. Petiolar node evenly ovular; node slightly wider than the peduncle, and evenly grading into the caudal cylinder, which the same width as the node. Postpetiole narrow (PWI 159–175) and subquadrate. Anterior margin of the postpetiole weakly convex and meets the lateral margins at a rounded ~90° angle; lateral margins parallel to each other; posterior margin flat. Metafemur moderately to strongly incrassate (FI 258–319).

Sculpture: median clypeal carina present, extending posteriorly nearly to the frontal triangle, and flanked on either side by two equally strong carinae. Lateral clypeal lobes with additional, weaker carinae; ground sculpture areolate. Antennal scapes shining through weak areolate ground sculpture. Cephalic dorsum areolate, with the areolae arranged into longitudinal rows by fine costulae. Lateral surfaces of head areolate, with sculpture similar to the dorsum of the head between the compound eye and the posterior margin of the head; sculpture between the compound eye and the mandibular insertion areolate-rugose. Ventral surface of head smooth and shining, with weak costulae. Pronotal neck areolate. Anterior face of the pronotum areolate. Lateral surface of the mesosoma areolate, with weak costulae overlying the ground sculpture on all surfaces except between the propodeal spiracle and the base of the propodeal spines. Dorsal surface of mesosoma areolate, with overlying rugose sculpture on the pronotum. Femora shining, with traces of weak areolate sculpture. Petiole and postpetiole predominantly areolate, but the anterior face of the postpetiole smooth and shining. First gastral tergite and sternite smooth and shining, with weak spectral iridescence.

Setae: antennal scapes and funiculi with short, adpressed pilosity. Dorsum of the head, pronotum, waist segments, and gaster with moderately abundant, erect, blunt-tipped setae, the longest of which are about the width of the compound eye. The head bears ~28, mesosoma ~16, petiole 6, postpetiole ~14, and first gastral tergite ~44 setae. Short, sparse pubescence present over the entire body, but difficult to detect against the densely sculptured integument.

Color: predominantly dark brown, with antennae, apices of mandibles, and legs testaceous. Sting testaceous yellow.

**Gyne:** Unknown.

**Male:** Unknown.

**Etymology:** Morphological, from the Latin ‘acutus’ (= sharp) + ‘spinosus’ (= thorny), a reference to the sharp, dorsally directed propodeal spines.

**Comments:** *Temnothorax acutispinosus* sp. nov. is known from a few collections in mid elevation mesic forest in Chiapas state in Mexico and Comayagua in Honduras. This species is apparently a canopy dweller, and probably nests arboreally: the types were collected from under epiphytes on a treefall or via canopy baiting. *Temnothorax acutispinosus* sp. nov. is closely related to the *rugosus* and *annexus* species groups from Mexico north of the Isthmus of Tehuantepec and the Southwestern United States, all of which are apparently arboreal.

### *altinodus* group overview

**Figure 94:**
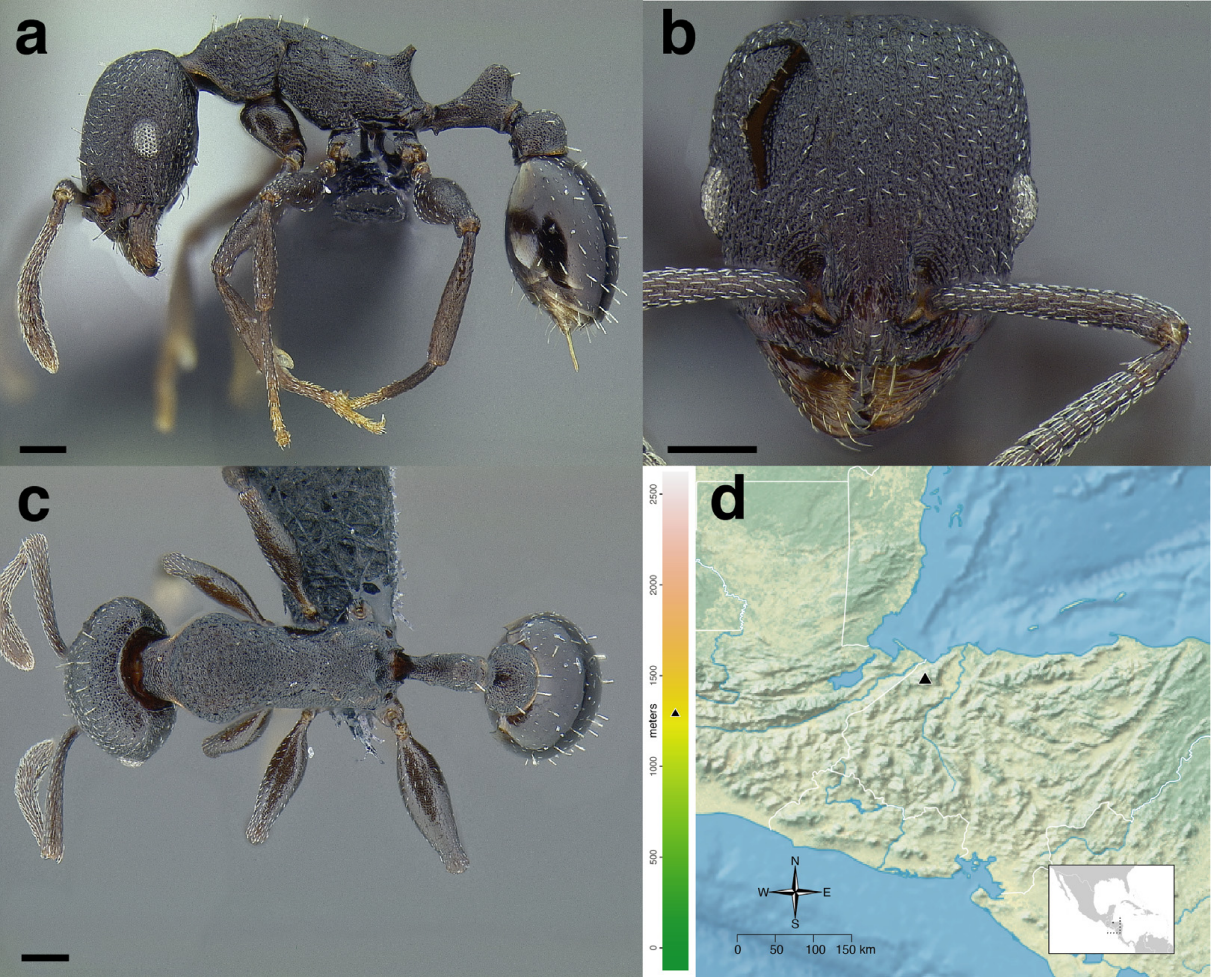
*Temnothorax altinodus* sp. nov. holotype worker (CASENT0617708). (A) Profile view. (B) Full-face view. (C) Dorsal view. (D) Geographical and elevational distribution of specimens examined. Type localities are represented by triangles, non-type localities are represented by circles. Bounding box in inset map shows location of main map. Scale bars 0.2 mm.

This group is monotypic, with the nominal *Temnothorax altinodus* sp. nov. being the only member. It is morphologically similar to the members of the *acuminatus* and *acutispinosus* groups, sharing a lack of setae on the propodeal dorsum. It can be distinguished from these groups by the larger subpetiolar tooth (small in the *acuminatus* group) and the incrassate femora (not incrassate in the *acuminatus* and *acutispinosus* groups). Known from a single collection in the mid elevations of Honduras (Fig. 94D), this species is apparently sister to the *fuscatus* and *pergandei* groups (Prebus, in prep.).

***Temnothorax altinodus* sp. nov.**

Distribution & worker: Fig. 94.

**Type material examined:** *Holotype worker:* HONDURAS: Cortés: Parque Nacional Cusuco, 15.48896°N 88.23439°W ± 70 m, 1,290 m, 1 June 2010, LLAMA#Go-C-06-4-03, mesophyll forest, beating vegetation (CASENT0617708) [CASC].

**Geographic range:** Honduras, mid elevations of the Central American Nucleus (Cortés) (Fig. 94D).

**Worker diagnosis:** *Temnothorax altinodus* sp. nov. can be separated from all other species in the *salvini* clade by the following character combination: antennal scapes short, failing to reach the posterior margin of the head capsule by about the maximum width of the scape (SI 83); mesosoma relatively elongate (WLI 148); dorsum of propodeum without erect setae; propodeal spines directed dorsally; hind femora strongly incrassate (FI 327); petiole with a weak transverse carina anterodorsally; petiolar node compact (NI 143); in dorsal view, apex of petiolar node narrower than caudal cylinder; petiolar node dorsally rounded; postpetiole moderately broad (PWI 202); integument predominantly black; setae on head, mesosoma, waist segments and gaster erect, moderately long, sparse and blunt (never long and tapering).

**Similar species:** *Temnothorax achii* sp. nov., *T. acuminatus* sp. nov., *T. acutispinosus* sp. nov., *T. ixili, T. subditivus, T. tuxtlanus* sp. nov., *T. xincai* sp. nov., members of the *augusti* and *pulchellus* groups, and species of the *sallei* clade. *Temnothorax altinodus* sp. nov. can be distinguished from similar appearing members of the *sallei* clade (e.g. *T. manni, T. mexicanus, T. punctithorax*) by the sculpture of the head: in similar looking *sallei* clade species, the head is mostly smooth and shining, with concentric costulae surrounding the antennal insertions; additionally, the medial lobe of the clypeus is typically smooth, with three distinct carinae, whereas it is densely costulate-rugulose in *T. altinodus* sp. nov. In contrast to all of the remaining species above, the petiole of *T. altinodus* sp. nov. has a weak transverse carina anterodorsally, a feature not found in other members of the *salvini* clade, except for *T. acuminatus* sp. nov., *T. tuxtlanus* sp. nov., *T. rugosus*, and members of the *annexus* group. *Temnothorax altinodus* sp. nov. is easily separated from *T. acutispinosus* sp. nov., *T. acuminatus* sp. nov. and *T. tuxtlanus* sp. nov. by the hind femora, which are incrassate in *T. altinodus* sp. nov.; it may be distinguished from *T. rugosus* and the members of the *annexus* group by the anterior margin of the clypeus, which entire in *T. altinodus* sp. nov., as opposed to medially emarginate.

**Worker measurements & indices (*n* = 1):** SL = 0.607; FRS = 0.223; CW = 0.773; CWb = 0.727; PoOC = 0.343; CL = 0.823; EL = 0.188; EW = 0.131; MD = 0.164; WL = 1.079; SPST = 0.252; MPST = 0.353; PEL = 0.38; NOL = 0.22; NOH = 0.154; PEH = 0.272; PPL = 0.190; PPH = 0.276; PW = 0.499; SBPA = 0.197; SPTI = 0.266; PEW = 0.157; PNW = 0.146; PPW = 0.317; HFL = 0.715; HFWmax = 0.209; HFWmin = 0.064; CS = 1.1385; ES = 0.254; SI = 83; OI = 22; CI = 88; WLI = 148; SBI = 27; PSI = 23; PWI = 202; PLI = 200; NI = 143; PNWI = 93; NLI = 58; FI = 327.

**Worker description:** In full-face view, head subquadrate, longer than broad (CI 88). Mandibles densely striate but shining and armed with five teeth: the apical-most well developed and acute, followed by a less developed preapical tooth and three equally developed smaller teeth. Anterior clypeal margin flat medially. Antennal scapes short: when fully retracted, failing to reach the posterior margin of the head capsule by about the maximum width of the scape (SI 83). Antennae 12-segmented; antennal club of composed of three segments, with the apical-most segment about one and a half times as long as the preceding two in combination. Frontal carinae moderately long, extending past the antennal toruli by about two times the maximum width of the antennal scape. Compound eyes weakly protruding past the lateral margins of the head capsule. Lateral margin of head weakly convex, forming a continuous arc from the mandibular insertions to the posterior margin of the head. Posterior head margin flat but rounding evenly into the lateral margins.

In profile view, compound eyes ovular and small (OI 22), with 11 ommatidia in longest row. Pronotal declivity indistinct, neck and anterior face of pronotum forming a ~120° angle. Mesosoma evenly rounded anteriorly and flat dorsally to the bases of the propodeal spines. Promesonotal suture extending from the posterior margin of the procoxal insertion to the mesothoracic spiracle, which is moderately well developed. Metanotal groove visible as a disruption of the sculpture laterally from where it arises between the mid- and hind coxae to where it ends in the poorly developed metathoracic spiracle, which is nearly indistinguishable against the ground sculpture. Propodeal spiracle well developed, directed posterolaterally, and separated from the propodeal declivity by about three spiracle diameters. Propodeal spines moderately well developed (PSI 23), about as long as the propodeal declivity, tapering evenly from the base, straight, and acute. Propodeal declivity weakly concave, forming a rounded ~120° angle with the base of the propodeal spines. Propodeal lobes rounded and weakly developed. Metapleural gland bulla moderately large, extending from the metacoxal insertion two thirds of the way to the propodeal spiracle. Petiole moderately long (PLI 200), with moderately well-developed tubercles anterodorsally. Subpetiolar process in the form of a small, posteriorly directed, blunt tooth; ventral margin of petiole flat concave posterior to it. Petiolar peduncle short: comprising about a third of the total length of the petiole. Petiolar node erect: transition between peduncle and node marked by a rounded angle of ~140°, resulting in a weakly concave anterior node face; anterior face forming a ~90° angle with the dorsal face, which is weakly convex; dorsal face meeting the posterior face at a ~100° angle; posterior face forming a ~90° angle with the caudal cylinder. Postpetiole evenly rounded anterodorsally, bulging before flattening posterodorsally; lobed ventrally.

In dorsal view, humeri developed and distinct: evenly rounded and wider than the rest of the mesosoma; mesothoracic spiracles weakly protruding past the lateral margins of the mesosoma, visible as slight angles where the pronotum meets the mesonotum. Promesonotal suture visible as a slight disruption in the ground sculpture. Metanotal groove absent: mesonotum and propodeum completely fused and lateral margins converging evenly to the bases of the propodeal spines. Propodeal spines broadly approximated basally and diverging apically, their apices separated from each other by about their length, the negative space between them “U” shaped. Petiolar peduncle with spiracles protruding past the lateral margins; peduncle broadened where they arise. Petiolar node subquadrate; node slightly broader than the peduncle, and evenly grading into the caudal cylinder, which is slightly wider than the node. Postpetiole moderately broad (PWI 202) and campaniform, articulating with the nearly the entire anterior margin of the gaster but leaving small angulate corners exposed. Anterior margin of the postpetiole weakly convex and evenly rounds into the lateral margins, which diverge slightly to the angulate posterior corners; posterior margin flat. Metafemur incrassate (FI 327).

Sculpture: median clypeal carina present, extending posteriorly to the frontal triangle, and flanked on either side by two equally strong carinae. Lateral clypeal lobes with additional, weaker carinae; ground sculpture weakly areolate. Antennal scapes areolate-costulate. Cephalic dorsum densely areolate-rugulose, with fine rugose sculpture overlying it. Lateral surfaces of sculptured similarly to the dorsum, but with areolate-rugulose sculpture becoming stronger between compound eye and mandibular insertion. Ventral surface of head longitudinally areolate-costulate. Pronotal neck weakly areolate. Lateral surfaces of mesosoma densely areolate, with fine rugose-costulate sculpture overlying it. Propodeal declivity weakly areolate. Dorsal surface of mesosoma densely areolate, with fine rugose overlying it. Femora shining through weak areolate sculpture. Petiole uniformly areolate; a weak carina present laterally, extending longitudinally from the anterodorsal flange to the caudal cylinder, passing through the petiolar spiracle. Postpetiole uniformly areolate. First gastral tergite weakly areolate on the basal quarter, otherwise smooth and shining, with weak spectral iridescence. First gastral sternite smooth and shining.

Setae: antennal scapes and funiculi with short, adpressed pilosity. Dorsum of the head, pronotum, waist segments, and gaster with sparse, short, erect, blunt-tipped setae, the longest of which are slightly less than the width of the compound eye. The head bears ~16, mesosoma ~8 restricted to the promesonotum, petiole 2, postpetiole ~4, and first gastral tergite ~20 setae. Short, sparse pubescence present over the entire body, but difficult to detect against the densely sculptured integument.

Color: predominantly black, with dark brown antennae, mandibles, and legs (excluding tarsi). Tarsi and sting testaceous yellow.

**Gyne:** Unknown.

**Male:** Unknown.

**Etymology:** Morphological, from the Latin ‘altus’ (= high, tall) + ‘nodus’ (= node), a reference to the very tall petiolar node.

**Comments:** *Temnothorax altinodus* sp. nov. is known only from the holotype worker, collected via beating vegetation in a mid-elevation mesophyll forest in northwestern Honduras. This species is most closely related to the exclusively arboreally nesting *fuscatus* species group, which ranges from Central Mexico to Costa Rica, and the *pergandei* group, ground nesting species ranging from the northern United States to Nicaragua (Prebus, in prep.).

### *annexus* group overview

**Figure 95:**
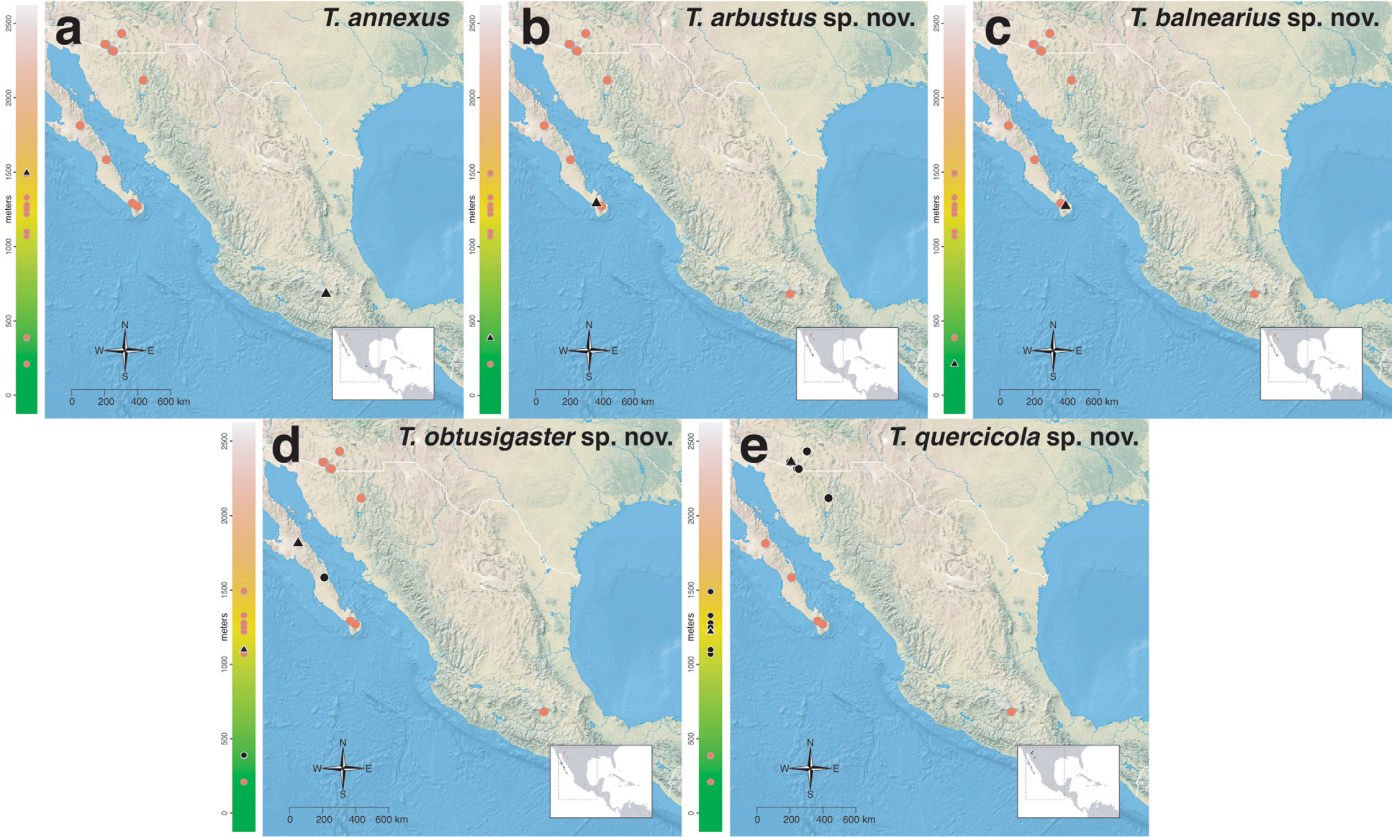
Geographical and elevational distribution of the *annexus* group. (A) *Temnothorax annexus* (B) *T. arbustus* sp. nov. (C) *T. balnearius* sp. nov. (D) *T. obtusigaster* sp. nov. (E) *T. quercicola* sp. nov. Colored scale to the left of each map represents elevation in meters. Points in black represent the species named in each subfigure, while points in red represent other members of the species group. Type localities are represented by triangles, non-type localities are represented by circles. Bounding box in inset map shows location of main map.

Consisting of five species (four of which are newly described here), the *annexus* group is a relatively small one, with a range spanning the low-to-mid elevations of the American southwest to central Mexico (Fig. 95). Although collections for many of these species are scant, all nest collections so far have been from habitats associated with vegetation, either from epiphytes (*Temnothorax annexus*) or from hollow branches and stems on live vegetation (*T. arbustus* sp. nov., *T. balnearius* sp. nov., *T. obtusigaster* sp. nov., and *T. quercicola* sp. nov.). As collections accumulate, this may prove to be the rule. *Temnothorax quercicola* sp. nov. has a long history of being conflated with *T. silvestrii*, to the extent that Creighton (1953) described the male and gyne of *T. quercicola* sp. nov. as *T. silvestrii*. The two species are superficially similar, but *T. silvestrii* belongs to another clade entirely (the *sallei* clade), in yet another example of convergent evolution in *Temnothorax*. See the comments under *T. quercicola* sp. nov. below for a more detailed discussion. These species are large, and typically have an emarginate clypeus, short petiolar peduncle, subquadrate petiolar node, and incrassate femora.

***Temnothorax annexus* (Baroni Urbani, 1978)**

**Figure 96:**
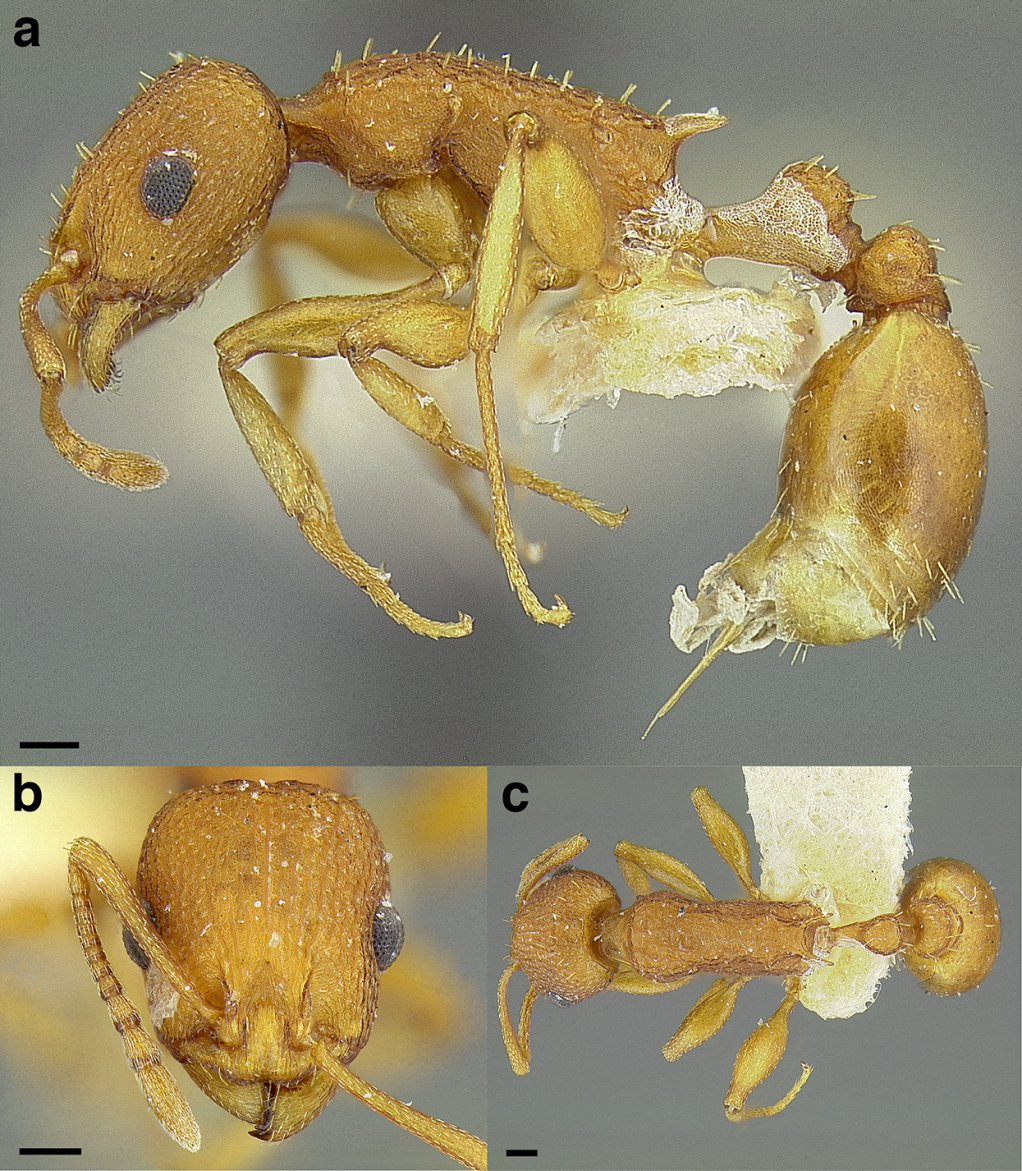
*Temnothorax annexus* lectotype worker (MCZENT00561747). (A) Profile view. (B) Full-face view. (C) Dorsal view. Scale bars 0.2 mm.

Distribution: Fig. 95A; worker: Fig. 96.

*Macromischa annectens* Wheeler, 1931: 11. Syntype workers. Cuernavaca, Mexico. One worker here designated **lectotype**.

*Leptothorax annexus* Baroni Urbani, 1978: 421. Transferred to *Leptothorax*. Nomen novum for *Leptothorax annectens* (Wheeler): 11 (nec *Leptothorax curvispinosus* ssp. *annectens* Wheeler, 1903b: 242).

*Temnothorax annexus* (Baroni Urbani): Bolton, 2003: 271. First combination in *Temnothorax*.

**Type material examined:** *Lectotype worker:* MEXICO: Morelos: Cuernavaca, 26 June 1929, E. Skwarra#Z867/Sk., ex *Tillandsia circinnata*, 1 worker (M.C.Z. co-type 2-3 16359, MCZENT00561747, bottom specimen on pin) [MCZC].

*Paralectotype workers:* same pin as lectotype, 1 worker (top specimen on pin); same data as lectotype, 2 teneral workers (M.C.Z. co-type 4-5 16359, MCZENT00561746) [MCZC].

**Geographic range:** South-Central Mexico, mid elevations of the Southern Sierra Madre (Fig. 95A).

**Worker diagnosis:** *Temnothorax annexus* can be separated from all other species in the *salvini* clade by the following character combination: subdecumbent setae present on gula; metanotal groove absent; propodeum bearing erect setae; propodeum not depressed; propodeal spines short, slightly shorter than the propodeal declivity (PSI 28–29); hind femora strongly incrassate (FI 338–349); petiole with a moderately long peduncle: peduncle comprising about half of the total length of the petiole; petiolar node erect and subquadrate, not overhanging the caudal cylinder; postpetiole narrow (PWI 169–173); first gastral tergite smooth and shining; integument testaceous; dorsum of head, mesosoma, waist segments and gaster with erect, short, blunt-tipped setae; legs without erect setae.

**Similar species:** *Temnothorax andersoni* (Mackay), *T. andrei* (Emery), *T. aureus* sp. nov., *T. aztecoides* sp. nov., *T. aztecus, T. carinatus* (Cole), *T. casanovai* sp. nov., *T. cokendolpheri* (Mackay), *T. goniops, T. nitens* (Emery), *T. paraztecus* sp. nov., *T. punctaticeps* (Mackay), *T. rugosus, T. subditivus* (light form), *T. tenuisculptus*, *T. terrigena, T. wardi* Snelling et al., and species of the *annexus, fuscatus*, and *silvestrii* groups. *Temnothorax annexus* can be separated from *T. andersoni, T. andrei, T. aztecoides* sp. nov., *T. aztecus, T. carinatus, T. cokendolpheri, T. goniops, T. nitens, T. terrigena, T. wardi*, and species of the *silvestrii* group by the strongly incrassate hind femora (FI > 330). Furthermore, it can be distinguished from *T. andersoni, T. andrei, T. aztecoides* sp. nov., *T. aztecus, T. carinatus, T. cokendolpheri, T. goniops, T. nitens, T. terrigena*, and the *silvestrii* group by the anterior clypeal margin, which is emarginate in *T. annexus*. The absence of erect, long, tapering setae on the body will separate *T. annexus* from *T. aztecoides* sp. nov., *T. aztecus*, and *T. paraztecus* sp. nov., while the erect, subquadrate petiolar node differentiates *T. annexus* from the *fuscatus* group, *T. punctaticeps, T. rugosus, T. subditivus*, and nearly all other species listed above. To separate *T. annexus* from other members of the *annexus* group, look for the following character combination: a continuous dorsal margin of the mesosoma in profile (propodeum not depressed as in *T. tenuisculptus*); decumbent setae on the gular region (*T. arbustus* sp. nov., *T. balnearius* sp. nov., and *T. obtusigaster* sp. nov. have erect setae); propodeal spines slightly shorter than the propodeal declivity (PSI ~ 29), pedunculate petiole, with the peduncle comprising about half the total petiole length, and heavily sculptured, predominantly areolate-rugose head and mesosoma will all distinguish *T. annexus* from *T. quercicola* sp. nov.

**Worker measurements & indices (*n* = 3):** SL = 0.759–0.766 (0.762); FRS = 0.261–0.302 (0.284); CW = 0.891–0.917 (0.904); CWb = 0.778–0.826 (0.807); PoOC = 0.350–0.378 (0.367); CL = 0.947–0.993 (0.973); EL = 0.218–0.238 (0.226); EW = 0.163–0.182 (0.173); MD = 0.236–0.262 (0.245); WL = 1.304–1.395 (1.345); SPST = 0.361–0.408 (0.387); MPST = 0.433–0.435 (0.434); PEL = 0.505–0.529 (0.514); NOL = 0.299–0.313 (0.304); NOH = 0.185–0.204 (0.198); PEH = 0.322–0.344 (0.336); PPL = 0.229–0.248 (0.241); PW = 0.572–0.622 (0.590); SBPA = 0.243–0.290 (0.272); SPTI = 0.329–0.340 (0.333); PEW = 0.230–0.234 (0.233); PNW = 0.250–0.273 (0.260); PPW = 0.389–0.405 (0.400); HFL = 0.835–0.850 (0.841); HFWmax = 0.251–0.274 (0.266); HFWmin = 0.072–0.081 (0.078); CS = 1.252–1.323 (1.294); ES = 0.300–0.329 (0.312); SI = 92–98 (94); OI = 23–25 (24); CI = 82–84 (83); WLI = 163–169 (167); SBI = 30–37 (34); PSI = 28–29 (29); PWI = 169–173 (172); PLI = 206–221 (213); NI = 147–162 (154); PNWI = 107–117 (112); NLI = 59; FI = 338–349 (342).

**Worker description:** In full-face view, head subquadrate, longer than broad (CI 82–84). Mandibles densely, finely striate but shining and armed with five teeth: the apical-most well developed and acute, followed by a less developed preapical tooth and three equally developed smaller teeth. Anterior clypeal margin emarginated medially. Antennal scapes moderately long: when fully retracted, just reaching the posterior margin of the head capsule (SI 92–98). Antennae 12-segmented; antennal club of composed of three segments, with the apical-most segment about one and a half times as long as the preceding two in combination. Frontal carinae short, extending past the antennal toruli by about one and a half times the maximum width of the antennal scape. Compound eyes moderately protruding past the lateral margins of the head capsule. Lateral margin of head weakly convex, forming a continuous arc from the mandibular insertions to the posterior margin of the head. Posterior head margin weakly emarginate medially but rounding evenly into the lateral margins.

In profile view, compound eyes ovular and moderately large (OI 23–25), with 13 ommatidia in longest row. Pronotal declivity distinct: dorsal margin of anterior face of pronotum marked by a carina; neck and anterior face of pronotum forming a ~120° angle. Mesosoma very weakly convex from where it joins the pronotal declivity to the propodeal spines, nearly flat. Promesonotal suture extending from the posterior margin of the procoxal insertion only to the mesothoracic spiracle, which is moderately well developed. Metanotal groove visible as a disruption of the sculpture laterally from where it arises between the mid- and hind coxae to where it ends in the poorly developed metathoracic spiracle, which is nearly indistinguishable against the ground sculpture. Propodeal spiracle well developed, directed posterolaterally, and separated from the propodeal declivity by about four spiracle diameters. Propodeal spines moderately well developed and moderately long (PSI 28–29), about as long as the propodeal declivity, flared at the base, downcurved, and blunt. Propodeal declivity weakly concave, forming a rounded ~110° angle with the base of the propodeal spines. Propodeal lobes rounded and weakly developed. Metapleural gland bulla small, extending from the metacoxal insertion halfway to the propodeal spiracle. Petiole moderately long (PLI 206–221), with weakly developed tubercles anterodorsally. Subpetiolar process in the form of a small, blunt tooth; ventral margin of petiole flat posterior to it. Petiolar peduncle moderately long: comprising about half of the total petiole length. Petiolar node robust, erect, and subquadrate: transition between peduncle and node marked by a rounded angle of ~120°; anterior face forming a ~110° angle with the dorsal face, which is weakly convex, nearly flat; dorsal face meeting the posterior face at a ~90° angle, which forms a ~100° angle with the caudal cylinder. Postpetiole flat anteriorly, bulging slightly anterodorsally before flattening posterodorsally; weakly lobed ventrally.

In dorsal view, humeri moderately well developed: evenly rounded and barely wider than the rest of the mesosoma; mesothoracic spiracles weakly protruding past the lateral margins of the mesosoma, visible as slight angles where the pronotum meets the mesonotum. Promesonotal suture represented by a disruption in the ground sculpture. Metanotal groove absent: mesonotum and propodeum completely fused and lateral margins converging evenly to the bases of the propodeal spines. Propodeal spines broadly approximated basally and weakly diverging apically, their apices separated from each other by slightly more than their length, the negative space between them “U” shaped. Petiolar peduncle with spiracles not protruding past the lateral margins. Petiolar node nearly evenly ovular, but posterior margin flattened; node broader than the peduncle, and evenly grading into the caudal cylinder, which is the same width as the node. Postpetiole narrow (PWI 169–173) and subquadrate. Anterior margin of the postpetiole flat and evenly rounds into the lateral margins, which diverge slightly to the rounded posterior corners; posterior margin flat. Metafemur strongly incrassate (FI 338–349).

Sculpture: median clypeal carina present, extending posteriorly to the frontal triangle, and flanked on either side by two equally strong carinae. Lateral clypeal lobes with additional, weaker carinae; ground sculpture shining through weak areolae. Antennal scapes areolate. Cephalic dorsum areolate, with coarse rugae over the ground sculpture; concentric costulae surrounding the antennal insertions; overlying sculpture primarily costate between the frontal carinae. Lateral surfaces of head areolate, with coarse rugae over the ground sculpture. Ventral surface of head smooth and shining anteromedially, but otherwise weakly areolate-costulate. Pronotal neck areolate. Lateral surfaces mesosoma areolate with coarse rugae over the ground sculpture, but with longitudinal rugae stronger than transverse rugae. Propodeal declivity areolate-rugulose. Dorsal surface of mesosoma sculptured similarly to the lateral surface, but stronger longitudinal rugae restricted to the pronotum. Femora finely, densely areolate. Petiole uniformly areolate; a weak carina present laterally, extending longitudinally from the petiolar spiracle to the caudal cylinder; weak rugae on the dorsal and posterior faces of the node. Postpetiole uniformly areolate, with weak rugulose sculpture on the lateral faces. First gastral tergite areolate, but with sculpture becoming weaker posteriorly, without spectral iridescence. First gastral sternite smooth and shining, without spectral iridescence.

Setae: antennal scapes and funiculi with short, adpressed pilosity. Dorsum of the head, pronotum, waist segments, and gaster with moderately abundant, erect, blunt-tipped setae, the longest of which are about half the width of the compound eye. The head bears ~18, mesosoma ~26, petiole 10, postpetiole ~16, and first gastral tergite ~20 setae. Short, sparse pubescence present over the entire body, but difficult to detect against the densely sculptured integument.

Color: predominantly testaceous, with apex of mandibles dark brown.

**Gyne:** Unknown.

**Male:** Unknown.

**Etymology:** In the original description, Wheeler (1931) states that this species, which he placed in *Macromischa*, nonetheless bears many features of the *rottenbergii* group of the Palearctic. Presumably ‘annectens’ (= connected) from Greek refers to the implied link between these taxa.

**Comments:** Known only from the type series, which was collected from *Tillandsia circinnata* epiphytes near Cuernavaca, Morelos, Mexico by Elizabeth Skwarra. *Temnothorax annexus* has close relatives in Baja California, Arizona, and Sonora that nest in dead vegetation, including live branches of oak trees. It is morphologically convergent with, but not particularly closely related to, members of the *rottenbergii* group of the Palearctic, or the *sallei* clade of southern North America, Central America, and the Greater Antilles (Baroni Urbani, 1978; Fontenla Rizo, 2000; Prebus, 2017).

***Temnothorax arbustus* sp. nov.**

**Figure 97:**
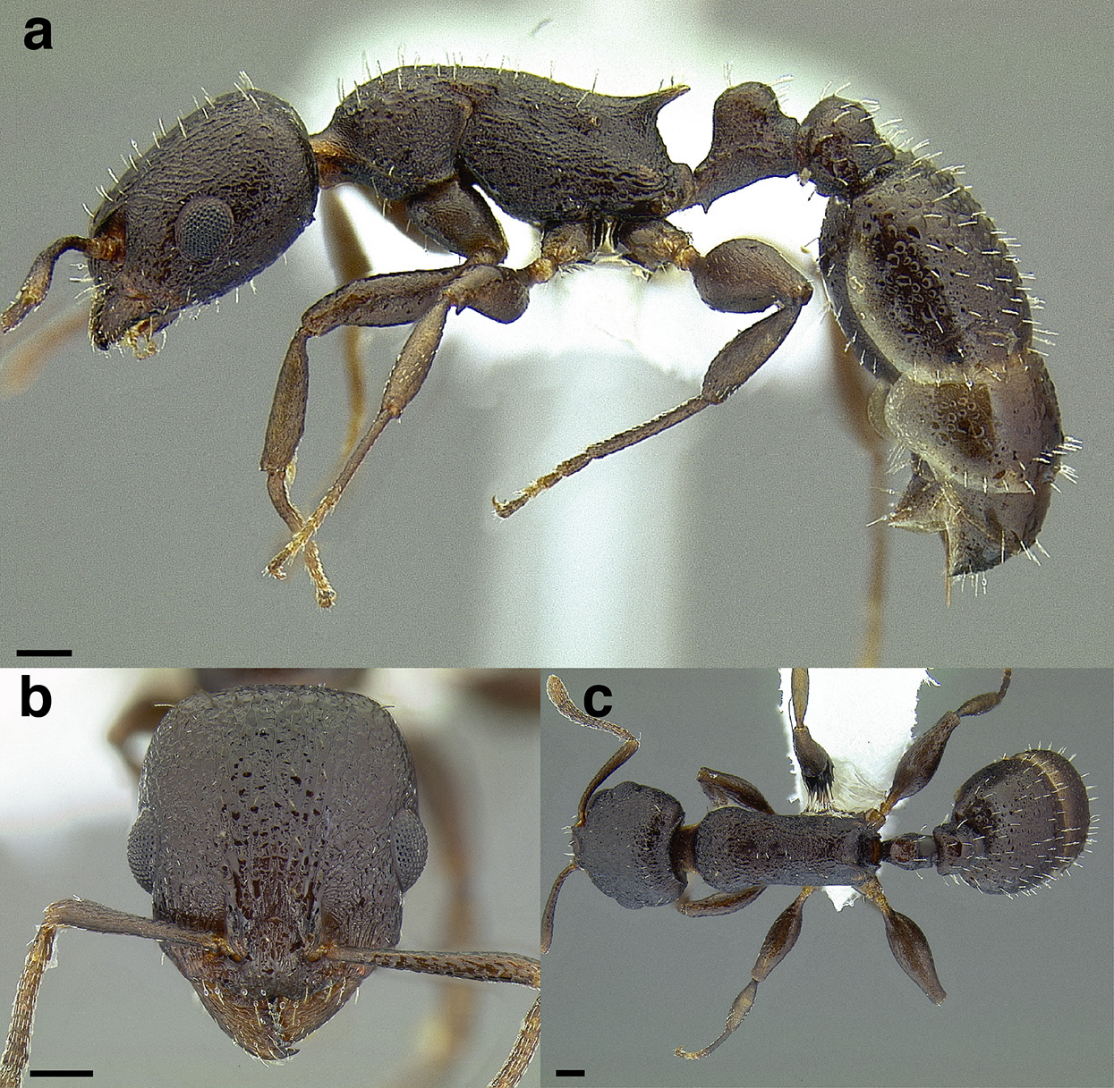
*Temnothorax arbustus* sp. nov. holotype worker (CASENT0758357). (A) Profile view. (B) Full-face view. (C) Dorsal view. Scale bars 0.2 mm.

Distribution: Fig. 95B; worker: Fig. 97.

*Temnothorax* sp. nr. *peninsularis* Prebus, 2017: 7. In phylogeny.

**Type material examined:** *Holotype worker:* MEXICO: Baja California Sur: 14 km NE Hwy 19 turnoff to Presa Santa Inez, 23.590000°N 110.091667°W, 385 m, 21 March 2014, R.A. Johnson#5277, dry forest with *Bursera, Jatropha, Quercus, Pachycereus*, organpipe, 5 cm dead branch *Bursera* (CASENT0758357) [CASC].

*Paratype workers:* MEXICO: Baja California Sur: same data as holotype, 1 worker (CASENT0733967) [UCDC] 2 workers (CASENT0869068) [UNAM]

**Non-type material examined:** MEXICO: Baja California Sur: same data as holotype, 2 workers (CASENT0869069) [RAJC].

**Geographic range:** Low elevations of Baja California Sur (Fig. 95B).

**Worker diagnosis:** *Temnothorax arbustus* sp. nov. can be separated from other species in the *salvini* clade by the following character combination: head relatively broad (CI 87–88); erect setae present on gula; metanotal groove absent; propodeum not depressed; propodeum bearing erect setae; propodeal spines short, slightly shorter than the propodeal declivity (PSI 26–28); hind femora strongly incrassate (FI 379–427); petiole with short peduncle: peduncle comprising about a quarter of the total length of the petiole; petiolar node erect and subquadrate, not overhanging the caudal cylinder in profile view; posterior margin of petiolar node distinct in profile view, about two thirds as long as dorsal margin; postpetiole narrow (PWI 154–163); first gastral tergite smooth and shining; integument dark brown; dorsum of head, mesosoma, waist segments and gaster with erect, short, blunt-tipped setae; legs without erect setae.

**Similar species:** *Temnothorax caguatan* Snelling et al., *T. obturator* (Wheeler), *T. politus, T. rugosus, T. subditivus, T. tenuisculptus, T. whitfordi* (Mackay), and other members of the *annexus* group. *Temnothorax arbustus* sp. nov. can be separated from all of the above species, except for *T. rugosus* and other members of the *annexus* group, by the medially emarginate anterior clypeal margin. Furthermore, it can be distinguished from *T. caguatan* and *T. whitfordi* by the 12-segmented antennae, which are 11-segmented in the latter two. Additionally, the absence of long, tapering setae on the legs and most other surfaces of the body will differentiate *T. arbustus* sp. nov. from *T. politus*. The erect, subquadrate petiolar node of *T. arbustus* sp. nov. contrasts with the node of *T. subditivus*, which is squamiform, and the node of *T. rugosus*, which is cuneiform to subcuneiform. The dorsal margin of the mesosoma is continuous in *T. arbustus* sp. nov. (as opposed to sinuate with a depressed propodeum in *T. tenuisculptus*). Within the *annexus* group, the following characters distinguish *T. arbustus* sp. nov. from its close relatives: gular region with erect setae, as opposed to absent (*T. quercicola* sp. nov.) or present but decumbent (*T. annexus*); head relatively broad (CI 87–88 vs. 80–84 in *T. balnearius*); a petiole with a distinct posterior face in lateral view, which is about two thirds as long as the dorsal face will separate *T. arbustus* sp. nov. from *T. obtusigaster* sp. nov., which has a very short posterior face of the petiole; the first gastral tergite of *T. arbustus* sp. nov. is not sculptured, as opposed to *T. obtusigaster* sp. nov., which has an areolate first gastral tergite.

**Worker measurements & indices (*n* = 2):** SL = 0.683–0.701 (0.692); FRS = 0.306–0.310 (0.308); CW = 0.928–0.969 (0.949); CWb = 0.848–0.895 (0.872); PoOC = 0.394–0.407 (0.401); CL = 0.962–1.028 (0.995); EL = 0.240–0.263 (0.252); EW = 0.196–0.212 (0.204); MD = 0.225–0.227 (0.226); WL = 1.357–1.445 (1.401); SPST = 0.376–0.380 (0.378); MPST = 0.416–0.439 (0.428); PEL = 0.455–0.477 (0.466); NOL = 0.271–0.279 (0.275); NOH = 0.196–0.209 (0.203); PEH = 0.351–0.376 (0.364); PPL = 0.294–0.335 (0.315); PW = 0.622–0.648 (0.635); SBPA = 0.284–0.300 (0.292); SPTI = 0.334–0.353 (0.344); PEW = 0.238–0.242 (0.240); PNW = 0.198–0.210 (0.204); PPW = 0.373–0.388 (0.381); HFL = 0.798–0.839 (0.819); HFWmax = 0.265–0.269 (0.267); HFWmin = 0.062–0.071 (0.067); CS = 1.329–1.409 (1.369); ES = 0.338–0.369 (0.354); SI = 78–81 (79); OI = 25–26 (26); CI = 87–88 (88); WLI = 160–161 (161); SBI = 33–34 (34); PSI = 26–28 (27); PWI = 154–163 (159); PLI = 136–162 (149); NI = 130–142 (136); PNWI = 83–87 (85); NLI = 58–60 (59); FI = 379–427 (403).

**Worker description:** In full-face view, head subquadrate, slightly longer than broad (CI 87-88). Mandibles densely, finely striate but shining and armed with five teeth: the apical-most well developed and acute, followed by a less developed preapical tooth and three equally developed smaller teeth. Anterior clypeal margin emarginated medially. Antennal scapes short: when fully retracted, failing to reach the posterior margin of the head capsule by about the maximum width of the antennal scape (SI 78–81). Antennae 12-segmented; antennal club of composed of three segments, with the apical-most segment about one and a half times as long as the preceding two in combination. Frontal carinae moderately long, extending past the antennal toruli by about two times the maximum width of the antennal scape. Compound eyes strongly protruding past the lateral margins of the head capsule. Lateral margin of head weakly convex, forming a continuous arc from the mandibular insertions to the posterior margin of the head. Posterior head margin weakly concave medially but rounding evenly into the lateral margins.

In profile view, compound eyes ovular and moderately large (OI 25–26), with 15 ommatidia in longest row. Pronotal declivity distinct: dorsal margin of anterior face of pronotum marked by a very weak carina; neck and anterior face of pronotum forming a ~120° angle. Mesosoma very weakly sinuate: weakly convex from where it joins the pronotal declivity to the propodeum, the propodeum slightly depressed and weakly concave. Promesonotal suture extending from the posterior margin of the procoxal insertion to the mesothoracic spiracle, which is moderately well developed. Metanotal groove visible as a disruption of the sculpture laterally from where it arises between the mid- and hind coxae to where it ends in the poorly developed metathoracic spiracle, which is nearly indistinguishable against the ground sculpture. Propodeal spiracle moderately well developed, directed posterolaterally, and separated from the propodeal declivity by about five spiracle diameters. Propodeal spines moderately well developed, but short (PSI 26–28), slightly shorter than the propodeal declivity, slightly flared at the base, straight, and blunt. Propodeal declivity weakly concave, forming a rounded ~100° angle with the base of the propodeal spines. Propodeal lobes rounded and weakly developed, but with an angulate dorsal margin. Metapleural gland bulla small, extending from the metacoxal insertion a third of the way to the propodeal spiracle. Petiole short (PLI 136–162), with tubercles anterodorsally. Subpetiolar process in the form of a moderately long, acute, triangular tooth; ventral margin of petiole weakly concave posterior to it. Petiolar peduncle short: comprising about a quarter of the total petiole length. Petiolar node robust, erect, and subquadrate: transition between peduncle and node marked by a rounded angle of ~110°; anterior face forming a ~100° angle with the dorsal face, which is weakly convex, nearly flat; dorsal face meeting the posterior face at a rounded ~90° angle; posterior face forms a ~110° angle with the caudal cylinder. Postpetiole flat anteriorly, bulging slightly anterodorsally before flattening posterodorsally; weakly lobed ventrally.

In dorsal view, humeri weakly developed: evenly rounded and slightly wider than the rest of the mesosoma; mesothoracic spiracles weakly protruding past the lateral margins of the mesosoma, visible as slight angles where the pronotum meets the mesonotum. Promesonotal suture represented weak sulcus and disruption in the ground sculpture. Metanotal groove represented by a weak sulcus. Propodeal spines broadly approximated basally and weakly diverging apically, their apices separated from each other by slightly more than their length, the negative space between them “U” shaped. Petiolar peduncle with spiracles weakly protruding past the lateral margins. Petiolar node nearly ovular, but posterior margin flattened; node the same width as the peduncle, and evenly grading into the caudal cylinder, which is slightly wider than the node. Postpetiole narrow (PWI 154–163) and subquadrate. Anterior margin of the postpetiole very weakly convex, nearly flat, and meet the lateral margins at a ~90° angle; lateral margins converge parallel to each other; posterior corners rounded; posterior margin flat. Metafemur strongly incrassate (FI 379–427).

Sculpture: median clypeal carina present, extending posteriorly to the frontal triangle, and flanked on either side by two slightly weaker carinae. Lateral clypeal lobes with additional, weaker carinae; ground sculpture weakly areolate. Antennal scapes areolate. Cephalic dorsum weakly areolate, with coarse rugae over the ground sculpture; concentric costulae surrounding the antennal insertions; sculpture between the frontal carinae costate. Lateral surfaces of head areolate, with coarse rugae over the ground sculpture. Ventral surface of head smooth and shining anteromedially, but otherwise weakly areolate-costulate. Pronotal neck areolate. Lateral surfaces mesosoma areolate, with costae over the ground sculpture; region between propodeal spiracle and base of propodeal spines areolate. Propodeal declivity areolate. Dorsal surface of mesosoma predominantly areolate, with rugulae that become very weak on the mesonotum. Femora finely, densely areolate. Petiole uniformly areolate; a weak carina present laterally, extending longitudinally from the petiolar spiracle to the caudal cylinder; areolate-rugulose on the dorsal and posterior faces of the node. Postpetiole uniformly areolate, with rugulose sculpture on the lateral faces. First gastral tergite and sternite smooth and shining, without spectral iridescence.

Setae: antennal scapes and funiculi with short, adpressed pilosity. Dorsum of the head, pronotum, waist segments, and gaster with moderately abundant, erect, blunt-tipped setae, the longest of which are about the width of the compound eye. The head bears ~38, mesosoma ~28, petiole 6, postpetiole ~18, and first gastral tergite ~90 setae. Short, sparse pubescence present over the entire body, but difficult to detect against the densely sculptured integument.

Color: predominantly dark brown, with antennae, mandibles, pronotal neck, legs (excluding femora), basal third of gastral sclerites (excluding the first gastral sclerites), and sting testaceous brown.

**Gyne:** Unknown.

**Male:** Unknown.

**Etymology:** Behavioural, from the Spanish ‘arbusta’ (= shrub), in reference to the dry forest and nesting site in which this species was collected.

**Comments:** Known only from the type specimens, which were extracted from a nest in a small hollow branch of *Bursera* in the subtropical dry forests of Baja California Sur by Bob Johnson. Like other members of the *annexus* group, this species most likely nests exclusively in dead plant material on live plants. *Temnothorax arbustus* sp. nov. was mistakenly reported as *T.* sp. nr. *peninsularis* in Prebus (2017). *Temnothorax peninsularis* (Wheeler) is a member of the distantly related *sallei* clade, which is morphologically convergent with members of the *salvini* clade (Prebus, in prep.). *Temnothorax peninsularis* was described from a dealate gyne but differs from other gynes in the *annexus* group by the absence a medial depression or notch in the anterior margin of the clypeus (Mackay, 2000; Wheeler, 1934, see CASENT0922107 on antweb.org).

***Temnothorax balnearius* sp. nov.**

**Figure 98:**
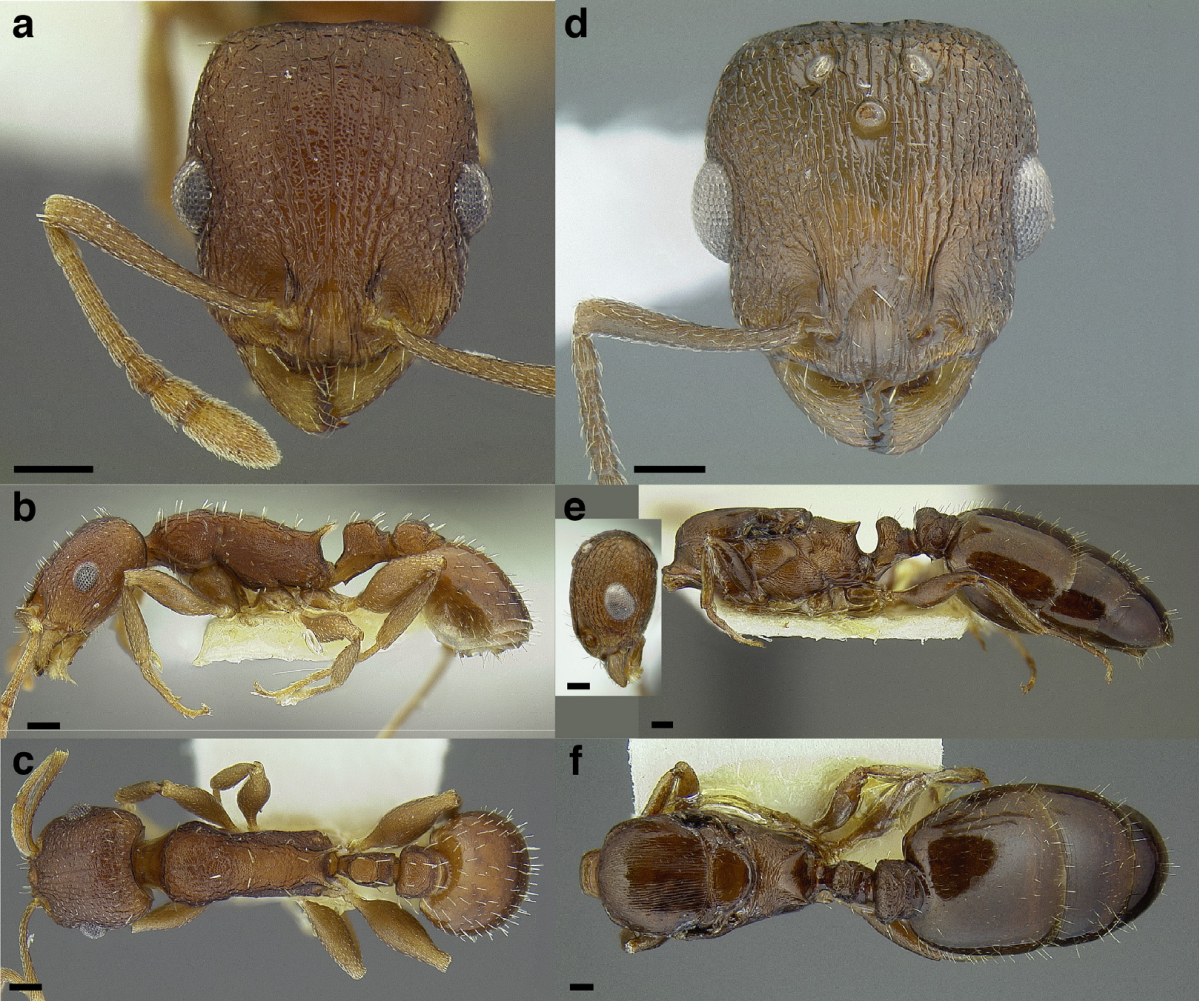
*Temnothorax balnearius* sp. nov. (A–C) Holotype worker (LACMENT323199). (A) Full-face view. (B) Profile view. (C) Dorsal view. (D–F) Paratype gyne (LACMENT323199). (D) Full-face view. (E) Profile view. (F) Dorsal view. Scale bars 0.2 mm.

Distribution: Fig. 95C; worker & gyne: Fig. 98.

**Type material examined:** *Holotype worker:* MEXICO: Baja California Sur: El Charro, 30 October 1969, E. L. Sleeper, ex dead *Agave* stalk (LACMENT323199, top specimen on pin) [LACM].

*Paratype workers and gyne:* same pin as holotype, 1 worker (middle specimen on pin) 1 dealate gyne (bottom specimen on pin); same data as holotype, 3 workers (LACMENT323198) [LACM] 3 workers (CASENT0758290) [LACM].

**Geographic range:** Low elevations of Baja California Sur (Fig. 95C).

**Worker diagnosis:** *Temnothorax balnearius* sp. nov. can be separated from other species in the *salvini* clade by the following character combination: head narrow (CI 80–84); erect setae present on gula; metanotal groove absent; propodeum not depressed; propodeum bearing setae; propodeal spines moderately short, slightly shorter than the propodeal declivity (PSI 24–27); hind femora strongly incrassate (FI 357–452); petiole with short peduncle: peduncle comprising about a quarter of the total length of the petiole; petiolar node erect and subquadrate, not overhanging the caudal cylinder in profile view; postpetiole narrow (PWI 153–180); first gastral tergite smooth and shining; integument medium brown; dorsum of head, mesosoma, waist segments and gaster with erect, short, blunt-tipped setae; legs without erect setae.

**Similar species:** *Temnothorax anaphalantus* Snelling et al., *T. andrei, T. caguatan, T. carinatus, T. cokendolpheri, T. nitens, T. pilicornis* sp. nov., *T. pseudandrei* Snelling et al., *T. rugosus, T. subditivus, T. tenuisculptus, T. wardi*, species of the *silvestrii* group, and other members of the *annexus* group. *Temnothorax balnearius* sp. nov. can be separated from all of the above species, except for *T. anaphalantus, T. rugosus, T. wardi*, and other members of the *annexus* group by the medially emarginate anterior clypeal margin. Furthermore, it can be distinguished from *T. caguatan* by the 12-segmented antennae, which are 11-segmented in the latter. The erect, subquadrate petiolar node of *T. balnearius* sp. nov. contrasts with the node of *T. subditivus*, which is squamiform, and the nodes of *T. nitens, T. rugosus* and *T. wardi*, which are cuneiform to subcuneiform. The dorsal margin of the mesosoma is continuous in *T. balnearius* sp. nov., as opposed to sinuate, with a depressed propodeum in *T. tenuisculptus*. The narrow postpetiole contrasts with the very broad postpetiole of *T. pilicornis* sp. nov. and *T. wardi* (PWI 153–180 vs. > 180). The massively incrassate hind femora will separate *T. balnearius* sp. nov. from all species listed above (except for *T. subditivus, T. tenuisculptus, T. rugosus*, and the *annexus* group), which have less incrassate hind femora (FI 357–452 in *T. balnearius* sp. nov. vs. < 300). Within the *annexus* group, the following characters distinguish *T. balnearius* sp. nov. from its close relatives: gular region with erect setae, as opposed to absent (*T. quercicola* sp. nov.) or present but decumbent (*T. annexus*); head relatively narrow (CI 80–84 vs. > 85 in *T. arbustus* sp. nov. and *T. obtusigaster* sp. nov.).

**Worker measurements & indices (*n* = 8):** SL = 0.625–0.699 (0.668); FRS = 0.241–0.293 (0.269); CW = 0.802–0.897 (0.855); CWb = 0.713–0.807 (0.769); PoOC = 0.334–0.400 (0.363); CL = 0.860–0.996 (0.928); EL = 0.194–0.229 (0.215); EW = 0.149–0.181 (0.166); MD = 0.208–0.246 (0.229); WL = 1.120–1.312 (1.219); SPST = 0.287–0.343 (0.315); MPST = 0.331–0.416 (0.382); PEL = 0.354–0.459 (0.407); NOL = 0.182–0.261 (0.234); NOH = 0.150–0.184 (0.167); PEH = 0.279–0.337 (0.308); PPL = 0.201–0.262 (0.239); PW = 0.508–0.599 (0.550); SBPA = 0.205–0.272 (0.241); SPTI = 0.256–0.316 (0.290); PEW = 0.188–0.226 (0.208); PNW = 0.181–0.244 (0.214); PPW = 0.299–0.380 (0.344); HFL = 0.708–0.805 (0.761); HFWmax = 0.223–0.257 (0.245); HFWmin = 0.054–0.072 (0.063); CS = 1.151–1.296 (1.233); ES = 0.275–0.320 (0.298); SI = 84–91 (87); OI = 23–25 (24); CI = 80–84 (83); WLI = 156–164 (158); SBI = 29–34 (31); PSI = 24–27 (26); PWI = 153–180 (166); PLI = 158–178 (171); NI = 121–154 (140); PNWI = 93–108 (103); NLI = 51–63 (57); FI = 357–452 (393).

**Worker description:** In full-face view, head subquadrate, longer than broad (CI 80–84). Mandibles densely, finely striate but shining and armed with five teeth: the apical-most well developed and acute, followed by a less developed preapical tooth and three equally developed smaller teeth. Anterior clypeal margin emarginated medially. Antennal scapes short: when fully retracted, failing to reach the posterior margin of the head capsule by about one and a half times the maximum width of the antennal scape (SI 84–91). Antennae 12-segmented; antennal club of composed of three segments, with the apical-most segment about one and a half times as long as the preceding two in combination. Frontal carinae short, extending past the antennal toruli by about one and a half times the maximum width of the antennal scape. Compound eyes moderately protruding past the lateral margins of the head capsule. Lateral margin of head weakly convex, forming a continuous arc from the mandibular insertions to the posterior margin of the head. Posterior head margin weakly concave medially but rounding evenly into the lateral marginsIn profile view, compound eyes ovular and moderately large (OI 23–25), with 13 ommatidia in longest row. Pronotal declivity distinct: dorsal margin of anterior face of pronotum marked by a weak carina; neck and anterior face of pronotum forming a ~120° angle. Mesosoma very weakly sinuate: weakly convex from where it joins the pronotal declivity to the propodeum, the propodeum slightly depressed and concave. Promesonotal suture extending from the posterior margin of the procoxal insertion only to the mesothoracic spiracle, which is moderately well developed. Metanotal groove visible as a disruption of the sculpture laterally from where it arises between the mid- and hind coxae to where it ends in the poorly developed metathoracic spiracle, which is nearly indistinguishable against the ground sculpture. Propodeal spiracle moderately well developed, directed posterolaterally, and separated from the propodeal declivity by about five spiracle diameters. Propodeal spines moderately well developed, but short (PSI 24–27), slightly shorter than the propodeal declivity, slightly flared at the base, thin, weakly downcurved, and acute. Propodeal declivity weakly concave, forming a rounded ~110° angle with the base of the propodeal spines. Propodeal lobes rounded and weakly developed. Metapleural gland bulla small, extending from the metacoxal insertion a third of the way to the propodeal spiracle. Petiole moderately long (PLI 158–178), with tubercles anterodorsally. Subpetiolar process in the form of a moderately long, acute, triangular tooth; ventral margin of petiole flat posterior to it. Petiolar peduncle short: comprising about a quarter of the total petiole length. Petiolar node robust, erect, and subquadrate: transition between peduncle and node marked by a rounded angle of ~120°; anterior face forming a ~120° angle with the dorsal face, which is weakly convex, nearly flat; dorsal face meeting the posterior face at a rounded ~90° angle; posterior face forms a ~110° angle with the caudal cylinder. Postpetiole flat anteriorly, bulging slightly anterodorsally before flattening posterodorsally; weakly lobed ventrally.

In dorsal view, humeri moderately well developed: evenly rounded and wider than the rest of the mesosoma; mesothoracic spiracles weakly protruding past the lateral margins of the mesosoma, visible as slight angles where the pronotum meets the mesonotum. Promesonotal suture represented by a weak sulcus. Metanotal groove absent: mesonotum and propodeum completely fused and lateral margins converging evenly to the bases of the propodeal spines. Propodeal spines broadly approximated basally and weakly diverging apically, their apices separated from each other by slightly more than their length, the negative space between them “U” shaped. Petiolar peduncle with spiracles weakly protruding past the lateral margins. Petiolar node nearly ovular, but posterior margin flattened; node broader than the peduncle, and evenly grading into the caudal cylinder, which is the same width as the node. Postpetiole narrow (PWI 153–180) and trapezoidal: slightly wider anteriorly than posteriorly. Anterior margin of the postpetiole flat and evenly rounds into the lateral margins, which converge slightly to the rounded posterior corners; posterior margin flat. Metafemur strongly incrassate (FI 357–452).

Sculpture: median clypeal carina present, extending posteriorly to the frontal triangle, and flanked on either side by two equally strong carinae. Lateral clypeal lobes with additional, weaker carinae; ground sculpture shining through weak areolae. Antennal scapes areolate. Cephalic dorsum areolate-rugulose, with rugae over the ground sculpture; concentric costulae surrounding the antennal insertions; costulate sculpture between the frontal carinae. Lateral surfaces of head areolate-rugulose, with fine rugae over the ground sculpture that become stronger between the compound eye and mandibular insertion. Ventral surface of head smooth and shining anteromedially, but otherwise weakly areolate. Pronotal neck weakly areolate. Lateral surfaces mesosoma areolate-rugulose, with fine costulae over the ground sculpture. Propodeal declivity areolate. Dorsal surface of mesosoma predominantly areolate, with fine costulae on the pronotum and fine strigulae on the propodeum. Femora finely, densely areolate. Petiole uniformly areolate; a weak carina present laterally, extending longitudinally from the petiolar spiracle to the caudal cylinder; weak rugulae on the dorsal and posterior faces of the node. Postpetiole uniformly areolate, with weak rugulose sculpture on the lateral faces. First gastral tergite and sternite smooth and shining, without spectral iridescence.

Setae: antennal scapes and funiculi with short, adpressed pilosity. Dorsum of the head, pronotum, waist segments, and gaster with moderately abundant, erect, blunt-tipped setae, the longest of which are about half the width of the compound eye. The head bears ~38, mesosoma ~30, petiole 6, postpetiole ~14, and first gastral tergite ~68 setae. Short, sparse pubescence present over the entire body, but difficult to detect against the densely sculptured integument.

Color: predominantly medium brown, with antennae, mandibles, clypeus, pronotal neck, and legs light brown.

**Gyne measurements & indices (*n* = 1):** SL = 0.724; FRS = 0.329; CW = 1.049; CWb = 0.950; PoOC = 0.395; CL = 1.037; EL = 0.341; EW = 0.254; MD = 0.212; WL = 1.882; SPST = 0.373; MPST = 0.495; PEL = 0.539; NOL = 0.245; NOH = 0.262; PEH = 0.438; PPL = 0.348; PW = 1.036; SBPA = 0.511; SPTI = 0.471; PEW = 0.307; PNW = 0.260; PPW = 0.548; HFL = 0.906; HFWmax = 0.254; HFWmin = 0.077; CS = 1.469; ES = 0.468; SI = 76; OI = 32; CI = 92; WLI = 198; SBI = 54; PSI = 20; PWI = 179; PLI = 155; NI = 94; PNWI = 85; NLI = 45; FI = 330.

**Gyne description:** In full-face view, head subquadrate, slightly longer than broad (CI 92). Mandibles densely striate but shining and armed with five teeth: the apical-most well developed, followed by a less developed preapical tooth and three equally developed smaller teeth. Anterior clypeal margin emarginated medially. Antennal scapes short: when fully retracted, failing to reach the posterior margin of the head capsule by about one and a half times the maximum width of the antennal scape (SI 76). Antennae 12-segmented; antennal club composed of three segments, with the apical-most segment about one and a half times as long as the preceding two in combination. Frontal carinae moderately long, extending past the antennal toruli by about two and a half times the maximum width of the antennal scape. Compound eyes strongly protruding past the lateral margins of the head capsule. Lateral margins of head convex behind the compound eyes; narrower and forming a converging arc between the compound eyes and the mandibular insertions. Posterior margin of head weakly concave medially, rounding evenly into the lateral margins.

In profile view, compound eyes ovular and large (OI 32), with 16 ommatidia in longest row. Mesoscutum rounded evenly anteriorly, covering the dorsal surface of the pronotum, and weakly convex dorsally, nearly flat. Dorsal plane of mesoscutellum even with the level of the mesoscutum. Posterior margin of metanotum extending past the posterior margin of the mesoscutum. Propodeal spiracle well developed, directed posterolaterally, and separated from the propodeal declivity by about four spiracle diameters. Propodeal spines stout and well developed (PSI 20), about as long as the propodeal declivity, tapering evenly from the base, directed posterodorsally, straight, and blunt. Propodeal declivity concave, forming a rounded ~120° angle with the base of the propodeal spines. Propodeal lobes rounded and weakly developed. Metapleural gland moderately large, extending from the metacoxal insertion halfway to the propodeal spiracle. Petiole short (PLI 155), with weakly developed tubercles anterodorsally. Subpetiolar process in the form of a blunt, triangular tooth, which grades evenly into the ventral margin of the petiole posteriorly. Petiolar peduncle short: comprising about a third of the total petiole length. Petiolar node robust and erect: transition between peduncle and node an evenly rounded ~90° angle, with a very slightly concave anterior node face; anterior face forming a rounded ~90° angle with the dorsal face, which is evenly convex; dorsal face rounding evenly into the posterior face; posterior face forms a ~100° angle with the caudal cylinder. Postpetiole evenly rounded anterodorsally, bulging before it transitions into the flattened posterodorsal face; ventral surface weakly lobed.

In dorsal view, mesoscutum covering pronotum anteriorly, but humeri visible laterally as rounded sclerites. Propodeal spines weakly diverging apically, their apices separated from each other by about two and a half times their length. Petiolar peduncle with spiracles not protruding past the lateral margins, but slightly narrowed anterior to them. Petiolar node subovate, slightly broadened transversely. Petiolar node wider than the peduncle, and evenly grading into the caudal cylinder, which is slightly narrower than the node. Postpetiole narrow (PWI 166), anteroposteriorly compressed, and subtrapezoidal: broader anteriorly than posteriorly. Anterior margin of postpetiole flat, with corners marked by rounded angles as it transitions to the lateral margins; lateral margins bulging anteriorly and converging posteriorly; posterior corners angulate; posterior margin flat. Metafemur strongly incrassate (FI 330).

Sculpture: median clypeal carina present, extending from the anterior margin to frontal triangle, and flanked by four weaker carinae. Lateral clypeal lobes with additional weak carinae; ground sculpture smooth and shining medially, but weakly areolate near the margins of the clypeus. Antennal scapes finely areolate-costulate. Cephalic dorsum with areolate ground sculpture that becomes smooth and shining medially, with rugose sculpture that becomes costate between the frontal carinae; fine concentric costulae surround the antennal insertions. Lateral surfaces of head areolate-rugulose, with rugose sculpture overlying the ground sculpture. Ventral surface of head smooth and shining medially, with weak areolate-rugulose sculpture laterally and posteriorly. Pronotal neck areolate-strigulate. Anterior face of pronotum weakly areolate. Lateral face of pronotum rugose. Anepisternum smooth and shining on the posterior half, otherwise anepisternum and katepisternum longitudinally areolate-costulate. Metapleuron and lateral face of propodeum costulate. Propodeal declivity weakly areolate. Mesoscutum with costulae flanking a medial strip of smooth and shining sculpture; lateral margins smooth and shining. Mesoscutellum smooth and shining, with traces of fine costulae laterally. Metanotum smooth and shining. Dorsum of propodeum strigulate. Femora shining, with traces of weak areolate sculpture. Petiole and postpetiole with finely areolate ground sculpture; petiolar node and postpetiole with coarse rugae overlying the ground sculpture. First gastral tergite shining, but with weak, indistinct sculpture. First gastral sternite smooth and shining.

Setae: antennal scapes and funiculi with short, adpressed pilosity. Dorsum of the head, pronotum, waist segments, and gaster with moderately abundant, erect, blunt-tipped setae, the longest of which are about a third of the width of the compound eye. Short, sparse pubescence present over the entire body, but difficult to detect against the densely sculptured integument.

Color: predominantly medium brown, with antennae, mandibles, clypeus, pronotal neck, and legs light brown.

**Male:** Unknown.

**Etymology:** Geographical, from the Latin ‘balnearius’ (= frequenting baths or bathhouses).

**Comments:** *Temnothorax balnearius* sp. nov.is known only from one collection in the low elevations of Baja California Sur, where it was collected from a dead *Agave* stalk near El Charro hot springs. The vegetation of this region is dominated by xeric scrub and low tropical deciduous forest.

***Temnothorax obtusigaster* sp. nov.**

**Figure 99:**
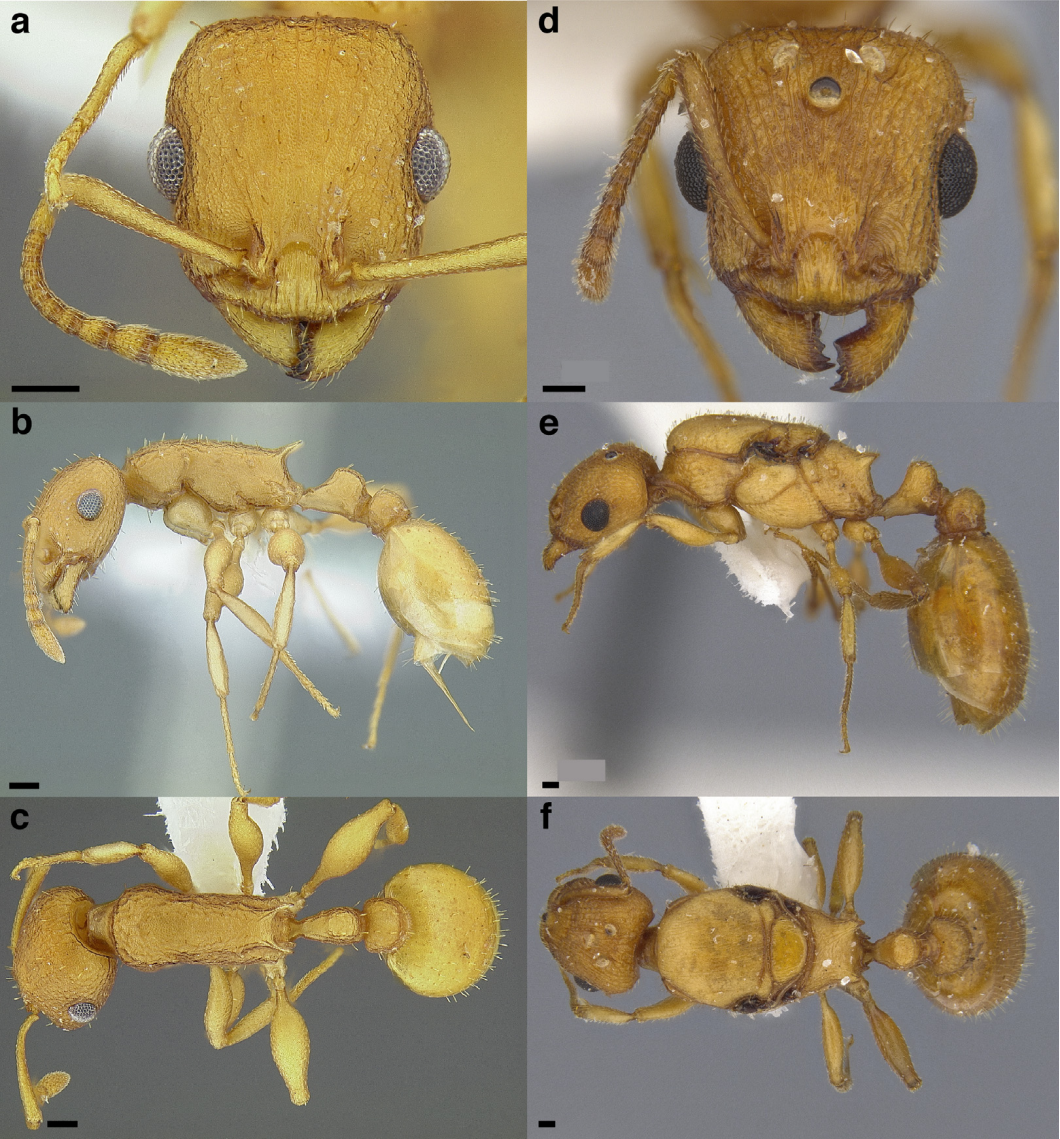
*Temnothorax obtusigaster* sp. nov. (A–C) Holotype worker (CASENT0758654). (A) Full-face view. (B) Profile view. (C) Dorsal view. (D–F) Paratype gyne (CASENT0869104). (D) Full-face view. (E) Profile view. (F) Dorsal view. Scale bars 0.2 mm.

Distribution: Fig. 95D; worker & gyne: Fig. 99.

*Leptothorax* sp. nr. *silvestrii* (in part): Johnson & Ward, 2002: 1023.

**Type material examined:** *Holotype worker:* MEXICO: Baja California Sur: 0.4 km S Sierra San Francisco, 27.600000°N 113.016667°W, 1,100 m, 20 February 1994, R.A. Johnson #BCS 197, 6 m high 3 cm dead branch *Quercus oblongifolia* (CASENT0758654, bottom specimen on pin) [CASC].

*Paratype workers and gyne:* same pin as holotype, 1 worker (CASENT0758654, top specimen on pin) [CASC]; same data as holotype, 1 dealate gyne, 2 workers (CASENT0869104) [CASC] 2 workers (CASENT0104939) [UCDC] 3 workers (MCZENT00561743) [MCZC] 3 workers (CASENT0758322) [MCZC]

**Non-type material examined:** same data as holotype, 100 workers (CASENT0869070-CASENT0869103) [RAJC]. MEXICO: Baja California Sur: 27 km SW Loreto, 9 November 1969, E.M. Fisher & E.L. Sleeper, 1 worker (LACMENT323251) [LACM] 1 worker (LACMENT323250) [LACM].

**Geographic range:** Low to mid elevations of Baja California Sur (Fig. 95D).

**Worker diagnosis:** *Temnothorax obtusigaster* sp. nov. can be separated from other species in the *salvini* clade by the following character combination: head relatively broad (CI 86–90); erect setae present on gula; metanotal groove absent; propodeum not depressed; propodeum bearing setae; propodeal spines short, slightly shorter than the propodeal declivity (PSI 28–29); hind femora strongly incrassate (FI 333–406); petiole with short peduncle: peduncle comprising about a third of the total length of the petiole; petiolar node erect and subquadrate, not overhanging the caudal cylinder in profile view; posterior margin of petiolar node indistinct in profile view, much shorter than the dorsal margin; postpetiole narrow (PWI 160–173); first gastral tergite areolate; integument yellow; dorsum of head, mesosoma, waist segments and gaster with erect, short, blunt-tipped setae; legs without erect setae.

**Similar species:** *Temnothorax anaphalantus, T. andrei, T. caguatan, T. carinatus, T. cokendolpheri, T. nitens, T. pilicornis* sp. nov., *T. pseudandrei, , T. rugosus, T. subditivus, T. tenuisculptus T. wardi*, species of the *silvestrii* group, other members of the *annexus* group. *Temnothorax obtusigaster* sp. nov. can be separated from all of the above species, except for *T. anaphalantus, T. rugosus, T. wardi* and other members of the *annexus* group, by the medially emarginate anterior clypeal margin. Furthermore, it can be distinguished from *T. caguatan* by the 12-segmented antennae, which are 11-segmented in the latter. The erect, subquadrate petiolar node of *T. obtusigaster* sp. nov. contrasts with the node of *T. subditivus*, which is squamiform, and the nodes of *T. rugosus* and *T. wardi*, which are cuneiform to subcuneiform. The dorsal margin of the mesosoma is continuous in *T. obtusigaster* sp. nov. (as opposed to sinuate with a depressed propodeum in *T. tenuisculptus*). The narrow postpetiole contrasts with the very broad postpetiole of *T. pilicornis* sp. nov. and *T. wardi* (PWI 160–173 vs. > 180). The massively incrassate hind femora will separate *T. obtusigaster* sp. nov. from all species listed above (except for *T. subditivus, T. tenuisculptus, T. rugosus*, and the *annexus* group), which have less incrassate hind femora (FI 333–406 in *T. obtusigaster* sp. nov. vs. < 300). Within the *annexus* group, the following characters distinguish *T. obtusigaster* sp. nov. from its close relatives: gular region with erect setae, as opposed to absent (*T. quercicola* sp. nov.) or present but decumbent (*T. annexus*); head relatively broad (CI 86–90 vs. 80–84 in *T. balnearius* sp. nov.); a petiole with an indistinct posterior face in lateral view (much shorter than the dorsal face) will separate *T. obtusigaster* sp. nov. from *T. arbustus* sp. nov., which has a relatively long posterior face of the petiole; the first gastral tergite of *T. obtusigaster* sp. nov. is areolate, as opposed to smooth in *T. arbustus* sp. nov.

**Worker measurements & indices (*n* = 5):** SL = 0.743–0.767 (0.753); FRS = 0.302–0.332 (0.322); CW = 0.950–1.026 (0.990); CWb = 0.863–0.923 (0.898); PoOC = 0.354–0.375 (0.364); CL = 0.991–1.051 (1.024); EL = 0.258–0.300 (0.275); EW = 0.177–0.223 (0.199); MD = 0.233–0.278 (0.256); WL = 1.364–1.433 (1.396); SPST = 0.389–0.410 (0.399); MPST = 0.412–0.457 (0.433); PEL = 0.511–0.526 (0.516); NOL = 0.279–0.314 (0.300); NOH = 0.169–0.205 (0.191); PEH = 0.337–0.390 (0.369); PPL = 0.264–0.313 (0.293); PW = 0.625–0.688 (0.658); SBPA = 0.260–0.310 (0.284); SPTI = 0.322–0.417 (0.375); PEW = 0.237–0.264 (0.249); PNW = 0.250–0.292 (0.276); PPW = 0.407–0.440 (0.417); HFL = 0.840–0.877 (0.858); HFWmax = 0.266–0.293 (0.281); HFWmin = 0.070–0.081 (0.077); CS = 1.359–1.442 (1.41); ES = 0.347–0.405 (0.375); SI = 81–87 (84); OI = 25–28 (27); CI = 86–90 (88); WLI = 150–158 (156); SBI = 30–34 (32); PSI = 28–29 (29); PWI = 160–173 (167); PLI = 164–195 (177); NI = 151–168 (157); PNWI = 105–117 (111); NLI = 54–61 (58); FI = 333–406 (367).

**Worker description:** In full-face view, head subquadrate, longer than broad (CI 86–90). Mandibles densely, finely striate but shining and armed with five teeth: the apical-most well developed and acute, followed by a less developed preapical tooth and three equally developed smaller teeth. Anterior clypeal margin emarginated medially. Antennal scapes moderately long: when fully retracted, just reaching the posterior margin of the head (SI 81–87). Antennae 12-segmented; antennal club of composed of three segments, with the apical-most segment about one and a half times as long as the preceding two in combination. Frontal carinae short, extending past the antennal toruli by about one and a half times the maximum width of the antennal scape. Compound eyes strongly protruding past the lateral margins of the head capsule. Lateral margin of head weakly convex, forming a continuous arc from the mandibular insertions to the posterior margin of the head. Posterior head margin weakly concave medially but rounding evenly into the lateral margins.

In profile view, compound eyes ovular and large (OI 25–28), with 13 ommatidia in longest row. Pronotal declivity distinct: dorsal margin of anterior face of pronotum marked by a carina; neck and anterior face of pronotum forming a ~110° angle. Mesosoma very weakly convex from where it joins the pronotal declivity to the base of the propodeal spines, nearly flat. Promesonotal suture extending from the posterior margin of the procoxal insertion to the mesothoracic spiracle, which is moderately well developed. Metanotal groove visible as a disruption of the sculpture laterally from where it arises between the mid- and hind coxae to where it ends in the poorly developed metathoracic spiracle, which is nearly indistinguishable against the ground sculpture. Propodeal spiracle moderately well developed, directed posterolaterally, and separated from the propodeal declivity by about four spiracle diameters. Propodeal spines moderately well developed, but short (PSI 28–29), slightly shorter than the propodeal declivity, slightly flared at the base, straight, and acute. Propodeal declivity straight, forming a rounded ~100° angle with the base of the propodeal spines. Propodeal lobes rounded and weakly developed, but dorsum slightly angulate. Metapleural gland bulla small, extending from the metacoxal insertion a third of the way to the propodeal spiracle. Petiole moderately long (PLI 164–195), with tubercles anterodorsally. Subpetiolar process in the form of a small, acute, triangular tooth which grades evenly into the ventral petiole margin posteriorly; ventral margin of petiole flat posterior to it. Petiolar peduncle short: comprising about a quarter of the total petiole length. Petiolar node robust, erect, and rounded-subquadrate: transition between peduncle and node marked by a rounded angle of ~120°; anterior face forming a ~100° angle with the dorsal face, which is strongly, evenly convex; dorsal face meeting the posterior face at a rounded ~100° angle; posterior face forms a ~100° angle with the caudal cylinder. Postpetiole convex anteriorly, bulging slightly anterodorsally before flattening posterodorsally; weakly lobed ventrally.

In dorsal view, humeri weakly developed: evenly rounded and slightly wider than the rest of the mesosoma; mesothoracic spiracles weakly protruding past the lateral margins of the mesosoma, visible as slight angles where the pronotum meets the mesonotum. Promesonotal suture represented by a weak sulcus and disruption in the ground sculpture. Metanotal groove represented by a disruption in the ground sculpture. Propodeal spines broadly approximated basally and weakly diverging apically, their apices separated from each other by slightly more than their length, the negative space between them “U” shaped. Petiolar peduncle with spiracles weakly protruding past the lateral margins. Petiolar node subquadrate: anterior margin flat medially, but corners rounding into the lateral margins; lateral margins rounding into the flat posterior margin; node broader than the peduncle, and evenly grading into the caudal cylinder, which is slightly narrower than the node. Postpetiole narrow (PWI 160–173) and subquadrate. Anterior margin of the postpetiole weakly convex, nearly flat, and meets the lateral margins at a rounded ~90° angle; lateral margins parallel to each other; posterior corners rounded; posterior margin flat. Metafemur strongly incrassate (FI 333–406).

Sculpture: median clypeal carina present, extending posteriorly to the frontal triangle, and flanked on either side by three equally strong carinae. Lateral clypeal lobes with additional, weaker carinae; ground sculpture shining through weak areolae. Antennal scapes areolate. Cephalic dorsum areolate-rugulose, with rugae over the ground sculpture; concentric costulae surrounding the antennal insertions; overlying sculpture becoming costate between the frontal carinae. Lateral surfaces of head areolate, with rugae over the ground sculpture. Ventral surface of head smooth and shining anteromedially, but otherwise weakly areolate with weak costulae. Pronotal neck weakly areolate. Lateral surfaces mesosoma areolate, with costulae over the ground sculpture; region between the propodeal spiracle and propodeal spines areolate, without costulae. Propodeal declivity areolate, with strigulae overlying the ground sculpture. Dorsal surface of mesosoma predominantly areolate, with rugae overlying the ground sculpture. Femora finely, densely areolate. Petiole uniformly areolate; a weak carina present laterally, extending longitudinally from the petiolar spiracle to the caudal cylinder; weak rugae on all faces of the node, except for the anterior face. Postpetiole uniformly areolate, with weak rugae on the lateral faces. First gastral tergite finely areolate, with sculpture slightly weakening posteriorly; without spectral iridescence. First gastral sternite weakly areolate, without spectral iridescence.

Setae: antennal scapes and funiculi with short, adpressed pilosity. Dorsum of the head, pronotum, waist segments, and gaster with moderately abundant, erect, blunt-tipped setae, the longest of which are about half the width of the compound eye. The head bears ~40, mesosoma ~26, petiole 8, postpetiole ~16, and first gastral tergite ~80 setae. Short, sparse pubescence present over the entire body, but difficult to detect against the densely sculptured integument.

Color: predominantly yellow, with masticatory margin of the mandibles dark brown.

**Gyne measurements & indices (*n* = 1):** SL = 0.812; FRS = 0.380; CW = 1.142; CWb = 1.032; PoOC = 0.388; CL = 1.091; EL = 0.336; EW = 0.275; MD = 0.251; WL = 2.047; SPST = 0.416; MPST = 0.496; PEL = 0.626; NOL = 0.291; NOH = 0.251; PEH = 0.505; PPL = 0.355; PW = 1.050; SBPA = 0.554; SPTI = 0.527; PEW = 0.298; PNW = 0.353; PPW = 0.558; HFL = 0.976; HFWmax = 0.245; HFWmin = 0.083; CS = 1.578; ES = 0.474; SI = 79; OI = 30; CI = 95; WLI = 198; SBI = 54; PSI = 20; PWI = 187; PLI = 176; NI = 116; PNWI = 118; NLI = 46; FI = 295.

**Gyne description:** In full-face view, head subquadrate, slightly longer than broad (CI 95). Mandibles densely striate but shining and armed with five teeth: the apical-most well developed, followed by a less developed preapical tooth and three equally developed smaller teeth. Anterior clypeal margin emarginated medially. Antennal scapes short: when fully retracted, failing to reach the posterior margin of the head capsule by about the maximum width of the scape (SI 79). Antennae 12-segmented; antennal club composed of three segments, with the apical-most segment about one and a half times as long as the e preceding two in combination. Frontal carinae moderately long, extending past the antennal toruli by about two times the maximum width of the antennal scape. Compound eyes strongly protruding past the lateral margins of the head capsule. Lateral margins of head convex behind the compound eyes, but parallel to each other between the mandibular insertions and the compound eyes. Posterior head margin concave medially, rounding evenly into the lateral margins.

In profile view, compound eyes ovular and large (OI 30), with 18 ommatidia in longest row. Mesoscutum rounded evenly anteriorly, covering the dorsal surface of the pronotum, and flat dorsally. Mesoscutellum on the same plane as the mesoscutum and flat dorsally. Posterior margin of metanotum extending slightly past the posterior margin of the mesoscutum. Propodeal spiracle well developed, directed posterolaterally, and separated from the propodeal declivity by about six spiracle diameters. Propodeal spines short and stout (PSI 20), about half as long as the propodeal declivity, tapering evenly from the base, directed posterodorsally, triangular, and blunt. Propodeal declivity straight and flat, forming a rounded ~100° angle with the base of the propodeal spines. Propodeal lobes rounded, but slightly angulate dorsally. Metapleural gland bulla small, extending from the metacoxal insertion halfway to the propodeal spiracle. Petiole moderately long (PLI 176), with weakly developed tubercles anterodorsally. Subpetiolar process in the form of a small, acute, triangular tooth, which grades evenly into the ventral margin of the petiole posteriorly. Petiolar peduncle short: comprising about a third of the total petiole length. Petiolar node robust and erect, subquadrate: transition between peduncle and node an evenly rounded ~120° angle; anterior face forming a rounded ~90° angle with the dorsal face, which evenly convex; dorsal face meeting the posterior face at a ~100° angle; posterior face forms a ~120° angle with the caudal cylinder. Postpetiole flat anteriorly, bulging slightly anterodorsally before it transitions into the short, flattened posterodorsal face; ventral surface weakly lobed.

In dorsal view, mesoscutum covering pronotum anteriorly, but humeri visible laterally as rounded sclerites. Propodeal spines diverging basally, but parallel apically, their apices separated from each other by about three times their length. Petiolar peduncle with spiracles protruding past the lateral margins, and slightly narrowed anterior to them. Petiolar node transversely ovular, anterior margin slightly broader than posterior margin. Petiolar node tapering apically, slightly wider than the peduncle, and evenly grading into the caudal cylinder, which is about as wide as the node. Postpetiole narrow (PWI 187), anteroposteriorly compressed, and slightly trapezoidal, with anterior broader than posterior. Anterior margin of postpetiole flat, with corners marked by rounded ~80° angles as it transitions to the lateral margins; lateral margins slightly converging; posterior corners rounded; posterior margin flat. Metafemur moderately incrassate (FI 295).

Sculpture: median clypeal carina present but fragmented, extending from the anterior margin nearly to frontal triangle, and flanked by multiple equally strong, fragmented carinae. Lateral clypeal lobes with additional weaker carinae; ground sculpture smooth and shining. Antennal scapes finely areolate-costulate. Cephalic dorsum areolate, with coarsely rugose sculpture overlying the ground sculpture, with costae stronger than transverse rugae; concentric costulae surround the antennal insertions. Lateral surfaces of head areolate, with coarsely rugose sculpture overlying the ground sculpture. Ventral surface of head weakly sculptured medially, with weak rugose sculpture laterally. Pronotal neck and anterior face of pronotum weakly areolate. Lateral surface of the pronotum sculptured similarly to the dorsum of the head, with rugae becoming weaker posteriorly; katepisternum and anepisternum weakly areolate, with weak overlying costulae; lateral surface of propodeum rugose, with sculpture becoming smooth over the metapleural gland bulla. Propodeal declivity weakly areolate-strigulate. Mesoscutum with fine costulae over finely areolate ground sculpture. Mesoscutellum mostly smooth and shining, with faint costulae. Metanotum weakly areolate. Propodeum areolate, with overlying strigulae. Femora areolate. Petiole uniformly areolate; strong rugae on all faces of the node. Postpetiole uniformly areolate, with strong overlying rugae. First gastral tergite and sternite uniformly covered in weak areolate sculpture.

Setae: antennal scapes and funiculi with short, decumbent pilosity. Dorsum of the head, pronotum, waist segments, and gaster with moderately abundant, erect, blunt-tipped setae, the longest of which are about the maximum width of the antennal scape. Short, sparse pubescence present over the entire body, but difficult to detect against the densely sculptured integument.

Color: predominantly yellow, with masticatory margin of the mandibles and wing bases dark brown.

**Male:** Unknown.

**Etymology:** Morphological, from the Latin ‘obtusus’ (= dull, blurred) + ‘gaster’ (= stomach), in reference to the areolate first gastral tergite and sternite.

**Comments:** *Temnothorax obtusigaster* sp. nov. is known from a couple of collections in Baja California Sur, Mexico, both of which were made at low-to-mid elevations in the mountainous interior of the peninsula. Like other species in the *annexus* group, this species appears to nest in vegetation: a nest was collected from *Quercus oblongifolia*.

***Temnothorax quercicola* sp. nov.**

**Figure 100:**
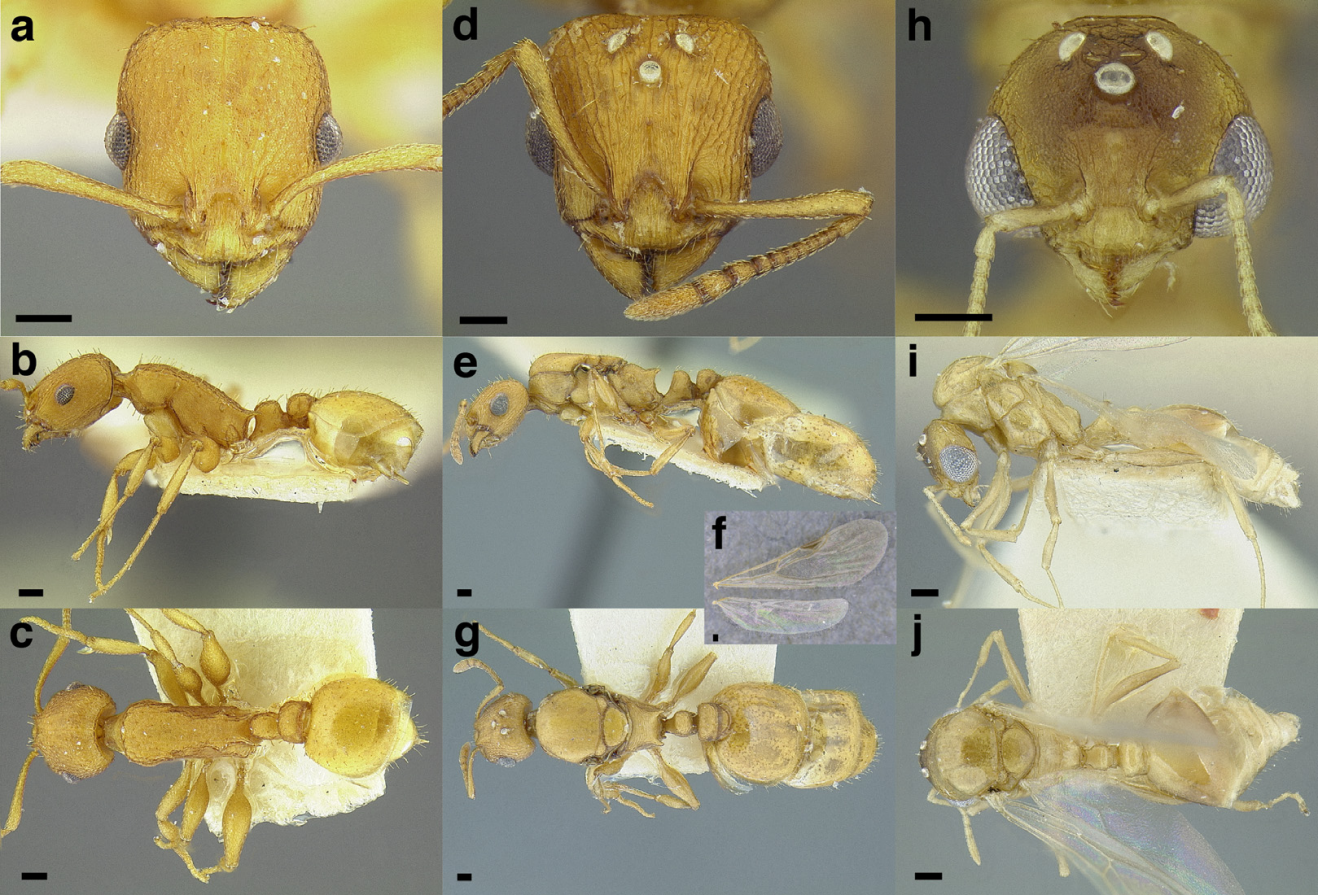
*Temnothorax quercicola* sp. nov. (A–C) Holotype worker (LACMENT323216). (A) Full-face view. (B) Profile view. (C) Dorsal view. (D–F) Paratype gyne (LACMENT323216). (D) Full-face view. (E) Profile view. (F) Dorsal view. (G) Gyne wings (CASENT0869108). (H–J) Paratype male (LACMENT323217). (H) Full-face view. (I) Profile view. (J) Dorsal view. Scale bars 0.2 mm.

Distribution: Fig. 95E; worker, gyne & male: Fig. 100.

*Leptothorax silvestrii* (Santschi, 1911): Creighton, 1953: 2 (misidentification). Worker, gyne, male. Arizona, U.S.A.

*Leptothorax silvestrii* (Santschi): Mackay, 2000: 207 (misidentification).

*Leptothorax silvestrii* (Santschi): Mackay & Mackay, 2002: 122 (misidentification).

*Temnothorax silvestrii* (Santschi): Snelling, Borowiec & Prebus, 2014: 52 (misidentification).

**Type material examined:** *Holotype worker:* U.S.A.: Arizona: Pima County: Baboquivari Mountains, Brown Cañon, 1,220 m, 19 September 1951, W.S. Creighton#245, in *Quercus emoryi* (LACMENT323216, middle specimen on pin) [LACM].

*Paratype workers and gyne:* U.S.A.: Arizona: same pin as holotype, 1 worker, 1 dealate gyne (top and bottom specimens on pin, respectively) [LACM]; same data as previous, 4 workers (CASENT0172599) [LACM] 3 workers (FMNHINS0000115817) [FMNH].

**Non-type material examined:** MEXICO: Sonora: Rancho San Fernando, E side Sierra de la Madera, 29.92833°N 109.4817°W, 1,490 m, 10 April 2010, T. VanDevender, RAJ#4559, rocky mountainside, Mexican blue oak woodland, 3 alate gynes (CASENT0869108) [CASC] 3 workers (CASENT0869108) [CASC].

U.S.A.: Arizona: Pima County: 1,280 m, Baboquivari Mountains, 3 September 1951, W.S. Creighton#546, in *Q. emoryi*, 2 workers, 1 male (LACMENT323217) [LACM]; same data as previous, except: 1 September 1951, W.S. Creighton, in *Q. emoryi* 617, 3 workers (LACMENT323213) [LACM] 3 workers (FMNHINS0000115818) [FMNH]; Baboquivari Mountains, Forestry Cabin, 1,070 m, 23 July 1951, W.S. Creighton#318, in *Q. emoryi*, 3 workers (LACMENT323214) [LACM] 3 workers (MCZENT00561744) [MCZC] 3 workers (FMNHINS0000115815) [FMNH] 1 dealate gyne, 1 worker (FMNHINS0000115823) [FMNH); Santa Catalina Mountains, Molino Cañon, 1,250 m, 5 November 1952, W.S. Creighton, in *Q. emoryi*, 3 workers (LACMENT323215) [LACM] 2 workers (CASENT0869107) [SIBR]; Molino Basin trailhead, 32.33751°N 110.69024°W ± 3 m, 1,330 m, 9 November 2019, M.M. Prebus, MMP#03350, grassland with scattered oaks, ex dead limb of *Quercus emoryi*, 3 workers (CASENT0868466-CASENT0868468) [MMPC]; Santa Cruz County: Ruby, 17 February 1946, N.Y.117144, 5V-582, nest in oak branch, 3 workers (CASENT0105865) [USNM] 3 workers (CASENT0758296) [USNM]; Pajarito Mountains, Yanks Canyon, 2 km E Sycamore Canyon turnoff, 31.433333°N 111.150000°W, 1,220 m, 6 April 1998, R.A. Johnson#AZ1330, oak woodland, 5 cm dead branch *Quercus grisea*, 2 workers (CASENT07583210) [UCDC], 1 dealate gyne, 2 workers (CASENT0869105) [SIBR] 3 workers (CASENT0869106) [SIBR].

**Geographic range:** Mid elevations of southern Arizona, U.S.A. & Sonora, Mexico (Fig. 95E).

**Worker Diagnosis:** *Temnothorax quercicola* sp. nov. can be separated from other species in the *salvini* clade by the following character combination: setae absent on gula; metanotal groove absent; propodeum not depressed; propodeum bearing erect setae; propodeal spines shorter than the propodeal declivity (PSI 22–30); hind femora strongly incrassate (FI 314–388); petiolar peduncle comprising about a third of the total length of the petiole; petiolar node erect and subquadrate, not overhanging the caudal cylinder in profile view; postpetiole narrow (PWI 164–183); first gastral tergite weakly areolate; integument yellow; dorsum of head, mesosoma, waist segments and gaster with erect, short, blunt-tipped setae; legs without erect setae.

**Similar species:** *Temnothorax anaphalantus, T. andrei, T. caguatan, T. carinatus, T. cokendolpheri, T. nitens, T. pilicornis* sp. nov., *T. pseudandrei, T. rugosus, T. subditivus, T. tenuisculptus, T. wardi*, species of the *silvestrii* group, and other members of the *annexus* group. *Temnothorax quercicola* sp. nov. can be separated from all of the above species, except for *T. anaphalantus, T. rugosus, T. wardi* and the other members of the *annexus* group, by the medially emarginate anterior clypeal margin. Furthermore, it can be distinguished from *T. caguatan* by the 12-segmented antennae, which are 11-segmented in the latter. The erect, subquadrate petiolar node of *T. quercicola* sp. nov. contrasts with the node of *T. subditivus*, which is squamiform, and the nodes of *T. nitens, T. rugosus* and *T. wardi*, which are cuneiform to subcuneiform. The dorsal margin of the mesosoma is continuous in *T. quercicola* sp. nov., as opposed to sinuate, with a depressed propodeum in *T. tenuisculptus*. The antennal scapes with subdecumbent setae contrast with those of *T. pilicornis* sp. nov., which has suberect setae. The massively incrassate hind femora will separate *T. quercicola* sp. nov. from all species listed above (except for *T. subditivus, T. tenuisculptus, T. rugosus*, and other members of the *annexus* group), which have less incrassate hind femora (FI 314–388 in *T. quercicola* sp. nov. vs. < 300). Within the *annexus* group, the following characters distinguish *T. quercicola* sp. nov. from its close relatives: gula without erect setae, as opposed to present but decumbent (*T. annexus*) or present and erect (*T. arbustus* sp. nov., *T. balnearius* sp. nov., and *T. obtusigaster* sp. nov.); petiolar peduncle short, comprising about a third of the total petiole length, vs. comprising about half in *T. annexus*; propodeal spines shorter than the propodeal declivity, vs. about as long as the propodeal declivity in *T. annexus*.

**Worker measurements & indices (*n* = 8):** SL = 0.631–0.696 (0.665); FRS = 0.255–0.281 (0.268); CW = 0.786–0.870 (0.837); CWb = 0.713–0.786 (0.747); PoOC = 0.301–0.346 (0.323); CL = 0.854–0.916 (0.883); EL = 0.186–0.244 (0.212); EW = 0.138–0.168 (0.155); MD = 0.191–0.239 (0.214); WL = 1.101–1.219 (1.161); SPST = 0.250–0.345 (0.295); MPST = 0.334–0.420 (0.373); PEL = 0.347–0.450 (0.395); NOL = 0.206–0.251 (0.224); NOH = 0.161–0.181 (0.172); PEH = 0.298–0.348 (0.324); PPL = 0.211–0.258 (0.241); PW = 0.508–0.569 (0.542); SBPA = 0.205–0.247 (0.226); SPTI = 0.252–0.305 (0.273); PEW = 0.195–0.22 (0.209); PNW = 0.193–0.229 (0.212); PPW = 0.338–0.381 (0.358); HFL = 0.671–0.771 (0.731); HFWmax = 0.220–0.248 (0.232); HFWmin = 0.059–0.071 (0.067); CS = 1.145–1.242 (1.189); ES = 0.255–0.327 (0.290); SI = 84–92 (89); OI = 22–26 (24); CI = 83–86 (85); WLI = 151–158 (155); SBI = 29–32 (30); PSI = 22–30 (25); PWI = 164–183 (172); PLI = 139–185 (165); NI = 116–144 (130); PNWI = 95–166 (109); NLI = 51–63 (57); FI = 314–388 (349).

**Worker description:** In full-face view, head subquadrate, longer than broad (CI 83–86). Mandibles densely, finely striate but shining and armed with five teeth: the apical-most well developed and acute, followed by a less developed preapical tooth and three equally developed smaller teeth. Anterior clypeal margin emarginated medially. Antennal scapes short: when fully retracted, failing to reach the posterior margin of the head capsule by about the maximum width of the antennal scape (SI 84–92). Antennae 12-segmented; antennal club of composed of three segments, with the apical-most segment about one and a half times as long as the preceding two in combination. Frontal carinae short, extending past the antennal toruli by about one and a half times the maximum width of the antennal scape. Compound eyes strongly protruding past the lateral margins of the head capsule. Lateral margin of head weakly convex, forming a continuous arc from the mandibular insertions to the posterior margin of the head. Posterior head margin weakly concave medially but rounding evenly into the lateral margins.

In profile view, compound eyes ovular and moderately large (OI 22–26), with 13 ommatidia in longest row. Pronotal declivity distinct: dorsal margin of anterior face of pronotum marked by a carina; neck and anterior face of pronotum forming a ~120° angle. Mesosoma very weakly convex from where it joins the pronotal declivity to the base of the propodeal spines. Promesonotal suture extending from the posterior margin of the procoxal insertion to the mesothoracic spiracle, which is moderately well developed. Metanotal groove visible as a disruption of the sculpture laterally from where it arises between the mid- and hind coxae to where it ends in the poorly developed metathoracic spiracle, which is nearly indistinguishable against the ground sculpture. Propodeal spiracle moderately well developed, directed posterolaterally, and separated from the propodeal declivity by about five spiracle diameters. Propodeal spines short (PSI 22–30), shorter than the propodeal declivity, slightly flared at the base, thin, straight, and acute. Propodeal declivity straight, forming a rounded ~100° angle with the base of the propodeal spines. Propodeal lobes rounded and weakly developed, but dorsum slightly angulate. Metapleural gland bulla small, extending from the metacoxal insertion halfway to the propodeal spiracle. Petiole moderately long (PLI 139–185), with tubercles anterodorsally. Subpetiolar process in the form of a small, blunt, triangular tooth which grades evenly into the ventral petiole margin posteriorly; ventral margin of petiole weakly concave posterior to it. Petiolar peduncle short: comprising about a third of the total petiole length. Petiolar node robust, erect, and subquadrate: transition between peduncle and node marked by a rounded angle of ~120°; anterior face forming a ~110° angle with the dorsal face, which is weakly convex, nearly flat; dorsal face meeting the posterior face at a rounded ~90° angle; posterior face forms a ~100° angle with the caudal cylinder. Postpetiole convex anteriorly, bulging slightly anterodorsally before flattening posterodorsally; weakly lobed ventrally.

In dorsal view, humeri weakly developed: evenly rounded and barely wider than the rest of the mesosoma; mesothoracic spiracles weakly protruding past the lateral margins of the mesosoma, visible as slight angles where the pronotum meets the mesonotum. Promesonotal suture represented by a weak sulcus. Metanotal groove absent: mesonotum and propodeum completely fused and lateral margins converging evenly to the bases of the propodeal spines. Propodeal spines broadly approximated basally and weakly diverging apically, their apices separated from each other by slightly more than their length, the negative space between them “U” shaped. Petiolar peduncle with spiracles weakly protruding past the lateral margins. Petiolar node nearly ovular, but posterior margin flattened; node broader than the peduncle, and evenly grading into the caudal cylinder, which is slightly narrower than the node. Postpetiole narrow (PWI 164–183) and subquadrate. Anterior margin of the postpetiole weakly convex, nearly flat, and meets the lateral margins at a ~90° angle; lateral margins parallel to each other; posterior corners rounded; posterior margin flat. Metafemur incrassate (FI 314–388).

Sculpture: median clypeal carina present, extending posteriorly to the frontal triangle, and flanked on either side by two weaker carinae. Lateral clypeal lobes with additional, weaker carinae; ground sculpture shining through weak areolae. Antennal scapes areolate. Cephalic dorsum areolate, with fine rugae over the ground sculpture; concentric costulae surrounding the antennal insertions; overlying sculpture becoming costate between the frontal carinae. Lateral surfaces of head areolate, with rugae over the ground sculpture. Ventral surface of head smooth and shining anteromedially, but otherwise weakly areolate with weak costulae. Pronotal neck weakly areolate. Lateral surfaces mesosoma areolate, with costulae over the ground sculpture; region between the propodeal spiracle and propodeal spines areolate, without costulae. Propodeal declivity areolate. Dorsal surface of mesosoma predominantly areolate, with fine costulae on the pronotum and rugae on the rest of the mesosoma. Femora finely, densely areolate. Petiole uniformly areolate; a weak carina present laterally, extending longitudinally from the petiolar spiracle to the caudal cylinder; weak rugae on all faces of the node. Postpetiole uniformly areolate, with rugulose sculpture on the lateral faces. First gastral tergite weakly, finely areolate, with sculpture weakening posteriorly; without spectral iridescence. First gastral sternite smooth and shining, without spectral iridescence.

Setae: antennal scapes and funiculi with short, adpressed pilosity. Dorsum of the head, pronotum, waist segments, and gaster with moderately abundant, erect, blunt-tipped setae, the longest of which are about the width of the compound eye. The head bears ~38, mesosoma ~38, petiole 10, postpetiole ~18, and first gastral tergite ~80 setae. Short, sparse pubescence present over the entire body, but difficult to detect against the densely sculptured integument.

Color: predominantly yellow, with masticatory margin of the mandibles dark brown.

**Gyne measurements & indices (*n* = 1):** SL = 0.783; FRS = 0.386; CW = 1.127; CWb = 1.022; PoOC = 0.366; CL = 1.086; EL = 0.354; EW = 0.297; MD = 0.220; WL = 2.001; SPST = 0.440; MPST = 0.495; PEL = 0.567; NOL = 0.293; NOH = 0.254; PEH = 0.538; PPL = 0.333; PW = 1.154; SBPA = 0.496; SPTI = 0.503; PEW = 0.312; PNW = 0.356; PPW = 0.591; HFL = 0.942; HFWmax = 0.282; HFWmin = 0.100; CS = 1.565; ES = 0.503; SI = 77; OI = 32; CI = 94; WLI = 196; SBI = 49; PSI = 22; PWI = 189; PLI = 170; NI = 115; PNWI = 114; NLI = 52; FI = 282.

**Gyne description:** In full-face view, head subquadrate, slightly longer than broad (CI 94). Mandibles densely striate but shining and armed with five teeth: the apical-most well developed, followed by a less developed preapical tooth and three equally developed smaller teeth. Anterior clypeal margin emarginated medially. Antennal scapes short: when fully retracted, failing to reach the posterior margin of the head capsule by about the maximum width of the scape (SI 77). Antennae 12-segmented; antennal club composed of three segments, with the apical-most segment about one and a half times as long as the preceding two in combination. Frontal carinae moderately long, extending past the antennal toruli by about two times the maximum width of the antennal scape. Compound eyes strongly protruding past the lateral margins of the head capsule. Lateral margins of head convex behind the compound eyes, but parallel to each other between the mandibular insertions and the compound eyes. Posterior head margin concave medially, rounding evenly into the lateral margins.

In profile view, compound eyes ovular and large (OI 32), with 22 ommatidia in longest row. Mesoscutum rounded evenly anteriorly, covering the dorsal surface of the pronotum, and flat dorsally. Mesoscutellum on the same plane as the mesoscutum and flat dorsally. Posterior margin of metanotum extending slightly past the posterior margin of the mesoscutum. Propodeal spiracle well developed, directed posterolaterally, and separated from the propodeal declivity by about four spiracle diameters. Propodeal spines short and stout (PSI 22), about half as long as the propodeal declivity, tapering evenly from the base, directed posterodorsally, triangular, and blunt. Propodeal declivity straight and flat, forming a rounded ~100° angle with the base of the propodeal spines. Propodeal lobes rounded, but slightly angulate dorsally. Metapleural gland bulla moderately large, extending from the metacoxal insertion two thirds of the way to the propodeal spiracle. Petiole moderately long (PLI 170), with weakly developed tubercles anterodorsally. Subpetiolar process in the form of a small, blunt, triangular tooth, which grades evenly into the ventral margin of the petiole posteriorly. Petiolar peduncle short: comprising about a third of the total petiole length. Petiolar node robust and erect, subquadrate: transition between peduncle and node an evenly rounded ~120° angle; anterior face forming a rounded ~90° angle with the dorsal face, which weakly convex; dorsal face meeting the posterior face at a ~110° angle; posterior face forms a ~120° angle with the caudal cylinder. Postpetiole flat anteriorly, bulging slightly anterodorsally before it transitions into the flattened posterodorsal face; ventral surface weakly lobed.

In dorsal view, mesoscutum covering pronotum anteriorly, but humeri visible laterally as rounded sclerites. Propodeal spines weakly diverging apically, their apices separated from each other by about three times their length. Petiolar peduncle with spiracles not protruding past the lateral margins, but slightly narrowed anterior to them. Petiolar node subquadrate, anterior margin slightly broader than posterior margin. Petiolar node tapering apically, slightly wider than the peduncle, and evenly grading into the caudal cylinder, which is narrower than the node. Postpetiole narrow (PWI 189), anteroposteriorly compressed, and subquadrate. Anterior margin of postpetiole flat, with corners marked by rounded ~90° angles as it transitions to the lateral margins; lateral margins parallel to each other; posterior corners rounded; posterior margin flat. Metafemur moderately incrassate (FI 282).

Sculpture: median clypeal carina present, extending from the anterior margin nearly to frontal triangle, and flanked by multiple weaker, indistinct carinae. Lateral clypeal lobes with additional weaker carinae; ground sculpture smooth and shining. Antennal scapes finely areolate-costulate. Cephalic dorsum areolate, with rugose sculpture overlying the ground sculpture; concentric costulae surround the antennal insertions. Lateral surfaces of head areolate, with costate sculpture overlying the ground sculpture; sculpture becoming rugose between the compound eye and mandibular insertion. Ventral surface of head smooth and shining medially, with weak areolate-rugulose sculpture laterally and posteriorly. Pronotal neck areolate. Anterior face of pronotum weakly areolate. Lateral surfaces of the mesosoma sculptured similarly to the dorsum of the head; anterior half of anepisternum areolate, without overlying rugose sculpture. Propodeal declivity weakly areolate- strigulate. Mesoscutum with costulate sculpture over finely areolate ground sculpture; a longitudinal strip of very weakly areolate sculpture present medially. Mesoscutellum smooth and shining, with weak costulae on the lateral margins. Metanotum finely areolate. Propodeum areolate, with fine strigulae. Femora shining through weak areolate sculpture. Petiole uniformly areolate; a weak carina present laterally, extending longitudinally from the petiolar spiracle to the caudal cylinder; weak reticulate rugae on all faces of the node. Postpetiole uniformly areolate, with weak rugose sculpture on the lateral faces. First gastral tergite and sternite predominantly smooth and shining, with weak spectral iridescence; base of first gastral tergite with traces of weak areolate sculpture.

Setae: antennal scapes and funiculi with short, adpressed pilosity. Dorsum of the head, pronotum, waist segments, and gaster with moderately abundant, erect, blunt-tipped setae, the longest of which are about a third of the width of the compound eye. Short, sparse pubescence present over the entire body, but difficult to detect against the densely sculptured integument.

Color: predominantly yellow, with masticatory margin of the mandibles and wing bases dark brown.

**Male measurements & indices (*n* = 1):** SL = 0.272; FRS = 0.176; CW = 0.792; CWb = 0.677; PoOC = 0.242; CL = 0.619; EL = 0.341; EW = 0.295; MD = 0.043; WL = 1.134; SPST = n/a; MPST = 0.352; PEL = 0.282; NOL = 0.174; NOH = 0.076; PEH = 0.224; PPL = 0.196; PW = 0.762; SBPA = n/a; SPTI = n/a; PEW = 0.180; PNW = 0.212; PPW = 0.325; HFL = 0.774; HFWmax = 0.137; HFWmin = 0.063; CS = 0.987; ES = 0.489; SI = 40; OI = 50; CI = 109; WLI = 168; SBI = n/a; PSI = n/a; PWI = 181; PLI = 144; NI = 229; PNWI = 118; NLI = 62; FI = 217.

**Male description:** In full-face view, head subovate, slightly broader than long (CI 109). Mandibles very weakly striate, shining, and armed with five teeth: the apical-most well developed, followed by a smaller preapical tooth and three roughly equally developed smaller teeth. Anterior clypeal margin entire and weakly convex. Antennal scapes very short: when fully retracted, failing to reach the posterior margin of the head capsule by about five times the maximum width of the antennal scape (SI 39). Antennae 13-segmented; antennal club composed of four segments, with the apical-most segment longer than the preceding two in combination. Frontal carinae moderately long, extending past the antennal toruli by about two and a half the maximum width of the antennal scape. Compound eyes strongly protruding past the lateral margins of the head capsule. Lateral margin of head convex behind the compound eyes; margin between the mandibular insertions and the anterior margin of the compound eye straight. Posterior head margin flat medially, rounding evenly into the lateral margins.

In profile view, compound eyes ovular and large (OI 50), with 24 ommatidia in the longest row. Mesoscutum bulging anteriorly, covering the dorsal surface of the pronotum, convex dorsally. Mesoscutellum on the same plane as the mesoscutum, convex dorsally. Posterior margin of metanotum extending beyond the posterior margin of the mesoscutellum. Propodeum weakly depressed, convex dorsally, and separated from the base of the propodeal angles by a deep sulcus. Propodeal spiracle moderately well developed, directed posterolaterally, and separated from the propodeal declivity by about four spiracle diameters. Propodeal spines absent but represented by blunt angles. Propodeal lobes rounded and weakly developed. Metapleural gland bulla very small, extending a quarter of the way between the insertion of the metacoxa and the propodeal spiracle. Petiole short (PLI 144), with weak tubercles anterodorsally. Subpetiolar process absent; ventral margin of petiole bulging slightly. Petiolar peduncle short: comprising about a third of the total petiole length. Petiolar node low and dorsally truncated, nearly cuneiform; the convergence of the anterior and dorsal faces marked by a ~110° angle; dorsal face very short, meeting the posterior face at a ~100° angle. Postpetiole flat anteriorly, rounded and slightly bulging anterodorsally, flattened posterodorsally, and concave ventrally.

In dorsal view, mesoscutum covering pronotum. Petiolar peduncle with spiracles slightly protruding past the lateral margins; transversely constricted anterior to them. Petiolar node slightly wider than the peduncle and the caudal cylinder; subquadrate when viewed dorsally. Postpetiole narrow (PWI 181) and subquadrate. Anterior margin of postpetiole weakly convex, with the anterior corners evenly rounding into the lateral margins; lateral margins parallel to each other; posterior margin of postpetiole flat. Metafemur not incrassate (FI 217).

Sculpture: median clypeal lobe with multiple indistinct carinae. Antennal scapes shining, with traces of weak areolate sculpture. Dorsum of head areolate, with weak, fine costulae between the frontal carinae. Lateral surface of head areolate, with weak, fine costulae posterior to the compound eye. Ventral surface of head weakly areolate, with weak, fine costulae. Pronotal neck very weakly areolate. Anterior face of pronotum weakly areolate. Lateral surface of pronotum predominantly areolate, with an anterior patch of smooth and shining sculpture. Katepisternum smooth and shining. Anepisternum predominantly smooth and shining, with weak areolate sculpture on the posterior half. Metapleuron and lateral face of propodeum areolate. Propodeal declivity weakly, finely transversely rugose. Dorsally, mesoscutum weakly areolate along the Mayrian furrows. Mesoscutellum predominantly smooth and shining, with traces of weak areolate sculpture laterally. Femora smooth and shining, with traces weak areolate sculpture. Petiole weakly areolate laterally, but smooth and shining dorsally. Dorsal surface of postpetiole shining, with traces of weak areolate sculpture laterally and on the posterior quarter. First gastral tergite and sternite smooth and shining, without spectral iridescence.

Setae: antennal scapes and funiculi with short, adpressed pilosity. Dorsum of the head, pronotum, waist segments, and gaster with moderately abundant, erect, blunt-tipped setae, the longest of which are about a quarter of the width of the compound eye. Short, sparse pubescence present over the entire body, but difficult to detect against the densely sculptured integument.

Color: predominantly yellow, with masticatory margin of the mandibles and interior of the ocellar triangle testaceous brown.

**Etymology:** Behavioural, from the Latin ‘quercus’ (= oak tree) + ‘colus’ (= dweller) in reference to the well-established nesting preference of this species.

**Comments:** *Temnothorax quercicola* sp. nov. is known from multiple collections at mid elevations in the sky islands of southern Arizona and Sonora. Allred (1982) reports *T. silvestrii*, which this species is often confused with, from Box Elder, Utah; I have not personally observed this collection, so I have excluded it from the range. This species appears to preferentially nest within hollow cavities of larger limbs of *Quercus emoryi* or *Q. grisea*, eschewing small twigs (Creighton, 1953). Colonies are monogynous and small, with 50–70 workers (Creighton, 1953). *Temnothorax quercicola* sp. nov. has a long history of being misidentified as *T. silvestrii*, a co-occurring, morphologically convergent member of the distantly related *sallei* clade. Interestingly, in an article detailing the ‘rediscovery’ of *T. silvestrii* (which was in fact *T. quercicola* sp. nov.), Creighton (1953) noted that specimens were sent to Heinrich Kutter at the NHMB to compare with types of *T. silvestrii* (see CASENT0913000). Kutter detailed a number of differences between the specimens of *T. quercicola* sp. nov. and the type, which he thought justified separating them from *T. silvestrii* (correctly, as it turns out). Unfortunately, Creighton dismissed these morphological disparities and stated that the type specimen fell within the range of observed variation among nests. *Temnothorax quercicola* sp. nov. most obviously differs from *T. silvestrii* by the incrassate femora, but the notched anterior margin of the clypeus and the blockier head shape are equally telling characters.

### *augusti* group overview

**Figure 101:**
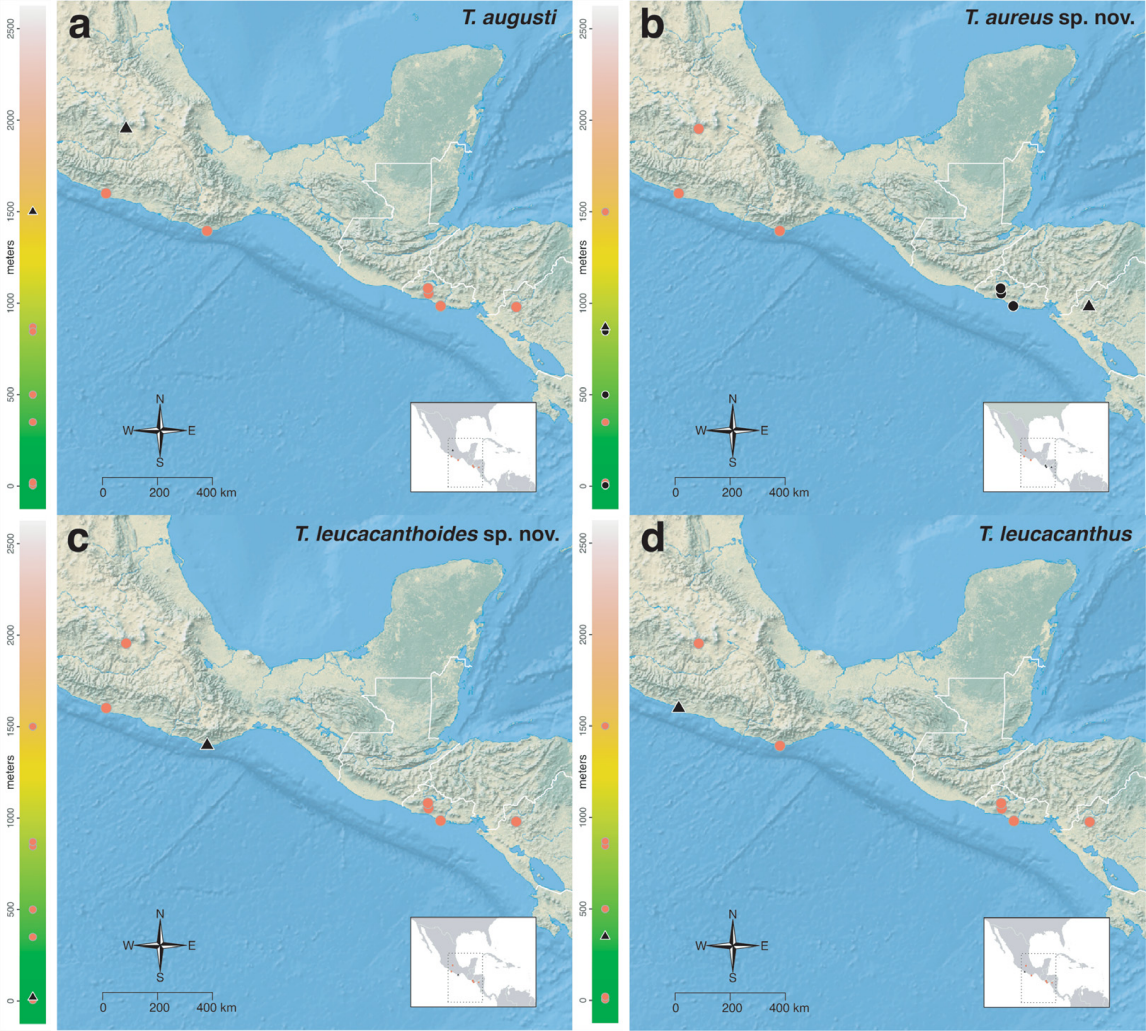
Geographical and elevational distribution of the *augusti* group. (A) *Temnothorax augusti* (B) *T. aureus* sp. nov. (C) *T. leucacanthoides* sp. nov. (D) *T. leucacanthus*. Colored scale to the left of each map represents elevation in meters. Points in black represent the species named in each subfigure, while points in red represent other members of the species group. Type localities are represented by triangles, non-type localities are represented by circles. Bounding box in inset map shows location of main map.

With four species (two described as new here), the *augusti* group is another small group with a range spanning the low-to-mid elevations of central Mexico to Central America (Fig. 101). Although little is known about the biology of the members of this group, the nominal *Temnothorax augusti* was collected from epiphytes, and *T. leucacanthoides* sp. nov. was collected from a dead stalk of Asteraceae; nesting in vegetation may prove to be the rule for this group as more collections are made. Members of this group may be confused with *T. casanovai* sp. nov. or *T. tenuisculptus*. They may be separated from these by the petiolar node that overhangs the caudal cylinder (perpendicular in *T. tenuisculptus*) and the faintly areolate first gastral tergite (strongly areolate in *T. casanovai* sp. nov.).

### *Temnothorax augusti* (Baroni Urbani, 1978)

**Figure 102:**
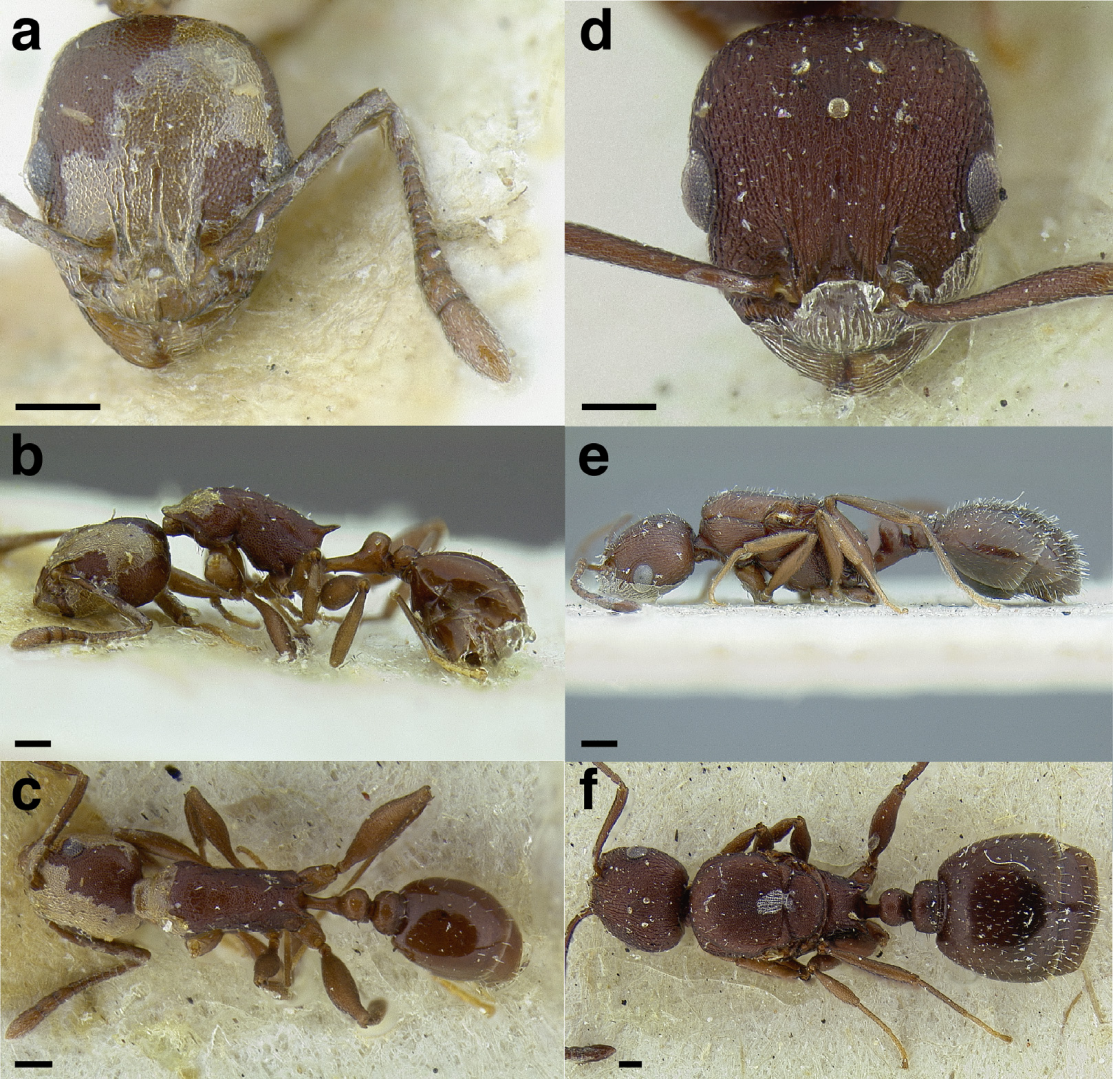
*Temnothorax augusti*. (A–C) ****Lectotype worker (MCZENT00021005). (A) Full-face view. (B) Profile view. (C) Dorsal view. (D-F) Gyne (MCZENT00577105). (D) Full-face view. (E) Profile view. (F) Dorsal view. Scale bars 0.2 mm.

Distribution: Fig. 101A; worker & gyne: Fig. 102.

*Leptothorax petiolatus* Forel, 1901a: 129. Syntype workers. Cuernavaca, Mexico. One worker here designated **lectotype**.

*Macromischa petiolata* (Forel): Emery, 1924: 247. First combination in *Macromischa* (nec

*Macromischa petiolata* Mayr, 1868: 15 (fossil species)).

*Macromischa foreli* Aguayo, 1931: 182. Nomen novum for *Macromischa petiolata* (Forel) (nec Mayr).

*Leptothorax augusti* Baroni Urbani, 1978: 423. Re-transfer to *Leptothorax*. Nomen novum for *Leptothorax foreli* (Aguayo) (nec *Leptothorax foreli* Santschi, 1907: 330).

*Temnothorax augusti* (Baroni Urbani): Bolton, 2003: 271. First combination in *Temnothorax*.

**Type material examined:** *Lectotype worker:* MEXICO: Morelos: Cuernavaca, [collection date unknown], [collector unspecified, but Wheeler (1931) states that he made the collection], in *Tillandsia*, M.C.Z. cotype 21005 (MCZENT00021005, top specimen on pin) [MCZC].

*Paralectotype worker*: same pin as lectotype (MCZENT00021005, bottom specimen on pin) [MCZC].

**Non-type material examined:** same data as previous, 1 dealate gyne (MCZENT00577105) [MCZC].

**Geographic range:** Mid elevations in South-Central Mexico (Morelos) (Fig. 101A).

**Worker diagnosis:** The following character combination separates *Temnothorax augusti* from all other species in the *salvini* clade: moderately long antennal scapes, which surpass the posterior margin of the head by about the maximum width of the scape (SI 103–117); mesosoma about one and a half times as long as the width of the head (WLI 143–150); propodeal spines shorter than the length of the propodeal declivity (PSI 29); petiolar node subquadrate, and overhangs the caudal cylinder of the petiole; postpetiole narrow, less than two times the width of the petiole in dorsal view (PWI 187–190); first gastral tergite with very faint areolate sculpture; erect setae on the dorsal surface of the head; integument medium brown.

**Similar species:** *Temnothorax acutispinosus* sp. nov., *T. subditivus, T. tenuisculptus, T. tuxtlanus* sp. nov., species of the *annexus*, *augusti*, *fuscatus*, and *salvini* groups. *Temnothorax augusti* can be distinguished from the first three species by the shape of the dorsal margin of the mesosoma in profile, which is predominantly flat: in *T. subditivus*, the mesosoma is strongly arched, while *T. tenuisculptus* and *T. tuxtlanus* sp. nov. have a sinuate profile. Petiolar node shape is also a useful character for separating *T. augusti* from the above taxa: *T. subditivus* and the potentially co-occurring members of the *salvini* group have a squamiform petiolar node, which is much broader than the peduncle in dorsal view (only slightly wider in *T. augusti*), and the nodes of the *annexus* group are erect and subquadrate, as opposed to leaning posteriorly in *T. augusti*; *T. tenuisculptus*, like *T. augusti*, has a subquadrate petiolar node, but it does not lean posteriorly over the caudal cylinder; *T. tuxtlanus* sp. nov. and *T. acutispinosus* sp. nov. have rounded petiolar nodes in profile view. *Temnothorax augusti* can also be distinguished from *T. tuxtlanus* sp. nov. and *T. acutispinosus* sp nov. by the presence of erect setae on the dorsum of the propodeum. The potentially co-occurring members of the *fuscatus* group have a low, elongate petiolar node in profile view. Finally, *T. augusti* can be separated from fellow members of the *augusti* group by the combination of short propodeal spines, which are shorter than the length of the propodeal declivity (as long as or longer than the declivity in other *augusti* group members, except *T. casanovai* sp. nov.), and the shining, faintly areolate first gastral tergite (which is strongly areolate and dull in *T. casanovai* sp. nov.).

**Worker measurements & indices (*n* = 2):** SL = 0.605–0.662 (0.634); FRS = 0.183–0.190 (0.187); CW = 0.624–0.630 (0.627); CWb = 0.566–0.588 (0.577); PoOC = 0.278–0.297 (0.288); CL = 0.693–0.737 (0.715); EL = 0.156–0.160 (0.158); EW = 0.118–0.153 (0.136); MD = 0.162–0.173 (0.168); WL = 0.812–0.884 (0.848); SPST = 0.239–0.252 (0.246); MPST = 0.247–0.263 (0.255); PEL = 0.306–0.340 (0.323); NOL = 0.177–0.212 (0.195); NOH = 0.116–0.124 (0.120); PEH = 0.208–0.225 (0.217); PPL = 0.137–0.153 (0.145); PPH = 0.186–0.193 (0.190); PW = 0.383–0.418 (0.401); SBPA = 0.144–0.152 (0.148); SPTI = 0.161–0.180 (0.171); PEW = 0.128–0.129 (0.129); PNW = 0.172–0.184 (0.178); PPW = 0.239–0.245 (0.242); HFL = 0.681–0.684 (0.683); HFWmax = 0.155–0.161 (0.158); HFWmin = 0.046–0.049 (0.048); CS = 0.913–0.957 (0.935); ES = 0.215–0.237 (0.226); SI = 103–117 (110); OI = 24–25 (24); CI = 80–82 (81); WLI = 143–150 (147); SBI = 25–26 (26); PSI = 29; PWI = 187–190 (188); PLI = 222–223 (223); NI = 153–171 (162); PNWI = 134–143 (139); NLI = 58–62 (60); FI = 329–337 (333).

**Worker description:** In full-face view, head subquadrate, longer than broad (CI 80–82). Mandibles densely, finely striate, weakly shining and armed with five teeth: the apical-most well developed and acute, followed by a less developed preapical tooth and three equally developed smaller teeth. Anterior clypeal margin weakly emarginated medially. Antennal scapes moderately long: when fully retracted, surpassing the posterior margin of the head capsule by about the maximum width of the scape (SI 103–117). Antennae 12-segmented; antennal club of composed of three segments, with the apical-most segment longer than the preceding two in combination. Frontal carinae moderately long, extending past the antennal toruli by about two and a half times the maximum width of the antennal scape. Compound eyes moderately protruding past the lateral margins of the head capsule. Lateral margin of head convex, forming a continuous arc from the mandibular insertions to the posterior margin of the head. Posterior head margin convex but rounding evenly into the lateral margins.

In profile view, compound eyes ovular and moderately large (OI 24–25), with 11 ommatidia in longest row. Pronotal declivity indistinct, but neck and anterior face of pronotum forming a ~120° angle. Mesosoma convex from where it joins the pronotal neck to the pronotal dorsum, then flat to the base of the propodeal spines. Promesonotal suture extending from the posterior margin of the procoxal insertion only to the mesothoracic spiracle, which is moderately well developed. Metanotal groove visible as a disruption of the sculpture laterally from where it arises between the mid- and hind coxae to where it ends in the poorly developed metathoracic spiracle, which is nearly indistinguishable against the ground sculpture, continuing dorsally as a disruption in the sculpture. Propodeal spiracle poorly developed, directed posterolaterally, and separated from the propodeal declivity by about four and a half spiracle diameters. Propodeal spines moderately well developed, but short (PSI 29), slightly shorter than the propodeal declivity, tapering evenly from the base, straight, and acute. Propodeal declivity straight, forming a rounded ~120° angle with the base of the propodeal spines. Propodeal lobes rounded and weakly developed. Metapleural gland bulla small, extending from the metacoxal insertion a third of the way to the propodeal spiracle. Petiole long (PLI 222–223), without tubercles anterodorsally. Subpetiolar process in the form of a very small, acute tooth; ventral margin of petiole weakly bulging medially. Petiolar peduncle long: comprising about two thirds of the petiole. Petiolar node robust and erect, nearly squamiform: transition between peduncle and node marked by a rounded angle of ~120°, resulting in a concave anterior node face; anterior face forming a ~100° angle with the dorsal face, which is weakly convex; dorsal face rounding evenly into the short posterior face, which forms a ~85° angle with the caudal cylinder, overhanging it. Postpetiole evenly rounded anteriorly, flattened dorsally, and weakly lobed ventrally.

In dorsal view, humeri developed: evenly rounded and wider than the rest of the mesosoma; mesothoracic spiracles not protruding past the lateral margins of the mesosoma. Metanotal groove absent: mesonotum and propodeum completely fused and lateral margins converging evenly to the bases of the propodeal spines. Propodeal spines narrowly approximated basally, joined by a transverse welt, and diverging apically, their apices separated from each other by about their length, the negative space between them “U” shaped. Petiolar peduncle with spiracles protruding past the lateral margins, broadened where they arise. Petiolar node campaniform: flattened posteriorly. Petiolar node broader than the peduncle, and about one and a quarter times as wide as the caudal cylinder. Postpetiole narrow (PWI 187–190) and campaniform, articulating with most of the anterior margin of the gaster, leaving small, angulate margins on each side exposed. Anterior margin of the postpetiole convex and evenly rounds into the lateral margins, which diverge to the angulate posterior corners; posterior margin flat. Metafemur strongly incrassate (FI 329–337).

Sculpture: median clypeal carina present, extending posteriorly nearly to the frontal triangle, and flanked on either side by two slightly weaker carinae. Lateral clypeal lobes with additional, weaker carinae; ground sculpture weakly areolate. Antennal scapes areolate. Cephalic dorsum areolate; costulae flanking the frontal carinae, and weak concentric costulae surrounding the antennal insertions. Lateral surfaces of head areolate, and weak rugulose sculpture between the compound eye and the mandibular insertion. Ventral surface of head weakly areolate. Mesosoma areolate-rugulose, with weak rugulae overlying it dorsally. Femora weakly areolate. Petiole and postpetiole predominantly smooth and shining, with traces of weak areolate sculpture. Gaster smooth and shining, with traces of areolate sculpture and spectral iridescence on the basal half of the first gastral tergite.

Setae: antennal scapes and funiculi with short, decumbent pilosity. Dorsum of the head, pronotum, waist segments, and gaster with moderately abundant, erect, blunt-tipped setae, the longest of which are about the width of the compound eye. The head bears ~40, mesosoma ~26, petiole 6, postpetiole ~10, and first gastral tergite ~40 setae. Short, sparse pubescence present over the entire body, but difficult to detect against the ground sculpture.

Color: predominantly medium brown, with yellowish tarsi.

**Gyne measurements & indices (*n* = 1):** SL = 0.746; FRS = 0.288; CW = 0.874; CWb = 0.819; PoOC = 0.361; CL = 0.931; EL = 0.255; EW = 0.197; MD = 0.204; WL = 1.437; SPST = 0.323; MPST = 0.416; PEL = 0.483; NOL = 0.224; NOH = 0.216; PEH = 0.373; PPL = 0.279; PPH = 0.311; PW = 0.859; SBPA = 0.371; SPTI = 0.378; PEW = 0.213; PNW = 0.300; PPW = 0.432; HFL = 0.900; HFWmax = 0.191; HFWmin = 0.070; CS = 1.285; ES = 0.354; SI = 91; OI = 28; CI = 88; WLI = 175; SBI = 45; PSI = 22; PWI = 203; PLI = 173; NI = 104; PNWI = 141; NLI = 46; FI = 273.

**Gyne description:** In full-face view, head subquadrate, longer than broad (CI 88). Mandibles densely, finely striate but weakly shining and armed with five teeth: the apical-most well developed, followed by a less developed preapical tooth and three equally developed smaller teeth. Anterior clypeal margin weakly emarginated medially. Antennal scapes moderately long: when fully retracted, just reaching the posterior margin of the head capsule (SI 91). Antennae 12-segmented; antennal club composed of three segments, with the apical-most segment longer than the preceding two in combination. Frontal carinae long, extending past the antennal toruli by about three times the maximum width of the antennal scape. Compound eyes moderately protruding past the lateral margins of the head capsule. Lateral margin of head evenly convex, converging from below the compound eyes to the mandibular insertions. Posterior head margin convex, rounding evenly into the lateral margins.

In profile view, compound eyes ovular and large (OI 28), with 22 ommatidia in longest row. Mesoscutum rounded evenly anteriorly, covering the dorsal surface of the pronotum, and flat dorsally. Mesoscutellum at the same level as the mesoscutum. Posterior margin of metanotum extending slightly past the posterior margin of the mesoscutum. Propodeal spiracle poorly developed, directed posterolaterally, and separated from the propodeal declivity by about five spiracle diameters. Propodeal spines stout and well developed, but short (PSI 22), about half as long as the propodeal declivity, tapering evenly from the base, directed posteriorly, straight, and blunt. Propodeal declivity straight and flat, forming a rounded ~100° angle with the base of the propodeal spines. Propodeal lobes rounded and very weakly developed. Metapleural gland bulla small, extending from the metacoxal insertion a third of the way to the propodeal spiracle. Petiole moderately long (PLI 173), without tubercles anterodorsally. Subpetiolar process in the form of a very small, acute tooth; ventral margin of the petiole bulging slightly medially. Petiolar peduncle long: comprising about two thirds of the petiole. Petiolar node robust and erect, nearly squamiform: transition between peduncle and node marked by a rounded angle of ~120°, resulting in a concave anterior node face; anterior face forming a ~100° angle with the dorsal face, which is weakly convex; dorsal face rounding evenly into the short posterior face, which forms a ~85° angle with the caudal cylinder, overhanging it. Postpetiole subquadrate: flat anteriorly, transitioning into the flattened dorsal face through a rounded angle; ventral surface weakly lobed.

In dorsal view, mesoscutum covering pronotum anteriorly, but humeri visible laterally as slightly angulate sclerites. Propodeal spines broadly approximated basally and diverging apically, their apices separated from each other by about twice their length. Petiolar peduncle with spiracles protruding past the lateral margins, the peduncle constricted anterior to them. Petiolar node subovate and transversely broad. Petiolar node broader than the peduncle, and about one and a half times as wide as the caudal cylinder. Postpetiole moderately broad (PWI 203), anteroposteriorly compressed, and subquadrate, articulating with most of the anterior margin of the gaster, leaving small, angulate margins on each side exposed. Anterior margin of postpetiole flat, with corners marked by rounded angles as it transitions to the lateral margins, which weakly diverge to the angulate posterior corners; posterior margin flat. Metafemur moderately incrassate (FI 273).

Sculpture: median clypeal carina present but flanked by numerous equally strong carinae. Lateral clypeal lobes with additional weaker carinae; ground sculpture weakly areolate. Antennal scapes areolate. Cephalic dorsum costulate, with areolate-rugulose ground sculpture; weak concentric costulae surrounding the antennal insertions. Lateral surfaces of head sculptured similarly to the dorsum. Ventral surface of head sculptured similarly to the rest of the head, but weaker. Pronotal neck areolate. Pronotum, metapleuron and lateral face of the propodeum sculptured similarly to the head dorsum. Anepisternum and katepisternum weakly areolate on their anterior quarters, transitioning into stronger areolate sculpture posteriorly. Propodeal declivity weakly areolate. Mesoscutum and mesoscutellum with longitudinal striae over areolate ground sculpture. Femora areolate. Petiole and postpetiole weakly areolate. First gastral tergite weakly areolate, with weak spectral iridescence. Surface of the first gastral sternite with weak spectral iridescence.

Setae: antennal scapes and funiculi with short, decumbent pilosity. Dorsum of the head, pronotum, waist segments, and gaster with moderately abundant, erect, blunt-tipped setae, the longest of which are about a third of the width of the compound eye. Short, sparse pubescence present over the entire body, but difficult to detect against the ground sculpture.

Color: predominantly medium brown, with yellowish tarsi.

**Male:** Unknown.

**Etymology:** Patronym, for the Swiss myrmecologist Auguste Forel, who originally described this species.

**Comments:** *Temnothorax augusti* is known only from the type series, which was collected from *Tillandsia* epiphytes near Cuernavaca, Morelos, Mexico by William Morton Wheeler. The epiphyte in which the nest series was collected was shared with two other ant species: *Crematogaster crinosa* Mayr, a common inhabitant of the canopy and disturbed areas in the Neotropics, and *Cephalotes wheeleri* (Forel), a much less common arboreal species known only from Central Mexico (Wheeler, 1910). The morphologically closest species to *T. augusti* is *T. casanovai* sp. nov., which was collected in neighboring Puebla state, Mexico from the limb of a pitahaya cactus, *Hylocereus undatus*, a hemiepiphytic species commonly cultivated for its fruit. Based on multiple morphological disparities between the two, including gross size, distance between the base of the propodeal spines, degree of sculpture on the first gastral tergite, and color, I choose to keep these species separate in the current revision. This issue should be revisited as more collections become available. This species is reported from Quintana Roo by Lachaud & Lachaud (2013); I have not personally examined these specimens, so I have excluded it from the range for the moment.

***Temnothorax aureus* sp. nov.**

**Figure 103:**
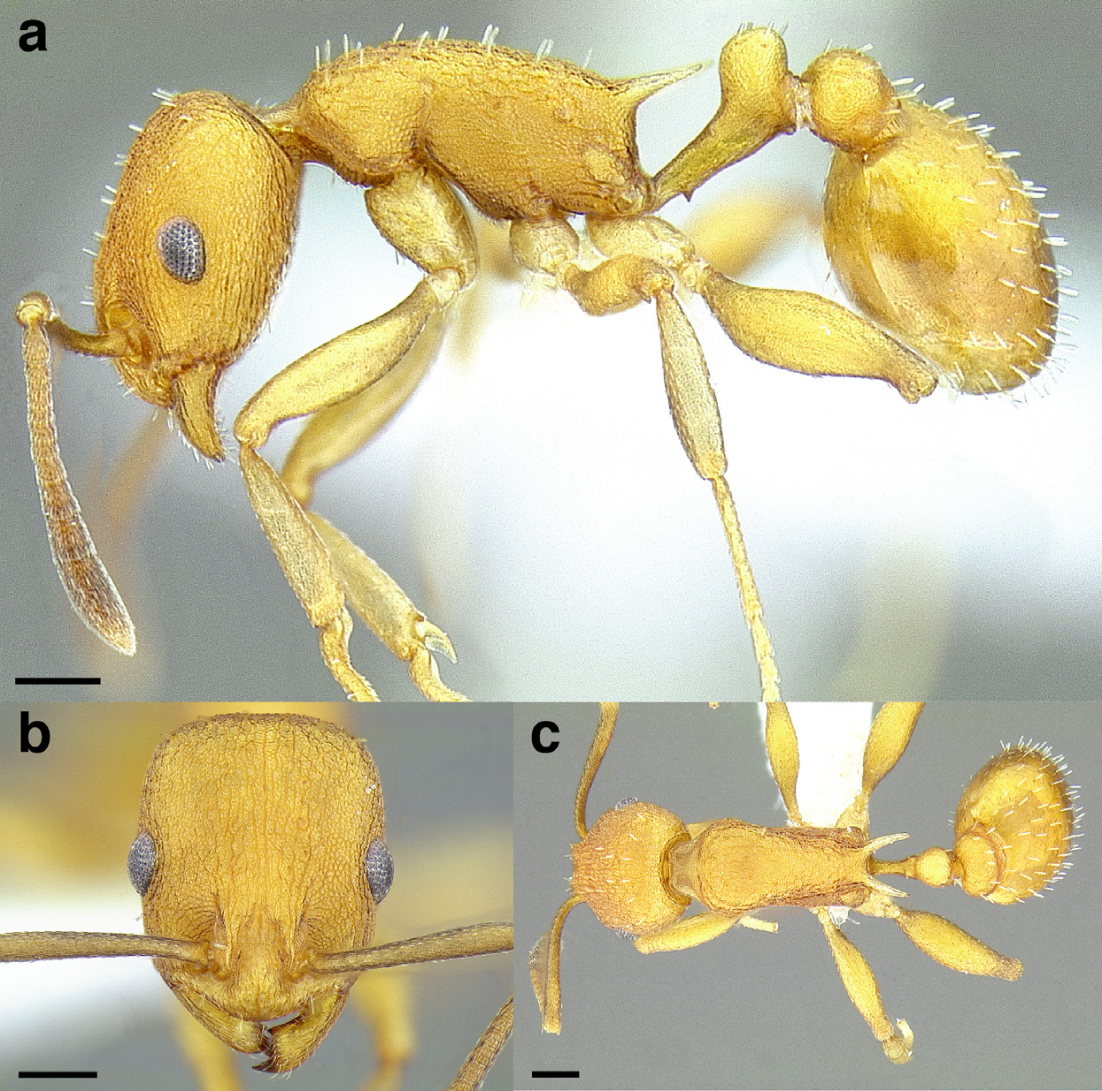
*Temnothorax aureus* sp. nov. holotype worker (CASENT0619363). (A) Full-face view. (B) Profile view. (C) Dorsal view. Scale bars 0.2 mm.

Distribution: Fig. 101B; worker: Fig. 103.

*Temnothorax* mmp13 Prebus, 2017: 8. Phylogeny.

**Type material examined:** *Holotype worker:* NICARAGUA: Estelí: 16 km N Estelí, 13.24082°N 86.35307°W ±100 m, 870 m, 25 April 2011, J. Longino#JTL7420-s, tropical dry scrub, strays (CASENT0619363) [CASC].

*Paratype worker:* NICARAGUA: Estelí: same data as holotype, 1 worker (CASENT0619364) [UCDC].

**Non-type material examined:** EL SALVADOR: La Libertad: Quezaltepeque, 500 m, 19 June 1985, D.Q. Cavagnaro & M.E. Irwin collectors, ANTC29707, 1 worker (CASENT0740646) [CASC]. La Paz: Isla Tasajera, 13.269°N 88.849°W, 31 July 2012, James K. Wetterer#161, forest, strays, 1 worker (CASENT0631217) [JTLC]. San Salvador: San Salvador, Jardín Botánico La Laguna, 13.669°N 89.247°W, 5 August 2012, James K. Wetterer#262, garden, 1 worker (CASENT0756095) [MCZC] 1 worker (CASENT0638812) [JTLC].

**Geographic range:** Low elevations of Southern El Salvador to Northwest Nicaragua (Fig. 101B).

**Worker diagnosis:** The following character combination separates *Temnothorax aureus* sp. nov. from all other species in the *salvini* clade: head relatively narrow (CI 77–81); antennal scapes moderately long, surpassing the posterior margin of the head by the maximum width of the scape (SI 106–108); mesosoma about one and a half times as long as the width of the head (WLI 147–152); propodeal spines about as long as the length of the propodeal declivity (PSI 36–40); petiolar node subquadrate, overhanging the caudal cylinder of the petiole; postpetiole moderately broad, more than two times the width of the petiole in dorsal view (PWI 205–218); erect setae on the dorsal surface of the head; integument testaceous yellow.

**Similar species:** *Temnothorax acutispinosus* sp. nov., *T. subditivus, T. tenuisculptus, T. tuxtlanus* sp. nov., species of the *annexus*, *augusti*, *fuscatus*, and *salvini* groups. *Temnothorax aureus* sp. nov. can be distinguished from the first three species by the shape of the dorsal margin of the mesosoma in profile, which is weakly, evenly convex: in *T. subditivus*, the mesosoma is strongly arched, while sinuate in *T. tenuisculptus* and *T. tuxtlanus* sp. nov. Petiolar node shape is also a useful character for separating *T. aureus* sp. nov. from the above taxa: *T. subditivus* and the potentially co-occurring members of the *salvini* group have a squamiform petiolar node, which is much broader than the peduncle in dorsal view (only slightly wider in *T. aureus* sp. nov.), and the nodes of the *annexus* group and *T. tenuisculptus* are erect and subquadrate, as opposed to leaning posteriorly in *T. aureus* sp. nov.; *T. tuxtlanus* sp. nov. and *T. acutispinosus* sp. nov. have rounded petiolar nodes in profile view. *Temnothorax aureus* sp. nov. can also be distinguished from *T. tuxtlanus* sp. nov. and *T. acutispinosus* sp. nov. by the presence of erect setae on the dorsum of the propodeum. The potentially co-occurring members of the *fuscatus* group have a low, elongate petiolar node in profile view. Finally, *T. aureus* sp. nov. can be separated from fellow members of the *augusti* group by the combination of long propodeal spines, which are about as long as the length of the propodeal declivity (shorter than the declivity in *T. augusti* and *T. casanovai* sp. nov.), the relatively narrow head (broader in *T. leucacanthoides* sp. nov.: CI 77–81 vs. > 84); relatively long antennal scapes (SI 106–108 vs. < 105 in *T. leucacanthus* and *T. leucacanthoides* sp. nov.).

**Worker measurements & indices (*n* = 4):** SL = 0.588–0.639 (0.621); FRS = 0.201–0.227 (0.216); CW = 0.606–0.649 (0.635); CWb = 0.546–0.601 (0.579); PoOC = 0.251–0.287 (0.278); CL = 0.676–0.752 (0.729); EL = 0.155–0.174 (0.165); EW = 0.112–0.123 (0.120); MD = 0.157–0.191 (0.179); WL = 0.802–0.909 (0.865); SPST = 0.310–0.337 (0.326); MPST = 0.260–0.271 (0.266); PEL = 0.324–0.380 (0.360); NOL = 0.144–0.215 (0.182); NOH = 0.131–0.145 (0.140); PEH = 0.245–0.255 (0.248); PPL = 0.162–0.196 (0.178); PPH = 0.202–0.221 (0.211); PW = 0.409–0.452 (0.433); SBPA = 0.174–0.195 (0.186); SPTI = 0.239–0.292 (0.270); PEW = 0.145–0.154 (0.150); PNW = 0.166–0.189 (0.180); PPW = 0.314–0.330 (0.320); HFL = 0.607–0.690 (0.665); HFWmax = 0.165–0.191 (0.176); HFWmin = 0.046–0.060 (0.055); CS = 0.884–0.977 (0.943); ES = 0.211–0.236 (0.225); SI = 106–108 (107); OI = 23–25 (24); CI = 77–81 (79); WLI = 147–152 (149); SBI = 30–35 (32); PSI = 36–40 (38); PWI = 205–218 (213); PLI = 194–213 (202); NI = 110–148 (129); PNWI = 114–123 (120); NLI = 44–58 (50); FI = 284–385 (326).

**Worker description:** In full-face view, head subquadrate, elongate (CI 77–81). Mandibles densely, finely striate but shining and armed with five teeth: the apical-most well developed and acute, followed by a less developed preapical tooth and three equally developed smaller teeth. Anterior clypeal margin evenly rounded medially. Antennal scapes moderately long: when fully retracted, surpassing the posterior margin of the head capsule by about the maximum width of the scape (SI 106–108). Antennae 12-segmented; antennal club of composed of three segments, with the apical-most segment slightly longer than the preceding two in combination. Frontal carinae moderately long, extending past the antennal toruli by about two times the maximum width of the antennal scape. Compound eyes moderately protruding past the lateral margins of the head capsule. Lateral margin of head very weakly convex, nearly flat, forming a continuous arc from the mandibular insertions to the posterior margin of the head. Posterior head margin medially weakly emarginate, but predominantly flat, rounding evenly into the lateral margins.

In profile view, compound eyes ovular and moderately large (OI 23–25), with 12 ommatidia in longest row. Pronotal declivity indistinct, but neck and anterior face of pronotum forming a ~120° angle; anterior face evenly rounding into the dorsal face. Mesosoma evenly, but weakly convex from where it joins the pronotal neck to the propodeal spines. Promesonotal suture extending from the posterior margin of the procoxal insertion only to the mesothoracic spiracle, which is moderately well developed. Metanotal groove visible as a disruption of the sculpture laterally from where it arises between the mid- and hind coxae to where it ends in the poorly developed metathoracic spiracle, which is nearly indistinguishable against the ground sculpture. Propodeal spiracle moderately well developed, directed posterolaterally, and separated from the propodeal declivity by about four spiracle diameters. Propodeal spines well developed and very long (PSI 36–40), slightly longer than the propodeal declivity, tapering evenly from the base, upturned at the tips, and acute. Propodeal declivity flat, forming a rounded ~110° angle with the base of the propodeal spines. Propodeal lobes rounded and weakly developed. Metapleural gland bulla moderately large, extending from the metacoxal insertion two-thirds of the way to the propodeal spiracle. Petiole long (PLI 194–213), without tubercles anterodorsally. Subpetiolar process in the form of an acute tooth, ventral margin of petiole very weakly bulging medially. Petiolar peduncle long: comprising two thirds the length of the petiole. Petiolar node robust and erect, nearly squamiform: transition between peduncle and node marked by a rounded angle of ~120°, resulting in a concave anterior node face; anterior face rounding evenly into the dorsal face, which is evenly convex and slightly bulging; dorsal face rounding evenly into the short posterior face, which forms a ~85° angle with the caudal cylinder, overhanging it. Postpetiole evenly rounded anterodorsally, rounding evenly into the flattened dorsal face; weakly lobed ventrally.

In dorsal view, dorsal margin of pronotum delimited from the pronotal declivity by a weak carina. Humeri developed: evenly rounded and wider than the rest of the mesosoma; mesothoracic spiracles very weakly protruding past the lateral margins of the mesosoma, visible as slight angles where the pronotum meets the mesonotum. Promesonotal suture visible as a disruption in the sculpture. Metanotal groove absent: mesonotum and propodeum completely fused and lateral margins converging evenly to the bases of the propodeal spines. Propodeal spines broadly approximated basally, diverging weakly apically, their apices separated from each other by slightly less than their length, the negative space between them “U” shaped. Petiolar peduncle with spiracles slightly protruding past the lateral margins; peduncle weakly constricted anterior to them. Petiolar node campaniform: very weakly convex posteriorly, nearly flat; node broader than the peduncle, and slightly wider than the caudal cylinder. Postpetiole subquadrate and moderately broad (PWI 205–218), articulating with most of the anterior margin of the gaster, leaving small, angulate margins on each side exposed. Anterior margin of the postpetiole flat, evenly rounding into the lateral margins, which are parallel to the angulate posterior corners; posterior margin broadly concave. Metafemur moderately to strongly incrassate (FI 284–385).

Sculpture: median clypeal carina present, extending posteriorly nearly to the frontal triangle, and slightly stronger than the numerous carinae that flank it. Lateral clypeal lobes with additional, weaker carinae; ground sculpture areolate. Antennal scapes areolate. Cephalic dorsum areolate, but with fine rugae overlying the ground sculpture, becoming costate between the frontal carinae; fine concentric costulae surrounding the antennal insertions. Lateral surfaces of head sculptured similarly to the dorsum of the head, but with rugae becoming costate posterior to the compound eye. Ventral surface of head shining through weak areolate-costulate sculpture. Mesosoma with areolate sculpture on the pronotal neck. Lateral surface of the mesosoma strongly areolate, with fine rugose-costate sculpture on the pronotum, on the border of the meso- and metapleurae, and on the propodeal declivity. Dorsal surface of mesosoma areolate, with rugose-costate sculpture overlying the ground sculpture. Femora areolate. Petiole and postpetiole predominantly areolate, but sculpture is weaker on the ventral surface of the petiolar peduncle. Basal half of first gastral tergite weakly, finely areolate-rugulose, with spectral iridescence, otherwise smooth and shining. First gastral sternite with spectral iridescence.

Setae: antennal scapes and funiculi with short, decumbent pilosity. Dorsum of the head, pronotum, waist segments, and gaster with moderately abundant, erect, blunt-tipped, nearly clavate setae, the longest of which are slightly shorter than the width of the compound eye. The head bears ~28, mesosoma ~24, petiole 4, postpetiole ~14, and first gastral tergite ~58 setae. Short, sparse pubescence present over the entire body, but difficult to detect against the ground sculpture and light integument.

Color: predominantly testaceous yellow, with masticatory margin of mandibles dark brown.

**Gyne:** Unknown.

**Male:** Unknown.

**Etymology:** Morphological, from the Latin ‘aureus’ (= golden).

**Comments:** *Temnothorax aureus* sp. nov. is apparently broadly distributed on the Pacific Slope of Central America but known only from collections of stray workers made at low elevations. This species closely resembles a lightly colored form *T. leucacanthus* and *T. leucacanthoides* sp. nov. from Southern Mexico; the latter species was found nesting in dead vegetation in littoral habitat.

***Temnothorax leucacanthoides* sp. nov.**

**Figure 104:**
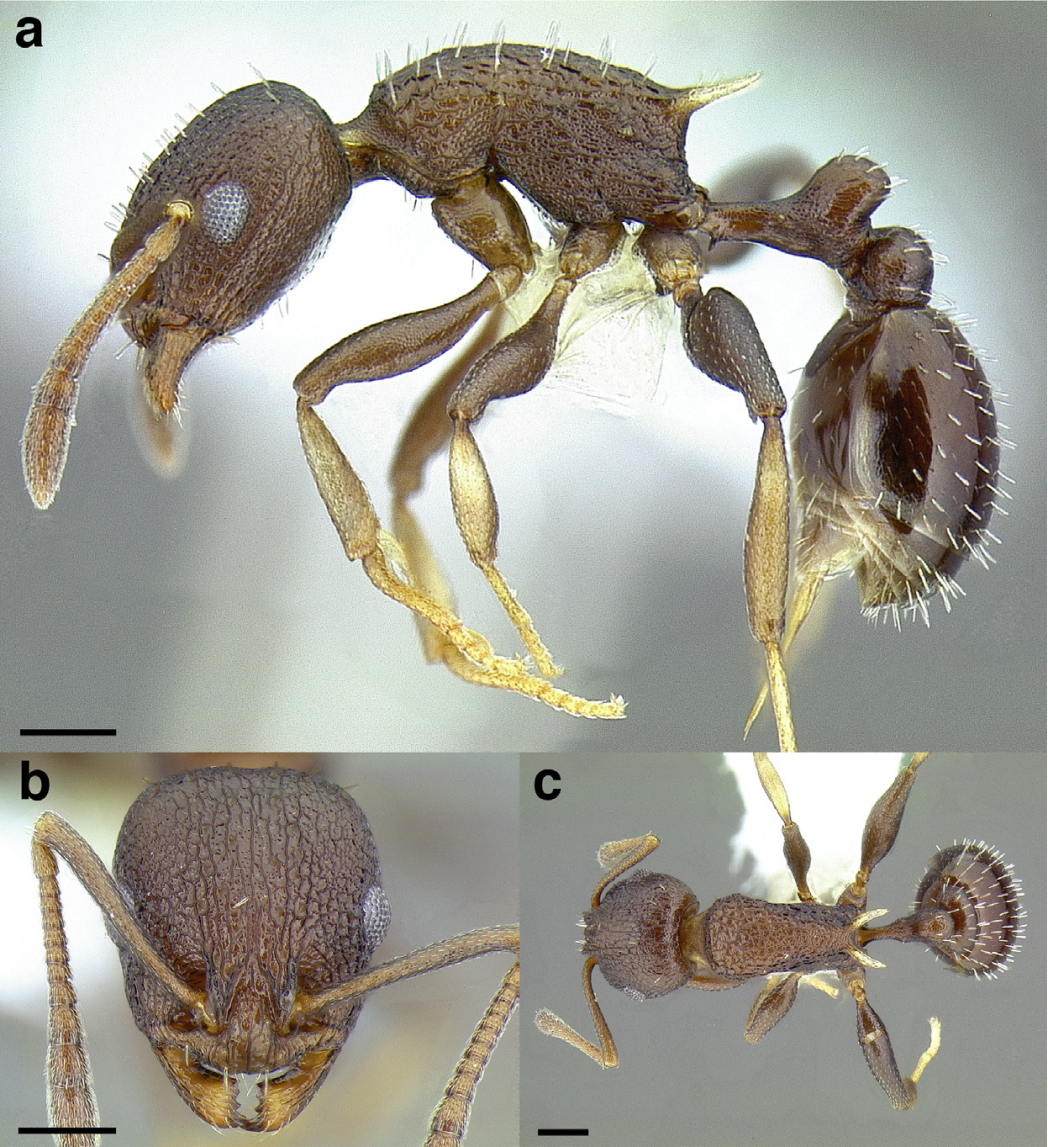
*Temnothorax leucacanthoides* sp. nov. holotype worker (CASENT0756102). (A) Full-face view. (B) Profile view. (C) Dorsal view. Scale bars 0.2 mm.

Distribution: Fig. 101C; worker: Fig. 104.

*Temnothorax* sp. nr. *augusti* Prebus, 2017: 8. Phylogeny.

**Type material examined:** *Holotype worker:* MEXICO: Oaxaca: Cerro Largo, 6 km W Puerto Angel, 20 m, 15.666667°N 96.550000°W, 22 March 2006, P.S. Ward#15573, littoral vegetation, ex dead stalk of Asteraceae (CASENT0756102, top specimen on pin) [CASC].

*Paratype workers:* same pin as holotype, 1 worker (CASENT0756102, bottom specimen on pin) [CASC]; same data as holotype, 1 worker (CASENT0732606) [UCDC] 1 worker (CASENT0758708) [UCDC] 3 workers (CASENT0916001) [MCZC].

**Geographic range:** Low elevations of southern Mexico (Oaxaca) (Fig. 101C).

**Worker diagnosis:** The following character combination separates *Temnothorax leucacanthoides* sp. nov. from all other species in the *salvini* clade: head relatively broad (CI 84–89); moderately long antennal scapes, which just reach the posterior margin of the head (SI 86–96); mesosoma about one and a half times as long as the width of the head (WLI 136–143); propodeal spines about as long as the length of the propodeal declivity (PSI 39–42); petiolar node subquadrate, overhanging the caudal cylinder of the petiole; postpetiole moderately to very broad, more than two times the width of the petiole in dorsal view (PWI 202–224); integument medium brown; erect setae on the dorsal surface of the head.

**Similar species:** *Temnothorax acutispinosus* sp. nov., *T. subditivus, T. tenuisculptus, T. tuxtlanus* sp. nov., species of the *annexus, augusti*, *fuscatus*, and *salvini* groups. *Temnothorax leucacanthoides* sp. nov. can be distinguished from the first three species by the shape of the dorsal margin of the mesosoma in profile, which is weakly, evenly convex (with the metanotal groove slightly impressed in some specimens): in *T. subditivus*, the mesosoma is strongly arched, while *T. tenuisculptus* and *T. tuxtlanus* sp. nov. have a sinuate profile. Petiolar node shape is also a useful character for separating *T. leucacanthoides* sp. nov. from the above taxa: *T. subditivus* and the potentially co-occurring members of the *salvini* group have a squamiform petiolar node, which is much broader than the peduncle in dorsal view (only slightly wider in *T. leucacanthoides* sp. nov.), and the nodes of the *annexus* group are erect and subquadrate, as opposed to leaning posteriorly in *T. leucacanthoides* sp. nov.; *T. tenuisculptus*, like *T. leucacanthoides* sp. nov., has a subquadrate petiolar node, but it does not lean posteriorly over the caudal cylinder; *T. tuxtlanus* sp. nov. and *T. acutispinosus* sp. nov. have rounded petiolar nodes in profile view. *Temnothorax leucacanthoides* sp. nov. can also be distinguished from *T. tuxtlanus* sp. nov. and *T. acutispinosus* sp. nov. by the presence of erect setae on the dorsum of the propodeum. The potentially co-occurring members of the *fuscatus* group have a low, elongate petiolar node in profile view. Finally, *T. leucacanthoides* sp. nov. can be separated from fellow members of the *augusti* group by the combination of long propodeal spines, which are about as long as the length of the propodeal declivity (shorter than the declivity in *T. augusti* and *T. casanovai* sp. nov.), the relatively broad head (narrower in all other *augusti* group species: CI > 84 vs. < 82); relatively short antennal scapes (SI 86–96 vs. > 105 in *T. augusti*, *T. aureus* sp. nov., *T. casanovai* sp. nov.).

**Worker measurements & indices (*n* = 7):** SL = 0.521–0.573 (0.547); FRS = 0.182–0.197 (0.189); CW = 0.618–0.658 (0.64); CWb = 0.578–0.611 (0.596); PoOC = 0.251–0.263 (0.256); CL = 0.680–0.704 (0.693); EL = 0.158–0.166 (0.162); EW = 0.112–0.126 (0.120); MD = 0.171–0.188 (0.179); WL = 0.815–0.863 (0.836); SPST = 0.319–0.360 (0.335); MPST = 0.249–0.276 (0.261); PEL = 0.349–0.376 (0.363); NOL = 0.181–0.206 (0.192); NOH = 0.122–0.150 (0.137); PEH = 0.240–0.260 (0.251); PPL = 0.154–0.187 (0.170); PPH = 0.198–0.225 (0.211); PW = 0.420–0.454 (0.437); SBPA = 0.159–0.184 (0.171); SPTI = 0.259–0.280 (0.267); PEW = 0.150–0.165 (0.156); PNW = 0.174–0.192 (0.184); PPW = 0.317–0.342 (0.330); HFL = 0.555–0.603 (0.583); HFWmax = 0.145–0.173 (0.162); HFWmin = 0.044–0.054 (0.048); CS = 0.920–0.957 (0.942); ES = 0.214–0.229 (0.221); SI = 86–96 (92); OI = 23–24 (24); CI = 84–89 (86); WLI = 136–143 (140); SBI = 28–30 (29); PSI = 39–42 (40); PWI = 202–224 (211); PLI = 198–244 (215); NI = 127–155 (141); PNWI = 112–123 (118); NLI = 50–55 (53); FI = 302–393 (341).

**Worker description:** In full-face view, head subquadrate, longer than broad (CI 84–89). Mandibles densely, finely striate but shining and armed with five teeth: the apical-most well developed and acute, followed by a less developed preapical tooth and three equally developed smaller teeth. Anterior clypeal margin flat medially. Antennal scapes moderately long: when fully retracted, just reaching the posterior margin of the head capsule (SI 86–96). Antennae 12-segmented; antennal club of composed of three segments, with the apical-most segment slightly longer than the preceding two in combination. Frontal carinae long, extending past the antennal toruli by about three times the maximum width of the antennal scape. Compound eyes moderately protruding past the lateral margins of the head capsule. Lateral margin of head weakly convex, forming a continuous arc from the mandibular insertions to the posterior margin of the head. Posterior head margin medially weakly emarginate, but predominantly flat, rounding evenly into the lateral margins.

In profile view, compound eyes ovular and moderately large (OI 23–24), with 10 ommatidia in longest row. Pronotal declivity distinct, neck and anterior face of pronotum forming a ~120° angle; anterior face and dorsal face meeting at a ~120° angle. Mesosoma evenly convex from where it joins the pronotal declivity to the propodeal spines, but the metanotal groove slightly impressed in some specimens. Promesonotal suture extending from the posterior margin of the procoxal insertion only to the mesothoracic spiracle, which is moderately well developed. Propodeal spiracle moderately well developed, directed posterolaterally, and separated from the propodeal declivity by about three and a half spiracle diameters. Propodeal spines well developed, and moderately long (PSI 39–42), about as long as the propodeal declivity, tapering evenly from the base, upturned at the tips, and acute. Propodeal declivity flat, forming a rounded ~110° angle with the base of the propodeal spines. Propodeal lobes rounded and weakly developed. Metapleural gland bulla small, extending from the metacoxal insertion halfway to the propodeal spiracle. Petiole long (PLI 198–244), without tubercles anterodorsally. Subpetiolar process in the form of an acute tooth, ventral margin of petiole very weakly bulging medially. Petiolar peduncle moderately long: half the length of the petiole. Petiolar node robust and erect, nearly squamiform: transition between peduncle and node marked by a rounded angle of ~120°, resulting in a concave anterior node face; anterior face rounding evenly into the dorsal face, which is evenly convex; dorsal face rounding evenly into the posterior face, which forms a ~80° angle with the caudal cylinder, overhanging it. Postpetiole flat anteriorly, rounding evenly into the flattened dorsal face; weakly lobed ventrally.

In dorsal view, dorsal margin of pronotum delimited from the pronotal declivity by a carina. Humeri developed: evenly rounded and wider than the rest of the mesosoma; mesothoracic spiracles very weakly protruding past the lateral margins of the mesosoma, visible as slight angles where the pronotum meets the mesonotum. Metanotal groove visible in some specimens as a slight disruption in the sculpture. Propodeal spines narrowly approximated basally, but diverging rapidly and becoming parallel apically, their apices separated from each other by slightly less than their length, the negative space between them “V” shaped. Petiolar peduncle with spiracles protruding past the lateral margins; peduncle constricted anterior to them. Petiolar node campaniform: very weakly convex posteriorly, nearly flat; node broader than the peduncle, and slightly wider than the caudal cylinder. Postpetiole subquadrate and moderately to strongly broad (PWI 202–224), articulating with most of the anterior margin of the gaster, leaving small, angulate margins on each side exposed. Anterior margin of the postpetiole very weakly convex, nearly flat, and evenly rounding into the lateral margins, which weakly diverge to the angulate posterior corners; posterior margin broadly concave. Metafemur strongly incrassate (FI 302–393).

Sculpture: median clypeal carina present, extending posteriorly to the level of the antennal toruli, and flanked on either side by two equally strong carinae. Lateral clypeal lobes with additional, weaker carinae; ground sculpture weakly areolate. Antennal scapes areolate. Cephalic dorsum areolate, but with rugae overlying the ground sculpture, becoming costate between the frontal carinae; concentric costulae surrounding the antennal insertions. Lateral surfaces of head sculptured similarly to the dorsum of the head, but with rugae becoming weaker posterior to the compound eye. Ventral surface of head predominantly smooth and shining. Mesosoma with areolate sculpture on the pronotal neck. Lateral surface of the mesosoma strongly areolate, with overlying rugose-costate sculpture on the pronotum, on the border of the meso- and metapleurae, and on the propodeal declivity. Dorsal surface of mesosoma areolate-rugulose, with rugae overlying the ground sculpture. Femora shining through weakly areolate sculpture. Petiole and postpetiole predominantly areolate, but sculpture is weaker on the petiolar peduncle, dorsal face of the petiolar node, and the anteromedial part of the postpetiole. First gastral tergite smooth and shining, with weak spectral iridescence. First gastral sternite smooth and shining, without spectral iridescence.

Setae: antennal scapes and funiculi with short, decumbent pilosity. Dorsum of the head, pronotum, waist segments, and gaster with moderately abundant, erect, blunt-tipped setae, the longest of which are about the width of the compound eye. The head bears ~32, mesosoma ~38, petiole 10, postpetiole ~20, and first gastral tergite ~72 setae. Short, sparse pubescence present over the entire body, but difficult to detect against the ground sculpture.

Color: predominantly medium brown, with mandibles, antennae, pronotal neck, tibiae, tarsi, propodeal spines, and sting testaceous yellow.

**Gyne:** Unknown.

**Male:** Unknown.

**Etymology:** Morphological, for the close resemblance to *Temnothorax leucacanthus*.

**Comments:** *Temnothorax leucacanthoides* sp. nov. is known from a single collection made by Phil Ward in Oaxaca state, Mexico. The type series was nesting in the dead stalk of a plant of the Asteraceae in littoral vegetation. *Temnothorax leucacanthoides* sp. nov. is morphologically close to *T. leucacanthus* from neighboring Guerrero state.

***Temnothorax leucacanthus* (Baroni Urbani, 1978)**

**Figure 105:**
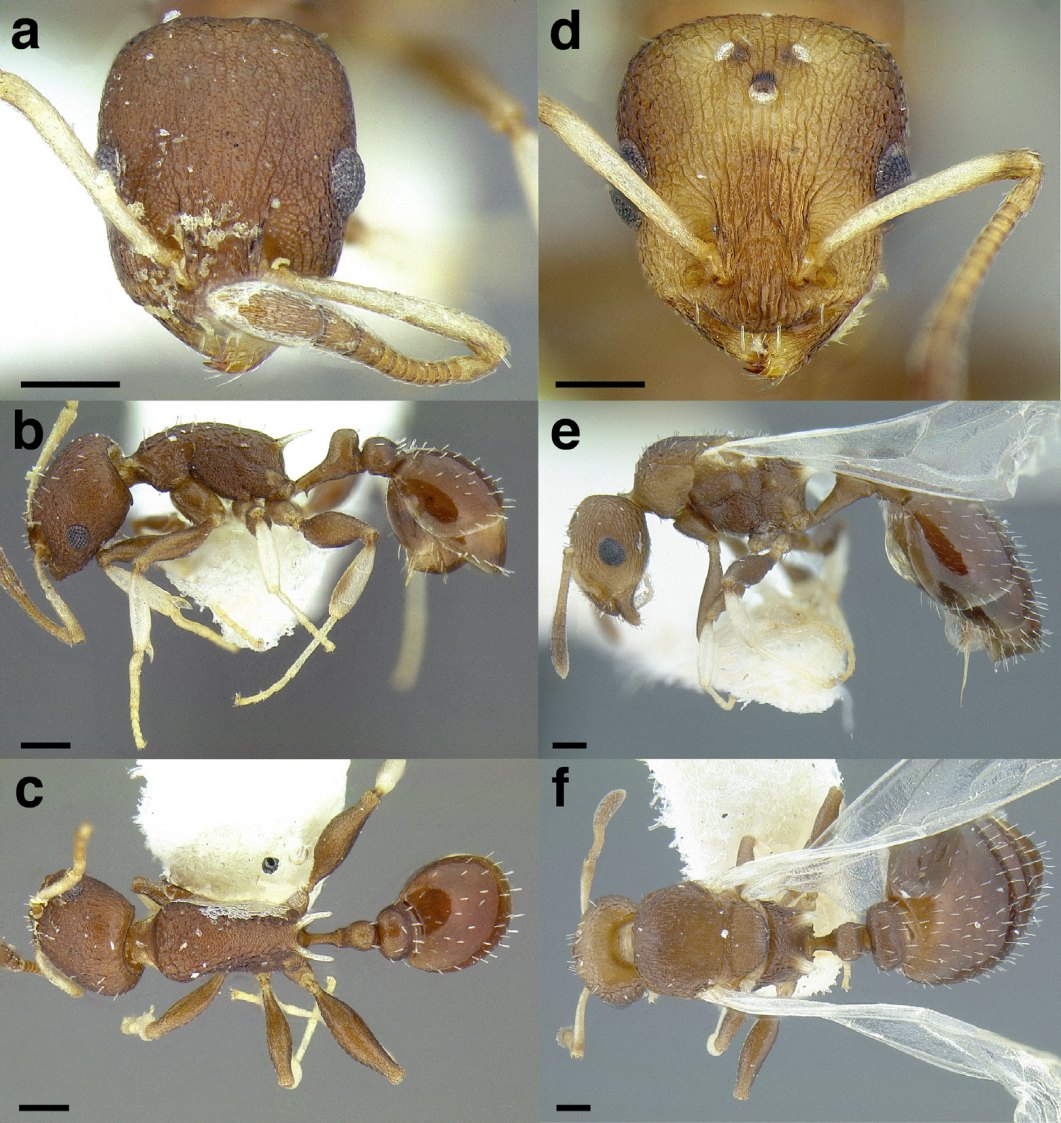
*Temnothorax leucacanthus*. (A–C) Holotype worker (MCZENT00032434). (A) Full-face view. (B) Profile view. (C) Dorsal view. (D–F) Paratype gyne (MCZENT00577109). (D) Full-face view. (E) Profile view. (F) Dorsal view. Scale bars 0.2 mm.

Distribution: Fig. 101D; worker & gyne: Fig. 105.

*Leptothorax leucacanthus* Baroni Urbani, 1978: 460, figs. 61, 103. Holotype worker & paratype gyne. Guerrero, Mexico.

*Temnothorax leucacanthus* (Baroni Urbani): Bolton, 2003: 271. First combination in *Temnothorax*.

**Type material examined:** *Holotype worker:* MEXICO: Guerrero: [locality unknown], [collection date unknown], W.V. Hagen, M.C.Z. Holotype 32434 (MCZENT00032434) [MCZC].

*Paratype workers and gyne:* same data as paratype, 1 worker (MCZENT00577108) [MCZC] 1 alate gyne (MCZENT577109) [MCZC] 1 worker (images of CASENT0912957 examined on antweb.org) [NHMB].

**Non-type material examined:** MEXICO: Guerrero: Acapulco, 7 September 1946, F. Bonet, 2 workers (LACMENT323192) [LACM].

**Geographic range:** Low elevations of southern Mexico (Guerrero) (Fig. 101D).

**Worker diagnosis:** The following character combination separates *Temnothorax leucacanthus* from all other species in the *salvini* clade: head relatively narrow (CI 80–81); moderately long antennal scapes, which just reach the posterior margin of the head (SI 96–103); mesosoma about one and a half times as long as the width of the head (WLI 143–148); propodeal spines about as long as the length of the propodeal declivity (PSI 38–40); petiolar node subquadrate, overhanging the caudal cylinder of the petiole; postpetiole moderately to very broad, more than two times the width of the petiole in dorsal view (PWI 204–224); erect setae on the dorsal surface of the head; integument medium brown.

**Similar species:** *Temnothorax acutispinosus* sp. nov., *Temnothorax subditivus, T. tenuisculptus, T. tuxtlanus* sp. nov., species of the *annexus*, *augusti*, *fuscatus*, and *salvini* groups. *Temnothorax leucacanthus* can be distinguished from the first three species by the shape of the dorsal margin of the mesosoma in profile, which is weakly, evenly convex: in *T. subditivus*, the mesosoma is strongly arched, while *T. tenuisculptus* and *T. tuxtlanus* sp. nov. have a sinuate profile. Petiolar node shape is also a useful character for separating *T. leucacanthus* from the above taxa: *T. subditivus* and the potentially co-occurring members of the *salvini* group have a squamiform petiolar node, which is much broader than the peduncle in dorsal view (only slightly wider in *T. leucacanthus*), and the nodes of the *annexus* group are erect and subquadrate, as opposed to leaning posteriorly in *T. leucacanthus*; *T. tenuisculptus*, like *T. leucacanthus*, has a subquadrate petiolar node, but it does not lean posteriorly over the caudal cylinder; *T. tuxtlanus* sp. nov. and *T. acutispinosus* sp. nov. have rounded petiolar nodes in profile view. *Temnothorax leucacanthus* can also be distinguished from *T. tuxtlanus* sp. nov. and *T. acutispinosus* sp. nov. by the presence of erect setae on the dorsum of the propodeum. The potentially co-occurring members of the *fuscatus* group have a low, elongate petiolar node in profile view. Finally, *T. leucacanthus* can be separated from fellow members of the *augusti* group by the combination of long propodeal spines, which are about as long as the length of the propodeal declivity (shorter than the declivity in *T. augusti* and *T. casanovai* sp. nov.), the relatively narrow head (broader in *T. leucacanthoides* sp. nov.: CI 80–81 vs. > 84); relatively short antennal scapes (SI 96–103 vs. > 105 in *T. augusti*, *T. aureus* sp. nov., *T. casanovai* sp. nov.).

**Worker measurements & indices (*n* = 4):** SL = 0.465–0.508 (0.489); FRS = 0.156–0.166 (0.161); CW = 0.505–0.571 (0.541); CWb = 0.453–0.524 (0.497); PoOC = 0.236–0.249 (0.244); CL = 0.564–0.645 (0.614); EL = 0.138–0.155 (0.145); EW = 0.096–0.119 (0.106); MD = 0.130–0.157 (0.147); WL = 0.646–0.775 (0.720); SPST = 0.248–0.312 (0.283); MPST = 0.214–0.248 (0.238); PEL = 0.270–0.318 (0.295); NOL = 0.146–0.166 (0.159); NOH = 0.123–0.146 (0.131); PEH = 0.197–0.226 (0.213); PPL = 0.140–0.178 (0.159); PPH = 0.160–0.183 (0.171); PW = 0.329–0.376 (0.360); SBPA = 0.126–0.152 (0.140); SPTI = 0.177–0.221 (0.201); PEW = 0.108–0.125 (0.118); PNW = 0.134–0.153 (0.144); PPW = 0.230–0.265 (0.252); HFL = 0.471–0.567 (0.519); HFWmax = 0.153–0.166 (0.160); HFWmin = 0.041–0.049 (0.045); CS = 0.735–0.847 (0.804); ES = 0.186–0.211 (0.198); SI = 96–103 (99); OI = 23–25 (25); CI = 80–81 (81); WLI = 143–148 (145); SBI = 27–30 (28); PSI = 38–40 (39); PWI = 204–224 (214); PLI = 168–209 (187); NI = 112–131 (122); PNWI = 111–131 (122); NLI = 52–56 (54); FI = 314–405 (357).

**Worker description:** In full-face view, head subquadrate, longer than broad (CI 80–81). Mandibles densely, finely striate but shining and armed with five teeth: the apical-most well developed and acute, followed by a less developed preapical tooth and three equally developed smaller teeth. Anterior clypeal margin evenly convex medially. Antennal scapes moderately long: when fully retracted, just reaching the posterior margin of the head capsule (SI 96–103). Antennae 12-segmented; antennal club of composed of three segments, with the apical-most segment longer than the preceding two in combination. Frontal carinae moderately long, extending past the antennal toruli by about two and a half times the maximum width of the antennal scape. Compound eyes moderately protruding past the lateral margins of the head capsule. Lateral margin of head weakly convex, forming a continuous arc from the mandibular insertions to the posterior margin of the head. Posterior head margin medially weakly emarginate, but predominantly flat, rounding evenly into the lateral margins.

In profile view, compound eyes ovular and moderately large (OI 23–25), with 11 ommatidia in longest row. Pronotal declivity distinct, neck and anterior face of pronotum forming a ~120° angle; anterior face and dorsal face meeting at a ~120° angle. Mesosoma evenly convex from where it joins the pronotal declivity to the propodeal spines. Promesonotal suture extending from the posterior margin of the procoxal insertion only to the mesothoracic spiracle, which is moderately well developed. Metanotal groove visible as a disruption of the sculpture laterally from where it arises between the mid- and hind coxae to where it ends in the poorly developed metathoracic spiracle, which is nearly indistinguishable against the ground sculpture. Propodeal spiracle moderately well developed, directed posterolaterally, and separated from the propodeal declivity by about three spiracle diameters. Propodeal spines well developed and moderately long (PSI 38–40), about as long as the propodeal declivity, tapering evenly from the base, straight, and acute. Propodeal declivity flat, forming a rounded ~100° angle with the base of the propodeal spines. Propodeal lobes rounded and weakly developed. Metapleural gland bulla moderately large, extending from the metacoxal insertion two thirds of the way to the propodeal spiracle. Petiole long (PLI 168–209), without tubercles anterodorsally. Subpetiolar process in the form of an acute tooth, ventral margin of petiole weakly bulging posteriorly. Petiolar peduncle long: comprising two thirds of the length of the petiole. Petiolar node robust and erect, nearly squamiform: transition between peduncle and node marked by a rounded angle of ~120°, resulting in a concave anterior node face; anterior face forming a ~100° angle with the dorsal face, which is weakly convex; dorsal face rounding evenly into the short posterior face, which forms a ~80° angle with the caudal cylinder, overhanging it. Postpetiole evenly rounded anteriorly, flattened dorsally, and weakly lobed ventrally.

In dorsal view, dorsal margin of pronotum delimited from the pronotal declivity by a weak carina. Humeri developed: evenly rounded and wider than the rest of the mesosoma; mesothoracic spiracles weakly protruding past the lateral margins of the mesosoma, visible as slight angles where the pronotum meets the mesonotum. Metanotal groove absent: mesonotum and propodeum completely fused and lateral margins converging evenly to the bases of the propodeal spines. Propodeal spines narrowly approximated basally, but diverging and becoming parallel apically, their apices separated from each other by less than their length, the negative space between them “U” shaped. Petiolar peduncle with spiracles protruding past the lateral margins; peduncle constricted anterior to them. Petiolar node campaniform: flattened posteriorly; node broader than the peduncle, and about the same width as the caudal cylinder. Postpetiole moderately to very broad (PWI 204–224) and campaniform, articulating with most of the anterior margin of the gaster, leaving small, angulate margins on each side exposed. Anterior margin of the postpetiole convex and evenly rounds into the lateral margins, which weakly diverge to the angulate posterior corners; posterior margin flat. Metafemur strongly incrassate (FI 314–405).

Sculpture: median clypeal carina present, extending posteriorly nearly to the frontal triangle, and flanked on either side by two equally strong carinae. Lateral clypeal lobes with additional, weaker carinae; ground sculpture weakly areolate. Antennal scapes areolate. Cephalic dorsum predominantly areolate, but with costulae overlying the ground sculpture. Lateral surfaces of head sculptured similarly to the dorsum of the head, but with rugulae between the compound eye and the mandibular insertion. Ventral surface of head weakly areolate. Mesosoma with areolate sculpture on the pronotal neck. Lateral surface of the pronotum very weakly areolate-costulate and shining. Lateral face of propodeum, meso- and metapleurae with stronger areolate sculpture; meso- and metapleurae with costulae over the ground sculpture. Propodeal declivity weakly areolate. Dorsal surface of pronotum with rugae over the areolate ground sculpture. Remainder of the mesosoma dorsum predominantly areolate, with weak costulae on the lateral margins. Femora weakly areolate. Petiole and postpetiole shining through weak areolate sculpture on all surfaces. First gastral tergite smooth and shining, with weak spectral iridescence. First gastral sternite smooth and shining, without spectral iridescence.

Setae: antennal scapes and funiculi with short, decumbent pilosity. Dorsum of the head, pronotum, waist segments, and gaster with moderately abundant, erect, clavate setae, the longest of which are about half the width of the compound eye. The head bears ~22, mesosoma ~22, petiole 2, postpetiole ~10, and first gastral tergite ~38 setae. Short, sparse pubescence present over the entire body, but difficult to detect against the ground sculpture.

Color: predominantly medium brown, with antennae (except for the club), tibiae, tarsi, propodeal spines, and sting light yellow.

**Gyne measurements & indices (*n* = 1):** SL = 0.555; FRS = 0.195; CW = 0.682; CWb = 0.646; PoOC = 0.273; CL = 0.720; EL = 0.210; EW = 0.164; MD = 0.152; WL = 1.105; SPST = 0.358; MPST = 0.326; PEL = 0.390; NOL = 0.218; NOH = 0.200; PEH = 0.276; PPL = 0.216; PPH = 0.267; PW = 0.697; SBPA = 0.343; SPTI = 0.305; PEW = 0.186; PNW = 0.233; PPW = 0.409; HFL = 0.648; HFWmax = 0.158; HFWmin = 0.049; CS = 1.006; ES = 0.292; SI = 86; OI = 29; CI = 90; WLI = 171; SBI = 53; PSI = 32; PWI = 220; PLI = 181; NI = 109; PNWI = 125; NLI = 56; FI = 322.

**Gyne description:** In full-face view, head subquadrate, slightly longer than broad (CI 90). Mandibles densely, finely striate but weakly shining and armed with five teeth: the apical-most well developed, followed by a less developed preapical tooth and three equally developed smaller teeth. Anterior clypeal margin evenly convex medially. Antennal scapes short: when fully retracted, failing to reach the posterior margin of the head capsule by about the maximum width of the antennal scape (SI 86). Antennae 12-segmented; antennal club composed of three segments, with the apical-most segment longer than the preceding two in combination. Frontal carinae moderately long, extending past the antennal toruli by about two and a half times the maximum width of the antennal scape. Compound eyes moderately protruding past the lateral margins of the head capsule. Lateral margin of head very weakly convex, nearly flat, diverging from the mandibular insertions to below the compound eyes. Posterior margin of head weakly convex, rounding evenly into the lateral margins.

In profile view, compound eyes ovular and large (OI 29), with 14 ommatidia in longest row. Mesoscutum rounded evenly anteriorly, not completely covering the dorsal surface of the pronotum, and flat dorsally. Mesoscutellum slightly depressed below the level of the mesoscutum. Posterior margin of metanotum extending slightly past the posterior margin of the mesoscutum. Propodeal spiracle moderately well developed, directed posterolaterally, and separated from the propodeal declivity by about four spiracle diameters. Propodeal spines stout and well developed, moderately long (PSI 32), about as long as the propodeal declivity, tapering evenly from the base, directed posteriorly, straight, and blunt. Propodeal declivity straight and flat, forming a rounded ~100° angle with the base of the propodeal spines. Propodeal lobes rounded and very weakly developed. Metapleural gland bulla small, extending from the metacoxal insertion halfway to the propodeal spiracle. Petiole long (PLI 181), without tubercles anterodorsally. Subpetiolar process in the form of a small, very acute tooth, which grades evenly into the ventral margin of the petiole posteriorly; ventral margin of petiole flat. Petiolar peduncle very long: comprising slightly more than half the length of the petiole. Petiolar node robust and erect, nearly squamiform: transition between peduncle and node marked by a rounded angle of ~130°, resulting in a concave anterior node face; anterior face forming a ~110° angle with the dorsal face, which is convex; dorsal face rounding evenly into the short posterior face, which forms a ~85° angle with the caudal cylinder, overhanging it. Postpetiole evenly rounded anterodorsally and flattened dorsally; ventral surface weakly lobed.

In dorsal view, mesoscutum leaving a small sliver of the anterior face of the pronotum exposed; humeri visible laterally as weakly angulate sclerites. Propodeal spines weakly diverging apically, their apices separated from each other by about twice their length. Petiolar peduncle with spiracles protruding past the lateral margins, the peduncle narrowed anterior to them. Petiolar node subovate, transversely elongated. Petiolar node wider than the peduncle, about one and a quarter times the width of the caudal cylinder. Postpetiole subquadrate and moderately broad (PWI 220), articulating with most of the anterior margin of the gaster, leaving small, angulate margins on each side exposed. Anterior margin of postpetiole weakly convex, with corners marked by rounded angles as it transitions to the lateral margins, which are parallel to the angulate posterior corners; posterior margin broadly concave. Metafemur strongly incrassate (FI 322).

Sculpture: median clypeal carina present but indistinct, flanked by three equally strong carinae on each side. Lateral clypeal lobes with additional weaker carinae; ground sculpture weakly areolate. Antennal scapes areolate. Cephalic dorsum areolate-rugulose, with rugose-costate sculpture overlying it; fine concentric costulae surrounding the antennal insertions. Lateral surfaces of head sculptured similarly to the dorsal surface, but with costae stronger. Ventral surface of head weakly transversely areolate-costulate. Pronotal neck areolate. Pronotum sculptured similarly to the lateral surface of the head. Lateral face of the propodeum, meso- and metapleurae longitudinally areolate-costulate, becoming weaker on the anterior half of the katepisternum and anterior quarter of the anepisternum. Propodeal declivity transversely areolate-strigulate. Mesoscutum and mesoscutellum with longitudinal striae over areolate ground sculpture. Femora shining through weak areolate sculpture. Petiole and postpetiole areolate, with weaker sculpture on the peduncle and ventral surface of the petiole. First gastral tergite very weakly areolate-costulate on the basal quarter, with faint traces of spectral iridescence. Surface of the first gastral sternite smooth and shining, with weak spectral iridescence.

Setae: antennal scapes and funiculi with short, decumbent pilosity. Dorsum of the head, pronotum, waist segments, and gaster with moderately abundant, erect, clavate setae, the longest of which are about a third of the width of the compound eye. Short, sparse pubescence present over the entire body, but difficult to detect against the ground sculpture.

Color: predominantly medium brown, with antennae (except for the club), tibiae, tarsi, wing bases, propodeal spines, and sting light yellow.

**Male:** Unknown.

**Etymology:** Morphological, presumably for the light color of the propodeal spines.

**Comments:** *Temnothorax leucacanthus* is known only from a couple of collections in Guerrero state, Mexico. Nothing is known about the biology of this species, but it closely resembles *T. leucacanthoides* sp. nov. from neighboring Oaxaca state, Mexico, which was collected from the dead stalk of a plant of the family Asteraceae in littoral vegetation.

### *casanovai* group overview

**Figure 106:**
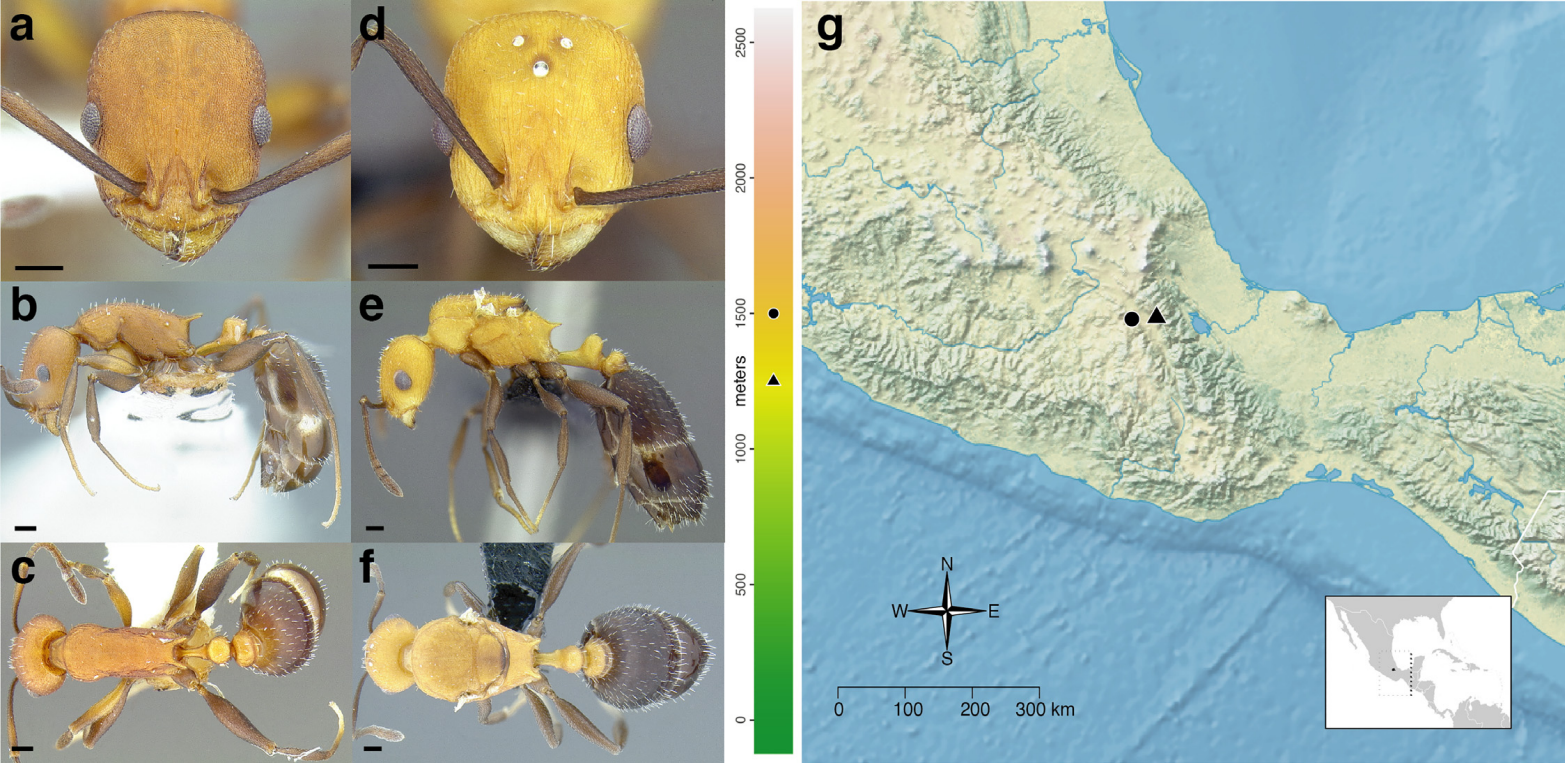
*Temnothorax casanovai* sp. nov. (A–C) Holotype worker (LACMENT323462). (A) Full-face view. (B) Profile view. (C) Dorsal view. (D–F) Paratype gyne (LACMENT323464). (D) Full-face view. (E) Profile view. (F) Dorsal view. (G) Geographical and elevational distribution of specimens examined. Type localities are represented by triangles, non-type localities are represented by circles. Bounding box in inset map shows location of main map. Scale bars 0.2 mm.

This group is monotypic, with the nominal *Temnothorax casanovai* sp. nov. being the only member. With only a couple of collections from mid elevation southern Mexico (Fig. 106G), this species is morphologically similar to the members of the *augusti* group, in that it has a subquadrate petiolar node that overhangs the caudal cylinder. It can be separated from them by the overall larger size of *T. casanovai* sp. nov., the shorter propodeal spines, smaller metapleural gland bulla, and longer antennal scapes. Despite the morphological similarities to the *augusti* group, this species is more closely related to *T. subditivus* (Prebus, in prep.).

***Temnothorax casanovai* sp. nov.**

Distribution, worker & gyne: Fig. 106.

**Type material examined:** *Holotype worker:* MEXICO: Puebla: San Rafael Coxcatlán, 1,250 m 18.350000°N 97.116667°W, April 2000, L. Rios Casanova, pitfall trap ZIII T7, (LACMENT323462) [LACM].

*Paratype workers:* MEXICO: Puebla: same data as holotype, 1 worker (LACMENT323463) [LACM]; same data as previous, except: July 2000, pitfall trap UII T16, 1 worker (LACMENT323460) [MCZC] 1 worker (LACMENT323461) [CASC].

**Non-type material examined:** MEXICO: Puebla: Zapotitlán de las Salinas, Jardín Botánico, 23 August 2003, L. Rios Casanova, LRC#2003-17, deteriorated terrace, in branch of Pitahaya, 1 alate gyne, 2 workers (LACMENT323464) [LACM] 3 workers (LACMENT323465) [LACM].

**Geographic range:** Mid elevations of south-central Mexico (Puebla) (Fig. 106G).

**Worker diagnosis:** The following character combination separates *Temnothorax casanovai* sp. nov. from all other species in the *salvini* clade: very long antennal scapes, which surpass the posterior margin of the head by about two times the maximum width of the scape (SI 108–121); mesosoma about one and a half times as long as the width of the head (WLI 151–161); propodeal spines short, about half as long as the propodeal declivity (PSI 28–32); metapleural gland bulla small, extending from the metacoxal insertion a third way to the propodeal spiracle; petiolar peduncle long, with the peduncle comprising a little more than half the length of the petiole; petiolar node subquadrate and erect, not overhanging the caudal cylinder of the petiole; postpetiole narrow to moderately broad, about two times the width of the petiole in dorsal view (PWI 181–203); first gastral tergite strongly areolate and dull, with abundant suberect setae; erect setae on the dorsal surface of the head; bicolored integument, testaceous with brown appendages and gaster.

**Similar species:** *Temnothorax subditivus*, *T. tenuisculptus*, and species of the *annexus* and *augusti* groups. *Temnothorax casanovai* sp. nov. can be separated from *T. augusti* and *T. aureus* sp. nov. by the petiolar node, which leans posteriorly in *T. augusti* and *T. aureus* sp. nov., overhanging the caudal cylinder. Furthermore, the first gastral tergite is weakly sculptured in *T. augusti* and is smaller than *T. casanovai* sp. nov.: WL < 0.9 mm. *Temnothorax casanovai* sp. nov. can be separated from *T. leucacanthus, T. leucacanthoides* sp. nov., *and T. aureus* sp. nov. by their longer propodeal spines, which are about the length of the propodeal declivity (PSI > 35) and their larger metapleural gland bullae, which extend from the metacoxal insertion more than halfway to the propodeal spiracle. The antennal scapes of *T. leucacanthus* and *T. leucacanthoides* sp. nov. are shorter than *T. casanovai* sp. nov., failing to surpass the posterior margin of the head (SI < 110), and also have smooth first gastral tergites. *Temnothorax casanovai* sp. nov. differs from *T. tenuisculptus* by the propodeal spines, which are about the length of the propodeal declivity in *T. tenuisculptus*, and are directed upward, forming a higher angle with the propodeal declivity; additionally, *T. tenuisculptus* has a shorter petiolar peduncle, comprising about a third of the total petiole length, and a depressed propodeum. *Temnothorax casanovai* sp. nov. can be separated from *T. subditivus* by the petiolar node in dorsal view, which is much broader than the peduncle in *T. subditivus*, as well as the first gastral tergite, which is smooth and shining in *T. subditivus. Temnothorax casanovai* sp. nov. can be separated from species in the *annexus* group by the pedunculate petiole, which always comprises less than half the total petiole length in the *annexus* group.

**Worker measurements & indices (*n* = 9):** SL = 0.668–0.830 (0.778); FRS = 0.199–0.248 (0.221); CW = 0.677–0.807 (0.737); CWb = 0.619–0.752 (0.677); PoOC = 0.317–0.390 (0.350); CL = 0.776–0.933 (0.855); EL = 0.168–0.197 (0.183); EW = 0.133–0.150 (0.140); MD = 0.178–0.230 (0.205); WL = 0.947–1.199 (1.053); SPST = 0.263–0.341 (0.311); MPST = 0.295–0.373 (0.335); PEL = 0.337–0.483 (0.408); NOL = 0.197–0.264 (0.238); NOH = 0.141–0.184 (0.158); PEH = 0.239–0.308 (0.270); PPL = 0.143–0.230 (0.180); PPH = 0.179–0.274 (0.222); PW = 0.439–0.559 (0.492); SBPA = 0.158–0.233 (0.183); SPTI = 0.180–0.293 (0.238); PEW = 0.138–0.192 (0.160); PNW = 0.199–0.281 (0.233); PPW = 0.269–0.362 (0.306); HFL = 0.761–0.944 (0.868); HFWmax = 0.176–0.218 (0.204); HFWmin = 0.053–0.065 (0.06); CS = 1.007–1.219 (1.104); ES = 0.236–0.270 (0.252); SI = 108–121 (115); OI = 22–24 (23); CI = 76–81 (79); WLI = 151–161 (156); SBI = 26–31 (27); PSI = 28–32 (30); PWI = 181–203 (191); PLI = 203–258 (229); NI = 132–186 (151); PNWI = 135–154 (145); NLI = 53–65 (58); FI = 317–363 (338).

**Worker description:** In full-face view, head subquadrate, elongate (CI 76–81). Mandibles densely, finely striate but weakly shining and armed with five teeth: the apical-most well developed and acute, followed by a less developed preapical tooth and three equally developed smaller teeth. Anterior clypeal margin emarginate medially. Antennal scapes very long: when fully retracted, surpassing the posterior margin of the head capsule by about two times the maximum width of the scape (SI 108–121). Antennae 12-segmented; antennal club of composed of three segments, with the apical-most segment as long as the preceding two in combination. Frontal carinae short, extending past the antennal toruli by about one and a half times the maximum width of the antennal scape. Compound eyes moderately protruding past the lateral margins of the head capsule. Lateral margin of head weakly convex, forming a continuous arc from the mandibular insertions to the posterior margin of the head. Posterior head margin evenly convex, rounding evenly into the lateral margins.

In profile view, compound eyes ovular and moderately large (OI 22–24), with 17 ommatidia in longest row. Pronotal declivity indistinct, but neck and anterior face of pronotum forming a ~120° angle; anterior face evenly rounding into the dorsal face. Mesosoma evenly, but weakly convex from where it joins the pronotal neck to the base of the propodeal spines. Promesonotal suture extending from the posterior margin of the procoxal insertion only to the mesothoracic spiracle, which is moderately well developed. Metanotal groove visible as a disruption of the sculpture laterally from where it arises between the mid- and hind coxae to where it ends in the poorly developed metathoracic spiracle, which is nearly indistinguishable against the ground sculpture. Propodeal spiracle weakly developed, directed posterolaterally, and separated from the propodeal declivity by about five spiracle diameters. Propodeal spines moderately well developed, but short (PSI 28–32), two thirds the length of the propodeal declivity, tapering evenly from the base, straight, and blunt. Propodeal declivity flat, forming a rounded ~100° angle with the base of the propodeal spines. Propodeal lobes rounded and weakly developed. Metapleural gland bulla small, extending from the metacoxal insertion a third way to the propodeal spiracle. Petiole long (PLI 203–258), without tubercles anterodorsally. Subpetiolar process in the form of small triangular tooth, ventral margin of petiole flat. Petiolar peduncle long: comprising a little more than half the length of the petiole. Petiolar node robust and erect, subquadrate: transition between peduncle and node marked by a rounded angle of ~120°, resulting in a concave anterior node face; anterior face meeting the dorsal face at a ~90° angle; dorsal face weakly convex, nearly flat, meeting the posterior face at a rounded angle of ~90°; posterior face forming a ~90° angle with the caudal cylinder. Postpetiole subquadrate and antero-posteriorly compressed, anterior face flat, bulging slightly anterodorsally before transitioning into the flattened posterodorsal face; weakly lobed ventrally.

In dorsal view, dorsal margin of pronotum weakly delimited from the pronotal declivity by a very weak carina. Humeri developed: evenly rounded and wider than the rest of the mesosoma; mesothoracic spiracles very weakly protruding past the lateral margins of the mesosoma, visible as slight angles where the pronotum meets the mesonotum. Promesonotal suture barely visible as a slight disruption in the ground sculpture. Metanotal groove absent: mesonotum and propodeum completely fused and lateral margins converging evenly to the bases of the propodeal spines. Propodeal spines narrowly approximated basally, their bases joined by a rounded transverse welt, diverging weakly apically, their apices separated from each other by about their length, the negative space between them “U” shaped. Petiolar peduncle with spiracles slightly protruding past the lateral margins; peduncle weakly constricted anterior to them. Petiolar node weakly campaniform: weakly convex posteriorly, more strongly convex anteriorly; node broader than the peduncle, and about one and half times as wide as the caudal cylinder. Postpetiole subquadrate and narrow to moderately broad (PWI 181–203), articulating with most of the anterior margin of the gaster, leaving small, angulate margins on each side exposed. Anterior margin of the postpetiole flat, evenly rounding into the lateral margins, which diverge very slightly to the angulate posterior corners; posterior margin broadly concave. Metafemur strongly incrassate (FI 317–363).

Sculpture: clypeus with eight parallel longitudinal carinae. Lateral clypeal lobes with additional, weaker carinae; ground sculpture areolate. Antennal scapes areolate. Cephalic dorsum areolate, but with very fine costulae overlying the ground sculpture; fine concentric costulae surrounding the antennal insertions. Lateral surfaces of head sculptured similarly to the dorsum of the head, but with rugulae becoming stronger and forming cross reticulations between the compound eye and mandibular insertions. Ventral surface of head shining through weak areolate-costulate sculpture. Mesosoma with areolate sculpture on the pronotal neck. Lateral surface of the mesosoma strongly areolate, with costulate sculpture on the pronotum the border of the meso- and metapleurae. Propodeal declivity weakly areolate. Dorsal surface of mesosoma areolate, with costulae overlying the ground sculpture. Femora areolate. Petiole and postpetiole predominantly weakly areolate, but sculpture is weaker on the ventral surface of the petiolar peduncle. First gastral tergite strongly areolate and dull, with spectral iridescence. First gastral sternite smooth and shining, with spectral iridescence.

Setae: antennal scapes and funiculi with short, decumbent pilosity. Dorsum of the head, pronotum, waist segments, and gaster with moderately abundant, erect, blunt-tipped, nearly clavate setae, the longest of which are about half the width of the compound eye. The head bears ~32, mesosoma ~48, petiole 10, postpetiole ~20, and first gastral tergite ~94 setae. Short, sparse pubescence present over the entire body, but difficult to detect against the ground sculpture and light integument.

Color: predominantly testaceous, with antennae, protibiae and protarsi, mid legs, hind legs, and gaster medium brown.

**Gyne measurements & indices (*n* = 1):** SL = 0.806; FRS = 0.263; CW = 0.869; CWb = 0.788; PoOC = 0.355; CL = 0.897; EL = 0.244; EW = 0.187; MD = 0.200; WL = 1.406; SPST = 0.324; MPST = 0.391; PEL = 0.464; NOL = 0.274; NOH = 0.209; PEH = 0.375; PPL = 0.212; PPH = 0.312; PW = 0.939; SBPA = 0.382; SPTI = 0.414; PEW = 0.221; PNW = 0.297; PPW = 0.422; HFL = 0.939; HFWmax = 0.204; HFWmin = 0.064; CS = 1.237; ES = 0.338; SI = 102; OI = 27; CI = 88; WLI = 178; SBI = 48; PSI = 23; PWI = 191; PLI = 219; NI = 131; PNWI = 134; NLI = 59; FI = 319.

**Gyne description:** In full-face view, head subquadrate, longer than broad (CI 88). Mandibles densely, finely striate but weakly shining and armed with five teeth: the apical-most well developed, followed by a less developed preapical tooth and three equally developed smaller teeth. Anterior clypeal margin emarginate medially. Antennal scapes very long: when fully retracted, surpassing the posterior margin of the head capsule by about one and a half times the maximum width of the antennal scape (SI 102). Antennae 12-segmented; antennal club composed of three segments, with the apical-most segment as long as the preceding two in combination. Frontal carinae moderately long, extending past the antennal toruli by about two times the maximum width of the antennal scape. Compound eyes moderately protruding past the lateral margins of the head capsule. Lateral margin of head weakly convex, diverging from the mandibular insertions to below the compound eyes. Posterior margin of head convex, rounding evenly into the lateral margins.

In profile view, compound eyes ovular and large (OI 27), with 20 ommatidia in longest row. Mesoscutum rounded evenly anteriorly, covering the dorsal surface of the pronotum, and flat dorsally. Mesoscutellum on the same level as the mesoscutum. Posterior margin of metanotum extending slightly past the posterior margin of the mesoscutum. Propodeal spiracle moderately well developed, directed posterolaterally, and separated from the propodeal declivity by about five spiracle diameters. Propodeal spines stout and moderately well developed, but short (PSI 23), about half as long as the propodeal declivity, tapering evenly from the base, directed posteriorly, straight, and blunt. Propodeal declivity straight and flat, forming a rounded ~100° angle with the base of the propodeal spines. Propodeal lobes rounded and very weakly developed. Metapleural gland bulla small, extending from the metacoxal insertion halfway to the propodeal spiracle. Petiole long (PLI 219), without tubercles anterodorsally. Subpetiolar process in the form of a very small, triangular, blunt tooth, which grades evenly into the ventral margin of the petiole posteriorly; ventral margin of petiole weakly bulging medially. Petiolar peduncle long: comprising slightly more than half the length of the petiole. Petiolar node robust and erect, nearly squamiform: transition between peduncle and node marked by a rounded angle of ~120°, resulting in a concave anterior node face; anterior face rounding evenly into the dorsal face, which is convex; dorsal face rounding evenly into posterior face, which forms a ~90° angle with the caudal cylinder. Postpetiole flat anteriorly, bulging anterodorsally before flattening posterodorsally; ventral surface weakly lobed.

In dorsal view, mesoscutum leaving a small sliver of the anterior face of the pronotum exposed; humeri visible laterally as rounded sclerites. Propodeal spines strongly diverging apically, their apices separated from each other by about two and a half times their length. Petiolar peduncle with spiracles protruding past the lateral margins, the peduncle slightly narrowed anterior to them. Petiolar node campaniform: anterior face strongly convex; posterior face very weakly convex, nearly flat. Petiolar node wider than the peduncle, about one and a quarter times the width of the caudal cylinder. Postpetiole subquadrate and narrow (PWI 191), articulating with most of the anterior margin of the gaster, leaving small, angulate margins on each side exposed. Anterior margin of postpetiole flat, with corners marked by rounded angles as it transitions to the lateral margins, which weakly diverge to the angulate posterior corners; posterior margin flat. Metafemur strongly incrassate (FI 319).

Sculpture: median clypeal carina present but indistinct, flanked by three slightly weaker carinae on each side. Lateral clypeal lobes with additional weaker carinae; ground sculpture weakly areolate. Antennal scapes areolate. Cephalic dorsum areolate, but with fine costae overlying the ground sculpture; fine concentric costulae surrounding the antennal insertions. Lateral surfaces of head sculptured similarly to the dorsum of the head. Ventral surface of head weakly areolate-costulate. Mesosoma with areolate sculpture on the pronotal neck. Lateral surface of the mesosoma strongly areolate, with rugulose-costulate sculpture on the pronotum the border of the meso- and metapleurae. Propodeal declivity weakly areolate, with fine costulae. Mesoscutum and mesoscutellum with costulae over areolate ground sculpture. Femora areolate. Petiole and postpetiole weakly areolate, becoming weaker on the dorsum of the peduncle. First gastral tergite strongly areolate, with spectral iridescence. First gastral sternite smooth and shining, with spectral iridescence.

Setae: antennal scapes and funiculi with short, decumbent pilosity. Dorsum of the head, pronotum, waist segments, and gaster with moderately abundant, erect, blunt-tipped, nearly clavate setae, the longest of which are about a third of the width of the compound eye. Short, sparse pubescence present over the entire body, but difficult to detect against the ground sculpture and light integument.

Color: predominantly testaceous, with antennae, metanotum, protibiae and protarsi, mid legs, hind legs, and gaster medium brown.

**Male:** unknown.

**Etymology:** Matronym, for the collector of the type series, Dr. Leticia Ríos Casanova.

**Comments:** *Temnothorax casanovai* sp. nov. is known from a couple of mid elevation collections in Puebla state, Mexico. Workers were taken from pitfall traps, and a nest was collected from the branch of a pitahaya cactus, *Hylocereus undatus*, a hemiepiphytic species commonly cultivated for its fruit.

### *fuscatus* group overview

**Figure 107:**
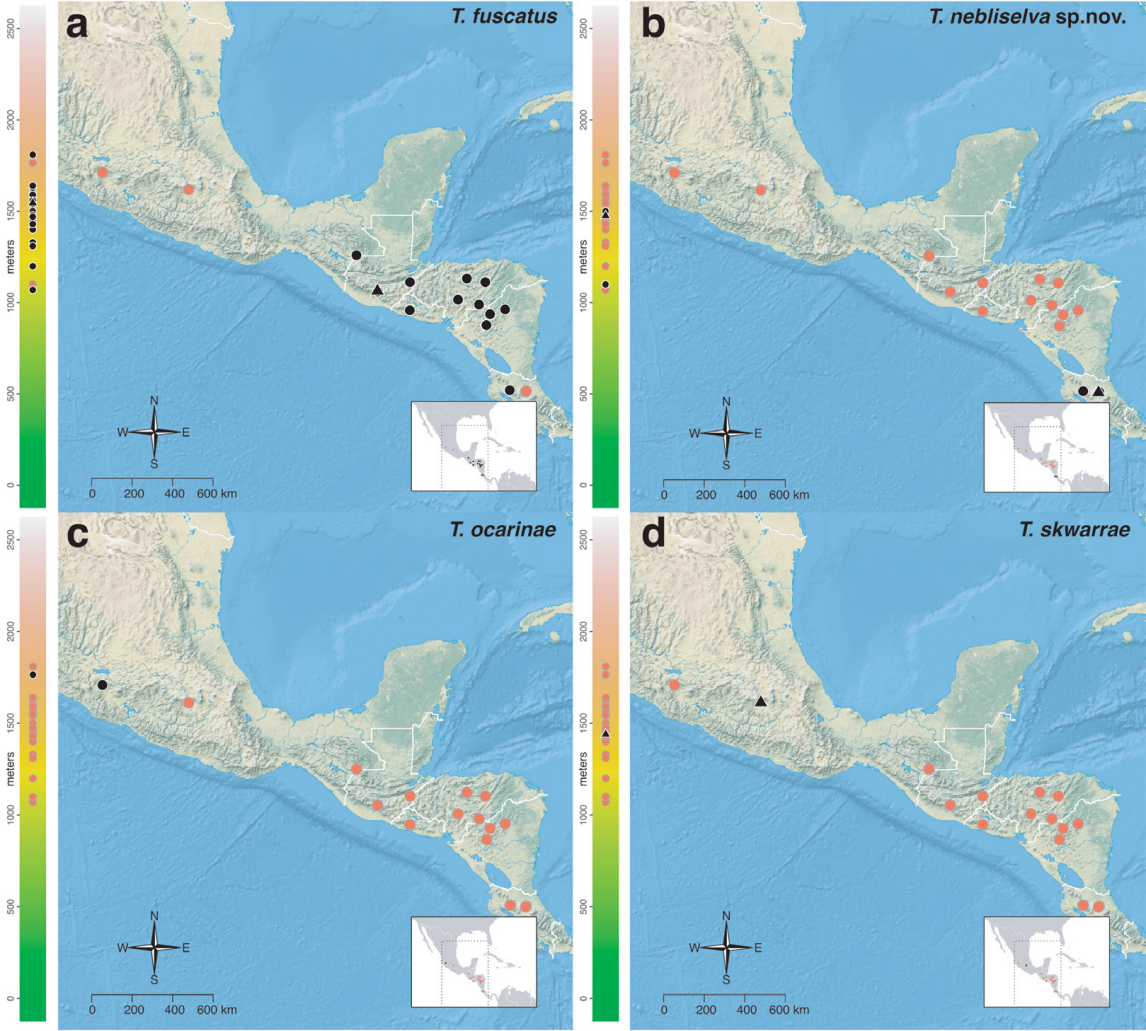
Geographical and elevational distribution of the *fuscatus* group. (A) *Temnothorax fuscatus* (B) *T. nebliselva* sp. nov. (C) *T. ocarinae* (D) *T. skwarrae*. Colored scale to the left of each map represents elevation in meters. Points in black represent the species named in each subfigure, while points in red represent other members of the species group. Type localities are represented by triangles, non-type localities are represented by circles. Bounding box in inset map shows location of main map.

With four species (one described as new here), the *fuscatus* group is another small group with a range spanning the mid-to-high elevations of central Mexico to southern Central America (Fig. 107). All nest collections of *fuscatus* group species have been from arboreal habitats, either nesting in vegetation (alive or dead) on live trees or in epiphytes. The low, long petiolar node distinguishes this group from nearly everything else in the *salvini* clade. Apparently, the *fuscatus* group is sister to the ground-nesting *pergandei* group (Prebus, in prep.).

***Temnothorax fuscatus* (Mann, 1920)**

**Figure 108:**
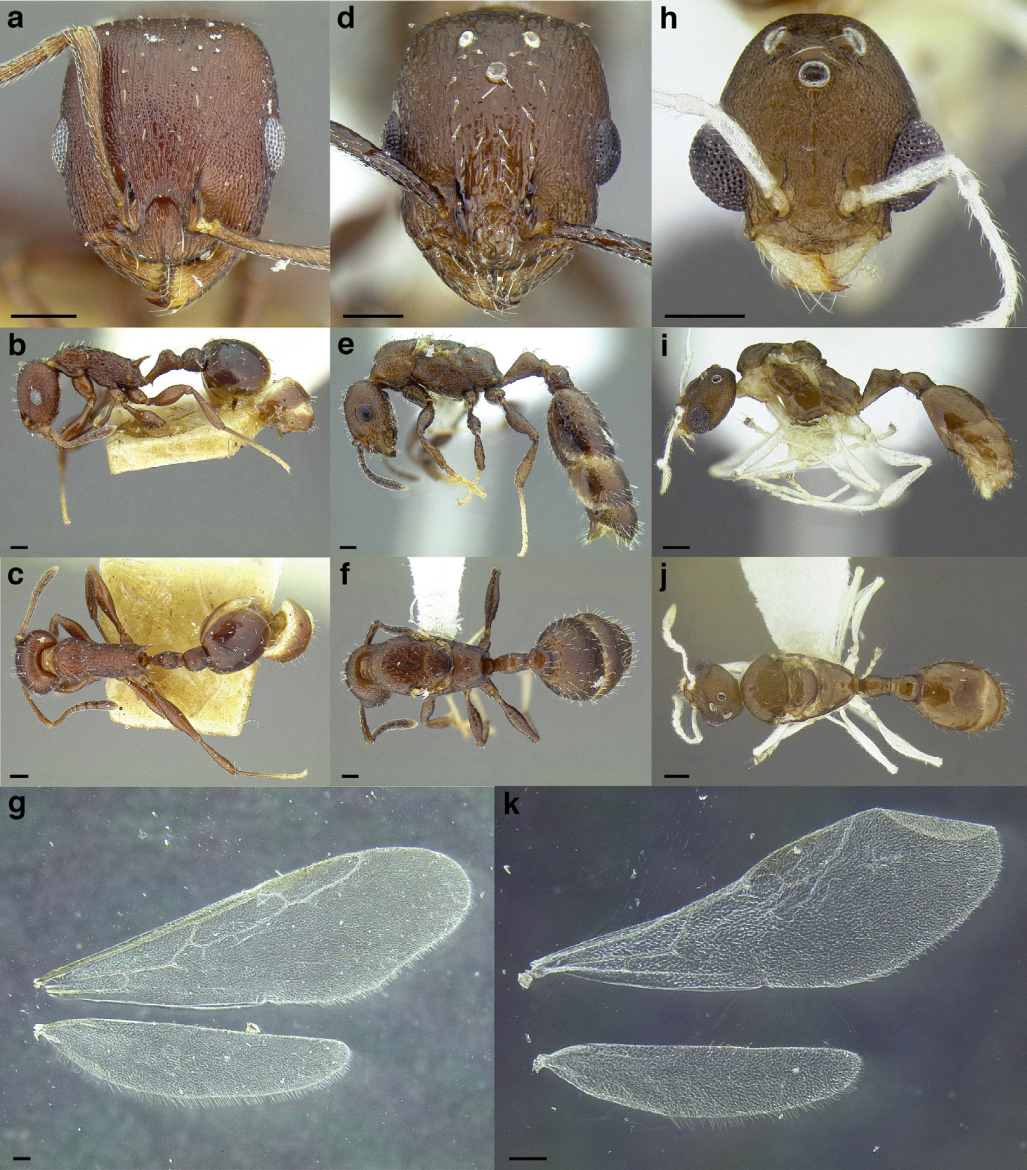
*Temnothorax fuscatus*. (A–C) Paralectotype worker (USNMENT00921897). (A) Full-face view. (B) Profile view. (C) Dorsal view. (D–G) gyne (CASENT0615617). (D) Full-face view. (E) Profile view. (F) Dorsal view. (G) Wings. (H–K) Male (CASENT0625205). (H) Full-face view. (I) Profile view. (J) Dorsal view. (K) Wings. Scale bars 0.2 mm.

**Figure 109:**
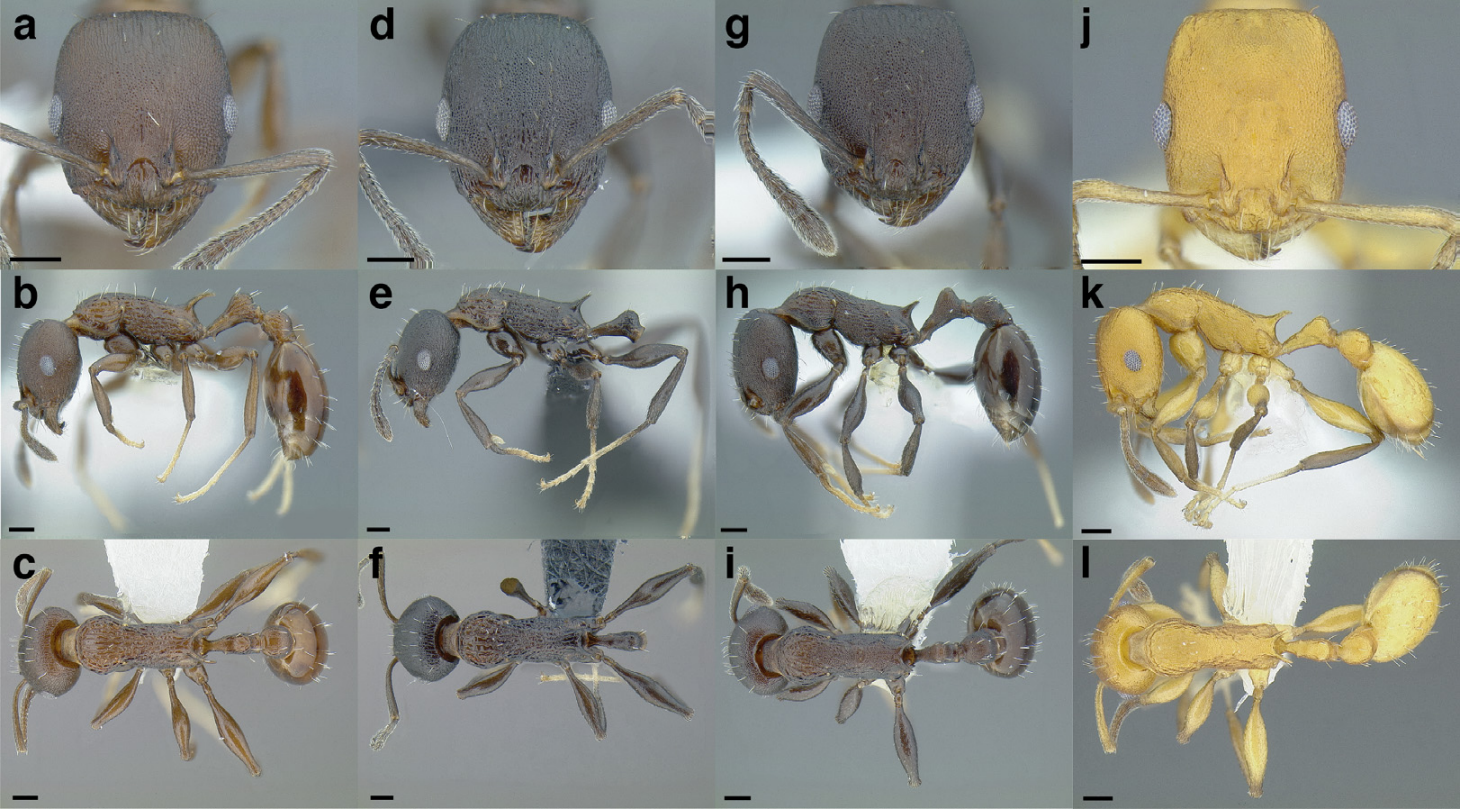
Comparison of *Temnothorax fuscatus* worker morphology. (A–C) Chiapas, Mexico (CASENT0916005). (A) Full-face view. (B) Profile view. (C) Dorsal view. (D–F) Zacapa, Guatemala (CASENT0614497). (D) Full-face view. (E) Profile view. (F) Dorsal view. (G-I) Olancho, Honduras (CASENT0625205). (G) Full-face view. (H) Profile view. (I) Dorsal view. (J–L) Jinotega, Nicaragua (CASENT0629158). (J) Full-face view. (K) Profile view. (L) Dorsal view. Scale bars 0.2 mm.

**Figure 110:**
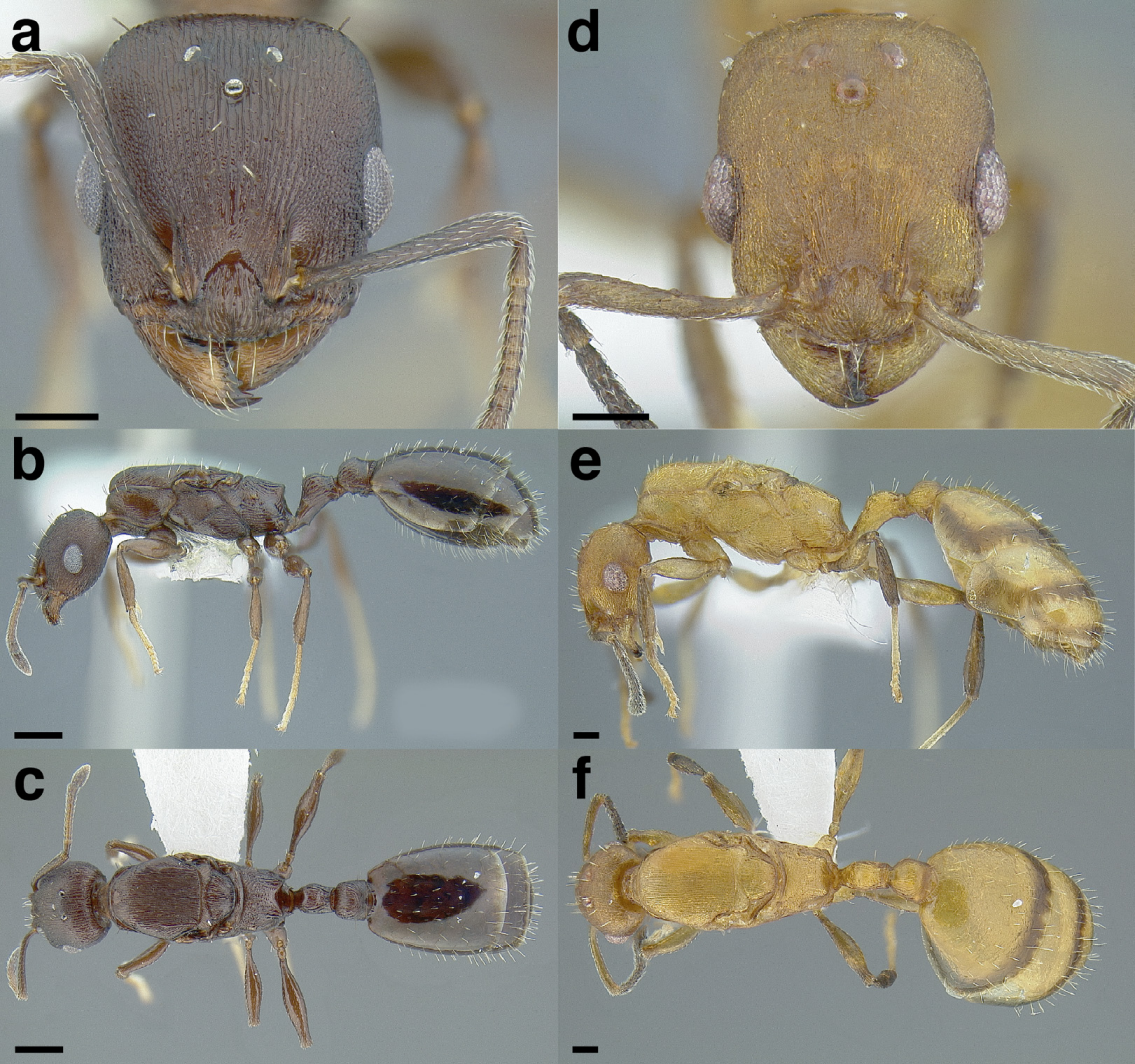
Comparison of *Temnothorax fuscatus* gyne morphology. (A–C) Chiapas, Mexico (CASENT0916005). (A) Full-face view. (B) Profile view. (C) Dorsal view. (D–F) Jinotega, Nicaragua (CASENT0629025). (D) Full-face view. (E) Profile view. (F) Dorsal view. Scale bars 0.2 mm.

Distribution: Fig. 107A; worker, gyne & male: Fig. 108; worker variability: Fig. 109; gyne variability: Fig. 110.

*Macromischa (Macromischa) fuscata* Mann, 1920: 420. Syntype workers. Antigua, Guatemala. One syntype here designated **lectotype**.

*Leptothorax fuscatus* (Mann): Baroni Urbani, 1978: 442. First combination in *Leptothorax*.

*Temnothorax fuscatus* (Mann): Bolton, 2003: 271. First combination in *Temnothorax*.

*Temnothorax* cf. *fuscatus* Prebus, 2017: 7. In phylogeny.

**Type material examined:** *Lectotype worker*: GUATEMALA: Sacatepéquez: Antigua, 21 December 1911, Wm. M. Wheeler, 1 worker (USNMENT00529240) [USNM].

*Paralectotype workers*: same data as lectotype: 3 workers, top worker with head missing (USNMENT00921897) [USNM].

**Non-type material examined:** COSTA RICA: Puntarenas: Monteverde, 1,190 m, 10.3°N 84.81667°W, 17 May 1989, J. Longino#2541, 1 worker (INBIOCRI001281867) [UCDC].

EL SALVADOR: La Libertad: Boquerón to Quetzaltepeque, 1,600 m, 24–27 January 1973, W.L. Brown, ravine cafetal, pines, 1 worker (MCZENT00581862) [MCZC].

GUATEMALA: [intercepted in quarantine, San Francisco, California, U.S.A.] 1 April 1944, ex Orchidaceae, 1 worker (LACMENT323190) [LACM]. Sacatepéquez: 5 km SE Antigua, 14.53990°N 90.70807°W ± 57 m, 1,810 m, 12 June 2009, LLAMA#Go-B-08-4-03, hardwood forest, beating vegetation, 1 worker (CASENT0610690) [UCDC]; same data as previous, except: LLAMA#Go-B-08-4-04, hardwood forest, beating vegetation, 2 workers (CASENT0610696, CASENT0610697) [JTLC]. Zacapa: 2 km SE La Unión, 14.94378°N 89.27747°W ± 104 m, 1,540 m, 13 May 2009, LLAMA#Go-B-03-1-01, cloud forest, beating vegetation, 1 worker (CASENT0614497) [UCDC].

HONDURAS: Francisco Morazán: Tegucigalpa vicinity, Jutiapa (near Parque Nacional La Tigra) 1,585 m, 23 May 1993, J. Rifkind, P. Gum, collectors, 1 dealate gyne (LACMENT323457) [LACM]. Olancho: 10 km N Catamacas, 14.94204°N 85.90979°W ± 50 m, 1,590 m, 9 May 2010, LLAMA#Go-C-02-1-03, cloud forest, beating vegetation, 1 worker (CASENT0614147) [UCDC]; same data as previous, except: 14.94116°N 85.90875°W ± 50 m, 1,560 m, 9 May 2010, LLAMA#Wm-C-02-1-05, cloud forest, ex sifted leaf litter, 1 worker (CASENT0616258) [JTLC]; 9 km NNW La Unión, 15.09658°N 86.74526°W ± 100 m, 1,50 m, 5 May 2010, J. Longino #JTL7004, pasture/pine forest edge, forager on vegetation, 1 worker (CASENT0615616) [JTLC]; same data as previous, except: 15.09691°N 86.74589°W ± 15 m, 1,450 m, 5 May 2010, J. Longino #JTL7005, pasture/pine forest edge, ex dead stem, 1 alate gyne (CASENT0615617) [UCDC] 2 workers, 1 alate gyne (CASENT0615618-CASENT0615620) [JTLC]; Parque Nacional La Muralla: 15.09957°N 86.73283°W ± 7 m, 1,640 m, 5 May 2010, P.S. Ward#16363, montane rainforest, ex dead twig (vine), 1 dealate gyne, 1 worker & 1 male (CASENT0625205) [JTLC] 1 dealate gyne & 1 worker (CASENT0758794) [UCDC] 2 dealate gynes & 1 worker (CASENT0758656) [PSWC] 2 workers & 1 male (CASENT0915981) [PSWC]; same data as previous, except: 15.09710°N 86.73904°W ± 100 m, 1,480 m, LLAMA#Go-C-01-1-01, cloud forest edge, beating vegetation, 1 worker (JTL288113) [JTLC]; same data as previous, except: 15.09685°N 86.7329°W ± 100 m, 1,630 m, LLAMA#Go-C-01-3-03, beating vegetation, 1 worker (CASENT0732588) [UCDC] 2 workers (JTL288179, JTL688876) [JTLC]; same data as previous, except: 15.09667°N 86.73800°W ± 100 m, 1,470 m, C. Weirauch, J. Mottern, M. Forthman#Weirauch-H13L41, yellow pan trap, 1 worker (JTL709204) [JTLC].

MEXICO: Chiapas: Tziscao, Lagos de Montebello 16.083333°N 91.683333°W, 1,500 m, 21 December 1991, P.S. Ward#11561, mixed tropical/temperate mesic forest, ex dead culm of sedge, 1 dealate gyne & 2 workers (CASENT0916005) [PSWC]; same data as previous, except: P.S. Ward#11559, mixed tropical/temperate mesic forest, ex dead twig, 1 dealate gyne (CASENT0758655) [UCDC].

NICARAGUA: Jinotega: Reserva Natural Datanlí El Diablo: 13.09908°N 85.86945°W ± 60 m, 1,330 m, 20 May 2011, LLAMA#Ba-D-04-2-06-01, at cookie bait, 1 worker (CASENT0732587) [UCDC] 1 worker (JTL682334) [JTLC]; 13.100368°N 85.86846°W ± 100 m, 1,330 m, 20 May 2011, LLAMA#Go-D-04-2-03, cloud forest, beating vegetation, 1 worker (CASENT0629157) [JTLC] 1 worker (CASENT0629158) [JTLC]; 13.09554°N 85.85844°W ± 100 m, 1,310 m, 20 May 2011, LLAMA#Go-D-04-3-02, montane wet forest, beating vegetation, 1 worker (CASENT0629161) [JTLC]; Reserva Natural Cerro Kilambé: 13.56751°N 85.69672°W ± 100 m, 1,430 m, 25 May 2011, LLAMA#Go-D-05-4-02, cloud forest, beating vegetation, 1 worker (CASENT0629262) [UCDC]; 13.56724°N 85.69775°W ± 150 m, 1,400 m, 23 May 2011, LLAMA#Wm-D-05-2-01, cloud forest, ex sifted leaf litter, 1 worker (CASENT0629307) [JTLC]; Parque Nacional Cerro Saslaya: 13.76941°N 85.02435°W ± 100 m, 1,070 m, 15 May 2011, LLAMA#Go-D-03-3-02, montane wet forest, beating vegetation, 1 alate gyne (CASENT0629025) [UCDC]. Nuevo Segovia: 9 km NW Jalapa, 13.97707°N 86.18907°W ± 100 m, 1,410 m, 30 May 2011, LLAMA#Go-D-06-2-03, oak cloud forest, beating vegetation, 1 worker (CASENT0629264) [UCDC].

**Geographic range:** Mid-to-high elevations of southern Mexico (Chiapas) to Costa Rica (Fig. 107A).

**Worker diagnosis:** *Temnothorax fuscatus* can be separated from all other species in the *salvini* clade by the following character combination: antennal scapes short: failing to reach the posterior margin of the head by about the maximum width of the antennal scape (SI 83–93); body elongate (WLI > 150); metanotal groove not strongly impressed; propodeal spines very long and acute, about one and a half times as long as the propodeal declivity (PSI 23–35); propodeal spines curved and directed posteriorly or posterodorsally, never directly upward; hind femora variable, but usually strongly incrassate (FI > 300); petiolar node low, elongate and rounded dorsally (NI 177–246); postpetiole narrow: greater than one and a half times as wide as the petiole in dorsal view, but less than twice as wide (PWI 165–193); setae on head, mesosoma, waist segments and gaster erect, moderately long, sparse and blunt (never long and tapering); integument variably colored, but always uniform (never bicolored).

**Similar species:** *Temnothorax acutispinosus* sp. nov., *T. subditivus, T. tenuisculptus, T. tuxtlanus* sp. nov., species of the *annexus*, *augusti*, *fuscatus*, and *salvini* groups. *Temnothorax fuscatus* can be distinguished from *T. tuxtlanus* sp. nov. by the incrassate femora (FI < 280 in *T. tuxtlanus* sp. nov.). The low, elongate petiolar node (NI > 180) will separate *T. fuscatus* from all species listed above, aside from the *salvini* group and most other members of the *fuscatus* group. The blunt-tipped setae and short antennal scapes, which fail to reach the posterior margin of the head, will separate *T. fuscatus* from the species of the *salvini* group, which have long, tapering setae and scapes that surpass the posterior margin of the head by at least one times the maximum width of the antennal scape (SI 83–89 vs. > 95). The long propodeal spines, which are longer than the propodeal declivity, as well as the uniformly colored integument, will distinguish *T. fuscatus* from *T. ocarinae* and *T. skwarrae*, which have very short propodeal spines and are bicolored. The acute propodeal spines will distinguish *T. fuscatus* from *T. nebliselva* sp. nov., which has truncate propodeal spines.

**Worker measurements & indices (*n* = 17):** SL = 0.544–0.652 (0.605); FRS = 0.245–0.282 (0.264); CW = 0.699–0.810 (0.761); CWb = 0.644–0.749 (0.696); PoOC = 0.286–0.373 (0.325); CL = 0.731–0.889 (0.819); EL = 0.176–0.214 (0.196); EW = 0.124–0.156 (0.141); MD = 0.166–0.231 (0.186); WL = 1.006–1.239 (1.132); SPST = 0.251–0.380 (0.322); MPST = 0.294–0.363 (0.321); PEL = 0.363–0.459 (0.424); NOL = 0.234–0.324 (0.277); NOH = 0.121–0.147 (0.134); PEH = 0.212–0.255 (0.235); PPL = 0.200–0.274 (0.236); PPH = 0.209–0.261 (0.242); PW = 0.431–0.531 (0.489); SBPA = 0.127–0.198 (0.170); SPTI = 0.233–0.316 (0.270); PEW = 0.141–0.170 (0.157); PNW = 0.153–0.228 (0.184); PPW = 0.246–0.312 (0.278); HFL = 0.715–0.856 (0.788); HFWmax = 0.140–0.197 (0.172); HFWmin = 0.044–0.077 (0.055); CS = 1.010–1.194 (1.105); ES = 0.239–0.292 (0.266); SI = 83–93 (87); OI = 22–26 (24); CI = 80–90 (85); WLI = 154–177 (163); SBI = 20–28 (24); PSI = 23–35 (29); PWI = 165–193 (177); PLI = 163–200 (180); NI = 177–246 (208); PNWI = 105–136 (117); NLI = 58–72 (65); FI = 231–407 (317).

**Worker description:** In full-face view, head subquadrate, longer than broad (CI 80–90). Mandibles densely, finely striate but shining and armed with five teeth: the apical-most well developed and acute, followed by a less developed preapical tooth and three equally developed smaller teeth. Anterior clypeal margin flat medially. Antennal scapes short: when fully retracted, failing to reach the posterior margin of the head capsule by about the maximum width of the antennal scape (SI 83–93). Antennae 12-segmented; antennal club of composed of three segments, with the apical-most segment as long as the preceding two in combination. Frontal carinae moderately long, extending past the antennal toruli by about two and a half times the maximum width of the antennal scape. Compound eyes weakly protruding past the lateral margins of the head capsule. Lateral margin of head weakly convex, forming a continuous arc from the mandibular insertions to the posterior margin of the head. Posterior head margin flat but rounding evenly into the lateral margins.

In profile view, compound eyes ovular and moderately large (OI 22–26), with 11 ommatidia in longest row. Pronotal declivity indistinct, neck and anterior face of pronotum forming a ~120° angle. Mesosoma slightly sinuate: very weakly convex from where it joins the pronotal neck to the propodeum, nearly flat; propodeum weakly depressed and rounds evenly into the base of the propodeal spines. Promesonotal suture extending from the posterior margin of the procoxal insertion to the mesothoracic spiracle, which is moderately well developed. Metanotal groove visible as a disruption of the sculpture laterally from where it arises between the mid- and hind coxae to where it ends in the poorly developed metathoracic spiracle, which is nearly indistinguishable from the ground sculpture. Propodeal spiracle moderately well developed, directed posterolaterally, and separated from the propodeal declivity by about five spiracle diameters. Propodeal spines well developed and very long (PSI 23–35), about one and a half times as long as the propodeal declivity, tapering evenly from the base, evenly downcurved, and acute. Propodeal declivity weakly concave, forming a rounded ~110° angle with the base of the propodeal spines. Propodeal lobes rounded and weakly developed. Metapleural gland bulla very large, extending from the metacoxal insertion three quarters of the way to the propodeal spiracle. Petiole moderately long (PLI 163–200), without tubercles anterodorsally. Subpetiolar process in the form of a moderately large, anteriorly curved, blunt tooth; ventral margin of petiole slightly bulging medially. Petiolar peduncle moderately long: comprising about half the length of the petiole. Petiolar node low and dorsally rounded: transition between peduncle and node very subtly marked by a rounded angle of ~150°, resulting in a weakly concave anterior node face; anterior face rounding evenly into dorsal and posterior faces. Postpetiole long; evenly rounded anteriorly, weakly convex dorsally, and lobed ventrally.

In dorsal view, humeri developed and distinct: evenly rounded and wider than the rest of the mesosoma; mesothoracic spiracles weakly protruding past the lateral margins of the mesosoma, visible as slight angles where the pronotum meets the mesonotum. Promesonotal suture visible as a disruption in the sculpture. Mesometanotal suture visible as a very weak sulcus. Propodeum depressed posterior to the metanotal groove, which is also represented by a very weak sulcus and disruption in the ground sculpture. Propodeal spines closely approximated basally and diverging apically, their apices separated from each other by about their length, the negative space between them “U” shaped. Petiolar peduncle with spiracles very weakly protruding past the lateral margins, and slightly constricted anterior to them. Petiolar node evenly ovular and slightly longitudinally elongate; node broader than the peduncle and evenly grading into the caudal cylinder, which is slightly narrower than the node. Postpetiole narrow (PWI 165–193) longitudinally elongate, and campaniform. Anterior margin of the postpetiole flat and evenly rounds into the lateral margins, which weakly diverge to the angulate posterior corners; posterior margin flat. Metafemur very incrassate (FI 231–407).

Sculpture: median clypeal carina present, extending posteriorly to the level of the anterior margins of the antennal insertions, and flanked on either side by two equally strong carinae. Lateral clypeal lobes with additional, weaker carinae; ground sculpture finely areolate-costulate. Antennal scapes areolate. Cephalic dorsum areolate, with costulae between the frontal carinae. Lateral surfaces of head areolate with costulae overlying the ground sculpture; costulae becoming stronger between the compound eye and the mandibular insertion. Ventral surface of the head weakly areolate. Pronotal neck areolate-strigulate. Lateral surfaces of the pronotum, mesopleurae, and lateral surface of propodeum costate, with weak areolate ground sculpture. Propodeal declivity weakly areolate, but smooth and shining between the propodeal spines. Dorsal surface of mesosoma sculptured similarly to the lateral surface. Femora weakly areolate. Petiole weakly areolate ventrally, otherwise sculptured similarly to the mesosoma. Postpetiole densely areolate, with fine costulate overlying the ground sculpture. First gastral tergite with fine areolate sculpture on the basal quarter, otherwise smooth and shining, with moderately strong spectral iridescence. First gastral sternite smooth and shining, with moderately strong spectral iridescence.

Setae: antennal scapes and funiculi with short, decumbent pilosity. Dorsum of the head, pronotum, waist segments, and gaster with sparse, erect, blunt-tipped setae, the longest of which are about the length of the compound eye. The head bears ~20, mesosoma ~18, petiole 6, postpetiole ~8, and first gastral tergite ~30 setae. Short, sparse pubescence present over the entire body, but difficult to detect against the densely sculptured integument.

Color: predominantly medium brown. Mandibles, antennae, pronotal neck, coxae, trochanters, femora, tibiae, and gastral sclerites (excluding the basalmost) testaceous. Tarsi and sting testaceous yellow.

**Gyne measurements & indices (*n* = 6):** SL = 0.573-0.646 (0.619); FRS = 0.286–0.321 (0.303); CW = 0.815–0.885 (0.852); CWb = 0.733–0.815 (0.771); PoOC = 0.334–0.362 (0.350); CL = 0.858–0.924 (0.898); EL = 0.238–0.275 (0.259); EW = 0.180–0.200 (0.189); MD = 0.161–0.199 (0.177); WL = 1.597–1.793 (1.701); SPST = 0.237–0.275 (0.262); MPST = 0.395–0.445 (0.419); PEL = 0.438–0.494 (0.471); NOL = 0.291–0.343 (0.317); NOH = 0.121–0.155 (0.145); PEH = 0.245–0.310 (0.278); PPL = 0.238–0.300 (0.264); PPH = 0.297–0.377 (0.342); PW = 0.754–0.822 (0.795); SBPA = 0.259–0.387 (0.327); SPTI = 0.274–0.317 (0.297); PEW = 0.193–0.236 (0.215); PNW = 0.231–0.290 (0.264); PPW = 0.343–0.394 (0.369); HFL = 0.789–0.865 (0.836); HFWmax = 0.174–0.197 (0.187); HFWmin = 0.059–0.070 (0.064); CS = 1.162–1.275 (1.220); ES = 0.330–0.366 (0.354); SI = 78–85 (80); OI = 28–31 (29); CI = 81–90 (86); WLI = 215–230 (221); SBI = 35–51 (42); PSI = 0–16 (8); PWI = 162–182 (172); PLI = 165–197 (179); NI = 194–264 (220); PNWI = 120–128 (123); NLI = 59–73 (67); FI = 263–313 (294).

**Gyne description:** In full-face view, head subquadrate, longer than broad (CI 81–90). antennal scapes short: when fully retracted, failing to reach the posterior margin of the head capsule by about the maximum width of the antennal scape (SI 78–85). Mandibles densely striate but shining and armed with six teeth: the apical-most well developed, followed by a less developed preapical tooth and four equally developed smaller teeth. Anterior clypeal margin weakly emarginated medially. Antennae 12-segmented; antennal club composed of three segments, with the apical-most segment as long as the preceding two in combination. Frontal carinae moderately long, extending past the antennal toruli by about two and a half times the maximum width of the antennal scape. Compound eyes moderately protruding past the lateral margins of the head capsule. Lateral margin of head evenly convex, diverging from the mandibular insertions to below the compound eyes. Posterior head margin flat, rounding evenly into the lateral margins.

In profile view, compound eyes ovular and large (OI 28–31), with 17 ommatidia in longest row. Pronotum with a distinct shape: longitudinally excavate laterally. Procoxae similarly shaped, with the anterior face strongly and evenly concave. Mesoscutum rounded evenly anteriorly, not fully covering the dorsal surface of the pronotum, and flat dorsally. Mesoscutellum on the same level as the mesoscutum but sloping posteriorly. Metanotum on the same plane as the mesoscutellum. Propodeum on the same plane as the previous sclerites. Propodeal spiracle well developed, directed posterolaterally, and separated from the propodeal declivity by about five spiracle diameters. Propodeal spines absent but represented as blunt angles. Propodeal declivity slightly concave. Propodeal lobes rounded and very weakly developed. Metapleural gland bulla large, extending from the metacoxal insertion three quarters of the way to the propodeal spiracle. Petiole moderately long (PLI 165–197), without tubercles anterodorsally. Subpetiolar process in the form of a moderately large, acute tooth, which grades evenly into the ventral margin of the petiole posteriorly. Petiolar peduncle short: comprising about a quarter of the total length of the petiole. Petiolar node low and dorsally rounded: transition between peduncle and node an even grade; anterior face meeting the dorsal face at a rounded ~120° angle; dorsal face rounding evenly into the posterior face. Postpetiole weakly convex anteriorly, bulging slightly anterodorsally before flattening posterodorsally; lobed ventrally.

In dorsal view, mesoscutum not fully covering pronotum anteriorly; humeri visible laterally as angulate sclerites. Mesoscutum with a distinctive shape: hexagonal, with anterior face narrow and weakly convex, lateral faces diverging posteriorly to the wing bases, then converging slightly to the posterior face, which is broader than the anterior face. Propodeal angles diverging apically. Petiolar peduncle with spiracles weakly protruding past the lateral margins. Petiolar node, when viewed posterodorsally, with node narrowing apically and the dorsal margin weakly emarginated. Petiolar node slightly narrower than the peduncle, and evenly grading into the caudal cylinder, which slightly narrower than the node. Postpetiole narrow (PWI 162–182), and subquadrate. Anterior margin of postpetiole flat, with corners marked by rounded angles as it transitions to the lateral margins, which are parallel to the angulate posterior corners; posterior margin flat. Metafemur moderately to strongly incrassate (FI 263–313).

Sculpture: median clypeal carina present, extending from the anterior margin to the level of the antennal insertions, ground sculpture longitudinally striate. Lateral clypeal lobes with additional carinae. Antennal scapes areolate-costulate. Cephalic dorsum sculptured similarly to the worker. Lateral surfaces of head sculptured similarly to the worker, but with rugose between the compound eye and the mandibular insertion. Ventral surface of head with weakly longitudinally areolate-costulate sculpture, which becomes weaker medially. Pronotal neck areolate-strigulate. Pronotum weakly areolate-costulate anteriorly, lateral faces smooth and shining through weak costulate sculpture. Anepisternum and katepisternum shining on their anterior quarters, transitioning into areolate-costulate sculpture posteriorly. Metapleuron strongly costulate, with weak areolate ground sculpture. Propodeal declivity smooth and shining. Mesoscutum costulate, with a small patch of smooth and shining sculpture anteromedially. Mesoscutellum costulate. Metanotum smooth and shining. Dorsum of propodeum coarsely costulate over areolate ground sculpture. Femora smooth and shining, with traces of weak areolate sculpture. Petiole shining and very weakly areolate ventrally; dorsum of peduncle and node smooth and shining. Dorsolateral face of petiole coarsely costulate over weak areolate sculpture. Postpetiole finely costulate, overlying areolate sculpture laterally and on the posterior quarter. First gastral tergite and sternite smooth and shining, with moderately strong spectral iridescence.

Setae: antennal scapes and funiculi with short, decumbent pilosity. Dorsum of the head, pronotum, waist segments, and gaster with sparse, erect, blunt-tipped setae, the longest of which are about half the length of the compound eye. Short, sparse pubescence present over the entire body, but difficult to detect against the densely sculptured integument.

Color: predominantly medium brown. Mandibles, antennae, pronotal neck, coxae, trochanters, femora, tibiae, and gastral sclerites (excluding the basalmost) testaceous. Wing bases, tarsi and sting testaceous yellow.

**Male measurements & indices (*n* = 1):** SL = 0.255; FRS = 0.158; CW = 0.593; CWb = 0.476; PoOC = 0.226; CL = 0.554; EL = 0.241; EW = 0.192; MD = 0.049; WL = 0.936; SPST = n/a; MPST = 0.265; PEL = 0.328; NOL = 0.226; NOH = 0.096; PEH = 0.191; PPL = 0.211; PPH = 0.167; PW = 0.589; SBPA = n/a; SPTI = n/a; PEW = 0.145; PNW = 0.196; PPW = 0.239; HFL = 0.631; HFWmax = 0.119; HFWmin = 0.055; CS = 0.753; ES = 0.337; SI = 54; OI = 45; CI = 86; WLI = 197; SBI = n/a; PSI = n/a; PWI = 165; PLI = 155; NI = 235; PNWI = 135; NLI = 69; FI = 216.

**Male description:** In full-face view, head subovate, longer than broad (CI 86). Mandibles smooth and shining, with traces of striae and armed with five teeth: the apical-most well developed, followed by a smaller preapical tooth and three roughly equally developed smaller teeth. Anterior clypeal margin weakly emarginate. Antennal scapes short: when fully retracted, failing to reach the posterior margin of the head capsule by about three times the maximum width of the antennal scape (SI 54). Antennae 13-segmented; antennal club composed of four segments, with the apical-most segment slightly longer than the preceding three in combination. Frontal carinae short, extending past the antennal toruli by one and a half times the maximum width of the antennal scape. Compound eyes strongly protruding past the lateral margins of the head capsule. Posterior head margin flat, rounding evenly into the lateral margins. Lateral margin of head convex behind the compound eyes, margin between the mandibular insertions and the anterior margin of the compound eye straight.

In profile view, anterior of head concave. Compound eyes ovular and large (OI 45), with 19 ommatidia in the longest row. Mesoscutum bulging anteriorly, covering the dorsal surface of the pronotum, and flat dorsally. Mesoscutellum on the same plane as the mesoscutum; dorsum evenly rounding into the posterior margin. Metanotum extending past the posterior margin of the mesoscutellum. Propodeal spiracle well developed, directed laterally, and separated from the propodeal declivity by about four spiracle diameters. Propodeal spines absent, indicated by weak, rounded angles on the dorsum of the propodeum. Propodeal lobes rounded and weakly developed. Metapleural gland bulla very large, extending three quarters of the way between the insertion of the metacoxa and the propodeal spiracle. Petiole moderately long (PLI 155), without tubercles anterodorsally. Subpetiolar process absent. Petiolar peduncle short: comprising less than a quarter of the total length of the petiole. Petiolar node low and rounded, the convergence of the anterior and posterodorsal faces marked by a rounded angle. Postpetiole evenly rounded anterodorsally, before flattening posteriorly; ventral surface concave.

In dorsal view, mesoscutum covering pronotum anteriorly, humeri not visible laterally. Petiolar peduncle with spiracles slightly protruding past the lateral margins. Petiolar node wider than the peduncle and the caudal cylinder. Postpetiole narrow (PWI 165), elongate, and campaniform. Anterior margin of postpetiole weakly convex, with the anterior corners evenly rounding into the lateral margins, which evenly diverge to the angulate posterior corners; posterior margin of postpetiole flat. Metafemur not incrassate (FI 216).

Sculpture: median clypeal carina absent. Antennal scapes shining through very weak areolate sculpture. Head areolate, with a smooth and shining central strip extending from the frontal triangle to the median ocellus. Area around the antennal insertions also smooth and shining. Lateral surface of head areolate. Ventral surface of head weakly areolate-costulate. Pronotal neck weakly areolate. Lateral surfaces of pronotum, anepisternum, katepisternum, and metapleuron smooth and shining, but katepisternum has traces of areolate sculpture. Propodeum weakly areolate laterally; propodeal declivity smooth and shining. Dorsally, mesoscutum weakly longitudinally areolate-costulate, with smooth and shining patches anteromedially and anterolaterally. Mesoscutellum smooth and shining. Femora smooth and shining. Petiole with shallow areolate sculpture ventrally, the dorsal surface of the node smooth and shining; dorsum of peduncle and dorsolateral faces of node areolate with overlying costulate sculpture. Dorsal surface of postpetiole smooth and shining. First gastral tergite and sternite smooth and shining.

Setae: antennal scapes and funiculi with short, decumbent pilosity. Dorsum of the head, pronotum, waist segments, and gaster with sparse, erect, tapering setae, the longest of which are about half the length of the compound eye. Short, sparse pubescence present over the entire body, but difficult to detect against the sculptured integument.

Color: predominantly medium brown. Mandibles, antennae, pronotum, legs, wing bases, and genitals light yellow.

**Etymology:** Morphological, Latin ‘fuscatus’ (= darkened), presumably for the dark integument of the type series.

**Figure 111:**
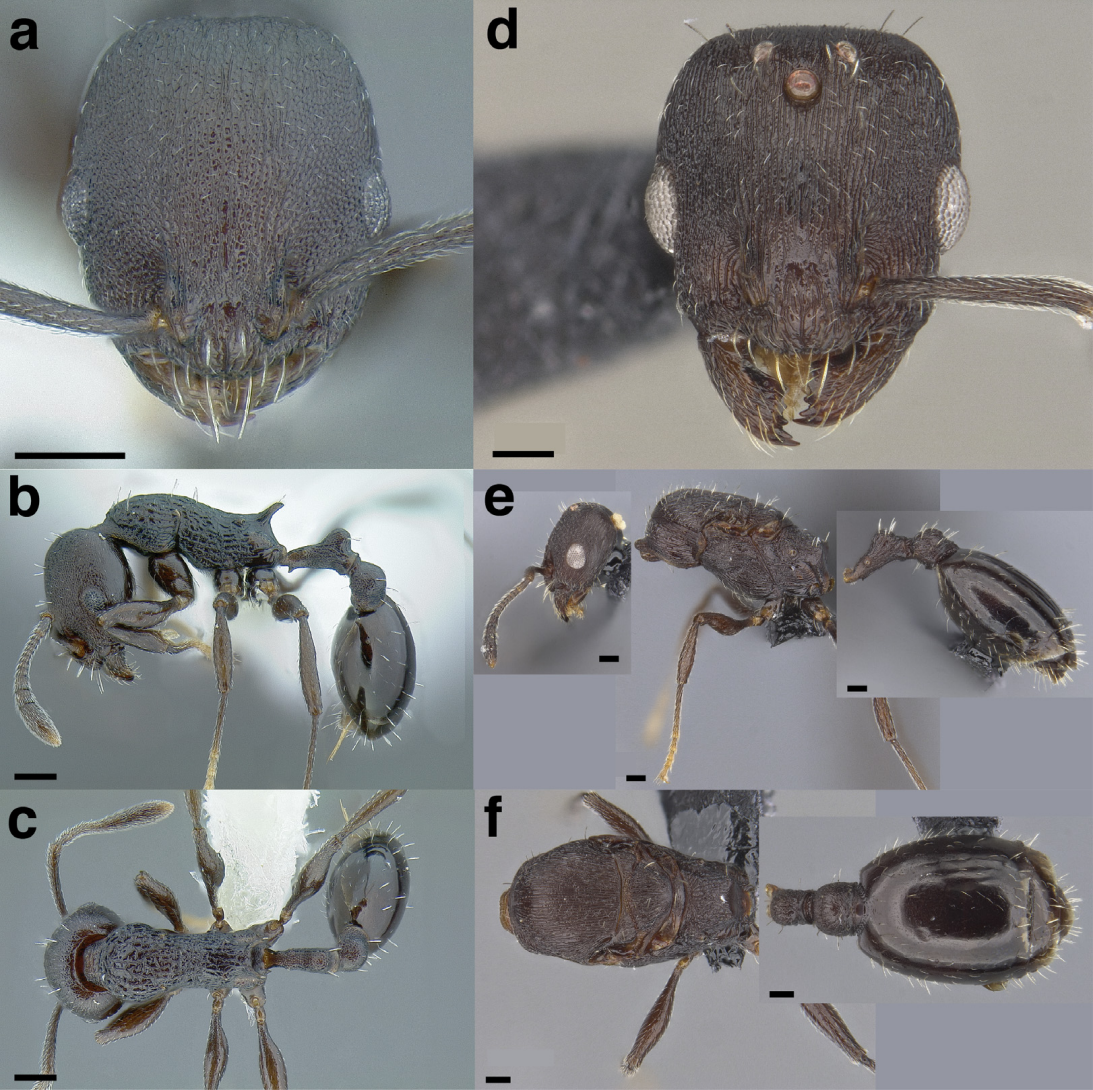
*Temnothorax nebliselva* sp. nov. (A–C) Worker (INB0003659261). (A) Full-face view. (B) Profile view. (C) Dorsal view. (D–F) Gyne (INB0003659261). (D) Full-face view. (E) Profile view. (F) Dorsal view. Scale bars 0.2 mm.

**Comments:** *Temnothorax fuscatus* is broadly distributed across mid-to-high elevation cloud forest habitats in Central America south of the Isthmus of Tehuantepec to Costa Rica. All nest collections of *T. fuscatus* have been taken from dead vegetation, often on live trees; canopy fogging has yielded many specimens as well. This species displays a high degree of morphological variability (Fig. 109 & Fig. 110); as our understanding of the geographic distribution of this variability improves, it may be revealed as a species complex. Typically, *T. fuscatus* is darkly colored, coarsely sculptured, and bears acute propodeal spines (Fig. 109A–109I). However, a nearly uniformly light colored morph is present near the southern end of its range, in Nicaragua and Costa Rica (Fig. 109J–109L). While costulae are present on the mesosoma of all specimens that I have examined, the degree of coarseness shows constant variability, and while some specimens bear smooth, glossy integument between the rugae, many show some degree of fine, areolate sculpture in addition (Fig. 109A, 109D, 109G, 109J). The propodeal spines, while always fairly long, vary from the typical strongly curved form to a posterodorsally directed, nearly straight and acute form (Fig. 109B vs. 109E, 109H). The gynes are morphologically variable as well: for example, the lateral faces of the pronotum and procoxae are concave in some specimens, as if they are excavated to accommodate the retracted profemora (Fig. 110B), but apparently this is not the rule (Fig. 108E, 110E). *Temnothorax fuscatus* most closely resembles *T. nebliselva* sp. nov. from Costa Rica, which has a moderately depressed propodeum, mesosomal integument that is smooth and shining between the costulae, and downwardly curved, blunt or truncated propodeal spines (Fig. 111A–111C). *Temnothorax ocarinae* and *T. skwarrae*, from Western Mexico (Jalisco state) and south-central Mexico respectively, are other apparently close relatives, based on their morphological affinities.

***Temnothorax nebliselva* sp. nov .**

Distribution: Fig. 107B; worker & gyne: Fig. 111.

**Type material examined:** *Holotype worker*: COSTA RICA: Heredia: 10 km NE Vara Blanca, 1,500 m, 10.23617°N 84.11767 °W, 10 February 2005, InBio-OET-ALAS transect, J. Longino#JTL5434 (CASENT0869139) [CASC].

*Paratype workers:* same data as previous, 3 workers (INB0003659289, CASENT0869137, CASENT0869138) [INBio] 1 worker (CASENT0869139) [CASC] 1 worker (CASENT0869161) [LACM] 1 worker (CASENT0869162) [MCZC] 2 workers (CASENT0869163, CASENT0869165) [USNM] 1 worker (CASENT0869164) [AMNH] 1 worker (CASENT0869166) [UCDC] 1 worker (CASENT0869167) [UNAM] 1 worker (CASENT0869168) [UVGC] 1 worker (CASENT0869169) [FSCA].

**Non-type material examined:** COSTA RICA: Heredia: 9 km NE Vara Blanca, 1,450–1,500 m, 10.23617°N 84.11767 °W, 9 February 2005, InBio-OET-ALAS transect, JTL5420, 1 worker & 1 dealate gyne (INB0003659261, CASENT0869067) [CASC] 2 workers (INB0003659262-3) [JTLC]; 10 km NE Vara Blanca, 1,500 m, 10.23617°N 84.11767 °W, 10 February 2005, InBio-OET-ALAS transect, J. Longino#JTL5433, 5 workers & 1 dealate gyne (INB0003659286-8, CASENT0869134-6) [JTLC]; same data as previous, except: 11 March 2005, InBio-OET-ALAS transect, J. Longino#JTL5506, 4 workers (INB0003659384, CASENT0869140-2) [JTLC]; 16 km SSE La Virgen, 10.266667°N 84.083333°W, 1,050–1,150 m, 19 February 2001, INBio-OET-ALAS transect 11/RG/DBM/001, 1 worker (INB0003225001) [JTLC]; Puntarenas: Monteverde: 10.300000°N 84.800000°W, 1,500 m, 17-25 May 2001, S. Yanoviak & J. Gering, pasture 4-1, canopy fogging study, 29 workers (JTLC000002967-JTLC000002995) [JTLC].

**Geographic range:** Mid elevations of Costa Rica (Fig. 107B).

**Worker diagnosis:** *Temnothorax nebliselva* sp. nov. can be separated from all other species in the *salvini* clade by the following character combination: antennal scapes short: failing to reach the posterior margin of the head by about the maximum width of the antennal scape (SI 84–89); body elongate (WLI > 150); metanotal groove not strongly impressed; propodeal spines very long, about one and a half times as long as the propodeal declivity (PSI 23–28), with tips truncate; propodeal spines curved and directed posteriorly or posterodorsally, never directly upward; hind femora moderately to strongly incrassate (FI 272–321); petiolar node high, nodiform and rounded dorsally (NI 208–260); postpetiole narrow: greater than one and a half times as wide as the petiole in dorsal view, but less than twice as wide (PWI 154–167); setae on head, mesosoma, waist segments and gaster erect, moderately long, sparse and blunt (never long and tapering); integument dark brown.

**Similar species:** *Temnothorax acutispinosus* sp. nov., *T. altinodus* sp. nov., *T. subditivus, T. tenuisculptus, T. tuxtlanus* sp. nov., species of the *annexus*, *augusti*, *fuscatus*, and *salvini* groups. *Temnothorax nebliselva* sp. nov. can be distinguished from *T. tuxtlanus* sp. nov. by the incrassate femora (FI < 280 in *T. tuxtlanus* sp. nov.). The elongate, dorsally rounded petiolar node (NI > 200) will separate *T. nebliselva* sp. nov. from other members of the *fuscatus* group, (petiolar node elongate and grading evenly into the petiolar peduncle), the *salvini* group (petiolar node elongate and grading evenly into the petiolar peduncle to squamiform), the *augusti* group (posteriorly leaning petiolar node), the *annexus* group and *T. tenuisculptus* (subquadrate petiolar node), *T. subditivus* (squamiform petiolar node), *T. acutispinosus* sp. nov., and *T. tuxtlanus* sp. nov. (shorter petiolar node: NI < 180). The blunt-tipped setae and short antennal scapes, which fail to reach the posterior margin of the head, will separate *T. nebliselva* sp. nov. from the species of the *salvini* group, which have long, tapering setae and scapes that surpass the posterior margin of the head by at least one times the maximum width of the antennal scape (SI 83–89 vs. > 95). The long, truncate propodeal spines, which are longer than the propodeal declivity, as well as the uniformly colored integument, will distinguish *T. nebliselva* sp. nov. from *T. fuscatus* (acute propodeal spines), *T. ocarinae* and *T. skwarrae*, (very short propodeal spines and bicolored).

**Worker measurements & indices (*n* = 3):** SL = 0.508–0.545 (0.526); FRS = 0.228–0.254 (0.241); CW = 0.617–0.688 (0.657); CWb = 0.570–0.625 (0.606); PoOC = 0.281–0.303 (0.293); CL = 0.680–0.729 (0.710); EL = 0.147–0.183 (0.166); EW = 0.106–0.127 (0.117); MD = 0.141–0.157 (0.151); WL = 0.899–0.981 (0.948); SPST = 0.211–0.270 (0.248); MPST = 0.267–0.287 (0.279); PEL = 0.333–0.373 (0.356); NOL = 0.239–0.255 (0.248); NOH = 0.098–0.120 (0.107); PEH = 0.191–0.218 (0.204); PPL = 0.179–0.198 (0.185); PPH = 0.189–0.202 (0.194); PW = 0.403–0.459 (0.422); SBPA = 0.141–0.170 (0.152); SPTI = 0.200–0.246 (0.216); PEW = 0.136–0.147 (0.143); PNW = 0.120–0.139 (0.128); PPW = 0.210–0.245 (0.230); HFL = 0.564–0.647 (0.617); HFWmax = 0.125–0.147 (0.136); HFWmin = 0.039–0.054 (0.045); CS = 0.91–0.99 (0.961); ES = 0.200–0.247 (0.225); SI = 84–89 (87); OI = 22–25 (23); CI = 84–86 (85); WLI = 155–158 (157); SBI = 23–27 (25); PSI = 23–28 (26); PWI = 154–167 (160); PLI = 186–202 (192); NI = 208–260 (234); PNWI = 86–95 (89); NLI = 67–72 (70); FI = 272–321 (304).

**Worker description:** In full-face view, head subquadrate, longer than broad (CI 84–86). Mandibles densely, finely striate but shining and armed with five teeth: the apical-most well developed and acute, followed by a less developed preapical tooth and three equally developed smaller teeth. Anterior clypeal margin evenly rounded. Antennal scapes short: when fully retracted, failing to reach the posterior margin of the head capsule by about the maximum width of the antennal scape (SI 84–89). Antennae 12-segmented; antennal club of composed of three segments, with the apical-most segment as long as the preceding two in combination. Frontal carinae moderately long, extending past the antennal toruli by about two and a half times the maximum width of the antennal scape. Compound eyes weakly protruding past the lateral margins of the head capsule. Lateral margin of head weakly convex, forming a continuous arc from the mandibular insertions to the posterior margin of the head. Posterior head margin flat but rounding evenly into the lateral margins.

In profile view, compound eyes ovular and moderately large (OI 22–25), with 11 ommatidia in longest row. Pronotal declivity indistinct, neck and anterior face of pronotum forming a ~120° angle. Mesosoma sinuate: evenly convex from where it joins the pronotal neck to the propodeum; propodeum depressed and rounds evenly into the base of the propodeal spines. Promesonotal suture extending from the posterior margin of the procoxal insertion to the mesothoracic spiracle, which is moderately well developed. Metanotal groove visible as a disruption of the sculpture laterally from where it arises between the mid- and hind coxae to where it ends in the poorly developed metathoracic spiracle, which is nearly indistinguishable from the ground sculpture. Propodeal spiracle moderately well developed, directed posterolaterally, and separated from the propodeal declivity by about five spiracle diameters. Propodeal spines well developed and very long (PSI 23–28), about one and a half times as long as the propodeal declivity, tapering evenly from the base, evenly curved, and apically truncate. Propodeal declivity weakly concave, forming a rounded ~110° angle with the base of the propodeal spines. Propodeal lobes rounded and weakly developed. Metapleural gland bulla very large, extending from the metacoxal insertion three quarters of the way to the propodeal spiracle. Petiole moderately long (PLI 186–202), without tubercles anterodorsally. Subpetiolar process in the form of a moderately large, triangular, acute tooth; ventral margin of petiole slightly bulging medially. Petiolar peduncle moderately long: comprising about half the length of the petiole. Petiolar node moderately high and dorsally rounded: transition between peduncle and node marked by a rounded angle of ~120°, resulting in a concave anterior node face; anterior face rounding evenly into dorsal and posterior faces. Postpetiole long; evenly rounded anteriorly, weakly convex dorsally, and flat to weakly lobed ventrally.

In dorsal view, humeri developed and distinct: evenly rounded and wider than the rest of the mesosoma; mesothoracic spiracles weakly protruding past the lateral margins of the mesosoma, visible as slight angles where the pronotum meets the mesonotum. Promesonotal suture visible as a disruption in the sculpture. Mesometanotal suture and metanotal groove obscured by ground sculpture. Propodeal spines closely approximated basally and diverging apically, their apices separated from each other by about their length, the negative space between them “U” shaped. Petiolar peduncle with spiracles very weakly protruding past the lateral margins, and slightly constricted anterior to them. Petiolar node weakly ovular and slightly longitudinally elongate; node slightly narrower than the peduncle and evenly grading into the caudal cylinder, which is slightly broader than the node. Postpetiole narrow (PWI 154–167) longitudinally elongate, and campaniform. Anterior margin of the postpetiole flat and evenly rounds into the lateral margins, which weakly diverge to the angulate posterior corners; posterior margin flat. Metafemur moderately to strongly incrassate (FI 272–321).

Sculpture: median clypeal carina present, extending posteriorly to the level of the anterior margins of the antennal insertions, and flanked on either side by two equally strong carinae. Lateral clypeal lobes with additional, weaker carinae; ground sculpture finely areolate-costulate. Antennal scapes areolate. Cephalic dorsum areolate, with costulae between the frontal carinae. Lateral surfaces of head areolate with costulae overlying the ground sculpture, become stronger between the compound eye and the mandibular insertion. Ventral surface weakly areolate. Pronotal neck finely areolate-strigulate. Lateral surfaces of the pronotum rugose-costate rugulae in the interspaces; mesopleurae and lateral surface of propodeum rugose, with weak areolate ground sculpture. Propodeal declivity weakly areolate, but smooth and shining between the propodeal spines. Dorsal surface of mesosoma rugose, with weak areolae in the interspaces. Femora weakly areolate, becoming smooth and shining medially. Petiole evenly weakly areolate-rugulose. Postpetiole densely areolate-rugulose. First gastral tergite smooth and shining, with moderately strong spectral iridescence. First gastral sternite smooth and shining, with moderately strong spectral iridescence.

Setae: antennal scapes and funiculi with short, decumbent pilosity. Dorsum of the head, pronotum, waist segments, and gaster with sparse, erect, blunt-tipped setae, the longest of which are about the length of the compound eye. The head bears ~12, mesosoma ~8, petiole 2, postpetiole ~6, and first gastral tergite ~14 setae. Short, sparse pubescence present over the entire body, but difficult to detect against the densely sculptured integument.

Color: predominantly medium brown. Mandibles, antennae, pronotal neck, coxae, trochanters, femora, tibiae, and gastral sclerites (excluding the basalmost) testaceous. Tarsi and sting testaceous yellow.

**Gyne measurements & indices (*n* = 1):** SL = 0.602; FRS = 0.328; CW = 0.851; CWb = 0.772; PoOC = 0.357; CL = 0.856; EL = 0.245; EW = 0.196; MD = 0.153; WL = 1.567; SPST = 0; MPST = 0.407; PEL = 0.47; NOL = 0.328; NOH = 0.128; PEH = 0.275; PPL = 0.248; PPH = 0.306; PW = 0.81; SBPA = 0.269; SPTI = 0.269; PEW = 0.215; PNW = 0.237; PPW = 0.347; HFL = 0.801; HFWmax = 0.161; HFWmin = 0.064; CS = 1.2; ES = 0.343; SI = 78; OI = 29; CI = 90; WLI = 203; SBI = 35; PSI = 0; PWI = 161; PLI = 190; NI = 256; PNWI = 110; NLI = 70; FI = 252.

**Gyne description:** In full-face view, head subquadrate, longer than broad (CI 90). Mandibles densely striate but shining and armed with five teeth: the apical-most well developed, followed by a less developed preapical tooth and three equally developed smaller teeth. Anterior clypeal margin flat medially. Antennal scapes short: when fully retracted, failing to reach the posterior margin of the head capsule by about the maximum width of the antennal scape (SI 78). Antennae 12-segmented; antennal club composed of three segments, with the apical-most segment as long as the preceding two in combination. Frontal carinae moderately long, extending past the antennal toruli by about two and a half times the maximum width of the antennal scape. Compound eyes moderately protruding past the lateral margins of the head capsule. Lateral margin of head evenly convex, converging from below the compound eyes to the mandibular insertions. head margin flat, rounding evenly into the lateral margins.

In profile view, compound eyes ovular and large (OI 28–31), with 17 ommatidia in longest row. Pronotum with a distinct shape: longitudinally excavate laterally. Mesoscutum rounded evenly anteriorly, not fully covering the dorsal surface of the pronotum, and flat dorsally. Mesoscutellum on the same level as the mesoscutum but sloping posteriorly. Metanotum and propodeum slightly depressed below the mesoscutellum. Propodeal spiracle well developed, directed posterolaterally, and separated from the propodeal declivity by about five spiracle diameters. Propodeal spines absent but represented as blunt angles. Propodeal declivity slightly concave. Propodeal lobes rounded and very weakly developed. Metapleural gland bulla large, extending from the metacoxal insertion three quarters of the way to the propodeal spiracle. Petiole moderately long (PLI 190), without tubercles anterodorsally. Subpetiolar process in the form of a small, acute tooth, which grades evenly into the ventral margin of the petiole posteriorly. Petiolar peduncle short: comprising about a third of the total length of the petiole. Petiolar node low and dorsally rounded: transition between peduncle and node an even grade; anterior face meeting the dorsal face at a rounded ~120° angle; dorsal face rounding evenly into the posterior face. Postpetiole weakly convex anteriorly, bulging slightly anterodorsally before flattening posterodorsally; lobed ventrally.

In dorsal view, mesoscutum not fully covering pronotum anteriorly; humeri visible laterally as angulate sclerites. Mesoscutum with a distinctive shape: hexagonal, with anterior face narrow and convex, lateral faces diverging posteriorly to the wing bases, then converging slightly to the posterior face, which is broader than the anterior face. Propodeal angles diverging apically. Petiolar peduncle with spiracles weakly protruding past the lateral margins. Petiolar node, when viewed posterodorsally, with node weakly narrowing apically. Petiolar node slightly narrower than the peduncle, and evenly grading into the caudal cylinder, which slightly narrower than the node. Postpetiole narrow (PWI 161), and campaniform. Anterior margin of postpetiole flat, with corners marked by rounded angles as it transitions to the lateral margins, which are parallel to the angulate posterior corners; posterior margin flat. Metafemur moderately incrassate (FI 252).

Sculpture: median clypeal carina present, but difficult to distinguish from the multitude of equally strong flanking carinae. Lateral clypeal lobes with additional strong carinae. Antennal scapes weakly areolate-costulate. Cephalic dorsum areolate-costulate, becoming costate between the frontal carinae. Ventral surface of head areolate-costulate. Pronotal neck weakly areolate-strigulate, with weak overlying rugae. Pronotum costate. Anepisternum densely rugulose, with costate sculpture ventrally and posteriorly. Katepisternum with predominantly costate. Metapleuron costate, with rugulose ground sculpture. Propodeal declivity weakly strigulate basally, with weak areolae between the propodeal angles. Mesoscutum costulate. Mesoscutellum costulate. Metanotum smooth and shining, with weak strigulate sculpture laterally. Dorsum of propodeum rugose. Femora smooth and shining, with traces of weak areolate sculpture. Petiole costate over areolate ground sculpture. Postpetiole costulate, overlying areolate sculpture. First gastral tergite and sternite smooth and shining, with moderately strong spectral iridescence.

Setae: antennal scapes and funiculi with short, decumbent pilosity. Dorsum of the head, pronotum, waist segments, and gaster with sparse, erect, tapering setae, the longest of which are about half the width of the compound eye. Short, sparse pubescence present over the entire body, but difficult to detect against the densely sculptured integument.

Color: predominantly medium brown. Mandibles, antennae, pronotal neck, coxae, trochanters, femora, tibiae, and gastral sclerites (excluding the basalmost) testaceous. Tarsi and sting testaceous yellow.

**Etymology:** Ecological, Spanish for the cloud forest habitat where this species has been collected.

**Comments:** *Temnothorax nebliselva* sp. nov. is known from a couple of localities in northern Costa Rica, both of which are montane cloud forest. The hand collections of this species, made by Jack Longino, were small monogynous nests in the internodes of a *Cecropia polyphlebia* sapling in a pasture abutting cloud forest. The other collections were made by sweeping vegetation and canopy fogging. Little else is known about the biology of this species. *Temnothorax nebliselva* sp. nov. most closely resembles the other members of the *fuscatus* group, especially *T. fuscatus*, which has a distribution primarily north of the Nicaraguan depression, save for a single collection of an aberrant light colored form collected in sympatry with *T. nebliselva* sp. nov. in Monteverde.

***Temnothorax ocarinae* (Baroni Urbani, 1978)**

**Figure 112:**
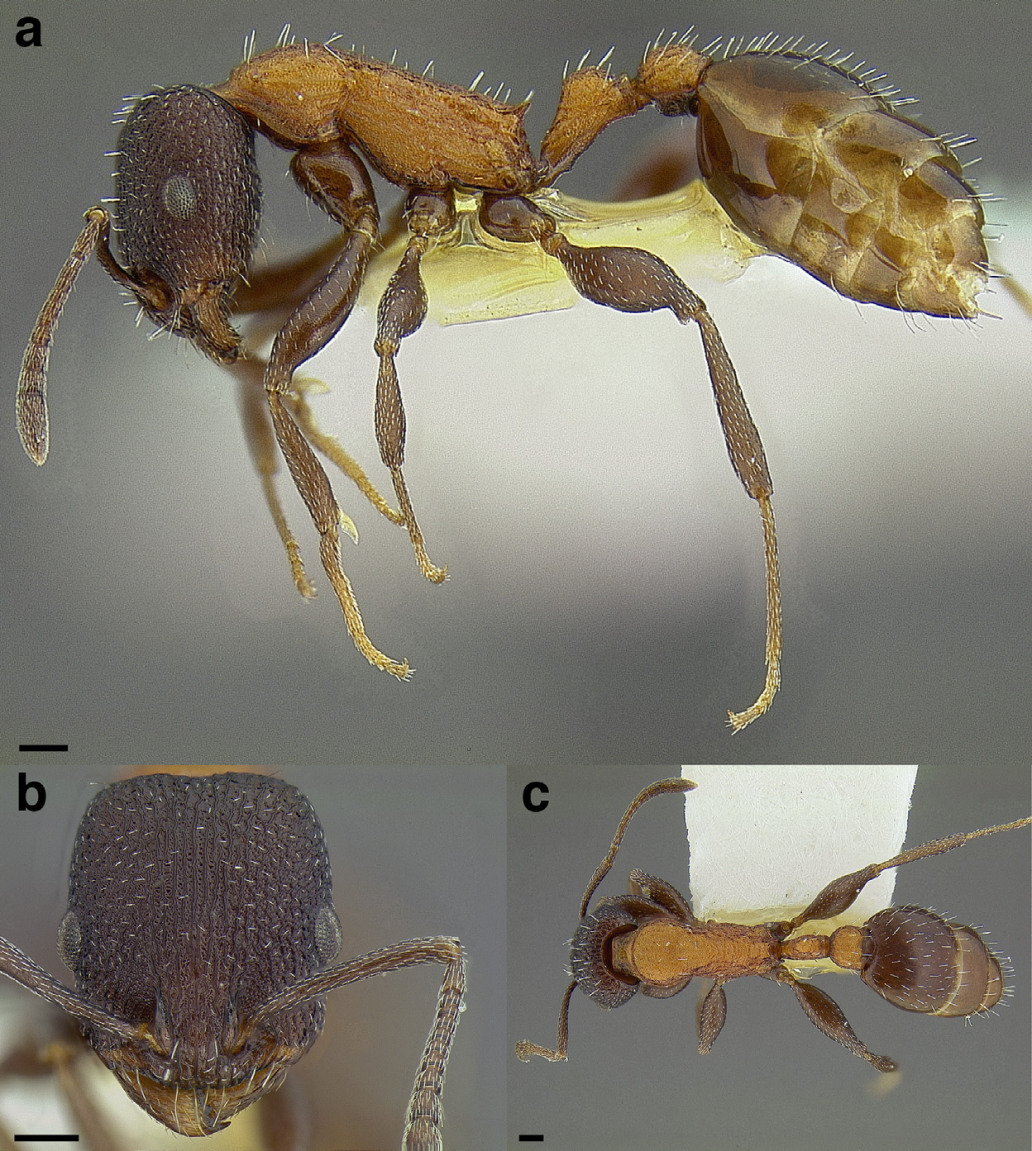
*Temnothorax ocarinae* holotype worker (USNMENT00531636). (A) Profile view. (B) Full-face view. (C) Dorsal view. Scale bars 0.2 mm.

Distribution: Fig. 107C; worker: Fig. 112.

*Leptothorax ocarinae* Baroni Urbani, 1978: 469, figs. 58, 131. Holotype worker. Mexico.

*Temnothorax ocarinae* (Baroni Urbani): Bolton, 2003: 272. First combination in *Temnothorax*.

**Type material examined:** *Holotype worker:* MEXICO: [intercepted in quarantine] at Nogales, Arizona, U.S.A., 23 October 1956, on orchid (USNMENT00531636) [USNM].

*Paratype worker:* same data as holotype, 1 worker (images of CASENT0912976 examined on antweb.org) [NHMB].

**Non-type material examined:** MEXICO: Jalisco: 27 km S. of Mazamitia, 5 December 1948, E.S. Ross, 1 worker (MCZENT00581861) [MCZC].

**Geographic distribution:** Mid elevation western Mexico (Jalisco) (Fig. 107C).

**Worker diagnosis:** *Temnothorax ocarinae* can be separated from all other species in the *salvini* clade by the following character combination: antennal scapes short: failing to reach the posterior margin of the head by about the maximum width of the antennal scape (SI 89–103); body elongate (WLI > 150); metanotal groove not strongly impressed; propodeal spines directed posterodorsally, never directly upward; propodeal spines short, much shorter than the length of the propodeal declivity (PSI 16–18); hind femora strongly incrassate (FI > 350); petiolar node low, elongate and rounded dorsally (NI 186–203); postpetiole moderately broad: greater than twice wide as the petiole in dorsal view, but less than 2.2 times as wide (PWI 208–209); setae on head, mesosoma, waist segments and gaster erect, moderately long, moderately dense and blunt (never long and tapering); integument bicolored: mesosoma and waist segments testaceous, otherwise medium brown.

**Similar species:** *Temnothorax acutispinosus* sp. nov., *T. huehuetenangoi, T. subditivus, T. tenuisculptus, T. tuxtlanus* sp. nov., species of the *annexus*, *augusti*, *fuscatus*, and *salvini* groups. *Temnothorax ocarinae* can be distinguished from *T. tuxtlanus* sp. nov. by the incrassate femora (FI < 280 in *T. tuxtlanus* sp. nov.). The low, elongate petiolar node (NI > 180) will separate *T. ocarinae* from all species listed above, aside from the *salvini* group and most other members of the *fuscatus* group. The moderately long, blunt-tipped setae will separate *T. ocarinae* from the species of the *salvini* group, which have long, tapering setae. The short propodeal spines, which are much shorter than the propodeal declivity, as well as the bicolored integument, will distinguish *T. ocarinae* from *T. fuscatus* and *T. nebliselva* sp. nov., which have longer propodeal spines and are uniformly colored. *Temnothorax ocarinae* can be separated from *T. skwarrae* by the relatively small, round compound eye, which is shorter than the distance between the compound eye and the mandibular insertion (about as long as the distance between the compound eye and mandibular insertion in *T. skwarrae*). Furthermore, *T. skwarrae* has a bright yellow gaster, as opposed to medium brown in *T. ocarinae*.

**Worker measurements & indices (*n* = 2):** SL = 0.633–0.729 (0.681); FRS = 0.214–0.275 (0.245); CW = 0.675–0.890 (0.783); CWb = 0.613–0.816 (0.715); PoOC = 0.342–0.407 (0.375); CL = 0.796–0.988 (0.892); EL = 0.160–0.206 (0.183); EW = 0.122–0.155 (0.139); MD = 0.179–0.232 (0.206); WL = 1.032–1.332 (1.182); SPST = 0.185–0.214 (0.200); MPST = 0.302–0.385 (0.344); PEL = 0.336–0.446 (0.391); NOL = 0.218–0.308 (0.263); NOH = 0.117–0.152 (0.135); PEH = 0.240–0.244 (0.242); PPL = 0.232–0.253 (0.243); PPH = 0.227–0.285 (0.256); PW = 0.435–0.578 (0.507); SBPA = 0.103–0.174 (0.139); SPTI = 0.121–0.163 (0.142); PEW = 0.138–0.173 (0.156); PNW = 0.152–0.208 (0.180); PPW = 0.289–0.359 (0.324); HFL = 0.681–0.894 (0.788); HFWmax = 0.191–0.251 (0.221); HFWmin = 0.050–0.060 (0.055); CS = 1.011–1.310 (1.161); ES = 0.221–0.284 (0.252); SI = 89–103 (96); OI = 22; CI = 77–83 (80); WLI = 163–168 (166); SBI = 17–21 (19); PSI = 16–18 (17); PWI = 208–209 (208); PLI = 145–176 (161); NI = 186–203 (194); PNWI = 110–120 (115); NLI = 65–69 (67); FI = 382–418 (400).

**Worker description:** In full-face view, head subquadrate, longer than broad (CI 77–83). Mandibles densely striate but shining and armed with five teeth: the apical-most well developed and acute, followed by a less developed preapical tooth and three equally developed smaller teeth. Anterior clypeal margin weakly emarginated medially. Antennal scapes short: when fully retracted, failing to reach the posterior margin of the head capsule by about the maximum width of the scape (SI 89–103). Antennae 12-segmented; antennal club of composed of three segments, with the apical-most segment about as long as the preceding two in combination. Frontal carinae moderately long, extending past the antennal toruli by about two times the maximum width of the antennal scape. Compound eyes moderately protruding past the lateral margins of the head capsule. Lateral margin of head very weakly convex, nearly flat, forming a continuous arc from the mandibular insertions to the posterior margin of the head. Posterior head margin weakly concave but rounding evenly into the lateral margins.

In profile view, compound eyes ovular and moderately large (OI 22), with 13 ommatidia in longest row. Pronotal declivity distinct, neck and anterior face of pronotum forming a ~90° angle; transition between the anterior and dorsal faces delimited by a transverse carina. Dorsal face of pronotum evenly rounding into the rest of the mesosoma, which is flat to the propodeal spines. Promesonotal suture extending from the posterior margin of the procoxal insertion only to the mesothoracic spiracle, which is moderately well developed. Metanotal groove visible as a disruption of the sculpture laterally from where it arises between the mid- and hind coxae to where it ends in the poorly developed metathoracic spiracle, which is nearly indistinguishable against the ground sculpture. Propodeal spiracle moderately well developed, directed posterolaterally, and separated from the propodeal declivity by about three spiracle diameters. Propodeal spines weakly developed and short (PSI 16–18), about a third as long as the propodeal declivity, tapering evenly from the base, straight, directed posterodorsally, and acute. Propodeal declivity flat, forming a ~120° angle with the base of the propodeal spines. Propodeal lobes dorsally angulate and small. Metapleural gland bulla moderately large, extending from the metacoxal insertion two thirds of the way to the propodeal spiracle. Petiole moderately long (PLI 145–176), without tubercles anterodorsally. Subpetiolar process in the form of a very small, blunt tooth; ventral margin of petiole flat posterior to it. Petiolar peduncle short: comprising about a quarter of the total length of the petiole. Petiolar node low and dorsally flat: transition between peduncle and node an even grade, resulting in a weakly concave anterior node face; anterior face meeting the dorsal face at a rounded 120° angle; dorsal face rounding evenly into the posterior face. Postpetiole long; anterior and dorsal faces forming an even convexity; lobed ventrally.

In dorsal view, humeri developed and distinct: evenly rounded and wider than the rest of the mesosoma; mesothoracic spiracles weakly protruding past the lateral margins of the mesosoma, visible as slight angles where the pronotum meets the mesonotum. Metanotal groove absent: mesonotum and propodeum completely fused and lateral margins converging evenly to the bases of the propodeal spines. Propodeal spines narrowly approximated basally and weakly diverging apically, their apices separated from each other by about their length, the negative space between them broadly “U” shaped. Petiolar peduncle with spiracles not protruding past the lateral margins. Petiolar node evenly ovular and slightly longitudinally elongate; node the same width as the peduncle and the caudal cylinder. Postpetiole moderately broad (PWI 208–209) and campaniform. Anterior margin of the postpetiole convex and evenly rounds into the lateral margins, which are parallel to each other; posterior corners angulate; posterior margin flat. Metafemur strongly incrassate (FI 382–418).

Sculpture: median clypeal carina present, extending posteriorly nearly to the frontal triangle, and flanked on either side by two equally strong carinae. Lateral clypeal lobes with additional, weaker carinae; ground sculpture finely areolate. Antennal scapes areolate-costulate. Cephalic dorsum densely areolate-rugulose, with overlying rugose sculpture, becoming costate between the frontal carinae. Lateral surfaces of head densely areolate-rugulose, with dense rugose sculpture overlying the ground sculpture. Ventral surface of head areolate-costulate. Pronotal neck weakly areolate. Lateral surface of the mesosoma areolate, with costae overlying the ground sculpture; area directly around propodeal spiracle smooth and shining. Propodeal declivity smooth, with a few coarse strigulae. Dorsal surface of mesosoma densely areolate, with coarse rugose sculpture overlying the ground sculpture. Femora areolate. Petiole densely areolate, with a coarse longitudinal carina extending from the anterodorsal flange to the caudal cylinder; rugose sculpture overlying the areolate ground sculpture dorsal to the carina. Postpetiole densely areolate, with rugose sculpture overlying the ground sculpture. First gastral sternite uniformly weakly areolate, with moderately strong spectral iridescence. First gastral sternite smooth and shining, with weak spectral iridescence.

Setae: antennal scapes and funiculi with short, adpressed pilosity. Dorsum of the head, pronotum, waist segments, and gaster with sparse, erect, blunt-tipped setae, the longest of which are about the length of the compound eye. The head bears ~36, mesosoma ~34, petiole 8, postpetiole ~16, and first gastral tergite ~64 setae. Short, sparse pubescence present over the entire body, but difficult to detect against the densely sculptured integument.

Color: bicolored. Head dark brown. Antennae, legs (except tarsi), and bases of the gastral sclerites testaceous brown. Mandibles, mesosoma, tarsi, waist segments, distal margins of the gastral sclerites, and sting testaceous yellow.

**Gyne:** Unknown.

**Male:** Unknown.

**Etymology:** Unknown.

**Comments:** Little is known about the biology or distribution of *Temnothorax ocarinae*. The holotype was taken in quarantine at the U.S.-Mexico border from an orchid. The only other specimen that I have examined is much smaller than the holotype and was collected from a high elevation site in Jalisco state, Mexico. This species most closely resembles *T. fuscatus* from Central America south of the Isthmus of Tehuantepec. Like *T. fuscatus*, *T. ocarinae* is likely arboreally nesting.

***Temnothorax skwarrae* (Wheeler, 1931)**

**Figure 113:**
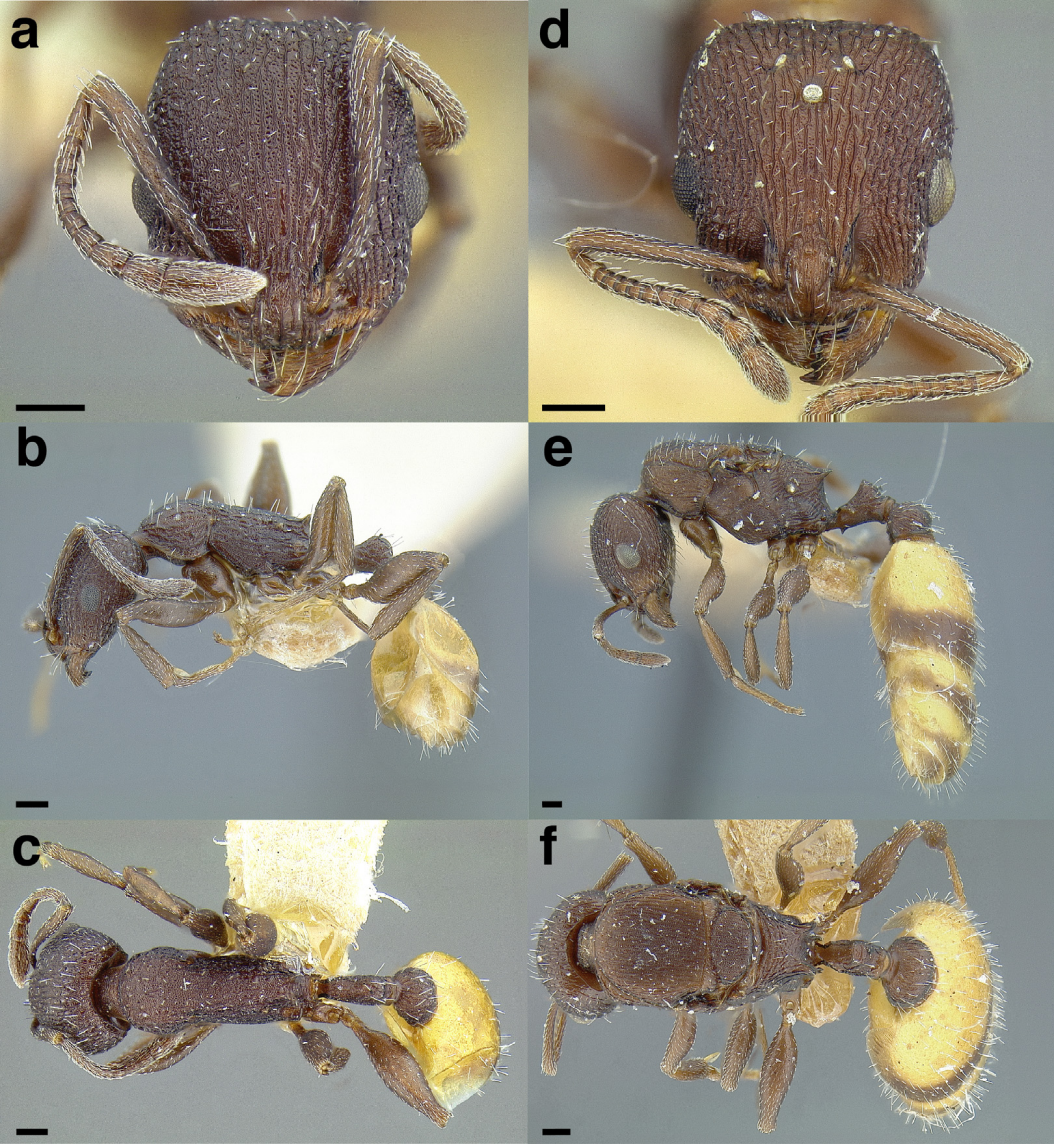
*Temnothorax skwarrae*. (A–C) Lectotype worker (MCZENT00016358). (A) Full-face view. (B) Profile view. (C) Dorsal view. (D–F) Paralectotype gyne (MCZENT00577117). (D) Full-face view. (E) Profile view. (F) Dorsal view. Scale bars 0.2 mm.

Distribution: Fig. 107D; worker & gyne: Fig. 113.

*Macromischa skwarrae* Wheeler, 1931: 10. Syntype workers and gyne. Cuernavaca, Mexico. One syntype worker here designated **lectotype**.

*Leptothorax skwarrae* (Wheeler): Baroni Urbani, 1978: 503. First combination in *Leptothorax*.

*Temnothorax skwarrae* (Wheeler): Bolton, 2003: 272. First combination in *Temnothorax*.

**Type material examined:** *Lectotype worker*: MEXICO: Morelos: Cuernavaca, 26 June 1929, E. Skwarra#Z857a/Sk. 1 worker (M.C.Z. co-type 10 16358, MCZENT00016358) [MCZC].

*Paralectotype workers and gyne*: same data as previous, except: E. Skwarra#Z879/Sk., ex *Tillandsia circinnata*, 1 dealate gyne (M.C.Z. co-type 916358, MCZENT00577117) [MCZC]; same data as previous, except: E. Skwarra#Z877, ex *Tillandsia circinnata*, 1 worker (images of FoCol0411 examined on antweb.org) [SMND] 1 teneral worker (images of FoCol0412 examined on antweb.org) [SMND]; same data as previous, except: E. Skwarra#Z807a/Sk., ex *Tillandsia circinnata* (images of M.C.Z. cotype 4-5 16358, S.N.M.U. type no. co 59607, USNMENT00532047 examined on the Smithsonian National Museum of Natural History Department of Entomology Collections website) [USNM].

**Geographic distribution:** Mid elevations of south-central Mexico (Morelos) (Fig. 107D).

**Worker diagnosis:** *Temnothorax skwarrae* can be separated from all other species in the *salvini* clade by the following character combination: antennal scapes short: failing to reach the posterior margin of the head by about the maximum width of the antennal scape (SI 92); body elongate (WLI > 150); metanotal groove not strongly impressed; propodeal spines directed posterodorsally, never directly upward; propodeal spines short, much shorter than the length of the propodeal declivity (PSI 18); hind femora strongly incrassate (FI > 350); petiolar node low, elongate and rounded dorsally (NI 244); postpetiole narrow: greater than one and a half times as wide as the petiole in dorsal view, but less than 2 times as wide (PWI 198); setae on head, mesosoma, waist segments and gaster erect, moderately long, moderately dense and blunt (never long and tapering); integument bicolored: dark brown with a yellow gaster.

**Similar species:** *Temnothorax acutispinosus* sp. nov., *T. subditivus, T. tenuisculptus, T. tuxtlanus* sp. nov., species of the *annexus*, *augusti*, *fuscatus*, and *salvini* groups. *Temnothorax skwarrae* can be distinguished from *T. tuxtlanus* sp. nov. by the incrassate femora (FI < 280 in *T. tuxtlanus* sp. nov.). The low, elongate petiolar node (NI > 180) will separate *T. skwarrae* from all species listed above, aside from the *salvini* group and most other members of the *fuscatus* group. The moderately long, blunt-tipped setae will separate *T. skwarrae* from the species of the *salvini* group, which have long, tapering setae. The short propodeal spines, which are much shorter than the propodeal declivity, as well as the bicolored integument, will distinguish *T. skwarrae* from *T. fuscatus* and *T. nebliselva* sp. nov., which have longer propodeal spines and are uniformly colored. *Temnothorax skwarrae* can be separated from *T. ocarinae* by the relatively large, oval compound eye, which is about as long as the distance between the compound eye and the mandibular insertion (shorter than the distance between the compound eye and mandibular insertion in *T. ocarinae*). Furthermore, *T. skwarrae* has a bright yellow gaster, as opposed to medium brown in *T. ocarinae*.

**Worker measurements & indices (*n* = 1):** SL = 0.737; FRS = 0.247; CW = 0.857; CWb = 0.804; PoOC = 0.387; CL = 0.965; EL = 0.219; EW = 0.174; MD = 0.218; WL = 1.270; SPST = 0.231; MPST = 0.368; PEL = 0.388; NOL = 0.342; NOH = 0.140; PEH = 0.248; PPL = 0.264; PPH = 0.291; PW = 0.558; SBPA = 0.164; SPTI = 0.189; PEW = 0.177; PNW = 0.145; PPW = 0.350; HFL = 0.880; HFWmax = 0.255; HFWmin = 0.069; CS = 1.287; ES = 0.306; SI = 92; OI = 24; CI = 83; WLI = 158; SBI = 20; PSI = 18; PWI = 198; PLI = 147; NI = 244; PNWI = 82; NLI = 88; FI = 370.

**Worker description:** In full-face view, head subquadrate, longer than broad (CI 83). Mandibles densely striate but shining and armed with five teeth: the apical-most well developed and acute, followed by a less developed preapical tooth and three equally developed smaller teeth. Anterior clypeal weakly emarginate medially. Antennal scapes short: when fully retracted, failing to reach the posterior margin of the head capsule by about the width of the antennal scape (SI 92). Antennae 12-segmented; antennal club of composed of three segments, with the apical-most segment slightly longer than the preceding two in combination. Frontal carinae moderately long, extending past the antennal toruli by about two times the maximum width of the antennal scape. Compound eyes moderately protruding past the lateral margins of the head capsule. Lateral margin of head weakly convex, forming a continuous arc from the mandibular insertions to the posterior margin of the head. Posterior head margin very weakly concave but rounding evenly into the lateral margins.

In profile view, compound eyes ovular and moderately large (OI 24), with 14 ommatidia in longest row. Pronotal declivity distinct, neck and anterior face of pronotum forming a rounded ~90° angle; transition between the anterior and dorsal faces delimited by a transverse carina. Dorsal face of pronotum evenly rounding into the rest of the mesosoma, which is flat to the propodeal spines. Promesonotal suture extending from the posterior margin of the procoxal insertion only to the mesothoracic spiracle, which is moderately well developed. Metanotal groove visible as a disruption of the sculpture laterally from where it arises between the mid- and hind coxae to where it ends in the poorly developed metathoracic spiracle, which is nearly indistinguishable against the ground sculpture. Propodeal spiracle moderately well developed, directed posterolaterally, and separated from the propodeal declivity by about four spiracle diameters. Propodeal spines weakly developed and short (PSI 18), about a third as long as the propodeal declivity, tapering evenly from the base, weakly downcurved, and acute. Propodeal declivity weakly concave, forming a rounded ~120° angle with the base of the propodeal spines. Propodeal lobes dorsally angulate and small. Metapleural gland bulla small, extending from the metacoxal insertion halfway to the propodeal spiracle. Petiole moderately long (PLI 147), with tubercles anterodorsally. Subpetiolar process in the form of a very small, blunt tooth; ventral margin of petiole flat posterior to it. Petiolar peduncle short: comprising about a quarter of the total length of the petiole. Petiolar node low and dorsally rounded transition between peduncle and node an even grade, resulting in a weakly concave anterior node face; anterior face meeting the dorsal face at a rounded ~120° angle; dorsal face rounding evenly into the posterior face. Postpetiole long; anterior and dorsal faces forming an even convexity; lobed ventrally.

In dorsal view, humeri developed and distinct: evenly rounded and wider than the rest of the mesosoma; mesothoracic spiracles weakly protruding past the lateral margins of the mesosoma, visible as slight angles where the pronotum meets the mesonotum. Metanotal groove absent: mesonotum and propodeum completely fused and lateral margins converging evenly to the bases of the propodeal spines. Propodeal spines narrowly approximated basally and weakly diverging apically, their apices separated from each other by about their length, the negative space between them broadly “U” shaped. Petiolar peduncle with spiracles not protruding past the lateral margins. Petiolar node ovular, slightly longitudinally elongate, and narrowed anteriorly; node the same width as the peduncle and the caudal cylinder. Postpetiole narrow (PWI 198) and campaniform. Anterior margin of the postpetiole convex and evenly rounds into the lateral margins, which are parallel to each other; posterior corners angulate; posterior margin weakly emarginate medially. Metafemur strongly incrassate (FI 370).

Sculpture: median clypeal carina present, extending posteriorly nearly to the frontal triangle, and flanked on either side by two equally strong carinae. Lateral clypeal lobes with additional, weaker carinae; ground sculpture areolate. Antennal scapes areolate. Cephalic dorsum densely areolate, with overlying reticulate sculpture; cross-reticulations weaker between the frontal carinae. Lateral surfaces of head densely areolate, with dense reticulate sculpture overlying the ground sculpture. Ventral surface of head longitudinally coarsely areolate-costulate. Pronotal neck areolate. Lateral surface of the mesosoma densely, finely areolate, with coarse longitudinal striae overlying the ground sculpture. Propodeal declivity weakly transversely areolate-costulate. Dorsal surface of mesosoma densely areolate, with coarse rugose sculpture overlying the ground sculpture on the pronotum and propodeum; mesonotal area without overlying sculpture. Femora areolate. Petiole densely areolate, with a coarse longitudinal carina extending from the anterodorsal flange to the caudal cylinder; reticulate sculpture overlying the areolate ground sculpture dorsal to the carina. Postpetiole densely areolate, with reticulate sculpture overlying the ground sculpture. First gastral sternite uniformly weakly areolate, with weak spectral iridescence. First gastral sternite smooth and shining, without spectral iridescence.

Setae: antennal scapes and funiculi with short, decumbent pilosity. Dorsum of the head, pronotum, waist segments, and gaster with sparse, erect, blunt-tipped setae, the longest of which are about the length of the compound eye. The head bears ~34, mesosoma ~30, petiole 8, postpetiole ~16, and first gastral tergite ~30 setae. Short, sparse pubescence present over the entire body, but difficult to detect against the densely sculptured integument.

Color: bicolored. Predominantly dark brown, with a testaceous yellow gaster. Mandibles and legs testaceous brown.

**Gyne measurements & indices (*n* = 1):** SL = 0.751; FRS = 0.335; CW = 1.056; CWb = 1.005; PoOC = 0.456; CL = 1.121; EL = 0.286; EW = 0.217; MD = 0.220; WL = 1.881; SPST = 0.430; MPST = 0.497; PEL = 0.525; NOL = 0.287; NOH = 0.239; PEH = 0.387; PPL = 0.379; PPH = 0.437; PW = 1.018; SBPA = 0.417; SPTI = 0.392; PEW = 0.263; PNW = 0.186; PPW = 0.576; HFL = 0.979; HFWmax = 0.267; HFWmin = 0.078; CS = 1.566; ES = 0.395; SI = 75; OI = 25; CI = 90; WLI = 187; SBI = 41; PSI = 23; PWI = 219; PLI = 139; NI = 120; PNWI = 71; NLI = 55; FI = 342.

**Gyne description:** In full-face view, head subquadrate, longer than broad (CI 90). Mandibles densely striate but shining and armed with five teeth: the apical-most well developed, followed by a less developed preapical tooth and three equally developed smaller teeth. Anterior clypeal margin weakly emarginated medially. Antennal scapes short: when fully retracted, failing to reach the posterior margin of the head capsule by about three times the maximum width of the scape (SI 75). Antennae 12-segmented; antennal club composed of three segments, with the apical-most segment slightly longer than the preceding two in combination. Frontal carinae moderately long, extending past the antennal toruli by about three times the maximum width of the antennal scape. Compound eyes moderately protruding past the lateral margins of the head capsule. Lateral margin of head convex posterior to the compound eyes, flat below the compound eyes to the mandibular insertions. Posterior head margin flat, rounding evenly into the lateral margins.

In profile view, compound eyes ovular and moderately large (OI 25), with 17 ommatidia in longest row. Mesoscutum rounded evenly anteriorly, not fully covering the dorsal surface of the pronotum, and flat dorsally. Mesoscutellum on the same level as the mesoscutum but sloping posteriorly. Metanotum on the same plane as the mesoscutellum. Propodeal spiracle well developed, directed posterolaterally, and separated from the propodeal declivity by about four spiracle diameters. Propodeal spines stout and well developed, but short (PSI 23), about a third as long as the propodeal declivity, tapering evenly from the base, directed posterodorsally, straight, and acute. Propodeal declivity straight and flat, forming a rounded ~110° angle with the base of the propodeal spines. Propodeal lobes dorsally angulate and small. Metapleural gland bulla moderately large, extending from the metacoxal insertion two thirds of the way to the propodeal spiracle. Petiole moderately long (PLI 139), with tubercles anterodorsally. Subpetiolar process in the form of a small, acute tooth, which grades evenly into the ventral margin of the petiole posteriorly. Petiolar peduncle short: comprising less than a quarter of the total length of the petiole. Petiolar node erect and cuneiform: transition between peduncle and node evenly rounded, resulting in a concave anterior node face; anterior face forming a very sharp ~90° angle with the dorsal face, which rounds evenly into the posterior face. Caudal cylinder long, about one and a half times the maximum width of the antennal scape. Postpetiole evenly rounded anterodorsally, bulging slightly before it transitions into the flattened dorsal face; ventral surface lobed.

In dorsal view, mesoscutum not fully covering the pronotum anteriorly; humeri visible laterally as angulate sclerites. Propodeal spines flat dorsally, weakly diverging apically, their apices separated from each other by about two times their length. Petiolar peduncle with spiracles not protruding past the lateral margins. Petiolar node, when viewed posterodorsally, subquadrate: apex narrower apically than the base, which is the same width as the caudal cylinder and peduncle. Postpetiole moderately broad (PWI 219) and campaniform. Anterior margin of postpetiole convex, rounding evenly into the lateral margins, which are parallel to each other; posterior corners angulate; posterior margin flat. Metafemur strongly incrassate (FI 342).

Sculpture: median clypeal carina present, extending from the anterior margin nearly to frontal triangle, and flanked by two equally strong carinae; lateral margins of median clypeal lobe with two carinae that are as strong as the median carina. Lateral clypeal lobes with additional weaker carinae; ground sculpture finely areolate. Antennal scapes areolate-costulate. Cephalic dorsum densely areolate, with overlying rugose sculpture, becoming costate between the frontal carinae. Lateral surfaces of head densely areolate, with dense predominantly costate sculpture overlying the ground sculpture. Ventral surface of head areolate-costulate. Pronotal neck areolate. Pronotum areolate anteriorly, lateral face areolate, with coarse costae overlying the ground sculpture. Anepisternum and katepisternum shining on their anterior quarters, transitioning into areolate-costulate sculpture posteriorly. Metapleuron and propodeum costate. Propodeal declivity weakly areolate. Mesoscutum and mesoscutellum costate, with areolate-rugulose sculpture between them. Metanotum areolate. Dorsum of propodeum strigulate over areolate ground sculpture. Femora weakly areolate. Petiole weakly areolate ventrally, as well as on the dorsal surface of the peduncle and node; coarse longitudinal carina extending from the anterodorsal flange to the caudal cylinder; rugose sculpture overlying the areolate ground sculpture dorsal to the carina. Postpetiole areolate, with concentric costulae overlying the ground sculpture. First gastral tergite densely, finely areolate, without spectral iridescence. Surface of the first gastral sternite smooth and shining, without spectral iridescence.

Setae: antennal scapes and funiculi with short, decumbent pilosity. Dorsum of the head, pronotum, waist segments, and gaster with sparse, erect, blunt-tipped setae, the longest of which are about half the width of the compound eye. Short, sparse pubescence present over the entire body, but difficult to detect against the densely sculptured integument.

Color: bicolored. Predominantly dark brown, with a testaceous yellow gaster. Mandibles and legs testaceous brown.

**Male:** Unknown.

**Etymology:** Matronym, named for Elizabeth Skwarra, who collected the type series.

**Comments:** The type series of *Temnothorax skwarrae* was collected from the epiphyte *Tillandsia circinnata* near Cuernavaca in Morelos state, Mexico. Very little is known about the biology of this species, but it resembles *T. ocarinae* from Jalisco state, and the cloud forest species *T. fuscatus*, which has a range in Central America from south of the Isthmus Tehuantepec to Costa Rica. This species, like *T. fuscatus*, is apparently arboreally nesting. *Temnothorax skwarrae* has also been reported from tropical dry forest habitat in Estacion de Biologia Chamela in Jalisco state (Dáttilo et al., 2020), but I have not personally investigated the specimens, so I exclude them here.

### *goniops* group overview

**Figure 114:**
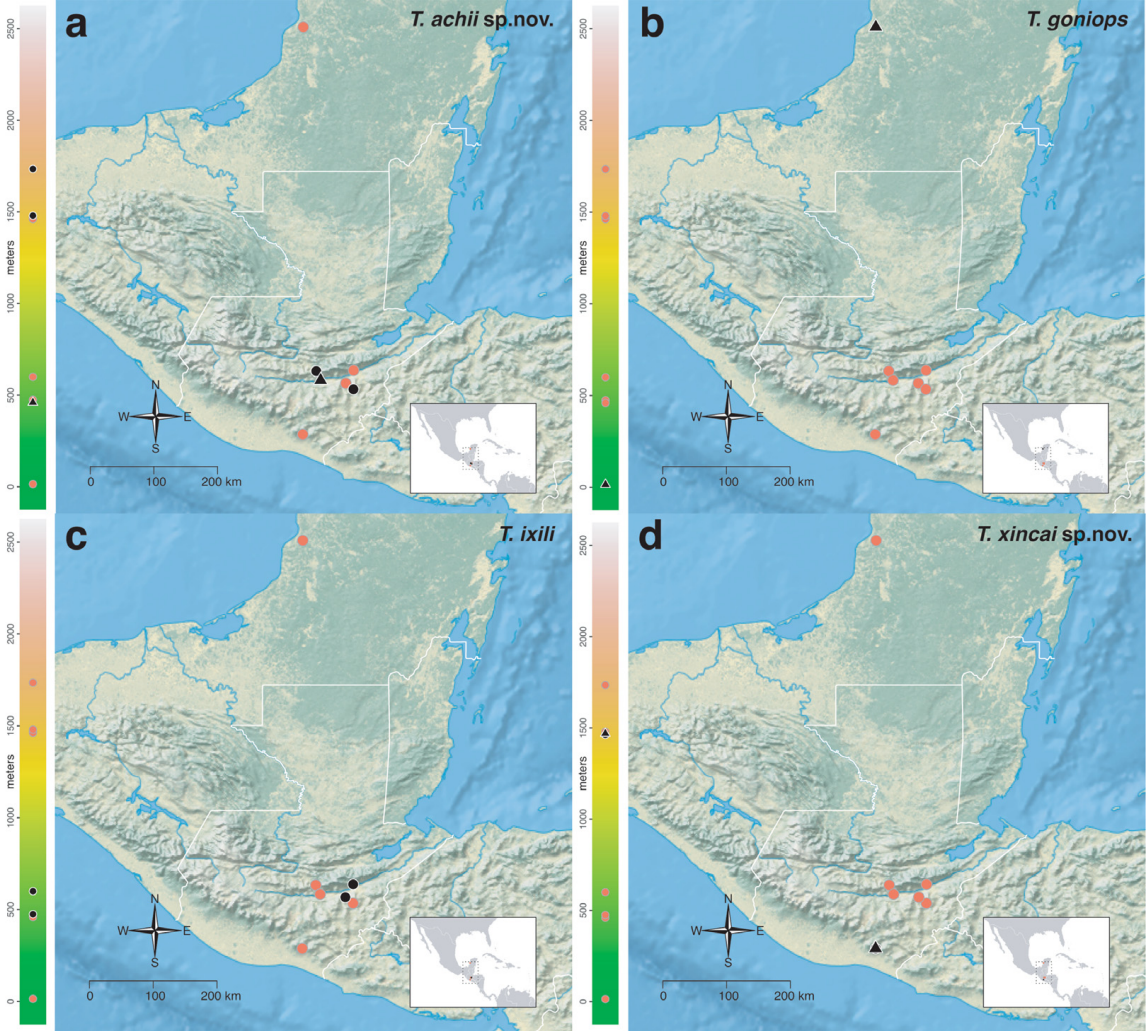
Geographical and elevational distribution of the *goniops* group. (A) *Temnothorax achii* sp. nov. (B) *T. goniops* (C) *T. ixili* (D) *T. xincai* sp. nov. Colored scale to the left of each map represents elevation in meters. Points in black represent the species named in each subfigure, while points in red represent other members of the species group. Type localities are represented by triangles, non-type localities are represented by circles. Bounding box in inset map shows location of main map. Map for *T. huehuetenangoi* not included due to the uncertainty of the collection locality.

With five species (two described as new here), the *goniops* group spans the low-to-mid elevations of Central America and the Yucatán Peninsula of Mexico (Fig. 114). Nest collections of the members of this group have been rare and restricted to those made from orchids in quarantine at United States ports of entry. Other collections have been made from leaf litter extractions. The generalized habitus of the members of this group make them easy to confuse with others in the *salvini* clade. They can be separated from other species with conservative morphology such as the *acuminatus* group, *T. acutispinosus* sp. nov., and *T. altinodus* sp. nov. by the presence of erect setae on the dorsum of the propodeum (absent in the preceding species), from *T. subditivus* by the petiolar node (squamiform in *T. subditivus*), and from members of the *pulchellus* group by geography (the *pulchellus* group spans the islands of the Caribbean and southern Florida).

***Temnothorax achii* sp. nov.**

**Figure 115:**
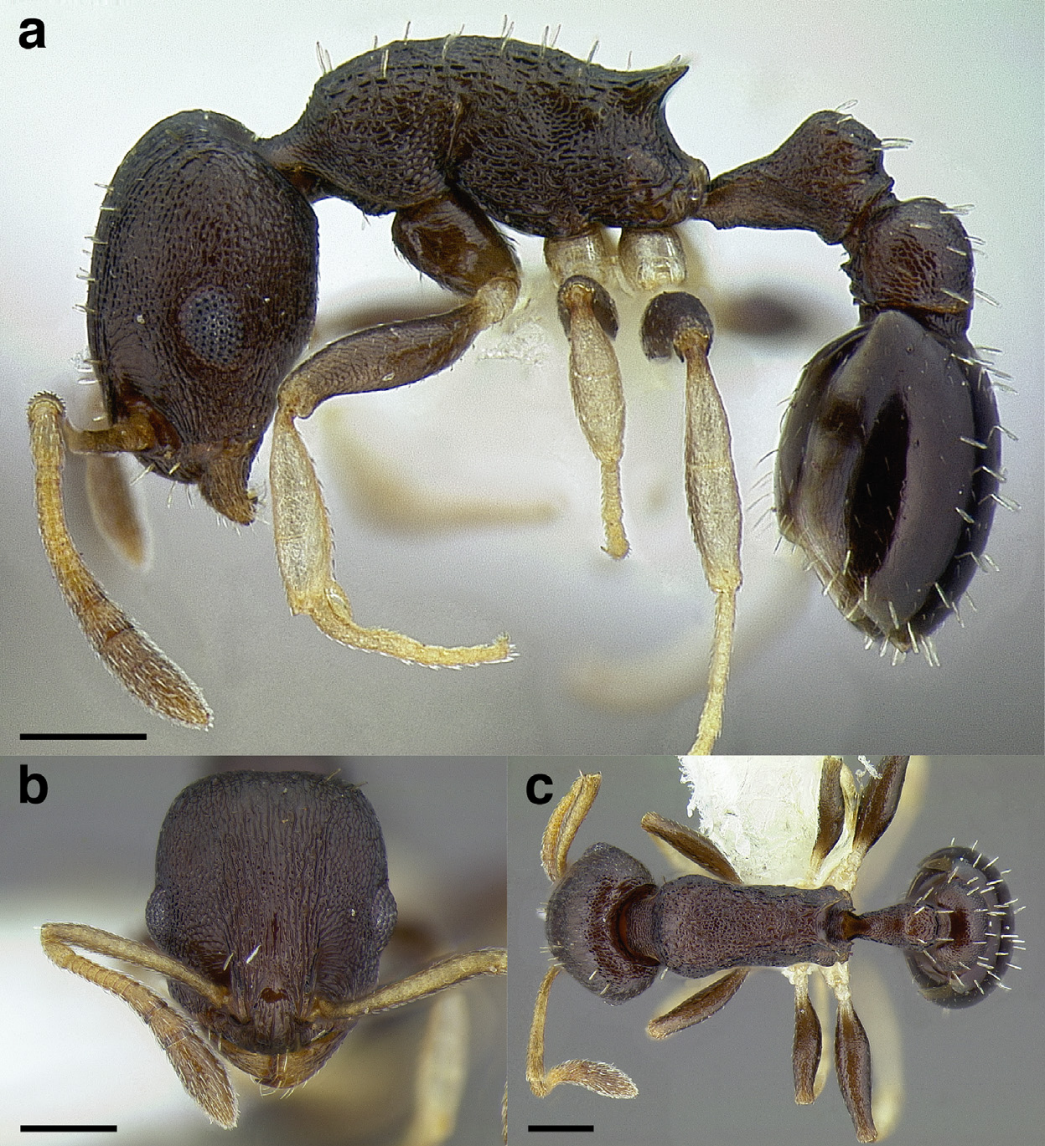
*Temnothorax achii* sp. nov. holotype worker (JTLC000009876). (A) Profile view. (B) Full-face view. (C) Dorsal view. Scale bars 0.2 mm.

Distribution: Fig. 114A; worker: Fig. 115.

*Temnothorax* mmp04 Prebus, 2017: 8. In phylogeny.

**Type material examined:** *Holotype worker:* GUATEMALA: El Progreso: 3.7 km SW Morazán, 14.9051°N -90.15362°W, 460 m, 7 July 2007, J. Longino #6002-s, tropical dry forest, ex sifted leaf litter (JTLC000009876) [CASC].

*Paratype workers:* same data as holotype, 1 worker (JTLC000009871) [MCZC] 1 worker (JTLC000009906) [USNM] 1 worker (CASENT0869159) [LACM] 1 worker (CASENT0869160) [UVGC]; same data as holotype, except: M.G. Branstetter #626-12, tropical dry scrub, ex sifted leaf litter, 1 worker (CASENT0600922) [UCDC].

**Non-type material examined:** GUATEMALA: Chiquimula: 15 km W Chiquimula, 14.77968°N 89.67686°W ± 50 m, 1,735 m, 27 April 2014, M. Barrios #MB1314, second growth oak cloud forest, ex sifted leaf litter, 1 worker (CASENT0758686) [UCDC]; Baja Verapaz: 1 km SSW La Cumbre, 15.033333°N 90.216667°W, 1,480 m, 19 November 2003, P.S. Ward#15097-14, mixed tropical/temperate mesic forest, sifted litter (leaf mold, rotten wood), 3 workers (CASENT0915976) [UCDC].

**Geographic range:** Low-to-mid elevations in central Guatemala (Baja Verapaz, Chiquimula, El Progreso) (Fig. 114A).

**Worker diagnosis:** *Temnothorax achii* sp. nov. can be separated from all other species in the *salvini* clade by the following character combination: antennal scapes short, failing to reach the posterior margin of the head capsule by about the maximum width of the scape (SI 85–90); compound eyes small (OI 22–26); body moderately compact (WLI 137–143); metanotal groove absent; propodeum not strongly depressed; propodeal spines shorter than the propodeal declivity and dentate (PSI 25–28); propodeal spines broadly approximated in dorsal view (SBI 28–34); hind femora weakly to moderately incrassate (FI 180–303); petiolar node erect and dorsally rounded, not overhanging the caudal cylinder in profile view; petiolar node compact, not elongate (NI 143–170); petiolar node very slightly broader than caudal cylinder in dorsal view (PNWI < 115); head sculpture areolate, with areolae arranged into columns by costulae; dorsum of postpetiole sculptured; antennal scapes with short, decumbent pilosity; setae on head, mesosoma, waist segments and gaster erect, short, sparse and blunt (never long and tapering); integument predominantly dark brown, with trochanters, meso- and metacoxae, tibiae, tarsi, and sting very light yellow.

**Similar species:** *Temnothorax acutispinosus* sp. nov., *T. altinodus* sp. nov., *T. tuxtlanus* sp. nov., *T. subditivus*, species of the *augusti*, *goniops*, and *pulchellus* groups, species of the *sallei* clade. *Temnothorax achii* sp. nov. can be distinguished from similar appearing members of the *sallei* clade (e.g., *T. manni, T. mexicanus, T. punctithorax*) by the petiole, which lacks a transverse carina anterodorsally. Unlike the long, dorsally directed propodeal spines of *T. acutispinosus* sp. nov., *T. achii* sp. nov. has short propodeal teeth that are directed posterodorsally. *Temnothorax achii* sp. nov. has a moderately broad postpetiole in dorsal view, whereas *T. tuxtlanus* sp. nov. has a relatively narrow postpetiole (PWI < 150). *Temnothorax altinodus* sp. nov. has comparatively strongly incrassate hind femora (FI 327). *Temnothorax achii* sp. nov. can be separated from *T. subditivus* and the *augusti* group by the structure of the petiolar node, which is erect, dorsally rounded, and only slightly broader than the caudal cylinder in dorsal view, as opposed to squamiform in *T. subditivus*, or leaning posteriorly over the caudal cylinder in the *augusti* group. *Temnothorax achii* sp. nov. can be distinguished from the *pulchellus* group by the combination of a relatively longer mesosoma (WLI > 135 vs. < 135 in most *pulchellus* group members). Some members of the *pulchellus* group may approach these dimensions (e.g., *T. agavicola* sp. nov., *T. albispinus, T. flavidulus, T. hippolytus* sp. nov., *T. laticrus* sp. nov., *T. terricola, T. torrei*, and *T. wilsoni* sp. nov.), but they can be separated from *T. achii* sp. nov. by their very broad postpetioles (PWI > 220). *Temnothorax achii* sp. nov. can be distinguished from other *goniops* group members by the short, dentate propodeal spines (longer than the propodeal declivity and spiniform in *T. goniops* and *T. huehuetenangoi*), the areolate-rugulose head and postpetiole (smooth and shining in *T. xincai* sp. nov.), and the relatively narrow, rugulose petiolar node (narrower, on average, in *T. achii* sp. nov. and uniformly areolate).

**Worker measurements & indices (*n* = 6):** SL = 0.375–0.444 (0.418); FRS = 0.165–0.181 (0.174); CW = 0.485–0.561 (0.524); CWb = 0.442–0.519 (0.479); PoOC = 0.220–0.253 (0.235); CL = 0.541–0.633 (0.582); EL = 0.114–0.146 (0.136); EW = 0.090–0.112 (0.104); MD = 0.113–0.142 (0.121); WL = 0.611–0.744 (0.668); SPST = 0.157–0.201 (0.177); MPST = 0.196–0.244 (0.220); PEL = 0.258–0.308 (0.286); NOL = 0.142–0.184 (0.161); NOH = 0.098–0.109 (0.102); PEH = 0.168–0.198 (0.185); PPL = 0.156–0.205 (0.176); PPH = 0.176–0.212 (0.195); PW = 0.308–0.389 (0.340); SBPA = 0.128–0.175 (0.145); SPTI = 0.169–0.235 (0.194); PEW = 0.136–0.152 (0.146); PNW = 0.148–0.184 (0.164); PPW = 0.265–0.309 (0.293); HFL = 0.402–0.474 (0.443); HFWmax = 0.081–0.129 (0.112); HFWmin = 0.040–0.046 (0.044); CS = 0.713–0.836 (0.77); ES = 0.159–0.199 (0.188); SI = 85–90 (87); OI = 22–26 (24); CI = 82–85 (82); WLI = 137–143 (139); SBI = 28–34 (30); PSI = 25–28 (26); PWI = 191–208 (200); PLI = 150–170 (163); NI = 143–170 (157); PNWI = 109–121 (112); NLI = 50–60 (56); FI = 180–303 (256).

**Worker description:** In full-face view, head subquadrate, longer than broad (CI 82–85). Mandibles densely, finely striate but shining and armed with five teeth: the apical-most well developed and acute, followed by a less developed preapical tooth and three equally developed smaller teeth. Anterior clypeal margin evenly convex medially. Antennal scapes short: when fully retracted, failing to reach the posterior margin of the head capsule by about the maximum width of the scape (SI 85–90). Antennae 12-segmented; antennal club of composed of three segments, with the apical-most segment longer than the preceding two in combination. Frontal carinae moderately long, extending past the antennal toruli by about three times the maximum width of the antennal scape. Compound eyes moderately protruding past the lateral margins of the head capsule. Lateral margin of head very weakly convex, nearly flat, forming a continuous low arc from the mandibular insertions to the posterior margin of the head. Posterior head margin weakly concave medially but rounding evenly into the lateral margins.

In profile view, compound eyes ovular, longitudinally elongate, and moderately large (OI 22–26), with 10 ommatidia in longest row. Pronotal declivity indistinct, but neck and anterior face of pronotum forming a ~120° angle. Mesosoma convex from where it joins the pronotal neck to the propodeal spines; propodeum slightly flatter, giving the dorsum of the mesosoma a weakly sinuate profile. Promesonotal suture extending from the posterior margin of the procoxal insertion to the mesothoracic spiracle, which is moderately well developed; continuing dorsally as a weak sulcus. Metanotal groove visible as a disruption of the sculpture laterally from where it arises between the mid- and hind coxae to where it ends in the poorly developed metathoracic spiracle, which is nearly indistinguishable against the ground sculpture. Propodeal spiracle moderately well developed, directed posterolaterally, and separated from the propodeal declivity by about two and a half spiracle diameters. Propodeal spines poorly developed and short (PSI 25–28), about two thirds the length of the propodeal declivity, flared at the base, straight, and acute. Propodeal declivity with weak carinae extending between the base of the propodeal spines and the propodeal lobes; declivity straight, forming a rounded ~120° angle with the base of the propodeal spines. Propodeal lobes rounded and weakly developed. Metapleural gland bulla small, extending from the metacoxal insertion halfway to the propodeal spiracle. Petiole moderately long (PLI 150–170), without tubercles anterodorsally. Subpetiolar process in the form of a small, triangular, acute tooth; ventral margin of petiole flat posteriorly to it. Petiolar peduncle short: comprising about a third of the petiole length. Petiolar node robust and rounded: node transitioning evenly into the petiolar peduncle, resulting in a concave anterior node face; anterior face rounding evenly into the dorsal face; dorsal face rounding evenly into the short posterior face, which forms a ~110° angle with the caudal cylinder. Postpetiole evenly rounded anteriorly, convex dorsally, and weakly lobed ventrally.

In dorsal view, humeri poorly developed: evenly rounded and only slightly wider than the rest of the mesosoma; mesothoracic spiracles not protruding past the lateral margins of the mesosoma. Promesonotal suture visible as a weak sulcus. Metanotal groove absent: mesonotum and propodeum completely fused and lateral margins converging evenly to the bases of the propodeal spines. Propodeal spines broadly approximated basally and diverging apically, their apices separated from each other by about their length, the negative space between them “U” shaped. Petiolar peduncle with spiracles very weakly protruding past the lateral margins, not noticeably constricted anterior to them. Petiolar node campaniform: evenly convex anteriorly, flattened posteriorly; node slightly wider than the peduncle, but narrower than the caudal cylinder. Postpetiole moderately broad (PWI 191–208) and campaniform, articulating with most of the anterior margin of the gaster, leaving small, angulate margins on each side exposed. Anterior margin of the postpetiole flat and evenly rounds into the lateral margins, which diverge slightly to the angulate posterior corners; posterior margin flat. Metafemur weakly to moderately incrassate (FI 180–303).

Sculpture: median clypeal carina present, extending posteriorly nearly to the frontal triangle, and flanked on either side by a slightly weaker carinae. Lateral clypeal lobes with additional, weaker carinae; ground sculpture very weakly areolate. Antennal scapes weakly areolate. Cephalic dorsum finely areolate, but areolae arranged into longitudinal columns by costulae which flank the frontal carinae medially and extend to the posterior margin of the head; concentric costulae surrounding the antennal insertions. Lateral surfaces of head uniformly weakly areolate-costulate, with dense rugose sculpture between the compound eye and the mandibular insertion. Ventral surface of head smooth and shining anteriorly, weakly areolate posteriorly. Mesosoma uniformly areolate-rugulose, but sculpture surrounding the propodeal spiracle costulate; propodeal declivity shining through very weak areolate sculpture. Femora shining through traces of shallow areolate sculpture, which becomes stronger distally. First gastral tergite and sternite smooth and shining, with weak spectral iridescence.

Setae: antennal scapes and funiculi with short, decumbent pilosity. Dorsum of the head, pronotum, waist segments, and gaster with moderately abundant, erect, blunt-tipped setae, the longest of which are about the width of the compound eye. The head bears ~28, mesosoma ~20, petiole 6, postpetiole ~12, and first gastral tergite ~46 setae. Longer, flexuous setae on the gular region, and the ventrites of the gaster. Short, sparse pubescence present over the entire body, but difficult to detect against the densely sculptured integument.

Color: predominantly dark brown, with mandibles and antennae testaceous yellow. Femora medium brown. Trochanters, Meso- and metacoxae, tibiae, tarsi, and sting very light yellow, nearly white.

**Gyne:** Unknown.

**Male:** Unknown.

**Etymology:** Geographical, from ‘achi’, one of the many Mayan dialects spoken by the indigenous people of Guatemala.

**Comments:** *Temnothorax achii* sp. nov. has been collected from Winkler leaf litter extractions in several localities in Central Guatemala, spanning low-to-mid elevation tropical and mesic forests. This species is closely related to *T. ixili*, which is also known from low to mid elevation leaf litter extractions and nests in *Oncidium* orchids. They have not been collected at the same locality, but collections of *T. ixili* and *T. achii* sp. nov. were taken about 15 km apart from each other. I consider them sympatric species.

***Temnothorax goniops* (Baroni Urbani, 1978)**

**Figure 116:**
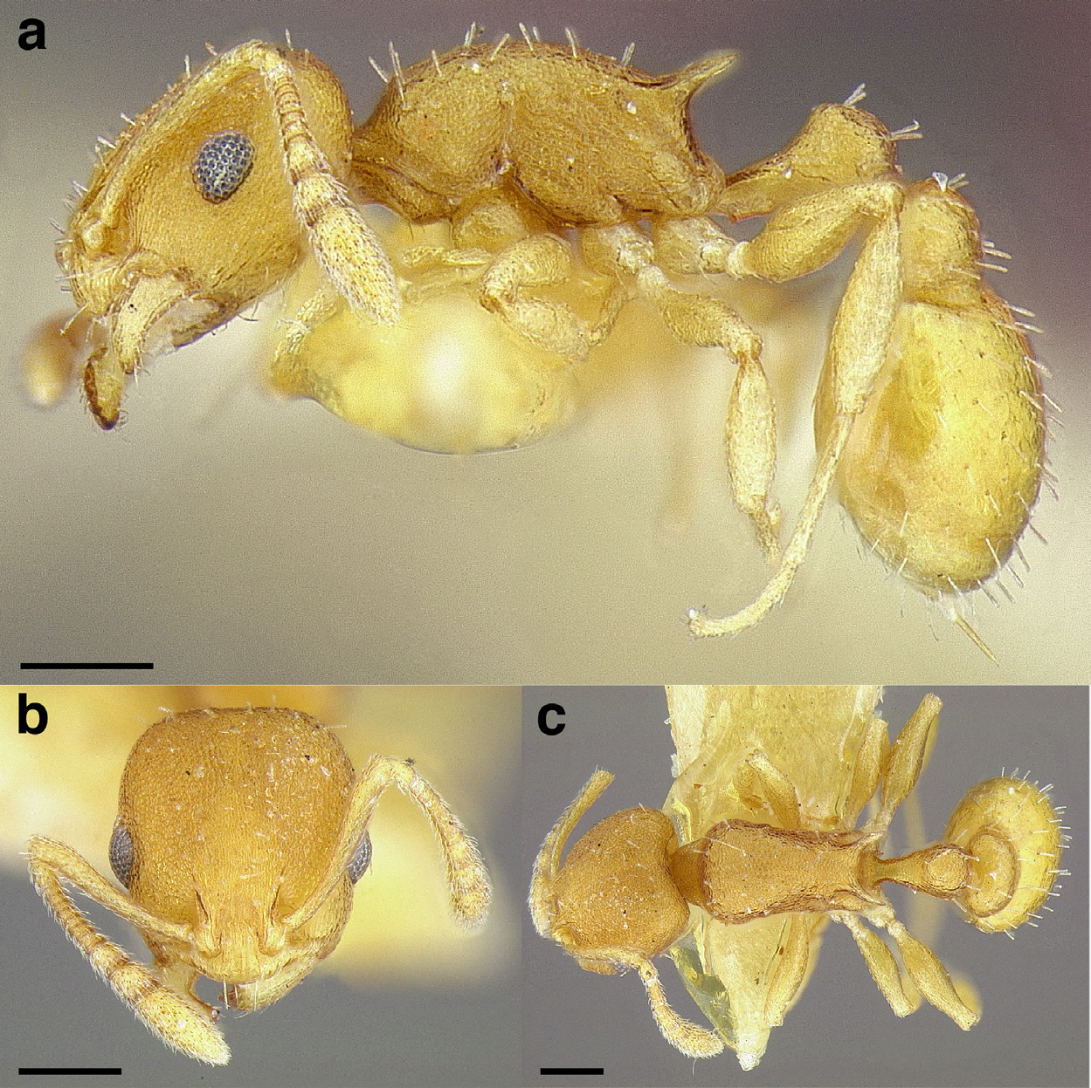
*Temnothorax goniops* holotype worker (MCZENT00032436). (A) Profile view. (B) Full-face view. (C) Dorsal view. Scale bars 0.2 mm.

Distribution: Fig. 114B; worker: Fig. 116.

*Leptothorax goniops* Baroni Urbani, 1978: 445, figs. 65, 100. Holotype worker. Campeche, Mexico.

*Temnothorax goniops* (Baroni Urbani): Bolton, 2003: 271. First combination in *Temnothorax*.

**Type material examined:** *Holotype worker:* MEXICO: Campeche: 10 km S Campeche, 28–29 June 1953, E.O. Wilson#125, 2nd growth thorn forest, strays, M.C.Z. Type 32436 (MCZ-ENT00032436, top) [MCZC].

*Paratype workers:* same pin as holotype, 1 worker (MCZ-ENT00032436, bottom) [MCZC]; same data as holotype, 1 worker (images of CASENT0912943 examined on antweb.org) [NHMB].

**Non-type material examined:** MEXICO: Chiapas: [no locality data], 8 March 1961, Jackson, 1 worker (CASENT0758363) [USNM].

**Geographic distribution:** Low elevations in southern and eastern Mexico (Fig. 114B).

**Worker diagnosis:** *Temnothorax goniops* can be separated from all other species in the *salvini* clade by the following character combination: antennal scapes short, failing to reach the posterior margin of the head capsule by about the width of the antennal scape (SI 80–83); compound eyes small (OI 22–24); body very compact (WLI < 130); metanotal groove absent; erect setae present on propodeum; propodeum not strongly depressed; propodeal spines about as long as the propodeal declivity (PSI 33–36). directed posterodorsally, and straight; propodeal spines broadly approximated in dorsal view (SBI > 30); hind femora weakly to moderately incrassate (FI 182–252); petiolar node erect and subquadrate, not overhanging the caudal cylinder in profile view; petiolar node compact, not elongate (NI 113–148); petiolar node broader than caudal cylinder in dorsal view (PNWI 123–140); postpetiole moderately broad (150 < PWI < 220); head sculpture uniformly areolate; antennal scapes with short, decumbent pilosity; setae on head, mesosoma, waist segments and gaster erect, short, sparse and blunt (never long and tapering); integument yellow.

**Similar species:** *Temnothorax aureus* sp. nov., *T. aztecus, T. paraztecus* sp. nov., *T. pilicornis* sp. nov., *T. rugosus, T. subditivus* (light form), *T. terrigena, T. torrei*, species of the *annexus* and *goniops* groups. *Temnothorax goniops* differs from *T. aztecus* and *T. paraztecus* sp. nov. by, among many other characters, its smaller size (WL < 0.7 mm vs. > 1 mm) and lack of long, tapering setae on head, mesosoma, legs, and gaster. *Temnothorax goniops* can be distinguished from *T. pilicornis* sp. nov. by the pilosity on the antennal scapes, which is decumbent in *T. goniops*, as opposed to subdecumbent in *T. pilicornis* sp. nov. The pedunculate petiole with an erect subquadrate node will separate *T. goniops* from *T. aztecus, T. paraztecus* sp. nov., and *T. subditivus*, which have squamiform petiolar nodes, *T. aureus* sp. nov., which has a posteriorly leaning petiolar node, and *T. rugosus*, which has a cuneiform to subcuneiform petiolar node. *Temnothorax goniops* differs from *T. torrei* by the presence of four erect setae on the dorsum of the petiolar node, as opposed to two. *Temnothorax goniops* can be distinguished from *T. terrigena* by the relatively long propodeal spines, which are longer than the propodeal declivity (shorter than the declivity in *T. terrigena*). The members of the *annexus* group all have a medially emarginate anterior clypeal margin, which is entire and convex in *T. goniops*. *Temnothorax goniops* can be separated from other members of the *goniops* group by the yellow integument (bicolored in *T. huehuetenangoi*, and predominantly dark brown in *T. ixili, T. achii* sp. nov., and *T. xincai* sp. nov.), continuous dorsal margin of the mesosoma (the propodeum is depressed in *T. xincai* sp. nov.), long propodeal spines (shorter than the propodeal declivity in *T. ixili* and *T. achii* sp. nov.), and the uniformly areolate dorsum of the head (smooth and shining in *T. huehuetenangoi*).

**Worker measurements & indices (*n* = 3):** SL = 0.378–0.395 (0.384); FRS = 0.156–0.160 (0.158); CW = 0.497–0.524 (0.510); CWb = 0.453–0.488 (0.472); PoOC = 0.212–0.214 (0.213); CL = 0.520–0.562 (0.544); EL = 0.120–0.130 (0.126); EW = 0.089–0.093 (0.092); MD = 0.116–0.125 (0.121); WL = 0.558–0.613 (0.594); SPST = 0.185–0.222 (0.206); MPST = 0.196–0.202 (0.198); PEL = 0.263–0.275 (0.267); NOL = 0.137–0.148 (0.143); NOH = 0.097–0.121 (0.109); PEH = 0.190–0.204 (0.197); PPL = 0.139–0.180 (0.165); PPH = 0.161–0.189 (0.172); PW = 0.317–0.341 (0.331); SBPA = 0.147–0.161 (0.155); SPTI = 0.219–0.260 (0.234); PEW = 0.137–0.144 (0.141); PNW = 0.174–0.201 (0.185); PPW = 0.273–0.288 (0.280); HFL = 0.311–0.396 (0.357); HFWmax = 0.089–0.109 (0.101); HFWmin = 0.042–0.049 (0.045); CS = 0.713–0.769 (0.744); ES = 0.167–0.175 (0.172); SI = 80–83 (81); OI = 22–24 (23); CI = 87; WLI = 123–129 (126); SBI = 32–33 (33); PSI = 33–36 (35); PWI = 194–204 (199); PLI = 146–198 (165); NI = 113–148 (132); PNWI = 123–140 (132); NLI = 50–56 (54); FI = 182–252 (225).

**Worker description:** In full-face view, head subquadrate, longer than broad (CI 87). Mandibles densely, finely striate but shining and armed with five teeth: the apical-most well developed and acute, followed by a less developed preapical tooth and three equally developed smaller teeth. Anterior clypeal margin evenly convex medially. Antennal scapes short: when fully retracted, failing to reach the posterior margin of the head capsule by about the width of the antennal scape (SI 80–83). Antennae 12-segmented; antennal club of composed of three segments, with the apical-most segment nearly twice as long as the preceding two in combination. Frontal carinae moderately long, extending past the antennal toruli by about two times the maximum width of the antennal scape. Compound eyes moderately protruding past the lateral margins of the head capsule. Lateral margin of head weakly convex, nearly flat, forming a continuous arc from the mandibular insertions to the posterior margin of the head. Posterior head margin weakly emarginate, but predominantly flat and rounding evenly into the lateral margins.

In profile view, compound eyes ovular and moderately large (OI 22–24), with 9 ommatidia in longest row. Pronotal declivity indistinct, but junction of anterior and dorsal faces marked by a change in ground sculpture; neck and anterior face of pronotum forming a ~120° angle; anterior face and dorsal face forming a rounded ~130° angle. Mesosoma evenly convex from where it joins the pronotal declivity to the propodeal spines. Promesonotal suture extending from the posterior margin of the procoxal insertion only to the mesothoracic spiracle, which is moderately well developed. Metanotal groove visible as a disruption of the sculpture laterally from where it arises between the mid- and hind coxae to where it ends in the poorly developed metathoracic spiracle, which is nearly indistinguishable against the ground sculpture. Propodeal spiracle moderately well developed, directed posterolaterally, and separated from the propodeal declivity by about four spiracle diameters. Propodeal spines well developed and long (PSI 33–36), longer than the propodeal declivity, flared at the base, slightly upcurved at the apices, and acute. Propodeal declivity flat, with a lamina between the base of the propodeal spine and the propodeal lobe, forming a rounded ~100° angle with the base of the propodeal spines. Propodeal lobes rounded and weakly developed. Metapleural gland bulla small, extending from the metacoxal insertion halfway to the propodeal spiracle. Petiole moderately long (PLI 146–198), without tubercles anterodorsally. Subpetiolar process in the form of a small, acute tooth; ventral margin of petiole flat posterior to it. Petiolar peduncle moderately long: comprising about half the length of the petiole. Petiolar node robust and subquadrate: transition between peduncle and node marked by a rounded angle of ~130°, resulting in a concave anterior node face; anterior face forming a ~100° angle with the dorsal face, which is weakly convex; dorsal face rounding evenly into the short posterior face, which forms a ~90° angle with the caudal cylinder. Postpetiole flat anteriorly, rounding into the weakly convex dorsal face; weakly lobed ventrally.

In dorsal view, humeri weakly developed: evenly rounded and slightly wider than the rest of the mesosoma; mesothoracic spiracles weakly protruding past the lateral margins of the mesosoma, visible as slight angles where the pronotum meets the mesonotum. Metanotal groove absent: mesonotum and propodeum completely fused and lateral margins converging evenly to the bases of the propodeal spines. Propodeal spines broadly approximated basally and diverging apically, their apices separated from each other by about their length, the negative space between them “U” shaped. Petiolar peduncle with spiracles not protruding past the lateral margins. Petiolar node campaniform: evenly rounded anteriorly, and very weakly convex posteriorly, nearly flat. Node wider than the peduncle, and about one and a quarter times as wide as the caudal cylinder. Postpetiole moderately broad (PWI 194–204) and campaniform, articulating with most of the anterior margin of the gaster, leaving small, angulate margins on each side exposed. Anterior margin of the postpetiole weakly emarginate medially, and evenly rounds into the lateral margins, which diverge slightly to the angulate posterior corners; posterior margin flat. Metafemur weakly to moderately incrassate (FI 182–252).

Sculpture: median clypeal carina present, extending posteriorly nearly to the frontal triangle, and flanked on either side by two equally strong carinae. Lateral clypeal lobes with additional, weaker carinae; ground sculpture weakly areolate. Antennal scapes areolate. Cephalic dorsum uniformly densely areolate, with areolae organized into longitudinal rows by costulae, which become stronger near the frontal carinae; fine, concentric costulae surrounding the antennal insertions. Lateral surface of head densely areolate, with rugulose sculpture between the compound eye and the mandibular insertion. Ventral surface of head weakly areolate. Mesosoma uniformly areolate laterally, with fine costulae on the lateral face of the propodeum, meso- and metapleurae. Propodeal declivity shining through weakly areolate sculpture. Dorsal surface of mesosoma uniformly areolate, with rugulose sculpture on the pronotum. Femora shining through weak areolate sculpture. Petiole and postpetiole uniformly areolate; petiolar node costulate. First gastral tergite smooth and shining, with weak spectral iridescence. First gastral sternite smooth and shining.

Setae: antennal scapes and funiculi with moderately long, decumbent pilosity. Dorsum of the head, pronotum, waist segments, and gaster with moderately abundant, erect, blunt-tipped setae, the longest of which are about the width of the compound eye. The head bears ~30, mesosoma ~20, petiole 6, postpetiole ~14, and first gastral tergite ~30 setae. Short, sparse pubescence present over the entire body, but difficult to detect against the lightly colored integument and dense sculpture.

Color: predominantly yellow, with masticatory margin of the mandible dark brown.

**Gyne:** Unknown.

**Male:** Unknown.

**Etymology:** Morphological, from the Greek ‘gonia’ (= angle) + ‘opsis’ (= sight), a reference to the weak anterior angle on the compound eyes.

**Comments:** Little is known about the biology of *Temnothorax goniops*. It appears to be distributed widely across Southern Mexico and the Yucatán peninsula: in addition to the type locality in Campeche and the collection from Chiapas noted by Baroni Urbani (1978), *T. goniops* has been reported from evergreen rainforest in Quintana Roo state, specifically the Mayan ruins at San Gervasio on the Isla de Cozumel by Reynoso-Campos, Rodríguez-Garza & Vásquez-Bolaños (2015), and the Reserva Ecologica de San Felipe Bacalar by Rodríguez-Garza (2015). Additionally, Vásquez-Bolaños (2011) records *T. goniops* from Yucatán state. I have not been able to examine these specimens personally, so I exclude them here.

***Temnothorax huehuetenangoi* (Baroni Urbani, 1978)**

Worker: Fig. 117.

**Figure 117:**
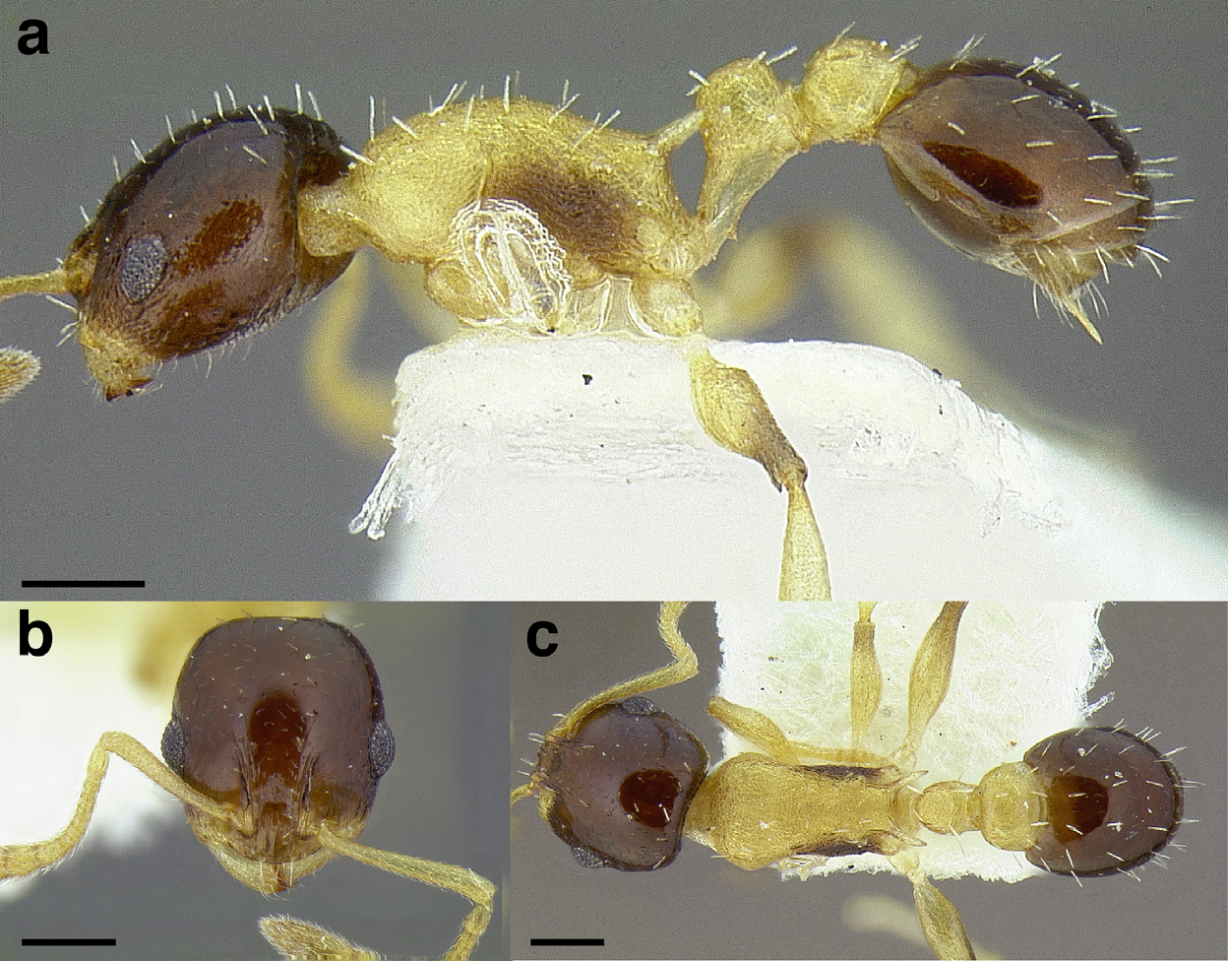
*Temnothorax huehuetenangoi* holotype worker (USNMENT00529517). (A) Profile view. (B) Full-face view. (C) Dorsal view. Scale bars 0.2 mm.

*Leptothorax huehuetenangoi* Baroni Urbani, 1978: 451, figs. 64, 106. Holotype worker. San de José, Guatemala.

*Temnothorax huehuetenangoi* (Baroni Urbani): Bolton, 2003: 271. First combination in *Temnothorax*.

**Type material examined:** *Holotype worker:* GUATEMALA: San de José [intercepted in quarantine, San Francisco, California, U.S.A.], 11 April 1946, SF 20724, 46-5347, ex *Odontoglossum bictoniense* (USNMENT00529517) [USNM].

**Geographic range:** Unknown. Intercepted in quarantine in cargo from Guatemala.

**Worker diagnosis:** *Temnothorax huehuetenangoi* can be separated from all other species in the *salvini* clade by the following character combination: antennal scapes short, failing to reach the posterior margin of the head capsule by about the width of the antennal scape (SI 84); compound eyes small (OI 24); head sculpture smooth and shining; body compact (WLI < 140); metanotal groove absent; erect setae present on propodeum; propodeum not strongly depressed; propodeal spines about as long as the propodeal declivity (PSI 32), directed posterodorsally, and straight; propodeal spines broadly approximated in dorsal view (SBI 30); hind femora moderately incrassate (FI 298); petiolar node erect and subquadrate, not overhanging the caudal cylinder in profile view; petiolar node compact, not elongate (NI 145); petiolar node barely broader than caudal cylinder in dorsal view (PNWI 117); postpetiole moderately broad (150 < PWI < 220); antennal scapes with short, decumbent pilosity; setae on head, mesosoma, waist segments and gaster erect, short, sparse and blunt (never long and tapering); integument bicolored: antennae, mandibles, mesosoma, legs, and waist segments mostly yellow, with head capsule, meso- and metapleurae, and gaster dark testaceous brown.

**Similar species:** *Temnothorax casanovai* sp. nov., *T. leucacanthoides* sp. nov., *T. leucacanthus, T. ocarinae, T. parvidentatus* sp. nov., *T. tenuisculptus*, species of the *goniops* and *pulchellus* groups. *Temnothorax huehuetenangoi* differs from all of the above species, except for *T. tenuisculptus, T. xincai* sp. nov. of the *goniops* group, and some members of the *pulchellus* group (e.g., *T. agavicola* sp. nov., *T. albispinus, T. laticrus* sp. nov., *T. torrei*, and *T. wettereri* sp. nov.) by the smooth and shining sculpture of the head. Additionally, the structure of the petiolar node, which is erect in *T. huehuetenangoi*, will distinguish it from *T. leucacanthus* and *T. leucacanthoides* sp. nov., in which the petiolar node leans posteriorly over the caudal cylinder. The short, sparse, blunt setae separates *T. huehuetenangoi* from *T. casanovai* sp. nov. *Temnothorax ocarinae* can differentiated by the relatively long petiolar node (NI ~ 194 vs. 145 in *T. huehuetenangoi*). The weakly incrassate femora distinguishes *T. huehuetenangoi* from many of the species above, including *T. tenuisculptus, T. casanovai* sp. nov., and *T. ocarinae*. The moderately broad postpetiole (in dorsal view) will separate *T. huehuetenangoi* from all members of the *pulchellus* group (PWI < 210). *Temnothorax huehuetenangoi* can be distinguished from other members of the *goniops* group by the bicolored integument (yellow in *T. goniops*, and predominantly dark brown in *T. ixili, T. achii* sp. nov., and *T. xincai* sp. nov.), the relatively long, spiniform propodeal spines (short and dentate in *T. ixili, T. achii* sp. nov., and *T. xincai* sp. nov.), and the smooth and shining sculpture of the head (areolate to rugulose in the rest of the *goniops* group, except *T. xincai* sp. nov.).

**Worker measurements & indices (*n* = 1):** SL = 0.392; FRS = 0.149; CW = 0.516; CWb = 0.465; PoOC = 0.206; CL = 0.538; EL = 0.130; EW = 0.094; MD = 0.110; WL = 0.633; SPST = 0.202; MPST = 0.211; PEL = 0.269; NOL = 0.161; NOH = 0.111; PEH = 0.182; PPL = 0.154; PPH = 0.176; PW = 0.332; SBPA = 0.141; SPTI = 0.207; PEW = 0.127; PNW = 0.148; PPW = 0.261; HFL = 0.420; HFWmax = 0.119; HFWmin = 0.040; CS = 0.734; ES = 0.177; SI = 84; OI = 24; CI = 86; WLI = 136; SBI = 30; PSI = 32; PWI = 206; PLI = 175; NI = 145; PNWI = 117; NLI = 60; FI = 298.

**Worker description:** In full-face view, head subquadrate, longer than broad (CI 86). Mandibles densely striate but shining and armed with five teeth: the apical-most well developed and acute, followed by a less developed preapical tooth and three equally developed smaller teeth. Anterior clypeal margin evenly convex medially Antennal scapes short: when fully retracted, failing to reach the posterior margin of the head capsule by about the maximum width of the scape (SI 84). Antennae 12-segmented; antennal club of composed of three segments, with the apical-most segment longer than the preceding two in combination. Frontal carinae long, extending past the antennal toruli by about three times the maximum width of the antennal scape. Compound eyes moderately protruding past the lateral margins of the head capsule. Lateral margin of head convex, forming a continuous arc from the mandibular insertions to the posterior margin of the head. Posterior head margin flat but rounding evenly into the lateral margins.

In profile view, compound eyes ovular, longitudinally elongate, and moderately large (OI 24), with 10 ommatidia in longest row. Pronotal declivity indistinct, neck and anterior face of pronotum forming a ~120° angle. Mesosoma convex from where it joins the pronotal neck to the propodeal spines, but propodeum slightly flattened, giving the dorsal margin a weakly sinuate shape. Promesonotal suture extending from the posterior margin of the procoxal insertion only to the mesothoracic spiracle, which is moderately well developed. Metanotal groove visible as a disruption of the sculpture laterally from where it arises between the mid- and hind coxae to where it ends in the poorly developed metathoracic spiracle, which is nearly indistinguishable against the ground sculpture. Propodeal spiracle well developed, directed posterolaterally, and separated from the propodeal declivity by about three spiracle diameters. Propodeal spines well developed and moderately long (PSI 32), about as long as the propodeal declivity, tapering evenly from the base, straight, and acute. Propodeal declivity flat, forming a rounded ~110° angle with the base of the propodeal spines. Propodeal lobes rounded and weakly developed. Metapleural gland bulla small, extending from the metacoxal insertion halfway to the propodeal spiracle. Petiole moderately long (PLI 175), without tubercles anterodorsally. Subpetiolar process in the form of a small, triangular, acute tooth; ventral margin of petiole weakly bulging medially. Petiolar peduncle short: comprising about a third of the petiole length. Petiolar node robust and subquadrate: transition between peduncle and node marked by a rounded angle of ~140°, resulting in a weakly concave anterior node face; anterior face forming a ~110° angle with the dorsal face, which is weakly convex; dorsal face weakly convex, forming a rounded ~90° angle with the posterior face, which forms a ~90° angle with the caudal cylinder. Postpetiole evenly rounded anteriorly, convex dorsally, and weakly lobed ventrally.

In dorsal view, humeri moderately well developed: evenly rounded and slightly wider than the rest of the mesosoma; mesothoracic spiracles weakly protruding past the lateral margins of the mesosoma, visible as slight angles where the pronotum meets the mesonotum. Metanotal groove absent: mesonotum and propodeum completely fused and lateral margins converging evenly to the bases of the propodeal spines. Propodeal spines broadly approximated basally and diverging apically, their apices separated from each other by about their length, the negative space between them “U” shaped. Petiolar peduncle with spiracles barely protruding past the lateral margins. Petiolar node evenly ovular, slightly longitudinally elongate, and narrowed anteriorly; node wider than the peduncle, and slightly broader than the caudal cylinder. Postpetiole moderately broad (PWI 206) and campaniform, articulating with most of the anterior margin of the gaster, leaving small, angulate margins on each side exposed. Anterior margin of the postpetiole flat and evenly rounds into the lateral margins, which slightly diverge to the angulate posterior corners; posterior margin flat. Metafemur moderately incrassate (FI 298).

Sculpture: median clypeal carina present, extending posteriorly nearly to the frontal triangle, and flanked on either side by two equally strong carinae. Lateral clypeal lobes with additional, weaker carinae; ground sculpture smooth and shining. Antennal scapes shining through weak areolate ground sculpture. Cephalic dorsum smooth and shining, but with coarse piligerous punctures; very fine costulae flanking the frontal carinae medially. Lateral surfaces of head with weak areolate sculpture posterior to the compound eye, fine rugulose sculpture surrounding the compound eye, and fine, dense rugose sculpture between the compound eye and the mandibular insertion. Ventral surface of head smooth and shining, but with weak areolate sculpture posteromedially. Pronotal neck areolate. Lateral surfaces mesosoma areolate, with the sculpture arranged into longitudinal rows on the pronotum by costulae. Propodeal declivity weakly areolate. Dorsal surface of mesosoma uniformly areolate. Femora weakly areolate and shining feebly. Petiole predominantly areolate, but smooth and shining ventrally. Postpetiole predominantly weakly areolate-costulate, but smooth and shining anteromedially. First gastral tergite smooth and shining, with weak spectral iridescence. First gastral sternite smooth and shining.

Setae: antennal scapes and funiculi moderately long, decumbent pilosity. Dorsum of the head, pronotum, waist segments, and gaster with moderately abundant, erect, blunt-tipped setae, the longest of which are slightly shorter than the length of the compound eye. The head bears ~34, mesosoma ~18, petiole 6, postpetiole ~10, and first gastral tergite ~30 setae. Long, subdecumbent pubescence surrounding the gular region. Short, sparse pubescence present over the entire body, but difficult to detect against the lightly colored integument and dense ground sculpture.

Color: predominantly yellow, with head capsule, meso- and metapleurae, and gaster (except for the basal quarter) dark testaceous brown. Distal quarters of the femora light brown.

**Gyne:** Unknown.

**Male:** Unknown.

**Etymology:** Geographical, a reference to the municipality of Huehuetenango in the highlands of Western Guatemala.

**Comments:** Very little is known about the biology of *Temnothorax huehuetenangoi*. It was intercepted in quarantine from the orchid *Odontoglossum bictoniense* (now *Rhynchostele bictoniensis*). The taxonomic affinities of this species remain unclear, although it is similar in habitus to *T. tenuisculptus*, a,lthough more lightly sculptured and bicolored.

***Temnothorax ixili* (Baroni Urbani, 1978)**

**Figure 118:**
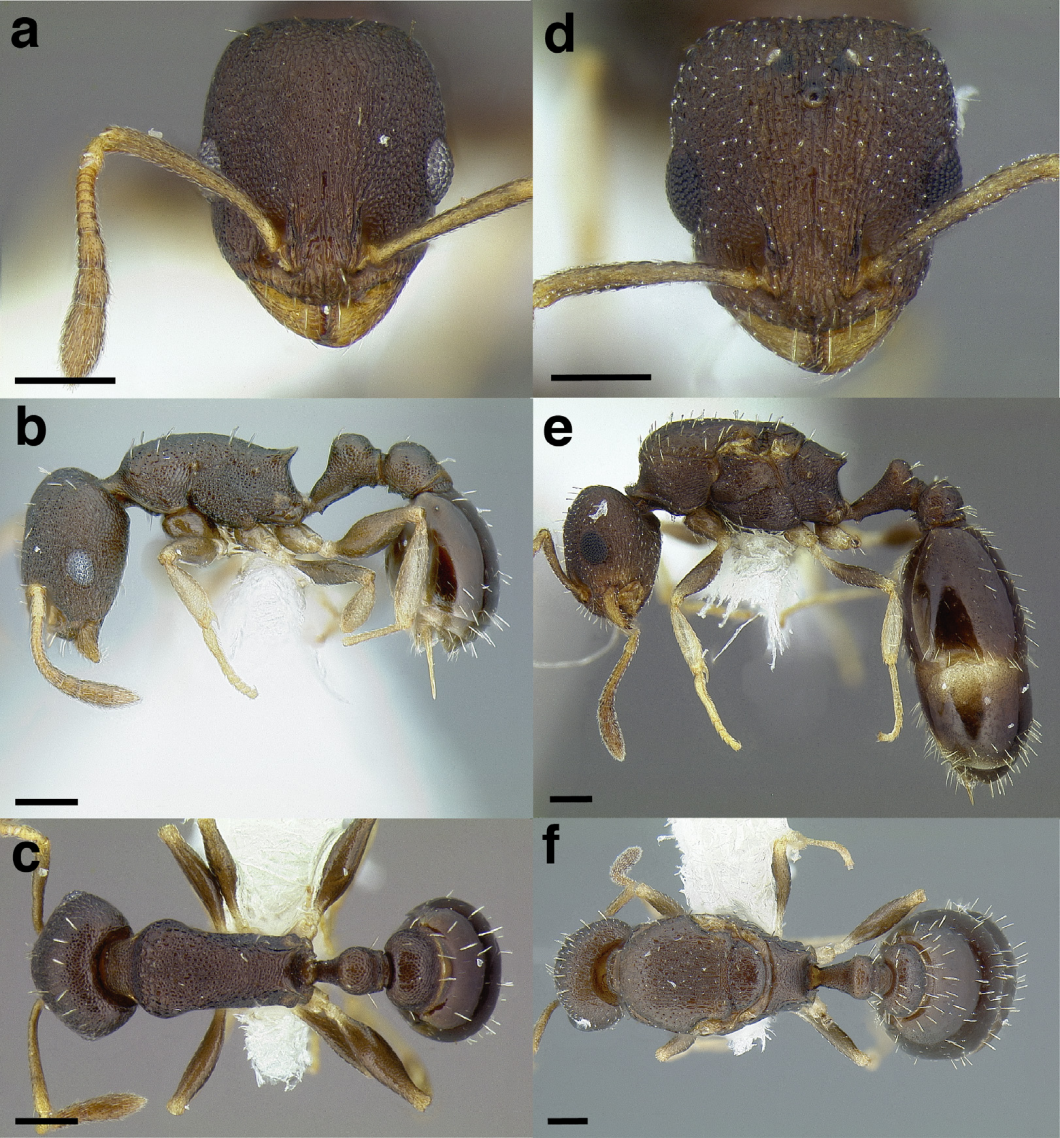
*Temnothorax ixili*. (A–C) Worker (CASENT0611678). (A) Full-face. (B) Profile view. (C) Dorsal view. (D–F) Gyne (CASENT0611679). (D) Full-face. (E) Profile view. (F) Dorsal view. Scale bars 0.2 mm.

Distribution: Fig. 114C; worker & gyne: Fig. 118.

*Leptothorax ixili* Baroni Urbani, 1978: 459, figs. 67, 111. Holotype worker. Guatemala.

*Temnothorax ixili* (Baroni Urbani): Bolton, 2003: 271. First combination in *Temnothorax*.

**Type material examined:** *Holotype worker:* GUATEMALA: [intercepted in quarantine: San Francisco, California, U.S.A.] 30 April 1946, SF 20968, 46-5352, *Oncidium* sp., pin includes teneral gyne which is missing a head (USNMENT00529550) [USNM].

*Paratype workers:* same data as holotype, 3 workers (CASENT0758364) [USNM] 3 workers (LACMENT323191) [LACM]; [intercepted in quarantine] 6 May 1938, EQR4815, *Oncidium* sp., 3 workers (bearing a label reading “PARATYPUS”, but unreported in Baroni Urbani, 1978, MCZ-ENT00581829) [MCZC] 1 worker (images of CASENT0912951 examined on antweb.org) [NHMB].

**Non-type material examined:** GUATEMALA: Zacapa: 7.5 km NE Teculután, 15.04367°N 89.67501°W ± 30 m, 475 m, 28 June 2009, J. Longino#JTL6795-s, stream gully in dry scrub, ex sifted leaf litter, 1 worker (CASENT0611673) [JTLC] 1 worker (CASENT0611669) [JTLC] 1 worker (CASENT0611665) [JTLC]; 16 km NE Teculután, 14.86383°N 89.78801°W ± 57 m, 600 m, 24 June 2009, C. Beza#MGB1338, tropical thorn scrub, ex sifted leaf litter, 1 dealate gyne (CASENT0611679) [JTLC] 1 worker (CASENT0611678) [JTLC] 1 worker (CASENT0732589) [JTLC].

**Geographic range:** Low elevations of Guatemala (Zacapa) (Fig. 114C).

**Worker diagnosis:** *Temnothorax ixili* can be separated from all other species in the *salvini* clade by the following character combination: antennal scapes moderately long, just reaching the posterior margin of the head capsule (SI 83–92); compound eyes small (OI 24–25); head sculpture areolate, with areolae arranged into columns by weak longitudinal striae; body moderately compact (WLI 137–148); metanotal groove absent; propodeum not strongly depressed; propodeal spines shorter than the propodeal declivity and dentate (PSI 22–26); propodeal spines broadly approximated in dorsal view (SBI 30–34); hind femora weakly to moderately incrassate (FI 245–300); petiolar node erect and dorsally rounded, not overhanging the caudal cylinder in profile view; petiolar node compact, not elongate (NI 137–160); petiolar node slightly broader than caudal cylinder in dorsal view (PNWI > 115); dorsum of postpetiole sculptured; antennal scapes with short, decumbent pilosity; setae on head, mesosoma, waist segments and gaster erect, short, sparse and blunt (never long and tapering); integument predominantly dark brown, with trochanters, meso- and metacoxae, tibiae, tarsi, and sting very light yellow.

**Similar species:** *Temnothorax acuminatus* sp. nov., *T. acutispinosus* sp. nov., *T. subditivus, T. tuxtlanus* sp. nov., species of the *augusti*, *goniops*, and *pulchellus* groups, species of the *sallei* clade. *Temnothorax ixili* can be distinguished from similar appearing members of the *sallei* clade (e.g., *T. manni, T. mexicanus, T. punctithorax*) by the petiole, which lacks a transverse carina anterodorsally. Unlike the long, dorsally directed propodeal spines of *T. acutispinosus* sp. nov., *T. ixili* has short propodeal teeth that are directed posterodorsally. *Temnothorax ixili* has a moderately broad postpetiole in dorsal view, whereas *T. tuxtlanus* sp. nov. has a relatively narrow postpetiole (PWI < 150). *Temnothorax altinodus* sp. nov. has comparatively strongly incrassate hind femora (FI 327). *Temnothorax ixili* can be separated from *T. subditivus* and the *augusti* group by the structure of the petiolar node, which is erect, dorsally rounded, and only slightly broader than the caudal cylinder in dorsal view, as opposed to squamiform in *T. subditivus*, or leaning posteriorly over the caudal cylinder in the *augusti* group. *Temnothorax ixili* can be distinguished from the *pulchellus* group by the combination of a relatively longer mesosoma (WLI > 135 vs. < 135 in most *pulchellus* group members) and the relatively broad petiolar node in dorsal view (PNWI > 115 vs. < 115 in most *pulchellus* group members). *Temnothorax hippolytus* sp. nov. from Cuba and *T. albispinus* from Puerto Rico may approach these dimensions, but they can be separated from *T. ixili* by their very broad postpetioles (PWI > 240), among other features. *Temnothorax ixili* can be distinguished from other *goniops* group members by the short, dentate propodeal spines (longer than the propodeal declivity and spiniform in *T. goniops* and *T. huehuetenangoi*), the sculptured head and postpetiole (smooth and shining in *T. xincai* sp. nov.), and the relatively broad, areolate petiolar node (narrower, on average, in *T. achii* sp. nov., and with overlying rugae).

**Worker measurements & indices (*n* = 9)**: SL = 0.407–0.451 (0.427); FRS = 0.152–0.172 (0.163); CW = 0.491–0.551 (0.524); CWb = 0.459–0.514 (0.485); PoOC = 0.215–0.251 (0.234); CL = 0.559–0.607 (0.583); EL = 0.130–0.143 (0.136); EW = 0.096–0.112 (0.104); MD = 0.100–0.130 (0.119); WL = 0.654–0.742 (0.693); SPST = 0.157–0.187 (0.170); MPST = 0.209–0.235 (0.221); PEL = 0.262–0.309 (0.288); NOL = 0.149–0.185 (0.167); NOH = 0.101–0.124 (0.113); PEH = 0.137–0.218 (0.189); PPL = 0.157–0.199 (0.176); PPH = 0.168–0.277 (0.204); PW = 0.325–0.365 (0.345); SBPA = 0.139–0.169 (0.154); SPTI = 0.171–0.226 (0.202); PEW = 0.117–0.151 (0.137); PNW = 0.149–0.194 (0.175); PPW = 0.254–0.324 (0.294); HFL = 0.421–0.480 (0.454); HFWmax = 0.115–0.138 (0.123); HFWmin = 0.040–0.049 (0.044); CS = 0.742–0.818 (0.776); ES = 0.178–0.197 (0.188); SI = 83–92 (88); OI = 24–25 (24); CI = 80–85 (83); WLI = 137–148 (143); SBI = 30–34 (32); PSI = 22–26 (25); PWI = 197–233 (214); PLI = 143–180 (164); NI = 137–160 (148); PNWI = 118–138 (128); NLI = 55–64 (58); FI = 245–300 (279).

**Worker description:** In full-face view, head subquadrate, longer than broad (CI 80–85). Mandibles densely, finely striate but shining and armed with five teeth: the apical-most well developed and acute, followed by a less developed preapical tooth and three equally developed smaller teeth. Anterior clypeal margin evenly convex medially. Antennal scapes short: when fully retracted, just reaching the posterior margin of the head capsule (SI 83–92). Antennae 12-segmented; antennal club of composed of three segments, with the apical-most segment slightly longer than the preceding two in combination. Frontal carinae moderately long, extending past the antennal toruli by about two and a half times the maximum width of the antennal scape. Compound eyes moderately protruding past the lateral margins of the head capsule. Lateral margin of head weakly convex, nearly flat, forming a continuous low arc from the mandibular insertions to the posterior margin of the head. Posterior head margin flat but rounding evenly into the lateral margins.

In profile view, compound eyes ovular, longitudinally elongate, and moderately large (OI 24–25), with 10 ommatidia in longest row. Pronotal declivity indistinct, but neck and anterior face of pronotum forming a ~120° angle. Mesosoma very weakly from where it joins the pronotal neck to the propodeal spines; propodeum slightly flatter, giving the dorsum of the mesosoma a weakly sinuate profile. Promesonotal suture extending from the posterior margin of the procoxal insertion only to the mesothoracic spiracle, which is moderately well developed. Metanotal groove visible as a disruption of the sculpture laterally from where it arises between the mid- and hind coxae to where it ends in the poorly developed metathoracic spiracle, which is nearly indistinguishable against the ground sculpture. Propodeal spiracle moderately well developed, directed posterolaterally, and separated from the propodeal declivity by about three spiracle diameters. Propodeal spines poorly developed and short (PSI 22–26), half the length of the propodeal declivity, triangular, flared at the base, slightly upturned, and acute. Propodeal declivity with weak carinae extending between the base of the propodeal spines and the propodeal lobes; declivity straight, forming a rounded ~110° angle with the base of the propodeal spines. Propodeal lobes rounded and weakly developed. Metapleural gland bulla small, extending from the metacoxal insertion slightly less than halfway to the propodeal spiracle. Petiole moderately long (PLI 143-180), without tubercles anterodorsally. Subpetiolar process in the form of a small, triangular, blunt tooth; ventral margin of petiole flat posterior to it. Petiolar peduncle short: comprising about a third of the petiole length. Petiolar node robust and subquadrate: transition between peduncle and node marked by a rounded angle of ~120°, resulting in a concave anterior node face; anterior face forming a ~90° angle with the dorsal face, which is convex; dorsal face rounding evenly into the short posterior face, which forms a ~100° angle with the caudal cylinder. Postpetiole evenly rounded anteriorly, convex dorsally, and weakly lobed ventrally.

In dorsal view, humeri poorly developed: evenly rounded and only slightly wider than the rest of the mesosoma; mesothoracic spiracles not protruding past the lateral margins of the mesosoma. Metanotal groove absent: mesonotum and propodeum completely fused and lateral margins converging evenly to the bases of the propodeal spines. Propodeal spines broadly approximated basally and diverging apically, their apices separated from each other by about twice their length, the negative space between them “U” shaped. Petiolar peduncle with spiracles very weakly protruding past the lateral margins, not noticeably constricted anterior to them. Petiolar node campaniform: evenly convex anteriorly, flattened posteriorly; node wider than the peduncle, and about one and quarter times as wide as the caudal cylinder. Postpetiole moderately to very broad (PWI 197–233) and campaniform, articulating with most of the anterior margin of the gaster, leaving small, angulate margins on each side exposed. Anterior margin of the postpetiole flat and evenly rounds into the lateral margins, which are parallel to the angulate posterior corners; posterior margin flat. Metafemur moderately incrassate (FI 245–300).

Sculpture: median clypeal carina present, extending posteriorly nearly to the frontal triangle, and flanked on either side by two slightly weaker carinae. Lateral clypeal lobes with additional, weaker carinae; ground sculpture weakly areolate. Antennal scapes weakly areolate. Cephalic dorsum evenly areolate and arranged into longitudinal columns by costulae between the frontal carinae. Lateral surfaces of head uniformly areolate, with dense rugulose sculpture between the compound eye and the mandibular insertion. Ventral surface of head smooth and shining anteriorly, weakly areolate posteriorly. Mesosoma and waist segments uniformly areolate, with areolae arranged into longitudinal rows by costulae on the lateral face of the pronotum, and very fine costulae on the dorsal face of the pronotum; propodeal declivity weakly areolate. Femora shining through traces of shallow areolate sculpture. First gastral tergite smooth and shining, with weak spectral iridescence. First gastral sternite smooth and shining.

Setae: antennal scapes and funiculi with short, decumbent pilosity. Dorsum of the head, pronotum, waist segments, and gaster with moderately abundant, erect, blunt-tipped setae, the longest of which are about the width of the compound eye. The head bears ~24, mesosoma ~20, petiole 6, postpetiole ~12, and first gastral tergite ~38 setae. Longer, flexuous setae on the gular region, and the ventrites of the gaster. Short, sparse pubescence present over the entire body, but difficult to detect against the densely sculptured integument.

Color: predominantly medium brown, with mandibles and antennae testaceous yellow. Trochanters, Meso- and metacoxae, tibiae, tarsi, and sting very light yellow, nearly white.

**Gyne measurements & indices (*n* = 1):** SL = 0.434; FRS = 0.192; CW = 0.585; CWb = 0.560; PoOC = 0.245; CL = 0.628; EL = 0.183; EW = 0.142; MD = 0.109; WL = 1.035; SPST = 0.202; MPST = 0.268; PEL = 0.352; NOL = 0.201; NOH = 0.121; PEH = 0.238; PPL = 0.196; PPH = 0.253; PW = 0.622; SBPA = 0.273; SPTI = 0.257; PEW = 0.172; PNW = 0.228; PPW = 0.376; HFL = 0.527; HFWmax = 0.118; HFWmin = 0.047; CS = 0.874; ES = 0.254; SI = 78; OI = 29; CI = 89; WLI = 185; SBI = 49; PSI = 20; PWI = 219; PLI = 180; NI = 166; PNWI = 133; NLI = 57; FI = 251.

**Gyne description:** In full-face view, head subquadrate, about as long as broad (CI 89). Antennal scapes short: when fully retracted, failing to reach the posterior margin of the head capsule by about the maximum width of the scape (SI 78). Antennae 12-segmented; antennal club composed of three segments, with the apical-most segment as long as the preceding two in combination. Mandibles densely striate but shining and armed with five teeth: the apical-most well developed, followed by a less developed preapical tooth and three equally developed smaller teeth. Anterior clypeal margin evenly convex medially. Frontal carinae moderately long, extending past the antennal toruli by about three times the maximum width of the antennal scape. Compound eyes moderately protruding past the lateral margins of the head capsule. Lateral margin of head evenly convex, converging from below the compound eyes to the mandibular insertions. Posterior head margin flat, rounding evenly into the lateral margins.

In profile view, compound eyes ovular and large (OI 29), with 14 ommatidia in longest row. Mesoscutum rounded evenly anteriorly, covering the dorsal surface of the pronotum, and flat dorsally. Mesoscutellum at the same level as the mesoscutum. Posterior margin of metanotum extending past the posterior margin of the mesoscutum. Propodeal spiracle well developed, directed posterolaterally, and separated from the propodeal declivity by about four spiracle diameters. Propodeal teeth stout, poorly developed, and short (PSI 20), about a third as long as the propodeal declivity, triangular, directed posteriorly, straight, and blunt. Propodeal declivity with carinae that extend from the base of the propodeal spine to the propodeal lobes; declivity straight and flat, forming a ~90° angle with the base of the propodeal spines. Propodeal lobes rounded and very weakly developed. Metapleural gland bulla small, extending from the metacoxal insertion halfway to the propodeal spiracle. Petiole moderately long (PLI 180), without tubercles anterodorsally. Subpetiolar process in the form of a small, triangular, blunt tooth, which grades evenly into the ventral margin of the petiole posteriorly; ventral margin of petiole flat. Petiolar peduncle moderately long: comprising slightly less than half the length of the petiole. Petiolar node erect: transition between peduncle and node evenly rounded, resulting in a very slightly concave anterior node face; anterior face forming a rounded ~90° angle with the dorsal face, which is short and evenly convex; dorsal face rounding evenly into the short posterior face, which forms a ~100° angle with the caudal cylinder. Postpetiole evenly rounded anterodorsally, bulging slightly before it transitions into the flattened dorsal face; ventral surface weakly lobed.

In dorsal view, mesoscutum covering pronotum anteriorly, but humeri visible laterally as slightly angulate sclerites. Propodeal spines weakly diverging apically, their apices separated from each other by about three and a half times their length. Petiolar peduncle with spiracles not protruding past the lateral margins. Petiolar node trapezoidal: widest anteriorly, lateral faces converge to the slightly narrower posterior face. Petiolar node wider than the peduncle, and about one and a half times as wide as the caudal cylinder. Postpetiole moderately broad (PWI 219) and campaniform, articulating with most of the anterior margin of the gaster, leaving small, angulate margins on each side exposed. Anterior margin of postpetiole flat, with corners marked by rounded angles as it transitions to the lateral margins, which weakly diverge to the angulate posterior corners; posterior margin flat. Metafemur moderately incrassate (FI 251).

Sculpture: median clypeal carina present, but indistinct against the densely areolate-costulate ground sculpture. Lateral clypeal lobes with additional weaker carinae. Antennal scapes weakly areolate. Cephalic dorsum densely areolate-costate. Lateral surfaces of head with sculptured similarly to the cephalic dorsum, but with rugose sculpture between the compound eye and mandibular insertion. Ventral surface of head weakly areolate-costulate. Pronotal neck areolate. Mesosoma longitudinally areolate-costulate, but sculpture weakening on the anepisternum and katepisternum. Propodeal declivity weakly areolate. Mesoscutum areolate-costulate, with small patches of weaker sculpture anteromedially, and surrounding the wing bases. Mesoscutellum smooth and shining medially, surrounded by weak areolate-costulate sculpture. Femora shining, with traces of weak areolate sculpture that become stronger distally. Petiole and postpetiole uniformly areolate, with sculpture weaker on the anterior face of the postpetiole. Gaster smooth and shining, with weak spectral iridescence. Surface of the first gastral sternite smooth and shining.

Setae: antennal scapes and funiculi with short, decumbent pilosity. Dorsum of the head, pronotum, waist segments, and gaster with moderately abundant, erect, blunt-tipped setae, the longest of which are about a third of the width of the compound eye. Longer, flexuous setae on the gular region, and the ventrites of the gaster. Short, sparse pubescence present over the entire body, but difficult to detect against the densely sculptured integument.

Color: predominantly medium brown, with mandibles and antennae testaceous yellow. Wing bases, trochanters, meso- and metacoxae, tibiae, tarsi, and sting very light yellow, nearly white.

**Male:** Unknown.

**Etymology:** Geographical, from ‘ixil’, one of the many Maya dialects spoken by the indigenous people of Guatemala.

**Comments:** *Temnothorax ixili* has been collected from Winkler leaf litter extractions at low elevations in Zacapa state, Guatemala. The type specimens were intercepted in quarantine from *Oncidium* orchids. The presence of a gyne in a leaf litter extract suggests that this species may nest terrestrially, or at least is not strictly arboreal like many of the *salvini* clade species.

***Temnothorax xincai* sp. nov.**

**Figure 119:**
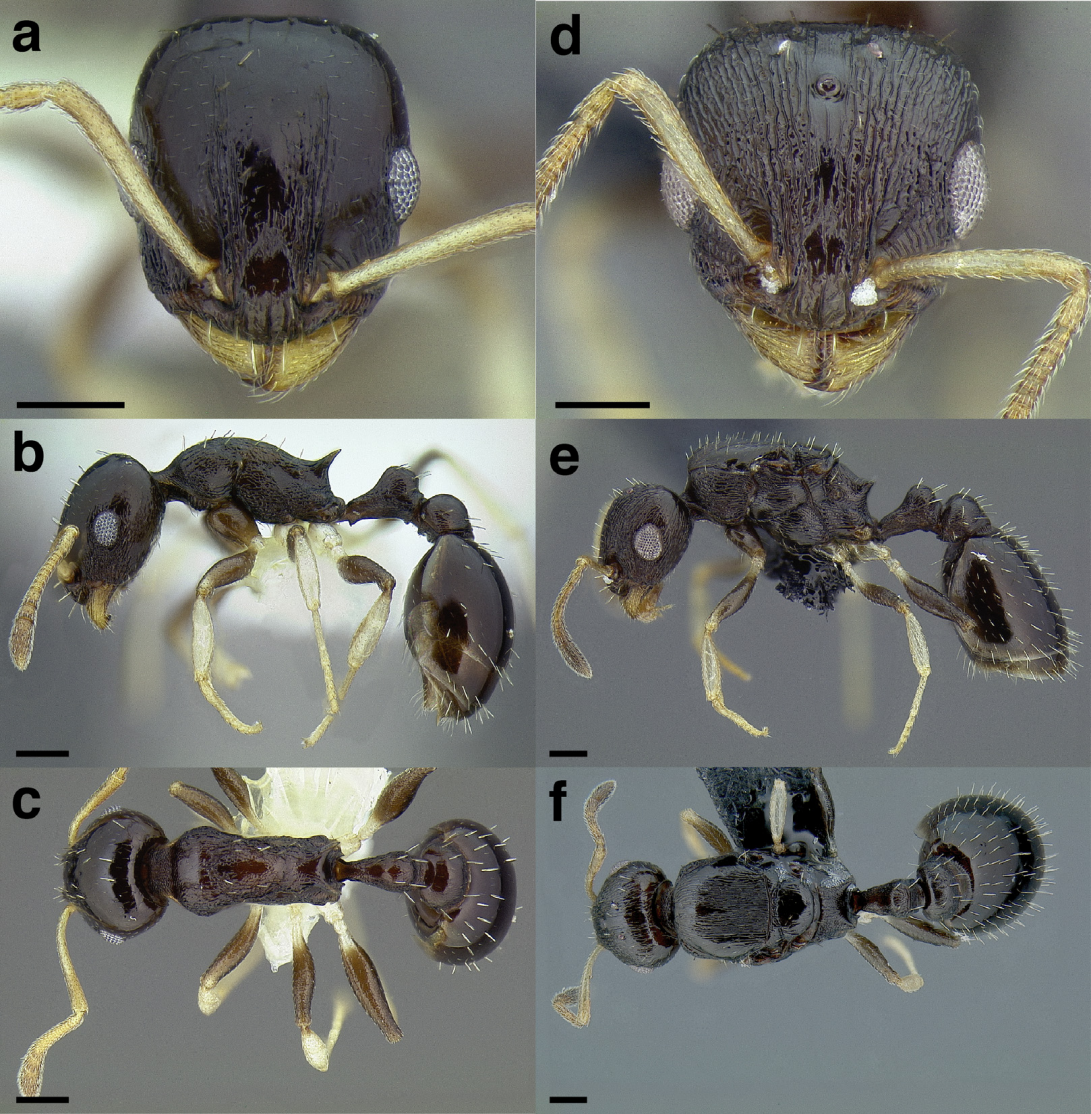
*Temnothorax xincai* sp. nov. (A–C) Holotype worker (CASENT0632983). (A) Full-face. (B) Profile view. (C) Dorsal view. (D–F) Gyne (CASENT0632988). (D) Full-face. (E) Profile view. (F) Dorsal view. Scale bars 0.2 mm.

Distribution: Fig. 114D; worker & gyne: Fig. 119.

**Type material examined:** *Holotype worker:* GUATEMALA: Santa Rosa: Volcán Tecuamburro, 14.15093°N 90.41435°W ± 20 m, 1,469 m, 19 April 2014, Barrios & Bustamonte #302, pine forest, ex sifted leaf litter (CASENT0632983) [CASC].

*Paratype workers:* same data as holotype, 1 worker (CASENT0869157) [USNM] 1 worker (CASENT0869158) [MCZC].

**Non-type material examined:** GUATEMALA: Santa Rosa: same data as holotype, except: 14.15160°N 90.41276°W ± 20 m, 1,462 m, 19 April 2014, Barrios & Bustamonte #304, pine forest, ex sifted leaf litter, 1 dealate gyne (CASENT0632988) [CASC].

**Geographic range:** Mid elevations of southern Guatemala (Santa Rosa) (Fig. 114D).

**Worker diagnosis:** *Temnothorax xincai* sp. nov. can be separated from all other species in the *salvini* clade by the following character combination: antennal scapes short, failing to reach the posterior margin of the head capsule by about the maximum width of the scape (SI 89); compound eyes small (OI 24); head sculpture mostly smooth and shining; body moderately compact (WLI 141); metanotal groove absent; propodeum moderately depressed; propodeal spines about as long as the propodeal declivity (PSI 21); propodeal spines broadly approximated in dorsal view (SBI ~ 29); hind femora moderately incrassate (FI 293); petiolar node erect and dorsally rounded, not overhanging the caudal cylinder in profile view; petiolar node compact, not elongate (NI 155); petiolar node slightly broader than caudal cylinder in dorsal view (PNWI < 122); dorsum of postpetiole smooth and shining; antennal scapes with short, decumbent pilosity; setae on head, mesosoma, waist segments and gaster erect, short, sparse and blunt (never long and tapering); integument predominantly dark brown, with trochanters, meso- and metacoxae, tibiae, tarsi, and sting very light yellow.

**Similar species:** *Temnothorax subditivus, T. tenuisculptus*, species of the *augusti*, *goniops*, and *pulchellus* groups, species of the *sallei* clade. *Temnothorax xincai* sp. nov. can be distinguished from similar appearing members of the *sallei* clade (e.g., *T. manni, T. mexicanus, T. punctithorax*) by the petiole, which lacks a transverse carina anterodorsally. *Temnothorax xincai* sp. nov. can be separated from *T. subditivus* and the *augusti* group by the structure of the petiolar node, which is erect, dorsally rounded, and only slightly broader than the caudal cylinder in dorsal view, as opposed to squamiform in *T. subditivus*, or leaning posteriorly over the caudal cylinder in the *augusti* group. In contrast to *T. tenuisculptus, T. xincai* sp. nov. has moderately incrassate hind femora (FI < 300). *Temnothorax xincai* sp. nov. can be distinguished from the *pulchellus* group by the combination of a relatively longer mesosoma (WLI > 135 vs. < 135 in most *pulchellus* group members). Some members of the *pulchellus* group may approach these dimensions (e.g., *T. agavicola* sp. nov., *T. albispinus, T. flavidulus, T. hippolytus* sp. nov., *T. laticrus* sp. nov., *T. terricola, T. torrei*, and *T. wilsoni* sp. nov.), but they can be separated from *T. xincai* sp. nov. by their very broad postpetioles (PWI > 220). *Temnothorax xincai* sp. nov. can be distinguished from other *goniops* group members by the relatively short, basally flared propodeal spines (PSI 21; longer than the propodeal declivity and spiniform in *T. goniops* and *T. huehuetenangoi*), the smooth and shining head and postpetiole (sculptured in *T. ixili and T. achii* sp. nov.).

**Worker measurements & indices (*n* = 1):** SL = 0.458; FRS = 0.188; CW = 0.560; CWb = 0.515; PoOC = 0.232; CL = 0.617; EL = 0.143; EW = 0.112; MD = 0.129; WL = 0.725; SPST = 0.153; MPST = 0.241; PEL = 0.298; NOL = 0.181; NOH = 0.117; PEH = 0.197; PPL = 0.170; PPH = 0.197; PW = 0.367; SBPA = 0.150; SPTI = 0.207; PEW = 0.139; PNW = 0.170; PPW = 0.295; HFL = 0.508; HFWmax = 0.117; HFWmin = 0.040; CS = 0.824; ES = 0.199; SI = 89; OI = 24; CI = 83; WLI = 141; SBI = 29; PSI = 21; PWI = 212; PLI = 175; NI = 155; PNWI = 122; NLI = 61; FI = 293.

**Worker description:** In full-face view, head subquadrate, longer than broad (CI 83). Antennal scapes short: when fully retracted, failing to reach the posterior margin of the head capsule by about the maximum width of the scape (SI 89). Mandibles densely, finely striate but weakly shining and armed with five teeth: the apical-most well developed and acute, followed by a less developed preapical tooth and three equally developed smaller teeth. Anterior clypeal margin evenly convex medially. Antennae 12-segmented; antennal club of composed of three segments, with the apical-most segment longer than the preceding two in combination. Frontal carinae short, extending past the antennal toruli by about one and a half times the maximum width of the antennal scape. Compound eyes moderately protruding past the lateral margins of the head capsule. Lateral margin of head weakly convex, forming a continuous arc from the mandibular insertions to the posterior margin of the head. Posterior head margin flat but rounding evenly into the lateral margins.

In profile view, compound eyes ovular and moderately large (OI 24), with 10 ommatidia in longest row. Pronotal declivity indistinct, neck and anterior face of pronotum forming a ~120° angle. Mesosoma sinuate: very strongly convex from where it joins the pronotal neck to the propodeum, which is flat and rounds evenly into the base of the propodeal spines. Promesonotal suture extending from the posterior margin of the procoxal insertion to the mesothoracic spiracle, which is moderately well developed; suture continues dorsally as a weak sulcus. Metanotal groove visible as a disruption of the sculpture laterally from where it arises between the mid- and hind coxae to where it ends in the poorly developed metathoracic spiracle, which is nearly indistinguishable against the ground sculpture. Propodeal spiracle moderately well developed, directed laterally, and separated from the propodeal declivity by about four spiracle diameters. Propodeal spines moderately well developed, but short (PSI 21), slightly shorter than the propodeal declivity, tapering evenly from the base, weakly downcurved near the apices, and acute. Propodeal declivity flat, forming a rounded ~120° angle with the base of the propodeal spines. Propodeal lobes rounded and weakly developed. Metapleural gland bulla small, extending from the metacoxal insertion halfway to the propodeal spiracle. Petiole moderately long (PLI 175), without tubercles anterodorsally. Subpetiolar process in the form of an acute, triangular tooth; ventral margin of petiole flat posterior to it. Petiolar peduncle short: comprising about a third of the length of the petiole. Petiolar node robust and erect, subquadrate: transition between peduncle and node marked by a rounded angle of ~120°, resulting in a concave anterior node face; anterior face meeting the dorsal face at a rounded angle of ~110°; dorsal face weakly convex, meeting the posterior face at a rounded ~90° angle; posterior face forms a ~90° angle with the caudal cylinder. Postpetiolar dorsum evenly rounded; weakly lobed ventrally.

In dorsal view, humeri developed: evenly rounded and wider than the rest of the mesosoma; mesothoracic spiracles protruding past the lateral margins of the mesosoma, visible as slight angles where the pronotum meets the mesonotum. Promesonotal suture indicated by a band of denser sculpture. Metanotal groove absent: mesonotum and propodeum completely fused and lateral margins converging evenly to the bases of the propodeal spines. Propodeal spines broadly approximated basally, bases joined by a rounded transverse welt, and diverging apically, their apices separated from each other by slightly less than their length, the negative space between them “U” shaped. Petiolar peduncle with spiracles not protruding past the lateral margins; peduncle not noticeably constricted anterior to them. Petiolar node ovular and longitudinally elongate, the anterior of the node narrower than the posterior margin; node tapering, so that the apex is narrower than the base. Base of node wider than the peduncle, and about one and quarter times as wide as the caudal cylinder. Postpetiole moderately broad (PWI 212) and campaniform, articulating with most of the anterior margin of the gaster, leaving small, angulate margins on each side exposed. Anterior margin of the postpetiole flat and evenly rounds into the lateral margins, which diverge slightly to the angulate posterior corners; posterior margin flat. Metafemur moderately incrassate (FI 293).

Sculpture: median clypeal carina present, extending from the anterior margin of the clypeus to the level of the antennal insertions, and flanked by two slightly weaker carinae. Lateral clypeal lobes with additional, weaker carinae; ground sculpture shining through weak areolate sculpture. Antennal scapes weakly areolate-costulate. Cephalic dorsum smooth and shining, with costulae flanking the frontal carinae medially, extending just beyond the level of the posterior margins of the compound eyes. Lateral surfaces of head predominantly smooth and shining, but with areolate sculpture on the ventral margin behind the compound eye and strongly rugose sculpture between the compound eye and mandibular insertion. Ventral surface of head weakly, finely areolate-costulate posteromedially, otherwise smooth and shining. Pronotal neck weakly areolate. Lateral surface of the pronotum rugulose anteriorly, and smooth and shining on the posterior half. Lateral surface of the rest of the mesosoma areolate, but with smooth and shining patches anterior and posterior to the propodeal spiracles. Propodeal declivity smooth and shining. Dorsal surface of pronotum finely costulate, but smooth and shining medially. Pronotum delineated from the mesonotum by a transverse strip of dense, rugulose sculpture. Mesonotum and propodeum weakly longitudinally areolate-costulate, but smooth and shining medially. Mesonotum delineated from the propodeum by an indistinct transverse sulcus. Femora shining, with weak areolate sculpture on the distal third. Petiole weakly areolate laterally, but smooth and shining anteriorly, and weakly areolate-costulate dorsally. Postpetiole predominantly smooth and shining, but with weakly areolate-strigulate on the posterodorsal half. First gastral tergite smooth and shining, with weak spectral iridescence. First gastral sternite smooth and shining.

Setae: antennal scapes and funiculi with short, decumbent pilosity. Dorsum of the head, pronotum, waist segments, and gaster with moderately abundant, erect, blunt-tipped setae, the longest of which are about the width of the compound eye. The head bears ~30, mesosoma ~20, petiole 6, postpetiole ~8, and first gastral tergite ~26 setae. Short, sparse pubescence present over the entire body, but difficult to detect against the areolate sculpture.

Color: predominantly dark brown, with mandibles and antennae testaceous yellow. Procoxae, sting, and femora testaceous. Trochanters, meso- and metacoxae, tibiae, and tarsi very pale yellow, nearly white.

**Gyne measurements & indices (*n* = 1):** SL = 0.483; FRS = 0.206; CW = 0.661; CWb = 0.620; PoOC = 0.231; CL = 0.636; EL = 0.210; EW = 0.160; MD = 0.115; WL = 1.083; SPST = 0.205; MPST = 0.290; PEL = 0.388; NOL = 0.168; NOH = 0.133; PEH = 0.240; PPL = 0.204; PPH = 0.290; PW = 0.633; SBPA = 0.296; SPTI = 0.275; PEW = 0.188; PNW = 0.221; PPW = 0.370; HFL = 0.584; HFWmax = 0.129; HFWmin = 0.050; CS = 0.938; ES = 0.290; SI = 78; OI = 31; CI = 97; WLI = 175; SBI = 48; PSI = 19; PWI = 197; PLI = 190; NI = 126; PNWI = 118; NLI = 43; FI = 258.

**Gyne description:** In full-face view, head subquadrate, about as long as broad (CI 97). Mandibles densely, finely striate but shining and armed with five teeth: the apical-most well developed, followed by a less developed preapical tooth and three equally developed smaller teeth. Anterior clypeal margin evenly convex medially. Antennal scapes short: when fully retracted, failing to reach the posterior margin of the head capsule by about half the maximum width of the scape (SI 78). Antennae 12-segmented; antennal club composed of three segments, with the apical-most segment longer than the preceding two in combination. Frontal carinae moderately long, extending past the antennal toruli by about two times the maximum width of the antennal scape. Compound eyes moderately protruding past the lateral margins of the head capsule. Lateral margin of head behind the compound eyes weakly convex; below the compound eyes, lateral margins converging to the mandibular insertions. Posterior head margin flat, rounding evenly into the lateral margins.

In profile view, compound eyes ovular and large (OI 31), with 16 ommatidia in longest row. Mesoscutum rounded evenly anteriorly, covering the dorsal surface of the pronotum, and flat dorsally. Mesoscutellum on the same level as the mesoscutum. Posterior margin of metanotum extending slightly past the posterior margin of the mesoscutum. Propodeal spiracle well developed, directed posterolaterally, and separated from the propodeal declivity by about four spiracle diameters. Propodeal spines stout and moderately well developed (PSI 19), about half as long as the propodeal declivity, triangular, directed posteriorly, straight, and acute. Propodeal declivity weakly concave, forming a rounded ~110° angle with the base of the propodeal spines. Propodeal lobes rounded and very weakly developed. Metapleural gland bulla moderately large, extending from the metacoxal insertion two thirds of the way to the propodeal spiracle. Petiole moderately long (PLI 190), without tubercles anterodorsally. Subpetiolar process in the form of a very small, triangular, very acute tooth, which grades evenly into the ventral margin of the petiole posteriorly; ventral margin of petiole very weakly bulging. Petiolar peduncle short: comprising about a third of the length of the petiole. Petiolar node robust and erect, evenly rounded: transition between peduncle and node indistinct, marked by a rounded angle of ~140°, resulting in a concave anterior node face; anterior face rounding evenly into the dorsal face, which is evenly convex; dorsal face rounding evenly into the posterior face, which forms a ~100° angle with the caudal cylinder. Postpetiole flat anteriorly, bulging slightly anterodorsally before flattening posterodorsally; ventral surface lobed.

In dorsal view, mesoscutum covering pronotum anteriorly, but humeri visible laterally as rounded sclerites. Propodeal spines weakly diverging apically, their apices separated from each other by about three times their length. Petiolar peduncle with spiracles not protruding past the lateral margins. Petiolar node campaniform: evenly convex anteriorly, and flat posteriorly; node tapering, so that the apex is narrower than the base. Base of node wider than the peduncle, and about one and quarter times as wide as the caudal cylinder. Postpetiole subquadrate, narrow (PWI 197), and articulating with most of the anterior margin of the gaster, leaving small, angulate margins on each side exposed. Anterior margin of postpetiole weakly convex, with corners marked by rounded angles as it transitions to the lateral margins, which are parallel to the angulate posterior corners; posterior margin flat. Metafemur moderately incrassate (FI 258).

Sculpture: median clypeal carina present, extending from the anterior margin of the clypeus to the level of the antennal insertions, and flanked by two slightly weaker carinae. Lateral clypeal lobes with additional weaker carinae; ground sculpture weakly areolate. Antennal scapes very weakly areolate-costulate. Cephalic dorsum costate with weaker cross reticulations; very fine concentric costulae surrounding the antennal insertions; strip of smooth and shining sculpture extending from the frontal triangle to the median ocellus; posterior to the median ocellus, the strip of smooth and shining sculpture broadening to the width separating the lateral ocelli. Lateral surfaces of head costate, with rugose sculpture surrounding the compound eye, and between the compound eye and the mandibular insertion. Ventral surface of head shining through weak areolate-costulate sculpture. Pronotal neck shining through weak areolate sculpture. Lateral surfaces of the mesosoma areolate-costulate, becoming weaker on the posteromedial part of the lateral face of the pronotum, anterior third of the mesopleurae, and in small patches anterior and posterior to the propodeal spiracle. Propodeal declivity weakly strigulate. Mesoscutum costulate, with smooth and shining sculpture in a small patch anteromedially, and in two patches near the wing bases. Mesoscutellum predominantly smooth and shining, with indications of weak costulae. Femora shining through traces of weak areolate sculpture. Petiole and postpetiole weakly longitudinally areolate-costulate laterally. Dorsal face of the peduncle weakly, finely areolate. Anterior face of the petiolar node smooth and shining; dorsal face of the node with fine concentric costulae. Postpetiole smooth and shining anteriorly; dorsal surface with fine concentric areolate-costulate sculpture. First gastral tergite smooth and shining, without spectral iridescence. First gastral sternite smooth and shining.

Setae: antennal scapes and funiculi with short, decumbent pilosity. Dorsum of the head, pronotum, waist segments, and gaster with moderately abundant, erect, blunt-tipped setae, the longest of which are about half the width of the compound eye. Short, sparse pubescence present over the entire body, but difficult to detect against the areolate sculpture.

Color: predominantly dark brown, with mandibles and antennae testaceous yellow. Procoxae, sting, and femora testaceous. Trochanters, meso- and metacoxae, tibiae, and tarsi very pale yellow, nearly white.

**Male:** Unknown.

**Etymology:** Geographical, from ‘xinca’, one of the many Mayan dialects spoken by the indigenous people of Guatemala.

**Comments:** *Temnothorax xincai* sp. nov. is known only from a couple of Winkler leaf litter extractions made in high elevation pine forests in southern Guatemala. This species is closely related to *T. ixili* and *T. achii* sp. nov., which are also known from low to high elevation leaf litter extractions and nests in *Oncidium* orchids.

### *misomoschus* group overview

**Figure 120:**
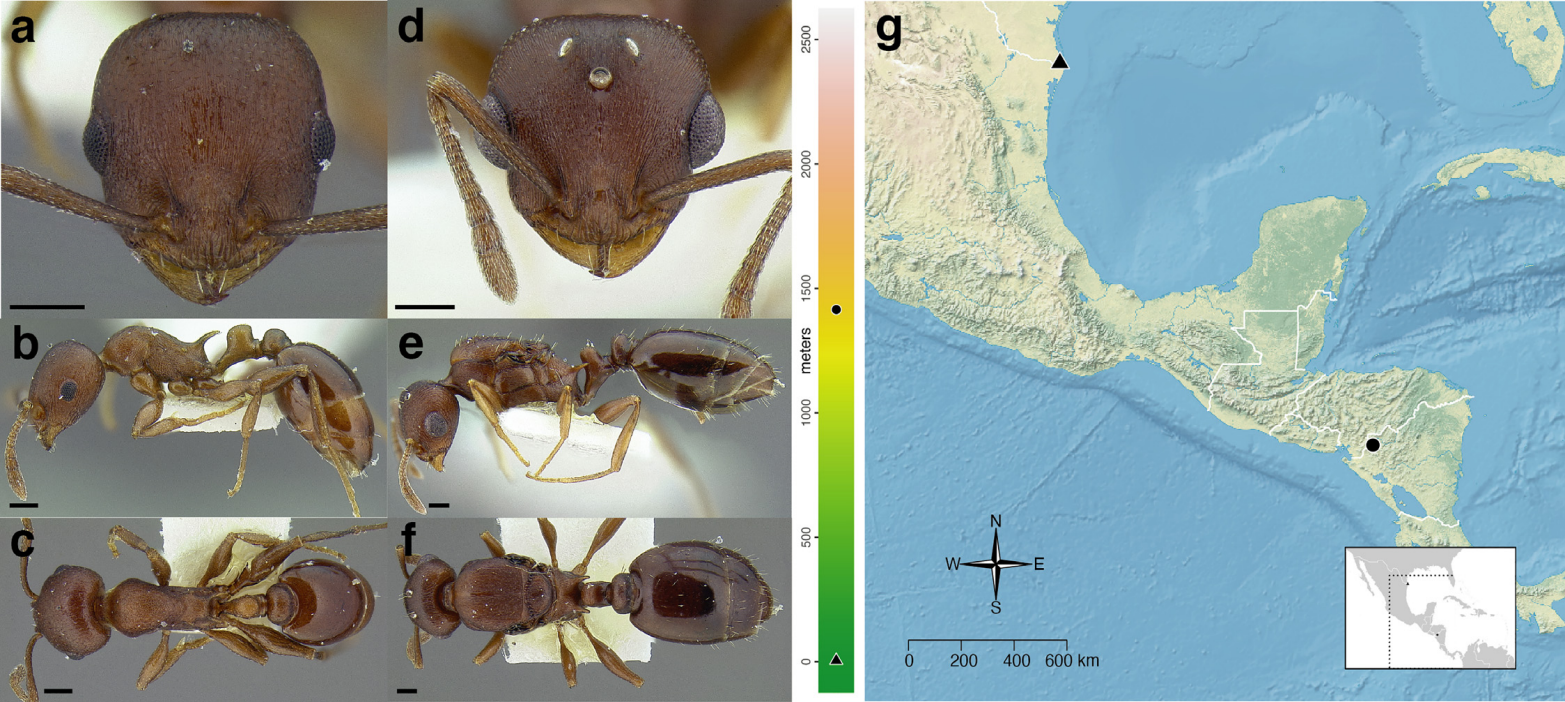
*Temnothorax misomoschus* sp. nov. (A–C) Holotype worker (LACMENT323430). (A) Full-face view. (B) Profile view. (C) Dorsal view. (D–F) Paratype gyne (LACMENT323429). (D) Full-face view. (E) Profile view. (F) Dorsal view. (G) Geographical and elevational distribution of specimens examined. Type localities are represented by triangles, non-type localities are represented by circles. Bounding box in inset map shows location of main map. Scale bars 0.2 mm.

This group is monotypic, with the nominal *Temnothorax misomoschus* sp. nov. being the only member. With its depressed propodeum, strongly curved propodeal spines, and nearly complete lack of standing setae, this species is distinctive in the *salvini* clade, not to mention the genus as a whole. Collected from only a couple of geographically disparate collections (southern Texas and Nicaragua; Fig. 120G), this species apparently has a large range, but we still know little about its biology. It is sister to the *goniops* group (Prebus, in prep.).

***Temnothorax misomoschus* sp. nov.**

Distribution, worker & gyne: Fig. 120.

*Temnothorax* mmp12 Prebus, 2017: 8. Phylogeny.

**Type material examined:** *Holotype worker:* U.S.A.: Texas: Cameron County: 16 km W Boca Chica, 11 January 1968, R.R. Snelling#1968-8 (LACMENT323430, top specimen on pin) [LACM].

*Paratype workers and gyne:* same pin as holotype, 2 workers (middle and bottom specimens on pin) (LACM); same data as holotype, 1 dealate gyne & 2 workers (LACMENT323429) [LACM].

**Non-type material examined:** NICARAGUA: Madriz: 8 km S Somoto, 13.40447°N 86.58267°W ± 50 m, 1,415 m, 22 April 2011, J. Longino#JTL7384-s, coffee farm, at bait, 1 worker (CASENT0619604) [UCDC].

**Geographic range:** Southern Texas and Nicaragua (Fig. 120G).

**Worker diagnosis:** The following character combination will separate *Temnothorax misomoschus* sp. nov. from all other species in the *salvini* clade: mesosoma compact (WLI 128–136); propodeum strongly depressed; propodeal spines longer than the propodeal declivity (PSI 33–38), and downwardly curved; hind femora strongly incrassate; petiolar node subquadrate and leans posteriorly over the caudal cylinder; postpetiole moderately broad; body devoid of erect setae, except for the clypeus and posterior margins of the gastral sclerites; integument medium brown, with tibiae and tarsi testaceous.

**Similar species:** It is difficult to imagine that this striking species could be confused with any other in *Temnothorax*, but *T. misomoschus* sp. nov. vaguely resembles *T. subditivus*; the lack of erect setae and the subquadrate petiolar node in *T. misomoschus* sp. nov. (as opposed to squamiform in *T. subditivus*) will distinguish the two.

**Worker measurements & indices (*n* = 6):** SL = 0.500–0.542 (0.515); FRS = 0.213–1.107 (0.370); CW = 0.655–0.693 (0.675); CWb = 0.610–0.645 (0.627); PoOC = 0.251–0.277 (0.264); CL = 0.686–0.719 (0.705); EL = 0.161–0.170 (0.166); EW = 0.120–0.137 (0.128); MD = 0.148–0.179 (0.162); WL = 0.793–0.864 (0.817); SPST = 0.262–0.322 (0.287); MPST = 0.240–0.290 (0.260); PEL = 0.338–0.366 (0.349); NOL = 0.190–0.232 (0.211); NOH = 0.144–0.155 (0.151); PEH = 0.237–0.269 (0.251); PPL = 0.147–0.178 (0.160); PPH = 0.206–0.293 (0.234); PW = 0.407–0.453 (0.423); SBPA = 0.143–0.180 (0.162); SPTI = 0.226–0.253 (0.237); PEW = 0.146–0.167 (0.158); PNW = 0.151–0.179 (0.166); PPW = 0.269–0.308 (0.290); HFL = 0.564–0.681 (0.598); HFWmax = 0.154–0.172 (0.160); HFWmin = 0.041–0.049 (0.046); CS = 0.957–1.005 (0.979); ES = 0.221–0.238 (0.230); SI = 81–84 (82); OI = 23–24 (23); CI = 86–90 (89); WLI = 128–136 (130); SBI = 23–28 (26); PSI = 33–38 (35); PWI = 174–192 (183); PLI = 199–236 (219); NI = 128–151 (139); PNWI = 99–109 (105); NLI = 55–64 (60); FI = 322–393 (353).

**Worker description:** In full-face view, head subquadrate, slightly longer than broad (CI 86–90). Mandibles densely striate but shining and armed with five teeth: the apical-most well developed and acute, followed by a less developed preapical tooth and three equally developed smaller teeth. Anterior clypeal margin flat medially. Antennal scapes short: when fully retracted, failing to reach the posterior margin of the head capsule by about two times the maximum width of the scape (SI 81–84). Antennae 12-segmented; antennal club of composed of three segments, with the apical-most segment slightly longer than the preceding two in combination. Frontal carinae moderately long, extending past the antennal toruli by about two times the maximum width of the antennal scape. Compound eyes moderately protruding past the lateral margins of the head capsule. Lateral margin of head convex, forming a continuous arc from the mandibular insertions to the posterior margin of the head. Posterior head margin flat but rounding evenly into the lateral margins.

In profile view, compound eyes ovular and moderately large (OI 23–24), with 15 ommatidia in longest row. Pronotal declivity indistinct, neck and anterior face of pronotum forming a ~120° angle. Mesosoma strongly sinuate: very strongly convex from where it joins the pronotal neck to the propodeum, which is concave and rounds evenly into the base of the propodeal spines. Promesonotal suture extending from the posterior margin of the procoxal insertion to the mesothoracic spiracle, which is moderately well developed; suture continues dorsally as an indistinct disruption in the ground sculpture. Metanotal groove visible as a disruption of the sculpture laterally from where it arises between the mid- and hind coxae to where it ends in the poorly developed metathoracic spiracle, which is nearly indistinguishable against the ground sculpture. Propodeal spiracle moderately well developed, directed posterolaterally, and separated from the propodeal declivity by about four spiracle diameters. Propodeal spines well developed and long (PSI 33–38), slightly longer than the propodeal declivity, tapering evenly from the base, very strongly downcurved, and acute. Propodeal declivity flat, forming a rounded ~110° angle with the base of the propodeal spines. Propodeal lobes rounded and weakly developed. Metapleural gland bulla small, extending from the metacoxal insertion halfway to the propodeal spiracle. Petiole long (PLI 199–236), without tubercles anterodorsally. Subpetiolar process in the form of a small, triangular tooth; ventral margin of petiole weakly concave posterior to it. Petiolar peduncle moderately long: comprising slightly less than half the length of the petiole. Petiolar node robust and erect, subquadrate: transition between peduncle and node marked by a rounded angle of ~120°, resulting in a concave anterior node face; anterior face rounding evenly into the dorsal face, which is evenly convex; dorsal face meeting the posterior face at a ~90° angle; posterior face which forms a ~85° angle with the caudal cylinder, overhanging it. Postpetiole flat anteriorly, bulging anterodorsally before flattening posterodorsally; weakly lobed ventrally.

In dorsal view, humeri developed: evenly rounded and wider than the rest of the mesosoma; mesothoracic spiracles not protruding past the lateral margins of the mesosoma. Promesonotal suture indicated by a disruption in the ground sculpture. Metanotal groove absent: mesonotum and propodeum completely fused and lateral margins converging evenly to the bases of the propodeal spines; propodeal spiracles protruding laterally. Propodeal spines narrowly approximated basally and weakly diverging apically, their apices separated from each other by slightly less than their length, the negative space between them “U” shaped. Petiolar peduncle with spiracles not protruding past the lateral margins; peduncle not noticeably constricted anterior to them. Petiolar node evenly ovular and slightly longitudinally elongate; node the same width as the peduncle, and evenly grading into the caudal cylinder, which is wider than the node. Postpetiole narrow (PWI 174–192) and campaniform, articulating with most of the anterior margin of the gaster, leaving small, angulate margins on each side exposed. Anterior margin of the postpetiole convex and evenly rounds into the lateral margins, which are evenly convex to the posterior corners; posterior margin flat. Metafemur strongly incrassate (FI 322–393).

Sculpture: median clypeal carina present but indistinct from the multiple, equally strong carinae that flank it. Lateral clypeal lobes with additional, weaker carinae; ground sculpture smooth and shining. Antennal scapes areolate-costulate. Cephalic dorsum very finely costulate, with very fine concentric costulae surrounding the antennal insertions. Lateral surfaces of head costulate over areolate sculpture, becoming rugulose between the compound eye and the mandibular insertion. Ventral surface of head weakly, finely areolate-costulate. Pronotal neck strigulate. Lateral surfaces of the mesosoma areolate, but meso- and metapleurae rugulose. Propodeal declivity weakly areolate. Dorsal surface of mesosoma areolate, with fine costulae over the promesonotum. Femora shining, with weak areolate sculpture on the distal third. Petiole and postpetiole shining through weak areolate sculpture, with ventral surface of petiole and anterior face of postpetiole very weakly areolate. Gaster smooth and shining, without spectral iridescence.

Setae: antennal scapes and funiculi with short, decumbent pilosity. Body apparently without erect setae, except for the clypeus, and sporadically on the postpetiole. Short, sparse pubescence present over the entire body.

Color: predominantly medium brown, with tibiae and tarsi testaceous.

**Gyne measurements & indices (*n* = 1):** SL = 0.588; FRS = 0.260; CW = 0.860; CWb = 0.754; PoOC = 0.262; CL = 0.780; EL = 0.272; EW = 0.219; MD = 0.145; WL = 1.358; SPST = 0.343; MPST = 0.353; PEL = 0.426; NOL = 0.227; NOH = 0.221; PEH = 0.379; PPL = 0.203; PPH = 0.372; PW = 0.728; SBPA = 0.350; SPTI = 0.342; PEW = 0.215; PNW = 0.265; PPW = 0.404; HFL = 0.769; HFWmax = 0.177; HFWmin = 0.044; CS = 1.144; ES = 0.382; SI = 78; OI = 33; CI = 97; WLI = 180; SBI = 46; PSI = 25; PWI = 188; PLI = 210; NI = 103; PNWI = 123; NLI = 53; FI = 402.

**Gyne description:** In full-face view, head subquadrate, about as long as broad (CI 97). Mandibles densely, finely striate but shining and armed with five teeth: the apical-most well developed, followed by a less developed preapical tooth and three equally developed smaller teeth. Anterior clypeal margin flat medially. Antennal scapes short: when fully retracted, failing to reach the posterior margin of the head capsule by about two times the maximum width of the scape (SI 78). Antennae 12-segmented; antennal club composed of three segments, with the apical-most segment as long as the preceding two in combination. Frontal carinae moderately long, extending past the antennal toruli by about three times the maximum width of the antennal scape. Compound eyes strongly protruding past the lateral margins of the head capsule. Lateral margin of head behind the compound eyes convex; below the compound eyes, lateral margins parallel, then converging to the mandibular insertions. Posterior head margin flat, rounding evenly into the lateral margins.

In profile view, compound eyes ovular and large (OI 33), with 20 ommatidia in longest row. Mesoscutum rounded evenly anteriorly, covering the dorsal surface of the pronotum, and flat dorsally. Mesoscutellum on the same level as the mesoscutum. Posterior margin of metanotum extending slightly past the posterior margin of the mesoscutum. Propodeal spiracle well developed, directed posterolaterally, and separated from the propodeal declivity by about five spiracle diameters. Propodeal spines stout and well developed, but short (PSI 25), about half as long as the propodeal declivity, tapering evenly from the base, directed posteriorly, very weakly downcurved at the tips, and acute. Propodeal declivity straight and flat, forming a rounded ~120° angle with the base of the propodeal spines. Propodeal lobes rounded and very weakly developed. Metapleural gland bulla small, extending from the metacoxal insertion halfway to the propodeal spiracle. Petiole long (PLI 210), without tubercles anterodorsally. Subpetiolar process in the form of a small, triangular, very blunt tooth, which grades evenly into the ventral margin of the petiole posteriorly. Petiolar peduncle short: comprising about a third of the length of the petiole. Petiolar node robust and erect, nearly squamiform: transition between peduncle and node indistinct, marked by a rounded angle of ~140°, resulting in a concave anterior node face; anterior face rounding evenly into the dorsal face, which is moderately convex; dorsal face rounding evenly into the posterior face, which forms a ~90° angle with the caudal cylinder. Postpetiole very flat anteriorly, bulging strongly anterodorsally before flattening posterodorsally; ventral surface weakly lobed.

In dorsal view, mesoscutum covering pronotum anteriorly, but humeri visible laterally as slightly angulate sclerites. Propodeal spines weakly diverging apically, their apices separated from each other by about twice their length. Petiolar peduncle with spiracles not protruding past the lateral margins. Petiolar node subquadrate, nearly trapezoidal: flattened anteriorly and posteriorly, with posterior face narrower. Petiolar node slightly wider than the peduncle and caudal cylinder. Postpetiole subquadrate, narrow (PWI 188), and articulating with most of the anterior margin of the gaster, leaving small, angulate margins on each side exposed. Anterior margin of postpetiole flat, with corners marked by rounded angles as it transitions to the lateral margins, which are evenly convex to the posterior corners; posterior margin flat. Metafemur strongly incrassate (FI 402).

Sculpture: median clypeal carina present, but indistinct from the multiple, equally strong carinae that flank it. Lateral clypeal lobes with additional weaker carinae; ground sculpture weakly areolate. Antennal scapes very finely costulate. Cephalic dorsum costulate over fine areolate sculpture; very fine concentric costulae surrounding the antennal insertions. Lateral surfaces of head with areolate-costulate sculpture forming whorls around posterior margin of the compound eye, with rugulose sculpture between the compound eye and the mandibular insertion. Ventral surface of head shining through weak areolate-costulate sculpture. Pronotal neck shining through weak strigulate sculpture. Lateral surfaces of the mesosoma finely longitudinally areolate-costulate, becoming weaker on the mesopleurae. Propodeal declivity shining and weakly areolate. Mesoscutum and mesoscutellum costulate over weak areolate ground sculpture. Femora shining through traces of weak areolate sculpture. Petiole weakly areolate and shining. Postpetiole smooth and shining anteriorly; dorsal surface weakly areolate-strigulate. First gastral tergite smooth and shining, with faint traces of spectral iridescence. First gastral sternite smooth and shining.

Setae: antennal scapes and funiculi with short, decumbent pilosity. Sparse, erect, blunt-tipped setae, on the dorsal surface of the head, mesosoma, postpetiole, and gaster. Short, sparse pubescence present over the entire body.

Color: Predominantly medium brown, with tibiae and tarsi testaceous.

**Male:** Unknown.

**Etymology:** Greek, ‘misos’ (= hatred) and ‘moschus’ (= musk). On 30 March 2021 a prototype rocket launched by SpaceX, an aerospace manufacturer and space transportation service founded by Elon Musk, exploded over the Lower Rio Grande Valley National Wildlife Refuge, which encompasses the type locality of *Temnothorax misomoschus* sp. nov. The explosion resulted in a rain of debris on the wildlife refuge, which is critical habitat for ocelots, migratory bird species, as well as the only known population of the Boca Chica flea beetle, *Chaetocnema rileyi*.

**Comments:** *Temnothorax misomoschus* sp. nov. is known from only a few of collections that span a large range. Roy Snelling collected the type series near Boca Chica, Texas; much later Jack Longino collected several workers at a cookie bait on a coffee farm in northwestern Nicaragua. Most recently Steven Wang collected and photographed a worker of this species found in Goliad, Texas. This species is closely related to the *goniops* species group, which spans Southern Mexico to Guatemala. Unfortunately, the circumstances of the type collection are unknown, but I suspect that *T. misomoschus* sp. nov. nests in the soil or the leaf litter, similar to species of the *goniops* group.

### *pastinifer* group overview

**Figure 121:**
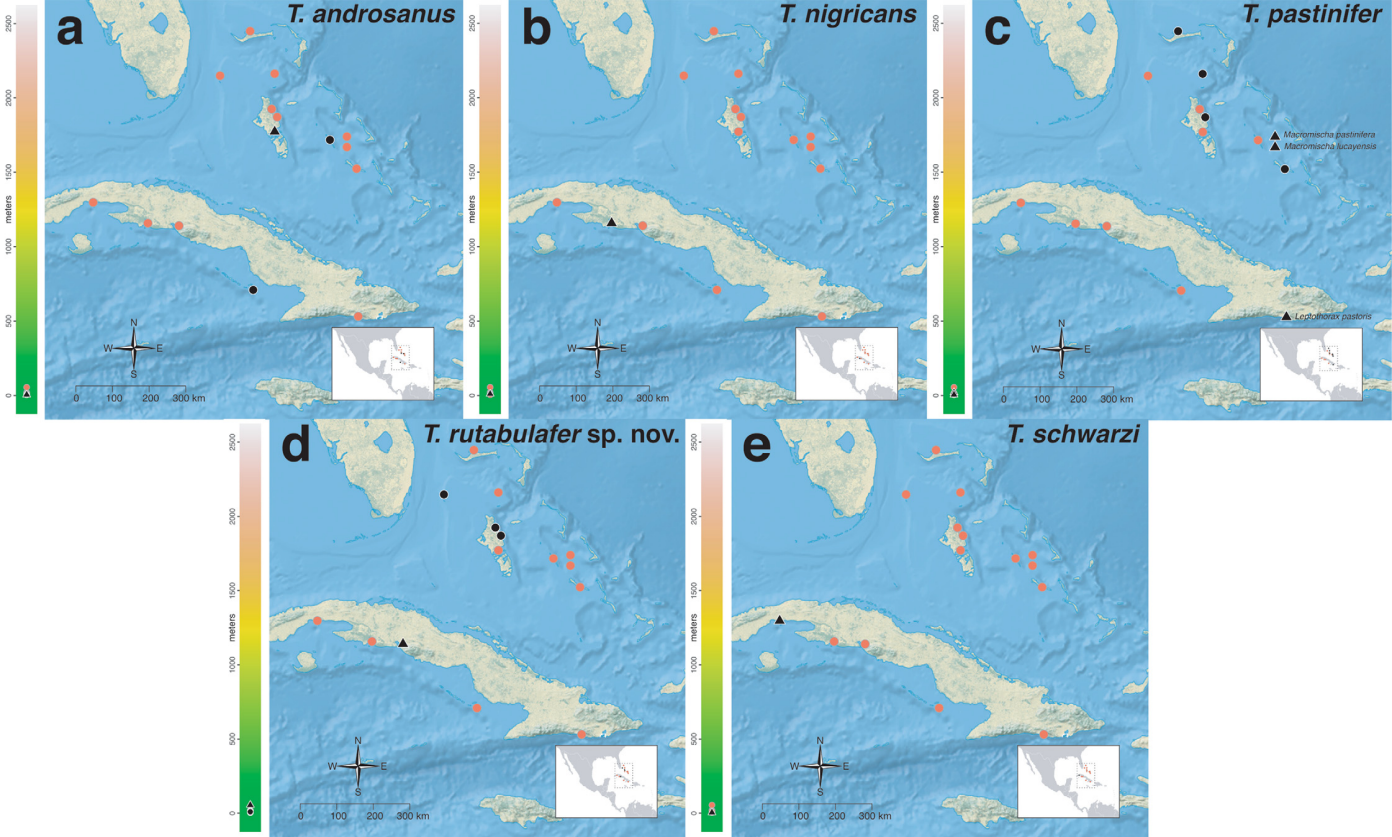
Geographical and elevational distribution of the *pastinifer* group. (A) *Temnothorax androsanus* (B) *T. nigricans* (C) *T. pastinifer* (D) *T. rutabulafer* sp. nov. (E) *T. schwarzi*. Colored scale to the left of each map represents elevation in meters. Points in black represent the species named in each subfigure, while points in red represent other members of the species group. Type localities are represented by triangles; if present, types of synonyms are named; non-type localities are represented by circles. Bounding box in inset map shows location of main map.

With five species (one described as new here), the *pastinifer* group spans the low elevations of the Bahamas and Cuba (Fig. 121). This group is likely composed of littoral specialists, as it has only been collected from low-lying habitats near the sea. Most nest collections have been taken from wood or woody fruits lying on the ground. These distinctive species have extremely arched mesosomata, broad postpetioles, and incrassate femora, and so are prime examples of the *Macromischa* syndrome. Members of the *pastinifer* group may be confused with *Temnothorax subditivus*, members of the *pulchellus* group, or morphologically convergent members of the *sallei* clade. Because of multiple character overlaps between these groups, the keys above and the ‘similar species’ sections below should be used to determine species group membership. The nominal *T. pastinifer* and *T. rutabulafer* sp. nov. have historically been conflated as a single species, probably due to a mixed pin prepared by W.M. Mann.

***Temnothorax androsanus* (Wheeler, 1905)**

**Figure 122:**
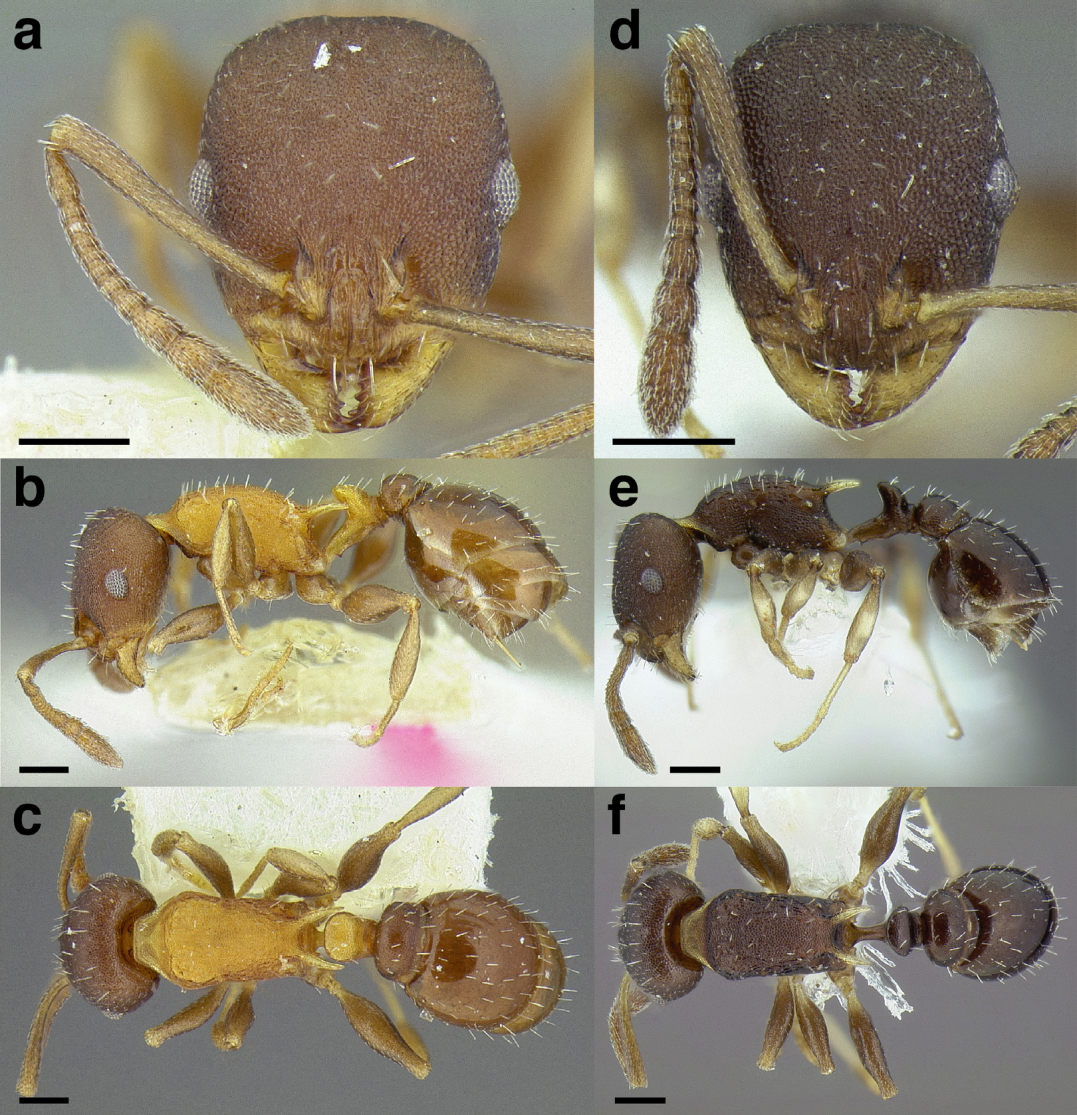
*Temnothorax androsanus*. (A–C) Lectotype worker (MCZENT00021003). (A) Full-face. (B) Profile view. (C) Dorsal view. (D–F) Dark form of *Temnothorax androsanus* (CASENT0758350). (D) Full-face. (E) Profile view. (F) Dorsal view. Scale bars 0.2 mm.

Distribution: Fig. 121A; worker & variability: Fig. 122.

*Macromischa androsana* Wheeler, 1905: 97, fig. K. Syntype workers. Bahamas. One syntype here designated **lectotype**.

*Leptothorax androsanus* (Wheeler): Baroni Urbani, 1978: 416. Gyne and male. First combination in *Leptothorax*.

*Temnothorax androsanus* (Wheeler): Bolton, 2003: 271. First combination in *Temnothorax*.

**Type material examined:** *Lectotype worker:* BAHAMAS: Andros Island: May–June 1904, W.M. Wheeler Collector, M.C.Z. Type 21003, Type No. A.M.N.H. (MCZENT00021003, bottom specimen on pin).

*Paralectotype workers:* same pin as lectotype (MCZENT00021003, remaining specimens on pin) [MCZC].

**Non-type material examined:** BAHAMAS: Exuma Cays: Staniel Key, May 1991, L. Morrison, leaf litter, 2 workers (CASENT0758350) [MCZC] 2 workers (CASENT0915979) [UCDC].

CUBA: Camagüey: Cayo Caguama, Jardines da la Reina, 1 April 1971, L. Armas, 1 worker (images of CASENT0914182 examined on antweb.org) [BMNH].

**Geographic range:** Low elevations from the Bahamas and Cuba (Fig. 121A).

**Worker diagnosis:** *Temnothorax androsanus* can be separated from all other species in the *salvini* clade by the following character combination: antennal scape moderately long, barely surpassing the posterior margin of head; anterior margin of clypeus entire; transition between anterior and dorsal faces of pronotum marked by a distinct angle; in profile view, mesosoma strongly convex dorsally and compact (WLI 117–128); in dorsal view, mesosoma posterior to the pronotum swollen, only slightly narrower than the pronotum; dorsum of propodeum with setae; in dorsal view, propodeal spines broadly approximated basally, negative space between them “U” shaped; propodeal spines longer than the propodeal declivity, directed posteriorly, and downcurved; hind femora moderately to strongly incrassate (FI 247–443); petiolar node squamiform; dorsum of petiole with four erect setae; in dorsal view, petiolar node moderately broader than the caudal cylinder (PNWI < 160); postpetiole very broad (PWI 219–262); dorsum of head uniformly areolate; dorsum of petiolar node and postpetiole shallowly areolate; setae on head, mesosoma, waist segments and gaster erect, moderately long, sparse and blunt (never long and tapering); dorsum of postpetiole with > 10 erect setae; integument bicolored: antennae, head capsule, postpetiole, and gaster medium brown; mandibles, clypeus, mesosoma, legs, and petiole testaceous yellow; tarsi and sting yellowish.

**Similar species:** *Temnothorax misomoschus* sp. nov., *T. subditivus*, and other members of the *pastinifer* group. *Temnothorax androsanus* can be distinguished from *T. subditivus* by the narrower petiolar node in dorsal view (PNWI < 160 vs. > 160 in *T. subditivus*), the somewhat shorter petiolar peduncle (~2/3 of the total petiole length in *T. androsanus* vs. ~ 3/4 in *T. subditivus*) and the sculpture of the head dorsum (uniformly areolate in *T. androsanus* vs. variable in *T. subditivus*, but never uniformly areolate). In contrast to *T. androsanus, T. misomoschus* sp. nov. is devoid of erect setae on most surfaces of the body, has a depressed propodeum, and a posteriorly leaning, subquadrate petiolar node. *Temnothorax androsanus* can be separated from other members of the *pastinifer* group by the areolate head (smooth and shining in *T. schwarzi*), flat to weakly convex anterior clypeus margin (strongly emarginate in *T. pastinifer*), and the angulate transition between the anterior and dorsal faces of the pronotum in profile view (transition indistinct in *T. nigricans*).

**Worker measurements & indices (*n* = 9):** SL = 0.467–0.528 (0.492); FRS = 0.161–0.206 (0.186); CW = 0.510–0.612 (0.564); CWb = 0.466–0.565 (0.521); PoOC = 0.214–0.253 (0.235); CL = 0.548–0.657 (0.600); EL = 0.112–0.151 (0.130); EW = 0.084–0.117 (0.098); MD = 0.134–0.160 (0.149); WL = 0.598–0.706 (0.643); SPST = 0.205–0.290 (0.248); MPST = 0.189–0.230 (0.210); PEL = 0.239–0.295 (0.259); NOL = 0.124–0.164 (0.146); NOH = 0.115–0.150 (0.134); PEH = 0.213–0.249 (0.225); PPL = 0.152–0.189 (0.166); PPH = 0.173–0.219 (0.200); PW = 0.338–0.404 (0.373); SBPA = 0.144–0.185 (0.161); SPTI = 0.216–0.262 (0.239); PEW = 0.125–0.152 (0.141); PNW = 0.190–0.225 (0.204); PPW = 0.309–0.346 (0.324); HFL = 0.417–0.511 (0.460); HFWmax = 0.125–0.155 (0.140); HFWmin = 0.035–0.053 (0.047); CS = 0.740–0.894 (0.821); ES = 0.154–0.210 (0.179); SI = 87–102 (95); OI = 20–23 (22); CI = 84–92 (87); WLI = 117–128 (124); SBI = 27–35 (31); PSI = 34–43 (38); PWI = 219–262 (231); PLI = 139–177 (157); NI = 94–140 (110); PNWI = 140–156 (145); NLI = 51–64 (57); FI = 247–443 (306).

**Worker description:** In full-face view, head subquadrate, longer than broad (CI 84–92). Mandibles moderately striate, shining, and armed with five teeth: the apical-most well developed, followed by a less developed preapical tooth and three equally developed smaller teeth. Anterior clypeal margin flat to weakly convex medially. Antennal scapes moderately long: when fully retracted, surpassing the posterior margin of the head capsule by about the maximum width of the antennal scape (SI 87–102). Antennae 12-segmented; antennal club of three segments, with the apical-most segment slightly longer than the preceding two in combination. Frontal carinae very short, extending past the antennal toruli by about one and a half times the maximum width of the antennal scape. Compound eyes moderately protruding past the lateral margins of the head capsule. Lateral margin of head weakly convex, converging evenly to the mandibular insertions. Posterior head margin flat to very slightly concave, rounding evenly into the lateral margins.

In profile view, compound eyes ovular and moderately large (OI 20–23), with 12 ommatidia in longest row. Pronotal declivity distinct: pronotal neck and anterior face of pronotum forming a rounded ~120° angle; anterior face and dorsal face forming a rounded ~120° angle. Mesosoma evenly, but weakly, convex dorsally from where it joins the anterior face of the pronotal declivity to the propodeal spines. Promesonotal suture extending from the posterior margin of the procoxal insertion only to the mesothoracic spiracle, which is barely visible against the ground sculpture. Metanotal groove nearly entirely absent: only visible as a faint disruption in the surface sculpture between meso- and metacoxal insertions to the minute metathoracic spiracle, which is nearly indistinguishable from the ground sculpture. Propodeal spiracle well developed, directed posterolaterally, and separated from the propodeal declivity by about two and a half spiracle diameters. Propodeal spines well developed and long (PSI 34–43), slightly longer than the propodeal declivity, tapering evenly from the base, evenly downcurved, and acute. Propodeal declivity weakly concave, forming a rounded ~100° angle with the base of the propodeal spines. Propodeal lobes rounded and weakly developed, but with a slightly angulate dorsal flange. Metapleural gland bulla large, extending from the metacoxal insertion three quarters of the way to the propodeal spiracle. Petiole moderately long (PLI 139–177), with peduncle evenly rounded anterodorsally. Subpetiolar process in the form of a weakly developed, blunt tooth, which grades evenly into the ventral margin of the petiole posteriorly. Ventral surface of petiole bulging medially. Petiolar peduncle long, comprising about two thirds of the total length of the petiole. Petiolar node squamiform: transition between peduncle and node marked by a rounded angle of ~90°, resulting in a weakly concave anterior node face that slightly overtops the peduncle; anterior face forming a slightly rounded ~90° angle with the dorsal face, which rounds evenly into the very short posterior face. Postpetiole evenly rounded and bulging anterodorsally, before flattening posterodorsally; weakly lobed ventrally.

In dorsal view, humeri very weakly developed: rounded and only slightly wider than the rest of the mesosoma; mesothoracic spiracles protruding past the lateral margins of the mesosoma, noticeable as slight angles where the pronotum joins the rest of the mesosoma. Metanotal groove absent: mesonotum and propodeum completely fused and converging evenly to the bases of the propodeal spines. Propodeal spines broadly approximated basally and diverging apically, their apices separated from each other by their length; negative space between them a “U”. Petiolar peduncle with spiracles protruding past the lateral margins, the peduncle broadened where they arise. Petiolar node squamiform: rounded anteriorly, with an indistinct posterior face, much wider than the peduncle, and narrowing into the caudal cylinder, which is narrower than the node. Postpetiole very broad (PWI 219–262) and campaniform, articulating with nearly the entire anterior margin of the gaster, but leaving angulate corners of the gaster exposed on each side. Anterior margin of the postpetiole broadly convex, meeting the lateral margins at rounded angles; lateral margins diverge evenly anteriorly, but flare laterally as they approach the gaster; posterior margin broadly concave. Metafemur moderately to strongly incrassate (FI 247–443).

Sculpture: median clypeal carina present, flanked by two equally strong carinae over weakly areolate-costulate ground sculpture. Antennal scapes weakly areolate and appearing dull. Cephalic dorsum and lateral surfaces of head densely areolate; ventral surface of head shining through weaker areolate sculpture. Mesosoma areolate-costulate, with cross-reticulations on the dorsal face of the pronotum; pronotal declivity lacks costulae; area between the propodeal spiracle and base of the propodeal spines, as well as the propodeal declivity, shining through weak areolae. Femora weakly shining through areolate sculpture. Petiolar peduncle, venter, and anterior face of the petiolar node shining through shallow areolate sculpture; dorsal face of the petiolar node more densely areolate. Dorsal surface of postpetiole dull with areolate sculpture. Gaster smooth and shining, without spectral iridescence on the first tergite. Surface of the first gastral sternite smooth and shining.

Setae: antennal scapes and funiculi with short, adpressed pilosity. Dorsum of head, mesosoma, waist segments and gaster with short, erect, blunt-tipped setae, the longest of which are roughly the width of the compound eye. The head bears ~38, mesosoma ~36, petiole 4, postpetiole ~14, and first gastral tergite ~46 setae. Sparse, adpressed pubescence present on the entire body, but may be difficult to discern from the dense ground sculpture on most of the body.

Color: antennae, head capsule, postpetiole, and gaster medium brown. Mandibles, clypeus, mesosoma, legs, and petiole testaceous yellow. Tarsi and sting are yellowish.

**Gyne:** see Baroni Urbani, 1978.

**Male:** see Baroni Urbani, 1978.

**Etymology:** Geographical, in reference to the type locality of Andros Island.

**Comments:** Similar to other members of the *pastinifer* and *pulchellus* groups (e.g., *T. pastinifer, T. rutabulafer* sp. nov., *T. torrei*), *T. androsanus* appears to have a Bahamian-Cuban distribution. This species, like other members of the *pastinifer* group, is only known from littoral habitats. Wheeler notes that the syntype workers were found under dead palmetto leaves near Crawl Creek in present day Southern Andros, but that the nests were not located. This species, like other members of the *pastinifer* and *pulchellus* groups, most likely nests in dead twigs in the leaf litter. A dark form of *T. androsanus* is present on the Exuma Cays: other than being uniformly dark brown, it differs from the type series by having relatively weakly incrassate femora, relatively narrow petiolar node, and having a smooth and shining dorsum of petiole and postpetiole (Figs. 122D–122F). If these two forms represent different species, they are surely close relatives. However, despite these morphological differences I unite the two forms as one species here, as I have not yet seen evidence of the two forms existing in sympatry.

***Temnothorax nigricans* (Baroni Urbani, 1978)**

**Figure 123:**
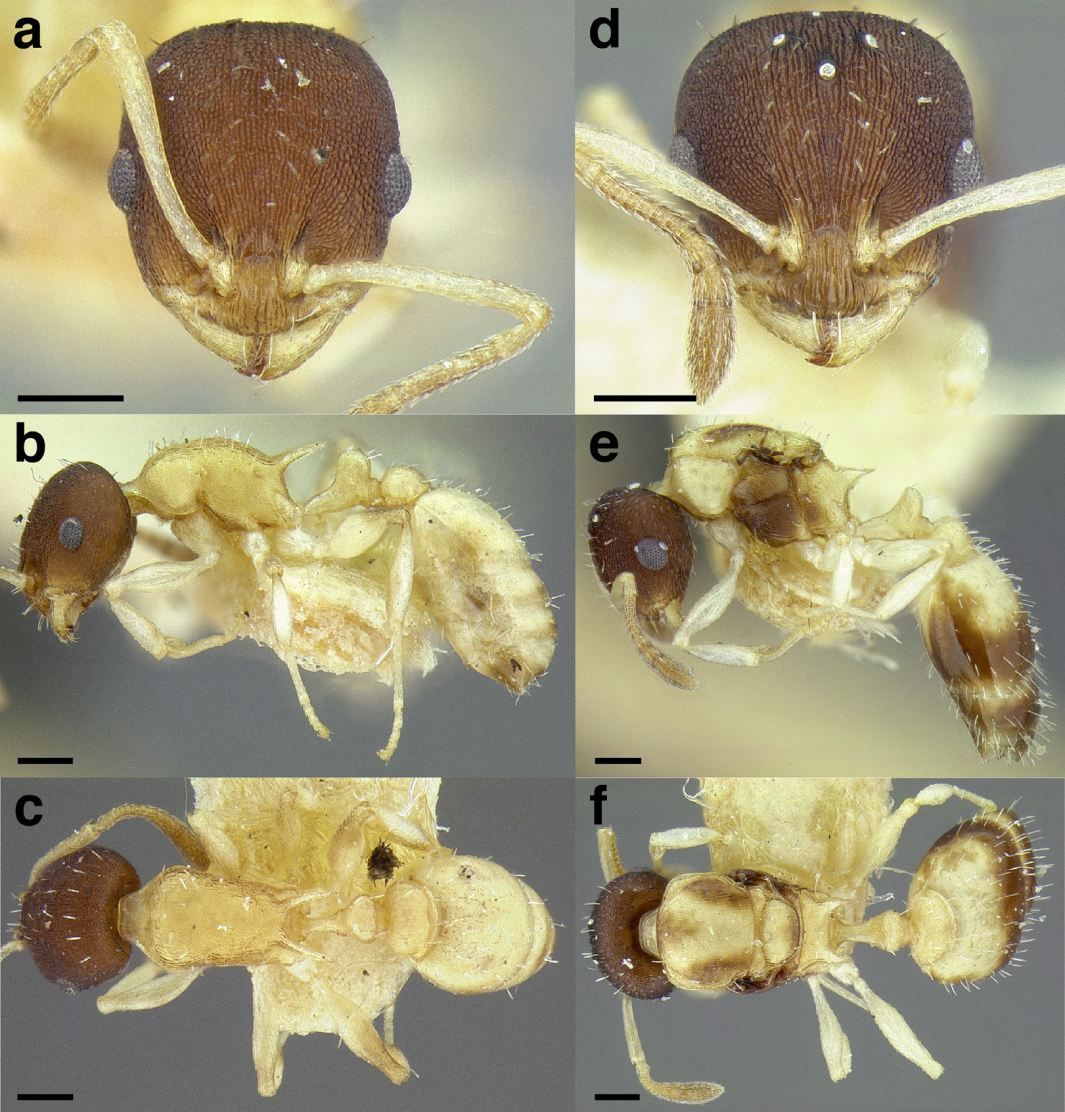
*Temnothorax nigricans*. (A–C) Lectotype worker (MCZENT00577111). (A) Full-face. (B) Profile view. (C) Dorsal view. (D–F) Paralectotype gyne (MCZENT00577113). (D) Full-face. (E) Profile view. (F) Dorsal view. Scale bars 0.2 mm.

Distribution: Fig. 121B; worker & gyne: Fig. 123.

*Macromischa melanocephala* Wheeler, 1931: 15. Syntype workers and gynes. Ensenada de Cochinos, Cuba. One syntype worker here designated **lectotype**.

*Leptothorax nigricans*: Baroni Urbani, 1978: 467. Transfer to *Leptothorax*. Nomen novum for *Leptothorax melanocephalum* (Wheeler) (nec *Leptothorax melanocephalum* Emery, 1870: 197).

*Temnothorax nigricans* (Baroni Urbani): Bolton, 2003: 271. First combination in *Temnothorax*.

**Type material examined:** *Lectotype worker:* CUBA: Artemisia: Hacienda Jiqui, Ensenada de Cochinos, 5 October 1929, J.G. Myers#C250, nest in fallen mahogany fruit, M.C.Z. Cotype 16365 (MCZENT00577110, middle specimen on pin) [MCZC].

*Paralectotype workers and gynes:* same pin as lectotype, 2 workers (top and bottom specimens on pin) [MCZC]; same data as lectotype, 3 workers (MCZENT00577111) [MCZC] 2 workers (MCZENT00577112) [MCZC] 2 dealate gynes (MCZENT00577113) [MCZC] 3 workers (MCZENT00577114) [MCZC] 3 workers (MCZENT00016365) [MCZC] 3 workers (CASENT0758264) [USNM] 3 workers (CASENT0758265) [USNM] 1 worker (images of CASENT0915381 examined on antweb.org) [MNHN].

**Geographic range:** Low elevations of Cuba (Artemisia) (Fig. 121B).

**Worker diagnosis:** *Temnothorax nigricans* can be separated from all other species in the *salvini* clade by the following character combination: antennal scapes very long, surpassing the posterior margin of head by about one and a half times the maximum width of the antennal scape (SI 92–100); anterior margin of clypeus entire; transition between anterior and dorsal faces of pronotum continuous, not marked by a transition or a weak transverse carina; in profile view, mesosoma strongly convex dorsally and compact (WLI 122–132); in dorsal view, mesosoma posterior to the pronotum swollen, only slightly narrower than the pronotum; dorsum of propodeum with setae; in dorsal view, propodeal spines broadly approximated basally, negative space between them “U” shaped; propodeal spines about as long as the propodeal declivity, directed posterodorsally, and weakly downcurved; hind femora weakly to moderately incrassate (FI 224–291); petiolar node weakly squamiform, dorsally rounded; dorsum of petiole with four erect setae; in dorsal view, petiolar node slightly broader than the caudal cylinder (PNWI < 160); postpetiole moderately to very broad (PWI 212–239); dorsum of head uniformly areolate; dorsum of postpetiole with > 10 erect setae; integument bicolored: antennal funiculus, clypeus, and distal third of the femora testaceous yellow; head capsule dark brown; mandibles, mesosoma, legs, waist segments, and gaster light yellow.

**Similar species:** *Temnothorax misomoschus* sp. nov., *T. subditivus*, and other members of the *pastinifer* group. *Temnothorax nigricans* can be distinguished from *T. subditivus* by the narrower petiolar node in dorsal view (PNWI < 160 vs. > 160 in *T. subditivus*), the somewhat shorter petiolar peduncle (~2/3 of the total petiole length in *T. nigricans* vs. ~ 3/4 in *T. subditivus*) and the sculpture of the head dorsum (uniformly areolate in *T. nigricans* vs. variable in *T. subditivus*, but never uniformly areolate). In contrast to *T. nigricans, T. misomoschus* sp. nov. is devoid of erect setae on most surfaces of the body, has a depressed propodeum, and a posteriorly leaning, subquadrate petiolar node. *Temnothorax nigricans* can be separated from other members of the *pastinifer* group by the areolate head (smooth and shining in *T. schwarzi*), weakly convex anterior clypeus margin (strongly emarginate in *T. pastinifer*), moderately long antennal scape, which surpasses the posterior margin of the head by about one and a half times the maximum width of the antennal scape (barely surpassing the posterior margin in *T. androsanus*), the continuous transition between the anterior and dorsal faces of the pronotum in profile view (transition distinct in *T. androsanus*). *Temnothorax nigricans* can also be separated from *T. rutabulafer* sp. nov. by the relatively narrow petiolar node in dorsal view (PNWI 130–149 vs. > 180 in *T. rutabulafer* sp. nov.).

**Worker measurements & indices (*n* = 7):** SL = 0.454–0.512 (0.477); FRS = 0.169–0.189 (0.179); CW = 0.502–0.573 (0.537); CWb = 0.452–0.524 (0.489); PoOC = 0.211–0.237 (0.226); CL = 0.532–0.602 (0.570); EL = 0.115–0.131 (0.123); EW = 0.090–0.104 (0.097); MD = 0.135–0.154 (0.142); WL = 0.574–0.667 (0.619); SPST = 0.244–0.289 (0.263); MPST = 0.192–0.233 (0.213); PEL = 0.212–0.278 (0.248); NOL = 0.131–0.177 (0.149); NOH = 0.105–0.141 (0.120); PEH = 0.182–0.234 (0.210); PPL = 0.131–0.158 (0.147); PPH = 0.163–0.198 (0.182); PW = 0.347–0.393 (0.369); SBPA = 0.143–0.175 (0.156); SPTI = 0.226–0.264 (0.244); PEW = 0.110–0.142 (0.124); PNW = 0.154–0.189 (0.173); PPW = 0.259–0.303 (0.283); HFL = 0.419–0.491 (0.465); HFWmax = 0.122–0.153 (0.136); HFWmin = 0.047–0.059 (0.054); CS = 0.718–0.825 (0.774); ES = 0.160–0.183 (0.172); SI = 92–100 (98); OI = 22–23 (22); CI = 84–89 (86); WLI = 122–132 (127); SBI = 30–35 (32); PSI = 41–45 (42); PWI = 212–239 (229); PLI = 159–180 (168); NI = 115–131 (124); PNWI = 130–149 (140); NLI = 55–71 (60); FI = 224–291 (254).

**Worker description:** In full-face view, head subquadrate, longer than broad (CI 84–89). Mandibles finely striate, shining, and armed with five teeth: the apical-most well developed, followed by a less developed preapical tooth and three equally developed smaller teeth. Anterior clypeal margin flat to evenly convex medially. Antennal scapes very long: when fully retracted, surpassing the posterior margin of the head capsule by about one and a half times the maximum width of the antennal scape (SI 92–100). Antennae 12-segmented; antennal club of three segments, with the apical-most segment longer than the preceding two in combination. Frontal carinae short, extending past the antennal toruli by about two times the maximum width of the antennal scape. Compound eyes moderately protruding past the lateral margins of the head capsule. Lateral margin of head weakly convex, converging evenly to the mandibular insertions. Posterior head margin flat to very slightly concave, rounding evenly into the lateral margins.

In profile view, compound eyes ovular and moderately large (OI 22–23), with 12 ommatidia in longest row. Pronotal declivity indistinct, but pronotal neck and anterior face of pronotum forming a rounded ~120° angle. Mesosoma evenly, but weakly, convex dorsally from where it joins the pronotal neck to the propodeal spines. Promesonotal suture extending from the posterior margin of the procoxal insertion only to the mesothoracic spiracle, which moderately well developed. Metanotal groove nearly entirely absent: only visible as a faint disruption in the surface sculpture between meso- and metacoxal insertions to the minute metathoracic spiracle, which is indistinguishable from the ground sculpture. Propodeal spiracle well developed, directed posterolaterally, and separated from the propodeal declivity by about two and a half spiracle diameters. Propodeal spines well developed and long (PSI 41–45), longer than the propodeal declivity, tapering evenly from the base, downcurved for most of their length but turning up slightly at the tips, and acute. Propodeal declivity weakly concave, forming a rounded ~110° angle with the base of the propodeal spines. Propodeal lobes rounded and weakly developed, but with an angulate dorsal flange. Metapleural gland bulla moderately large, extending from the metacoxal insertion two thirds of the way to the propodeal spiracle. Petiole moderately long (PLI 159–180), without tubercles anterodorsally. Subpetiolar process in the form of a weakly developed, acute tooth, which grades evenly into the ventral margin of the petiole posteriorly. Ventral surface of petiole bulging medially. Petiolar peduncle long, comprising about two thirds of the total length of the petiole. Petiolar node squamiform: transition between peduncle and node marked by a rounded angle of ~110°; anterior face of the node is straight and does not overhang the peduncle; anterior face rounds evenly into the strongly convex dorsal face, which rounds evenly into the posterior face. Postpetiole evenly rounded anterodorsally, before flattening posterodorsally; flat ventrally.

In dorsal view, humeri very weakly developed: rounded and only slightly wider than the rest of the mesosoma; mesothoracic spiracles not protruding past the lateral margins of the mesosoma. Metanotal groove absent: mesonotum and propodeum completely fused and converging evenly to the bases of the propodeal spines. Propodeal spines broadly approximated basally and diverging apically, their apices separated from each other by slightly less than their length; negative space between them a “U”. Petiolar peduncle with spiracles not protruding past the lateral margins. Petiolar node squamiform: rounded anteriorly, with a flattened posterior face, much wider than the peduncle, and narrowing into the caudal cylinder, which is narrower than the node. Postpetiole moderately to very broad (PWI 212–239) and campaniform, articulating with nearly the entire anterior margin of the gaster, but leaving angulate corners of the gaster exposed on each side. Anterior margin of the postpetiole broadly convex, meeting the lateral margins at rounded angles; lateral margins diverging evenly posteriorly; posterior margin flat. Metafemur weakly to moderately incrassate (FI 224–291).

Sculpture: median clypeal carina present, flanked by two slightly weaker carinae over areolate-costulate ground sculpture. Antennal scapes weakly areolate and dull. Cephalic dorsum and lateral surfaces of head with dense areolae arranged into longitudinal rows by costulae; ventral surface shining through weaker areolate sculpture. Pronotal neck areolate. Lateral face of pronotum with sculpture similar to the cephalic dorsum. Lateral face of propodeum, meso- and metapleurae uniformly areolate. Dorsally, mesosoma sculptured similarly to the cephalic dorsum. Areolate sculpture weaker on the propodeal declivity. Femora weakly shining through areolate sculpture. Petiolar peduncle, venter, anterior, and posterior faces of the petiolar node weakly shining through shallow areolate sculpture; dorsal face of the petiolar node more densely areolate. Dorsal surface of postpetiole dull with areolate sculpture and costulae. Gaster smooth and shining, with weak spectral iridescence on the first tergite. Surface of the first gastral sternite smooth and shining.

Setae: antennal scapes and funiculi with short, adpressed pilosity. Dorsum of head, mesosoma, waist segments and gaster with short, erect, blunt-tipped setae, the longest of which are roughly the width of the compound eye. The head bears ~32, mesosoma ~22, petiole 6, postpetiole ~10, and first gastral tergite ~36 setae. Sparse, adpressed pubescence present on the entire body, but may be difficult to discern from the dense ground sculpture and light integument on most of the body.

Color: funiculus of the antenna, clypeus, and distal third of the femora testaceous yellow. Head capsule dark brown. Mandibles, mesosoma, legs, waist segments, and gaster light yellow.

**Gyne measurements & indices (*n* = 2):** SL = 0.469–0.520 (0.495); FRS = 0.212–0.230 (0.221); CW = 0.637–0.687 (0.662); CWb = 0.590–0.646 (0.618); PoOC = 0.245–0.251 (0.248); CL = 0.627–0.675 (0.651); EL = 0.173–0.183 (0.178); EW = 0.132–0.158 (0.145); MD = 0.155–0.160 (0.158); WL = 0.903–1.008 (0.956); SPST = 0.259–0.269 (0.264); MPST = 0.262–0.265 (0.264); PEL = 0.287–0.317 (0.302); NOL = 0.160–0.175 (0.168); NOH = 0.131–0.164 (0.148); PEH = 0.257–0.287 (0.272); PPL = 0.182–0.187 (0.185); PPH = 0.226–0.250 (0.238); PW = 0.554–0.628 (0.591); SBPA = 0.287–0.345 (0.316); SPTI = 0.273–0.312 (0.293); PEW = 0.149–0.174 (0.162); PNW = 0.194–0.213 (0.204); PPW = 0.371–0.432 (0.402); HFL = 0.535–0.589 (0.562); HFWmax = 0.128–0.144 (0.136); HFWmin = 0.056–0.059 (0.058); CS = 0.904–0.984 (0.944); ES = 0.239–0.262 (0.251); SI = 79–80 (80); OI = 26–27 (27); CI = 94–96 (95); WLI = 153–156 (155); SBI = 49–53 (51); PSI = 27–29 (28); PWI = 248–249 (249); PLI = 153–174 (164); NI = 107–122 (114); PNWI = 122–130 (126); NLI = 55–56 (55); FI = 229–244 (236).

**Gyne description:** In full-face view, head subquadrate, about as long as broad (CI 94–96). Mandibles densely, finely striate, weakly shining, and armed with five teeth: the apical-most well developed, followed by a less developed preapical tooth and three equally developed smaller teeth. Anterior clypeal margin flat to weakly emarginated medially. Antennal scapes moderately long: when fully retracted, just reaching the posterior margin of the head capsule (SI 79–80). Antennae 12-segmented; antennal club composed of three segments, with the apical-most longer than the preceding two in combination. Frontal carinae moderately long, extending past the antennal toruli by about two times the maximum width of the antennal scape. Compound eyes moderately protruding past the lateral margins of the head capsule. Lateral margin of head evenly convex, converging from below the compound eyes to the mandibular insertions. Posterior head margin flat, rounding evenly into the lateral margins.

In profile view, compound eyes ovular and large (OI 26–27), with 17 ommatidia in longest row. Mesoscutum rounded evenly anteriorly, barely covering the dorsal surface of the pronotum, and weakly convex dorsally. Mesoscutellum slightly depressed below the level of the mesoscutum. Posterior margin of metanotum extending slightly past the posterior margin of the mesoscutum, the two sclerites form an even convexity. Propodeal spiracle well developed, directed posterolaterally, and separated from the propodeal declivity by about three and a half spiracle diameters. Propodeal spines stout, well developed, and moderately long (PSI 27–29), about as long as the propodeal declivity, tapering evenly from the base, directed posteriorly, straight, and acute. Propodeal declivity weakly concave, forming a rounded ~90° angle with the base of the propodeal spines. Propodeal lobes rounded and weakly developed, but with a flange that is slightly angulate dorsally. Metapleural gland bulla moderately large, extending from the metacoxal insertion two thirds of the way to the propodeal spiracle. Petiole moderately long (PLI 153–174), with peduncle evenly rounded anterodorsally. Subpetiolar process in the form of a weakly developed, acute tooth, which grades evenly into the ventral margin of the petiole posteriorly. Petiolar peduncle long, comprising about two thirds of the total length of the petiole. Petiolar node squamiform: transition between peduncle and node marked by a rounded angle of ~120°; anterior face transitioning joining the short dorsal face at a ~90° angle, which rounds evenly into the posterior face. Postpetiole evenly rounded anterodorsally, bulging slightly before it transitions into the flattened dorsal face; ventral surface weakly lobed.

In dorsal view, mesoscutum covering pronotum anteriorly, but humeri visible laterally as rounded sclerites. Propodeal spines weakly diverging basally but parallel apically, their apices separated from each other by about one and a half times their length; negative space between them a broad “U”. Petiolar peduncle with spiracles weakly protruding past the lateral margins. Petiolar node squamiform: flat anteriorly, with lateral faces strongly convex, and with a flattened posterior face, much wider than the peduncle, and narrowing into the caudal cylinder, which is narrower than the node. Postpetiole very broad (PWI 248–249) and campaniform, articulating with most of the anterior margin of the gaster, leaving small, angulate margins on each side exposed. Anterior margin of postpetiole weakly convex, with corners marked by rounded angles as it transitions to the lateral margins, which evenly diverge to the angulate posterior corners; posterior margin flat. Metafemur weakly incrassate (FI 229–244).

Sculpture: median clypeal lobe with about seven equally strong fine carinae, extending from the anterior margin nearly to frontal triangle. Lateral clypeal lobes with additional fine carinae; ground sculpture weakly areolate. Antennal scapes weakly shining through areolate sculpture. Cephalic dorsum and lateral surfaces of head with dense costulae over areolate sculpture; ventral surface shining through weaker areolate-costulate sculpture. Pronotal neck areolate. Pronotum, a posterior two thirds of the anepisternum and katepisternum, and lateral face of the propodeum with sculpture similar to that of the cephalic dorsum, but slightly weaker. Propodeal declivity weakly areolate-costulate. Mesoscutum and mesoscutellum with sculpture similar to that of the cephalic dorsum. Mesoscutellum smooth and shining medially, surrounded by costulae and areolae. Femora shining through weak areolate sculpture. Petiole with weak areolate sculpture on all surfaces, becoming stronger on the dorsoposterior surface of the node. Postpetiole densely areolate dorsally. Gaster predominantly smooth and shining, but with the anterior of the first gastral tergite finely areolate-costulate, and with faint spectral iridescence. Surface of the first gastral sternite smooth and shining.

Setae: antennal scapes and funiculi with short, adpressed pilosity. Dorsum of head, mesosoma, waist segments and gaster with short, erect, blunt-tipped setae, the longest of which are roughly three quarters of the compound eye width. Sparse, adpressed pubescence present on the entire body, but may be difficult to discern from the dense ground sculpture and light integument on most of the body.

Color: funiculus of the antenna, clypeus, and distal third of the femora testaceous yellow. Meso- and metapleurae, anteromedial and posterolateral portions of the mesoscutum, posterior edge of the mesoscutellum, metanotum, and posterior third of all gastral sclerites testaceous. Head capsule dark brown. Mandibles, mesosoma, legs, waist segments, and gaster light yellow.

**Male:** Unknown.

**Etymology:** Morphological, from the Latin ‘nigricans’ (= blackish), presumably in reference to the dark head.

**Comments:** In the original description by Wheeler (1931), the type series of *Temnothorax nigricans* was collected from the ground in a fruit of *Swietenia mahogani*, a hardwood species endemic to the Caribbean islands and southern Florida. The type locality is in northern Artemisia province on the west side of Cuba, which experiences a dry season typical of many islands in the Caribbean but receives substantial precipitation year-round, leading to this area being classified as having a tropical rainforest climate. In addition to the type locality, this species has been reported from numerous other localities across the island by Alayo (1974), including Ciénaga de Zapata, Cienfuegos, Caibarién, Buenos Aires, Escambray, and Seibabo. I have only been able to examine the original nest series, but the presence of multiple gynes suggests that this species may be polygynous. In his remarks on this species, Wheeler (1931) mused about the resemblance of the structure of the mesosoma to *Antillaemyrmex* Mann (a now defunct genus applied to what is presently referred to as the *pulchellus* group + *T. allardycei* (Mann)) and in petiolar shape to *Macromischa* (probably referring *T. pastinifer*). Our current understanding is that *Macromischa*, as defined by Mann (1920) with *T. purpuratus* (Roger) as the type for the genus, is polyphyletic: namely *T. subditivus*, *T. androsanus*, *T. pastinifer*, *T. fuscatus*, and *T. salvini* are all more closely related to *T. terricola* than they are to *T. purpuratus* (see Prebus, 2017), mostly concurring with Fontenla Rizo (2000), which inferred phylogenetic relationships within *Temnothorax* using morphology.

***Temnothorax pastinifer* (Emery, 1894)**

**Figure 124:**
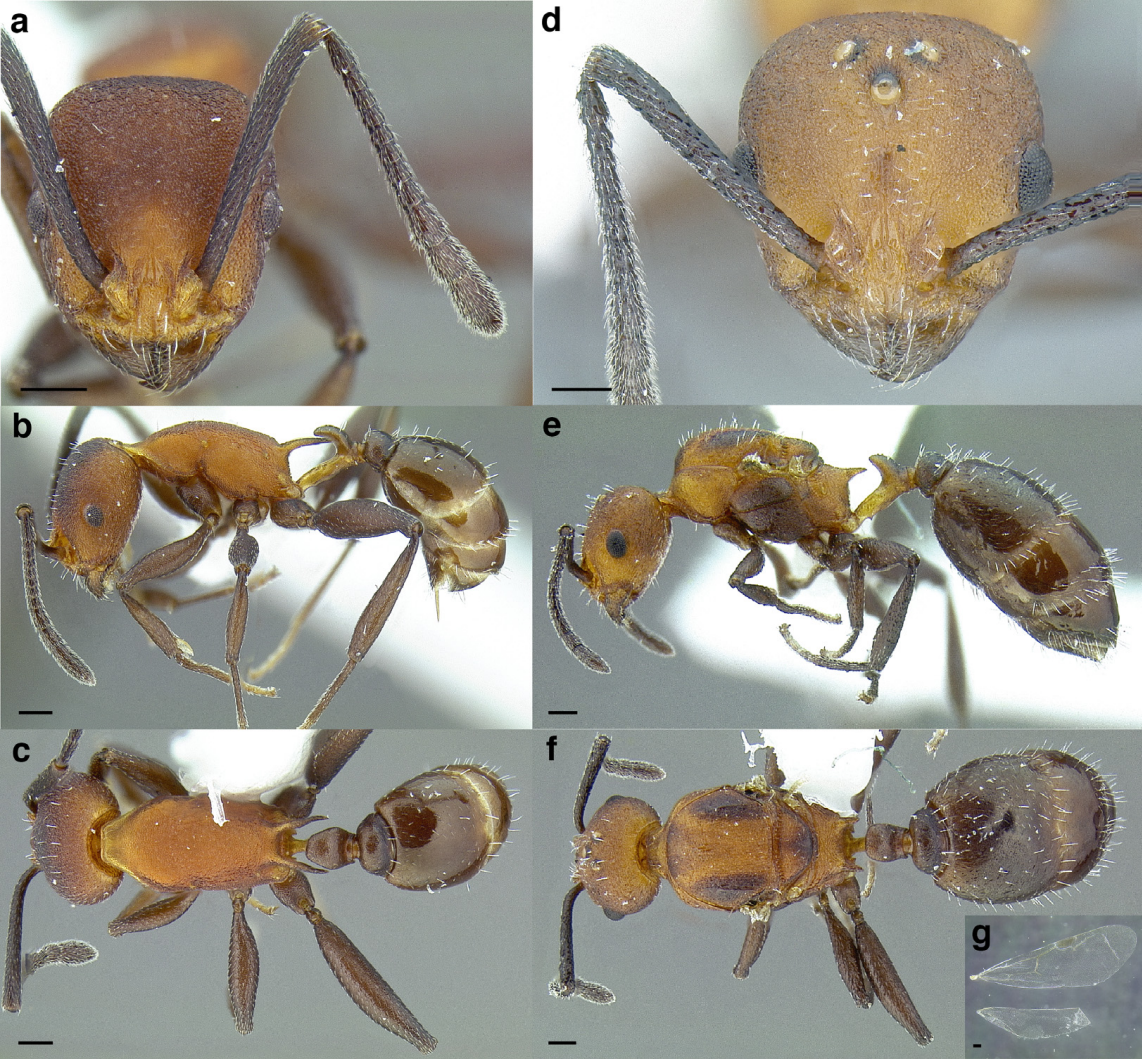
*Temnothorax pastinifer*. (A–C) Worker (CASENT0758861). (A) Full-face. (B) Profile view. (C) Dorsal view. (D–G) Gyne (CASENT0758861). (D) Full-face. (E) Profile view. (F) Dorsal view. (G) Wings. Scale bars 0.2 mm.

Distribution: Fig. 121C; worker & gyne: Fig. 124.

*Macromischa pastinifera* Emery, 1894: 164, pl. 1, fig. 18. Syntype workers. Bahamas. One syntype worker here designated **lectotype**.

*Macromischa lucayensis* Forel, 1901b: 340. Holotype gyne; junior synonym of *Temnothorax pastinifer* by Baroni Urbani, 1978: 471.

*Macromischa pastinifera* var. *opacipes* Wheeler, 1905: 96, fig. 1. Syntype workers; junior synonym of *Temnothorax pastinifer* by Smith, 1954: 6.

*Leptothorax pastinifer* (Emery): Baroni Urbani, 1978: 470 (in part) . First combination in *Leptothorax*.

*Leptothorax pastoris* Baroni Urbani, 1978: 475. Holotype worker; junior synonym of *Temnothorax pastinifer* by Fontenla Rizo, 1997: 51.

*Temnothorax pastinifer* (Emery): Bolton, 2003: 271. First combination in *Temnothorax*.

**Type material examined:** *Lectotype worker of* Macromischa pastinifera: BAHAMAS: Bahama Isola, [no collection date], Pergande No. 40, 1 worker (images of CASENT0904718 examined on antweb.org) [MSNG].

*Macromischa lucayensis holotype alate gyne:* BAHAMAS: [no locality data], [no collection date] 21, A. Forel, 1 alate gyne (images of CASENT0908986 examined on antweb.org) [MHNG].

*Leptothorax pastoris paratype worker:* CUBA: Santiago de Cuba: Siboney, [no collection date], I. Garcia#I-72, BMNH(E) 1014997, 1 worker (images of CASENT09101798 examined on antweb.org) [NHMUK].

**Type material not examined:** *Macromischa pastinifera var. opacipes syntype workers:* BAHAMAS: South Andros Island: Crawl Creek, Wheeler, syntype workers of *Macromischa pastinifera* var. *opacipes* [AMNH].

**Non-type material examined:** BAHAMAS: Berry Islands: Great Harbour Cay, Bamboo Cay, site 37, 29 January 2005, John Mangold, in wood, 1 alate gyne & 3 workers (CASENT0758857) [ABS] 4 workers (CASENT0758858) [ABS] 1 alate gyne & 3 workers (CASENT0758859) [ABS] 1 alate gyne & 3 workers (CASENT0758860) [ABS] 1 alate gyne & 3 workers (CASENT0758861) [ABS] 1 alate gyne & 3 workers (CASENT0758862) [ABS]; Exuma: 2 km SE Georgetown, 23.4833°N 75.75°W, 14 December 2006, J. Piovia-Scott, littoral vegetation, ground forager(s), 2 workers (CASENT0732596) [UCDC] 1 worker (CASENT0106606) [UCDC] 2 workers (CASENT0915977) [PSWC]; North Andros: Fresh Creek, [no collection date], W.M. Mann, 2 workers (CASENT0869170) [USNM]; West Grand Bahama: Water Cay, Wickham, [no collection date], [no collector data], 40, 1 worker (CASENT0758268) [USNM] 1 dealate gyne, postpetiole and gaster missing (CASENT0758269) [USNM].

**Geographic range:** Low elevations of the Bahamas and Cuba (Fig. 121C).

**Worker diagnosis:** *Temnothorax pastinifer* can be separated from all other species in the *salvini* clade by the following character combination: antennal scape very long, surpassing the posterior margin of the head by about a quarter of the total scape length; dorsum of head uniformly areolate; anterior margin of clypeus strongly emarginate medially; transition between anterior and dorsal faces of pronotum indistinct; in profile view, mesosoma strongly convex dorsally and moderately compact (WLI 133–143); in dorsal view, mesosoma posterior to the pronotum swollen, only slightly narrower than the pronotum; postpetiole moderately to very broad (PWI 174–227); dorsum of propodeum without setae; in dorsal view, propodeal spines broadly approximated basally, negative space between them “U” shaped; propodeal spines longer than the propodeal declivity, directed posteriorly, and downcurved; hind femora moderately to strongly incrassate (FI 269–320); petiolar node squamiform and arched anteriorly; in dorsal view, petiolar node moderately broader than the caudal cylinder (PNWI < 160); setae on head, mesosoma, waist segments and gaster erect, moderately long, sparse and blunt (never long and tapering); dorsum of petiole with two erect setae; postpetiole with < 6 erect setae; integument bicolored: mandibles, head capsule, mesosoma, peduncle and anterior face of petiolar node red; remainder of the body dark brown.

**Similar species:** *Temnothorax misomoschus* sp. nov., *T. subditivus*, and other members of the *pastinifer* group. *Temnothorax pastinifer* can be distinguished from *T. subditivus* by the narrower petiolar node in dorsal view (PNWI < 160 vs. > 160 in *T. subditivus*) and the sculpture of the head dorsum (uniformly areolate in *T. pastinifer* vs. variable in *T. subditivus*, but never uniformly areolate). In contrast to *T. pastinifer, T. misomoschus* sp. nov. is devoid of erect setae on most surfaces of the body, has a depressed propodeum, and a posteriorly leaning, subquadrate petiolar node. *Temnothorax pastinifer* can be separated from other members of the *pastinifer* group by the areolate head (smooth and shining in *T. schwarzi*), and the strongly emarginate anterior margin of the clypeus, which is entire to weakly impressed in all other members of the *pastinifer* group, except for *T. schwarzi*.

**Worker measurements & indices (*n* = 10):** SL = 0.828–0.923 (0.878); FRS = 0.224–0.322 (0.274); CW = 0.617–0.826 (0.776); CWb = 0.554–0.772 (0.716); PoOC = 0.282–0.366 (0.339); CL = 0.661–0.864 (0.823); EL = 0.142–0.180 (0.169); EW = 0.108–0.141 (0.126); MD = 0.165–0.239 (0.211); WL = 0.773–1.058 (0.985); SPST = 0.293–0.496 (0.413); MPST = 0.234–0.305 (0.285); PEL = 0.250–0.418 (0.360); NOL = 0.159–0.248 (0.221); NOH = 0.141–0.233 (0.204); PEH = 0.217–0.340 (0.303); PPL = 0.145–0.188 (0.167); PPH = 0.150–0.252 (0.221); PW = 0.438–0.582 (0.548); SBPA = 0.166–0.258 (0.230); SPTI = 0.229–0.359 (0.324); PEW = 0.125–0.183 (0.168); PNW = 0.176–0.294 (0.244); PPW = 0.217–0.413 (0.359); HFL = 0.784–1.035 (0.961); HFWmax = 0.172–0.225 (0.206); HFWmin = 0.060–0.082 (0.071); CS = 0.885–1.204 (1.128); ES = 0.196–0.245 (0.232); SI = 116–127 (120); OI = 20–22 (21); CI = 84–89 (87); WLI = 133–143 (137); SBI = 30–34 (32); PSI = 38–50 (42); PWI = 174–227 (215); PLI = 172–235 (212); NI = 91–121 (109); PNWI = 138–161 (145); NLI = 55–68 (61); FI = 269–320 (288).

**Worker description:** In full-face view, head subquadrate, longer than broad (CI 84–89). Mandibles densely, finely striate, but shining and armed with five teeth: the apical-most well developed and acute, followed by a less developed preapical tooth and three equally developed smaller teeth. Anterior clypeal margin strongly emarginated medially. Antennal scapes very long: when fully retracted, surpassing the posterior margin of the head capsule by about a quarter of their total length (SI 116–127). Antennae 12-segmented; antennal club of composed of three segments, with the apical-most segment as long as the preceding two in combination. Torular laminae strongly developed and triangular, roughly as wide as the distance that separates them. Frontal carinae short, extending past the torular laminae by less than the maximum width of the antennal scape. Compound eyes moderately protruding past the lateral margins of the head capsule. Lateral margin of head very weakly convex, forming a continuous arc from the mandibular insertions to the posterior margin of the head. Posterior head margin flat but rounding evenly into the lateral margins.

In profile view, compound eyes ovular and moderately large (OI 20–22), with 15 ommatidia in the longest row. Pronotal declivity indistinct, neck and anterior face of pronotum forming a ~130° angle. Mesosoma evenly convex from where it joins the pronotal neck to the propodeal spines, but pronotum is slightly planar. Promesonotal suture extending from the posterior margin of the procoxal insertion only to the mesothoracic spiracle, which is well developed. Metanotal groove visible as a disruption of the sculpture laterally from where it arises between the mid- and hind coxae to where it ends in the poorly developed metathoracic spiracle, which is nearly indistinguishable against the ground sculpture. Propodeal spiracle well developed, directed posteriorly, and separated from the propodeal declivity by about three and a half spiracle diameters. Propodeal spines well developed and long (PSI 38–50), longer than the propodeal declivity, tapering evenly from the base, strongly downcurved, and acute. In some specimens, the spines are abruptly bent at the basal third, and the apical two thirds are straight. Propodeal declivity straight, forming a rounded ~110° angle with the base of the propodeal spines. Propodeal lobes rounded and weakly developed, but with a slightly angulate dorsal flange. Metapleural gland bulla moderately large, extending from the metacoxal insertion two thirds of the way to the propodeal spiracle. Petiole long (PLI 172–235), without tubercles anterodorsally. Subpetiolar process in the form of a small, acute tooth; ventral margin of petiole bulging slightly posterior to it. Petiolar peduncle long: petiolar node comprising only about a quarter of the petiolar dorsum. Petiolar node strongly squamiform: transition between peduncle and node abrupt: marked by a rounded angle of ~90°; anterior face of the node is strongly concave and overhangs the peduncle; anterior face forming a ~90° angle with the very short dorsal face, which rounds evenly into the convex posterior face. Postpetiole evenly rounded and slightly bulging anteriorly, flattened dorsally, and weakly lobed ventrally. Sting very well developed, slightly longer than the first gastral sternite.

In dorsal view, humeri strongly developed and distinct: when viewed at an oblique angle, the dorsal face of the pronotum transitions to the lateral face through an angle; mesothoracic spiracles weakly protruding past the lateral margins of the mesosoma, visible as slight angles where the pronotum meets the mesonotum. Promesonotal suture visible as a slight disruption in the surface sculpture. Metanotal groove absent: mesonotum and propodeum completely fused and lateral margins converging evenly to the bases of the propodeal spines. Propodeal spines broadly approximated basally and diverging apically, but slightly in-curved at the tips in some specimens. Spine apices separated from each other by about two thirds of their length, the negative space between them an elongated “U”. Petiolar peduncle with spiracles strongly protruding past the lateral margins, but not noticeably constricted anterior to them. Petiolar node, when viewed posterodorsally, spade-shaped: dorsal margin flat, meeting the lateral margins at an angle; lateral margins evenly convex and widest medially. Petiolar node wider than the peduncle, and evenly grading into the caudal cylinder, which is narrower than the node. Postpetiole moderately to very broad (PWI 174–227) and campaniform, articulating with the nearly the entire anterior margin of the gaster, but leaving angulate corners of the gaster exposed on each side. Anterior margin of the postpetiole broadly convex and evenly rounds into the lateral margins, which diverge to the angulate posterior corners; posterior margin broadly concave. Metafemur moderately to strongly incrassate (FI 269–).

Sculpture: median clypeal carina present, extending posteriorly to the level of the antennal toruli, and flanked on either side by two weaker carinae. Lateral clypeal lobes with additional, weaker carinae; ground sculpture areolate. Antennal scapes densely areolate. All surfaces of the head densely areolate, but sculpture becomes weaker ventrally. Nearly all surfaces of the mesosoma densely areolate, with very fine, predominantly costulate sculpture overlying the ground sculpture; propodeal declivity shallowly areolate. Femora densely areolate. Petiole shining through weak areolate sculpture on most surfaces, but in some specimens the posterior face and base of the petiolar node opaque, with denser areolae. Postpetiole sculpture matching that of the posterior face of the petiolar node. Gaster with traces of shallow areolate sculpture on the basal quarter of the first gastral tergite, which is otherwise smooth and shining, with weak to moderate spectral iridescence; first gastral sternite smooth and shining, with weak to moderate spectral iridescence.

Setae: antennal scapes and funiculi with short, decumbent pilosity, with several longer setae apically. Dorsum of the head, pronotum, waist segments, and gaster with sparse, erect, blunt-tipped setae, the longest of which are about the width of the compound eye. The head bears ~20, mesosoma ~6 restricted to the pronotum, petiole 2, postpetiole ~6, and first gastral tergite ~12 setae. Short, sparse pubescence present over the entire body, but difficult to detect against the ground sculpture.

Color: mandibles, head capsule, mesosoma, peduncle and anterior face of petiolar node red; remainder of the body dark brown.

**Gyne measurements & indices (*n* = 2):** SL = 0.856–0.895 (0.876); FRS = 0.323–0.332 (0.328); CW = 0.897–0.906 (0.902); CWb = 0.815–0.839 (0.827); PoOC = 0.359–0.362 (0.361); CL = 0.897–0.900 (0.899); EL = 0.216–0.237 (0.227); EW = 0.168–0.180 (0.174); MD = 0.214–0.219 (0.217); WL = 1.443–1.465 (1.454); SPST = 0.301–0.394 (0.348); MPST = 0.362–0.386 (0.374); PEL = 0.480–0.510 (0.495); NOL = 0.239–0.260 (0.250); NOH = 0.235–0.239 (0.237); PEH = 0.380–0.389 (0.385); PPL = 0.207; PPH = 0.323; PW = 0.822–0.934 (0.878); SBPA = 0.410–0.422 (0.416); SPTI = 0.361–0.423 (0.392); PEW = 0.224–0.247 (0.236); PNW = 0.273–0.312 (0.293); PPW = 0.524; HFL = 0.935–1.026 (0.981); HFWmax = 0.168–0.211 (0.190); HFWmin = 0.083–0.084 (0.084); CS = 1.265–1.288 (1.276); ES = 0.300–0.327 (0.314); SI = 105–107 (106); OI = 24–25 (25); CI = 91–94 (92); WLI = 172–180 (176); SBI = 49–52 (50); PSI = 21–27 (24); PWI = 234; PLI = 232; NI = 100–111 (105); PNWI = 122–126 (124); NLI = 50–51 (50); FI = 200–254 (227).

**Gyne description:** In full-face view, head subquadrate, about as long as broad (CI 91–94). Mandibles densely, finely striate, but shining and armed with five teeth: the apical-most well developed and acute, followed by a less developed preapical tooth and three equally developed smaller teeth. Anterior clypeal margin weakly strongly emarginated medially. Antennal scapes very long: when fully retracted, surpassing the posterior margin of the head capsule by about a quarter of their total length (SI 105–107). Antennae 12-segmented; antennal club composed of three segments, with the apical-most segment as long as the preceding two in combination. Torular laminae strongly developed and triangular, roughly as wide as the distance that separates them. Frontal carinae short, extending past the torular laminae by about the maximum width of the antennal scape. Compound eyes moderately protruding past the lateral margins of the head capsule. Lateral margin of head evenly convex, converging from below the compound eyes to the mandibular insertions. Posterior head margin flat, rounding evenly into the lateral margins.

In profile view, compound eyes ovular and moderately large (OI 24–25), with 25 ommatidia in longest row. Mesoscutum rounded evenly anteriorly, covering the dorsal surface of the pronotum, and weakly convex dorsally. Mesoscutellum slightly depressed below the level of the mesoscutum. Posterior margin of metanotum extending slightly past the posterior margin of the mesoscutum. Propodeal spiracle well developed, directed posterolaterally, and separated from the propodeal declivity by about three and a half spiracle diameters. Propodeal spines stout and moderately well developed, but short (PSI 21–27), about a third as long as the propodeal declivity, tapering evenly from the base, directed posteriorly, slightly downcurved, and acute. Propodeal declivity straight and flat, forming a rounded ~100° angle with the base of the propodeal spines. Propodeal lobes rounded and very weakly developed. Metapleural gland bulla moderately large, extending from the metacoxal insertion two thirds of the way to the propodeal spiracle. Petiole long (PLI 232), without tubercles anterodorsally. Subpetiolar process in the form of a small, very acute tooth; ventral margin of petiole bulging slightly posterior to it. Petiolar peduncle long: petiolar node comprising only about a quarter of the petiolar dorsum. Petiolar node strongly squamiform: transition between peduncle and node abrupt: marked by a rounded angle of ~90°; anterior face of the node is strongly concave and overhangs the peduncle; anterior face forming a ~90° angle with the very short dorsal face, which rounds evenly into the convex posterior face. Postpetiole evenly rounded and slightly bulging anteriorly, flattened dorsally, and weakly lobed ventrally.

In dorsal view, mesoscutum covering pronotum anteriorly, but humeri visible laterally as rounded sclerites. Propodeal spines short and weakly diverging apically, their apices separated from each other by about one and a half times their length, the negative space between them a truncated “U”. Petiolar peduncle with spiracles strongly protruding past the lateral margins, but not noticeably constricted anterior to them. Petiolar node, when viewed posterodorsally, spade-shaped: dorsal margin flat, meeting the lateral margins at an angle; lateral margins evenly convex and widest medially. Petiolar node wider than the peduncle, and evenly grading into the caudal cylinder, which is narrower than the node. Postpetiole very broad (PWI 234) and campaniform, articulating with the nearly the entire anterior margin of the gaster, but leaving angulate corners of the gaster exposed on each side. Anterior margin of postpetiole broadly convex and evenly rounding into the lateral margins, which diverge to the angulate posterior corners; posterior margin broadly concave. Metafemur weakly to moderately incrassate (FI 200–254).

Sculpture: median clypeal carina present, extending from the anterior margin of the clypeus to the level of the antennal toruli, and flanked two by weaker carinae. Lateral clypeal lobes with additional weaker carinae; ground sculpture areolate. Antennal scapes densely areolate. All surfaces of the head densely areolate and costulate, but sculpture becomes weaker ventrally. Nearly all surfaces of the mesosoma densely areolate, with fine, predominantly costulate sculpture overlying the ground sculpture; mesopleurae and propodeal declivity shallowly areolate. Femora appearing dull and shallowly areolate. Petiole shining through weak areolate sculpture on most surfaces, but in some specimens the posterior face and base of the petiolar node dull, with denser areolae. Postpetiole sculpture matching that of the posterior face of the petiolar node. Gaster with traces of shallow areolate sculpture on the basal eighth of the first gastral tergite, which is otherwise smooth and shining, with weak to moderate spectral iridescence; first gastral sternite smooth and shining, with weak to moderate spectral iridescence.

Setae: antennal scapes and funiculi with short, decumbent pilosity, with several longer setae apically. Dorsum of the head, pronotum, waist segments, and gaster with sparse, erect, blunt-tipped setae, the longest of which are about half the width of the compound eye. Short, sparse pubescence present over the entire body, but difficult to detect against the ground sculpture.

Color: mandibles, head capsule, mesosoma, peduncle and anterior face of petiolar node red; mesopleurae dark brown, mesoscutum with dark brown patches anteromedially and posterolaterally, and mesoscutellum dark brown medially; remainder of the body dark brown.

**Male:** Unknown. Mann, 1929 misidentified and described a male of *T. rutabulafer* sp. nov. as *T. pastinifer*.

**Etymology:** Morphological, from the Latin ‘pastinum’, a two-pronged digging instrument used for digging + ‘-fer’ (= bearing); presumably a reference to the long propodeal spines.

**Comments:** This species is found in the Bahamas and mainland Cuba, known primarily from collections made in littoral habitats. Baroni Urbani (1978) mistakenly states that the type locality of *Temnothorax pastinifer* is Bermuda. As for the presence of *T. pastinifer* on Cuba, I have only been able to confirm this by inspection of the type of *T. pastoris* (Baroni Urbani), from the coastal village of Siboney, Santiago de Cuba province. Beginning with Mann (1929), this species has a long history of being conflated with *T. rutabulafer* sp. nov., which is broadly sympatric with *T. pastinifer*. Most of the Cuban specimens and many of the Bahamian specimens that I have examined have proven to be *T. rutabulafer* sp. nov. When examining the collections at the United States National Museum collection at the Smithsonian, the source of this confusion became clear: Mann had collected both species in the same sample from North Andros and subsequently mounted one specimen of *T. rutabulafer* sp. nov. on the same pin with two specimens of *T. pastinifer* (CASENT0758267 & CASENT0869170, specimens on pin separated in this study). When Mann was describing the gyne and male of *T. pastinifer* (which are, in fact, *T. rutabulafer* sp. nov.) in his 1929 article from collections made from Cuba by Creighton, he apparently mistook this mixed pin for a representation of variation within the species. This mistake was replicated by Baroni Urbani (1978), who attempted to reconcile what he interpreted as morphological variation in a redescription of what is apparently a chimaera of the two species. The illustration of *T. pastinifer* in the article is apparently an attempt to blend the two species as well, showing a specimen with an emarginate anterior margin of the clypeus in dorsal view (anterior margin is described as weakly convex in the main article) and with a narrow petiolar node: characters that are consistent with *T. pastinifer*. However, the illustration in profile is apparently *T. rutabulafer* sp. nov., with abundant erect setae on the petiole (*T. pastinifer* only has two setae); the illustration also appears to misrepresent the propodeal spines, which are either strongly downcurved along their entire length, or abruptly bent at the basal third in *T. pastinifer* (more or less straight and posteriorly directed in *T. rutabulafer* sp. nov.).

***Temnothorax rutabulafer* sp. nov.**

**Figure 125:**
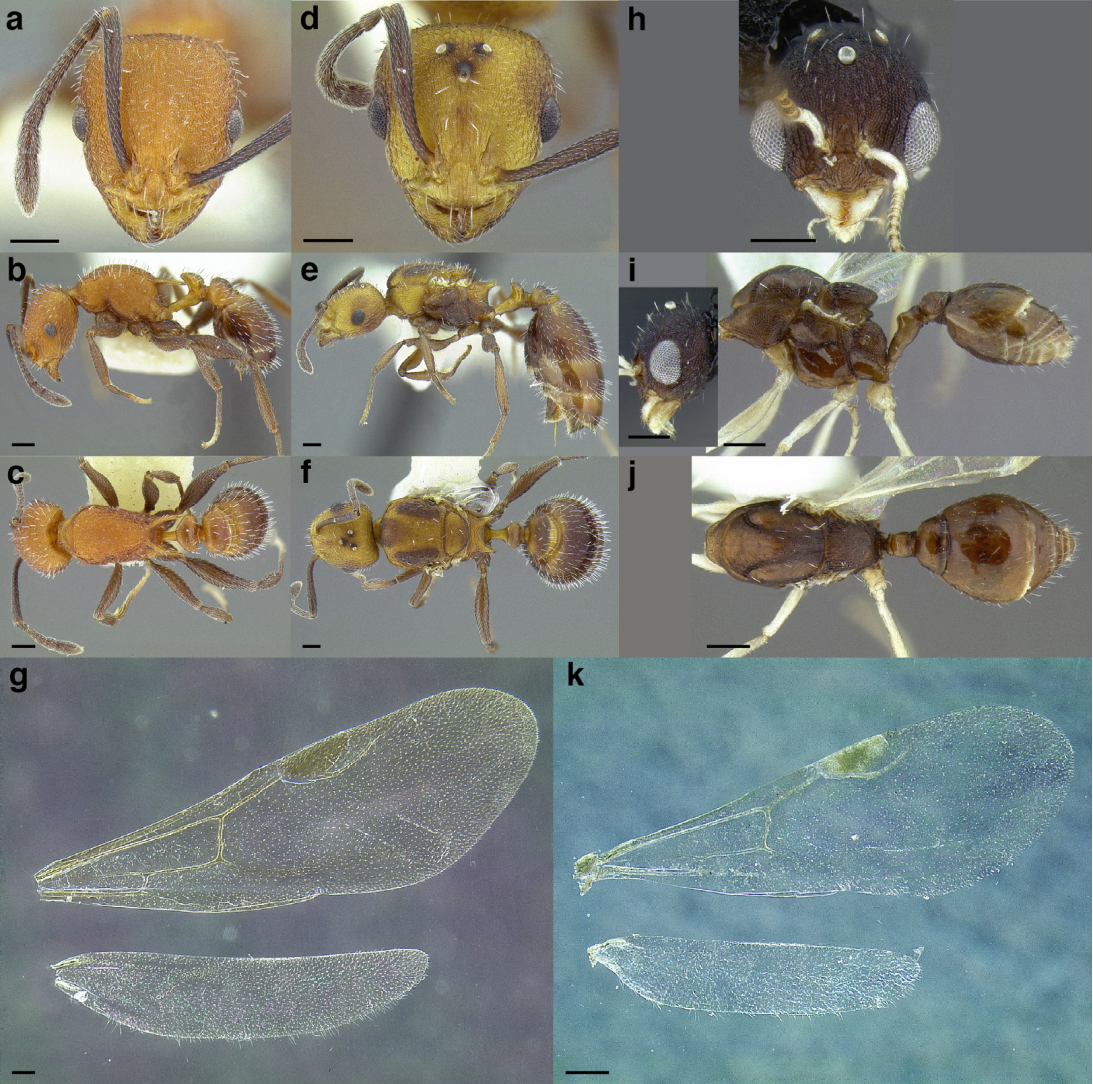
*Temnothorax rutabulafer* sp. nov. (A–C) Holotype worker (CASENT0758266). (A) Full-face. (B) Profile view. (C) Dorsal view. (D–G) Paratype gyne (CASENT0758831). (D) Full-face. (E) Profile view. (F) Dorsal view. (G) Wings. (H–K) Paratype male (CASENT0756085). (H) Full-face. (I) Profile view. (J) Dorsal view. (K) Wings. Scale bars 0.2 mm.

Distribution: Fig. 121d; worker, gyne & male: Fig. 125.

*Macromischa pastinifera* (Emery): Mann, 1929: 161 (in part). Gyne and male of *Temnothorax rutabulafer* described as *Temnothorax pastinifer*.

*Leptothorax pastinifer* (Emery): Baroni Urbani, 1978: 470 (in part). Worker of *Temnothorax rutabulafer* described as *Temnothorax pastinifer*.

*Leptothorax pastinifer* (Emery): Fontenla Rizo, 1997: 51 (in part). Workers of *Temnothorax rutabulafer* described as *Temnothorax pastinifer*.

**Type material examined:** *Holotype worker:* CUBA: Cienfuegos: Soledad, 20 November 1927, W.S. Creighton, (CASENT0758266, middle specimen on pin) [USNM].

*Paratype workers, gyne, and male:* same pin as holotype, 2 workers (top and bottom specimens on pin) [USNM]; same data as holotype: 1 dealate gyne, 1 male, 1 worker (CASENT0758230) [USNM] 34 workers (CASENT0758277-89) [USNM] 3 workers (MCZENT00581833) [MCZC].

**Non-type material examined:** BAHAMAS: Bimini: South Bimini Island, May 1951, Cazier & Gertsch, 1 worker (CASENT0758274) [USNM] 1 worker (CASENT0758275) [USNM]; June 1951, Cazier, C. & P. Vaurie, 1 male (CASENT0756085) [USNM] 1 worker (CASENT0758270) [USNM] 1 worker (CASENT0758272) [USNM] 1 dealate gyne (CASENT0756086) [USNM]; July 1951, C. & P. Vaurie, 1 worker (CASENT0758276) [USNM] 1 worker (CASENT0758273) [USNM] 1 worker (CASENT0758271) [USNM]. Central Andros: Fresh Creek, Andros Island, [no collection date], W.M. Mann, 1 worker (MCZENT00581832) [MCZC] 1 worker (CASENT0758267) [USNM]. North Andros: North Andros Island, north of Stafford Creek bridge, west side of road, rocky hill in from road, site B, 14 November 1996, M. Deyrup & L. Davis, dense unburned coppice, nest in 2 cm twig on ground, 1 alate gyne, 4 workers (CASENT0758831) [ABS] 5 workers (CASENT0758832) [ABS] 5 workers (CASENT0758833) [ABS] 5 workers (CASENT0758834) [ABS] 5 workers (CASENT0758835) [ABS] 5 workers (CASENT0758836) [ABS] 5 workers (CASENT0758837) [ABS] 1 alate gyne & 4 workers (CASENT0758838) [ABS] 5 workers (CASENT0758839) [ABS] 5 workers (CASENT0758840) [ABS] 5 workers (CASENT0758841) [ABS] 5 workers (CASENT0758842) [ABS] 5 workers (CASENT0758843) [ABS] 5 workers (CASENT0758844) [ABS] 5 workers (CASENT0758845) [ABS] 5 workers (CASENT0758846) [ABS]; same data as previous, except: 2 km N of Forfar, roadside coppice, in small branch on ground, 1 dealate gyne & 4 workers (CASENT0758847) [ABS] 4 workers (CASENT0758848) [ABS] 4 workers (CASENT0758849) [ABS] 4 workers (CASENT0758850) [ABS] 4 workers (CASENT0758851) [ABS] 4 workers (CASENT0758852) [ABS]; north of Stafford Creek bridge, west side of road, rocky hill in from road, dense unburned coppice, site B, 17 November 1996, M. Deyrup & L. Davis, nest in 2 cm twig on ground, 4 workers (CASENT0758853) [ABS] 4 workers (CASENT0758854) [ABS] 4 workers (CASENT0758855) [ABS] 3 workers (CASENT0758856) [ABS].

CUBA: Cienfuegos: Soledad, 18 November 1927, W.S. Creighton, 1 alate gyne & 3 workers (CASENT0759978) [FSCA]; Soledad, Blanco’s Woods, 22 July 1953, E.O. Wilson#Berl, 1 worker (MCZENT00581834) [MCZC] 3 workers (MCZENT00581835) [MCZC]; same data as previous, except: 12 June 1953, 1 teneral worker (MCZENT00581836) [MCZC].

**Geographic range:** Low elevations of the Bahamas and Cuba (Fig. 121D).

**Worker Diagnosis:** *Temnothorax rutabulafer* sp. nov. can be separated from all other species in the *salvini* clade by the following character combination: antennal scape very long, surpassing the posterior margin of the head by about two times the maximum width of the antennal scape; dorsum of head uniformly areolate; anterior margin of clypeus weakly convex to weakly emarginate medially; transition between anterior and dorsal faces of pronotum indistinct but marked by a change in sculpture; in profile view, mesosoma strongly convex dorsally and moderately compact (WLI 131–137); in dorsal view, mesosoma posterior to the pronotum swollen, only slightly narrower than the pronotum; dorsum of propodeum with setae; in dorsal view, propodeal spines broadly approximated basally, negative space between them “U” shaped; propodeal spines longer than the propodeal declivity, directed posteriorly, and downcurved; hind femora moderately to strongly incrassate (FI 271–344); petiolar node squamiform and weakly arched anteriorly; dorsum of petiole with twelve erect setae; in dorsal view, petiolar node much broader than the caudal cylinder (PNWI > 180); postpetiole very broad (PWI 234–266), with < 6 erect setae; setae on head, mesosoma, waist segments and gaster erect, moderately long, dense and blunt (never long and tapering); integument bicolored: mandibles, head capsule, mesosoma, peduncle, anterior face of petiolar node, lateral face of postpetiole, distal four tarsi, and base of gaster red; remainder of the body dark brown.

**Similar species:** *Temnothorax misomoschus* sp. nov., *T. subditivus*, and other members of the *pastinifer* group. *Temnothorax rutabulafer* sp. nov. can be distinguished from *T. subditivus* by the sculpture of the head dorsum (uniformly areolate in *T. androsanus* vs. variable in *T. subditivus*, but never uniformly areolate), and the very broad postpetiole (PWI < 220 in *T. subditivus*). In contrast to *T. rutabulafer* sp. nov., *T. misomoschus* sp. nov. is devoid of erect setae on most surfaces of the body, has a depressed propodeum, and a posteriorly leaning, subquadrate petiolar node. *Temnothorax pastinifer* sp. nov. can be separated from other members of the *pastinifer* group by the areolate head (smooth and shining in *T. schwarzi*), the abundant and dense erect setae on all surfaces of the body, and the very broad petiolar node (PNWI < 160 in all other *pastinifer* group species, except *T. schwarzi*).

**Worker measurements & indices (*n* = 14):** SL = 0.555–0.688 (0.639); FRS = 0.170–0.242 (0.192); CW = 0.536–0.728 (0.660); CWb = 0.488–0.676 (0.607); PoOC = 0.224–0.308 (0.276); CL = 0.579–0.752 (0.690); EL = 0.115–0.160 (0.142); EW = 0.090–0.123 (0.107); MD = 0.149–0.203 (0.183); WL = 0.654–0.899 (0.816); SPST = 0.290–0.406 (0.353); MPST = 0.230–0.300 (0.273); PEL = 0.279–0.361 (0.327); NOL = 0.128–0.199 (0.170); NOH = 0.126–0.204 (0.174); PEH = 0.219–0.297 (0.268); PPL = 0.145–0.208 (0.178); PPH = 0.165–0.265 (0.231); PW = 0.402–0.558 (0.500); SBPA = 0.152–0.260 (0.218); SPTI = 0.233–0.408 (0.318); PEW = 0.115–0.172 (0.153); PNW = 0.223–0.367 (0.303); PPW = 0.292–0.427 (0.377); HFL = 0.563–0.749 (0.663); HFWmax = 0.152–0.195 (0.173); HFWmin = 0.050–0.062 (0.057); CS = 0.778–1.052 (0.951); ES = 0.160–0.222 (0.195); SI = 100–114 (106); OI = 20–21 (21); CI = 84–90 (88); WLI = 131–137 (135); SBI = 31–40 (36); PSI = 40–48 (43); PWI = 234–266 (247); PLI = 160–208 (185); NI = 89–133 (98); PNWI = 178–224 (198); NLI = 42–57 (52); FI = 271–344 (302).

**Worker description:** In full-face view, head subquadrate, longer than broad (CI 84–90). Mandibles densely, finely striate, but shining and armed with five teeth: the apical-most well developed and acute, followed by a less developed preapical tooth and three equally developed smaller teeth. Anterior clypeal margin weakly convex to weakly emarginate medially. Antennal scapes very long: when fully retracted, surpassing the posterior margin of the head capsule by about three times the maximum width of the antennal scape (SI 100–14). Antennae 12-segmented; antennal club of composed of three segments, with the apical-most segment slightly longer than the preceding two in combination. Torular laminae not strongly developed: narrower than the distance that separates them. Frontal carinae short, extending past the antennal toruli by about the maximum width of the antennal scape. Lateral margin of head weakly convex, forming a continuous arc from the mandibular insertions to the posterior margin of the head. Posterior head margin flat but rounding evenly into the lateral margins. Compound eyes moderately protruding past the lateral margins of the head capsule.

In profile view, compound eyes ovular and moderately large (OI 20–21), with 15 ommatidia in the longest row. Pronotal declivity indistinct, but transition from the anterior to dorsal faces of the pronotum marked by a change in sculpture, from finely areolate to longitudinally rugose; neck and anterior face of pronotum forming a ~120° angle. Mesosoma evenly convex from where it joins the pronotal neck to the propodeal spines. Promesonotal suture extending from the posterior margin of the procoxal insertion only to the mesothoracic spiracle, which is well developed. Metanotal groove visible as a faint disruption of the sculpture laterally from where it arises between the mid- and hind coxae to where it ends in the poorly developed metathoracic spiracle, which is nearly indistinguishable from the ground sculpture. Propodeal spiracle well developed, directed posteriorly, and separated from the propodeal declivity by about two and a half spiracle diameters. Propodeal spines well developed (PSI 40–48), longer than the propodeal declivity, tapering evenly from the base, directed straight posteriorly, slightly upcurved at the tips in some specimens, and acute. Propodeal declivity flat, forming a rounded ~70° angle with the base of the propodeal spines. Propodeal lobes rounded and weakly developed. Metapleural gland bulla small, extending from the metacoxal insertion halfway to the propodeal spiracle. Petiole moderately long (PLI 160–208), without tubercles anterodorsally. Subpetiolar process in the form of a small, blunt tooth; ventral margin of petiole bulging slightly immediately anterior to where the petiolar node arises. Petiolar peduncle moderately long: petiolar node comprising about a third of the petiolar dorsum. Petiolar node strongly squamiform: transition between peduncle and node abrupt: marked by a rounded angle of ~90°; anterior face of the node is strongly concave and overhangs the peduncle; anterior face forming a ~90° angle with the very short dorsal face, which rounds evenly into the convex posterior face. Postpetiole evenly convex anterodorsally, and convex ventrally.

In dorsal view, humeri moderately well developed: evenly rounded and slightly wider than the rest of the mesosoma; mesothoracic spiracles protruding past the lateral margins of the mesosoma, visible as angles where the pronotum meets the mesonotum. Promesonotal suture visible as a slight disruption in the surface sculpture. Metanotal groove absent: mesonotum and propodeum completely fused and lateral margins converging evenly to the bases of the propodeal spines. Propodeal spines broadly approximated basally and strongly diverging in basally, becoming parallel apically before slightly diverging again at the tips. Apices of the propodeal spines separated from each other by about three quarters of their length, the negative space between them “U” shaped. Petiolar peduncle with spiracles weakly protruding past the lateral margins, but not noticeably constricted anterior to them. When viewed posterodorsally, the petiolar node spade-shaped as in *Temnothorax pastinifer*, but very broad transversely, nearly twice as wide as the caudal cylinder at its broadest point. Postpetiole very broad (PWI 234–266), anteroposteriorly compressed, and campaniform, articulating with the nearly the entire anterior margin of the gaster, but leaving angulate corners of the gaster exposed on each side. Anterior margin of the postpetiole broadly convex and evenly rounds into the lateral margins, which diverge slightly to the angulate posterior corners; posterior margin broadly concave. Metafemur moderately to strongly incrassate (FI 271–344).

Sculpture: median clypeal carina present, extending posteriorly nearly to the frontal triangle, and flanked on either side by two slightly weaker carinae. Lateral clypeal lobes with additional, weaker carinae; ground sculpture areolate. Antennal scapes densely areolate. All surfaces of the head densely areolate with costulae, but sculpture becomes weaker ventrally. Mesosoma uniformly densely areolate, but lateral face of the pronotum and dorsum of mesosoma with strong rugae; lateral face of the propodeum, meso- and metapleurae with areolae arranged into rows by costulae. Femora areolate and opaque. Petiole shining through weak areolate sculpture on nearly all surfaces, but base and dorsolateral portions of the petiolar node more densely areolate and appearing dull. Postpetiole weakly shining though shallow areolate sculpture. Gaster smooth and shining, but with moderately strong spectral iridescence on the first gastral tergite and sternite.

Setae: antennal scapes and funiculi with short, decumbent pilosity. Dorsum of the head, pronotum, waist segments, and gaster with abundant, erect, blunt-tipped setae, the longest of which are about the length of the compound eye. The head dorsum bears ~68, dorsum of mesosoma ~66, petiole 12, postpetiole ~34, and first gastral tergite ~110 setae. Short, sparse pubescence present over the entire body, but difficult to detect against the dense ground sculpture.

Color: mandibles, head capsule, mesosoma, peduncle, anterior face of petiolar node, lateral face of postpetiole, distal four tarsi, and base of gaster red; remainder of the body dark brown.

**Gyne measurements & indices (*n* = 2):** SL = 0.679–0.685 (0.682); FRS = 0.225–0.239 (0.232); CW = 0.789–0.812 (0.801); CWb = 0.731–0.751 (0.741); PoOC = 0.291–0.309 (0.300); CL = 0.774–0.776 (0.775); EL = 0.198–0.199 (0.199); EW = 0.147–0.152 (0.150); MD = 0.179–0.190 (0.185); WL = 1.263–1.280 (1.272); SPST = 0.338–0.386 (0.362); MPST = 0.336–0.339 (0.338); PEL = 0.397–0.421 (0.409); NOL = 0.197–0.203 (0.200); NOH = 0.171–0.183 (0.177); PEH = 0.317–0.360 (0.339); PPL = 0.200–0.233 (0.217); PPH = 0.336–0.349 (0.343); PW = 0.793–0.828 (0.811); SBPA = 0.408–0.442 (0.425); SPTI = 0.374–0.428 (0.401); PEW = 0.204–0.218 (0.211); PNW = 0.276–0.300 (0.288); PPW = 0.529–0.552 (0.541); HFL = 0.772–0.780 (0.776); HFWmax = 0.159–0.160 (0.160); HFWmin = 0.063–0.070 (0.067); CS = 1.118–1.139 (1.129); ES = 0.273–0.274 (0.273); SI = 90–94 (92); OI = 24–25 (24); CI = 94–97 (96); WLI = 168–175 (172); SBI = 56–59 (57); PSI = 26–31 (28); PWI = 253–259 (256); PLI = 181–199 (190); NI = 108–119 (113); PNWI = 135–138 (136); NLI = 47–51 (49); FI = 227–254 (241).

**Gyne description:** In full-face view, head subquadrate, about as long as broad (CI 94–97). Mandibles densely, finely striate, but shining and armed with five teeth: the apical-most well developed, followed by a less developed preapical tooth and three equally developed smaller teeth. Anterior clypeal margin flat medially. Antennal scapes very long: when fully retracted, surpassing the posterior margin of the head capsule by about two times the maximum width of the scape (SI 90–94). Antennae 12-segmented; antennal club composed of three segments, with the apical-most segment slightly longer than the preceding two in combination. Torular laminae not strongly developed: narrower than the distance that separates them. Frontal carinae moderately short, extending past the antennal toruli by about two times the maximum width of the antennal scape. Compound eyes moderately protruding past the lateral margins of the head capsule. Lateral margin of head evenly convex, converging from below the compound eyes to the mandibular insertions. Posterior head margin flat, rounding evenly into the lateral margins.

In profile view, compound eyes ovular and moderately large (OI 24–25), with 16 ommatidia in longest row. Mesoscutum rounded evenly anteriorly, covering the dorsal surface of the pronotum, and weakly convex dorsally. Mesoscutellum slightly depressed below the level of the mesoscutum. Posterior margin of metanotum extending slightly past the posterior margin of the mesoscutum. Propodeal spiracle well developed, directed posterolaterally, and separated from the propodeal declivity by about two and a half spiracle diameters. Propodeal spines stout, well developed, and moderately long (PSI 26–31), about as long as the propodeal declivity, tapering evenly from the base, directed posteriorly, straight, and blunt. Propodeal declivity weakly concave, forming a rounded ~100° angle with the base of the propodeal spines. Propodeal lobes rounded and very weakly developed. Metapleural gland bulla small, extending from the metacoxal insertion halfway to the propodeal spiracle. Petiole moderately long (PLI 181–199), with weakly developed tubercles anterodorsally. Subpetiolar process in the form of a small, acute tooth; ventral margin of petiole bulging slightly posterior to it. Petiolar peduncle moderately long: petiolar node comprising about a third of the petiolar dorsum. Petiolar node strongly squamiform, but not as extreme as in the worker: transition between peduncle and node abrupt: marked by a rounded angle of ~110°; anterior face of the node is strongly concave but does not overhang the peduncle; anterior face forming a ~90° angle with the very short dorsal face, which rounds evenly into the convex posterior face. Postpetiole evenly rounded anterodorsally, bulging slightly before it transitions into the flattened dorsal face; ventral surface weakly lobed.

In dorsal view, mesoscutum covering pronotum anteriorly, but humeri visible laterally as rounded sclerites. Propodeal spines weakly diverging apically, their apices separated from each other by about one and a half times their length. Petiolar peduncle with spiracles strongly protruding past the lateral margins, but not noticeably constricted anterior to them. When viewed posterodorsally, the petiolar node spade-shaped but not as transversely broad as in the worker; dorsal margin weakly emarginated. Postpetiole very broad (PWI 253–259), anteroposteriorly compressed, and campaniform, articulating with most of the anterior margin of the gaster, leaving small, angulate margins on each side exposed. Anterior margin of postpetiole broadly convex and evenly rounding into the lateral margins, which diverge to the angulate posterior corners; posterior margin broadly concave. Metafemur weakly to moderately incrassate (FI 227–254).

Sculpture: median clypeal carina present, extending from the anterior margin nearly to frontal triangle, and flanked by three weaker carinae on each side. Lateral clypeal lobes with additional weaker carinae; ground sculpture areolate. Antennal scapes densely areolate. All surfaces of the head densely areolate and costulate, but sculpture becomes weaker ventrally. Nearly all surfaces of the mesosoma densely areolate, with costulae overlying the ground sculpture, but mesopleurae, lateral face of the propodeum, and propodeal declivity lack costulae. Femora appearing dull and shallowly areolate. Petiole shining through weak areolate sculpture on most surfaces, but the posterior face and base of the petiolar node dull, with denser areolae. Postpetiole sculpture matching that of the posterior face of the petiolar node. Gaster with shallow areolate sculpture on the basal third of the first gastral tergite, which is otherwise smooth and shining, with weak to moderate spectral iridescence; first gastral sternite smooth and shining, with moderate spectral iridescence. First gastral sternite smooth and shining with weak spectral iridescence.

Setae: antennal scapes and funiculi with short, decumbent pilosity. Dorsum of the head, pronotum, waist segments, and gaster with abundant, erect, blunt-tipped setae, the longest of which are about half the length of the compound eye. Short, sparse pubescence present over the entire body, but difficult to detect against the dense ground sculpture.

Color: mandibles, head capsule, mesosoma, peduncle, anterior face of petiolar node, lateral face of postpetiole, distal four tarsi, and base of gaster red; mesopleurae and ventral half of propodeum dark brown, and mesoscutum with dark brown patches anteromedially and posterolaterally.

**Male measurements & indices (*n* = 2):** SL = 0.194–0.212 (0.203); FRS = 0.103–0.160 (0.132); CW = 0.585–0.601 (0.593); CWb = 0.508–0.524 (0.516); PoOC = 0.225–0.245 (0.235); CL = 0.512–0.521 (0.517); EL = 0.214–0.237 (0.226); EW = 0.172–0.172 (0.172); MD = 0.062–0.069 (0.066); WL = 0.916–0.938 (0.927); SPST = n/a; MPST = 0.216–0.227 (0.222); PEL = 0.255–0.260 (0.258); NOL = 0.156–0.183 (0.170); NOH = 0.041–0.050 (0.046); PEH = 0.131–0.134 (0.133); PPL = 0.147–0.151 (0.149); PPH = 0.172–0.177 (0.175); PW = 0.446–0.458 (0.452); SBPA = n/a; SPTI = n/a; PEW = 0.122–0.126 (0.124); PNW = 0.129–0.146 (0.138); PPW = 0.305–0.322 (0.314); HFL = 0.766–0.830 (0.798); HFWmax = 0.070–0.082 (0.076); HFWmin = 0.039–0.042 (0.041); CS = 0.769–0.780 (0.774); ES = 0.300–0.323 (0.312); SI = 38–40 (39); OI = 38–42 (40); CI = 98–102 (100); WLI = 179–180 (180); SBI = n/a; PSI = n/a; PWI = 242–264 (253); PLI = 172–173 (173); NI = 312–446 (379); PNWI = 102–120 (111); NLI = 60–72 (66); FI = 167–210 (188).

**Male description:** In full-face view, head subovate, about as long as broad (CI 98–102). Mandibles smooth and shining and armed with two teeth: the apical-most well developed and acute, followed by a smaller preapical tooth and a series of weak crenulae. Anterior clypeal margin entire and flat. Antennal scapes short: when fully retracted, failing to reach the posterior margin of the head capsule by about their length (SI 38–40). Antennae 13-segmented; antennal club composed of four segments, with the apical-most segment longer than the preceding two in combination. Frontal carinae short, extending past the antennal toruli by two times the maximum width of the antennal scape. Compound eyes strongly protruding past the lateral margins of the head capsule. Posterior and lateral head margins indistinct, forming an even convexity.

In profile view, compound eyes ovular and large (OI 38–42), with 20 ommatidia in the longest row. Mesoscutum slightly bulging anteriorly, but not completely covering the dorsal surface of the pronotum, evenly convex from where it joins the petiolar neck to the mesoscutellum. Mesoscutellum depressed slightly below the level of the mesoscutum; posterior margin overhanging the posterior margin of the metanotum. Propodeal spiracle well developed, directed posterolaterally, and separated from the propodeal declivity by about three spiracle diameters. Propodeal spines absent. Propodeal lobes rounded and weakly developed. Metapleural gland bulla small, extending a third of the way between the insertion of the metacoxa and the propodeal spiracle. Petiole moderately long (PLI 172–173), with weakly developed tubercles anterodorsally. Subpetiolar process a weakly developed, blunt triangular tooth. Petiolar peduncle very long: comprising two thirds of the total length of the petiole. Petiolar node low and rounded, the convergence of the anterior and dorsal faces marked by a rounded angle. Postpetiole evenly rounded anterodorsally, flattened dorsally, and with a lobed, concave ventral surface.

In dorsal view, mesoscutum not completely covering pronotum anteriorly: juncture of the pronotal neck and the humeri visible. Petiolar peduncle with spiracles strongly protruding past the lateral margins, the peduncle broadened where they arise. Petiolar node wider than the peduncle; petiole narrowing posterior to the node, before widening again to the caudal cylinder, which is about the same width as the node. Postpetiole very broad (PWI 242–264) and campaniform, articulating with most of the anterior margin of the gaster. Anterior margin of postpetiole convex, the anterior corners evenly rounding into the lateral margins, which evenly diverge to the angulate posterior corners; posterior margin of postpetiole flat. Metafemur not incrassate (FI 167–210).

Sculpture: clypeal carinae indistinguishable from the areolate ground sculpture. Antennal scapes smooth and shining. Head areolate, with weak costulae. Pronotum areolate, but with smooth patches dorsolaterally. Katepisternum mostly smooth and shining, but with areolate sculpture where it joins the anepisternum. Anepisternum with the anterior half smooth and shining; posterior half is areolate. Ventral half of the metapleuron smooth and shining; dorsal half areolate. Propodeum areolate on all surfaces. Mesoscutum with two broad strips of smooth and shining sculpture anteriorly, which flank a patch of finely areolate sculpture medially; the two strips converge posteriorly and extend to the border with the mesoscutellum; two broad patches of smooth and shining sculpture flank the Mayrian furrows; otherwise mesoscutum is finely areolate. Mesoscutellum densely areolate and costulate. Femora smooth and shining, with traces weak areolate sculpture distally. Petiole with shallow areolate sculpture at the base of the petiolar node, otherwise smooth and shining. Dorsal surface of postpetiole shining, with traces of areolate sculpture posteromedially. Gaster smooth and shining, without traces of spectral iridescence.

Setae: antennal scapes and funiculi with short, decumbent pilosity. Dorsum of the head, pronotum, waist segments, and gaster with abundant, erect, blunt-tipped setae, the longest of which are about a third of the length of the compound eye. Short, sparse pubescence present over the entire body, but difficult to detect against the dense ground sculpture.

Color: antennal funiculi testaceous yellow. Antennal scapes, mandibles, and legs light yellow, nearly white. Head capsule, Mayrian furrows, mesoscutellum, and propodeum dark brown. Remainder of body testaceous.

**Etymology:** Morphological, from the Latin ‘rutabulum’ (= ladle) + ‘-fer’ (= bearing), in reference to the shape of the petiole.

**Comments:** *Temnothorax rutabulafer* sp, nov., like *T. pastinifer*, is broadly distributed among the Bahamas and Cuba, occurring primarily in littoral habitats, but apparently found in lowland rainforest as well. Nest collections from North Andros island were made from small dead twigs in leaf litter, similar to the majority of species from the *pulchellus* and *pastinifer* groups. This species has historically been confused with *T. pastinifer*; for more information see the taxonomic notes under that species.

***Temnothorax schwarzi* (Mann, 1920)**

**Figure 126:**
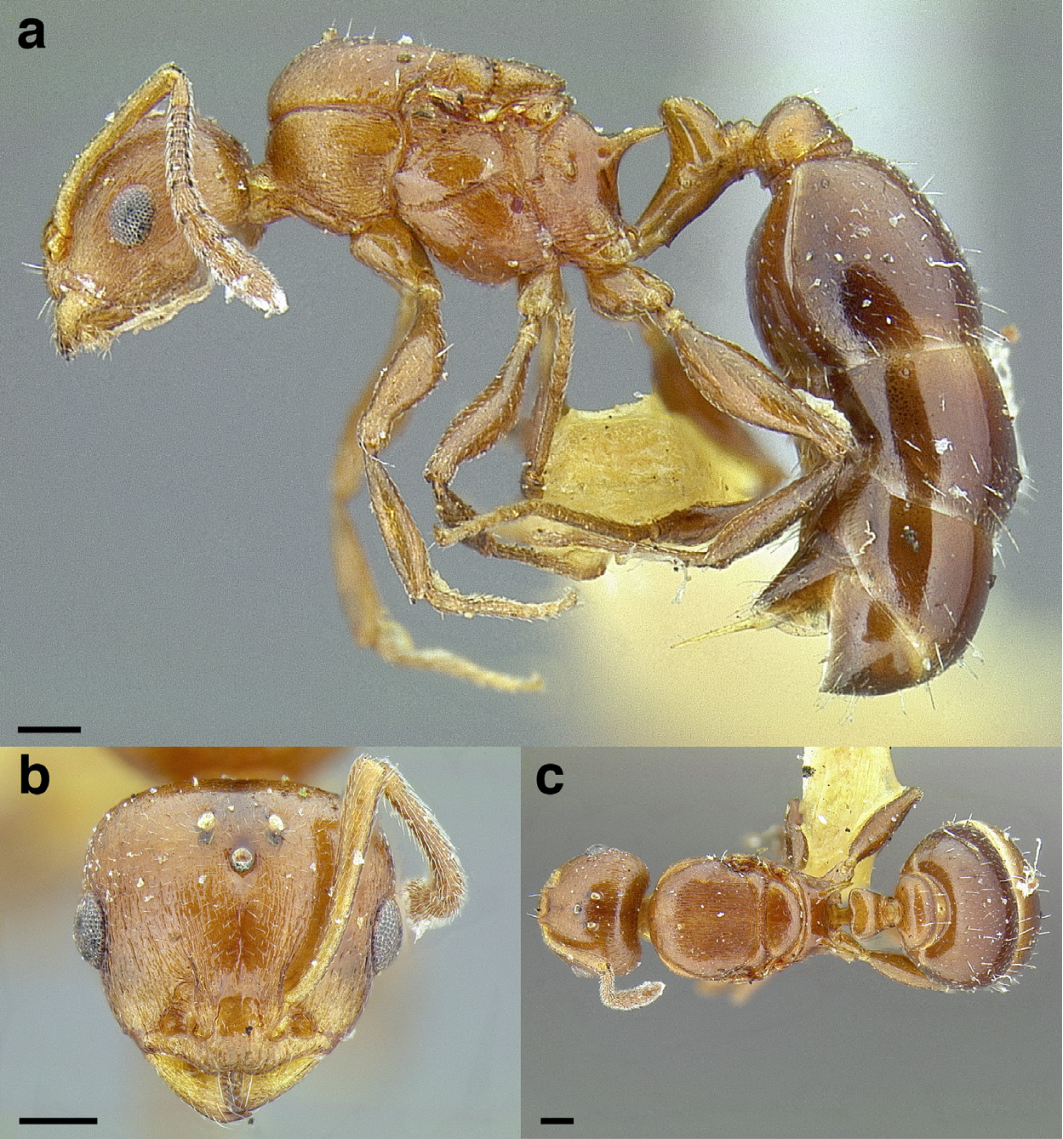
*Temnothorax schwarzi* holotype gyne (USNMENT00532012). (A) Profile view. (B) Full-face view. (C) Dorsal view. Scale bars 0.2 mm.

Distribution: Fig. 121E; gyne: Fig. 126.

*Macromischa (Macromischa) schwarzi* Mann, 1920: 415. Holotype gyne. Cayamas, Cuba.

*Leptothorax schwarzi* (Mann): Baroni Urbani, 1978: 497. Worker described. First combination in *Leptothorax*.

*Temnothorax schwarzi* (Mann): Bolton, 2003: 272. First combination in *Temnothorax*.

**Type material examined:** *Holotype gyne:* CUBA: Artemisia: Cayamas, [no collection date], E.A. Schwarz, USNM Type No. 56181, 1 dealate gyne (USNMENT00532012) [USNM].

**Geographic range:** Cuba (Artemisia) (Fig. 121E).

**Worker Diagnosis:** Mann described this species from a single dealate gyne, collected in the present-day Cuban province of Mayabeque. Subsequently, Baroni Urbani described workers of *Temnothorax schwarzi* from several localities in the adjacent Pinar del Rio province in his 1978 revision, based on details of the petiolar node and microsculpture. However, workers and gynes of this species have not yet been recorded from a single nest collection, so there remains some doubt as to how the holotype and Baroni Urbani’s *T. schwarzi* are related. For the purposes of diagnosis, I refer to Baroni Urbani’s concept of the workers: among the species closely related to *Temnothorax pastinifer*, only the workers of *T. schwarzi* have the combination of smooth integument on the dorsum of the head and a strongly squamiform petiolar node than overhangs the petiolar peduncle anteriorly. This species may be confused with *T. rutabulafer* sp. nov., but the latter has a flat to weakly emarginate anterior margin of the clypeus, and densely sculptured head and mesosoma.

**Worker:** See Baroni Urbani (1978).

**Gyne measurements & indices (*n* = 1):** SL = 0.680; FRS = 0.246; CW = 0.870; CWb = 0.814; PoOC = 0.297; CL = 0.780; EL = 0.215; EW = 0.161; MD = 0.183; WL = 1.265; SPST = 0.348; MPST = 0.329; PEL = 0.446; NOL = 0.196; NOH = 0.217; PEH = 0.345; PPL = 0.199; PPH = 0.320; PW = 0.782; SBPA = 0.379; SPTI = 0.456; PEW = 0.200; PNW = 0.328; PPW = 0.509; HFL = 0.731; HFWmax = 0.180; HFWmin = 0.070; CS = 1.204; ES = 0.296; SI = 84; OI = 25; CI = 104; WLI = 155; SBI = 47; PSI = 28; PWI = 255; PLI = 224; NI = 90; PNWI = 164; NLI = 44; FI = 257.

**Gyne description:** In full-face view, head subquadrate, slightly broader than long (CI 104). Mandibles densely striate but shining and armed with five teeth: the apical-most well developed, followed by a less developed preapical tooth and three equally developed smaller teeth. Anterior clypeal margin emarginated medially. Antennal scapes very long: when fully retracted, surpassing the posterior margin of the head capsule by about one and a half times the maximum width of the scape (SI 84). Antennae 12-segmented; antennal club composed of three segments, with the apical-most segment longer than the preceding two in combination. Frontal carinae moderately long, extending past the antennal toruli by about two times the maximum width of the antennal scape. Compound eyes moderately protruding past the lateral margins of the head capsule. Lateral margin of head evenly convex, converging from below the compound eyes to the mandibular insertions. Posterior head margin flat, rounding evenly into the lateral margins.

In profile view, compound eyes ovular and moderately large (OI 25), with 17 ommatidia in longest row. Mesoscutum rounded evenly anteriorly, covering the dorsal surface of the pronotum, and flat dorsally. Mesoscutellum slightly higher than the level of the mesoscutum. Posterior margin of metanotum extending slightly past the posterior margin of the mesoscutum. Propodeal spiracle well developed, directed posterolaterally, and separated from the propodeal declivity by about four spiracle diameters. Propodeal spines stout, well developed, and moderately long (PSI 28), about as long as the propodeal declivity, tapering evenly from the base, directed posteriorly, weakly downcurved, and acute. Propodeal declivity weakly concave, forming a rounded ~110° angle with the base of the propodeal spines. Propodeal lobes rounded and very weakly developed, but with a slightly angulate dorsal flange. Metapleural gland bulla small, extending from the metacoxal insertion halfway to the propodeal spiracle. Petiole long (PLI 224), without tubercles anterodorsally. Subpetiolar process in the form of a small, very acute tooth, ventral margin of the petiole bulging very weakly posterior to the process. Petiolar peduncle very long: petiolar node comprising about a third of the petiolar dorsum. Petiolar node squamiform: transition between peduncle and node abrupt: marked by a rounded angle of ~110°; anterior face of the node is straight and does not overtop the peduncle; anterior face forming a ~90° angle with the very short dorsal face, which rounds evenly into the convex posterior face. Postpetiole with a flat anterior face, which rounds evenly into the flat dorsal face; ventral surface concave.

In dorsal view, mesoscutum covering pronotum anteriorly, but humeri visible laterally as rounded sclerites. Propodeal spines broadly approximated basally and evenly diverging along their entire length, their apices separated from each other by about two times their length. Petiolar peduncle with spiracles strongly protruding past the lateral margins. Petiolar node, when viewed posterodorsally, roughly trapezoidal: widest apically, tapering to the caudal cylinder, and with the flat dorsal margin weakly emarginated. Broadest part of the petiolar node much wider than the peduncle and the caudal cylinder. Postpetiole very broad (PWI 255), strongly anteroposteriorly compressed, and subquadrate. Postpetiole articulates with most of the anterior margin of the gaster, but leaves small, angulate margins on each side exposed. Anterior margin of postpetiole flat, with corners marked by rounded angles as it transitions to the lateral margins, which are parallel to the angulate posterior corners; posterior margin broadly concave. Metafemur moderately incrassate (FI 257).

Sculpture: median clypeal carina present, extending from the anterior margin to the level of the antennal toruli and flanked on either side by additional less distinct carinae; lateral margins of median clypeal lobe with two carinae that are as strong as the medial carina. Lateral clypeal lobes with additional weaker carinae; ground sculpture weakly areolate and shining. Antennal scapes weakly areolate. Cephalic dorsum weakly costulate and shining, with a median sulcus extending from the frontal triangle nearly to the median ocellus. Lateral surfaces of head with stronger costulate sculpture, becoming rugose between the compound eye and the mandibular insertion. Ventral surface of head with weakly costulate. Pronotal neck areolate. Lateral face of the pronotum shining, with very weak areolate-costulate ground sculpture. Anterior third of katepisternum smooth and shining, transitioning into weak areolate-costulate sculpture posteriorly. Anepisternum smooth and shining. Mesopleuron costulate. Propodeum smooth and shining. Mesoscutum predominantly costulate, surrounding a smooth and shining anteromedial patch; smooth and shining patches laterally. Mesoscutellum smooth and shining medially, surrounded by weak areolae. Femora smooth and shining, with traces of weak areolate sculpture distally. Petiole with weak areolate sculpture ventrally, otherwise smooth and shining. Postpetiole shining through weak areolate sculpture anteriorly, with stronger areolate sculpture laterally and dorsally. Gaster predominantly smooth and shining, but first gastral tergite and sternite weakly coriarious.

Setae: antennal scapes and funiculi with short, decumbent pilosity, with several longer setae apically. Dorsum of the head, pronotum, waist segments, and gaster with very sparse, erect, blunt-tipped setae, the longest of which are about half the width of the compound eye. The head bears ~6, mesosoma ~10 restricted to the mesoscutum, petiole 0, postpetiole ~4, and first gastral tergite ~32 setae. Short, sparse pubescence present over the entire body.

Color: nearly uniformly ferruginous red, with slightly darker longitudinal strips on the mesoscutum. Gaster slightly darker than the rest of the body.

**Male:** Unknown.

**Etymology:** Patronym, after the collector of the holotype gyne, E.A. Schwarz.

**Comments:** *Temnothorax schwarzi* is known only from low lying coastal areas in western Cuba. The holotype gyne was collected in Cayería las Cayamas, a small peninsula in the Gulf of Batabano. The workers of *T. schwarzi* described by Baroni Urbani were collected from the Guanahacabibes peninsula and two other localities in the province of Pinar del Rio. This species, like other members of the *pastinifer* group, appears to be tolerant of littoral habitats. While this species is undoubtedly closely related to *T. pastinifer*, the association of workers and gynes has not yet been definitively made. Although I tend to agree with Baroni Urbani (1978) opinion that the specimens mentioned above are conspecific, an inventory of Cuban species has not been made for several decades. I suspect that there may be a trove of species yet to be described from the island, especially in museum collections, and in the leaf litter of any remnant low elevation forest.

### *pergandei* group overview

**Figure 127:**
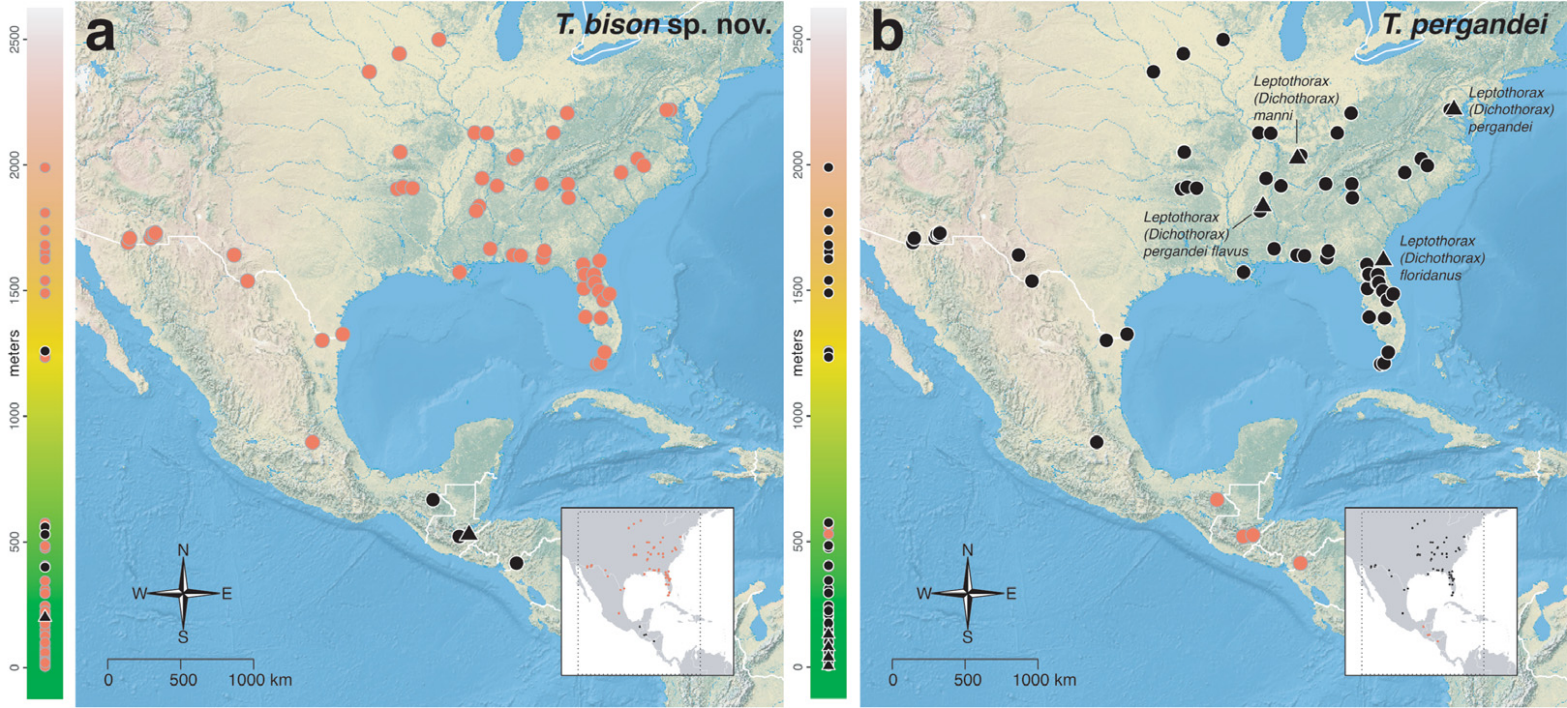
Geographical and elevational distribution of the *pergandei* group. (A) *Temnothorax bison* sp. nov. (B) *T. pergandei*. The colored scale to the left of each map represents elevation in meters. Points in black represent the species named in each subfigure, while points in red represent other members of the species group. Type localities are represented by triangles; if present, types of synonyms are named; non-type localities are represented by circles. Bounding box in inset map shows location of main map.

This group is composed of two species, *Temnothorax pergandei* and *T. bison* sp. nov., which are found at all elevations from eastern North America to Central America (Fig. 127). For much of its history, the nominal species of this group was placed in its own subgenus (Dichothorax). All nest collections have been made from soil or dead vegetation on the ground; this appears to be a primarily terrestrial group. United by a strongly depressed propodeum, long setae on all surfaces of the body, and reduced or absent propodeal spines, the members of the *pergandei* group are unlikely to be confused with any co-occurring *Temnothorax* species (although they appear to be morphologically convergent with some species of the Mediterranean region of Europe).

***Temnothorax bison* sp. nov.**

**Figure 128:**
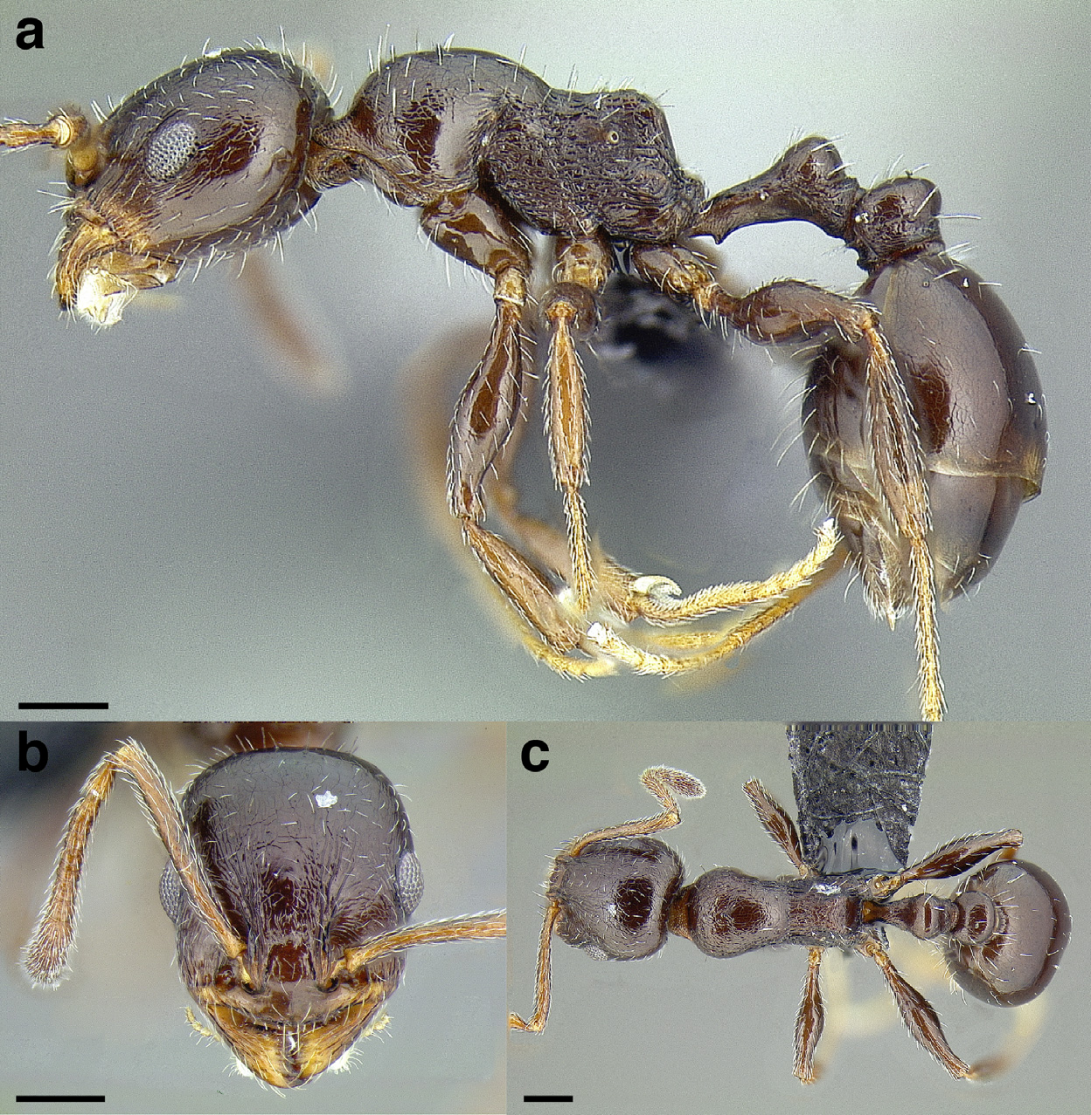
*Temnothorax bison* sp. nov. holotype worker (CASENT0636947). (A) Profile view. (B) Full-face view. (C) Dorsal view. Scale bars 0.2 mm.

Distribution: Fig. 127A; worker: Fig. 128.

*Temnothorax* mmp05 Prebus, 2017: 8. Phylogeny.

**Type material examined:** *Holotype worker:* GUATEMALA: Zacapa: Zacapa, finca near University Landivar campus, 14.99336°N 89.54607°W, 200 m, 18–21 June 2015, Z. Falin#GUATIF15-188, dry thorn scrub forest, flight intercept trap (CASENT0636947) [CASC].

*Paratype worker:* same data as holotype, 1 worker (CASENT0636946) [UCDC].

**Non-type material examined:** GUATEMALA: El Progreso: 3.7 km SW Morazán, 14.907267°N 90.1548°W, 530 m, 7 July 2007, J. Longino#6021-s, scrubby xeric vegetation, on ground, 1 worker (JTLC000010341) [JTLC] 1 worker (JTLC000010340) [UVGC]; 5 km W El Rancho, 14.916667°N 90.066667°W, 400 m, 17 November 2003, A.L. Wild #AW2082, tropical dry forest, 1 worker (CASENT0915984) [USNM].

MEXICO: Chiapas: Yaxoquintela, 16.966667°N 91.783333°W, 560 m, 28 August 1978, John E. Rawlins, 1 worker (MCZ-ENT00541468) [MCZC].

NICARAGUA: Madriz: 8 km S Somoto, 13.40699°N 86.59106°W ±50 m, 1,260 m, 23 April 2011, J. Longino#JTL7396, dry oak woodland, at bait, 1 worker (CASENT0619615) [UCDC] 1 worker (CASENT0619345) [LACM].

**Geographic range:** Low-to-mid elevations, southern Mexico to Nicaragua (Fig. 127A).

**Worker diagnosis:** *Temnothorax bison* sp. nov. can be separated from all other species in the *salvini* clade by the following character combination: promesonotum evenly convex; metanotal groove deeply impressed and propodeum depressed below the level of the promesonotum; propodeal spines absent: dorsal face of propodeum transitioning evenly in to the propodeal declivity; in profile view, postpetiole bulging anterodorsally and flat posterodorsally; in dorsal view, petiolar node 1.3 to 1.6 times as broad as caudal cylinder; integument predominantly dark brown; setae on head, mesosoma, legs, waist segments and gaster erect, moderately long, abundant and tapering.

**Similar species:** this striking species is difficult to confuse with any other species in *Temnothorax*, except the closely related *T. pergandei*. The absence of propodeal teeth, the structure of the postpetiole, which is evenly convex dorsally in *T. pergandei*, and the relative width of the petiolar node in dorsal view (1.1 to 1.3 times as broad as the caudal cylinder in *T. pergandei*) will separate the two species.

**Worker measurements & indices (*n* = 8):** SL = 0.535–0.675 (0.596); FRS = 0.171–0.244 (0.214); CW = 0.602–0.748 (0.678); CWb = 0.548–0.686 (0.627); PoOC = 0.229–0.282 (0.253); CL = 0.651–0.783 (0.710); EL = 0.158–0.207 (0.184); EW = 0.114–0.136 (0.124); MD = 0.143–0.178 (0.158); WL = 0.816–0.990 (0.901); SPST = 0.119–0.158 (0.141); MPST = 0.242–0.336 (0.300); PEL = 0.311–0.413 (0.366); NOL = 0.196–0.227 (0.211); NOH = 0.094–0.129 (0.112); PEH = 0.175–0.239 (0.209); PPL = 0.156–0.193 (0.173); PPH = 0.217–0.278 (0.248); PW = 0.395–0.513 (0.450); SBPA = n/a; SPTI = 0.163–0.211 (0.186); PEW = 0.141–0.176 (0.161); PNW = 0.197–0.282 (0.238); PPW = 0.234–0.314 (0.270); HFL = 0.555–0.732 (0.648); HFWmax = 0.126–0.157 (0.141); HFWmin = 0.046–0.060 (0.052); CS = 0.874–1.069 (0.982); ES = 0.215–0.273 (0.246); SI = 87–99 (95); OI = 23–27 (25); CI = 84–92 (88); WLI = 141–150 (144); SBI = n/a; PSI = 14–18 (16); PWI = 149–184 (167); PLI = 194–232 (212); NI = 167–209 (189); PNWI = 125–164 (147); NLI = 53–63 (58); FI = 254–292 (275).

**Worker description:** In full-face view, head subquadrate, longer than broad (CI 84–92). Mandibles densely striate but shining and armed with five teeth: the apical-most well developed and acute, followed by a less developed preapical tooth and three equally developed smaller teeth. Anterior clypeal margin weakly convex medially. Antennal scapes very long: when fully retracted, surpassing the posterior margin of the head capsule by about two times the maximum width of the scape (SI 87–99). Antennae 12-segmented; antennal club of composed of three segments, with the apical-most segment as long as the preceding two in combination. Frontal carinae short, extending past the antennal toruli by about one and a half times the maximum width of the antennal scape. Compound eyes moderately protruding past the lateral margins of the head capsule. Lateral margin of head weakly convex, forming a continuous arc from the mandibular insertions to the posterior margin of the head. Posterior head margin weakly convex, rounding evenly into the lateral margins.

In profile view, compound eyes elongate ovular and moderately large (OI 23–27), with 11 ommatidia in longest row. Pronotal declivity indistinct, neck and anterior face of pronotum forming a ~120° angle. Mesosoma with promesonotum forming an even convexity; the propodeum depressed below the level of the promesonotum and dorsally concave. Promesonotal suture extending from the posterior margin of the procoxal insertion to the mesothoracic spiracle, which is moderately well developed, then continuing to the dorsal surface as a very weak sulcus. Metanotal groove visible as a disruption of the sculpture laterally from where it arises between the mid- and hind coxae to where it ends in the poorly developed metathoracic spiracle, which is nearly indistinguishable against the ground sculpture; continuing dorsally as a distinct sulcus. Propodeal spiracle well developed, directed posterolaterally, and separated from the propodeal declivity by about three spiracle diameters. Propodeal spines absent, but with a cuticular flange extending from the propodeal dorsum to the propodeal lobes in their place. Propodeal declivity flat, forming a rounded ~100° angle with propodeal dorsum. Propodeal lobes rounded and weakly developed. Metapleural gland bulla moderately large, extending from the metacoxal insertion two thirds of the way to the propodeal spiracle. Petiole long (PLI 194–232), without tubercles anterodorsally. Subpetiolar process in the form of a small, triangular, blunt tooth, which continues as a low carina to the caudal cylinder; ventral margin of petiole posterior to the subpetiolar process strongly concave. Petiolar peduncle moderately long: comprising about half the length of the petiole. Petiolar node cuneiform but rounded dorsally: transition between peduncle and node marked by a rounded angle of ~150°, resulting in a weakly concave anterior node face; anterior face forming a broadly rounded ~60° angle with the posterior face; caudal cylinder short, about half the maximum width of the antennal scape. Postpetiole weakly convex anteriorly, rounding evenly into the dorsal face, which bulges strongly before flattening posteriorly; lobed ventrally.

In dorsal view, humeri weakly developed: evenly rounded and wider than the rest of the mesosoma, but not distinct from it; mesothoracic spiracles weakly protruding past the lateral margins of the mesosoma, visible as slight angles where the pronotum meets the mesonotum. Promesonotal suture visible as a weak sulcus. Metanotal strongly impressed, distinctly dividing the promesonotum from the propodeum. Propodeal spines absent, but flanges diverging at the apex of the propodeal dorsum. Petiolar peduncle with spiracles weakly protruding past the lateral margins, but not noticeably constricted anterior to them. Petiolar node, when viewed posterodorsally, trapezoidal and apically broadened, the apex weakly convex; caudal cylinder narrower than the apex of the node. Postpetiole narrow (PWI 149–184) and trapezoidal: widest anteriorly. Anterior margin of the postpetiole weakly convex and meeting the lateral margins at a rounded angle; lateral margins converge moderately to the angulate posterior corners; posterior margin broadly concave. Metafemur moderately incrassate (FI 254–292).

Sculpture: median clypeal carina present, extending posteriorly to the level of the anterior margins of the antennal insertions. Lateral clypeal lobes with additional, weaker carinae; ground sculpture smooth and shining. Antennal scapes shining through weak coriarious ground sculpture. Cephalic dorsum predominantly coriarious, with costulae flanking the frontal carinae; concentric costulae surrounding the antennal insertions, extending from the apices of the frontal carinae, to the anterior margins of the lateral clypeal lobes. Lateral surfaces of head with weak coriarious sculpture posterior to the compound eye, denser coriarious sculpture surrounding the compound eye, and stronger costulae between the compound eye and the mandibular insertion. Ventral surface of head smooth and shining. Pronotal neck areolate. Lateral surface of the pronotum areolate-coriarious on the anterior third, otherwise smooth and shining. Lateral face of the propodeum, meso- and metapleurae predominantly costate, over areolate ground sculpture; area around propodeal spiracle finely areolate. Propodeal declivity smooth and shining. Dorsal surface of promesonotum coriarious. Dorsum of propodeum strongly rugose. Femora smooth and shining. Petiole smooth and shining ventrally; node weakly coriarious; dorsum of peduncle finely areolate. Postpetiole weakly coriarious. First gastral tergite weakly coriarious; first gastral sternite smooth and shining. Spectral iridescence absent.

Setae: antennal scapes and funiculi with moderately long, decumbent pilosity. Dorsum of the head, pronotum, waist segments, and gaster with moderately abundant, erect, tapering setae, the longest of which are about the length of the compound eye. The head bears ~40, mesosoma ~38, petiole ~14, postpetiole ~20, and first gastral tergite ~60 setae. Pubescence is long, coarse, difficult to distinguish from the setae, and present over the entire body except for the ventral surface of the petiole.

Color: predominantly dark brown, with mandibles, antennae, tibiae and tarsi testaceous.

**Gyne:** Unknown.

**Male:** Unknown.

**Etymology:** Morphological, in reference to the humpbacked appearance of the worker.

**Comments:** Despite multiple collections, very little is known about the biology of *Temnothorax bison* sp. nov. It appears to inhabit low-to-mid elevation tropical dry forest or xeric scrub habitats and forage terrestrially, much like its close relative, *T. pergandei*.

***Temnothorax pergandei* (Emery, 1895)**

**Figure 129:**
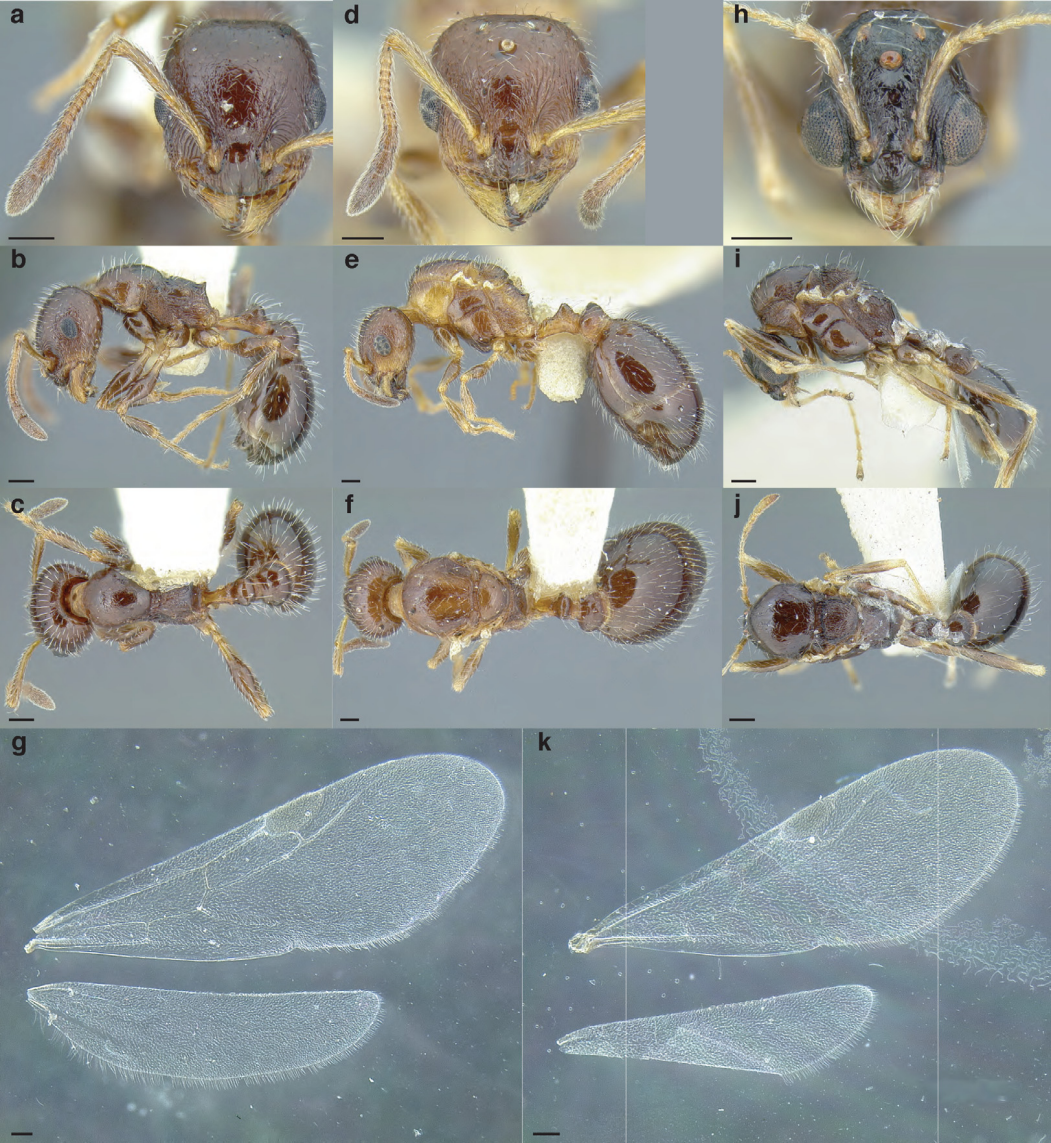
*Temnothorax pergandei*. (A–C) Worker (CASENT0758291). (A) Full-face view. (B) Profile view. (C) Dorsal view. (D–G) Gyne (CASENT0758229). (D) Full-face view. (E) Profile view. (F) Dorsal view. (G) Wings. (H–K) Male (CASENT0758229). (H) Full-face view. (I) Profile view. (J) Dorsal view. (K) Wings. Scale bars 0.2 mm.

**Figure 130:**
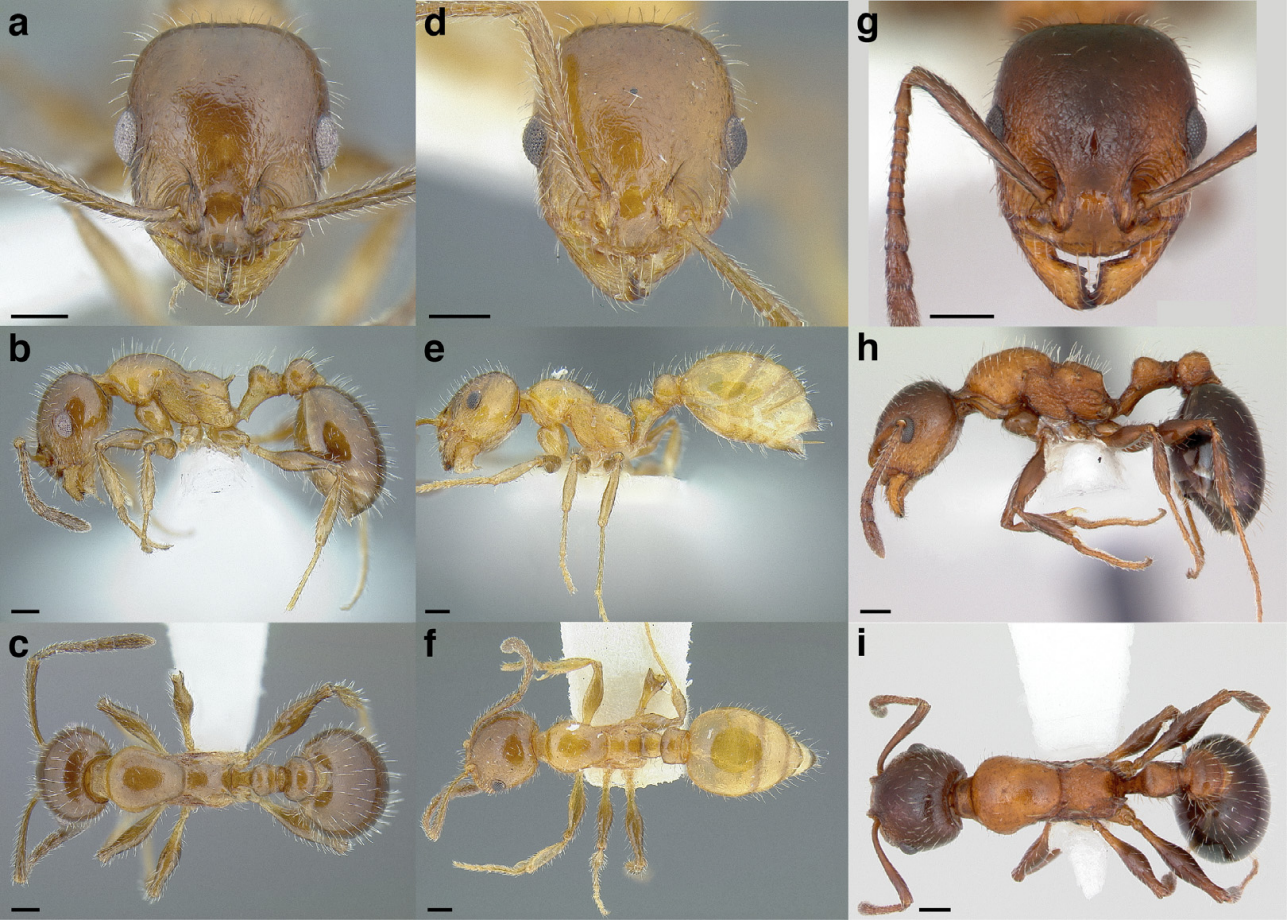
Comparison of *Temnothorax pergandei* worker morphology. (A–C) Missouri, U.S.A. (MCZENT00583596). (A) Full-face view. (B) Profile view. (C) Dorsal view. (D–F) Mississippi, U.S.A. (USNMENT00921850). (D) Full-face view. (E) Profile view. (F) Dorsal view. (G–I) Arizona, U.S.A. (CASENT0172989). (G) Full-face view. (H) Profile view. (I) Dorsal view. Scale bars 0.2 mm.

**Figure 131:**
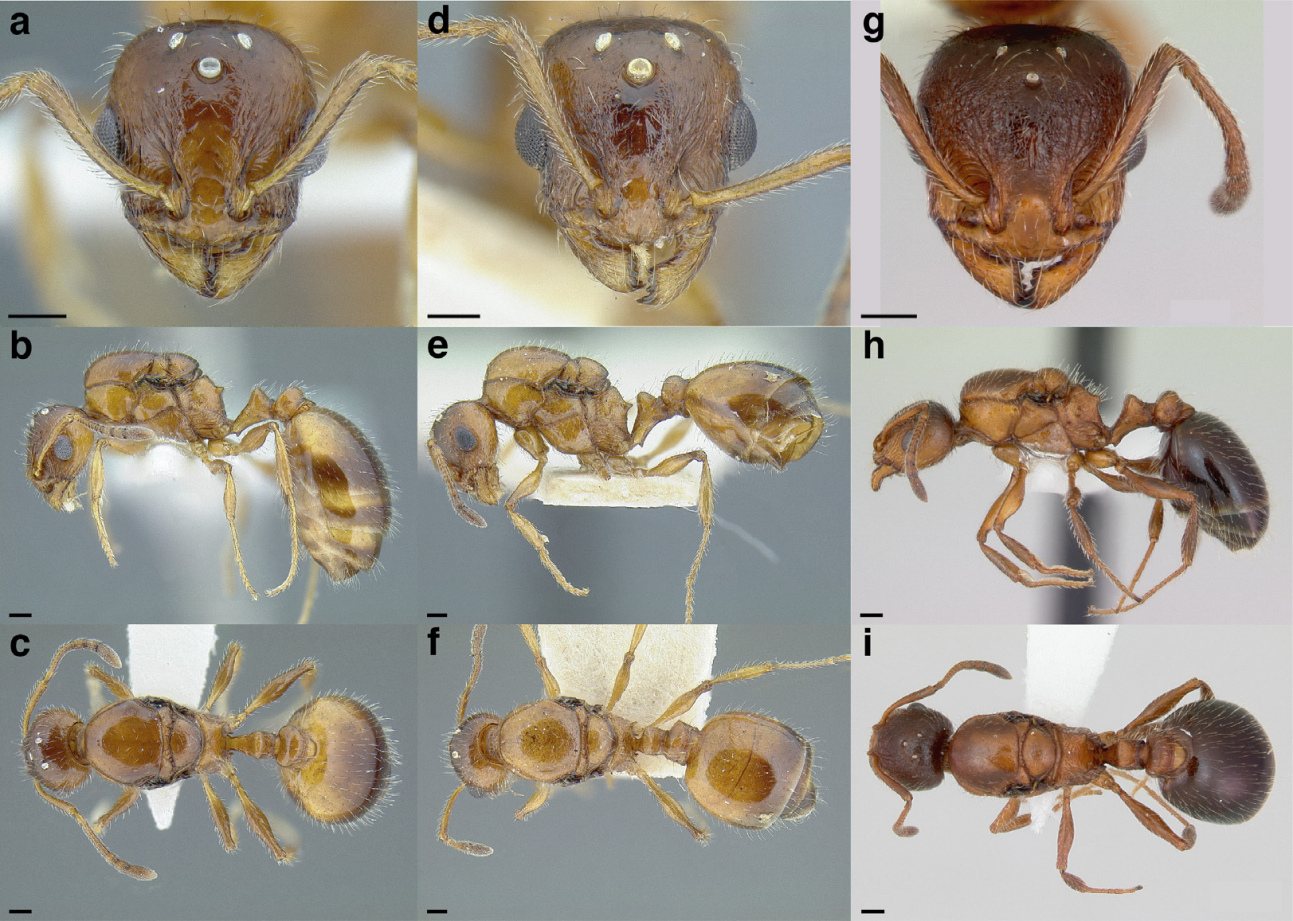
Comparison of *Temnothorax pergandei* gyne morphology. (A–C) Missouri, U.S.A. (MCZENT00583596). (A) Full-face view. (B) Profile view. (C) Dorsal view. (D–F) Mississippi, U.S.A. (USNMENT00529209). (D) Full-face view. (E) Profile view. (F) Dorsal view. (G–I) Arizona, U.S.A. (CASENT0172990). (G) Full-face view. (H) Profile view. (I) Dorsal view. Scale bars 0.2 mm.

Distribution: Fig. 127B; worker, gyne & male: Fig. 129; worker variability: Fig. 130; gyne variability: Fig. 131.

*Leptothorax (Dichothorax) pergandei* Emery, 1895: 323, pl. 8, fig. 13. Syntype workers. Washington, D.C., U.S.A. One syntype worker here designated **lectotype**.

*Leptothorax (Dichothorax) floridanus* Emery, 1895: 323. Syntype workers. Junior synonym of *Temnothorax pergandei* by Mackay, 1993: 289. One syntype here designated **lectotype**.

*Leptothorax (Dichothorax) pergandei* Emery: Wheeler, 1903b: 257: gyne and male.

*Leptothorax (Dichothorax) pergandei flavus* Smith, 1929: 549. Syntype workers. Adaton, Mississippi. Junior synonym of *Temnothorax pergandei* by Creighton, 1950: 260. One syntype worker here designated **lectotype**.

*Leptothorax (Dichothorax) manni* Wesson, 1935: 208. Syntype workers, gyne, and male. Nashville, Tennessee. Junior synonym of *Temnothorax pergandei* by Wesson, 1939: 180. One syntype worker here designated **lectotype**.

*Leptothorax (Dichothorax) pergandei* Emery: Wheeler & Wheeler, 1960: 23: larva.

*Temnothorax pergandei* (Emery): Bolton, 2003: 272. First combination in *Temnothorax*.

**Type material examined:** *Lectotype worker of* Leptothorax (Dichothorax) pergandei: U.S.A.: District of Columbia: Washington, D.C.: September 1910, [no collector data], no. 54078 U.S.N.M. cotype, (USNMENT00922524) [USNM].

*Paralectotype workers of* Leptothorax (Dichothorax) pergandei: same data as lectotype: 1 worker (USNMENT00922508) [USNM] 1 worker (USNMENT00922509) [USNM] 1 worker (USNMENT00922515) [USNM] 1 worker (USNMENT00922517) [USNM] 1 worker (USNMENT00922518) [USNM] 1 worker (USNMENT00922510) [USNM] 1 worker (USNMENT00922511) [USNM] 1 worker (USNMENT00922522) [USNM] 1 worker (USNMENT00922523) [USNM] 1 worker (USNMENT00922525) [USNM] 1 worker (USNMENT00532783) [USNM] 1 worker (images of CASENT0904767 examined on antweb.org) [MSNG].

*Lectotype worker of* Leptothorax (Dichothorax) pergandei flavus: U.S.A.: Mississippi: Oktibbeha County: Adaton, [no collection date], M.R. Smith, cotype 52535 U.S.N.M., (USNMENT00921850, top specimen) [USNM].

*Paralectotype workers and gyne of* Leptothorax (Dichothorax) pergandei flavus: same pin as lectotype (bottom specimen) [USNM]; same data as lectotype: 1 dealate gyne (USNMENT00529209) [USNM] 2 workers (USNMENT00529210) [USNM] 3 workers, top missing gaster, middle missing head (USNMENT00921851) [USNM] 3 workers (USNMENT00921852) [USNM] 3 workers (USNMENT00921849) [USNM].

*Lectotype worker of* Leptothorax (Dichothorax) floridanus: U.S.A.: Florida: Highlands County: Ft. George, 18 July 1882, [no collector data], No. 37, No. 54079, U.S.N.M. cotype (USNMENT00532780) [USNM].

*Paralectotype workers of* Leptothorax (Dichothorax) floridanus: same data as lectotype: 1 worker (USNMENT00921856) [USNM] 1 worker (USNMENT00921857) [USNM] 1 worker (images of CASENT0904768 examined on antweb.org) [MSNG].

*Lectotype worker of* Leptothorax (Dichothorax) manni: U.S.A.: Tennessee: Davidson County: Nashville, [no collection date], [no collector data] (top specimen on pin, missing gaster) (images of USNMENT00529639 examined on the Smithsonian National Museum of Natural History Department of Entomology Collections website) [USNM].

*Paralectotype gyne and male of* Leptothorax (Dichothorax) manni: same pin as lectotype: 1 male, 1 dealate gyne (images of USNMENT0529639 examined on the Smithsonian National Museum of Natural History Department of Entomology Collections website).

**Non-type material examined:** MEXICO: Hidalgo: 1.8 km NE Tezontepec, 20.20574°N 99.27112°W ± 50 m, 1,990 m, 18 July 2005, P. Hernández#05IIaTna311, xerophyll scrub, pitfall, 3 workers (CASENT0608344- CASENT0608346) [JTLC]. Nuevo León: 33 mi east of General Bravo, 25.93661°N 98.674200°W, 245 m, 12 March 1953, W.S. Creighton, 4 workers (CASENT0759990) [FSCA].

U.S.A.: Alabama: Colbert County: Cane Creek Canyon Nature Preserve, 34.62333°N 87.80278°W, 245 m, 2 June 2014, J.A. MacGown, upland hardwood forest, 1 worker (MEM243844) [MEM]; Mobile County: Citronelle, 31.09046°N 88.22798°W, 95 m, 11 June 1924, W.S. Creighton, 3 workers (CASENT0867001) [FSCA]. Arizona: Cochise County: 1 km S junction of Route 533 on State Line Road, 11 September 1997, 1,280 m, S.P. Cover#SPC4993, overgrazed desert with scattered *Ephedra*, mesquite and *Acacia*, bare hole entrance in *Hilaria* patch, fine sand w/ clay, 1 male & 1 worker (MCZENT00583595) [MCZC]; Chiricahua Mountains, Cave Creek Canyon, 0.6 km N of Southwestern Research Station, 31.88883°N 109.20433°W, 1,740 m, 15 August 2001, S.P. Cover#SPC6341, oak-juniper woodland, nest in base of grass clump, 1 worker (CASENT0758667) [UCDC]; Chiricahua Mountains, Paradise cemetery, 31.93167°N 109.20833°W, 1,682 m, 4 August 2001, R.A. Johnson#RAJ2539, juniper grassland, small tumulus nest, 3 workers (CASENT0869114) [RAJC] 4 workers (CASENT0869115) [RAJC]; Chiricahua Mountains, Tex Canyon, 31.68167°N 109.32°W, 1,625 m, 12 August 2002, R.A. Johnson#RAJ2913, oak-juniper-mesquite woodland, stray foragers, 4 workers (CASENT0869113) [RAJC]; Santa Cruz County: Sonoita, 31.67833°N 39.11667°W, 1,490 m, S.L. Heydon, sweeping roadside, 1 worker & 1 dealate gyne (CASENT0731541) [MMPC]; Patagonia Mtns. 0.4 mi N Jct FSR 135 on FSR 49, 31.43382°N 110.72094°W, 1,645 m, 31 August 1999, S.P. Cover#SPC5709, juniper and Emory oak woodland, 25’ tall on gentle W-facing slope, sandy loam, found when excavating 5,708, grassy open slope, 1 worker & 1 dealate gyne (MCZENT00583600) [MCZC] 2 workers (MCZENT00583601) [MCZC] 2 workers (MCZENT00583602) [MCZC]; same data as previous, except: S.P. Cover#SPC5704, found when digging 5,705, at edge of grassy gap in sun, 1 worker & 1 dealate gyne (MCZENT00583603) [MCZC] 2 workers (MCZENT00583604) [MCZC] 2 workers (MCZENT00583605) [MCZC] 2w (CASENT0869110) [RAJC] 3w (CASENT0869111) [RAJC] 3w (CASENT0869112) [RAJC]; 1 mi S American Peak, 31.43333°N 110.72167°W, 1,625 m, 30 September 2005, R.A. Johnson#RAJ3598, oak-pinyon-juniper-grassland, in soil of excavated *Trachymyrmex* nest, 2 workers (CASENT0869116) [RAJC]. Arkansas: Garland County: Hot Springs, 18 October 1932, D.E. Read, 1 dealate gyne & 2 workers (CASENT0758293) [USNM]; Montgomery County: Ouchita Mountains, 2.4 km NW junction Route 27N on Route 270 (in Mt. Ida). 14 May1996, S.P. Cover#SPC4663, *Quercus alba*, *Q. falcata*, *Ulmus alata*, shortleaf pine, 18 m tall on gentle slope, rotten branch on litter surface in shade, 2 workers, 1 dealate gyne (MCZENT00583597) [MCZC] 2 workers (MCZENT00583598) [MCZC]; Polk County: Ouachita Mountains Biological Station, 34.45806°N 93.99889°W, 475 m, 23 June 2017, J.G. Hill, in glade mixed hardwood/pine forest, 1 worker (MEM244952) [MEM]. Florida: Alachua County: Doyle Conner Bldg., Gainesville, 29.63499°N 82.37152°W, 30 m, 1 May 1974, F.W. Mead, blacklight trap, 1 male (CASENT0867165) [FSCA]; Gainesville, 29.65163°N 82.32484°W, 30 m, 22 April 1971, F.W. Mead, blacklight trap, 1 male (CASENT0867167) [FSCA]; Citrus County: Citrus Wildlife Management Area, 8.5 km W junction Route 41 on Route 44 on Sand Road 3 April 1995, S.P. Cover#SPC4390, open longleaf pine-turkey oak sandhill forest, to 15 m tall, rotten pine stub 5 cm in diameter in open sparsely grassy gap, pure sand, 1 worker & 1 dealate gyne (MCZENT00583599) [MCZC]; Southern Ridge Sandhill, Archbold Biological Station, 21.18667°N 81.35200°W, 60 m 24 September 2010, S.P. Cover#SPC8411, pine-oak forest, nest in rotten stick in leaf litter, 1 worker (CASENT0758668) [UCDC]; Columbia County: Osceola National Forest, 30.20198°N 82.45857°W, 50 m, 28 October 1976, J.R. Wiley, blacklight trap, 3 males (CASENT0867164) [FSCA]; same data as previous, except: 12 December 1976, C. Ross, blacklight trap, 1 male (CASENT0867166) [FSCA]; Liberty County: Torreya State Park, 30.56652°N 84.94780°W, 55 m, 30 August 1978, L.A. Stange, blacklight trap, 2 males (CASENT0867162) [FSCA]; Marion County: 11 mi E of Okla. R., Ocala National Forest, 29.16676°N 81.79165°W, 45 m, 14 October 1959, H.A. Denmark, *Pinus clausa*, 14 workers & 1 dealate gyne (CASENT0759991- CASENT0759995) [FSCA]; Juniper Springs, Ocala National Forest, 29.18387°N 81.712023°W, 5 m, 27 June 1960, H.A. Denmark, *Pinus clausa*, 1 worker (CASENT0759996) [FSCA]; same data as previous, except: 28 June 1960, H.A. Denmark, *Pinus clausa*, 1 worker (CASENT0867011) [FSCA]; same data as previous, except: 26 November 1960, H.A. Denmark, *Pinus clausa*, 3 workers (CASENT0867004-CASENT0867006) [FSCA]; same data as previous, except: 28 November 1960, H.A. Denmark, *Pinus clausa*, 1 worker (CASENT0867007) [FSCA]; same data as previous, except: 21 December 1959, H.A. Denmark, *Pinus clausa*, 4 workers (CASENT0867008, CASENT0867009, CASENT0867016, CASENT0867017) [FSCA]; same data as previous, except: 21 December 1959, H.A. Denmark, *Pinus clausa*, 2 workers (CASENT0867008, CASENT0867009) [FSCA]; same data as previous, except: 20 May 1960, H.A. Denmark, *Pinus clausa*, 7 workers (CASENT0867010, CASENT0867012- CASENT0867015) [FSCA]; Monroe County: Cape Sable, 25.27290°N 81.12147°W, 5 m, 4 April 1958, H.V. Weems, 1 worker (CASENT0867002) [FSCA]; Little Torch Key, Torchwood Hammock, end of Pirates Rd., 24.65442°N 81.38783°W, 12 m, 13 March 2010, C.S. Moreau#CSM1380b, 1 worker (CASENT0759999) [FSCA]; Saddlebunch Keys, 24.60515°N 81.59398°W, 5 m, 29 December 1959, H.V. Weems, on *Flaveria linearis*, 1 male (CASENT0759989) [FSCA]; Okaloosa County: 4.5 mi NW Holt, A & M Res. Sta., Blackwater River State Forest, 30.73583°N 86.81628°W, 20 m, 1 September 1979, L.A. Stange, blacklight trap, 1 male (CASENT0867163) [FSCA]; Fam Biol. Sta., 3 mi NW Holt, 30.73137°N 86.80135°W, 20 m, 8 August 1979, L.A. Stange, blacklight trap, 2 males (CASENT0867161) [FSCA]; Orange County: Indian River City, Titusville Well Field, 0.2 km S Route 50, 28.53333°N 80.81483°W, 20 m, 1 March 2001, S.P. Cover#SPC6168, oak-sand pine scrub, hollow stick buried in litter, 1 worker (CASENT0758666) [UCDC]; Wekiwa Springs State Park, 28.71065°N 81.46084°W, 20 m, 20 November 1993, Z. Prusak, sandhill, 1 dealate gyne & 2 workers (CASENT0867107) [FSCA]; Osceola County: 7.4 mi S of Narcoossee, 28.20869°N 81.17210°W, 25 m, 1 February 1959, R.E. Woodruff, in malt trap, 2 workers (CASENT0867043) [FSCA]; Putnam County: 29.62652°N 81.77870°W, 20 m, 9 August 1948, A. Van Pelt, *P. palustris* - *Q. cinerea*, 3 workers (CASENT0867018) [FSCA]; same data as previous, except: 4 September 1948, A. Van Pelt, hydric hammock, 63 workers (CASENT0867019-CASENT0867039) [FSCA]; same data as previous, except: 10 December 1948, A. Van Pelt, Turkey oak, 6 workers (CASENT0867040, CASENT0867041) [FSCA]; same data as previous, except: 16 October 1948, A. Van Pelt, Q. spp., Leon, 14 workers & 1 dealate gyne (CASENT0867042, CASENT0867044-CASENT0867047, CASENT0867055, CASENT0867056) [FSCA]; same data as previous, except: 11 October 1948, A. Van Pelt, *P. palustris - Q. cinerea*, 19 workers (CASENT0867048-CASENT0867054) [FSCA]; same data as previous, except: 3 August 1948, A. Van Pelt, Pong pine-Fetterbush, 3 workers (CASENT0867058) [FSCA]; same data as previous, except: 9 August 1948, A. Van Pelt, *P. palustris - Q. cinerea*, 32 workers (CASENT0867062-CASENT0867072) [FSCA]; same data as previous, except: 3 September 1948, A. Van Pelt, *Quercus* spp., 18 workers (CASENT0867073, CASENT0867080, CASENT0867082-CASENT0867085) [FSCA]; same data as previous, except: 21 September 1948, A. Van Pelt, *P. palustris - Q. cinerea*, 25 workers (CASENT0867074- CASENT0867079, CASENT0867081, CASENT0867096, CASENT0867097) [FSCA]; ]; same data as previous, except: 2 October 1948, A. Van Pelt, mesic hammock, 14 workers (CASENT0867087-CASENT0867091) [FSCA]; same data as previous, except: 11 October 1948, A. Van Pelt, *P. palustris - Q. cinerea*, 19 workers (CASENT0867048-CASENT0867054) [FSCA]; same data as previous, except: 28 October 1948, A. Van Pelt, *P. palustris*, 2 workers (CASENT0867059) [FSCA]; same data as previous, except: 14 November 1948, A. Van Pelt, mesic hammock, 13 workers (CASENT0867098-CASENT0867102) [FSCA]; same data as previous, except: 5 December 1948, A. Van Pelt, *P. clausa - Q*. spp., 5 workers (CASENT0867060-CASENT0867061) [FSCA]; same data as previous, except: 23 April 1949, A. Van Pelt, *P. palustris - Q. cinerea*, 2 workers (CASENT0867057) [FSCA]; same data as previous, except: 6 September 1949, A. Van Pelt, *Q. cinerea*, 11 workers & 1 dealate gyne (CASENT0867092-CASENT0867095) [FSCA]; same data as previous, except: 17 January 1950, A. Van Pelt, *P. serotina - Desmoth*, 10 workers (CASENT0867086, CASENT0867103-CASENT0867105) [FSCA]; Sarasota County: Myakka River State Park, 27.24028°N 82.31518°W, 5 m, 1 February 1990, J. Longino#JTL2604, 2 workers (CASENT0867106) [FSCA]; Seminole County: 21st District, Lot#172, 30.9613°N 84.85753°W, 35 m, 13 June 1953, P.B. Kannowski#PBK775, pine-oak, on sand, 3 workers (CASENT0867003) [FSCA]; Walton County: 0.3 km S of the junction of AFB 211 on AFB 214 at Indigo Pond, Elgin Airforce Base, 30.69655°N 86.33200°W, 70 m, 12 June 2013, S.P. Cover#SPC8557, pine-oak forest, in rotten pine branch, 1 worker (CASENT0758669) [UCDC]. Georgia: Clarke County, Athens, 05 April 1975, P. Decelles#750405-2, 1 dealate gyne & 2 workers (CASENT0758292) [USNM]; Rabun County: Tallulah Gorge State Park, 34.73722°N 83.39306°W, 575 m, 5 June 2016, J.G. Hill, hardwood forest, mountain trail, 1 worker (MEM245145) [MEM]; Whitfield County: Pinhoti trail, Mt. Sinai, 34.739°N 85.0167°W, 485 m, 30 March 2012, D. Booher, hardwood forest, SW facing slope, ex *Carya* nut, 1 worker (CASENT0758874) [UGCA]. Illinois: Pope County: Herod, 37.58033°N 88.43616°W, 135 m, 6 November 1942, Ross & Sanders#INHS439.048, ground litter, 1 worker (CASENT0759704) [INHS]; Union County: Giant City, 15 May 1968, R.E. Levy, 3 workers (CASENT0758308) [UCDC]. Iowa: Boone County: Boone, 42.05971°N 93.88023°W, 345 m, 11 May 1941, Wm. Buren, 1 worker (CASENT0867168) [FSCA]; Clayton County: Elkader, 23 June 1941, W.F. Buren, 2 males & 1 worker (CASENT0758291) [USNM] 1 male & 1 alate gyne (CASENT0758229) [USNM]; Mill County: Glenwood, 41.04694°N 95.742511°W, 310 m, June 1940, Wm. Buren, 1 worker (CASENT0867160) [FSCA]. Kentucky: Madison County: Berea, 37.60083°N 84.29972°W, 295 m, 22 July 2013, D.M. Daugherty, Lindgren funnel trap baited with IPS lure, 1 worker (MEM245110) [MEM]. Louisiana: Jefferson County: Jean Lafitte National Historical Park and Preserve, 29.76972°N 90.13°W, 5 m, 16 June 2016, X. Chen, on tallow root, 1 worker (MEM243841) [MEM]. Mississippi: Winston County: Tombigbee National Forest, 33.21556°N 89.10417°W, 210 m, 6 June 2012, J.A. MacGown, sloped hardwood forest, in *Carya glabra* nut, 4 workers, 1 alate gyne, 1 male (CASENT0868827, CASENT0868956-CASENT0868960) [MEM]. Missouri: Barry County: 4 miles WSW of Eagle Rock, 36.51972°N 93.82028°W, 405 m, 14 June 2007, L.E. Watrous, from glade soil, 1 worker (MEM244988) [MEM]; Ozark Mtns. 14 km S junction Route 76/86 on Route 112 at FSR 1037, 19 May 1996. S.P. Cover#SPC4759, black oak, white oak forest 18–21 m tall, in *Quercus borealis* acorn on litter surface in light shade, 1 worker & 1 dealate gyne (MCZENT00583596) [MCZC]. New Mexico: Hidalgo County: 1,234 m, 16 km N Rodeo, 31.9774°N 109.0356°W, G.W. Forister, 9 September 2010, 1 worker (CASENT0758313) [UCDC]. North Carolina: Orange County: 9 km NNE Hillsborough, 36.15000°N 79.06667°W, 175 m, 22 July 1983, J. Longino#JTL22Jul83/stray, piedmont woodland, 3 workers (LACMENT141184-LACMENT141186) [JTLC]; Stanly County: Morrow Mountain State Park, 9 October 2011, B. Guenard, hand collecting, 1 worker (CASENT0758261) [MMPC] 1 worker (CASENT0732597) [UCDC]; Wake County: park at turnoff on Tanager, 35.759970°N 78.688698°W, 2 June 2008, S.B. Menke#SBM_272, pitfall P8, 2 workers (CASENT0758310) [UCDC]. Ohio: Adams: Abner Hollow Rd., Green Twp., 38.71561°N 83.43808°W, 170 m, 21 August 1997, G.A. Coovert, 2 workers (CASENT0759997, CASENT0759998) [FSCA]. Tennessee: Davidson County: Hendersonville, 36.33587°N 86.56801°W, 175 m, 19 June 2011, B.E. Boudinot#BEB000439, suburban, lawn and fields, 1 worker (TESC00009756) [BEBC]; McNairy County: Big Hill Pond State Park, 35.04417°N 88.73167°W, 150 m, 9 July 2012, J.G. Hill, sloped hardwood forest, 1 worker (MEM245132) [MEM]. Texas: Brewster County: Chisos Mountains, The Basin, 1,540 m, 27 July 1970, P. Ward#3785-6, open vegetation pine-oak-juniper, ex honey bait, 3 workers (CASENT0915983) [PSWC] 3 workers (CASENT0758355) [PSWC] 2 workers (CASENT0758307) [UCDC]; Cameron County: Laguna Atacosa National Wildlife Refuge, 5 m, 15 December 1984, P.S. Ward#7197-2, coastal mesquite scrub, ground forager(s), 2 workers (CASENT0758356) [PSWC] 1 worker (CASENT0758309) [PSWC]; Jeff Davis County: 38 km S Kent, 15 August 1996, L.A. Baptiste, sweeping vegetation, 1 worker (CASENT0758311) [UCDC] 1 worker (CASENT0758312) [UCDC]. Virginia: Fairfax County: Vienna, 38.89835°N 77.26515°W, 100 m, 22 June 1983, J. Longino#JTL22Jun83/Vienna, waste ground in urban area, strays, 4 workers (LACMENT141180-LACMENT141183) [JTLC].

**Geographic range:** All elevations, eastern U.S.A. as far north as Michigan, as far west as Arizona, and south as far as Hidalgo, Mexico (Fig. 127B).

**Worker diagnosis:** *Temnothorax pergandei* can be separated from all other species in the *salvini* clade by the following character combination: promesonotum evenly convex; metanotal groove deeply impressed and propodeum depressed below the level of the promesonotum; propodeal spines present and typically represented by small teeth; in profile view, postpetiole evenly convex dorsally, with a small flat area posteriorly; in dorsal view, petiolar node 1.1 to 1.3 times as broad as caudal cylinder; setae on head, mesosoma, legs, waist segments and gaster erect, moderately long, abundant and tapering; integument variously colored: may be uniformly dark brown, uniformly yellow, or bicolored.

**Similar species:** This striking species is difficult to confuse with any other species in *Temnothorax*, except the closely related *T. bison* sp. nov. The presence of propodeal teeth, the structure of the postpetiole, which bulges anterodorsally in *T. bison* sp. nov., and the relative width of the petiolar node in dorsal view (1.3 to 1.6 times as broad as the caudal cylinder in *T. bison* sp. nov.) will separate the two species.

**Worker measurements & indices (*n* = 20):** SL = 0.543-0.708 (0.631); FRS = 0.213-0.283 (0.248); CW = 0.643–0.853 (0.726); CWb = 0.597–0.784 (0.663); PoOC = 0.262–0.307 (0.284); CL = 0.701–0.860 (0.766); EL = 0.165–0.227 (0.189); EW = 0.106–0.158 (0.132); MD = 0.141–0.199 (0.172); WL = 0.847–1.135 (1.003); SPST = 0.109–0.222 (0.177); MPST = 0.278–0.399 (0.325); PEL = 0.346–0.500 (0.403); NOL = 0.195–0.272 (0.226); NOH = 0.099–0.156 (0.128); PEH = 0.187–0.275 (0.234); PPL = 0.171–0.255 (0.224); PPH = 0.240–0.344 (0.294); PW = 0.413–0.557 (0.473); SBPA = 0.118–0.228 (0.194); SPTI = 0.134–0.264 (0.220); PEW = 0.158–0.225 (0.191); PNW = 0.178–0.262 (0.228); PPW = 0.257–0.372 (0.322); HFL = 0.609–0.798 (0.703); HFWmax = 0.137–0.186 (0.160); HFWmin = 0.051–0.068 (0.059); CS = 0.948–1.214 (1.046); ES = 0.226–0.306 (0.255); SI = 84–104 (95); OI = 23–26 (24); CI = 82–91 (87); WLI = 142–157 (151); SBI = 20–33 (29); PSI = 13–21 (18); PWI = 156–183 (169); PLI = 155–213 (181); NI = 144–245 (178); PNWI = 111–133 (120); NLI = 51–64 (56); FI = 239–315 (271).

**Worker description:** In full-face view, head subquadrate, longer than broad (CI 82–91). Mandibles densely striate but shining and armed with five teeth: the apical-most well developed and acute, followed by a less developed preapical tooth and three equally developed smaller teeth. Anterior clypeal margin weakly convex medially. Antennal scapes moderately long: when fully retracted, surpassing the posterior margin of the head capsule by about the maximum width of the scape (SI 84–04). Antennae 12-segmented; antennal club of composed of three segments, with the apical-most segment about as long as the preceding two in combination. Frontal carinae short and apices directed laterally, extending past the antennal toruli by about the maximum width of the antennal scape. Compound eyes moderately protruding past the lateral margins of the head capsule. Lateral margin of head convex, forming a continuous arc from the mandibular insertions to the posterior margin of the head. Posterior head margin flat but rounding evenly into the lateral margins.

In profile view, compound eyes ovular and moderately large (OI 23–26), with 16 ommatidia in longest row. Pronotal declivity indistinct, neck and anterior face of pronotum forming a ~120° angle. Mesosoma with promesonotum forming an even convexity; metanotal groove deeply impressed, distinctly separating the promesonotum from the propodeum, which is depressed below the level of the promesonotum and dorsally convex. Promesonotal suture extending from the posterior margin of the procoxal insertion to the mesothoracic spiracle, which is moderately well developed, then continuing to the dorsal surface as a very weak sulcus. Propodeal spiracle well developed, directed posterolaterally, and separated from the propodeal declivity by about three spiracle diameters. Propodeal spines poorly developed and short (PSI 13–21), in the form of small triangular teeth, angled posterodorsally. Propodeal declivity flat, forming a ~120° angle with the base of the propodeal spines. Propodeal lobes rounded and weakly developed. Metapleural gland bulla large, extending from the metacoxal insertion three quarters of the way to the propodeal spiracle. Petiole moderately long (PLI 155–213), without tubercles anterodorsally. Subpetiolar process in the form of a small, triangular, blunt tooth, which continues as a low carina to the caudal cylinder; ventral margin of petiole posterior to the subpetiolar process strongly concave. Petiolar peduncle moderately long: comprising about half of the petiole. Petiolar node robust and cuneiform: transition between peduncle and node marked by a rounded angle of ~130°; anterior face forming a rounded ~90° angle with the posterior face; caudal cylinder long: about the as long as the maximum width of the antennal scape. Postpetiole weakly convex anteriorly, rounding evenly into the dorsal face, which bulges before flattening posteriorly; lobed ventrally.

In dorsal view, humeri weakly developed: evenly rounded and wider than the rest of the mesosoma, but not distinct from it; mesothoracic spiracles weakly protruding past the lateral margins of the mesosoma, visible as slight angles where the pronotum meets the mesonotum. Promesonotal suture visible as a weak sulcus. Metanotal strongly impressed, distinctly dividing the promesonotum from the propodeum. Propodeal spines very short, but broadly approximated basally and diverging apically, their apices separated from each other by about five times their length. Petiolar peduncle with spiracles protruding past the lateral margins, but not noticeably constricted anterior to them. Petiolar node, when viewed posterodorsally, trapezoidal and apically broadened, the apex flat; caudal cylinder narrower than the apex of the node. Postpetiole narrow (PWI 156–183) and subquadrate. Anterior margin of the postpetiole weakly convex and meeting the lateral margins at a ~95° angle; lateral margins parallel to the angulate posterior corners; posterior margin broadly concave. Metafemur moderately to strongly incrassate (FI 239–315).

Sculpture: median clypeal carina present, extending posteriorly to the level of the anterior margins of the antennal toruli, and flanked on either side by one weaker carina. Lateral clypeal lobes with additional, weaker carinae; ground sculpture smooth and shining on the posterior half of the median clypeal lobe, and weakly areolate anteriorly. Antennal scapes shining through weak coriarious ground sculpture. Cephalic dorsum predominantly weakly coriarious; concentric costulae surrounding the antennal insertions, extending from the apices of the frontal carinae, to the anterior margins of the lateral clypeal lobes. Lateral surfaces of head predominantly weakly coriarious, with strong costulae between the compound eye and the mandibular insertion. Ventral surface of head smooth and shining. Pronotal neck areolate. Lateral surface of the pronotum and the surface surrounding the propodeal spiracle weakly coriarious. Lateral face of the propodeum, meso- and metapleurae with strong costulae. Dorsal surface of promesonotum weakly coriarious; dorsal surface of propodeum areolate. Femora smooth and shining. Petiole shining ventrally through weak coriarious sculpture; dorsum of petiolar peduncle and lateral face of petiolar node with weak costulae; petiolar node otherwise weakly coriarious. Postpetiole weakly coriarious. First gastral tergite and sternite weakly coriarious, without spectral iridescence.

Setae: antennal scapes and funiculi with long, subdecumbent pilosity. Dorsum of the head, pronotum, waist segments, and gaster with abundant, erect, flexuous, tapering setae, the longest of which are slightly longer than the length of the compound eye. The head bears ~36, mesosoma ~32, petiole 18, postpetiole ~28, and first gastral tergite ~66 setae. Pubescence is long, coarse, difficult to distinguish from the setae, and present over the entire body except for the ventral surface of the petiole.

Color: predominantly medium brown, with mandibles and tarsi testaceous yellow; masticatory margins of the mandibles dark brown.

**Gyne measurements & indices (*n* = 9):** SL = 0.633–0.728 (0.680); FRS = 0.270–0.327 (0.301); CW = 0.848–0.963 (0.906); CWb = 0.755–0.868 (0.816); PoOC = 0.289–0.339 (0.315); CL = 0.821–0.943 (0.880); EL = 0.244–0.284 (0.267); EW = 0.173–0.222 (0.198); MD = 0.142–0.195 (0.171); WL = 1.405–1.554 (1.488); SPST = 0.203–0.274 (0.240); MPST = 0.352–0.458 (0.407); PEL = 0.453–0.515 (0.492); NOL = 0.212–0.319 (0.262); NOH = 0.135–0.181 (0.162); PEH = 0.293–0.362 (0.328); PPL = 0.229–0.291 (0.259); PPH = 0.370–0.439 (0.406); PW = 0.786–0.903 (0.835); SBPA = 0.341–0.405 (0.377); SPTI = 0.307–0.439 (0.370); PEW = 0.233–0.277 (0.258); PNW = 0.256–0.328 (0.295); PPW = 0.384–0.465 (0.429); HFL = 0.793–0.896 (0.852); HFWmax = 0.160–0.185 (0.177); HFWmin = 0.063–0.085 (0.072); CS = 1.166–1.340 (1.256); ES = 0.343–0.395 (0.366); SI = 79–89 (83); OI = 27–32 (29); CI = 90–96 (93); WLI = 178–188 (183); SBI = 41–49 (46); PSI = 14–18 (16); PWI = 161–170 (166); PLI = 175–223 (191); NI = 143–233 (163); PNWI = 107–119 (114); NLI = 46–63 (53); FI = 209–283 (248).

**Gyne description:** In full-face view, head subquadrate, slightly longer than broad (CI 90–96). Mandibles densely striate but shining and armed with five teeth: the apical-most well developed, followed by a less developed preapical tooth and three equally developed smaller teeth. Anterior clypeal margin flat medially. Antennal scapes moderately long: when fully retracted, just reaching the posterior margin of the head capsule (SI 79–89). Antennae 12-segmented; antennal club composed of three segments, with the apical-most segment as long as the preceding two in combination. Frontal carinae moderately long, directed laterally at their apices, and extending past the antennal toruli by about three times the maximum width of the antennal scape. Compound eyes moderately protruding past the lateral margins of the head capsule. Lateral margin of head evenly convex, converging from below the compound eyes to the mandibular insertions. Posterior head margin flat, rounding evenly into the lateral margins.

In profile view, compound eyes ovular and large (OI 27–32), with 20 ommatidia in longest row. Mesoscutum rounded evenly anteriorly, not quite covering the dorsal surface of the pronotum, and weakly convex dorsally. Mesoscutellum on the same level of the mesoscutum, and evenly convex dorsally. Posterior margin of metanotum extending slightly past the posterior margin of the mesoscutum. Propodeal spiracle well developed, directed posterolaterally, and separated from the propodeal declivity by about two and a half spiracle diameters. Propodeal spines short and stout (PSI 14–18), represented by posterodorsally directed, blunt, triangular teeth, the bases of which are attached to the propodeal declivity by flanges. Propodeal declivity straight and flat, forming a ~120° angle with the base of the propodeal spines. Propodeal lobes rounded and weakly developed. Metapleural gland bulla large, extending from the metacoxal insertion three quarters of the way to the propodeal spiracle. Petiole moderately long (175–223), without tubercles anterodorsally. Subpetiolar process in the form of a small, triangular, blunt tooth, which continues as a low carina to the caudal cylinder; ventral margin of petiole posterior to the subpetiolar process strongly concave. Petiolar peduncle moderately long: comprising about half the length of the petiole. Petiolar node robust and cuneiform: transition between peduncle and node marked by a rounded angle of ~130°; anterior face forming a rounded ~90° angle with the posterior face; caudal cylinder long: about the as long as the maximum width of the antennal scape. Postpetiole weakly convex anteriorly, rounding evenly into the dorsal face, which bulges before flattening posteriorly; lobed ventrally.

In dorsal view, mesoscutum not fully covering pronotum anteriorly; humeri evenly rounded laterally. Propodeal teeth diverging apically, their apices separated from each other by about six times their length. Petiolar peduncle with spiracles protruding past the lateral margins, but not noticeably constricted anterior to them. Petiolar node, when viewed posterodorsally, trapezoidal and apically broadened, the apex weakly emarginate; caudal cylinder narrower than the apex of the node. Postpetiole narrow (PWI 161–170), and subquadrate. Anterior margin of postpetiole weakly convex, with corners marked by rounded angles as it transitions to the lateral margins, which are parallel to the angulate posterior corners; posterior margin broadly concave. Metafemur weakly to moderately incrassate (FI 209–283).

Sculpture: median clypeal carina present, extending from the anterior margin nearly to the level of the anterior margins of the antennal insertions; flanked by weaker, indistinct carinae near the lateral margins of median clypeal lobe. Lateral clypeal lobes with additional weaker carinae; ground sculpture smooth and shining on the median clypeal lobe medially but becoming weakly areolate-coriarious laterally. Antennal scapes weakly coriarious. Cephalic dorsum predominantly costulate over smooth and shining sculpture, with moderately strong striae flanking the frontal carinae; a median strip of smooth sculpture extends from the frontal triangle to the median ocellus, then expands to encompass the middle of the ocellar triangle and extends to the posterior head margin; concentric costulae surrounding the antennal insertions, extending from the frontal carinae to the anterior margin of the lateral clypeal lobes. Lateral surfaces of head with weak coriarious sculpture posterior to the compound eye, denser rugose sculpture surrounding the compound eye, extending between the compound eye and the mandibular insertion. Ventral surface of head weakly costulate. Pronotal neck areolate. Lateral face of the pronotum weakly coriarious, with costulae on the ventral third. Anepisternum smooth and shining on its anterior half, but weakly coriarious on its posterior half; katepisternum predominantly weakly coriarious. Metapleuron and lateral face of propodeum with costulae. Propodeal declivity transversely coriarious. Mesoscutum with predominantly longitudinal coriarious sculpture, with a patch of transverse, finely costulate sculpture anteromedially, and a shallow sulcus posteromedially. Mesoscutellum smooth and shining medially, surrounded by weak coriarious sculpture. Femora smooth and shining. Petiole shining ventrally through weak coriarious sculpture; dorsum of petiolar peduncle and lateral face of petiolar node with weak costulae; petiolar node otherwise weakly coriarious. Postpetiole coriarious, but with sculpture weaker anteromedially. First gastral tergite and sternite weakly coriarious, without spectral iridescence.

Setae: antennal scapes and funiculi with long, subdecumbent pilosity. Dorsum of the head, pronotum, waist segments, and gaster with abundant, erect, flexuous, tapering setae, the longest of which are about the width of the compound eye. Pubescence is long, coarse, difficult to distinguish from the setae, and present over the entire body except for the ventral surface of the petiole.

Color: predominantly medium brown, with mandibles, genae, antennal scapes, antennal funiculus, pronotum, propodeum, tibiae, tarsi and venter of petiole testaceous yellow; masticatory margins of the mandibles dark brown.

**Male measurements & indices (*n* = 2):** SL = 0.261–0.324 (0.293); FRS = 0.133–0.151 (0.142); CW = 0.552–0.592 (0.572); CWb = 0.466–0.475 (0.471); PoOC = 0.251–0.266 (0.259); CL = 0.561–0.603 (0.582); EL = 0.240–0.267 (0.254); EW = 0.196–0.209 (0.203); MD = 0.038–0.041 (0.040); WL = 1.062–1.169 (1.116); SPST = 0.188–0.216 (0.202); MPST = 0.310–0.378 (0.344); PEL = 0.334–0.335 (0.335); NOL = 0.194–0.258 (0.226); NOH = 0.076–0.078 (0.077); PEH = 0.160–0.166 (0.163); PPL = 0.202–0.215 (0.209); PPH = 0.216–0.260 (0.238); PW = 0.600–0.616 (0.608); SBPA = 0.224–0.257 (0.241); SPTI = 0.224–0.257 (0.241); PEW = 0.163–0.182 (0.173); PNW = 0.142–0.197 (0.170); PPW = 0.255–0.268 (0.262); HFL = 0.819–0.891 (0.855); HFWmax = 0.093–0.098 (0.096); HFWmin = 0.051; CS = 0.747–0.777 (0.762); ES = 0.338–0.372 (0.355); SI = 56–68 (62); OI = 45–48 (47); CI = 79–83 (81); WLI = 228–246 (237); SBI = 48–54 (51); PSI = 18; PWI = 140–164 (152); PLI = 156–165 (161); NI = 249–339 (294); PNWI = 87–108 (98); NLI = 58–77 (68); FI = 182–192 (187).

**Male description:** In full-face view, head elongate and subovate, longer than broad (CI 79–83). Mandibles very weakly striate, shining, and armed with five teeth: the apical-most well developed, followed by a smaller preapical tooth and three roughly equally developed smaller teeth. Anterior clypeal margin entire and weakly convex. Antennal scapes short: when fully retracted, failing to reach the posterior margin of the head capsule by about two times the maximum width of the scape (SI 56–68). Antennae 13-segmented; antennal club composed of four segments, with the apical-most segment slightly longer than the preceding two in combination. Frontal carinae short, extending past the antennal toruli by two times the maximum width of the antennal scape. Compound eyes strongly protruding past the lateral margins of the head capsule. Margin between the anterior margin of the compound eye and the mandibular insertions straight. Posterior head margin flat convex, evenly rounding into the lateral margins.

In profile view, compound eyes ovular and large (OI 45–48), with 30 ommatidia in the longest row. Mesoscutum bulging anteriorly, covering the dorsal surface of the pronotum, and evenly convex dorsally. Mesoscutellum on the same level of the mesoscutum and evenly rounded dorsally. Posterior margin of metanotum extending beyond the posterior margin of scutellum. Propodeal spiracle well developed, directed laterally, and separated from the propodeal declivity by about four spiracle diameters. Propodeal spines absent but indicated by angulate flanges on the dorsal and declivitous faces of the propodeum. Propodeal lobes rounded and weakly developed. Metapleural gland bulla large, extending three quarters of the way between the insertion of the metacoxa and the propodeal spiracle. Petiole moderately long (PLI 156–165), without anterodorsally. Subpetiolar process absent; ventral margin of petiole concave. Petiolar peduncle short: comprising about a third of the total length of the petiole. Petiolar node low and cuneiform, the convergence of the anterior and posterior faces marked by a rounded angle. Postpetiole evenly rounded anterodorsally, flattened dorsally, and with a lobed, concave ventral surface.

In dorsal view, mesoscutum covering pronotum anteriorly, humeri not visible. Petiolar peduncle with spiracles strongly protruding past the lateral margins, the peduncle broadened where they arise. Petiolar node slightly wider than the peduncle; petiole narrowing posterior to the node, before widening again to the caudal cylinder, which is about the same width as the node. Postpetiole narrow (PWI 140–164) and campaniform. Anterior margin of postpetiole convex, with the anterior corners evenly rounding into the lateral margins, which are parallel to the angulate posterior corners; posterior margin of postpetiole broadly concave. Metafemur not incrassate (FI 182–192).

Sculpture: median clypeal carina absent. Lateral clypeal lobes with weak, indistinct carinae, ground sculpture weakly coriarious. Antennal scapes smooth and shining. Head predominantly weakly areolate-coriarious, with a central strip of smooth and shining sculpture extending from the frontal triangle to the median ocellus. Lateral surface of head weakly coriarious. Ventral surface of head smooth and shining anteriorly, transversely rugulose posteriorly. Pronotal neck areolate. Lateral surfaces of pronotum, anepisternum, katepisternum, predominantly smooth and shining, but with anterior third of pronotum densely rugose. Metapleuron and lateral face of propodeum longitudinally rugose. Dorsally, mesoscutum weakly coriarious, the Mayrian furrows moderately well impressed. Mesoscutellum smooth and shining medially, becoming coriarious laterally. Femora smooth and shining. Petiole costulate ventrally; lateral faces of the peduncle and node longitudinally rugose, the posterodorsal surface of the petiole smooth and shining. Dorsal surface of postpetiole shining. First gastral tergite and sternite weakly coriarious.

Setae: antennal scapes and funiculi with long, subdecumbent pilosity. Dorsum of the head, pronotum, waist segments, and gaster with abundant, erect, flexuous, tapering setae, the longest of which are about half the width of the compound eye. Pubescence is long, coarse, difficult to distinguish from the setae, and present over the entire body except for the ventral surface of the petiole.

Color: predominantly medium brown, with mandibles, genae, antennal scapes, antennal funiculus, and tarsi testaceous yellow; masticatory margins of the mandibles dark brown.

**Etymology:** Patronym, in honor of Theodore Pergande, a myrmecologist who sent his extensive collections to Forel and Emery.

**Comments:** *Temnothorax pergandei* is often found in open, sparsely vegetated habitats throughout its range. With its rapid movements and constricted mesosoma, *T. pergandei* presents itself in the field as distinctly *Pheidole*-like, often foraging for small arthropods such as aphids and heteropterans during the diurnal hours (Wheeler, 1903b). Wheeler observed that workers of *T. pergandei* are territorial, showing aggression toward workers from other colonies. It often nests directly in the ground, with a very indistinct nest entrance that is typically only discoverable by following foraging workers back to it, much like other ground nesting *Temnothorax*. This is by no means the rule, though: nests have been collected under the bark of pine stumps (Smith, 1929), in rotten sticks on the ground (this study), or under moss mats and grass roots (Wheeler, 1903b). Emery (1895) states that Pergande collected *T. pergandei* in association with a *Monomorium minimum* Buckley nest, but this observation appears to be spurious; most collections of *T. pergandei* have been found apart from other ant species. Nests are small and monogynous, with 75 to 250 workers (Smith, 1929, Wesson, 1935; Heinze, Hoelldobler & Trenkle, 1995). Records of mating flights have been made from late spring in the vicinity of Austin, Texas, U.S.A. (Wheeler, 1903b), and early summer near Portal, Arizona, U.S.A. (Heinze, Hoelldobler & Trenkle, 1995). Gynes attract males with secretions from the poison gland, and typically mate with males only once in swarming flights (Heinze, Hoelldobler & Trenkle, 1995). *Temnothorax pergandei* is a highly morphologically variable species with a broad range, with a great deal of diversity in integument coloration, sculpturation, and propodeal spine length (Fig. 130 & Fig. 131). These qualities have led to a profusion of names, with the morphologically similar but geographically distant *T. floridanu*s appearing alongside the original description of *T. pergandei* (Emery, 1895). However, little sense has been made of the different forms, as there is often a great deal of intranidal and geographic variation (Creighton, 1950). Mackay (1993) synonymized *T. floridanus* under *T. pergandei* following a morphometric analysis of specimens throughout the range of both. I have found that the yellow form, formerly the subspecies *T. pergandei flavus*, is paraphyletic with respect to the typical dark form, as is the case with bicolored specimens. Most of the genetic structure that I have observed in the phylogeny of *T. pergandei* is explained by geography, with distinct Eastern, Central Great Plains, Northwestern and Southwestern clades, with the Eastern clade corresponding roughly to the Ozarks, the Appalachians and everything east of them. Whether these clades indicate distinct species is a question left to further analysis, but I cannot yet discern a distinct morphological pattern that supports separation.

### *pilicornis* group overview

**Figure 132:**
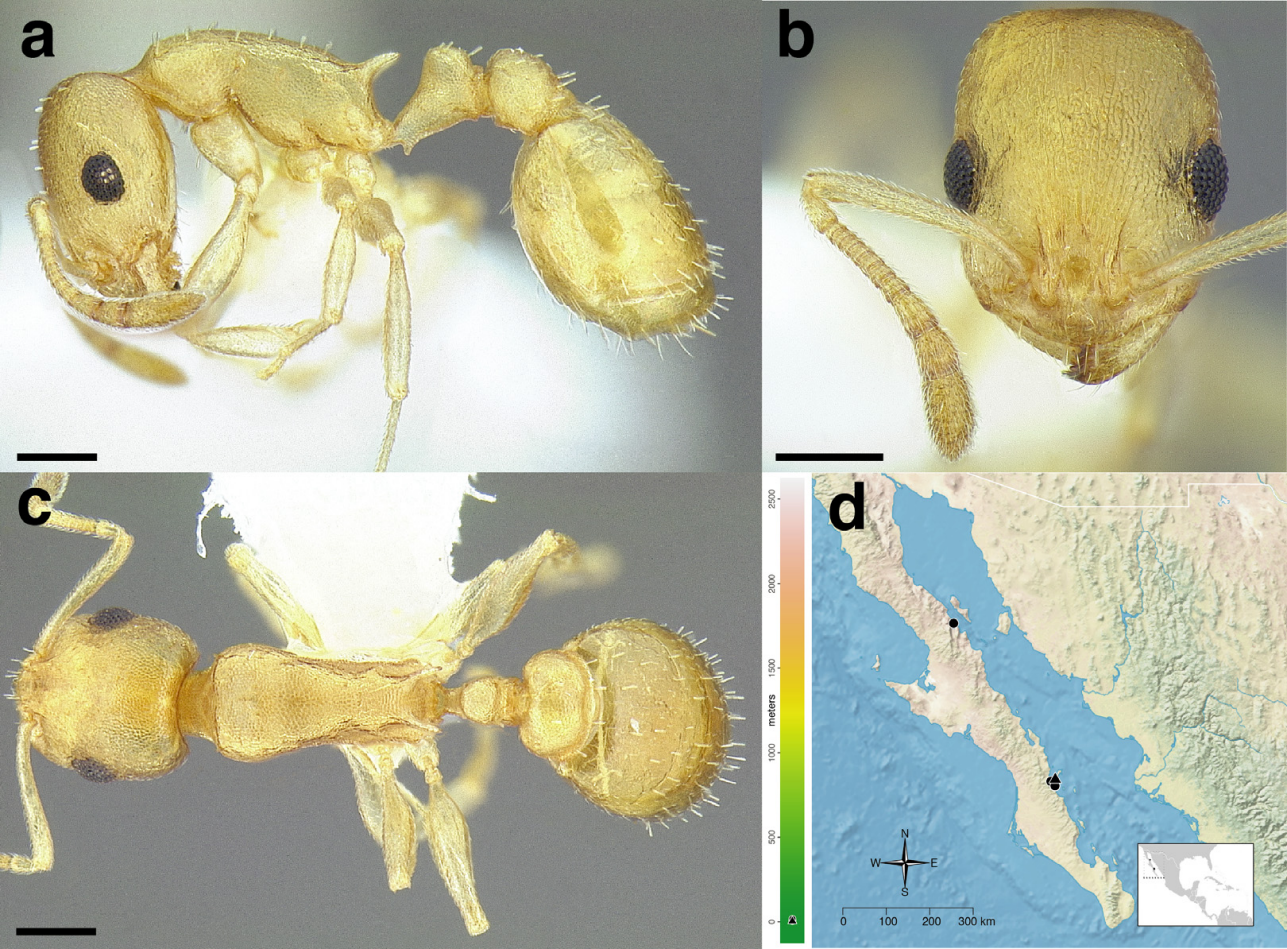
*Temnothorax pilicornis* sp. nov. holotype worker (CASENT0756186). (A) Profile view. (B) Full-face view. (C) Dorsal view. (D) Geographical and elevational distribution of specimens examined. Type localities are represented by triangles, non-type localities are represented by circles. Bounding box in inset map shows location of main map. Scale bars 0.2 mm.

This group is monotypic, with the nominal *Temnothorax pilicornis* sp. nov. being the only member. Known only from coastal habitats and offshore islands of Baja California (Fig. 132D), this species has a generalized *Temnothorax* habitus, save for the extremely broad postpetiole. It can be separated from most similar appearing species be the combination of the broad postpetiole and erect setae on the antennal scapes.

***Temnothorax pilicornis* sp. nov.**

Worker & distribution: Fig. 132.

*Leptothorax* sp. BCA-6. Johnson & Ward, 2002: 1023.

**Type material examined:** *Holotype worker:* MEXICO: Baja California Sur: Isla Carmen, S end, 25.866667°N 111.216667°W, 20 m, 18 July 1999, A.M. Boulton #245, pitfall trap (CASENT0756186, top specimen on pin) [CASC].

*Paratype worker:* same pin as holotype, 1 worker (bottom specimen on pin) [CASC].

**Non-type material examined:** MEXICO: Baja California Sur: Isla Carmen, S end, 25.866667°N 111.216667°W, 20 m, 13–18 July 1999, R. Aalbu & K. Brown, A. Boulton acc. no. RA-25, oatmeal pitfall trap, 3 workers (CASENT0756185) [CASC]; same data as previous, except: A. Boulton acc. no. RA-24, 1 worker (CASENT0756184) [LACM]; same data as previous, except: A. Boulton acc. no. RA-12, 1 worker (CASENT0756182) [MCZC]; same data as previous, except: R. Aalbu, A. Boulton acc. no. RA-15, pitfall trap, 1 worker (CASENT0756183) [USNM]. Isla Ventana, 29.000000°N 113.516667°W, 12 May - 2 July1999, F. Sanchez-Piñero#FS72, pitfall trap, 1 worker (CASENT0756196) [UCDC]. Baja California Sur: Isla Danzante, 25.783333°N 111.250000°W, 10 m, 13–18 July 1999, R. Aalbu, A. Boulton acc. no. RA-32, pitfall trap, 3 workers (CASENT0756187) [UCDC]; same data as previous, except: A. Boulton acc. no. RA-34, 1 worker missing head (CASENT0756188) [UCDC]; same data as previous, except: A. Boulton acc. no. RA-39, 3 workers, top worker missing gaster and postpetiole (CASENT0756189) [UCDC]; same data as previous, except: 18 July 1999, A.M. Boulton#244, pitfall trap, 1 worker (CASENT0756190) [UCDC]; Isla Mestiza, 25.833333°N 111.316666°W, 10 m, 13–18 July 1999, R. Aalbu, A. Boulton acc. no. RA-53, pitfall trap, 2 workers (CASENT0756192) [UCDC]; same data as previous, except: A. Boulton acc. no. RA-48, 1 worker (CASENT0756191) [UCDC]; Isla Pardo, 25.733333°N 111.216667°W, 10 m, 18 July 1999, A.M. Boulton #242, pitfall trap, 3 workers (CASENT0756194) [UCDC]; same data as previous, except: 13–18 July 1999, R. Aalbu, A. Boulton acc. no. RA-54, pitfall trap, 1 worker (CASENT0756193) [UCDC]; Isla Tijeras 25.750000°N 111.233333°W, 20 m, 13–18 July 1999, R. Aalbu, A. Boulton acc. no. RA-68 pitfall trap, 1 worker(CASENT0756195) [UCDC].

**Geographic range:** Low elevations of Baja California (Fig. 132D).

**Worker diagnosis:** *Temnothorax pilicornis* sp. nov. can be separated from all other species in the *salvini* clade by the following character combination: compound eyes large (OI ≥ 27); propodeal spines shorter than the propodeal declivity (PSI 27–29) and directed posteriorly, forming a rounded ~90° angle with the declivity; hind femora moderately to strongly incrassate (FI 282–323); petiolar node erect and subquadrate, not overhanging the caudal cylinder in profile view; postpetiole moderately broad (PWI 207–223); base of first gastral tergite sculptured; antennal scapes with dense suberect pilosity; setae on head, mesosoma, waist segments and gaster erect, short, sparse and blunt; integument light yellow.

**Similar species:** *Temnothorax anaphalantus, T. andrei, T. carinatus, T. cokendolpheri, T. goniops, T. nitens, T. pseudandrei, T. wardi*, species of the *annexus* and *silvestrii* groups. *Temnothorax pilicornis* sp. nov. can be separated from *T. andrei, T. anaphalantus, T. carinatus, T. cokendolpheri, T. nitens, T. pseudandrei* and the *annexus* group by the very broad postpetiole (PWI > 200). *Temnothorax pilicornis* sp. nov. can be further separated from the *annexus* group, as well as *T. wardi* by the entire and evenly convex anterior clypeal margin, which is emarginate in the latter two. The sculpture, petiole, and propodeal spines of *T. carinatus*, *T. cokendolpheri*, and *T. wardi* differ from *T. pilicornis* sp. nov. as well, with the former three having weak areolate-costulate sculpture on the head (generally with a medial strip of smooth sculpture), a less pedunculate petiole, and the spines reduced to weak angles. *Temnothorax pilicornis* sp. nov. can be separated from members of the *silvestrii* group by the placement of the compound eyes, which are separated from the mandibular insertions by about their total length in *T. pilicornis* sp. nov., but by more than their total length in the *silvestrii* group; the posterior margin of the head is convex in the *silvestrii* group, but flat in *T. pilicornis* sp. nov.; the anterior clypeal margin is flat in the *silvestrii* group, but convex in *T. pilicornis* sp. nov. This species differs from *T. goniops* and *T. terrigena* by the subdecument pilosity on the antennal scapes, which is decumbent in *T. goniops* and *T. terrigena*.

**Worker measurements & indices (*n* = 5):** SL = 0.423–0.490 (0.460); FRS = 0.156–0.188 (0.173); CW = 0.517–0.602 (0.561); CWb = 0.469–0.549 (0.508); PoOC = 0.222–0.245 (0.235); CL = 0.606–0.689 (0.644); EL = 0.149–0.170 (0.161); EW = 0.118–0.139 (0.125); MD = 0.111–0.157 (0.138); WL = 0.702–0.817 (0.755); SPST = 0.192–0.219 (0.206); MPST = 0.209–0.255 (0.233); PEL = 0.266–0.319 (0.291); NOL = 0.143–0.182 (0.164); NOH = 0.096–0.122 (0.105); PEH = 0.202–0.242 (0.224); PPL = 0.188–0.213 (0.199); PPH = 0.198–0.241 (0.212); PW = 0.344–0.396 (0.364); SBPA = 0.142–0.187 (0.162); SPTI = 0.192–0.245 (0.213); PEW = 0.144–0.183 (0.161); PNW = 0.144–0.183 (0.163); PPW = 0.314–0.39 (0.348); HFL = 0.451–0.542 (0.501); HFWmax = 0.126–0.142 (0.137); HFWmin = 0.042–0.049 (0.046); CS = 0.772–0.894 (0.830); ES = 0.209–0.237 (0.224); SI = 89–96 (91); OI = 26–28 (27); CI = 77–80 (79); WLI = 148–150 (149); SBI = 29–34 (32); PSI = 27–29 (27); PWI = 207–223 (216); PLI = 130–168 (147); NI = 140–175 (156); PNWI = 100–106 (101); NLI = 53–61 (56); FI = 282–323 (300).

**Worker description:** In full-face view, head subquadrate, elongate (CI 77–80). Mandibles densely, finely striate but shining and armed with five teeth: the apical-most well developed and acute, followed by a less developed preapical tooth and three equally developed smaller teeth. Anterior clypeal margin evenly convex medially. Antennal scapes short: when fully retracted, failing to reach the posterior margin of the head capsule by about the maximum width of the scape (SI 89–96). Antennae 12-segmented; antennal club of composed of three segments, with the apical-most segment longer than the preceding two in combination. Frontal carinae long, extending past the antennal toruli by about four times the maximum width of the antennal scape. Compound eyes moderately protruding past the lateral margins of the head capsule. Lateral margin of head very weakly convex, nearly flat. Posterior head margin flat but rounding evenly into the lateral margins.

In profile view, compound eyes ovular and large (OI 26–28), with 12 ommatidia in longest row. Pronotal declivity distinct, neck and anterior face of pronotum forming a ~110° angle; anterior face and dorsal face forming a rounded ~110° angle. Mesosoma very weakly convex from the pronotal declivity to the propodeal spines, nearly flat; propodeum very weakly depressed, so that the dorsal margin dips slightly anterior to the base of the propodeal spines. Promesonotal suture extending from the posterior margin of the procoxal insertion only to the mesothoracic spiracle, which is weakly developed. Metanotal groove visible as a disruption of the sculpture laterally from where it arises between the mid- and hind coxae to where it ends in the poorly developed metathoracic spiracle, which is nearly indistinguishable against the ground sculpture. Propodeal spiracle poorly developed, directed posterolaterally, and separated from the propodeal declivity by about four spiracle diameters. Propodeal spines moderately well developed and moderately long (PSI 27–29), about as long as the propodeal declivity, flared at the base, straight, directed posterodorsally, and blunt. Propodeal declivity flat, forming a rounded ~90° angle with the base of the propodeal spines. Propodeal lobes rounded and weakly developed. Metapleural gland bulla small, extending from the metacoxal insertion halfway to the propodeal spiracle. Petiole short (PLI 130–168), without tubercles anterodorsally. Subpetiolar process in the form of a moderately large, blunt tooth, with a posterior flange that extends along most of the ventral face of the petiole. Petiolar peduncle short: petiolar node covering most of the petiolar dorsum. Petiolar node robust: node transitioning evenly to the petiolar peduncle anteriorly, with a weakly concave anterior face; anterior face forming a ~100° angle with the dorsal face, which is weakly convex; dorsal face rounding evenly into the short posterior face, which forms a ~100° angle with the caudal cylinder. Postpetiole evenly rounded anteriorly, flattened dorsally, and lobed ventrally.

In dorsal view, transition between the dorsum and the anterior face of the pronotum marked by a distinct carina. Humeri developed and distinct: anterior face of the pronotum meeting the lateral face at a distinct ~90° angle; mesothoracic spiracles weakly protruding past the lateral margins of the mesosoma, visible as slight angles where the pronotum meets the mesonotum. Promesonotal suture indicated by a change in sculpture and slight change in color of the integument. Metanotal groove absent: mesonotum and propodeum completely fused and lateral margins converging evenly to the bases of the propodeal spines. Propodeal spines broadly approximated basally and diverging apically, their apices separated from each other by about their length, the negative space between them “U” shaped. Petiolar peduncle with spiracles not protruding past the lateral margins. Petiolar node campaniform: rounded anteriorly and very weakly convex posterior, nearly flat; node slightly wider than the peduncle, and evenly grading into the caudal cylinder, which is wider than the node. Postpetiole moderately broad (PWI 207–223) and campaniform, articulating with the nearly the entire anterior margin of the gaster, but leaving angulate corners of the gaster exposed on each side. Anterior margin of the postpetiole broadly convex and evenly rounds into the lateral margins, which diverge slightly to the angulate posterior corners; posterior margin broadly concave. Metafemur moderately to strongly incrassate (FI 282–323).

Sculpture: median clypeal carina present but may be difficult to distinguish from the costulate ground sculpture; extending posteriorly to the level of the antennal toruli and flanked on either side by two equally strong carinae. Lateral clypeal lobes with additional, weaker carinae; ground sculpture densely areolate-costulate. Antennal scapes shining through weak areolate ground sculpture. Cephalic dorsum densely areolate-costulate. Lateral surfaces sculptured similarly to the dorsum, but with stronger rugulae between the compound eye and the mandibular insertion. Ventral surface of head smooth and shining through weak areolate sculpture. Mesosoma with areolate sculpture on the pronotal neck. Lateral surface mesosoma densely areolate. Propodeal declivity densely areolate. Dorsal surface of pronotum with costulae over dense areolate ground sculpture; remainder of the mesosoma densely areolate, with costulae on the lateral margins and strigulae directly anterior to the base of the propodeal spines. Femora shining through weak areolate sculpture. Petiole and postpetiole uniformly areolate; petiole with a weak carina that extends from the spiracle to the caudal cylinder. Gaster very weakly areolate on the basal quarter of the first gastral tergite; otherwise smooth and shining, without spectral iridescence.

Setae: antennal scapes and funiculi with short, subdecumbent pilosity. Dorsum of the head, pronotum, waist segments, and gaster with moderately abundant, erect, blunt-tipped setae, some of which are nearly clavate. The longest setae are about half the width of the compound eye. The head dorsum bears ~28, mesosoma ~16, petiole 6, postpetiole ~12, and first gastral tergite ~34 setae. Longer, tapering setae are present on the ventral surface of the head, propleuron, procoxae, and ventral surface of the gaster. Short, sparse pubescence present over the entire body, but is difficult to detect against the lightly colored integument and dense sculpture.

Color: predominantly light yellow; masticatory margin of the mandible dark brown.

**Gyne:** Unknown.

**Male:** Unknown.

**Etymology:** Morphological, from the Latin ‘pilus’ (= hair) + ‘cornis’ (= horned), in reference to the dense suberect pilosity on the antennal scapes.

**Comments:** *Temnothorax pilicornis* sp. nov. is known only from the islands off the coast of Baja California, where it has been collected from pitfall traps. This species has large eyes and lightly colored integument, which are typical of crepuscular species in xerophytic habitats. At first glance, this species is very similar to *T. andrei* or *T. carinatus*, but the broad post-petiole and areolate-costulate dorsum of the head is an indication of its true affiliation with the *salvini* clade.

### *pulchellus* group overview

**Figure 133:**
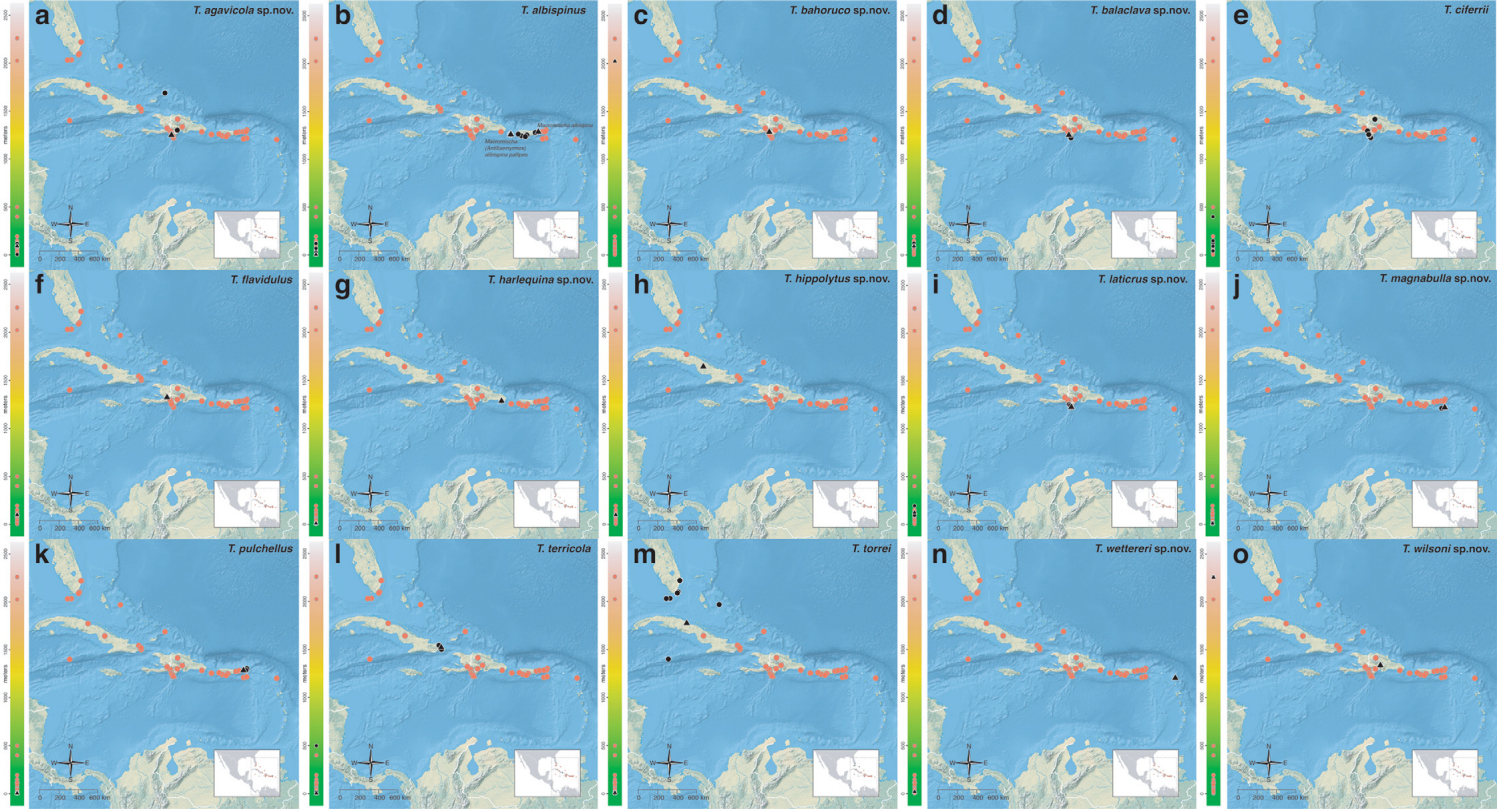
Geographical and elevational distribution of the *pulchellus* group. (A) *Temnothorax agavicola* sp. nov. (B) *T. albispinus*. (C) *T. bahoruco* sp. nov. (D) *T. balaclava* sp. nov. (E) *T. ciferrii* (F) *T. flavidulus* (G) *T. harlequina* sp. nov. (H) *T. hippolytus* sp. nov. (I) *T. laticrus* sp. nov. (J) *T. magnabulla* sp. nov. (K) *T. pulchellus* (L) *T. terricola* (M) *T. torrei* (N) *T. wettereri* sp. nov. (O) *T. wilsoni* sp. nov. Colored scale to the left of each map represents elevation in meters. Points in black represent the species named in each subfigure, while points in red represent other members of the species group. Type localities are represented by triangles; if present, types of synonyms are named; non-type localities are represented by circles. Bounding box in inset map shows location of main map.

With fifteen species (nine described as new here), the *pulchellus* group is the largest in the *salvini* clade. Primarily inhabiting the low elevations of the Caribbean islands and southern Florida, this group has a peculiar bimodal elevational distribution, inhabiting some of the highest points on the island of Hispaniola, but as of yet without any records of mid-elevation collections (Fig. 133). Nest collections of this group, while rare, suggest nesting habits similar to the *pastinifer* group, i.e., within dead wood and vegetation on or near the ground, often in leaf litter. The general habitus of the members of the *pulchellus* group is another example of the *Macromischa* syndrome, with most species having extremely arched mesosomata, often with enlarged femora, and always with broad postpetioles. These characters broadly overlap with the *pastinifer* group, *Temnothorax subditivus*, and some members of the *sallei* clade (e.g., the *sallei* and *iris* subclades sensu Prebus (2017)). Use the keys above and the ‘similar species’ sections below to find specific distinguishing characters.

***Temnothorax agavicola* sp. nov.**

**Figure 134:**
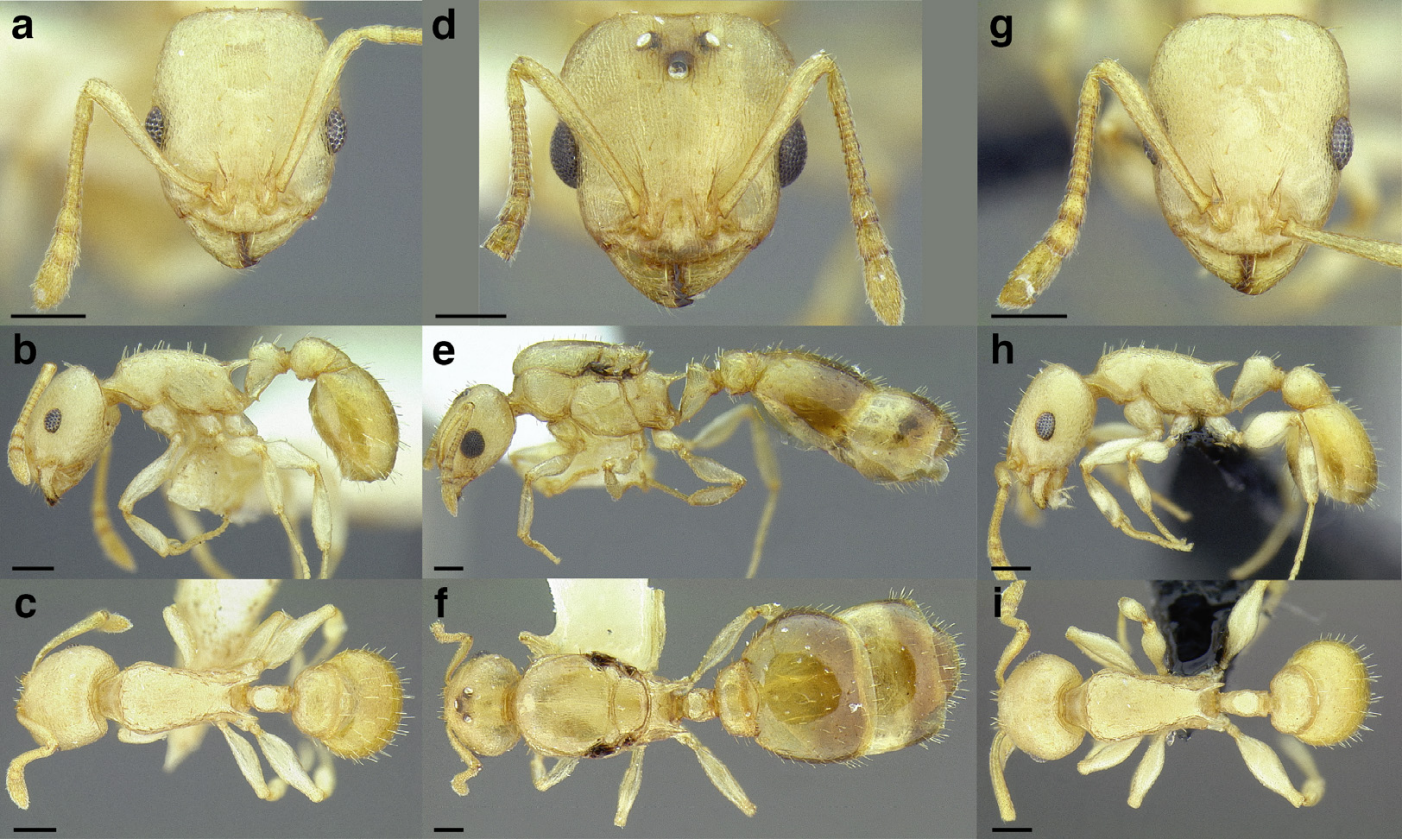
*Temnothorax agavicola* sp. nov. (A-C) Holotype worker (MCZENT00510559). (A) Full-face view. (B) Profile view. (C) Dorsal view. (D–F) Paratype gyne (MCZENT00510541). (D) Full-face view. (E) Profile view. (F) Dorsal view. (G–I) Morphological variant, Providenciales, Turks and Caicos Islands (CASENT0756096). (G) Full-face view. (H) Profile view. (I) Dorsal view. Scale bars 0.2 mm.

Distribution: Fig. 133A; worker, gyne & variability: Fig. 134.

**Type material examined:** *Holotype worker:* DOMINICAN REPUBLIC: Pedernales: Jaragua National Park 18.02571°N 71.64732°W ± 5 m, 92 m 1 April 2012, D. Lubertazzi#DL03475:001, dry forest, downed wood (MCZENT00510559) [MCZC].

*Paratype workers and gynes:* same data as holotype, except: D. Lubertazzi #DL03479:003, dry forest, under Agave, 4 workers & 1 dealate gyne (MCZENT00510560-MCZENT00510562) [MCZC]; same data as holotype, except: D. Lubertazzi#DL03474:003, dry forest, downed wood, 1 dealate gyne (MCZENT00510541) [MCZC].

**Non-type material examined:** DOMINICAN REPUBLIC: Barahona: 28 km N Barahona, 165 m, 18 August 1988, M.A. Ivie, T.K. Philips & K.A. Johnson, ex rotten agave, 1 worker (CASENT0758360) [USNM]. TURKS AND CAICOS ISLANDS: Providenciales: Old Airport Road, 21.775°N 72.261°W, 5 m, 9 July 2010, J.K. Wetterer #JKW-2010-154, tropical scrub by store, 1 worker (CASENT0756096) [MCZC] 1 worker (CASENT0758672) [UCDC] 1 worker (CASENT0758819) [USNM].

**Geographic range:** Low elevations of Hispaniola and the Turks and Caicos Islands (Fig. 133A).

**Worker diagnosis:** *Temnothorax agavicola* sp. nov. can be separated from all other species in the *salvini* clade by the following character combination: head in full face view with posterior margin weakly concave; mesosoma moderately compact; in profile view, dorsum of mesosoma weakly convex; metanotal groove not impressed; propodeum not depressed below the level of the promesonotum; propodeal spines about as long as the propodeal declivity, directed posteriorly, and straight; hind femora moderately incrassate; in dorsal view, propodeal spines broadly approximated basally, the negative space between them “U” shaped; in dorsal view, apex of petiolar node narrower than the caudal cylinder of the petiole; petiolar node flat dorsally; petiolar node with four erect setae dorsally; postpetiole very broad: greater than 2.6 times the width of the petiole; dorsum of head uniformly areolate; setae on head, mesosoma, waist segments and gaster erect, moderately long, sparse and blunt (never long and tapering); integument light yellow.

**Similar species:** *Temnothorax albispinus, T. laticrus* sp. nov., *T. torrei, T. wettereri* sp. nov., and members of the *pastinifer* group. *Temnothorax agavicola* sp. nov. can be separated from all members of the *pastinifer* group by the apically narrow petiolar node, which not squamiform and always narrower than the caudal cylinder of the petiole in dorsal view. The propodeal spines, which are longer than the propodeal declivity and relatively narrow hind femora will distinguish *T. agavicola* sp. nov. from *T. laticrus* sp. nov., which has propodeal spines that are shorter than the declivity and extremely incrassate hind femora. The erect setae count of four on the dorsum of the petiolar node differentiates *T. agavicola* sp. nov. from *T. torrei*, which has only two along the posterior margin. The weakly concave posterior margin of the head of *T. agavicola* sp. nov. contrasts with *T. albispinus*, which has a flat to weakly convex posterior head margin. The body of *T. wettereri* sp. nov. is mostly devoid of erect setae, whereas *T. agavicola* sp. nov. has more than ten erect setae on the dorsum of the mesosoma.

**Worker measurements & indices (*n* = 9):** SL = 0.474–0.500 (0.486); FRS = 0.169–0.199 (0.182); CW = 0.516–0.588 (0.552); CWb = 0.478–0.544 (0.509); PoOC = 0.248–0.267 (0.260); CL = 0.593–0.655 (0.626); EL = 0.121–0.145 (0.132); EW = 0.093–0.105 (0.101); MD = 0.136–0.159 (0.148); WL = 0.663–0.750 (0.700); SPST = 0.208–0.257 (0.220); MPST = 0.214–0.234 (0.224); PEL = 0.267–0.305 (0.281); NOL = 0.153–0.204 (0.180); NOH = 0.099–0.118 (0.105); PEH = 0.189–0.214 (0.196); PPL = 0.189–0.229 (0.208); PPH = 0.239–0.255 (0.248); PW = 0.343–0.402 (0.371); SBPA = 0.137–0.169 (0.154); SPTI = 0.176–0.220 (0.196); PEW = 0.127–0.146 (0.137); PNW = 0.117–0.149 (0.133); PPW = 0.358–0.392 (0.373); HFL = 0.444–0.543 (0.491); HFWmax = 0.118–0.162 (0.141); HFWmin = 0.044–0.053 (0.050); CS = 0.775–0.871 (0.822); ES = 0.170–0.198 (0.183); SI = 91–99 (95); OI = 21–23 (22); CI = 80–83 (81); WLI = 133–141 (137); SBI = 28–31 (30); PSI = 28–39 (32); PWI = 249–295 (272); PLI = 118–150 (136); NI = 147–191 (171); PNWI = 92–102 (96); NLI = 53–76 (64); FI = 236–361 (287).

**Worker description:** In full-face view, head subquadrate, longer than broad (CI 80–83). Mandibles weakly striate, shining, and armed with five small teeth: the apical-most well developed, followed by a smaller preapical tooth, which is followed by three equally developed smaller teeth. Anterior clypeal margin entire and evenly rounded. Antennal scapes moderately long: when fully retracted, just reaching the posterior margin of the head capsule (SI 91–99). Antennae 12-segmented; antennal club of three segments, with the apical-most segment slightly longer than the preceding two in combination. Frontal carinae moderately long, extending past the antennal toruli by about two times the maximum width of the antennal scape. Compound eyes moderately protruding past the lateral margins of the head capsule. Lateral margin of head weakly convex, nearly flat, and forming a continuous arc from the posterior of the head to the mandibular insertions. Posterior head margin weakly concave but transitioning into the lateral margin through a rounded corner, giving the head a boxy appearance.

In profile view, compound eyes ovular, moderately large (OI 21–23), with 9 ommatidia in longest row. Pronotal declivity distinct, neck and anterior face of pronotum forming a ~110° angle; anterior and dorsal faces of pronotum forming a ~130° angle. Mesosoma very weakly convex from the pronotal declivity to the propodeal spines, nearly flat. Promesonotal suture extending from the posterior margin of the procoxal insertion only to the mesothoracic spiracle, which is well developed. Metanotal groove visible as a disruption of the sculpture laterally from where it arises between the mid- and hind coxae to where it ends in the poorly developed metathoracic spiracle, which is nearly indistinguishable against the ground sculpture. Propodeal spiracle poorly developed, directed posterolaterally, and separated from the propodeal declivity by about two spiracle diameters. Propodeal spines well developed and moderately long (PSI 28–39), about as long as the propodeal declivity, tapering evenly from the base, straight, and acute. Propodeal declivity weakly concave, forming a ~80° angle with the base of the propodeal spines. Propodeal lobes rounded and weakly developed. Metapleural gland bulla small, extending from the metacoxal insertion halfway to the propodeal spiracle. Petiole short (PLI 118–150), without tubercles anterodorsally. Subpetiolar process in the form of a small, acute tooth. Petiolar peduncle short: petiolar node covering most of the petiolar dorsum. Petiolar node robust: grading evenly into the petiolar peduncle anteriorly, resulting in a slightly concave anterior node face; anterior margin forming a ~100° angle with the dorsal surface, which is weakly convex; dorsal face rounding evenly into the short posterior face, which forms a ~110° angle with the caudal cylinder. Postpetiole evenly rounded anteriorly, flattened dorsally, and weakly lobed ventrally.

In dorsal view, humeri weakly developed: evenly rounded and slightly wider than the rest of the mesosoma; mesothoracic spiracles visible as a very slight angle where the pronotum meets the mesonotum. Metanotal groove absent: mesonotum and propodeum completely fused and converging evenly to the bases of the propodeal spines. Propodeal spines broadly approximated basally, but diverging apically, their apices separated from each other by about their length; negative space between them “U” shaped. Petiolar peduncle with spiracles slightly protruding past the lateral margins, and subtly constricted anterior to them. Petiolar node evenly ovular; node slightly wider than the peduncle, and evenly grading into the caudal cylinder, which is slightly wider than the node. Postpetiole very broad (PWI 249–295) and campaniform, articulating with the entire anterior margin of the gaster. Anterior margin of the postpetiole convex, evenly rounding into the lateral margins, which diverge to the angulate posterior corners; posterior margin broadly concave. Metafemur weakly to strongly incrassate (FI 236–361).

Sculpture: median clypeal carina present, extending posteriorly to the level of the antennal toruli, and flanked on either side by two equally strong carinae. Lateral clypeal lobes with additional, weaker carinae; ground sculpture smooth and shining. Antennal scapes shining through weak areolate ground sculpture. Cephalic dorsum evenly areolate, with the areolae arranged into longitudinal rows separated by costulae; ventral surface of head weakly areolate and shining. Pronotal neck areolate. Lateral surfaces of the pronotum shining, with weak rugulae. Mesopleurae and lateral surface of propodeum areolate. Propodeal declivity shining through weak areolate sculpture. Dorsal surface of mesosoma with costulae over areolate ground sculpture. Femora shining, with weak areolate sculpture on the distal third. Petiole smooth and shining ventrally, with weak areolate sculpture on all other surfaces. Postpetiole with weak areolate sculpture becoming stronger on the posterior third. Gaster smooth and shining, without spectral iridescence.

Setae: antennal scapes and funiculi with short, adpressed pilosity. Dorsum of head, pronotum, waist segments and gaster with moderately abundant, erect, blunt-tipped setae, the longest of which are about the length of the compound eye. The head bears ~28, mesosoma ~24, petiole 4, postpetiole ~18, and first gastral tergite ~48 setae. Short, sparse pubescence present over the entire body, but difficult to detect against the lightly colored integument.

Color: predominantly light yellow, with the masticatory margin of the mandibles darker.

**Gyne measurements & indices (*n* = 2):** SL = 0.567–0.573 (0.570); FRS = 0.229–0.249 (0.239); CW = 0.746–0.764 (0.755); CWb = 0.703–0.708 (0.706); PoOC = 0.295–0.307 (0.301); CL = 0.728–0.740 (0.734); EL = 0.208–0.223 (0.216); EW = 0.173–0.188 (0.181); MD = 0.160–0.166 (0.163); WL = 1.157–1.196 (1.177); SPST = 0.283–0.308 (0.296); MPST = 0.285–0.313 (0.299); PEL = 0.405–0.413 (0.409); NOL = 0.169–0.185 (0.177); NOH = 0.146–0.152 (0.149); PEH = 0.279–0.302 (0.291); PPL = 0.227–0.234 (0.231); PPH = 0.319–0.341 (0.330); PW = 0.703–0.720 (0.712); SBPA = 0.314–0.324 (0.319); SPTI = 0.32; PEW = 0.192–0.197 (0.195); PNW = 0.228–0.234 (0.231); PPW = 0.541–0.550 (0.546); HFL = 0.631–0.651 (0.641); HFWmax = 0.148–0.150 (0.149); HFWmin = 0.058–0.059 (0.059); CS = 1.072–1.073 (1.073); ES = 0.295–0.317 (0.306); SI = 81; OI = 27–30 (29); CI = 95–97 (96); WLI = 165–169 (167); SBI = 45–46 (45); PSI = 24–26 (25); PWI = 279–282 (280); PLI = 173–182 (178); NI = 111–127 (119); PNWI = 119–119 (119); NLI = 41–46 (43); FI = 254–255 (255).

**Gyne description:** In full-face view, head subquadrate, slightly longer than broad (CI 95–97). Mandibles weakly striate, shining, and armed with five small teeth: the apical-most well developed, followed by a less developed preapical tooth and three equally developed smaller teeth. Anterior clypeal margin flat medially. Antennal scapes moderately long: when fully retracted, just reaching the posterior margin of the head capsule (SI 81). Antennae 12-segmented; antennal club of three segments, with the apical-most segment slightly longer than the preceding two in combination. Frontal carinae very short, extending past the antennal toruli by two times the maximum width of the antennal scape. Compound eyes moderately protruding past the lateral margins of the head capsule. Lateral margins of head converging evenly from the expanded temples to the mandibular insertions. Posterior head margin concave medially, with temples rounding evenly into the lateral margins.

In profile view, compound eyes large (OI 27–30), ovular, with 16 ommatidia in longest row. Mesoscutum rounded evenly anteriorly, covering the dorsal surface of the pronotum, and flat dorsally. Mesoscutellum slightly depressed below the level of the mesoscutum, not overhanging the metanotum. Propodeal spiracle well developed, directed anteroposteriorly, and separated from the propodeal declivity by about four spiracle diameters. Propodeal spines stout and well developed, but short (PSI 24–26), about two thirds as long as the propodeal declivity, tapering evenly from the base, directed posteriorly, upturned, and blunt. Propodeal declivity weakly concave and forming a ~90° angle with the base of the propodeal spines. Propodeal lobes weakly developed, with the dorsal margin slightly angulate. Petiole moderately long (PLI 173–182), without tubercles anterodorsally. Subpetiolar process in the form of a small, very acute tooth, which grades evenly into the ventral margin of the petiole posteriorly; ventral margin of the petiole slightly bulging. Petiolar peduncle short: petiolar node covering most of the petiolar dorsum. Petiolar node erect: transition between peduncle and node evenly rounded, resulting in a very slightly concave anterior node face; anterior face forming a sharp ~100° angle with the dorsal face, which is short; dorsal face rounding evenly into the posterior face, which forms a ~140° angle with the caudal cylinder. Postpetiole evenly rounded anterodorsally, forming a slight convexity before it transitions into the flattened dorsal face; ventral surface lobed.

In dorsal view, mesoscutum covering pronotum anteriorly, but humeri visible laterally as rounded sclerites. Propodeal spines diverging apically, their apices separated from each other by about two times their length. Petiolar peduncle with spiracles slightly protruding past the lateral margins; node emarginated anterodorsally, dorsal face transitioning into the lateral faces with a rounded ~90° angle, which taper into the indistinct posterior face. Petiolar node slightly wider than the peduncle, and evenly grading into the caudal cylinder, which narrower than the node. Postpetiole very broad (PWI 279–282), anteroposteriorly compressed, and campaniform, articulating with most of the anterior margin of the gaster, leaving small, weakly angulate margins on each side exposed. Anterior margin of the postpetiole weakly convex, with corners evenly rounding into the lateral margins, which evenly diverge to the angulate posterior corners; posterior margin broadly concave. Metafemur moderately incrassate (FI 254–255).

Sculpture: median clypeal carina present, extending from the anterior margin to the level of the antennal toruli, and flanked two equally strong carinae. Lateral clypeal lobes with additional weaker carinae; ground sculpture smooth and shining. Antennal scapes shining through very weakly areolate ground sculpture. Cephalic dorsum shining with costulae over weak areolate ground sculpture; ventral surface of head shining through weak, indistinct areolate sculpture. Pronotal neck areolate. Pronotum with costulae over weak areolate ground sculpture. Anepisternum and katepisternum shining through weak areolate ground sculpture. Propodeum with weak areolae and costulae laterally; declivity areolate. Mesoscutum with weak costulae over weak areolate ground sculpture. Femora smooth and shining, with traces weak areolate sculpture distally. Peduncle of petiole smooth and shining ventrally, with shallow areolate sculpture on all other surfaces. Postpetiole weakly areolate. Gaster smooth and shining, without spectral iridescence. Surface of the first gastral sternite smooth and shining.

Setae: antennal scapes and funiculi with short, adpressed pilosity. Dorsum of head, pronotum, waist segments and gaster with moderately abundant, erect, blunt-tipped setae, the longest of which are about a third of the width of the compound eye. Short, sparse pubescence present over the entire body, but difficult to detect against the lightly colored integument.

Color: predominantly light yellow, with the masticatory margin of the mandibles and wing bases darker.

**Male:** Unknown.

**Etymology:** Behavioral, from *Agave* (plant genus) + ‘colere’ (dweller) from Latin, in reference to two collections of this species being made from dead stalks of *Agave* spp.

**Comments:** *Temnothorax agavicola* sp. nov. is known from three collections from southern Hispaniola, which were made from dead agave stalks and downed wood in a dry forest habitat. Other details of the biology of this species are unknown but are probably similar to other members of the terricolous pan-Caribbean *pulchellus* group. The specimens from the Turks and Caicos Islands vary morphologically from the Hispaniola material that I have examined; with more incrassate femora (the widest point of the hind femur 3 to 3.6 times as wide as the narrowest point vs. 2.4 to 3 in the Hispaniolan populations), overall larger body size (WL 0.73 to 0.75 mm vs. 0.66 to 0.69 mm), and more posteriorly directed propodeal spines, this population may prove to be different species (Figs. 134G–134I). A phylogenetic analysis provides evidence that these two populations are sister to each other (Prebus, in prep.).

*Temnothorax albispinus* (Wheeler, 1908)

**Figure 135:**
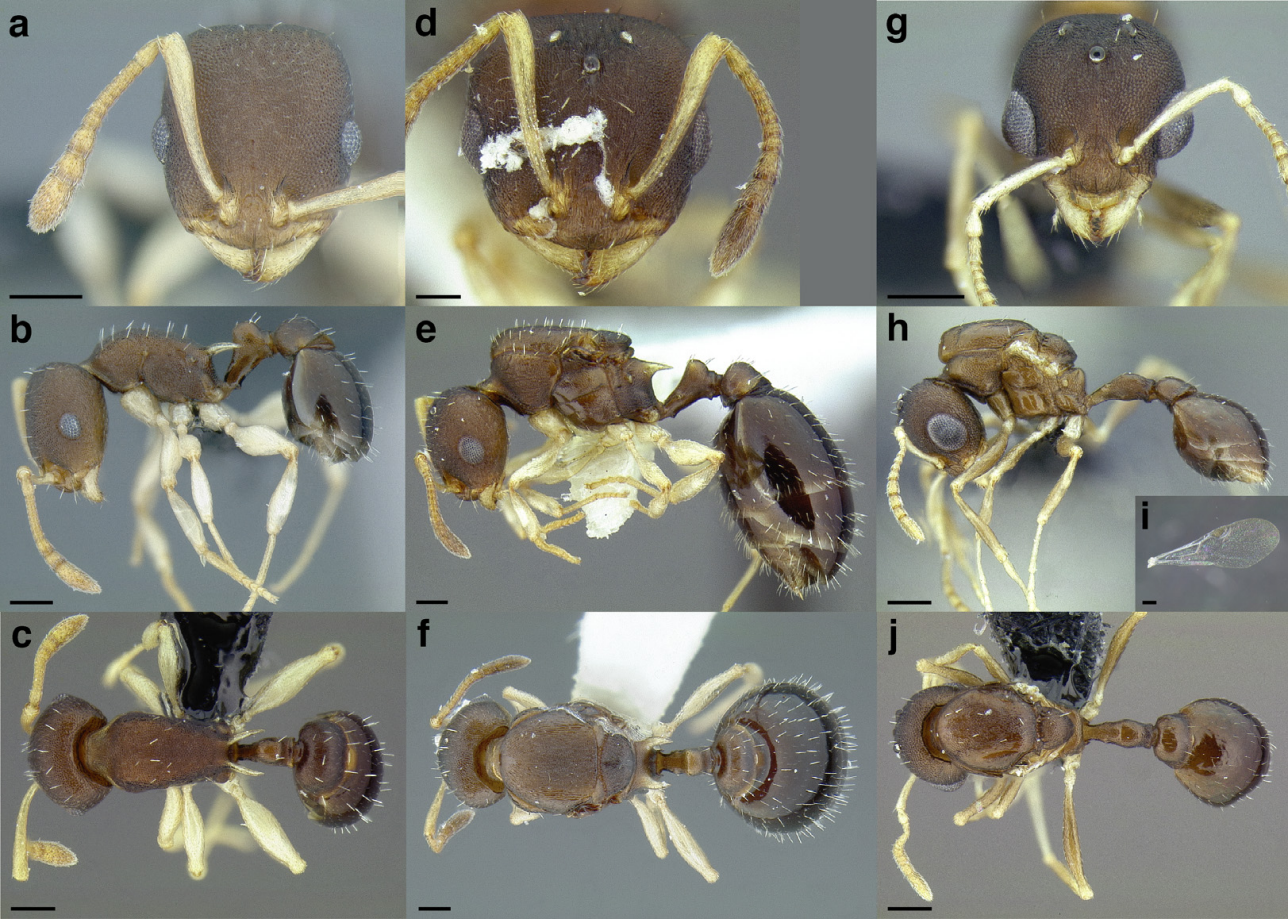
*Temnothorax albispinus*. (A–C) Worker (CASENT0756097). (A) Full-face view. (B) Profile view. (C) Dorsal view. (D–F) Gyne (MCZENT00505431). (D) Full-face view. (E) Profile view. (F) Dorsal view. (G–J) Male (CASENT0756098). (G) Full-face view. (H) Profile view. (I) Forewing. (J) Dorsal view. Scale bars 0.2 mm.

**Figure 136:**
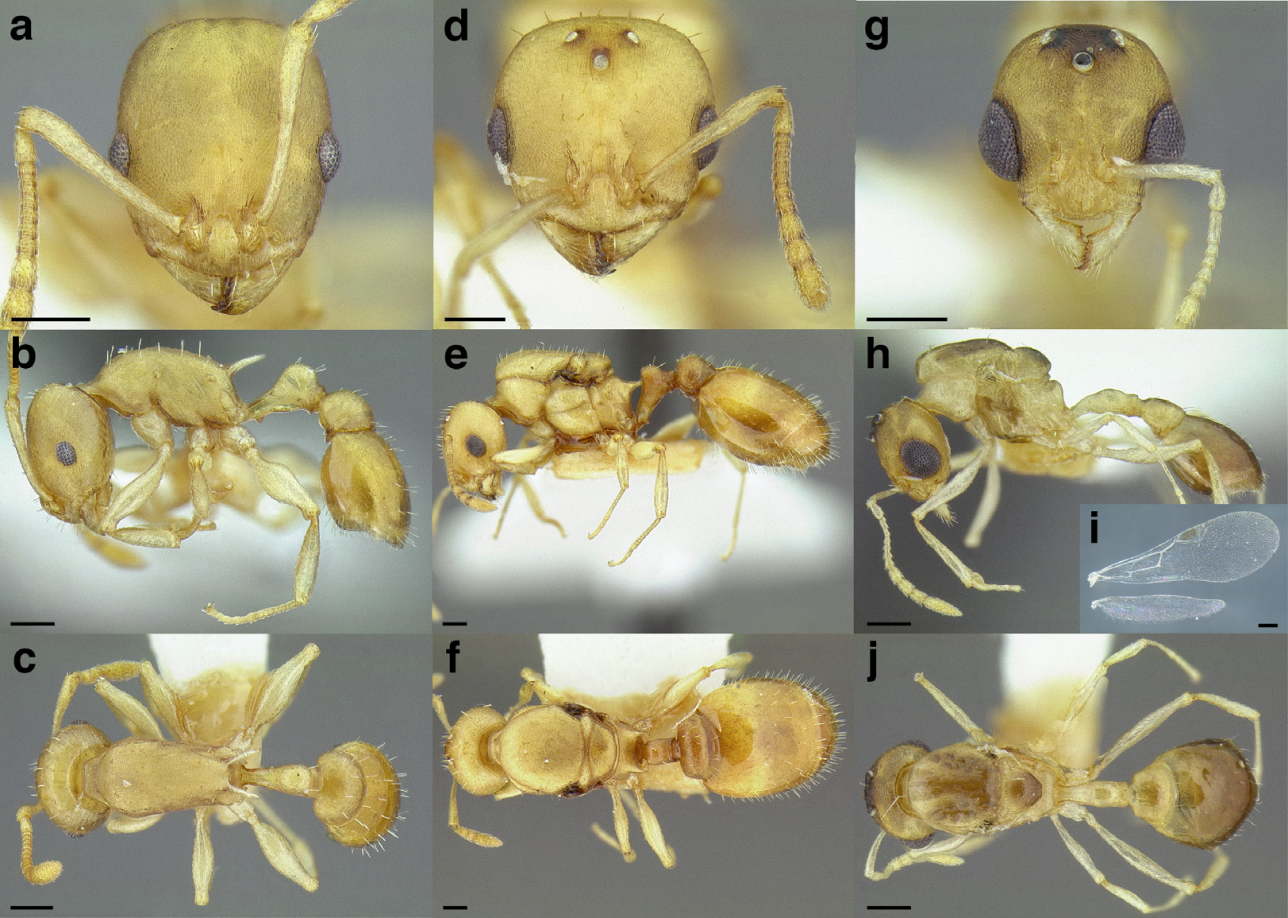
Light form of *Temnothorax albispinus*. (A–C) Worker (LACMENT323245). (A) Full-face view. (B) Profile view. (C) Dorsal view. (D–F) Gyne (LACMENT323245). (D) Full-face view. (E) Profile view. (F) Dorsal view. (G–J) Male (LACMENT323246). (G) Full-face view. (H) Profile view. (I) Wings. (J) Dorsal view. Scale bars 0.2 mm.

Distribution: Fig. 133B; worker, gyne & male: Fig. 135; variability: Fig. 136.

*Macromischa albispina* Wheeler, 1908: 139, pl. 11, figs. 3, 4. Syntype workers and gyne. U.S.A., Puerto Rico. One syntype worker here designated **lectotype**.

*Macromischa (Antillaemyrmex) albispina* (Wheeler): Mann, 1920: 408. First combination in *Macromischa (Antillaemyrmex)*.

*Macromischa (Antillaemyrmex) albispina* var. *pallipes* Mann, 1920: 424. *Nomen nudum*.

*Antillaemyrmex albispina* (Wheeler): Wheeler, 1931: 27. First combination in *Antillaemyrmex*.

*Antillaemyrmex albispina* ssp. *pallipes* (Mann): Wheeler, 1931: 27. Syntype workers and gyne; junior synonym of *Temnothorax albispinus* by Baroni Urbani, 1978: 413. One syntype worker here designated **lectotype**.

*Leptothorax albispina* (Wheeler): Baroni Urbani, 1978: 413. First combination in *Leptothorax*.

*Temnothorax albispinus* (Wheeler): Bolton, 2003: 271. First combination in *Temnothorax*.

**Type material examined:** *Lectotype worker of* Macromischa albispina: U.S.A.: Puerto Rico: Culebra Island, W.M. Wheeler, TYPE No. A.M.N.H., specimen furthest from pin (images of M.C.Z. Type 1-3 21013 examined on the MCZ Type Database website) [MCZC].

*Lectotype worker of* Macromischa (Antillaemyrmex) alibispina pallipe*s:* U.S.A.: Puerto Rico: Isla de Mona: Mona Passage, top specimen (images of MCZ Type 1-2 21014 syntype worker examined on the MCZ Type Database website) [MCZC].

**Non-type material examined:** U.S.A.: Puerto Rico: Coamo Springs, 1 worker, 1 dealate gyne (LACMENT323175) [LACM]; Guánica Forest 17.960000°N 66.870000°W, 28 August 2000, J.A. Torres, ex sifted leaf litter, 1 dealate gyne & 2 workers (LACMENT323245) [LACM]; same data as previous, except: 1 January 2000 J.A. Torres, ex sifted leaf litter, 4 workers (LACMENT323247) [LACM]; Guánica Forest, 17.840000°N 66.860000°W, 14 June 1997, M. Canals, lite trap, 1 male (LACMENT323241) [LACM] 1 male (LACMENT323242) [LACM] 1 male (LACMENT323246) [LACM]; 17.972000°N 66.868000°W, 120 m, 20 September 1998, R.R. Snelling#98-239, #1998-239, subtropical deciduous forest, ex rotten stick in leaf litter, 2 workers (LACMENT323243) [LACM]; 17.966667°N 66.883333°W, 0–50 m, 30 May 1993, J. Longino#JTL3493-s, dry forest, sifted leaf litter sample, taken from moist litter in ravine, 1 worker (LACMENT002310) [LACM] 2 workers (LACMENT002311) [UCDC]; same data as previous, except: J. Longino#JTL3494-s, dry forest, strays from first site, 1 worker (LACMENT002269) [LACM]. Guánica Forest, 7 June 1991, J. Torres#592, 2 workers, 1 dealate gyne (LACMENT323244) Isla de Mona: 30 June 1982, J.A. Torres#65, 3 workers (LACMENT323169) [LACM]; Maygüez: Isla de Mona, 4 July 1982, J.A. Torres#255, 2 workers (LACMENT323170) [LACM]; Isla de Mona, 5 min E of antenna, 18.100000°N 67.927000°W, 3 June 2006, J.K. Wetterer#272, 2 workers (MCZENT00505426) 2 workers (MCZENT00505427) [MCZC]; Isla de Mona, 50 min E of antenna, 18.090000°N 67.910000°W, 3 June 2006, J.K. Wetterer#282, 1 dealate gyne (MCZENT00505431) [MCZC]; Isla de Mona, Los Cerezos, 18.089000°N 67.900000°W, 3 June 2006, James K. Wetterer#277, forest, 1 worker (CASENT0756097) [MMPC] 1 male (CASENT0756098) [MMPC]; Isla de Mona, near antenna, 18.910000°N 67.936000°W, 40 m, 2 June 2006, J.K. Wetterer#JKW-2006-250, 1 worker (CASENT0758674) [UCDC]; Isla de Mona, 21-26 February 2014, 3 workers (CASENT0758358) [USNM]; Isla de Mona, 11-31 August 1944, Harry A. Beatty, 1 worker (CASENT0758359) [USNM]; Cayo Luis Peña, 10 Sept 1982, J.A. Torres#51b, 3 workers (LACMENT323171) [LACM]; Isla Piñeras, Humacao 11 March 1982, J.A. Torres#109, 3 workers (LACMENT323172) [LACM]; Cayo Ratones, 17 July 1982, J.A. Torres#252, 1 worker (LACMENT323173) [LACM]; Caja de Muertos, 27 September 1980, coll. J.A. Torres#51, 3 workers (LACMENT323174) [LACM] 3 workers (CASENT0759975) [FSCA].

**Geographic range:** Low elevations of Puerto Rico and its small outlying islands (Fig. 133B).

**Worker diagnosis:** *Temnothorax albispinus* can be separated from all other species in the *salvini* clade by the following character combination: head in full face view with posterior margin flat to slightly convex; mesosoma compact; in profile view, dorsum of mesosoma evenly convex; metanotal groove not impressed; propodeum not depressed below the level of the promesonotum; propodeum bearing standing setae dorsally; propodeal spines about as long as the propodeal declivity, directed posteriorly, and straight to downcurved; in dorsal view, propodeal spines broadly approximated basally, the negative space between them “U” shaped; hind femora moderately to strongly incrassate; petiolar node rounded dorsally; in dorsal view, apex of petiolar node narrower or slightly wider than the caudal cylinder of the petiole; petiolar node with four erect setae dorsally; postpetiole very broad: greater than 2.5 times the width of the petiole; median clypeal carina well-defined; dorsum of head uniformly areolate; mesosoma bearing more than ten erect setae dorsally; setae on head, mesosoma, waist segments and gaster erect, moderately long, sparse and blunt (never long and tapering); integument variously colored: either predominantly dark brown, with light yellow antennal funiculus, tibiae, tarsi, and propodeal spines, or femora and antennal scapes yellow in addition to the preceding body parts, or uniformly yellow.

**Similar species:** *Temnothorax agavicola* sp. nov., *T. laticrus* sp. nov., *T. torrei, T. wettereri* sp. nov., and members of the *pastinifer* group. *Temnothorax albispinus* can be separated from most members of the *pastinifer* group, by the petiolar node, which not squamiform and relatively narrow in dorsal view: 0.9 to 1.1 times the width of the caudal cylinder of the petiole in *T. albispinus* vs. greater than 1.3 times in the *pastinifer* group. The petiolar node shape, in addition to the long propodeal spines and relatively narrow hind femora will distinguish *T. albispinus* from *T. laticrus* sp. nov. The erect setae count of four on the dorsum of the petiolar node differentiates *T. albispinus* from *T. torrei*, which has only two along the posterior margin. The flat to weakly convex posterior margin of the head of *T. albispinus* contrasts with *T. agavicola* sp. nov., which has a weakly concave posterior head margin. The body of *T. wettereri* sp. nov. is mostly devoid of erect setae, whereas *T. albispinus* has more than ten erect setae on the dorsum of the mesosoma.

**Worker measurements & indices (*n* = 28):** SL = 0.432–0.530 (0.487); FRS = 0.167–0.216 (0.190); CW = 0.518–0.641 (0.578); CWb = 0.456–0.590 (0.525); PoOC = 0.233–0.294 (0.265); CL = 0.563–0.694 (0.637); EL = 0.120–0.156 (0.139); EW = 0.091–0.116 (0.103); MD = 0.120–0.179 (0.147); WL = 0.608–0.763 (0.691); SPST = 0.190–0.276 (0.245); MPST = 0.184–0.250 (0.224); PEL = 0.259–0.350 (0.317); NOL = 0.161–0.231 (0.195); NOH = 0.093–0.147 (0.120); PEH = 0.172–0.238 (0.214); PPL = 0.171–0.240 (0.208); PPH = 0.209–0.297 (0.246); PW = 0.332–0.416 (0.386); SBPA = 0.125–0.180 (0.154); SPTI = 0.171–0.259 (0.218); PEW = 0.119–0.169 (0.146); PNW = 0.116–0.176 (0.148); PPW = 0.334–0.434 (0.385); HFL = 0.431–0.534 (0.492); HFWmax = 0.120–0.157 (0.141); HFWmin = 0.044–0.058 (0.050); CS = 0.738–0.937 (0.844); ES = 0.169–0.212 (0.191); SI = 86–97 (93); OI = 22–24 (23); CI = 79–87 (82); WLI = 123–138 (132); SBI = 25–33 (29); PSI = 31–39 (36); PWI = 247–281 (265); PLI = 121–178 (153); NI = 141–216 (164); PNWI = 85–117 (102); NLI = 56–78 (62); FI = 245–320 (283).

**Worker description:** In full–face view, head subquadrate, longer than broad (CI 79–87). Mandibles weakly striate, shining, and armed with five teeth: the apical-most well developed, followed by a four roughly equally developed smaller teeth. Anterior clypeal margin entire to very slightly concave medially. Antennal scapes moderately long: when fully retracted, just reaching the posterior margin of the head capsule (SI 86–97). Antennae 12-segmented; antennal club of three segments, with the apical-most segment longer than the preceding two in combination. Frontal carinae very short, extending past the antennal toruli by the maximum width of the antennal scape. Compound eyes moderately protruding past the lateral margins of the head capsule. Lateral margin of head weakly convex, converging evenly to the mandibular insertions. Posterior head margin flat to very slightly convex, rounding evenly into the lateral margins.

In profile view, compound eyes ovular and moderately large (OI 22–24), with 11 ommatidia in longest row. Pronotal declivity indistinct, neck and anterior face of pronotum forming a rounded ~120° angle. Mesosoma evenly, but weakly, convex dorsally from where it joins the pronotal neck to the propodeal spines. Promesonotal suture extending from the posterior margin of the procoxal insertion only to the mesothoracic spiracle, which is well developed. Metanotal groove nearly entirely absent: only visible as a faint disruption in the surface sculpture between meso- and metacoxal insertions to the minute metathoracic spiracle, which is nearly indistinguishable against the ground sculpture. Propodeal spiracle well developed, directed posterolaterally, and separated from the propodeal declivity by about three spiracle diameters. Propodeal spines well developed and moderately long (PSI 31–39), about as long as the propodeal declivity, tapering evenly from the base, evenly downcurved, and acute. Propodeal declivity straight and flat, forming a rounded ~100° angle with the base of the propodeal spines. Propodeal lobes rounded and very weakly developed. Metapleural gland bulla small, extending from the metacoxal insertion halfway to the propodeal spiracle. Petiole moderately long (PLI 121–178), without tubercles anterodorsally. Subpetiolar process in the form of a weakly developed, blunt tooth, which grades evenly into the ventral margin of the petiole posteriorly. Petiolar peduncle short, comprising about one third of the total length of the petiole. Petiolar node robust and erect: transition between peduncle and node marked by a rounded angle of ~120°, resulting in a strongly concave anterior node face; anterior face forming a rounded ~90° angle with the dorsal face, which is evenly convex; dorsal face rounding evenly into the posterior face, which is straight and forms a ~100° angle with the caudal cylinder. Postpetiole evenly rounded anterodorsally, before flattening posterodorsally; weakly lobed ventrally.

In dorsal view, humeri weakly developed: rounded and only slightly wider than the rest of the mesosoma; mesothoracic spiracles not protruding past the lateral margins of the mesosoma. Metanotal groove absent: mesonotum and propodeum completely fused and converging evenly to the bases of the propodeal spines. Propodeal spines broadly approximated basally and diverging apically, their apices separated from each other by slightly more than their length; negative space between them “U” shaped. Petiolar peduncle with spiracles slightly protruding past the lateral margins, the peduncle subtly constricted anterior to them. Petiolar node campaniform: rounded anteriorly, flatter posteriorly, slightly wider than the peduncle, and evenly grading into the caudal cylinder, which is slightly wider than the node. Postpetiole very broad (PWI 247–281) and campaniform, articulating with the entire anterior margin of the gaster. Anterior margin of the postpetiole broadly convex, with corners evenly rounding into the lateral margins, which evenly diverge to the angulate posterior corners; posterior margin broadly concave. Metafemur moderately to strongly incrassate (FI 245–320).

Sculpture: median clypeal carina present, flanked by additional faint carinae over weakly areolate ground sculpture. Antennal scapes weakly areolate and dull. Cephalic dorsum and lateral surfaces of head densely areolate; ventral surface shining through weaker areolate sculpture. Mesosoma with uniformly areolate sculpture, with the areolae subtly arranged into longitudinal rows on the dorsal faces of the pronotum, meso- and metapleurae; area directly anterior to the propodeal spiracle, as well as the area between the propodeal spiracle and base of the propodeal spines shining through weaker sculpture. Femora shining through weak areolate sculpture. Petiole smooth and shining ventrally, with shallow areolate sculpture surrounding the base of the petiolar node, which is mostly smooth and shining dorsally. Dorsal surface of postpetiole dull with weak costulae and shallow areolate sculpture. Gaster smooth and shining, with extensive spectral iridescence on the first tergite. Surface of the first gastral sternite smooth and shining, with weaker spectral iridescence.

Setae: antennal scapes and funiculi with short, adpressed pilosity. Dorsum of head, mesosoma, waist segments and gaster with short, erect, blunt-tipped setae, the longest of which are roughly the width of the compound eye. The head bears ~28, mesosoma ~24, petiole 4, postpetiole ~12, and first gastral tergite ~44 setae. Sparse, adpressed pubescence present on the entire body, but only apparent on the gaster due to the dense ground sculpture on the rest of the body.

Color: head, mesosoma (except for propodeal spines), waist segments (except for strigil), and gaster are uniformly dark brown. Mandibles, extremities, and strigil are pale yellow.

**Gyne measurements & indices (*n* = 4):** SL = 0.561–0.613 (0.591); FRS = 0.224–0.254 (0.243); CW = 0.776–0.807 (0.787); CWb = 0.715–0.758 (0.731); PoOC = 0.278–0.315 (0.301); CL = 0.716–0.775 (0.756); EL = 0.210–0.224 (0.217); EW = 0.160–0.185 (0.169); MD = 0.146–0.154 (0.150); WL = 1.153–1.262 (1.206); SPST = 0.282–0.328 (0.302); MPST = 0.304–0.342 (0.321); PEL = 0.384–0.466 (0.438); NOL = 0.212–0.265 (0.244); NOH = 0.131–0.176 (0.159); PEH = 0.256–0.310 (0.288); PPL = 0.210–0.297 (0.251); PPH = 0.316–0.359 (0.336); PW = 0.715–0.779 (0.757); SBPA = 0.354–0.374 (0.363); SPTI = 0.293–0.345 (0.327); PEW = 0.182–0.206 (0.196); PNW = 0.189–0.212 (0.201); PPW = 0.492–0.553 (0.533); HFL = 0.629–0.674 (0.655); HFWmax = 0.122–0.151 (0.139); HFWmin = 0.051–0.061 (0.057); CS = 1.073–1.142 (1.109); ES = 0.293–0.313 (0.301); SI = 78–86 (81); OI = 26–28 (27); CI = 92–100 (97); WLI = 161–172 (165); SBI = 48–52 (50); PSI = 23–28 (25); PWI = 260–284 (273); PLI = 148–189 (177); NI = 151–162 (154); PNWI = 100–105 (103); NLI = 55–57 (56); FI = 234–256 (245).

**Gyne description:** In full-face view, head subquadrate, roughly as long as broad (CI 92–100). Mandibles weakly striate, shining, and armed with five teeth: the apical-most well developed, followed by a four roughly equally developed smaller teeth. Anterior clypeal very slightly concave medially. Antennal scapes moderately long: when fully retracted, just reaching the posterior margin of the head capsule (SI 78–86). Antennae 12-segmented; antennal club of three segments, with the apical-most segment as long as the preceding two in combination. Frontal carinae very short, extending past the antennal toruli by one and a half times the maximum width of the antennal scape. Compound eyes moderately protruding past the lateral margins of the head capsule. Lateral margin of head slightly convex, converging evenly to the mandibular insertions. Posterior head margin flat to very slightly convex, rounding evenly into the lateral margins.

In profile view, compound eyes teardrop-ovular and large (OI 26–28), with 16 ommatidia in longest row. Mesoscutum rounded evenly anteriorly, covering the dorsal surface of the pronotum, and flat dorsally. Mesoscutellum depressed slightly below the level of the mesoscutum; posterior margin of mesoscutellum even with the posterior margin of the metanotum. Propodeal spiracle well developed, directed posterolaterally, and separated from the propodeal declivity by about four spiracle diameters. Propodeal spines stout and well developed, but short (PSI 23–28), about half as long as the propodeal declivity, tapering evenly from the base, straight, and directed posteriorly. Propodeal declivity straight and flat, forming a rounded ~90° angle with the base of the propodeal spines. Propodeal lobes rounded and very weakly developed. Metapleural gland bulla small, extending from the metacoxal insertion halfway to the propodeal spiracle. Petiole moderately long (PLI 148–189), without tubercles anterodorsally. Subpetiolar process in the form of a weakly developed, blunt triangular tooth, which grades evenly into the ventral margin of the petiole posteriorly. Petiolar peduncle short; comprising about one third of the total length of the petiole. Petiolar node erect: transition between peduncle and node evenly rounded, resulting in a very slightly concave anterior node face; anterior face forming a sharp ~90° angle with the dorsal face, which is evenly convex and short; dorsal face rounding evenly into the posterior face, which forms a ~130° angle with the caudal cylinder. Postpetiole evenly rounded anteriorly, dorsal face weakly bulging anterodorsally before flattening posteriorly; ventral surface lobed.

In dorsal view, mesoscutum covering pronotum anteriorly, but humeri visible laterally as rounded sclerites. Propodeal spines weakly diverging proximally, but converging distally, their apices separated from each other by about one and a half times their length. Petiolar peduncle with spiracles slightly protruding past the lateral margins, the peduncle broadened where they arise. Petiolar node weakly convex anteriorly, its shape roughly trapezoidal; posterior face of node indistinct. Petiolar node slightly wider than the peduncle, and evenly grading into the caudal cylinder, which is roughly the same width as the node. Postpetiole very broad (PWI 260–284), anteroposteriorly compressed, and campaniform, articulating with most of the anterior margin of the gaster, leaving small, angulate margins on each side of the gaster exposed. Anterior margin of postpetiole weakly convex, with corners evenly rounding into the lateral margins, which evenly diverge to the angulate posterior corners; posterior margin broadly concave. Metafemur weakly to moderately incrassate (FI 234–256).

Sculpture: median clypeal carina present, flanked by additional equally strong carinae over weakly areolate ground sculpture. Antennal scapes weakly areolate and moderately shining. Cephalic dorsum densely areolate, with weak longitudinal striae. Ventral surface of head weakly, but densely, areolate. Pronotal neck areolate. Pronotum, propodeum, and posterior halves of the anepisternum and katepisternum sculptured like the cephalic dorsum; anterior halves of anepisternum and katepisternum smooth and shining. Mesoscutum costulate with weak areolate sculpture; a small patch of smooth and shining sculpture present anteromedially. Mesoscutellum densely areolate, with a central strip of weaker, shining sculpture. Femora smooth and shining, with traces weak areolate sculpture on the distal quarter. Petiole covered with shallow areolate sculpture, which is weaker ventrally and on the angulate dorsal surface of the node. Dorsal surface of postpetiole dull, with weak costulae and shallow areolate sculpture. Gaster smooth and shining, with extensive spectral iridescence on the first, second, and third tergites. Surface of the and first gastral sternite smooth and shining, with weaker spectral iridescence.

Setae: antennal scapes and funiculi with short, adpressed pilosity. Dorsum of head, mesosoma, waist segments and gaster with short, erect, blunt-tipped setae, the longest of which are roughly a third of the width of the compound eye. Sparse, adpressed pubescence present on the entire body, but only apparent on the gaster due to the dense ground sculpture on the rest of the body.

Color: head, mesosoma (except for propodeal spines), waist segments (except for strigil), and gaster are uniformly dark brown. Mandibles, extremities, and strigil are pale yellow.

**Male measurements & indices (*n* = 4):** SL = 0.312–0.338 (0.322); FRS = 0.137–0.177 (0.152); CW = 0.504–0.574 (0.534); CWb = 0.449–0.494 (0.470); PoOC = 0.190–0.202 (0.196); CL = 0.479–0.516 (0.499); EL = 0.209–0.228 (0.217); EW = 0.172–0.182 (0.177); MD = 0.056–0.069 (0.063); WL = 0.698–0.765 (0.737); SPST = n/a; MPST = 0.141–0.172 (0.156); PEL = 0.295–0.349 (0.322); NOL = 0.191–0.217 (0.203); NOH = 0.042–0.058 (0.050); PEH = 0.120–0.150 (0.130); PPL = 0.154–0.193 (0.174); PPH = 0.158–0.198 (0.181); PW = 0.445–0.519 (0.467); SBPA = n/a; SPTI = n/a; PEW = 0.115–0.130 (0.122); PNW = 0.123–0.155 (0.137); PPW = 0.262–0.339 (0.313); HFL = 0.523–0.649 (0.580); HFWmax = 0.069–0.084 (0.078); HFWmin = 0.036–0.039 (0.038); CS = 0.693–0.752 (0.719); ES = 0.295–0.315 (0.305); SI = 67–70 (69); OI = 42–43 (42); CI = 90–96 (94); WLI = 152–163 (157); SBI = n/a; PSI= n/a; PWI = 228–277 (256); PLI = 153–213 (186); NI = 341–481 (412); PNWI = 99–119 (112); NLI = 59–68 (63); FI = 177–221 (204).

**Male description:** In full-face view, head subovate, slightly longer than broad (CI 90-96). Mandibles weakly striate, shining, and armed with five teeth: the apical-most well developed, followed by a four roughly equally developed smaller teeth, and the basal-most two separated from the rest by a brief diastema. Anterior clypeal margin entire and evenly convex. Antennal scapes moderately long: when fully retracted, just reaching the posterior margin of the head capsule (SI 67–70). Antennae 13-segmented; antennal club of four segments, with the apical-most segment as long as the preceding two in combination. Frontal carinae very short, extending past the antennal toruli by one and a half times the maximum width of the antennal scape. Compound eyes strongly protruding past the lateral margins of the head capsule. Lateral margin of head convex, margin between the anterior margin of the compound eye and the mandibular insertions straight. Posterior head margin very slightly convex, rounding evenly into the lateral margins.

In profile view, compound eyes ovular and large (OI 42–43), with 19 ommatidia in the longest row. Mesoscutum bulging anteriorly, covering the dorsal surface of the pronotum, flat dorsally, and rounded posteriorly. Mesoscutellum depressed slightly below the level of the mesoscutum, overhanging the very small metanotum. Propodeal spiracle well developed, directed posterolaterally, and separated from the propodeal declivity by about three spiracle diameters. Propodeal spines absent, indicated by weak carinae on the dorsal and declivitous faces of the propodeum, which converge into a rounded angle. Propodeal lobes rounded and weakly developed. Metapleural gland bulla small, extending from the metacoxal insertion halfway to the propodeal spiracle. Petiole long (PLI 153–213), without tubercles anterodorsally where it articulates with the mesosoma. Subpetiolar process absent. Petiolar peduncle short: comprising about one third of the total length of the petiole. Petiolar node low and rounded, the convergence of the anterior and dorsal faces marked by a rounded angle. Postpetiole evenly rounded anterodorsally, flattened dorsally, and with a lobed, concave ventral surface.

In dorsal view, mesoscutum covering pronotum anteriorly, humeri barely visible laterally as slivers of rounded sclerites. Petiolar peduncle with spiracles slightly protruding past the lateral margins, the peduncle broadened where they arise. Petiolar node slightly wider than the peduncle; petiole narrowed posterior to the node, before widening again to the caudal cylinder, which is slightly narrower than the node. Postpetiole very broad (PWI 228–277) and campaniform, articulating with most of the anterior margin of the gaster, leaving small, rounded margins on each side exposed. Anterior margin of postpetiole weakly convex, with the anterior corners evenly rounding into the lateral margins, which evenly diverge to the angulate posterior corners; posterior margin of postpetiole broadly concave. Metafemur not incrassate (FI 177–221).

Sculpture: median clypeal carina present, flanked by additional weak carinae over weakly areolate ground sculpture. Antennal scapes weakly shining through shallow areolate sculpture. Head uniformly densely areolate. Lateral surfaces of pronotum and propodeum moderately shining through weak areolate sculpture. Anepisternum and katepisternum smooth and shining with weak areolate sculpture on their posterior third. Dorsally, mesoscutum weakly areolate between the Mayrian furrows, with a small patch of smooth sculpture anteromedially; otherwise smooth and shining. Mesoscutellum weakly areolate. Femora smooth and shining, with traces weak areolate sculpture along the entire length. Petiole covered with shallow areolate sculpture, with the dorsal surface of the node smooth and shining. Dorsal surface of postpetiole shining, with traces of shallow areolate sculpture, especially on the posterior third. Gaster smooth and shining, with weak spectral iridescence on the first gastral tergite.

Setae: antennal scapes and funiculi with short, adpressed pilosity. Dorsum of head, mesosoma, waist segments and gaster with short, erect, blunt-tipped setae, the longest of which are roughly a quarter of the width of the compound eye. Sparse, adpressed pubescence present on the entire body, but only apparent on the gaster due to the dense ground sculpture on the rest of the body.

Color: head, mesosoma, waist segments, and gaster are uniformly dark brown. Mandibles and extremities are pale yellow.

**Etymology:** Morphological, from the Latin ‘albus’ (= white) + ‘spinus’ (= spined), in reference to the lightly colored integument of the propodeal spines.

**Comments:** *Temnothorax albispinus* is known mostly from collections made on xerophytic islands surrounding Puerto Rico; known only from a couple of localities on the main island: the vicinity of Coamo Springs, and a light form from Guánica State Forest. Only one record of their nesting habits has appeared in the literature: Wheeler (1908), when describing the type series, noted: “a single colony which was found nesting in a small cavity in the ground in the shade of a thicket, where some of the workers were moving about slowly over the dead leaves and twigs.” Additional collections of the light form have been from either from soil beneath a stone, from rotten sticks in the leaf litter, or from Winkler extraction of leaf litter. An unpublished study by Snelling & Torres notes that it is attracted to tuna fish baits placed on the soil but has never been observed at baits placed on vegetation, even in localities where it is common. Apparently heat tolerant, workers have been observed foraging at baits during the midday hours, when soil surface temperatures probably exceed 45 °C (R. R. Snelling & J.A. Torres, 2001, unpublished data). Males have been collected at light traps in Guánica State Forest, but queens have only been collected from nests or from sifting leaf litter. Based on the presence of multiple dealate gynes in nest series, *T. albispinus* may be polygynous, but only dissections of the gynes can rule out functional monogyny. This species, like other members of the *pulchellus* group, is probably ground or leaf litter nesting. Populations from Mona Island, which correspond to subspecies *pallipes* Wheeler, appear to be highly variable in the amount of infuscation on their appendages, ranging from the entirety of the legs being light yellow to the femora and antennal scapes being medium brown. However, another difference between *T. albispinus pallipes* and *T. albispinus s.s*. noted by Wheeler (1931), the absence of sculpture on the dorsal surface of the petiolar node, appears to occur only in the Isla de Mona populations. The amount of sculpture on the first gastral tergite appears to vary throughout the range of *T. albispinus* as well: at the extreme end, the anterior 2/3 of the tergite is weakly sculptured in the Coamo Springs worker. Nest series of workers indicate that the extent of femoral incrassation is at least somewhat dependent on worker size. The light form of this species has been determined as *T. torrei* in Torres & Snelling, 1997 and in the unpublished manuscript by Snelling & Torres, which is a review of the ants of Puerto Rico (Fig. 136). It is easily separated from *T. torrei* by the larger size and presence of four erect setae on the dorsum of the petiole. Recognition of this species also explains what was interpreted as a disjunct distribution of *T. torrei* in Torres & Snelling, 1997. *Temnothorax torrei*, like many other *Temnothorax* species, has a Bahamian-Cuban distribution; it has never been collected from the island of Hispaniola. This species has long been suspected to have close relationships to other members of the *salvini* clade: specifically, *T. androsanus*, *T. pastinifer*, and *T. subditivus* (Wheeler, 1908). Baroni Urbani (1978) lumped them into the heterogeneous *pulchellus* group. In the first formal morphology-based phylogenetic work including this species, Fontenla Rizo (2000), while not including all of the taxa in Baroni Urbani’s *pulchellus* group, corroborated these relationships to some degree, with the caveat that *T. terricola* and *T. torrei* fell outside of this group. Combined molecular and morphological analyses (M. Prebus, 2021, unpublished data) mostly agree with Fontenla Rizo (2000).

***Temnothorax bahoruco* sp. nov.**

**Figure 137:**
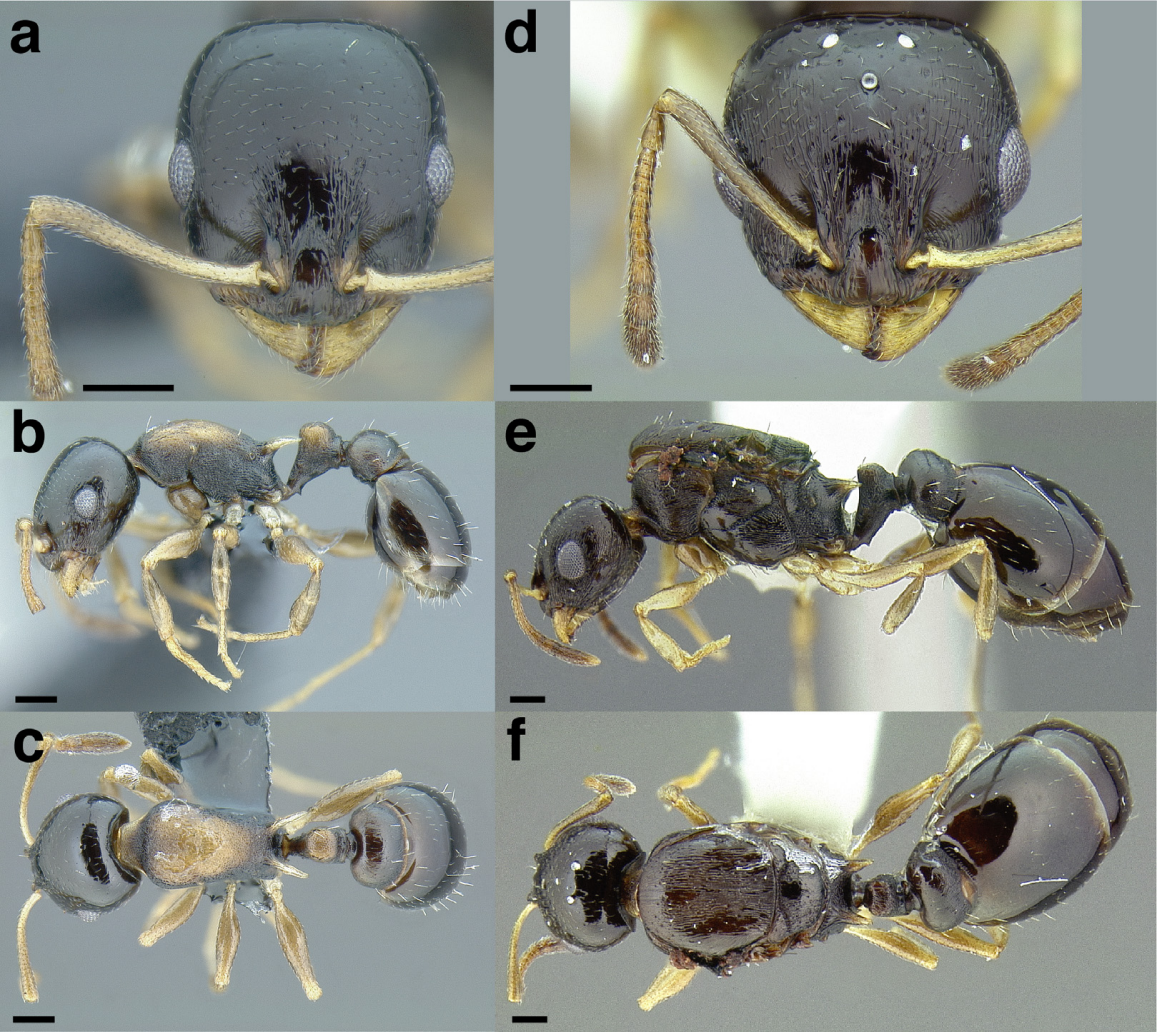
*Temnothorax bahoruco* sp. nov. (A-C) Holotype worker (CASENT0758817). (A) Full-face view. (B) Profile view. (C) Dorsal view. (D-F) Paratype gyne (MCZENT00521421). (D) Full-face view. (E) Profile view. (F) Dorsal view. Scale bars 0.2 mm.

Distribution: Fig. 133C; worker & gyne: Fig. 137.

**Type material examined:** *Holotype worker:* DOMINICAN REPUBLIC: Pedernales: Sierra de Bahoruco National Park, 18.29217°N 71.69448°W ± 30 m, 2,025 m, 30 March 2014, D. Lubertazzi#DL03802, open pine forest, in dead tree trunk and soil, 1 worker (CASENT0758817) [MCZC].

*Paratype workers and gyne:* same data as holotype, 1 worker (CASENT0758816) [MCZC] 1 worker (CASENT0758678) [UCDC]; same data as previous, except: D. Lubertazzi#DL03803:003, open pine forest, in dead tree trunk and soil, 1 dealate gyne (MCZENT00521421) [MCZC].

**Geographic range:** High elevations of Hispaniola (Fig. 133C).

**Worker diagnosis:** *Temnothorax bahoruco* sp. nov. can be separated from all other species in the *salvini* clade by the following character combination: anterior clypeal margin flat; in profile view, dorsum of mesosoma weakly convex; metanotal groove not impressed; propodeum not depressed below the level of the promesonotum; propodeum bearing standing setae dorsally; propodeal spines about as long as the propodeal declivity, directed posterodorsally, and weakly downcurved; in dorsal view, propodeal spines broadly approximated, the negative space between them “U” shaped; hind femora weakly incrassate; petiole 1.4 times the length of the postpetiole; in dorsal view, apex of petiolar node narrower to slightly broader than the caudal cylinder of the petiole; postpetiole very broad: greater than or equal to 2.4 times the width of the petiole; dorsum of head smooth and shining; petiolar node with four erect setae dorsally; setae on head, mesosoma, waist segments and gaster erect, moderately long, sparse and blunt (never long and tapering); integument tricolored: predominantly dark brown, with the mandibles, and propodeal spines light yellow; legs testaceous with light yellow joints and tarsi.

**Similar species:** *Temnothorax balaclava* sp. nov., *T. ciferrii, T. harlequina* sp. nov., *T. hippolytus* sp. nov., *T. magnabulla* sp. nov., *T. pulchellus, T. schwarzi, T. terricola*, and *T. wilsoni* sp. nov. The erect setae count of four on the dorsum of the petiolar node differentiates *T. bahoruco* sp. nov. from *T. terricola* and *T. hippolytus* sp. nov., which have only two along the posterior margin. The presence of erect setae on the propodeum distinguishes *T. bahoruco* sp. nov. from *T. pulchellus*. The relatively small metapleural gland bulla of *T. bahoruco* sp. nov., which reaches halfway to from the metacoxal insertion to the propodeal spiracle, distinguishes it from *T. magnabulla* sp. nov., in which it extends three quarters of the way or more. *Temnothorax balaclava* sp. nov. and *T. harlequina* sp. nov. can be separated most easily by the color of the integument: *T. harlequina* sp. nov. has a light yellow gaster which strongly contrasts with the darker integument of the rest of the body, whereas the gaster of *T. bahoruco* sp. nov. is always light dark brown. The mesosoma is of *T. bahoruco* sp. nov. is less arched and more densely sculptured in comparison to *T. harlequina* sp. nov. *Temnothorax bahoruco* sp. nov. could also be confused with *T. schwarzi* due to the smooth and shining head capsule, but the relatively narrow apex of the petiolar node of *T. bahoruco* sp. nov. will separate the two. The medially flat anterior clypeal margin of *T. bahoruco* sp. nov. will differentiate it from *T. wilsoni* sp. nov. and *T. balaclava* sp. nov., which have emarginate and rounded anterior clypeal margins, respectively. *Temnothorax bahoruco* sp. nov. can be further separated from *T. balaclava* sp. nov. by the relatively shorter petiole, which is about 1.4 times the length of the postpetiole, versus 1.5 to 1.6 times in *T. balaclava* sp. nov.

**Worker measurements & indices (*n* = 3):** SL = 0.530–0.560 (0.546); FRS = 0.215–0.235 (0.225); CW = 0.616–0.641 (0.631); CWb = 0.569–0.604 (0.589); PoOC = 0.268–0.289 (0.275); CL = 0.651–0.693 (0.675); EL = 0.138–0.152 (0.147); EW = 0.112–0.118 (0.115); MD = 0.145–0.168 (0.160); WL = 0.733–0.805 (0.772); SPST = 0.256–0.263 (0.261); MPST = 0.241–0.252 (0.246); PEL = 0.312–0.338 (0.329); NOL = 0.189–0.217 (0.199); NOH = 0.122–0.144 (0.135); PEH = 0.225–0.254 (0.241); PPL = 0.218–0.242 (0.232); PPH = 0.253–0.271 (0.259); PW = 0.402–0.441 (0.422); SBPA = 0.196–0.213 (0.204); SPTI = 0.244–0.315 (0.283); PEW = 0.161–0.168 (0.164); PNW = 0.154–0.177 (0.169); PPW = 0.401–0.436 (0.419); HFL = 0.510–0.532 (0.523); HFWmax = 0.126–0.137 (0.132); HFWmin = 0.044–0.055 (0.050); CS = 0.895–0.944 (0.926); ES = 0.194–0.211 (0.205); SI = 92–93 (93); OI = 22; CI = 86–89 (87); WLI = 129–133 (131); SBI = 34–36 (35); PSI = 32–36 (34); PWI = 245–261 (255); PLI = 140–143 (142); NI = 131–178 (149); PNWI = 94–110 (103); NLI = 56–70 (61); FI = 244–286 (268).

**Worker description:** In full-face view, head subquadrate, longer than broad (CI 86–89). Mandibles weakly striate, shining, and armed with five teeth: the apical-most well developed, followed by a less developed preapical tooth and three equally-developed smaller teeth. Anterior clypeal margin flat medially. Antennal scapes moderately long: when fully retracted, just barely surpassing the posterior margin of the head capsule (SI 92–93). Antennae 12-segmented; antennal club of three segments, with the apical-most segment slightly longer than the preceding two in combination. Frontal carinae moderately long, extending past the antennal toruli by about two times the maximum width of the antennal scape. Compound eyes moderately protruding past the lateral margins of the head capsule. Lateral margin of head weakly convex, forming a continuous arc from the posterior of the head, converging below the compound eye to the mandibular insertions. Posterior head margin weakly concave to flat, rounding evenly into the lateral margins.

In profile view, compound eyes ovular and moderately large (OI 22), with 9 ommatidia in longest row. Pronotal declivity indistinct, neck and anterior face of pronotum forming a rounded ~120° angle. Mesosoma evenly convex dorsally from where it joins the pronotal neck to the propodeal spines. Promesonotal suture extending from the posterior margin of the procoxal insertion only to the mesothoracic spiracle, which is well developed. Metanotal groove visible as a disruption of the sculpture laterally from where it arises between the mid- and hind coxae to where it ends in the poorly developed metathoracic spiracle, which is nearly indistinguishable against the ground sculpture. Propodeal spiracle well developed, directed posterolaterally, and separated from the propodeal declivity by about two and a half spiracle diameters. Propodeal spines well developed and moderately long (PSI 32–36), about as long as the propodeal declivity, tapering evenly from the base, weakly downcurved, and acute. Propodeal declivity straight and flat, forming a rounded ~100° angle with the base of the propodeal spines. Propodeal lobes rounded and weakly developed. Metapleural gland bulla small, extending from the metacoxal insertion halfway to the propodeal spiracle. Petiole moderately long (PLI 140–143), without tubercles anterodorsally. Subpetiolar process in the form of a small, acute tooth; ventral margin of petiole weakly concave posterior to it. Petiolar peduncle short, comprising about one third the total length of the petiole. Petiolar node robust: transition between peduncle and node marked by a rounded angle of ~140°, resulting in a weakly concave anterior node face; anterior face forming a rounded, ~100° angle with the dorsal surface, which is evenly convex; dorsal surface rounding evenly into the short posterior declivity, which forms a ~100° angle with the caudal cylinder. Postpetiole evenly rounded dorsally, and weakly lobed ventrally.

In dorsal view, humeri weakly developed: evenly rounded and slightly wider than the rest of the mesosoma; mesothoracic spiracles weakly protruding past the lateral margins of the mesosoma, visible as slight angles where the pronotum meets the mesonotum. Metanotal groove absent: mesonotum and propodeum completely fused and converging evenly to the bases of the propodeal spines. Propodeal spines broadly approximated basally and diverging apically, their apices separated from each other by slightly more than their length; negative space between them “U” shaped. Petiolar peduncle with spiracles protruding past the lateral margins, peduncle slightly narrowed anterior to them. Petiolar node evenly ovular, but slightly flatted posteriorly; node slightly wider than the peduncle, and evenly grading into the caudal cylinder, which is slightly narrower than the node. Postpetiole very broad (PWI 245–261) and campaniform, articulating with the nearly the entire anterior margin of the gaster. Anterior margin of the postpetiole flat and evenly rounds into the lateral margins, which diverge to the angulate posterior corners; posterior margin broadly concave. Metafemur weakly to moderately incrassate (FI 244–286).

Sculpture: median clypeal carina present, extending posteriorly nearly to the frontal triangle, and flanked on either side by two equally strong carinae. Lateral clypeal lobes with additional, weaker carinae; ground sculpture smooth and shining. Antennal scapes smooth and shining through weak areolate ground sculpture. Cephalic dorsum smooth and shining, but with covered with coarse piligerous punctures; costulae flanking the frontal carinae. Lateral surfaces of head with weak areolate sculpture posterior to the compound eye, dense rugose sculpture surrounding the compound eye, and weak rugae between the compound eye and the mandibular insertion. Ventral surface of head smooth and shining. Pronotal neck areolate. Lateral surface of the pronotum with weak costulate that are joined by rugulae. Mesopleurae and lateral surface of propodeum areolate-rugulose, with weaker sculpture between the propodeal spiracle and the propodeal spines. Dorsal surface of mesosoma uniformly areolate-rugulose. Femora shining, with weak areolate sculpture on the distal quarter. Dorsal surface of peduncle and ventral surface of petiole smooth and shining, with weak areolate sculpture on all other surfaces. Postpetiole smooth and shining, with weak areolate sculpture on the posterior quarter. Gaster smooth and shining, without spectral iridescence.

Setae: antennal scapes and funiculi with short, adpressed pilosity. Dorsum of head, pronotum, waist segments and gaster with moderately abundant, erect, blunt-tipped setae, the longest of which are about the width of the compound eye. The head bears ~24, mesosoma ~10, petiole 4, postpetiole ~10, and first gastral tergite ~20 setae. Short, sparse pubescence present over the entire body.

Color: predominantly dark brown, with the mandibles, bases of antennal scapes, and propodeal spines light yellow. Distal parts of the antennal scape, antennal funiculus, dorsum of mesosoma, and petiolar node testaceous. Legs testaceous with light yellow joints and tarsi.

**Gyne measurements & indices (*n* = 1):** SL = 0.570; FRS = 0.270; CW = 0.794; CWb = 0.736; PoOC = 0.288; CL = 0.746; EL = 0.244; EW = 0.188; MD = 0.147; WL = 1.304; SPST = 0.296; MPST = 0.296; PEL = 0.416; NOL = 0.200; NOH = 0.167; PEH = 0.315; PPL = 0.222; PPH = 0.391; PW = 0.807; SBPA = 0.354; SPTI = 0.356; PEW = 0.251; PNW = 0.233; PPW = 0.528; HFL = 0.673; HFWmax = 0.129; HFWmin = 0.053; CS = 1.109; ES = 0.338; SI = 77; OI = 30; CI = 99; WLI = 177; SBI = 48; PSI = 23; PWI = 210; PLI = 187; NI = 120; PNWI = 93; NLI = 48; FI = 243.

**Gyne description:** In full-face view, head subquadrate, about as long as broad (CI 99). Mandibles striate but shining and armed with five teeth: the apical-most well developed, followed by a less developed preapical tooth and three equally-developed smaller teeth. Anterior clypeal margin flat medially. Antennal scapes moderately long: when fully retracted, just reaching the posterior margin of the head capsule (SI 77). Antennae 12-segmented; antennal club composed of three segments, with the apical-most segment as long as the preceding two in combination. Frontal carinae moderately long, extending past the antennal toruli by about three times the maximum width of the antennal scape. Compound eyes moderately protruding past the lateral margins of the head capsule. Lateral margin of head evenly convex, converging from below the compound eyes to the mandibular insertions. Posterior head margin flat, rounding evenly into the lateral margins.

In profile view, compound eyes ovular and large (OI 30), with 18 ommatidia in the longest row. Mesoscutum rounded evenly anteriorly, covering the dorsal surface of the pronotum, and flat dorsally. Mesoscutellum on the same level as the mesoscutum, not overhanging the metanotum. Posterior margin of metanotum extending past the posterior margin of the mesoscutum. Propodeal spiracle well developed, directed posterolaterally, and separated from the propodeal declivity by about four spiracle diameters. Metapleural gland bulla small, extending from the metacoxal insertion halfway to the propodeal spiracle. Propodeal spines stout and well developed, but short (PSI 23), about as two thirds as long as the propodeal declivity, tapering evenly from the base, directed posteriorly, straight, and blunt. Propodeal declivity straight and flat, forming a rounded ~100° angle with the base of the propodeal spines. Propodeal lobes rounded and weakly developed. Petiole moderately long (PLI 187), without tubercles anterodorsally. Subpetiolar process in the form of a small, triangular, very acute tooth, which grades evenly into the ventral margin of the petiole posteriorly. Petiolar peduncle short: petiolar node covering most of the petiolar dorsum. Petiolar node erect: transition between peduncle and node evenly rounded, resulting in a very slightly concave anterior node face; anterior face forming a sharp ~90° angle with the dorsal face, which is short. Dorsal face of petiolar node joins the posterior face at a ~120° angle. Posterior face of petiolar node forms a ~120° angle with the caudal cylinder. Postpetiole evenly rounded anterodorsally, bulging slightly before it transitions into the flattened dorsal face; ventral surface weakly convex.

In dorsal view, mesoscutum covering pronotum anteriorly, but humeri visible laterally as rounded sclerites. Propodeal spines diverging apically, their apices separated from each other by about two times their length. Petiolar peduncle with spiracles protruding past the lateral margins. Petiolar node trapezoidal: widest anteriorly, and emarginated anterodorsally; lateral faces converge to the posterior face. Petiolar node slightly narrower than the peduncle, and evenly grading into the caudal cylinder, which is slightly wider than the node. Postpetiole moderately broad (PWI 210), anteroposteriorly compressed, and subquadrate, articulating with most of the anterior margin of the gaster, leaving small, angulate margins on each side exposed. Anterior margin of postpetiole broadly concave, with corners evenly rounded as they transitions to the lateral margins, which are roughly parallel to the angulate posterior corners; posterior margin broadly concave. Metafemur weakly incrassate (FI 243).

Sculpture: median clypeal carina present, extending from the anterior margin nearly to frontal triangle, and flanked by weaker, indistinct carinae; lateral margins of median clypeal lobe with two carinae that are as strong as the medial carina. Lateral clypeal lobes with additional weaker carinae; ground sculpture smooth and shining. Antennal scapes smooth and shining. Cephalic dorsum with costulae, which become weaker posteriorly; a strip of smooth sculpture present medially, surrounding a central carina, which extends from the frontal triangle nearly to the median ocellus. Lateral surfaces of head with weak areolate sculpture posterior to the compound eye, dense rugose sculpture surrounding the compound eye, and rugae between the compound eye and the mandibular insertion. Ventral surface of head with weak rugae and areolae. Pronotal neck areolate. Pronotum with weak areolate ground sculpture arranged into longitudinal rows and separated by superficial striae. Anepisternum and katepisternum shining on their anterior halves, transitioning into weak costulae over weak areolae posteriorly. Propodeum areolate laterally and on the propodeal declivity; sculpture weaker between the propodeal spiracle and the base of the propodeal spine. Mesoscutum with costulae which are weaker anteromedially and laterally. Mesoscutellum smooth and shining medially, surrounded by dense striae and areolae. Femora smooth and shining, with traces of weak areolate sculpture distally. Petiole areolate laterally and on the dorsal surface of the node; peduncle smooth and shining dorsally; base of petiolar node with rugulae. Postpetiole smooth and shining anteriorly, areolate laterally and on the posterior half. Gaster smooth and shining, without spectral iridescence. Surface of the first gastral sternite smooth and shining.

Setae: antennal scapes and funiculi with short, adpressed pilosity. Dorsum of head, pronotum, waist segments and gaster with moderately abundant, erect, blunt-tipped setae, the longest of which are about half the width of the compound eye. Short, sparse pubescence present over the entire body.

Color: predominantly dark brown, with the mandibles, bases of antennal scapes, and propodeal spines light yellow. Distal parts of the antennal scape and antennal funiculus testaceous. Legs testaceous with light yellow joints and tarsi.

**Male:** Unknown.

**Etymology:** Bahoruco, a word from the Macorix language of the people of the northern coast of Hispaniola meaning “within the forest”, a reference to the mountain range and habitat in which the type specimens were collected.

**Comments:** *Temnothorax bahoruco* sp. nov. is known only from high elevation *Pinus occidentalis* forests in the Sierra de Bahoruco, where workers and a dealate gyne were collected from a dead tree trunk and the surrounding soil. Other details of the biology of this species are unknown but are probably similar to other members of the terricolous pan-Caribbean *pulchellus* group. In the limited number of specimens that I observed from this series, this species displays some variation in coloration of the mesosoma and waist segments, ranging from being completely dark brown to dorsally testaceous. This taxon is part of a species complex with *T. balaclava* sp. nov., *T. ciferrii*, and *Temnothorax wilsoni* sp. nov., all of which have only been collected from allopatric populations in mountains and low-lying dry forest from the southern parts of Hispaniola. The worker is morphologically quite similar to *T. wilsoni* sp. nov. and *T. balaclava* sp. nov., differing primarily in the conformation of the clypeus, coloration, and sculptural details. The gyne differs from these two species by the concave anterior margin of the postpetiole in dorsal view.

***Temnothorax balaclava* sp. nov.**

**Figure 138:**
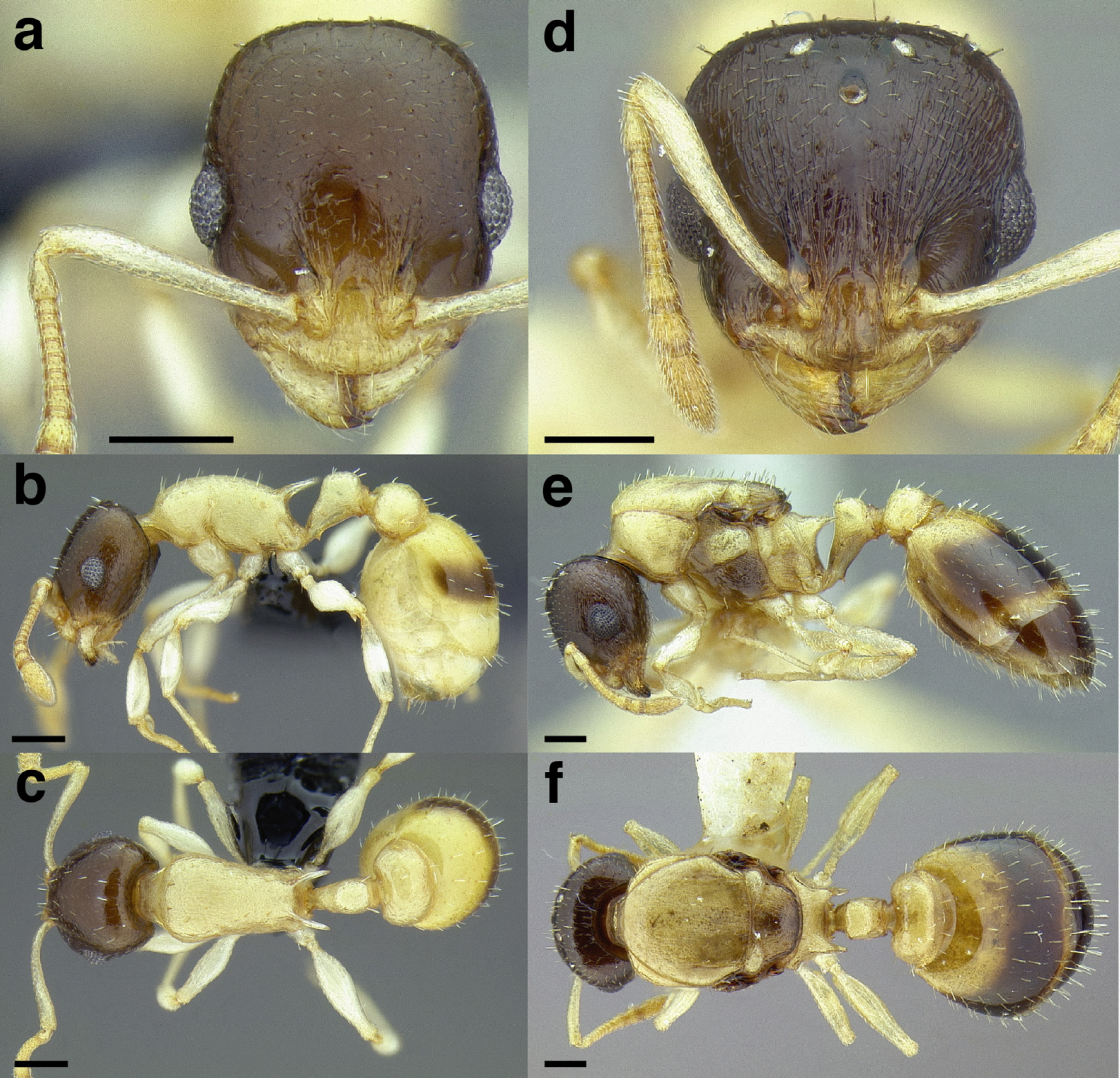
*Temnothorax balaclava* sp. nov. (A–C) Holotype worker (CASENT0758262). (A) Full-face view. (B) Profile view. (C) Dorsal view. (D–F) Paratype gyne (MCZENT00510550). Scale bars 0.2 mm.

Distribution: Fig. 133D; worker & gyne: Fig. 138.

**Type material examined:** *Holotype worker:* DOMINICAN REPUBLIC: Pedernales: Jaragua National Park, 18.02571°N 71.64732°W ± 5 m, 92 m, 1 April 2012, D. Lubertazzi#DL03474, dry forest, downed wood (CASENT0758262) [MCZC].

*Paratype workers & gyne:* same data as holotype, 1 worker (CASENT0756153) [UCDC] 2 workers (CASENT4010177) [CASC] 2 workers (CASENT4010178) [USNM]; same data as previous, except: D. Lubertazzi#DL03476:001, dry forest, in soil under downed wood, 2 workers (CASENT4010179) [LACM]; same data as previous, except: D. Lubertazzi #DL03477:001, dry forest, under dead agave, 2 workers (CASENT4010180) [MNHNSD]; same data as previous, except: D. Lubertazzi#DL03477:002, dry forest, under dead agave, 2 workers (MCZENT00510544) [MCZC]; same data as previous, except: D. Lubertazzi#DL03477:003, dry forest, under dead agave, 2 workers (MCZENT00510545) [MCZC]; same data as previous, except: D. Lubertazzi#DL03477:004, dry forest, under dead agave, 1 dealate gyne (MCZENT00510546) [MCZC].

**Non-type material examined:** DOMINICAN REPUBLIC: Pedernales: Jaragua National Park, 17.792206°N 71.46109°W ± 5 m, 125 m, 26 March 2012, D. Lubertazzi#DL03383:003, xeric scrub on limestone, in soil and litter at base of tree, 1 worker (MCZENT00510535) [MCZC]; same data as previous, except: D. Lubertazzi#DL03388:001, xeric scrub on limestone, sifted soil and litter under half buried branch, 1 worker (MCZENT00510536) [MCZC]; same data as previous, except: 17.79249°N 71.46385°W ± 13 m, 126 m 30 March 2012, D. Lubertazzi#DL03411:001, dry forest, litter at base of large tree, 1 worker (MCZENT00510537) [MCZC]; same data as previous, except: Jaragua National Park access road, 17.78166°N 71.44007°W ± 8 m, 87 m 29 March 2012, D. Lubertazzi#DL03433:001, dry forest, under downed wood, 1 worker (MCZENT00510538) [MCZC]; same data as previous, except: Jaragua National Park, 18.02571°N 71.64732°W ± 9 m, 92 m 2 April 2012, D. Lubertazzi #DL03497:001, dry forest, downed wood, 2 workers (MCZENT00510548) [MCZC]; same data as previous, except: D. Lubertazzi#DL03497:002, dry forest, downed wood, 2 workers (MCZENT00510549) [MCZC]; same data as previous, except: 18.02571°N 71.64732°W ± 11 m, 92 m 31 March 2012, D. Lubertazzi#DL03501:001, dry forest, litter, 1 dealate gyne (MCZENT00510550) [MCZC] 1 worker (MCZENT00510551) [MCZC]; same data as previous, except: #DL03501:003, dry forest, litter, 2 workers (MCZENT00510552) [MCZC].

**Geographic range:** Low elevations of Hispaniola (Fig. 133D).

**Worker diagnosis:** *Temnothorax balaclava* sp. nov. can be separated from all other species in the *salvini* clade by the following character combination: anterior clypeal margin evenly convex; in profile view, dorsum of mesosoma weakly convex; metanotal groove not impressed; propodeum not depressed below the level of the promesonotum; propodeum bearing standing setae dorsally; propodeal spines about as long as the propodeal declivity, and directed posterodorsally; in dorsal view, propodeal spines broadly approximated, the negative space between them “U” shaped; hind femora moderately incrassate; petiole 1.5 to 1.6 times the length of the postpetiole; in dorsal view, apex of petiolar node narrower to slightly broader than the caudal cylinder of the petiole; postpetiole very broad: greater than or equal to 2.4 times the width of the petiole; dorsum of head smooth and shining; petiolar node with four erect setae dorsally; setae on head, mesosoma, waist segments and gaster erect, moderately long, sparse and blunt (never long and tapering); integument bicolored: predominantly yellow, with the head capsule (excluding clypeus) and posterior third of the first gastral tergite dark brown.

**Similar species:** *Temnothorax balaclava* sp. nov., *T. ciferrii, T. harlequina* sp. nov., *T. hippolytus* sp. nov., *T. magnabulla* sp. nov., *T. nigricans, T. pulchellus, T. schwarzi, T. terricola*, and *T. wilsoni* sp. nov. The erect setae count of four on the dorsum of the petiolar node differentiates *T. balaclava* sp. nov. from *T. terricola* and *T. hippolytus* sp. nov., which have only two along the posterior margin. The presence of erect setae on the propodeum distinguishes *T. balaclava* sp. nov. from *T. pulchellus*. The relatively small metapleural gland bulla of *T. balaclava* sp. nov., which reaches halfway to from the metacoxal insertion to the propodeal spiracle, distinguishes it from *T. magnabulla* sp. nov., in which it extends three quarters of the way or more. *Temnothorax balaclava* sp. nov. and *T. harlequina* sp. nov. can be separated most easily by the color of the integument: *T. harlequina* sp. nov. has a light yellow gaster which strongly contrasts with the darker integument of the rest of the body, whereas the gaster, waist segments, and mesosoma and of *T. balaclava* sp. nov. is always light yellow, and the gaster bears a dark brown band across the posterior third of the gaster. The mesosoma is of *T. balaclava* sp. nov. is less arched and more densely sculptured in comparison to *T. harlequina* sp. nov. *Temnothorax balaclava* sp. nov. could also be confused with *T. schwarzi* due to the smooth and shining head capsule, but the relatively narrow apex of the petiolar node of *T. balaclava* sp. nov. will separate the two. *Temnothorax nigricans* is superficially similar due to the nearly identical integument coloring, but can be separated from *T. balaclava* sp. nov. by the areolate head sculpture and dorsally broader petiolar node. The medially rounded anterior clypeal margin of *T. balaclava* sp. nov. will differentiate it from *T. wilsoni* sp. nov. and *T. bahoruco* sp. nov., which have emarginate and flat anterior clypeal margins, respectively. *Temnothorax balaclava* sp. nov. can be further separated from *T. bahoruco* sp. nov. by the relatively longer petiole, which is 1.5 to 1.6 times the length of the postpetiole, versus 1.4 times in *T. bahoruco* sp. nov.

**Worker measurements & indices (*n* = 7):** SL = 0.407–0.471 (0.434); FRS = 0.165–0.212 (0.185); CW = 0.470–0.559 (0.515); CWb = 0.425–0.518 (0.473); PoOC = 0.201–0.240 (0.220); CL = 0.511–0.612 (0.559); EL = 0.121–0.140 (0.130); EW = 0.093–0.114 (0.105); MD = 0.114–0.149 (0.130); WL = 0.561–0.675 (0.614); SPST = 0.220–0.247 (0.231); MPST = 0.194–0.223 (0.207); PEL = 0.247–0.299 (0.277); NOL = 0.131–0.186 (0.161); NOH = 0.092–0.116 (0.101); PEH = 0.161–0.200 (0.178); PPL = 0.163–0.202 (0.178); PPH = 0.190–0.228 (0.207); PW = 0.315–0.376 (0.342); SBPA = 0.130–0.172 (0.150); SPTI = 0.135–0.294 (0.216); PEW = 0.120–0.155 (0.135); PNW = 0.118–0.151 (0.135); PPW = 0.322–0.375 (0.345); HFL = 0.399–0.454 (0.430); HFWmax = 0.110–0.127 (0.119); HFWmin = 0.042–0.047 (0.045); CS = 0.681–0.824 (0.752); ES = 0.171–0.197 (0.182); SI = 86–96 (92); OI = 23–26 (24); CI = 83–87 (85); WLI = 126–133 (130); SBI = 29–34 (32); PSI = 35–39 (38); PWI = 242–268 (257); PLI = 148–166 (155); NI = 139–190 (160); PNWI = 94–109 (100); NLI = 52–63 (58); FI = 243–293 (268).

**Worker description:** In full-face view, head subquadrate, longer than broad (CI 83–87). Mandibles weakly striate, shining, and armed with five teeth: the apical-most well developed, followed by a less developed preapical tooth and three equally-developed smaller teeth. Anterior clypeal margin evenly convex. Antennal scapes moderately long: when fully retracted, just barely surpassing the posterior margin of the head capsule (SI 86–96). Antennae 12-segmented; antennal club of three segments, with the apical-most segment slightly longer than the preceding two in combination. Frontal carinae moderately long, extending past the antennal toruli by about two times the maximum width of the antennal scape. Compound eyes moderately protruding past the lateral margins of the head capsule. Lateral margin of head weakly convex, forming a continuous arc from the posterior of the head, converging below the compound eye to the mandibular insertions. Posterior head margin weakly concave to flat, rounding evenly into the lateral margins.

In profile view, compound eyes ovular and moderately large (OI 23–26), with 9 ommatidia in longest row. Pronotal declivity indistinct, neck and anterior face of pronotum forming a rounded ~120° angle. Mesosoma evenly convex dorsally from where it joins the pronotal neck to the propodeal spines. Promesonotal suture extending from the posterior margin of the procoxal insertion only to the mesothoracic spiracle, which is well developed. Metanotal groove visible as a disruption of the sculpture laterally from where it arises between the mid- and hind coxae to where it ends in the poorly developed metathoracic spiracle, which is nearly indistinguishable against the ground sculpture. Propodeal spiracle well developed, directed posterolaterally, and separated from the propodeal declivity by about two and a half spiracle diameters. Propodeal spines well developed and moderately long (PSI 35–39), about as long as the propodeal declivity, tapering evenly from the base, straight, and acute. Propodeal declivity straight and flat, forming a rounded ~90° angle with the base of the propodeal spines. Propodeal lobes rounded and weakly developed. Metapleural gland bulla small, extending from the metacoxal insertion halfway to the propodeal spiracle. Petiole moderately long (PLI 148–166), without tubercles anterodorsally. Subpetiolar process in the form of a weakly developed, acute tooth; ventral margin of petiole weakly concave posterior to it. Petiolar peduncle short: petiolar node covering most of the petiolar dorsum. Petiolar node robust: grading evenly into the petiolar peduncle anteriorly, anterior face weakly concave; anterior face forming a rounded, ~110° angle with the dorsal surface, which is weakly convex and nearly flat; dorsal surface rounding evenly into the short posterior declivity, which forms a ~100° angle with the caudal cylinder. Postpetiole evenly rounded dorsally, and weakly lobed ventrally.

In dorsal view, humeri weakly developed: evenly rounded and slightly wider than the rest of the mesosoma; mesothoracic spiracles weakly protruding past the lateral margins of the mesosoma, visible as slight angles where the pronotum meets the mesonotum. Metanotal groove absent: mesonotum and propodeum completely fused and converging evenly to the bases of the propodeal spines. Propodeal spines broadly approximated basally and diverging apically, their apices separated from each other by about their length; negative space between them “U” shaped. Petiolar peduncle with spiracles protruding past the lateral margins, peduncle slightly narrowed anterior to them. Petiolar node evenly ovular, but slightly flatted posteriorly; node slightly wider than the peduncle, and evenly grading into the caudal cylinder, which is the same width as the node. Postpetiole very broad (PWI 242–268) and campaniform, articulating with the nearly the entire anterior margin of the gaster. Anterior margin of the postpetiole weakly concave, evenly rounding into the lateral margins, which diverge to the angulate posterior corners; posterior margin broadly concave. Metafemur weakly to moderately incrassate (FI 243–293).

Sculpture: median clypeal carina present, extending posteriorly nearly to the frontal triangle, and flanked on either side by two equally strong carinae. Lateral clypeal lobes with additional, weaker carinae; ground sculpture smooth and shining. Antennal scapes shining through weak areolate ground sculpture. Cephalic dorsum smooth and shining, but with coarse piligerous punctures; costulae flanking the frontal carinae. Lateral surfaces of head with weak areolate sculpture posterior to the compound eye, dense rugose sculpture surrounding the compound eye, and weak rugae between the compound eye and the mandibular insertion. Ventral surface of head smooth and shining. Pronotal neck areolate. Lateral surface of the pronotum shining through weak costulae. Mesopleurae and anterior half of the lateral face of the propodeum areolate, but propodeum smooth and shining between the propodeal spiracle and the propodeal spines. Dorsal surface of mesosoma with weak costulae on the pronotum and lateral margins over areolate ground sculpture. Femora shining, with weak areolate sculpture on the distal quarter. Petiole smooth and shining ventrally, with areolate sculpture on all other surfaces. Dorsal and lateral surfaces of petiolar node shining through very weak sculpture. A very weak carina present on the petiolar node laterally, extending from the petiolar spiracle to the caudal cylinder. Postpetiole smooth and shining, with weak areolate sculpture on the posterior quarter. Gaster smooth and shining, without spectral iridescence.

Setae: antennal scapes and funiculi with short, adpressed pilosity. Dorsum of head, pronotum, waist segments, and gaster with moderately abundant, erect, blunt-tipped setae, the longest of which are about the width of the compound eye. The head bears ~30, mesosoma ~20, petiole 4, postpetiole ~12, and first gastral tergite ~32 setae. Short, sparse pubescence present over the entire body, but difficult to detect against the lightly colored integument.

Color: predominantly yellow, with the head capsule (excluding clypeus) and posterior third of the first gastral tergite dark brown.

**Gyne measurements & indices (*n* = 2):** SL = 0.491–0.498 (0.495); FRS = 0.244–0.253 (0.249); CW = 0.681–0.691 (0.686); CWb = 0.639–0.649 (0.644); PoOC = 0.259–0.268 (0.264); CL = 0.661–0.692 (0.677); EL = 0.184–0.191 (0.188); EW = 0.153–0.162 (0.158); MD = 0.137–0.139 (0.138); WL = 1.066–1.080 (1.073); SPST = 0.253–0.278 (0.266); MPST = 0.270–0.290 (0.280); PEL = 0.365–0.380 (0.373); NOL = 0.18; NOH = 0.135–0.144 (0.140); PEH = 0.262–0.263 (0.263); PPL = 0.187–0.199 (0.193); PPH = 0.282–0.302 (0.292); PW = 0.646–0.653 (0.650); SBPA = 0.311–0.314 (0.313); SPTI = 0.295–0.303 (0.299); PEW = 0.176–0.184 (0.180); PNW = 0.201–0.204 (0.203); PPW = 0.470–0.477 (0.474); HFL = 0.566–0.589 (0.578); HFWmax = 0.124–0.135 (0.130); HFWmin = 0.051–0.054 (0.053); CS = 0.980–0.985 (0.982); ES = 0.261–0.272 (0.266); SI = 77; OI = 27–28 (27); CI = 92–98 (95); WLI = 166–167 (167); SBI = 48–49 (49); PSI = 24–26 (25); PWI = 259–267 (263); PLI = 191–195 (193); NI = 125–133 (129); PNWI = 109–116 (113); NLI = 47–49 (48); FI = 243–250 (247).

**Gyne description:** In full-face view, head subquadrate, about as long as broad (CI 92–98). Mandibles weakly striate, shining, and armed with five teeth: the apical-most well developed, followed by a less developed preapical tooth and three equally-developed smaller teeth. Anterior clypeal margin weakly convex medially. Antennal scapes moderately long: when fully retracted, just reaching the posterior margin of the head capsule (SI 77). Antennae 12-segmented; antennal club of three segments, with the apical-most segment as slightly longer than the preceding two in combination. Frontal carinae moderately long, extending past the antennal toruli by about four times the maximum width of the antennal scape. Compound eyes protruding past the lateral margins of the head capsule. Lateral margin of head evenly convex, converging from below the compound eyes to the mandibular insertions. Posterior head margin weakly convex, rounding evenly into the lateral margins.

In profile view, compound eyes ovular and large (OI 27–28), with 16 ommatidia in longest row. Mesoscutum rounded evenly anteriorly, covering the dorsal surface of the pronotum, and flat dorsally; mesoscutellum on the same level as the mesoscutum, not overhanging the metanotum. Propodeal spiracle well developed, directed posterolaterally, and separated from the propodeal declivity by about spiracle diameters. Propodeal spines stout and well developed, but short (PSI 24–26), about two thirds as long as the propodeal declivity, tapering evenly from the base, straight, and directed posteriorly. Propodeal declivity weakly concave, forming a rounded ~90° angle with the base of the propodeal spines. Propodeal lobes rounded and weakly developed. Metapleural gland bulla small, extending from the metacoxal insertion halfway to the propodeal spiracle. Petiole long (PLI 191–195), without tubercles anterodorsally where it articulates with the mesosoma; subpetiolar process in the form of a moderately well developed, acute, triangular tooth, which grades evenly into the ventral margin of the petiole posteriorly. Petiolar peduncle short: comprising about one third of the total length of the petiole. Petiolar node erect: transition between peduncle and node evenly rounded, resulting in a very slightly concave anterior node face; anterior face forming a sharp ~90° angle with the dorsal face, which is short and evenly rounds into the posterior face, which forms a ~110° angle with the caudal cylinder. Postpetiole evenly rounded anterodorsally, bulging slightly before it transitions into the flattened dorsal face; ventral surface weakly lobed.

In dorsal view, mesoscutum covering pronotum anteriorly, but humeri visible laterally as rounded sclerites. Propodeal spines weakly diverging apically, their apices separated from each other by about one and a half times their length. Petiolar peduncle with spiracles not protruding past the lateral margins. Petiolar node widest anteriorly, and emarginated anterodorsally; lateral faces converge to the indistinct posterior face. Petiolar node slightly broader than the peduncle, and evenly grading into the caudal cylinder, which slightly narrower than the node. Postpetiole very broad (PWI 259–267), anteroposteriorly compressed, and campaniform, articulating with most of the anterior margin of the gaster, leaving small, angulate margins on each side exposed. Anterior margin of the postpetiole weakly concave, evenly rounded as it transitions to the lateral margins, which evenly diverge to the angulate posterior corners; posterior margin broadly concave. Metafemur weakly incrassate (FI 243–250).

Sculpture: median clypeal carina present, extending from the anterior margin nearly to frontal triangle, and flanked by weaker, indistinct carinae; lateral margins of median clypeal lobe with two carinae that are as strong as the medial carina. Lateral clypeal lobes with additional weaker carinae; ground sculpture smooth and shining. Antennal scapes shining through weak areolate sculpture. Cephalic dorsum with costulae, which become weaker posteriorly; a strip of smooth sculpture present medially, surrounding a central carina, which extends from the frontal triangle nearly to the median ocellus; weak concentric costulae surrounding the antennal insertions. Lateral surfaces of head with weak areolate sculpture posterior to the compound eye, dense rugose sculpture surrounding the compound eye, with costulae superimposed over this sculpture. Ventral surface of head shining, with weak rugae. Pronotal neck areolate. Pronotum with weak areolate ground sculpture arranged into longitudinal rows and separated by superficial costulae. Anepisternum and katepisternum shining on their anterior halves, transitioning into weak costulae posteriorly. Propodeum with stronger costulae laterally; propodeal declivity weakly areolate. Mesoscutum with costulae over weak areolate ground sculpture surrounding a smooth and shining central strip which extends over half of the sclerite from the anterior margin; mesoscutum also with smooth and shining patches laterally. Mesoscutellum smooth and shining medially, surrounded by weak costulae and areolae. Femora smooth and shining, with traces of weak areolate sculpture distally. Petiole with weak areolate sculpture laterally, and on the dorsoposterior surface of the node; dorsal surface of the peduncle and the anterior face of the node shining through weak sculpture. Postpetiole smooth and shining anteriorly, areolate laterally and on the posterior half. Gaster smooth and shining, without spectral iridescence. Surface of the first gastral sternite smooth and shining.

Setae: antennal scapes and funiculi with short, adpressed pilosity. Dorsum of head, pronotum, waist segments, and gaster with moderately abundant, erect, blunt-tipped setae, the longest of which are about a third of the width of the compound eye. Short, sparse pubescence present over the entire body, but difficult to detect against the lightly colored integument.

Color: predominantly yellow, with the head capsule (excluding clypeus), anepisternum, median of the mesoscutellum, and posterior halves of the gastral tergite dark brown.

**Male:** Unknown.

**Etymology:** From the headgear used by British troops during the Battle of Balaclava during the Crimean war, in reference to the distinctive coloration of the cephalic integument.

**Comments:** *Temnothorax balaclava* sp. nov. is known only from low elevation xerophytic forest in Jaragua National Park in southwestern Dominican Republic, where partial nests were collected from partially buried wood, leaf litter, and soil. Other details of the biology of this species are unknown but are probably similar to other members of the terricolous pan-Caribbean *pulchellus* group. This taxon is part of a species complex with *T. bahoruco* sp. nov., *T. ciferrii*, and *T. wilsoni* sp. nov., all of which have only been collected from allopatric populations in mountains and low-lying dry forest from the southern parts of Hispaniola. The worker is morphologically quite similar to *T. bahoruco* sp. nov. and *T. wilsoni* sp. nov., differing primarily in the conformation of the clypeus, coloration, and sculptural details.

*Temnothorax ciferrii* (Menozzi & Russo, 1930)

**Figure 139:**
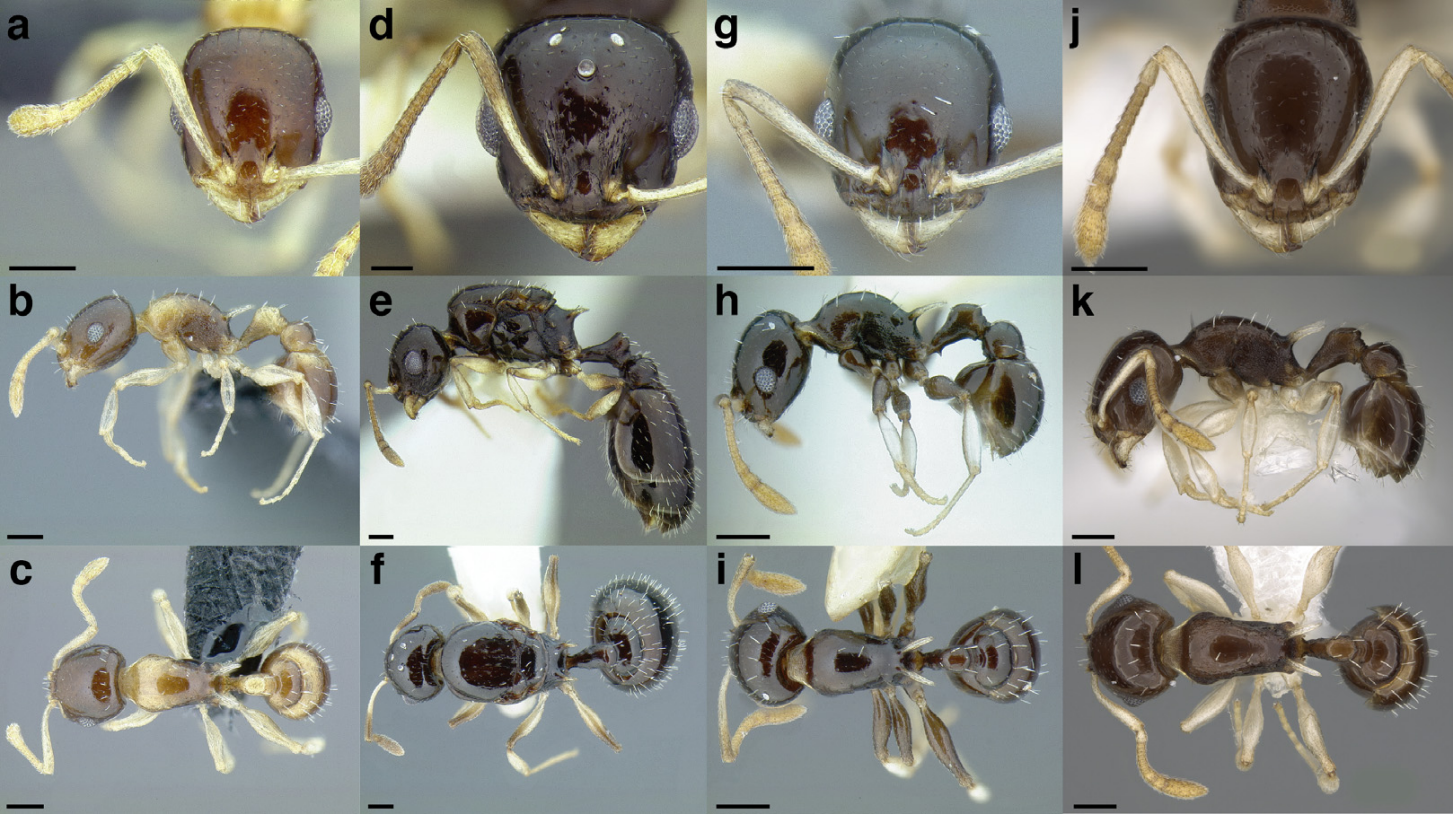
*Temnothorax ciferrii*. (A–C) Worker (CASENT0758829). (A) Full-face view. (B) Profile view. (C) Dorsal view. (D–F) Gyne (MCZENT00521295). (D) Full-face view. (E) Profile view. (F) Dorsal view. Morphological variation: (G–I) worker with dark femora (MCZENT00510533). (G) Full-face view. (H) Profile view. (I) Dorsal view. (J–L) Worker with light femora (CASENT0916003). (J) Full-face view. (K) Profile view. (L) Dorsal view. Scale bars 0.2 mm.

Distribution: Fig. 133E; worker, gyne & variability: Fig. 139.

*Macromischa (Antillaemyrmex) ciferrii* Menozzi & Russo, 1930: 160 fig. 5. Syntype workers. Cayos Siete Hermanos, Dominican Republic.

*Antillaemyrmex ciferrii* (Menozzi & Russo): Wheeler, 1931: 32. First combination in *Antillaemyrmex*.

*Leptothorax ciferrii* (Menozzi & Russo): Baroni Urbani, 1978: 433. First combination in *Leptothorax*.

*Temnothorax ciferrii* (Menozzi & Russo): Bolton, 2003: 271. First combination in *Temnothorax*.

**Type material not examined:** *Syntype workers:* DOMINICAN REPUBLIC: Monti Cristi: Isole Los Siete Hermanos [IEGG].

**Non-type material examined:** DOMINICAN REPUBLIC: Independencia: La Florida, ESE Jimani, S. Lago Limon, 45 m, 14 April 1992, M.A. Ivie, leaf litter (CASENT0916003) [UCDC]; Pedernales: Jaragua National Park: 17.79249°N 71.46385°W ± 18 m, 126 m, 30 March 2012, D. Lubertazzi#DL03412:001, dry forest, litter at base of large stump, 2 workers (MCZENT00510533) [MCZC]; same data as previous, except: 18.025710°N 71.647320°W ± 11 m, 92 m, 2 April 2012, D. Lubertazzi#DL03498:002, dry forest, soil/litter around and under stump, 1 worker (MCZENT00510534) [MCZC]; same data as previous, except: 18.073320°N 71.652030°W ± 120 m, 400 m, 27 March 2014, D. Lubertazzi#DR03769:001, transitional forest, Winkler sample 29, 1 dealate gyne (MCZENT00521295) [MCZC]; Valverde: 12 km S Mao, 25 July 1978, R.O. Schuster, 6 workers (CASENT0758330, CASENT0758828-CASENT0758830, CASENT0758875) [UCDC].

**Geographic range:** Low elevations of Hispaniola (Fig. 133E).

**Worker diagnosis:** *Temnothorax ciferrii* can be separated from all other species in the *salvini* clade by the following character combination: head in full face view with posterior margin flat to slightly convex; antennal scapes very long, surpassing the posterior margin of the head by about two times the maximum width of the antennal scape; compound eyes relatively small, with nine to eleven ommatidia in the longest row; in profile view, dorsum of mesosoma evenly, strongly convex; pronotal declivity indistinct; metanotal groove not impressed; propodeum not depressed below the level of the promesonotum; propodeum bearing standing setae dorsally; propodeal spines longer than the propodeal declivity, directed posteriorly, and weakly downcurved; hind femora weakly to moderately incrassate; petiolar node flat and elongate dorsally; transition of dorsal to posterior face of petiolar node marked by an angle; in dorsal view, apex of petiolar node narrower or slightly wider than the caudal cylinder of the petiole; postpetiole very broad: greater than or equal to 2.3 times the width of the petiole; dorsum of head smooth and shining; petiolar node with four erect setae dorsally; setae on head, mesosoma, waist segments and gaster erect, moderately long, sparse and blunt (never long and tapering); integument variously colored: either predominantly dark brown, with the antennae, pronotal neck, propodeal spines, and tarsi testaceous, mandibles and tibiae nearly white, or: femora, meso- and metacoxae light yellow in addition to the previous color scheme, or: pronotum, procoxa, petiolar node, and base of gaster in yellow in addition to the previous color scheme.

**Similar species:** *Temnothorax flavidulus, T. harlequina* sp. nov., *T. hippolytus* sp. nov., *T. magnabulla* sp. nov., *T. pulchellus* and *T. terricola*. The erect setae count of four on the dorsum of the petiolar node differentiates *T. ciferrii* from *T. terricola* and *T. hippolytus* sp. nov., which have only two along the posterior margin. The flat relatively small compound eyes, with nine to eleven ommatidia in the longest row, and dark integument separate *T. ciferrii* from *T. flavidulus*, which has 12 ommatidia in the longest row of the compound eye and is uniformly light yellow. The presence of erect setae on the propodeum distinguishes *T. ciferrii* from *T. pulchellus*. The strongly convex dorsal margin of the mesosoma and angulate transition between the dorsal and posterior faces of the petiolar node distinguishes *T. ciferrii* from *T. magnabulla* sp. nov., in which the mesosoma is flatter and the petiolar node transition is evenly rounded. *Temnothorax ciferrii* and *T. harlequina* sp. nov. can be separated most easily by the color of the integument: *T. harlequina* sp. nov. has a light yellow gaster, whereas the gaster of *T. ciferrii* is always dark brown, or brown with the anterior quarter of the first gastral tergite yellowish. The structure of the clypeus may also be used to separate the two species: *T. harlequina* sp. nov. has an evenly convex anterior margin of the clypeus, whereas *T. ciferrii* has a medially flattened anterior clypeal margin. The medial two pairs of setae on the anterior clypeus margin may also be used to separate *T. harlequina* sp. nov. and *T. ciferrii*: in *T. ciferrii*, the setae flanking the central pair are thickened to nearly twice the width of the central pair, whereas in *T. harlequina* sp. nov. they are nearly the same width.

**Worker measurements & indices (*n* = 9):** SL = 0.405–0.462 (0.435); FRS = 0.146–0.190 (0.172); CW = 0.482–0.548 (0.505); CWb = 0.437–0.501 (0.454); PoOC = 0.194–0.214 (0.202); CL = 0.505–0.576 (0.535); EL = 0.115–0.140 (0.127); EW = 0.094–0.108 (0.102); MD = 0.122–0.139 (0.132); WL = 0.532–0.630 (0.578); SPST = 0.210–0.277 (0.236); MPST = 0.202–0.235 (0.214); PEL = 0.251–0.297 (0.269); NOL = 0.133–0.182 (0.151); NOH = 0.079–0.101 (0.092); PEH = 0.149–0.181 (0.168); PPL = 0.138–0.184 (0.156); PPH = 0.179–0.207 (0.196); PW = 0.303–0.366 (0.332); SBPA = 0.123–0.155 (0.138); SPTI = 0.210–0.276 (0.228); PEW = 0.116–0.142 (0.132); PNW = 0.111–0.134 (0.122); PPW = 0.300–0.349 (0.319); HFL = 0.387–0.450 (0.418); HFWmax = 0.109–0.132 (0.120); HFWmin = 0.039–0.056 (0.045); CS = 0.690–0.789 (0.722); ES = 0.165–0.193 (0.178); SI = 91–104 (96); OI = 24–25 (25); CI = 82–87 (85); WLI = 122–131 (127); SBI = 28–33 (30); PSI = 39–45 (41); PWI = 229–270 (243); PLI = 146–187 (173); NI = 148–194 (164); PNWI = 83–110 (93); NLI = 48–62 (56); FI = 227–321 (268).

**Worker description:** In full-face view, head subquadrate, longer than broad (CI 82–87). Mandibles weakly striate, shining, and armed with five teeth: the apical-most well developed, followed by a less developed preapical tooth and three equally developed smaller teeth. Anterior clypeal margin entire and evenly rounded. Antennal scapes very long: when fully retracted, extending past the posterior margin of the head capsule by about two times the maximum width of the scape (SI 91–104). Antennae 12-segmented; antennal club of three segments, with the apical-most segment about as long as the preceding two in combination. Frontal carinae short, extending past the antennal toruli by about one and a half times the maximum width of the antennal scape. Compound eyes moderately protruding past the lateral margins of the head capsule. Lateral margin of head evenly convex, forming a continuous arc from the posterior of the head to the mandibular insertions. Posterior head margin weakly convex, rounding evenly into the lateral margins.

In profile view, compound eyes ovular and moderately large (OI 24–25), with 9 ommatidia in longest row. Pronotal declivity indistinct, neck and anterior face of pronotum forming a ~120° angle. Mesosoma strongly convex from where it joins the pronotal neck to the propodeal spines. Promesonotal suture extending from the posterior margin of the procoxal insertion only to the mesothoracic spiracle, which is well developed. Metanotal groove visible as a disruption of the sculpture laterally from where it arises between the mid- and hind coxae to where it ends in the poorly developed metathoracic spiracle. Propodeal spiracle poorly developed, directed posterolaterally, and separated from the propodeal declivity by about three spiracle diameters. Propodeal spines well developed and long (PSI 39–45), about one and a quarter times the length of the propodeal declivity, tapering evenly from the base, weakly downcurved, and acute. Propodeal declivity straight and flat, angled anteriorly so that it continues the even curve of dorsal surface of the mesosoma, forming a ~100° angle with the base of the propodeal spines. Propodeal lobes rounded and weakly developed. Metapleural gland bulla small, extending from the metacoxal insertion one quarter of the way to the propodeal spiracle. Petiole moderately long (PLI 146–187), without tubercles anterodorsally. Subpetiolar process in the form of a small, very acute tooth; ventral margin of petiole weakly concave posterior to it. Petiolar peduncle short: comprising roughly a third of the length of the petiole. Petiolar node robust: transition between peduncle and node marked by a rounded angle of ~150°, resulting in a weakly concave anterior node face; anterior face forming a ~120° angle with the dorsal face, which is long and weakly convex, nearly flat; dorsal face rounding evenly into the short posterior face, which forms a ~110° angle with the caudal cylinder. Postpetiole evenly rounded anteriorly, flattened dorsally, and weakly lobed ventrally.

In dorsal view, humeri not well developed: evenly rounding into the rest of the mesosoma; mesothoracic spiracles not protruding past the lateral margins of the mesosoma. Metanotal groove absent: mesonotum and propodeum completely fused and converging evenly to the bases of the propodeal spines. Propodeal spines closely approximated basally, and strongly diverging apically, their apices separated from each other by a little less than their length; negative space between them “U” shaped. Petiolar peduncle with spiracles slightly protruding past the lateral margins, peduncle not noticeably constricted anterior to them. Petiolar node evenly ovate and narrowest anteriorly, slightly wider than the peduncle, and evenly grading into the caudal cylinder, which is slightly wider than the node. Postpetiole very broad (PWI 229–270) and campaniform, articulating with nearly the entire anterior margin of the gaster. Anterior margin of the postpetiole broadly convex, with the corners evenly rounding into the lateral margins, which diverge to the angulate posterior corners; posterior margin broadly concave. Metafemur weakly to strongly incrassate (FI 227–321).

Sculpture: median clypeal carina present, extending posteriorly nearly to frontal triangle. Lateral clypeal lobes with additional, weaker carinae; ground sculpture smooth and shining. Antennal scapes shining through weak ground sculpture. Cephalic dorsum smooth and shining. Pronotal neck areolate. Mesosoma with weak rugulae on the lateral face of the pronotum, mesopleurae, metapleurae and propodeal declivity; weak areolate sculpture surrounding the metanotal groove laterally; otherwise smooth and shining. Femora smooth and shining. Petiole smooth and shining ventrally, with very weak areolate sculpture on the peduncle and the lateral surfaces of the petiolar node; the dorsal surface of the node smooth and shining. Postpetiole entirely smooth and shining, with weak areolate sculpture on the posterior quarter. Gaster smooth and shining, without spectral iridescence.

Setae: antennal scapes and funiculi with short, adpressed pilosity. Dorsum of head, pronotum, waist segments and gaster with sparse, erect, blunt-tipped setae, the longest of which are about the length of the compound eye. The head bears ~24, mesosoma ~14, petiole 4, postpetiole ~10, and first gastral tergite ~20 setae. Short, sparse pubescence present over the entire body.

Color: predominantly dark brown, but with the antennae, pronotal neck, propodeal spines, and tarsi testaceous; the mandibles and tibiae nearly white.

**Gyne measurements & indices (*n* = 1)**: SL = 0.552; FRS = 0.246; CW = 0.729; CWb = 0.68; PoOC = 0.259; CL = 0.667; EL = 0.209; EW = 0.165; MD = 0.144; WL = 1.141; SPST = 0.311; MPST = 0.299; PEL = 0.421; NOL = 0.225; NOH = 0.152; PEH = 0.272; PPL = 0.220; PPH = 0.332; PW = 0.696; SBPA = 0.350; SPTI = 0.310; PEW = 0.194; PNW = 0.194; PPW = 0.521; HFL = 0.653; HFWmax = 0.130; HFWmin = 0.052; CS = 1.014; ES = 0.292; SI = 81; OI = 29; CI = 102; WLI = 168; SBI = 51; PSI = 27; PWI = 269; PLI = 191; NI = 148; PNWI = 100; NLI = 53; FI = 250.

**Gyne description:** In full-face view, head subquadrate, slightly broader than long (CI 102). Mandibles weakly striate, shining, and armed with five teeth: the apical-most well developed, followed by a less developed preapical tooth and three equally developed smaller teeth. Anterior clypeal margin flat medially. Antennal scapes long: when fully retracted, surpassing the posterior margin of the head capsule by about one and a half times the maximum width of the antennal scape (SI 81). Antennae 12-segmented; antennal club of three segments, with the apical-most segment as long as the preceding two in combination. Frontal carinae long, extending past the antennal toruli by about five times the maximum width of the antennal scape. Compound eyes moderately protruding past the lateral margins of the head capsule. Lateral margin of head evenly convex, forming a continuous arc from the posterior of the head, but narrowing as they join the mandibular insertions. Posterior head margin very slightly convex, rounding evenly into the lateral margins.

In profile view, compound eyes teardrop-ovular and large (OI 29), with 16 ommatidia in the longest row. Mesoscutum rounded evenly anteriorly, covering the dorsal surface of the pronotum, and flat dorsally. Mesoscutellum on the same plane as the mesoscutum, and evenly rounded posteriorly. Posterior margin of metanotum extending slightly past the posterior margin of the mesoscutum. Propodeal spiracle poorly developed, directed posterolaterally, and separated from the propodeal declivity by about 4 spiracle diameters. Propodeal spines stout and well developed, but short (PSI 27), about half as long as the propodeal declivity, tapering evenly from the base, directed posteriorly, very slightly downcurved, and acute. Metapleural gland bulla small, extending from the metacoxal insertion a quarter of the way to the propodeal spiracle. Propodeal declivity weakly concave, forming a ~90° angle with the base of the propodeal spines. Propodeal lobes rounded and weakly developed. Petiole moderately long (PLI 191), without tubercles anterodorsally. Subpetiolar process a small, very acute tooth, which grades evenly into the ventral margin of the petiole posteriorly. Petiolar peduncle short: petiolar node covering most of the petiolar dorsum. Petiolar node erect: transition between peduncle and node evenly rounded, resulting in a concave anterior node face; anterior face of node forming a sharp ~90° angle with the dorsal face, which even rounds into the posterior margin, which forms a rounded ~100° angle with the caudal cylinder. Postpetiole evenly rounded anterodorsally, flattened dorsally; ventral surface weakly lobed.

In dorsal view, mesoscutum covering pronotum anteriorly, but humeri visible laterally as rounded sclerites. Propodeal spines straight and directed posteriorly, not diverging, their apices separated by about one and a half times their length. Petiolar peduncle with spiracles slightly protruding past the lateral margins. Petiolar node trapezoidal and widest anteriorly, with the anterodorsal margin slightly emarginated. Petiolar node slightly narrower than the peduncle, and evenly grading into the caudal cylinder, which is roughly the same width as the node. Postpetiole very broad (PWI 269), somewhat anteroposteriorly compressed, and campaniform, articulating with most of the anterior margin of the gaster, leaving small, angulate margins on each side exposed. Anterior margin of the postpetiole flat, with corners evenly rounding into the lateral margins, which evenly diverge to the angulate posterior corners; posterior margin medially emarginate. Metafemur moderately incrassate (FI 250).

Sculpture: median clypeal carina present, extending posteriorly from the anterior clypeal margin to the antennal toruli; flanked by two equally strong carinae. Lateral clypeal lobes with additional weaker carinae; ground sculpture smooth and shining. Antennal scapes shining through very weak, indistinct areolate ground sculpture. Cephalic dorsum predominantly shining, but with weak costulate sculpture surrounding the frontal carinae, and rugose sculpture surrounding the compound eye, extending to the mandibular insertions. Pronotal neck strongly areolate. Pronotum smooth and shining with traces of weak areolate sculpture. Katepisternum smooth and shining; anepisternum mostly smooth and shining, but with dense areolate sculpture dorsally. Propodeum with dense areolate sculpture laterally, with superimposed rugae that continue onto the ventroposterior surface; otherwise propodeum smooth and shining. Mesoscutum predominantly smooth and shining, but with weak costulae posteromedially. Mesoscutellum smooth and shining. Femora smooth and shining, with traces weak areolate sculpture. Petiolar peduncle smooth and shining anteroventrally. Lateral surfaces of petiole with areolate-rugulose sculpture; dorsal surface entirely smooth and shining. Dorsal surface of postpetiole smooth and shining, with weak areolate sculpture on the posterior quarter, and on the lateral surfaces. Gaster smooth and shining, with very weak spectral iridescence. Surface of the first gastral sternite smooth and shining.

Setae: antennal scapes and funiculi with short, adpressed pilosity. Dorsum of head, pronotum, waist segments and gaster with sparse, erect, blunt-tipped setae, the longest of which are about a third of the width of the compound eye. Short, sparse pubescence present over the entire body.

Color: predominantly dark brown, but with the antennae, pronotal neck, propodeal spines, and tarsi testaceous; the mandibles and tibiae nearly white. Femora light brown distally, grading into light yellow proximally.

**Male:** Unknown.

**Etymology:** Patronym, in honor of the collector of the type series, R. Ciferri, Director of the Moca National Agronomic Station.

**Comments:** *Temnothorax ciferrii* is known from a few geographically distinct collections. The type series was collected from the Los Siete Hermanos islands, a group of small, uninhabited islands off the northern coast of Hispaniola near Monte Cristi, which are dominated by mangroves and xerophytic vegetation. Two additional a series of workers and a dealate gyne were collected from Jaragua National Park in southern Hispaniola. The details of the type collection are not known, but the latter two were made from soil in low lying dry forest and leaf litter in mid-elevation transitional forest, respectively. The biology of this species remains unknown but is likely to be similar to other members of the ground or litter nesting, pan-Caribbean *pulchellus* group. As noted by Baroni Urbani (1978), this species closely resembles *T. flavidulus*, which is known only from the type specimens collected from Manneville, Haiti. Baroni Urbani (1978) uses the conformation of the postpetiole as a character that distinguishes *T. flavidulus* from *T. ciferrii*, with *T. flavidulus* having more angulate anterior corners of the postpetiole. My observations of the types of *T. flavidulus* offer several more distinguishing characteristics, for example the eye of *flavidulus* is distinctly larger than that of *T. ciferrii*. *Temnothorax ciferrii* is part of a large complex of species endemic to the island of Hispaniola and its smaller outlying islands, including *T. flavidulus*, *T. wilsoni* sp. nov., *T. balaclava* sp. nov., and *T. bahoruco* sp. nov., none of which have been collected in sympatry. While it is possible to distinguish *T. ciferrii* from each of these taxa based on morphology, it remains to be seen whether this putative species is simply a variant of a widespread, morphologically diverse species. I have observed a couple of color variants of *T. ciferrii*, which were also noted by Baroni Urbani (1978): both have dark mesosomata, but the degree of infuscation on the legs varies (Fig. 139G–139L). In the first formal morphology-based phylogenetic work including this species, Fontenla Rizo (2000), while not including all of the taxa in Baroni Urbani’s *pulchellus* group, corroborated these relationships to some degree, with the caveat that *T. terricola* and *T. torrei* fell outside of this group. Combined molecular and morphological analyses (Prebus, in prep.) mostly agree with Fontenla’s analysis. The type specimens of *T. ciferrii* were not examined in this revision, so while the original description of *T. ciferrii* closely matches the characteristics of the more southern collections, these may be yet another, distinct member of the *flavidulus* complex.

*Temnothorax flavidulus* (Wheeler & Mann, 1914)

**Figure 140:**
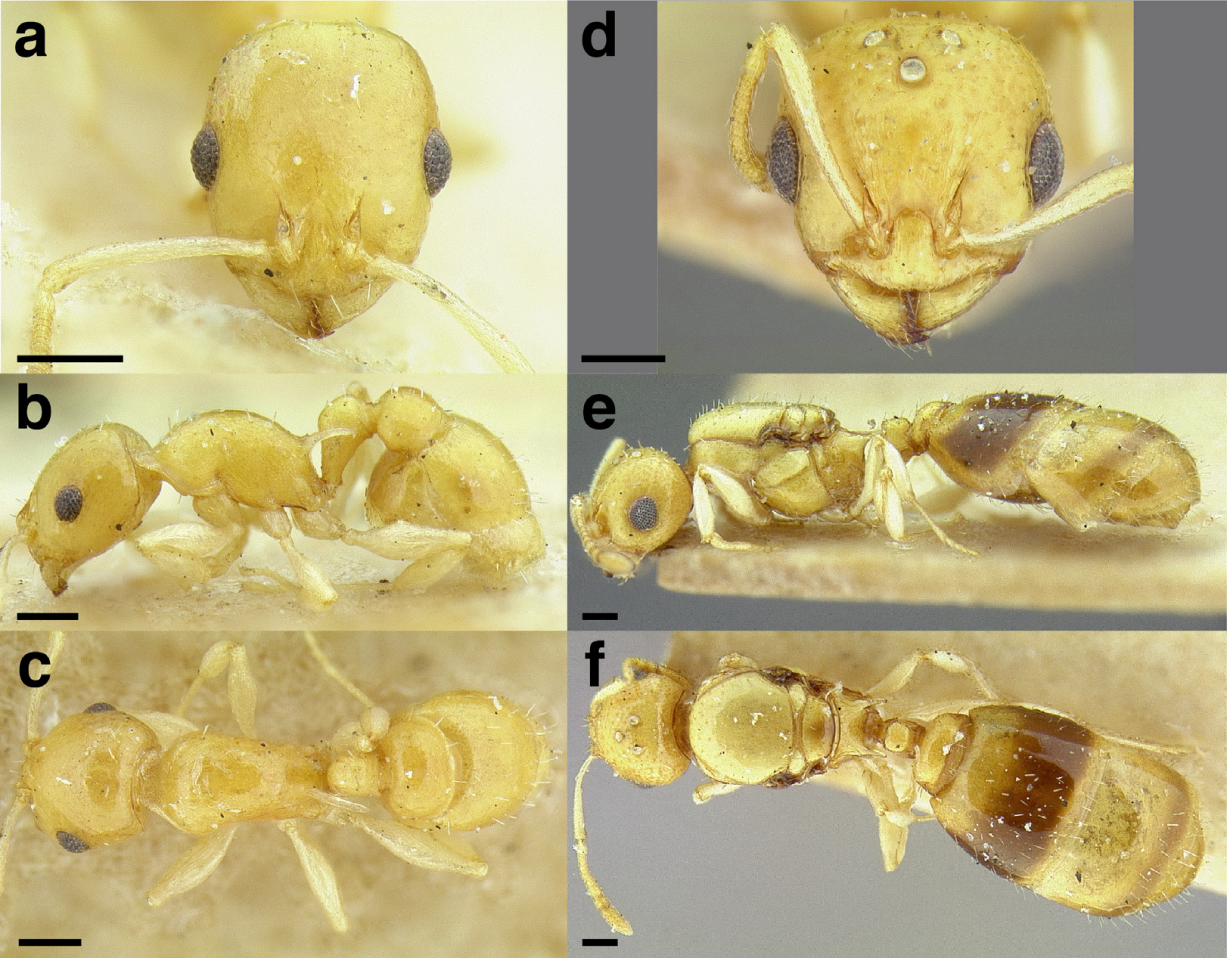
*Temnothorax flavidulus*. (A–C) Lectotype worker (MCZENT00021015). (A) Full-face view. (B) Profile view. (C) Dorsal view. (D–F) Paralectotype gyne (MCZENT00021015). (D) Full-face view. (E) Profile view. (F) Dorsal view. Scale bars 0.2 mm.

Distribution: Fig. 133F; worker & gyne: Fig. 140.

*Macromischa flavidula* Wheeler & Mann, 1914: 37 fig. 15. Syntype workers and gyne. Manneville, Haiti. One syntype worker here designated **lectotype**.

*Macromischa (Antillaemyrmex) flavidula* (Wheeler & Mann): Mann, 1920: 408. First combination in *Macromischa (Antillaemyrmex)*.

*Antillaemyrmex flavidulus* (Wheeler & Mann): Wheeler, 1931: 32. First combination in *Antillaemyrmex*.

*Leptothorax flavidulus* (Wheeler & Mann): Baroni Urbani, 1978: 441. First combination in *Leptothorax*.

*Temnothorax flavidulus* (Wheeler & Mann): Bolton, 2003: 271. First combination in *Temnothorax*.

**Type material examined:** *Lectotype worker of* Macromischa flavidula: HAITI: Ouest: Manneville, [no collection date], W.M. Mann, M.C.Z. Cotype 2-4 21015 (MCZENT00021015, top specimen, closest to pin).

*Paralectotype workers and gyne of* Macromischa flavidula: same pin as lectotype: 1 worker & 1 dealate gyne (MCZENT00021015, top specimen furthest from pin and bottom, respectively) [MCZC]; same data as lectotype: 1 worker (CASENT0758362) [USNM] 2 workers (CASENT0758361) [USNM].

**Geographic range:** Low elevations of Hispaniola (Fig. 133F).

**Worker diagnosis:** *Temnothorax flavidulus* can be separated from all other species in the *salvini* clade by the following character combination: compound eyes relatively large, with twelve ommatidia in the longest row; in profile view, dorsum of mesosoma evenly, strongly convex; pronotal declivity indistinct; metanotal groove not impressed; propodeum not depressed below the level of the promesonotum; propodeum bearing standing setae dorsally; propodeal spines longer than the propodeal declivity, directed posteriorly, and weakly downcurved; hind femora weakly to moderately incrassate; petiolar node flat and elongate dorsally; transition of dorsal to posterior face of petiolar node marked by an angle; in dorsal view, apex of petiolar node narrower or slightly wider than the caudal cylinder of the petiole; postpetiole very broad: greater than or equal to 2.2 times the width of the petiole; dorsum of head smooth and shining; petiolar node with four erect setae dorsally; setae on head, mesosoma, waist segments and gaster erect, moderately long, sparse and blunt (never long and tapering); integument predominantly light yellow.

**Similar species:** *Temnothorax agavicola* sp. nov., *T. ciferrii, T. harlequina* sp. nov., *T. hippolytus* sp. nov., *T. magnabulla* sp. nov., *T. pulchellus* and *T. terricola*. The erect setae count of four on the dorsum of the petiolar node differentiates *T. flavidulus* from *T. terricola* and *T. hippolytus* sp. nov., which have only two along the posterior margin. The flat relatively large compound eyes, with twelve ommatidia in the longest row, and light integument separate *T. flavidulus* from *T. ciferrii*, which has nine to eleven ommatidia in the longest row of the compound eye and is variously colored, but never light yellow. The presence of erect setae on the propodeum distinguishes *T. flavidulus* from *T. pulchellus*. The strongly convex dorsal margin of the mesosoma and angulate transition between the dorsal and posterior faces of the petiolar node distinguishes *T. flavidulus* from *T. magnabulla* sp. nov., in which the mesosoma is flatter and the petiolar node transition is evenly rounded. *Temnothorax flavidulus* and *T. harlequina* sp. nov. can be separated most easily by the color of the integument: *T. harlequina* sp. nov. has a light yellow gaster, which contrasts with a darker mesosoma and head, whereas *T. flavidulus* is uniformly yellow. The medial two pairs of setae on the anterior clypeus margin may also be used to separate *T. harlequina* sp. nov. and *T. flavidulus*: in *T. flavidulus*, the setae flanking the central pair are thickened to nearly twice the width of the central pair, whereas in *T. harlequina* sp. nov. they are nearly the same width. *Temnothorax flavidulus* may also be confused with *T. agavicola* sp. nov., but the head sculpture of this species is areolate, in contrast the smooth and shining sculpture of *T. flavidulus*.

**Worker measurements & indices (*n* = 2):** SL = 0.427–0.450 (0.442); FRS = 0.160–0.185 (0.170); CW = 0.492–0.529 (0.506); CWb = 0.444–0.472 (0.454); PoOC = 0.182–0.206 (0.195); CL = 0.530–0.552 (0.537); EL = 0.134–0.145 (0.140); EW = 0.102–0.117 (0.109); MD = 0.123–0.143 (0.138); WL = 0.572–0.604 (0.596); SPST = 0.236–0.272 (0.256); MPST = 0.201–0.249 (0.221); PEL = 0.261–0.302 (0.278); NOL = 0.142–0.176 (0.155); NOH = 0.100–0.111 (0.104); PEH = 0.165–0.185 (0.173); PPL = 0.151–0.194 (0.170); PPH = 0.202–0.229 (0.212); PW = 0.338–0.350 (0.342); SBPA = 0.147–0.164 (0.151); SPTI = 0.231–0.26 (0.240); PEW = 0.130–0.155 (0.139); PNW = 0.136–0.156 (0.143); PPW = 0.325–0.351 (0.342); HFL = 0.405–0.471 (0.433); HFWmax = 0.104–0.132 (0.122); HFWmin = 0.041–0.054 (0.047); CS = 0.712–0.748 (0.723); ES = 0.189–0.201 (0.194); SI = 93–101 (98); OI = 26–27 (27); CI = 83–86 (85); WLI = 124–136 (131); SBI = 32–35 (33); PSI = 41–45 (43); PWI = 223–262 (247); PLI = 156–180 (164); NI = 131–176 (150); PNWI = 91–120 (104); NLI = 51–60 (56); FI = 231–298 (262).

**Worker description:** In full-face view, head subquadrate, longer than broad (CI 83–86). Mandibles weakly striate, shining, and armed with five teeth: the apical-most well developed, followed by a smaller preapical tooth, which is followed by three equally developed smaller teeth. Anterior clypeal margin entire and evenly rounded. Antennal scapes moderately long: when fully retracted, extending past the posterior margin of the head capsule by about the maximum width of the scape (SI 93–101). Antennae 12-segmented; antennal club of three segments, with the apical-most segment slightly longer than the preceding two in combination. Frontal carinae moderately long, extending past the antennal toruli by about three times the maximum width of the antennal scape. Compound eyes moderately protruding past the lateral margins of the head capsule. Lateral margin of head evenly convex, forming a continuous arc from the posterior of the head to the mandibular insertions. Posterior head margin flat, rounding evenly into the lateral margins.

In profile view, compound eyes ovular and moderately large (OI 26–27), with 12 ommatidia in longest row. Pronotal declivity indistinct, neck and anterior face of pronotum forming a ~120° angle. Mesosoma evenly rounded from where it joins the pronotal neck to the propodeal spines. Promesonotal suture extending from the posterior margin of the procoxal insertion only to the mesothoracic spiracle, which is well developed. Metanotal groove visible as a disruption of the sculpture laterally from where it arises between the mid- and hind coxae to where it ends in the poorly developed metathoracic spiracle. Propodeal spiracle poorly developed, directed posterolaterally, and separated from the propodeal declivity by about three spiracle diameters. Propodeal spines well developed and long (PSI 41–45), about one and a half times as long as the propodeal declivity, tapering evenly from the base, weakly downcurved, and acute. Propodeal declivity straight and flat, forming a rounded ~90° angle with the base of the propodeal spines. Propodeal lobes rounded and weakly developed. Metapleural gland bulla small, extending from the metacoxal insertion a quarter of the way to the propodeal spiracle. Petiole moderately long (PLI 156–180), without tubercles anterodorsally. Subpetiolar process in the form of a small, acute tooth; ventral margin of petiole weakly concave posterior to it. Petiolar peduncle short: petiolar node covering most of the petiolar dorsum. Petiolar node robust: grading evenly into the petiolar peduncle anteriorly, resulting in a weakly concave anterior node face; anterior margin forming a ~120° angle with the dorsal face, which is long and weakly convex, nearly flat; dorsal face forming a ~110° angle with the short posterior declivity. Postpetiole evenly rounded anteriorly, flattened dorsally, and weakly lobed ventrally.

In dorsal view, humeri developed and distinct: evenly rounded and wider than the rest of the mesosoma; mesothoracic spiracles weakly protruding past the lateral margins of the mesosoma, visible as slight angles where the pronotum meets the mesonotum. Metanotal groove absent: mesonotum and propodeum completely fused and converging evenly to the bases of the propodeal spines. Propodeal spines broadly approximated basally and diverging apically, their apices separated from each other by about two thirds their length; negative space between them “U” shaped. Petiolar node evenly ovate and narrowest anteriorly, but transition from dorsal to anterior face indistinct; node slightly wider than the peduncle, and evenly grading into the caudal cylinder, which is slightly wider than the node. Postpetiole very broad (PWI 223–262) and campaniform, articulating with nearly the entire anterior margin of the gaster. Anterior margin of the postpetiole broadly convex, nearly flat, with the corners evenly rounding into the lateral margins, which diverge to the angulate posterior corners; posterior margin broadly concave, nearly flat. Metafemur weakly to moderately incrassate (FI 231–298).

Sculpture: median clypeal carina present, extending posteriorly nearly to the level of the antennal toruli, and flanked on either side by two equally strong carinae. Lateral clypeal lobes with additional, weaker carinae; ground sculpture smooth and shining. Antennal scapes shining through weak areolate ground sculpture. Cephalic dorsum smooth and shining, with weak areolate sculpture on the posterolateral surface of the head, behind the compound eyes. Pronotal neck areolate. Mesosoma with weak and indistinct sculpture on the posterodorsal surface of the pronotum, weak costulae on the lateral margin of the pronotum, and weak areolate sculpture on the mesopleurae, metapleurae and lateral surfaces of the propodeum; otherwise smooth and shining. Femora smooth and shining through weak areolate sculpture. Petiole smooth and shining ventrally, with very weak areolate sculpture on all other surfaces. Postpetiole entirely smooth and shining, with weak areolate sculpture on the posterior quarter. Gaster smooth and shining, without spectral iridescence.

Setae: antennal scapes and funiculi with short, adpressed pilosity. Dorsum of head, pronotum, waist segments and gaster with sparse, erect, blunt-tipped setae, the longest of which are about the width of the compound eye. The head bears ~12, mesosoma ~8, petiole 4, postpetiole ~6, and first gastral tergite ~10 setae. Short, sparse pubescence present over the entire body, but difficult to detect against the lightly colored integument.

Color: entirely light yellow, except for the slightly infuscated masticatory margin of the mandible.

**Gyne measurements & indices (*n* = 1):** SL = 0.536; FRS = 0.248; CW = 0.723; CWb = 0.647; PoOC = 0.249; CL = 0.675; EL = 0.219; EW = 0.179; MD = 0.148; WL = 1.114; SPST = 0.253; MPST = 0.245; PEL = 0.363; NOL = 0.216; NOH = 0.137; PEH = 0.274; PPL = 0.208; PPH = 0.321; PW = 0.704; SBPA = 0.334; SPTI = 0.328; PEW = 0.212; PNW = 0.206; PPW = 0.514; HFL = 0.594; HFWmax = 0.146; HFWmin = 0.058; CS = 0.985; ES = 0.309; SI = 83; OI = 31; CI = 96; WLI = 172; SBI = 52; PSI = 23; PWI = 242; PLI = 175; NI = 158; PNWI = 97; NLI = 60; FI = 252.

**Gyne description:** In full-face view, head subquadrate, slightly broader than long (CI 96). Mandibles weakly striate, shining, and armed with five small teeth, with the apical-most well developed. Anterior clypeal margin weakly emarginated medially. Antennal scapes moderately long: when fully retracted, surpassing the posterior margin of the head capsule by about the maximum width of the antennal scape (SI 83). Antennae 12-segmented; antennal club of three segments, with the apical-most segment slightly longer than the preceding two in combination. Frontal carinae long, extending past the antennal toruli by about three times the maximum width of the antennal scape. Compound eyes moderately protruding past the lateral margins of the head capsule. Lateral margin of head evenly convex, forming a continuous arc from the posterior of the head, but narrowing as they join the mandibular insertions. Posterior head margin very slightly convex, rounding evenly into the lateral margins.

In profile view, compound eyes ovular and large (OI 31), with 18 ommatidia in longest row. Mesoscutum rounded evenly anteriorly, covering the dorsal surface of the pronotum, and flat dorsally. Mesoscutellum on the same plane as the mesoscutum, and evenly rounded posteriorly; not overhanging the metanotum. Propodeal spiracle well developed, directed posterolaterally, and separated from the propodeal declivity by about four spiracle diameters. Propodeal spines stout and well developed, but short (PSI 23), about half as long as the propodeal declivity, tapering evenly from the base, directed posteriorly, very slightly downcurved, and acute. Propodeal declivity straight and flat, forming a rounded ~90° angle with the base of the propodeal spines. Propodeal lobes rounded and weakly developed. Petiole moderately long (PLI 175), without tubercles anterodorsally where it articulates with the mesosoma. Subpetiolar process in the form of a small, very acute tooth; ventral margin of petiole weakly concave posterior to it. Petiolar peduncle short: petiolar node covering most of the petiolar dorsum. Petiolar node robust and subquadrate: transition between peduncle and node evenly rounded, resulting in a very slightly concave anterior node face; anterior face of node forming a sharp ~90° angle with the dorsal face; dorsal face nearly flat, forming a rounded ~90° angle with the posterior face; posterior face forms a ~90° angle with the caudal cylinder. Postpetiole evenly rounded anterodorsally, flattened dorsally, ventral surface weakly lobed.

In dorsal view, mesoscutum covering pronotum anteriorly, but humeri visible laterally as rounded sclerites. Propodeal spines weakly diverging medially, their apices separated from each other by about one and a half times their length. Petiolar peduncle with spiracles very slightly protruding past the lateral margins. Petiolar node trapezoidal and widest anteriorly, and weakly emarginated anterodorsally; node slightly broader than the peduncle, and evenly grading into the caudal cylinder, which is slightly narrower than the node. Postpetiole very broad (PWI 242), somewhat anteroposteriorly compressed, and campaniform, articulating with most of the anterior margin of the gaster, leaving small, angulate margins on each side exposed. Anterior margin of the postpetiole broadly concave, with corners evenly rounding into the lateral margins, which evenly diverge to the angulate posterior corners; posterior margin broadly concave. Metafemur moderately incrassate (FI 252).

Sculpture: median clypeal carina present, extending posteriorly from the anterior clypeal margin to the level of the antennal toruli; flanked by two equally strong carinae. Lateral clypeal lobes with additional weaker carinae; ground sculpture smooth and shining. Antennal scapes shining through very weak, indistinct areolate ground sculpture. Cephalic dorsum predominantly shining, but with weak costulae surrounding the frontal carinae, and rugose sculpture surrounding the compound eye, extending to the mandibular insertions. Pronotal neck areolate. Pronotum smooth and shining with traces of weak areolate sculpture. Katepisternum smooth and shining; anepisternum mostly smooth and shining, but with dense areolate sculpture dorsally. Propodeum with dense areolate sculpture laterally, with superimposed rugae that continue onto the ventro-posterior surface; otherwise the propodeum smooth and shining. Mesoscutum predominantly smooth and shining, but with weak costulae posteromedially. Mesoscutellum smooth and shining. Femora smooth and shining, with traces weak areolate sculpture. Peduncle of petiole smooth and shining anteroventrally. Lateral surfaces of petiole with areolate-rugulose sculpture; dorsal surface entirely smooth and shining. Dorsal surface of postpetiole smooth and shining, with weak areolate sculpture on the posterior quarter, and on the lateral surfaces. Gaster smooth and shining, with very weak spectral iridescence. Surface of the first gastral sternite smooth and shining.

Setae: antennal scapes and funiculi with short, adpressed pilosity. Dorsum of head, pronotum, waist segments and gaster with sparse, erect, blunt-tipped setae, the longest of which are about a third of the width of the compound eye. Short, sparse pubescence present over the entire body, but difficult to detect against the lightly colored integument.

Color: entirely light yellow, except for the slightly infuscated posteromedial portion of the mesoscutellum, posterior two thirds of the first gastral tergite, and masticatory margin of the mandible.

**Male:** Unknown.

**Etymology:** Morphological, from the Latin flavidulus (= yellowish), in reference to the predominantly yellow integument of this species.

**Comments:** *Temnothorax flavidulus* is only known from the type specimens, collected from under a stone in Manneville, Haiti, which is contemporarily known as Thomazeau. This locality lies in the Hispaniolan rift valley near Étang Saumâtre, a large, brackish lake. Thomazeau lies in an arid part of the rift valley, and the surrounding area is dominated by dry forest and dry steppe. The biology of this species remains unknown but is likely to be similar to other members of the terricolous *pulchellus* group. The larger eyes and lightly colored integument are typical of other xerophilic *Temnothorax*. A member of the *pulchellus* group, sensu Baroni Urbani (1978) and Fontenla Rizo (2000). As noted by Baroni Urbani (1978), this species closely resembles *T. ciferrii*, which is known only from a few collections in xerophytic habitats of the Dominican Republic. Baroni Urbani (1978) uses the conformation of the postpetiole as a character that distinguishes *flavidulus* from *ciferrii*, with *flavidulus* having more angulate anterior corners of the postpetiole. The depiction of the postpetiole in the Baroni Urbani (1978) is a bit misleading, though: the anterior corners of the postpetiole are not quite as squared as in the drawing, especially when all of the worker specimens are considered. *Temnothorax flavidulus* is part of a large complex of species endemic to the island of Hispaniola and its smaller outlying islands, including *T. bahoruco* sp. nov., *T. balaclava* sp. nov., *T. ciferrii*, *T. harlequina* sp. nov., *T. wilsoni* sp. nov., and none of which have been collected in sympatry. While it is possible to distinguish *T. flavidulus* from each of these taxa based on morphology, it remains to be seen whether this putative species is simply a variant of a widespread, morphologically diverse species.

***Temnothorax harlequina* sp. nov.**

**Figure 141:**
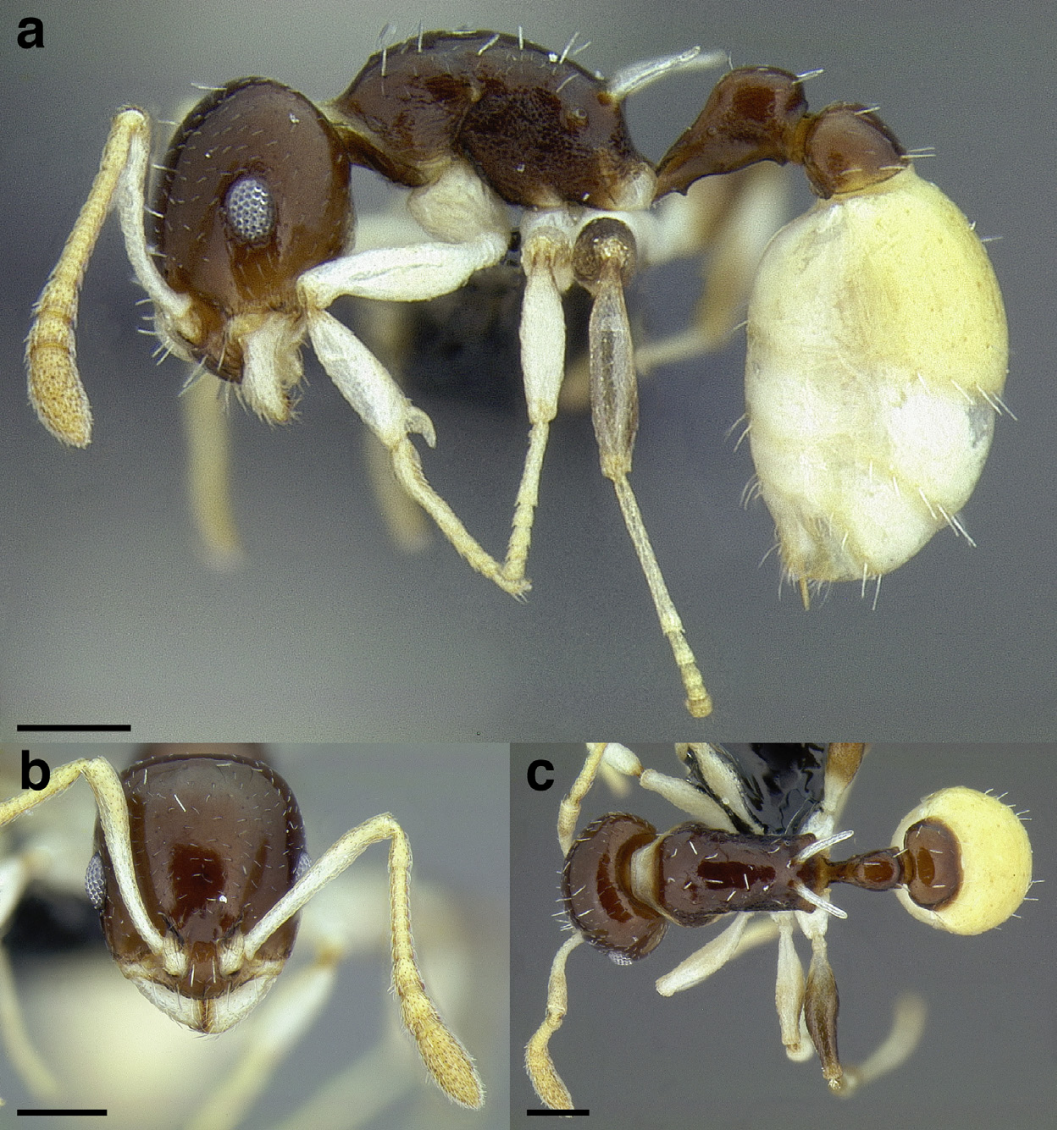
*Temnothorax harlequina* sp. nov. holotype worker (CASENT0756091). (A) Profile view. (B) Full-face view. (C) Dorsal view. Scale bars 0.2 mm.

Distribution: Fig. 133G; worker: Fig. 141.

**Type material examined:** *Holotype worker:* DOMINICAN REPUBLIC: La Altagracia: Parque Nacional del Este, Caseta de Guaraguao, 18.32715°N 68.80733°W, 10 m, 30 March 2004, S.P. Cover#SPCDR-151, evergreen coastal forest 5-10 m tall on coralline rock, relatively undisturbed native vegetation, nest fragment in dead, dry stick 2 cm in diameter on litter surface in shade; dense, shady tree clump to 6 m tall (CASENT0756091) [MCZC].

*Paratype workers:* same data as holotype, 1 worker (CASENT0758822) [MCZC] 1 worker (CASENT0758823) [MCZC] 1 worker (CASENT0758824) [CASC]; same data as holotype, except: S.P. Cover#SPC DR-152, evergreen coastal forest 5–10 m tall on coralline rock; relatively undisturbed native vegetation; nest fragment in dead, dry stick 2 cm in diameter on litter surface in shade; dense, shady tree clump to 6 m tall, 1 worker (CASENT0756156) [MCZC] 1 worker (CASENT0758709) [UCDC] 1 worker (CASENT0758825) [USNM] 1 worker (CASENT0758826) [MNHNSD] 1 worker (CASENT0758827) [LACM] 1 worker (MCZENT00531354) [MCZC] 1 worker (MCZENT00531355) [MCZC].

**Geographic range:** Low elevations of Hispaniola (Fig. 133G).

**Worker diagnosis:** *Temnothorax harlequina* sp. nov. can be separated from all other species in the *salvini* clade by the following character combination: head in full face view with posterior margin slightly convex; antennal scapes moderately long, surpassing the posterior margin of the head by about the maximum width of the antennal scape; anterior margin of the clypeus evenly convex; compound eyes relatively small, with eleven ommatidia in the longest row; in profile view, dorsum of mesosoma evenly, strongly convex; pronotal declivity indistinct; metanotal groove not impressed; propodeum not depressed below the level of the promesonotum; propodeum bearing standing setae dorsally; propodeal spines longer than the propodeal declivity, directed posteriorly, and slightly upturned; hind femora weakly to moderately incrassate; petiolar node flat and elongate dorsally; transition of dorsal to posterior face of petiolar node marked by a rounded angle; in dorsal view, apex of petiolar node narrower or slightly wider than the caudal cylinder of the petiole; postpetiole very broad: greater than or equal to 2.2 times the width of the petiole; dorsum of head smooth and shining; on the anterior margin of the clypeus, setae flanking the central pair are about the same width as the central pair; petiolar node with four erect setae dorsally; setae on head, mesosoma, waist segments and gaster erect, moderately long, sparse and blunt (never long and tapering); integument bicolored: head (except for the lateral lobes of the clypeus, mandibles and antennae), mesosoma (except for the pronotal neck and propodeal spines), metafemora (except for the proximalmost third) and waist segments medium brown; otherwise very light yellow.

**Similar species:** *Temnothorax ciferrii, T. flavidulus, T. hippolytus* sp. nov., *T. magnabulla* sp. nov., *T. pulchellus* and *T. terricola*. The bicolored integument of *T. harlequina* sp. nov., with its light yellow gaster, is unique within the *pulchellus* group and will distinguish *T. harlequina* sp. nov. from any similar species. Additionally, the erect setae count of four on the dorsum of the petiolar node differentiates *T. harlequina* sp. nov. from *T. terricola* and *T. hippolytus* sp. nov., which ha9*ve only two along the posterior margin. The flat relatively small compound eyes, with eleven ommatidia in the longest row, and bicolored integument separate *T. harlequina* sp. nov. from *T. flavidulus*, which has 12 ommatidia in the longest row of the compound eye and is uniformly light yellow. The presence of erect setae on the propodeum distinguishes *T. harlequina* sp. nov. from *T. pulchellus*. The strongly convex dorsal margin of the mesosoma and angulate transition between the dorsal and posterior faces of the petiolar node distinguishes *T. harlequina* sp. nov. from *T. magnabulla* sp. nov., in which the mesosoma is flatter and the petiolar node transition is evenly rounded. *Temnothorax ciferrii* and *T. harlequina* sp. nov. can be separated most easily by the color of the integument: *T. harlequina* sp. nov. has a light yellow gaster, whereas the gaster of *T. ciferrii* is always dark brown, or brown with the anterior quarter of the first gastral tergite yellowish. The structure of the clypeus may also be used to separate the two species: *T. harlequina* sp. nov. has an evenly convex anterior margin of the clypeus, whereas *T. ciferrii* has a medially flattened anterior clypeal margin. The medial two pairs of setae on the anterior clypeus margin may also be used to separate *T. harlequina* sp. nov. and *T. ciferrii*: in *T. ciferrii*, the setae flanking the central pair are thickened to nearly twice the width of the central pair, whereas in *T. harlequina* sp. nov. they are nearly the same width.

**Worker measurements & indices (*n* = 9):** SL = 0.438–0.504 (0.467); FRS = 0.170–0.220 (0.195); CW = 0.475–0.574 (0.533); CWb = 0.432–0.524 (0.483); PoOC = 0.204–0.235 (0.220); CL = 0.496–0.595 (0.555); EL = 0.113–0.138 (0.126); EW = 0.088–0.107 (0.100); MD = 0.112–0.159 (0.13); WL = 0.550–0.661 (0.608); SPST = 0.226–0.283 (0.255); MPST = 0.183–0.213 (0.195); PEL = 0.248–0.304 (0.278); NOL = 0.131–0.166 (0.151); NOH = 0.090–0.111 (0.102); PEH = 0.172–0.199 (0.185); PPL = 0.134–0.166 (0.153); PPH = 0.170–0.215 (0.192); PW = 0.323–0.393 (0.361); SBPA = 0.115–0.165 (0.141); SPTI = 0.243–0.301 (0.269); PEW = 0.116–0.150 (0.137); PNW = 0.127–0.170 (0.145); PPW = 0.284–0.355 (0.319); HFL = 0.403–0.485 (0.447); HFWmax = 0.112–0.132 (0.122); HFWmin = 0.042–0.057 (0.047); CS = 0.680–0.822 (0.751); ES = 0.157–0.190 (0.174); SI = 92–101 (97); OI = 23–25 (23); CI = 85–88 (87); WLI = 123–128 (125); SBI = 27–31 (29); PSI = 40–45 (42); PWI = 222–245 (234); PLI = 161–196 (182); NI = 128–169 (147); PNWI = 93–116 (105); NLI = 50–62 (54); FI = 230–300 (257).

**Worker description:** In full-face view, head subquadrate, longer than broad (CI 85–88). Mandibles weakly striate, shining and armed with five teeth: the apical-most well developed, followed by a less developed preapical tooth and three equally developed smaller teeth. Anterior clypeal margin entire and evenly rounded. Antennal scapes moderately long: when fully retracted, extending past the posterior margin of the head capsule by about the maximum width of the scape (SI 92–101). Antennae 12-segmented; antennal club of three segments, with the apical-most segment slightly longer than the preceding two in combination. Frontal carinae short, extending past the antennal toruli by about the maximum width of the antennal scape. Compound eyes moderately protruding past the lateral margins of the head capsule. Lateral margin of head evenly convex, forming a continuous arc from the posterior of the head to the mandibular insertions. Posterior head margin weakly convex, rounding evenly into the lateral margins.

In profile view, compound eyes ovular and moderately large (OI 23–25), with 11 ommatidia in longest row. Pronotal declivity indistinct, neck and anterior face of pronotum forming a ~130° angle. Mesosoma evenly convex from where it joins the neck to the propodeal spines. Promesonotal suture extending from the posterior margin of the coxal insertion only to the mesothoracic spiracle, which is well developed. Metanotal groove visible as a disruption of the sculpture laterally from where it arises between the mid- and hind coxae to where it ends in the poorly developed metathoracic spiracle. Propodeal spiracle well developed, directed posterolaterally, and separated from the propodeal declivity by about two and a half spiracle diameters. Propodeal spines well developed and long (PSI 40–45), about one and a half times the length of the propodeal declivity, tapering evenly from the base, straight for most of their length but slightly upturned at the tips, and acute. Propodeal declivity straight and flat, forming a ~110° angle with the base of the propodeal spines. Propodeal lobes rounded and weakly developed. Metapleural gland bulla small, extending from the metacoxal insertion a quarter of the way to the propodeal spiracle. Petiole moderately long (PLI 161–196), without tubercles anterodorsally. Subpetiolar process in the form of a very small, acute tooth; ventral margin of the petiole slightly concave posterior to it. Petiolar peduncle short: most of the dorsal surface of the petiole composed of the node. Petiolar node robust: transition between peduncle and node evenly rounded, resulting in a very weakly concave anterior node face; anterior face forming a ~120° angle with the dorsal face, which is long, weakly convex, and nearly flat; dorsal face forming a ~100° angle with the short posterior face, which forms a ~130° angle with the caudal cylinder. Postpetiole evenly rounded anteriorly, flat dorsally, and weakly lobed ventrally.

In dorsal view, humeri developed and distinct: evenly rounded and wider than the rest of the mesosoma; mesothoracic spiracles protruding past the lateral margins of the mesosoma, visible as slight angles where the pronotum meets the mesonotum. Metanotal groove absent: mesonotum and propodeum completely fused and converging evenly to the bases of the propodeal spines. Propodeal spines closely approximated basally and strongly diverging apically, their apices separated from each other by about their length; negative space between them in the form of a “V”. Petiolar peduncle with spiracles slightly protruding past the lateral margins, peduncle not noticeably constricted anterior to them. Petiolar node evenly rounded, slightly wider than the peduncle, and evenly grading into the caudal cylinder, which is slightly wider than the node. Postpetiole very broad (PWI 222–245) and campaniform, articulating with nearly the entire anterior margin of the gaster. Anterior margin of the postpetiole broadly convex, with the corners evenly rounding into the lateral margins, which diverge to the angulate posterior corners; posterior margin medially emarginate. Metafemur weakly to moderately incrassate (FI 230–300).

Sculpture: median clypeal carina present, extending posteriorly to the level of the antennal insertions. Lateral clypeal lobes with additional, weaker carinae; ground sculpture smooth and shining. Antennal scapes shining through weak ground sculpture. Cephalic dorsum smooth and shining, with weak sculpture near the anterior margins of the compound eye. Pronotal neck areolate. Mesosoma with lateral face of the pronotum, mesopleurae, metapleurae and propodeal declivity areolate; otherwise smooth and shining. Femora shining through weak areolate sculpture. Petiole smooth and shining ventrally, with very weak areolate sculpture on the peduncle and the lateral surfaces of the petiolar node; the dorsal surface of the smooth and shining. Postpetiole entirely smooth and shining. Gaster smooth and shining, without spectral iridescence.

Setae: antennal scapes and funiculi with adpressed pilosity. Dorsum of head, pronotum, waist segments and gaster with abundant, erect, blunt-tipped setae, the longest of which are about the width of the compound eye. The head bears ~22, mesosoma ~18, petiole 4, postpetiole ~10, and first gastral tergite ~8 setae. Pubescence present on the entire body but more difficult to detect on lightly colored surfaces.

Color: strongly bicolored: head (except for the lateral lobes of the clypeus, mandibles and antennae), mesosoma (except for the pronotal neck and propodeal spines), metafemora (except for the proximalmost third) and waist segments medium brown. Otherwise very light yellow.

**Gyne:** Unknown.

**Male:** Unknown.

**Etymology:** Morphological, from obsolete French: Harlequin, a pantomime fool with brightly colored clothing. The specific epithet refers to the similarity in color pattern to the sympatric ant species *Pheidole harlequina*.

**Comments:** *Temnothorax harlequina* sp. nov. is only known from the type locality, Parque Nacional del Este, where a nest was located in a dead, dry stick on top of the leaf litter in dry forest. Stefan Cover noted that where the workers were foraging there was a similarly bicolored salticid spider present. This color scheme is apparently common among the arthropods of Hispaniola and montane Mesoamerica: for example, among the ant species *Pheidole harlequina* Wilson, *Linepithema keiteli* (Forel), *Cephalotes flavigaster* de Andrade, *C. unimaculatus* (Smith), and an undescribed species of *Crematogaster* display a similar pattern on Hispaniola (see AntWiki); in montane Honduras, *Pheidole balatro* Longino, *P. zinnia* Longino, *Brachymyrmex bicolor* Ortiz-Sepúlveda et al., and *Tapinoma* JTL003 exhibit similar coloration (J. T. Longino, 2021, personal communications). The biology of this species remains unknown but is likely to be similar to other members of the ground or litter nesting, pan-Caribbean *pulchellus* group. While this species is closely related to other terricolous Antillean species, namely *T. albispinus*, *T. wettereri* sp. nov., *T. pulchellus, T. magnabulla* sp. nov., and *T. laticrus* sp. nov. (Prebus, in prep.). However, it is morphologically similar to members of the *flavidulus* complex, which are apparently restricted to the island of Hispaniola and its smaller outlying islands.

***Temnothorax hippolytus* sp. nov.**

**Figure 142:**
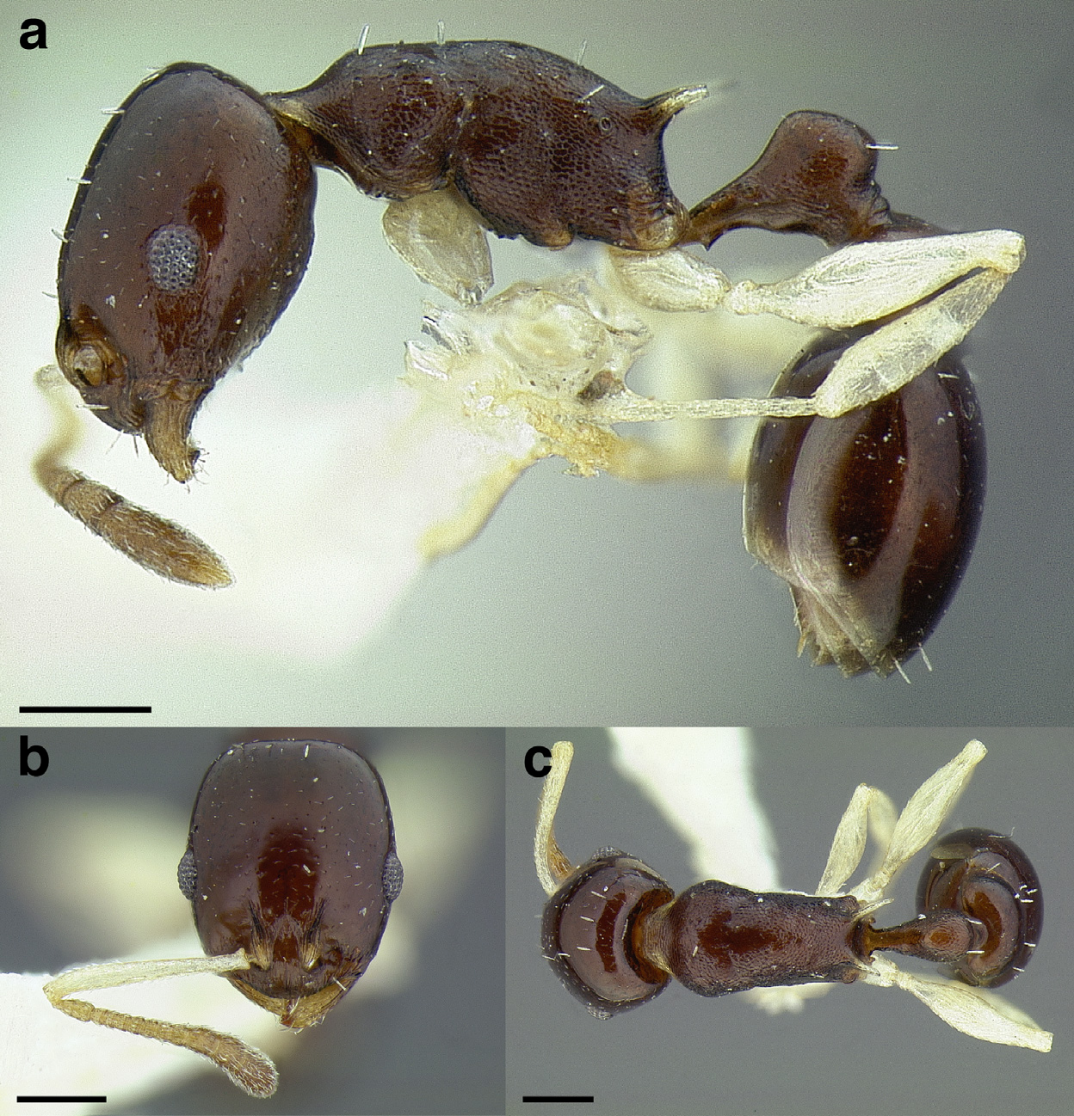
*Temnothorax hippolytus* sp. nov. holotype worker (LACMENT323469). (A) Profile view. (B) Full-face view. (C) Dorsal view. Scale bars 0.2 mm.

Distribution: Fig. 133H; worker: Fig. 142.

**Type material examined:** *Holotype worker:* CUBA: Camagüey: El Dieciocho, Francisco, March 1974, L.B. Zayas (LACMENT323469) [LACM].

**Geographic range:** Cuba (Fig. 133H).

**Worker diagnosis:** *Temnothorax hippolytus* sp. nov. can be separated from all other species in the *salvini* clade by the following character combination: head in full face view ovular; promesonotal suture deeply impressed and extending to the dorsal surface of the mesosoma; metanotal groove not impressed; propodeum not depressed below the level of the promesonotum; propodeum bearing standing setae dorsally; propodeal spines slightly shorter than the propodeal declivity, and directed posterodorsally; hind femora moderately incrassate; petiolar node subquadrate: dorsal face transitioning to posterior face through a rounded ~90° angle; postpetiole broad: about 2.5 times the width of the petiole; dorsum of head smooth and shining; petiolar node with only two erect setae dorsally; setae on head, mesosoma, waist segments and gaster erect, short, sparse and blunt (never long and tapering); integument predominantly dark brown; yellow antennae, mandibles, pronotal neck, propodeal spines, and legs.

**Similar species:** *Temnothorax ciferrii, T. harlequina* sp. nov., *T. terricola*, and *T. torrei. Temnothorax hippolytus* sp. nov. can be distinguished from *T. ciferrii* and *T. harlequina* sp. nov. by the setae count of two on the dorsum of the petiolar node, versus four. *Temnothorax hippolytus* sp. nov. is closely related to *T. torrei* and *T. terricola*, but the smooth and shining head sculpture can be used to separate *T. hippolytus* sp. nov. from *T. torrei*, in which the head sculpture is at least somewhat areolate. *Temnothorax hippolytus* sp. nov. can be differentiated from *T. terricola* by the ovular head shape (boxy in *T. terricola*), the deeply impressed promesonotal suture, and the more angulate transition between the dorsal and posterior faces of the petiolar node.

**Worker measurements & indices (*n* = 1):** SL = 0.467; FRS = 0.166; CW = 0.514; CWb = 0.467; PoOC = 0.253; CL = 0.583; EL = 0.114; EW = 0.088; MD = 0.141; WL = 0.635; SPST = 0.197; MPST = 0.207; PEL = 0.278; NOL = 0.137; NOH = 0.115; PEH = 0.193; PPL = 0.161; PPH = 0.218; PW = 0.346; SBPA = 0.162; SPTI = 0.240; PEW = 0.136; PNW = 0.156; PPW = 0.339; HFL = 0.470; HFWmax = 0.124; HFWmin = 0.049; CS = 0.759; ES = 0.158; SI = 100; OI = 21; CI = 80; WLI = 136; SBI = 35; PSI = 31; PWI = 249; PLI = 173; NI = 119; PNWI = 115; NLI = 49; FI = 253.

**Worker description:** In full-face view, head ovular, longer than broad (CI 80). Mandibles striate, shining, and armed with five teeth: the apical-most well developed, followed by a smaller preapical tooth, which is followed by three equally developed smaller teeth. Anterior clypeal margin entire and evenly rounded. Antennal scapes moderately long: when fully retracted, just reaching the posterior margin of the head capsule (SI 100). Antennae 12-segmented; antennal club of three segments, with the apical-most segment one and a half times longer than the preceding two in combination. Frontal carinae moderately long, extending past the antennal toruli by about two and a half times the maximum width of the antennal scape. Compound eye moderately protruding past the lateral margin of the head capsule. Lateral margin of head evenly convex, forming a continuous arc from the posterior of the head to the mandibular insertions. Posterior head margin flat, rounding evenly into the lateral margins.

In profile view, compound eyes ovular and small (OI 21), with 9 ommatidia in longest row. Pronotal declivity distinct, with neck and anterior face of pronotum forming a ~130° angle; anterior and dorsal faces of pronotum forming a rounded ~120° angle. Mesosoma arched from the pronotal declivity to the propodeal spines, but the pronotum is flat, giving the dorsal margin an angulate appearance. Promesonotal suture extending from the posterior margin of the procoxal insertion only to the dorsum of the mesosoma through the mesothoracic spiracle, which is poorly developed. Metanotal groove visible as a disruption of the sculpture laterally from where it arises between the mid- and hind coxae to where it ends in the poorly developed metathoracic spiracle. Propodeal spiracle poorly developed, directed posterolaterally, and separated from the propodeal declivity by about three and half spiracle diameters. Propodeal spines well developed and short (PSI 31), slightly shorter than the propodeal declivity, tapering evenly from the base, slightly upturned at the tips, and acute. Propodeal declivity straight and flat, forming a ~120° angle with the base of the propodeal spines. Propodeal lobes rounded and weakly developed. Metapleural gland bulla small, extending from the metacoxal insertion a third of the way to the propodeal spiracle. Petiole moderately long (PLI 173), with weakly developed denticles anterodorsally. Subpetiolar process in the form of a small, acute tooth, which grades gradually into the ventral margin of the petiole posteriorly, which is slightly concave posterior to it. Petiolar peduncle short: comprising about a third of the petiolar dorsum. Petiolar node robust and subquadrate: transition between peduncle and node evenly rounded, resulting in a slightly concave anterior node face; anterior face forming a ~110° angle with the dorsal face, which is weakly convex, nearly flat, and long; dorsal face forming a rounded ~90° angle with the posterior face, which forms a ~90° angle with the caudal cylinder. Postpetiole evenly rounded dorsally; ventral surface flat.

In dorsal view, humeri developed and distinct: evenly rounded and wider than the rest of the mesosoma; mesothoracic spiracles not protruding past the lateral margins of the mesosoma. Promesonotal suture nearly complete but obscured medially. Metanotal groove absent: mesonotum and propodeum completely fused and converging evenly to the bases of the propodeal spines. Propodeal spines broadly approximated basally and diverging apically, their apices separated from each other by a little more than their length; negative space between them “U” shaped. Petiolar peduncle with spiracles protruding past the lateral margins, peduncle slightly constricted anterior to them. Petiolar node evenly ovate and narrowest anteriorly; node slightly wider than the peduncle, and evenly grading into the caudal cylinder, which is slightly narrower than the node. Postpetiole very broad (PWI 249) and campaniform, articulating with nearly the entire anterior margin of the gaster. Anterior margin of the postpetiole flat, with the corners evenly rounding into the lateral margins, which diverge to the angulate posterior corners; posterior margin broadly concave. Metafemur moderately incrassate (FI 253).

Sculpture: median clypeal carina present, extending posteriorly nearly to the level of the antennal toruli, and flanked on either side by two equally strong carinae. Lateral clypeal lobes with additional, weaker carinae; ground sculpture weakly costulate. Antennal scapes shining through weak areolate ground sculpture. Cephalic dorsum smooth and shining, with weak areolate sculpture flanking the frontal carinae and on the posterolateral surface of the head, behind the compound eyes. Lateral surface of head weakly rugulose between the compound eye and the mandibular insertion. Ventral surface of head smooth and shining. Pronotal neck areolate-strigulate. Lateral face of pronotum areolate, but sculpture weaker on the posterior half. Mesopleurae, metapleurae, and lateral face of propodeum areolate, but sculpture weaker between the propodeal spiracle and the base of the propodeal spines, and on the propodeal declivity. Dorsum of mesosoma predominantly areolate, but a smooth and shining patch of sculpture present medially, that spans the promesonotal suture. Femora shining through weak areolate sculpture, which becomes stronger on the distal third. Petiole smooth and shining ventrally, with very weak areolate on the petiolar node; dorsal face of peduncle smooth and shining. Postpetiole entirely smooth and shining, with weak areolate sculpture on the posterior quarter. Gaster smooth and shining, with very weak spectral iridescence.

Setae: antennal scapes and funiculi with short, adpressed pilosity. Dorsum of head, pronotum, waist segments and gaster with sparse, erect, nearly clavate setae, the longest of which are about the width of the compound eye. The head dorsum bears ~16, mesosoma dorsum ~8, petiole 2, postpetiole ~4, and first gastral tergite ~8 setae. Short, sparse pubescence present over the entire body, but difficult to detect against the lightly colored integument.

Color: predominantly dark brown, nearly black, with yellow antennae, mandibles, pronotal neck, propodeal spines, and legs.

**Gyne:** Unknown.

**Male:** Unknown.

**Etymology:** Morphological, from the Ancient Greek ‘Hippolyta’, the queen of the Amazons, who wore a magical girdle given by her father Ares. This is a reference to the deep promesonotal impression, a feature not found in any closely related species.

**Comments:** *Temnothorax hippolytus* sp. nov. is known only from the holotype, which was collected in Camagüey province, Cuba. The specific locality data is obscure and needs verification. Nothing is known about the collection details, but this species, like all other documented species of the *pulchellus* group, is probably ground or leaf litter nesting. *Temnothorax hippolytus* sp. nov. bears two erect setae on the dorsum of the petiolar node, a feature it shares in common with other Caribbean endemics of the *pulchellus* group, to which it is most likely closely related, namely: *T. terricola* and *T. torrei*.

***Temnothorax laticrus* sp. nov.**

**Figure 143:**
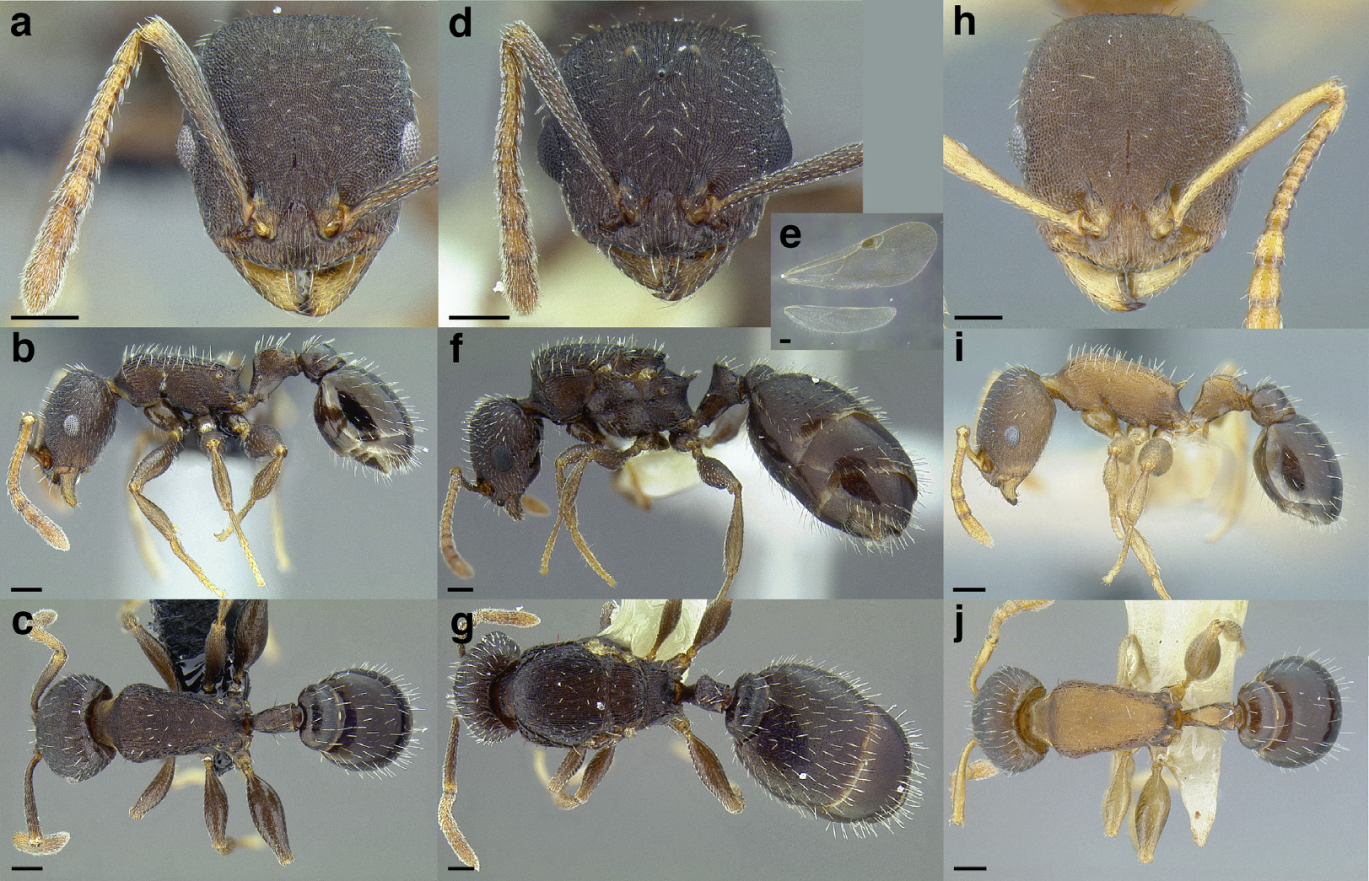
*Temnothorax laticrus* sp. nov. (A–C) Holotype worker (CASENT0758263). (A) Full-face view. (B) Profile view. (C) Dorsal view. (D–G) Paratype gyne (MCZENT00510556). (D) Full-face view. (E) Wings. (F) Profile view. (G) Dorsal view. (H–J) Bicolored form (MCZENT00594613). (H) Full-face view. (I) Profile view. (J) Dorsal view. Scale bars 0.2 mm.

Distribution: Fig. 133I; worker, gyne & variability: Fig. 143.

*Temnothorax* dr02 Prebus, 2017: 8. In phylogeny.

**Type material examined:** *Holotype worker:* DOMINICAN REPUBLIC: Pedernales: Jaragua National Park, 17.792206°N 71.46109°W ± 16 m, 125 m, 26 March 2012, D. Lubertazzi #DL03387, xeric scrub on limestone, half buried branch (3.5 cm diameter) and in soil (CASENT0758263) [MCZC].

*Paratype workers and gyne:* same data as holotype, 1 worker (CASENT0733974) [UCDC] 1 worker (CASENT4010181) [USNM] 1 worker (CASENT4010182) [MNHNSD] 1 worker (CASENT4010183) [CASC] 1 alate gyne (MCZENT00510556) [MCZC]; same data as holotype, except: #DL03388, xeric scrub on limestone, sifted soil and litter under half buried branch, 1 worker (CASENT4010184) [LACM] 1 worker (MCZENT00510558) [MCZC].

**Non-type material examined:** DOMINICAN REPUBLIC: Pedernales: 11 km E of Pedernales on route 44, 5 km N, Jaragua National Park, 18.025556°N 71.647500°W, 94 m, 1 April 2012, Gary D. Alpert, dry scrub forest, deep crack between large boulders, Davis sifter, leaf litter, 1 worker (MCZENT00594613) [MCZC]; Jaragua National Park, 17.930967°N 71.528600°W, 194 m, 27 March 2012, Gary D. Alpert#WP004, dry forest, nest in soil, open area, 1 worker (MCZENT00512198) [MCZC].

**Geographic range:** Low elevations of Hispaniola (Fig. 133I).

**Worker diagnosis:** *Temnothorax laticrus* sp. nov. can be separated from all other species in the *salvini* clade by the following character combination: anterior margin of the clypeus medially emarginate; metanotal groove not impressed; propodeum not depressed below the level of the promesonotum; propodeum bearing standing setae dorsally; propodeal spines shorter than the propodeal declivity, and directed posterodorsally; hind femora strongly incrassate; in dorsal view, apex of petiolar node laterally compressed, and much narrower than the caudal cylinder of the petiole; petiolar node with more than four erect setae dorsally (usually 12); postpetiole very broad: greater than or equal to 2.3 times the width of the petiole; setae on head, mesosoma, waist segments and gaster erect, moderately long, abundant and blunt (never long and tapering); integument variously colored: either predominantly dark brown, or bicolored, with the antennae, mandibles, mesosoma, legs, and petiole testaceous.

**Similar species:** It is difficult to imagine that this species would be confused with any other in the genus. *Temnothorax laticrus* sp. nov. vaguely resembles some of the more densely sculptured members of the *pastinifer* group, but it is easily separated from them by the short propodeal spines and laterally compressed petiolar node.

**Worker measurements & indices (*n* = 7):** SL = 0.625–0.690 (0.659); FRS = 0.246–0.273 (0.259); CW = 0.689–0.747 (0.721); CWb = 0.652–0.695 (0.675); PoOC = 0.300–0.319 (0.310); CL = 0.737–0.766 (0.753); EL = 0.147–0.172 (0.157); EW = 0.114–0.128 (0.122); MD = 0.180–0.201 (0.188); WL = 0.886–0.949 (0.917); SPST = 0.154–0.185 (0.171); MPST = 0.266–0.287 (0.278); PEL = 0.377–0.395 (0.386); NOL = 0.227–0.254 (0.242); NOH = 0.127–0.144 (0.133); PEH = 0.233–0.255 (0.249); PPL = 0.179–0.222 (0.206); PPH = 0.249–0.275 (0.261); PW = 0.487–0.517 (0.504); SBPA = 0.149–0.171 (0.159); SPTI = 0.142–0.176 (0.163); PEW = 0.177–0.196 (0.186); PNW = 0.136–0.174 (0.156); PPW = 0.412–0.447 (0.432); HFL = 0.576–0.649 (0.619); HFWmax = 0.217–0.237 (0.226); HFWmin = 0.055–0.060 (0.057); CS = 1.022–1.078 (1.051); ES = 0.204–0.234 (0.218); SI = 94–102 (98); OI = 19–22 (21); CI = 88–91 (90); WLI = 133–138 (136); SBI = 22–25 (23); PSI = 17–20 (19); PWI = 228–243 (233); PLI = 175–217 (188); NI = 165–194 (183); PNWI = 74–92 (84); NLI = 60–66 (63); FI = 369–420 (395).

**Worker description:** In full-face view, head subquadrate, longer than broad (CI 88–91). Mandibles weakly striate, shining, and armed with five teeth: the apical-most well developed, followed by a less developed preapical tooth and three equally-developed smaller teeth. Anterior clypeal margin entire and flat medially. Antennal scapes moderately long: when fully retracted, extending past the posterior margin of the head capsule by about the maximum width of the scape (SI 94–102). Antennae 12-segmented; antennal club of three segments, with the apical-most segment slightly longer than the preceding two in combination. Frontal carinae short, extending past the antennal toruli by about the maximum width of the antennal scape. Compound eyes moderately protruding past the lateral margins of the head capsule. Lateral margin of head evenly convex, forming a continuous arc from the posterior of the head to the mandibular insertions. Posterior margin of head flat, rounding evenly into the lateral margins.

In profile view, compound eyes ovular, moderately large (OI 19–22), with 12 ommatidia in longest row. Pronotal declivity indistinct, neck and anterior face of pronotum forming a ~120° angle. Pronotum evenly rounded from where it joins the neck to the dorsal margin. Mesosoma flat dorsally for most of its length, but propodeum descends to the base of the propodeal spines, beginning just dorsally to the propodeal spiracle. Promesonotal suture extending from the posterior margin of the procoxal insertion only to the mesothoracic spiracle, which is well developed. Metanotal groove visible as a disruption of the sculpture laterally from where it arises between the mid- and hind coxae to where it ends in the poorly developed metathoracic spiracle. Propodeal spiracle well developed, directed posterolaterally, and separated from the propodeal declivity by about three spiracle diameters. Propodeal spines poorly developed and small (PSI 17–20), about half the length of the propodeal declivity, tapering evenly from the base, straight, and blunt; represented as blunt teeth on the rounded angle where the dorsal surface of the propodeum meets the propodeal declivity. Propodeal declivity weakly concave, forming a ~110° angle with the base of the propodeal spines. Propodeal lobes weakly developed, with the dorsal margin slightly angulate. Metapleural gland bulla small, extending from the metacoxal insertion halfway to the propodeal spiracle. Petiole moderately long (PLI 175–217), without tubercles anterodorsally. Subpetiolar process in the form of a broad triangular tooth, forming a keel posteriorly which grades gradually into the ventral margin of the petiole, so that the process extends along about two thirds of the ventral margin. Petiolar peduncle short: most of the dorsal surface of the petiole composed of the node. Petiolar node robust: transition between peduncle and node evenly rounded, resulting in a slightly concave anterior node face; anterior face forming a ~110° angle with the dorsal face, which is long and weakly convex, nearly flat; dorsal face rounding evenly into the posterior margin, which forms a ~140° angle with the caudal cylinder. Postpetiole evenly rounded anteriorly, flat dorsally, and lobed ventrally.

In dorsal view, humeri moderately developed: evenly rounded and slightly wider than the rest of the mesosoma; mesothoracic spiracles weakly protruding past the lateral margins of the mesosoma, visible as slight angles where the pronotum meets the mesonotum. Metanotal groove absent: mesonotum and propodeum completely fused and converging evenly to the bases of the propodeal teeth. Propodeal teeth broadly approximated basally and diverging apically, their apices separated from each other by about twice their length; negative space between them an apically truncated “U”. Petiole strongly compressed laterally, without spiracles protruding past the lateral margins of the peduncle. Petiolar node roughly ovate: narrowest anteriorly and truncate, rounded posteriorly. Petiolar node slightly wider than the peduncle, and evenly grades into the caudal cylinder, which is slightly wider than the node. Postpetiole very broad (PWI 228–243) and campaniform, articulating with nearly the entire anterior margin of the gaster. Anterior margin of the postpetiole broadly concave, with the corners evenly rounding into the lateral margins, which diverge only slightly to the angulate posterior corners; posterior margin broadly concave. Metafemur strongly incrassate (FI 369–420).

Sculpture: clypeus with multiple (roughly 6) longitudinal carinae medially. Lateral lobes of clypeus with similar, weaker carinae; ground sculpture weakly areolate. Antennal scapes densely areolate, weakly shining. Cephalic dorsum areolate, with areolae arranged into parallel longitudinal rows; rugulae present near the mandibular insertions and on the ventral surface of the head. Mesosoma predominantly areolate, with costulae present on the dorsal and lateral surfaces of the pronotum, as well as the metapleuron. Femora shining through weaker areolate sculpture. Petiole smooth and shining ventrally, with areolate sculpture laterally and dorsally; the dorsal surface of the petiole with weak costulae over the areolate ground sculpture. Dorsal surface of postpetiole with weak costulae anteromedially, which grades into areolate sculpture on the posterior half. Gaster smooth and shining, with strong spectral iridescence. Surface of the first gastral sternite smooth and shining, with weaker spectral iridescence.

Setae: antennal scapes with adpressed pilosity; funiculus with subdecumbent pilosity. Dorsum of head, pronotum, waist segments and gaster with abundant, erect, blunt-tipped setae, the longest of which are about the length of the compound eye. The head bears ~40, mesosoma ~54, petiole 12, postpetiole ~18, and first gastral tergite ~68 setae. Pubescence present on the entire body but more difficult to detect on strongly sculptured surfaces.

Color: head (except for the mandibles and antennal funiculi), mesosoma, legs, and gaster are mostly dark brown. Mandibles, antennal funiculi, trochanters, and tarsi testaceous.

**Gyne measurements & indices (*n* = 1):** SL = 0.711; FRS = 0.302; CW = 0.865; CWb = 0.811; PoOC = 0.329; CL = 0.813; EL = 0.221; EW = 0.165; MD = 0.176; WL = 1.306; SPST = 0.276; MPST = 0.328; PEL = 0.460; NOL = 0.256; NOH = 0.174; PEH = 0.344; PPL = 0.192; PPH = 0.327; PW = 0.811; SBPA = 0.354; SPTI = 0.336; PEW = 0.222; PNW = 0.250; PPW = 0.525; HFL = 0.732; HFWmax = 0.194; HFWmin = 0.058; CS = 1.218; ES = 0.304; SI = 88; OI = 25; CI = 100; WLI = 161; SBI = 44; PSI = 21; PWI = 236; PLI = 240; NI = 147; PNWI = 113; NLI = 56; FI = 334.

**Gyne description:** In full-face view, head subquadrate, roughly as long as broad (CI 100). Mandibles weakly striate, shining, and armed with five teeth: the apical-most well developed, followed by a less developed preapical tooth and three equally-developed smaller teeth. Anterior clypeal margin emarginated medially. Frontal carinae short, extending past the antennal toruli by one and a half times the maximum width of the antennal scape. Compound eyes moderately protruding past the lateral margins of the head capsule. Antennal scapes very long: when fully retracted, extending past the posterior margin of the head capsule by about two times the maximum scape width (SI 88). Antennae 12-segmented; antennal club of three segments, with the apical-most segment as long as the preceding two in combination. Lateral margin of head slightly convex, converging evenly to the mandibular insertions. Posterior margin of head slightly convex, rounding evenly into the lateral margins.

In profile view, compound eyes ovular and moderately large (OI 25), with 16 ommatidia in longest row. Mesoscutum rounded evenly anteriorly, but just barely covering the dorsal surface of the pronotum, and weakly convex dorsally. Mesoscutellum on the same level as the mesoscutum, not overhanging the metanotum. Propodeal spiracle well developed, directed posterolaterally, and separated from the propodeal declivity by about four spiracle diameters. Propodeal spines poorly developed, but longer than in the worker (PSI 21), about as half as long as the propodeal declivity, tapering evenly from the base, directed posterodorsally, straight, and blunt. Propodeal declivity concave and forming a ~120° angle with the base of the propodeal spines. Propodeal lobes weakly developed, the dorsal margin with a slightly angulate flange. Metapleural gland bulla small, extending from the metacoxal insertion halfway to the propodeal spiracle. Petiolar peduncle moderately long: comprising about half of the petiolar length. Petiolar node cuneiform: transition between peduncle and node evenly rounded, resulting in a concave anterior node face; anterior face forming a very sharp ~80° angle with the dorsal face, which is indistinguishable from the posterior face; together, they form a ~130° angle with the caudal cylinder. Postpetiole subquadrate: weakly convex anteriorly, meeting the dorsal surface at a rounded 90° angle, flattened dorsally, and with a convex ventral surface.

In dorsal view, mesoscutum barely covering pronotum anteriorly, leaving much of the humeri visible laterally. Humeri well developed: the anterior margin broadly convex, meeting the lateral margins at a ~110° angle. Propodeal spines flattened dorsally, broadly approximate basally, and slightly diverging apically, their apices separated from each other by about two and a half times their length. Petiolar peduncle with spiracles slightly protruding past the lateral margins, not noticeably constricted anterior to them. Petiolar node cuneiform, forming a convex transverse ridge anterodorsally; slightly wider than the peduncle, and evenly grading into the caudal cylinder, which is roughly the same width as the node. Postpetiole very broad (PWI 236), anteroposteriorly compressed, and campaniform, articulating with most of the anterior margin of the gaster, leaving small angulate margins on each side exposed. Anterior margin of postpetiole broadly concave, with corners evenly rounding into the lateral margins, which diverge slightly to the angulate posterior corners; posterior margin broadly concave. Metafemur strongly incrassate, but to a lesser extent than the worker (FI 334).

Sculpture: clypeus with multiple (roughly 6) longitudinal carinae medially. Lateral lobes of clypeus with similar, weaker carinae; ground sculpture weakly areolate. Antennal scapes areolate; weakly shining. Cephalic dorsum densely areolate, with costulae; rugulae around the compound eyes and on the ventral surface of the head stronger than in the worker. Lateral sclerites of the mesosoma costulate over areolate ground sculpture, but weaker on the anterior half of the katepisternum. Mesoscutum and mesoscutellum with strong costulae with weaker cross-reticulations; mesoscutum with a small patch of smooth sculpture anteromedially. Femora shining through weak areolate sculpture. Petiole smooth and shining ventrally; dorsal surface of peduncle and anterior surface of node with weak rugae over weak areolate ground sculpture; dorsal surface of node rugose. Dorsal surface of postpetiole dull with costulae anteriorly, grading into areolate sculpture posteriorly. Gaster smooth and shining, with extensive spectral iridescence on all visible sclerites.

Setae: antennal scapes with adpressed pilosity; funiculus with subdecumbent pilosity. Dorsum of head, pronotum, waist segments and gaster with abundant, erect, blunt-tipped setae, the longest of which are about two-thirds the width of the compound eye. Pubescence present on the entire body but more difficult to detect on strongly sculptured surfaces.

Color: head (except for the mandibles and antennal funiculi), mesosoma, legs, and gaster are mostly dark brown. Mandibles, antennal funiculi, wing bases, trochanters, and tarsi testaceous.

**Male:** Unknown.

**Etymology:** Morphological, from the Latin ‘latus’ (= broad) + ‘crus’ (= leg), in reference to the notably incrassate femora in the worker of this species.

**Comments:** *Temnothorax laticrus* sp. nov. is only known from lowland dry forests in Jaragua National Park in the Dominican Republic, where it apparently nests in rotten sticks in the leaf litter or directly in the soil. Little else is known about the biology of this species, but it is probably similar to other members of the terricolous pan-Caribbean *pulchellus* group. There is evidently some color variation between populations: the non-type specimens observed in this study are distinctly bicolored, with the dorsal surface of the head, postpetiole, and gaster dark brown, and the rest of the body testaceous (Figs. 143H–143J).

***Temnothorax magnabulla* sp. nov.**

**Figure 144:**
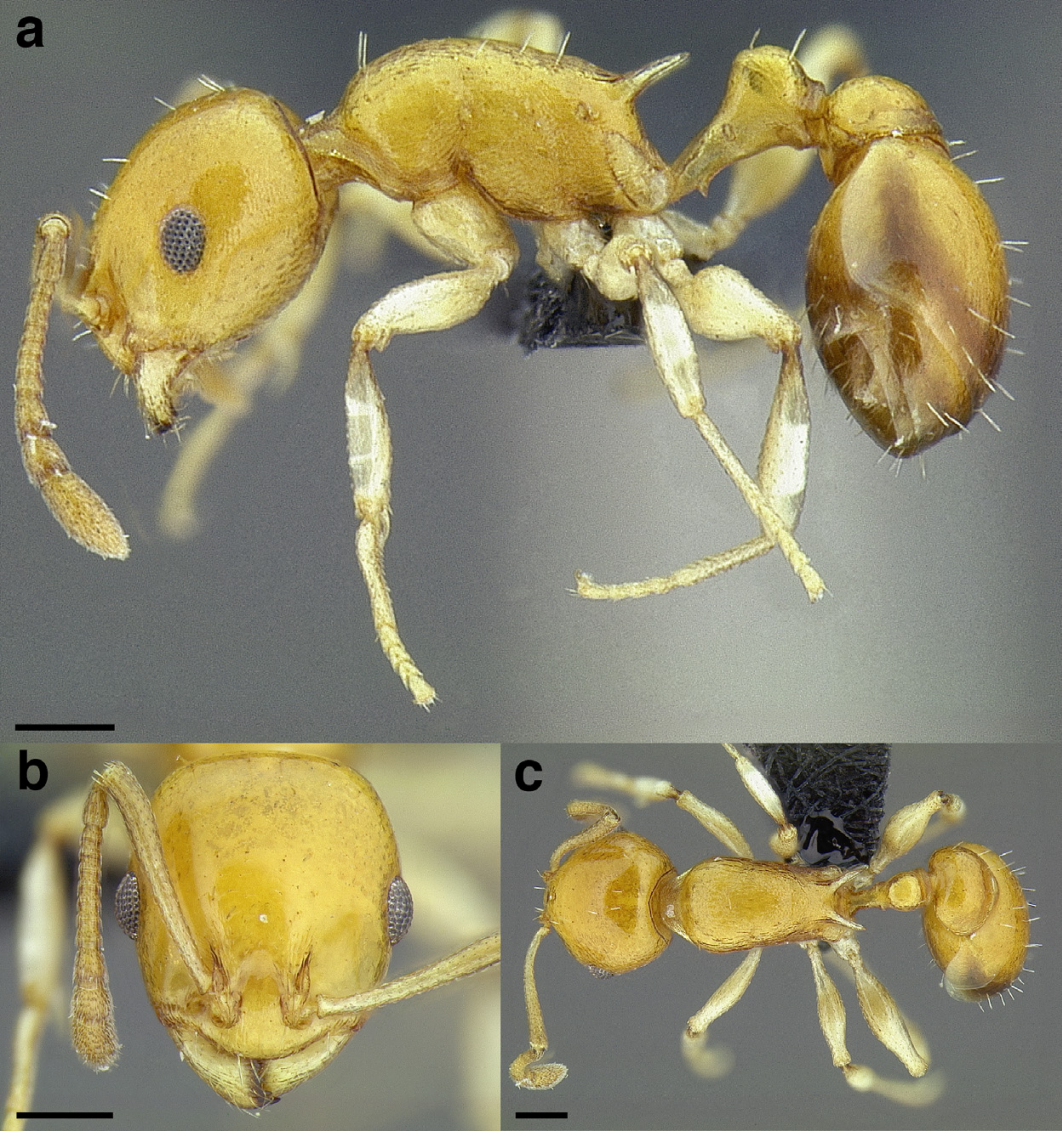
*Temnothorax magnabulla* sp. nov. holotype worker (CASENT0756093). (A) Profile view. (B) Full-face view. (C) Dorsal view. Scale bars 0.2 mm.

Distribution: Fig. 133J; worker: Fig. 144.

**Type material examined:** *Holotype worker:* U.S. VIRGIN ISLANDS: St. Croix: Knight: Routes 60 & 82, 17.7551°N 64.5937°W, 31 October 2005, J.K. Wetterer#160 (CASENT0756093) [USNM].

*Paratype workers:* U.S. VIRGIN ISLANDS: St. Croix: same data as holotype, 1 worker (CASENT0758673) [UCDC] 1 worker (LACMENT323205) [LACM].

**Non-type material examined:** U.S. VIRGIN ISLANDS: St. Croix, Hesselberg, S end Shore Drive, 17.694°N 64.891°W, 3 November 2005, J.K. Wetterer#201, 1 worker (LACMENT323206) [LACM].

**Geographic range:** U.S. Virgin Islands (Fig. 133J).

**Worker diagnosis:** *Temnothorax magnabulla* sp. nov. can be separated from all other species in the *salvini* clade by the following character combination: dorsum of head smooth and shining; in profile view, dorsum of mesosoma not strongly convex; pronotal declivity indistinct; metanotal groove not impressed; propodeum not depressed below the level of the promesonotum; propodeum bearing standing setae dorsally; propodeal spines about as long as the propodeal declivity, directed posterodorsally, and straight; hind femora weakly to moderately incrassate; petiolar node flat and relatively short dorsally; transition of dorsal to posterior face of petiolar node evenly rounded; in dorsal view, apex of petiolar node narrower or the same width as the caudal cylinder of the petiole; postpetiole very broad: greater than or equal to 2.4 times the width of the petiole; petiolar node with four erect setae dorsally; setae on head, mesosoma, waist segments and gaster erect, moderately long, sparse and blunt (never long and tapering); integument predominantly yellow: head (except for the mandibles), mesosoma, and gaster are mostly testaceous yellow, with the posterior margins of the gastral sclerites medium brown; coxae, legs, and mandibles are pale yellow.

**Similar species:** *Temnothorax agavicola* sp. nov., *T. ciferrii, T. flavidulus, T. harlequina* sp. nov., *T. hippolytus* sp. nov., *T. pulchellus* and *T. terricola*. The erect setae count of four on the dorsum of the petiolar node differentiates *T. magnabulla* sp. nov. from *T. terricola* and *T. hippolytus* sp. nov., which have only two along the posterior margin. The flat relatively small compound eyes, with eleven ommatidia in the longest row, and darker yellow integument separate *T. magnabulla* sp. nov. from *T. flavidulus*, which has twelve ommatidia in the longest row of the compound eye and is uniformly light yellow. The presence of erect setae on the propodeum distinguishes *T. magnabulla* sp. nov. from *T. pulchellus*. The weakly convex dorsal margin of the mesosoma and rounded transition between the dorsal and posterior faces of the petiolar node distinguishes *T. magnabulla* sp. nov. from *T. flavidulus, T. ciferrii*, and *T. harlequina* sp. nov., in which the mesosoma is strongly convex and the petiolar node transition is angulate. *Temnothorax magnabulla* sp. nov. and *T. harlequina* sp. nov. can be separated most easily by the color of the integument: *T. harlequina* sp. nov. has a light yellow gaster, which contrasts with a darker mesosoma and head, whereas *T. magnabulla* sp. nov. is uniformly yellow. *Temnothorax magnabulla* sp. nov. may also be confused with *T. agavicola* sp. nov. but the head sculpture of this species is areolate, in contrast the smooth and shining sculpture of *T. magnabulla* sp. nov.

**Worker measurements (*n* = 4):** SL = 0.458–0.533 (0.507); FRS = 0.182–0.215 (0.199); CW = 0.555–0.642 (0.607); CWb = 0.507–0.591 (0.557); PoOC = 0.238–0.261 (0.252); CL = 0.583–0.667 (0.636); EL = 0.119–0.150 (0.140); EW = 0.091–0.118 (0.107); MD = 0.147–0.165 (0.154); WL = 0.638–0.760 (0.719); SPST = 0.203–0.243 (0.228); MPST = 0.207–0.233 (0.223); PEL = 0.293–0.336 (0.321); NOL = 0.163–0.203 (0.180); NOH = 0.110–0.126 (0.120); PEH = 0.187–0.219 (0.207); PPL = 0.182–0.217 (0.199); PPH = 0.213–0.264 (0.235); PW = 0.340–0.394 (0.379); SBPA = 0.140–0.182 (0.162); SPTI = 0.191–0.239 (0.219); PEW = 0.135–0.164 (0.153); PNW = 0.130–0.164 (0.151); PPW = 0.327–0.393 (0.367); HFL = 0.454–0.508 (0.494); HFWmax = 0.122–0.159 (0.142); HFWmin = 0.042–0.061 (0.053); CS = 0.799–0.925 (0.875); ES = 0.165–0.209 (0.194); SI = 90–92 (91); OI = 21–23 (22); CI = 87–89 (88); WLI = 126–132 (129); SBI = 27–32 (29); PSI = 31–32 (32); PWI = 237–242 (239); PLI = 149–170 (162); NI = 139–161 (151); PNWI = 96–100 (98); NLI = 50–60 (56); FI = 251–290 (270).

**Worker description:** In full-face view, head subquadrate, slightly longer than broad (CI 87–89). Mandibles weakly striate, shining, and armed with five teeth: the apical-most well developed, followed by a smaller preapical tooth, which is followed by three equally developed smaller teeth. Anterior clypeal margin entire and evenly convex. Antennal scapes moderately long: when fully retracted, extending past the posterior margin of the head capsule by about the maximum width of the scape (SI 90–92). Antennae 12-segmented; antennal club of three segments, with the apical-most segment slightly longer than the preceding two in combination. Frontal carinae short, extending past the antennal toruli by one and a half times the maximum width of the antennal scape. Compound eyes moderately protruding past the lateral margins of the head capsule. Lateral margin of head evenly convex, forming a continuous arc from the posterior of the head to the mandibular insertions. Posterior head margin slightly convex, rounding evenly into the lateral margins.

In profile view, compound eyes ovular and moderately large (OI 21–23), with 11 ommatidia in longest row. Pronotal declivity indistinct, neck and anterior face of pronotum forming a rounded ~130° angle. Mesosoma convex dorsally, but flatter on the propodeum. Promesonotal suture extending from the posterior margin of the procoxal insertion only to the mesothoracic spiracle, which is well developed. Metanotal groove barely visible dorsally as a slight indentation and disruption of the weak sculpture. Propodeal spiracle well developed, directed posterolaterally, and separated from the propodeal declivity by about three spiracle diameters. Propodeal spines well developed and moderately long (PSI 31–32), about as long as the propodeal declivity, straight, tapering evenly from the base, and acute. Propodeal declivity straight and flat, forming a rounded ~90° angle with the base of the propodeal spines. Propodeal lobes rounded and very weakly developed. Metapleural gland bulla large, extending from the metacoxal insertion three quarters of the way to the propodeal spiracle. Petiole moderately long (PLI 149–170), without tubercles anterodorsally where it articulates with the mesosoma. Subpetiolar process in the form of a weakly developed, acute tooth, which grades evenly into the ventral margin of the petiole posteriorly; ventral surface of petiole very slightly concave posterior to the process. Petiolar peduncle moderately long: comprising about half the total length of the petiole. Petiolar node robust and erect: transition between peduncle and node marked by a rounded angle of ~120°, resulting in a concave anterior node face; anterior face forming a rounded ~90° angle with the dorsal face, which is evenly convex and moderately long; dorsal face rounding evenly into the posterior face, which forms a ~120° angle with the caudal cylinder. Postpetiole evenly rounded dorsally; weakly lobed ventrally.

In dorsal view, humeri weakly developed: rounded and only slightly protruding past the rest of the mesosoma; mesothoracic spiracles protruding past the lateral margins of the mesosoma, visible as slight angles where the pronotum meets the mesonotum. Mesonotum and propodeum separated by a very weak depression, their margins converging evenly to the bases of the propodeal spines. Propodeal spines broadly approximate basally and diverging apically, their apices separated from each other by slightly more than their length; negative space between them a basally narrowed “U”. Petiolar peduncle with spiracles protruding past lateral margins, peduncle constricted anterior to them. Petiolar node campaniform: narrowest anteriorly, flat posteriorly, with the posterior corners rounded; slightly wider than the peduncle, evenly grading into the caudal cylinder, which is slightly wider than the node. Postpetiole very broad (PWI 237–242) and campaniform, articulating with nearly the entire anterior margin of the gaster. Anterior margin of the postpetiole flat, with the corners evenly rounding into the lateral margins, which evenly diverge to the angulate posterior corners; posterior margin broadly concave. Metafemur moderately incrassate (FI 251–290).

Sculpture: median clypeal carina present, but very weak: barely visible against the smooth and shining ground sculpture, flanked by additional slightly stronger carinae. Antennal scapes shining through very weak, indistinct ground sculpture. Cephalic dorsum predominantly smooth and shining, with weak costulae medial to the frontal carinae, faint areolate sculpture surrounding the compound eyes, and weak areolate sculpture on the posterolateral margins of the head. Pronotal neck areolate. Mesosoma predominantly smooth and shining, with weak areolate sculpture and rugulae present on the lateral margins of the pronotum, meso-, and metapleurae; dorsally, predominantly weakly areolate, with weak costulae present on the pronotum; mesonotum with weaker sculpture than the rest of the mesosoma. Femora shining through very weak traces of areolate sculpture. Petiole with very shallow areolate sculpture on most surfaces, but petiolar node smooth and shining dorsally. Dorsal surface of postpetiole smooth and shining, with weak areolate sculpture on the posterior quarter. Gaster smooth and shining, without spectral iridescence. Surface of the first gastral sternite smooth and shining.

Setae: antennal scapes and funiculus with short, adpressed pilosity. Dorsum of head, pronotum, waist segments and gaster with sparse, short, erect, blunt-tipped setae, the longest of which are slightly less than the length of the compound eye. The head bears ~20, mesosoma ~10, petiole 4, postpetiole ~6, and first gastral tergite ~10 setae. Sparse, adpressed, very short pubescence present on the entire body, but nearly undetectable against the lightly colored integument.

Color: head (except for the mandibles), mesosoma, and gaster are mostly testaceous yellow, with the posterior margins of the gastral sclerites medium brown. Coxae, legs, and mandibles are pale yellow.

**Gyne:** Unknown.

**Male:** Unknown.

**Etymology:** Morphological, from the Latin ‘magna’ (= large) + bulla (= bubble-like cavity), in reference to the enlarged metapleural gland bulla.

**Comments:** *Temnothorax magnabulla* sp. nov. is known only from two collections made from the island of St. Croix in the U.S. Virgin Islands. St. Croix receives substantially more precipitation on the western part of the island, with the eastern end dominated by xerophytic scrub; the two collections were made at the extreme eastern and western ends of the island. The biology of this species is unknown but is likely to be similar to other members of the Antillean, terricolous *pulchellus* group. This species is closely related to other terricolous Antillean species, namely *T. albispinus*, *T. wettereri* sp. nov., *T. pulchellus*, *T. harlequina* sp. nov., and *T. laticrus* sp. nov.

*Temnothorax pulchellus* (Emery, 1894)

**Figure 145:**
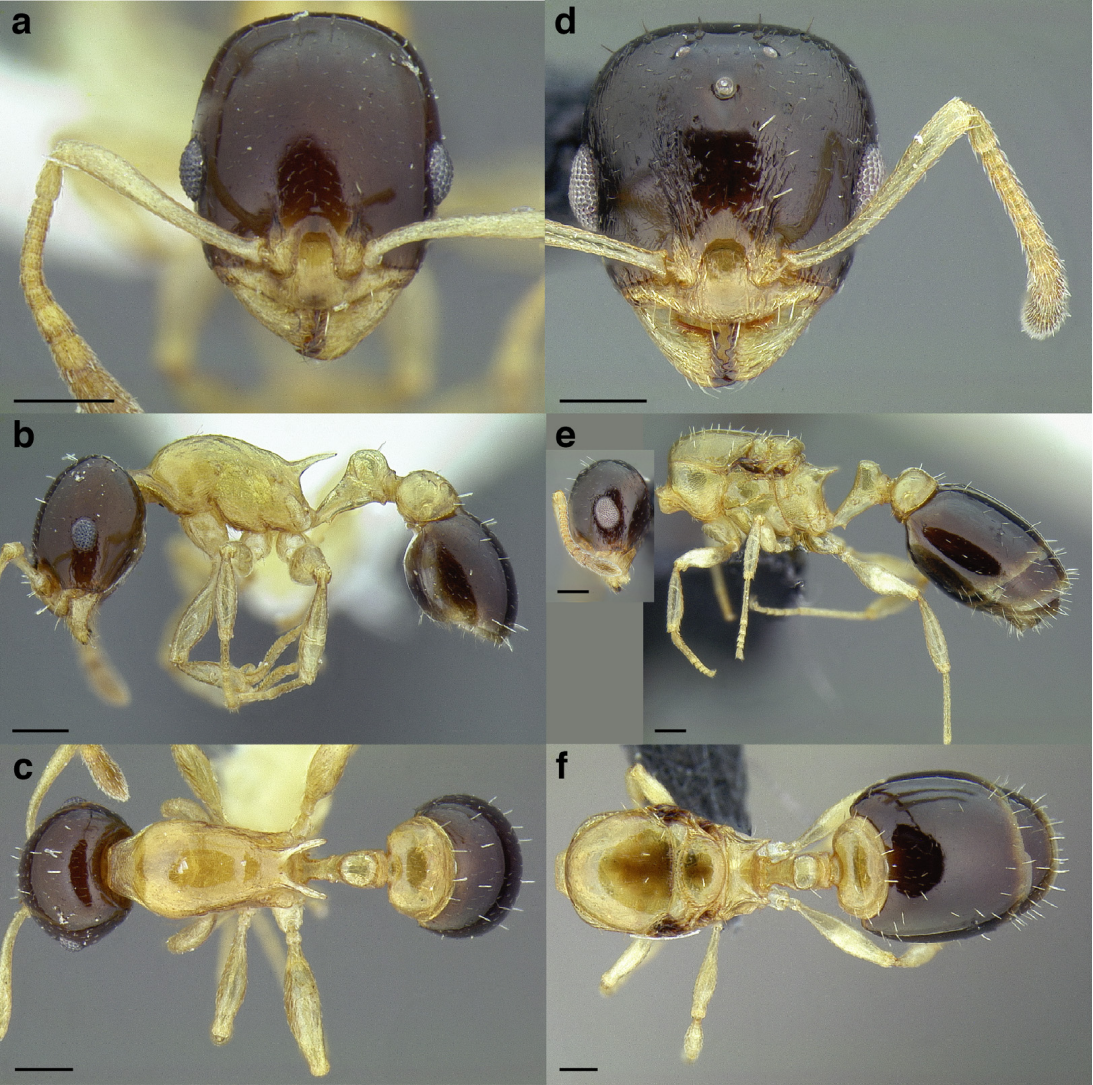
*Temnothorax pulchellus*. (A–C) Worker (LACMENT323204). (A) Full-face view. (B) Profile view. (C) Dorsal view. (D–F) Gyne (LACMENT323203). (D) Full-face view. (E) Profile view. (F) Dorsal view. Scale bars 0.2 mm.

Distribution: Fig. 133K; worker & gyne: Fig. 145.

*Macromischa pulchella* Emery, 1894: 162. Syntype worker and gyne. St. Thomas, U.S. Virgin Islands. One syntype worker here designated **lectotype**.

*Macromischa (Antillaemyrmex) pulchella* (Emery): Mann, 1920: 408. First combination in *Macromischa (Antillaemyrmex)*.

*Antillaemyrmex pulchellus* (Emery): Wheeler, 1931: 32. First combination in *Antillaemyrmex*.

*Leptothorax pulchellus* (Emery): Baroni Urbani, 1978: 482. First combination in *Leptothorax*.

*Temnothorax pulchellus* (Emery): Bolton, 2003: 272. First combination in *Temnothorax*.

**Type material examined:** *Lectotype worker of* Macromischa pulchella: U.S. VIRGIN ISLANDS: St. Thomas: Eggers, [no collection date], [no collector data] (images of CASENT0904717 examined on antweb.org) [MSNG].

**Non-type material examined:** BRITISH VIRGIN ISLANDS: Guana Island: Muskmelon Beach, 18.486°N 64.579°W, 0–5 m, 4 October 2002, R.R. Snelling#RRS02-209, subtropical dry forest: ex sifted mixed litter, 2 workers & 1 gyne (LACMENT323203) [LACM]; same data as previous, except: 12 October 2002, R.R. Snelling#RRS02-228, subtropical dry forest: ex sifted mixed litter, 3 workers (LACMENT323204) [LACM]; same data as previous, except: ~2 m, 18.48°N 64.58°W, 4 October 2002, R.R. Snelling#RRS02-210, strays in litter, 3 workers (LACMENT323208) [LACM].

U.S. VIRGIN ISLANDS: St. John: Privateer Bay, N end of bay, 18.338°N 64.666°W, 12 November 2005, J.K. Wetterer#JKW-2005-321, 1 worker (LACMENT323207) [LACM].

**Geographic range:** U.S. and British Virgin Islands (Fig. 133K).

**Worker diagnosis:** *Temnothorax pulchellus* can be separated from all other species in the *salvini* clade by the following character combination: in profile view, dorsum of mesosoma not strongly convex; pronotal declivity indistinct; metanotal groove not impressed; propodeum not depressed below the level of the promesonotum; propodeum not bearing standing setae dorsally; propodeal spines longer than propodeal declivity, directed posterodorsally, and slightly downcurved; hind femora weakly to moderately incrassate; in dorsal view, apex of petiolar node narrower to slightly broader the caudal cylinder of the petiole; postpetiole very broad: greater than or equal to 2.4 times the width of the petiole; dorsum of head smooth and shining; petiolar node with two erect setae dorsally; setae on head, mesosoma, waist segments and gaster erect, moderately long, sparse and blunt (never long and tapering); integument bicolored: head (except for the clypeus and mandibles) and gaster dark brown; rest of body pale yellow.

**Similar species:** *Temnothorax ciferrii, T. harlequina* sp. nov., *T. hippolytus* sp. nov., and *T. terricola*. The erect setae count of two on the dorsum of the petiolar node differentiates *T. pulchellus* from *T. harlequina* sp. nov. and *T. ciferrii*, which have four dorsally. The absence of erect setae on the propodeum distinguishes *T. pulchellus* from *T. hippolytus* sp. nov. and *T. terricola*, as well as the long propodeal spines.

**Worker measurements & indices (*n* = 9):** SL = 0.382–0.516 (0.470); FRS = 0.163–0.211 (0.195); CW = 0.457–0.603 (0.547); CWb = 0.407–0.542 (0.493); PoOC = 0.189–0.263 (0.236); CL = 0.492–0.635 (0.586); EL = 0.108–0.141 (0.129); EW = 0.075–0.104 (0.096); MD = 0.118–0.158 (0.136); WL = 0.521–0.690 (0.629); SPST = 0.169–0.262 (0.224); MPST = 0.169–0.222 (0.206); PEL = 0.229–0.337 (0.295); NOL = 0.140–0.193 (0.170); NOH = 0.096–0.129 (0.115); PEH = 0.165–0.212 (0.193); PPL = 0.158–0.205 (0.188); PPH = 0.185–0.243 (0.214); PW = 0.286–0.385 (0.351); SBPA = 0.123–0.156 (0.143); SPTI = 0.170–0.225 (0.208); PEW = 0.119–0.154 (0.138); PNW = 0.106–0.152 (0.132); PPW = 0.299–0.395 (0.356); HFL = 0.383–0.502 (0.455); HFWmax = 0.105–0.140 (0.129); HFWmin = 0.039–0.057 (0.049); CS = 0.653–0.860 (0.786); ES = 0.146–0.193 (0.178); SI = 94–97 (95); OI = 22–26 (23); CI = 82–86 (84); WLI = 126–131 (128); SBI = 27–31 (29); PSI = 31–39 (36); PWI = 242–273 (258); PLI = 135–177 (157); NI = 131–161 (148); PNWI = 89–104 (95); NLI = 53–65 (58); FI = 232–298 (265).

**Worker description**: In full-face view, head subquadrate, longer than broad (CI 82–86). Mandibles weakly striate, shining, and armed with five teeth: the apical-most well developed, followed by a less developed preapical tooth and three equally-developed smaller teeth. Anterior clypeal margin entire and evenly convex medially. Antennal scapes moderately long: when fully retracted, extending past the posterior margin of the head capsule by about the maximum width of the scape (SI 94–97). Antennae 12-segmented; antennal club of three segments, with the apical-most segment slightly longer than the preceding two in combination. Frontal carinae very short, extending past the antennal toruli by the maximum width of the antennal scape. Compound eyes moderately protruding past the lateral margins of the head capsule. Lateral margin of head evenly convex, forming a continuous arc from the posterior of the head to the mandibular insertions. Posterior head margin flat to very slightly convex, rounding evenly into the lateral margins.

In profile view, compound eyes ovular and moderately large (OI 22–26), with 11 ommatidia in longest row. Pronotal declivity indistinct, neck and anterior face of pronotum forming a rounded ~140° angle. Mesosoma convex dorsally from where it joins the pronotal neck to the propodeal spines. Promesonotal suture extending from the posterior margin of the procoxal insertion only to the mesothoracic spiracle, which is well developed. Metanotal groove visible in profile in some specimens as a small concavity between the promesonotum and propodeum; visible in all specimens as a faint disruption in the surface sculpture between meso- and metacoxal insertions to the minute metathoracic spiracle. Propodeal spiracle well developed, directed posterolaterally, and separated from the propodeal declivity by about three spiracle diameters. Propodeal spines well developed and moderately long (PSI 31–39), about as long as the propodeal declivity, tapering evenly from the base, and acute. Propodeal declivity straight and flat, forming a rounded ~100° angle with the base of the propodeal spines. Propodeal lobes rounded and very weakly developed. Metapleural gland bulla small, extending from the metacoxal insertion halfway to the propodeal spiracle. Petiole moderately long (PLI 135–177), without tubercles anterodorsally where it articulates with the mesosoma. Subpetiolar process in the form of a small, blunt tooth, which grades evenly into the ventral margin of the petiole posteriorly; ventral surface of petiole slightly concave posterior to the process. Petiolar peduncle moderately long: comprising about half of the total length of the petiole. Petiolar node robust and erect: transition between peduncle and node marked by a rounded angle of ~120°, resulting in a strongly concave anterior node face; anterior face forming a rounded ~100° angle with the dorsal face, which is evenly convex and moderately long; dorsal face rounding evenly into the posterior face, which forms a ~120° angle with the caudal cylinder. Postpetiole evenly rounded dorsally; weakly lobed ventrally.

In dorsal view, humeri weakly developed: rounded and only slightly protruding past the rest of the mesosoma; mesothoracic spiracles protruding past the lateral margins of the mesosoma, visible as slight angles where the pronotum meets the mesonotum. Mesonotum and propodeum separated by a very weak depression, their margins converging evenly to the bases of the propodeal spines. Propodeal spines closely approximate basally and diverging apically, their apices separated from each other by slightly more than their length; negative space between them a basally rounded “V”. Petiolar peduncle with spiracles protruding past the lateral margins; node oviform and narrowest anteriorly, slightly wider than the peduncle, and evenly grading into the caudal cylinder, which is slightly wider than the node. Postpetiole very broad (PWI 242–273) and campaniform, articulating with the entire anterior margin of the gaster, not leaving anterior corners of gaster exposed. Anterior margin of the postpetiole broadly convex, with corners evenly rounding into the lateral margins, which evenly diverge to the angulate posterior corners; posterior margin broadly concave. Metafemur weakly to moderately incrassate (FI 232–298).

Sculpture: median clypeal carina present, flanked by additional weaker carinae over a smooth and shining ground sculpture. Antennal scapes shining through very weakly areolate ground sculpture. Cephalic dorsum predominantly smooth and shining, except for fine piligerous punctures and weak areolate sculpture posterior to the compound eyes, which extends to the posteroventral surface of the head. Pronotal neck areolate. Mesosoma predominantly smooth and shining dorsally; weak areolate sculpture and rugulae present on the lateral margins of the pronotum, meso-, and metapleurae. Femora shining through very weak traces of areolate sculpture. Peduncle of petiole smooth and shining ventrally, with very shallow areolate sculpture surrounding the base of the petiolar node, which is smooth and shining dorsally. Dorsal surface of postpetiole smooth and shining, with weak areolate sculpture on the posterior margin. Gaster smooth and shining, without spectral iridescence. Surface of the first gastral sternite smooth and shining.

Setae: antennal scapes and funiculi with short, adpressed pilosity. Dorsum of head, pronotum, waist segments and gaster with sparse, short, erect, blunt-tipped setae, the longest of which are slightly less than the width of the compound eye. The head bears ~18, mesosoma ~2, petiole 2, postpetiole ~10, and first gastral tergite ~14 setae. Sparse, adpressed pubescence present on the entire body.

Color: head (except for the clypeus and mandibles) and gaster are dark brown. The rest of the body pale yellow.

**Gyne measurements & indices (*n* = 1):** SL = 0.577; FRS = 0.249; CW = 0.750; CWb = 0.690; PoOC = 0.278; CL = 0.707; EL = 0.208; EW = 0.160; MD = 0.168; WL = 1.134; SPST = 0.278; MPST = 0.301; PEL = 0.438; NOL = 0.192; NOH = 0.173; PEH = 0.302; PPL = 0.195; PPH = 0.351; PW = 0.697; SBPA = 0.354; SPTI = 0.349; PEW = 0.210; PNW = 0.212; PPW = 0.580; HFL = 0.611; HFWmax = 0.153; HFWmin = 0.062; CS = 1.044; ES = 0.288; SI = 84; OI = 28; CI = 98; WLI = 164; SBI = 51; PSI = 25; PWI = 276; PLI = 225; NI = 111; PNWI = 101; NLI = 44; FI = 247.

**Gyne description:** In full-face view, head subquadrate, roughly as long as broad (CI 98). Mandibles weakly striate, shining, and armed with five teeth: the apical-most well developed, followed by a less developed preapical tooth and three equally-developed smaller teeth. Anterior clypeal margin very slightly concave medially. Antennal scapes moderately long: when fully retracted, just reaching the posterior margin of the head capsule (SI 84). Antennae 12-segmented; antennal club of three segments, with the apical-most segment as long as the preceding two in combination. Frontal carinae very short, extending past the antennal toruli by one and a half times the maximum width of the antennal scape. Compound eyes moderately protruding past the lateral margins of the head capsule. Lateral margin of head evenly convex, forming a continuous arc from the posterior of the head to the mandibular insertions. Posterior head margin very slightly convex, rounding evenly into the lateral margins.

In profile view, compound eyes ovular and large (OI 28), with 16 ommatidia in longest row. Mesoscutum rounded evenly anteriorly, covering the dorsal surface of the pronotum, and flat dorsally. Mesoscutellum on the same plane as the mesoscutum but inclined posteriorly; posterior margin of mesoscutellum even with the posterior margin of the metanotum. Propodeal spiracle well developed, directed posterolaterally, and separated from the propodeal declivity by about three and a half spiracle diameters. Propodeal spines stout and well developed, but short (PSI 25), about half as long as the propodeal declivity, tapering evenly from the base, straight, and directed posteriorly. Metapleural gland bulla small, extending from the metacoxal insertion halfway to the propodeal spiracle. Propodeal declivity weakly concave, forming a rounded ~100° angle with the base of the propodeal spines. Propodeal lobes rounded and very weakly developed. Petiole long (PLI 225), without tubercles anterodorsally where it articulates with the mesosoma. Subpetiolar process in the form of a weakly developed, acute triangular tooth, which grades evenly into the ventral margin of the petiole posteriorly. Petiolar peduncle moderately long: comprising about half of the total length of the petiole. Petiolar node erect: transition between the peduncle and the node evenly rounded, resulting in a concave anterior node face; anterior face forming a sharp ~90° angle with the dorsal face, which is nearly flat; dorsal face rounding evenly into the posterior face, which forms a ~130° angle with the caudal cylinder. Postpetiole evenly rounded anterodorsally, flattened dorsally; ventral surface lobed.

In dorsal view, mesoscutum covering pronotum anteriorly, but humeri visible laterally as rounded sclerites. Propodeal spines diverging apically, their apices separated from each other by about two times their length. Petiolar peduncle with spiracles protruding past the lateral margins, the peduncle broadened where they arise. Petiolar node weakly convex anteriorly, but forming angles with the lateral margins, its shape roughly trapezoidal. Petiolar node slightly wider than the peduncle, and evenly grading into the caudal cylinder, which is roughly the same width as the node. Postpetiole very broad (PWI 276), anteroposteriorly compressed, and campaniform, articulating with most of the anterior margin of the gaster, leaving small, angulate margins on each side of the gaster exposed. Anterior margin of the postpetiole weakly concave medially, with corners evenly rounding into the lateral margins, which evenly diverge to the angulate posterior corners; posterior margin broadly concave. Metafemur weakly incrassate (FI 247).

Sculpture: median clypeal carina present, but very short and weak: extending from the anterior margin only about a quarter of the way to the frontal triangle; flanked by additional stronger carinae; smooth and shining through weak areolate sculpture medially. Antennal scapes shining through very weakly areolate ground sculpture. Cephalic dorsum shining through traces of costulate ground sculpture; weak areolae present around the antennal insertions and posterior to the compound eyes. Pronotal neck evenly areolate. Lateral surfaces of pronotum with weak areolae and striae, which become stronger ventrally. Anepisternum and katepisternum smooth and shining. Propodeum with weak areolate sculpture and costulae laterally, but smooth and shining between the propodeal spines. Mesoscutum predominantly smooth and shining, but with weak costulae surrounding a medial patch of smooth and shining sculpture. Mesoscutellum smooth and shining, with weak costulate sculpture laterally. Femora smooth and shining, with traces of weak areolate sculpture. Peduncle of petiole smooth and shining ventrally, with shallow areolate sculpture dorsally. Areolate sculpture surrounding the petiolar node, which is smooth and shining dorsally. Dorsal surface of postpetiole smooth and shining, with weak areolate sculpture on the posterior quarter, and lateral surfaces. Gaster smooth and shining, without spectral iridescence. Surface of the first gastral sternite smooth and shining.

Setae: antennal scapes and funiculi with short, adpressed pilosity. Dorsum of head, pronotum, waist segments and gaster with sparse, short, erect, blunt-tipped setae, the longest of which are about half the width of the compound eye. Sparse, adpressed pubescence present on the entire body.

Color: head (except for the clypeus and mandibles), wing bases, and gaster are dark brown. The rest of the body pale yellow.

**Male:** Unknown.

**Etymology:** Morphological, from the Latin ‘pulchellus’ (= pretty little), in reference to the coloration and size of this species.

**Comments:** *Temnothorax pulchellus* is known only from the British and U.S. Virgin Islands of Guana and St. John, respectively, both of which are a mixture of tropical xeric scrubland and tropical moist forest. The biology of this species is unknown but is likely to be similar to other members of the Antillean, terricolous *pulchellus* group. Both Baroni Urbani (1978) and Emery (1894) note that the workers of this species are very small; this observation may have been biased by the small amount of material available to them (e.g. Baroni Urbani, 1978 only observed one worker syntype). During this revision, I was able to look at two additional nest series, and the variation in the size of the workers of *pulchellus* (WL 0.521–0.690) appears to be similar to that of *albispinus* (WL 0.608–0.715). This species is closely related to other terricolous Antillean species, namely *T. albispinus*, *T. harlequina* sp. nov., *T. laticrus* sp. nov. *T. magnabulla* sp. nov., and *T. wettereri* sp. nov.

***Temnothorax terricola* (Mann, 1920)**

**Figure 146:**
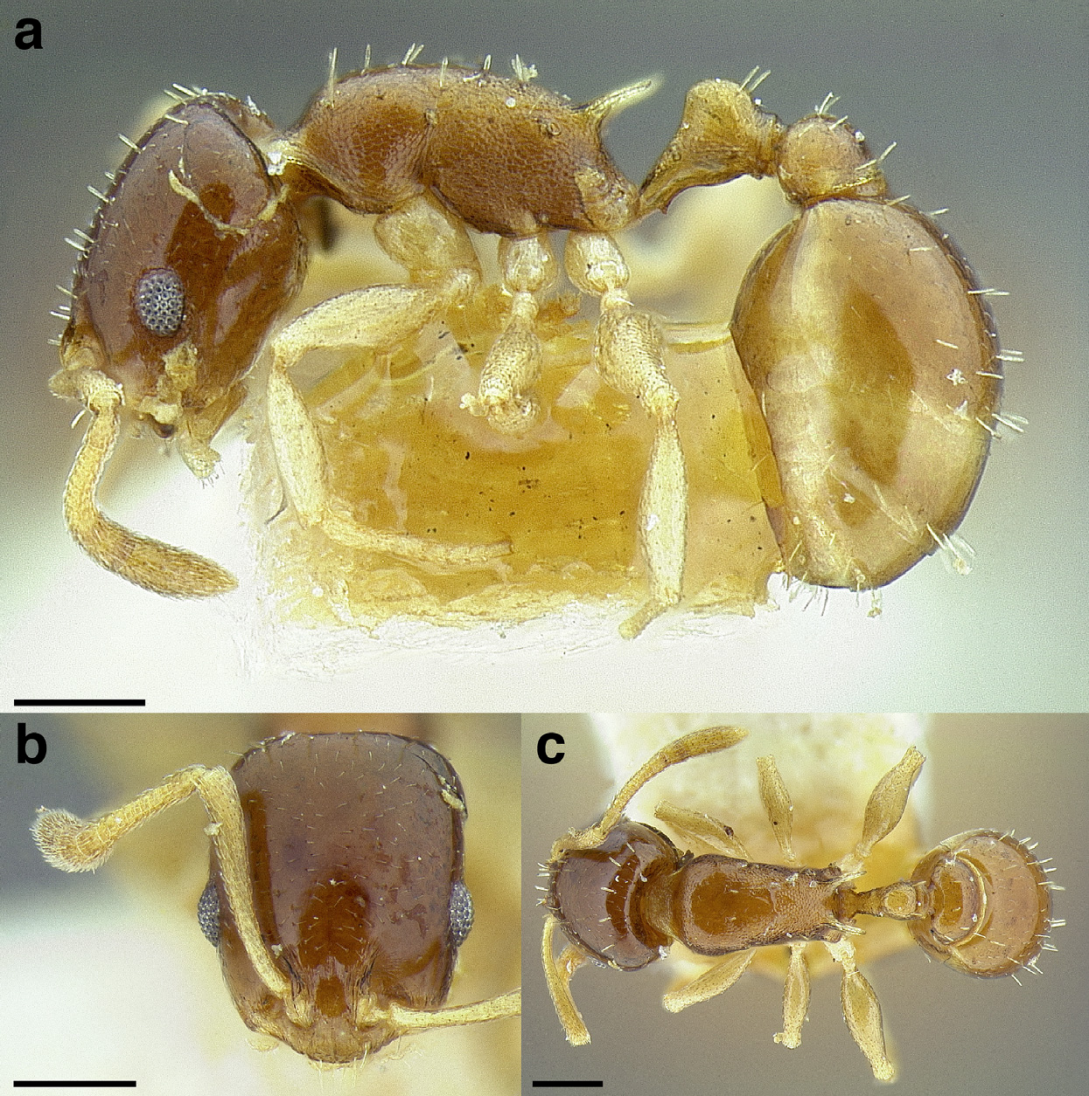
*Temnothorax terricola* lectotype worker (USNMENT00922871). (A) Profile view. (B) Full-face view. (C) Dorsal view. Scale bars 0.2 mm.

Distribution: Fig. 133L; worker: Fig. 146.

*Macromischa (Antillaemyrmex) terricola*: Mann, 1920: 423, fig. 7. Syntype workers and gyne. Baracoa, Cuba. One syntype worker here designated **lectotype**.

*Antillaemyrmex terricola* (Mann): Wheeler, 1931: 32. First combination in *Antillaemyrmex*.

*Leptothorax terricolus* (Mann): Baroni Urbani, 1978: 518. First combination in *Leptothorax*.

*Temnothorax terricola* (Mann): Bolton, 2003: 272. First combination in *Temnothorax*.

**Type material examined:** *Lectotype worker of* Macromischa (Antillaemyrmex) terricola: CUBA: Guantanamo: Baracoa, 1918, W.M. Mann, Cotype U.S.N.M. No. 54153 (USNMENT00922871, top specimen, furthest from pin) [USNM].

*Paralectotype workers and gyne:* same pin as lectotype (USNMENT00922871, remaining specimens on pin) [USNM]; same data as lectotype: 1 gyne, head and mesosoma missing, 3 workers, head of 2 workers missing (USNMENT00922870) [USNM] 4 workers, head of 1 worker missing (USNMENT00532116) [USNM] 3 workers (USNMENT00922871) [USNM].

**Non-type material examined:** CUBA: Guantanamo: 1 km NE Alto de Cotilla, 20.183333°N 74.483333°W, 500 m, 24 August 2001, P.S. Ward#14453-6, pine forest, sifted litter (leaf mold, rotten wood), 1 worker (CASENT0712509) [UCDC] 1 worker (CASENT0758707) [UCDC] 3 workers (CASENT0758300) [UCDC]. Same data as previous, except: 6 km NW La Vega de Taco, 20.55°N 74.716667°W, 20 m, 23 August 2001, P.S. Ward#14441-1, rainforest edge, ground foragers, 2 workers (CASENT0915980) [UCDC].

**Geographic range:** Low elevations of Cuba (Fig. 133L).

**Worker diagnosis:** *Temnothorax terricola* can be separated from all other species in the *salvini* clade by the following character combination: head in full face view blocky; promesonotal suture shallowly impressed; metanotal groove not impressed; propodeum not depressed below the level of the promesonotum; propodeum bearing standing setae dorsally; propodeal spines about as long as the propodeal declivity, and directed posterodorsally; hind femora weakly incrassate; petiolar node rounded: dorsal face transitioning to posterior face through a broad curve; petiolar node with only two erect setae dorsally; postpetiole broad: more than 2.2 times the width of the petiole; dorsum of head smooth and shining; setae on head, mesosoma, waist segments and gaster erect, short, sparse and blunt (never long and tapering); head, mesosoma, waist segments, and gaster are uniformly testaceous; mandibles and extremities light brown.

**Similar species:** *Temnothorax ciferrii, T. harlequina* sp. nov., *T. hippolytus* sp. nov*.*, and *T. torrei. Temnothorax terricola* can be distinguished from *T. ciferrii* and *T. harlequina* sp. nov. by the setae count of two on the dorsum of the petiolar node, versus four. *Temnothorax terricola* is closely related to *T. torrei* and *T. hippolytus* sp. nov., but the smooth and shining head sculpture can be used to separate *T. terricola* from *T. torrei*, in which the head sculpture is at least somewhat areolate. *Temnothorax terricola* can be differentiated from *T. hippolytus* sp. nov. by the boxy head shape (ovular in *T. hippolytus* sp. nov.), the shallowly impressed promesonotal suture, and the less angulate transition between the dorsal and posterior faces of the petiolar node.

**Worker measurements (*n* = 14):** SL = 0.349–0.401 (0.380); FRS = 0.130–0.164 (0.145); CW = 0.401–0.471 (0.450); CWb = 0.360–0.441 (0.416); PoOC = 0.213–0.243 (0.229); CL = 0.471–0.551 (0.514); EL = 0.099–0.126 (0.110); EW = 0.069–0.101 (0.084); MD = 0.094–0.122 (0.108); WL = 0.493–0.597 (0.552); SPST = 0.147–0.204 (0.176); MPST = 0.161–0.220 (0.182); PEL = 0.200–0.251 (0.220); NOL = 0.130–0.164 (0.145); NOH = 0.074–0.099 (0.090); PEH = 0.140–0.170 (0.155); PPL = 0.129–0.160 (0.141); PPH = 0.136–0.175 (0.157); PW = 0.268–0.306 (0.290); SBPA = 0.116–0.147 (0.133); SPTI = 0.147–0.188 (0.165); PEW = 0.099–0.123 (0.110); PNW = 0.096–0.120 (0.109); PPW = 0.236–0.277 (0.258); HFL = 0.310–0.370 (0.348); HFWmax = 0.089–0.131 (0.108); HFWmin = 0.036–0.053 (0.044); CS = 0.596–0.702 (0.673); ES = 0.134–0.177 (0.152); SI = 87–99 (92); OI = 21–25 (23); CI = 76–84 (81); WLI = 129–137 (133); SBI = 29–34 (32); PSI = 27–36 (32); PWI = 215–255 (235); PLI = 128–187 (157); NI = 140–201 (162); PNWI = 89–111 (100); NLI = 60–77 (66); FI = 202–285 (248).

**Worker description:** In full-face view, head subquadrate, longer than broad (CI 76–84). Mandibles weakly striate, shining, and armed with five teeth: the apical-most well developed, followed by a smaller preapical tooth, which is followed by three equally developed smaller teeth. Anterior clypeal margin entire and evenly rounded. Antennal scapes short: when fully retracted, failing to reach the posterior margin of the head capsule by about the maximum width of the scape (SI 87–99). Antennae 12-segmented; antennal club of three segments, with the apical-most segment one and a half times longer than the preceding two in combination. Frontal carinae short, extending past the antennal toruli by one and a half times the maximum width of the antennal scape. Compound eyes weakly protruding past the lateral margins of the head capsule. Lateral margin of head very weakly convex, nearly flat, giving the head a boxy appearance. Posterior head margin flat to very slightly concave, rounding evenly into the lateral margins.

In profile view, compound eyes ovular and moderately large (OI 21–25), with 9 ommatidia in longest row. Pronotal declivity distinct, with neck and anterior face of pronotum forming a rounded ~130° angle; anterior and dorsal faces of pronotum forming a rounded ~140° angle. Mesosoma evenly, but weakly, convex dorsally from where it joins the pronotal declivity to the propodeal spines, nearly flat. Promesonotal suture extending from the posterior margin of the procoxal insertion only to the mesothoracic spiracle, which is well developed. Metanotal groove nearly entirely absent: only visible as a faint disruption in the surface sculpture between meso- and metacoxal insertions to the minute metathoracic spiracle, which is nearly indistinguishable against the ground sculpture. Propodeal spiracle weakly developed, directed posterolaterally, and separated from the propodeal declivity by about three and a half spiracle diameters. Propodeal spines well developed, moderately long (PSI 27–36), about as long as the propodeal declivity, tapering evenly from the base, very weakly downcurved, and acute. Propodeal declivity straight and flat, forming a rounded ~100° angle with the base of the propodeal spines. Propodeal lobes rounded and very weakly developed. Metapleural gland bulla small, extending from the metacoxal insertion halfway to the propodeal spiracle. Petiole moderately long (PLI 128–187), without tubercles anterodorsally. Subpetiolar process in the form of a weakly developed, acute, triangular tooth; ventral margin of petiole strongly concave posterior to it. Petiolar peduncle short, comprising about a quarter of the total length of the petiole. Petiolar node robust: grading evenly into the petiolar peduncle anteriorly, anterior face concave; anterior face forming a rounded, ~90° angle with the dorsal face, which is evenly, strongly convex; dorsal face rounding evenly into the posterior face, which forms a ~110° angle with the caudal cylinder. Postpetiole evenly rounded anterodorsally, before flattening posterodorsally; weakly lobed ventrally.

In dorsal view, humeri weakly developed: rounded and only slightly wider than the rest of the mesosoma; mesothoracic spiracles weakly protruding past the lateral margins of the mesosoma, visible as slight angles where the pronotum meets the mesonotum. Metanotal groove absent: mesonotum and propodeum completely fused and converging evenly to the bases of the propodeal spines. Propodeal spines broadly approximated basally and diverging apically, their apices separated from each other about their length; negative space between them a “U”. Petiolar peduncle with spiracles barely protruding past the lateral margins; not constricted anterior to them. Petiolar node ovular, subtly narrowed anteriorly, slightly wider than the peduncle, and evenly grading into the caudal cylinder, which is the same width as the node. Postpetiole moderately to very broad (PWI 215–255) and campaniform, articulating with nearly the entire anterior margin of the gaster, leaving small angulate margins on each side exposed. Anterior margin of the postpetiole flat, with corners evenly rounding into the lateral margins, which evenly diverge to the angulate posterior corners; posterior margin broadly concave. Metafemur weakly to moderately incrassate (FI 202–285).

Sculpture: median lobe of clypeus compressed between the closely approximated frontal lobes. Median clypeal carina present, flanked two additional equally developed carinae over smooth and shining ground sculpture. Antennal scapes shining through weak sculpture. Cephalic dorsum predominantly smooth and shining, with weak costulate sculpture medial to the frontal carinae, faint areolate sculpture surrounding the compound eyes and between the eyes and the mandibular insertions. Pronotal neck areolate. Mesosoma with lateral surfaces and dorsum of propodeum shining through weak areolate sculpture; dorsum of promesonotum and propodeal declivity smooth and shining. Femora shining through very weak areolate sculpture. Petiole smooth and shining ventrally, with shallow areolate sculpture surrounding the base of the petiolar node, which is mostly smooth and shining dorsally. Dorsal surface of postpetiole smooth and shining, with shallow areolate sculpture on the posterior quarter areolate. Gaster smooth and shining, without spectral iridescence on the first tergite. Surface of the first gastral sternite smooth and shining.

Setae: antennal scapes and funiculi with short, adpressed pilosity. Dorsum of head, mesosoma, waist segments and gaster with short, erect, blunt-tipped setae, some of which are nearly clavate; the longest setae are roughly the width of the compound eye. The head bears ~32, mesosoma ~16, petiole 2, postpetiole ~10, and first gastral tergite ~26 setae. Sparse, adpressed pubescence present on the entire body, but only apparent on the darker colored integument.

Color: head, mesosoma, waist segments, and gaster are uniformly testaceous. Mandibles and extremities are light brown.

**Gyne:** Too damaged to describe.

**Male:** Unknown.

**Etymology:** Behavioural, from the Latin ‘terra’ (= earth) + ‘colere’ (= dwelling), in reference to the nesting habitat of this species.

**Comments:** *Temnothorax terricola* is mostly known from the Guantanamo region in eastern Cuba. The type series was collected from under a stone in low elevation pine forest near Baracoa. Two more series were collected by Phil Ward: stray ground foragers were collected at bread crumb baits on the edge of disturbed rainforest just north of Baracoa, and several more workers were recovered from Winkler leaf litter extraction in mid-elevation mixed *Pinus cubensis* and broadleaf evergreen forest. This species was reported from the Nipe-Sagua-Baracoa mountain range in Cuba in a survey by Ferrer & Triana (2004), which is consistent with the previous collections; I have not been able to personally examine these specimens, however. This species, like other members of the *pulchellus* group, is probably ground or leaf litter nesting. The original description by Mann (1920) notes that this species has a dark brown to black head and gaster. Perhaps the intensity of the integumental coloration in the type series has faded with time, but all of the specimens from the type series that I have observed are dark to medium brown. The two series collected by Phil Ward are substantially lighter, with the head and gaster medium brown, while the rest of the body is light brown; the lateral surface of the pronotum is also slightly less densely sculptured than the type series, shining through weak areolate sculpture. This species has long been suspected to have close relationships to other members of the *salvini* clade, specifically members of the *pulchellus* group: *T. albispinus*, *T. ciferrii, T. flavidulus*, and *T. pulchellus*. In the first formal morphology-based phylogenetic work including this species, Fontenla Rizo (2000), while not including all of the taxa in Baroni Urbani’s *pulchellus* group, corroborated these relationships to some degree, with the caveat that *T. terricola* and *T. torrei* fell outside of this group. Combined molecular and morphological analyses (Prebus, in prep.) mostly agree with Fontenla Rizo (2000).

***Temnothorax torrei* (Aguayo, 1931)**

**Figure 147:**
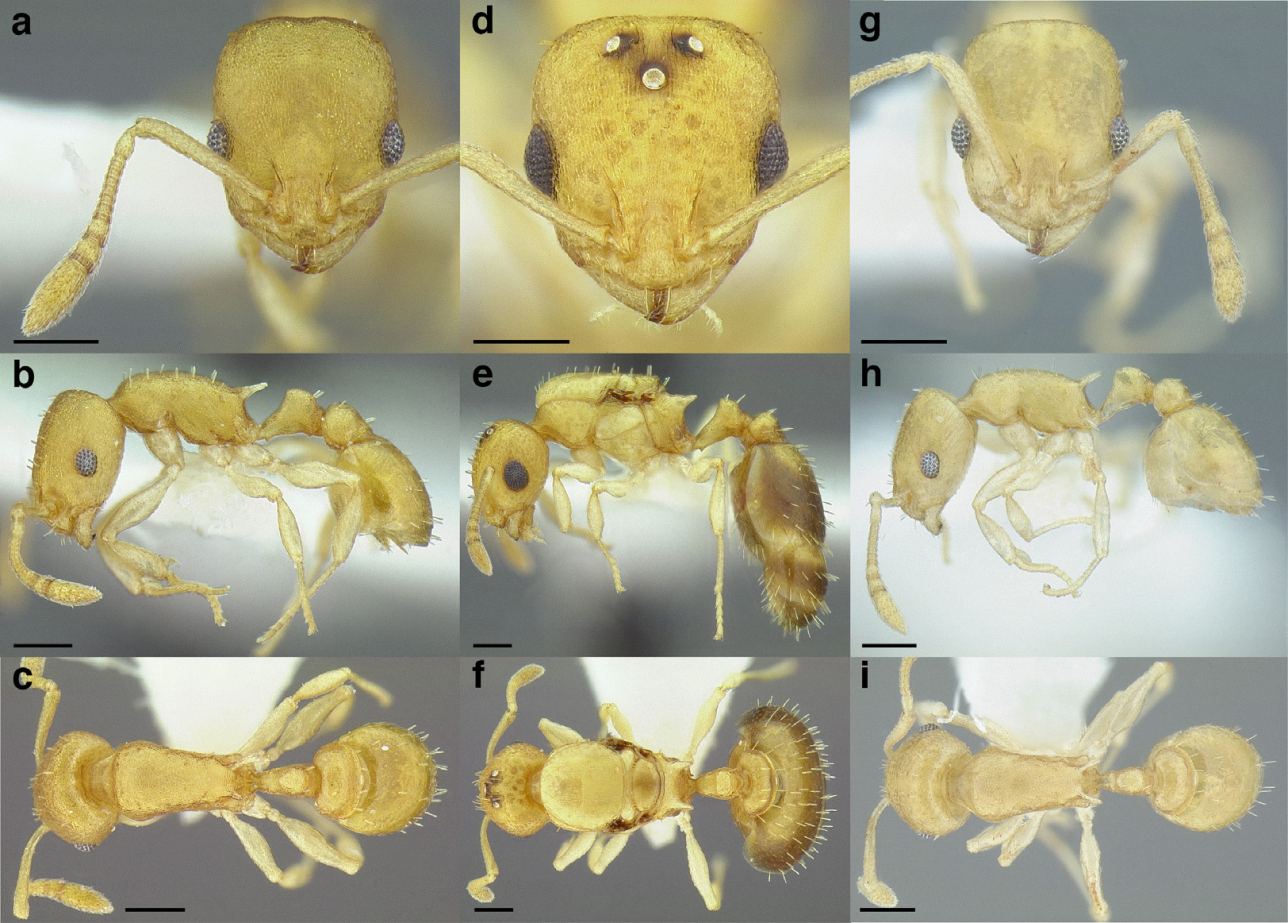
*Temnothorax torrei*. (A–C) Worker (MCZENT00583611). (A) Full-face view. (B) Profile view. (C) Dorsal view. (D–F) Gyne (MCZENT00583612). (D) Full-face view. (E) Profile view. (F) Dorsal view. (G–I) Morphological variant, Staniel Key, Bahamas (CASENT0758351). (G) Full-face view. (H) Profile view. (I) Dorsal view. Scale bars 0.2 mm.

Distribution: Fig. 133M; worker, gyne & variability: Fig. 147.

*Macromischa (Antillaemyrmex) torrei* Aguayo, 1931: 178. Holotype worker, by monotypy. Santa Clara Province, Cuba.

*Leptothorax torrei* (Aguayo): Baroni Urbani, 1978: 520. First combination in *Leptothorax*.

*Temnothorax torrei* (Aguayo): Bolton, 2003: 272. First combination in *Temnothorax*.

**Type material examined:** *Holotype worker:* CUBA: Villa Clara: Caibarién, February 1931, P.Q. Bermudez#135, M.C.Z. Type No. 16562 (MCZENT00016562) [MCZC].

**Non-type material examined:** BAHAMAS: Exuma: Staniel Key, 24.170633°N 76.439227°N, 5 m, 1 May 1991, L. Morrison#ANTC43808, leaf litter, 1 worker (CASENT0758351) [UCDC] 1 worker (CASENT0758352) [UCDC].

CAYMAN ISLANDS: Grand Cayman: 1 km up Mastic trail, 19.32000°N 81.19800°W, 8 March 2008, J.K. Wetterer#JKW-2008-03-08-32, 1 worker (CASENT0868475) [MMPC].

U.S.A.: Florida: Broward County: 4 mi S Boca Raton, 1 February 1959, H.A. Denmark, *Pinus clausa* debris, 1 dealate gyne (CASENT0867193) [FSCA]; Deerfield Beach, 17 July 1959, H.A. Denmark, *Pinus clausa* debris, 1 dealate gyne (CASENT0758306) [USNM]; Monroe County: Key Largo, Crocodile Lake National Wildlife Refuge, Post 220, 25.19603°N 80.35386°W, 4 m, 8 June 2011, C.S. Moreau#CSM2104, 1 worker (images of FMNHINS0000088654 examined on antweb.org) [FMNH]; Key Largo Hammock Botanical State Park, post 99, 25.27380°N 80.30365°W, 10 m, 11 April 2011, C.S. Moreau#CSM2216, 1 worker (CASENT0733969) [UCDC]; same data as previous, except: 25.17705°N 80.365217°W, 17 m, 11 April 2011, C.S. Moreau#CSM2208, 1 worker (CASENT0759988) [FSCA]; N. Key Largo, 25.3°N 80.28333°W, 5 m, 7 July 1986, M. Deyrup, tropical hammock, telephone pole #100, 1 alate gyne (images of CASENT0104056 examined on antweb.org) [ABS] 1 worker (images of CASENT0104057 examined on antweb.org) [ABS]; Key Vaca, Marathon, 13 July 1986, Klimaszewski & Peck, hammock litter, 1 worker (MCZENT00583611) [MCZC] 1 worker (MCZENT00583612) [MCZC]; Watson Hammock, Big Pine Key, 10 m, 16 September 1982, P.S. Ward#5749A, ground forager(s), hardwood hammock, 1 worker (CASENT0104776) [UCDC].

**Geographic range:** Low elevations: Southern Florida, Florida Keys, The Bahamas, Cayman Islands, and Cuba (Fig. 133M).

**Worker diagnosis:** *Temnothorax torrei* can be separated from all other species in the *salvini* clade by the following character combination: metanotal groove not impressed; propodeum not depressed below the level of the promesonotum; propodeum bearing standing setae dorsally; propodeal spines about as long as the propodeal declivity, and directed posterodorsally; hind femora weakly incrassate; petiolar node with only two erect setae dorsally; postpetiole very broad: 2.3 or greater times the width of the petiole; dorsum of head sculptured, although there may be a longitudinal strip of smooth and shining sculpture medially; setae on head, mesosoma, waist segments and gaster erect, short, sparse and blunt (never long and tapering); head, mesosoma, waist segments, and gaster uniformly testaceous yellow; mandibles and extremities pale yellow.

**Similar species:** *Temnothorax ciferrii, T. harlequina* sp. nov., *T. hippolytus* sp. nov*.*, and *T. terricola. Temnothorax torrei* can be distinguished from *T. ciferrii* and *T. harlequina* sp. nov. by the setae count of two on the dorsum of the petiolar node, versus four. *Temnothorax torrei* is closely related to *T. terricola* and *T. hippolytus* sp. nov., but the sculptured dorsum of the head can be used to separate them: *T. terricola* and *T. hippolytus* sp. nov. have smooth and shining heads.

**Worker measurements & indices (*n* = 4):** SL = 0.310–0.375 (0.345); FRS = 0.120–0.145 (0.138); CW = 0.389–0.440 (0.425); CWb = 0.362–0.410 (0.393); PoOC = 0.202–0.239 (0.225); CL = 0.465–0.515 (0.501); EL = 0.092–0.109 (0.102); EW = 0.071–0.085 (0.078); MD = 0.104–0.119 (0.111); WL = 0.467–0.541 (0.516); SPST = 0.138–0.209 (0.177); MPST = 0.161–0.189 (0.179); PEL = 0.200–0.250 (0.226); NOL = 0.123–0.168 (0.146); NOH = 0.077–0.110 (0.092); PEH = 0.140–0.176 (0.159); PPL = 0.124–0.166 (0.144); PPH = 0.135–0.214 (0.169); PW = 0.255–0.300 (0.283); SBPA = 0.112–0.151 (0.130); SPTI = 0.166–0.209 (0.184); PEW = 0.104–0.129 (0.118); PNW = 0.098–0.129 (0.118); PPW = 0.234–0.327 (0.287); HFL = 0.290–0.354 (0.327); HFWmax = 0.084–0.104 (0.097); HFWmin = 0.036–0.041 (0.039); CS = 0.595–0.666 (0.643); ES = 0.128–0.148 (0.141); SI = 86–93 (88); OI = 21–22 (22); CI = 125–131 (128); WLI = 129–135 (131); SBI = 30–37 (33); PSI = 30–39 (34); PWI = 225–279 (243); PLI = 139–185 (159); NI = 153–164 (159); PNWI = 94–110 (100); NLI = 61–68 (65); FI = 210–278 (249).

**Worker description**: In full-face view, head subquadrate, longer than broad (CI 125–131). Mandibles weakly striate, shining, and armed with five teeth: the apical-most well developed, followed by a smaller preapical tooth, which is followed by three equally developed smaller teeth. Anterior clypeal margin entire and evenly rounded. Median lobe of clypeus compressed between the closely approximated frontal lobes. Antennal scapes short: when fully retracted, failing to reach the posterior margin of the head capsule by about the maximum width of the scape (SI 86–93). Antennae 12-segmented; antennal club of three segments, with the apical-most segment one and a half times longer than the preceding two in combination. Frontal carinae short, extending past the antennal toruli by one and a half times the maximum width of the antennal scape. Compound eye weakly protruding past the lateral margin of the head capsule. Lateral margins of head very weakly convex, nearly flat, giving the head a boxy appearance. Posterior head margin weakly concave, rounding evenly into the lateral margins.

In profile view, compound eye ovular and moderately large (OI 21–22), with 9 ommatidia in longest row. Pronotal declivity distinct, with neck and anterior face of the pronotum forming a rounded ~110°; anterior and dorsal faces of the pronotum forming a rounded ~130° angle. Mesosoma evenly, but weakly, convex dorsally from where it joins the pronotal declivity to the propodeal spines. Promesonotal suture extending from the posterior margin of the procoxal insertion only to the mesothoracic spiracle, which is well developed. Metanotal groove nearly entirely absent: only visible as a faint disruption in the surface sculpture between meso- and metacoxal insertions to the minute metathoracic spiracle, which is nearly indistinguishable against the ground sculpture. Propodeal spiracle weakly developed, directed posterolaterally, and separated from the propodeal declivity by about three and a half spiracle diameters. Propodeal spines well developed, moderately long (PSI 30–39), about as long as the propodeal declivity, tapering evenly from the base, straight, and acute. Propodeal declivity straight and flat, forming a rounded ~90° angle with the base of the propodeal spines. Propodeal lobes rounded and very weakly developed. Metapleural gland bulla small, extending from the metacoxal insertion halfway to the propodeal spiracle. Petiole moderately long (PLI 139–185), without tubercles anterodorsally. Subpetiolar process in the form of a weakly developed, acute, triangular tooth; ventral margin of petiole strongly concave posterior to it. Petiolar peduncle short, comprising about a third of the total length of the petiole. Petiolar node robust: grading evenly into the petiolar peduncle anteriorly, anterior face concave; anterior face forming a rounded, ~90° angle with the dorsal face, which is evenly, strongly convex; dorsal face rounding evenly into the posterior face, which forms a ~110° angle with the caudal cylinder. Postpetiole evenly rounded anterodorsally, before flattening posterodorsally; weakly lobed ventrally.

In dorsal view, humeri weakly developed: rounded and only slightly wider than the rest of the mesosoma; mesothoracic spiracles weakly protruding past the lateral margins of the mesosoma, visible as slight angles where the pronotum meets the mesonotum. Metanotal groove absent: mesonotum and propodeum completely fused and converging evenly to the bases of the propodeal spines. Propodeal spines broadly approximated basally and diverging apically, their apices separated from each other about their length; negative space between them “U” shaped. Petiolar peduncle with spiracles barely protruding past the lateral margins, peduncle not constricted anterior to them. Petiolar node ovular, subtly narrowed anteriorly, slightly wider than the peduncle, and evenly grading into the caudal cylinder, which is slightly wider than the node. Postpetiole very broad (PWI 225–279) and campaniform, articulating with nearly the entire anterior margin of the gaster, leaving small angulate margins on each side exposed. Anterior margin of the postpetiole flat, with corners evenly rounding into the lateral margins, which evenly diverge to the angulate posterior corners; posterior margin broadly concave. Metafemur weakly to moderately incrassate (FI 210–278).

Sculpture: median clypeal carina present, flanked two additional equally developed carinae over weakly areolate ground sculpture. Antennal scapes shining through weak, indistinct sculpture. Cephalic dorsum uniformly areolate. Mesosoma uniformly areolate, except for propodeal declivity which is smooth and shining. Femora moderately shining through weak areolate sculpture. Petiole smooth and shining ventrally, with shallow areolate sculpture surrounding the base of the petiolar node, which is shining dorsally through weak, indistinct sculpture. Dorsal surface of postpetiole smooth and shining, with shallow areolate sculpture on the posterior third areolate. Gaster smooth and shining, without spectral iridescence on the first tergite. Surface of the first gastral sternite smooth and shining.

Setae: antennal scapes and funiculi with short, adpressed pilosity. Dorsum of head, mesosoma, waist segments and gaster with short, erect, blunt-tipped setae; the longest setae are roughly the length of the compound eye. The head dorsum bears ~26, mesosoma dorsum ~14, petiole 2, postpetiole ~6, and first gastral tergite ~20 setae. Sparse, adpressed pubescence present on the entire body, but difficult to detect against the lightly colored integument.

Color: head, mesosoma, waist segments, and gaster uniformly testaceous yellow. Mandibles and extremities pale yellow.

**Gyne measurements & indices (*n* = 2):** SL = 0.393–0.401 (0.397); FRS = 0.171–0.186 (0.179); CW = 0.556–0.565 (0.561); CWb = 0.526–0.533 (0.530); PoOC = 0.222–0.231 (0.227); CL = 0.572–0.58 (0.576); EL = 0.178–0.179 (0.179); EW = 0.136–0.145 (0.141); MD = 0.104–0.105 (0.105); WL = 0.878–0.888 (0.883); SPST = 0.206–0.214 (0.210); MPST = 0.218–0.226 (0.222); PEL = 0.307–0.311 (0.309); NOL = 0.167–0.177 (0.172); NOH = 0.108–0.108 (0.108); PEH = 0.211–0.221 (0.216); PPL = 0.146–0.153 (0.15); PPH = 0.234–0.244 (0.239); PW = 0.514–0.531 (0.523); SBPA = 0.280–0.282 (0.281); SPTI = 0.252–0.256 (0.254); PEW = 0.152–0.169 (0.161); PNW = 0.185–0.192 (0.189); PPW = 0.379–0.382 (0.381); HFL = 0.426–0.437 (0.432); HFWmax = 0.106–0.114 (0.110); HFWmin = 0.043–0.047 (0.045); CS = 0.812–0.823 (0.818); ES = 0.247–0.251 (0.249); SI = 75; OI = 30; CI = 109; WLI = 165–169 (167); SBI = 53–54 (53); PSI = 23–24 (24); PWI = 224–251 (238); PLI = 203–210 (207); NI = 155–164 (159); PNWI = 114–122 (118); NLI = 54–57 (56); FI = 243–247 (245).

**Gyne description:** In full-face view, head trapezoidal, longer than broad (CI 109). Mandibles weakly striate, shining, and armed with five teeth: the apical-most well developed, followed by a less developed preapical tooth and three equally developed smaller teeth. Anterior clypeal margin evenly rounded. Antennal scapes short: when fully retracted, failing to reach the posterior margin of the head capsule by about the maximum width of the antennal scape (SI 75). Antennae 12-segmented; antennal club of three segments, with the apical-most segment one and a half times longer than the preceding two in combination. Frontal carinae moderately long, extending past the antennal toruli by about three times the maximum width of the antennal scape. Compound eye moderately protruding past the lateral margin of the head capsule. Lateral margins of head weakly convex; temples enlarged, converging evenly to the mandibular insertions. Posterior head margin weakly convex, rounding evenly into the lateral margins.

In profile view, compound eye teardrop-ovular and large (OI 30), with 16 ommatidia in longest row. Mesoscutum rounded evenly anteriorly, covering the dorsal surface of the pronotum, and flat dorsally. Mesoscutellum on the same level as the mesoscutum, not overhanging the metanotum. Propodeal spiracle well developed, directed posterolaterally, and separated from the propodeal declivity by about two spiracle diameters. Propodeal spines stout and short (PSI 23–24), about a third as long as the propodeal declivity, widely flared at the base, straight, and directed posteriorly. Propodeal declivity straight and flat, forming a rounded ~90° angle with the base of the propodeal spines. Propodeal lobes rounded and very weakly developed. Metapleural gland bulla small, extending from the metacoxal insertion halfway to the propodeal spiracle. Petiole long (PLI 203–210), without tubercles anterodorsally. Subpetiolar process a very weakly developed, tiny, triangular tooth, which grades evenly into the ventral margin of the petiole posteriorly. Petiolar peduncle short: comprising about a third of the total length of the petiole. Petiolar node erect: transition between peduncle and node evenly rounded, resulting in a concave anterior node face; anterior face forming a sharp ~90° angle with the dorsal face, which is short and evenly rounds into the posterior face, which forms a ~110° angle with the caudal cylinder. Postpetiole subquadrate: transition between anterior face and flat dorsal face marked by a broadly rounded ~90° angle; ventral surface weakly lobed.

In dorsal view, mesoscutum covering pronotum anteriorly, but humeri visible laterally as rounded sclerites. Propodeal spines very weakly diverging apically, their apices separated from each other by about twice their length. Petiolar peduncle with spiracles not protruding past the lateral margins. Petiolar node weakly trapezoidal: widest anteriorly, with lateral sides converging only slightly to the posterior face, and weakly emarginated anterodorsally. Petiolar node slightly narrower than the peduncle, and evenly grading into the caudal cylinder, which slightly broader than the node. Postpetiole very broad (PWI 224–251), anteroposteriorly compressed, and subquadrate, articulating with most of the anterior margin of the gaster, leaving small, angulate margins on each side exposed. Anterior margin of the postpetiole flat, evenly rounded as it transitions to the lateral margins, which are parallel as they continue to the angulate posterior corners; posterior margin emarginated medially. Femora weakly incrassate (FI 243–247).

Sculpture: median clypeal carina present, extending from the anterior margin to the level of the antennal insertions, and flanked by weaker, indistinct carinae; lateral margins of median clypeal lobe with two carinae that are as strong as the medial carina. Lateral clypeal lobes with additional weaker carinae; ground sculpture weakly areolate. Antennal scapes weakly shining through areolate sculpture. Cephalic dorsum with weak costulae over areolate sculpture; ventral surface of head with slightly weaker, indistinct sculpture. Pronotal neck areolate. Lateral surfaces of pronotum weakly shining through superficial areolate ground sculpture. Anepisternum and katepisternum mostly smooth and shining, with margins weakly, indistinctly sculptured. Propodeum areolate laterally, with weak costulae over the metapleural gland bulla; propodeal declivity weakly areolate. Mesoscutum with costulae over weak areolate ground sculpture surrounding a smooth and shining central strip which extends over half of the sclerite from the anterior margin; mesoscutum also with smooth and shining patches laterally. Mesoscutellum smooth and shining medially, surrounded by weak costulae and areolae. Femora smooth and shining, with traces of weak areolate sculpture distally. Petiole with weak areolate sculpture ventrally and on the dorsal face of the peduncle, areolate on all other surfaces. Postpetiole areolate. Gaster smooth and shining, without spectral iridescence. Surface of the first gastral sternite smooth and shining.

Setae: antennal scapes and funiculi with short, adpressed pilosity. Dorsum of head, mesosoma, waist segments and gaster with short, erect, blunt-tipped setae; the longest setae are roughly a third of the width of the compound eye. Sparse, adpressed pubescence present on the entire body, but difficult to detect against the lightly colored integument.

Color: head, mesosoma, waist segments, and gaster uniformly testaceous yellow. Mandibles and extremities pale yellow. Integument within the ocellar triangle and the wing bases infuscated.

**Male:** Unknown.

**Etymology:** Patronym, in honor of Carlos de la Torre, who sent the type series to C. G. Aguayo for description.

**Comments:** Known primarily from stray ground foragers and specimens collected via leaf litter sifting, *Temnothorax torrei* ranges from the Cayman Islands to Cuba, the Florida Keys and southern Florida. *Temnothorax torrei* is also most likely present on the islands of the Bahamas. This species, like other members of the *pulchellus* group, is probably ground or leaf litter nesting. The original description states that is species was found among terrestrial snail shells collected by Bermudez in “Buenavista”, Remedios, Santa Clara Province, Cuba. The collection data label on the holotype specimen from the MCZ does not match this verbal description but Caibarién, the locality indicated, is located roughly 18 km north of Buenavista, on the coast. Perhaps the label data indicates the closest city to the collection locality. This species is most closely related to *T. terricola*. These species have only been collected in allopatry and differ primarily color and/or extent of areolate vs. smooth sculpturation. Future collections from the Bahamas and Cuba may reveal that these two “species” represent morphologically divergent populations, which are united by intermediate forms. For example, a morphological variant of *T. torrei* was collected from Staniel Key, The Bahamas, with very weak head sculpture (Figs. 147G–147I). *Temnothorax hippolytus* sp. nov. is also a member of this complex but differs from the other members by the more well-defined promesonotal suture, predominantly smooth and shining sculpture, and dark brown coloration. The illustration of *T. torrei* in Baroni Urbani (1978) embellishes certain aspects of the holotype specimen, which bears many fewer setae than depicted.

***Temnothorax wettereri* sp. nov.**

**Figure 148:**
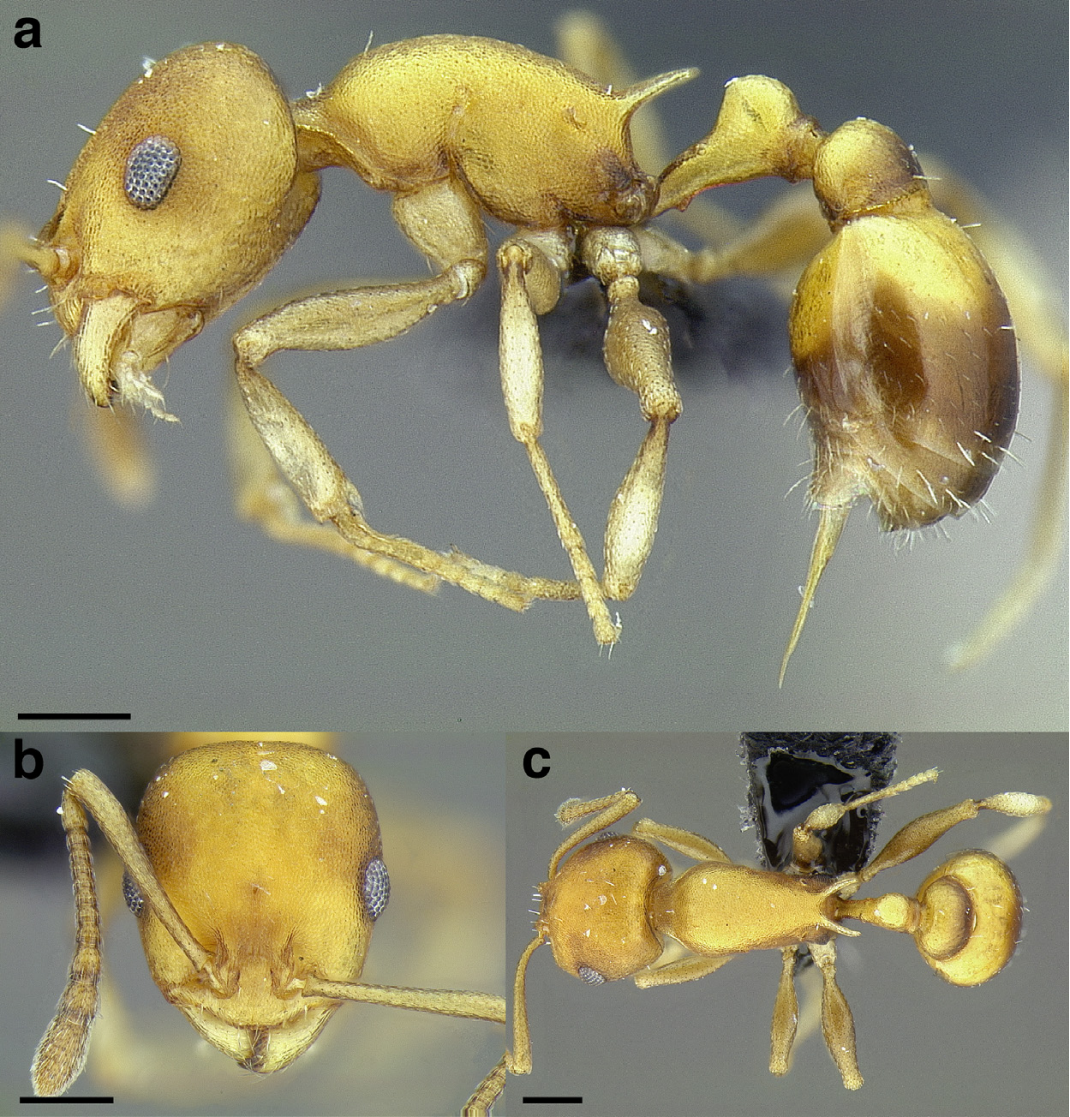
*Temnothorax wettereri* sp. nov. holotype worker (CASENT0756094). (A) Profile view. (B) Full-face view. (C) Dorsal view. Scale bars 0.2 mm.

Distribution: Fig. 133N; worker: Fig. 148.

**Type material examined:** *Holotype worker:* ANTIGUA AND BARBUDA: Barbuda: Gut Road, 4 km W of town, 17.63°N 61.783°W, 20 m, 11 June 2007, J.K. Wetterer#JKW-2007-483 (CASENT0756094) [USNM].

*Paratype workers:* same data as holotype, 1 worker (CASENT0758675) [UCDC] 1 worker (CASENT0758818) [MCZC].

**Geographic range:** Barbuda (Fig. 133N).

**Worker diagnosis:** *Temnothorax wettereri* sp. nov. can be separated from all other species in the *salvini* clade by the following character combination: head in full face view with posterior margin flat to slightly convex; mesosoma compact; in profile view, dorsum of mesosoma evenly convex; metanotal groove not impressed; propodeum not depressed below the level of the promesonotum; propodeum without standing setae dorsally; propodeal spines slightly longer than the propodeal declivity, directed posterodorsally, and very slightly downcurved; in dorsal view, propodeal spines broadly approximated basally, the negative space between them “U” shaped; hind femora weakly to moderately incrassate; petiolar node rounded dorsally; in dorsal view, apex of petiolar node the same width as or slightly broader than the caudal cylinder of the petiole; postpetiole very broad: greater than 2.4 times the width of the petiole; median clypeal carina obscured by areolate ground sculpture; dorsum of head uniformly areolate; mesosoma bearing less than ten erect setae dorsally; setae on head, mesosoma, waist segments and gaster erect, moderately long, sparse and blunt (never long and tapering); integument uniformly light brown, with the surface of the metapleural gland, posterior margin of the postpetiole, and posterior halves of the gastral sclerites darker brown; mandibles, tibiae, and tarsi pale yellow.

**Similar species:** *Temnothorax agavicola* sp. nov., *T. albispinus, T. laticrus* sp. nov., *T. torrei*, and members of the *pastinifer* group. *Temnothorax wettereri* sp. nov. can be separated from most members of the *pastinifer* group by the petiolar node, which not squamiform and relatively narrow in dorsal view: 1 to 1.1 times the width of the caudal cylinder of the petiole in *T. albispinus* versus greater than 1.3 times in the *pastinifer* group. The petiolar node shape, in addition to the long propodeal spines and relatively narrow hind femora will distinguish *T. wettereri* sp. nov. from *T. laticrus* sp. nov. The lack of erect setae on the dorsum of the petiolar node differentiates *T. wettereri* sp. nov. from all of the above species. The flat to weakly convex posterior margin of the head of *T. wettereri* sp. nov. contrasts with *T. agavicola* sp. nov., which has a weakly concave posterior head margin. The body of *T. wettereri* sp. nov. is mostly devoid of erect setae, whereas *T. albispinus* has more than ten erect setae on the dorsum of the mesosoma.

**Worker measurements (*n* = 2):** SL = 0.476–0.519 (0.494); FRS = 0.177–0.186 (0.181); CW = 0.550–0.578 (0.565); CWb = 0.505–0.532 (0.519); PoOC = 0.221–0.247 (0.231); CL = 0.585–0.619 (0.601); EL = 0.136–0.145 (0.141); EW = 0.105–0.109 (0.107); MD = 0.153–0.162 (0.157); WL = 0.619–0.646 (0.630); SPST = 0.225–0.241 (0.234); MPST = 0.190–0.212 (0.203); PEL = 0.276–0.295 (0.283); NOL = 0.169–0.187 (0.179); NOH = 0.104–0.118 (0.112); PEH = 0.188–0.206 (0.196); PPL = 0.153–0.180 (0.167); PPH = 0.193–0.210 (0.202); PW = 0.334–0.360 (0.350); SBPA = 0.136–0.140 (0.138); SPTI = 0.189–0.204 (0.199); PEW = 0.119–0.138 (0.130); PNW = 0.135–0.145 (0.139); PPW = 0.291–0.326 (0.311); HFL = 0.455–0.486 (0.470); HFWmax = 0.114–0.119 (0.117); HFWmin = 0.040–0.046 (0.043); CS = 0.798–0.842 (0.820); ES = 0.191–0.199 (0.195); SI = 93–98 (95); OI = 24; CI = 86–87 (86); WLI = 120–123 (121); SBI = 26–27 (27); PSI = 36–38 (37); PWI = 236–245 (239); PLI = 163–182 (170); NI = 153–163 (159); PNWI = 100–113 (107); NLI = 61–65 (63); FI = 259–285 (275).

**Worker description:** In full-face view, head subquadrate, longer than broad (CI 86–87). Mandibles striate, weakly shining, and armed with five teeth: the apical-most well developed, followed by a smaller preapical tooth, which is followed by three equally developed smaller teeth. Anterior clypeal margin flat to very slightly concave medially. Antennal scapes moderately long: when fully retracted, extending past the posterior margin of the head capsule by about the maximum width of the antennal scape (SI 93–98). Antennae 12-segmented; antennal club of three segments, with the apical-most segment longer than the preceding two in combination. Frontal carinae very short, extending past the antennal toruli by the maximum width of the antennal scape. Compound eyes moderately protruding past the lateral margins of the head capsule. Lateral margin of head weakly convex, converging evenly to the mandibular insertions. Posterior head margin flat to slightly convex, rounding evenly into the lateral margins.

In profile view, compound eyes ovular and moderately large (OI 24), with 11 ommatidia in longest row. Pronotal declivity indistinct, neck and anterior face of pronotum forming a rounded ~120° angle. Mesosoma evenly, but weakly, convex dorsally from where it joins the pronotal neck to the propodeal spines. Promesonotal suture extending from the posterior margin of the procoxal insertion only to the mesothoracic spiracle, which is well developed. Metanotal groove nearly entirely absent: only visible as a faint disruption in the surface sculpture between meso- and metacoxal insertions to the minute metathoracic spiracle, which is nearly indistinguishable against the ground sculpture. Propodeal spiracle well developed, directed posterolaterally, and separated from the propodeal declivity by about three spiracle diameters. Propodeal spines well developed and moderately long (PSI 36–38), about as long as the propodeal declivity, tapering evenly from the base, very slightly downcurved at the apical quarter, and acute. Propodeal declivity weakly concave, forming a rounded ~110° angle with the base of the propodeal spines. Propodeal lobes rounded and very weakly developed. Metapleural gland bulla small, extending from the metacoxal insertion halfway to the propodeal spiracle. Petiole moderately long (PLI 163–182), without tubercles anterodorsally; subpetiolar process in the form of a weakly developed, blunt tooth; ventral surface of petiole slightly concave posterior to the process. Petiolar peduncle moderately long: comprising about half the total length of the petiole. Petiolar node robust and erect: transition between peduncle and node marked by a rounded angle of ~120°, resulting in a strongly concave anterior node face; anterior face forming a rounded ~90° angle with the dorsal face, which is evenly convex and short; dorsal face rounding evenly into the posterior face, which forms a ~100° angle with the caudal cylinder. Postpetiole evenly rounded anterodorsally, before flattening posterodorsally; weakly lobed ventrally.

In dorsal view, humeri weakly developed: rounded and only slightly protruding past the rest of the mesosoma; mesothoracic spiracles protruding past the lateral margins of the mesosoma, visible as slight angles where the pronotum meets the mesonotum. Metanotal groove absent: mesonotum and propodeum completely fused and converging evenly to the bases of the propodeal spines. Propodeal spines broadly approximated basally and diverging apically, their apices separated from each other roughly by their length; negative space between them “U” shaped. Petiolar peduncle with spiracles slightly protruding past the lateral margins. Petiolar node weakly campaniform, with the posterior margins rounded, slightly wider than the peduncle, and evenly grading into the caudal cylinder, which is slightly wider than the node. Postpetiole very broad (PWI 236–245) and campaniform, articulating with the nearly the entire anterior margin of the gaster, leaving rounded angles of the gaster on each side exposed anteriorly. Anterior margin of the postpetiole broadly convex, with corners evenly rounding into the lateral margins, which evenly diverge to the angulate posterior corners; posterior margin broadly concave. Metafemur moderately incrassate (FI 259–285).

Sculpture: median clypeal carina present but very weak, extending halfway from the anterior clypeal margin to the frontal triangle before becoming obscured by areolate ground sculpture. Antennal scapes weakly areolate and dull. Cephalic dorsum and lateral surfaces of head densely areolate. Ventral surface of head shining through weaker areolate sculpture. Mesosoma evenly, densely areolate. Femora shining through weak areolate sculpture. Peduncle of petiole smooth and shining ventrally, with shallow areolate sculpture surrounding the base of the petiolar node, which is weakly shining through weak areolate sculpture dorsally. Dorsal surface of postpetiole dull with weak costulae and shallow areolae. Gaster smooth and shining, with very weak spectral iridescence on the first tergite. Surface of the first gastral sternite smooth and shining.

Setae: antennal scapes and funiculi with short, adpressed pilosity. Setae sparse on all body surfaces: dorsum of head with short, erect, blunt-tipped setae, the longest of which are roughly the ½ the width of the compound eye. The head bears ~16, mesosoma ~4, postpetiole ~2, and first gastral tergite ~4 erect setae. Sparse, adpressed pubescence present on the entire body, but only apparent on the gaster due to the dense ground sculpture on the rest of the body.

Color: uniformly light brown, with the surface of the metapleural gland, posterior margin of the postpetiole, and posterior halves of the gastral sclerites darker brown. Mandibles, tibiae, and tarsi are pale yellow.

**Gyne:** Unknown.

**Male:** Unknown.

**Etymology:** Patronym, in honor of the collector of the type series, James K. Wetterer.

**Comments:** *Temnothorax wettereri* sp. nov. is known only from the type series. Barbuda is a low-lying coral limestone island with little topographical variation, supporting primarily tropical scrub vegetation. The climate is tropical marine, with little seasonal temperature variation. The natural history of this species remains unknown but is likely to be similar to other members of the ground and leaf litter nesting *pulchellus* group.

***Temnothorax wilsoni* sp. nov.**

**Figure 149:**
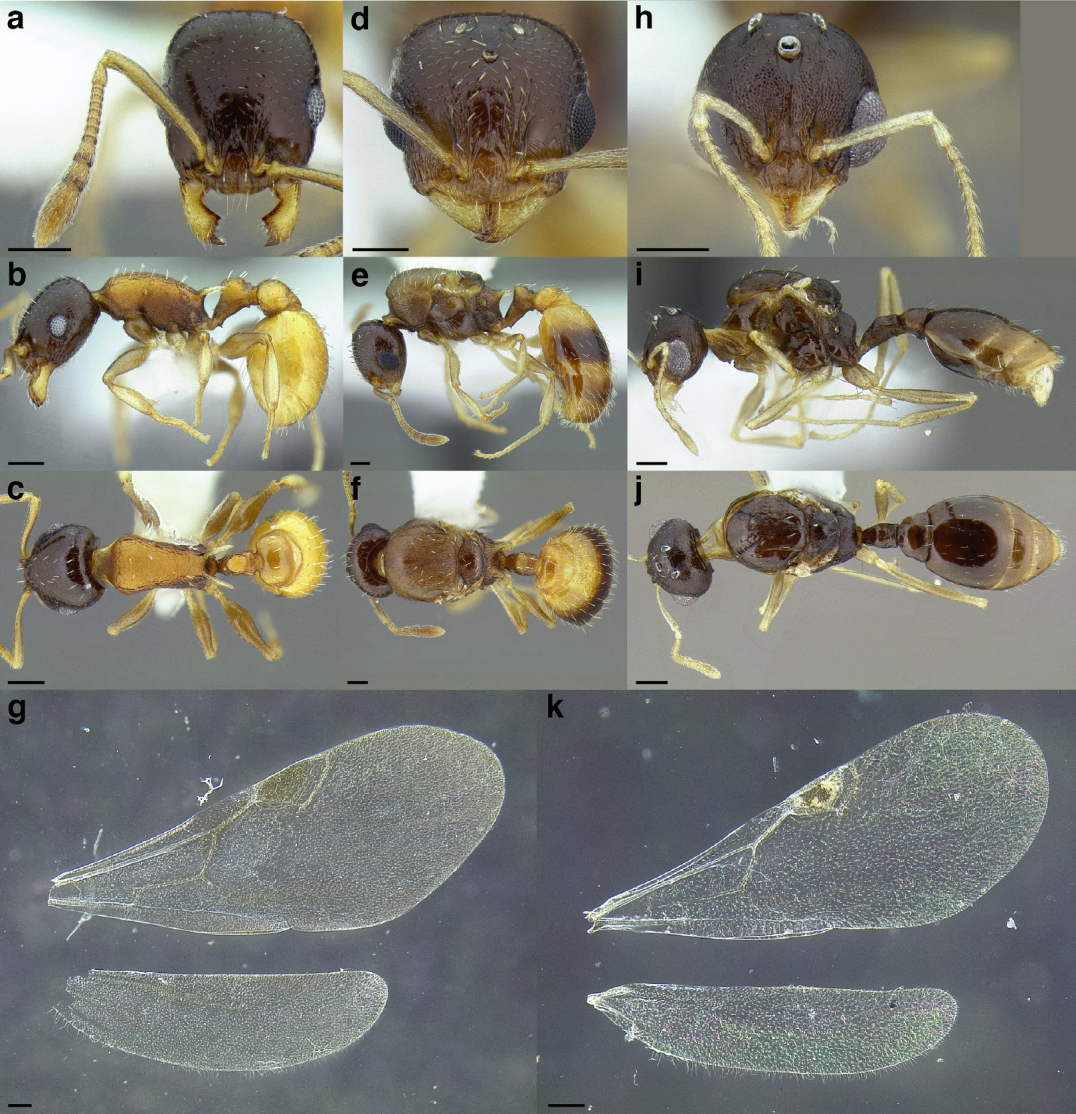
*Temnothorax wilsoni* sp. nov. (A–C) Holotype worker (MCZENT00583608). (A) Full-face view. (B) Profile view. (C) Dorsal view. (D–G) Paratype gyne (CASENT0758813). (D) Full-face view. (E) Profile view. (F) Dorsal view. (G) Wings. (H–K) Paratype male (CASENT0758813). (H) Full-face view. (I) Profile view. (J) Dorsal view. (K) Wings. Scale bars 0.2 mm.

Distribution: Fig. 133O; worker, gyne & male: Fig. 149.

**Type material examined:** *Holotype worker:* DOMINICAN REPUBLIC: La Vega: Valle Nuevo Scientific Reserve, 18.77800°N 70.63950°W ± 5 m, 2,255 m, 3 December 2003, S.P. Cover#SPCDR-53, pine woodland, nest under rock (MCZENT00583608, top specimen on pin) [MCZC].

*Paratype workers and gyne:* same pin as holotype (bottom specimen on pin) [MCZC]; same data as holotype, 2 workers (MCZENT00583609) [MCZC], 1 worker (CASENT0756092) [MNHNSD], 1 dealate gyne (MCZENT00583610) [MCZC].

**Non-type material examined:** DOMINICAN REPUBLIC: La Vega: same data as holotype, except: Valle Nuevo, Estación Moscoso Puello, 8.769000°N 70.639667°W, 2,262 m, M. Deyrup, open hillside, under rock, *Lepto*. nest #3, 1 male, 1 alate gyne, 2 workers (CASENT0758813) [ABS]; same data as holotype, except: S.P. Cover #SPC DR-48, open pine-juniper woodland, nest under rock, 1 worker (CASENT0758670) [UCDC]; same data as holotype, except: S.P. Cover #SPC DR-51, pine woodland with grassy understory to 12 m tall on steep S facing slope; superficial nest under rock in shade, sandy clay, 1 worker (CASENT0758820) [MCZC] 1 worker (CASENT0758821) [MCZC]; same data as holotype, except: S.P. Cover #SPC DR-59, 1 worker (CASENT0756090) [MCZC]; Valle Nuevo Scientific Reserve, 18.770683°N 70.639633°W ± 8 m, 2,262 m, Lloyd R. Davis, Jr.#031203-3, under rock, 1 worker (MCZENT00513364) [MCZC] same data as previous, except:# 031203-4, under rock, 1 worker (MCZENT00513365) [MCZC]; same data as previous, except: #031203-7, under rock, 1 worker (MCZENT00513366) [MCZC]; same data as previous, except: #031203-9, under rock, 1 worker (MCZENT00513358) [MCZC]; Valle Nuevo, 2,262 m, N 18°46.14′, W 070°38.38′, Estac. Moscoso Puello, open hillside, M. Deyrup, under rock, *Lepto*. nest #1, Wilson Harvard Expedition, 5 workers (MCZENT00513144) [MCZC] 5 workers (MCZENT00513145) [MCZC] 5 workers (CASENT0758814) [ABS] 5 workers (CASENT0758815) [ABS]; Valle Nuevo, Estación Moscoso Puello, 2,262 m, N 18.769000°N 70.639667°W, M. Deyrup, open hillside, under rock, *Lepto*. nest #2, 5 workers (MCZENT00513146) [MCZC] 5 workers (MCZENT00513147) [MCZC] 5 workers (MCZENT00513148) [ABS] 5 workers (MCZENT00513149) [MCZC] 5 workers (MCZENT00513150) [MCZC] 5 workers (MCZENT00513151) [MCZC] 5 workers (MCZENT00513152) [MCZC] 5 workers (MCZENT00513153) [MCZC] 5 workers (MCZENT00513154) [ABS] 5 workers (MCZENT00513155) [ABS].

**Geographic range:** High elevations of Hispaniola (Fig. 133O).

**Worker diagnosis:** *Temnothorax wilsoni* sp. nov. can be separated from all other species in the *salvini* clade by the following character combination: anterior clypeal margin medially emarginate; in profile view, dorsum of mesosoma weakly convex; metanotal groove not impressed; propodeum not depressed below the level of the promesonotum; propodeum bearing standing setae dorsally; propodeal spines longer than the propodeal declivity, directed posteriorly, and weakly downcurved; in dorsal view, propodeal spines broadly approximated, the negative space between them “U” shaped; hind femora weakly to strongly incrassate; in dorsal view, apex of petiolar node narrower than the caudal cylinder of the petiole; petiolar node with four erect setae dorsally; postpetiole very broad: greater than or equal to 2.3 times the width of the petiole; dorsum of head smooth and shining; setae on head, mesosoma, waist segments and gaster erect, moderately long, sparse and blunt (never long and tapering);integument tricolored: predominantly testaceous yellow, with a dark brown head capsule; femora, postpetiole, and lower half of the mesosomal pleurites testaceous; gaster yellow, often with two posterolateral slightly darker spots on the first gastral tergite.

**Similar species:** *Temnothorax bahoruco* sp. nov., *T. balaclava* sp. nov., *T. ciferrii, T. harlequina* sp. nov., *T. hippolytus* sp. nov., *T. magnabulla* sp. nov., *T. pulchellus, T. schwarzi*, and *T. terricola*. The erect setae count of four on the dorsum of the petiolar node differentiates *T. wilsoni* sp. nov. from *T. terricola* and *T. hippolytus* sp. nov., which have only two along the posterior margin. The presence of erect setae on the propodeum distinguishes *T. wilsoni* sp. nov. from *T. pulchellus*. The relatively small metapleural gland bulla of *T. wilsoni* sp. nov., which reaches two thirds of the way to from the metacoxal insertion to the propodeal spiracle, distinguishes it from *T. magnabulla* sp. nov., in which it extends three quarters of the way or more. *Temnothorax wilsoni* sp. nov. and *T. harlequina* sp. nov. can be separated most easily by the color of the integument: *T. harlequina* sp. nov. has a light yellow gaster which strongly contrasts with the darker integument of the rest of the body, whereas the gaster of *T. wilsoni* sp. nov. is always medium yellow, or bears dark spots; additionally, the dorsum of the mesosoma and waist segments of *T. wilsoni* sp. nov. are always testaceous yellow. The mesosoma is of *T. wilsoni* sp. nov. is less arched and more densely sculptured in comparison to *T. harlequina* sp. nov. *Temnothorax wilsoni* sp. nov. could also be confused with *T. schwarzi* due to the smooth and shining head capsule, but the relatively narrow apex of the petiolar node of *T. wilsoni* sp. nov. will separate the two. The medially emarginate anterior clypeal margin of *T. wilsoni* sp. nov. will differentiate it from *T. balaclava* sp. nov. and *T. bahoruco* sp. nov.

**Worker measurements & indices (*n* = 10):** SL = 0.462–0.537 (0.505); FRS = 0.202–0.231 (0.213); CW = 0.548–0.628 (0.595); CWb = 0.512–0.579 (0.546); PoOC = 0.234–0.273 (0.251); CL = 0.596–0.671 (0.636); EL = 0.136–0.165 (0.150); EW = 0.104–0.133 (0.120); MD = 0.134–0.164 (0.148); WL = 0.692–0.781 (0.731); SPST = 0.226–0.289 (0.256); MPST = 0.219–0.274 (0.233); PEL = 0.293–0.348 (0.318); NOL = 0.158–0.187 (0.175); NOH = 0.102–0.125 (0.111); PEH = 0.190–0.231 (0.211); PPL = 0.171–0.209 (0.188); PPH = 0.212–0.245 (0.231); PW = 0.367–0.428 (0.393); SBPA = 0.147–0.187 (0.171); SPTI = 0.251–0.316 (0.287); PEW = 0.144–0.177 (0.161); PNW = 0.119–0.157 (0.141); PPW = 0.346–0.408 (0.376); HFL = 0.474–0.530 (0.504); HFWmax = 0.122–0.150 (0.136); HFWmin = 0.045–0.062 (0.050); CS = 0.810–0.915 (0.864); ES = 0.188–0.228 (0.210); SI = 88–98 (93); OI = 23–26 (24); CI = 84–88 (86); WLI = 129–138 (134); SBI = 29–34 (31); PSI = 29–39 (35); PWI = 225–241 (234); PLI = 146–187 (170); NI = 145–168 (157); PNWI = 77–96 (88); NLI = 52–61 (55); FI = 240–313 (270).

**Worker description:** In full-face view, head subquadrate, longer than broad (CI 84–88). Mandibles densely striate but shining and armed with five teeth: the apical-most well developed and acute, followed by a less developed preapical tooth and three equally developed smaller teeth. Anterior clypeal margin weakly emarginated medially. Antennal scapes moderately long: when fully retracted, just reaching the posterior margin of the head capsule (SI 88–98). Antennae 12-segmented; antennal club of composed of three segments, with the apical-most segment slightly longer than the preceding two in combination. Frontal carinae moderately long, extending past the antennal toruli by about two times the maximum width of the antennal scape. Compound eyes moderately protruding past the lateral margins of the head capsule. Lateral margin of head weakly convex, forming a continuous arc from the mandibular insertions to the posterior margin of the head. Posterior head margin flat but rounding evenly into the lateral margins.

In profile view, compound eyes ovular and moderately large (OI 23–26), with 9 ommatidia in longest row. Pronotal declivity indistinct, neck and anterior face of pronotum forming a ~120° angle. Mesosoma weakly convex from where it joins the pronotal neck to the propodeal spines. Promesonotal suture extending from the posterior margin of the procoxal insertion only to the mesothoracic spiracle, which is moderately well developed. Metanotal groove visible as a disruption of the sculpture laterally from where it arises between the mid- and hind coxae to where it ends in the poorly developed metathoracic spiracle, which is nearly indistinguishable against the ground sculpture. Propodeal spiracle well developed, directed posterolaterally, and separated from the propodeal declivity by about three spiracle diameters. Propodeal spines well developed and moderately long (PSI 29–39), about as long as the propodeal declivity, tapering evenly from the base, weakly downcurved, and acute. Propodeal declivity weakly concave, forming a rounded ~100° angle with the base of the propodeal spines. Propodeal lobes rounded and weakly developed. Metapleural gland bulla moderately large, extending from the metacoxal insertion two thirds of the way to the propodeal spiracle. Petiole moderately long (PLI 146–187), without tubercles anterodorsally. Subpetiolar process in the form of a small, acute tooth; ventral margin of petiole weakly concave posterior to it. Petiolar peduncle short: petiolar node covering most of the petiolar dorsum. Petiolar node robust: transition between peduncle and node marked by a rounded angle of ~140°, resulting in a weakly concave anterior node face; anterior face forming a ~90° angle with the dorsal face, which is weakly convex; dorsal face rounding evenly into the short posterior face, which forms a ~110° angle with the caudal cylinder. Postpetiole evenly rounded anteriorly, flattened dorsally, and weakly lobed ventrally.

In dorsal view, humeri developed and distinct: evenly rounded and wider than the rest of the mesosoma; mesothoracic spiracles weakly protruding past the lateral margins of the mesosoma, visible as slight angles where the pronotum meets the mesonotum. Metanotal groove absent: mesonotum and propodeum completely fused and lateral margins converging evenly to the bases of the propodeal spines. Propodeal spines broadly approximated basally and diverging apically, their apices separated from each other by about their length, the negative space between them “U” shaped. Petiolar peduncle with spiracles protruding past the lateral margins, but not noticeably constricted anterior to them. Petiolar node evenly ovular; node the same width as the peduncle, and evenly grading into the caudal cylinder, which is wider than the node. Postpetiole very broad (PWI 225–241) and campaniform, articulating with the nearly the entire anterior margin of the gaster. Anterior margin of the postpetiole convex and evenly rounds into the lateral margins, which diverge to the angulate posterior corners; posterior margin broadly concave. Metafemur weakly to strongly incrassate (FI 240–313).

Sculpture: median clypeal carina present, extending posteriorly nearly to the frontal triangle, and flanked on either side by two equally strong carinae. Lateral clypeal lobes with additional, weaker carinae; ground sculpture smooth and shining. Antennal scapes shining through weak areolate ground sculpture. Cephalic dorsum smooth and shining, but with coarse piligerous punctures; costulae flanking the frontal carinae. Lateral surfaces of head with weak areolate sculpture posterior to the compound eye, dense rugulose sculpture surrounding the compound eye, and weak rugae between the compound eye and the mandibular insertion. Ventral surface of head smooth and shining. Mesosoma with areolate sculpture on the pronotal neck. Lateral surfaces of the pronotum, mesopleurae, and anterior half of the lateral surface of propodeum areolate, but smooth and shining between the propodeal spiracle and the propodeal spines. Dorsal surface of mesosoma with weak costulae on the pronotum and lateral margins over areolate ground sculpture. Femora shining, with weak areolate sculpture on the distal third. Petiole smooth and shining ventrally, with areolate sculpture on all other surfaces; a weak carina present laterally, extending longitudinally from the petiolar spiracle to the caudal cylinder. Postpetiole smooth and shining, with weak areolate sculpture on the posterior quarter. Gaster smooth and shining, without spectral iridescence.

Setae: antennal scapes and funiculi with short, decumbent pilosity. Dorsum of the head, pronotum, waist segments, and gaster with moderately abundant, erect, blunt-tipped setae, the longest of which are about the width of the compound eye. The head bears ~30, mesosoma ~18, petiole 4, postpetiole ~14, and first gastral tergite ~36 setae. Short, sparse pubescence present over the entire body, but difficult to detect against the lightly colored integument.

Color: predominantly testaceous yellow, with a dark brown head capsule. The femora, postpetiole, and lower half of the mesosomal pleurites are testaceous. Two slightly darker spots are present on the first gastral tergite posterolaterally.

**Gyne measurements & indices (*n* = 2):** SL = 0.559–0.565 (0.562); FRS = 0.267–0.268 (0.268); CW = 0.772–0.779 (0.776); CWb = 0.704–0.720 (0.712); PoOC = 0.271–0.272 (0.272); CL = 0.707–0.726 (0.717); EL = 0.234–0.239 (0.237); EW = 0.175–0.190 (0.183); MD = 0.135–0.142 (0.139); WL = 1.212–1.239 (1.226); SPST = 0.288–0.306 (0.297); MPST = 0.285–0.303 (0.294); PEL = 0.424–0.433 (0.429); NOL = 0.202–0.213 (0.208); NOH = 0.142–0.143 (0.143); PEH = 0.294–0.299 (0.297); PPL = 0.191–0.211 (0.201); PPH = 0.299–0.322 (0.311); PW = 0.748–0.838 (0.793); SBPA = 0.354–0.356 (0.355); SPTI = 0.333–0.367 (0.350); PEW = 0.199–0.207 (0.203); PNW = 0.221–0.234 (0.228); PPW = 0.487–0.535 (0.511); HFL = 0.646–0.654 (0.650); HFWmax = 0.135–0.141 (0.138); HFWmin = 0.052–0.058 (0.055); CS = 1.058–1.083 (1.070); ES = 0.327–0.329 (0.328); SI = 78–79 (79); OI = 30–31 (31); CI = 99–100 (99); WLI = 172–172 (172); SBI = 49–51 (50); PSI = 23–25 (24); PWI = 245–258 (252); PLI = 205–222 (214); NI = 141–150 (146); PNWI = 111–113 (112); NLI = 48–49 (48); FI = 243–260 (251).

**Gyne description:** In full-face view, head subquadrate, about as long as broad (CI 99–100). Mandibles densely striate but shining and armed with five teeth: the apical-most well developed, followed by a less developed preapical tooth and three equally developed smaller teeth. Anterior clypeal margin weakly emarginated medially. Antennal scapes moderately long: when fully retracted, just reaching the posterior margin of the head capsule (SI 78–79). Antennae 12-segmented; antennal club composed of three segments, with the apical-most segment as long as the preceding two in combination. Frontal carinae moderately long, extending past the antennal toruli by about three times the maximum width of the antennal scape. Compound eyes moderately protruding past the lateral margins of the head capsule. Lateral margin of head evenly convex, converging from below the compound eyes to the mandibular insertions. Posterior head margin flat, rounding evenly into the lateral margins.

In profile view, compound eyes ovular and large (OI 30–31), with 17 ommatidia in longest row. Mesoscutum rounded evenly anteriorly, covering the dorsal surface of the pronotum, and flat dorsally. Mesoscutellum slightly depressed below the level of the mesoscutum. Posterior margin of metanotum extending slightly past the posterior margin of the mesoscutum. Propodeal spiracle well developed, directed posterolaterally, and separated from the propodeal declivity by about three spiracle diameters. Propodeal spines stout and well developed, but short (PSI 23–25), about as two thirds as long as the propodeal declivity, tapering evenly from the base, directed posteriorly, straight, and blunt. Propodeal declivity straight and flat, forming a rounded ~90° angle with the base of the propodeal spines. Propodeal lobes rounded and very weakly developed. Metapleural gland bulla moderately large, extending from the metacoxal insertion two thirds of the way to the propodeal spiracle. Petiole long (PLI 205–222), without tubercles anterodorsally. Subpetiolar process in the form of a small, very acute tooth, which grades evenly into the ventral margin of the petiole posteriorly. Petiolar peduncle short: petiolar node covering most of the petiolar dorsum. Petiolar node erect: transition between peduncle and node evenly rounded, resulting in a very slightly concave anterior node face; anterior face forming a very sharp ~80° angle with the dorsal face, which is short; dorsal face rounding evenly into the posterior face, which forms a ~100° angle with the caudal cylinder. Postpetiole evenly rounded anterodorsally, bulging slightly before it transitions into the flattened dorsal face; ventral surface weakly lobed.

In dorsal view, mesoscutum covering pronotum anteriorly, but humeri visible laterally as rounded sclerites. Propodeal spines weakly diverging apically, their apices separated from each other by about one and a half times their length. Petiolar peduncle with spiracles not protruding past the lateral margins. Petiolar node widest anteriorly, and emarginated anterodorsally; lateral faces converge to the indistinct posterior face. Petiolar node slightly narrower than the peduncle, and evenly grading into the caudal cylinder, which is about the same width as the node. Postpetiole very broad (PWI 245–258), anteroposteriorly compressed, and campaniform, articulating with most of the anterior margin of the gaster, leaving small, angulate margins on each side exposed. Anterior margin of postpetiole weakly convex, with corners marked by rounded angles as it transitions to the lateral margins, which evenly diverge to the angulate posterior corners; posterior margin broadly concave. Metafemur weakly to moderately incrassate (FI 243–260).

Sculpture: median clypeal carina present, extending from the anterior margin nearly to frontal triangle, and flanked by weaker, indistinct carinae; lateral margins of median clypeal lobe with two carinae that are as strong as the medial carina. Lateral clypeal lobes with additional weaker carinae; ground sculpture smooth and shining. Antennal scapes smooth and shining. Cephalic dorsum mostly smooth and shining, with costulae flanking the frontal carinae, and a median carina extending from the frontal triangle nearly to the median ocellus; weak concentric costulae surrounding the antennal insertions. Lateral surfaces of head with weak areolate sculpture posterior to the compound eye, dense rugulose sculpture surrounding the compound eye, and rugae between the compound eye and the mandibular insertion. Ventral surface of head with weak rugulae. Pronotal neck areolate. Pronotum with weak areolate ground sculpture arranged into longitudinal rows and separated by superficial costulae. Anepisternum and katepisternum shining on their anterior halves, transitioning into weak costulae posteriorly. Propodeum with stronger costulae laterally. Propodeal declivity weakly areolate. Mesoscutum with costulae over weak areolate ground sculpture surrounding a smooth and shining central strip which extends over half of the sclerite from the anterior margin; smooth and shining patches laterally. Mesoscutellum smooth and shining medially, surrounded by weak costulae and areolae. Femora smooth and shining, with traces of weak areolate sculpture distally. Petiole with weak areolate sculpture laterally, and on the dorsoposterior surface of the node. Dorsal surface of the peduncle and the anterior face of the node shining through weak sculpture. Postpetiole smooth and shining anteriorly, areolate laterally and on the posterior half. Gaster smooth and shining, without faint traces of spectral iridescence. Surface of the first gastral sternite smooth and shining.

Setae: antennal scapes and funiculi with short, decumbent pilosity. Dorsum of the head, pronotum, waist segments, and gaster with moderately abundant, erect, blunt-tipped setae, the longest of which are about a third of the width of the compound eye. Short, sparse pubescence present over the entire body, but difficult to detect against the lightly colored integument.

Color: head capsule, anepisternum, propodeum, mesoscutellum, waist segments (except petiolar node), posterior half of the first gastral tergite and the following tergites dark brown. The mandibles, antennae, pronotum, katepisternum, mesoscutum, propodeal spines, petiolar node, and femora testaceous. Postpetiole and basal half of first gastral tergite yellow.

**Male measurements & indices (*n* = 1):** SL = 0.329; FRS = 0.143; CW = 0.563; CWb = 0.494; PoOC = 0.204; CL = 0.514; EL = 0.248; EW = 0.172; MD = 0.038; WL = 0.926; SPST = 0; MPST = 0.231; PEL = 0.322; NOL = 0.225; NOH = 0.055; PEH = 0.154; PPL = 0.180; PPH = 0.197; PW = 0.501; SBPA = n/a; SPTI = n/a; PEW = 0.143; PNW = 0.155; PPW = 0.301; HFL = 0.681; HFWmax = 0.058; HFWmin = 0.036; CS = 0.751; ES = 0.334; SI = 67; OI = 44; CI = 96; WLI = 187; SBI = n/a; PSI = n/a; PWI = 210; PLI = 179; NI = 409; PNWI = 108; NLI = 70; FI = 161.

**Male description:** In full-face view, head subovate, slightly longer than broad (CI 96). Mandibles very weakly striate, shining, and armed with five teeth: the apical-most well developed, followed by a smaller preapical tooth and three roughly equally developed smaller teeth. Anterior clypeal margin entire and weakly convex. Antennal scapes moderately long: when fully retracted, just reaching the posterior margin of the head capsule (SI 67). Antennae 13-segmented; antennal club composed of four segments, with the apical-most segment as long as the preceding two in combination. Frontal carinae short, extending past the antennal toruli by two times the maximum width of the antennal scape. Compound eyes strongly protruding past the lateral margins of the head capsule. Lateral margin of head convex, margin between the anterior margin of the compound eye and the mandibular insertions straight. Posterior head margin evenly convex, rounding evenly into the lateral margins.

In profile view, compound eyes ovular and large (OI 44), with 22 ommatidia in the longest row. Mesoscutum bulging anteriorly, covering the dorsal surface of the pronotum, flat dorsally, and rounded posteriorly. Mesoscutellum depressed slightly below the level of the mesoscutum; posterior margin even with the posterior margin of the metanotum, not overhanging it. Propodeal spiracle well developed, directed posterolaterally, and separated from the propodeal declivity by about three spiracle diameters. Propodeal spines absent, indicated by weak carinae on the dorsal and declivitous faces of the propodeum, which converge into a rounded angle. Propodeal lobes rounded and weakly developed. Metapleural gland bulla small, extending halfway between the insertion of the metacoxa and the propodeal spiracle. Petiole moderately long (PLI 179), without tubercles anterodorsally. Subpetiolar process absent. Petiolar peduncle short: comprising less than a quarter of the total length of the petiole. Petiolar node low and rounded, the convergence of the anterior and dorsal faces marked by a rounded angle. Postpetiole evenly rounded anterodorsally, flattened dorsally, and with a lobed, concave ventral surface.

In dorsal view, mesoscutum covering pronotum anteriorly, humeri barely visible laterally as slivers of rounded sclerites. Petiolar peduncle with spiracles slightly protruding past the lateral margins, the peduncle broadened where they arise. Petiolar node wider than the peduncle; petiole narrowing posterior to the node, before widening again to the caudal cylinder, which is about the same width as the node. Postpetiole moderately broad (PWI 210) and campaniform, articulating with most of the anterior margin of the gaster, leaving small angulate margins on each side exposed. Anterior margin of postpetiole convex, with the anterior corners evenly rounding into the lateral margins, which evenly diverge to the angulate posterior corners; posterior margin of postpetiole broadly concave. Metafemur not incrassate (FI 161).

Sculpture: median clypeal carina present but very weak and rounded, flanked by additional weak carinae over smooth and shining ground sculpture. Antennal scapes smooth and shining. Head areolate, with weak costulae flanking the frontal carinae and a central strip of weak sculpture extending from the frontal triangle to the median ocellus. Lateral surfaces of pronotum, anepisternum, katepisternum, and metasternum smooth and shining. Propodeum weakly areolate laterally, nearly smooth on the dorsum and declivity. Dorsally, mesoscutum weakly costulate along the Mayrian furrows, otherwise smooth and shining. Mesoscutellum costulate, but sculpture weaker medially. Femora smooth and shining, with traces weak areolate sculpture distally. Petiole with shallow areolate sculpture ventrally, the dorsal surface of the node smooth and shining. Dorsal surface of postpetiole shining, with traces of strigulae, especially on the posterior quarter. Gaster smooth and shining.

Setae: antennal scapes and funiculi with short, decumbent pilosity. Dorsum of the head, pronotum, waist segments, and gaster with moderately abundant, erect, blunt-tipped setae, the longest of which are about a quarter of the width of the compound eye. Short, sparse pubescence present over the entire body, but difficult to detect against the integument.

Color: predominantly dark brown, with mandibles, antennae, legs, and genitalia yellow. Pronotum, wing bases, coxae, and gastral segments (excluding the first) testaceous.

**Etymology:** Patronym, named in honor of E.O. Wilson, a naturalist and myrmecologist of unmeasurable influence, whose expedition to Hispaniola in 2003 recovered all of the known specimens of *Temnothorax wilsoni* sp. nov. used in this study.

**Comments:** *Temnothorax wilsoni* sp. nov. is known only from high elevations sites in Valle Nuevo National Park in the Dominican Republic with open *Pinus occidentalis* forests, where several nests were collected from under stones in the Cordillera Central. All nest collections contained only one dealate gyne, suggesting that this species may be monogynous. Other details of the biology of this species remain unknown but are probably similar to other members of the terricolous pan-Caribbean *pulchellus* group. This species displays variation in coloration of the gaster within nest series, ranging from having two testaceous spots posterolaterally on the first gastral tergite to being completely yellow. This species may form a species complex with *T. bahoruco* sp. nov., *T. balaclava* sp. nov., and *T. ciferrii*, all of which have only been collected from allopatric populations in mountains and low-lying dry forest from the southern parts of Hispaniola. It is morphologically quite similar to *T. bahoruco* sp. nov. and *T. balaclava* sp. nov., differing primarily in the conformation of the clypeus and in coloration.

### *rugosus* group overview

**Figure 150:**
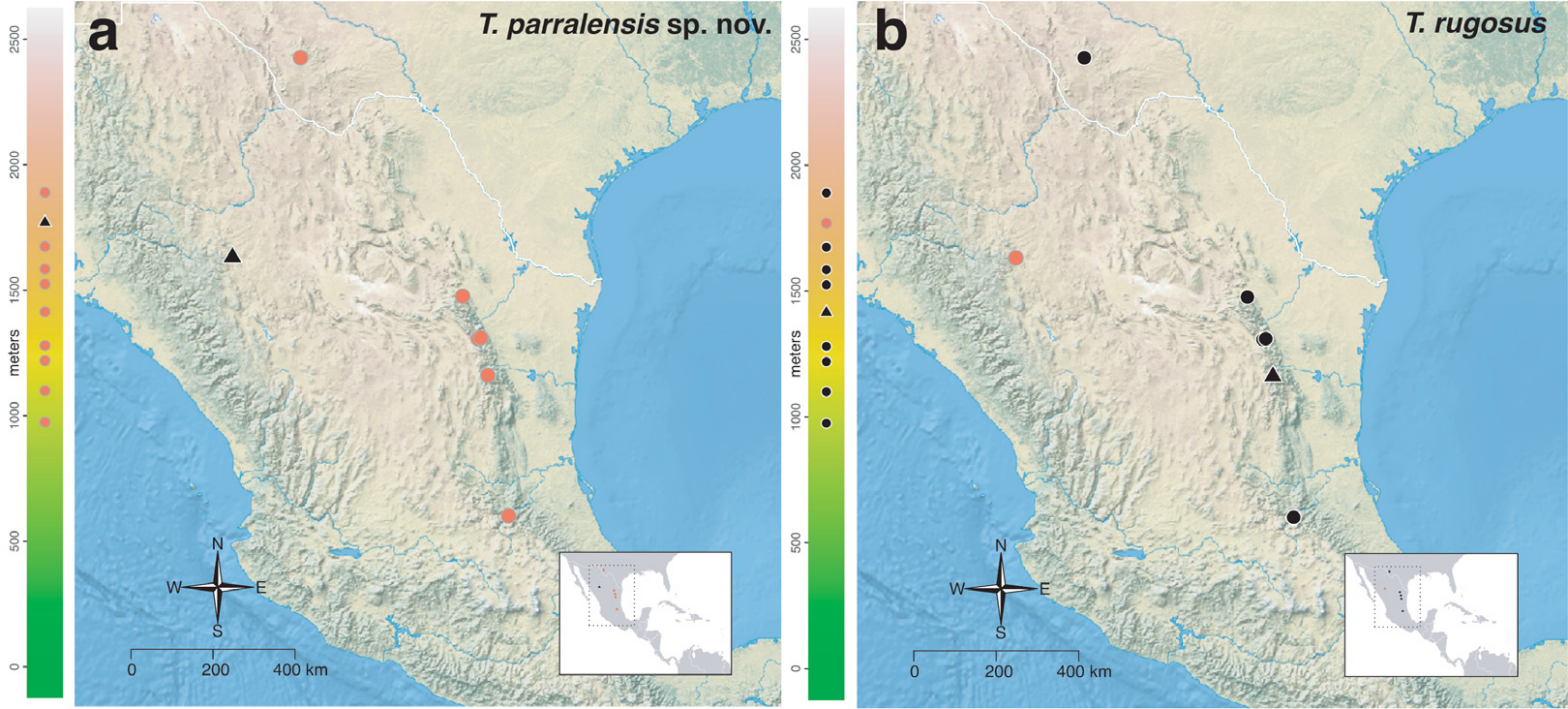
Geographical and elevational distribution of the *rugosus* group. (A) *Temnothorax parralensis* sp. nov. (B) *T. rugosus*. Colored scale to the left of each map represents elevation in meters. Points in black represent the species named in each subfigure, while points in red represent other members of the species group. Type localities are represented by triangles, non-type localities are represented by circles. Bounding box in inset map shows location of main map.

This group is composed of two species, *Temnothorax parralensis* sp. nov. and *T. rugosus*, which are found at mid-to-high elevations in the southern United States to central Mexico (Fig. 150). Members of this group have been collected from dead branches on live trees, mostly *Quercus* spp. The species of the *rugosus* group, with their arboreal nests, large size, coarse sculpturing, and somewhat incrassate femora, are very similar in habitus to the *annexus* group, but differ from them by their relatively short propodeal spines and shape of their petiolar nodes (cuneiform to truncate).

***Temnothorax parralensis* sp. nov.**

**Figure 151:**
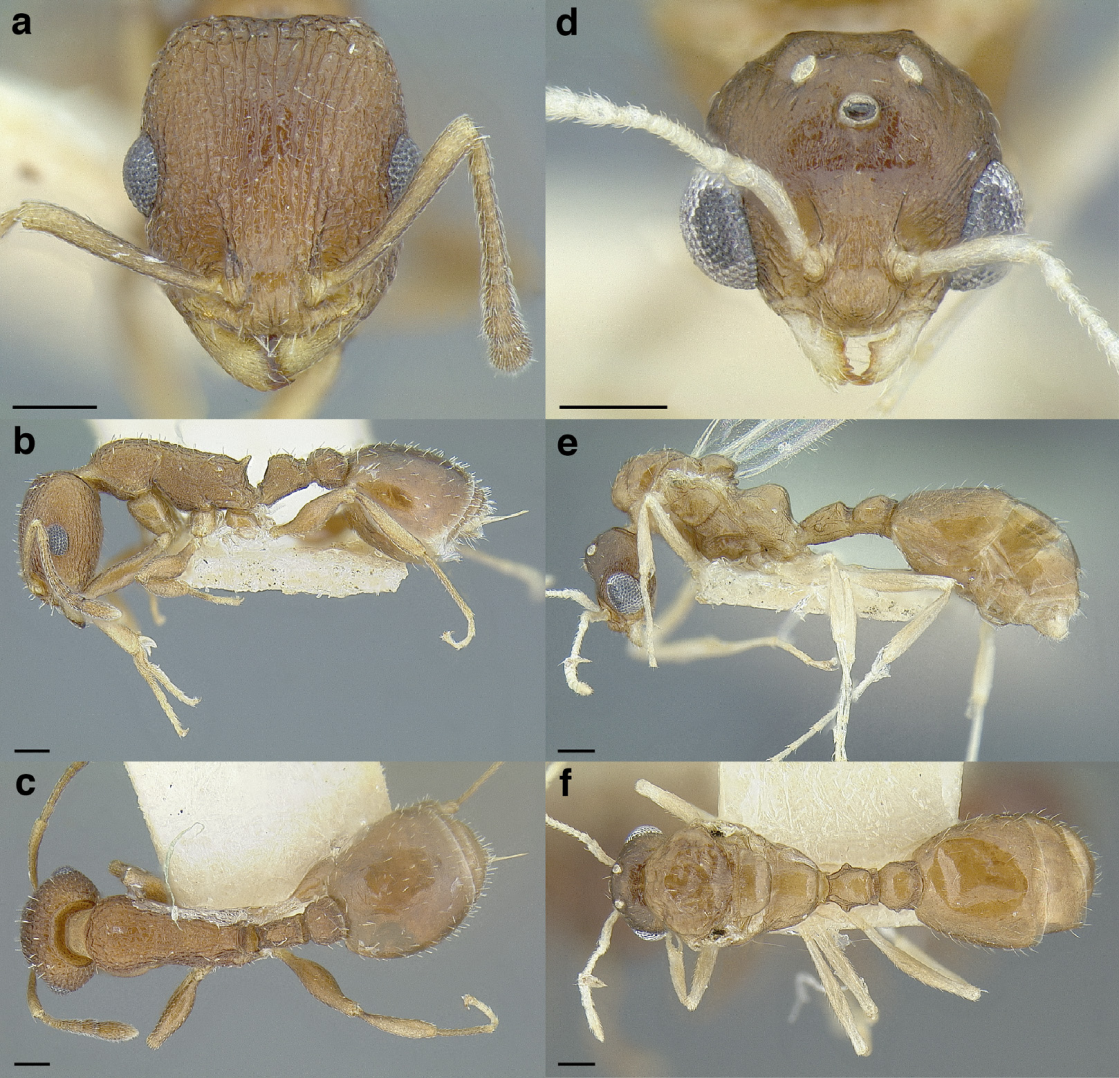
*Temnothorax parralensis* sp. nov. (A–C) Holotype worker (LACMENT323355). (A) Full-face view. (B) Profile view. (C) Dorsal view. (D–F) Paratype male (LACMENT323356). (D) Full-face view. (E) Profile view. (F) Dorsal view. Scale bars 0.2 mm.

Distribution: Fig. 150A; worker & male: Fig. 151.

**Type material examined:** *Holotype:* MEXICO: Chihuahua: 55 km south of Parral, 1,770 m, 2 May 1953, W.S. Creighton, in *Quercus grisea* (LACMENT323355, bottom specimen on pin) [LACM].

*Paratype workers and males:* same pin as holotype, 1 worker & 1 male (middle and top specimens on pin) [LACM]; same collection data as holotype, 2 males, 1 worker (LACMENT323351) [LACM] 1 male, 3 workers (LACMENT323352) [MCZC] 1 male, 2 workers (LACMENT323353) [LACM] 2 males, 1 worker (LACMENT323354) [UCDC] 1 male, 3 workers (LACMENT323356) [LACM] 1 male, 2 workers (LACMENT323357) [USNM].

**Geographic range:** Mid elevations in northern Mexico (Chihuahua) (Fig. 150A).

**Worker diagnosis:** *Temnothorax parralensis* sp. nov. can be separated from all other species in the *salvini* clade by the following character combination: anterior clypeal margin emarginate; metanotal groove absent; propodeum not depressed, and bearing setae dorsally; petiolar node flat dorsally; hind femora moderately incrassate; in dorsal view, postpetiole narrow, less than 1.5 times the width of the petiole; dorsum of head, mesosoma, waist segments and gaster with erect, short, blunt-tipped setae; legs without erect setae; integument predominantly testaceous.

**Similar species:** *Temnothorax rugosus, T. tricarinatus* Emery, species of the *annexus* and *silvestrii* groups. *Temnothorax parralensis* sp. nov. may be separated from *T. tricarinatus* by the moderately incrassate femora (not apparent in smaller workers), and the weakly sculptured first gastral tergite (which is smooth and shining in *T. tricarinatus*). Many of the preceding characters, in combination, are shared with members of the *annexus* species group, which *T. parralensis* sp nov. is closely related to. *Temnothorax parralensis* sp. nov. can be separated from the species of the *annexus* group by the relatively narrow postpetiole (PWI < 150) and the propodeal spines, which are short (PSI < 22) and dorsally directed; all species in the *annexus* group have broader postpetioles (PWI > 150) and longer (PSI ≥ 22), posterodorsally directed propodeal spines. The areolate first gastral tergite may suggest an affiliation with the *silvestrii* group, but these species never have incrassated femora. The closest relative of *T. parralensis* sp. nov. is *T. rugosus*, which always has a cuneiform to subcuneiform petiolar node.

**Worker measurements & indices (*n* = 5)**: SL = 0.494–0.555 (0.518); FRS = 0.228–0.258 (0.238); CW = 0.712–0.771 (0.737); CWb = 0.637–0.692 (0.659); PoOC = 0.265–0.295 (0.284); CL = 0.760–0.837 (0.788); EL = 0.198–0.227 (0.211); EW = 0.147–0.166 (0.154); MD = 0.191–0.217 (0.206); WL = 0.939–1.063 (1.004); SPST = 0.161–0.219 (0.193); MPST = 0.268–0.306 (0.290); PEL = 0.296–0.343 (0.318); NOL = 0.147–0.226 (0.188); NOH = 0.116–0.140 (0.131); PEH = 0.242–0.277 (0.257); PPL = 0.195–0.212 (0.204); PPH = 0.220–0.262 (0.235); PW = 0.415–0.470 (0.441); SBPA = 0.147–0.177 (0.164); SPTI = 0.178–0.213 (0.194); PEW = 0.173–0.196 (0.184); PNW = 0.080–0.103 (0.090); PPW = 0.245–0.284 (0.262); HFL = 0.556–0.624 (0.585); HFWmax = 0.148–0.176 (0.163); HFWmin = 0.052–0.066 (0.055); CS = 1.017–1.111 (1.053); ES = 0.272–0.304 (0.288); SI = 77–82 (79); OI = 27–28 (27); CI = 83–84 (84); WLI = 146–157 (152); SBI = 23–27 (25); PSI = 17–21 (19); PWI = 140–145 (143); PLI = 141–176 (156); NI = 127–169 (143); PNWI = 45–55 (49); NLI = 49–66 (59); FI = 265–332 (295).

**Worker description:** In full-face view, head subquadrate, longer than broad (CI 83–84). Mandibles densely, finely striate but shining and armed with five teeth: the apical-most well developed and acute, followed by a less developed preapical tooth and three equally developed smaller teeth. Anterior clypeal margin emarginated medially. Antennal scapes short: when fully retracted, failing to reach the posterior margin of the head capsule by about two times the maximum width of the antennal scape (SI 77–82). Antennae 12-segmented; antennal club of composed of three segments, with the apical-most segment about one and a half times as long as the preceding two in combination. Frontal carinae moderately long, extending past the antennal toruli by about three times the maximum width of the antennal scape. Compound eyes moderately protruding past the lateral margins of the head capsule. Lateral margin of head weakly convex, forming a continuous arc from the mandibular insertions to the posterior margin of the head. Posterior head margin concave medially, rounding evenly into the lateral margins.

In profile view, compound eyes ovular and moderately large (OI 27–28), with 14 ommatidia in longest row. Pronotal declivity distinct: dorsal margin of anterior face of pronotum marked by a weak carina and change in sculpture; neck and anterior face of pronotum forming a ~120° angle. Mesosoma very weakly sinuate: promesonotum forming an even convexity from where it joins the pronotal declivity; propodeum weakly depressed below the level of the promesonotum and descending to the base of the propodeal spines on the posterior quarter. Promesonotal suture extending from the posterior margin of the procoxal insertion to the mesothoracic spiracle, which is well developed. Metanotal groove visible as a disruption of the sculpture laterally from where it arises between the mid- and hind coxae to where it ends in the poorly developed metathoracic spiracle, which is nearly indistinguishable against the ground sculpture. Propodeal spiracle moderately well developed, directed posterolaterally, and separated from the propodeal declivity by about four spiracle diameters. Propodeal spines weakly developed and short (PSI 17–21), about half as long as the propodeal declivity, flared at the base, stout, triangular, and blunt. Propodeal declivity straight, forming a rounded ~150° angle with the base of the propodeal spines. Propodeal lobes rounded and weakly developed. Metapleural gland bulla small, extending from the metacoxal insertion a third of the way to the propodeal spiracle. Petiole moderately long (PLI 141–176), with tubercles anterodorsally. Subpetiolar process in the form of a blunt, triangular tooth which grades evenly into the ventral petiole margin posteriorly. Petiolar peduncle short: comprising about a quarter of the total petiole length. Petiolar node truncate dorsally; anterior face forming a ~100° angle with the dorsal face, which is flat; dorsal face meeting the posterior face at a ~120° angle; very short posterior face forms a ~120° angle with the caudal cylinder. Postpetiole evenly convex anterodorsally; weakly lobed ventrally.

In dorsal view, humeri developed: angulate anterolaterally and slightly wider than the rest of the mesosoma; mesothoracic spiracles protruding past the lateral margins of the mesosoma, visible as slight angles where the pronotum meets the mesonotum. Promesonotal suture represented by a weak sulcus and disruption in the ground sculpture. Metanotal groove absent: mesonotum and propodeum completely fused and lateral margins converging evenly to the bases of the propodeal spines. Propodeal spines broadly approximated basally and diverging apically, their apices separated from each other by about twice their length, the negative space between them “U” shaped. Petiolar peduncle with spiracles weakly protruding past the lateral margins. Petiolar node narrowed apically and flat dorsally, narrower than the peduncle and the caudal cylinder. Postpetiole narrow (PWI 140–145) and campaniform. Anterior margin of the postpetiole convex and rounds evenly into the lateral margins; lateral margins parallel to each other; posterior corners rounded; posterior margin flat. Metafemur moderately to strongly incrassate (FI 265–332).

Sculpture: median clypeal carina present, extending posteriorly to the frontal triangle, and flanked on either side by three weaker carinae. Lateral clypeal lobes with additional, weaker carinae; ground sculpture shining through weak areolae. Antennal scapes areolate. Cephalic dorsum areolate-rugulose, with costae over the ground sculpture, which become rugose on the posterior sixth of the head and between the frontal area and lateral margins of the head; concentric rugulae surrounding the antennal insertions. Lateral surfaces of head areolate-rugulose, with coarse rugae over the ground sculpture. Ventral surface of head smooth and shining medially, but otherwise weakly areolate with weak costulae. Pronotal neck areolate. Lateral surfaces of mesosoma weakly areolate, with coarse costae over the ground sculpture. Propodeal declivity areolate-strigulate. Dorsal surface of mesosoma with coarse rugae; propodeum with areolate ground sculpture. Femora finely, weakly areolate. Petiole uniformly areolate; a weak carina present laterally, extending longitudinally from the petiolar spiracle to the caudal cylinder; rugae on lateral faces of the node. Postpetiole uniformly areolate, with rugose sculpture on the dorsal and lateral surfaces. First gastral tergite shining through weak areolate sculpture; without spectral iridescence. First gastral sternite smooth and shining, without spectral iridescence.

Setae: antennal scapes and funiculi with short, subdecumbent pilosity. Dorsum of the head, pronotum, waist segments, and gaster with moderately abundant, erect, blunt-tipped setae, the longest of which are about half the width of the compound eye. The head bears ~50, mesosoma ~26, petiole 8, postpetiole ~32, and first gastral tergite ~72 setae. Short, sparse pubescence present over the entire body, but difficult to detect against the densely sculptured integument.

Color: predominantly testaceous, with tibiae, tarsi, and sting testaceous yellow.

**Male measurements & indices (*n* = 2):** SL = 0.188–0.241 (0.215); FRS = 0.128–0.160 (0.144); CW = 0.559–0.650 (0.605); CWb = 0.447–0.571 (0.509); PoOC = 0.189–0.241 (0.215); CL = 0.467–0.576 (0.522); EL = 0.226–0.268 (0.247); EW = 0.199–0.211 (0.205); MD = 0.040–0.041 (0.041); WL = 0.841–0.974 (0.908); SPST = n/a; MPST = 0.237–0.300 (0.269); PEL = 0.234–0.288 (0.261); NOL = 0.143–0.189 (0.166); NOH = 0.065–0.099 (0.082); PEH = 0.183–0.208 (0.196); PPL = 0.160–0.205 (0.183); PPH = 0.201–0.228 (0.215); PW = 0.492–0.632 (0.562); SBPA = n/a; SPTI = n/a; PEW = 0.174–0.202 (0.188); PNW = 0.164–0.188 (0.176); PPW = 0.241–0.280 (0.261); HFL = 0.611–0.758 (0.685); HFWmax = 0.079–0.101 (0.090); HFWmin = 0.033–0.039 (0.036); CS = 0.681–0.859 (0.770); ES = 0.326–0.374 (0.350); SI = 42; OI = 43–48 (46); CI = 96–99 (97); WLI = 171–188 (179); SBI = n/a; PSI = n/a; PWI = 139; PLI = 140–146 (143); NI = 191–220 (205); PNWI = 93–94 (94); NLI = 61–66 (63); FI = 239–259 (249).

**Male description:** In full-face view, head subovate, about as broad as long (CI 96–99). Mandibles very weakly striate, shining, and armed with four teeth: the apical-most well developed, followed by three smaller preapical teeth. Anterior clypeal margin entire and weakly convex. Antennal scapes short: when fully retracted, failing to reach the posterior margin of the head capsule by about half the length of the antennal scape (SI 42). Antennae 13-segmented; antennal club composed of four segments, with the apical-most segment longer than the preceding two in combination. Frontal carinae moderately long, extending past the antennal toruli by about two times the maximum width of the antennal scape. Compound eyes strongly protruding past the lateral margins of the head capsule. margin of head convex behind the compound eyes; margins between the anterior margin of the compound eye and the mandibular insertions converging. Posterior head margin convex, rounding evenly into the lateral margins.

In profile view, compound eyes ovular and large (OI 43–48), with 24 ommatidia in the longest row. Mesoscutum bulging anteriorly, covering the dorsal surface of the pronotum, convex dorsally. Mesoscutellum on the same plane as the mesoscutum, convex dorsally. Posterior margin of metanotum extending slightly beyond the posterior margin of the mesoscutellum. Propodeum strongly depressed, flat dorsally, and meeting the propodeal declivity at a broadly rounded ~90° angle. Propodeal spiracle moderately well developed, directed posterolaterally, and separated from the propodeal declivity by about three spiracle diameters. Propodeal spines absent. Propodeal lobes rounded and weakly developed. Metapleural gland bulla small, extending a quarter of the way between the insertion of the metacoxa and the propodeal spiracle. Petiole short (PLI 140–146), with tubercles anterodorsally. Subpetiolar process absent; ventral margin of petiole bulging slightly. Petiolar peduncle short: comprising about a third of the total petiole length. Petiolar node low and rounded; the convergence of the anterior and dorsal faces marked by a ~120° angle. Postpetiole flat anteriorly, meeting the dorsal face at ~120° angle; weakly concave ventrally.

In dorsal view, mesoscutum covering pronotum. Petiolar peduncle with spiracles strongly protruding past the lateral margins. Petiolar node about as wide as the peduncle, and wider than the caudal cylinder. Postpetiole narrow (PWI 139) and campaniform. Anterior margin of postpetiole convex, with the anterior corners evenly rounding into the lateral margins; lateral margins parallel to each other; posterior margin of postpetiole flat. Metafemur weakly to moderately incrassate (FI 239–259).

Sculpture: median clypeal lobe with multiple concentric carinae anteriorly. Antennal scapes weakly areolate. Dorsum of head weakly areolate, with weak, fine costulae between the frontal carinae. Lateral surface of head areolate, with weak, fine concentric costulae surrounding the compound eye. Ventral surface of head weakly areolate. Pronotal neck very weakly areolate. Anterior face of pronotum weakly areolate. Lateral surfaces of pronotum and mesopleurae predominantly smooth and shining, with weak, indistinct sculpture near the borders of the sclerites. Metapleuron and lateral face of propodeum areolate. Propodeal declivity weakly areolate, with weak, fine costulae. Dorsally, mesoscutum and mesoscutellum smooth and shining, with weak areolate sculpture along the Mayrian furrows. Propodeum weakly areolate. Femora weakly areolate. Petiole weakly areolate with coarse rugae laterally, but smooth and shining dorsally. Dorsal surface of postpetiole shining, with traces of weak areolate sculpture laterally and on the posterior quarter. First gastral tergite weakly, indistinctly sculptured, but mostly smooth and shining, without spectral iridescence. First gastral sternite smooth and shining, without spectral iridescence.

Setae: antennal scapes and funiculi with short, subdecumbent pilosity. Dorsum of the head, pronotum, waist segments, and gaster with moderately abundant, erect, blunt-tipped setae, the longest of which are about a quarter of the width of the compound eye. Short, sparse pubescence present over the entire body, but difficult to detect against the densely sculptured integument.

Color: predominantly testaceous, with antennae, legs, and genitalia light yellow. Head capsule slightly darker than the rest of the body.

**Etymology**: Geographical, from ‘Parral’, the type collection locality + ‘-ensis’ (= native to).

**Comments:** *Temnothorax parralensis* sp. nov., like its closest relatives *T. rugosus* and the *annexus* species group, appears to prefer nesting in hollow oak branches at mid elevations.

***Temnothorax rugosus* (Mackay, 2000)**

**Figure 152:**
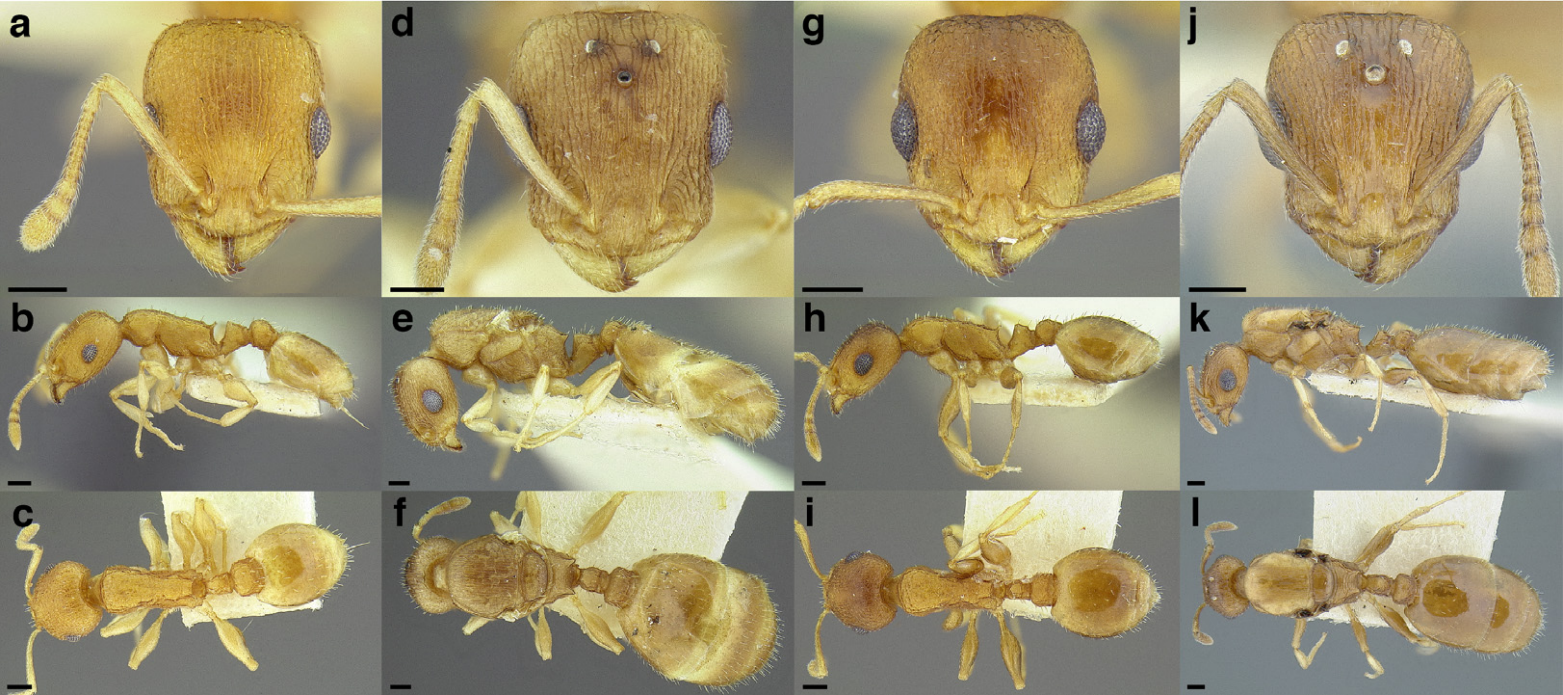
*Temnothorax rugosus*. (A–C) Worker (LACMENT323296). (A) Full-face view. (B) Profile view. (C) Dorsal view. (D–F) Gyne (LACMENT323337). (D) Full-face view. (E) Profile view. (F) Dorsal view. Morphological variant, Texas, U.S.A. (G–I) worker (LACMENT323260). (G) Full-face view. (H) Profile view. (I) Dorsal view. (J–L) Gyne (LACMENT323259). (J) Full-face view. (K) Profile view. (L) Dorsal view. Scale bars 0.2 mm.

Distribution: Fig. 150B; worker, gyne & variability: Fig. 152.

*Leptothorax rugosus* Mackay, 2000: 399, figs. 18, 160. Worker. Nuevo León, Mexico.

*Temnothorax rugosus* (Mackay): Bolton, 2003: 272. First combination in *Temnothorax.*,

**Type material examined:** *Paratype worker:* MEXICO: Nuevo León: El Salto (Zaragosa), 1,415 m, 10 June 1988, W. Mackay#WM11012-10 (images of UTEPENT05451 examined on antweb.org) [UTEP].

**Non-type material examined:** MEXICO: Hidalgo: 11 km west of Jacala, 1,585 m, 14 January 1952, W.S. Creighton, in *Quercus polymorpha*, 3 workers, 1 dealate gyne (LACMENT323267) [LACM] 4 workers (LACMENT323268) [LACM] 4 workers (LACMENT323269) [LACM] 4 workers (LACMENT323270) [LACM] 4 workers (LACMENT323272) [LACM] 3 workers (LACMENT323333) [LACM]; same data as previous, except: in oak twig, 3 workers (LACMENT323280) [LACM] 2 workers (LACMENT323324) [LACM] 3 workers, 1 dealate gyne (LACMENT323326) [LACM] 3 workers, 1 dealate gyne (LACMENT323327) [LACM] 3 workers, 1 dealate gyne (LACMENT323328) [LACM] 3 workers, 1 dealate gyne (LACMENT323271) [LACM]; same data as previous, except: in oak limb, 4 workers, 1 dealate gyne (LACMENT323329) [LACM] 4 workers, 1 dealate gyne (LACMENT323330) [LACM]. Nuevo León: Monterrey, Chipinque Mesa, 1,280 m, 21 January 1952, W.S. Creighton, in *Quercus clivicola*, 3 workers (LACMENT323302) [LACM] 4 workers (LACMENT323303) [LACM] 4 workers (LACMENT323306) [LACM] 4 workers (LACMENT323307) [LACM] 4 workers (LACMENT323314) [LACM] 4 workers (LACMENT323325) [LACM]; same data as previous, except: in *Quercus canbyi*, 4 workers (LACMENT323308) [LACM] 4 workers (LACMENT323309) [LACM] 4 workers (LACMENT323310) [LACM] 4 workers (LACMENT323311) [LACM]; 22 January 1952, in *Quercus clivicola*, 3 workers, 1 dealate gyne (LACMENT323286) [LACM] 4 workers (LACMENT323287) [LACM] 4 workers (LACMENT323288) [LACM] 4 workers (LACMENT323289) [LACM] 4 workers (LACMENT323290) [LACM] 4 workers (LACMENT323291) [LACM]; same data as previous, except: 1,280 m, 18 February 1952, in *Quercus canbyi*, 4 workers (LACMENT323297) [LACM] 4 workers (LACMENT323298) [LACM] 4 workers (LACMENT323299) [LACM] 4 workers (LACMENT323300) [LACM] 4 workers (LACMENT323301) [LACM] 4 workers (LACMENT323304) [LACM]; same data as previous, except: 975 m, 19 February 1952, in *Quercus* sp., 3 workers, 1 alate gyne (LACMENT323337) [LACM] 4 workers (LACMENT323334) [LACM] 4 workers (LACMENT323335) [LACM] 4 workers (LACMENT323336) [LACM] 4 workers (LACMENT323338) [LACM]; same data as previous, except: in *Quercus clivicola*, 3 workers, 1 dealate gyne (LACMENT323273) [LACM] 4 workers (LACMENT323274) [LACM] 3 workers, 1 dealate gyne (LACMENT323276) [LACM] 4 workers (LACMENT323279) [LACM]; same data as previous, except: in *Quercus canbyi*, 4 workers (LACMENT323275) [LACM] 4 workers (LACMENT323277) [LACM] 3 workers (LACMENT323278) [LACM]; same data as previous, except: 1,100 m, in *Cercis reniformis*, 4 workers (LACMENT323292) [LACM] 4 workers (LACMENT323293) [LACM] 4 workers (LACMENT323294) [LACM] 4 workers (LACMENT323295) [LACM] 4 workers (LACMENT323296) [LACM] 4 workers (LACMENT323361) [LACM]; same data as previous, except: 1,220 m, in *Quercus canbyi*, 3 workers (LACMENT323281) [LACM] 4 workers (LACMENT323282) [LACM] 4 workers (LACMENT323283) [LACM] 4 workers (LACMENT323284) [LACM] 4 workers (LACMENT323285) [LACM] 3 workers, 1 dealate gyne (LACMENT323322) [LACM]; same data as previous, except: 1,280 m, 21 April 1952, in *Quercus clivicola*, (LACMENT323305) [LACM] 4 workers (LACMENT323318) [LACM] 4 workers (LACMENT323319) [LACM] 4 workers (LACMENT323320) [LACM]; same data as previous, except: in *Quercus canbyi*, 4 workers (LACMENT323312) [LACM] 4 workers (LACMENT323313) [LACM] 4 workers (LACMENT323315) [LACM] 4 workers (LACMENT323316) [LACM] 4 workers (LACMENT323317) [LACM] 4 workers (LACMENT323321) [LACM] 4 workers (LACMENT323323) [LACM]; 10 km west of Iturbide, 1,890 m, 16 February 1952, W.S. Creighton, in *Quercus clivicola*, 4 workers (LACMENT323358) [LACM] 4 workers (LACMENT323360) [LACM] 4 workers (LACMENT323362) [LACM] 4 workers (LACMENT323363) [LACM] 4 workers (LACMENT323364) [LACM] 4 workers (LACMENT323365) [LACM] 4 workers (LACMENT323366) [LACM] 3 workers, 1 dealate gyne (LACMENT323367) [LACM]; 3 km west of Iturbide, 1,675 m, 16 February 1952, W.S. Creighton, in hollow stem of *Rhus* sp., 4 workers (LACMENT323359) [LACM].

U.S.A.: Texas: Jeff Davis County: Davis Mountains State Park, 1,525 m, 11 May 1953, W.S. Creighton, in *Quercus grisea*, 3 workers (LACMENT323257) [LACM]; same data as previous, except: in deciduous oak, 2 workers (LACMENT323258) [LACM]; same data as previous, except: 16 May 1953, W.S. Creighton, in *Quercus grisea*, 3 workers (LACMENT323252) [LACM] 2 workers (LACMENT323253) [LACM] 4 workers (LACMENT323254) [LACM] 3 workers (LACMENT323255) [LACM] 3 workers (LACMENT323256) [LACM] 3 workers, 1 dealate gyne (LACMENT323259) [LACM] 4 workers (LACMENT323260) [LACM] 4 workers (LACMENT323261) [LACM] 4 workers (LACMENT323262) [LACM] 4 workers (LACMENT323263) [LACM]; same data as previous, except: 20 May 1952, W.S. Creighton, in *Quercus grisea*, 4 workers (LACMENT323264) [LACM] 4 workers (LACMENT323265) [LACM] 4 workers (LACMENT323266) [LACM] 3 workers (LACMENT323331) [LACM] 2 workers (LACMENT323332) [LACM] 3 workers (LACMENT323339) [LACM].

**Geographic range:** Mid-to-high elevations of southwestern Texas to Hidalgo State, Mexico (Fig. 150B).

**Worker diagnosis:** *Temnothorax rugosus* can be separated from all other species in the *salvini* clade by the following character combination: anterior clypeal margin emarginate; metanotal groove absent; propodeum not depressed; hind femora weakly to strongly incrassate; petiole cuneiform to subcuneiform; dorsum of head, mesosoma, waist segments and gaster with erect, short, blunt-tipped setae; legs without erect setae; integument testaceous yellow, with legs lighter yellow.

**Similar species:** *Temnothorax acuminatus* sp. nov., *T. nitens, T. tricarinatus*, species of the *annexus* and *silvestrii* groups. *Temnothorax rugosus* may be separated from *T. nitens* and *T. tricarinatus* by the moderately incrassate femora (not apparent in smaller workers), and the weakly sculptured first gastral tergite (which is smooth and shining in *T. nitens* and *T. tricarinatus*). Many of the preceding characters, in combination, are shared with members of the *annexus* species group, which *T. rugosus* is closely related to. *Temnothorax rugosus* can be separated from the species of the *annexus* group by the cuneiform petiolar node, which is laterally compressed in dorsal view; all species in the *annexus* group have quadrate to subquadrate petiolar nodes. Finally, the areolate first gastral tergite is similar to members of the *silvestrii* group, but members of that group never have incrassate femora, and always have subquadrate petiolar nodes.

**Worker measurements & indices (*n* = 27)**: SL = 0.465–0.602 (0.527); FRS = 0.207–0.279 (0.228); CW = 0.610–0.800 (0.700); CWb = 0.563–0.733 (0.642); PoOC = 0.265–0.325 (0.288); CL = 0.701–0.874 (0.773); EL = 0.157–0.255 (0.198); EW = 0.116–0.166 (0.142); MD = 0.173–0.217 (0.197); WL = 0.824–1.095 (0.948); SPST = 0.141–0.219 (0.182); MPST = 0.203–0.334 (0.281); PEL = 0.242–0.362 (0.291); NOL = 0.147–0.230 (0.178); NOH = 0.105–0.180 (0.133); PEH = 0.226–0.303 (0.260); PPL = 0.170–0.289 (0.211); PPH = 0.210–0.306 (0.244); PW = 0.368–0.502 (0.428); SBPA = 0.135–0.213 (0.160); SPTI = 0.148–0.241 (0.187); PEW = 0.149–0.217 (0.178); PNW = 0.066–0.117 (0.090); PPW = 0.245–0.393 (0.290); HFL = 0.484–0.680 (0.578); HFWmax = 0.148–0.209 (0.177); HFWmin = 0.049–0.081 (0.059); CS = 0.914–1.170 (1.029); ES = 0.219–0.327 (0.269); SI = 77–89 (82); OI = 24–29 (26); CI = 80–87 (83); WLI = 139–157 (148); SBI = 22–29 (25); PSI = 17–22 (19); PWI = 140–181 (163); PLI = 113–176 (139); NI = 105–173 (135); PNWI = 38–67 (50); NLI = 49–75 (61); FI = 225–354 (301).

**Worker description:** In full-face view, head subquadrate, longer than broad (CI 80–87). Mandibles densely, finely striate but shining and armed with five teeth: the apical-most well developed and acute, followed by a less developed preapical tooth and three equally developed smaller teeth. Anterior clypeal margin emarginated medially. Antennal scapes short: when fully retracted, failing to reach the posterior margin of the head capsule by about two times the maximum width of the antennal scape (SI 77–89). Antennae 12-segmented; antennal club of composed of three segments, with the apical-most segment about one and a half times as long as the preceding two in combination. Frontal carinae short, extending past the antennal toruli by about two times the maximum width of the antennal scape. Compound eyes moderately protruding past the lateral margins of the head capsule. margin of head weakly convex, forming a continuous arc from the mandibular insertions to the posterior margin of the head. Posterior head margin concave medially, rounding evenly into the lateral margins.

In profile view, compound eyes ovular and moderately large (OI 24–29), with 13 ommatidia in longest row. Pronotal declivity distinct: dorsal margin of anterior face of pronotum marked by a weak carina and change in sculpture; neck and anterior face of pronotum forming a ~120° angle. Mesosoma very weakly sinuate: promesonotum forming an even convexity from where it joins the pronotal declivity; propodeum weakly depressed below the level of the promesonotum and descending sharply to the base of the propodeal spines on the posterior quarter. Promesonotal suture extending from the posterior margin of the procoxal insertion to the mesothoracic spiracle, which is well developed. Metanotal groove visible as a disruption of the sculpture laterally from where it arises between the mid- and hind coxae to where it ends in the poorly developed metathoracic spiracle, which is nearly indistinguishable against the ground sculpture. Propodeal spiracle moderately well developed, directed posterolaterally, and separated from the propodeal declivity by about four spiracle diameters. Propodeal spines weakly developed and short (PSI 17–22), about a third as long as the propodeal declivity, flared at the base, stout, triangular, and acute. Propodeal declivity straight, forming a rounded ~120° angle with the base of the propodeal spines. Propodeal lobes rounded and weakly developed. Metapleural gland bulla small, extending from the metacoxal insertion a third of the way to the propodeal spiracle. Petiole moderately long (PLI 113–176), with tubercles anterodorsally. Subpetiolar process in the form of a blunt, triangular tooth which grades evenly into the ventral petiole margin posteriorly. Petiolar peduncle short: comprising about a quarter of the total petiole length. Petiolar node truncate but dorsum is extremely angled, so that in many specimens it appears cuneiform: anterior face forming a sharp ~90° angle with the dorsal face, which is flat; dorsal face meeting the posterior face at a sharp ~120° angle; very short posterior face forms a ~110° angle with the caudal cylinder. Postpetiole evenly convex anterodorsally; weakly lobed ventrally.

In dorsal view, humeri developed: angulate anterolaterally and slightly wider than the rest of the mesosoma; mesothoracic spiracles protruding past the lateral margins of the mesosoma, visible as slight angles where the pronotum meets the mesonotum. Promesonotal suture represented by a weak sulcus and disruption in the ground sculpture. Metanotal groove absent: mesonotum and propodeum completely fused and lateral margins converging evenly to the bases of the propodeal spines. Propodeal spines broadly approximated basally and diverging apically, their apices separated from each other by about twice their length, the negative space between them “U” shaped. Petiolar peduncle with spiracles weakly protruding past the lateral margins. Petiolar node, when viewed at posterodorsal angle, cuneiform: narrowed apically and flat dorsally; node narrower than the peduncle and the caudal cylinder. Postpetiole narrow (PWI ~ 163) and campaniform. Anterior margin of the postpetiole convex and rounds evenly into the lateral margins; lateral margins parallel to each other; posterior corners rounded; posterior margin flat. Metafemur moderately to strongly incrassate (FI 225–354).

Sculpture: median clypeal carina present, extending posteriorly to the frontal triangle, and flanked on either side by three weaker carinae. Lateral clypeal lobes with additional, weaker carinae; ground sculpture shining through weak areolae. Antennal scapes areolate. Cephalic dorsum areolate, with costa over the ground sculpture, which become rugose on the posterior sixth of the head; concentric rugulae surrounding the antennal insertions. Lateral surfaces of head areolate, with coarse rugae over the ground sculpture. Ventral surface of head smooth and shining medially, but otherwise weakly areolate with weak costulae. Pronotal neck areolate. Lateral surfaces of mesosoma weakly areolate, with coarse, costae over the ground sculpture; region between the propodeal spiracle and propodeal spines areolate, without rugae. Propodeal declivity areolate, with fine strigulae. Dorsal surface of mesosoma weakly areolate, with coarse costae that are rugose on the pronotum and the propodeum. Femora finely, weakly areolate. Petiole uniformly areolate; a weak carina present laterally, extending longitudinally from the petiolar spiracle to the caudal cylinder; rugae on lateral faces of the node. Postpetiole uniformly areolate, with rugose sculpture on the dorsal and lateral surfaces. First gastral tergite shining through weak areolate sculpture; without spectral iridescence. First gastral sternite smooth and shining, without spectral iridescence.

Setae: antennal scapes and funiculi with short, subdecumbent pilosity. Dorsum of the head, pronotum, waist segments, and gaster with moderately abundant, erect, blunt-tipped setae, the longest of which are about half the width of the compound eye. The head bears ~50, mesosoma ~26, petiole 8, postpetiole ~32, and first gastral tergite ~72 setae. Short, sparse pubescence present over the entire body, but difficult to detect against the densely sculptured integument.

Color: predominantly testaceous yellow, with legs lighter yellow.

**Gyne measurements & indices (*n* = 5):** SL = 0.518–0.600 (0.556); FRS = 0.264–0.309 (0.291); CW = 0.783–0.831 (0.802); CWb = 0.726–0.782 (0.746); PoOC = 0.304–0.319 (0.312); CL = 0.824–0.880 (0.851); EL = 0.233–0.277 (0.253); EW = 0.171–0.207 (0.191); MD = 0.169–0.196 (0.183); WL = 1.324–1.477 (1.401); SPST = 0.202–0.233 (0.223); MPST = 0.322–0.383 (0.354); PEL = 0.312–0.382 (0.355); NOL = 0.204–0.228 (0.213); NOH = 0.145–0.168 (0.155); PEH = 0.300–0.351 (0.319); PPL = 0.215–0.238 (0.224); PPH = 0.314–0.376 (0.345); PW = 0.783–0.838 (0.802); SBPA = 0.300–0.391 (0.338); SPTI = 0.268–0.340 (0.310); PEW = 0.222–0.269 (0.237); PNW = 0.120–0.185 (0.146); PPW = 0.378–0.465 (0.413); HFL = 0.644–0.718 (0.667); HFWmax = 0.166–0.189 (0.178); HFWmin = 0.053–0.069 (0.062); CS = 1.138–1.216 (1.171); ES = 0.319–0.381 (0.349); SI = 71–77 (75); OI = 28–33 (30); CI = 85–90 (88); WLI = 182–200 (188); SBI = 41–52 (45); PSI = 15–17 (16); PWI = 165–186 (174); PLI = 145–176 (158); NI = 121–146 (138); PNWI = 52–79 (62); NLI = 53–67 (60); FI = 242–313 (289).

**Gyne description:** In full-face view, head subquadrate, longer than broad (CI 85–90). Mandibles densely striate but shining and armed with five teeth: the apical-most well developed, followed by a less developed preapical tooth and three equally developed smaller teeth. Anterior clypeal margin emarginated medially. Antennal scapes short: when fully retracted, failing to reach the posterior margin of the head capsule by about two times the maximum width of the scape (SI 71–77). Antennae 12-segmented; antennal club composed of three segments, with the apical-most segment about one and a half times as long as the preceding two in combination. Frontal carinae moderately long, extending past the antennal toruli by about two and a half times the maximum width of the antennal scape. Compound eyes strongly protruding past the lateral margins of the head capsule. Lateral margins of head convex behind the compound eyes, but parallel to each other between the compound eyes and the mandibular insertions. Posterior head margin weakly concave medially, rounding evenly into the lateral margins.

In profile view, compound eyes ovular and large (OI 28–33), with 17 ommatidia in longest row. Mesoscutum rounded evenly anteriorly, covering the dorsal surface of the pronotum, and flat dorsally. Mesoscutellum on the same plane as the mesoscutum and flat dorsally. Posterior margin of metanotum extending slightly past the posterior margin of the mesoscutum. Propodeal spiracle well developed, directed posterolaterally, and separated from the propodeal declivity by about four spiracle diameters. Propodeal spines stout, weakly developed, and short (PSI 15–17), about a third as long as the propodeal declivity, flared at the base, triangular, and blunt. Propodeal declivity straight and flat, forming a rounded ~120° angle with the base of the propodeal spines. Propodeal lobes rounded. Metapleural gland bulla small, extending from the metacoxal insertion halfway to the propodeal spiracle. Petiole moderately long (PLI 145–176), with tubercles anterodorsally. Subpetiolar process in the form of a blunt, triangular tooth, which grades evenly into the ventral margin of the petiole posteriorly. Petiolar peduncle short: comprising about a quarter of the total petiole length. Petiolar node truncate but dorsum is extremely angled, so that in many specimens it appears cuneiform: anterior face forming a sharp ~90° angle with the dorsal face, which is flat; dorsal face meeting the posterior face at a sharp ~120° angle; very short posterior face forms a ~110° angle with the caudal cylinder. Postpetiole evenly convex anterodorsally, flat posterodorsally; weakly lobed ventrally.

In dorsal view, mesoscutum covering pronotum anteriorly, but humeri visible laterally as rounded sclerites. Propodeal spines weakly diverging apically, their apices separated from each other by about three times their length. Petiolar peduncle with spiracles weakly protruding past the lateral margins. Petiolar node, when viewed posterodorsally, tapering apically so the apex is about half the width of the base; dorsal margin emarginate medially. Postpetiole narrow (PWI 165–186), anteroposteriorly compressed, and subquadrate. Anterior margin of postpetiole weakly convex but emarginated medially; anterior corners marked by rounded ~100° angles as it transitions to the lateral margins; lateral margins parallel to each other; posterior corners rounded; posterior margin weakly concave. Metafemur weakly to strongly incrassate (FI 242–313).

Sculpture: median clypeal carina present, extending from the anterior margin nearly to frontal triangle, and flanked on either side by three equally strong carinae. Lateral clypeal lobes with additional weaker carinae; ground sculpture weakly areolate. Antennal scapes finely areolate. Cephalic dorsum areolate, with costae over the ground sculpture, which become rugose on the posterior sixth of the head; concentric rugulae surrounding the antennal insertions. Lateral surfaces of head areolate, with coarse rugae over the ground sculpture. Ventral surface of head smooth and shining medially, but otherwise weakly areolate with weak costulae. Pronotal neck areolate. Anterior face of pronotum weakly areolate. Lateral surfaces of the mesosoma sculptured similarly to the dorsum of the head. Propodeal declivity areolate-strigulate. Mesoscutum and mesoscutellum with fine costulae over finely areolate ground sculpture. Metanotum finely areolate. Propodeum areolate, with coarse costae. Femora shining through weak areolate sculpture. Petiole uniformly areolate; a weak carina present laterally, extending longitudinally from the petiolar spiracle to the caudal cylinder; coarse rugae on lateral faces of the node. Postpetiole uniformly areolate, with weak rugose sculpture on the dorsal and lateral faces. First gastral tergite shining through weak areolate sculpture; without spectral iridescence. First gastral sternite smooth and shining.

Setae: antennal scapes and funiculi with short, subdecumbent pilosity. Dorsum of the head, pronotum, waist segments, and gaster with moderately abundant, erect, blunt-tipped setae, the longest of which are about a third of the width of the compound eye. Short, sparse pubescence present over the entire body, but difficult to detect against the densely sculptured integument.

Color: predominantly testaceous yellow, with legs lighter yellow. Wing bases dark brown.

**Etymology**: Morphological, from the Latin ‘rugosus’ (= wrinkled), in reference to the rugose head and mesosoma (Mackay, 2000).

**Comments:** From Mackay (2000): “The type series was collected in a trap baited with Vienna sausage, placed in oak trees, about 2-meter height. The habitat was a very steep, south-facing slope of a mountain, covered with oak trees. The specimens were collected within 20 meters distant from one another.” After inspecting the collections of the LACM, I found that W.S. Creighton collected *Temnothorax rugosus* in a number of localities spanning northern Mexico and southwestern Texas, in addition to the type locality near Monterrey. Like members of the *annexus* group, which it is closely affiliated with, *T. rugosus* nests arboreally in the branches a number of different *Quercus* spp. (including *Q. canbyi, Q. clivicola, Q. grisea*, and *Q. polymorpha*), as well as *Cercis reniformis* and *Rhus* spp. at mid elevations. *Temnothorax rugosus* is also recorded as being collected from *Tillandsia carlos-hankii* in oak-pine forest near Ixtepeji, Oaxaca (Franco Méndez, 2008). The femora of *Temnothorax rugosus*, as noted above, tend to be incrassate, but the degree of swollenness appears to scale with the size of the worker, with smaller workers having less incrassate femora. *Temnothorax rugosus* shows some variability in petiolar node shape, eye size, and sculpture among populations (Figs. 152G–152L). In the petiolar node, there appears to be a complete range of variation between completely cuneiform, with no differentiation between the dorsal and posterior declivitous face, to subcuneiform, with a very short but distinct posterior face and a deeply sloping dorsal face.

### *salvini* group overview

**Figure 153:**
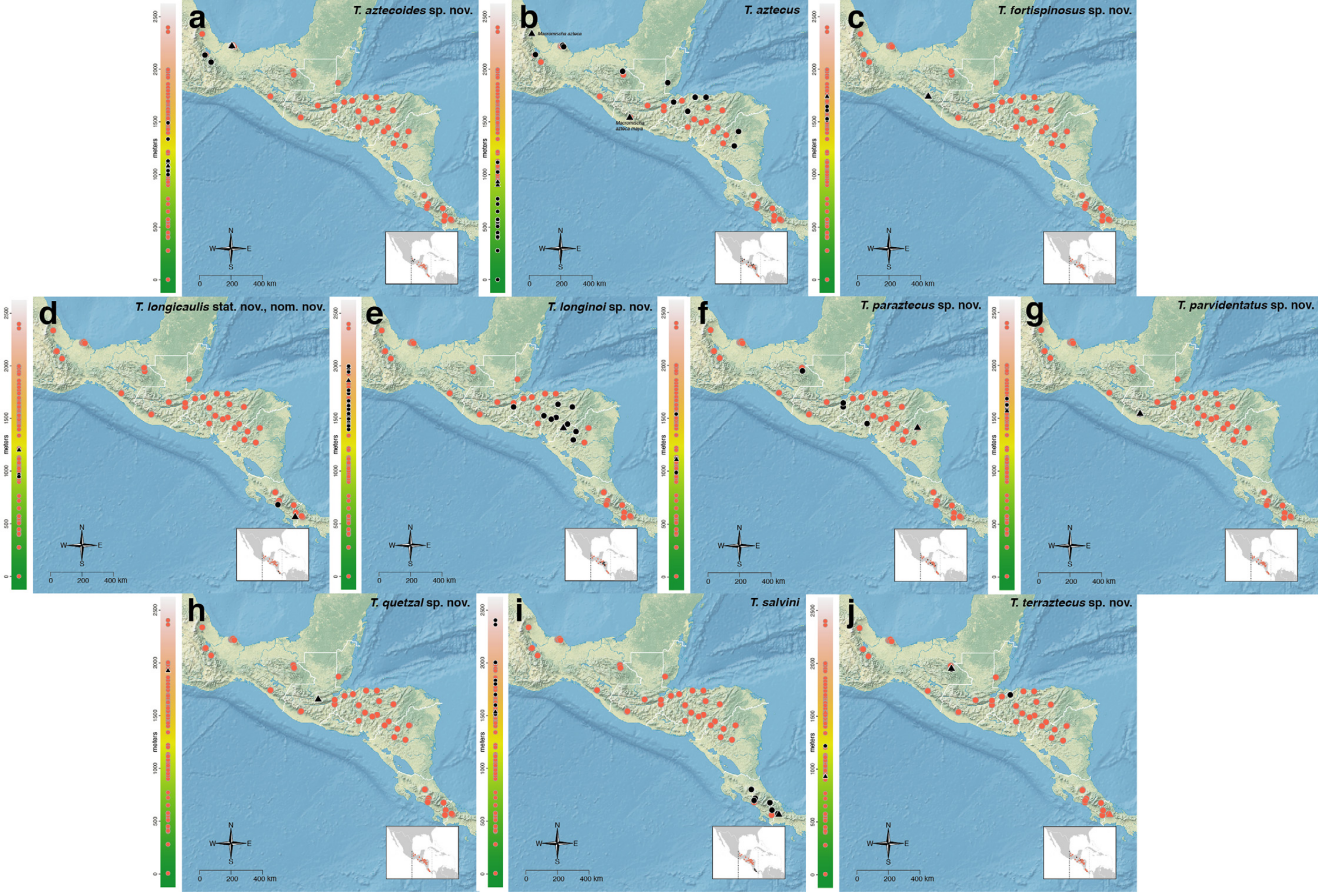
Geographical and elevational distribution of the *salvini* group. (A) *Temnothorax aztecoides* sp. nov. (B) *T. aztecus* (C) *T. fortispinosus* sp. nov. (D) *T. longicaulis* stat. nov., nom. nov. (E) *T. longinoi* sp. nov. (F) *T. paraztecus* sp. nov. (G) *T. parvidentatus* sp. nov. (H) *T. quetzal* sp. nov. (I) *T. salvini* (J) *T. terraztecus* sp. nov. Colored scale to the left of each map represents elevation in meters. Points in black represent the species named in each subfigure, while points in red represent other members of the species group. Type localities are represented by triangles; if present, types of synonyms are named; non-type localities are represented by circles. Bounding box in inset map shows location of main map.

With ten species (eight described as new here), the *salvini* group is the second largest in the *salvini* clade. Up until a recent species delimitation study (Prebus, 2021), this group was thought to contain two species, *T. aztecus* and *T. salvini*, but upon including specimens from across its considerable geographic range and morphological variability, this group was found to contain an additional seven species. Following publication of this study, an additional species was discovered that had been overlooked in collections (*T. terraztecus* sp. nov.). The members of the *salvini* group inhabit all elevations throughout their range in southern Mexico and Central America (Fig. 153), but individual species appear to have an affinity for nearly discrete elevational ranges when their geographic ranges overlap with other members of the *salvini* group. Prebus, 2021 illuminated some of the biogeographic history of the salvini group as well: it was inferred to have arisen in the northern part of its current range in habitats associated with contemporary mid-to-high elevations in the Miocene, approximately 13 Ma. Subsequently, the *salvini* group expanded into the southern Central American Cordilleras at the Miocene-Pliocene transition, coinciding with mountain building in that region 8–5 Ma, where today it is represented by *T. salvini* and *T. longicaulis* stat. nov., nom. nov. Additionally, the *salvini* group expanded into low elevation habitats during the same transition period, with the widespread *T. aztecus* being the only member of the group inhabiting these habitats today. The nesting habits of the species of the *salvini* group, where they are known, are almost exclusively arboreal, nesting in vines, under epiphytes, or in dead vegetation suspended in the canopy. The one exception may be *T. terraztecus* sp. nov., which has only been collected from leaf litter extractions from a couple of localities. While the species within the *salvini* group can be difficult to tease apart morphologically, as a group they are quite distinctive, with long, tapering setae on all surfaces of the body, flat to slightly sinuate mesosomata in profile, and strongly sculptured head and mesosomata.

***Temnothorax aztecoides* sp. nov.**

**Figure 154:**
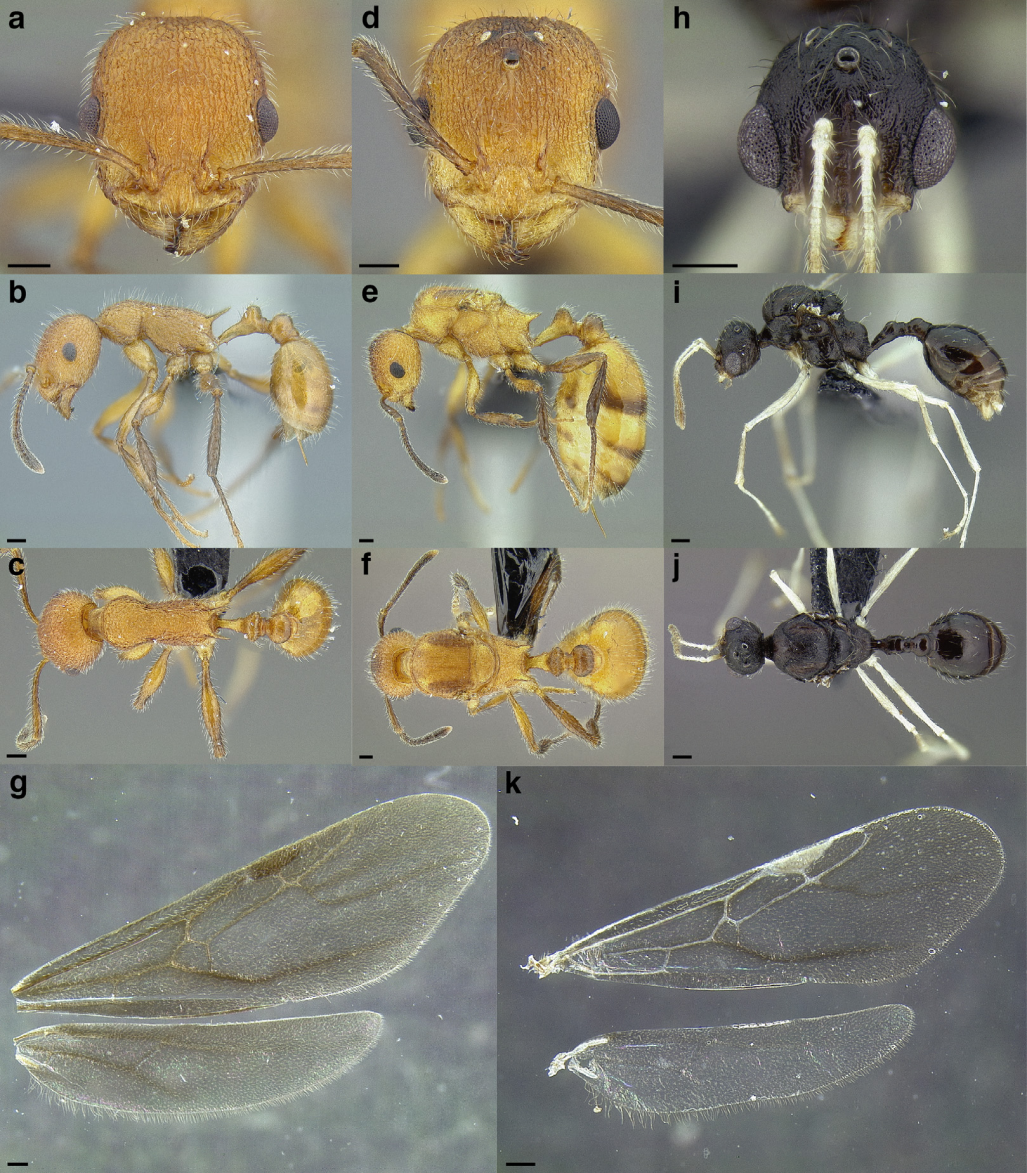
*Temnothorax aztecoides* sp. nov. (A–C) Holotype worker (CASENT0758791). (A) Full-face view. (B) Profile view. (C) Dorsal view. (D–F) Paratype gyne (CASENT0758789). (D) Full-face view. (E) Profile view. (F) Dorsal view. (G) Wings. (H–K) Paratype male (CASENT0758790). (H) Full-face view. (I) Profile view. (J) Dorsal view. (K) Wings. Scale bars 0.2 mm.

Distribution: Fig. 153A; worker, gyne & male: Fig. 154.

*Temnothorax salvini* grp 3 Prebus, 2021: 12. In phylogeny.

**Type material examined:** *Holotype worker:* MEXICO: Veracruz: Ruiz Cortinez, 13 km NE San Andrés Tuxtla, 18.53235°N 95.14477°W ± 20 m, 1,080 m ± 5 m, 2 June 2016, M.M. Prebus#MMP02634, montane rainforest, ex hollow twig hanging from tree (CASENT0758791) [CASC].

*Paratype workers, males, and gynes:* same data as holotype, 1 worker (CASENT0758792) [CASC] 1 dealate gyne (CASENT0758789) [CASC] 1 male (CASENT0758790) [CASC] 1 alate gyne (CASENT0869605) [CASC] 1 alate gyne (CASENT0869606) [USNM] 1 alate gyne (CASENT0869607) [MCZC] 1 alate gyne (CASENT0869608) [LACM] 1 alate gyne (CASENT0869609) [UCDC] 1 male (CASENT0869610) [USNM] 1 male (CASENT0869611) [UCDC] 1 male (CASENT0869612) [MCZC] 1 male (CASENT0869613) [LACM] 1 worker (CASENT0869614) [USNM] 1 worker (CASENT0869615) [UNAM] 1 worker (CASENT0869616) [MCZC] 1 worker (CASENT0869617) [LACM] 1 worker (CASENT0869618) [UCDC] 1 worker (CASENT0869619) [UVGC] 1 worker (CASENT0869620) [MCZC] 1 worker (CASENT0869621) [AMNH] 1 worker (CASENT0869622) [UNAM] 1 worker (CASENT0869623) [USNM] 1 worker (CASENT0869624) [LACM] 1 worker (CASENT0869625) [AMNH] 1 worker (CASENT0869626) [FSCA].

**Non-type material examined:** MEXICO: Oaxaca: 13 km S Valle Nacional, 17.65947°N 96.33438°W ± 20 m, 1,340 m ± 5 m, 15 August 2014, M.M. Prebus#01593, wet broadleaf forest, nest in dead hollow vine, 1 dealate gyne (CASENT0733317) [UCDC] 1 worker (CASENT0733318) [UCDC] 1 worker (CASENT0733319) [UCDC]; Veracruz: 10 km NNE San Andrés Tuxtla, 18.53514°N 95.19022°W ± 8 m, 1,030 m, 5 June 2016, P.S. Ward#PSW17623, montane rainforest, ex dead twig of *Bursera*, ca. 2.5 m above ground, 1w (CASENT0642924) [UCDC]; same data as previous, except: 18.53613°N 95.19097°W ± 13 m, 1,015 m ± 5 m, 5 June 2016, M.M. Prebus#MMP02692, montane rainforest, ex dead vine hanging from tree, 1 alate gyne, 59 workers (CASENT0757622) [MMPC]; 12 km NE San Andrés Tuxtla, 18.53438°N 95.1497°W ± 250 m, 1,130 m ± 50 m, 4 June 2016, ADMAC#Go-F-02-1-01, montane wet forest, beating vegetation, 1 worker (CASENT0640387) [JTLC] 1 worker (CASENT0640716) [UCDC]; same data as previous, except: 18.55793°N 95.19018°W ± 7 m, 1,495 m ± 5 m, 6 June 2016, M.M. Prebus#MMP02713, montane rainforest, ex dead stick, 1 alate gyne, 30 workers (CASENT0757637) [MMPC]; Ruiz Cortínez, 13 km NE San Andrés Tuxtla, 18.53443°N 95.14966°W ± 20 m, 1,115 m ± 5 m, 2 June 2016, M.M. Prebus#MMP02638, montane rainforest, ex hollow twig on live tree, 1 dealate gyne (CASENT0757144) [MCZC]; same data as previous, except: 18.53245°N 95.14465°W ± 10 m, 1,080 m ± 5 m, 2 June 2016, M.M. Prebus#MMP02633, montane rainforest, stray on low vegetation, 1 worker (CASENT0757137) [USNM]; same data as previous, except: 18.53450°N 95.14944°W ± 15 m, 1,115 m ± 5 m, 2 June 2016, M.M. Prebus#MMP02640, montane rainforest, ex dead vine, 1 alate gyne, 14 workers (CASENT0757571) [MMPC]; same data as previous, except: 18.53434°N 95.13850°W ± 100 m, 1,040 m ± 5 m, , 23June 2016, M.M. Prebus#MMP02647, montane rainforest, ex dead vine, 29 workers (CASENT0757579) [MMPC]; same data as previous, except: M.M. Prebus#MMP02648, montane rainforest edge, ex dead vine, 37 workers (CASENT0757580) [MMPC]; same data as previous, except: M.M. Prebus#MMP02649, montane rainforest edge, ex dead vine, 1 worker (CASENT0758684) [UCDC] 1 worker (CASENT0758785) [UNAM]; same data as previous, except: M.M. Prebus#MMP02659, montane rainforest edge, ex dead stick hanging from vine, 13 workers (CASENT0757591) [MMPC]; same data as previous, except: M.M. Prebus#MMP02660, montane rainforest edge, ex dead stick hanging from vine, 11 males, 60 workers (CASENT0757592) [MMPC]; same data as previous, except: 18.53469°N 95.13954°W ± 10 m, 1,065 m ± 5 m, 4 June 2016, M.M. Prebus#MMP02677, montane rainforest, ex dead stick hanging from tree, 1 alate gyne, 77 workers (CASENT0757608) [MMPC]; same data as previous, except: 18.53434098°N 95.13842097°W ± 9 m, 1,070 m ± 5 m, 5 June 2016, M.M. Prebus#MMP02684, montane rainforest edge, ex dead branch, 1 alate gyne, 2 males, 36 workers (CASENT0757613) [MMPC]; same data as previous, except: Prebus#MMP02685, montane rainforest edge, stray worker, 1 worker (CASENT0757615) [MMPC]; same data as previous, except: 18.53613°N 95.19097°W ± 13 m, 1,015 m ± 5 m, 5 June 2016, M.M. Prebus#MMP02692, montane rainforest, ex dead vine hanging from tree, 1 alate gyne, 59 workers (CASENT0757622) [MMPC]; same data as previous, except: 18.53°N 95.15°W, 1,131 m, 5 June 2016, K.W. Gray#KWG160605-01, montane rainforest, in log, 6 workers (CASENT0868850-CASENT0868855) [KWGC].

**Geographic range:** Mid elevations in southern Mexico (Los Tuxtlas and the Southern Sierra Madre) (Fig. 153A).

**Worker diagnosis:** *Temnothorax aztecoides* sp. nov. can be separated from all other species in the *salvini* clade by the following character combination: larger species: WL > 1.25 mm; dorsum of mesosoma weakly sinuate; metanotal groove not impressed; propodeum not strongly depressed below the level of the promesonotum; propodeal spines present and longer than the propodeal declivity; subpetiolar tooth acutely spiniform and shorter than the setae that arises from the peduncle directly above; petiolar node strongly squamiform: in dorsal view, petiolar node greater than or equal to 1.6 times as broad as caudal cylinder; dorsum of head with predominantly rugose over areolate sculpture; dorsum of petiolar node and postpetiole sculptured; setae on head, mesosoma, legs, waist segments and gaster erect to suberect, long, abundant and tapering; integument predominantly yellow.

**Similar species:** Fellow members of the *salvini* group. *Temnothorax aztecoides* sp. nov. can be separated from other members of the *salvini* group by the strongly squamiform petiolar node (petiolar node less than or equal to 1.5 times as broad as the caudal cylinder in *T. longinoi* sp. nov., *T. quetzal* sp. nov., *T. fortispinosus* sp. nov., *T. parvidentatus* sp. nov., and *T. salvini*), yellow integument (*T. longinoi* sp. nov., *T. longicaulis* stat. nov., nom. nov*., T. quetzal* sp. nov., *T. fortispinosus* sp. nov., *T. parvidentatus* sp. nov., and *T. salvini* are variously colored, but never uniformly yellow), sculptured dorsum of the petiolar node and postpetiole (smooth in *T. aztecus* and *T. paraztecus* sp. nov.), relatively small subpetiolar tooth (longer than the setae that arises directly above it in *T. aztecus* and *T. paraztecus* sp. nov.), and long propodeal spines (shorter than the propodeal declivity in *T. paraztecus* sp. nov.).

**Worker measurements & indices (*n* = 7):** SL = 0.849–0.905 (0.880); FRS = 0.272–0.305 (0.292); CW = 0.889–0.973 (0.937); CWb = 0.792–0.852 (0.822); PoOC = 0.345–0.395 (0.373); CL = 0.912–0.980 (0.955); EL = 0.208–0.240 (0.224); EW = 0.153–0.171 (0.164); MD = 0.220–0.268 (0.238); WL = 1.263–1.388 (1.336); SPST = 0.421–0.479 (0.458); MPST = 0.338–0.394 (0.368); PEL = 0.490–0.580 (0.524); NOL = 0.252–0.295 (0.284); NOH = 0.149–0.175 (0.163); PEH = 0.268–0.309 (0.292); PPL = 0.231–0.282 (0.251); PW = 0.592–0.624 (0.610); SBPA = 0.270–0.307 (0.291); SPTI = 0.326–0.363 (0.347); PEW = 0.186–0.213 (0.200); PNW = 0.308–0.353 (0.331); PPW = 0.316–0.358 (0.333); HFL = 0.995–1.065 (1.031); HFWmax = 0.189–0.224 (0.207); HFWmin = 0.065–0.077 (0.072); CS = 1.249–1.342 (1.300); ES = 0.294–0.324 (0.306); SI = 105–109 (107); OI = 23–24 (24); CI = 85–88 (86); WLI = 158–168 (162); SBI = 34–37 (35); PSI = 33–36 (34); PWI = 160–179 (167); PLI = 191–223 (209); NI = 150–195 (175); PNWI = 157–176 (165); NLI = 49–59 (54); FI = 271–307 (288).

**Worker description:** In full-face view, head subquadrate, longer than broad (CI 85–88). Mandibles finely striate but shining and armed with five teeth: the apical-most well developed and acute, followed by a less developed preapical tooth and three equally developed smaller teeth. Anterior clypeal margin weakly convex medially. Antennal scapes very long: when fully retracted, surpassing the posterior margin of the head capsule by about three times the maximum width of the antennal scape (SI 105–09). Antennae 12-segmented; antennal club of composed of three segments, with the apical-most segment slightly longer than the preceding two in combination. Frontal carinae moderately long, extending past the antennal toruli by about two times the maximum width of the antennal scape. Compound eyes strongly protruding past the lateral margins of the head capsule. Lateral margin of head convex, forming a continuous arc from the mandibular insertions to the posterior margin of the head. Posterior head margin flat but rounding evenly into the lateral margins.

In profile view, compound eyes ovular and moderately large (OI 23–24), with 17 ommatidia in longest row. Pronotal declivity indistinct, neck and anterior face of pronotum forming a ~120° angle. Anterior face of pronotum evenly rounding into dorsal face. Dorsum of mesosoma flat from where it joins the anterior face of the pronotum to the propodeal spines. Promesonotal suture extending from the posterior margin of the procoxal insertion only to the mesothoracic spiracle, which is moderately well developed. Metanotal groove visible as a disruption of the sculpture laterally from where it arises between the mid- and hind coxae to where it ends in the poorly developed metathoracic spiracle, which is nearly indistinguishable against the ground sculpture. Propodeal spiracle weakly developed, directed posterolaterally, and separated from the propodeal declivity by about five spiracle diameters. Propodeal spines well developed and very long (PSI 33–36), longer than the propodeal declivity, tapering evenly from the base, acute, and straight but slightly upturned at the apices. Propodeal declivity weakly concave, forming a rounded ~120° angle with the base of the propodeal spines. Propodeal lobes rounded and weakly developed. Metapleural gland bulla moderately large, extending from the metacoxal insertion two thirds of the way to the propodeal spiracle. Petiole long (PLI 191–223), without tubercles anterodorsally. Subpetiolar process in the form of a small, spiniform, acute, anteriorly curved tooth; ventral margin of petiole weakly concave posterior to it but bulging slightly medially. Petiolar peduncle very long: comprising about two thirds of the total petiole length. Petiolar node rounded-squamiform: transition between peduncle and node marked by a rounded angle of ~120°; anterior face rounding evenly into the posterior face, the dorsal margin evenly convex; posterior face forms a ~100° angle with the caudal cylinder. Postpetiole evenly rounded anterodorsally, bulging before flattening posterodorsally; concave ventrally.

In dorsal view, humeri developed and distinct: evenly rounded and slightly wider than the rest of the mesosoma; mesothoracic spiracles protruding past the lateral margins of the mesosoma, visible as slight angles where the pronotum meets the mesonotum. Promesonotal suture visible as a slight disruption in the ground sculpture. Metanotal groove very faintly impressed, visible as a slight disruption in the ground sculpture. Propodeal spines broadly approximated basally and nearly parallel apically, their apices separated from each other by slightly less than their length, the negative space between them “U” shaped. Petiolar peduncle with spiracles slightly protruding past the lateral margins, but not noticeably constricted anterior to them. Petiolar node, when viewed posterodorsally, trapezoidal: much broader apically than basally; apical margin flat; node broader than the peduncle and the caudal cylinder. Postpetiole narrow (PWI 160–179) and campaniform. Anterior margin of the postpetiole convex, evenly rounding into the lateral margins, which bulge slightly anteriorly, are weakly constricted medially, then diverge slightly again to the angulate posterior corners; posterior margin flat. Metafemur moderately to strongly incrassate (FI 271–307).

Sculpture: median clypeal carina present, extending posteriorly to the frontal triangle, and flanked on either side by multiple slightly weaker carinae. Lateral clypeal lobes with additional, weaker carinae; ground sculpture weakly areolate. Antennal scapes areolate-costulate. Cephalic dorsum densely rugose over areolate sculpture; fine concentric costulae surrounding the antennal insertions. Lateral surfaces of head sculptured similarly to the dorsum, but rugae forming whorls around the compound eyes. Ventral surface of head smooth and shining over the gular region, otherwise with weak costulae over weak areolate ground sculpture. Pronotal neck areolate. Lateral surfaces of the mesosoma sculptured similarly to the head, but with weak areolate sculpture between the propodeal spiracle and the base of the propodeal spine. Propodeal declivity weakly rugulose. Dorsal surface of mesosoma sculptured similarly to the cephalic dorsum and the lateral mesosoma surface. Femora shining through weak areolate sculpture. Petiole finely areolate on all surfaces but the ventral surface of the peduncle, which is smooth and shining; areolae becoming weaker on the node. Postpetiole shining through weak areolate sculpture. First gastral tergite shining but anterior quarter often weakly, indistinctly sculptured, with very weak spectral iridescence. First gastral sternite smooth and shining, without spectral iridescence.

Setae: antennal scapes and funiculi with long, suberect pilosity. Dorsum of the head, pronotum, waist segments, and gaster with abundant, erect, tapering, flexuous setae, the longest of which are about one and a half times the length of the compound eye and are directed toward the midline of the body. The head bears >80, mesosoma >80, petiole ~32, postpetiole ~40, and first gastral tergite >80 setae. Pubescence present over the entire body, which is nearly as long as the setae.

Color: predominantly testaceous yellow; antennae, mandibles, tibiae, and first tarsomere testaceous.

**Gyne measurements & indices (*n* = 3):** SL = 0.850–0.923 (0.880); FRS = 0.313–0.354 (0.328); CW = 1.034–1.081 (1.050); CWb = 0.909–0.963 (0.932); PoOC = 0.371–0.408 (0.390); CL = 0.976–1.045 (0.999); EL = 0.247–0.274 (0.258); EW = 0.184–0.194 (0.188); MD = 0.236–0.249 (0.240); WL = 1.687–1.839 (1.754); SPST = 0.456–0.495 (0.476); MPST = 0.424–0.509 (0.459); PEL = 0.593–0.629 (0.608); NOL = 0.309–0.350 (0.323); NOH = 0.174–0.231 (0.194); PEH = 0.322–0.410 (0.362); PPL = 0.265–0.323 (0.294); PW = 0.899–1.029 (0.958); SBPA = 0.444–0.520 (0.478); SPTI = 0.407–0.493 (0.459); PEW = 0.239–0.271 (0.254); PNW = 0.370–0.471 (0.409); PPW = 0.418–0.490 (0.442); HFL = 1.004–1.162 (1.080); HFWmax = 0.187–0.201 (0.192); HFWmin = 0.079–0.086 (0.083); CS = 1.397–1.486 (1.432); ES = 0.339–0.371 (0.353); SI = 92–96 (94); OI = 24–25 (25); CI = 92–95 (93); WLI = 183–191 (188); SBI = 48–54 (51); PSI = 25–28 (27); PWI = 166–181 (174); PLI = 195–224 (207); NI = 152–178 (168); PNWI = 154–174 (161); NLI = 51–56 (53); FI = 220–239 (232).

**Gyne description:** In full-face view, head subquadrate, longer than broad (CI 92-95). Mandibles finely striate but shining and armed with five teeth: the apical-most well developed, followed by a less developed preapical tooth and three equally developed smaller teeth. Anterior clypeal margin flat medially. Antennal scapes very long: when fully retracted, surpassing the posterior margin of the head capsule by about two times the maximum width of the scape (SI 92–96). Antennae 12-segmented; antennal club composed of three segments, with the apical-most segment about as long as the preceding two in combination. Frontal carinae short, extending past the antennal toruli by about the maximum width of the antennal scape. Compound eyes strongly protruding past the lateral margins of the head capsule. Lateral margin of head evenly convex, converging from below the compound eyes to the mandibular insertions. Posterior head margin flat, rounding evenly into the lateral margins.

In profile view, compound eyes ovular and large (OI 24–25), with 21 ommatidia in longest row. Mesoscutum rounded evenly anteriorly, not fully covering the dorsal surface of the pronotum, and flat dorsally. Anterior margin of mesoscutellum on the same level as the mesoscutum; dorsum flat. Posterior margin of metanotum extending past the posterior margin of the mesoscutellum. Propodeal spiracle moderately well developed, directed posterolaterally, and separated from the propodeal declivity by about five spiracle diameters. Propodeal spines stout and well developed, but short (PSI 25–28), about two thirds as long as the propodeal declivity, tapering evenly from the base, directed posterodorsally, straight, and acute. Propodeal declivity weakly concave, forming a rounded ~120° angle with the base of the propodeal spines. Propodeal lobes rounded and very weakly developed. Metapleural gland bulla small, extending from the metacoxal insertion halfway to the propodeal spiracle. Petiole long (PLI 195–224), without tubercles anterodorsally. Subpetiolar process in the form of tiny, acute tooth, which grades evenly into the ventral margin of the petiole posteriorly. Petiolar peduncle moderately long: comprising half of the total petiole length. Petiolar node squamiform: transition between peduncle and node a rounded ~120° angle; anterior face rounding evenly into the posterior face, which forms a ~110° angle with the caudal cylinder. Postpetiole evenly rounded anterodorsally, bulging before flattening posterodorsally; concave ventrally.

In dorsal view, mesoscutum not fully covering pronotum anteriorly; humeri visible laterally as rounded sclerites. Mesoscutum evenly rounded anteriorly; anterior margin rounding evenly into the lateral margins; lateral margins diverging to the wing bases, then converging to the convex posterior margin. Propodeal spines strongly diverging basally, but parallel to each other apically, their apices separated from each other by about one and a half times their length. Petiolar peduncle with spiracles weakly protruding past the lateral margins. Petiolar node, when viewed posterodorsally, roughly trapezoidal: broader apically than basally; apical margin convex; node broader than the peduncle and the caudal cylinder. Postpetiole narrow (PWI 166–181) and subquadrate. Anterior margin of postpetiole weakly convex, with corners rounding evenly into the lateral margins; lateral margins weakly converging posteriorly; posterior margin broadly concave. Metafemur weakly incrassate (FI 220–239).

Sculpture: median clypeal carina present, extending from the anterior margin to frontal triangle, and flanked by multiple slightly weaker carinae. Lateral clypeal lobes with additional weaker carinae; ground sculpture weakly areolate. Antennal scapes areolate-costulate. Cephalic dorsum densely rugose over areolate ground sculpture, becoming costate between the frontal carinae; fine concentric costulae surrounding the antennal insertions. Lateral surfaces of head sculptured similarly to the dorsum. Ventral surface of head weakly costulate. Pronotal neck areolate. Lateral surfaces of the mesosoma sculptured similarly to the head. Propodeal declivity transversely rugose. Mesoscutum and mesoscutellum areolate, with fine costulate sculpture. Metanotum finely areolate. Propodeum coarsely rugose. Femora predominantly smooth and shining; basal quarter weakly areolate. Petiole finely longitudinally areolate-costulate on nearly all surfaces, but smooth and shining on the ventral surface of the petiole. Postpetiole weakly, finely areolate. First gastral tergite with fine areolate-costulate sculpture on the basal half; otherwise smooth and shining with weak spectral iridescence. First gastral sternite weakly areolate basally; otherwise smooth and shining, without spectral iridescence.

Setae: antennal scapes and funiculi with long, suberect pilosity. Dorsum of the head, pronotum, waist segments, and gaster with abundant, erect, tapering, flexuous setae, the longest of which are about the width of the compound eye. Pubescence present over the entire body, which is nearly as long as the setae.

Color: predominantly testaceous yellow. Antennae, masticatory margin of mandibles, tibiae, and first tarsomere dark brown. Interior of ocellar triangle, lateral margins of mesoscutum, wing bases, apex of femora, tarsi, dorsum of petiole and postpetiole, posterior margins of gastral tergites, and sting testaceous.

**Male measurements & indices (*n* = 1):** SL = 0.227; FRS = 0.086; CW = 0.686; CWb = 0.556; PoOC = 0.249; CL = 0.597; EL = 0.262; EW = 0.200; MD = 0.068; WL = 1.206; SPST = n/a; MPST = 0.312; PEL = 0.406; NOL = 0.234; NOH = 0.070; PEH = 0.183; PPL = 0.203; PW = 0.777; SBPA = n/a; SPTI = n/a; PEW = 0.176; PNW = 0.160; PPW = 0.271; HFL = 1.004; HFWmax = 0.095; HFWmin = 0.056; CS = 0.855; ES = 0.362; SI = 41; OI = 42; CI = 93; WLI = 217; SBI = n/a; PSI = n/a; PWI = 154; PLI = 200; NI = 334; PNWI = 91; NLI = 58; FI = 170.

**Male description:** In full-face view, head subovate, slightly longer than broad (CI 93). Mandibles shining through very weak striae, and armed with five teeth: the apical-most well developed, followed by a smaller preapical tooth and three roughly equally developed smaller teeth. Anterior clypeal margin entire and weakly convex. Antennal scapes short: when fully retracted, failing to reach the posterior margin of the head capsule by about the length of the scape (SI 41). Antennae 13-segmented; antennal club composed of four segments, with the apical-most segment about as long as the preceding two in combination. Frontal carinae short, extending past the antennal toruli by about the maximum width of the antennal scape. Compound eyes strongly protruding past the lateral margins of the head capsule. Lateral margin of head convex, margin between the anterior margin of the compound eye and the mandibular insertions straight. Posterior head margin evenly convex, rounding evenly into the lateral margins.

In profile view, compound eyes ovular and large (OI 46), with 24 ommatidia in the longest row. Mesoscutum bulging anteriorly, fully covering the dorsal surface of the pronotum, convex dorsally. Mesoscutellum depressed slightly below the level of the mesoscutum, convex dorsally. Posterior margin of metanotum extending beyond the posterior margin of the mesoscutellum. Propodeum strongly depressed; flat dorsally and transitioning into the propodeal declivity through a broadly rounded ~95° angle. Propodeal spiracle moderately well developed, directed posterolaterally, and separated from the propodeal declivity by about four spiracle diameters. Propodeal spines absent. Propodeal lobes rounded and weakly developed. Metapleural gland bulla small, extending a sixth of the way between the insertion of the metacoxa and the propodeal spiracle. Petiole moderately long (PLI 200), without tubercles anterodorsally. Subpetiolar process a minute, acute tooth; ventral margin of petiole bulging slightly posterior to it. Petiolar peduncle moderately long: comprising about half of the total petiole length. Petiolar node low and truncated dorsally. Postpetiole evenly rounded anterodorsally, bulging slightly before flattening dorsally, and concave ventrally.

In dorsal view, mesoscutum fully covering pronotum anteriorly; humeri not visible laterally. Petiolar peduncle with spiracles strongly protruding past the lateral margins. Petiolar node narrower than the peduncle where the spiracles arise, but broader than the caudal cylinder; trapezoidal when view at a posterodorsal aspect, the apex flat and narrower than the base. Postpetiole narrow (PWI 154) and campaniform. Anterior margin of postpetiole weakly convex, with the anterior corners evenly rounding into the lateral margins, which evenly diverge to the angulate posterior corners; posterior margin of postpetiole flat. Metafemur not incrassate (FI 170).

Sculpture: median clypeal carina present, extending from the anterior margin of the clypeus to the frontal triangle, flanked on either side by one weaker carina. Antennal scapes shining. Dorsum of head areolate, with weak rugae between the compound eye and antennal insertion. Lateral surface of head areolate, with weak rugae between the compound eye and mandibular insertion. Ventral surface of head shining, with weak areolae. Pronotal neck weakly areolate. Anterior face of pronotum shining through weak areolate sculpture. Lateral surface of pronotum predominantly weakly areolate. Katepisternum weakly areolate, with sculpture becoming weaker on the posterior two thirds. Anepisternum predominantly smooth and shining, with weak areolate sculpture around the margins. Metapleuron predominantly smooth and shining, with a small patch of weak areolate sculpture over the metapleural gland bulla. Lateral face of the propodeum areolate. Propodeal declivity smooth and shining. Dorsally, mesoscutum areolate-costulate, with a smooth and shining strip anteromedially. Mesoscutellum predominantly areolate-costulate, but sculpture weaker medially. Femora smooth and shining. Petiole predominantly smooth and shining, but base of node weakly, indistinctly sculptured; dorsum of peduncle weakly rugulose. Dorsal surface of postpetiole shining, with traces of weak areolate sculpture laterally and on the posterior quarter. First gastral tergite and sternite smooth and shining, without spectral iridescence.

Setae: antennal scapes and funiculi with long, suberect pilosity. Dorsum of the head, pronotum, waist segments, and gaster with abundant, erect, tapering, flexuous setae, the longest of which are about one and a half times the width of the compound eye. Pubescence present over the entire body, which is nearly as long as the setae.

Color: predominantly dark brown, with light yellow, nearly white antennae, mouthparts, legs, and genitalia.

**Etymology:** Morphological, from ‘aztecus’ + Latin ‘-oides’ (= resembling), in reference to the superficial similarity between this species and *Temnothorax aztecus*.

**Comments:** *Temnothorax aztecoides* sp. nov. is known from several collections made from mid elevation wet forests in Veracruz and Oaxaca States, Mexico. This species apparently nests primarily in hollow twigs and vines suspended from live trees, and forages arboreally like many of its close relatives in the *salvini* species group. *Temnothorax aztecoides* sp. nov. is not particularly closely related to *T. aztecus* (having shared a common ancestor approximately 10 Ma (Prebus, 2021)), but the two species are very similar in appearance and have overlapping geographical ranges, especially in the Los Tuxtlas mountain complex (and probably the southern Sierra Madre) in Mexico (Figs. 153A & 153B). *Temnothorax aztecoides* sp. nov. appears to inhabit higher elevations (>1,000 m), while *T. aztecus* inhabits lower elevations (<1,200 m) (Figs. 153A & 153B).

***Temnothorax aztecus* (Wheeler, 1931)**

**Figure 155:**
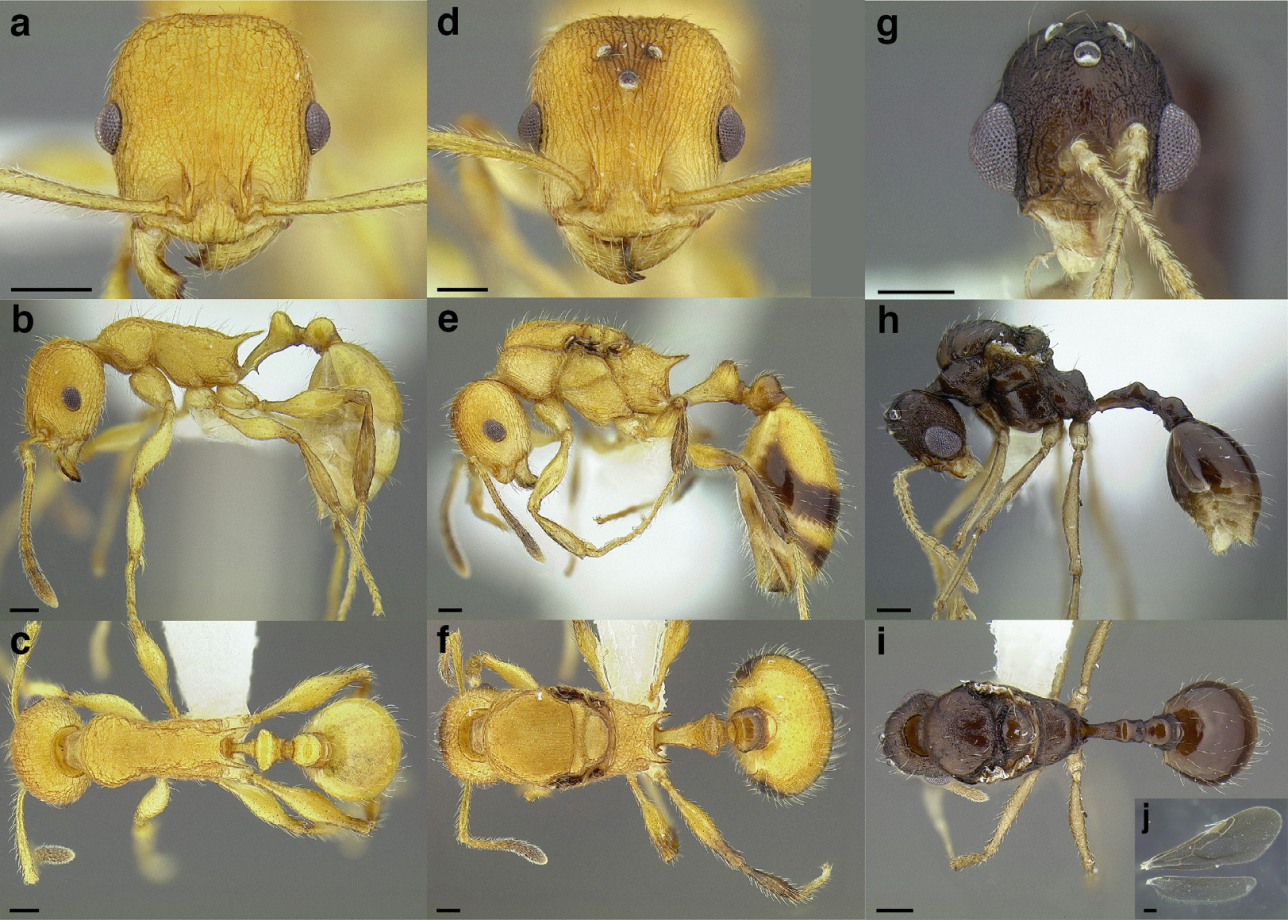
*Temnothorax aztecus*. (A–C) Worker (CASENT0758793). (A) Full-face view. (B) Profile view. (C) Dorsal view. (D–F) Gyne (JTLC000014408). (D) Full-face view. (E) Profile view. (F) Dorsal view. (G–J) Male (CASENT0609756). (G) Full-face view. (H) Profile view. (I) Dorsal view. (J) Wings. Scale bars 0.2 mm.

Distribution: Fig. 153B; worker, gyne & male: Fig. 155.

*Macromischa azteca* Wheeler, 1931: 8. Syntype workers, gyne, and males: Mirador, Mexico. One syntype worker here designated **lectotype**.

*Macromischa azteca maya* Wheeler, 1937: 458. Syntype workers and gynes. Mocá, Guatemala. Junior synonym of *Temnothorax aztecus* by Baroni Urbani, 1978: 425. One syntype worker here designated **lectotype**.

*Leptothorax aztecus* (Wheeler): Baroni Urbani, 1978: 425. First combination in *Leptothorax*.

*Temnothorax aztecus* (Wheeler): Bolton, 2003: 271. First combination in *Temnothorax*.

**Type material examined:** *Lectotype worker of* Macromischa azteca: MEXICO: Veracruz: Mirador, 10 May 1929, E. Skwarra#Z435/Sk, top specimen on pin (images of M.C.Z. Type 16357 examined on the MCZ Type Database website) [MCZC].

*Paralectotype workers of* Macromischa azteca: same pin as lectotype (M.C.Z. Type 16357, remaining workers on pin) [MCZC]; same data as previous: 3 workers (LACMENT323181) [LACM]; same data as previous, except: E. Skwarra#Z420/Sk., 3 workers (LACMENT181967) [LACM].

*Lectotype worker of* Macromischa azteca maya: GUATEMALA: Sololá: Mocá: 22 March 1935, W.M. Wheeler (images of M.C.Z. co-type 22801 examined on the M.C.Z. Type Database website).

*Paralectotype workers of* Macromischa azteca maya: 3 workers (syntypes of *Macromischa azteca maya*, LACMENT181956) [LACM].

**Non-type material examined:** BELIZE: Cayo: Chiquibul National Park, Doyle’s Delight, Dry Creek Trail, 16.483333°N 89.050000°W, 950–1,100 m, 20–22 August 2007, P. Kovarik#2007-09, yellow pan trap, 1 worker (CASENT0614827) [UCDC].

GUATEMALA: Izabal: 16 km ESE Morales: 15.41109°N 88.71184°W ± 108 m, 440 m, 19 May 2009, LLAMA#Go-B-04-3-01, 2° lowland rainforest, beating vegetation, 1 worker (CASENT0613028) [JTLC]; 15.40780°N 88.69698°W ± 210 m, 530 m, 19 May 2009, LLAMA#Go-B-04-3-04, 2° lowland rainforest, beating vegetation, 1 worker (CASENT0758692) [UCDC]; 15.41109°N 88.71184°W ± 58 m, 440 m, 19 May 2009, LLAMA#Ba-B-04-3-02-07, 2° lowland rainforest, at bait, 1 worker (CASENT0610593) [JTLC]; 15.41186°N 88.71071°W ± 28 m, 410 m, 19 May 2009, LLAMA#Wm-B-04-2-04, 2° lowland rainforest, ex sifted leaf litter, 1 worker (CASENT0611481) [JTLC].

HONDURAS: Atlántida: 10 km SSW Tela, 15.69843°N 87.47256°W ± 20 m, 550 m, 17 June 2010, LLAMA#Wm-C-08-2-03, tropical wet forest, ex sifted leaf litter, 1 worker (CASENT0618641) [JLTC]; 15.69479°N 87.47707°W ± 20 m, 720 m, 17 June 2010, LLAMA#Wm-C-08-1-10, tropical wet forest, ex sifted leaf litter, 1 worker (CASENT0618595) [JTLC]; 12 km SW La Ceiba, 15.69134°N 86.86137°W ± 20 m, 280 m, 19 June 2010, LLAMA#Wa-C-09-2-40, tropical rainforest, ex sifted leaf litter, 1 worker (CASENT0624888) [JTLC]. Comayagua: Parque National Cerro Azul Meambar: 14.86991°N 87.89879°W ± 20 m, 1,120 m, 20 May 2010, LLAMA#Wa-C-04-1-46, 1 worker (CASENT0615293) [JTLC]; 14.86979°N 87.89874°W ± 20 m, 1,120 m, 20 May 2010, LLAMA#Wa-C-04-1-49, 1 worker (CASENT0758693) [UCDC]; same data as previous, except: 14.886993°N 87.90479°W ± 110 m, 770 m, 21 May 2010, LLAMA#Wm-C-04-2-01, montane rainforest, ex sifted leaf litter, 1 worker (CASENT0617422) [JTLC].

MEXICO: Chiapas: Lago Metzabok: 17.12562°N 91.63086°W ± 300 m, 570 m, 6 June 2008, LLAMA#Go-A-06-1-03, lowland wet forest, beating vegetation, 1 worker (CASENT0609815) [JTLC]; 17.12366°N 91.63716°W, 575 m, 5 June 2008, LLAMA Wa-A-06-2-18, lowland wet forest, ex sifted leaf litter, 1 dealate gyne (JTLC000014408) [JTLC]; 17.12365°N 91.63748°W, 575 m, 5 June 2008, LLAMA Wa-A-06-2-25, lowland wet forest, ex sifted leaf litter, 1 dealate gyne (JTLC000014413) [JTLC]; 17.12380°N 91.63635°W, ± 100 m, 575 m, 8 June 2008, D.J. Cox#0142, 1 worker (CASENT0609754) [JTLC] 1 worker (CASENT0609755) [JTLC] 1 worker (CASENT0758793) [JTLC] 1 worker (CASENT0758691) [UCDC] 1 male (CASENT0609756) [JTLC]. Oaxaca: Uluapan, 4 km NE Ayautla, 18.05906°N 96.64564°W ± 250 m, 400 m, 10 June 2016, ADMAC#Go-F-03-1-03, lowland rainforest, beating vegetation, 1 worker (CASENT0640555) [JTLC]; Veracruz: Los Tuxtlas: 26 July 1974, R.L. Jeanne, forest, on understory vegetation, 1 worker (LACMENT323179) [LACM]; 23 July 1974, R.L. Jeanne, forest, on understory vegetation, 1 worker (LACMENT323180) [LACM] 1 worker (CASENT0759976) [FSCA]; 1 km SW Los Tuxtlas Biological Station, 18.57863°N 95.08113°W ± 40 m, 450 m ± 10 m, 16 August 2014, M.M. Prebus#MMP01643, lowland tropical rainforest, beating vegetation, 1 worker (CASENT0733469) [MMPC]; 18.58090°N 95.08085°W ± 20 m, 410 m ± 5 m, 16 August 2014, M.M. Prebus#MMP01648, lowland tropical rain forest, nest in small twig, 42 workers (CASENT0732975) [MMPC]; 0.5 km SW Los Tuxtlas Biological Station, 18.58272°N 95.07763°W ± 20 m, 280 m ± 5 m 16 August 2014, M.M. Prebus#MMP01650, lowland tropical rainforest, beating vegetation, 1 worker (CASENT0733501) [MMPC].

NICARAGUA: Matagalpa: Reserva Natural Cerro Musún: 12.96392°N 85.23242°W ± 100 m, 900 m, 3 May 2011, LLAMA#Go-D-01-1-03, montane wet forest, beating vegetation, 1 worker (CASENT0627684) [JTLC] 1 worker (CASENT0627794) [JTLC]; 12.95949°N 85.22562°W ± 100 m, 650 m, 3 May 2011, LLAMA#Go-D-01-2-03, tropical wet forest, beating vegetation, 1 worker (CASENT0627800) [JTLC] 1 worker (CASENT0732561) [UCDC] 1 worker (CASENT0627802) [JTLC] 1 worker (CASENT0627801) [JTLC] 1 dealate gyne (CASENT0627698) [JTLC]; RAAN: PN Cerro Saslaya, 13.77361°N 84.98650°W ± 20 m, 510 m, 8 May 2011, LLAMA#Wm-D-02-1-05, tropical wet forest, ex sifted leaf litter, 1 worker (CASENT0628778) [JTLC].

**Geographic range:** Low-to-mid elevations of southern Mexico to Nicaragua (Fig. 153B).

**Worker diagnosis:** *Temnothorax aztecus* can be separated from all other species in the *salvini* clade by the following character combination: smaller species: WL < 1.17 mm; dorsum of mesosoma weakly sinuate; metanotal groove not impressed; propodeum not strongly depressed below the level of the promesonotum; propodeal spines present and longer than the propodeal declivity; subpetiolar tooth acutely spiniform and longer than the setae that arise from the peduncle directly above; petiolar node strongly squamiform: in dorsal view, petiolar node greater than or equal to 1.6 times as broad as caudal cylinder; dorsum of head predominantly costate-rugose over areolate ground sculpture; dorsum of petiolar node, postpetiole and entire first gastral tergite smooth and shining; setae on head, mesosoma, legs, waist segments and gaster erect to suberect, long, abundant and tapering; integument predominantly yellow.

**Similar species:** Fellow members of the *salvini* group. *Temnothorax aztecus* can be separated from other members of the *salvini* group by the strongly squamiform petiolar node (petiolar node less than or equal to 1.5 times as broad as the caudal cylinder in *T. longinoi* sp. nov., *T. quetzal* sp. nov., *T. fortispinosus* sp. nov., *T. parvidentatus* sp. nov., and *T. salvini*), yellow integument (*T. longinoi* sp. nov., *T. longicaulis* stat. nov., nom. nov., *T. quetzal* sp. nov., *T. fortispinosus* sp. nov., *T. parvidentatus* sp. nov., and *T. salvini* are variously colored, but never uniformly yellow), smooth dorsum of the petiolar node, postpetiole, and gaster (lightly sculptured in *T. aztecoides* sp. nov.), relatively large subpetiolar tooth (shorter than the setae that arises directly above it in *T. aztecoides* sp. nov.), and long propodeal spines (shorter than the propodeal declivity in *T. paraztecus* sp. nov.).

**Worker measurements & indices (*n* = 20):** SL = 0.706–0.798 (0.745); FRS = 0.219–0.262 (0.247); CW = 0.741–0.838 (0.802); CWb = 0.642–0.738 (0.701); PoOC = 0.278–0.328 (0.300); CL = 0.742–0.831 (0.799); EL = 0.167–0.225 (0.201); EW = 0.132–0.161 (0.144); MD = 0.188–0.220 (0.205); WL = 1.024–1.168 (1.104); SPST = 0.359–0.479 (0.404); MPST = 0.288–0.352 (0.323); PEL = 0.397–0.464 (0.439); NOL = 0.217–0.282 (0.245); NOH = 0.138–0.181 (0.155); PEH = 0.248–0.293 (0.264); PPL = 0.188–0.247 (0.210); PW = 0.492–0.548 (0.523); SBPA = 0.189–0.243 (0.225); SPTI = 0.255–0.334 (0.295); PEW = 0.140–0.172 (0.159); PNW = 0.258–0.309 (0.281); PPW = 0.225–0.275 (0.254); HFL = 0.756–0.897 (0.823); HFWmax = 0.177–0.202 (0.191); HFWmin = 0.047–0.071 (0.061); CS = 1.013–1.15 (1.101); ES = 0.233–0.303 (0.273); SI = 103–111 (106); OI = 23–27 (25); CI = 85–90 (88); WLI = 152–163 (157); SBI = 28–36 (32); PSI = 32–42 (37); PWI = 150–168 (159); PLI = 182–226 (209); NI = 120–177 (159); PNWI = 161–199 (177); NLI = 49–62 (56); FI = 273–379 (316).

**Worker description:** In full-face view, head subquadrate, longer than broad (CI 85–90). Mandibles finely striate but shining and armed with five teeth: the apical-most well developed and acute, followed by a less developed preapical tooth and three equally developed smaller teeth. Anterior clypeal margin flat medially. Antennal scapes moderately long: when fully retracted, surpassing the posterior margin of the head capsule by about the maximum width of the antennal scape (SI 103–111). Antennae 12-segmented; antennal club of composed of three segments, with the apical-most segment as long as the preceding two in combination. Frontal carinae moderately long, extending past the antennal toruli by about two times the maximum width of the antennal scape. Compound eyes strongly protruding past the lateral margins of the head capsule. Lateral margin of head weakly convex, forming a continuous arc from the mandibular insertions to the posterior margin of the head. Posterior head margin flat but rounding evenly into the lateral margins.

In profile view, compound eyes ovular and moderately large (OI 23–27), with 17 ommatidia in longest row. Pronotal declivity indistinct, neck and anterior face of pronotum forming a ~120° angle. Anterior face of pronotum evenly rounding into dorsal face. Promesonotum very weakly convex to the faint metanotal groove. Propodeum weakly convex. Promesonotal suture extending from the posterior margin of the procoxal insertion only to the mesothoracic spiracle, which is moderately well developed. Metanotal groove visible as a disruption of the sculpture laterally from where it arises between the mid- and hind coxae to where it ends in the poorly developed metathoracic spiracle, which is nearly indistinguishable against the ground sculpture. Propodeal spiracle well developed, directed posterolaterally, and separated from the propodeal declivity by about four spiracle diameters. Propodeal spines well developed and very long (PSI 32–42), slightly longer than the propodeal declivity, tapering evenly from the base, straight, and acute. Propodeal declivity weakly concave, forming a rounded ~120° angle with the base of the propodeal spines. Propodeal lobes rounded and weakly developed. Metapleural gland bulla moderately large, extending from the metacoxal insertion two thirds of the way to the propodeal spiracle. Petiole long (PLI 182–226), without tubercles anterodorsally. Subpetiolar process in the form of a moderately large, spiniform, acute tooth; ventral margin of petiole weakly concave posterior to it. Petiolar peduncle very long: comprising about two thirds of the total petiole length. Petiolar node squamiform: transition between peduncle and node marked by a rounded angle of ~120°; anterior face forming a ~90° angle with the very short, strongly convex dorsal face; dorsal face rounding evenly into the posterior face, which forms a ~90° angle with the caudal cylinder. Postpetiole evenly rounded anterodorsally, bulging before flattening posterodorsally; concave ventrally.

In dorsal view, humeri developed and distinct: evenly rounded and about one and a half times wider than the rest of the mesosoma; mesothoracic spiracles weakly protruding past the lateral margins of the mesosoma, visible as slight angles where the pronotum meets the mesonotum. Promesonotal suture visible as a darker band and disruption in the ground sculpture. Metanotal groove moderately well impressed. Propodeal spines broadly approximated basally and diverging apically, their apices separated from each other by about their length, the negative space between them “U” shaped. Petiolar peduncle with spiracles slightly protruding past the lateral margins, but not noticeably constricted anterior to them. Petiolar node, when viewed posterodorsally, trapezoidal: broader apically than basally; apical margin flat; node broader than the peduncle and the caudal cylinder. Postpetiole narrow (PWI 150–168) and campaniform. Anterior margin of the postpetiole convex, evenly rounding into the lateral margins, which diverge to the angulate posterior corners; posterior margin flat. Metafemur moderately to strongly incrassate (FI 273–379).

Sculpture: median clypeal carina present, extending posteriorly to the frontal triangle, and flanked on either side by multiple slightly weaker carinae. Lateral clypeal lobes with additional, weaker carinae; ground sculpture weakly areolate. Antennal scapes weakly areolate. Cephalic dorsum areolate, with predominantly costate-rugose sculpture overlying the ground sculpture; fine concentric costulae surrounding the antennal insertions. Lateral surfaces of head sculptured similarly to the dorsum, but rugae forming whorls around the compound eyes and rugose sculpture coarser between the compound eye and the mandibular insertion. Ventral surface of head smooth and shining, with weak costulae. Pronotal neck areolate. Lateral surfaces of the mesosoma sculptured similarly to the head, with costulae. Propodeal declivity strigulate. Dorsal surface of mesosoma areolate, with costate-rugose sculpture on the pronotum and strigulae on the propodeum. Femora shining through weak areolate sculpture. Petiole finely areolate on all surfaces but the node, which is smooth and shining. Postpetiole smooth and shining dorsally, weakly areolate laterally. First gastral tergite and sternite smooth and shining, without spectral iridescence.

Setae: antennal scapes and funiculi with moderately long, suberect pilosity. Dorsum of the head, pronotum, waist segments, and gaster with abundant, suberect, tapering, flexuous setae, the longest of which are slightly longer than the length of the compound eye and are directed toward the midline of the body. The head bears ~40, mesosoma ~42, petiole 4, postpetiole ~18, and first gastral tergite ~84 setae. Pubescence present over the entire body, which is nearly as long as the setae.

Color: predominantly yellow; masticatory margin of the mandible dark brown; tibiae and distal quarter of femora testaceous yellow.

**Gyne measurements & indices (*n* = 2):** SL = 0.756–0.757 (0.757); FRS = 0.293–0.311 (0.302); CW = 0.912–0.923 (0.918); CWb = 0.803–0.814 (0.809); PoOC = 0.332–0.348 (0.340); CL = 0.859–0.915 (0.887); EL = 0.247–0.250 (0.249); EW = 0.182–0.184 (0.183); MD = 0.205–0.237 (0.221); WL = 1.556–1.609 (1.583); SPST = 0.386–0.431 (0.409); MPST = 0.419–0.426 (0.423); PEL = 0.579–0.589 (0.584); NOL = 0.338–0.344 (0.341); NOH = 0.185–0.202 (0.194); PEH = 0.349–0.364 (0.357); PPL = 0.267–0.269 (0.268); PW = 0.857–0.864 (0.861); SBPA = 0.412–0.440 (0.426); SPTI = 0.383–0.419 (0.401); PEW = 0.225–0.239 (0.232); PNW = 0.315–0.388 (0.352); PPW = 0.376–0.394 (0.385); HFL = 0.913–0.947 (0.930); HFWmax = 0.188–0.191 (0.190); HFWmin = 0.064–0.071 (0.068); CS = 1.233–1.272 (1.252); ES = 0.338–0.342 (0.340); SI = 93–94 (94); OI = 27–28 (27); CI = 89–93 (91); WLI = 194–198 (196); SBI = 51–55 (53); PSI = 25–27 (26); PWI = 157–175 (166); PLI = 215–221 (218); NI = 167–186 (177); PNWI = 140–162 (151); NLI = 58–58 (58); FI = 265–298 (282).

**Gyne description:** In full-face view, head subquadrate, longer than broad (CI 89–93). Mandibles finely striate but shining and armed with five teeth: the apical-most well developed, followed by a less developed preapical tooth and three equally developed smaller teeth. Anterior clypeal margin flat medially. Antennal scapes moderately long: when fully retracted, surpassing the posterior margin of the head capsule by about the maximum width of the scape (SI 93–94). Antennae 12-segmented; antennal club composed of three segments, with the apical-most segment slightly shorter than the preceding two in combination. Frontal carinae moderately long, extending past the antennal toruli by about two times the maximum width of the antennal scape. Compound eyes strongly protruding past the lateral margins of the head capsule. Lateral margin of head evenly convex, converging from below the compound eyes to the mandibular insertions. Posterior head margin flat, rounding evenly into the lateral margins.

In profile view, compound eyes ovular and large (OI 27–28), with 17 ommatidia in longest row. Mesoscutum rounded evenly anteriorly, not fully covering the dorsal surface of the pronotum, and weakly convex dorsally. Anterior margin of mesoscutellum on the same plane as the mesoscutum but sloping posteriorly. Posterior margin of metanotum extending past the posterior margin of the mesoscutum. Propodeal spiracle very well developed, directed posterolaterally, and separated from the propodeal declivity by about five spiracle diameters. Propodeal spines stout and well developed, but short (PSI 25–27), about two thirds as long as the propodeal declivity, tapering evenly from the base, directed posteriorly, straight, and acute. Propodeal declivity straight and flat, forming a rounded ~120° angle with the base of the propodeal spines. Propodeal lobes rounded and very weakly developed, but slightly angulate dorsally. Metapleural gland bulla small, extending from the metacoxal insertion halfway to the propodeal spiracle. Petiole long (PLI 215–221), without tubercles anterodorsally. Subpetiolar process in the form of moderately long, spiniform, very acute tooth, which grades evenly into the ventral margin of the petiole posteriorly. Petiolar peduncle moderately long: comprising half of the total petiole length. Petiolar node squamiform: transition between peduncle and node an evenly rounded ~120° angle; anterior face forming a rounded ~110° angle with the dorsal face, which is short; dorsal face rounding evenly into the posterior face, which forms a ~110° angle with the caudal cylinder. Postpetiole evenly rounded anterodorsally, bulging before flattening posterodorsally; concave ventrally.

In dorsal view, mesoscutum not fully covering pronotum anteriorly; humeri visible laterally as rounded sclerites. Mesoscutum evenly rounded anteriorly; anterior margin rounding evenly into the lateral margins; lateral margins diverging to the wing bases, then converging through a curve to the convex posterior margin. Propodeal spines strongly diverging basally, but parallel to each other apically, their apices separated from each other by about one and a half times their length. Petiolar peduncle with spiracles weakly protruding past the lateral margins. Petiolar node, when viewed posterodorsally, roughly trapezoidal: broader apically than basally; apical margin convex; node broader than the peduncle and the caudal cylinder. Postpetiole narrow (PWI 157–175) and subquadrate. Anterior margin of postpetiole flat, with corners marked by rounded angles as it transitions to the lateral margins; lateral margins parallel to each other; posterior margin flat. Metafemur moderately incrassate (FI 265–298).

Sculpture: median clypeal carina present, extending from the anterior margin to frontal triangle, and flanked by multiple slightly weaker carinae. Lateral clypeal lobes with additional weaker carinae; ground sculpture weakly areolate. Antennal scapes weakly areolate. Cephalic dorsum areolate, with costate-rugose sculpture overlying the ground sculpture; fine concentric costulae surrounding the antennal insertions. Lateral surfaces of head sculptured similarly to the dorsum, but rugae forming whorls posterior to the compound eyes. Ventral surface of head smooth and shining, with weak costulae. Pronotal neck areolate. Lateral surfaces of the mesosoma sculptured similarly to the head, with costae over areolate sculpture; anepisternum with a small patch of smooth and shining sculpture anteriorly. Propodeal declivity strigulate. Mesoscutum and mesoscutellum areolate, with costulae and weak strigulae on the propodeum. Dorsum of propodeum coarsely rugose. Femora predominantly smooth and shining; basal quarter weakly areolate. Petiole finely longitudinally areolate-costulate on all surfaces, but sculpture on node is weaker. Postpetiole predominantly smooth and shining, with weak areolate sculpture laterally and on the posterior quarter. First gastral tergite with weak, fine costulae on the basal quarter; otherwise smooth and shining with weak spectral iridescence. First gastral sternite smooth and shining, without spectral iridescence.

Setae: antennal scapes and funiculi with moderately long, suberect pilosity. Dorsum of the head, pronotum, waist segments, and gaster with abundant, suberect, tapering, flexuous setae, the longest of which are slightly longer than the width of the compound eye. Pubescence present over the entire body, which is nearly as long as the setae.

Color: predominantly yellow; masticatory margin of the mandible, wing bases, and distal quarters of the gastral sclerites dark brown; tibiae and distal quarter of femora testaceous yellow. Tips of the propodeal spines and dorsum of waist segments testaceous.

**Male measurements & indices (*n* = 1):** SL = 0.186; FRS = 0.109; CW = 0.600; CWb = 0.473; PoOC = 0.206; CL = 0.514; EL = 0.246; EW = 0.179; MD = 0.056; WL = 0.951; SPST = n/a; MPST = 0.255; PEL = 0.334; NOL = 0.205; NOH = 0.06; PEH = 0.15; PPL = 0.157; PW = 0.587; SBPA = n/a; SPTI = n/a; PEW = 0.144; PNW = 0.138; PPW = 0.224; HFL = 0.858; HFWmax = 0.095; HFWmin = 0.048; CS = 0.73; ES = 0.336; SI = 39; OI = 46; CI = 92; WLI = 201; SBI = n/a; PSI = n/a; PWI = 156; PLI = 213; NI = 342; PNWI = 96; NLI = 61; FI = 198.

**Male description:** In full-face view, head subovate, slightly longer than broad (CI 92). Mandibles very weakly striate, shining, and armed with five teeth: the apical-most well developed, followed by a smaller preapical tooth and three roughly equally developed smaller teeth. Anterior clypeal margin entire and weakly convex. Antennal scapes short: when fully retracted, failing to reach the posterior margin of the head capsule by about five times the maximum width of the antennal scape (SI 39). Antennae 13-segmented; antennal club composed of four segments, with the apical-most segment slightly longer than the preceding two in combination. Frontal carinae short, extending past the antennal toruli by about the maximum width of the antennal scape. Compound eyes strongly protruding past the lateral margins of the head capsule. Lateral margin of head convex, margin between the anterior margin of the compound eye and the mandibular insertions straight. Posterior head margin evenly convex, rounding evenly into the lateral margins.

In profile view, compound eyes ovular and large (OI 46), with 24 ommatidia in the longest row. Mesoscutum bulging anteriorly, but not fully covering the dorsal surface of the pronotum, convex dorsally. Mesoscutellum depressed slightly below the level of the mesoscutum, convex dorsally. Posterior margin of metanotum extending beyond the posterior margin of the mesoscutellum. Propodeum strongly depressed; flat dorsally and meeting the propodeal declivity at a distinct ~100° angle. Propodeal spiracle moderately well developed, directed posterolaterally, and separated from the propodeal declivity by about three spiracle diameters. Propodeal spines absent. Propodeal lobes rounded and weakly developed. Metapleural gland bulla small, extending a quarter of the way between the insertion of the metacoxa and the propodeal spiracle. Petiole moderately long (PLI 218), without tubercles anterodorsally. Subpetiolar process a small, acute, triangular tooth; ventral margin of petiole bulging slightly posterior to it. Petiolar peduncle moderately long: comprising about half of the total petiole length. Petiolar node low and rounded, nearly cuneiform; the convergence of the anterior and dorsal faces marked by a rounded ~100° angle. Postpetiole evenly rounded anterodorsally, flattened dorsally, and concave ventrally.

In dorsal view, mesoscutum not fully covering pronotum anteriorly; humeri visible laterally as slivers of rounded sclerites. Petiolar peduncle with spiracles slightly protruding past the lateral margins. Petiolar node slightly wider than the peduncle and the caudal cylinder; trapezoidal when view at a posterodorsal aspect, the apex flat and broader than the base. Postpetiole narrow (PWI 156) and campaniform. Anterior margin of postpetiole flat, with the anterior corners evenly rounding into the lateral margins, which evenly diverge to the angulate posterior corners; posterior margin of postpetiole flat. Metafemur not incrassate (FI 198).

Sculpture: median clypeal lobe with three equally strong carinae. Antennal scapes shining, with traces of weak areolate sculpture. Dorsum of head areolate, with rugulae between the compound eye and antennal insertion. Lateral surface of head areolate, with rugulae surrounding the compound eye. Ventral surface of head shining, with weak costulae. Pronotal neck areolate. Anterior face of pronotum smooth and shining. Lateral surface of pronotum predominantly smooth and shining, with a posteromedial patch of finely areolate and costulate sculpture, and weak areolate sculpture near the dorsal and ventral margins. Katepisternum weakly areolate, with smooth and shining patches near the dorsal and ventral margins. Anepisternum smooth and shining. Metapleuron predominantly smooth and shining, with a small patch of weak areolate sculpture over the metapleural gland bulla. Lateral face of the propodeum weakly rugulose. Propodeal declivity weakly areolate. Dorsally, mesoscutum weakly areolate, with smooth and shining patches abutting the Mayrian furrows laterally. Mesoscutellum predominantly smooth and shining, with traces of weak costulate sculpture. Femora smooth and shining, with traces weak areolate sculpture. Petiole predominantly weakly areolate, but node smooth and shining. Dorsal surface of postpetiole shining, with traces of weak areolate sculpture laterally and on the posterior quarter. First gastral tergite and sternite smooth and shining, without spectral iridescence.

Setae: antennal scapes and funiculi with moderately long, suberect pilosity. Dorsum of the head, pronotum, waist segments, and gaster with abundant, suberect, tapering, flexuous setae, the longest of which are as long as the width of the compound eye. Pubescence present over the entire body, which is nearly as long as the setae.

Color: Predominantly testaceous brown, with yellow antennae, mouthparts, legs, and genitalia.

**Etymology:** Geographical, in reference to the type series locality in the former Aztec empire.

**Comments:** *Temnothorax aztecus* nests arboreally in hollow twigs and vines in the mid to low elevation tropical wet forests of Mesoamerica, from the Isthmus of Tehuantepec to the Nicaraguan depression. This species has also been collected in Winkler leaf litter extractions, suggesting that there may be some flexibility in nesting preference. Foragers are frequently collected on low vegetation. *Temnothorax aztecus* is not particularly closely related to *T. aztecoides* sp. nov. (having shared a common ancestor approximately 10 Ma (Prebus, 2021)), but the two species are very similar in appearance and have overlapping geographical ranges, especially in the Los Tuxtlas mountain complex (and probably the southern Sierra Madre) in Mexico (Figs. 153A & 153B). *Temnothorax aztecus* appears to inhabit lower elevations (<1,200 m), while *T. aztecoides* sp. nov. inhabits higher elevations (>1,000 m) (Figs. 153A & 153B). The closest relative of *T. aztecus* is the morphologically similar *T. paraztecus* sp. nov., with which it shared a common ancestor a little more than 7 Ma (Prebus, 2021). *Temnothorax aztecus* and *T. paraztecus* sp. nov. also overlap geographically in the Central American Nucleus, but *T. paraztecus* sp. nov., similar to *T. aztecoides* sp. nov. inhabits a generally higher elevational range (>950 m) than *T. aztecus* (Figs. 153B & 153F) Inhabiting a large present-day geographical range, *T. aztecus* is the only member of the *salvini* species group that is found below 900 m (Fig. 153B). Following speciation from the lineage leading to *T. paraztecus*, the *T. aztecus* lineage was inferred to have expanded into lower elevation habitats during the Miocene-Pliocene transition around 7.5 Ma (Prebus, 2021).

***Temnothorax fortispinosus* sp. nov.**

**Figure 156:**
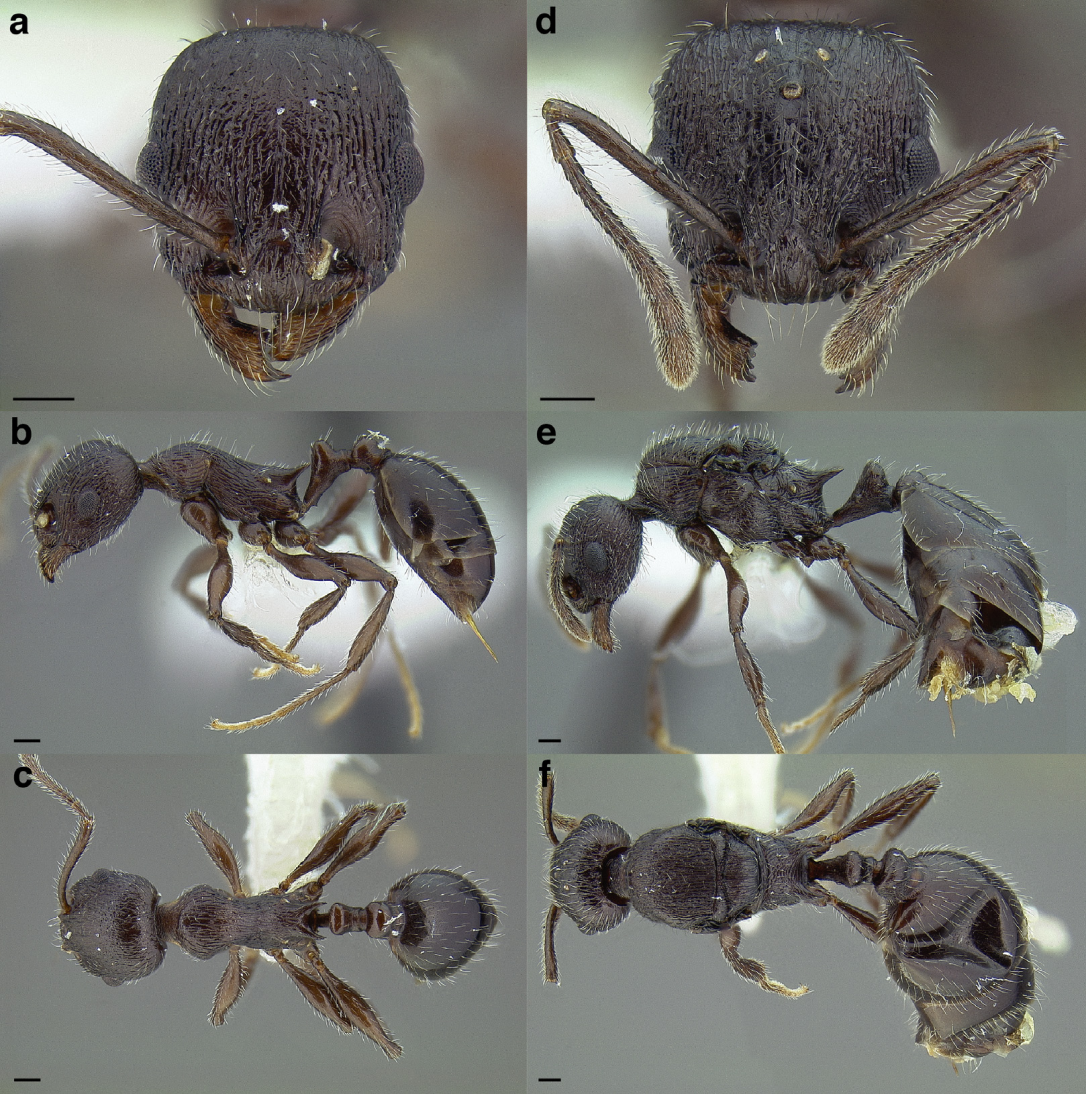
*Temnothorax fortispinosus* sp. nov. (A–C) Holotype worker (JTLC000010282). (A) Full-face view. (B) Profile view. (C) Dorsal view. (D–F) Paratype gyne (JTLC000010282). (D) Full-face view. (E) Profile view. (F) Dorsal view. Scale bars 0.2 mm.

Distribution: Fig. 153C; worker & gyne: Fig. 156.

*Temnothorax salvini* grp 2, Prebus, 2021: 12. In phylogeny.

**Type material examined:** *Holotype worker:* MEXICO: Chiapas: 2.6 km SE Custepec, 15.71648°N 92.94423°W, 1,740 m, 18 July 2007, J. Longino#6078, *Liquidambar* 2nd growth forest, nest in dead stick (JTLC000010282, top specimen on pin) [CASC].

*Paratype gyne and worker*: same pin as holotype, 1 dealate gyne (bottom specimen on pin) [CASC]; same data as holotype, 1 worker (JTLC000010283) [UCDC].

**Non-type material examined:** MEXICO: Chiapas: 2.6 km SE Custepec, 15.71648°N 92.94423°W, 1,740 m, 18 July 2007, J. Longino#JTL6077, *Liquidambar* 2nd growth forest, nest in dead stick, 1 worker (JTLC000010281) [JTLC]; J. Longino#JTL6079, *Liquidambar* 2nd growth forest, nest in dead stick, 1 worker (JTLC000010284) [JTLC]; 15.72298°N 92.94493°W, 1,650 m, 18 July 2007, R.S. Anderson#2007-021, ridgetop oak forest, ex sifted leaf litter, 1 worker (CASENT0601966) [JTLC]; 2 km SE Custepec, 15.72097°N 92.95059°W ± 50 m, 1,520 m, 17 May 2008, LLAMA#Wa-A-02-1-23, mesophyll forest, ex sifted leaf litter, 1 worker (CASENT0758699) [UCDC]; same data as previous, except: 15.72099°N 92.95054°W, 1,520 m, 18 May 2008, LLAMA#Go-A-02-1-01, mesophyll forest, beating vegetation, 1 worker (CASENT0609693) [JTLC] 1 worker (CASENT0609694) [JTLC]; same data as previous, except: 15.72219°N 92.94640°W, 1,610 m, 19 May 2008, LLAMA Ba-A-02-3-04-06, mesophyll forest, at bait, 1 worker (JTLC000014251) [MCZC]; Custepec, 15.72196°N 92.95037°W, 1,530 m, 19 May 2008, J. Longino#JTL6278-s, mesophyll forest, foragers, 1 worker, (JTLC000007469) [JTLC]; Darci Andresen#FBI B7, 10 July 1998 (LACMENT323459) [LACM].

**Geographic range:** Mid elevations of southern Mexico (Chiapas) (Fig. 153C).

**Worker diagnosis:** *Temnothorax fortispinosus* sp. nov. can be separated from all other species in the *salvini* clade by the following character combination: dorsum of mesosoma sinuate and predominantly longitudinally striate; metanotal groove not impressed; propodeum not strongly depressed below the level of the promesonotum; propodeal spines shorter than the propodeal declivity; integument smooth and shining between propodeal spines; subpetiolar tooth small and triangular: shorter than the setae that arise from the peduncle directly above; petiolar node weakly squamiform: in dorsal view, petiolar node more than or equal to 1.2 times as broad as caudal cylinder, but less than or equal to 1.5 times as broad; setae on head, mesosoma, legs, waist segments and gaster erect to suberect, moderately long, abundant and tapering; integument dark brown.

**Similar species:** Fellow members of the *salvini* group. *Temnothorax fortispinosus* sp. nov. can be separated from other members of the *salvini* group by the weakly squamiform petiolar node (petiolar node more than or equal to 1.6 times as broad as the caudal cylinder in *T. aztecus, T. aztecoides* sp. nov., *T. longicaulis* stat. nov., nom. nov*.*, and *T. quetzal* sp. nov., but less than 1.3 times in *T. salvini*), relatively small subpetiolar tooth (longer than the setae that arises directly above it in *T. longinoi* sp. nov.), short propodeal spines (longer than the propodeal declivity in *T. quetzal* sp. nov.), and smooth integument between the propodeal spines (sculptured in *T. parvidentatus* sp. nov. and *T. salvini*).

**Worker measurements & indices (*n* = 5):** SL = 0.815–0.881 (0.861); FRS = 0.281–0.296 (0.288); CW = 0.916–0.974 (0.948); CWb = 0.819–0.877 (0.850); PoOC = 0.344–0.377 (0.363); CL = 0.921–0.971 (0.944); EL = 0.212–0.242 (0.229); EW = 0.152–0.183 (0.165); MD = 0.205–0.218 (0.211); WL = 1.204–1.271 (1.245); SPST = 0.301–0.352 (0.323); MPST = 0.346–0.382 (0.361); PEL = 0.435–0.486 (0.465); NOL = 0.247–0.284 (0.266); NOH = 0.167–0.193 (0.177); PEH = 0.300–0.316 (0.309); PPL = 0.225–0.247 (0.234); PW = 0.560–0.612 (0.588); SBPA = 0.243–0.267 (0.257); SPTI = 0.260–0.291 (0.277); PEW = 0.178–0.195 (0.185); PNW = 0.218–0.256 (0.239); PPW = 0.279–0.306 (0.297); HFL = 0.930–0.988 (0.953); HFWmax = 0.176–0.204 (0.190); HFWmin = 0.062–0.070 (0.066); CS = 1.281–1.363 (1.323); ES = 0.294–0.328 (0.312); SI = 99–107 (101); OI = 23–24 (24); CI = 89–91 (90); WLI = 143–152 (147); SBI = 28–32 (30); PSI = 24–28 (26); PWI = 156–167 (161); PLI = 182–215 (199); NI = 136–160 (151); PNWI = 119–139 (129); NLI = 53–61 (57); FI = 279–306 (288).

**Worker description:** In full-face view, head subquadrate, longer than broad (CI 89–91). Mandibles finely striate but shining and armed with five teeth: the apical-most well developed and acute, followed by a less developed preapical tooth and three equally developed smaller teeth. Anterior clypeal margin flat medially. Antennal scapes moderately long: when fully retracted, surpassing the posterior margin of the head capsule by about the maximum width of the antennal scape (SI 99–107). Antennae 12-segmented; antennal club of composed of three segments, with the apical-most segment slightly longer than the preceding two in combination. Frontal carinae moderately long, extending past the antennal toruli by about two times the maximum width of the antennal scape. Compound eyes moderately protruding past the lateral margins of the head capsule. Lateral margin of head weakly convex, forming a continuous arc from the mandibular insertions to the posterior margin of the head. Posterior head margin flat but rounding evenly into the lateral margins.

In profile view, compound eyes ovular and moderately large (OI 23–24), with 17 ommatidia in longest row. Pronotal declivity indistinct, neck and anterior face of pronotum forming a ~120° angle. Anterior face of pronotum evenly rounding into dorsal face. Mesosoma sinuate: propodeum slightly depressed below the level of the promesonotum, but dorsal outline is continuous; metanotal groove not impressed. Promesonotal suture extending from the posterior margin of the procoxal insertion only to the mesothoracic spiracle, which is moderately well developed. Metanotal groove visible as a disruption of the sculpture laterally from where it arises between the mid- and hind coxae to where it ends in the poorly developed metathoracic spiracle, which is nearly indistinguishable against the ground sculpture. Propodeal spiracle moderately well developed, directed posterolaterally, and separated from the propodeal declivity by about five spiracle diameters. Propodeal spines stout and short (PSI 24–28), three quarters as long as the propodeal declivity, flared at the base, straight, and acute. Propodeal declivity flat, forming a rounded ~120° angle with the base of the propodeal spines. Propodeal lobes rounded and weakly developed. Metapleural gland bulla large, extending from the metacoxal insertion three quarters of the way to the propodeal spiracle. Petiole long (PLI 182–215), without tubercles anterodorsally. Subpetiolar process in the form of a small, triangular, acute tooth, which grades evenly into the ventral margin posteriorly; ventral margin of petiole slightly bulging posterior to it. Petiolar peduncle short: comprising about a third of the total petiole length. Petiolar node rounded-cuneiform: transition between peduncle and node marked by a rounded angle of ~130°; anterior face evenly rounding into the short, strongly convex dorsal face; dorsal face rounding evenly into the posterior face, which forms a ~95° angle with the caudal cylinder. Postpetiole evenly rounded anterodorsally, bulging before flattening posterodorsally; concave ventrally.

In dorsal view, humeri developed and distinct: evenly rounded and about one and a half times wider than the rest of the mesosoma; mesothoracic spiracles weakly protruding past the lateral margins of the mesosoma, visible as slight angles where the pronotum meets the mesonotum. Promesonotal suture not visible. Metanotal groove not visible. Propodeal spines broadly approximated basally and weakly diverging apically, their apices separated from each other by about their length, the negative space between them “U” shaped. Petiolar peduncle with spiracles slightly protruding past the lateral margins, but not noticeably constricted anterior to them. Petiolar node, when viewed posterodorsally, weakly trapezoidal: slightly broader apically than basally; apical margin weakly convex; node broader than the peduncle and the caudal cylinder. Postpetiole narrow (PWI 156–167) and subquadrate. Anterior margin of postpetiole weakly convex, transitioning into the lateral margins through a rounded angle; lateral margins bulging slightly anteriorly, weakly constricted medially, and weakly diverging posteriorly; posterior margin flat. Metafemur moderately to strongly incrassate (FI 279–306).

Sculpture: median clypeal carina present, extending posteriorly to the frontal triangle, and flanked on either side by multiple slightly weaker carinae. Lateral clypeal lobes with additional, weaker carinae; ground sculpture weakly areolate. Antennal scapes weakly areolate. Cephalic dorsum areolate, with costate-rugose sculpture overlying the ground sculpture. Lateral surfaces of head sculptured similarly to the dorsum, but sculpture becoming rugose and coarse between the compound eye and the mandibular insertion. Ventral surface of head smooth and shining anteriorly, with areolate-rugulose sculpture posterolaterally. Pronotal neck transversely areolate-costulate. Lateral surfaces of the mesosoma with costae over ground sculpture that is much more weakly areolate than the head; region between the propodeal spiracle and the base of the propodeal spine smooth and shining. Propodeal declivity predominantly smooth and shining, with traces of weak areolae. Dorsal surface of mesosoma sculptured similarly to the lateral surfaces. Femora shining through weak areolate sculpture. Petiole finely areolate on all surfaces but the node, which is smooth and shining. Postpetiole smooth and shining dorsally, weakly areolate laterally and on the posterior quarter. First gastral tergite and sternite smooth and shining, without spectral iridescence.

Setae: antennal scapes and funiculi with moderately long, suberect pilosity. Dorsum of the head, pronotum, waist segments, and gaster with abundant, suberect, tapering, flexuous setae, the longest of which are about the length of the compound eye and are directed toward the midline of the body. The head bears ~60, mesosoma ~80, petiole ~14, postpetiole ~22, and first gastral tergite ~84 setae. Pubescence present over the entire body, which is nearly as long as the setae.

Color: predominantly dark brown; mandibles, antennal club, pronotal neck, and tarsi testaceous brown.

**Gyne measurements & indices (*n* = 1):** SL = 0.901; FRS = 0.355; CW = 1.121; CWb = 1.037; PoOC = 0.440; CL = 1.077; EL = 0.298; EW = 0.219; MD = 0.205; WL = 1.804; SPST = 0.479; MPST = 0.471; PEL = 0.610; NOL = 0.326; NOH = 0.265; PEH = 0.461; PPL = 0.286; PW = 0.984; SBPA = 0.504; SPTI = 0.487; PEW = 0.261; PNW = 0.357; PPW = 0.442; HFL = 1.057; HFWmax = 0.211; HFWmin = 0.080; CS = 1.576; ES = 0.408; SI = 87; OI = 26; CI = 96; WLI = 174; SBI = 49; PSI = 27; PWI = 169; PLI = 213; NI = 123; PNWI = 137; NLI = 53; FI = 264.

**Gyne description:** In full-face view, head subquadrate, slightly longer than broad (CI 96). Mandibles finely striate but shining and armed with five teeth: the apical-most well developed, followed by a less developed preapical tooth and three equally developed smaller teeth. Anterior clypeal margin very weakly emarginate medially. Antennal scapes moderately long: when fully retracted, surpassing the posterior margin of the head capsule by about half the maximum width of the scape (SI 87). Antennae 12-segmented; antennal club composed of three segments, with the apical-most segment as long as the preceding two in combination. Frontal carinae short, extending past the antennal toruli by about one and a half times the maximum width of the antennal scape. Compound eyes strongly protruding past the lateral margins of the head capsule. Lateral margin of head evenly convex, converging from below the compound eyes to the mandibular insertions. Posterior head margin flat, rounding evenly into the lateral margins.

In profile view, compound eyes ovular and large (OI 26), with 20 ommatidia in longest row. Mesoscutum rounded evenly anteriorly, not fully covering the dorsal surface of the pronotum, and weakly convex dorsally. Mesoscutellum convex; anterior margin on the same plane as the mesoscutum but sloping posteriorly. Posterior margin of metanotum extending past the posterior margin of the mesoscutum. Propodeal spiracle very well developed, directed posterolaterally, and separated from the propodeal declivity by about four spiracle diameters. Propodeal spines well developed, but short (PSI 27), about three quarters as long as the propodeal declivity, flared at the base, directed posteriorly, straight, and acute. Propodeal declivity straight and flat, forming a rounded ~110° angle with the base of the propodeal spines. Propodeal lobes rounded and very weakly developed. Metapleural gland bulla small, extending from the metacoxal insertion halfway to the propodeal spiracle. Petiole long (PLI 213), without tubercles anterodorsally. Subpetiolar process in the form of a tiny, blunt tooth, which grades evenly into the ventral margin of the petiole posteriorly. Petiolar peduncle moderately long: comprising half of the total petiole length. Petiolar node cuneiform-squamiform: transition between peduncle and node an evenly rounded ~120° angle; anterior face forming a rounded ~60° angle with posterior face, which forms a ~110° angle with the caudal cylinder. Postpetiole weakly convex anteriorly, evenly rounded anterodorsally, bulging before flattening posterodorsally; concave ventrally.

In dorsal view, mesoscutum not fully covering pronotum anteriorly; humeri visible laterally as rounded sclerites. Mesoscutum evenly rounded anteriorly; anterior margin rounding evenly into the lateral margins; lateral margins diverging to the wing bases, then converging through a curve to the convex posterior margin. Propodeal spines strongly diverging basally, but converging slightly apically, their apices separated from each other by about one and a half times their length. Petiolar peduncle with spiracles protruding past the lateral margins. Petiolar node, when viewed posterodorsally, weakly trapezoidal: slightly broader apically than basally; apical margin convex; node broader than the peduncle and the caudal cylinder. Postpetiole narrow (PWI 169) and subquadrate. Anterior margin of postpetiole flat, with corners marked by rounded ~90° angles as it transitions to the lateral margins; lateral margins parallel to each other; posterior margin flat. Metafemur moderately incrassate (FI 264).

Sculpture: median clypeal carina present, extending from the anterior margin to frontal triangle, and flanked by multiple equally strong carinae. Lateral clypeal lobes with additional weaker carinae; ground sculpture weakly areolate. Antennal scapes weakly areolate. Cephalic dorsum areolate, with costate-rugose sculpture overlying the ground sculpture; fine concentric costulae surrounding the antennal insertions. Lateral surfaces of head sculptured similarly to the dorsum, but sculpture becoming rugose near the compound eye, and between the compound eye and mandibular insertion. Ventral surface of sculptured similarly to the dorsum, but weaker. Pronotal neck areolate-strigulate. Lateral surfaces of the mesosoma sculptured similarly to the head, but areolate sculpture much weaker; katepisternum and anepisternum with costae becoming weaker anteriorly. Propodeal declivity strigulate. Mesoscutum and mesoscutellum areolate, with costulate sculpture. Metanotum finely costulate. Propodeum with coarse concentric costae. Femora predominantly smooth and shining, with traces of weak areolate sculpture. Petiole predominantly finely areolate, with fine costulae at the base of the node; sculpture on node is weaker. Anterior face of postpetiole smooth and shining, otherwise finely areolate. First gastral tergite with weak, fine costulae on the basal quarter; otherwise smooth and shining with weak spectral iridescence. First gastral tergite and sternite smooth and shining, without spectral iridescence.

Setae: antennal scapes and funiculi with moderately long, suberect pilosity. Dorsum of the head, pronotum, waist segments, and gaster with abundant, suberect, tapering, flexuous setae, the longest of which are about the width of the compound eye. Pubescence present over the entire body, which is nearly as long as the setae.

Color: predominantly dark brown; mandibles, antennal club, pronotal neck, and tarsi testaceous brown.

**Male:** Unknown.

**Etymology:** Morphological, from the Latin ‘fortis’ (= robust) + ‘spinosus’ (= thorned), in reference to the stout propodeal spines.

**Comments:** *Temnothorax fortispinosus* sp. nov. is only known from several collections from mid elevation forest in the type locality of Custepec, Chiapas State, Mexico. This species, like many of its close relatives in the *salvini* species group, is probably arboreally nesting. The closest relative of *T. fortispinosus* sp. nov., *T. quetzal* sp. nov., is also known only from a single locality in the Central American Nucleus, but at a relatively high elevation (1,925 m; Figs. 153C & 153H). These two morphologically distinctive species shared a common ancestor around 8 Ma, and together are sister to the remainder of the *salvini* group (Prebus, 2021). The type specimens came from a nest in a dead stick, and additional specimens have been collected from leaf litter extractions.

***Temnothorax longicaulis* stat. nov., nom. nov.**

**Figure 157:**
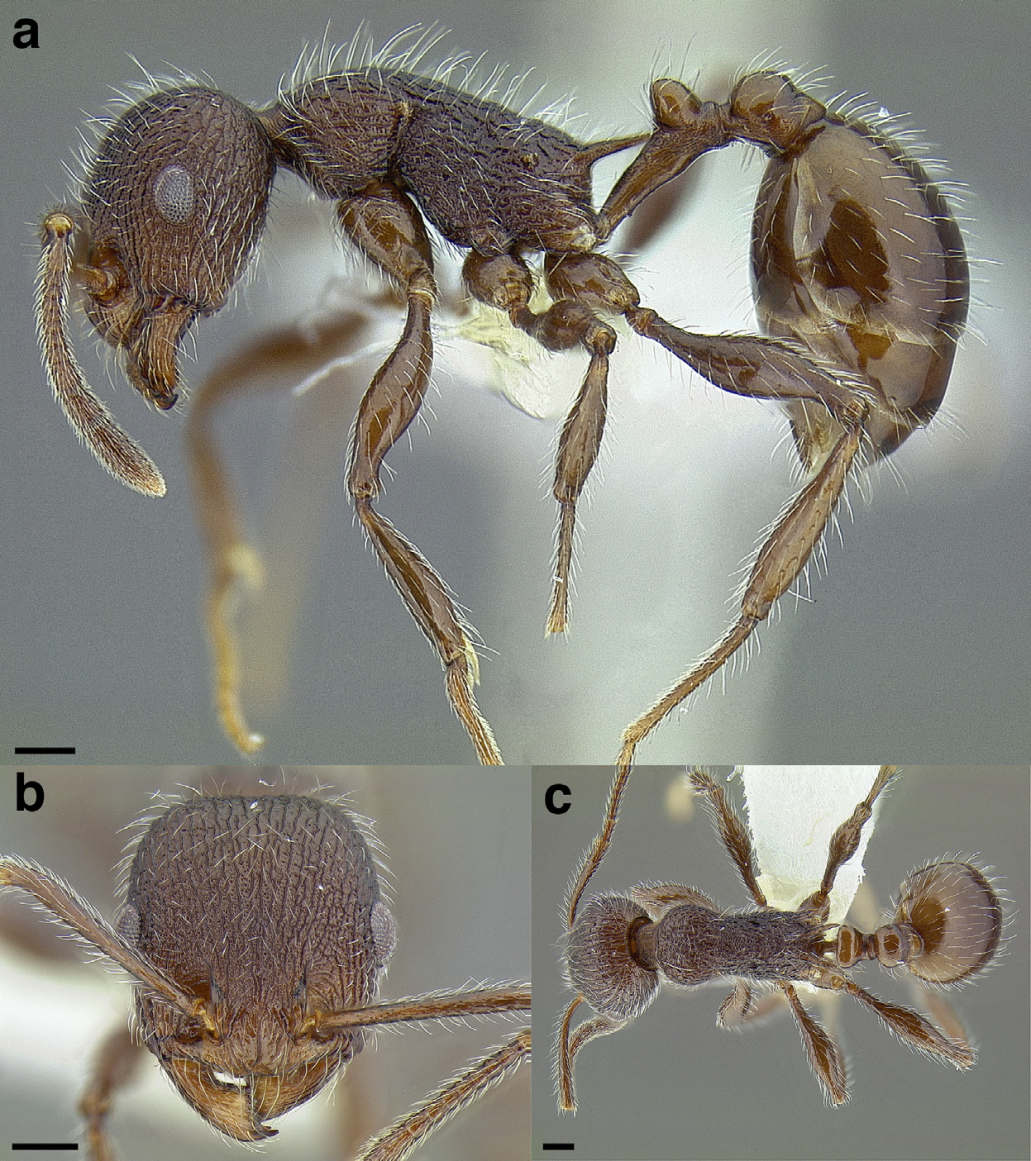
*Temnothorax longicaulis* stat. nov., nom. nov. neotype worker (CASENT0758334). (A) Profile view. (B) Full-face view. (C) Dorsal view. Scale bars 0.2 mm.

Distribution: Fig. 153D; worker: Fig. 157.

*Macromischa salvini obscurior* Forel, 1899: 57. Syntype workers. Volcan de Chiriqui, Panama. Types assumed lost. One worker here designated neotype.

*Leptothorax salvini obscurior* (Forel): Baroni Urbani, 1978: 495. First combination in *Leptothorax*. Junior secondary homonym of *Temnothorax tuberum obscurior* Dalla Torre, 1893: 128.

*Temnothorax salvini* grp 6, Prebus, 2021: 12. In phylogeny.

**Type material examined:** *Neotype worker:* COSTA RICA: Puntarenas: 4 km SSE San Vito, 8.783333°N 82.966667°W, 1,200 m, 18 March 1990, P.S. Ward#10564-2, second-growth rainforest, on low vegetation (CASENT0758334, top specimen on pin) [MHNG].

*Paraneotype worker:* same pin as neotype (bottom specimen on pin) [MHNG].

**Non-type material examined:** COSTA RICA: Puntarenas: 5 km S San Vito, 8.78333°N 82.96667°W ± 2,000 m, 1,200 m, 22–26 August 2010, Pollet & DeBraekeleer #12811, montane wet forest, ex pan trap, 1 worker (CASENT0615221) [JTLC]; 4 km SSE San Vito, 8.783333°N 82.966667°W, 1,200 m, 18 March 1990, P.S. Ward#10571, edge of second-growth rainforest, ex dead twig of *Cassia*, 3 workers (CASENT0010847) [UCDC]; same data as previous, except: 27 March 1990, P.S. Ward#10658-2, rainforest edge, on low vegetation, 1 worker (CASENT0758335) [UCDC]. San José: Cerro Plano, 9.48561°N 83.96196°N ± 20 m, 970 m, 6 July 2015, Irene Calderon#Go-E-06-1-01, pasture/cloud forest edge, beating vegetation, 1 worker (CASENT0758740) [UCDC] 1 worker (CASENT0631383) [JTLC]; Ranchos Tinamú, 9.48574°N 83.96119°W ± 110 m, 950 m, 4 July 2015, P.S. Ward #PSW17398-23, rainforest edge, on low vegetation, 1 worker (CASENT0758671) [UCDC].

**Geographic range:** Low-to-mid elevations of Costa Rica (Fig. 153D).

**Worker diagnosis:** *Temnothorax longicaulis* stat. nov., nom. nov. can be separated from all other species in the *salvini* clade by the following character combination: dorsum of mesosoma very weakly sinuate; metanotal groove not impressed; propodeum not strongly depressed below the level of the promesonotum; propodeal spines present and longer than the propodeal declivity; subpetiolar tooth minute and triangular; petiolar node strongly squamiform: in dorsal view, petiolar node greater than or equal to 1.6 times as broad as caudal cylinder; setae on head, mesosoma, legs, waist segments and gaster erect to suberect, long, abundant and tapering; integument predominantly dark brown.

**Similar species:** Fellow members of the *salvini* group. *Temnothorax longicaulis* stat. nov., nom. nov. can be separated from other members of the *salvini* group by the strongly squamiform petiolar node (petiolar node less than or equal to 1.5 times as broad as the caudal cylinder in *T. longinoi* sp. nov., *T. quetzal* sp. nov., *T. fortispinosus* sp. nov., *T. parvidentatus* sp. nov., and *T. salvini*), dark brown integument (*T. aztecus, T. aztecoides* sp. nov., *T. paraztecus* sp. nov., *T. parvidentatus* sp. nov., and *T. quetzal* sp. nov. are variously colored, but never uniformly dark brown), relatively small subpetiolar tooth (longer than the setae that arises directly above it in *T. aztecus, T. longinoi* sp. nov., and *T. paraztecus* sp. nov.), and long propodeal spines (shorter than the propodeal declivity in *T. salvini*).

**Worker measurements & indices (*n* = 6)**: SL = 0.737–0.845 (0.797); FRS = 0.257–0.308 (0.286); CW = 0.805–0.903 (0.875); CWb = 0.714–0.785 (0.768); PoOC = 0.316–0.340 (0.325); CL = 0.824–0.897 (0.875); EL = 0.210–0.233 (0.226); EW = 0.136–0.163 (0.153); MD = 0.198–0.235 (0.211); WL = 1.081–1.231 (1.189); SPST = 0.340–0.433 (0.390); MPST = 0.332–0.376 (0.355); PEL = 0.391–0.505 (0.463); NOL = 0.259–0.291 (0.276); NOH = 0.129–0.167 (0.154); PEH = 0.249–0.285 (0.275); PPL = 0.208–0.261 (0.241); PPH = 0.244–0.281 (0.271); PW = 0.510–0.590 (0.558); SBPA = 0.200–0.233 (0.217); SPTI = 0.257–0.339 (0.295); PEW = 0.156–0.182 (0.173); PNW = 0.266–0.334 (0.308); PPW = 0.257–0.310 (0.290); HFL = 0.858–0.997 (0.939); HFWmax = 0.180–0.203 (0.195); HFWmin = 0.054–0.073 (0.068); CS = 1.126–1.232 (1.206); ES = 0.278–0.312 (0.303); SI = 101–108 (104); OI = 25; CI = 87–89 (88); WLI = 151–157 (155); SBI = 26–30 (28); PSI = 31–35 (33); PWI = 160–172 (167); PLI = 179–215 (193); NI = 162–201 (180); PNWI = 164–193 (178); NLI = 53–66 (60); FI = 270–333 (290).

**Worker description:** In full-face view, head subquadrate, longer than broad (CI 87–89). Mandibles finely striate but shining and armed with five teeth: the apical-most well developed and acute, followed by a less developed preapical tooth and three equally developed smaller teeth. Anterior clypeal margin flat medially. Antennal scapes moderately long: when fully retracted, surpassing the posterior margin of the head capsule by about half the maximum width of the antennal scape (SI 101–108). Antennae 12-segmented; antennal club of composed of three segments, with the apical-most segment about one and a half times as long as the preceding two in combination. Frontal carinae short: extending past the antennal toruli by about one and a half times the maximum width of the antennal scape. Compound eyes protruding past the lateral margins of the head capsule. Lateral margin of head weakly convex, forming a continuous arc from the mandibular insertions to the posterior margin of the head. Posterior head margin weakly concave medially but rounding evenly into the lateral margins.

In profile view, compound eyes ovular and moderately large (OI 25), with 15 ommatidia in longest row. Pronotal declivity indistinct, neck and anterior face of pronotum forming a ~120° angle. Mesosoma very weakly sinuate: promesonotum weakly convex from where it joins the pronotal neck to the weakly impressed mesometanotal sulcus; metanotum very weakly convex; propodeum slightly depressed below the level of the promesonotum, and very weakly convex. Promesonotal suture extending from the posterior margin of the procoxal insertion to the mesothoracic spiracle, which is well developed, then continuing dorsally as a weak sulcus. Metanotal groove visible as a disruption of the sculpture laterally from where it arises between the mid- and hind coxae to the poorly developed metathoracic spiracle, which is nearly indistinguishable against the ground sculpture; continuing dorsally as a faint impression to the anterior margin of the metanotum. Propodeal spiracle well developed, directed posterolaterally, and separated from the propodeal declivity by about three and a half spiracle diameters. Propodeal spines moderately well developed and moderately long (PSI 31–35), about as long as the propodeal declivity, flared at the base, straight, and acute. Propodeal declivity weakly concave, forming a rounded ~95° angle with the base of the propodeal spines. Propodeal lobes rounded and weakly developed. Metapleural gland bulla small, extending from the metacoxal insertion halfway to the propodeal spiracle. Petiole moderately long (PLI 179–215), without tubercles anterodorsally. Subpetiolar process in the form of a minute, acute, triangular tooth; ventral margin of petiole weakly bulging posterior to it. Petiolar peduncle very long: comprising about three quarters the total length of the petiole. Petiolar node erect and squamiform: transition between peduncle and node marked by a rounded angle of ~130°; anterior face forming a sharp ~70° angle with the posterior face; posterior face forms a ~100° angle with the caudal cylinder. Postpetiole evenly rounded anterodorsally, bulging before flattening posterodorsally; concave ventrally.

In dorsal view, humeri developed and distinct: evenly rounded and wider than the rest of the mesosoma; mesothoracic spiracles weakly protruding past the lateral margins of the mesosoma, visible as slight angles where the pronotum meets the mesonotum. Promesonotal suture not visible. Metanotum delineated anteriorly and posteriorly bye weak sulci and a disruption in ground sculpture. Propodeal spines narrowly approximated basally and weakly diverging apically, their apices separated from each other by about their length, the negative space between them “U” shaped. Petiolar peduncle with spiracles weakly protruding past the lateral margins. Petiolar node, when viewed posterodorsally, trapezoidal: broader apically than basally; node broader than the peduncle and the caudal cylinder. Postpetiole narrow (PWI 160–172) and campaniform. Anterior margin of the postpetiole convex and evenly rounds into the lateral margins; lateral margins parallel to each other; posterior corners narrowly rounded; posterior margin flat. Metafemur moderately to strongly incrassate (FI 270–333).

Sculpture: median clypeal carina present, extending posteriorly nearly to the frontal triangle, and flanked on either side by three irregular carinae. Lateral clypeal lobes with additional, weaker carinae; ground sculpture weakly areolate. Antennal scapes finely areolate. Cephalic dorsum densely areolate, with coarse rugose sculpture overlying the ground sculpture. Lateral surfaces of head sculptured similarly to the dorsum. Ventral surface of head shining, with weaker areolae and costulae. Pronotal neck areolate. Anterior face of pronotum coarsely areolate; lateral face coarsely costate. Lateral face of meso- and metapleurae coarsely rugose over weak areolate sculpture. Propodeal declivity finely strigulate. Dorsal surface of mesosoma weakly areolate, with rugose sculpture overlying the ground sculpture. Femora weakly areolate. Petiole predominantly weakly areolate, with anterior and posterior faces of node shining through very weak areolate sculpture. Postpetiole predominantly smooth and shining, with ventrolateral surface and posterior quarter finely areolate. First gastral tergite and sternite smooth and shining, without spectral iridescence.

Setae: antennal scapes and funiculi with moderately long, subdecumbent pilosity. Dorsum of the head, pronotum, waist segments, and gaster with abundant, suberect, tapering, flexuous setae, the longest of which are slightly longer than the length of the compound eye and are directed toward the midline of the body. The head bears ~40, mesosoma ~40, petiole ~6, postpetiole ~18, and first gastral tergite ~44 setae. Pubescence present over the entire body, which is nearly as long as the setae.

Color: head and mesosoma predominantly dark brown. Antennae, mandibles, pronotal neck, legs, and basal two thirds of gastral sclerites (excluding the first gastral sclerites) testaceous. Sting testaceous yellow.

**Gyne:** Unknown.

**Male:** Unknown.

**Etymology:** Replacement name for *Temnothorax obscurior*, which is occupied by a species from North Africa. From the Latin ‘longus’ (=long) + ‘caulis’ (=stalk or stem of plant), in reference to the elongate petiolar peduncle of this species.

**Comments:** *Temnothorax longicaulis* stat. nov., nom. nov. is known from several collections from low-to-mid elevation wet forests in southern Mesoamerica. Workers have been found several times foraging on low vegetation. *Temnothorax longicaulis* stat. nov., nom. nov. is apparently restricted to the Pacific slope of the southern Central American cordilleras, where it may be found sympatrically with *T. salvini*. This latter species is also restricted to southern Central America, but its range also includes the Caribbean slope, and has a generally higher elevational range (>1,500 m vs. <1,300 m, Figs. 153D & 153I). *Temnothorax longicaulis* stat. nov., nom. nov. and *T. salvini* are sister species, having shared a common ancestor around 5 Ma which had dispersed to the southern Central American cordilleras from the Central American Nucleus during a mountain building phase in southern Central America during the Miocene-Pliocene transition (Prebus, 2021). These two species together are closely related to the morphologically similar *T. longinoi* sp. nov., which is restricted to the Central American Nucleus. *Temnothorax longicaulis* stat. nov., nom. nov., like its close relatives in the *salvini* species group, is arboreally nesting and foraging.

***Temnothorax longinoi* sp. nov.**

**Figure 158:**
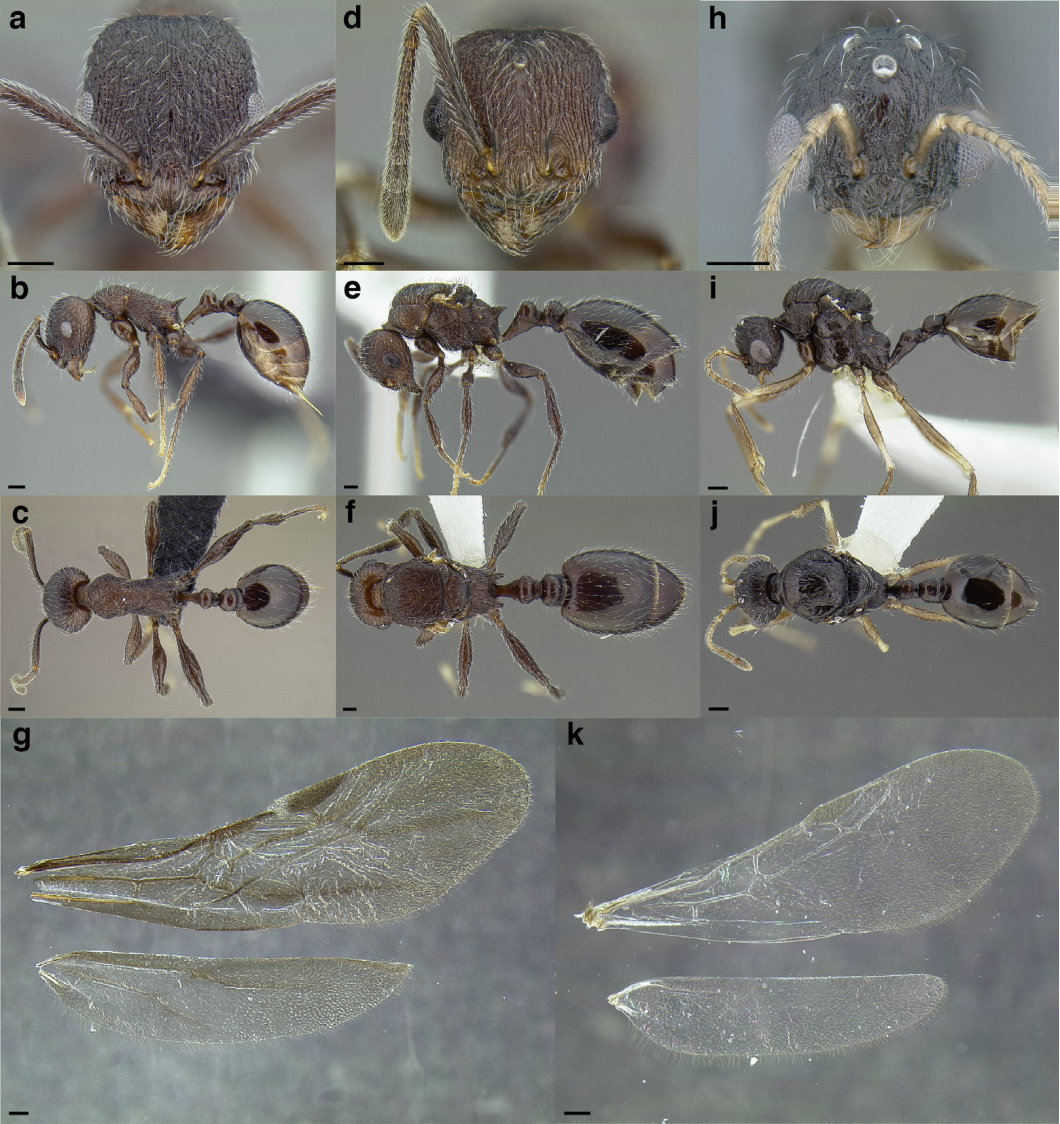
*Temnothorax longinoi* sp. nov. (A–C) Holotype worker (CASENT0758892). (A) Full-face view. (B) Profile view. (C) Dorsal view. (D–G) Paratype gyne (CASENT0619354). (D) Full-face view. (E) Profile view. (F) Dorsal view. (G) Wings. (H–K) Paratype male (CASENT0619355). (H) Full-face view. (I) Profile view. (J) Dorsal view. (K) Wings. Scale bars 0.2 mm.

**Figure 159:**
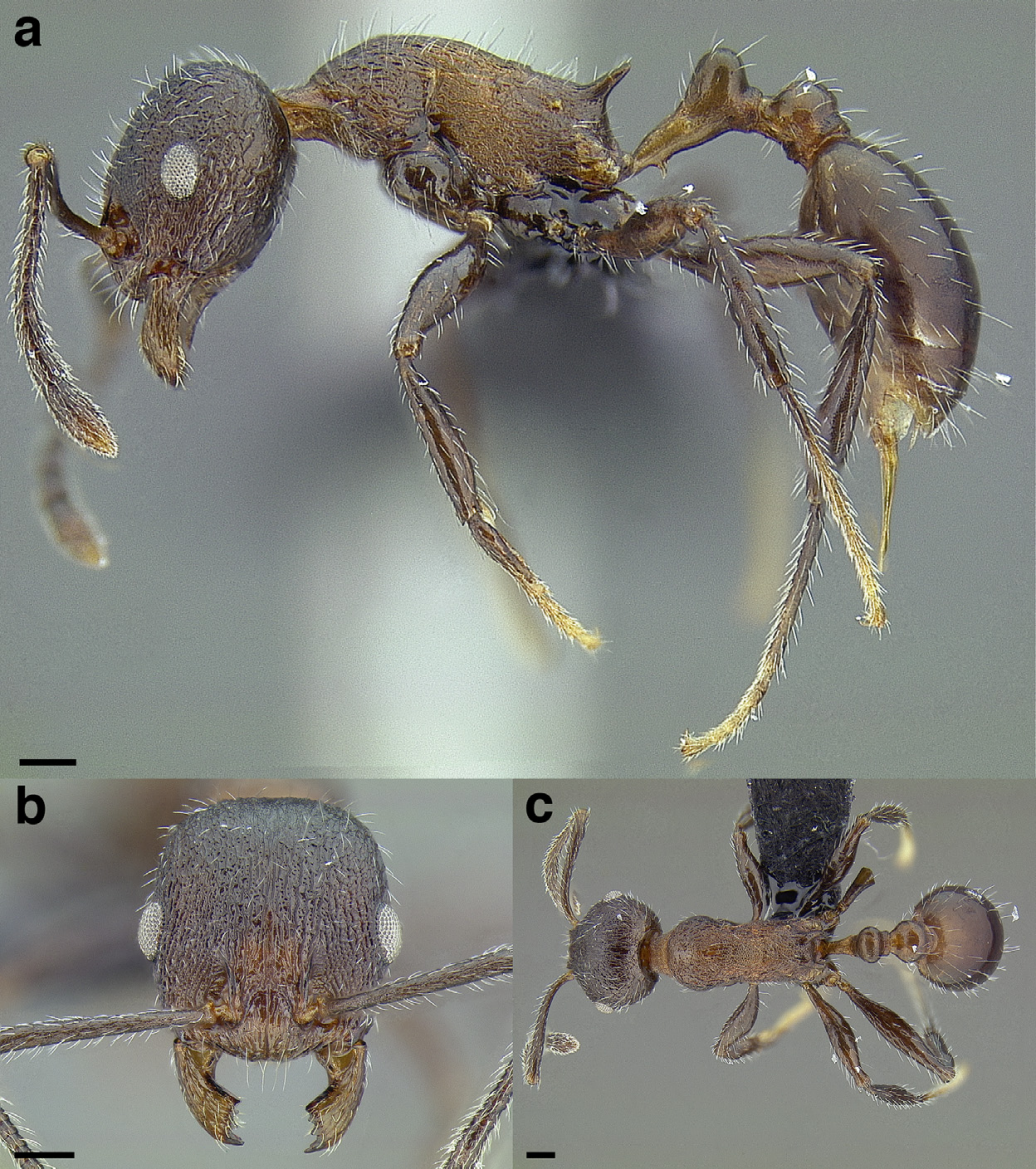
Bicolored morphological variant worker of *Temnothorax longinoi* sp. nov. Comayagua, Honduras (CASENT0615331). (A) Profile view. (B) Full-face view. (C) Dorsal view. Scale bars 0.2 mm.

Distribution: Fig. 153E; worker, gyne & male: Fig. 158; variability: Fig. 159.

*Temnothorax salvini* (Forel): Prebus, 2017: 7 (misidentification). In phylogeny.

*Temnothorax salvini* grp 5, Prebus, 2021: 12. In phylogeny.

**Type material examined:** *Holotype worker:* NICARAGUA: Nueva Segovia: 14 km NNE Ocotal, 13.75412°N 86.42565°W ± 20 m, 1,860 m, 24 April 2011, J. Longino#JTL7413, low 2nd growth vegetation, nest in dead stem (CASENT0758892) [CASC].

*Paratype workers, male, and gyne:* same data as holotype, 1 worker (CASENT0758893) [CASC] 1 worker (CASENT0619621) [USNM] 1 male (CASENT0758894) [CASC] 1 male (CASENT0619355) [UCDC] 1 alate gyne (CASENT0619354) [CASC].

**Non-type material examined:** GUATEMALA: Zacapa: 2 km SE La Unión, 14.94654°N 89.27600°W ± 105 m, 1,550 m, 13 May 2009, LLAMA#Go-B-03-1-03, cloud forest, beating vegetation, 1 worker (CASENT0614501) [UCDC]; same data as previous, except: 14.95359°N 89.27686°W ± 108 m, 1,430 m, LLAMA#Go-B-03-2-05, 1 worker (CASENT0614503) [JTLC] 1 worker (CASENT0758695) [UCDC].

HONDURAS: Comayagua: 9 km E Comayagua, 14.44776°N 87.55019°W ± 60 m, 1,740 m, 17 May 2010, LLAMA#Ba-C-03-3-03-09, secondary pine-oak-liquidambar forest, at bait, 1 worker (CASENT0616341) [JTLC]; same data as previous, except: 14.45971°N 87.54623°W ± 4 km, 1,990 m, 17 May 2010, B. Boudinot#BEB178, mesophyll forest, on vegetation, 1 worker (CASENT0615769) [JTLC] 1 worker (CASENT0615770) [JTLC]; same data as previous, except: 10 km E Comayagua, 14.45971°N 87.54623°W ± 100 m, 1,980 m, 16 May 2010, LLAMA#Go-C-03-1-01, cloud forest, beating vegetation, 1 worker (CASENT0615344) [JTLC]; same data as previous, except: 14.45975°N 87.5460°W ± 20 m, 2,000 m, 15 May 2010, LLAMA#Wa-C-03-1-03, cloud forest, ex sifted leaf litter, 1 worker (CASENT0615331) [UCDC]; same data as previous, except: 14.46049°N 87.54481°W ± 20 m, 2,000 m, 15 May 2010, LLAMA#Wa-C-03-1-39, cloud forest, ex sifted leaf litter, 1 dealate gyne (CASENT0615339) [JTLC]; Francisco Morazán: Reserva Biologia El Chile, 14.35174°N 86.84488°W ± 30 m, 1,500 m, 14 May 2009, R. Anderson#RSA2009-030, cloud forest, ex sifted leaf litter, 1 worker (CASENT0611710) [UCDC]; Olancho: 10 km N Catacamas, 14.94402°N 85.91081°W ± 75 m, 1,670 m, 9 May 2010, LLAMA#Go-C-02-1-02, cloud forest, beating vegetation, 1 worker (CASENT0614144) [UCDC] 1 worker (CASENT0614145) [JTLC]; same data as previous, except: 14.94204°N 85.90979°W ± 50 m, 1,590 m, 9 May 2010, LLAMA#Go-C-02-1-03, cloud forest, beating vegetation, 1 worker (CASENT0614146) [JTLC]; 11 km N Catacamas, 14.94838°N 85.91439°W ± 4 km, 2,000 m, 16 May 2010, B.E. Boudinot#BEB000164, 1 worker (TESC000008211) [UCDC] 1 worker (TESC000008213) [UCDC]; Parque Nacional La Muralla, 15.09658°N 86.74526°W ± 100 m, 1,450 m, 5 May 2010, LLAMA#Go-C-01-3-02, pine-mesophyll forest, beating vegetation, 1 dealate gyne (CASENT0615048) [JTLC]; same data as previous, except: 15.09685°N 86.73290°W ± 100 m, 1,630 m, 5 May 2010, LLAMA#Go-C-01-3-03, cloud forest w/pine, beating vegetation, 1 worker (CASENT0758696) [UCDC]; same data as previous, except: 15.09578°N 86.73738°W ± 50 m, 1,450 m, 2 May 2010, P.S. Ward#16325, montane rainforest edge, ex dead twig of vine, 1 worker (CASENT0758331) [UCDC] 1 worker (CASENT0625203) [UCDC]; same data as previous, except: P.S. Ward#16326-02, on low vegetation, montane rainforest edge, 1 worker (CASENT0915982) [UCDC]; same data as previous, except: 15.09715°N 86.73859°W ± 50 m, 1,470 m, 2 May 2010, P.S. Ward#16329-03, on low vegetation, montane rainforest edge, 2 workers (CASENT0758353) [UCDC]; same data as previous, except: 15.09965°N 86.74072°W ± 20 m, 1,530 m, 2 May 2010, LLAMA#Wa-C-01-1-36, cloud forest, ex sifted leaf litter, 1 worker (CASENT0615009) [JTLC]; same data as previous, except: 15.09683°N 86.73008°W ± 30 m, 1,550 m, 3 May 2010, P.S. Ward#16337, ex dead twig, montane rainforest, 2 workers (CASENT0758332) [UCDC] 1 dealate gyne, 2 workers (CASENT0758354) [UCDC] 1 worker (CASENT0625204) [JTLC]; same data as previous, except: 15.09721°N 86.73840°W ± 30 m, 1,480 m, 4 May 2010, J. Longino#JTL6970, cloud forest clearing, forager, 1 worker (CASENT0615578) [JTLC]; same data as previous, except: 15.09557°N 86.73859°W ± 1 km, 1,500 m, 6 May 2010, B. Boudinot#BEB174, cloud forest, 1 worker (CASENT0615716) [JTLC]; Tegucigalpa: Parque Nacional La Tigra, 1,770 m, 24 May 1993, Rifkind & Gum, cloud forest, 1 worker (LACMENT323458) [LACM].

NICARAGUA: Nueva Segovia: 14 km NNE Ocotal, 13.75412°N 86.42565°W ± 20 m, 1,860 m, 24 April 2011, J. Longino#7413, low second growth vegetation, nest in dead stem, 1 worker (CASENT0619621) [JTLC]; same data as previous, except: 13.75449°N 86.42272°W ± 500 m, 1,945 m, 24 April 2011, J. Longino#JTL7417.1, pasture/cloud forest edge, strays, 1 worker (CASENT0619360) [JTLC]; same data as previous, except: 13.98118°N 86.18879°W ± 10 m, 1,625 m, 28 May 2011, LLAMA#Wa-D-06-1-12, oak cloud forest, ex sifted leaf litter, 1 worker (CASENT0629286) [JTLC]; Jinotega: RN Datanlí el Diablo, 13.10474°N 85.86748°W ± 10 m, 1,400 m, 18 May 2011, LLAMA#Wa-D-04-2-32, cloud forest, ex sifted leaf litter, 1 worker (CASENT0629203) [JTLC]; same data as previous, except: 13.10394°N 85.86829°W ± 10 m, 1,400 m, 18 May 2011, LLAMA#Wa-D-04-2-07, cloud forest, ex sifted leaf litter, 1 worker (CASENT0629194) [JTLC]; RN Cerro Kilambé, 1357023°N 85.69737°W ± 20 m, 1,500 m, 23 May 2011, LLAMA#Wa-D-05-1-25, cloud forest, ex sifted leaf litter, 1 worker (CASENT0629233) [JTLC]; 13.98068°N 86.18874°W ± 100 m, 1,500 m, 24 May 2011, LLAMA#Go-D-06-1-01, cloud forest, beating vegetation, 1 worker (CASENT0732601) [UCDC]; same data as previous, except: 13.56751°N 85.69672°W ± 100 m, 1,430 m, 25 May 2011, LLAMA#Go-D-05-4-02, cloud forest, beating vegetation, 1 worker (CASENT0629261) [JTLC].

**Geographic range:** Mid-to-high elevations, Guatemala to Nicaragua (Fig. 153E).

**Worker diagnosis:** *Temnothorax longinoi* sp. nov. can be separated from all other species in the *salvini* clade by the following character combination: dorsum of mesosoma flat; metanotal groove not impressed; propodeum not strongly depressed below the level of the promesonotum; propodeal spines straight and about as long as the propodeal declivity; subpetiolar tooth small long and acute: longer than the setae that arise from the peduncle directly above; petiolar node weakly squamiform: in dorsal view, petiolar node less than or equal to 1.6 times as broad as caudal cylinder; setae on head, mesosoma, legs, waist segments and gaster erect to suberect, long, abundant and tapering; integument uniformly dark brown to weakly bicolored; bicolored form with clypeus, mandibles, mesosoma, and waist segments testaceous brown.

**Similar species:** Fellow members of the *salvini* group. *Temnothorax longinoi* sp. nov. can be separated from other members of the *salvini* group by the weakly squamiform petiolar node (petiolar node more than or equal to 1.6 times as broad as the caudal cylinder in *T. aztecus, T. aztecoides* sp. nov., *T. longicaulis* stat. nov., nom. nov., and *T. paraztecus* sp. nov.), relatively large subpetiolar tooth (shorter than the setae that arises from the dorsum of the petiolar peduncle directly above it in *T. quetzal* sp. nov., *T. fortispinosus* sp. nov., *T. parvidentatus* sp. nov., and *T. salvini*), and short propodeal spines (as long as or longer than the propodeal declivity in *T. quetzal* sp. nov., *T. aztecoides* sp. nov., and *T. aztecus*).

**Worker measurements & indices (*n* = 18):** SL = 0.738–0.869 (0.814); FRS = 0.247–0.292 (0.270); CW = 0.795–0.933 (0.876); CWb = 0.703–0.834 (0.783); PoOC = 0.310–0.358 (0.333); CL = 0.782–0.927 (0.874); EL = 0.189–0.244 (0.228); EW = 0.142–0.169 (0.158); MD = 0.178–0.249 (0.204); WL = 1.012–1.281 (1.164); SPST = 0.267–0.355 (0.325); MPST = 0.293–0.359 (0.332); PEL = 0.359–0.477 (0.433); NOL = 0.217–0.291 (0.260); NOH = 0.118–0.188 (0.159); PEH = 0.230–0.309 (0.272); PPL = 0.185–0.252 (0.224); PPH = 0.223–0.307 (0.275); PW = 0.469–0.606 (0.549); SBPA = 0.180–0.264 (0.230); SPTI = 0.216–0.306 (0.268); PEW = 0.145–0.193 (0.177); PNW = 0.208–0.301 (0.262); PPW = 0.234–0.330 (0.288); HFL = 0.808–0.999 (0.930); HFWmax = 0.160–0.217 (0.185); HFWmin = 0.050–0.071 (0.063); CS = 1.103–1.298 (1.220); ES = 0.262–0.321 (0.307); SI = 99–109 (104); OI = 24–27 (25); CI = 84–94 (90); WLI = 142–157 (149); SBI = 25–33 (29); PSI = 26–31 (28); PWI = 152–176 (163); PLI = 166–216 (194); NI = 139–198 (165); PNWI = 141–161 (148); NLI = 53–64 (60); FI = 262–326 (294).

**Worker description:** In full-face view, head subquadrate, longer than broad (CI 84–94). Antennal scapes very long: when fully retracted, surpassing the posterior margin of the head capsule by about three times the maximum width of the antennal scape (SI 99–109). Antennae 12-segmented; antennal club of composed of three segments, with the apical-most segment about as long as the preceding two in combination. Frontal carinae short: extending past the antennal toruli by about one and a half times the maximum width of the antennal scape. Compound eyes moderately protruding past the lateral margins of the head capsule. Lateral margin of head weakly convex, forming a continuous arc from the mandibular insertions to the posterior margin of the head. Posterior head margin weakly concave medially but rounding evenly into the lateral margins.

In profile view, compound eyes ovular and moderately large (OI 24–27), with 14 ommatidia in longest row. Pronotal declivity indistinct, neck and anterior face of pronotum forming a ~110° angle. Mesosoma very weakly sinuate: promesonotum weakly convex from where it joins the pronotal neck to the weakly impressed mesometanotal sulcus; metanotum weakly convex; propodeum slightly depressed below the mesonotum, and flat to the base of the propodeal spines. Promesonotal suture extending from the posterior margin of the procoxal insertion to the mesothoracic spiracle, which is well developed, then continuing dorsally as a weak sulcus. Metanotal groove visible as a disruption of the sculpture laterally from where it arises between the mid- and hind coxae to the poorly developed metathoracic spiracle, which is nearly indistinguishable against the ground sculpture; continuing dorsally as a faint impression to the anterior margin of the metanotum. Propodeal spiracle well developed, directed posterolaterally, and separated from the propodeal declivity by about four spiracle diameters. Propodeal spines moderately well developed and moderately long (PSI 26–31), about as long as the propodeal declivity, flared at the base, straight, and acute. Propodeal declivity flat, forming a rounded ~120° angle with the base of the propodeal spines. Propodeal lobes rounded and weakly developed. Metapleural gland bulla moderately large, extending from the metacoxal insertion two thirds of the way to the propodeal spiracle. Petiole moderately long (PLI 166–216), without tubercles anterodorsally. Subpetiolar process in the form of a moderately long, anteriorly curved, acute spiniform tooth; ventral margin of petiole weakly bulging posterior to it. Petiolar peduncle moderately long: comprising about half of the total length of the petiole. Petiolar node erect and rounded dorsally: transition between peduncle and node marked by a rounded angle of ~140°, resulting in a weakly concave anterior node face; anterior face rounding evenly into the dorsal face, which is evenly convex; dorsal face rounding evenly into the posterior face, which forms a ~100° angle with the caudal cylinder. Postpetiole evenly rounded anterodorsally, bulging before flattening posterodorsally; concave ventrally.

In dorsal view, humeri developed and distinct: evenly rounded and wider than the rest of the mesosoma; mesothoracic spiracles weakly protruding past the lateral margins of the mesosoma, visible as slight angles where the pronotum meets the mesonotum. Promesonotal suture visible as a faint sulcus and disruption in the ground sculpture. Metanotum delineated anteriorly and posteriorly by weak sulci and disruption in the ground sculpture. Propodeal spines narrowly approximated basally and diverging apically, their apices separated from each other by about their length, the negative space between them “U” shaped. Petiolar peduncle with spiracles protruding past the lateral margins, but not noticeably constricted anterior to them. Petiolar node, when viewed posterodorsally, rounded-trapezoidal and weakly squamiform: about one and a half times as broad apically as the base; node broader than the peduncle and the caudal cylinder. Postpetiole narrow (PWI 152–176) and subquadrate. Anterior margin of the postpetiole flat but evenly rounding into the lateral margins; lateral margins parallel to each other; posterior corners angulate; posterior margin flat. Metafemur moderately to strongly incrassate (FI 262–326).

Sculpture: median clypeal carina present but indistinct, extending posteriorly nearly to the frontal triangle, and flanked on either side by three indistinct carinae. Lateral clypeal lobes with additional, weaker carinae; ground sculpture weakly areolate. Antennal scapes finely areolate. Cephalic dorsum finely areolate, with predominantly rugose-costate sculpture overlying the ground sculpture; concentric costulae surrounding the base of the antennal insertions. Lateral surfaces of head sculptured similarly to the dorsum. Ventral surface of head shining, with shallow areolae, and weak costulae. Pronotal neck areolate. Lateral face of pronotum weakly areolate, with coarse costae. Lateral face of pronotum, meso- and metapleuron coarsely rugose over areolate sculpture. Propodeal declivity weakly areolate, with strigulae. Dorsal surface of mesosoma weakly areolate, with costate sculpture overlying the ground sculpture, becoming rugose on the pronotum and propodeum. Femora shallowly areolate. Dorsum of the petiolar peduncle, anterior, dorsal and posterior faces of the petiolar node, and base of the lateral face of the node weakly areolate; otherwise shining. Postpetiole with lateral faces and posterodorsal quarter shallowly areolate; otherwise shining. First gastral tergite and sternite smooth and shining, with weak spectral iridescence.

Setae: antennal scapes and funiculi with moderately long, subdecumbent pilosity. Dorsum of the head, pronotum, waist segments, and gaster with abundant, suberect, tapering, flexuous setae, the longest of which are slightly longer than the length of the compound eye and are directed toward the midline of the body. The head bears > 80, mesosoma ~80, petiole 22, postpetiole ~30, and first gastral tergite > 80 setae. Pubescence present over the entire body, which is nearly as long as the setae.

Color: head and mesosoma predominantly testaceous brown. Dorsum of head, postpetiole and first gastral sclerites dark brown. Mandibles, tarsi, basal two thirds of remaining gastral sclerites, and sting testaceous yellow.

**Gyne measurements & indices (*n* = 2):** SL = 0.770–0.819 (0.795); FRS = 0.291–0.298 (0.295); CW = 0.974–1.001 (0.988); CWb = 0.873–0.879 (0.876); PoOC = 0.326–0.348 (0.337); CL = 0.907–0.917 (0.912); EL = 0.271–0.281 (0.276); EW = 0.199–0.205 (0.202); MD = 0.210–0.212 (0.211); WL = 1.646–1.704 (1.675); SPST = 0.343–0.377 (0.360); MPST = 0.424–0.440 (0.432); PEL = 0.541–0.598 (0.570); NOL = 0.304–0.333 (0.319); NOH = 0.204–0.208 (0.206); PEH = 0.353–0.395 (0.374); PPL = 0.281–0.292 (0.287); PPH = 0.417–0.439 (0.428); PW = 0.922–1.006 (0.964); SBPA = 0.430–0.447 (0.439); SPTI = 0.401–0.429 (0.415); PEW = 0.238–0.240 (0.239); PNW = 0.345–0.394 (0.370); PPW = 0.412–0.515 (0.464); HFL = 1.033–1.043 (1.038); HFWmax = 0.181–0.187 (0.184); HFWmin = 0.063–0.077 (0.070); CS = 1.332–1.333 (1.332); ES = 0.371–0.384 (0.377); SI = 88–94 (91); OI = 28–29 (28); CI = 95–97 (96); WLI = 187–195 (191); SBI = 49–51 (50); PSI = 20–23 (22); PWI = 172–216 (194); PLI = 193–205 (199); NI = 149–160 (155); PNWI = 144–166 (155); NLI = 56–56 (56); FI = 243–287 (265).

**Gyne description:** In full-face view, head subquadrate, slightly longer than broad (CI 95–97). Mandibles weakly striate, shining and armed with five teeth: the apical-most well developed, followed by a less developed preapical tooth and three equally developed smaller teeth. Anterior clypeal margin flat medially. Antennal scapes very long: surpassing the posterior margin of the head capsule by about two times the maximum width of the antennal scape (SI 88–94). Antennae 12-segmented; antennal club composed of three segments, with the apical-most segment as long as the preceding two in combination. Frontal carinae moderately long: extending past the antennal toruli by about two times the maximum width of the antennal scape. Compound eyes moderately protruding past the lateral margins of the head capsule. Lateral margin of head evenly convex, converging from below the compound eyes to the mandibular insertions. Posterior head margin weakly concave but rounding evenly into the lateral margins.

In profile view, compound eyes ovular and moderately large (OI 28–29), with 20 ommatidia in longest row. Mesoscutum rounded evenly anteriorly, not fully covering the dorsal surface of the pronotum, and flat dorsally. Mesoscutellum on the same plane as the mesoscutum, but rounded posteriorly. Posterior margin of metanotum extending slightly beyond the posterior margin of the mesoscutum. Propodeal spiracle well developed, directed posterolaterally, and separated from the propodeal declivity by about four spiracle diameters. Propodeal spines stout and well developed, but short (PSI 20–23), about as two thirds as long as the propodeal declivity, tapering evenly from the base, directed posteriorly, straight, and acute. Propodeal declivity weakly concave, forming a rounded ~110° angle with the base of the propodeal spines. Propodeal lobes rounded and weakly developed. Metapleural gland bulla moderately large, extending from the metacoxal insertion two thirds of the way to the propodeal spiracle. Petiole moderately long (PLI 193–205), without tubercles anterodorsally. Subpetiolar process in the form of a moderately long, anteriorly curved, acute spiniform tooth; ventral margin of petiole weakly bulging posterior to it. Petiolar peduncle moderately long: comprising about half of the total petiole length. Petiolar node erect and rounded dorsally: transition between peduncle and node marked by a rounded angle of ~140°, resulting in a weakly concave anterior node face; anterior face rounding evenly into the dorsal face, which is evenly convex; dorsal face rounding evenly into the posterior face, which forms a ~100° angle with the caudal cylinder. Postpetiole evenly rounded anterodorsally, bulging before flattening posterodorsally; concave ventrally.

In dorsal view, mesoscutum not fully covering pronotum anteriorly; humeri visible laterally as rounded sclerites. Propodeal spines weakly diverging apically, their apices separated from each other by about twice their length. Petiolar peduncle with spiracles protruding past the lateral margins, but not noticeably constricted anterior to them. Petiolar node, when viewed posterodorsally, rounded-trapezoidal and weakly squamiform: about one and a half times as broad apically as the base; node broader than the peduncle and the caudal cylinder. Postpetiole narrow (PWI 172–216) and subquadrate. Anterior margin of postpetiole nearly flat, with corners evenly rounding into the lateral margins; lateral margins parallel to each other; posterior corners angulate; posterior margin flat. Metafemur weakly to moderately incrassate (FI 243–287).

Sculpture: median clypeal carina present, extending from the anterior margin nearly to frontal triangle, and flanked on either side by three equally strong carinae. Lateral clypeal lobes with additional weaker carinae; ground sculpture weakly areolate. Antennal scapes finely areolate. Cephalic dorsum finely areolate, with rugose-costate sculpture overlying the ground sculpture; antennal insertions surrounded by concentric costulae. Lateral surfaces of head sculptured similarly to the dorsum; rugae becoming stronger between the compound eye and mandibular insertion. Ventral surface of head with weak areolae and weak costulae. Pronotal neck areolate-strigulate. Pronotum weakly areolate anteriorly and costate laterally. Anterior half of anepisternum weakly areolate, with fine costulae on the posterior half. Katepisternum coarsely rugose. Metapleuron and lateral face of propodeum with coarse costae. Propodeal declivity weakly areolate, with fine costulae. Mesoscutum with costae over areolate ground sculpture; a small patch of finely areolate sculpture anteromedially. Mesoscutellum costulate. Metanotum finely areolate. Dorsal face of propodeum coarsely rugose. Femora shallowly areolate. Dorsum of the petiolar peduncle, anterior, dorsal and posterior faces of the petiolar node, and base of the lateral face of the node shallowly areolate; otherwise shining. Postpetiole with lateral faces and posterodorsal quarter shallowly areolate; otherwise shining. First gastral tergite and sternite smooth and shining, with weak spectral iridescence.

Setae: antennal scapes and funiculi with moderately long, subdecumbent pilosity. Dorsum of the head, pronotum, waist segments, and gaster with abundant, suberect, tapering, flexuous setae, the longest of which are slightly longer than the width of the compound eye. Pubescence present over the entire body, which is nearly as long as the setae.

Color: head and mesosoma predominantly testaceous brown. Dorsum of head, postpetiole and first gastral sclerites dark brown. Mandibles, wing bases, and tarsi testaceous yellow.

**Male measurements & indices (*n* = 2):** SL = 0.241–0.248 (0.245); FRS = 0.116–0.143 (0.130); CW = 0.699–0.753 (0.726); CWb = 0.567–0.609 (0.588); PoOC = 0.244–0.255 (0.250); CL = 0.581–0.617 (0.599); EL = 0.267–0.303 (0.285); EW = 0.201–0.218 (0.210); MD = 0.053–0.056 (0.055); WL = 1.156–1.268 (1.212); SPST = n/a; MPST = 0.287–0.318 (0.303); PEL = 0.405–0.428 (0.417); NOL = 0.214–0.249 (0.232); NOH = 0.078–0.103 (0.091); PEH = 0.192–0.222 (0.207); PPL = 0.210–0.226 (0.218); PPH = 0.231–0.240 (0.236); PW = 0.774–0.786 (0.780); SBPA = n/a; SPTI = n/a; PEW = 0.166–0.172 (0.169); PNW = 0.215–0.232 (0.224); PPW = 0.263–0.268 (0.266); HFL = 0.961–1.032 (0.997); HFWmax = 0.109–0.112 (0.111); HFWmin = 0.060–0.066 (0.063); CS = 0.858–0.918 (0.888); ES = 0.368–0.412 (0.390); SI = 41–43 (42); OI = 43–45 (44); CI = 98–99 (98); WLI = 204–208 (206); SBI = n/a; PSI = n/a; PWI = 156–158 (157); PLI = 179–204 (192); NI = 208–319 (263); PNWI = 125–140 (132); NLI = 53–58 (56); FI = 170–182 (176).

**Male description:** In full-face view, head subovate, about as long as broad (CI 98–99). Mandibles shining through very weak striae and armed with five teeth: the apical-most well developed, followed by a smaller preapical tooth and three roughly equally developed smaller teeth. Anterior clypeal margin entire and flat. Antennal scapes short: when fully retracted, failing to reach the posterior margin of the head capsule by about the length of the scape (SI 41–43). Antennae 13-segmented; antennal club composed of four segments, with the apical-most segment slightly shorter than the preceding two in combination. Frontal carinae short, extending past the antennal toruli by about the maximum width of the antennal scape, but continuing on to the median ocellus as weak carinae. Compound eyes strongly protruding past the lateral margins of head capsule. Lateral margin of head convex, margin between the anterior margin of the compound eye and the mandibular insertions converging. Posterior head margin evenly convex, rounding evenly into the lateral margins.

In profile view, compound eyes ovular and large (OI 43–45), with 24 ommatidia in the longest row. Mesoscutum bulging anteriorly, fully covering the dorsal surface of the pronotum, convex dorsally. Mesoscutellum depressed slightly below the level of the mesoscutum, convex dorsally. Posterior margin of mesoscutellum extending beyond the posterior margin of the metanotum. Propodeum strongly depressed, with a declivitous face anteriorly and flat posteriorly; transitioning into the propodeal declivity through a broadly rounded ~95° angle. Propodeal spiracle moderately well developed, directed posterolaterally, and separated from the propodeal declivity by about five spiracle diameters. Propodeal spines absent. Propodeal lobes rounded and weakly developed. Metapleural gland bulla moderately large, extending two thirds of the way between metacoxal insertion and propodeal spiracle. Petiole moderately long (PLI 179–204), without tubercles anterodorsally. Subpetiolar process a minute, acute, triangular tooth; ventral margin of petiole bulging slightly posterior to it. Petiolar peduncle moderately long: comprising about half of the total petiole length. Petiolar node low and rounded dorsally. Postpetiole evenly rounded anterodorsally, bulging slightly before flattening dorsally; concave ventrally.

In dorsal view, mesoscutum fully covering pronotum anteriorly; humeri not visible laterally. Petiolar peduncle with spiracles strongly protruding past the lateral margins. Petiolar node narrower than the peduncle where the spiracles arise, but slightly broader than the caudal cylinder; rounded-trapezoidal when view at a posterodorsal aspect, the apex broader than the base. Postpetiole narrow (PWI 156–158) and campaniform. Anterior margin of postpetiole weakly convex, with the anterior corners evenly rounding into the lateral margins; lateral margins parallel to each other; posterior corners angulate; posterior margin of postpetiole flat. Metafemur not incrassate (FI 170–182).

Sculpture: median clypeal lobe with fine, concentric costulae. Antennal scapes shining through weak areolae. Dorsum of head areolate, with very fine costulae overlying the ground sculpture. Lateral surface of head areolate, with rugulae forming whorls around compound eye. Ventral surface of head shining, with weak areolae and rugulae. Pronotal neck weakly areolate. Anterior face of pronotum shining through weak areolate sculpture. Lateral surface of pronotum predominantly weakly areolate, with weak costulae overlying the ground sculpture. Katepisternum smooth and shining medially, with weak areolate sculpture on the anterior and posterior margins. Anepisternum smooth and shining on the anterior half, posterior half weakly areolate. Metapleuron and lateral face of propodeum densely areolate-rugulose. Propodeal declivity areolate. Dorsally, mesoscutum predominantly shining through weak, indistinct sculpture; Mayrian furrows surrounded by dense, irregular areolate sculpture. Mesoscutellum irregularly areolate costulate. Femora smooth and shining. Petiole predominantly weakly areolate, but dorsum of node shining and indistinctly sculptured. Dorsal surface of postpetiole shining medially, otherwise weakly areolate. First gastral tergite and sternite smooth and shining, without spectral iridescence.

Setae: antennal scapes and funiculi with moderately long, subdecumbent pilosity. Dorsum of the head, pronotum, waist segments, and gaster with abundant, suberect, tapering, flexuous setae, the longest of which are about as long as the width of the compound eye. Pubescence present over the entire body, which is nearly as long as the setae.

Color: head and mesosoma predominantly testaceous brown. Dorsum of head, postpetiole and first gastral sclerites dark brown. Mandibles, antennae, legs, and genitalia testaceous yellow.

**Etymology:** Patronym, in honor of Jack Longino, who collected the type specimens and whose initiatives were responsible for much of the material used in this study.

**Comments:** *Temnothorax longinoi* sp. nov. is common in mid-to-high elevation cloud forests of Mesoamerica between the Isthmus of Tehuantepec and the Nicaraguan depression (Fig. 153E). Like many of its close relatives in the *salvini* species group, it nests in hollow stems and forages arboreally. *Temnothorax longinoi* sp. nov. is morphologically similar to *T. longicaulis* stat. nov., nom. nov. and *T. salvini*. Together, these species are the sister group to *T. longinoi* sp. nov., having shared a common ancestor with it around 8 Ma before dispersing to the southern Central American cordilleras during the Miocene-Pliocene transition (Prebus, 2021). This species is morphologically uniform across its range, but a weakly bicolored form is known from Honduras (Fig. 159).

***Temnothorax paraztecus* sp. nov.**

**Figure 160:**
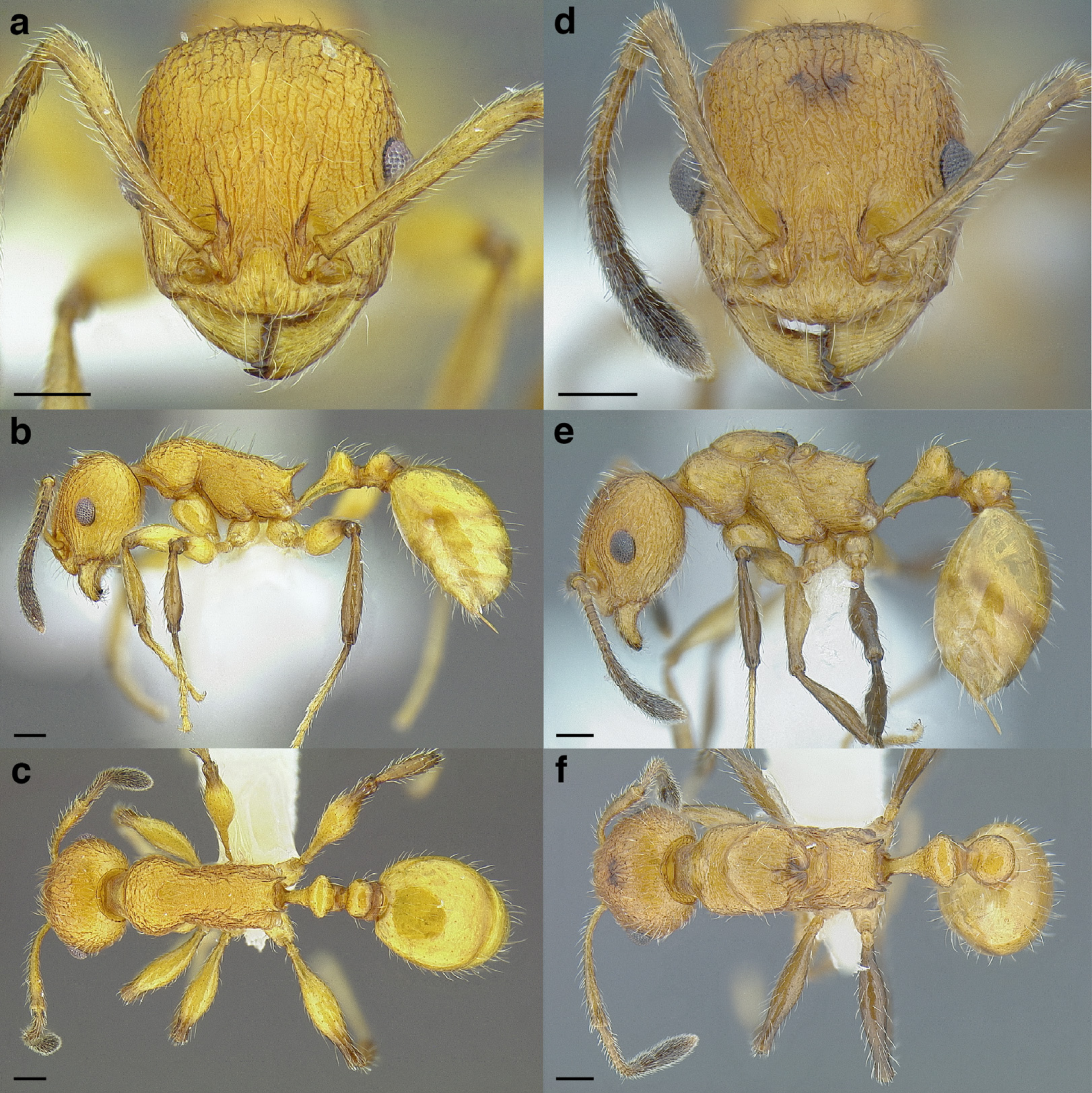
*Temnothorax paraztecus* sp. nov. (A–C) Holotype worker (CASENT0629030). (A) Full-face view. (B) Profile view. (C) Dorsal view. (D-F) Ergatogyne (CASENT0614500). (D) Full-face view. (E) Profile view. (F) Dorsal view. Scale bars 0.2 mm.

Distribution: Fig. 153F; worker & ergatogyne: Fig. 160.

*Temnothorax salvini* grp 7, Prebus, 2021: 12. In phylogeny.

**Type material examined:** *Holotype worker:* NICARAGUA: Jinotega: Parque Nacional Cerro Saslaya, 13.76880°N 85.02107°W ± 100 m, 1,110 m, 15 May 2011, LLAMA#Go-D-03-3-03, ridgetop cloud forest, beating vegetation (CASENT0629030) [CASC].

*Paratype worker:* same data as holotype, 1 worker (CASENT0758802) [UCDC] 1 worker (CASENT0629031) [MCZC].

**Non-type material examined:** GUATEMALA: Zacapa: 2 km SE La Unión, 14.94378°N 89.27747°W ± 104 m, 1,540 m, 13 May 2009, LLAMA#Go-B-03-1-01, cloud forest, beating vegetation, 1 ergatogyne (CASENT0614500) [JTLC] 1 worker (CASENT0614499) [UCDC].

HONDURAS: Comayagua: PN Cerro Azul Meambar, 14.87096°N 87.89920°W ± 20 m, 1,120 m, 20 May 2010, LLAMA#Wa-C-04-1-30, ridgetop cloud forest, ex sifted leaf litter, 1 worker (CASENT0617083) [JTLC].

MEXICO: Chiapas: Nahá, 16.96291°N 91.59335°W ± 300 m, 985 m, 10 June 2008, LLAMA#Go-A-07-1-02, mesophyll forest, beating vegetation, 1 worker (CASENT0608774) [JTLC].

**Geographic range:** Low-to-mid elevations, southern Mexico to Nicaragua (Fig. 153F).

**Worker diagnosis:** *Temnothorax paraztecus* sp. nov. can be separated from all other species in the *salvini* clade by the following character combination: smaller species: WL < 1.17 mm; dorsum of mesosoma weakly sinuate; metanotal groove not impressed; propodeum not strongly depressed below the level of the promesonotum; propodeal spines shorter than the propodeal declivity; subpetiolar tooth acutely spiniform and equal to or longer than the setae that arise from the peduncle directly above; petiolar node strongly squamiform: in dorsal view, petiolar node greater than or equal to 1.7 times as broad as caudal cylinder; dorsum of head rugose-costate; dorsum of petiolar node, postpetiole and entire first gastral tergite smooth and shining; setae on head, mesosoma, legs, waist segments and gaster erect to suberect, long, abundant and tapering; integument predominantly yellow.

**Similar species:** Fellow members of the *salvini* group. *Temnothorax paraztecus* sp. nov. can be separated from other members of the *salvini* group by the strongly squamiform petiolar node (petiolar node less than or equal to 1.5 times as broad as the caudal cylinder in *T. longinoi* sp. nov., *T. quetzal* sp. nov., *T. fortispinosus* sp. nov., *T. parvidentatus* sp. nov., and *T. salvini*), yellow integument (*T. longinoi* sp. nov., *T. longicaulis* stat. nov., nom. nov*., T. quetzal* sp. nov., *T. fortispinosus* sp. nov., *T. parvidentatus* sp. nov., and *T. salvini* are variously colored, but never uniformly yellow), smooth dorsum of the petiolar node, postpetiole, and gaster (lightly sculptured in *T. aztecoides* sp. nov.), relatively large subpetiolar tooth (shorter than the setae that arises directly above it in *T. aztecoides* sp. nov.), and short propodeal spines (longer than the propodeal declivity in *T. aztecus*).

**Worker measurements & indices (*n* = 4)**: SL = 0.715-0.758 (0.736); FRS = 0.233–0.256 (0.242); CW = 0.786–0.845 (0.823); CWb = 0.690–0.754 (0.728); PoOC = 0.302–0.323 (0.314); CL = 0.787–0.831 (0.811); EL = 0.187–0.205 (0.196); EW = 0.133–0.146 (0.139); MD = 0.198–0.218 (0.204); WL = 1.078–1.15 (1.107); SPST = 0.285–0.349 (0.311); MPST = 0.302–0.319 (0.312); PEL = 0.413–0.447 (0.428); NOL = 0.234–0.271 (0.256); NOH = 0.128–0.159 (0.145); PEH = 0.248–0.294 (0.270); PPL = 0.189–0.229 (0.211); PPH = 0.238–0.290 (0.264); PW = 0.511–0.529 (0.523); SBPA = 0.208–0.239 (0.229); SPTI = 0.218–0.292 (0.253); PEW = 0.156–0.179 (0.171); PNW = 0.285–0.308 (0.300); PPW = 0.247–0.276 (0.265); HFL = 0.822–0.863 (0.836); HFWmax = 0.190–0.206 (0.196); HFWmin = 0.059–0.067 (0.064); CS = 1.084–1.170 (1.133); ES = 0.257–0.273 (0.265); SI = 100–104 (101); OI = 22–24 (23); CI = 87–93 (90); WLI = 145–159 (152); SBI = 30–33 (31); PSI = 26–30 (28); PWI = 152–158 (155); PLI = 186–219 (205); NI = 101–183 (156); PNWI = 172–183 (175); NLI = 56–64 (60); FI = 292–322 (305).

**Worker description:** In full-face view, head subquadrate, longer than broad (CI 87–93). Mandibles finely striate but shining and armed with five teeth: the apical-most well developed and acute, followed by a less developed preapical tooth and three equally developed smaller teeth. Anterior clypeal margin weakly convex. Antennal scapes very long: when fully retracted, surpassing the posterior margin of the head capsule by about two times the maximum width of the antennal scape (SI 100–104). Antennae 12-segmented; antennal club of composed of three segments, with the apical-most segment about one and a half times as long as the preceding two in combination. Frontal carinae moderately long: extending past the antennal toruli by about two and a half times the maximum width of the antennal scape. Compound eyes protruding past the lateral margins of the head capsule. Lateral margin of head weakly convex, forming a continuous arc from the mandibular insertions to the posterior margin of the head. Posterior head margin weakly concave medially but rounding evenly into the lateral margins.

In profile view, compound eyes ovular and moderately large (OI 22–24), with 14 ommatidia in longest row. Pronotal declivity indistinct, neck and anterior face of pronotum forming a ~120° angle. Promesonotum weakly convex, with the remainder of the mesosoma weakly sinuate; metanotal groove faintly impressed; propodeum weakly convex to the base of the propodeal spines. Promesonotal suture extending from the posterior margin of the procoxal insertion to the mesothoracic spiracle, which is moderately well developed. Metanotal groove visible as a disruption of the sculpture laterally from where it arises between the mid- and hind coxae to the poorly developed metathoracic spiracle, which is nearly indistinguishable against the ground sculpture; continuing dorsally as a weak impression. Propodeal spiracle well developed, directed posterolaterally, and separated from the propodeal declivity by about four spiracle diameters. Propodeal spines moderately well developed, but short (PSI 26–30), about three quarters as long as the propodeal declivity, weakly upturned, and acute. Propodeal declivity flat, forming a rounded ~120° angle with the base of the propodeal spines. Propodeal lobes rounded and weakly developed. Metapleural gland bulla moderately large, extending from the metacoxal insertion two thirds of the way to the propodeal spiracle. Petiole moderately long (PLI 186–219), without tubercles anterodorsally. Subpetiolar process in the form of a small, acute tooth; ventral margin of petiole weakly bulging posterior to it. Petiolar peduncle moderately long: comprising about half the total length of the petiole. Petiolar node erect and narrowly rounded dorsally; squamiform: transition between peduncle and node marked by a rounded angle of ~130°; anterior face forming a rounded ~60° angle with the posterior face; posterior face forms a ~100° angle with the caudal cylinder. Postpetiole evenly rounded anterodorsally, bulging before flattening posterodorsally; concave ventrally.

In dorsal view, humeri developed and distinct: evenly rounded and wider than the rest of the mesosoma; mesothoracic spiracles weakly protruding past the lateral margins of the mesosoma, visible as slight angles where the pronotum meets the mesonotum. Metanotal groove weakly impressed and visible as a disruption in the ground sculpture. Propodeal spines narrowly approximated basally and parallel to each other apically, their apices separated from each other by about their length, the negative space between them “U” shaped. Petiolar peduncle with spiracles weakly protruding past the lateral margins, but not noticeably constricted anterior to them. Petiolar node, when viewed posterodorsally, trapezoidal: broader apically than basally; node broader than the peduncle and the caudal cylinder. Postpetiole narrow (PWI 152–158) and campaniform. Anterior margin of the postpetiole weakly convex and evenly rounds into the lateral margins; lateral margins parallel to each other but slightly bulging anteriorly; posterior corners narrowly rounded; posterior margin broadly concave. Metafemur strongly incrassate (FI 292–322).

Sculpture: median clypeal carina present, extending posteriorly nearly to the frontal triangle, and flanked on either side by three slightly weaker carinae. Lateral clypeal lobes with additional, weaker carinae; ground sculpture weakly areolate. Antennal scapes finely areolate. Cephalic dorsum densely areolate, with rugose-costate sculpture overlying the ground sculpture; fine concentric costulae surrounding antennal bases. Lateral surfaces of head sculptured similarly to the dorsum but becoming coarser between the compound eye and the mandibular insertion. Ventral surface of head shining, with weaker costulae. Pronotal neck areolate. Anterior face of pronotum weakly areolate-strigulate; lateral face shining through weakly areolate sculpture, with rugose sculpture on the anterior half, becoming costate on the posterior half. Lateral face of propodeum, meso- and metapleurae with coarsely rugose sculpture over densely areolate sculpture. Propodeal declivity with fine strigulae. Dorsal surface of mesosoma areolate, with costate sculpture overlying the ground sculpture; costae on pronotum concentric anteriorly; propodeum rugose. Femora smooth and shining, but basal quarter finely areolate. Petiole shining through weak areolate sculpture ventrally and on the dorsal surface of the peduncle, otherwise smooth and shining. Postpetiole predominantly smooth and shining, with weak areolate sculpture on the ventrolateral faces and posterior quarter dorsally. First gastral tergite and sternite smooth and shining, without spectral iridescence.

Setae: antennal scapes and funiculi with long, subdecumbent pilosity. Dorsum of the head, pronotum, waist segments, and gaster with abundant, suberect, tapering, flexuous setae, the longest of which are about one and a half times the length of the compound eye and are directed toward the midline of the body. The head bears ~40, mesosoma ~40, petiole ~10, postpetiole ~10, and first gastral tergite ~80 setae. Pubescence present over the entire body, which is nearly as long as the setae.

Color: head and mesosoma predominantly testaceous yellow. Antennal funiculus, masticatory margin of mandibles, apex of femora, tibiae, and base of basalmost tarsi testaceous.

**Gyne:** Unknown, but see comments below for discussion of ergatogyne.

**Male:** Unknown.

**Etymology:** Systematic, from the Ancient Greek ‘para’ (= alongside) and ‘aztecus’, in reference to the phylogenetic position of this species as sister to *Temnothorax aztecus*.

**Comments:** *Temnothorax paraztecus* sp. nov. inhabits cloud forest habitats in Mesoamerica south of the Isthmus of Tehuantepec and north of the Nicaraguan depression. Little is known about its biology, but it forages arboreally, having been collected several times by beating vegetation. The closest relative of *T. paraztecus* sp. nov. is the morphologically similar *T. aztecus*, with which it shared a common ancestor a little more than 7 Ma (Prebus, 2021). *Temnothorax paraztecus* sp. nov. and *T. aztecus* also overlap geographically in the Central American Nucleus, but *T. paraztecus* sp. nov., similar to *T. aztecoides* sp. nov. inhabits a generally higher elevational range (>950 m) than *T. aztecus* (Figs. 153B & 153F). Rarely collected, this species apparently inhabits a large present-day geographical range throughout the Central American Nucleus (Fig. 153F). Although the gyne of *T. paraztecus* sp. nov. remains unknown, an ergatogyne was collected in Guatemala, distinguished from workers by the tell-tale expanded tergites on the mesosoma, lack of wing scars, and infuscation on the dorsum of the head marking the positions of where the ocelli would be on a gyne (Figs. 160D–160F). Because the material of this species remains scant, I did not perform a dissection of this specimen to determine whether the ergatogyne retained reproductive function (ergatoid gyne) or not (intercaste) (Peeters, 1991).

***Temnothorax parvidentatus* sp. nov.**

**Figure 161:**
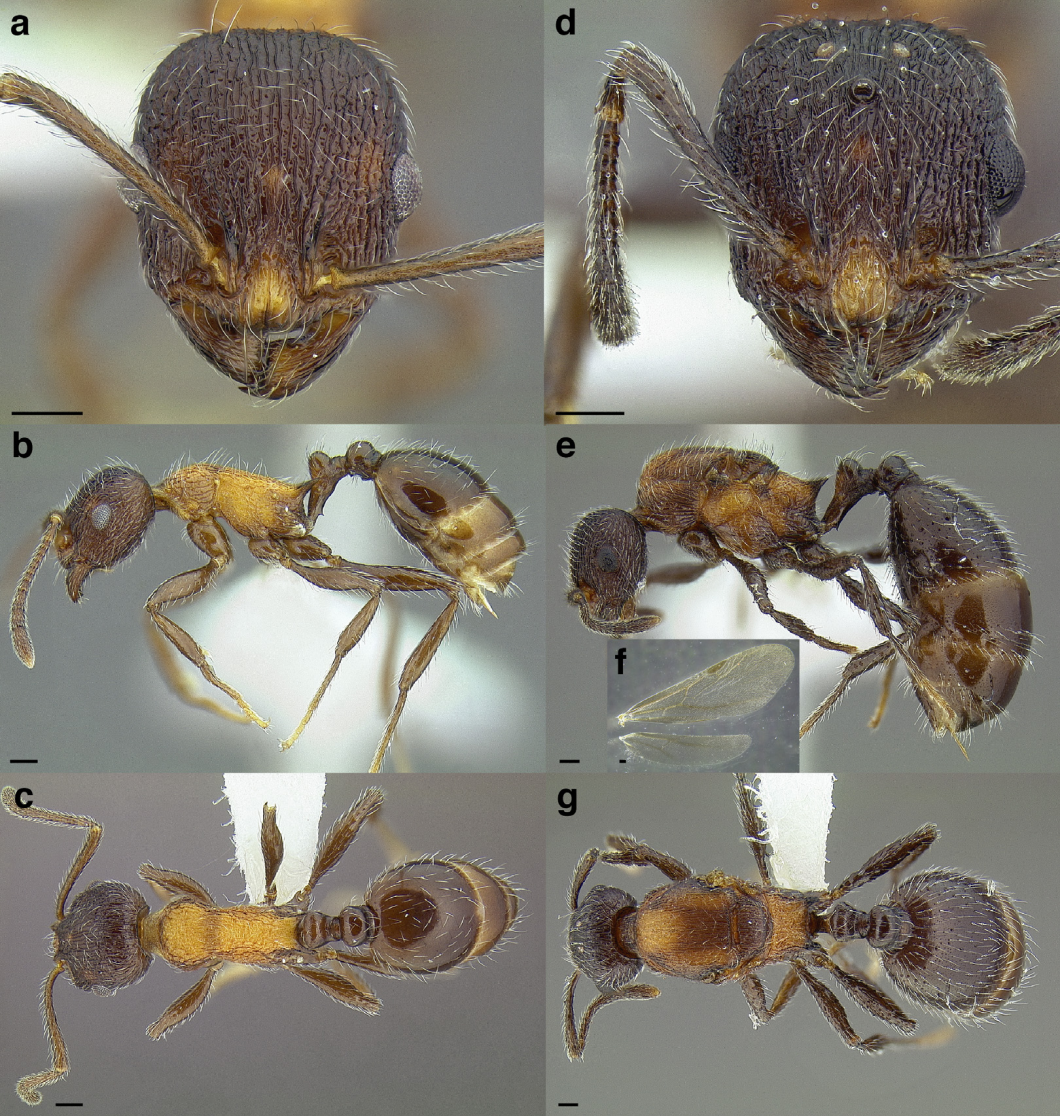
*Temnothorax parvidentatus* sp. nov. (A-C) Holotype worker (CASENT0756087). (A) Full-face view. (B) Profile view. (C) Dorsal view. (D–G) Paratype gyne (CASENT0612665). (D) Full-face view. (E) Profile view. (F) Wings. (G) Dorsal view. Scale bars 0.2 mm.

Distribution: Fig. 153G; worker & gyne: Fig. 161.

*Temnothorax salvini* grp 4, Prebus, 2021: 12. In phylogeny.

**Type material examined:** *Holotype worker:* GUATEMALA: Suchitepéquez: 4 km S Volcán Atitlán, 14.54800°N 91.19369°W ± 20 m, 1,570 m, 16 June 2009, J. Longino#JTL6717, cloud forest, ex dead twig (CASENT0756087) [CASC].

*Paratype workers and gynes:* same data as holotype, 1 alate gyne (CASENT0612665) [CASC] 1 worker (CASENT0612667) [MCZC]; same data as holotype, except: 14.54804°N 91.19108°W ± 300 m, 1,580 m, 15 June 2009, J. Longino#JTL6715, cloud forest, ex live stem, 1 worker (CASENT0758698) [UCDC] 1 worker (CASENT0758786) [USNM] 1 dealate gyne (CASENT0612659) [USNM] 1 worker (CASENT0612660) [AMNH] 1 worker (CASENT0612661) [UVGC] 1 worker (CASENT0612662) [UNAM] 1 worker (CASENT0612663) [MCZC] 1 worker (CASENT0612664) [LACM].

**Non-type material examined:** GUATEMALA: Guatemala: Origin: Guatemala City, Quarantine, San Francisco, California, U.S.A., 21 May 1946, ex *Odontoglossum grande*, 1 worker (LACMENT323431) [LACM] 1 worker (LACMENT323432) [LACM]; Suchitepéquez: 4 km S Volcán Atitlán, 14.54914°N 91.19357°W ± 55 m, 1,625 m, 16 June 2009, LLAMA#Go-B-09-1-01, cloud forest, beating vegetation, 1 worker (CASENT0610699) [USNM]; same data as previous, except: 14.55096°N 91.19352°W ± 106 m, 1,685 m, 16 June 2009, LLAMA#Go-B-09-2-01, cloud forest, beating vegetation, 1 worker (CASENT0610702) [JTLC].

**Geographic range:** Mid elevations of Guatemala (Fig. 153G).

**Worker diagnosis:** *Temnothorax parvidentatus* sp. nov. can be separated from all other species in the *salvini* clade by the following character combination: smaller species: WL less than 1.18 mm; dorsum of mesosoma weakly convex and irregularly rugose; metanotal groove not impressed; propodeum not strongly depressed below the level of the promesonotum; propodeal spines about as long as the propodeal declivity; integument sculptured between propodeal spines; subpetiolar tooth small and spiniform: shorter than the setae that arise from the peduncle directly above; petiolar node weakly squamiform: in dorsal view, petiolar node more than or equal to 1.4 times as broad as caudal cylinder, but less than or equal to 1.5 times as broad; setae on head, mesosoma, legs, waist segments and gaster erect to suberect, moderately long, abundant and tapering; integument bicolored: antennae, base of mandibles, head capsule (excluding clypeus), propodeal spine tips, legs (excluding tarsi beyond first tarsomere), waist segments, first gastral tergite and sternite, and the distal half of the remaining gastral sclerites testaceous brown; clypeus, mesosoma, tarsi (excluding first tarsomere), and basal halves of the gastral sclerites (excluding the first gastral tergite and sternite) testaceous yellow.

**Similar species:** Fellow members of the *salvini* group. *Temnothorax parvidentatus* sp. nov. can be separated from other members of the *salvini* group by the weakly squamiform petiolar node (petiolar node more than or equal to 1.6 times as broad as the caudal cylinder in *T. aztecus, T. aztecoides* sp. nov., *T. longicaulis* stat. nov., nom. nov., and *T. paraztecus* sp. nov., but less than 1.3 times in *T. salvini*), relatively small subpetiolar tooth (longer than the setae that arises directly above it in *T. longinoi* sp. nov.), short propodeal spines (longer than the propodeal declivity in *T. quetzal* sp. nov.), and sculptured integument between the propodeal spines (smooth and shining in *T. fortispinosus* sp. nov.).

**Worker measurements & indices (*n* = 6):** SL = 0.730–0.821 (0.794); FRS = 0.225–0.282 (0.259); CW = 0.756–0.906 (0.858); CWb = 0.672–0.807 (0.760); PoOC = 0.311–0.341 (0.330); CL = 0.779–0.892 (0.860); EL = 0.179–0.230 (0.211); EW = 0.131–0.167 (0.153); MD = 0.205–0.256 (0.225); WL = 1.001–1.18 (1.132); SPST = 0.269–0.363 (0.327); MPST = 0.300–0.365 (0.334); PEL = 0.368–0.499 (0.433); NOL = 0.213–0.252 (0.232); NOH = 0.120–0.167 (0.153); PEH = 0.230–0.295 (0.268); PPL = 0.199–0.251 (0.228); PW = 0.466–0.572 (0.538); SBPA = 0.200–0.263 (0.238); SPTI = 0.225–0.303 (0.269); PEW = 0.142–0.186 (0.166); PNW = 0.210–0.280 (0.243); PPW = 0.229–0.308 (0.279); HFL = 0.804–0.931 (0.889); HFWmax = 0.151–0.180 (0.170); HFWmin = 0.060–0.076 (0.066); CS = 1.062–1.253 (1.19); ES = 0.245–0.313 (0.287); SI = 97–109 (105); OI = 23–26 (24); CI = 86–90 (88); WLI = 146–151 (149); SBI = 30–33 (31); PSI = 27–31 (29); PWI = 161–178 (168); PLI = 177–221 (190); NI = 138–178 (153); PNWI = 141–151 (146); NLI = 47–58 (54); FI = 237–283 (260).

**Worker description:** In full-face view, head subquadrate, longer than broad (CI 86–90). Mandibles finely striate but shining and armed with five teeth: the apical-most well developed and acute, followed by a less developed preapical tooth and three equally developed smaller teeth. Anterior clypeal margin weakly emarginate medially. Antennal scapes very long: when fully retracted, surpassing the posterior margin of the head capsule by about three times the maximum width of the antennal scape (SI 97–109). Antennae 12-segmented; antennal club of composed of three segments, with the apical-most segment slightly longer than the preceding two in combination. Frontal carinae moderately long, extending past the antennal toruli by about two times the maximum width of the antennal scape. Compound eyes strongly protruding past the lateral margins of the head capsule. Lateral margin of head convex, forming a continuous arc from the mandibular insertions to the posterior margin of the head. Posterior head margin flat but rounding evenly into the lateral margins.

In profile view, compound eyes ovular and moderately large (OI 23–26), with 17 ommatidia in longest row. Pronotal declivity indistinct, neck and anterior face of pronotum forming a ~100° angle. Anterior face of pronotum evenly rounding into dorsal face. Dorsum of mesosoma very weakly convex, nearly flat from where it joins the anterior face of the pronotum to the propodeal spines. Promesonotal suture extending from the posterior margin of the procoxal insertion only to the mesothoracic spiracle, which is moderately well developed. Metanotal groove visible as a disruption of the sculpture laterally from where it arises between the mid- and hind coxae to where it ends in the poorly developed metathoracic spiracle, which is nearly indistinguishable against the ground sculpture. Propodeal spiracle moderately well developed, directed posterolaterally, and separated from the propodeal declivity by about five spiracle diameters. Propodeal spines moderately well developed and moderately long (PSI 27–31), as long as the propodeal declivity, tapering evenly from the base, acute, straight, and directed posterodorsally. Propodeal declivity weakly concave, forming a rounded ~120° angle with the base of the propodeal spines. Propodeal lobes rounded and weakly developed, but dorsal margin slightly angulate. Metapleural gland bulla moderately large, extending from the metacoxal insertion two thirds of the way to the propodeal spiracle. Petiole moderately long (PLI 177–221), without tubercles anterodorsally. Subpetiolar process in the form of a small, spiniform, acute, anteriorly curved tooth; ventral margin of petiole weakly concave posterior to it but bulging slightly medially. Petiolar peduncle moderately long: comprising about half the total petiole length. Petiolar node rounded-squamiform: transition between peduncle and node marked by a rounded angle of ~130°; anterior face rounding evenly into the posterior face, the dorsal margin evenly convex; posterior face forms a ~110° angle with the caudal cylinder. Postpetiole evenly rounded anterodorsally, bulging before flattening posterodorsally; concave ventrally.

In dorsal view, humeri developed and distinct: evenly rounded and slightly wider than the rest of the mesosoma; mesothoracic spiracles protruding past the lateral margins of the mesosoma, visible as angles where the pronotum meets the mesonotum. Promesonotal suture visible as a slight disruption in the ground sculpture and a dark transverse band. Metanotal groove absent. Propodeal spines broadly approximated basally and slightly diverging apically, their apices separated from each other by about their length, the negative space between them “U” shaped. Petiolar peduncle with spiracles slightly protruding past the lateral margins, but not noticeably constricted anterior to them. Petiolar node, when viewed posterodorsally, trapezoidal: broader apically than basally; apical margin weakly convex; node broader than the peduncle and the caudal cylinder. Postpetiole narrow (PWI 161–178) and subquadrate. Anterior margin of the postpetiole weakly convex, evenly rounding into the lateral margins, which bulge slightly anteriorly, and weakly converge posteriorly; posterior margin flat. Metafemur weakly to moderately incrassate (FI 237–283).

Sculpture: median clypeal carina present, extending posteriorly to the frontal triangle, and flanked on either side by two equally strong carinae. Lateral clypeal lobes with additional, weaker carinae; ground sculpture shining but very weakly areolate. Antennal scapes areolate-costulate. Cephalic dorsum densely rugose over areolate sculpture, becoming costate between the frontal carinae; fine concentric costulae surrounding the antennal insertions. Lateral surfaces of head sculptured similarly to the dorsum. Ventral surface of head shining through weak areolate sculpture over the gular region, otherwise with weak costulae over weak areolate ground sculpture. Pronotal neck areolate. Lateral surfaces of the mesosoma sculptured similarly to the head, but propodeum with costulae over smooth ground sculpture, and weak areolate sculpture between the propodeal spiracle and the base of the propodeal spine. Propodeal declivity weakly strigulate. Dorsal surface of mesosoma sculptured similarly to the cephalic dorsum and the lateral mesosoma surface. Femora shining through weak areolate sculpture. Petiole shining and weakly areolate on all surfaces but the ventral surface of the peduncle, which is smooth and shining; areolae becoming even weaker on the node. Postpetiole smooth and shining dorsally, but with weak areolate sculpture on the posterior quarter and on the lateral faces. First gastral tergite smooth and shining, with weak spectral iridescence. First gastral sternite smooth and shining, with very weak spectral iridescence.

Setae: antennal scapes and funiculi with long, suberect pilosity. Dorsum of the head, pronotum, waist segments, and gaster with abundant, erect, tapering, flexuous setae, the longest of which are about one and a quarter times the length of the compound eye and are directed toward the midline of the body. The head bears >80, mesosoma ~68, petiole ~18, postpetiole ~24, and first gastral tergite >80 setae. Pubescence present over the entire body, which is nearly as long as the setae.

Color: bicolored. antennae, base of mandibles, head capsule (excluding clypeus), propodeal spine tips, legs (excluding tarsi beyond first tarsomere), waist segments, first gastral tergite and sternite, and the distal half of the remaining gastral sclerites testaceous brown. Clypeus, mesosoma, tarsi (excluding first tarsomere), and basal halves of the gastral sclerites (excluding the first gastral tergite and sternite) testaceous yellow.

**Gyne measurements & indices (*n* = 1):** SL = 0.828; FRS = 0.307; CW = 0.976; CWb = 0.873; PoOC = 0.348; CL = 0.923; EL = 0.258; EW = 0.175; MD = 0.231; WL = 1.652; SPST = 0.407; MPST = 0.408; PEL = 0.595; NOL = 0.283; NOH = 0.201; PEH = 0.374; PPL = 0.270; PW = 0.950; SBPA = 0.442; SPTI = 0.412; PEW = 0.248; PNW = 0.363; PPW = 0.394; HFL = 1.033; HFWmax = 0.177; HFWmin = 0.069; CS = 1.335; ES = 0.346; SI = 95; OI = 26; CI = 95; WLI = 189; SBI = 51; PSI = 25; PWI = 159; PLI = 220; NI = 141; PNWI = 146; NLI = 48; FI = 257.

**Gyne description:** In full-face view, head subquadrate, longer than broad (CI 95). Mandibles finely striate but shining and armed with five teeth: the apical-most well developed, followed by a less developed preapical tooth and three equally developed smaller teeth. Anterior clypeal margin emarginated medially. Antennal scapes very long: when fully retracted, surpassing the posterior margin of the head capsule by about two and a half times the maximum width of the scape (SI 95). Antennae 12-segmented; antennal club composed of three segments, with the apical-most segment about as long as the preceding two in combination. Frontal carinae long, extending past the antennal toruli by about three times the maximum width of the antennal scape. Compound eyes strongly protruding past the lateral margins of the head capsule. Lateral margin of head evenly convex, converging from below the compound eyes to the mandibular insertions. Posterior head margin flat, rounding evenly into the lateral margins.

In profile view, compound eyes ovular and large (OI 26), with 21 ommatidia in longest row. Mesoscutum rounded evenly anteriorly, not fully covering the dorsal surface of the pronotum, and weakly convex dorsally. Anterior margin of mesoscutellum on the same level as the mesoscutum; dorsum sloping downward posteriorly. Posterior margin of metanotum extending past the posterior margin of the mesoscutellum. Propodeal spiracle moderately well developed, directed posterolaterally, and separated from the propodeal declivity by about four spiracle diameters. Propodeal spines stout, well developed, and moderately long (PSI 25), about as long as the propodeal declivity, tapering evenly from the base, directed posterodorsally, straight, and acute. Propodeal declivity weakly concave, forming a rounded ~120° angle with the base of the propodeal spines. Propodeal lobes rounded and very weakly developed, but slightly angulate dorsally. Metapleural gland bulla moderately large, extending from the metacoxal insertion two thirds of the way to the propodeal spiracle. Petiole long (PLI 220), without tubercles anterodorsally. Subpetiolar process in the form of an acute, spiniform tooth, which grades evenly into the ventral margin of the petiole posteriorly. Petiolar peduncle moderately long: comprising about half of the total petiole length. Petiolar node rounded-squamiform: transition between peduncle and node a rounded ~150° angle; anterior face rounding evenly into the posterior face, which forms a ~120° angle with the caudal cylinder. Postpetiole evenly rounded anterodorsally, bulging before flattening posterodorsally; concave ventrally.

In dorsal view, mesoscutum not fully covering pronotum anteriorly; humeri visible laterally as rounded sclerites. Mesoscutum evenly rounded anteriorly; anterior margin rounding evenly into the lateral margins; lateral margins diverging to the wing bases, then converging through a curve to the convex posterior margin. Propodeal spines strongly diverging basally, but parallel to each other apically, their apices separated from each other by about two times their length. Petiolar peduncle with spiracles weakly protruding past the lateral margins. Petiolar node, when viewed posterodorsally, roughly trapezoidal: slightly broader apically than basally; apical margin convex; node broader than the peduncle and the caudal cylinder. Postpetiole narrow (PWI 159) and subquadrate. Anterior margin of postpetiole weakly convex, with corners rounding evenly into the lateral margins; lateral margins parallel to each other; posterior margin flat. Metafemur moderately incrassate (FI 257).

Sculpture: median clypeal carina present, extending from the anterior margin to frontal triangle, and flanked on either side by two slightly weaker carinae. Lateral clypeal lobes with additional weaker carinae; ground sculpture weakly areolate. Antennal scapes weakly areolate-costulate. Cephalic dorsum densely rugose over areolate ground sculpture, becoming costate between the frontal carinae. Lateral surfaces of head sculptured similarly to the dorsum, but with rugae stronger between the compound eye and the mandibular insertion. Ventral surface of head weakly costulate, becoming strigulate posteriorly. Pronotal neck areolate. Anterior face of pronotum shining and weakly sculptured; humeri rugose; lateral faces coarsely costate over weak areolate ground sculpture. Katepisternum predominantly shining, with weak costulae on the posterior third. Anepisternum finely, weakly costulate over areolate ground sculpture. Metapleuron and lateral surface of propodeum costate. Propodeal declivity strigulate. Mesoscutum and mesoscutellum areolate, with costulate sculpture; mesoscutum with a small patch of weak areolate sculpture anteromedially. Metanotum finely areolate. Propodeum coarsely rugose. Femora predominantly smooth and shining. Petiole finely longitudinally areolate-costulate on nearly all surfaces, but smooth and shining on the ventral surface of the petiole; sculpture weaker on the dorsum of the node. Postpetiole predominantly shining through weak areolate sculpture; areolae becoming stronger on the posterior quarter and on the lateral faces. First gastral tergite smooth and shining, with weak spectral iridescence. First gastral sternite weakly areolate basally, otherwise smooth and shining, with weak spectral iridescence.

Setae: antennal scapes and funiculi with long, suberect pilosity. Dorsum of the head, pronotum, waist segments, and gaster with abundant, erect, tapering, flexuous setae, the longest of which are about as long as the width of the compound eye. Pubescence present over the entire body, which is nearly as long as the setae.

Color: bicolored. Antennae, base of mandibles, head capsule (excluding median lobe of clypeus), wing bases, propodeal spine tips, legs (excluding tarsi beyond first tarsomere), waist segments, first gastral tergite and sternite, and the distal half of the remaining gastral sclerites testaceous brown. Median lobe of clypeus, mesosoma, tarsi (excluding first tarsomere), and basal halves of the gastral sclerites (excluding the first gastral tergite and sternite) testaceous yellow.

**Male:** Unknown.

**Etymology:** Morphological, from the Latin ‘parvus’ (= small) + ‘dentatus’ (= toothed), in reference to the relatively small subpetiolar tooth.

**Comments:** *Temnothorax parvidentatus* sp. nov. is known from a few collections in mid elevation cloud forests in Guatemala; additional specimens were collected from the orchid *Odontoglossum grande* in quarantine. Apparently restricted to the Central American Nucleus, this species is sister to the geographically widespread group consisting of *T. aztecus*, *T. longicaulis* stat. nov., nom. nov., *T. longinoi* sp. nov., *T. paraztecus* sp. nov., and *T. salvini*, with which it shared a common ancestor a little more than 9 Ma (Prebus, 2021). *Temnothorax parvidentatus* sp. nov. has been found in dead twigs, live hollow stems, and by beating vegetation. Like many of its close relatives in the *salvini* species group, this species apparently nests and forages arboreally.

***Temnothorax quetzal* sp. nov.**

**Figure 162:**
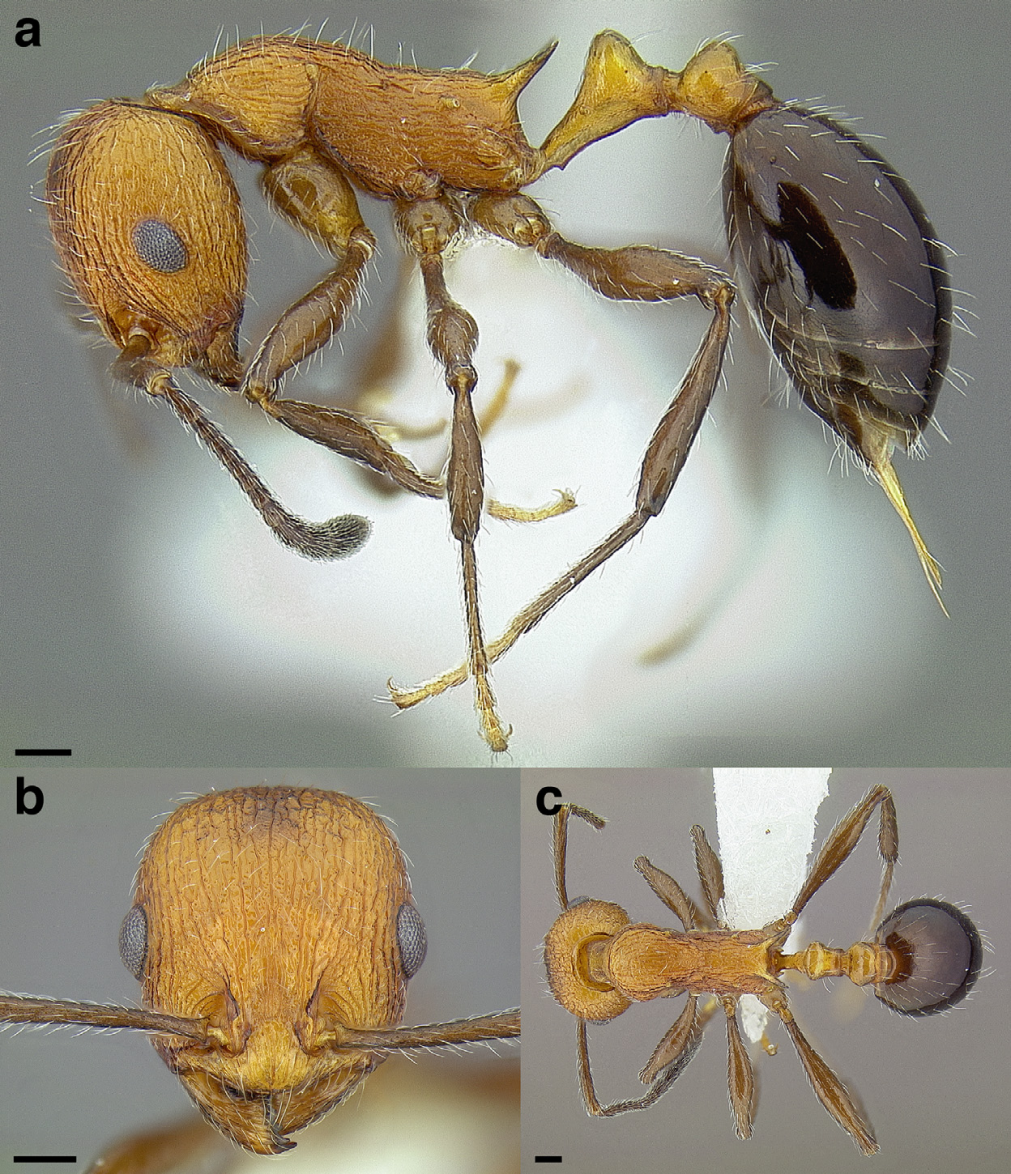
*Temnothorax quetzal* sp. nov. holotype worker (CASENT0614495). (A) Profile view. (B) Full-face view. (C) Dorsal view. Scale bars 0.2 mm.

Distribution: Fig. 153H; worker: Fig. 162.

*Temnothorax salvini* grp 1, Prebus, 2021: 12. In phylogeny.

**Type material examined:** *Holotype worker:* GUATEMALA: Baja Verapaz: Biotopo Quetzal, 15.20885°N 99.21592°W ± 207 m, 1,925 m, 9 May 2009, LLAMA#Go-B-02-4-03, cloud forest, beating vegetation (CASENT0614495) [CASC].

*Paratype worker:* same data as holotype, 1 worker (CASENT0614496) [UCDC].

**Geographic range:** high elevations of Guatemala (Baja Verapaz) (Fig. 153H).

**Worker diagnosis:** *Temnothorax quetzal* sp. nov. can be separated from all other species in the *salvini* clade by the following character combination: dorsum of mesosoma sinuate; metanotal groove not impressed; propodeum not strongly depressed below the level of the promesonotum; propodeal spines upturned and longer than the propodeal declivity; subpetiolar tooth small and triangular: shorter than the setae that arise from the peduncle directly above; petiolar node weakly squamiform: in dorsal view, petiolar node less than or equal to 1.5 times as broad as caudal cylinder; setae on head, mesosoma, legs, waist segments and gaster erect to suberect, long, abundant and tapering; integument bicolored; predominantly testaceous yellow; antennae, femora, and tibiae testaceous; gaster dark brown.

**Similar species:** Fellow members of the *salvini* group. *Temnothorax quetzal* sp. nov. can be separated from other members of the *salvini* group by the weakly squamiform petiolar node (petiolar node more than or equal to 1.6 times as broad as the caudal cylinder in *T. aztecus, T. aztecoides* sp. nov., *T. longicaulis* stat. nov., nom. nov*.*, and *T. paraztecus* sp. nov.), relatively small subpetiolar tooth (longer than the setae that arises directly above it in *T. longinoi* sp. nov.), and long propodeal spines (as long as or shorter than the propodeal declivity in *T. fortispinosus* sp. nov., *T. parvidentatus* sp. nov., and *T. salvini*).

**Worker measurements & indices (*n* = 2):** SL = 0.973–0.978 (0.976); FRS = 0.291–0.298 (0.295); CW = 1.000–1.002 (1.001); CWb = 0.883–0.905 (0.894); PoOC = 0.371–0.381 (0.376); CL = 0.999–1.018 (1.009); EL = 0.232–0.255 (0.244); EW = 0.167; MD = 0.249–0.256 (0.253); WL = 1.367–1.375 (1.371); SPST = 0.439–0.450 (0.445); MPST = 0.386–0.404 (0.395); PEL = 0.520–0.527 (0.524); NOL = 0.290–0.303 (0.297); NOH = 0.180–0.209 (0.195); PEH = 0.335–0.348 (0.342); PPL = 0.256–0.271 (0.264); PW = 0.595–0.618 (0.607); SBPA = 0.256–0.264 (0.260); SPTI = 0.368–0.451 (0.410); PEW = 0.202–0.212 (0.207); PNW = 0.295–0.299 (0.297); PPW = 0.322–0.332 (0.327); HFL = 1.187–1.201 (1.194); HFWmax = 0.195–0.202 (0.199); HFWmin = 0.075–0.078 (0.077); CS = 1.383–1.414 (1.398); ES = 0.316–0.339 (0.327); SI = 108–111 (109); OI = 23–24 (23); CI = 88–89 (89); WLI = 151–156 (153); SBI = 29; PSI = 32–33 (32); PWI = 157–159 (158); PLI = 192–206 (199); NI = 145–161 (153); PNWI = 141–146 (144); NLI = 56–57 (57); FI = 259–260 (259).

**Worker description:** In full-face view, head subquadrate, longer than broad (CI 88–89). Mandibles finely, densely striate but shining and armed with five teeth: the apical-most well developed and acute, followed by a less developed preapical tooth and three equally developed smaller teeth. Anterior clypeal margin convex medially. Antennal scapes moderately long: when fully retracted, surpassing the posterior margin of the head capsule by about the maximum width of the antennal scape (SI 108–111). Antennae 12-segmented; antennal club of composed of three segments, with the apical-most segment slightly longer than the preceding two in combination. Frontal carinae short, extending past the antennal toruli by about one and a half times the maximum width of the antennal scape. Compound eyes moderately protruding past the lateral margins of the head capsule. Lateral margin of head weakly convex, forming a continuous arc from the mandibular insertions to the posterior margin of the head. Posterior head margin weakly convex, rounding evenly into the lateral margins.

In profile view, compound eyes ovular and moderately large (OI 23–24), with 18 ommatidia in longest row. Pronotal declivity indistinct, neck and anterior face of pronotum forming a ~120° angle. Anterior face of pronotum evenly rounding into dorsal face. Promesonotum very weakly convex to the weakly impressed metanotal groove. Propodeum flat and slightly depressed below the level of the promesonotum. Promesonotal suture extending from the posterior margin of the procoxal insertion to the mesothoracic spiracle, which is moderately well developed; continuing dorsally as a faint disruption in the ground sculpture. Metanotal groove visible as a disruption of the sculpture laterally from where it arises between the mid- and hind coxae to where it ends in the poorly developed metathoracic spiracle, which is nearly indistinguishable against the ground sculpture. Propodeal spiracle moderately well developed, directed posterolaterally, and separated from the propodeal declivity by about five spiracle diameters. Propodeal spines well developed and long (PSI 32–33), slightly longer than the propodeal declivity, tapering evenly from the base, upturned, and acute. Propodeal declivity weakly concave, forming a rounded ~120° angle with the base of the propodeal spines. Propodeal lobes rounded and weakly developed. Metapleural gland bulla small, extending from the metacoxal insertion halfway to the propodeal spiracle. Petiole long (PLI 192–206), without tubercles anterodorsally. Subpetiolar process in the form of a small, triangular, acute tooth, which grades into the ventral margin of the petiole posteriorly; ventral margin of petiole weakly bulging posterior to it. Petiolar peduncle moderately long: comprising about half of the total petiole length. Petiolar node cuneiform-squamiform: transition between peduncle and node marked by a rounded angle of ~120°; anterior face forming a rounded ~80° angle with the posterior face, which forms a ~120° angle with the caudal cylinder. Postpetiole evenly rounded anterodorsally, strongly bulging before flattening posterodorsally; concave ventrally.

In dorsal view, humeri developed and distinct: evenly rounded and about one and a quarter times wider than the rest of the mesosoma; mesothoracic spiracles weakly protruding past the lateral margins of the mesosoma, visible as slight angles where the pronotum meets the mesonotum. Promesonotal suture visible as a slight disruption in the ground sculpture. Metanotal groove moderately well impressed. Propodeal spines broadly approximated basally and strongly diverging apically, their apices separated from each other by about their length, the negative space between them “V” shaped. Petiolar peduncle with spiracles slightly protruding past the lateral margins, but not noticeably constricted anterior to them. Petiolar node, when viewed posterodorsally, trapezoidal: broader apically than basally; apical margin weakly emarginate; node broader than the peduncle and the caudal cylinder. Postpetiole narrow (PWI 157–159) and trapezoidal. Anterior margin of the postpetiole weakly convex, transitioning into the lateral margins through a rounded angle; lateral margins bulging slightly anteriorly before converging to the angulate posterior corners; posterior margin flat. Metafemur moderately incrassate (FI 259–260).

Sculpture: median clypeal carina present, extending posteriorly to the level of the antennal insertions, and flanked on either side by multiple slightly weaker carinae. Lateral clypeal lobes with additional, weaker carinae; ground sculpture weakly areolate. Antennal scapes weakly areolate. Cephalic dorsum areolate, with rugose sculpture overlying the ground sculpture; fine concentric costulae surrounding the antennal insertions; rugae becoming costate from the antennal insertions to the posterior quarter of the head, cross reticulations becoming stronger beyond this point. Lateral surface of head sculptured similarly to the dorsum, with cross reticulations becoming stronger posterior to the compound eye. Ventral surface of head smooth and shining, with weak costulae. Pronotal neck areolate. Lateral surfaces of the mesosoma sculptured similarly to the head, but with areolae very weak on the lateral face of the pronotum, shining between rugae; area between propodeal spiracle and propodeal spine very weakly sculptured and shining. Propodeal declivity shining, with weak strigulae. Dorsal surface of mesosoma areolate, with rugose-costate sculpture. Femora shining through weak areolate sculpture. Petiole finely areolate on all surfaces but the node, which is smooth and shining. Postpetiole smooth and shining dorsally, weakly areolate laterally. First gastral tergite and sternite smooth and shining, without spectral iridescence.

Setae: antennal scapes and funiculi with moderately long, suberect pilosity. Dorsum of the head, pronotum, waist segments, and gaster with abundant, suberect, tapering, flexuous setae, the longest of which are about the length of the compound eye and are directed toward the midline of the body. The head bears ~50, mesosoma ~36, petiole 10, postpetiole ~16, and first gastral tergite ~84 setae. Pubescence present over the entire body, which is nearly as long as the setae.

Color: bicolored. Predominantly testaceous yellow; antennae, femora, and tibiae testaceous; gaster dark brown.

**Gyne:** Unknown.

**Male:** Unknown.

**Etymology:** Geographical, based on the type locality of Biotopo Quetzal, in Baja Verapaz, Guatemala.

**Comments:** *Temnothorax quetzal* sp. nov. is known only from the type specimens, collected via beating vegetation in cloud forest. The closest relative of *T. quetzal* sp. nov., *T. fortispinosus* sp. nov., is also known only from a single locality in the Central American Nucleus, but at mid elevations (1,520–1,740 m; Figs. 153C & 153H). These two morphologically distinctive species shared a common ancestor around 8 Ma, and together are sister to the remainder of the *salvini* group (Prebus, 2021). Like other members of the *salvini* species group, *T. quetzal* sp. nov. is likely arboreally nesting.

***Temnothorax salvini* (Forel, 1899)**

**Figure 163:**
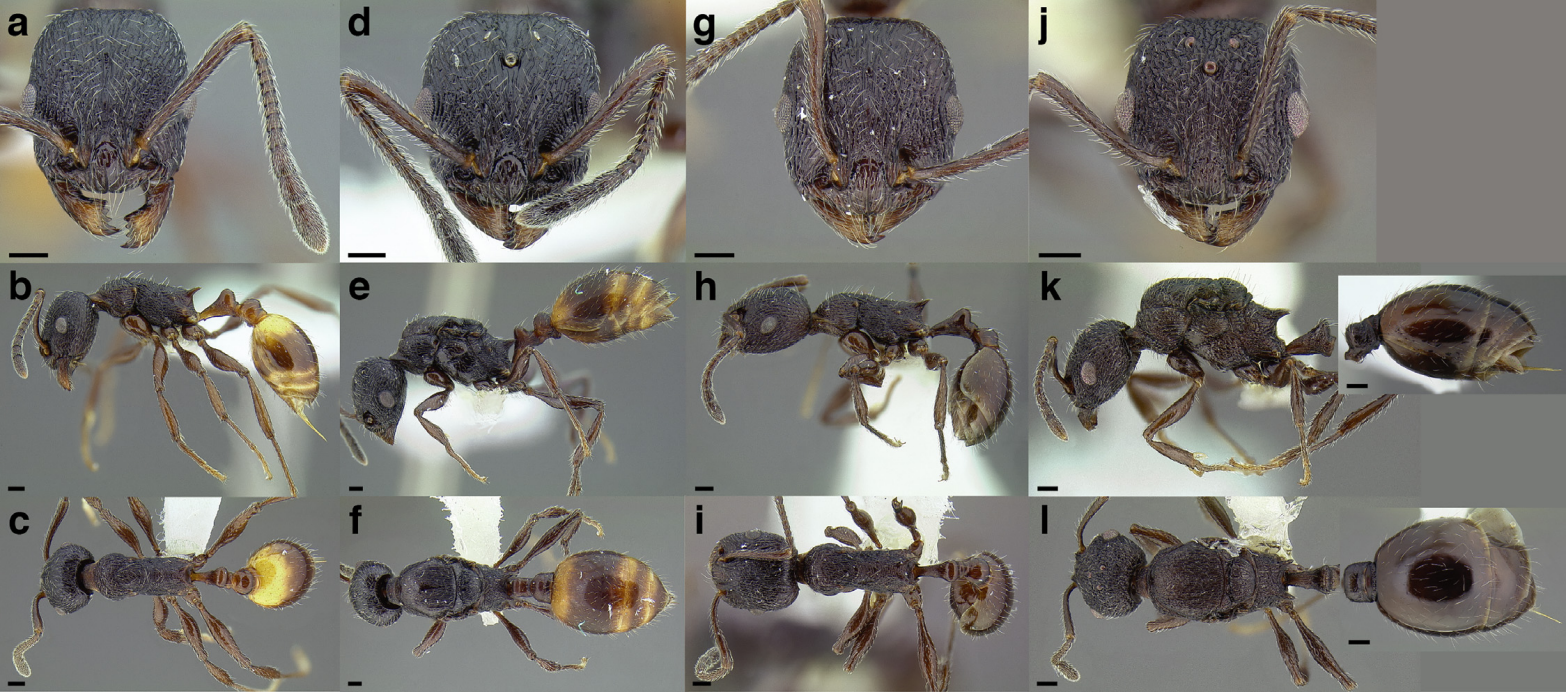
*Temnothorax salvini*. (A–C) Worker (CASENT0756074). (A) Full-face view. (B) Profile view. (C) Dorsal view. (D–F) Gyne (JTLC000001501). (D) Full-face view. (E) Profile view. (F) Dorsal view. (G–L) Dark form worker, Cartago, Costa Rica (LACMENT323467). (G) Full-face view. (H) Profile view. (I) Dorsal view. (J–L) Dark form gyne, Chiriquí, Panama (CASENT0740647). (J) Full-face view. (K) Profile view. (L) Dorsal view. Scale bars 0.2 mm.

Distribution: Fig. 153I; worker, gyne & variability: Fig. 163.

*Macromischa salvini* Forel, 1899: 57, pl. 3, fig. 18. Syntype workers. Volcán de Chiriqui, Panama. One syntype worker here designated **lectotype**.

*Leptothorax salvini* (Forel): Baroni Urbani, 1978: 494. First combination in *Leptothorax*.

*Temnothorax salvini* (Forel): Bolton, 2003: 272. First combination in *Temnothorax*.

**Type material examined:** *Lectotype worker:* PANAMA: Chiriquí: Volcán de Chiriquí, 1,220–1,830 m, Champion#99-304, leftmost specimen (images of BMNH (E) 1014995, CASENT0901796 examined on antweb.org) [BMNH].

*Paralectotype worker*: same data as lectotype, 1 worker, severely damaged (images of CASENT0908989 examined on antweb.org) [MHNG].

**Non-type material examined:** COSTA RICA: Heredia: 8 km N Vol. Barba, 10.2°N 84.1°W, 1,830 m, 4–14 July 1986, J. Longino#JTL1328, wet forest, in base of *Tillandsia*, 10 workers (INBIOCRI002281235-INBIOCRI002281237, INBIOCRI002281239) [JTLC] 2 workers (INBIOCRI002281238) [UCDC]; 8 km ENE Vara Blanca, 10.2°N 84.1°W, 1,800 m, 16 April 2002, J. Longino#JTL4654, montane wet forest, forager, 1 worker (JTLC000001493) [UCDC]; 6 km ENE Vara Blanca: 10.183333°N 84.116667°W, 2,000 m, 18 April 2002, J. Longino#JTL4559, montane wet forest treefall, ex live stem Araliaceae, 1 worker and 1 dealate gyne (JTLC000001499) [JTLC] 2 workers (JTLC000001500) [JTLC]; same data as previous, except: J. Longino#JTL4660, montane wet forest treefall, nest in epiphytes, 1 worker & 1 dealate gyne (JTLC000001501) [JTLC] 1 worker (CASENT0756074) [JTLC]; 10.18333°N 84.11667°W ± minute, 2,000 m 18 April 2002, J. Longino#4659, montane wet forest treefall, ex live stem Araliaceae, 1 worker (CASENT0758697) [UCDC] 1 worker (CASENT0758295) [JTLC]. Cartago: 4 km NE Canon, 2,360 m, 22 May 1995, J. Rifkind, 3 workers (LACMENT323466) [LACM] 2 workers (LACMENT323467) [LACM]. Puntarenas: Cerro Gemelo, 9.05°N 82.93°N, 2,400 m, 3 July 1995, parataxonomist course#12-APC-95, oak forest, branch fall, 1 worker (INBIOCRI001280795) [UCDC]. San José: 7 km SSE Santa María, 9.58434°N 83.95101°W ± 300 m, 1,510 m, 28 June 2015, ADMAC#Go-E-05-1-01, road through cloud forest/pasture, beating vegetation, 1 worker (CASENT0632189) [UCDC] 1 worker (CASENT0632190) [JTLC]; 2 km E San Gerardo, 9.466667°N 83.583333°W, 1,600 m, 4 August 1985, P.S. Ward #7810-4, montane rainforest, on low vegetation, 1 worker (CASENT0758333) [UCDC].

PANAMA: Chiriquí: 3 km south Cerro Punta, 1,700 m, 24 January 1987, E.S. Ross, ANTC29709 (CASENT0740648) [CASC]; same data as previous, except: ANTC29708, 1 dealate gyne (CASENT0740647) [CASC].

**Geographic range:** Mid-to-high elevations, Costa Rica to Panama (Fig. 153I).

**Worker diagnosis:** *Temnothorax salvini* can be separated from all other species in the *salvini* clade by the following character combination: dorsum of mesosoma weakly sinuate; metanotal groove not impressed; propodeum not strongly depressed below the level of the promesonotum; propodeal spines shorter than the propodeal declivity; subpetiolar tooth small and triangular: shorter than the setae that arises from the peduncle directly above; petiolar node very weakly squamiform: in dorsal view, petiolar node more than or equal to 1 time as broad as caudal cylinder, but less than or equal to 1.5 times as broad; setae on head, mesosoma, legs, waist segments and gaster erect to suberect, moderately long, abundant and tapering; head and mesosoma predominantly dark brown, nearly black; antennae, mandibles, promesonotal suture, legs, dorsum of waist segments, and distal margins of gastral sclerites testaceous; venter of waist segments, basal margins of gastral sclerites, and sting light yellow.

**Similar species:** Fellow members of the *salvini* group. *Temnothorax salvini* can be separated from other members of the *salvini* group by the very weakly squamiform petiolar node (petiolar node more than or equal to 1.6 times as broad as the caudal cylinder in *T. aztecus, T. aztecoides* sp. nov., *T. longicaulis* stat. nov., nom. nov*.*, and *T. paraztecus* sp. nov.), relatively small subpetiolar tooth (longer than the setae that arises directly above it in *T. longinoi* sp. nov.), short propodeal spines (longer than the propodeal declivity in *T. quetzal* sp. nov.).

**Worker measurements & indices (*n* = 9):** SL = 0.801-0.909 (0.866); FRS = 0.258–0.337 (0.308); CW = 0.836–1.031 (0.958); CWb = 0.735–0.930 (0.860); PoOC = 0.349–0.428 (0.390); CL = 0.901–1.067 (1.001); EL = 0.199–0.269 (0.240); EW = 0.152–0.209 (0.172); MD = 0.203–0.281 (0.232); WL = 1.124–1.450 (1.306); SPST = 0.311–0.380 (0.345); MPST = 0.324–0.430 (0.378); PEL = 0.398–0.511 (0.457); NOL = 0.222–0.314 (0.274); NOH = 0.142–0.170 (0.158); PEH = 0.262–0.336 (0.306); PPL = 0.195–0.291 (0.246); PW = 0.487–0.655 (0.599); SBPA = 0.179–0.329 (0.232); SPTI = 0.244–0.401 (0.294); PEW = 0.166–0.232 (0.208); PNW = 0.205–0.297 (0.252); PPW = 0.263–0.368 (0.334); HFL = 0.855–1.062 (0.996); HFWmax = 0.177–0.220 (0.204); HFWmin = 0.062–0.079 (0.070); CS = 1.186–1.452 (1.360); ES = 0.275–0.374 (0.326); SI = 96–109 (101); OI = 23–26 (24); CI = 82–89 (86); WLI = 146–158 (152); SBI = 22–35 (27); PSI = 23–28 (27); PWI = 156–171 (161); PLI = 158–210 (187); NI = 148–196 (174); PNWI = 101–145 (122); NLI = 53–64 (60); FI = 273–344 (291).

**Worker description:** In full-face view, head subquadrate, longer than broad (CI 82–89). Mandibles densely striate but shining and armed with five teeth: the apical-most well developed and acute, followed by a less developed preapical tooth and three equally developed smaller teeth. Anterior clypeal margin weakly emarginated medially. Antennal scapes moderately long: when fully retracted, surpassing the posterior margin of the head capsule by about the maximum width of the antennal scape (SI 96–109). Antennae 12-segmented; antennal club of composed of three segments, with the apical-most segment slightly longer than the preceding two in combination. Lateral margin of head weakly convex, forming a continuous arc from the mandibular insertions to the posterior margin of the head. Posterior head margin flat but rounding evenly into the lateral margins. Frontal carinae short: extending past the antennal toruli by about one and a half times the maximum width of the antennal scape. Compound eyes protruding past the lateral margins of the head capsule.

In profile view, compound eyes ovular and moderately large (OI 23–26), with 14 ommatidia in longest row. Pronotal declivity indistinct, neck and anterior face of pronotum forming a ~110° angle. Mesosoma very weakly convex from where it joins the pronotal neck to the weakly impressed metanotal groove; propodeum flat to the base of the propodeal spines. Promesonotal suture extending from the posterior margin of the procoxal insertion to the mesothoracic spiracle, which is well developed, then continuing dorsally as a weak sulcus. Metanotal groove visible as a disruption of the sculpture laterally from where it arises between the mid- and hind coxae to the poorly developed metathoracic spiracle, which is nearly indistinguishable against the ground sculpture; continuing dorsally as a faint impression. Propodeal spiracle well developed, directed posterolaterally, and separated from the propodeal declivity by about four spiracle diameters. Propodeal spines moderately well developed, but short (PSI 23–28), about two thirds as long as the propodeal declivity, flared at the base, weakly downcurved, and acute. Propodeal declivity flat, forming a rounded ~120° angle with the base of the propodeal spines. Propodeal lobes rounded and weakly developed, but slightly angulate dorsally. Metapleural gland bulla small, extending from the metacoxal insertion halfway to the propodeal spiracle. Petiole moderately long (PLI 158–210), without tubercles anterodorsally. Subpetiolar process in the form of a very small, triangular tooth, which grades evenly into the ventral surface of the petiole posteriorly; ventral margin of petiole weakly bulging posterior to it. Petiolar peduncle short: comprising about a third of the total length of the petiole. Petiolar node erect and narrowly rounded, nearly cuneiform: transition between peduncle and node marked by a rounded angle of ~140°, resulting in a weakly concave anterior node face; anterior face forming a rounded ~100° angle with the dorsal face, which is evenly convex; dorsal face rounding evenly into the posterior face, which forms a ~100° angle with the caudal cylinder. Postpetiole evenly rounded anterodorsally, bulging before flattening posterodorsally; concave ventrally.

In dorsal view, humeri developed and distinct: evenly rounded and wider than the rest of the mesosoma; mesothoracic spiracles weakly protruding past the lateral margins of the mesosoma, visible as slight angles where the pronotum meets the mesonotum. Promesonotal suture visible as a faint sulcus. Metanotal groove weakly impressed and visible as a disruption in the ground sculpture. Propodeal spines broadly approximated basally and weakly diverging apically, their apices separated from each other by about one and a half times their length, the negative space between them “U” shaped. Petiolar peduncle with spiracles protruding past the lateral margins, but not noticeably constricted anterior to them. Petiolar node, when viewed posterodorsally, trapezoidal: broader apically than basally; node broader than the peduncle and the caudal cylinder. Postpetiole narrow (PWI 156–171) and subquadrate. Anterior margin of the postpetiole flat but evenly rounds into the lateral margins; lateral margins parallel; posterior corners narrowly rounded; posterior margin broadly concave. Metafemur moderately to strongly incrassate (FI 273–344).

Sculpture: median clypeal carina present, extending posteriorly nearly to the frontal triangle, and flanked on either side by two slightly weaker carinae. Lateral clypeal lobes with additional, weaker carinae; ground sculpture smooth and shining. Antennal scapes weakly areolate. Cephalic dorsum weakly areolate, with predominantly rugose-costate sculpture overlying the ground sculpture. Lateral surfaces of head sculptured similarly to the dorsum, but rugae forming whorls around the compound eyes. Ventral surface of head shining, with weaker costulae. Pronotal neck areolate. Anterior face of pronotum coarsely areolate; lateral face coarsely costate. Lateral face of pronotum, meso- and metapleuron coarsely rugose over weak areolate sculpture. Propodeal declivity weakly areolate. Dorsal surface of mesosoma weakly areolate, with costate sculpture overlying the ground sculpture; costae on pronotum concentric anteriorly; rugae on propodeum weaker and transverse. Femora smooth and shining. Petiole shining through very weak areolate sculpture ventrally and on the dorsal surface of the peduncle, otherwise smooth and shining; a weak carina extends longitudinally from the propodeal spiracle to the caudal cylinder. Postpetiole smooth and shining. First gastral tergite and sternite smooth and shining, without spectral iridescence.

Setae: antennal scapes and funiculi with moderately long, subdecumbent pilosity. Dorsum of the head, pronotum, waist segments, and gaster with abundant, suberect, tapering, flexuous setae, the longest of which are slightly longer than the length of the compound eye and are directed toward the midline of the body. The head bears ~40, mesosoma ~42, petiole 4, postpetiole ~18, and first gastral tergite ~84 setae. Pubescence present over the entire body, which is nearly as long as the setae.

Color: head and mesosoma predominantly dark brown, nearly black. Antennae, mandibles, promesonotal suture, legs, dorsum of waist segments, and distal margins of gastral sclerites testaceous. Venter of waist segments, basal margins of gastral sclerites, and sting light yellow.

**Gyne measurements & indices (*n* = 1):** SL = 0.892; FRS = 0.333; CW = 1.083; CWb = 0.975; PoOC = 0.410; CL = 1.079; EL = 0.297; EW = 0.208; MD = 0.221; WL = 1.738; SPST = 0.406; MPST = 0.435; PEL = 0.556; NOL = 0.329; NOH = 0.199; PEH = 0.400; PPL = 0.296; PW = 0.925; SBPA = 0.386; SPTI = 0.388; PEW = 0.282; PNW = 0.303; PPW = 0.471; HFL = 1.157; HFWmax = 0.205; HFWmin = 0.07; CS = 1.515; ES = 0.401; SI = 91; OI = 26; CI = 90; WLI = 178; SBI = 40; PSI = 23; PWI = 167; PLI = 188; NI = 165; PNWI = 107; NLI = 59; FI = 293.

**Gyne description:** In full-face view, head subquadrate, longer than broad (CI 90). Mandibles finely striate but shining and armed with five teeth: the apical-most well developed, followed by a less developed preapical tooth and three equally developed smaller teeth. Anterior clypeal margin weakly emarginated medially. Antennal scapes moderately long: surpassing the posterior margin of the head capsule by about the maximum width of the antennal scape (SI 91). Antennae 12-segmented; antennal club composed of three segments, with the apical-most segment as long as the preceding two in combination. Frontal carinae short: extending past the antennal toruli by about one and a half times the maximum width of the antennal scape. Compound eyes moderately protruding past the lateral margins of the head capsule. Lateral margin of head evenly convex, converging from below the compound eyes to the mandibular insertions. Posterior head margin weakly concave but rounding evenly into the lateral margins.

In profile view, compound eyes ovular and moderately large (OI 26), with 20 ommatidia in longest row. Mesoscutum rounded evenly anteriorly, not fully covering the dorsal surface of the pronotum, and weakly convex dorsally. Mesoscutellum slightly depressed below the level of the mesoscutum and weakly convex dorsally. Posterior margin of metanotum extending beyond the posterior margin of the mesoscutum. Propodeal spiracle well developed, directed posterolaterally, and separated from the propodeal declivity by about four spiracle diameters. Propodeal spines stout and well developed, but short (PSI 23), about as two thirds as long as the propodeal declivity, tapering evenly from the base, directed posteriorly, straight, and acute. Propodeal declivity weakly concave, forming a rounded ~100° angle with the base of the spines. Propodeal lobes rounded and weakly developed, but slightly angulate dorsally. Metapleural gland bulla small, extending from the metacoxal insertion halfway to the propodeal spiracle. Petiole moderately long (PLI 188), without tubercles anterodorsally. Subpetiolar process in the form of a minute, blunt tooth, which grades evenly into the ventral margin of the petiole posteriorly. Petiolar peduncle short: comprising about a third of the total petiole length. Petiolar node erect and narrowly rounded, nearly cuneiform: transition between peduncle and node marked by a rounded angle of ~140°, resulting in a weakly concave anterior node face; anterior face forming a rounded ~100° angle with the dorsal face, which is evenly convex; dorsal face rounding evenly into the posterior face, which forms a ~100° angle with the caudal cylinder. Postpetiole evenly rounded anterodorsally, bulging before flattening posterodorsally; lobed ventrally.

In dorsal view, mesoscutum not fully covering pronotum anteriorly; humeri visible laterally as angulate sclerites. Mesoscutum with a distinctive shape: hexagonal, with anterior margin narrow and weakly convex, lateral margins diverging posteriorly to the wing bases, then converging slightly to the posterior margin, which is broader than the anterior margin. Propodeal spines weakly diverging apically, their apices separated from each other by about twice their length. Petiolar peduncle with spiracles protruding past the lateral margins, but not noticeably constricted anterior to them. Petiolar node, when viewed posterodorsally, trapezoidal: broader apically than basally; node slightly broader than the peduncle and the caudal cylinder. Postpetiole narrow (PWI 167) and campaniform. Anterior margin of postpetiole nearly flat, with corners evenly rounding into the lateral margins, which diverge slightly to the angulate posterior corners; posterior margin flat. Metafemur moderately incrassate (FI 293).

Sculpture: median clypeal carina present, extending from the anterior margin nearly to frontal triangle, and flanked two equally strong carinae. Lateral clypeal lobes with additional weaker carinae; ground sculpture superficially areolate. Antennal scapes weakly areolate. Cephalic dorsum weakly areolate, with rugose-costate sculpture overlying the ground sculpture; antennal insertions surrounded by concentric costulae. Lateral surfaces of head sculptured similarly to the dorsum, but rugae forming whorls around the compound eye, and reticulation becoming stronger between the compound eye and mandibular insertion. Ventral surface of head mostly smooth and shining, with weak costulae. Pronotal neck areolate. Pronotum smooth and shining anteriorly and costate laterally. Anepisternum predominantly smooth and shining, with superficial costae. Katepisternum smooth and shining, with superficial costae that become stronger posteriorly. Metapleuron and lateral face of propodeum with stronger costae. Propodeal declivity strigulate. Mesoscutum with costulae over areolate ground sculpture; a small patch of finely areolate sculpture anteromedially. Mesoscutellum costulate. Metanotum finely areolate. Dorsal face of propodeum coarsely rugose. Femora smooth and shining. Petiole with fine costulae ventrally, and on the dorsal surface of the peduncle; base of node with stronger costulae; apex of node smooth and shining. Postpetiole smooth and shining anteriorly, very finely areolate laterally. First gastral tergite and sternite smooth and shining, without spectral iridescence.

Setae: antennal scapes and funiculi with moderately long, subdecumbent pilosity. Dorsum of the head, pronotum, waist segments, and gaster with abundant, suberect, tapering, flexuous setae, the longest of which are as long as the width of the compound eye. Pubescence present over the entire body, which is nearly as long as the setae.

Color: head and mesosoma predominantly dark brown, nearly black. Antennae, mandibles, promesonotal suture, legs, dorsum of waist segments, and distal margins of gastral sclerites testaceous. Venter of waist segments, basal margins of gastral sclerites, and sting light yellow.

**Male:** Unknown.

**Etymology:** Patronym, presumably in honor of English naturalist Osbert Salvin, who collected in Mesoamerica during the 19^th^ century.

**Comments:** *Temnothorax salvini*, as I now recognize it, is known only from a handful of collections made from mid-to-high elevation cloud forest in Costa Rica and Panama. Jack Longino collected nests from under epiphytes on a tree fall, and additional foraging workers have been collected by beating vegetation. The nest collection contained a single dealate queen. *Temnothorax salvini* is restricted to the southern Central America cordilleras, with its range encompassing the Caribbean and Pacific slopes, with a generally higher elevational range than its sister species *T. longicaulis* stat. nov., nom. nov. (>1,500 m vs. <1,300 m, Figs. 153D & 153I), which is restricted to the Pacific slope. *Temnothorax salvini* and *T. longicaulis* stat. nov., nom. nov. shared a common ancestor around 5 Ma which had dispersed to the southern Central American cordilleras from the Central American Nucleus during a mountain building phase in southern Central America during the Miocene-Pliocene transition (Prebus, 2021). These two species together are closely related to the morphologically similar *T. longinoi* sp. nov., which is restricted to the Central American Nucleus. Like its close relatives in the *salvini* species group, *T. salvini* likely nests and forages arboreally. There is a completely dark form of *T. salvini* from Costa Rica (Figs. 163G–163L).

***Temnothorax terraztecus* sp. nov.**

**Figure 164:**
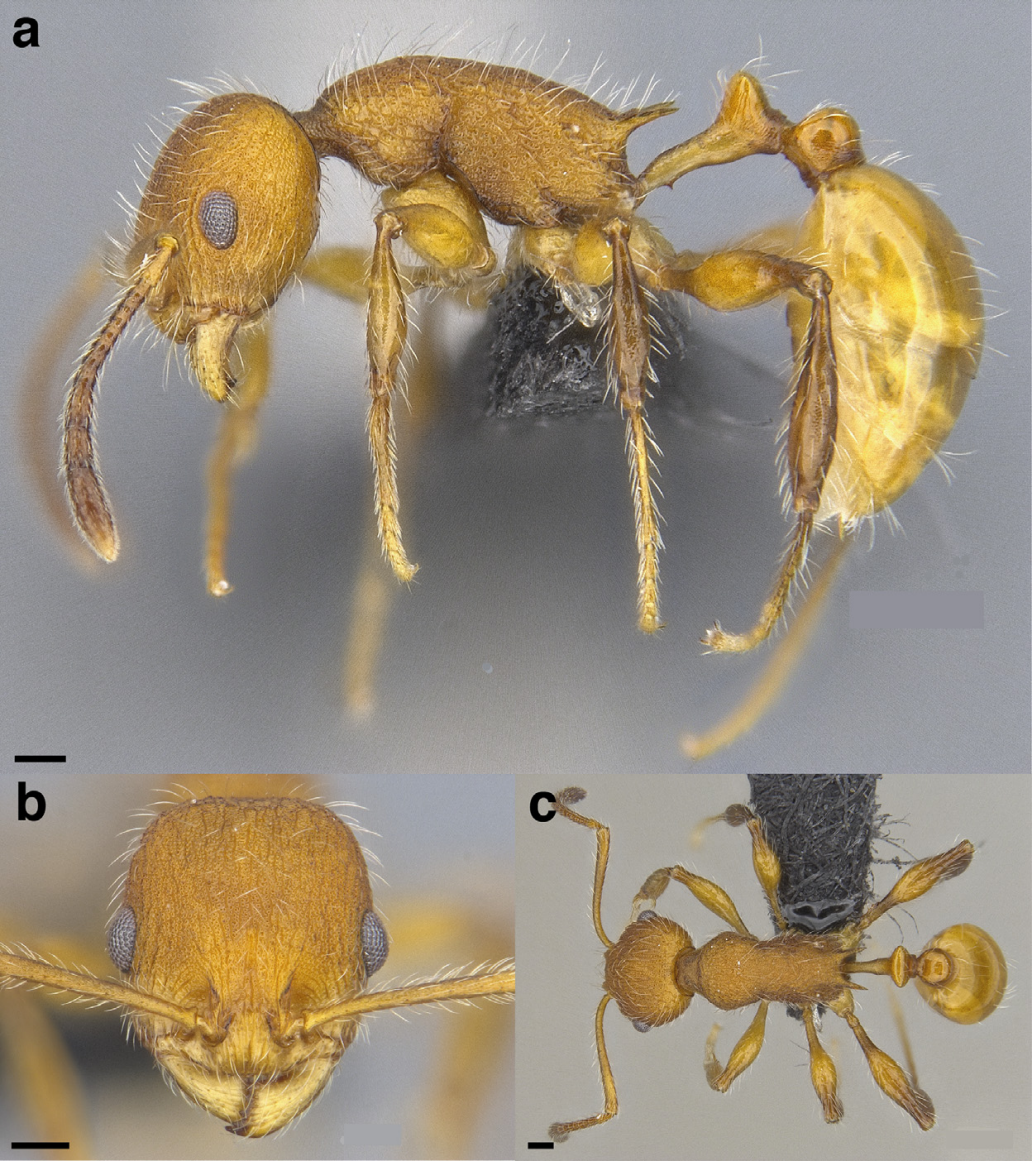
*Temnothorax terraztecus* sp. nov. holotype worker (CASENT0868966). (A) Profile view. (B) Full-face view. (C) Dorsal view. Scale bars 0.2 mm.

Distribution: Fig. 153J; worker: Fig. 164.

**Type material examined:** *Holotype worker:* MEXICO: Chiapas: Nahá, 16.94884°N 91.59484°W ±50 m, 930 m, 8 June 2008, LLAMA Wa-A-07-2-28, mesophyll forest, ex sifted leaf litter, 1 worker (CASENT0868966) [CASC].

*Paratype workers:* same data as holotype, 1 worker (CASENT0868965) [MCZC], 1 worker (CASENT0868824) [UCDC].

**Non-type material examined:** MEXICO: Chiapas: Nahá, 16.94881°N 91.59466°W 930 m, 8 June 2008, LLAMA Wa-A-07-2-24, mesophyll forest, ex sifted leaf litter, 1 worker (JTLC000014496) [USNM].

HONDURAS: Cortés: PN Cusuco, 15.48754°N 88.23678°W ±60 m, 1,220 m, 31 May 2010, LLAMA Wm-C-06-2-03, mesophyll forest, ex sifted leaf litter, 1 worker (CASENT0868823) [UCDC].

**Geographic range:** Low-to-mid elevations, southern Mexico to Honduras (Fig. 153J).

**Worker diagnosis:** *Temnothorax terraztecus* sp. nov. can be separated from all other species in the *salvini* clade by the following character combination: smaller species: WL < 1.17 mm; dorsum of mesosoma arched in profile view; metanotal groove not impressed; propodeum not strongly depressed below the level of the promesonotum; propodeal spines present and about as long as, or slightly longer than, the propodeal declivity; subpetiolar tooth acutely spiniform and shorter than the setae that arises from the peduncle directly above; petiolar node strongly squamiform: in dorsal view, petiolar node greater than 1.6 times as broad as caudal cylinder; dorsum of head predominantly areolate, with weak rugae; dorsum of petiolar node, postpetiole and entire first gastral tergite smooth and shining; setae on head, mesosoma, legs, waist segments and gaster erect to suberect, long, abundant and tapering; integument predominantly yellow to testaceous.

**Similar species:** Fellow members of the *salvini* group. *Temnothorax terraztecus* sp. nov. can be separated from other members of the *salvini* group by the arched dorsal profile of the mesosoma in profile view (flat to slightly sinuate in all other *salvini* group members); squamiform petiolar node (petiolar node less than or equal to 1.5 times as broad as the caudal cylinder in *T. longinoi* sp. nov., *T. quetzal* sp. nov., *T. fortispinosus* sp. nov., *T. parvidentatus* sp. nov., and *T. salvini*); yellow to testaceous integument (*T. longinoi* sp. nov., *T. longicaulis* stat. nov., nom. nov*., T. quetzal* sp. nov., *T. fortispinosus* sp. nov., *T. parvidentatus* sp. nov., and *T. salvini* are variously colored, but never uniformly yellow); smooth dorsum of the petiolar node, postpetiole, and gaster (sculptured in *T. aztecoides* sp. nov.); relatively small subpetiolar tooth (longer than the setae that arises directly above it in *T. aztecus* and *T. paraztecus* sp. nov.); short propodeal spines (longer than the propodeal declivity in *T. aztecoides* sp. nov.).

**Worker measurements & indices (*n* = 5):** SL = 0.704–0.767 (0.748); FRS = 0.213–0.242 (0.226); CW = 0.715–0.845 (0.784); CWb = 0.637–0.733 (0.694); PoOC = 0.280–0.331 (0.309); CL = 0.721–0.819 (0.777); EL = 0.180–0.201 (0.193); EW = 0.120–0.140 (0.126); MD = 0.163–0.198 (0.181); WL = 0.926–1.111 (1.029); SPST = 0.284–0.385 (0.323); MPST = 0.276–0.323 (0.303); PEL = 0.373–0.429 (0.404); NOL = 0.225–0.255 (0.245); NOH = 0.147–0.174 (0.160); PEH = 0.251–0.279 (0.270); PPL = 0.176–0.206 (0.197); PW = 0.468–0.535 (0.505); SBPA = 0.190–0.233 (0.214); SPTI = 0.231–0.275 (0.258); PEW = 0.137–0.159 (0.151); PNW = 0.235–0.297 (0.263); PPW = 0.233–0.261 (0.248); HFL = 0.752–0.884 (0.810); HFWmax = 0.166–0.189 (0.180); HFWmin = 0.057–0.064 (0.061); CS = 0.998–1.143 (1.083); ES = 0.240–0.271 (0.256); SI = 105–111 (108); OI = 23–24 (24); CI = 88–91 (89); WLI = 145–152 (148); SBI = 29–33 (31); PSI = 30–35 (31); PWI = 160–170 (165); PLI = 198–213 (206); NI = 147–158 (153); PNWI = 167–188 (174); NLI = 59–62 (61); FI = 272–316 (298);

**Worker description:** In full-face view, head subquadrate, longer than broad (CI 88–91). Mandibles finely striate but shining and armed with five teeth: the apical-most well developed and acute, followed by a less developed preapical tooth and three equally developed smaller teeth. Anterior clypeal margin weakly convex medially. Antennal scapes very long: when fully retracted, surpassing the posterior margin of the head capsule by about three times the maximum width of the antennal scape (SI 105–111). Antennae 12-segmented; antennal club of composed of three segments, with the apical-most segment slightly longer than the preceding two in combination. Frontal carinae moderately long, extending past the antennal toruli by about two times the maximum width of the antennal scape. Compound eyes strongly protruding past the lateral margins of the head capsule. Lateral margin of head convex, forming a continuous arc from the mandibular insertions to the posterior margin of the head. Posterior head margin flat but rounding evenly into the lateral margins.

In profile view, compound eyes ovular and moderately large (OI 23–24), with 17 ommatidia in longest row. Pronotal declivity indistinct, neck and anterior face of pronotum forming a ~110° angle. Anterior face of pronotum evenly rounding into dorsal face. Dorsum of mesosoma evenly arched from where it joins the anterior face of the pronotum to the propodeal spines. Promesonotal suture extending from the posterior margin of the procoxal insertion only to the mesothoracic spiracle, which is moderately well developed. Metanotal groove visible as a disruption of the sculpture laterally from where it arises between the mid- and hind coxae to where it ends in the poorly developed metathoracic spiracle, which is nearly indistinguishable against the ground sculpture. Propodeal spiracle weakly developed, directed posterolaterally, and separated from the propodeal declivity by about five spiracle diameters. Propodeal spines moderately well developed (PSI 30–35), about as long as or slightly longer than the propodeal declivity, tapering evenly from the base, acute, and straight. Propodeal declivity weakly concave, forming a rounded ~110° angle with the base of the propodeal spines. Propodeal lobes rounded and weakly developed. Metapleural gland bulla small, extending from the metacoxal insertion to a little more than half of the way to the propodeal spiracle. Petiole long (PLI 198–213), without tubercles anterodorsally. Subpetiolar process in the form of a small, spiniform, acute tooth; ventral margin of petiole weakly concave posterior to it but bulging slightly medially. Petiolar peduncle very long: comprising about two thirds of the total petiole length. Petiolar node rounded-squamiform: transition between peduncle and node marked by a rounded angle of ~120°; anterior face rounding evenly into the posterior face, the dorsal margin evenly convex; posterior face forms a ~100° angle with the caudal cylinder. Postpetiole evenly rounded anterodorsally, bulging before flattening posterodorsally; concave ventrally.

In dorsal view, humeri developed and distinct: evenly rounded and slightly wider than the rest of the mesosoma; mesothoracic spiracles protruding past the lateral margins of the mesosoma, visible as slight angles where the pronotum meets the mesonotum. Promesonotal suture visible as a slight disruption in the ground sculpture. Metanotal groove not apparent. Propodeal spines broadly approximated basally and evenly diverging, their apices separated from each other by about their length, the negative space between them “U” shaped. Petiolar peduncle with spiracles slightly protruding past the lateral margins, but not noticeably constricted anterior to them. Petiolar node, when viewed posterodorsally, trapezoidal: much broader apically than basally; apical margin slightly emarginate medially; node broader than the peduncle and the caudal cylinder. Postpetiole narrow (PWI 160–170) and campaniform. Anterior margin of the postpetiole convex, evenly rounding into the lateral margins, which bulge slightly anteriorly, are weakly constricted medially, then diverge slightly again to the angulate posterior corners; posterior margin flat. Metafemur moderately to strongly incrassate (FI 272–316).

Sculpture: median clypeal carina present, extending posteriorly to the frontal triangle, and flanked on either side by multiple slightly weaker carinae. Lateral clypeal lobes with additional, weaker carinae; ground sculpture smooth. Antennal scapes areolate-costulate. Cephalic dorsum areolate with weak overlying rugose sculpture; fine concentric costulae surrounding the antennal insertions. Lateral surfaces of head sculptured similarly to the dorsum, but weak rugae forming whorls around the compound eyes. Ventral surface of head smooth and shining. Pronotal neck areolate. Lateral surfaces of the mesosoma densely rugose, becoming weaker between the propodeal spiracle and the base of the propodeal spine. Propodeal declivity weakly areolate, with weak strigulae. Dorsal surface of mesosoma sculptured similarly to the cephalic dorsum. Femora shining through weak areolate sculpture. Petiole weakly areolate on all surfaces, but sculpture becoming smooth and shining on the node. Postpetiole smooth and shining. First gastral tergite smooth and shining, with weak spectral iridescence. First gastral sternite smooth and shining, without spectral iridescence.

Setae: antennal scapes and funiculi with long, suberect pilosity. Dorsum of the head, pronotum, waist segments, and gaster with abundant, erect, tapering, flexuous setae, the longest of which are about one and a half times the length of the compound eye and are directed toward the midline of the body. The head bears >80, mesosoma >80, petiole ~32, postpetiole ~40, and first gastral tergite >80 setae. Pubescence present over the entire body, which is nearly as long as the setae.

Color: predominantly yellow to testaceous; antennal funiculus, tibiae, and first tarsomere testaceous.

**Etymology:** Behavioral and morphological, from Latin ‘terra-’ (=earth) + ‘aztecus’, in reference to the superficial similarity between this species and *Temnothorax aztecus* and the circumstances of collection.

**Comments:** *Temnothorax terraztecus* sp. nov. is known from several collections made from low-to-mid elevation wet forests in Chiapas, Mexico and Cortés, Honduras. This species is only known from leaf litter extracts, despite collections of other members of the *salvini* group being collected sympatrically from beating vegetation or arboreal nest collections. *Temnothorax terraztecus* sp. nov. may represent the only known non-arboreally nesting member of the *salvini* group.

### *subditivus* group

**Figure 165:**
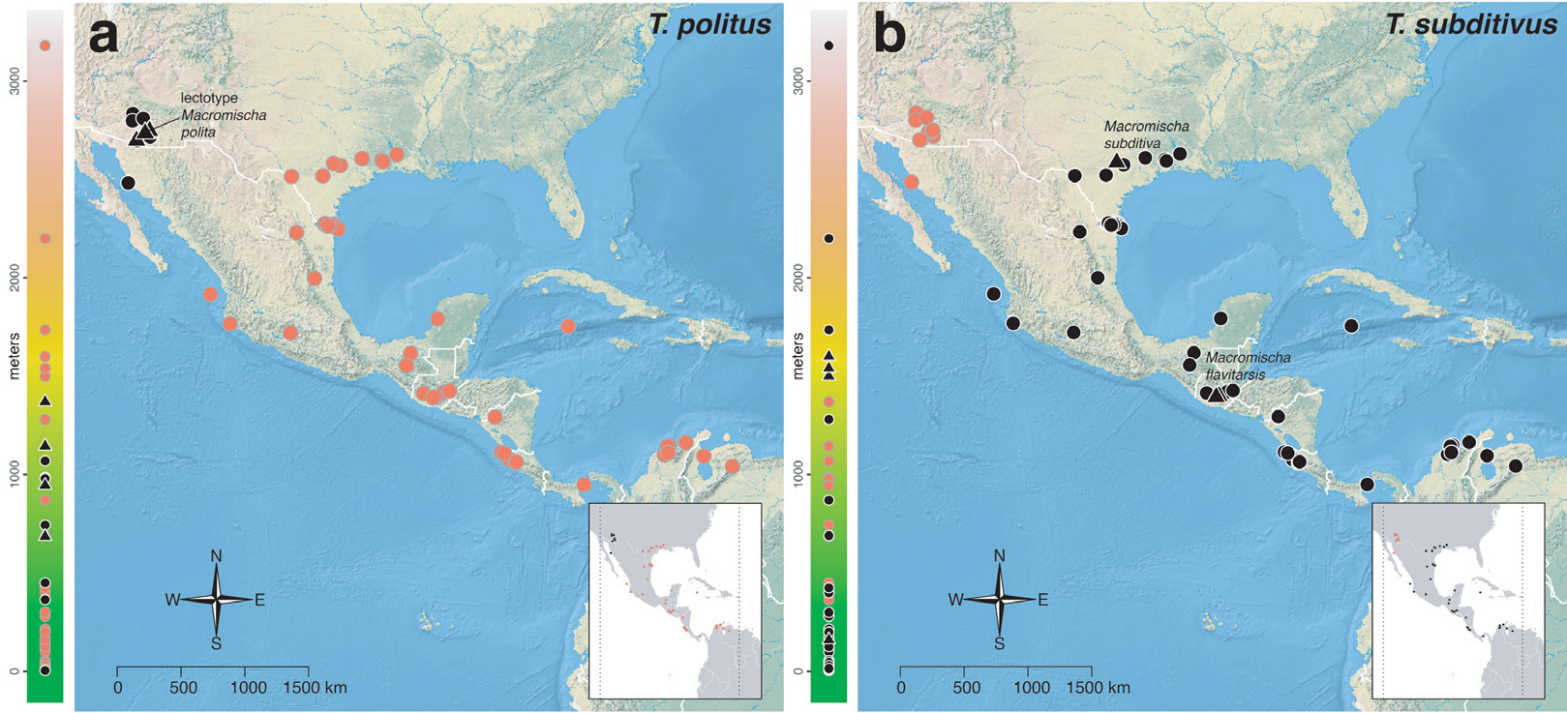
Geographical and elevational distribution of the *subditivus* group. (A) *Temnothorax politus* (B) *T. subditivus*. Colored scale to the left of each map represents elevation in meters. Points in black represent the species named in each subfigure, while points in red represent other members of the species group. Type localities are represented by triangles; if present, types of synonyms are named; non-type localities are represented by circles. Bounding box in inset map shows location of main map.

This group is composed of two species, *Temnothorax politus* and *T. subditivus*, which together have a vast range spanning all elevations, from the southern United States to northern South America (Fig. 165). Typically found nesting in dead vegetation, under bark, or in the leaf litter, the finer details of the biology of this group remain unknown. All nest collections so far have been monogynous. The morphology of *T. subditivus* is extremely variable across its range and may prove to be a species complex with additional sampling and further investigation. As a species group, *T. politus* is easy to distinguish from other Nearctic *Temnothorax*, but the morphological characteristics and range of *T. subditivus* overlap broadly with the *pulchellus* group, the *pastinifer* group, and members of the *sallei* clade with characters of the *Macromischa* syndrome. The keys above and the ‘similar species’ sections below should be used to distinguish it from lookalikes.

***Temnothorax politus* (Smith, 1939)**

**Figure 166:**
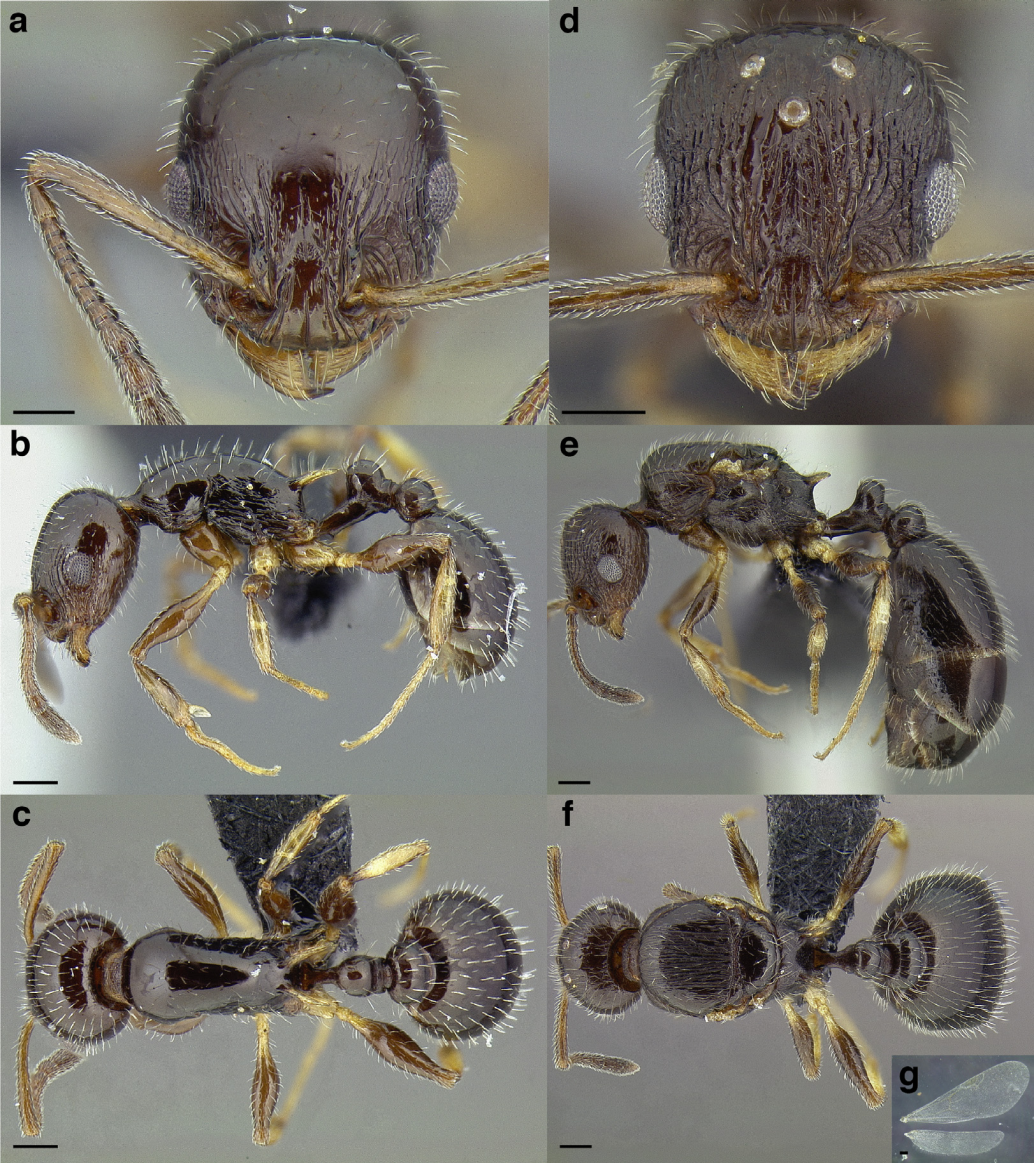
*Temnothorax politus*. (A–C) Worker (CASENT0756845). (A) Full-face view. (B) Profile view. (C) Dorsal view. (D–F) Gyne (CASENT0758021). (D) Full-face view. (E) Profile view. (F) Dorsal view. (G) Wings. Scale bars 0.2 mm.

Distribution: Fig. 165A; worker & gyne: Fig. 166.

*Macromischa polita* Smith, 1939: 506, pl. 1, fig. B. Syntype workers. Arizona, U.S.A. One syntype worker here designated **lectotype**.

*Leptothorax politus* (Smith): Baroni Urbani, 1978: 478, figs. 54 & 104. First combination in *Leptothorax*.

*Temnothorax politus* (Smith): Bolton, 2003: 272. First combination in *Temnothorax*.

**Type material examined:** *Lectotype worker of* Macromischa polita: U.S.A.: Arizona: 6.5 km up Santa Cruz River from Tucson, 13 September 1938, R.G. Wesson, M.C.Z. cotype 23781 (MCZENT00023781, top specimen on pin) [MCZC].

*Paralectotype workers of* Macromischa polita: same pin as lectotype, 1 worker (MCZENT00023781, bottom specimen on pin); Sabino Canyon, 8 October 1938, 945 m, R.G. Wesson#I03 I, 1 worker (No. 53249 U.S.N.M. Cotype, USNMENT00528925) [USNM] 1 worker (No. 53249 U.S.N.M. Cotype, USNMENT00922558) [USNM] 1 worker (CASENT0105625) [USNM]; W of Baboquivari Peak, 50 km E of Sells, 25 September 1938 1,070–1,220 m, R.G. Wesson#I30k, 1 worker (No. 53249 U.S.N.M. Cotype, USNMENT00922559) [USNM]; Catalina Mountains, 11 September 1938, 1,370 m, R.G. Wesson#24j, 2 workers (No. 53249 U.S.N.M. Cotype, LACMENT323200) [LACM].

**Non-type material examined:** MEXICO: Sonora: 9 km NNE Punta Narragansett, Isla Tiburón, 5 m, 28.950000°N 112.216667°W, 15 December 1997, P.S. Ward#13464-6, Sonoran desert, ex spider middens under stone, body parts (CASENT0758314-CASENT0758317) [UCDC]; same data as previous, P.S. Ward#13465-3, Sonoran desert, ex spider middens under stone, 1 head capsule (CASENT0758318) [UCDC]; same data as previous, P.S. Ward#13472-6, Sonoran desert wash, ex middens of ant, *Solenopsis xyloni*?, body parts (CASENT0758319, CASENT0758320) [UCDC].

U.S.A.: Arizona: [no collection locality], [no collection date], R.G. Wesson#99L, 1 worker (CASENT0758294) [USNM]; Pima Co.: Forestry Cabin, Baboquivari Mountains, 1,070 m, 9 April 1963, W.S. Creighton, on mesquite, nest not found, 1 worker (LACMENT323202) [LACM] 1 worker (CASENT0759979) [FSCA]; 4.5 km N Vail, 32.03537°N 110.67832°W ± 9 m, 985 m ± 5 m, 2 May 2016, M.M. Prebus#MMP02493, desert wash, nest in hollow *Populus* twig in leaf litter, 1 dealate gyne & 2 workers (CASENT0756844-CASENT0756846) [MMPC] 1 worker (CASENT0758681) [UCDC]; same data as previous, 32.03607°N 110.67967°W ± 4 m, 985 m ± 5 m, 1 May 2016, M.M. Prebus#MMP02500, desert wash, *Populus* leaf litter, 1 worker (CASENT0756857) [MMPC]; same data as previous, 32.03653°N 110.68096°W ± 5 m, 985 m ± 5 m, 1 May 2016, M.M. Prebus#MMP02501, desert wash, *Populus* leaf litter, 1 worker (CASENT0756864) [MMPC]; same data as previous, 32.04394°N 110.69148°W ± 4 m, 985 m ± 5 m, 1 May 2016, M.M. Prebus#MMP02503, desert wash, *Vachellia* leaf litter, 1 worker (CASENT0756873) [MMPC]; Cienega Creek Preserve, 32.03341°N 110.67424°W ± 5 m, 980 m ± 5 m, 5 Aug 2016, M.M. Prebus#MMP2912, gallery forest, ex dead log in beetle burrows, 1 alate gyne (CASENT0758021) [MMPC]. Pinal Co.: Boyce-Thompson Arboretum, 33.28°N 111.15667°W, 744 m, 15 September 2007, R.A. Johnson#RAJ3991, solitary forager on rock face, 1 worker (CASENT0869117) [RAJC]; Gilliland Ranch, NE of Phoenix, 17 May 1940, Citrus sweeps, Lot No. 40-12517, Lot I, [no collector data], 1 worker (LACMENT323201) [LACM]; 13.24 km NE Maricopa, 33.107150°N 111.918500°W, 365 m, 27 August 2002, Manda G. Cattaneo, PFT 628(B25)-Native, Voucher: EPA Proj. No. X82974701 “Assessment of Environmental Impact of Bt Cotton”, pitfall trap, 1 worker (MCZENT00541469) [MCZC]; same data as previous, PFT 626(B25)-Native, pitfall trap, 1 worker (MCZENT00541470) [MCZC]; same data as previous, PFT 656(B25)-Native, pitfall trap, 1 worker (MCZENT00541471) [MCZC].

**Geographic range:** Low-to-mid elevations, southern Arizona, U.S.A. & Sonora, Mexico (Fig. 165A).

**Worker diagnosis:** *Temnothorax politus* can be separated from all other species in the *salvini* clade by the following character combination: posterior margin of head convex; in profile view, dorsum of mesosoma strongly convex; pronotal declivity not marked by an angle as it transitions from the anterior face of the pronotum to the dorsal face; propodeum not depressed below the level of the promesonotum; propodeal spines longer than the propodeal declivity, directed posterodorsally, and weakly downcurved; hind femora weakly to strongly incrassate; petiolar node node robust and erect, nearly squamiform and dorsally rounded, not leaning posteriorly over the caudal cylinder of the petiole; postpetiole narrow to moderately broad: greater than 1.7 times the width of the caudal cylinder of the petiole; legs and nearly all other surfaces of the body covered in long, tapering, suberect setae; antennal scape moderately long, surpassing the posterior margin of the head by about the maximum width of the antennal scape; integument predominantly dark brown, nearly black.

**Similar species:** Due to the predominantly smooth and shining sculpture, dark coloration, and long, tapering setae, *Temnothorax politus* is unlikely to be confused with any other species in the Nearctic. *Temnothorax tenuisculptus* and less sculptured specimens of *T. subditivus* may be confused with *T. politus*, but both of these have short, blunt setae.

**Worker measurements & indices (*n* = 7):** SL = 0.424–0.555 (0.503); FRS = 0.170–0.235 (0.210); CW = 0.468–0.655 (0.577); CWb = 0.419–0.602 (0.526); PoOC = 0.201–0.282 (0.255); CL = 0.529–0.715 (0.648); EL = 0.121–0.180 (0.150); EW = 0.102–0.129 (0.116); MD = 0.119–0.208 (0.157); WL = 0.581–0.855 (0.744); SPST = 0.213–0.318 (0.276); MPST = 0.207–0.297 (0.247); PEL = 0.239–0.355 (0.302); NOL = 0.150–0.223 (0.179); NOH = 0.094–0.223 (0.133); PEH = 0.165–0.249 (0.216); PPL = 0.114–0.169 (0.143); PPH = 0.132–0.206 (0.168); PW = 0.282–0.431 (0.371); SBPA = 0.108–0.161 (0.143); SPTI = 0.165–0.267 (0.229); PEW = 0.101–0.163 (0.133); PNW = 0.120–0.185 (0.159); PPW = 0.188–0.286 (0.246); HFL = 0.407–0.579 (0.513); HFWmax = 0.098–0.155 (0.136); HFWmin = 0.028–0.061 (0.046); CS = 0.684–0.960 (0.851); ES = 0.172–0.241 (0.208); SI = 92–101 (96); OI = 23–27 (25); CI = 79–84 (81); WLI = 135–147 (141); SBI = 26–28 (27); PSI = 35–39 (37); PWI = 175–192 (185); PLI = 196–250 (212); NI = 100–171 (141); PNWI = 113–127 (120); NLI = 52–67 (60); FI = 243–350 (302).

**Worker description:** In full-face view, head subquadrate, longer than broad (CI 79–84). Mandibles densely, finely striate but shining and armed with five teeth: the apical-most well developed and acute, followed by a less developed preapical tooth and three equally developed smaller teeth. Anterior clypeal margin flat medially. Antennal scapes moderately long: when fully retracted, surpassing the posterior margin of the head capsule by about the maximum width of the antennal scape (SI 92–101). Antennae 12-segmented; antennal club of composed of three segments, with the apical-most segment slightly longer than the preceding two in combination. Frontal carinae moderately long, extending past the antennal toruli by about three and a half times the maximum width of the antennal scape. Compound eyes moderately protruding past the lateral margins of the head capsule. Lateral margin of head weakly convex, forming a continuous arc from the mandibular insertions to the posterior margin of the head. Posterior head margin flat but rounding evenly into the lateral margins.

In profile view, compound eyes ovular and moderately large (OI 23–27), with 12 ommatidia in longest row. Pronotal declivity indistinct, neck and anterior face of pronotum forming a ~120° angle. Mesosoma predominantly evenly convex from where it joins the pronotal neck to the propodeal spines; propodeum very weakly depressed, so that the dorsal margin dips slightly anterior to the base of the propodeal spines. Promesonotal suture extending from the posterior margin of the procoxal insertion only to the mesothoracic spiracle, which is moderately well developed. Metanotal groove visible as a disruption of the sculpture laterally from where it arises between the mid- and hind coxae to where it ends in the poorly developed metathoracic spiracle. Propodeal spiracle moderately well developed, directed posterolaterally, and separated from the propodeal declivity by about four spiracle diameters. Propodeal spines well developed and very long (PSI 35–39), longer than the propodeal declivity, tapering evenly from the base, very weakly downcurved, and acute. Propodeal declivity flat, forming a rounded ~100° angle with the base of the propodeal spines. Propodeal lobes rounded and weakly developed. Metapleural gland bulla small, extending from the metacoxal insertion halfway to the propodeal spiracle. Petiole long (PLI 196–250), without tubercles anterodorsally. Subpetiolar process in the form of a small, acute tooth; ventral margin of petiole nearly flat posterior to it. Petiolar peduncle moderately long: comprising about half of the petiole. Petiolar node robust and erect, nearly squamiform: transition between peduncle and node marked by a rounded angle of ~120°; anterior face forming a rounded ~90° angle with the short, evenly convex dorsal face; dorsal face rounding evenly into the short posterior face, which forms a ~95° angle with the caudal cylinder. Postpetiole evenly rounded anteriorly, flattened dorsally, and weakly lobed ventrally.

In dorsal view, humeri developed: evenly rounded and slightly wider than the rest of the mesosoma; mesothoracic spiracles weakly protruding past the lateral margins of the mesosoma, visible as angles where the pronotum meets the mesonotum. Metanotal groove absent: mesonotum and propodeum completely fused and lateral margins converging evenly to the bases of the propodeal spines. Propodeal spines narrowly approximated basally and diverging apically, their apices separated from each other by slightly less than their length, the negative space between them “V” shaped. Petiolar peduncle with spiracles weakly protruding past the lateral margins, and slightly broader where they arise. Petiolar node nearly evenly transversely ovular, but slightly flattened posteriorly; node wider than the peduncle and the caudal cylinder. Postpetiole narrow (PWI 175–192) and transversely rectangular, articulating with most of the anterior margin of the gaster. Anterior margin of the postpetiole flat and meets the lateral margins at a rounded ~90° angle; lateral margins parallel to the angulate posterior corners; posterior margin flat. Metafemur moderately to strongly incrassate (FI 243–350).

Sculpture: median clypeal carina present, extending posteriorly to the level of the antennal toruli, and flanked on either side by two equally strong carinae. Lateral clypeal lobes with additional, weaker carinae; ground sculpture smooth and shining. Antennal scapes shining through very weak areolate ground sculpture. Cephalic dorsum predominantly smooth and shining except for piligerous punctures, but with fine costulae flanking the frontal carinae medially, and concentric costulae surrounding the antennal insertions. Lateral surfaces of head with costulae over weak areolate sculpture surrounding the compound eyes and between the eyes and the mandibular insertions. Ventral surface of head smooth and shining except for a narrow strip of areolate sculpture surrounding the median sulcus. Mesosoma with weak areolate sculpture on the pronotal neck. Lateral surface of the pronotum smooth and shining except for fine costulae along the ventral margin. Mesopleuron, metapleuron and anterior half of the lateral surface of propodeum predominantly smooth and shining, but with weak costulae. Propodeal declivity smooth and shining. Dorsal surface of mesosoma predominantly smooth and shining, but with weak costulae on the propodeum leading into the bases of the propodeal spines. Femora smooth and shining. Petiole uniformly smooth and shining. Dorsum of postpetiole smooth and shining, with weak areolate sculpture on the posterior quarter. Gaster smooth and shining, without spectral iridescence.

Setae: antennal scapes and funiculi with short, subdecumbent pilosity. Dorsum of the head, pronotum, waist segments, and gaster with abundant, erect, tapering setae, the longest of which are about the length of the compound eye. The dorsum of the head bears ~82, mesosoma ~84, petiole 12, postpetiole ~26, and first gastral tergite ~140 setae. Short, sparse pubescence absent.

Color: predominantly dark brown, nearly black. The mandibles, propodeal spines, coxae, bases of the femora, tibiae, and tarsi testaceous yellow. The antennae and remainder of the femora testaceous brown.

**Gyne measurements & indices (*n* = 1):** SL = 0.620; FRS = 0.294; CW = 0.796; CWb = 0.742; PoOC = 0.316; CL = 0.800; EL = 0.216; EW = 0.175; MD = 0.184; WL = 1.298; SPST = 0.326; MPST = 0.374; PEL = 0.444; NOL = 0.222; NOH = 0.189; PEH = 0.366; PPL = 0.203; PPH = 0.309; PW = 0.761; SBPA = 0.337; SPTI = 0.380; PEW = 0.206; PNW = 0.274; PPW = 0.409; HFL = 0.693; HFWmax = 0.149; HFWmin = 0.057; CS = 1.142; ES = 0.304; SI = 84; OI = 27; CI = 93; WLI = 175; SBI = 45; PSI = 25; PWI = 199; PLI = 219; NI = 117; PNWI = 133; NLI = 50; FI = 261.

**Gyne description:** In full-face view, head subquadrate, slightly longer than broad (CI 93). Mandibles densely, finely striate but shining and armed with five teeth: the apical-most well developed, followed by a less developed preapical tooth and three equally developed smaller teeth. Anterior clypeal margin emarginated medially. Antennal scapes moderately long: when fully retracted, just reaching the posterior margin of the head capsule (SI 84). Antennae 12-segmented; antennal club composed of three segments, with the apical-most segment as long as the preceding two in combination. Frontal carinae moderately long, extending past the antennal toruli by about two and a half times the maximum width of the antennal scape. Compound eyes moderately protruding past the lateral margins of the head capsule. Lateral margin of head evenly convex, converging from below the compound eyes to the mandibular insertions. Posterior head margin convex, rounding evenly into the lateral margins.

In profile view, compound eyes ovular and large (OI 27), with 16 ommatidia in longest row. Mesoscutum rounded evenly anteriorly, covering the dorsal surface of the pronotum, and flat dorsally. Mesoscutellum slightly higher than the level of the mesoscutum. Posterior margin of metanotum extending past the posterior margin of the mesoscutum. Propodeal spiracle well developed, directed posterolaterally, and separated from the propodeal declivity by about four spiracle diameters. Propodeal spines stout and well developed, but short (PSI 25), about as two thirds as long as the propodeal declivity, tapering evenly from the base, directed posteriorly, slightly downcurved, and acute. Propodeal declivity straight and flat, forming a rounded ~100° angle with the base of the propodeal spines. Propodeal lobes rounded and very weakly developed. Metapleural gland bulla small, extending from the metacoxal insertion halfway to the propodeal spiracle. Petiole long (PLI 219), without tubercles anterodorsally. Subpetiolar process in the form of a small, very acute tooth; ventral margin of petiole nearly flat. Petiolar peduncle moderately long: comprising half the length of the petiole. Petiolar node robust and erect: transition between peduncle and node marked by a rounded angle of ~130°; anterior face forming a rounded ~90° angle with the short, evenly convex dorsal face; dorsal face rounding evenly into the short posterior face, which forms a ~110° angle with the caudal cylinder. Postpetiole evenly rounded anterodorsally, bulging before it transitions into the flattened dorsal face; ventral surface weakly lobed.

In dorsal view, mesoscutum covering pronotum anteriorly, but humeri visible laterally as rounded sclerites. Propodeal spines diverging apically, their apices separated from each other by about two times their length. Petiolar peduncle with spiracles protruding past the lateral margins, the peduncle broadened where they arise. Petiolar node transversely ovular. Petiolar node wider than the peduncle, and evenly grading into the caudal cylinder, which is slightly narrower than the node. Postpetiole narrow (PWI 199) and transversely rectangular, articulating with most of the anterior margin of the gaster, leaving angulate margins on each side exposed. Anterior margin of postpetiole very weakly convex, nearly convex with corners marked by rounded angles as it transitions to the lateral margins, which are parallel to the angulate posterior corners; posterior margin flat. Metafemur moderately incrassate (FI 261).

Sculpture: median clypeal carina present, extending from the anterior margin nearly to the level of the antennal toruli; lateral margins of median clypeal lobe with two carinae that are slightly weaker than the medial carina. Lateral clypeal lobes with additional weaker carinae; ground sculpture weakly areolate. Antennal scapes shining through weak areolate sculpture. Cephalic dorsum rugose-costate, over weak areolate ground sculpture; concentric costulae surrounding the antennal insertions. Lateral surfaces of head sculptured similarly to the dorsum of the head. Ventral surface of head with rugulae over very weak areolate ground sculpture. Pronotal neck areolate. Pronotum with costulae, becoming very weak medially. Anepisternum and katepisternum smooth and shining medially, but with weak costulae along the margins of the sclerites. Metapleuron and lateral face of the propodeum costate. Area between the propodeal spiracle and base of propodeal spines, as well as the propodeal declivity, smooth and shining. Propodeal declivity weakly areolate. Mesoscutum costulate, with smooth and shining patches laterally. Mesoscutellum smooth and shining medially, surrounded by weak costulae and areolae laterally. Femora shining, with traces of weak areolate sculpture. Petiole with very fine areolate sculpture ventrolaterally, around the base of the petiolar node. Longitudinal carina running from the petiolar spiracle to the caudal cylinder. Dorsal surface of the node with weak costulae. Dorsum of postpetiole weakly areolate anteriorly, posterior margin weakly strigulate. Sclerites of the gaster shining but dulled by very fine areolae, as found on the ventrolateral surface of the petiole. Spectral iridescence absent.

Setae: antennal scapes and funiculi with short, subdecumbent pilosity. Dorsum of the head, pronotum, waist segments, and gaster with abundant, erect, tapering setae, the longest of which are about the width of the compound eye. Short, sparse pubescence absent.

Color: predominantly dark brown, nearly black. The mandibles, propodeal spines, coxae, bases of the femora, tibiae, and tarsi testaceous yellow. The antennae and remainder of the femora testaceous brown.

**Male:** Unknown.

**Etymology:** Morphological, from the Latin ‘politus’ (= polished), in reference to the smooth and shining integument of this species.

**Comments:** *Temnothorax politus* is known only from the hot deserts and sky islands of southern Arizona and Sonora, Mexico. In desert habitats, it is apparently restricted to washes, irrigation canals, and other riparian habitats (Smith, 1939). In the original description, it was noted that Wesson collected this species at higher elevations in the Santa Catalina Mountains near Tucson, but no habitat data was associated with this occurrence. Smith (1939) also recorded that this species nests under the bark of cottonwood trees near the ground; I have personally collected several nests from ~12 cm diameter fallen cottonwood branches in gallery forest, as well as ~3 cm diameter hollow twigs in cottonwood leaf litter on the banks of a desert wash. This species is probably active in the nocturnal or crepuscular hours, at least in warm desert habitats: pitfall traps that I left overnight in Cienega Creek Preserve near Vail, Arizona yielded several workers, although I observed none foraging diurnally in the pitfall localities. The type series consisted of multiple localities across Arizona. Here, I designate a specimen from the Santa Cruz river near Tucson as the lectotype, leaving the remainder of the type series as paralectotypes.

***Temnothorax subditivus* (Wheeler, 1903a)**

**Figure 167:**
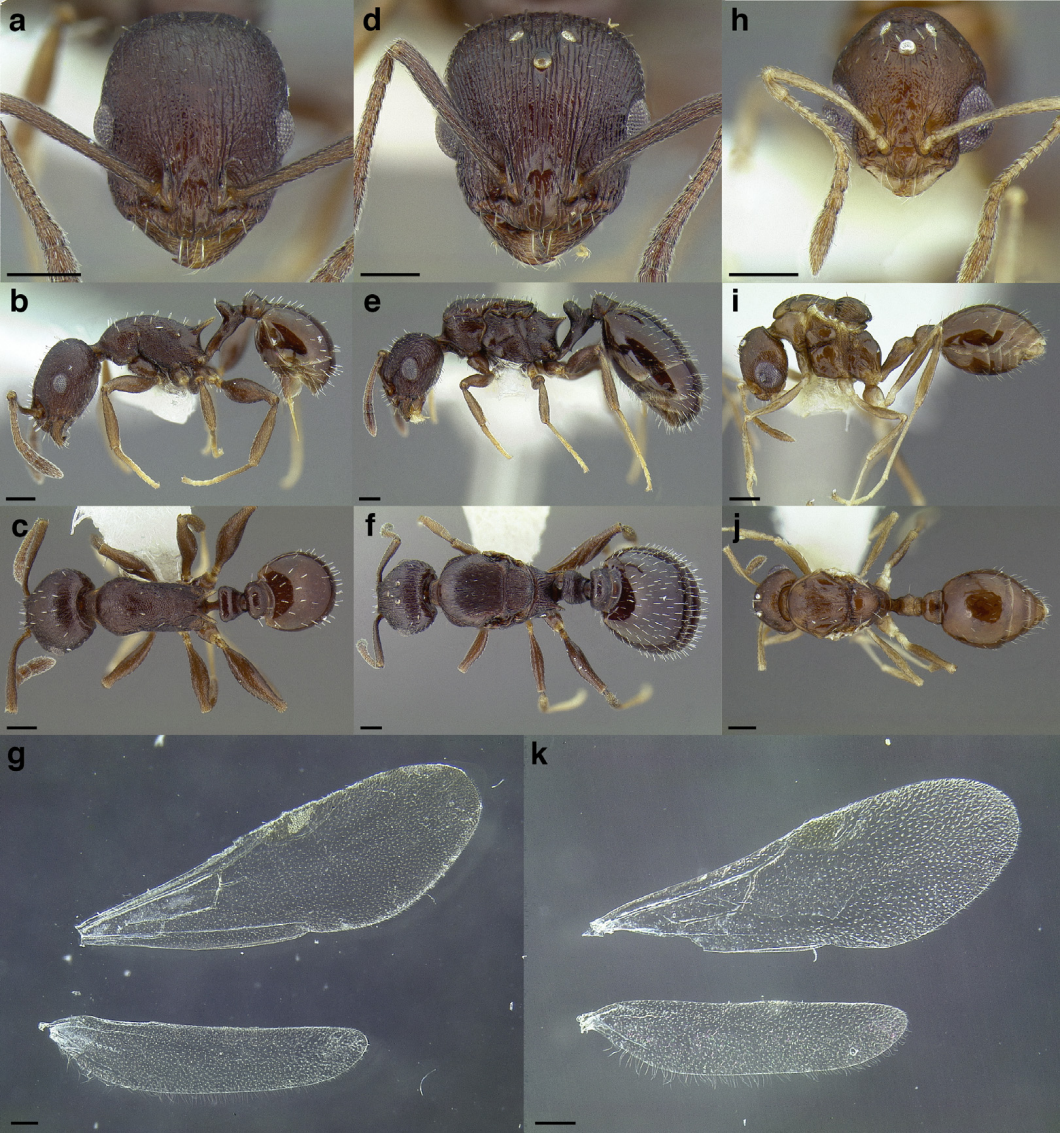
*Temnothorax suditivus*. (A–C) Worker (CASENT0758340). (A) Full-face view. (B) Profile view. (C) Dorsal view. (D–G) Gyne (CASENT0758338). (D) Full-face view. (E) Profile view. (F) Dorsal view. (G) Wings. (H–K) Male (CASENT0915978). (H) Full-face view. (I) Profile view. (J) Dorsal view. (K) Wings. Scale bars 0.2 mm.

**Figure 168:**
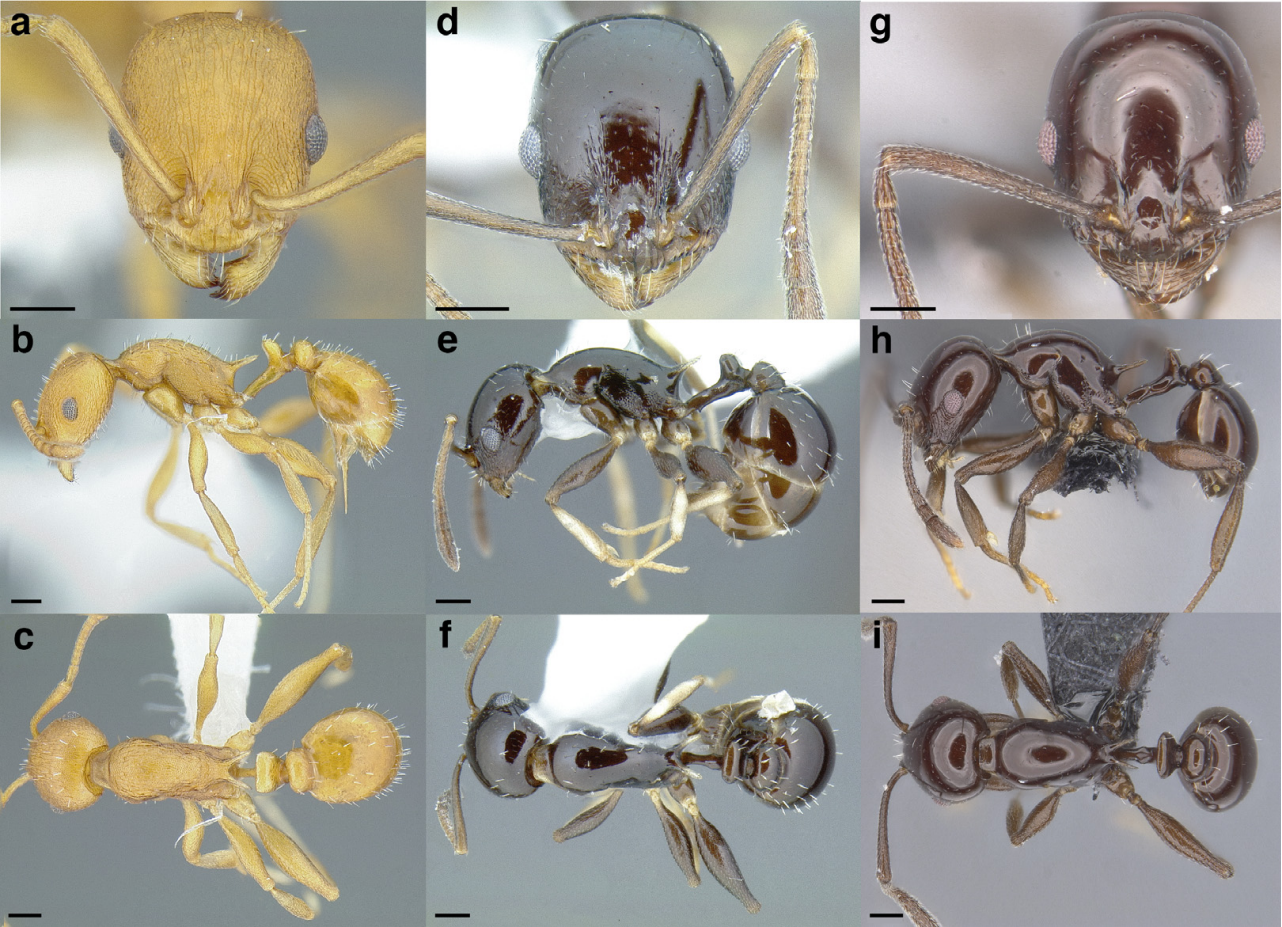
Morphological variability in *Temnothorax suditivus* workers. (A–C) Estelí, Nicaragua (CASENT0619368). (A) Full-face view. (B) Profile view. (C) Dorsal view. (D–F) Jalisco, Mexico (CASENT0758299). (D) Full-face view. (E) Profile view. (F) Dorsal view. (G–I) Grand Cayman, Cayman Islands (CASENT0868474). (G) Full-face view. (H) Profile view. (I) Dorsal view. Scale bars 0.2 mm.

Distribution: Fig. 165B; worker, gyne & male: Fig. 167; variability: Fig. 168.

*Macromischa subditiva* Wheeler, 1903a: 99, fig. 5. Syntype workers. Texas, U.S.A. One syntype worker here designated **lectotype**. Wheeler, 1931: 15 (Gyne and male of *Macromischa flavitarsis* Mann), Smith, 1939: 503. (Male).

*Macromischa laevissima* Wheeler, 1911: 205. Holotype worker. Veracruz, Mexico. Junior synonym of *Temnothorax subditivus* by Baroni Urbani, 1978: 512.

*Macromischa (Macromischa) subditiva* (Wheeler): Mann, 1920: 408. First combination in *Macromischa (Macromischa)*.

*Macromischa (Macromischa) flavitarsis* Mann, 1920: 420. Syntype workers and gynes. Guatemala. Junior synonym of *Temnothorax subditivus* by Baroni Urbani, 1978: 512. One syntype worker here designated **lectotype**.

*Macromischa luciliae* Mann, 1935: 35, fig. 1. Syntype workers. Guatemala. Junior synonym of *Temnothorax subditivus* by Baroni Urbani, 1978: 512. One syntype worker here designated **lectotype**.

*Leptothorax subditivus* (Wheeler): Baroni Urbani, 1978: 512. First combination in *Leptothorax*.

*Temnothorax subditivus* (Wheeler): Bolton, 2003: 272. First combination in *Temnothorax*.

*Temnothorax* mmp14 Prebus, 2017: 8. Phylogeny.

**Type material examined:** *Lectotype worker of* Macromischa subditiva: U.S.A.: Texas: Travis County: Austin, 15 May 1903, on bark of *Salix nigra*, Type No. A.M.N.H., M.C.Z. Type 2-1 16371 (MCZENT00016371, middle specimen on pin) [MCZC].

*Paralectotype workers of* Macromischa subditiva: same pin as lectotype, 2 workers (top and bottom specimens on pin) [MCZC]; same data as lectotype, 3 workers (MCZENT00578574) [MCZC]; [no locality data], [no collection date], W.M. Wheeler, Type No. A.M.N.H., Type 16371, 1 worker (MCZENT00578573) [MCZC].

*Lectotype worker of* Macromischa flavitarsis: GUATEMALA: Sacatepéquez: Antigua, 24 December 1911, W.M. Wheeler, M.C.Z. Cotype 16368, (MCZENT00016368, top specimen on pin) [MCZC].

*Paralectotype workers and gynes of* Macromischa flavitarsis: same pin as lectotype, (bottom specimen on pin) [MCZC]; same data as lectotype, 3 alate gynes (MCZENT00534455) [MCZC]; Guatemala City, 17 December 1911, W.M. Wheeler, 5 workers, (USNMENT00921848) [USNM] 3 workers (USNMENT00921847) [USNM] 5 workers (USNMENT00529208) [USNM]; Antigua, 21 December 1911, M.C.Z. Cotype 16368, 3 workers (MCZENT00534456) [MCZC]; Antigua, 20 December 1911, W.M. Wheeler, 3 workers (LACMENT323232) [LACM].

*Lectotype worker of* Macromischa luciliae: [no department], intercepted at Hololulu, Hawaii, U.S.A. [quarantine], 20 October 1932, on *Oncidium splendidum* roots from Guatemala cargo, Cotype 50802 U.S.N.M. (images of USNMENT00529613 examined on the Smithsonian Type Specimen Database).

**Type material not examined:** *Macromischa laevissima holotype worker:* MEXICO: Veracruz: La Buena Ventura, near Santa Rosa, A. Petrunkewitch [AMNH].

**Non-type material examined:** CAYMAN ISLANDS: Grand Cayman: 1 km up Mastic trail, 19.32000°N 81.19800°W, 5 m, 8 March 2008, #32, J.K. Wetterer, 1 worker (CASENT0868784) [UCDC] 1 worker (CASENT0868474) [MMPC].

COLOMBIA: La Guajira: Caribbean Region, 2 km S Rio Hacha, 18 January 1937, P. Hummelinck#294, 1 worker (MCZENT00583614) [MCZC]; Magdalena: Santa Marta 40 m, 11.250000°N 74.216667°W, 15 August 1985, P.S. Ward#7940, tropical thorn woodland, dead stalk of *Manihot* sp., 1 alate gyne, 1 dealate gyne & 1 worker (CASENT0758338) [PSWC] 1 male & 2 workers (CASENT0756100) [PSWC]; Villa Culebra, near Bonda, 10 km E Santa Marta, October 1985, H.G. Müller, low vegetation, 2 workers (CASENT0758339) [PSWC]; Bahia de Neguangue, Tayrona Pk., ca 25 km NE Santa Marta, 8 October 1985, H.G. Müller, low vegetation, 2 workers (CASENT0758340) [PSWC]; same data as previous, 30 September 1985, H.G. Müller, 3 workers (CASENT0758344) [PSWC]; Bahia Concha, Tayrona Pk., 10 km NE Santa Marta, 23 June 1985, H.G. Müller, ex vegetation, 3 workers (CASENT0758341) [PSWC]; mouth of Rio Fundación, Cienaga Grande de Sta Marta, 10.716667°N 74.416667°W, 29 August 1985, H.G. Müller, 3 workers (CASENT0758342) [PSWC]; Bahia de Cinto, Parque Nacional Tayrona, 30 km E Santa Marta 11.333333°N 74.066667°W, 14 April 1986, H.G. Müller, 1 worker (CASENT0758343) [PSWC]; Punta de Betin, Santa Marta, October 1985, 11.250000°N 74.216667°W, H.G. Müller, low vegetation, 2 workers (CASENT0758345) [PSWC] 1 alate gyne (CASENT0756101) [PSWC]; Punta de Betin, Santa Marta, November-December 1985, 11.250000°N 74.216667°W, H.G. Müller, 2 workers (CASENT0758346) [PSWC]; same data as previous, pan traps, 2 workers (CASENT0758347) [PSWC]; Punta de Betin, Santa Marta, January-March 1986, 11.250000°N 74.216667°W, H.G. Müller, 2 workers (CASENT0758348) [PSWC]; Bahia de Neguange, Parque Nacional Tayrona, 25 km NE Santa Marta 11.350000°N 74.083333°W, 16 June 1985, H.G. Müller, 1 worker (CASENT0758349) [PSWC]; 2 km E Orihueca, 10.850000°N 74.150000°W, 20 m, 17 Aug 1985, J. Longino#845, 1 dealate gyne, 1 worker (LACMENT141164) [UCDC] 1 worker (LACMENT141165) [JTLC] 2 workers (LACMENT323176) [LACM] 3 workers (CASENT0869131–3) [MMPC].

COSTA RICA: Guancaste: Isla San José, 10.866667°N 85.900000°W, 100 m, 22 October 1992, D.M. Olson, 3 workers (CASENT0758337) [UCDC]; S. Naranjo ridge, Santa Rosa National Park, 10.8°N 85.68333°W, 100 m, 14 July 1985, J. Longino#JTL0461,xeric scrub on rocky ridge, nest in dead branch <0.5 m high, 1 dealate gyne, 1 male, 2 workers (INBIOCRI002281233–4) [JTLC] 1 male & 3 workers (CASENT0869127–30) [MMPC]; Paloverde National Park, August 1984, I.H. Rathet, ex *Acacia* thorn, 3 workers (LACMENT323177) [LACM]; Puntarenas: Guacimal, road to Monteverde, 10.21667°N 84.85°W, 400 m, 20 July 1984, J. Longino#JTL20Jul84/2, roadside vegetation, strays from tree trunks, 1 worker (INBIOCRI002281232) [JTLC]; La Pita, 10.16°N 84.90°W, 200 m, 13 July 1984, J. Longino#JTL13Jul84/pm, sweep sample, 1 worker (INBIOCRI002281229) [LACM]; La Pita, road to Monteverde, 10.16667°N 84.91667°W, 120 m, 13 July 1984, J. Longino#JTL13Jul84/am, sweeping vegetation, 2 workers (INBIOCRI002281230, INBIOCRI002281231) [JTLC].

GUATEMALA: El Progreso: San Agustin Acasaguastlan, near road to San Cristobal Acasaguastlan, 14.91777°N 89.94683°W, 280 m, 18 June 2015, Z. Falin#GUAT1F15149, fogging *Pereskia* trunk/foliage, 1 worker (JTL-SV00859.3) [JTLC]. Esquintla: Origin: San Jose, in quarantine, San Francisco, California, U.S.A., 18 April 1949, ex *Odontoglossum grande*, 1 worker (LACMENT323230) [LACM]. Guatemala: Origin: Guatemala City, quarantine, San Francisco, California, U.S.A., 25 April 1947, ex *Oncidium splendidum*, 1 male (LACMENT323223) [LACM] 1 dealate gyne (LACMENT323226) [LACM] 1 worker (LACMENT323227) [LACM]; [no locality data], in quarantine, San Francisco, California, U.S.A., 25 March 1947, ex *Oncidium splendidum*, 3 workers (MCZENT00581856) [MCZC]. Totonicapan: Xeabaj I, road between antennae, 14.83126°N 91.40299°W, 3.180 m, 7 June 2015, Z. Falin#GUAT1F15049, under bark, *Pinus* stump, 1 worker (CASENT0636941) [JTLC]. Zacapa: Zacapa, finca nr. Univ. Landivar campus, 14.99336°N 89.54607°W, 200 m, 21 June 2015, Z. Falin#GUAT1F15188, flight intercept trap, 1 worker (JTL-SV00862.3) [JTLC].

MEXICO: Campeche: 10 km E Campeche, 28–29 August 1953, E.O. Wilson#141, 2 workers (MCZENT00581851) [MCZC]; Chiapas: 8 km SE Salto de Agua, 17.51615°N 92.30164°W ± 300 m, 100 m, 100 m, 16 June 2008, LLAMA#Go-A-08-2-04, 2° wet forest, beating vegetation, 1 worker (CASENT0609888) [UCDC]; 6 km SE San Cristobal on Las Casas, 2,195 m, 10 August 1978, John E. Rawlins, 1 worker (MCZENT00581852) [MCZC]; Jalisco: Estación De Biolgía Chamela, 19.497778°N 105.044167°W, 135 m, 31 March–4 April 2012, D. Dubovikoff#CHUNAM1, 1 worker (CASENT0758297) [UNAM] 1 worker (CASENT0758298) [UNAM] 1 worker (CASENT0758299) [UNAM]; Michoacán: 8 km SW Tiquicheo, 425 m, 8 July 1970, E. Fisher & P. Sullivan, 1 dealate gyne (LACMENT323468) [LACM]; Nayarit: Maria Magdalena, Isla Tres Marias, 23 March 1964, R.R. Snelling, 1 worker (LACMENT323229) [LACM]. Nuevo León: Chipinque Mesa, Monterrey, 1,280 m, 21 January 1952, W.S. Creighton, in *Quercus fusiformis*, 3 workers (LACMENT323234) [LACM] 3 workers (CASENT0759986) [FSCA]; Tamaulipas: Antigue Morelos, 9 July 1969, S.&J. Peck#Ber167, Berlese of palm-thorn forest litter, 1 dealate gyne (MCZENT00583613) [MCZC]; Veracruz: Mirador, 16 March 1929, E. Skwarra#Z154a/Sk., *Conostegia xalapensis*, 3 workers (MCZENT00581853) [MCZC]; same data as previous, except: 18 April 1929, E. Skwarra#Z196c/Sk., 1 dealate gyne (MCZENT00581854) [MCZC]; same data as previous, except: 3 May 1929, E. Skwarra#Z333/Sk., *Conostegia xalapensis*, 3 males (MCZENT00581855) [MCZC]; same data as previous, except: 4 May 29, E. Skwarra, 3 workers (LACMENT323231) [LACM]; same data as previous, except: E. Skwarra#Z348/SK, 3 males & 2 alate queens (MCZENT00730929-30) [MCZC].

NICARAGUA: Estelí: 16 km N Estelí, 13.24082°N 86.35307°W ± 100 m, 870 m, 25 April 2011, J. Longino#JTL7420-s, tropical dry scrub, strays, 1 worker (CASENT0619627) [JTLC]; same data as previous, except: J. Longino#JTL7421-s, tropical dry scrub, at bait, 3 workers (CASENT0619366-CASENT0619368) [JTLC].

PANAMA: Panamá, Juan Díaz, 1938, N.A. Weber#775, 2 workers (LACMENT323178) [LACM].

U.S.A.: Louisiana: Beauregard Parish: DeRidder, 24 June 1942, W.F. Buren, 3 workers (CASENT0867155) [FSCA]; same data as previous, except: 10 July 1942, W.F. Buren, 1 alate gyne, 3 males, 8w (CASENT0867156-CASENT0867159) [FSCA]; same data as previous, except: 3 September 1942, W.F. Buren, 3 workers (LACMENT323224) [LACM]. Texas: Bastrop County: Bastrop State Park, 21 September 1979, P. Ward#3825-2, honey bait on ground, pine-oak forest, 1 worker (CASENT0758323) [UCDC]; Bexar County: San Antonio, 28 April 1979, P.S. Ward#3298, foraging on low vegetation, 1 worker (CASENT0758324) [UCDC]; Cameron County: Brownsville, 24 October 1942, [no collector data], 4 workers (LACMENT323225) [LACM]; La Feria, *Pandora* stem, 16 April 1973, R.R. Snelling#RRS73-12, 3 workers (LACMENT323228) [LACM]; La Feria, 26 January 1973, R.M. Duffield, 3 workers (CASENT0750538-CASENT0750540) [FSCA]; Resaca de la Palma S.P., 25.99596°N 97.56622°W ± 50 m, 10 m, 5 November 2013, R. & C. Anderson#RSA2013TX05, *Celtis*, ex sifted leaf litter, 1 worker (JTL713352) [JTLC]; Sabal Palm Grove Sanctuary, 25.84994°N 97.41850°W ± 50 m, 10 m, 4 November 2013, R. & C. Anderson#RSA2013TX02, hardwood forest, ex sifted leaf litter, 1 worker (CASENT0635918) [JTLC]; In quarantine from Harlingen, San Bernadino, San Bernadino County, California, 12 November 1961, Young et al. California Department of Agriculture#61215-15, ex *Washingtonia robusta*, 3 workers (LACMENT323233) [LACM]; Hardin County: 4.75 km E of US69/287, 0.30 km N of FM 420, 15 September 1979, S.P. Lewis#SPL89, 1 worker (MCZENT00730931) [MCZC]; 2.06 km W of Silsbee, 0.4 km N of Texas 327, 15 September 1979, S.P. Lewis#SPL97, 1 worker (MCZENT00730932) [MCZC]; same data as previous, except: S.P. Lewis#SPL99, 1 worker (MCZENT00730933) [MCZC]; Hidalgo County: Bentsen Rio Grande State Park 12–13 March 1980, semi-dry deciduous forest, P.S. Ward#3928, by water hole, on rotten log, 1 worker (CASENT0758328) [UCDC]; Bentsen Rio Grande State Park, 26.18308°N 98.38243°W ± 50 m, 35 m, 5 November 2013, R. & C. Anderson#RSA2013TX06, dry cedar elm, ex sifted leaf litter, 1 worker (JTL713359) [JTLC]; Mission, 30 m, 15 December 1984, P.S. Ward#7185, roadside edge, ex dead twig of undetermined tree, 1 dealate gyne, 1 worker & 1 male (CASENT0915978) [PSWC] 2 workers (CASENT0758327) [UCDC] 1 worker (CASENT0758702) [UCDC]; Progreso, 11 January 1966, W.S. Creighton, 1 dealate gyne, 3 workers (CASENT0759985) [FSCA]; Santa Ana National Wildlife Refuge, 30 m, 14 December 1984, P.S. Ward#7167-2, subtropical thorn woodland, on low vegetation, 1 worker (CASENT0758326) [UCDC]; same data as previous, except: 13 December 1984, J. Longino#JTL0047, mesic woodland, nest in dead stick, 1 worker (LACMENT141166) [JTLC] 3 workers (CASENT0869120 -2) [MMPC]; same data as previous, except: 14 December 1984, J. Longino#JTL0076, dead stalks of *Arundo donax* along riverbank, single-queen colony, 1 worker (JTL0002485) [JTLC] 1 dealate gyne & 3 workers (CASENT0869123-6) [MMPC]. Kinney County: Sycamore Creek at Hwy90, 29.38333°N 100.7°W, 300 m, 16 December 2004, J. Longino#JTL5400-s, live oak woodland, sifted leaf litter, 1 worker (JTLC000006433) [JTLC]. Travis County: Austin, 15 April 1979, P.S. Ward#PSW3234, dead twig of *Morus*, 5 workers (CASENT0758322, CASENT0758336) [UCDC]; Brackenridge Field Station, Austin 19 June 1978, D.H. Feener#Z715b, 1 male & 1 worker (MCZENT00581859) [MCZC] same data as previous, Z714b, Z713 b, 1 alate gyne & 1 worker (MCZENT00581858) [MCZC]; same data as previous, 21 August 1981, P.S. Ward#5133, on *Ulmus* trunk, semi-deciduous woodland, 2 workers (CASENT0758325) [UCDC]; Walker County, Sam Houston National Forest, 18 km SW junction Route 30 on Farm Road 1791, 2 May 1993, S.P. Cover#3495, mature shortleaf-loblolly pine forest, 15–18 m tall with well-developed mixed deciduous understory to 12 m, in standing pine bark (stump remnant) in shade, 2 workers (MCZENT00581857) [MCZC].

VENEZUELA: Lara: Quibor, Jimenez, 8 July 1979, R.W. Brooks, A.A. Grigarick, J. McLaughlin, R.O. Schuster, 1 male (CASENT0758329) [UCDC]. Zulia: Maracaibo vic., 1941, R.G. Wesson, dry thorn scrub, 6 workers (MCZENT00730934–6) [MCZC].

**Geographic range:** all elevations, southern U.S.A. to Colombia & Venezuela, including the Cayman Islands (Fig. 165B).

**Worker diagnosis:** *Temnothorax subditivus* can be separated from all other species in the *salvini* clade by the following character combination: posterior margin of head convex; antennal scape moderately long, surpassing the posterior margin of the head by about the maximum width of the antennal scape; in profile view, dorsum of mesosoma strongly convex; pronotal declivity not marked by an angle as it transitions from the anterior face of the pronotum to the dorsal face; propodeum not depressed below the level of the promesonotum; propodeal spines as long as or longer than the propodeal declivity, directed posterodorsally, and straight; hind femora moderately to strongly incrassate; petiolar node node robust and erect, squamiform, and not leaning posteriorly over the caudal cylinder of the petiole; postpetiole moderately broad: greater than 1.7 times the width of the caudal cylinder of the petiole; setae on head, mesosoma, waist segments and gaster erect, moderately long, sparse and blunt (never long and tapering).

**Similar species:** *Temnothorax subditivus* is extremely variable across its vast range, and may be confused with *T. goniops, T. pilicornis* sp. nov., *T. tenuisculptus*, and members of the *augusti* and *pastinifer* groups. It can be separated from all of the above, except the *pastinifer* group, by the extremely squamiform petiolar node, which in dorsal view is much broader than the caudal cylinder of the petiole. When viewed dorsally, the closely approximated propodeal spines, the bases of which are united by a transverse carina, will distinguish *T. subditivus* from all members of the *pastinifer* group.

**Worker measurements & indices (*n* = 15):** SL = 0.528–0.704 (0.610); FRS = 0.184–0.237 (0.209); CW = 0.569–0.712 (0.645); CWb = 0.525–0.657 (0.592); PoOC = 0.257–0.322 (0.285); CL = 0.644–0.810 (0.717); EL = 0.153–0.198 (0.172); EW = 0.117–0.146 (0.130); MD = 0.138–0.189 (0.164); WL = 0.745–1.003 (0.836); SPST = 0.247–0.368 (0.296); MPST = 0.255–0.318 (0.283); PEL = 0.301–0.417 (0.346); NOL = 0.168–0.240 (0.207); NOH = 0.100–0.174 (0.142); PEH = 0.201–0.277 (0.243); PPL = 0.120–0.166 (0.144); PPH = 0.148–0.206 (0.183); PW = 0.361–0.470 (0.419); SBPA = 0.117–0.167 (0.144); SPTI = 0.179–0.300 (0.247); PEW = 0.124–0.163 (0.143); PNW = 0.212–0.298 (0.251); PPW = 0.246–0.32 (0.285); HFL = 0.574–0.837 (0.670); HFWmax = 0.138–0.208 (0.168); HFWmin = 0.049–0.066 (0.055); CS = 0.848–1.062 (0.951); ES = 0.213–0.269 (0.237); SI = 95–109 (103); OI = 24–26 (25); CI = 79–86 (83); WLI = 134–153 (141); SBI = 21–28 (24); PSI = 30–45 (35); PWI = 179–216 (199); PLI = 218–273 (241); NI = 117–210 (148); PNWI = 160–208 (175); NLI = 53–70 (60); FI = 261–359 (304).

**Worker description:** In full-face view, head subquadrate, slightly longer than broad (CI 79–86). Mandibles densely, finely striate but shining and armed with five teeth: the apical-most well developed and acute, followed by a less developed preapical tooth and three equally developed smaller teeth. Anterior clypeal margin evenly convex medially. Antennal scapes moderately long: when fully retracted, surpassing the posterior margin of the head capsule by slightly more than the maximum width of the scape (SI 95–109). Antennae 12-segmented; antennal club of composed of three segments, with the apical-most segment longer than the preceding two in combination. Frontal carinae long, extending past the antennal toruli by about three and a half times the maximum width of the antennal scape. Compound eyes moderately protruding past the lateral margins of the head capsule. Lateral margin of head moderately convex, forming a continuous arc from the mandibular insertions to the posterior margin of the head. Posterior head margin convex, rounding evenly into the lateral margins.

In profile view, compound eyes ovular and moderately large (OI 24–26), with 14 ommatidia in longest row. Pronotal declivity indistinct, but neck and anterior face of pronotum forming a ~120° angle. Mesosoma predominantly evenly convex from where it joins the pronotal neck to the propodeal spines; propodeum very weakly depressed, so that the dorsal margin dips slightly anterior to the base of the propodeal spines. Promesonotal suture extending from the posterior margin of the procoxal insertion only to the mesothoracic spiracle, which is weakly developed. Metanotal groove visible as a disruption of the sculpture laterally from where it arises between the mid- and hind coxae to where it ends in the poorly developed metathoracic spiracle, which is nearly indistinguishable against the ground sculpture. Propodeal spiracle poorly developed, directed posterolaterally, and separated from the propodeal declivity by about five spiracle diameters. Propodeal spines well developed and moderately long (PSI 30–45), about as long as the propodeal declivity, flared at the base, straight, directed posterodorsally, and acute. Propodeal declivity flat, forming a rounded ~110° angle with the base of the propodeal spines. Propodeal lobes rounded and weakly developed. Metapleural gland bulla small, extending from the metacoxal insertion halfway to the propodeal spiracle. Petiole long (PLI 218–273), without tubercles anterodorsally. Subpetiolar process in the form of a small, acute tooth, the ventral face of the petiole weakly bulging below the petiolar node. Petiolar peduncle very long: comprising about three quarters of the petiole. Petiolar node squamiform: node arising abruptly from the petiolar peduncle, forming a rounded ~100° angle with it; anterior face rounding evenly into the very short dorsal face, which is strongly convex; dorsal face rounding evenly into the posterior face, which forms a ~90° angle with the caudal cylinder. Postpetiole evenly rounded anteriorly, flattened dorsally, and lobed ventrally. Sting extremely well developed: longer than the first gastral sternite.

In dorsal view, humeri well developed and distinct: rounded and wider than the rest of the mesosoma; mesothoracic spiracles weakly protruding past the lateral margins of the mesosoma, visible as slight angles where the pronotum meets the mesonotum. Metanotal groove absent: mesonotum and propodeum completely fused and lateral margins converging evenly to the bases of the propodeal spines. Propodeal spines narrowly approximated basally and joined by a transverse welt; diverging apically, their apices separated from each other by about their length, the negative space between them “V” shaped. Petiolar peduncle with spiracles slightly protruding past the lateral margins. Petiolar node squamiform: transversely ovular when viewed dorsally; node much wider than the peduncle, and a little less than twice as broad as the caudal cylinder. Postpetiole narrow to moderately broad (PWI 179-216) and campaniform, articulating with most of the anterior margin of the gaster, but leaving angulate corners of the gaster exposed on each side. Anterior margin of the postpetiole weakly convex, nearly flat, and evenly rounding into the lateral margins, which diverge slightly to the angulate posterior corners; posterior margin broadly concave. Metafemur moderately to strongly incrassate (FI 261–359).

Sculpture: median clypeal carina present, extending posteriorly to the level of the antennal toruli, and flanked on either side by two equally strong carinae. Lateral clypeal lobes with additional, weaker carinae; ground sculpture shining through weak costulae. Antennal scapes dully shining through weak areolate ground sculpture. Cephalic dorsum predominantly with weak costae, but with indications of weak areolae laterally; median strip and posterior half of the head smooth and shining. Lateral surfaces shallowly areolate, but with stronger rugae surrounding the compound eye, and between the eye and the mandibular insertion. Ventral surface of head smooth and shining, with weak costulae. Mesosoma with areolate sculpture on the pronotal neck. Lateral faces of mesosoma with weak areolae and costulae, which become stronger on the lateral face of the propodeum and meso- and metapleuron. Propodeal declivity shining through shallow areolae. Dorsal surface of pronotum with very weak costulae and areolae, which becomes stronger laterally; the border between the pronotum and mesonotum indicated by a transverse strip of stronger sculpture. Remainder of the mesosoma smooth and shining medially, but with areolate sculpture laterally and with a transverse strip of areolate sculpture directly anterior to the base of the propodeal spines. Femora weakly shining through shallow areolate sculpture. Petiole and postpetiole predominantly smooth and shining; dorsum of the postpetiole with a strip of shallow areolate sculpture along the posterior border. Gaster smooth and shining, without spectral iridescence.

Setae: antennal scapes and funiculi with short, decumbent pilosity. Dorsum of the head, pronotum, waist segments, and gaster with abundant, erect, blunt-tipped setae, the longest of which are about the width of the compound eye. The head dorsum bears ~44, mesosoma ~34, petiole 10, postpetiole ~18, and first gastral tergite ~82 setae. Finer, blunt setae are present on the ventral surface of the head, propleuron, procoxae, and ventral surface of the gaster. Short, sparse pubescence present over the entire body, but is difficult to detect against the dense sculpture.

Color: Predominantly testaceous brown; tarsi and sting yellow.

**Gyne measurements & indices (*n* = 3):** SL = 0.603–0.703 (0.644); FRS = 0.242–0.265 (0.253); CW = 0.732–0.812 (0.782); CWb = 0.673–0.752 (0.722); PoOC = 0.292–0.33 (0.305); CL = 0.765–0.838 (0.795); EL = 0.225–0.254 (0.244); EW = 0.166–0.196 (0.182); MD = 0.165–0.176 (0.170); WL = 1.187–1.320 (1.251); SPST = 0.307–0.402 (0.345); MPST = 0.345–0.379 (0.367); PEL = 0.390–0.440 (0.420); NOL = 0.209–0.288 (0.243); NOH = 0.181–0.195 (0.188); PEH = 0.323–0.342 (0.331); PPL = 0.180–0.224 (0.206); PPH = 0.269–0.305 (0.290); PW = 0.671–0.820 (0.741); SBPA = 0.339–0.408 (0.369); SPTI = 0.349–0.406 (0.370); PEW = 0.185–0.243 (0.212); PNW = 0.297–0.323 (0.307); PPW = 0.403–0.441 (0.416); HFL = 0.706–0.800 (0.740); HFWmax = 0.146–0.168 (0.157); HFWmin = 0.051–0.059 (0.055); CS = 1.056–1.160 (1.120); ES = 0.308–0.352 (0.335); SI = 83–95 (89); OI = 29–30 (30); CI = 88–96 (91); WLI = 166–178 (173); SBI = 48–55 (51); PSI = 25–30 (28); PWI = 166–218 (199); PLI = 192–217 (204); NI = 115–148 (129); PNWI = 133–163 (146); NLI = 53–67 (58); FI = 264–300 (284).

**Gyne description:** In full-face view, head subquadrate, slightly longer than broad (CI 88–96). Mandibles densely striate, dull, and armed with five teeth: the apical-most well developed, followed by a four roughly equally developed smaller teeth. Anterior clypeal margin very slightly emarginate medially. Antennal scapes moderately long: when fully retracted, just reaching the posterior margin of the head capsule (SI 83–95). Antennae 12-segmented; antennal club of composed of three segments, with the apical-most segment as long as than the preceding two in combination. Frontal carinae long, extending past the antennal toruli by about four times the maximum width of the antennal scape. Compound eyes moderately protruding past the lateral margins of the head capsule. Lateral margin of head evenly convex, converging evenly to the mandibular insertions. Posterior margin of head weakly convex, rounding evenly into the lateral margins.

In profile view, compound eyes ovular and large (OI 29–30), with 18 ommatidia in longest row. Mesoscutum rounded evenly anteriorly, covering the dorsal surface of the pronotum, and flat dorsally. Mesoscutellum on the same level as the mesoscutum; posterior margin of metanotum extending past the posterior margin of the mesoscutellum. Propodeal spiracle weakly developed, directed posterolaterally, and separated from the propodeal declivity by about five spiracle diameters. Propodeal spines stout and well developed, but short (PSI 25–30), about half as long as the propodeal declivity, flared at the base, weakly downcurved, directed posteriorly, and acute. Propodeal declivity straight and flat, forming a rounded ~100° angle with the base of the propodeal spines. Propodeal lobes rounded and very weakly developed. Metapleural gland bulla small, extending from the metacoxal insertion halfway to the propodeal spiracle. Petiole long (PLI 192–217), without tubercles anterodorsally. Subpetiolar process in the form of a weakly developed, acute tooth, which grades evenly into the ventral margin of the petiole posteriorly. Petiolar peduncle very long: comprising about two thirds of the total length of the petiole. Petiolar node squamiform: node arising abruptly from the petiolar peduncle, forming a rounded ~120° angle with it; anterior face rounding evenly into the very short dorsal face, which is strongly convex; dorsal face rounding evenly into the posterior face, which forms a ~90° angle with the caudal cylinder. Postpetiole evenly rounded anteriorly, dorsal face bulging anterodorsally before flattening posteriorly; ventral surface lobed.

In dorsal view, mesoscutum covering pronotum anteriorly, but humeri visible laterally as rounded sclerites. Propodeal spines diverging basally, but parallel distally, their apices separated from each other by about two and a half times their length. Petiolar peduncle with spiracles protruding past the lateral margins, the peduncle broadened where they arise. Petiolar node squamiform: transversely oval when viewed dorsally; node much wider than the peduncle, and a about one and a half times as broad as the caudal cylinder. Postpetiole narrow to moderately broad (PWI 166–218), anteroposteriorly compressed, and subquadrate, articulating with most of the anterior margin of the gaster, leaving small, angulate margins on each side of the gaster exposed. Anterior margin of postpetiole very weakly convex, nearly flat, with corners evenly rounding into the lateral margins, which evenly diverge to the angulate posterior corners; posterior margin weakly concave. Metafemur moderately incrassate (FI 264–300).

Sculpture: median clypeal carina present, extending nearly to the frontal triangle, and flanked by additional weaker carinae over weakly areolate ground sculpture. Antennal scapes areolate and weakly shining. Cephalic dorsum densely costate, over weak areolate sculpture. Lateral surface of head with denser rugae forming whorls around the compound eye and forming transverse reticulations between the compound eye and the mandibular insertions. Ventral surface of head weakly costulate. Pronotal neck areolate. Pronotum, propodeum, and posterior two thirds of the anepisternum and katepisternum sculptured like the cephalic dorsum; anterior thirds of anepisternum and katepisternum smooth and shining. Propodeal declivity shallowly areolate. Mesoscutum costulate. Mesoscutellum costulate, with a patch of smooth and shining sculpture posteromedially. Femora shining through weak areolate sculpture. Petiole weakly areolate, with the anterior face of the node smooth and shining, and the dorsum transversely striate. Dorsal surface of postpetiole shallowly areolate. Gaster smooth and shining, only faint traces of spectral iridescence on the first gastral tergite and sternite.

Setae: antennal scapes and funiculi with short, decumbent pilosity. Dorsum of the head, pronotum, waist segments, and gaster with abundant, erect, blunt-tipped setae, the longest of which are about a third of the width of the compound eye. Finer, blunt setae are present on the ventral surface of the head, propleuron, procoxae, and ventral surface of the gaster. Short, sparse pubescence present over the entire body, but is difficult to detect against the dense sculpture.

Color: predominantly testaceous brown; tarsi yellow.

**Male measurements & indices (*n* = 3):** SL = 0.368–0.421 (0.402); FRS = 0.107–0.142 (0.126); CW = 0.505–0.537 (0.519); CWb = 0.439–0.473 (0.455); PoOC = 0.219–0.234 (0.225); CL = 0.503–0.529 (0.514); EL = 0.217–0.228 (0.222); EW = 0.175–0.191 (0.184); MD = 0.033–0.051 (0.040); WL = 0.810–0.877 (0.841); SPST = n/a; MPST = 0.268–0.295 (0.280); PEL = 0.273–0.289 (0.282); NOL = 0.151–0.186 (0.167); NOH = 0.039–0.072 (0.056); PEH = 0.134–0.161 (0.145); PPL = 0.123–0.154 (0.140); PPH = 0.170–0.181 (0.174); PW = 0.511–0.558 (0.537); SBPA = n/a; SPTI = n/a; PEW = 0.131–0.184 (0.149); PNW = 0.157–0.219 (0.182); PPW = 0.248–0.276 (0.261); HFL = 0.596–0.679 (0.634); HFWmax = 0.076–0.090 (0.083); HFWmin = 0.044–0.053 (0.048); CS = 0.694–0.738 (0.712); ES = 0.310–0.321 (0.314); SI = 81–96 (89); OI = 42–46 (44); CI = 86–90 (88); WLI = 179–190 (185); SBI = n/a; PSI = n/a; PWI = 150–195 (178); PLI = 188–222 (203); NI = 258–387 (313); PNWI = 119–127 (122); NLI = 55–64 (59); FI = 158–205 (175).

**Male description:** In full-face view, head subglobular, slightly longer than broad (CI 86–90). Mandibles smooth and shining and armed with five teeth: the apical-most well developed and acute, followed by a less developed preapical tooth and three equally developed smaller crenulae. Anterior clypeal margin evenly rounded medially. Antennal scapes moderately long: when fully retracted, just reaching the posterior margin of the head capsule (SI 81–96). Antennae 13-segmented; antennal club of composed of three segments, with the apical-most segment as long as than the preceding three in combination. Frontal carinae long, extending past the antennal toruli by about four times the maximum width of the antennal scape. Compound eyes strongly protruding past the lateral margins of the head capsule. Posterior margin of head strongly convex, not differentiated from lateral margins.

In profile view, compound eyes ovular and very large (OI 42–46), with 22 ommatidia in longest row. Mesoscutum rounded evenly anteriorly, covering the dorsal surface of the pronotum, and flat dorsally. Mesoscutellum inflated and much higher than the mesoscutum; posterior margin of mesoscutellum overhanging the posterior margin of the metanotum. Propodeal spiracle weakly developed, directed posterolaterally, and separated from the propodeal declivity by about four spiracle diameters. Propodeal spines absent. Propodeum evenly rounded. Propodeal lobes rounded and very weakly developed. Metapleural gland bulla small, extending from the metacoxal insertion halfway to the propodeal spiracle. Petiole long (PLI 188–222), without tubercles anterodorsally. Subpetiolar process absent; ventral margin of petiole weakly bulging medially. Petiolar peduncle very long: comprising about two thirds of the total length of the petiole. Petiolar node low and cuneiform: node evenly grading into the petiolar peduncle anteriorly; anterior face meeting the posterior face at a ~90° angle. Postpetiole evenly rounded anteriorly, flattened dorsally; ventral surface concave.

In dorsal view, mesoscutum covering pronotum anteriorly; humeri barely visible laterally. Propodeal spines absent. Petiolar peduncle with spiracles protruding past the lateral margins, the peduncle broadened where they arise. Petiolar node cuneiform: dorsum flat when viewed posterodorsally, and narrower than the caudal cylinder. Postpetiole narrow (PWI 150–195) and campaniform, articulating with most of the anterior margin of the gaster, leaving angulate margins on each side of the gaster exposed. Anterior margin of postpetiole weakly concave, nearly flat, with corners evenly rounding into the lateral margins, which evenly diverge to the angulate posterior corners; posterior margin weakly concave. Metafemur not incrassate (FI 158–205).

Sculpture: median clypeal carina present, extending nearly the level of the antennal toruli, and flanked by additional weaker carinae over smooth ground sculpture. Antennal scapes weakly areolate and shining. Cephalic dorsum densely areolate, with costulae radiating from the frontal triangle. Lateral surface of head with denser rugae forming whorls around the compound eye. Ventral surface of head weakly areolate. Pronotal neck weakly areolate. Pronotum smooth and shining. Anterior half of the katepisternum and lateral portions of the anepisternum smooth and shining, otherwise areolate. Metapleuron areolate. Lateral face of propodeum areolate; propodeal declivity smooth and shining. Median strip of mesoscutum costulate over weak areolate sculpture; smooth and shining laterally. Mesoscutellum smooth and shining medially; lateral faces costulate over weak areolate sculpture. Femora shining through weak areolate sculpture, which becomes stronger distally. Petiole weakly areolate, with the anterior face of the node smooth and shining. Dorsal surface of postpetiole smooth and shining, with posterior quarter shallowly areolate. Gaster smooth and shining, without spectral iridescence.

Setae: antennal scapes and funiculi with short, decumbent pilosity. Dorsum of the head, pronotum, waist segments, and gaster with abundant, erect, blunt-tipped setae, the longest of which are about a third of the width of the compound eye. Finer, blunt setae are present on the ventral surface of the gaster. Short, sparse pubescence present over the entire body, but is difficult to detect against the sculpture.

Color: predominantly testaceous brown; mandibles, antennae, coxae, tarsi, and genitalia yellow.

**Etymology:** Systematic, from the Latin ‘subditivus’ (= spurious, counterfeit). W.M. Wheeler originally assigned this species to *Macromischa* with reservations; the species epithet is likely a reflection of his doubts.

**Comments:** *Temnothorax subditivus* has the largest range of any *salvini* clade species, which encompasses much of southern North America, all of Mesoamerica, parts of northern South America, and the Cayman Islands. *Temnothorax subditivus* is also highly morphologically variable, with forms that are uniformly dark brown to uniformly yellow, densely sculptured to mostly smooth and shining (Fig. 168). *Temnothorax subditivus* inhabits dry forests and disturbed habitats from sea level to high elevations, and is often found nesting in hollow, dead vegetation, under the bark of trees, or in epiphytes, but may also be found in rock crevices or abandoned termite burrows (this study; Achury, Chacón de Ulloa & Arcila, 2008; Creighton, 1965; Escalante-Jiménez & Vasquez-Bolaños, 2015; Gutiérrez-Martínez & Naranjo, 2016; Pérez-Sánchez, Lattke & Viloria Petit, 2012; Rojas, Fragoso & Mackay, 2014; Wheeler, 1903a). Colonies are small, with 12 to 145 workers, and monogynous (Creighton, 1965). They are apparently trophic generalists, like many *Temnothorax* for which food preference is known, but favour insects (Creighton, 1965). The male pupae bear bizarre appendages with unknown function (Schumann, 1992). *Temnothorax subditivus* is most closely related to *T. politus* of the Sonoran Desert. The high degree of morphological variability and broad range have led taxonomists to assign a large number of names to this species, but the phylogeny of this species shows a great deal of geographical structure, with a pectinate series from the northern part of the range to the southern part (Prebus in prep.). This pattern could be attributed to either isolation by distance or to a recent radiation of closely related species. Increased sampling along with species delimitation analysis across the range of *T. subditivus* should elucidate whether this taxon represents a broadly distributed morphologically and ecologically variable species or a species complex.

### *tenuisculptus* group overview

**Figure 169:**
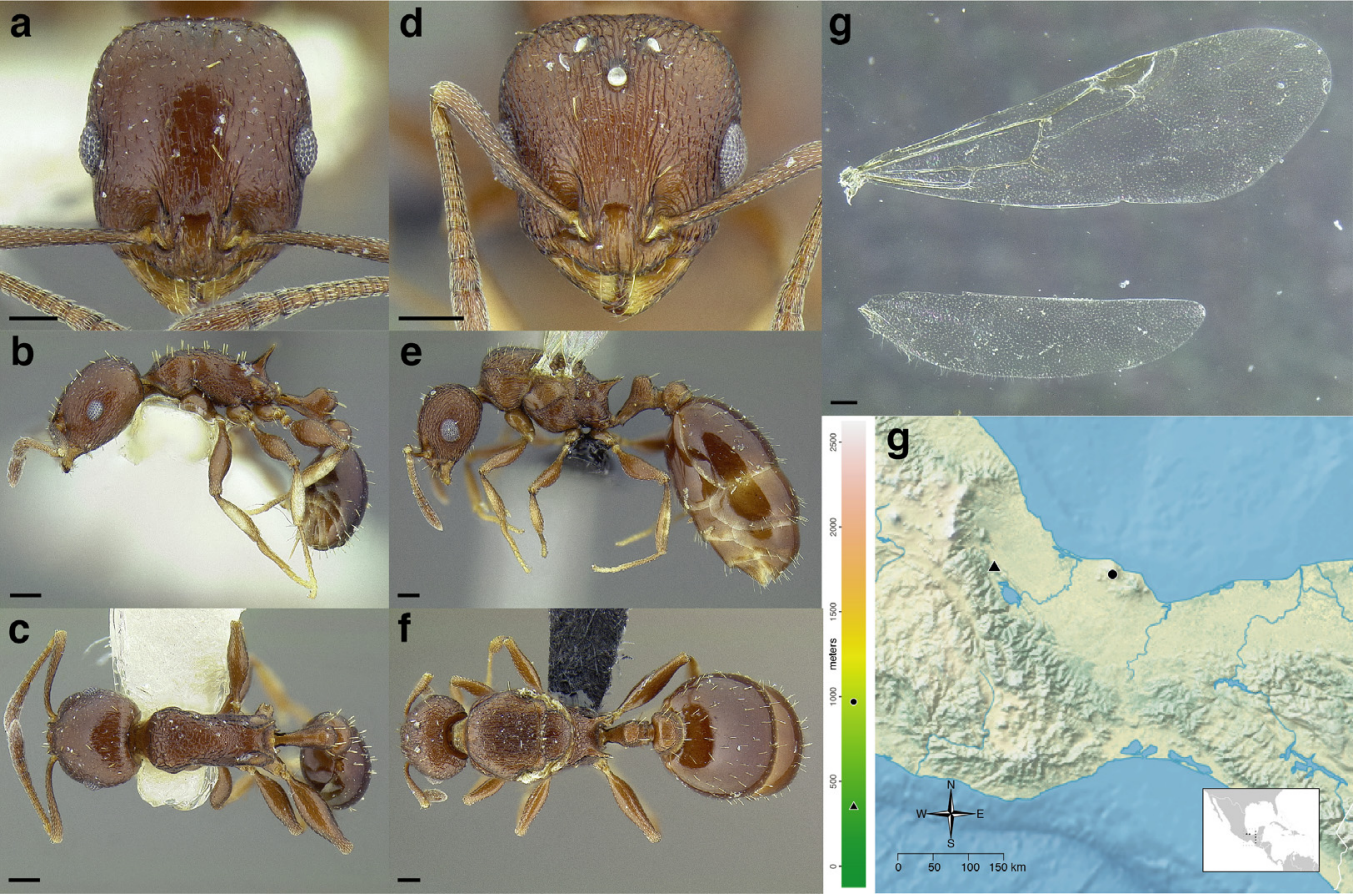
*Temnothorax tenuisculptus*. (A–C) Holotype worker (MCZENT00032435). (A) Full-face view. (B) Profile view. (C) Dorsal view. (D–G) Gyne (CASENT0756078). (D) Full-face view. (E) Profile view. (F) Dorsal view. (G) Wings. (H) Geographical and elevational distribution of specimens examined. Type locality is represented by a triangle, non-type localities are represented by circles. Bounding box in inset map shows location of main map. Scale bars 0.2 mm.

This group is monotypic, with the nominal *Temnothorax tenuisculptus* being the only member. The range of this species as we currently understand it is small, restricted to the low elevations of the Southern Sierra Madre and Los Tuxtlas in southern Mexico (Fig. 169H). It is morphologically similar to the members of the *augusti* group and is contained within its geographical range. Unlike the members of the *augusti* group, the petiolar node of *T. tenuisculptus* is erect, not leaning posteriorly. This species is sister to the *annexus* group (Prebus, in prep.).

***Temnothorax tenuisculptus* (Baroni Urbani, 1978)**

Distribution, worker & gyne: Fig. 169.

*Leptothorax tenuisculptus* Baroni Urbani, 1978: 517. Holotype worker. Pueblo Nuevo, Veracruz, Mexico.

*Temnothorax tenuisculptus* (Baroni Urbani): Bolton, 2003: 272. First combination in *Temnothorax*.

**Type material examined:** *Holotype worker:* MEXICO: Veracruz: Pueblo Nuevo, near Tetzonapa, E.O. Wilson 9 August 1953, rainforest, 1 worker (M.C.Z. Type 32435, MCZENT00032435, top specimen on pin) [MCZC].

*Paratype workers:* same pin as holotype, 1 worker (bottom specimen on pin) [MCZC]; same data as holotype, 1 worker (M.C.Z. Type 32435, MCZENT00578578) [MCZC] 1 worker (images of CASENT0913005 examined on antweb.org) [NHMB].

**Non-type material examined:** MEXICO: San Luis Potosí: [quarantine], 13 April 1976, J. Kline & R. Eads #ANTC43726, *Quercus* sp. with *Epidendrum* sp. orchid plant, 7 alate gynes & 3 workers (CASENT0756076-CASENT0756084) [USNM]. Veracruz: 9 km NE San Andrés Tuxtla, 18.51232°N 95.16729°W ± 4 m, 970 m ± 5 m 4 June 2016, M.M. Prebus#MMP02671, mixed tropical temperate mesic forest, between leaves and stalk of *Tillandsia*, 4 workers (CASENT0757196-CASENT0757198, CASENT0758683) [UCDC].

**Geographic range:** Low elevations of southern Mexico (Veracruz) (Fig. 169H).

**Worker diagnosis:** *Temnothorax tenuisculptus* can be separated from all other species in the *salvini* clade by the following character combination: in profile view, dorsum of mesosoma strongly sinuate; propodeum depressed below the level of the promesonotum; hind femora strongly incrassate; petiolar node subquadrate, not leaning posteriorly; postpetiole moderately broad, greater than 1.5 times the width of the caudal cylinder of the petiole, but less than 2.2 times; setae on head, mesosoma, waist segments and gaster erect, moderately long, sparse and blunt (never long and tapering);integument predominantly testaceous brown, with apex of mandibles, pedicel of antenna, pronotal neck, tibiae, tarsi, and sting testaceous yellow.

**Similar species:** *Temnothorax acutispinosus* sp. nov., *T. tuxtlanus* sp. nov., *T. xincai* sp. nov., members of the *augusti* and *subditivus* groups. *Temnothorax tenuisculptus* can be separated from all of the above species, except for the *augusti* and *subditivus* groups by the strongly incrassate hind femora. The petiolar node, which is erect in *T. tenuisculptus*, can be used to distinguish it from similar appearing members of the *augusti* group, which have posteriorly leaning petiolar nodes. Due to the smooth integument of the head, *T. tenuisculptus* may be confused with *T. politus*, but the short, blunt tipped setae and sinuate mesosoma of the former will separate it from *T. politus*, which has long, tapering setae and an arched mesosoma. Similarly, *T. tenuisculptus* may be mistaken for some of the more lightly sculptured specimens of *T. subditivus* but differs from it by the sinuate mesosoma (uniformly convex in *T. subditivus*) and the subquadrate petiolar node (squamiform in *T. subditivus*).

**Worker measurements & indices (*n* = 9):** SL = 0.501–0.592 (0.554); FRS = 0.171–0.205 (0.191); CW = 0.575–0.698 (0.654); CWb = 0.516–0.643 (0.604); PoOC = 0.255–0.312 (0.283); CL = 0.647–0.767 (0.720); EL = 0.134–0.169 (0.154); EW = 0.096–0.127 (0.114); MD = 0.158–0.189 (0.170); WL = 0.748–0.919 (0.857); SPST = 0.216–0.317 (0.267); MPST = 0.244–0.320 (0.279); PEL = 0.300–0.388 (0.353); NOL = 0.147–0.199 (0.176); NOH = 0.119–0.159 (0.139); PEH = 0.196–0.255 (0.222); PPL = 0.156–0.215 (0.178); PW = 0.372–0.456 (0.420); SBPA = 0.133–0.194 (0.166); SPTI = 0.205–0.274 (0.250); PEW = 0.131–0.164 (0.153); PNW = 0.136–0.202 (0.170); PPW = 0.234–0.288 (0.266); HFL = 0.535–0.668 (0.621); HFWmax = 0.151–0.199 (0.180); HFWmin = 0.042–0.056 (0.050); CS = 0.840–1.023 (0.964); ES = 0.182–0.230 (0.211); SI = 89–97 (92); OI = 21–23 (22); CI = 80–86 (84); WLI = 138–145 (142); SBI = 23–30 (27); PSI = 27–34 (31); PWI = 167–180 (174); PLI = 174–233 (199); NI = 111–147 (128); PNWI = 94–126 (111); NLI = 40–57 (50); FI = 348–392 (361).

**Worker description:** In full-face view, head subquadrate, longer than broad (CI 80–86). Mandibles densely striate but shining and armed with five teeth: the apical-most well developed and acute, followed by a less developed preapical tooth and three equally developed smaller teeth. Anterior clypeal margin evenly convex medially. Antennal scapes moderately long: when fully retracted, failing to reach the posterior margin of the head capsule by about the width of the scape (SI 89–97). Antennae 12-segmented; antennal club of composed of three segments, with the apical-most segment about one and a half times as long as the preceding two in combination. Frontal carinae moderately long, extending past the antennal toruli by about twice the maximum width of the antennal scape. Compound eyes weakly protruding past the lateral margins of the head capsule. Lateral margin of head weakly convex, forming a continuous arc from the mandibular insertions to the posterior margin of the head. Posterior head margin flat but rounding evenly into the lateral margins.

In profile view, compound eyes ovular and moderately large (OI 21–23), with 10 ommatidia in longest row. Pronotal declivity indistinct, neck and anterior face of pronotum forming a ~120° angle. Mesosoma evenly convex from where it joins the pronotal neck to the propodeum. Promesonotal suture extending from the posterior margin of the procoxal insertion to the mesothoracic spiracle, which is moderately well developed, then continuing dorsally as a disruption in the ground sculpture. Metanotal groove visible as a weak sulcus and disruption of the sculpture laterally from where it arises between the mid- and hind coxae to where it ends in the poorly developed metathoracic spiracle, which is nearly indistinguishable against the ground sculpture. Propodeal spiracle moderately well developed, directed posterolaterally, and separated from the propodeal declivity by about three and a half spiracle diameters. Propodeal spines well developed and very long (PSI 27–34), slightly longer than the propodeal declivity, flared at the base, straight, and acute. Propodeal declivity weakly concave, forming a rounded ~120° angle with the base of the propodeal spines. Propodeal lobes rounded and weakly developed. Metapleural gland bulla large, extending from the metacoxal insertion three quarters of the way to the propodeal spiracle. Petiole moderately long (PLI 174–233), without tubercles anterodorsally. Subpetiolar process in the form of a small, acute, spiniform tooth; ventral margin of petiole flat posterior to it. Petiolar peduncle moderately long: comprising a little more than a third of the total petiole length. Petiolar node robust, erect, and subquadrate: transition between peduncle and node marked by a rounded angle of ~110°; anterior face forming a ~100° angle with the dorsal face, which is weakly convex; dorsal face meeting the posterior face at a rounded ~90° angle; posterior face forms a ~90° angle with the caudal cylinder. Postpetiole evenly rounded anterodorsally; weakly lobed ventrally.

In dorsal view, humeri developed and distinct: evenly rounded and slightly wider than the rest of the mesosoma; mesothoracic spiracles weakly protruding past the lateral margins of the mesosoma, visible as slight angles where the pronotum meets the mesonotum. Promesonotal suture visible as a disruption in the ground sculpture. Metanotal groove absent: mesonotum and propodeum completely fused and lateral margins converging evenly to the bases of the propodeal spines. Propodeal spines narrowly approximated basally and diverging apically, their apices separated from each other by slightly less than their length, the negative space between them “V” shaped. Petiolar peduncle with spiracles slightly protruding past the lateral margins, and slightly constricted anterior to them. Petiolar node ovular, slightly narrower anteriorly and flattened posteriorly; node the same width as the peduncle, and evenly grading into the caudal cylinder, which is about the same width as the node. Postpetiole narrow (PWI 167–180) and subquadrate. Anterior margin of the postpetiole weakly convex, meeting the lateral margins at a rounded ~90° angle; lateral margins parallel to each other; lateral margin meeting the posterior margin at a ~90° angle; posterior margin flat. Metafemur strongly incrassate (FI 348–392).

Sculpture: median clypeal carina present, extending posteriorly to the level of the antennal insertions, and flanked on either side by one slightly weaker carina. Lateral clypeal lobes with additional, weaker carinae; ground sculpture smooth and shining. Antennal scapes finely areolate-costulate. Cephalic dorsum weakly sculptured: finely costulate between the frontal carinae; fine concentric costulae surrounding the antennal insertions; weak areolate sculpture on the dorsolateral margins behind the compound eyes and on the posterior quarter of the head, otherwise shining. Lateral surfaces of head weakly areolate, with fine concentric costulae around the compound eyes, and fine rugulose sculpture between the compound eye and mandibular insertion. Ventral surface of head smooth and shining medially, with weak costulae and areolae laterally. Pronotal neck areolate. Lateral face of the pronotum areolate; the remainder of the lateral surface of the mesosoma finely rugulose. Propodeal declivity weakly areolate. Dorsal surface of mesosoma finely areolate, with rugulae overlying the ground sculpture; rugulose sculpture much weaker on the propodeum. Femora shining through weak areolate sculpture. Petiole predominantly weakly areolate, but smooth and shining on the ventral surface of the peduncle. Postpetiole weakly areolate, with sculpture becoming stronger on the posterior quarter and the lateral faces. First gastral tergite and sternite smooth and shining, with weak spectral iridescence.

Setae: antennal scapes and funiculi with short, adpressed pilosity. Dorsum of the head, pronotum, waist segments, and gaster with moderately abundant, erect, stout, blunt-tipped setae, the longest of which are about the width of the compound eye. The head bears ~30, mesosoma ~22, petiole 6, postpetiole ~8, and first gastral tergite ~18 setae. Short, sparse, adpressed pubescence present over the entire body, but difficult to detect against the densely sculptured integument.

Color: predominantly testaceous brown, with apex of mandibles, pedicel of antenna, pronotal neck, tibiae, tarsi, and sting testaceous yellow.

**Gyne measurements & indices (*n* = 2):** SL = 0.625–0.634 (0.630); FRS = 0.236–0.249 (0.243); CW = 0.815–0.834 (0.825); CWb = 0.756–0.757 (0.757); PoOC = 0.328–0.333 (0.331); CL = 0.826–0.829 (0.828); EL = 0.230–0.247 (0.239); EW = 0.187–0.204 (0.196); MD = 0.155–0.171 (0.163); WL = 1.321–1.322 (1.322); SPST = 0.340–0.344 (0.342); MPST = 0.344–0.351 (0.348); PEL = 0.478–0.483 (0.481); NOL = 0.216–0.219 (0.218); NOH = 0.173–0.193 (0.183); PEH = 0.317–0.325 (0.321); PPL = 0.229–0.235 (0.232); PW = 0.832–0.833 (0.833); SBPA = 0.361–0.370 (0.366); SPTI = 0.366–0.376 (0.371); PEW = 0.214–0.224 (0.219); PNW = 0.216; PPW = 0.392–0.408 (0.400); HFL = 0.774–0.798 (0.786); HFWmax = 0.171–0.193 (0.182); HFWmin = 0.058–0.076 (0.067); CS = 1.170–1.171 (1.170); ES = 0.324–0.349 (0.336); SI = 83–84 (83); OI = 28–30 (29); CI = 91–92 (91); WLI = 175; SBI = 48–49 (48); PSI = 26; PWI = 182–183 (183); PLI = 203–211 (207); NI = 113–125 (119); PNWI = 96–101 (99); NLI = 45–45 (45); FI = 254–295 (274).

**Gyne description:** In full-face view, head subquadrate, slightly longer than broad (CI 91–92). Mandibles densely striate but shining and armed with five teeth: the apical-most well developed, followed by a less developed preapical tooth and three equally developed smaller teeth. Anterior clypeal margin weakly emarginated medially. Antennal scapes moderately long: when fully retracted, just reaching the posterior margin of the head capsule (SI 83–84). Antennae 12-segmented; antennal club composed of three segments, with the apical-most segment about one and a half times as long as the preceding two in combination. Frontal carinae moderately long, extending past the antennal toruli by about two and a half times the maximum width of the antennal scape. Compound eyes moderately protruding past the lateral margins of the head capsule. Lateral margins of head convex behind the compound eyes, but parallel to each other between the compound eyes and the mandibular insertions. Posterior head margin flat, rounding evenly into the lateral margins.

In profile view, compound eyes ovular and large (OI 28–30), with 16 ommatidia in longest row. Mesoscutum rounded evenly anteriorly, covering the dorsal surface of the pronotum, and weakly convex dorsally, nearly flat. Mesoscutellum very slightly depressed below the level of the mesoscutum and gently sloping posteriorly. Posterior margin of metanotum extending slightly past the posterior margin of the mesoscutum. Propodeal spiracle well developed, directed posterolaterally, and separated from the propodeal declivity by about four spiracle diameters. Propodeal spines stout and well developed, but short (PSI 26), about as two thirds as long as the propodeal declivity, tapering evenly from the base, directed posterodorsally, straight, and acute. Propodeal declivity straight and flat, forming a rounded ~120° angle with the base of the propodeal spines. Propodeal lobes rounded and very weakly developed. Metapleural gland bulla large, extending from the metacoxal insertion three quarters of the way to the propodeal spiracle. Petiole long (PLI 203–211), without tubercles anterodorsally. Subpetiolar process in the form of a small, triangular tooth, which grades evenly into the ventral margin of the petiole posteriorly. Petiolar peduncle moderately long: comprising a little more than a third of the total petiole length. Petiolar node robust and erect: transition between peduncle and node an evenly rounded ~130° angle, resulting in a very slightly concave anterior node face; anterior face forming a rounded ~100° angle with the dorsal face, which is flat; dorsal face meeting the posterior face at a ~100° angle; posterior face forms a ~90° angle with the caudal cylinder. Postpetiole evenly rounded anterodorsally, bulging slightly before it transitions into the flattened posterodorsal face; ventral surface weakly lobed.

In dorsal view, mesoscutum covering pronotum anteriorly, but humeri visible laterally as rounded sclerites. Propodeal spines weakly diverging apically, their apices separated from each other by about one and a half times their length. Petiolar peduncle with spiracles not protruding past the lateral margins, but slightly narrowed anterior to them. Petiolar node subquadrate, slightly broadened transversely. Petiolar node slightly narrower than the peduncle, and evenly grading into the caudal cylinder, which is about the same width as the node. Postpetiole narrow (PWI 182–183), anteroposteriorly compressed, and subquadrate. Anterior margin of postpetiole flat, with corners marked by rounded angles as it transitions to the lateral margins; lateral margins parallel to each other; posterior corners angulate; posterior margin broadly flat. Metafemur moderately incrassate (FI 254–295).

Sculpture: median clypeal carina present, extending from the anterior margin nearly to frontal triangle, and flanked by weaker, indistinct carinae. Lateral clypeal lobes with additional weaker carinae; ground sculpture smooth and shining. Antennal scapes finely areolate-costulate. Cephalic dorsum areolate, with rugose sculpture that becomes costate between the frontal carinae; concentric costulae surround the antennal insertions. Lateral surfaces of head areolate, with rugose sculpture overlying the ground sculpture. Ventral surface of head smooth and shining medially, with weak areolate-rugulose sculpture laterally and posteriorly. Pronotal neck areolate. Anterior face of pronotum shining and weakly, indistinctly sculptured. Lateral face of pronotum costate. Anepisternum and katepisternum shining on their anterior halves, transitioning into weak areolate-costulate sculpture posteriorly. Metapleuron areolate, with costulae. Propodeum weakly areolate laterally. Propodeal declivity weakly areolate. Mesoscutum with costulae over areolate ground sculpture; a patch of finely areolate sculpture present anteromedially. Mesoscutellum longitudinally areolate-costulate. Metanotum areolate. Propodeum areolate, with weak rugae. Femora shining, with traces of weak areolate sculpture. Petiole uniformly finely areolate, with parallel rugae that are dorsoventrally oriented around the petiolar node; dorsum of node rugose. Postpetiole shining through weak areolate sculpture anteriorly, areolate laterally and dorsally; fine concentric costulae dorsally. First gastral tergite and sternite smooth and shining, with weak spectral iridescence.

Setae: antennal scapes and funiculi with short, adpressed pilosity. Dorsum of the head, pronotum, waist segments, and gaster with moderately abundant, erect, stout, blunt-tipped setae, the longest of which are about half the width of the compound eye. Short, sparse, adpressed pubescence present over the entire body, but difficult to detect against the densely sculptured integument.

Color; predominantly testaceous brown, with apex of mandibles, pedicel of antenna, pronotal neck, tibiae, tarsi, and sting testaceous yellow.

**Male:** Unknown.

**Etymology:** Morphological, from the Latin ‘tenuis’ (= weak) + ‘sculptus’ (= sculptured), probably in reference to the smooth and shining integument of the head.

**Comments:** This species is only known from a few collections, including lowland rainforest in Veracruz, Mexico; a nest from between the leaves of *Tillandsia* in mid-elevation mesic forest in Veracruz, and a nest in an oak branch bearing *Epidendrum* orchids, which was intercepted in a shipment from Mexico at the U.S. border by customs officials. This is an arboreally nesting species, similar to the species of the *annexus* species group, with which it is closely related. Very little morphological variation can be seen between the three collections of this species that I have observed.

### *terrigena* group overview

**Figure 170:**
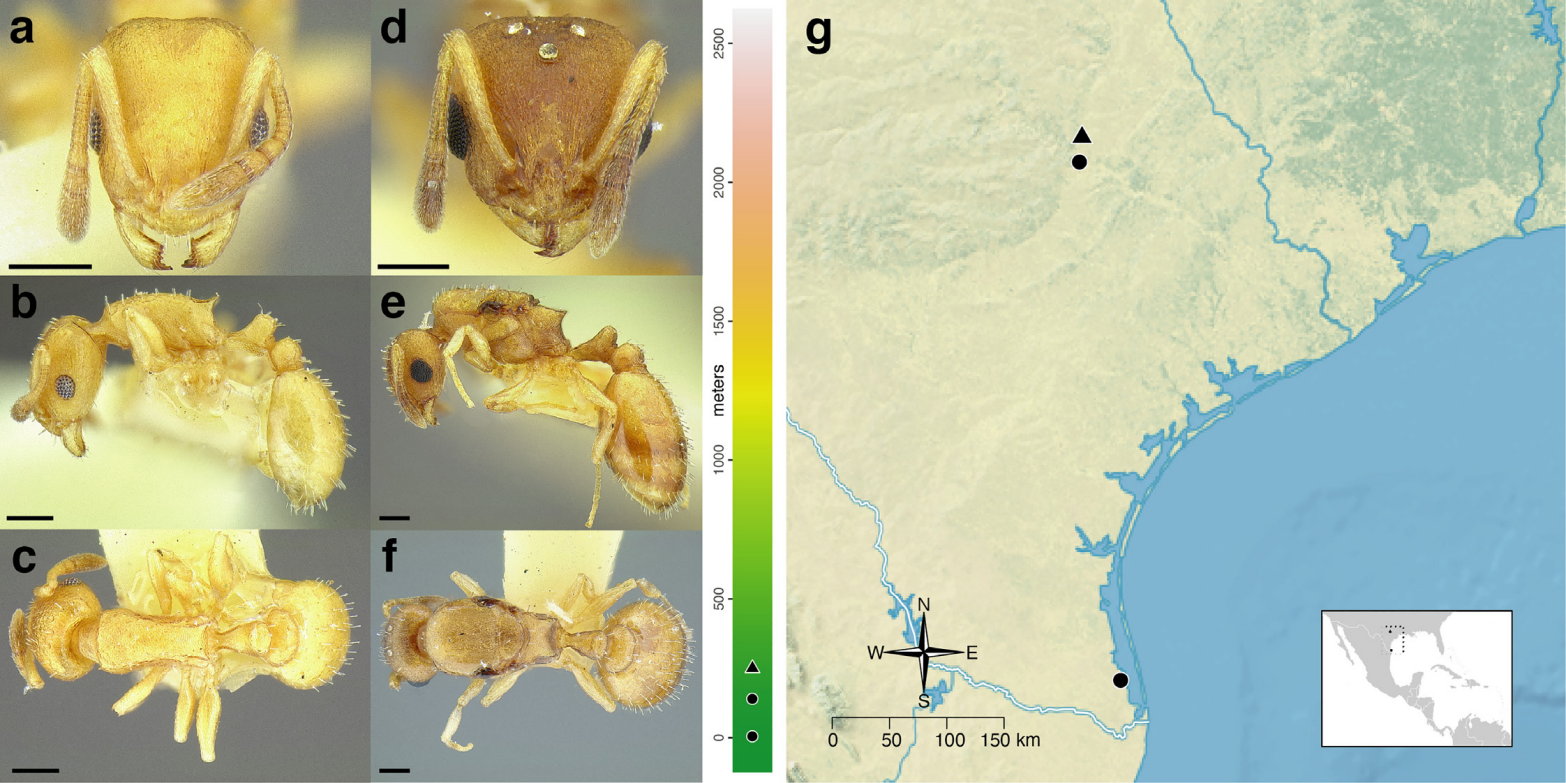
*Temnothorax terrigena*. (A–C) Lectotype worker (MCZENT00522078). (A) Full-face view. (B) Profile view. (C) Dorsal view. (D–F) Paralectotype gyne (MCZENT00522078). (D) Full-face view. (E) Profile view. (F) Dorsal view. (G) Geographical and elevational distribution of specimens examined. Type locality is represented by a triangle, non-type localities are represented by circles. Bounding box in inset map shows location of main map. Scale bars 0.2 mm.

This group is monotypic, with the nominal *Temnothorax terrigena* being the only member. The range of *T. terrigena* small, restricted to the low elevations of the southern United States and northern Mexico (Fig. 170G). Apparently, this species is ground nesting, and the generalized habitus has led to confusion about its relationships historically, but the moderately arched mesosoma dorsum and broad postpetiole are telltale signs of its membership in the *salvini* clade. *Temnothorax terrigena* may be confused with members of the *andrei* and *sallei* clades, and other members of the *salvini* clade. See the ‘similar species’ section below to distinguish this species.

***Temnothorax terrigena* (Wheeler, 1903b)**

Distribution, worker & gyne: Fig. 170.

*Leptothorax terrigena* Wheeler, 1903b: 254. Syntype workers and dealate gyne: McNeil, Texas, U.S.A. One syntype worker here designated **lectotype**.

*Leptothorax (Myrafant) terrigena* (Wheeler): Smith, 1979: 1395. First combination in *Leptothorax (Myrafant)*.

*Temnothorax terrigena* (Wheeler): Bolton, 2003: 271. First combination in *Temnothorax*.

**Type material examined:** *Lectotype worker of Leptothorax terrigena*: U.S.A.: Texas: Travis County, McNeil, 22 February 1902, co-type A.M.N.H., bottom specimen on pin (M.C.Z. Type 21038, MCZENT00522078) [MCZC].

*Paralectotype workers and gyne of Leptothorax terrigena*: same pin as lectotype, 1 dealate gyne, 1 worker (top and middle specimens on pin) [MCZC]; same data as lectotype, 4 workers (M.C.Z. Type 21038, MCZENT00031038) [MCZC] 3 workers (LACMENT181910) [LACM] 3 workers (M.C.Z. Type 21038, USNMENT00528965) [USNM] 3 workers (CASENT0105624) [USNM].

**Non-type material examined:** MEXICO: Tamaulipas: Victoria, [intercepted in quarantine], 7 November 1947, [no collector data], Lar-45405, 47-16324, on Cycad plant, 1 dealate gyne & 7 workers (CASENT0758302-CASENT0758305) [USNM].

U.S.A.: Texas: Cameron County: Laguna Atacosa National Wildlife Refuge, 5 m, 15 December 1984, P.S. Ward#7197-3, coastal mesquite scrub, ground foragers, 1 worker (CASENT0104775) [UCDC]; Travis County: Austin, [no collection date], W.M. Wheeler, 2 workers (CASENT0758301) [USNM].

**Geographic range:** Low elevations, Texas, U.S.A. to Tamaulipas, Mexico (Fig. 170G).

**Worker diagnosis:** *Temnothorax terrigena* can be separated from all other species in the *salvini* clade by the following character combination: in profile view, dorsum of mesosoma weakly convex; metanotal groove not impressed; propodeum not depressed below the level of the promesonotum; propodeal spines shorter than the propodeal declivity and directed posterodorsally; hind femora weakly to moderately incrassate; petiolar node subquadrate, not leaning posteriorly, and rounded dorsally; petiolar node as long as, or only slightly longer than high; posterior face of petiolar node about the same height as the anterior face; postpetiole moderately broad, greater than 1.5 times the width of the caudal cylinder of the petiole, but less than 2.2 times; setae on head, mesosoma, waist segments and gaster erect, short, sparse and blunt (never long and tapering); integument predominantly yellow.

**Similar species:** *Temnothorax andrei, T. carinatus, T. goniops, T. pilicornis* sp. nov., *T. rugosus, T. subditivus* (light form), *T. torrei*. *Temnothorax terrigena* differs from *T. pilicornis* sp. nov. by the pilosity on the antennal scapes and the shape of the petiolar node: the scape setae are subdecumbent and the posterior face of the petiolar node is much shorter than the anterior face in *T. pilicornis* sp. nov. The pedunculate petiole with an erect subquadrate node will separate *T. terrigena* from *T. subditivus*, which has a squamiform petiolar node. *Temnothorax rugosus* has a cuneiform to subcuneiform petiolar node, as opposed to the erect and compact petiolar node of *T. terrigena*. *Temnothorax terrigena* differs from *T. torrei* by the presence of four erect setae on the dorsum of the petiolar node, as opposed to two. *Temnothorax terrigena* can be distinguished from *T. goniops* by the relatively short propodeal spines, which are shorter than the propodeal declivity (longer than the declivity in *T. goniops*). *Temnothorax andrei* has a relatively narrow postpetiole, and both *T. andrei* and *T. carinatus* differ from *T. terrigena* by their head sculpture, which typically has a medial strip of smooth and shining sculpture, as opposed to the uniformly areolate head of *T. terrigena*.

**Worker measurements & indices (*n* = 14):** SL = 0.349–0.403 (0.378); FRS = 0.133–0.165 (0.152); CW = 0.425–0.482 (0.456); CWb = 0.395–0.457 (0.423); PoOC = 0.208–0.233 (0.219); CL = 0.511–0.561 (0.536); EL = 0.102–0.131 (0.117); EW = 0.080–0.101 (0.089); MD = 0.104–0.123 (0.112); WL = 0.539–0.634 (0.586); SPST = 0.128–0.163 (0.143); MPST = 0.177–0.222 (0.194); PEL = 0.211–0.265 (0.228); NOL = 0.101–0.134 (0.118); NOH = 0.080–0.110 (0.100); PEH = 0.159–0.224 (0.198); PPL = 0.140–0.180 (0.156); PW = 0.274–0.314 (0.298); SBPA = 0.127–0.154 (0.141); SPTI = 0.140–0.180 (0.165); PEW = 0.113–0.146 (0.130); PNW = 0.134–0.171 (0.154); PPW = 0.237–0.284 (0.256); HFL = 0.311–0.405 (0.362); HFWmax = 0.102–0.129 (0.116); HFWmin = 0.038–0.050 (0.044); CS = 0.651–0.738 (0.691); ES = 0.142–0.178 (0.161); SI = 83–93 (89); OI = 21–26 (23); CI = 76–82 (79); WLI = 135–143 (138); SBI = 30–36 (33); PSI = 21–28 (24); PWI = 180–215 (198); PLI = 123–161 (147); NI = 98–148 (118); PNWI = 106–134 (119); NLI = 43–61 (52); FI = 225–300 (268).

**Worker description:** In full-face view, head subquadrate and elongate (CI 76–82). Mandibles densely striate but shining and armed with five teeth: the apical-most well developed and acute, followed by a less developed preapical tooth and three equally developed smaller teeth. Anterior clypeal margin strongly convex medially. Antennal scapes short: when fully retracted, failing to reach the posterior margin of the head capsule by about two times the maximum width of the scape (SI 83–93). Antennae 12-segmented; antennal club of composed of three segments, with the apical-most segment about twice as long as the preceding two in combination. Frontal carinae short, extending past the antennal toruli by about one and a half times the maximum width of the antennal scape. Compound eyes moderately protruding past the lateral margins of the head capsule. Lateral margin of head very weakly convex, nearly flat, forming a continuous arc from the mandibular insertions to the posterior margin of the head. Posterior head margin concave medially but rounding evenly into the lateral margins.

In profile view, compound eyes ovular and moderately large (OI 21–26), with 8 ommatidia in longest row. Pronotal declivity distinct: neck and anterior face of pronotum forming a ~110° angle; anterior face and dorsum of mesosoma forming a ~120° angle. Mesosoma very weakly convex from where it joins the pronotal neck to the propodeal spines. Promesonotal suture extending from the posterior margin of the procoxal insertion only to the mesothoracic spiracle, which is moderately well developed. Metanotal groove visible as a disruption of the sculpture laterally from where it arises between the mid- and hind coxae to where it ends in the poorly developed metathoracic spiracle, which is nearly indistinguishable against the ground sculpture. Propodeal spiracle weakly developed, directed posterolaterally, and separated from the propodeal declivity by about four spiracle diameters. Propodeal spines weakly developed and short (PSI 21–28), about half as long as the propodeal declivity, flared at the base, triangular, and acute. Propodeal declivity flat, forming a rounded ~120° angle with the base of the propodeal spines. Propodeal lobes rounded and weakly developed. Metapleural gland bulla moderately large, extending from the metacoxal insertion two thirds of the way to the propodeal spiracle. Petiole short (PLI 123–161), without tubercles anterodorsally. Subpetiolar process in the form of a moderately large, blunt, triangular tooth which grades evenly into the ventral margin of the petiole posteriorly; ventral margin of petiole flat posterior to it. Petiolar peduncle short: comprising about a third of the total petiole length. Petiolar node robust, erect, and rounded-subquadrate: transition between peduncle and node marked by a rounded angle of ~120°; anterior face forming a ~100° angle with the dorsal face, which is convex; dorsal face meeting the posterior face at a rounded ~90° angle; posterior face forms a ~90° angle with the caudal cylinder. Postpetiole strongly convex anteriorly, weakly convex dorsally, and weakly lobed ventrally.

In dorsal view, humeri developed and distinct: wider than the rest of the mesosoma; mesothoracic spiracles weakly protruding past the lateral margins of the mesosoma, visible as slight angles where the pronotum meets the mesonotum. Metanotal groove absent: mesonotum and propodeum completely fused and lateral margins converging evenly to the bases of the propodeal spines. Propodeal spines broadly approximated basally and diverging apically, their apices separated from each other by about twice their length, the negative space between them “U” shaped. Petiolar peduncle with spiracles weakly protruding past the lateral margins, but not noticeably constricted anterior to them. Petiolar node campaniform and antero-posteriorly compressed: convex anteriorly and flat posteriorly; node slightly wider than the peduncle and caudal cylinder. Postpetiole moderately broad (PWI 180–215) and subtrapezoidal, articulating with the nearly the entire anterior margin of the gaster, but leaving small angles on either side exposed. Anterior margin of the postpetiole weakly convex and evenly rounds into the lateral margins; lateral margins converge slightly to the rounded posterior corners; posterior margin broadly concave. Metafemur weakly to moderately incrassate (FI 225–300).

Sculpture: median clypeal carina present but weak, extending posteriorly nearly to the frontal triangle, and flanked on either side by two equally strong carinae. Lateral clypeal lobes with additional, weaker carinae; ground sculpture weakly areolate. Antennal scapes weakly areolate. Cephalic dorsum and lateral surfaces of head uniformly areolate. Ventral surface of head shining through weak areolae. Pronotal neck and anterior face of the pronotum areolate. Lateral surfaces of mesosoma and shining through weak areolate sculpture. Propodeal declivity areolate. Dorsal surface of mesosoma uniformly areolate. Femora shining, with traces of weak areolate sculpture. Petiole and postpetiole areolate, with anterior and dorsal surfaces of petiolar node, and dorso-medial surface of postpetiole smooth and shining. First gastral tergite and sternite smooth and shining, without spectral iridescence.

Setae: antennal scapes and funiculi with short, adpressed pilosity. Dorsum of the head, pronotum, waist segments, and gaster with sparse, erect, blunt-tipped, nearly clavate setae, the longest of which are about the half the width of the compound eye. The head bears ~28, mesosoma ~16, petiole 6, postpetiole ~10, and first gastral tergite ~36 setae. Short, sparse pubescence present over the entire body, but difficult to detect against the lightly colored integument.

Color: predominantly yellow, with masticatory margin of mandibles dark brown; antennal club testaceous yellow.

**Gyne measurements & indices (*n* = 2):** SL = 0.437; FRS = 0.190–0.202 (0.196); CW = 0.596–0.605 (0.601); CWb = 0.541–0.554 (0.548); PoOC = 0.214–0.221 (0.218); CL = 0.599–0.613 (0.606); EL = 0.189–0.209 (0.199); EW = 0.154; MD = 0.108–0.112 (0.110); WL = 0.969–0.989 (0.979); SPST = 0.191–0.207 (0.199); MPST = 0.249–0.266 (0.258); PEL = 0.310–0.319 (0.315); NOL = 0.152–0.165 (0.159); NOH = 0.115–0.133 (0.124); PEH = 0.247–0.268 (0.258); PPL = 0.163–0.211 (0.187); PW = 0.575–0.601 (0.588); SBPA = 0.288–0.306 (0.297); SPTI = 0.257–0.268 (0.263); PEW = 0.161–0.202 (0.182); PNW = 0.216–0.217 (0.217); PPW = 0.339–0.346 (0.343); HFL = 0.466–0.482 (0.474); HFWmax = 0.099–0.124 (0.112); HFWmin = 0.043–0.044 (0.044); CS = 0.841–0.861 (0.851); ES = 0.266–0.286 (0.276); SI = 79–81 (80); OI = 32–33 (32); CI = 90; WLI = 179; SBI = 53–55 (54); PSI = 19–21 (20); PWI = 168–215 (191); PLI = 147–196 (171); NI = 124–132 (128); PNWI = 107–135 (121); NLI = 49–52 (50); FI = 230–282 (256).

**Gyne description:** In full-face view, head subquadrate, slightly longer than broad (CI 90). Mandibles densely, finely striate but shining and armed with five teeth: the apical-most well developed, followed by a less developed preapical tooth and three equally developed smaller teeth. Anterior clypeal margin strongly convex medially. Antennal scapes short: when fully retracted, failing to reach the posterior margin of the head capsule by about twice the maximum scape width (SI 79–81). Antennae 12-segmented; antennal club composed of three segments, with the apical-most segment about twice as long as the preceding two in combination. Frontal carinae short, extending past the antennal toruli by about the maximum width of the antennal scape. Compound eyes moderately protruding past the lateral margins of the head capsule. Lateral margin of head evenly convex, converging from below the compound eyes to the mandibular insertions. Posterior head margin flat, rounding evenly into the lateral margins.

In profile view, compound eyes ovular and large (OI 32–33), with 16 ommatidia in longest row. Mesoscutum rounded evenly anteriorly, covering the dorsal surface of the pronotum, and flat dorsally. Mesoscutellum on the same plane as the mesoscutum. Posterior margin of metanotum extending past the posterior margin of the mesoscutum. Propodeal spiracle moderately well developed, directed posterolaterally, and separated from the propodeal declivity by about four spiracle diameters. Propodeal spines stout and moderately well developed, but short (PSI ~ 20), about a third as long as the propodeal declivity, flared at the base, triangular, and acute. Propodeal declivity straight and flat, forming a ~120° angle with the base of the propodeal spines. Propodeal lobes rounded and very weakly developed. Metapleural gland bulla small, extending from the metacoxal insertion halfway to the propodeal spiracle. Petiole moderately long (PLI 147–196), without tubercles anterodorsally. Subpetiolar process in the form of a moderately large, triangular, blunt tooth, which grades evenly into the ventral margin of the petiole posteriorly. Petiolar peduncle moderately long: comprising about half of the total petiole length. Petiolar node erect and squamiform: transition between peduncle and node a rounded ~130° angle; anterior face forming a ~90° angle with the brief dorsal face; dorsal face meeting the posterior face at a ~90° angle; posterior face forms a ~90° angle with the caudal cylinder. Postpetiole flat anteriorly, bulging slightly anterodorsally, flattened posterodorsally; ventral surface weakly lobed.

In dorsal view, mesoscutum covering pronotum anteriorly, but humeri visible laterally as angulate sclerites. Propodeal spines parallel apically, their apices separated from each other by about three times their length. Petiolar peduncle with spiracles weakly protruding past the lateral margins. Petiolar node transversely campaniform: rounded anteriorly and flattened posteriorly; subquadrate when viewed posterodorsally, the dorsum of the node weakly convex. Petiolar node slightly broader than the peduncle and caudal cylinder. Postpetiole moderately broad (PWI 168–215), anteroposteriorly compressed, and subtrapezoidal, articulating with most of the anterior margin of the gaster, leaving small, angulate margins on each side exposed. Anterior margin of postpetiole flat, with corners marked by rounded angles as it transitions to the lateral margins, which converge slightly to the angulate posterior corners; posterior margin flat. Metafemur weakly to moderately incrassate (FI 230–282).

Sculpture: median clypeal carina present, extending from the anterior margin nearly to frontal triangle, and flanked by two weaker, indistinct carinae. Lateral clypeal lobes with additional weaker carinae; ground sculpture weakly areolate. Antennal scapes weakly areolate. Cephalic dorsum areolate, with weak costulae overlying the ground sculpture. Lateral surfaces of head areolate, with fine costulae between the compound eye and mandibular insertion. Ventral surface of head smooth and shining, with weak costulae. Pronotal neck and anterior face of pronotum areolate. Pronotum with areolate ground sculpture arranged into longitudinal rows by superficial costulae. Katepisternum shining medially, otherwise weakly areolate. Anepisternum areolate. Metapleurae and lateral face of propodeum areolate, with costulae that become stronger over the metapleural gland bulla. Propodeal declivity areolate. Mesoscutum predominantly with costulae over areolate ground sculpture; a patch of smooth and shining sculpture anteromedially. Mesoscutellum with costulae over areolate ground sculpture. Femora shining, with traces of weak areolate sculpture. Petiole and postpetiole uniformly areolate, but sculpture becomes weaker on the anterior face of the petiolar node and anterior face of postpetiole. First gastral tergite and sternite smooth and shining, without spectral iridescence.

Setae: antennal scapes and funiculi with short, adpressed pilosity. Dorsum of the head, pronotum, waist segments, and gaster with sparse, erect, blunt-tipped, nearly clavate setae, the longest of which are about a third of the width of the compound eye. Short, sparse pubescence present over the entire body, but difficult to detect against the lightly colored integument.

Color: predominantly yellow; with antennae, dorsomedial surface of head, posteromedial surface of mesoscutum, metanotum, propodeal declivity, caudal cylinder of the petiole, caudal cylinder of the postpetiole, and distal quarters of the gastral sclerites testaceous yellow. Wing bases and masticatory margin of mandibles dark brown.

**Male:** Unknown.

**Etymology:** Behavioural, from the Latin ‘terra’ (= earth) + ‘genus’ (= born), presumably a reference to the terrestrial nesting and foraging habits of this species.

**Comments:** *Temnothorax terrigena*, which inhabits a range from the Chisos Mountains in southwestern Texas through central Texas, and south to Tamaulipas state in Mexico, apparently inhabits a broad range of habitats, including pinyon forests, desert canyons, high Douglas fir/pine forests, and semiarid plains (van Pelt, 1983, Wheeler, 1903a). It is common in the type locality of Austin, Texas, where it has been collected in litter samples at the Brackenridge Field Laboratory (Feener, 1981). In the original description (Wheeler, 1903b), *T. terrigena* was noted as nesting under stones in black clay soil. Wheeler (1903b) observed workers foraging diurnally and that this species may possibly be pleisiobiotic with other myrmicines: Wheeler collected a nest from a midden pile of the fungus farming ant *Mycetomoellerius turrifex* (Wheeler), and another from a *Pheidole tepicana* Pergande colony. The references to *T. terrigena* from Hidalgo state by Hernández & Castaño-Meneses (2010), Hernández (2013), Vásquez-Bolaños (2015) are incorrect: the exemplar specimen from the study by Hernández & Castaño-Meneses deposited in the UNAM collection proved to be an undescribed species of the *tricarinatus* species group (sensu Mackay, 2000) upon examination.

## Supplemental Information

10.7717/peerj.11514/supp-1 Supplemental Information 1 Raw measurements and collection data for all specimens used in the study.

10.7717/peerj.11514/supp-2 Supplemental Information 2 All named New World *Temnothorax* species with their associated clade memberships, based on Prebus (2017).

## Additional Information and Declarations

### Field Study Permissions

The following information was supplied relating to field study approvals (i.e., approving body and any reference numbers): Field studies were approved by the Secretaria de Medio Ambiente y Recursos Naturales (Mexico; license numbers FAUT-0018 & FAUT-0280), Natural Resources, Parks and Recreation Department (Arizona, U.S.A.), and the Ministerio de Medio Ambiente y Recursos Naturales (Dominican Republic; license number: VAPB-02742).

### Data Availability

The following information was supplied regarding data availability: The raw measurements and collection information, a list of all described species of Temnothorax from the Americas and their associated clades, as recovered in Prebus, 2017 and a study in progress are available in the Supplemental Files. The R script and associated files used to generate the elevation range and geographical distribution maps for the study and two example files are available at Dryad DOI 10.25338/B87W6W.

### New Species Registration

The following information was supplied regarding the registration of a newly described species: Publication LSID: urn:lsid:zoobank.org:pub:F8C827C6-7475-4AF0-B67E-E50786131273 *Temnothorax acuminatus* sp. nov.: urn:lsid:zoobank.org:act:A9B33BCC-C3C0-4A08-A372-844CC2DC1029 *Temnothorax tuxtlanus* sp. nov.: urn:lsid:zoobank.org:act:52AC0BF9-4D74-4D55-9534-5301914B6E89 *Temnothorax acutispinosus* sp. nov.: urn:lsid:zoobank.org:act:05EA9F36-F838-4331-9601-58EC5393C237 *Temnothorax altinodus* sp. nov.: urn:lsid:zoobank.org:act:403FB613-2F8B-4C3D-9801-9669E7DAD87F *Temnothorax arbustus* sp. nov.: urn:lsid:zoobank.org:act:A2419DCD-DA3B-41A1-9CA4-FBB8C5D3BF3E *Temnothorax balnearius* sp. nov.: urn:lsid:zoobank.org:act:F5383574-D914-460A-97BF-D465A00CCA2B *Temnothorax obtusigaster* sp. nov.: urn:lsid:zoobank.org:act:F37C01F0-ADFA-4493-B953-800EB3F6D2B0 *Temnothorax quercicola* sp. nov.: urn:lsid:zoobank.org:act:AA27D744-E327-4A6E-BC1B-A4298B743860 *Temnothorax aureus* sp. nov.: urn:lsid:zoobank.org:act:CEA21155-C3EE-46A1-863A-E05A93BE9421 *Temnothorax leucacanthoides* sp. nov.: urn:lsid:zoobank.org:act:25BBD7A1-CB88-45EA-8808-452CB6466A4F *Temnothorax casanovai* sp. nov.: urn:lsid:zoobank.org:act:E8D4E176-F7C1-4222-92F2-D9E6FAECA64F *Temnothorax misomoschus* sp. nov.: urn:lsid:zoobank.org:act:1F25F9DB-07E5-45A3-B96A-6C11AD1AF37C *Temnothorax nebliselva* sp. nov.: urn:lsid:zoobank.org:act:D52A149B-2C5C-4F32-9FDA-BD54F2663809 *Temnothorax achii* sp. nov.: urn:lsid:zoobank.org:act:0340AA27-740C-47D9-9925-CEE1136FD8BF *Temnothorax xincai* sp. nov.: urn:lsid:zoobank.org:act:7AFD7F16-D394-4339-9024-58F9EDF25371 *Temnothorax rutabulafer* sp. nov.: urn:lsid:zoobank.org:act:5DCE8226-9C5E-42F5-A4C4-86A7D89CBC5D *Temnothorax pilicornis* sp. nov.: urn:lsid:zoobank.org:act:4E21ED68-777E-42C2-8150-5BABDB875E4B *Temnothorax bison* sp. nov.: urn:lsid:zoobank.org:act:F59A4882-C8C7-4C0C-A340-F2DC34D2EB0A *Temnothorax agavicola* sp. nov.: urn:lsid:zoobank.org:act:B75128CF-3EF5-4A54-836C-78D2467CC2B2 *Temnothorax bahoruco* sp. nov.: urn:lsid:zoobank.org:act:3F4A8C33-A52C-46A1-9D1E-6045483D3A5A *Temnothorax balaclava* sp. nov.: urn:lsid:zoobank.org:act:7371C17B-9CB9-4B95-974D-698A5C8A4D0E *Temnothorax harlequina* sp. nov.: urn:lsid:zoobank.org:act:3EF66CE1-DEEF-4899-B201-B98922D25BA6 *Temnothorax hippolytus* sp. nov.: urn:lsid:zoobank.org:act:5FB1108F-9F0D-4A50-91C5-EF15C5CCF106 *Temnothorax laticrus* sp. nov.: urn:lsid:zoobank.org:act:55901403-8078-4FCE-95C5-B5E6BB276AFE *Temnothorax magnabulla* sp. nov.: urn:lsid:zoobank.org:act:EA3B533C-2B35-4B1B-956A-0DDB39C634A2 *Temnothorax wettereri* sp. nov.: urn:lsid:zoobank.org:act:34AB3259-2072-4D21-9969-D7DA47F761AE *Temnothorax wilsoni* sp. nov.: urn:lsid:zoobank.org:act:9206D88F-5B66-447F-9427-62602B2C065B *Temnothorax parralensis* sp. nov.: urn:lsid:zoobank.org:act:ECFE3D3B-DB7B-4FEF-9038-A17AFFBD1AF7 *Temnothorax aztecoides* sp. nov.: urn:lsid:zoobank.org:act:6247BABA-BB2A-41CD-BCFB-BFAC66252022 *Temnothorax fortispinosus* sp. nov.: urn:lsid:zoobank.org:act:0659C21D-40BE-4ECC-BA43-F38C163D0A0B *Temnothorax longicaulis* sp. nov.: urn:lsid:zoobank.org:act:F26D1C85-41F5-413A-835F-080D5AE40763 *Temnothorax longinoi* sp. nov.: urn:lsid:zoobank.org:act:791ADD76-E440-4997-8230-6FC83B444C9B *Temnothorax paraztecus* sp. nov.: urn:lsid:zoobank.org:act:F8DC8385-B911-4C87-A611-44C799154A98 *Temnothorax parvidentatus* sp. nov.: urn:lsid:zoobank.org:act:E27676C3-85CB-4CE3-A04B-99F4316DF3E1 *Temnothorax quetzal* sp. nov.: urn:lsid:zoobank.org:act:A4112861-0551-457F-82AB-E857DB069FE3 *Temnothorax terraztecus* sp. nov.: urn:lsid:zoobank.org:act:7EB02FA9-C6D9-4029-8D26-D4E855100C16.
